# Connectivity mapping of angiotensin-PPAR interactions involved in the amelioration of non-alcoholic steatohepatitis by Telmisartan

**DOI:** 10.1038/s41598-019-40322-1

**Published:** 2019-03-08

**Authors:** Jung Gyu Park, Jong Soo Mok, Young In Han, Tae Sub Park, Keon Wook Kang, Cheol Soo Choi, Hee Dong Park, Joonghoon Park

**Affiliations:** 1 LG Chem R&D Campus, Daejeon, Korea; 2 Graduate School of International Agricultural Technology, Seoul National University, Seoul, Korea; 3 Institute of Green Bio Science and Technology, Seoul National University, Seoul, Korea; 4 College of pharmacy, Seoul National University, Seoul, Korea; 5 Korea mouse metabolic phenotyping center, Lee Gil Ya cancer and diabetes institute, Gachon University School of Medicine, Seongnam-si, Republic of Korea; 6 Endocrinology, Internal Medicine, Gachon University Gil Medical Center, Seongnam-si, Republic of Korea

## Abstract

Nonalcoholic fatty liver disease (NAFLD) is a global health problem that is associated with various metabolic disorders. Telmisartan is a potential treatment for NAFLD due to its ability to improve insulin sensitivity and decrease hepatic fat accumulation via modulation of PPARγ, and to suppress hepatic fibrosis by blocking angiotensin II receptors. However, the underlying mechanisms of action of telmisartan have yet to be fully elucidated. In the present study, diabetic nonalcoholic steatohepatitis (NASH) mice (STAM mice) received daily administrations of telmisartan for 6 weeks to assess the improvements in NASH. Hepatic transcriptome analyses revealed that the amelioration of NASH likely occurred through the regulation of inflammatory- and fibrosis-related gene responses. An integrated network analysis including transcriptional and non-transcriptional genes regulated by telmisartan showed that the NAFLD pathway is interconnected with the dysregulated RAS-PPAR-NFκB pathways. The downstream targets of PPARα, PPARδ, and RELA in this network significantly overlapped with telmisartan-induced differentially expressed genes (DEGs), which were verified in palmitate-treated Hepa1c1c7 cell line. This transcriptome approach accompanied with cell-based molecular analyses provided the opportunity to understand the fundamental molecular mechanisms underpinning the therapeutic effects of telmisartan, and will contribute to the establishment of a novel pharmacological treatment for NASH patients.

## Introduction

NAFLD is a global health problem with a prevalence of approximately 30% in Western countries^1^, and a rapidly increasing prevalence (with a trend towards a younger onset) in Asian countries^2^. NAFLD is highly associated with metabolic disorders such as obesity, insulin resistance, type 2 diabetes mellitus, dyslipidemia, and hypertension^3^. Additionally, NAFLD covers a broad spectrum of pathological abnormalities ranging from simple steatosis and NASH to advanced fibrosis and cirrhosis^4^. Furthermore, NASH is recognized as a significant risk factor for hepatocellular carcinoma (HCC)^5,6^.

A decade ago, it was proposed that NASH developed due to hepatic steatosis followed by the production of gut-derived endotoxins^7^. More recently, it was proposed that numerous factors act in concert to induce NASH, including genetic predisposition, abnormal lipid metabolism, oxidative stress, lipotoxicity, mitochondrial dysfunction, altered production of cytokines and adipokines, gut dysbiosis, and endoplasmic reticulum stress^3^. However, the pathogenesis of NASH has yet to be fully elucidated. Transcriptional profiling studies with cohorts stratified based on histological liver parameters have demonstrated that several genes involved in the Wnt pathway, metabolism, cellular proliferation and extracellular matrix (ECM) organization are dysregulated during the progression of NAFLD^8,9^. Additionally, an elegant study by Lefebvre *et al*.^4^, which investigated NASH disease activity using whole genome profiling, revealed that gastric bypass, which is a surgical procedure that effectively improves NASH, significantly normalizes ECM homeostasis-associated genes. Thus, transcriptomic investigations have elucidated the genetic contributors to NAFLD progression, and also provided an opportunity to establish novel pharmacological and/or medical treatment options.

Pharmacological agents, such as PPARγ activators, lipid-lowering agents, cytoprotective agents, and antioxidants have been used to treat NASH patients^10^. However, no optimal therapeutic strategy has yet been established; thus, there is a need for novel NASH treatment modalities. Previous studies have suggested that the renin-angiotensin system (RAS) may play a critical role in the progression of NAFLD, because activation of this system potentiates the accumulation of triglycerides, decreases hepatic fatty acid oxidation, alters very low-density lipoprotein secretion, and increases *de novo* lipogenesis in the liver^11^. Additionally, the RAS-mediated activation of hepatic stellate cells results in the acquisition of a myofibroblast-like phenotype^12^. Taken together, these findings indicate that suppression of the RAS may be a potentially effective treatment for NAFLD. Telmisartan is an angiotensin II receptor (AGTR1) antagonist used for the management of hypertension, which is the principle effector of RAS. Recently, it was demonstrated that telmisartan is a bifunctional molecule that activates PPARγ and blocks angiotensin II receptors^13^. This unique feature allows telmisartan to improve insulin sensitivity and decrease hepatic fat accumulation via the modulation of PPARγ, as well as suppress hepatic fibrosis by blocking angiotensin II receptors^14,15^. Clinical trials have shown that telmisartan improves fibrosis and the NAFLD activity score (NAS) in patients with NASH or NAFLD, and thus has beneficial effects on fatty liver patients^16,17^. However, the molecular mechanisms of telmisartan, and the interaction between the RAS and PPAR, have yet to be fully investigated.

In the present study, telmisartan efficiently prevented the development of NASH in STAM mice. Additionally, hepatic transcriptomic analyses revealed that the amelioration of NASH likely occurred via regulation of inflammatory- and fibrosis-related responses, and an integrated analysis of transcriptional and non-transcriptional genes regulated by telmisartan identified cross-talk between angiotensin-PPAR-NFκB pathways that could contribute to the effects of telmisartan on NASH. This alternative approach to assessing the transcriptome accompanied with the cell-based molecular analyses provided the opportunity to elucidate the underlying molecular mechanisms of the therapeutic effects of telmisartan and will contribute to the establishment of novel pharmacological treatments for NASH patients.

## Results

### Telmisartan-induced amelioration of NASH in STAM mice

**Figure 1:**
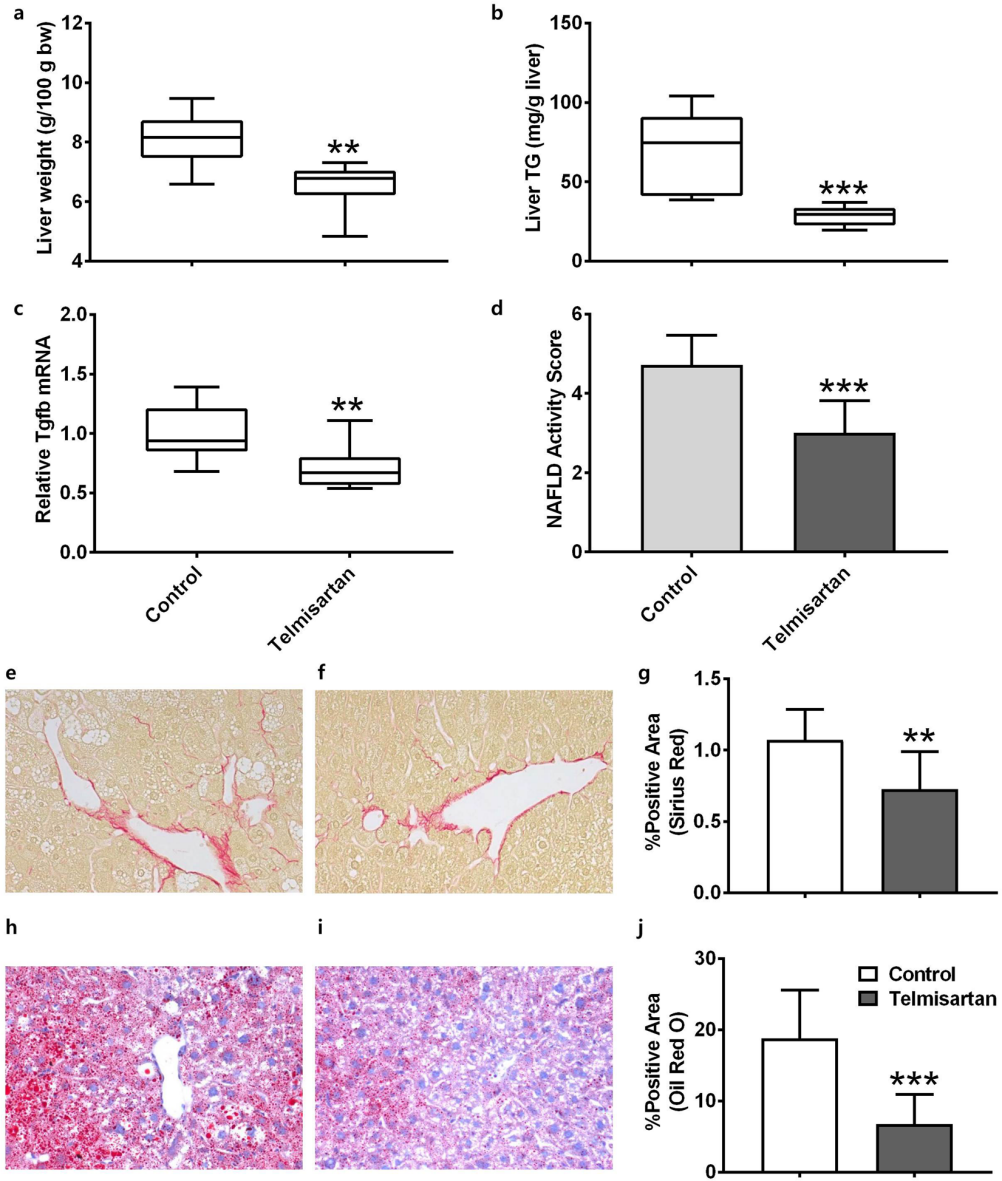
Telmisartan-induced improvements of NASH in STAM mice. Relative liver weights of the mice (**a**). Triglyceride levels in the liver tissues (**b**). mRNA expression levels of *Tgfb* gene (**c**). NAFLD activity score (**d**). Lipid accumulation in vehicle- (**e**) and telmisartan- (**f**) treated livers and quantification of positive areas (%) of Sirius red in liver tissues (**g**). Degrees of fibrosis in vehicle- (**h**) and telmisartan- (**i**) treated livers and quantification of positive areas (%) of oil red O in liver tissues (**j**). Images were captured under 200 × magnification. Horizon bars in the box plots indicate mean values and whiskers indicate minimum and maximum values. Bar graph values are presented as means ± standard deviation (SD). n = 7 per group; **p < 0.01, ***p < 0.001 vs. control.

The pharmacological effects of telmisartan were evaluated in STAM mice from the steatosis stage (6 weeks of age) to the fibrosis stage (12 weeks of age). After 6 weeks of treatment, the bodyweights of the vehicle and telmisartan-treated mice did not differ significantly (19.4 ± 3.2 and 19.5 ± 2.3 g, respectively; *p* = 0.4963). In blood chemistry analyses, hypertension-related parameters including plasma triglyceride (TG) and low-density lipoprotein (LDL) were significantly reduced (*p* < 0.05), and high-density lipoprotein (HDL) was significantly increased (*p* = 0.0007) by telmisartan (Table 1). The liver/bodyweight ratios of the vehicle and telmisartan-treated mice were 8.07 ± 0.92 and 6.49 ± 0.83 g/100 g bodyweight, respectively, which indicates that telmisartan significantly reduced relative liver weight compared to the vehicle (*p* = 0.027; Fig. 1a). Consistent with the liver/bodyweight ratio, the liver TG levels of the vehicle and telmisartan-treated mice were 67.6 ± 25.8 and 28.1 ± 6.2 mg/g liver, respectively, which shows that liver TG levels were significantly reduced by telmisartan compared to vehicle (*p* = 0.0003; Fig. 1b). The relative mRNA levels of fibrosis-related *Tgfb* in the vehicle and telmisartan groups were 1.00 ± 0.23 and 0.72 ± 0.19, respectively, which indicates that telmisartan significantly decreased *Tgfb* expression (*p* = 0.015; Fig. 1c). Histological examinations revealed that the telmisartan-treated mice exhibited reduction of liver steatosis, lobular inflammation as well as hepatocyte ballooning compared to vehicle control. The NAS values in the vehicle and telmisartan groups were 4.71 ± 0.76 and 3.00 ± 0.82, respectively, which indicates that telmisartan significantly reduced the NAS relative to the vehicle (*p* = 0.0008; Fig. 1d, Supplementary Table 1). Furthermore, the percentages of Sirius red-positive areas in the vehicle and telmisartan groups were 1.07 ± 0.22 and 0.73 ± 0.26, respectively, which shows that telmisartan significantly reduced the degree of liver fibrosis compared to vehicle (*p* = 0.01; Fig. 1e–g). Oil red O-positive areas in the vehicle and telmisartan groups were 18.77 ± 6.81% and 6.76 ± 4.17%, respectively, which indicates that the presence of vesicular fat in telmisartan-treated liver tissues was significantly reduced (*p* = 0.0009; Fig. 1h–j). Taken together, these findings indicate that 6 weeks of daily treatment with telmisartan efficiently prevented fibrosis and lipid accumulation in the liver and ameliorated NASH in STAM mice.

**Table 1:** Analyses of blood chemistry in telmisartan-treated STAM mice.

	Control	Telmisartan	*p*-value
ALT (U/L)	43.1 ± 21.5	26.7 ± 4.1	0.011
Liver TG (mg/dL)	107.2 ± 37.6	63.8 ± 21.1	0.0036
TBIL (mg/dL)	0.135 ± 0.071	0.122 ± 0.089	0.3719
Plasma TG (mg/dL)	930.2 ± 274.9	578.1 ± 398.3	0.0185
TCHO (mg/dL)	167.2 ± 15.5	178.0 ± 20.2	0.1027
HDL (mg/dL)	65.3 ± 13.4	86.1 ± 9.7	0.0007
LDL (mg/dL)	15.2 ± 11.0	8.1 ± 3.6	0.04
BUN (mg/dL)	33.4 ± 9.7	68.1 ± 16.5	<0.0001
CREA (mg/dL)	0.285 ± 0.065	0.417 ± 0.197	0.0385
GLU (mg/dL)	691.9 ± 83.7	881.0 ± 119.3	0.0004

ALT: alanine aminotransferase, TG: triglyceride, TBIL: total bilirubin, TCHO: total cholesterol, HDL: high-density lipoprotein, LDL: low-density lipoprotein, BUN: blood urea nitrogen, CREA: creatinine, GLU: glucose, N = 9~10 animals per group.

### Transcriptional genes-regulated by telmisartan

**Figure 2:**
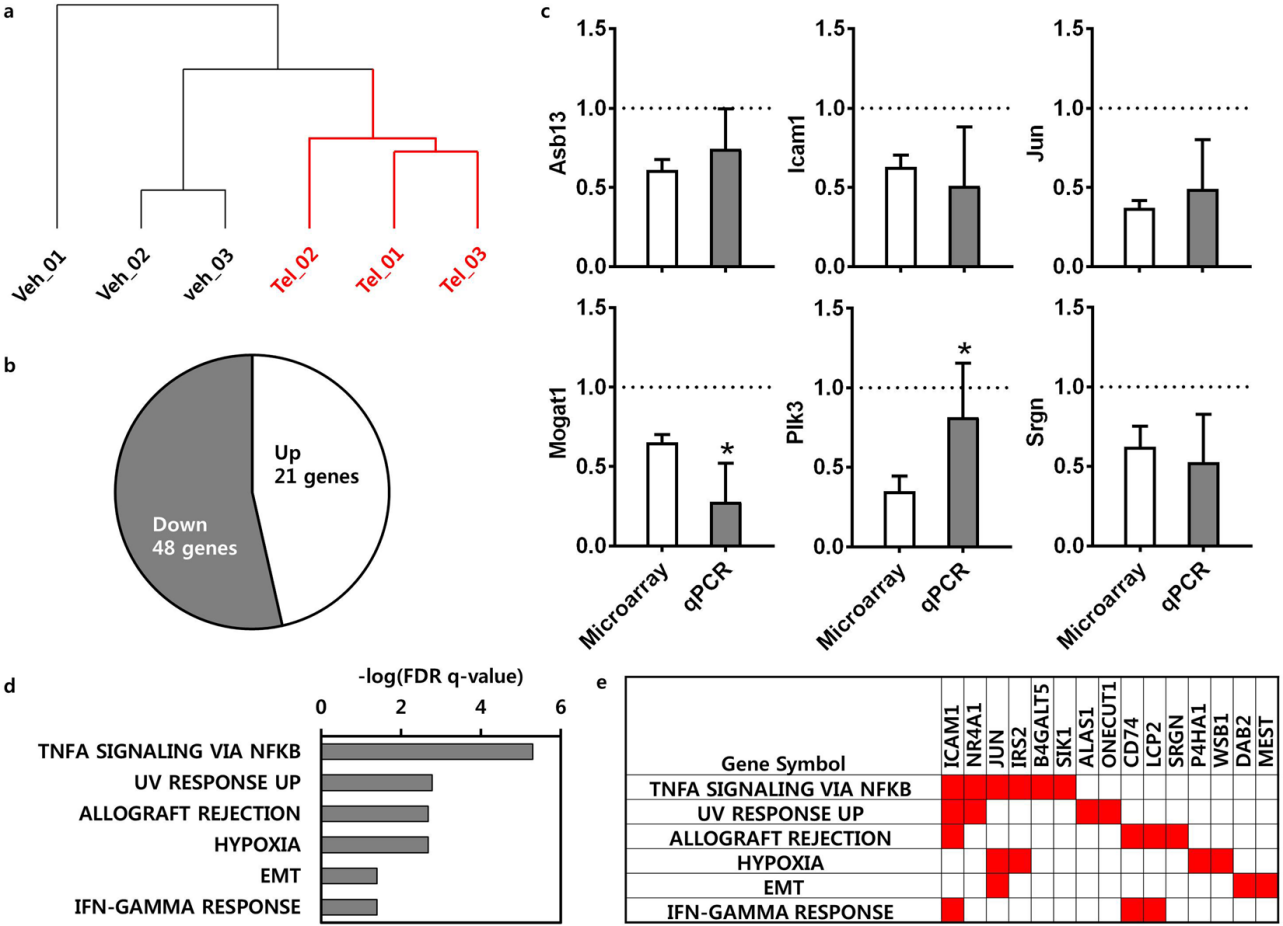
Transcriptional genes-regulated by telmisartan. Unsupervised hierarchical clustering of probes in selected liver tissues (n = 3); veh is vehicle control (black) and Tel is telmisartan treatment (red) (**a**). DEGs induced by telmisartan in the liver tissues; up-regulated genes were counted in the white area, and down-regulated genes in the gray area, in the pie graph (**b**). qRT-PCR validation of microarray results. Bar graph values are presented as means ± SD. Grid line indicates *Gapdh*-normalized mRNA level of control. n = 6 per group; *p < 0.05 vs. microarray (**c**). Biological states or processes down-regulated by telmisartan in the liver tissues; data are presented as –log_10_ (FDR *q*-value) (**d**). Enriched gene set in biological states or processes down-regulated by telmisartan (**e**).

Transcriptomic analyses of the liver tissues from randomly selected mice (three per group) were performed to identify the differentially expressed transcripts due to telmisartan. After normalization, a total of 31,873 probes with signals common to all samples were subjected to hierarchical clustering; the liver transcriptomes of the vehicle and telmisartan-treated groups exhibited distinct clusters (Fig. 2a, Supplementary Table 2). Overall, 69 DEGs exhibited a significant change of at least 1.2-fold up- or down-regulation due to telmisartan compared to vehicle (*p* < 0.05). Of these DEGs, 21 were up-regulated and 48 were down-regulated by telmisartan (Fig. 2b, Supplementary Table 3). To validate the microarray result, the expression levels of the down-regulated genes by telmisartan were verified by quantitative RT-PCR (qRT-PCR), those included ankyrin repeat and SOCS box containing 13 (*Asb13*), intercellular adhesion molecule 1 (*Icam1*), *Jun*, monoacylglycerol O-acyltransferase 1 (*Mogat1*), polo like kinase 3 (*Plk3*), and Serglycin (*Srgn*). qRT-PCR confirmed the consistent reduction of the mRNA levels of these genes by telmisartan compared to vehicle control in the liver tissues (Fig. 2c). The up- and down-regulated genes were separately applied to functional enrichment using Gene Set Enrichment Analysis [GSEA; false discovery rate (FDR) *q* < 0.05] to clarify whether the molecular functions were activated or inhibited by telmisartan. There were no functions enriched with up-regulated genes. In the contrary, several inflammatory- and fibrosis-related functions were down-regulated by telmisartan, included TNFα signaling via NFκB (*q* = 4.99E-06), allograft rejection (*q* = 1.65E-03), the IFNγ response (*q* = 3.93E-02), and epithelial-mesenchymal transition (EMT; *q* = 3.94E-02; Fig. 2d, Supplementary Table 4). A total of 15 down-regulated genes were enriched in these functions (Fig. 2e), and these genes were considered to be the differentially expressed as well as functionally enriched genes that were transcriptionally regulated by telmisartan. Subsequently, these genes were used as transcriptional genes regulated by telmisartan to construct the regulatory network.

### Non-transcriptional genes regulated by telmisartan

**Figure 3:**
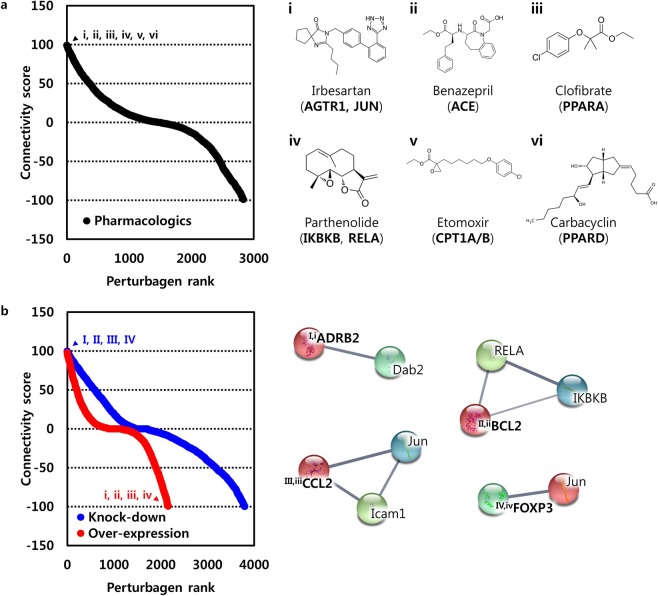
Non-transcriptional genes-regulated by telmisartan. (Left) Connectivity score and rank of pharmacologic; black dotted line indicates pharmacologic perturbagens. (Right) Chemical structures of top ranked pharmacologic perturbagens were depicted and their corresponding target genes were described in parentheses (**a**). (Left) Connectivity score and rank of genetic perturbagens; blue dotted line indicates genetic perturbagens by knock-down, and red dotted line indicates genetic perturbagens by overexpression. (Right) Strongly paired top ranked genes were depicted. Upper-case characters indicate non-transcriptional genes regulated by telmisartan; lower-case characters indicate transcriptional genes regulated by telmisartan; bold characters indicate strongly paired genes. Interaction between gene products were depicted by edges (**b**).

The identification of transcriptional-regulated genes due to the perturbation of non-transcription factors is challenging and could have a serious negative impact on the construction of a precise pathway and/or network for understanding the molecular mechanisms of drugs. To identify non-transcriptional-regulated genes associated with telmisartan, 40 telmisartan-induced DEGs were queried in the Connectivity Map (CMap). CMap analyses were conducted using the Touchstone signature dataset, which was generated by pharmacological perturbation for identification of drugs and their target genes, as well as by paired genetic perturbation through knockdown or over-expression. As shown in Table 2, CMap analyses revealed that the telmisartan-induced DEGs were connected with irbesartan (angiotensin receptor antagonist, connectivity score: 99.98), benazepril (angiotensin converting enzyme inhibitor, connectivity score: 99.96), clofibrate (PPAR receptor agonist, connectivity score: 99.96), parthenolide (NFκB pathway inhibitor and adiponectin receptor agonist, connectivity score: 99.88), etomoxir (carnitine palmitoyltransferase inhibitor, connectivity score: 99.87), and carbacyclin (PPARδ receptor activator, connectivity score: 99.85) (Fig. 3a, Supplementary Table 5). The associated protein targets of the drugs retrieved from CMap were as follows: AGTR1 and JUN for irbesartan, ACE for benazepril, PPARα and LPL for clofibrate, IκBKB and RELA for parthenolide, CPT1A and CPT1B for etomoxir, and PPARδ for carbacyclin. Next, the paired genetic perturbagens that were transcriptionally similar to the telmisartan-induced DEGs in CMap were investigated (Fig. 3b, Supplementary Table 6); the strongly paired genes included *FOXP3* (connectivity score by knockdown/over-expression: 98.92/−97.31), *CCL2* (connectivity score: 91.16/−98.32), *ADRB2* (connectivity score: 99.73/−97.86), and *BCL10* (connectivity score: 97.23/−96.73). This approach identified 11 target genes of pharmacological perturbagens and 10 genetic perturbagens; these genes were regarded as non-transcriptional-regulated genes by telmisartan. Subsequently, these genes were used to construct the regulatory network of telmisartan in combination with the transcriptional-regulated genes.

**Table 2:** Pharmacologic and genetic perturbagens connected with telmisartan-induced DEGs.

Cell line	Perturbagen	Description	Target(s)	Connectivity score
A375	irbesartan	angiotensin receptor antagonist, liver bile acid transporter inhibitor	AGTR1, JUN, SLC10A1	99.98
PC3	benazepril	angiotensin converting enzyme inhibitor	ACE	99.96
HT29	clofibrate	PPAR receptor agonist	PPARA, LPL	99.96
A549	parthenolide	NFkB pathway inhibitor, adiponectin receptor agonist	ADIPOR2, IKBKB, RELA	99.88
VCAP	etomoxir	carnitine palmitoyltransferase inhibitor, carnitine O-palmitoyltransferase inhibitor, fatty acid oxidation inhibitor	CPT1A, CPT1B	99.87
HEPG2	carbacyclin	IP receptor activator, PPARbeta receptor activator	PPARD, PTGDR, PTGER1, PTGER2, PTGER3, PTGER4, PTGFR, PTGIR, TBXA2R	99.85
PC3	KD	Forkhead boxes, forkhead box P3	FOXP3	98.92
	OE			−97.31
VCAP	KD	Chemokine ligands, chemokine (C-C motif) ligand 2	CCL2	91.16
	OE			−98.32
A375	KD	B-cell CLL/lymphoma 10	BCL10	97.23
	OE			−96.73
HT29	KD	GPCR/Class A: Adrenoceptors: beta, adrenergic, beta-2-, receptor, surface	ADRB2	99.73
	OE			−97.86

KD: knock-down, OE: over-expression.

### Telmisartan-induced regulatory network for improvement of NASH

**Figure 4:**
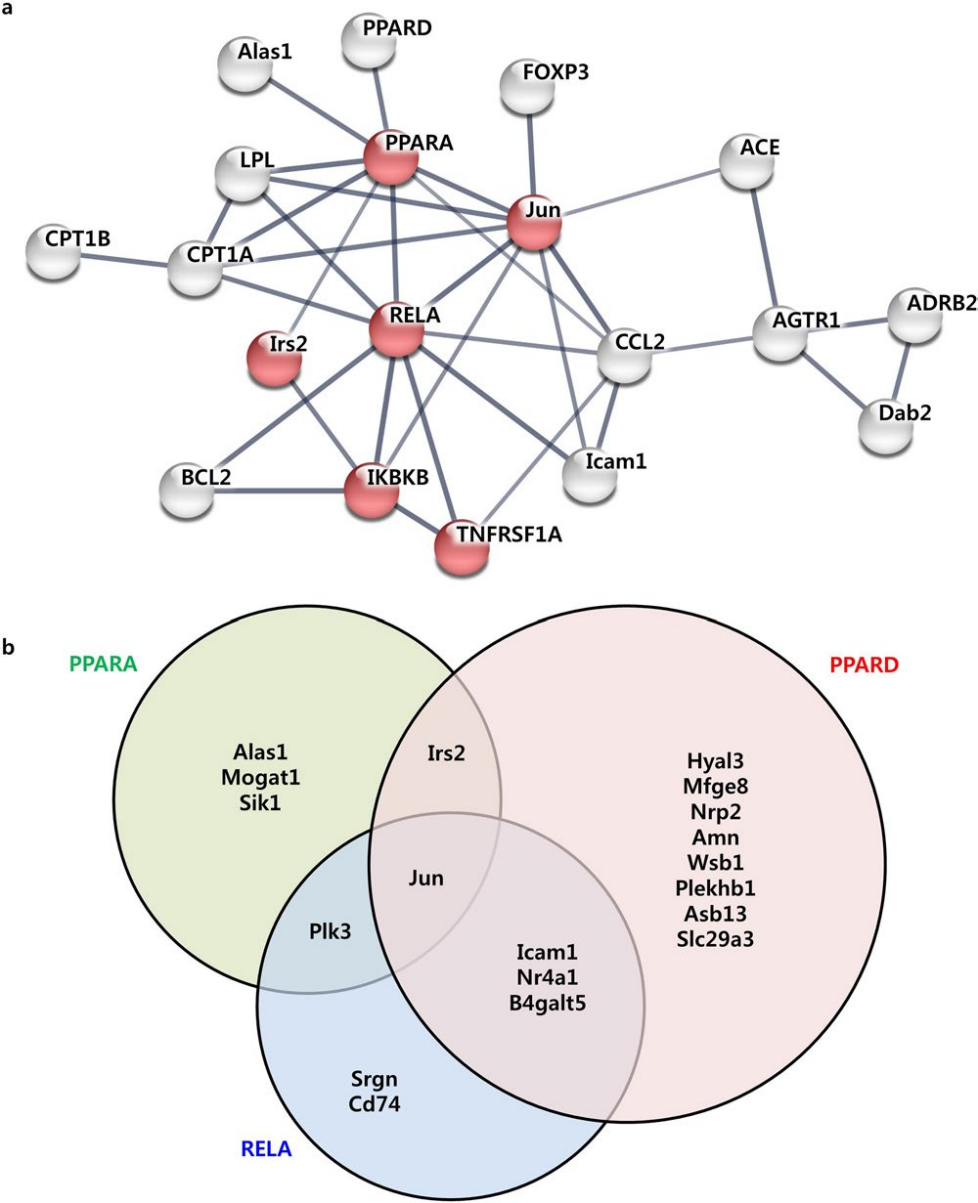
Angiotensin-PPAR-NFκB signaling pathway-associated regulatory network induced by telmisartan. STRING PPI network constructed with transcriptional and non-transcriptional genes regulated by telmisartan. Red nodes indicated genes enriched in the NAFLD pathway. Upper-case characters in the nodes indicate non-transcriptional regulated genes from CMap analyses, lower-case characters indicate transcriptional genes regulated by telmisartan (**a**). Downstream targets of transcription factors in the network. Venn diagram shows direct targets of PPARA in green circle, PPARD in red, or RELA in blue from telmisartan-induced DEGs (**b**).

**Figure 5:**
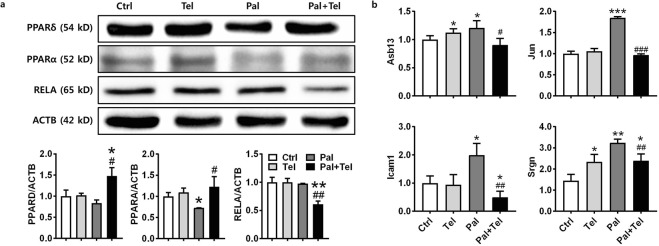
Regulation of PPARα, PPARδ, and RELA pathway by telmisartan in lipotoxicity-induced Hepa1c1c7 cells. Western blotting of differentially expressed PPARα, PPARδ, and RELA by telmisartan in Hepa1c1c7 cells with or without palmitate. Expression values were normalized with ACTB. (**a**). Differential expression of target genes of PPARα, PPARδ, or RELA by telmisartan in Hepa1c1c7 cells with or without palmitate. Expression values were normalized with *Gapdh* (**b**). Bar graph values are presented as means ± SD, n = 3; *p < 0.05, **p < 0.01, ***p < 0.001 vs. control; ^#^*p* < 0.05, ^##^*p* < 0.01, ^###^*p* < 0.001 vs. palmitate. Ctrl: vehicle control, Tel: telmisartan at 10 µM, Pal: palmitate at 0.2 mM.

The transcriptional and non-transcriptional genes regulated by telmisartan were assessed using STRING and a protein-protein interaction (PPI) network was constructed. A PPI network was generated with 19 protein nodes encoded by telmisartan-regulated genes (Fig. 4a). Of the nodes in the network, six were functionally enriched in the NAFLD pathway (*q* = 2.81E-07), which was located at the center of this network and interconnected with the PPAR signaling pathway (*q* = 2.81E-07), TNFα signaling pathway (*q* = 6.18E-08), and angiotensin signaling pathway. To understand whether this network influenced the expression of telmisartan-induced DEGs, the associations of the transcription factors in the network with the DEGs were investigated using ChIP-X enrichment analysis (ChEA). Interestingly, PPARα, PPARδ, and RELA were significantly associated with 6, 14, and 7 telmisartan-induced DEGs, respectively (Fig. 4b, Supplementary Table 7), which implies that these genes were down-regulated by PPARα, PPARδ, and RELA binding, respectively. Therefore, these three transcription factors appeared to play the essential role as network regulators, exerting an influence on the telmisartan-induced STRING network. To verify whether the telmisartan modulates PPARα, PPARδ and RELA to influence NAFLD network, the protein levels of these transcription factors and the mRNA levels of their downstream target genes were evaluated in Hepa1c1c7 cells. As shown in Fig. 5a, telmisartan alone did not change the protein levels of PPARα, PPARδ and RELA in Hepa1c1c7 cells. In the contrary, palmitate slightly decreased PPARα and PPARδ; furthermore, telmisartan in palmitate-treated Hepa1c1c7 cells significantly increased the levels of PPARα and PPARδ (*p* < 0.05), and decreased RELA (*p* < 0.01; Supplementary Fig. 1). Coincidently, the mRNA levels of the downstream target genes of these transcription factors were inversely correlated with the protein levels of the transcription factors (Fig. 5b). The mRNA levels of *Asb13*, *Icam1* and *Jun* genes, which were predicted to be down-regulated by PPARα and/or PPARδ in ChEA, were significantly increased by palmitate (*p* < 0.05); however, as PPARα and PPARδ increased by co-treatment of telmisartan, the mRNA levels of these genes were significantly decreased (*p* < 0.05). In contrast, palmitate-induced increase of *Srgn* mRNA was significantly reduced (*p* < 0.01) as the RELA decreased by telmisartan. Taken together, the findings of the constructed regulatory network in conjunction with the transcriptional and non-transcriptional genes identified as being regulated by telmisartan indicate that the AGTR1-mediated angiotensin pathway interacted with the PPAR-NFκB signaling pathway, and that the NAFLD pathway was down-regulated through PPARα, PPARδ, and RELA as transcriptional regulators to ameliorate NASH in STAM mice.

## Discussion

Activation of the PPARγ signaling pathway improves insulin resistance, dyslipidemia, adipokine secretion, inflammation, cell proliferation and hepatic steatosis^18,19^. Blockade of the RAS pathway improves oxidative stress, inflammation, and cell proliferation, and also leads to improvements in hepatic fibrosis^20^. Thus, modulation of the PPARγ and RAS pathways would likely confer significant advantages for NASH patients. Based on previous research and the present findings, the bifunctional pharmacological activities of telmisartan, as an angiotensin II receptor antagonist and PPARγ partial agonist, significantly ameliorate NAFLD activity, alter hepatic fat accumulation, and improve hepatic fibrosis. Other PPARγ agonists, such as pioglitazone, also attenuate hepatic steatosis, inflammation, and fibrosis to a degree similar to that of telmisartan, but also affect systemic characteristics such as lipid metabolism and body weight such that rats treated with pioglitazone exhibit increases in body weight and subcutaneous fat. In contrast, telmisartan is associated with mild loss of body weight accompanied by marked decreases in subcutaneous inguinal and epididymal visceral fat^18^. These features differentiate telmisartan and pioglitazone in terms of therapeutic efficacy for NASH patients. Telmisartan also exerts dissociable effects on hepatic steatosis and energy expenditure to those of ordinary angiotensin II receptor antagonists, such as valsartan^13^. These differences may be due to differences in chemical structure. Conventional angiotensin II receptor antagonists in clinical use today are biphenyl tetrazole derivatives, whereas telmisartan is a non-tetrazole derivative that resembles pioglitazone^21^. This unique structural feature appears to grant telmisartan the ability to regulate both carbohydrate and lipid metabolism, which led to improvements in fatty liver and reductions in triglyceride levels, without weight gain, in the present study.

Gene expression profiling analysis at different stages of various diseases represents a sensitive method for elucidating the molecular processes that underlie pathological states. The reversal of NASH and fibrosis by telmisartan seen in the present study suggests that at least some of the transcriptomic alterations were reversible, which allowed for the identification of putative target genes that may potentially be effective against pathological processes. The unsupervised hierarchical clustering of the present hepatic transcriptome data revealed a clear dissociation between the vehicle and telmisartan treatments. Telmisartan induced subtle changes in global gene expression levels, and this may have been due to the adaptive nature of the pathological response to gene expression^4^. However, it is noteworthy that telmisartan appeared to reduce the activities of essential genes that are associated with the inflammatory response and hepatic fibrosis. Of these genes, *Icam1* is important in the inflammatory process of livers with NASH, and thus may be a useful marker for the diagnosis of NASH^22,23^. Increased expression of *Irs2* is associated with steatohepatitis in obese individuals^24^ and seems to be a critical regulator of the synthesis and oxidation of fatty acids in the livers of rats with NASH^25^. Additionally, *Onecut1* and *Cd74* were down-regulated in telmisartan-treated liver tissues but, in contrast to the present findings, these genes were inhibited during hepatic steatosis induction^26,27^. Thus, whether the activities of *Onecut1* and *Cd74* are regulated differentially depending on the NASH induction conditions needs to be clarified.

It is well known that gene activity is regulated by transcription, RNA processing, post-translational modification, and/or PPIs. In the present study, several telmisartan-regulated genes were identified at the transcription level and their cellular and molecular functions were shown to attenuate NASH progression. However, it was challenging to understand the therapeutic mechanisms underlying the effects of telmisartan on NASH, and the interaction between angiotensin II receptors and the PPAR signaling pathway, using only transcriptional-regulated genes. The target genes of transcription factors can be efficiently revealed by knockout expression profiling because transcriptional-regulated genes would be directly regulated by perturbations in transcription factors^28^. However, it would be extremely difficult to investigate the activity of non-transcriptional-regulated genes by gene expression profiling^29^. Thus, CMap provides a significant opportunity to elucidate disease-drug and/or drug-drug connections at the transcription level due to massive pharmacological or genetic perturbations that, in turn, may aid in the identification of the modes of action of certain candidate drugs, and repurpose existing drugs for alternative indications^30,31^. Furthermore, pharmacological or genetic perturbation expression profiles in CMap could provide insight into the transcriptional responses of genes that are regulated in a non-transcriptional manner.

To investigate this hypothesis, the transcriptional genes that were dysregulated by telmisartan were queried in CMap. Surprisingly, CMap revealed that well-known telmisartan associated signaling molecules such as AGTR1, ACE, PPARα, and PPARδ exhibited a pharmacological connection, while other interacting molecules had a genetic connection to telmisartan-induced DEGs; these had never been previously identified by conventional gene expression analyses. Thus, in combination with the transcriptional genes regulated by telmisartan, the telmisartan network that ameliorated NASH in the livers of STAM mice, and which harbored the NAFLD pathway that was interconnected with the RAS and the PPAR-NFκB signaling pathways, was successfully generated. Furthermore, retrospective ChEAs of transcription factors in this network implied that PPARα, PPARδ, and RELA likely play important role in differentially control of target gene activity during the reversal of NASH by telmisartan. AGTR1 has been known to activate the NFκB machinery through MAPK/ERK pathway^32,33^. RelA, p65 subunit of NFκB, also has been known to down-regulate the PPARα^34^ and PPARδ^35^ activity by inhibitory binding. It implies that RelA would be a master regulator of the core transcriptional circuit by telmisartan and mediate RAS-PPAR pathway. Interestingly, PPARγ was not identified among the transcriptional and non-transcriptional genes regulated by telmisartan. PPAR isoforms display tissue-specific expressions. For example, PPARγ is dominant in adipose tissue, whereas PPARδ is found in various tissues and has been identified in high levels in skeletal muscle^36^. The potential agonism of PPARγ by telmisartan was suggested by PPRE-dependent transcription in cells that were similar to pioglitazone-treated cells^14,18^. However, telmisartan also induces anti-fibrotic and anti-obesity effects through PPARδ-dependent pathways^37,38^ and enacts anti-hepatic fibrosis and anti-dyslipidemic effects through PPARα-dependent pathways^4,39^. Therefore, telmisartan appears to inhibit NASH progression by PPARγ activation as well as by partial activation of PPARα and PPARδ through AGTR1 antagonism, resulting in down-regulation of genes related with inflammation and fibrosis in STAM mice.

There are some potential pitfalls to consider. First, STAM mice used in this study represent diabetic, male NASH in human, and it does not explain non-diabetic NASH or female NASH patients. Long-term HFD without streptozotocin (STZ) treatment could be an alternative model for non-diabetic NASH with variable onset and characteristics to improve the clinical relevance of the study. Moreover, STAM mice are known as hypertension insensitive and maybe inappropriate to evaluate the anti-hypertensive effects by telmisartan^40^; however, telmisartan effectively controlled the strong hypertension predictors including plasma TG, LDL and HDL at dose level used in this study. Second, there was no comparison between wild type and STAM mice to evaluate the effect of telmisartan. However, the *in vitro* experiment with Hepa1c1c7 cells demonstrated that telmisartan-induced amelioration of NASH would be steatosis/steatochepatitis-specific and there would be low possibility to observe the transcriptional effect of telmisartan in wild type animals. Third, CMap has limited coverage of perturbagens. Although the coverage has been dramatically increased in the next generation CMap with L1000 platform^30^, it is still retrospective and novel targets which do not have matched perturbagens would be difficult to be connected with biological states. This limitation needs to be improved by expanding the coverage of genetic perturbagens with appropriate test system.

In conclusion, telmisartan efficiently prevented the development of NASH in STAM mice. Additionally, hepatic transcriptomic analyses revealed that the amelioration of NASH possibly occurred via the regulation of inflammatory- and fibrosis-related responses. Integrated analyses of transcriptional and non-transcriptional genes regulated by telmisartan identified cross-talk between the angiotensin-PPAR-NFκB signaling pathways, which could have contributed to the pharmacological effects of telmisartan on NASH. This alternative transcriptomic approach accompanied with the cell-based molecular analyses provided the opportunity to understand the fundamental molecular mechanisms underlying the therapeutic effects of telmisartan, and will contribute to the establishment of novel pharmacological treatments for patients with NASH.

## Methods

### Animal experiment

NASH was induced in C57BL/6 J male mice, as described previously^41^. Briefly, at 2 days after birth, the mice received a single subcutaneous injection of 200 μg STZ (Sigma, St. Louis, MO, USA). Then, after 4 weeks of age, they received 60 kcal% fat HFD32 chow (CLEA Japan Inc., Tokyo, Japan) *ad libitum* and were assigned to receive either vehicle or telmisartan. Telmisartan (Sigma) was dissolved in 0.5% (v/v) carboxymethyl cellulose (Sigma) and orally administered (5 mg/kg/day) to the mice from 6 to 12 weeks of age (n = 7 per group). At termination, liver tissues were obtained from the mice and stored until further analysis. This study was approved by the Institutional Animal Care and Use Committees of Seoul National University (SNU-170912-22) and was conducted in accordance with the approved guidelines.

### Biochemical analysis

Blood chemistry was analyzed using automated chemistry analyzer (Hitachi, Tokyo, Japan), those included ALT, TBIL, plasma TG, TCHO, HDL, LDL, BUM, CREA, and GLU according to Park *et al*.^42^. Total lipid in the liver tissues was extracted using a 2:1 chloroform:methanol solution (v/v) and the TG contents were measured with a Triglyceride E-test kit (Wako, Osaka, Japan) according to the manufacturer’s instructions.

### qRT-PCR

To measure the expression levels of *Tgfb* and ribosomal protein lateral stalk subunit P0 (*36B4*) genes, total RNA was extracted from the liver tissues using RNAiso (Takara, Tokyo, Japan) and cDNA was prepared with Moloney murine leukemia virus reverse transcriptase (Invitrogen, Carlsbad, CA, USA). cDNA was amplified with the ABI 7700 sequence‐detector system (Applied Biosystems, Foster City, CA, USA) using a set of primers and probes that corresponded to *Tgfb* and *36B4* (endogenous control)^41^. To measure the expression levels of *Asb13*, *Icam1*, *Jun*, *Mfge*, *Mogat1*, *Plk3*, and *Srgn* genes, total RNA from the liver tissues (six per group) or cells was extracted using a RNeasy Mini kit (Qiagen) according to the manufacturer’s instructions. cDNA was prepared using SuperScript III Reverse Transcriptase (Invitrogen) and qRT-PCR was performed on a StepOnePlus Real-Time PCR System (Applied Biosystems) with Power SYBR Green PCR Master Mix (Applied Biosystems). Gene expression levels were analyzed by ΔΔC_T_ method using *Gapdh* as an internal control. Primers referred from PrimerBank (https://pga.mgh.harvard.edu/primerbank/index.html)^43^ were summarized in Supplementary Table 8.

### Histological analyses

Water-soluble glycol and resin-embedded liver sections were cut at a thickness of 5 μm, air-dried, fixed in acetone, and then stained with a hematoxylin and eosin solution (Wako). The NAS was evaluated semi-quantitatively, as described previously^44^, the degree of liver fibrosis was assessed with Sirius-red staining, and the presence of vesicular fat in the liver tissues was confirmed using oil red O.

### Microarray analyses

Total RNA was extracted from the frozen liver tissues with a RNeasy mini kit (Qiagen). Following quantitative and qualitative evaluations performed with BioAnalyzer (Agilent, Santa Clara, CA, USA), RNA samples with an RNA integrity number (RIN) ≥ 6.7 and A260/A280 values ≥ 1.88 were subjected to cDNA synthesis, performed with the GeneChip WT cDNA synthesis and amplification kit (Applied Biosystems). Next, the cDNA was fragmented and biotin-labeled using GeneChip WT terminal labeling kit (Applied Biosystems), and approximately 5.5 μg of labeled cDNA was hybridized to the Affymetrix GeneChip Mouse Gene 2.0 ST Array (Affymetrix, Santa Clara, CA, USA) at 45 °C for 16 h. The hybridized arrays were scanned on a GCS3000 Scanner (Affymetrix) and all data analyses were performed with the GeneChip Command Console Software (Affymetrix). All data were normalized using the robust multi-array average (RMA) approach and hierarchical clustering of the expressed probes was performed using GenePattern (https://genepattern.broadinstitute.org). The distance between clusters was computed with Pearson correlations and global gene expression profiling was conducted in triplicate for the vehicle control and telmisartan treatment groups.

### Telmisartan gene signature

Telmisartan-induced DEGs in the liver tissues were identified using a fold change cutoff of 1.2; these genes were compared to the vehicle control group using independent *t*-tests with a *p*-value of 0.05 taken to indicate statistical significance. Next, the telmisartan-induced DEGs were used as seeds to generate a telmisartan gene signature. First, the DEGs were analyzed using GSEA software (http://software.broadinstitute.org/gsea)^45^ to determine the molecular mechanisms of action of telmisartan. Next, the telmisartan-induced DEGs were computationally overlapped with the Molecular Signature Database (MSigDb) using a FDR *q*-value cutoff of 0.05; DEGs enriched in a certain molecular signature were regarded as transcriptional-regulated genes by telmisartan. Next, the transcriptional connections between telmisartan-induced DEGs and chemical and genetic perturbagens were assessed with CMap (https://clue.io)^30^, which is a catalog of transcriptional responses following pharmacological or genetic (knock-down by shRNA or over-expression by transgenesis) perturbations of cell lines; the pharmacological (and their target genes) or genetic perturbagens associated with telmisartan-induced DEGs were considered non-transcriptional-regulated genes by telmisartan. These transcriptional- and non-transcriptional-regulated gene sets were used as the telmisartan gene signature.

### Regulatory networks

A system-wide understanding of the cellular functions induced by telmisartan was obtained using the STRING protein-protein association network database (https://string-db.org)^46^. A telmisartan gene signature generated by GSEA and CMap analyses was used to construct both the experimental and predicted interactions of the signature molecules using a confidence level of 0.7. To verify the experimental relevance of the network, transcription factors in the constructed network were associated with telmisartan-induced DEGs using the ChEA gene set library^47^, which is a comprehensive resource for targets of transcription factors in various cell types, mammalian organisms, and microarray platforms, as determined by ChIP-seq.

### Cell experiment

Hepa1c1c7 murine hepatoma cells (Korean Cell Line Bank, Korea) were maintained in αMEM medium without nucleosides (ThermoFisher Scientific, Waltham, MA, USA) with 10% FBS (ThermoFisher Scientific) at 37 °C, 5% CO_2_. After reaching 70% confluency, Hepa1c1c7 cells were treated with 0.2 mM palmitate (Sigma) for 9 h to induce lipotoxicity. The concentration of palmitate was determined by cell viability assay which did not cause significant cell death. Telmisartan at 10 µM was treated for 24 h after palmitate treatment according to Li *et al*.^36^.

### Western blotting

Total protein collected from cells was subject to dodecyl sulfate-poly acrylamide electrophoresis, and then transferred to nitrocellulose membranes. Membranes were incubated with anti-PPARδ, PPARα (1:500) and anti-RELA (1:250) antibodies (ThermoFisher Scientific). Expression levels of proteins were analyzed by ImageJ (https://imagej.nih.gov/ij/) using ACTB as an internal control.

### Statistical analyses

All animal data were analyzed with Student *t*-tests or Mann-Whitney U tests depending on the homogeneity of variance of the data. All statistical analyses were performed using Prism software (ver. 7.03; GraphPad Software, Inc., San Diego, CA, USA) with *p*-values < 0.05 considered to indicate statistical significance. All measurements are reported as means ± standard deviation (SD).

## Supplementary information

Supplementary Information

## Data Availability

The dataset generated during the current study are available in the Gene Expression Omnibus repository, https://www.ncbi.nlm.nih.gov/geo/query/acc.cgi?acc=GSE120937.
