# Supplementary material for: Connectivity mapping of angiotensin-PPAR interactions involved in the amelioration of non-alcoholic steatohepatitis by Telmisartan
Source: Sci Rep. 2019 Mar 8;9:4003. doi: 10.1038/s41598-019-40322-1 (PMC6408578; doi:10.1038/s41598-019-40322-1)
Supplement: Supplementary file 1 — Supplementary Information [file 41598_2019_40322_MOESM1_ESM.pdf]

Connectivity mapping of angiotensin-PPAR interactions involved in the amelioration of non-alcoholic steatohepatitis by Telmisartan

Jung Gyu Park[1], Jong Soo Mok[2], Young In Han[3], Tae Sub Park[2,3], Keon Wook Kang[4], Cheol Soo Choi[5,6], Hee Dong Park[1], Joonghoon Park[2,3*]

[1]LG Chem R&D Campus, Korea

[2]Graduate School of International Agricultural Technology, Seoul National University, Korea

[3]Institute of Green Bio Science and Technology, Seoul National University, Korea

[4]College of pharmacy, Seoul National University, Korea

[5]Korea mouse metabolic phenotyping center, Lee Gil Ya cancer and diabetes institute, Gachon University School of Medicine, Republic of Korea.

[6]Endocrinology, Internal Medicine, Gachon University Gil Medical Center, Republic of Korea.

*Correspondence: **joonghoon@snu.ac.kr**

Supplementary Information

Supplementary Tables

Supplementary Table 1. NAFLD activity score

Supplementary Table 2. Normalized probeset

Supplementary Table 3. Differentially expressed genes by telmisartan in STAM mice liver

Supplementary Table 4. Gene set enrichment analysis of telmisartan-induced DEGs

Supplementary Table 5. Chemical perturbagens connected with telmisartan-induced DEGs

Supplementary Table 6. Strongly paired genetic pertubagens connected with telmisartan-treated DEGs

Supplementary Table 7. ChIP-X enrichment analysis of transcription factors to telmisartan-induced DEGs

Supplementary Table 8. Primer information for qRT-PCR

Supplementary Information

Supplementary Figure

Supplementary Figure 1. Uncropped Western blots with original markings

Full scanned 9% SDS-PAGE gels for Western blots of PPARδ (a), PPARα (b), RELA (c) and ACTB (d). Some gel images were cropped to merge with size marker while removing samples unrelated to study which were ran on the same gel. Black dashed boxes indicate cropped region shown in respective figure. Ctrl: vehicle control, Tel: telmisartan at 10 µM, Pal: palmitate at 0.2 mM.

Supplementary Table 1. NAFLD activity score

Group	No. of animals	Score											NAS (mean ± SD)		
		Steatosis				Lobular inflammation				Hepatocyte ballooning					
		0	1	2	3	0	1	2	3	0	1	2			
Control	7	-	7	-	-	-	-	5	2	-	4	3	4.7	±	0.76
Telmisartan	7	1	6	-	-	1	4	2	-	1	5	1	3.0	±	0.82

Definition of NAS Components		
Item	Score	Extent
Steatosis	0	<5%
	1	5-33%
	2	>33-66%
	3	>66%
Lobular Inflammation	0	No foci
	1	<2 foci/200x
	2	2-4 foci/200x
	3	>4 foci/200x
HepatocyteBallooning	0	None
	1	Few balloon cells
	2	Many cells/prominent ballooning

Supplementary Table 2. Normalized probeset

Probeset ID	Gene Symbol	Gene Description	Ctrl_01	Ctrl_02	Ctrl_03	Tel_01	Tel_02	Tel_03
17478785	Gm7367	1110014K08Rik pseudogene	13.53366	11.85275	13.14471	11.50086	10.46921	12.48125
17336636	Agpat1	1-acylglycerol-3-phosphate O-acyltransferase 1 (lysophosphatidic acid acyltransfer	15.55277	18.62954	21.10832	18.62954	17.28885	18.62954
17383104	Agpat2	1-acylglycerol-3-phosphate O-acyltransferase 2 (lysophosphatidic acid acyltransfer	267.0433	204.0756	179.556	231.0258	201.7955	199.639
17242474	Agpat3	1-acylglycerol-3-phosphate O-acyltransferase 3	268.6399	267.4805	261.7707	244.761	246.3917	245.8271
17333222	Agpat4	1-acylglycerol-3-phosphate O-acyltransferase 4 (lysophosphatidic acid acyltransfer	3.804245	4.270663	5.179777	5.455419	5.324441	4.004217
17499560	Agpat5	1-acylglycerol-3-phosphate O-acyltransferase 5 (lysophosphatidic acid acyltransfer	20.78787	19.42873	19.00767	19.42873	16.31956	17.90251
17439622	Agpat9	1-acylglycerol-3-phosphate O-acyltransferase 9	50.31233	50.31233	53.92155	50.31233	39.12747	34.98077
17388499	Accs	1-aminocyclopropane-1-carboxylate synthase (non-functional)	6.766313	6.981127	7.698994	8.457844	8.410141	8.395347
17388522	Accsl	1-aminocyclopropane-1-carboxylate synthase (non-functional)-like	4.08303	3.972535	2.809708	2.600141	3.166129	3.106801
17457171	Bpgm	2,3-bisphosphoglycerate mutase	35.68485	29.32156	29.32156	29.32156	19.91895	24.48914
17256404	Cnp	2,3-cyclic nucleotide 3 phosphodiesterase	11.24366	9.52527	9.40384	9.730406	9.213696	10.02368
17423463	Decr1	2,4-dienoyl CoA reductase 1, mitochondrial	152.694	151.8655	144.0874	134.2176	124.4455	126.441
17416325	Dhcr24	24-dehydrocholesterol reductase	892.4639	1115.945	1128.679	991.0541	941.0255	816.7892
17342581	Decr2	2-4-dienoyl-Coenzyme A reductase 2, peroxisomal	847.4298	906.4917	846.1268	851.9116	783.4243	808.8621
17452126	Oas1a	2-5 oligoadenylate synthetase 1A	7.85483	4.872189	6.552929	9.960513	5.48933	5.911278
17441759	Oas1b	2-5 oligoadenylate synthetase 1B	5.963916	5.364598	7.814535	9.085967	7.814535	8.433667
17452097	Oas1c	2-5 oligoadenylate synthetase 1C	4.52137	4.146287	4.542291	4.64066	2.782767	4.619735
17441788	Oas1d	2-5 oligoadenylate synthetase 1D	3.062691	3.263862	2.659804	2.659804	3.295255	3.010681
17452089	Oas1e	2-5 oligoadenylate synthetase 1E	3.990197	4.245075	4.072063	3.147726	4.269952	3.270407
17441768	Oas1f	2-5 oligoadenylate synthetase 1F	2.802841	3.401733	4.302872	4.767986	3.513844	3.592355
17452115	Oas1g	2-5 oligoadenylate synthetase 1G	7.53376	15.196	8.185894	8.581798	12.8405	10.17121
17441777	Oas1h	2-5 oligoadenylate synthetase 1H	2.177541	2.351221	2.391209	1.907866	2.403705	2.307339
17452054	Oas2	2-5 oligoadenylate synthetase 2	17.22629	27.11219	40.00227	32.34722	26.42644	28.98832
17452070	Oas3	2-5 oligoadenylate synthetase 3	15.70716	19.71798	18.62954	21.92812	17.58435	18.64452
17441051	Oasl1	2-5 oligoadenylate synthetase-like 1	26.64975	23.59043	31.50655	34.57051	26.96115	33.8762
17441037	Oasl2	2-5 oligoadenylate synthetase-like 2	11.78271	14.64331	22.72377	16.23595	14.64331	14.55164
17548963	Gm17764	28S ribosomal protein S14, mitochondrial-like	12.92973	14.38876	14.1656	14.6214	12.38309	12.7511
17241643	Ado	2-aminoethanethiol (cysteamine) dioxygenase	34.9127	27.7957	22.77675	26.703	30.97731	28.09186
17296548	Tcstv1	2-cell-stage, variable group, member 1	2.265674	2.307739	1.726009	1.715346	2.266145	1.941901
17290211	Tcstv3	2-cell-stage, variable group, member 3	5.015835	3.214691	2.411683	3.07906	4.562486	3.673767
17338118	Dnph1	2-deoxynucleoside 5-phosphate N-hydrolase 1	25.55671	27.14341	26.94377	28.99295	27.18088	29.96113
17463898	Dera	2-deoxyribose-5-phosphate aldolase homolog (C. elegans)	106.9416	97.89478	84.54749	107.511	80.90125	100.2856
17304794	Hacl1	2-hydroxyacyl-CoA lyase 1	542.177	591.3711	657.2325	659.1258	581.1553	384.6333
17503910	Ogfod1	2-oxoglutarate and iron-dependent oxygenase domain containing 1	42.32593	40.7817	41.04942	41.09383	43.45981	39.93301
17442464	Ogfod2	2-oxoglutarate and iron-dependent oxygenase domain containing 2	24.9191	20.58812	21.84846	20.99567	17.33745	21.1615
17273485	Ogfod3	2-oxoglutarate and iron-dependent oxygenase domain containing 3	29.29312	28.93493	27.09867	24.10934	27.89124	27.41721
17529871	Pxylp1	2-phosphoxylose phosphatase 1	17.09734	16.23595	18.33871	19.08583	17.62523	19.22325
17488076	LOC101055953	39S ribosomal protein L41, mitochondrial-like	3.654236	5.689295	6.992896	3.170785	5.360286	4.265604
17419980	Hmgcl	3-hydroxy-3-methylglutaryl-Coenzyme A lyase	261.4969	309.4645	270.1712	268.4017	272.9249	267.2878
17295233	Hmgcr	3-hydroxy-3-methylglutaryl-Coenzyme A reductase	80.60738	57.27803	87.4781	48.03153	83.67125	47.88958
17290173	Hmgcs1	3-hydroxy-3-methylglutaryl-Coenzyme A synthase 1	514.0839	358.1053	448.508	349.5076	474.0975	296.3771
17400862	Hmgcs2	3-hydroxy-3-methylglutaryl-Coenzyme A synthase 2	2976.993	3434.109	3376.681	3029.744	2700.311	2724.602
17381892	Hacd1	3-hydroxyacyl-CoA dehydratase 1	16.27426	17.18538	17.4474	15.82781	14.29917	14.03016
17325198	Hacd2	3-hydroxyacyl-CoA dehydratase 2	280.2771	330.0956	382.1831	285.0162	298.333	302.2168
17528229	Hacd3	3-hydroxyacyl-CoA dehydratase 3	241.3562	267.21	299.0121	269.5576	267.21	239.1928
17427051	Hacd4	3-hydroxyacyl-CoA dehydratase 4	6.109228	5.26298	5.853096	5.628537	4.113282	4.298103
17347650	Haao	3-hydroxyanthranilate 3,4-dioxygenase	459.471	376.2949	396.6465	436.8608	467.7812	483.7608
17324643	Bdh1	3-hydroxybutyrate dehydrogenase, type 1	171.2084	186.5036	163.2705	154.3862	145.3039	164.4836
17402846	Bdh2	3-hydroxybutyrate dehydrogenase, type 2	15.99834	18.02528	13.63951	15.94011	12.24508	15.76376
17466908	Hibadh	3-hydroxyisobutyrate dehydrogenase	391.5628	409.4973	360.5286	404.8911	365.5969	491.6814
17466919	Hibadh	3-hydroxyisobutyrate dehydrogenase	11.55255	11.55255	7.719246	11.50086	10.39156	8.952248
17212792	Hibch	3-hydroxyisobutyryl-Coenzyme A hydrolase	121.8219	130.5718	127.9282	142.1441	97.70564	145.4007
17519550	Hmgcll1	3-hydroxymethyl-3-methylglutaryl-Coenzyme A lyase-like 1	8.993982	6.76275	7.060205	6.781103	6.117448	6.991393
17226049	Kdsr	3-ketodihydrosphingosine reductase	73.65186	104.0873	104.9218	91.87306	68.74534	76.72208
17309802	Oxct1	3-oxoacid CoA transferase 1	11.32815	11.96277	11.8576	11.85842	11.96277	14.8062
17429685	Oxct2a	3-oxoacid CoA transferase 2A	2.769758	3.339703	3.009972	3.339703	3.56497	3.146221
17418154	Oxct2b	3-oxoacid CoA transferase 2B	2.798454	5.314043	3.139054	3.299026	3.546092	2.314836
17303599	Oxsm	3-oxoacyl-ACP synthase, mitochondrial	13.18522	13.30116	13.07773	14.13187	13.39423	15.482
17402681	Papss1	3-phosphoadenosine 5-phosphosulfate synthase 1	36.37865	26.68709	27.14473	27.14473	30.96679	24.94433
17358690	Papss2	3-phosphoadenosine 5-phosphosulfate synthase 2	441.6715	493.8024	666.3748	589.8564	400.2645	593.4571
17408273	Phgdh	3-phosphoglycerate dehydrogenase	12.60068	12.7106	10.09935	12.86384	17.4905	11.74366
17349803	Gm6756	3-phosphoglycerate dehydrogenase pseudogene	2.914705	2.940701	4.021629	4.130367	2.621695	3.140621
17508568	Gm8096	3-phosphoglycerate dehydrogenase pseudogene	6.46455	6.779505	8.67095	8.286139	7.854612	8.376398
17341690	Pdpk1	3-phosphoinositide dependent protein kinase 1	77.46163	77.65194	71.79592	71.79592	61.47466	64.07812
17313427	LOC102634709	40S ribosomal protein S14	16.68766	10.93878	17.98348	13.97504	17.67086	21.06447
17524967	Gm6607	40S ribosomal protein S20 pseudogene	2.67402	2.770065	2.893027	3.880143	2.68751	2.827329
17547978	LOC102638850	40S ribosomal protein S21 pseudogene	60.47974	62.68464	69.98384	66.01171	60.20619	63.5945
17325933	LOC102641930	40S ribosomal protein S24-like	3.381258	3.883991	3.040334	2.927826	3.263862	3.659001
17349588	LOC102638785	40S ribosomal protein S25	3.685448	2.627717	3.420293	3.356854	4.205799	2.968636
17512752	LOC102642963	40S ribosomal protein S26-like	215.9106	242.6428	329.4099	244.3559	270.13	251.562
17440754	Gm3511	40S ribosomal protein S28 pseudogene	129.9678	175.7877	229.4538	168.8841	231.4409	182.2127
17512474	LOC105243270	40S ribosomal protein S2-like	1498.476	1476.064	1623.577	1535.891	1682.476	1730.34
17358223	LOC102638448	40S ribosomal protein S7 pseudogene	1024.284	971.2709	935.8607	975.4067	1004.993	1105.007
17322750	Abat	4-aminobutyrate aminotransferase	488.0787	463.8646	444.0043	503.9057	419.1811	495.7607
17510264	Haus8	4HAUS augmin-like complex, subunit 8	15.83637	19.16059	17.23596	16.7586	12.39348	15.83637
17359504	Hoga1	4-hydroxy-2-oxoglutarate aldolase 1	120.9578	153.0946	136.7095	119.6677	136.4274	135.673
17428731	Hpdl	4-hydroxyphenylpyruvate dioxygenase-like	5.009199	5.672835	5.187107	4.366811	4.72587	6.212009
17452578	Hpd	4-hydroxyphenylpyruvic acid dioxygenase	2483.691	2736.147	2775.304	2874.071	2624.724	3057.125
17246860	Nipsnap1	4-nitrophenylphosphatase domain and non-neuronal SNAP25-like protein homolo	239.0022	229.8964	222.725	237.2444	218.1036	250.4467
17238199	Sdr9c7	4short chain dehydrogenase/reductase family 9C, member 7	49.92394	30.08516	20.55561	28.29451	24.40424	22.40921
17351168	Htr4	5 hydroxytryptamine (serotonin) receptor 4	3.850538	4.044777	5.003335	4.240928	3.993933	5.213673

17520073	Nt5e	5 nucleotidase, ecto	34.15394	34.11986	59.25151	36.95865	39.96236	41.26481
17520177	Mthfs	5, 10-methenyltetrahydrofolate synthetase	7.576754	7.828359	7.811123	9.46131	9.013891	8.334179
17529569	Mthfsl	5, 10-methenyltetrahydrofolate synthetase-like	133.7662	115.3721	113.2	120.7149	153.1626	134.4448
17421550	Mthfr	5,10-methylenetetrahydrofolate reductase	33.98839	23.45608	22.06197	23.02102	25.56645	19.23631
17271911	Nt5c	5,3-nucleotidase, cytosolic	62.3795	70.49456	66.2317	69.43254	73.82359	74.57809
17250307	Nt5m	5,3-nucleotidase, mitochondrial	14.57749	14.31296	15.90568	17.00244	12.51043	12.65864
17520492	Xrn1	5-3 exoribonuclease 1	34.02893	34.63791	32.12757	35.30596	38.82382	31.88531
17377283	Xrn2	5-3 exoribonuclease 2	56.54039	51.60075	54.99887	58.01041	59.61031	55.47785
17213990	Atic	5-aminoimidazole-4-carboxamide ribonucleotide formyltransferase/IMP cyclohydrc	35.7148	48.6322	39.15705	37.78024	33.11424	37.76061
17522848	Azi2	5-azacytidine induced gene 2	43.91829	34.68476	41.73917	38.5912	49.33228	44.2161
17460603	Hmces	5-hydroxymethylcytosine (hmC) binding, ES cell specific	10.5291	14.99802	12.00572	11.66965	11.56746	11.56746
17289656	Htr1a	5-hydroxytryptamine (serotonin) receptor 1A	3.334568	3.168676	3.336695	3.720483	3.728305	3.252322
17529218	Htr1b	5-hydroxytryptamine (serotonin) receptor 1B	4.743559	5.48933	5.48933	4.606401	5.48933	6.035319
17420113	Htr1d	5-hydroxytryptamine (serotonin) receptor 1D	7.186205	6.597594	8.145538	7.085109	6.918983	6.714407
17331431	Htr1f	5-hydroxytryptamine (serotonin) receptor 1F	3.407822	2.991668	3.229319	2.991668	3.133381	2.939641
17301926	Htr2a	5-hydroxytryptamine (serotonin) receptor 2A	6.393164	7.105273	5.726882	6.891414	6.062698	6.615224
17225138	Htr2b	5-hydroxytryptamine (serotonin) receptor 2B	4.503667	5.775116	4.639406	5.332566	4.288818	5.075078
17538411	Htr2c	5-hydroxytryptamine (serotonin) receptor 2C	2.76389	3.279919	3.110141	2.501373	2.722685	2.915615
17526717	Htr3a	5-hydroxytryptamine (serotonin) receptor 3A	4.555218	5.308153	4.841654	4.776123	4.626624	5.55921
17526729	Htr3b	5-hydroxytryptamine (serotonin) receptor 3B	7.062113	6.697673	6.182799	7.419233	5.053875	5.064957
17435570	Htr5a	5-hydroxytryptamine (serotonin) receptor 5A	8.663981	10.78138	7.103204	6.384705	6.732956	9.050202
17226364	Htr5b	5-hydroxytryptamine (serotonin) receptor 5B	3.793002	6.069384	4.654996	4.816783	3.75442	5.323223
17431853	Htr6	5-hydroxytryptamine (serotonin) receptor 6	7.80728	5.669769	5.369359	5.893644	4.923281	6.466926
17364169	Htr7	5-hydroxytryptamine (serotonin) receptor 7	3.759029	3.985774	3.99745	3.757205	3.486501	3.34647
17290565	Mtr	5-methyltetrahydrofolate-homocysteine methyltransferase	21.17882	21.68101	18.39625	19.6908	13.00325	23.56412
17294066	Mtrr	5-methyltetrahydrofolate-homocysteine methyltransferase reductase	19.03496	20.36486	21.05236	20.78067	19.28517	20.27261
17240199	Nt5dc1	5-nucleotidase domain containing 1	50.37863	53.17403	44.92697	50.67984	43.4297	50.52201
17298364	Nt5dc2	5-nucleotidase domain containing 2	15.32102	21.97605	27.68591	17.69414	16.38041	13.96061
17236227	Nt5dc3	5-nucleotidase domain containing 3	31.54479	24.62326	19.13696	17.63965	21.90897	23.82443
17418168	Nt5c1a	5-nucleotidase, cytosolic IA	7.325159	6.560474	6.836415	7.519465	7.401358	6.687004
17274031	Nt5c1b	5-nucleotidase, cytosolic IB	3.489814	3.429409	3.237365	3.415673	3.485873	3.634306
17365421	Nt5c2	5-nucleotidase, cytosolic II	39.71577	54.80061	66.48606	41.16568	42.69455	42.95458
17467128	Nt5c3	5-nucleotidase, cytosolic III	24.44758	31.51223	25.95265	25.73286	29.03598	26.12067
17269498	Nt5c3b	5-nucleotidase, cytosolic IIIB	7.988751	8.345567	7.814535	7.814535	6.134858	6.850503
17318428	Oplah	5-oxoprolinase (ATP-hydrolysing)	227.1214	235.8719	220.0199	225.5416	179.4154	165.7621
17249328	Phykpl	5-phosphohydroxy-L-lysine phospholyase	26.89862	26.00992	30.10122	29.29799	24.21585	29.38504
17388099	LOC102632821	60S acidic ribosomal protein P1-like	9.302574	8.79571	8.467112	10.74706	7.972156	9.689619
17548211	LOC102632821	60S acidic ribosomal protein P1-like	17.80944	14.89597	14.13079	21.66073	12.99018	16.57124
17548369	LOC102637947	60S acidic ribosomal protein P1-like	36.23595	37.37522	30.60319	37.75642	38.34243	40.15367
17548373	LOC102637947	60S acidic ribosomal protein P1-like	42.70639	43.65122	38.76116	42.43492	39.11093	43.19613
17548597	LOC102641848	60S ribosomal protein L15-like	253.9604	228.5174	192.5608	208.6281	202.5354	208.6281
17352102	Gm6133	60S ribosomal protein L17 pseudogene	18.4099	19.28036	22.58229	27.89452	21.26811	20.89332
17476693	LOC102638521	60S ribosomal protein L23a	9.793944	6.996096	6.474864	7.867199	4.272469	9.074512
17548840	LOC102636101	60S ribosomal protein L39 pseudogene	5.765837	5.871585	5.906649	6.596085	5.763	6.082692
17286228	LOC105245385	60S ribosomal protein L7a-like	61.20171	64.80261	70.50482	80.67389	58.0933	79.97072
17538590	Pfkfb1	6-phosphofructo-2-kinase/fructose-2,6-biphosphatase 1	60.37712	64.32288	88.41321	92.23676	107.3531	96.72655
17226676	Pfkfb2	6-phosphofructo-2-kinase/fructose-2,6-biphosphatase 2	24.1381	26.6215	27.36045	31.89238	28.73705	30.63502
17381630	Pfkfb3	6-phosphofructo-2-kinase/fructose-2,6-biphosphatase 3	16.89624	23.42781	18.65874	16.73156	19.03615	14.08951
17522110	Pfkfb4	6-phosphofructo-2-kinase/fructose-2,6-biphosphatase 4	5.75055	6.72032	7.315315	5.592138	5.888948	5.985451
17502249	Pgls	6-phosphogluconolactonase	43.5014	39.8299	49.63583	52.17729	42.55244	50.85508
17526813	Pts	6-pyruvoyl-tetrahydropterin synthase	49.19876	57.04387	59.01705	51.293	58.35644	62.68333
17485357	Dhcr7	7-dehydrocholesterol reductase	67.54514	63.61478	89.69589	83.67763	88.62796	71.52571
17461625	Ogg1	8-oxoguanine DNA-glycosylase 1	9.248119	14.14657	13.52644	15.88849	15.15508	11.73048
17519088	Adam10	a disintegrin and metallopeptidase domain 10	95.05035	88.58927	96.61665	84.23571	81.36125	78.36894
17256991	Adam11	a disintegrin and metallopeptidase domain 11	12.44798	17.1755	26.61062	16.77522	13.79793	13.0674
17497246	Adam12	a disintegrin and metallopeptidase domain 12 (meltrin alpha)	5.433644	5.433644	5.433644	5.433644	5.94353	5.672678
17407017	Adam15	a disintegrin and metallopeptidase domain 15 (metargidin)	11.16375	15.19126	14.93743	12.29739	11.99	12.97929
17280207	Adam17	a disintegrin and metallopeptidase domain 17	44.09307	36.08482	36.30132	40.65188	37.15437	36.21888
17508202	Adam18	a disintegrin and metallopeptidase domain 18	3.649857	3.158107	3.304541	3.816526	3.715046	4.077599
17248754	Adam19	a disintegrin and metallopeptidase domain 19 (meltrin beta)	4.27638	5.963503	4.629544	4.03095	5.024604	5.085607
17452204	Adam1a	a disintegrin and metallopeptidase domain 1a	4.057834	4.684516	3.194345	4.281926	6.32388	4.638409
17452201	Adam1b	a disintegrin and metallopeptidase domain 1b	2.908812	4.047132	2.823276	4.083001	4.615158	4.038137
17307815	Adam2	a disintegrin and metallopeptidase domain 2	3.433405	3.370167	3.880762	3.668576	3.668576	3.078783
17500707	Adam20	a disintegrin and metallopeptidase domain 20	2.543562	2.454212	3.350114	2.304206	2.804163	2.220785
17282363	Adam21	a disintegrin and metallopeptidase domain 21	3.613307	3.150695	3.959285	3.873452	3.871708	4.291107
17445467	Adam22	a disintegrin and metallopeptidase domain 22	3.270633	3.341486	3.342228	3.97857	3.748673	3.024905
17213608	Adam23	a disintegrin and metallopeptidase domain 23	45.19809	46.26598	59.11736	49.55745	47.17989	25.02174
17500697	Adam24	a disintegrin and metallopeptidase domain 24 (testase 1)	4.628357	4.203439	4.036878	3.864093	3.624822	3.843285
17500702	Adam25	a disintegrin and metallopeptidase domain 25 (testase 2)	2.44103	2.42871	1.972465	1.898013	2.759544	2.346906
17509005	Adam26a	a disintegrin and metallopeptidase domain 26A (testase 3)	2.406006	2.285991	1.898013	1.982183	2.285991	2.285991
17509001	Adam26b	a disintegrin and metallopeptidase domain 26B	3.11096	2.386093	2.209822	2.574853	2.763008	2.60189
17308099	Adam28	a disintegrin and metallopeptidase domain 28	4.440124	4.165819	4.291316	3.761583	3.359299	3.959724
17509428	Adam29	a disintegrin and metallopeptidase domain 29	2.473116	2.393945	2.335902	2.707047	2.573693	2.722364
17508224	Adam3	a disintegrin and metallopeptidase domain 3 (cyritestin)	4.75795	3.962827	4.447609	3.286268	5.118177	4.311291
17400849	Adam30	a disintegrin and metallopeptidase domain 30	3.062691	2.462203	2.400425	2.681462	2.919599	2.400425
17508278	Adam32	a disintegrin and metallopeptidase domain 32	2.822708	3.457981	3.076628	3.40395	2.920388	2.933474
17391803	Adam33	a disintegrin and metallopeptidase domain 33	8.953179	9.52362	10.34413	7.583795	8.403673	9.58707
17509013	Adam34	a disintegrin and metallopeptidase domain 34	2.974238	2.919862	2.587843	2.664565	2.440505	2.284724
17500712	Adam39	a disintegrin and metallopeptidase domain 39	3.024968	2.391026	3.339703	2.957524	3.723992	3.565341
17282343	Adam4	a disintegrin and metallopeptidase domain 4	2.705003	3.249573	3.241519	5.573602	3.491604	3.037175
17508253	Adam5	a disintegrin and metallopeptidase domain 5	2.968636	2.51809	2.782767	2.6815	2.782767	2.782767
17279542	Adam6a	a disintegrin and metallopeptidase domain 6A	3.874956	3.811344	3.113527	4.271344	3.860105	3.504333
17279539	Adam6b	a disintegrin and metallopeptidase domain 6B	2.212182	2.239691	1.884472	2.667374	2.239691	2.14716
17308058	Adam7	a disintegrin and metallopeptidase domain 7	3.211217	2.862846	2.587125	2.838148	3.374234	2.943291

17497525	Adam8	a disintegrin and metallopeptidase domain 8	4.86136	4.937155	3.723011	4.741258	5.445294	4.655932
17508300	Adam9	a disintegrin and metallopeptidase domain 9 (meltrin gamma)	133.8302	131.5703	132.3845	115.404	114.7396	109.4183
17331705	Adamts1	a disintegrin-like and metallopeptidase (reprolysin type) with thrombospondin typ	27.22028	22.29456	27.25899	24.40052	23.81618	22.83476
17336086	Adamts10	a disintegrin-like and metallopeptidase (reprolysin type) with thrombospondin typ	11.78933	12.30472	12.10961	11.28958	12.55056	10.39833
17310368	Adamts12	a disintegrin-like and metallopeptidase (reprolysin type) with thrombospondin typ	8.349848	7.260556	9.079797	7.15891	7.15891	6.319016
17368422	Adamts13	a disintegrin-like and metallopeptidase (reprolysin type) with thrombospondin typ	12.35189	11.13863	9.787565	11.37883	8.958828	12.31487
17241181	Adamts14	a disintegrin-like and metallopeptidase (reprolysin type) with thrombospondin typ	10.81926	14.03736	12.53708	12.39048	12.53708	12.38739
17525224	Adamts15	a disintegrin-like and metallopeptidase (reprolysin type) with thrombospondin typ	7.473997	7.097003	6.826802	6.478815	5.619298	6.489422
17294187	Adamts16	a disintegrin-like and metallopeptidase (reprolysin type) with thrombospondin typ	6.279806	7.673421	7.529113	5.978444	6.153341	8.01732
17479041	Adamts17	a disintegrin-like and metallopeptidase (reprolysin type) with thrombospondin typ	10.32514	10.7052	10.09165	10.63509	11.39738	11.94289
17513240	Adamts18	a disintegrin-like and metallopeptidase (reprolysin type) with thrombospondin typ	3.225382	3.742286	3.596566	3.596566	3.940667	4.031146
17350876	Adamts19	a disintegrin-like and metallopeptidase (reprolysin type) with thrombospondin typ	5.637361	4.509352	4.699607	4.865405	4.849278	5.689848
17249206	Adamts2	a disintegrin-like and metallopeptidase (reprolysin type) with thrombospondin typ	19.04777	21.43624	18.10655	22.44621	17.85059	23.5973
17320737	Adamts20	a disintegrin-like and metallopeptidase (reprolysin type) with thrombospondin typ	3.623616	3.911874	4.42467	3.961893	3.961893	3.961893
17449488	Adamts3	a disintegrin-like and metallopeptidase (reprolysin type) with thrombospondin typ	4.240223	3.660473	3.403408	3.722418	4.70003	3.722418
17219248	Adamts4	a disintegrin-like and metallopeptidase (reprolysin type) with thrombospondin typ	6.528538	6.429159	8.873234	6.221662	6.458325	5.226735
17331720	Adamts5	a disintegrin-like and metallopeptidase (reprolysin type) with thrombospondin typ	5.625885	6.010646	5.184878	5.884745	5.990889	5.08388
17289602	Adamts6	a disintegrin-like and metallopeptidase (reprolysin type) with thrombospondin typ	8.318826	12.19662	15.18284	10.17911	9.764729	10.50679
17520254	Adamts7	a disintegrin-like and metallopeptidase (reprolysin type) with thrombospondin typ	8.518444	7.698994	6.922773	7.4755	6.170982	7.698994
17515703	Adamts8	a disintegrin-like and metallopeptidase (reprolysin type) with thrombospondin typ	5.192359	4.951995	4.623835	4.689389	5.425214	4.975391
17469136	Adamts9	a disintegrin-like and metallopeptidase (reprolysin type) with thrombospondin typ	14.56264	21.542	18.19538	16.24969	14.75007	17.23596
17231423	Akap12	A kinase (PRKA) anchor protein (gravin) 12	15.88188	16.89717	16.15815	18.32181	18.10342	19.06877
17434414	Akap9	A kinase (PRKA) anchor protein (yotiao) 9	67.22798	67.22798	64.01642	61.2208	67.22798	64.41523
17267584	Akap1	A kinase (PRKA) anchor protein 1	158.2136	158.3233	154.5607	149.9606	166.3053	143.2062
17263929	Akap10	A kinase (PRKA) anchor protein 10	22.47766	24.08762	26.38288	26.54411	28.51027	24.28847
17308829	Akap11	A kinase (PRKA) anchor protein 11	39.33332	46.11678	50.9507	46.88615	50.84838	55.23229
17479205	Akap13	A kinase (PRKA) anchor protein 13	85.02764	88.99054	80.78077	73.3828	80.78077	79.02187
17541028	Akap14	A kinase (PRKA) anchor protein 14	5.769795	5.426267	4.496336	5.759206	5.719504	5.69936
17540950	Akap17b	A kinase (PRKA) anchor protein 17B	14.6554	9.575315	6.572535	8.670829	9.862272	9.616154
17414364	Akap2	A kinase (PRKA) anchor protein 2	16.39382	14.65052	15.46248	13.49553	14.48663	15.24992
17463308	Akap3	A kinase (PRKA) anchor protein 3	2.663512	3.263862	2.940497	3.009972	3.269727	2.88936
17532786	Akap4	A kinase (PRKA) anchor protein 4	5.146851	5.48933	5.129684	4.672064	5.48933	5.252412
17276552	Akap5	A kinase (PRKA) anchor protein 5	19.50457	20.22854	18.81746	20.22307	19.75875	23.0132
17275436	Akap6	A kinase (PRKA) anchor protein 6	3.650793	3.945177	3.945177	3.816499	3.521964	4.283648
17239856	Akap7	A kinase (PRKA) anchor protein 7	15.27584	20.54549	19.57052	15.61175	16.167	22.29441
17343371	Akap8	A kinase (PRKA) anchor protein 8	36.88008	49.39284	38.64286	45.49512	48.16356	40.23279
17343387	Akap8l	A kinase (PRKA) anchor protein 8-like	24.82772	37.5669	34.15275	37.5669	32.71408	41.90353
17481684	Akip1	A kinase (PRKA) interacting protein 1	42.90518	43.23986	48.51873	35.68807	35.35029	36.00561
17378628	Aar2	AAR2 splicing factor homolog (S. cerevisiae)	23.82196	22.41636	25.83079	26.0628	23.29454	19.55438
17277697	Adck1	aarF domain containing kinase 1	21.79114	28.73287	25.45382	28.15326	24.02859	24.92307
17457524	Adck2	aarF domain containing kinase 2	11.84889	9.79829	12.46698	13.13787	11.84889	12.85965
17230408	Adck3	aarF domain containing kinase 3	170.3727	146.1322	108.3376	193.2695	135.7718	184.8562
17475542	Adck4	aarF domain containing kinase 4	29.15073	34.15678	33.31662	32.4593	33.66272	32.00244
17312511	Adck5	aarF domain containing kinase 5	93.204	89.26607	83.05874	86.0677	63.2139	59.32466
17231973	Ahi1	Abelson helper integration site 1	5.347483	5.347483	5.193294	6.268846	5.037821	5.38367
17435946	Abhd1	abhydrolase domain containing 1	7.544838	12.71192	13.86033	13.86033	8.725392	14.5691
17330692	Abhd10	abhydrolase domain containing 10	9.590393	9.516031	8.300395	9.778072	14.70271	10.70572
17443154	Abhd11	abhydrolase domain containing 11	43.01447	39.83101	35.28199	43.87478	47.3404	41.49168
17453506	Abhd11os	abhydrolase domain containing 11, opposite strand	14.52	14.11845	14.92822	16.99608	16.79582	16.08529
17392730	Abhd12	abhydrolase domain containing 12	37.19482	31.19753	40.17065	40.3294	37.58799	37.66885
17275986	Abhd12b	abhydrolase domain containing 12B	6.171709	6.480073	5.997204	6.491537	6.768612	7.759157
17498892	Abhd13	abhydrolase domain containing 13	32.65156	32.65156	26.38873	34.78106	27.0465	32.65156
17530686	Abhd14a	abhydrolase domain containing 14A	13.83403	13.42007	13.42007	13.72087	15.70814	13.45873
17521211	Abhd14b	abhydrolase domain containing 14b	196.5388	190.881	197.6398	182.8897	204.7061	184.6142
17253297	Abhd15	abhydrolase domain containing 15	29.7317	30.60361	41.95585	39.14131	39.19207	42.51087
17336939	Abhd16a	abhydrolase domain containing 16A	32.66831	42.7209	39.90451	34.58777	48.70068	42.49968
17381039	Abhd16b	abhydrolase domain containing 16B	3.843179	3.703322	4.072797	4.092578	3.650211	4.05036
17243014	Abhd17a	abhydrolase domain containing 17A	45.5535	35.94627	37.60571	39.09521	41.6643	44.2842
17358132	Abhd17b	abhydrolase domain containing 17B	35.89647	36.74357	37.55596	44.95657	33.96672	40.11973
17492978	Abhd17c	abhydrolase domain containing 17C	172.164	153.8969	163.4868	146.0248	160.4511	145.951
17479354	Abhd2	abhydrolase domain containing 2	158.6452	242.7521	309.6726	170.1649	251.9459	166.1992
17352680	Abhd3	abhydrolase domain containing 3	137.6328	141.5374	128.4925	123.3086	111.0565	115.6619
17300251	Abhd4	abhydrolase domain containing 4	137.9307	162.8726	148.2769	130.8464	112.3986	114.3482
17523416	Abhd5	abhydrolase domain containing 5	54.65532	47.68653	52.70727	52.2946	53.85506	45.65687
17297059	Abhd6	abhydrolase domain containing 6	154.6593	182.2191	211.5434	154.9899	113.9234	149.0053
17510311	Abhd8	abhydrolase domain containing 8	11.7832	13.54896	10.0218	12.05392	7.843296	12.04398
17268227	Abi3	ABI gene family, member 3	18.27855	18.27855	28.13609	18.66288	16.83988	13.20278
17326318	Abi3bp	ABI gene family, member 3 (NESH) binding protein	5.418224	7.343816	7.169189	6.321163	5.173426	6.753007
17382156	Abi1	abl-interactor 1	70.75401	72.4483	70.02946	66.20646	75.28184	73.28063
17213446	Abi2	abl-interactor 2	19.73603	18.99806	21.50932	14.68987	15.62149	16.35852
17383139	Abo	ABO blood group (transferase A, alpha 1-3-N-acetylgalactosaminyltransferase, trar	8.397035	7.698994	7.554572	8.313696	8.185724	7.698994
17239501	Abracl	ABRA C-terminal like	11.45376	8.321768	7.59179	6.886209	6.915243	8.731382
17240621	Aim1	absent in melanoma 1	36.14139	39.80368	49.84925	49.73743	44.44133	49.57325
17419669	Aim1l	absent in melanoma 1-like	6.791263	6.874048	8.225709	8.225709	8.225709	10.67571
17219643	Aim2	absent in melanoma 2	3.021343	3.292971	3.963321	4.326499	4.29322	3.88884
17442719	Aacs	acetoacetyl-CoA synthetase	37.93112	25.82775	31.60016	28.67417	34.18591	26.34231
17443523	Ache	acetylcholinesterase	12.33555	13.06568	10.56294	11.45384	11.62622	14.33222
17527123	Acat1	acetyl-Coenzyme A acetyltransferase 1	938.7974	941.1429	843.8425	818.4894	816.0818	802.8686
17340967	Acat2	acetyl-Coenzyme A acetyltransferase 2	43.569	35.86339	41.53052	38.97994	36.25681	46.72977
17340953	Acat3	acetyl-Coenzyme A acetyltransferase 3	164.7061	198.2394	163.9952	202.4245	173.1874	207.8429
17523042	Acaa1a	acetyl-Coenzyme A acyltransferase 1A	78.49452	89.21906	92.96785	104.6218	77.91936	88.46286
17532063	Acaa1b	acetyl-Coenzyme A acyltransferase 1B	473.5064	614.6537	570.1944	540.3162	365.26	430.1542
17351811	Acaa2	acetyl-Coenzyme A acyltransferase 2 (mitochondrial 3-oxoacyl-Coenzyme A thiola:	943.6186	945.0586	826.2531	912.4047	885.771	905.1543
17254395	Acaca	acetyl-Coenzyme A carboxylase alpha	82.38787	81.73658	85.70753	79.1902	89.05718	76.05125

17274027	Gm5182	acetyl-Coenzyme A carboxylase alpha pseudogene	1.981894	2.466457	2.732251	2.220785	2.188921	1.973218
17440826	Acacb	acetyl-Coenzyme A carboxylase beta	36.51557	44.36254	79.67416	51.93733	31.8968	31.96276
17539734	Asmt	acetylserotonin O-methyltransferase	20.13007	19.72277	18.07783	20.76209	21.32243	19.17241
17243839	Ascl1	achaete-scute complex homolog 1 (Drosophila)	3.016154	3.584023	2.961073	3.44468	2.976589	2.38003
17498188	Ascl2	achaete-scute complex homolog 2 (Drosophila)	4.800518	5.446729	5.033772	5.386134	4.775825	4.70192
17498193	Ascl2	achaete-scute complex homolog 2 (Drosophila)	3.149363	2.904057	2.856285	3.791556	3.88009	3.400437
17494926	Ascl3	achaete-scute complex homolog 3 (Drosophila)	3.97744	3.247726	3.718678	4.588237	3.956322	3.288592
17236150	Ascl4	achaete-scute complex homolog 4 (Drosophila)	4.916972	4.059663	5.0578	5.386134	4.645584	4.066084
17322200	Aaas	achalasia, adrenocortical insufficiency, alacrimia	29.91153	30.69454	30.53327	28.28274	29.41642	33.398
17280483	Acp1	acid phosphatase 1, soluble	56.67183	66.37693	59.16779	53.33387	74.09427	52.98896
17373222	Acp2	acid phosphatase 2, lysosomal	102.2729	99.93582	100.4943	87.75199	90.6346	80.88498
17524930	Acp5	acid phosphatase 5, tartrate resistant	94.07699	103.1336	90.02851	91.21948	101.4503	88.30174
17400759	Acp6	acid phosphatase 6, lysophosphatidic	43.73844	51.57107	44.48509	43.18026	42.91619	42.15962
17530406	Acpp	acid phosphatase, prostate	18.27577	25.74785	17.8792	18.27577	22.92596	14.98409
17490196	Acpt	acid phosphatase, testicular	10.78489	13.70956	12.17644	10.67504	10.8924	10.46507
17518172	Anp32a	acidic (leucine-rich) nuclear phosphoprotein 32 family, member A	42.56483	53.09853	52.18199	46.07652	52.21962	53.716
17413821	Anp32b	acidic (leucine-rich) nuclear phosphoprotein 32 family, member B	37.80749	41.27539	43.76968	41.68539	46.02577	34.34314
17400449	Anp32e	acidic (leucine-rich) nuclear phosphoprotein 32 family, member E	14.68142	12.70936	14.62009	14.06025	14.14814	13.38634
17231431	Armt1	acidic residue methyltransferase 1	13.47301	11.96219	11.46361	13.31518	10.48228	10.96178
17314855	Asic1	acid-sensing (proton-gated) ion channel 1	4.456342	5.34815	5.055662	5.103971	4.978634	4.978634
17266732	Asic2	acid-sensing (proton-gated) ion channel 2	3.731977	5.102343	3.911956	4.585344	3.856361	3.721618
17435335	Asic3	acid-sensing (proton-gated) ion channel 3	6.686633	8.492092	7.187749	6.171709	5.942912	6.171709
17214564	Asic4	acid-sensing (proton-gated) ion channel family member 4	11.24366	10.87626	11.19048	9.394589	10.3912	10.3912
17398441	Asic5	acid-sensing (proton-gated) ion channel family member 5	14.79564	15.07588	13.20672	14.8176	14.32149	14.69996
17274649	Adi1	acireductone dioxygenase 1	84.7364	91.00602	80.24002	83.61617	81.36125	78.35047
17412730	Aco1	aconitase 1	307.7127	284.0321	280.2771	284.9964	263.8474	261.361
17313394	Aco2	aconitase 2, mitochondrial	211.7444	266.2892	314.948	287.327	214.4846	279.9373
17314222	Acr	acrosin prepropeptide	6.613711	7.158364	7.398896	7.176299	6.819425	7.331915
17515998	Acrv1	acrosomal vesicle protein 1	3.201615	2.8896	3.163146	2.878725	2.957196	3.310112
17354831	Ablim3	actin binding LIM protein family, member 3	32.5404	39.10524	38.49155	51.32323	39.46905	36.09958
17436907	Afap1	actin filament associated protein 1	16.96877	15.50434	16.10269	15.48436	15.39127	15.95725
17354810	Afap1l1	actin filament associated protein 1-like 1	17.92846	15.40151	18.12778	22.59403	18.54386	16.54232
17365883	Afap1l2	actin filament associated protein 1-like 2	6.863812	7.024473	5.756891	5.30249	6.000114	5.855815
17444593	Arpc1a	actin related protein 2/3 complex, subunit 1A	84.60963	72.82389	73.77141	71.07646	73.92498	77.1471
17444609	Arpc1b	actin related protein 2/3 complex, subunit 1B	5.747601	8.67612	6.584619	5.419689	5.395176	6.32445
17214149	Arpc2	actin related protein 2/3 complex, subunit 2	484.358	489.21	450.7207	426.5526	448.2159	419.1259
17442096	Arpc3	actin related protein 2/3 complex, subunit 3	91.06722	116.3131	101.2944	96.8265	105.6757	113.9305
17461638	Arpc4	actin related protein 2/3 complex, subunit 4	111.2946	107.7915	106.8276	112.8905	127.49	111.2946
17218254	Arpc5	actin related protein 2/3 complex, subunit 5	69.44168	87.4587	77.24716	86.14708	80.17052	78.99229
17370511	Arpc5l	actin related protein 2/3 complex, subunit 5-like	34.87666	30.12084	30.44197	30.39474	31.02609	32.83949
17404515	Actrt3	actin related protein T3	8.845661	8.276782	7.954162	9.073505	9.675727	8.845661
17513995	Acta1	actin, alpha 1, skeletal muscle	24.90459	23.17681	21.01678	20.79623	21.69168	26.84274
17364098	Acta2	actin, alpha 2, smooth muscle, aorta	36.30711	36.74434	36.95916	44.55977	23.78846	32.10509
17389510	Actc1	actin, alpha, cardiac muscle 1	4.460731	3.121826	3.418942	4.155004	3.936545	4.164317
17454753	Actb	actin, beta	463.6557	465.6332	440.2777	444.3738	412.846	385.9072
17289813	Actbl2	actin, beta-like 2	6.168672	4.084671	4.719618	4.39432	4.146632	5.203997
17468183	Actg2	actin, gamma 2, smooth muscle, enteric	13.1346	11.07473	10.14601	10.27602	12.74836	12.42333
17273061	Actg1	actin, gamma, cytoplasmic 1	24.82772	15.10129	13.88938	18.49649	19.62866	16.56114
17500992	Actg-ps1	actin, gamma, pseudogene 1 [Source:MGI Symbol;Acc:MGI:87907]	79.79464	73.61817	102.2495	74.59464	70.87869	62.87001
17365911	Ablim1	actin-binding LIM protein 1	13.72715	17.52813	18.49649	19.79192	15.55558	16.1586
17436877	Ablim2	actin-binding LIM protein 2	12.72461	16.85635	11.38632	10.133	7.391463	11.78582
17316775	Abra	actin-binding Rho activating protein	4.975288	3.827839	3.194699	3.485873	3.382406	3.588249
17290603	Actn2	actinin alpha 2	4.29852	2.76995	2.611104	2.807313	2.835219	3.195651
17361362	Actn3	actinin alpha 3	7.153652	6.84718	7.073479	7.208654	6.773117	7.869817
17488565	Actn4	actinin alpha 4	168.1622	148.3951	157.3693	147.7782	142.881	134.5595
17282226	Actn1	actinin, alpha 1	71.4925	87.36963	100.098	68.80827	65.29316	67.04324
17485376	LOC102636989	actin-like	5.329795	6.281243	6.259121	4.780296	5.551086	4.227398
17416160	LOC102642973	actin-like	553.9876	481.3392	599.7594	453.0291	446.0835	413.2096
17378238	Actl10	actin-like 10	6.223042	6.143837	6.268716	5.974633	6.223042	7.566048
17521507	Actl11	actin-like 11	3.399235	4.080172	3.399235	2.606909	2.782767	3.084657
17396733	Actl6a	actin-like 6A	40.46857	27.53663	30.70773	32.5948	37.52812	30.15029
17443596	Actl6b	actin-like 6B	4.064252	4.296673	3.944931	4.534269	4.227955	4.870669
17414327	Actl7a	actin-like 7a	7.413013	11.71951	9.011267	8.51685	6.735476	9.387624
17425421	Actl7b	actin-like 7b	2.284962	2.599601	2.447824	2.480412	2.616521	2.464919
17336082	Actl9	actin-like 9	3.681312	3.530053	3.492645	4.457145	3.233709	3.571921
17492466	Arpin	actin-related protein 2/3 complex inhibitor	20.75806	27.59414	28.40317	28.29451	23.81156	22.60578
17534493	Actrt1	actin-related protein T1	4.436432	3.234965	3.891941	3.430955	3.720045	3.898935
17433661	Actrt2	actin-related protein T2	5.031679	4.464628	4.697262	4.518175	4.386682	5.11651
17330918	Alcam	activated leukocyte cell adhesion molecule	64.4331	61.40865	51.54784	44.08757	69.76928	65.4985
17233600	Ascc1	activating signal cointegrator 1 complex subunit 1	15.21687	19.22205	20.37842	19.02929	26.8988	19.28758
17246825	Ascc2	activating signal cointegrator 1 complex subunit 2	36.84278	38.11429	34.28819	36.07056	30.62016	33.84381
17233149	Ascc3	activating signal cointegrator 1 complex subunit 3	101.392	73.35178	72.58553	75.84278	78.57617	71.43999
17314972	Atf1	activating transcription factor 1	13.85524	18.02453	17.84243	16.88277	16.69241	13.81267
17386591	Atf2	activating transcription factor 2	51.44967	46.8946	45.77253	53.48936	41.47787	50.24019
17231033	Atf3	activating transcription factor 3	20.18903	31.56601	37.56429	21.43966	14.00083	13.56757
17313100	Atf4	activating transcription factor 4	81.17649	73.26539	71.92692	55.56945	64.10967	50.3933
17490391	Atf5	activating transcription factor 5	264.2501	280.4753	319.6856	250.669	293.694	237.2181
17229556	Atf6	activating transcription factor 6	180.2443	171.0519	189.3472	136.6688	161.9697	134.934
17336660	Atf6b	activating transcription factor 6 beta	30.31479	33.82223	32.81424	30.06139	27.39878	21.1615
17322261	Atf7	activating transcription factor 7	21.42703	40.43661	43.71874	31.73296	39.17361	35.00114
17322263	Atf7	activating transcription factor 7	39.35186	40.85979	42.02861	36.54723	41.60428	40.96103
17463812	Atf7ip	activating transcription factor 7 interacting protein	71.25849	65.87321	93.58919	74.74577	74.72453	70.49456
17463829	Atf7ip	activating transcription factor 7 interacting protein	19.42873	15.59563	18.90283	22.29456	17.71501	17.77367
17322806	Atf7ip2	activating transcription factor 7 interacting protein 2	2.872586	2.67402	2.380393	2.545043	2.67402	2.654739

17462676	Aicda	activation-induced cytidine deaminase	3.642174	3.292903	3.351693	3.142908	2.841928	3.158085
17291131	Abt1	activator of basal transcription 1	23.10223	26.2408	26.2408	24.29454	28.12088	25.58223
17440728	Aym1	activator of yeast meiotic promoters 1	12.64916	11.84216	17.93518	24.29454	13.06948	19.80702
17266107	Abr	active BCR-related gene	11.63428	16.95231	15.22531	11.36527	10.36494	10.80637
17385437	Acvr1	activin A receptor, type 1	32.14829	24.25516	26.14748	23.95027	19.70883	26.19755
17315152	Acvr1b	activin A receptor, type 1B	19.49889	18.35982	21.01649	29.22781	26.53749	19.94949
17385422	Acvr1c	activin A receptor, type IC	3.263862	3.913285	3.419227	3.703322	3.263862	3.339703
17315133	Acvrl1	activin A receptor, type II-like 1	10.36156	10.60527	12.64171	10.60527	9.648469	10.23721
17370598	Acvr2a	activin receptor IIA	65.72225	61.07034	52.56388	54.01919	59.73274	61.57915
17523081	Acvr2b	activin receptor IIB	13.68297	20.21802	13.60099	11.91313	12.24431	12.71236
17317983	Arc	activity regulated cytoskeletal-associated protein	16.52238	18.88227	17.97251	20.63698	15.91836	18.47638
17394774	Adnp	activity-dependent neuroprotective protein	50.98426	55.28208	57.89633	57.69762	38.92756	52.6182
17527271	Acsbg1	acyl-CoA synthetase bubblegum family member 1	6.313631	6.393429	6.993962	7.133005	6.724234	7.506654
17346369	Acsbg2	acyl-CoA synthetase bubblegum family member 2	4.623675	5.037088	5.230867	4.988444	5.937024	6.936029
17268010	Acsf2	acyl-CoA synthetase family member 2	384.8147	427.9149	413.5965	414.5378	269.9971	287.7137
17506443	Acsf3	acyl-CoA synthetase family member 3	50.41461	51.86035	60.406	56.75517	44.92697	55.04692
17500996	Acsl1	acyl-CoA synthetase long-chain family member 1	1645.42	1590.219	1384.51	1614.118	1141.797	1273.594
17545051	Acsl4	acyl-CoA synthetase long-chain family member 4	109.7959	104.6276	127.0515	91.45816	119.7474	94.35912
17360462	Acsl5	acyl-CoA synthetase long-chain family member 5	440.8885	407.6141	412.1741	357.7232	394.0705	449.0711
17249640	Acsl6	acyl-CoA synthetase long-chain family member 6	7.66566	8.288379	8.037351	7.891229	7.45506	8.885235
17482272	Acsm1	acyl-CoA synthetase medium-chain family member 1	240.6162	247.7257	212.8274	243.9768	247.7257	210.7473
17482247	Acsm2	acyl-CoA synthetase medium-chain family member 2	4.395691	4.128392	4.887376	4.069524	4.232999	4.131999
17482310	Acsm3	acyl-CoA synthetase medium-chain family member 3	248.8979	232.4538	220.6598	262.9728	258.968	278.0499
17482290	Acsm4	acyl-CoA synthetase medium-chain family member 4	4.218646	3.989395	3.900097	4.218646	4.662068	4.819956
17482230	Acsm5	acyl-CoA synthetase medium-chain family member 5	138.168	173.8115	151.252	163.9452	172.238	150.023
17392690	Acss1	acyl-CoA synthetase short-chain family member 1	8.240032	10.05897	9.128261	7.915717	7.147605	7.362471
17378359	Acss2	acyl-CoA synthetase short-chain family member 2	107.1182	96.76745	92.49414	114.9609	105.7145	107.5043
17393283	Acss2os	acyl-CoA synthetase short-chain family member 2, opposite strand	4.01461	3.896611	4.20927	5.551625	3.657404	4.885005
17244705	Acss3	acyl-CoA synthetase short-chain family member 3	76.71251	89.49592	104.8251	96.80301	57.84499	105.5769
17277140	Acot1	acyl-CoA thioesterase 1	46.16124	111.6144	131.9301	74.14204	45.87448	48.0955
17316153	Acot10	acyl-CoA thioesterase 10	9.714165	12.97821	9.388274	10.41665	9.991145	8.487678
17427860	Acot11	acyl-CoA thioesterase 11	13.0305	14.46596	12.83356	12.83312	10.52618	11.83026
17289061	Acot12	acyl-CoA thioesterase 12	322.1503	365.5285	347.4063	343.4271	329.1041	333.7722
17291355	Acot13	acyl-CoA thioesterase 13	110.703	116.6821	124.2249	113.4403	116.8691	137.8896
17277134	Acot2	acyl-CoA thioesterase 2	395.8829	751.3597	1155.851	383.5099	194.7285	208.5024
17277152	Acot3	acyl-CoA thioesterase 3	16.65034	30.9492	64.4742	29.31831	28.85961	19.19276
17277146	Acot4	acyl-CoA thioesterase 4	21.02553	40.06665	39.79816	30.48273	24.00645	25.07087
17277161	Acot5	acyl-CoA thioesterase 5	3.648719	3.648719	3.565341	3.854036	3.929811	4.468281
17277170	Acot6	acyl-CoA thioesterase 6	7.723421	10.46231	9.518538	10.08536	9.733172	9.179783
17422117	Acot7	acyl-CoA thioesterase 7	24.20243	21.8621	27.6261	23.03427	21.83469	27.53281
17394275	Acot8	acyl-CoA thioesterase 8	49.36101	42.63097	39.90009	40.11139	30.03869	37.22345
17539064	Acot9	acyl-CoA thioesterase 9	8.004382	9.301543	15.10548	9.488237	9.488237	9.535377
17536538	Awat1	acyl-CoA wax alcohol acyltransferase 1	4.335024	3.152355	3.428268	3.138489	4.002052	4.117675
17543460	Awat2	acyl-CoA wax alcohol acyltransferase 2	4.393833	4.488365	3.775375	4.529871	5.621792	4.563498
17425211	Acnat1	acyl-coenzyme A amino acid N-acyltransferase 1	65.87184	70.4052	77.28733	84.18376	81.60581	65.3629
17425202	Acnat2	acyl-coenzyme A amino acid N-acyltransferase 2	48.27586	66.18214	86.85217	81.68674	86.35154	59.21579
17220166	Acbd3	acyl-Coenzyme A binding domain containing 3	72.07915	70.61095	58.10659	52.68973	59.20676	53.9436
17257067	Acbd4	acyl-Coenzyme A binding domain containing 4	17.65102	23.94363	26.38397	23.97485	23.25244	21.84089
17367576	Acbd5	acyl-Coenzyme A binding domain containing 5	390.5626	351.6948	302.0346	332.2326	310.5475	389.9053
17218377	Acbd6	acyl-Coenzyme A binding domain containing 6	25.66855	18.76068	24.19539	22.07069	32.65744	21.96828
17366393	Acbd7	acyl-Coenzyme A binding domain containing 7	3.858473	3.901503	5.043656	4.33163	4.039593	4.228688
17452264	Acad10	acyl-Coenzyme A dehydrogenase family, member 10	29.71542	29.55827	43.27524	33.31823	33.47008	35.90294
17521014	Acad11	acyl-Coenzyme A dehydrogenase family, member 11	520.5634	536.6955	516.8959	491.2147	382.5117	428.0751
17452248	Acad12	acyl-Coenzyme A dehydrogenase family, member 12	10.88251	11.14829	11.19079	11.1804	8.826858	10.93863
17525163	Acad8	acyl-Coenzyme A dehydrogenase family, member 8	86.49651	83.58082	85.04179	93.96034	74.59722	80.92031
17396941	Acad9	acyl-Coenzyme A dehydrogenase family, member 9	49.65544	53.50456	46.38286	55.54403	39.81555	46.25059
17223903	Acadl	acyl-Coenzyme A dehydrogenase, long-chain	617.3479	750.7611	779.962	650.3714	614.8156	698.3275
17411323	Acadm	acyl-Coenzyme A dehydrogenase, medium chain	1215.648	1420.93	1303.993	1219.962	1007.427	1141.314
17451649	Acads	acyl-Coenzyme A dehydrogenase, short chain	292.2785	287.8932	280.8566	265.4433	240.2845	245.3011
17484014	Acadsb	acyl-Coenzyme A dehydrogenase, short/branched chain	145.0393	123.336	95.38298	122.1754	107.6467	109.3591
17265129	Acadvl	acyl-Coenzyme A dehydrogenase, very long chain	389.6568	389.5352	372.2944	369.733	353.2021	329.435
17272241	Acox1	acyl-Coenzyme A oxidase 1, palmitoyl	2033.642	2233.066	2345.972	2006.576	1738.359	1802.383
17303412	Acox2	acyl-Coenzyme A oxidase 2, branched chain	271.5455	290.5857	245.245	299.6947	277.6243	290.6099
17436839	Acox3	acyl-Coenzyme A oxidase 3, pristanoyl	37.0759	32.8131	34.42751	36.08982	31.22739	25.65663
17375935	Acoxl	acyl-Coenzyme A oxidase-like	5.114311	6.171709	6.059589	6.171709	5.564299	5.091497
17457555	Agk	acylglycerol kinase	14.55568	17.16526	20.36303	15.61705	20.33101	17.16526
17285586	Aoah	acyloxyacyl hydrolase	5.541897	5.844785	6.443301	6.595491	4.285222	5.56106
17531130	Apeh	acylpeptide hydrolase	56.20085	58.02771	52.16608	56.53726	47.67265	52.4998
17282689	Acyp1	acylphosphatase 1, erythrocyte (common) type	22.10468	22.38143	25.22636	23.8005	24.19874	20.68123
17261393	Acyp2	acylphosphatase 2, muscle type	10.81176	8.107966	7.155834	6.661606	5.227967	8.426071
17308083	Adamdec1	ADAM-like, decysin 1	9.900834	10.36652	11.1367	7.837698	10.43523	12.44031
17415130	Adamtsl1	ADAMTS-like 1	4.430716	4.738564	4.276676	3.867845	4.809144	3.341834
17415136	Adamtsl1	ADAMTS-like 1	5.780603	6.121563	5.703154	7.093364	5.465904	5.780603
17368476	Adamtsl2	ADAMTS-like 2	17.46579	23.00093	20.93057	22.16129	18.88973	22.46878
17479769	Adamtsl3	ADAMTS-like 3	4.884023	5.51993	5.985742	5.51993	5.728312	6.680842
17407829	Adamtsl4	ADAMTS-like 4	12.59571	13.60599	14.48502	9.691299	12.22905	13.91366
17242879	Adamtsl5	ADAMTS-like 5	10.8887	11.59684	13.85899	10.50016	9.804065	7.990856
17246904	Ap1b1	adaptor protein complex AP-1, beta 1 subunit	78.69227	67.45718	79.49874	83.14629	62.2961	71.80289
17505426	Ap1g1	adaptor protein complex AP-1, gamma 1 subunit	153.8947	148.5227	139.1889	133.2027	128.7626	113.9455
17306638	Ap1g2	adaptor protein complex AP-1, gamma 2 subunit	13.524	12.10549	15.73342	11.1571	13.07628	11.24366
17524703	Ap1m2	adaptor protein complex AP-1, mu 2 subunit	4.566377	4.566377	5.49306	4.79779	4.480905	4.692771
17453809	Ap1s1	adaptor protein complex AP-1, sigma 1	30.34864	28.35596	32.12223	22.93295	25.0148	30.38569
17304246	Appl1	adaptor protein, phosphotyrosine interaction, PH domain and leucine zipper conta	49.01665	41.33638	41.37708	48.56214	47.56637	45.40408
17243576	Appl2	adaptor protein, phosphotyrosine interaction, PH domain and leucine zipper conta	25.98649	30.90161	32.44423	27.3021	30.02787	33.6077

17376466	Ap5s1	adaptor-related protein 5 complex, sigma 1 subunit	29.05008	28.49032	29.67919	28.11913	30.66795	28.51462
17410227	Ap1ar	adaptor-related protein complex 1 associated regulatory protein	97.21587	106.0308	123.3428	94.97132	88.78475	94.14157
17539467	Ap1s2	adaptor-related protein complex 1, sigma 2 subunit	3.842912	4.178224	3.842912	4.20927	3.842912	3.37765
17490452	Ap2a1	adaptor-related protein complex 2, alpha 1 subunit	48.8972	40.66174	54.16954	43.31606	48.11417	44.04114
17484966	Ap2a2	adaptor-related protein complex 2, alpha 2 subunit	212.6829	177.567	169.027	180.5194	172.83	165.7322
17254210	Ap2b1	adaptor-related protein complex 2, beta 1 subunit	78.15358	79.66312	81.70713	78.85444	77.67724	77.44982
17323929	Ap2m1	adaptor-related protein complex 2, mu 1 subunit	227.2159	248.8789	265.9387	223.0711	218.4107	207.6817
17474136	Ap2s1	adaptor-related protein complex 2, sigma 1 subunit	86.43657	104.1161	111.3942	105.5652	110.1038	116.4026
17289207	Ap3b1	adaptor-related protein complex 3, beta 1 subunit	106.2626	116.6967	116.6967	110.5021	116.6967	112.9453
17492772	Ap3b2	adaptor-related protein complex 3, beta 2 subunit	5.310214	5.792194	5.918411	4.894156	5.027113	4.981154
17243057	Ap3d1	adaptor-related protein complex 3, delta 1 subunit	149.388	151.1985	186.9463	131.7665	130.0498	122.0882
17303941	Ap3m1	adaptor-related protein complex 3, mu 1 subunit	92.80946	128.1625	101.0083	125.0378	107.8214	149.2017
17508111	Ap3m2	adaptor-related protein complex 3, mu 2 subunit	12.67927	12.34641	11.54135	12.99919	14.37002	12.99919
17350369	Ap3s1	adaptor-related protein complex 3, sigma 1 subunit	9.487842	6.124788	5.093898	6.835088	7.515348	6.790275
17504019	Ap3s1-ps2	adaptor-related protein complex 3, sigma 1 subunit, pseudogene 2 [Source:MGI S	21.83151	18.11898	10.14826	13.78436	5.120306	18.90021
17492457	Ap3s2	adaptor-related protein complex 3, sigma 2 subunit	32.84359	36.1231	33.3187	37.45717	39.30961	31.00631
17356477	Ap5b1	adaptor-related protein complex 5, beta 1 subunit	12.7322	13.92196	13.39423	14.90051	14.65184	17.86978
17299454	Ap5m1	adaptor-related protein complex 5, mu 1 subunit	32.34005	32.69436	28.37051	27.94678	30.39917	27.47181
17444216	Ap5z1	adaptor-related protein complex 5, zeta 1 subunit	29.35796	23.41531	24.67127	18.23768	22.0525	24.98603
17502423	Ap1m1	adaptor-related protein complex AP-1, mu subunit 1	47.51134	49.16501	59.20881	53.13977	47.22045	60.56364
17224738	Ap1s3	adaptor-related protein complex AP-1, sigma 3	5.193006	6.078941	5.617429	5.617429	4.986543	6.182241
17401246	Ap4b1	adaptor-related protein complex AP-4, beta 1	50.37269	38.80122	46.95738	45.33615	42.17248	48.96945
17375742	Ap4e1	adaptor-related protein complex AP-4, epsilon 1	9.880883	11.92089	12.107	11.93412	10.79896	11.83986
17443766	Ap4m1	adaptor-related protein complex AP-4, mu 1	27.22254	29.63335	26.71948	24.38311	32.42962	27.83889
17275392	Ap4s1	adaptor-related protein complex AP-4, sigma 1	8.764428	9.248568	9.491657	7.981998	9.689351	11.96775
17377940	Asxl1	additional sex combs like 1	15.62095	21.09487	25.55281	21.29481	19.06494	19.93332
17273604	Asxl2	additional sex combs like 2 (Drosophila)	27.79378	37.31749	38.00665	39.15949	37.04944	38.41772
17348879	Asxl3	additional sex combs like 3 (Drosophila)	5.366532	5.495421	5.014758	5.269149	5.411881	4.72289
17436629	Add1	adducin 1 (alpha)	47.00949	45.03735	47.81562	41.91777	50.82795	42.86802
17460366	Add2	adducin 2 (beta)	8.630595	8.634365	7.514984	8.594359	8.066708	8.420524
17360312	Add3	adducin 3 (gamma)	45.04297	37.06254	46.64401	33.45291	36.94712	34.00064
17513771	Aprt	adenine phosphoribosyl transferase	50.42265	70.05665	71.06809	79.21545	59.53557	60.83206
17349369	Apc	adenomatosis polyposis coli	37.47275	36.09782	39.13425	32.40692	39.33604	38.50356
17235300	Apc2	adenomatosis polyposis coli 2	12.13742	11.88508	11.37899	11.10371	10.4471	9.931076
17351196	Apcdd1	adenomatosis polyposis coli down-regulated 1	8.027663	8.299359	8.994543	11.14489	8.984075	6.582657
17227094	Adora1	adenosine A1 receptor	48.50159	70.98243	77.21397	59.91644	56.92143	66.61245
17234339	Adora2a	adenosine A2a receptor	12.13187	16.88933	9.758978	9.488237	9.96438	10.74736
17250660	Adora2b	adenosine A2b receptor	11.95051	15.27987	14.42851	20.49486	14.7941	15.28262
17394063	Ada	adenosine deaminase	8.919853	8.339321	9.035973	8.471515	7.844981	9.294131
17397091	Adad1	adenosine deaminase domain containing 1 (testis specific)	2.858959	3.053577	3.516816	3.336487	3.868274	3.628869
17506126	Adad2	adenosine deaminase domain containing 2	5.147779	6.030928	5.991055	6.030928	6.030928	6.158291
17399496	Adar	adenosine deaminase, RNA-specific	35.61097	38.30737	38.47955	39.07814	39.34313	38.97832
17242281	Adarb1	adenosine deaminase, RNA-specific, B1	27.35267	24.80708	26.40794	23.95459	22.62899	27.92219
17285036	Adarb2	adenosine deaminase, RNA-specific, B2	3.224689	3.300718	2.686672	3.63136	3.482951	3.203969
17513194	Adat1	adenosine deaminase, tRNA-specific 1	5.48933	7.055948	5.617429	5.48933	6.295409	6.092186
17231733	Adat2	adenosine deaminase, tRNA-specific 2	16.19829	16.84677	13.51397	19.22671	16.84677	18.74976
17235368	Adat3	adenosine deaminase, tRNA-specific 3	26.34103	28.19212	27.98923	22.84901	22.00104	22.72298
17375203	Adal	adenosine deaminase-like	28.58151	31.5601	25.97137	26.36112	22.49231	28.04672
17297576	Adk	adenosine kinase	652.7108	654.1193	632.9115	659.3086	649.673	695.9983
17401126	Ampd1	adenosine monophosphate deaminase 1	4.482271	4.888731	5.2758	5.546278	4.898228	5.650858
17409184	Ampd2	adenosine monophosphate deaminase 2	35.96945	26.74505	28.81024	27.42786	25.47659	28.16575
17481776	Ampd3	adenosine monophosphate deaminase 3	10.48228	10.48228	10.91031	12.75738	10.48228	9.665605
17247183	Adcy1	adenylate cyclase 1	6.287864	6.859817	5.4899	6.960592	5.379567	6.287864
17218965	Adcy10	adenylate cyclase 10	3.214156	3.214156	3.236625	3.351995	3.263862	3.669873
17294086	Adcy2	adenylate cyclase 2	5.874557	7.941437	6.775183	5.790107	6.174062	7.733492
17273714	Adcy3	adenylate cyclase 3	7.580694	10.40172	8.276209	9.824813	9.707905	9.402341
17306877	Adcy4	adenylate cyclase 4	11.57885	14.40962	22.30863	17.188	13.50686	17.97899
17325206	Adcy5	adenylate cyclase 5	7.363134	8.766737	8.351469	7.698994	9.399018	7.006189
17321263	Adcy6	adenylate cyclase 6	21.99098	34.46237	36.84431	28.3827	29.18125	37.07488
17503616	Adcy7	adenylate cyclase 7	9.91079	15.09648	10.93067	8.062292	10.99968	10.93067
17317533	Adcy8	adenylate cyclase 8	3.348906	4.538136	3.212597	3.306603	3.182411	3.268163
17327720	Adcy9	adenylate cyclase 9	50.41636	53.65981	52.56826	50.96306	46.47265	53.78344
17340404	Adcyap1	adenylate cyclase activating polypeptide 1	3.501444	4.414806	4.1753	4.330189	3.505985	4.141375
17458841	Adcyap1r1	adenylate cyclase activating polypeptide 1 receptor 1	10.90245	10.54794	9.488237	8.566762	9.933898	9.488237
17369911	Ak1	adenylate kinase 1	9.315415	9.851135	9.573723	13.45817	9.859196	12.06294
17418904	Ak2	adenylate kinase 2	183.8328	206.6277	179.0862	191.9265	166.8583	202.6898
17363779	Ak3	adenylate kinase 3	514.4368	505.0667	484.0694	525.2644	482.612	470.5291
17415932	Ak4	adenylate kinase 4	94.29308	82.01523	92.49798	81.39171	91.72035	85.13733
17411235	Ak5	adenylate kinase 5	4.747342	5.137726	4.843601	4.722039	5.120306	5.133455
17278415	Ak7	adenylate kinase 7	3.910383	3.736429	3.958901	3.946408	3.670305	4.364208
17368741	Ak8	adenylate kinase 8	12.47977	13.54464	12.67303	12.52501	11.64937	15.10925
17232802	Ak9	adenylate kinase 9	4.729114	5.120306	4.690039	4.873371	5.207641	5.010727
17313199	Adsl	adenylosuccinate lyase	35.26365	35.36107	34.72902	47.08416	34.10355	36.43189
17279349	Adssl1	adenylosuccinate synthetase like 1	39.05594	45.41415	49.5492	49.72364	36.05731	49.39524
17230256	Adss	adenylosuccinate synthetase, non muscle	122.445	115.8296	113.5418	119.2611	115.4577	109.6354
17433528	Ajap1	adherens junction associated protein 1	6.171709	4.801649	5.075395	4.994638	3.731459	5.495459
17484409	Adgra1	adhesion G protein-coupled receptor A1	7.944685	8.822637	8.467112	8.467112	10.11848	9.829922
17500301	Adgra2	adhesion G protein-coupled receptor A2	18.97424	23.26759	21.88833	23.29067	19.09681	19.50457
17447979	Adgra3	adhesion G protein-coupled receptor A3	93.24213	70.65697	76.04967	63.74896	56.75911	59.24206
17312146	Adgrb1	adhesion G protein-coupled receptor B1	9.123011	7.782103	8.799918	6.893134	7.200365	8.64137
17419097	Adgrb2	adhesion G protein-coupled receptor B2	7.870689	9.015578	7.611654	7.431276	8.297806	9.967488
17221878	Adgrb3	adhesion G protein-coupled receptor B3	2.953407	2.953407	2.790551	2.953407	2.953407	2.897904
17442834	Adgrd1	adhesion G protein-coupled receptor D1	4.148961	5.397981	7.234834	4.950245	4.687244	5.94283
17370471	Adgrd2-ps	adhesion G protein-coupled receptor D2, pseudogene	6.832909	7.86938	6.694935	7.616721	7.449017	7.263677

17339013	Adgre1	adhesion G protein-coupled receptor E1	43.65878	55.09922	53.78045	45.65117	46.52648	54.73417
17338617	Adgre4	adhesion G protein-coupled receptor E4	39.8893	49.96098	54.04316	42.51323	45.75032	35.12874
17510948	Adgre5	adhesion G protein-coupled receptor E5	83.37656	108.2483	154.9609	96.42531	91.95142	81.85564
17337750	Adgrf1	adhesion G protein-coupled receptor F1	4.509789	3.709745	3.853769	4.639277	4.509789	5.189156
17345029	Adgrf2	adhesion G protein-coupled receptor F2	6.128356	6.943774	6.943774	6.943774	6.992896	6.943774
17446669	Adgrf3	adhesion G protein-coupled receptor F3	5.585127	5.252157	7.06701	5.00167	4.675336	4.835181
17345016	Adgrf4	adhesion G protein-coupled receptor F4	3.102304	3.218682	3.35874	3.316083	3.65563	3.440647
17337770	Adgrf5	adhesion G protein-coupled receptor F5	46.28491	43.96123	58.83774	50.39155	36.56167	44.44749
17504190	Adgrg1	adhesion G protein-coupled receptor G1	10.64047	15.78158	22.53038	15.26409	10.53107	9.327432
17539271	Adgrg2	adhesion G protein-coupled receptor G2	7.131275	7.48779	8.467112	8.466232	8.341114	7.169189
17504207	Adgrg3	adhesion G protein-coupled receptor G3	12.78012	12.7649	17.46785	15.55256	8.427472	13.05047
17534982	Adgrg4	adhesion G protein-coupled receptor G4	3.007553	3.154278	2.78323	2.407073	3.435762	3.007553
17504174	Adgrg5	adhesion G protein-coupled receptor G5	4.579468	4.836254	5.404009	6.541085	4.351793	5.382849
17239435	Adgrg6	adhesion G protein-coupled receptor G6	4.970014	6.038415	5.120306	6.943774	4.652319	3.843578
17331059	Adgrg7	adhesion G protein-coupled receptor G7	2.685461	3.550691	2.880921	3.347792	3.296443	2.845259
17502994	Adgrl1	adhesion G protein-coupled receptor L1	7.001383	18.69603	11.45813	13.82799	12.39013	12.44885
17411103	Adgrl2	adhesion G protein-coupled receptor L2	63.11539	59.34029	62.38641	65.76828	68.29178	51.91178
17438530	Adgrl3	adhesion G protein-coupled receptor L3	4.211984	4.612823	3.620277	3.263681	3.816949	3.241946
17403605	Adgrl4	adhesion G protein-coupled receptor L4	49.85305	35.28101	45.44627	37.09724	19.79879	23.29454
17294565	Adgrv1	adhesion G protein-coupled receptor V1	4.220137	4.100927	4.226138	4.326529	4.316285	4.20927
17401650	Amigo1	adhesion molecule with Ig like domain 1	13.51585	14.56385	16.33076	13.02399	10.25539	14.37958
17320967	Amigo2	adhesion molecule with Ig like domain 2	24.31711	23.99696	25.94582	27.19784	26.36112	26.36112
17521621	Amigo3	adhesion molecule with Ig like domain 3	6.909302	8.319461	7.427568	5.988522	8.016486	7.347228
17516718	Amica1	adhesion molecule, interacts with CXADR antigen 1	4.728098	5.001809	5.95487	4.237402	4.686627	5.070015
17380729	Adrm1	adhesion regulating molecule 1	42.65994	47.55384	46.7139	39.87824	44.57182	33.57953
17407396	Adrm1	adhesion regulating molecule 1	110.6562	114.9442	117.4929	112.1822	112.1822	103.0228
17392676	Apmap	adipocyte plasma membrane associated protein	54.42698	56.16658	50.79061	44.29095	54.87054	53.71004
17537865	Arxes1	adipocyte-related X-chromosome expressed sequence 1	2.038701	2.245863	2.459594	2.28791	2.203321	2.875833
17537861	Arxes2	adipocyte-related X-chromosome expressed sequence 2	2.623968	3.076863	3.569644	3.009471	2.571428	2.773793
17493436	Aamdc	adipogenesis associated Mth938 domain containing	22.53335	21.48667	18.03716	21.13087	22.53335	23.9161
17378898	Adig	adipogenin	3.462322	4.064985	3.729055	4.603177	4.265397	3.729055
17217455	Adipor1	adiponectin receptor 1	137.733	128.1554	136.3055	131.1278	129.3997	126.194
17470301	Adipor2	adiponectin receptor 2	492.483	515.3897	501.9118	535.3876	434.7403	459.1017
17324404	Adipoq	adiponectin, C1Q and collagen domain containing	4.830525	3.855559	4.247577	4.528804	4.429028	4.209474
17355619	Adnp2	ADNP homeobox 2	13.17252	12.66215	11.95233	11.50086	8.421108	11.77039
17517934	Adpgk	ADP-dependent glucokinase	35.87458	28.43193	26.82061	24.61319	25.62677	24.16672
17264295	Adprm	ADP-ribose/CDP-alcohol diphosphatase, manganese dependent	15.79351	14.93857	15.45391	18.34837	17.50651	15.60688
17330373	Adprh	ADP-ribosylarginine hydrolase	22.62114	16.09042	17.29784	17.23011	17.47088	15.65292
17263379	Arf1	ADP-ribosylation factor 1	133.7495	140.2338	134.8611	148.866	131.7683	150.4083
17548910	LOC102632770	ADP-ribosylation factor 1 pseudogene	552.2279	501.2755	540.7695	451.1114	497.0027	472.893
17257165	Arf2	ADP-ribosylation factor 2	8.707147	12.46836	11.41501	15.33302	15.535	9.682436
17321326	Arf3	ADP-ribosylation factor 3	27.66136	33.5193	26.14857	27.46914	28.71674	23.96455
17297913	Arf4	ADP-ribosylation factor 4	210.6202	158.047	147.1333	150.6921	164.4961	158.9016
17304235	Arf4os	ADP-ribosylation factor 4, opposite strand [Source:MGI Symbol;Acc:MGI:3642411]	5.493707	5.947486	6.501759	6.587906	6.684826	6.31744
17456485	Arf5	ADP-ribosylation factor 5	68.42777	54.4002	58.63296	50.93551	75.21351	66.86874
17275925	Arf6	ADP-ribosylation factor 6	80.78301	83.64689	84.22111	81.09101	80.89788	85.83654
17380892	Arfgap1	ADP-ribosylation factor GTPase activating protein 1	55.35071	56.8812	47.388	54.89603	61.63253	52.76722
17373262	Arfgap2	ADP-ribosylation factor GTPase activating protein 2	111.784	114.7476	112.1363	115.6723	126.062	107.4156
17319806	Arfgap3	ADP-ribosylation factor GTPase activating protein 3	48.18626	16.65877	20.78571	20.2639	20.28259	21.09106
17221228	Arfgef1	ADP-ribosylation factor guanine nucleotide-exchange factor 1(brefeldin A-inhibite	211.2621	196.0652	174.6182	186.8556	186.6782	191.1843
17379767	Arfgef2	ADP-ribosylation factor guanine nucleotide-exchange factor 2 (brefeldin A-inhibite	126.7885	109.3409	120.7609	116.2349	106.4166	117.882
17406383	Arfip1	ADP-ribosylation factor interacting protein 1	60.42694	58.7929	53.46489	51.061	58.23522	54.84355
17406395	Arfip1	ADP-ribosylation factor interacting protein 1	2.67402	3.723166	4.801649	4.692608	4.352065	6.430184
17494559	Arfip2	ADP-ribosylation factor interacting protein 2	36.62102	28.77073	29.31099	28.84461	31.43805	37.64718
17395852	Arfrp1	ADP-ribosylation factor related protein 1	40.74892	35.10294	40.32856	42.39347	31.79526	39.97788
17236378	Arl1	ADP-ribosylation factor-like 1	104.632	95.37836	82.29712	84.39116	99.63702	87.73618
17287454	Arl10	ADP-ribosylation factor-like 10	14.57749	13.09769	14.93857	11.43449	9.586472	12.00098
17301108	Arl11	ADP-ribosylation factor-like 11	11.7615	9.996895	11.03244	14.27303	15.09355	11.93296
17537632	Arl13a	ADP-ribosylation factor-like 13A	6.224034	5.65909	6.194896	5.932458	6.709755	5.409743
17331365	Arl13b	ADP-ribosylation factor-like 13B	12.19992	12.51494	14.03512	14.15235	14.31296	22.3746
17398272	Arl14	ADP-ribosylation factor-like 14	4.725837	5.136337	5.959784	5.209807	5.345222	4.00866
17389093	Arl14ep	ADP-ribosylation factor-like 14 effector protein	24.33026	22.96126	21.54005	22.96126	20.08421	19.73425
17350401	Arl14epl	ADP-ribosylation factor-like 14 effector protein-like	8.452218	9.613755	7.984821	11.25583	9.883623	9.579298
17290033	Arl15	ADP-ribosylation factor-like 15	48.33479	43.3993	52.4696	44.97402	38.64474	38.94132
17273121	Arl16	ADP-ribosylation factor-like 16	11.24366	11.05196	12.0363	13.35456	11.42031	11.24366
17361988	Arl2	ADP-ribosylation factor-like 2	16.60758	17.48675	15.8412	17.49632	17.84799	15.05103
17504112	Arl2bp	ADP-ribosylation factor-like 2 binding protein	18.40658	21.33204	24.12605	19.18571	20.35174	18.02004
17365403	Arl3	ADP-ribosylation factor-like 3	47.40082	59.18132	59.18132	62.67508	58.07076	59.18132
17280810	Arl4a	ADP-ribosylation factor-like 4A	86.7559	71.09469	73.66973	76.5452	67.89437	80.63253
17275155	Arl4aos	ADP-ribosylation factor-like 4A, opposite strand	3.527436	3.544495	3.339703	3.762079	3.339703	3.437763
17225360	Arl4c	ADP-ribosylation factor-like 4C	15.68779	16.5515	18.11647	15.98149	21.86346	18.68113
17256823	Arl4d	ADP-ribosylation factor-like 4D	53.20131	55.60721	50.55283	57.82254	75.07236	76.30523
17385272	Arl5a	ADP-ribosylation factor-like 5A	212.3073	203.1934	229.89	199.9395	235.4309	221.8945
17367192	Arl5b	ADP-ribosylation factor-like 5B	77.92531	98.37024	88.11587	79.92029	93.455	104.3821
17268681	Arl5c	ADP-ribosylation factor-like 5C	3.817928	4.993184	4.529589	4.751029	5.964578	5.247863
17331311	Arl6	ADP-ribosylation factor-like 6	7.571295	7.870689	7.30265	8.411599	6.63842	8.253751
17495466	Arl6ip1	ADP-ribosylation factor-like 6 interacting protein 1	329.5423	296.9364	277.8958	288.3546	263.6957	316.3044
17442474	Arl6ip4	ADP-ribosylation factor-like 6 interacting protein 4	69.67649	66.42802	65.95865	62.9821	65.94037	71.80289
17461124	Arl6ip5	ADP-ribosylation factor-like 6 interacting protein 5	60.64429	61.1237	60.54349	57.44502	52.06926	48.6946
17370843	Arl6ip6	ADP-ribosylation factor-like 6 interacting protein 6	13.66895	15.21097	15.8277	17.7605	15.19137	17.22008
17217580	Arl8a	ADP-ribosylation factor-like 8A	47.22766	58.89528	43.79275	70.55322	42.60114	61.90011
17461423	Arl8b	ADP-ribosylation factor-like 8B	159.5373	152.9716	139.3404	144.9961	124.7858	135.8528
17438459	Arl9	ADP-ribosylation factor-like 9	3.130558	3.122497	3.152246	2.965818	3.49606	2.791712
17507573	Adprhl1	ADP-ribosylhydrolase like 1	7.498801	7.173166	6.992896	9.431257	7.068627	8.103583

17430023	Adprhl2	ADP-ribosylhydrolase like 2	19.52797	17.62059	20.95801	25.36118	21.66884	15.62225
17481057	Art1	ADP-ribosyltransferase 1	5.690203	8.161702	7.118583	7.16601	7.915223	8.596861
17493906	Art2a-ps	ADP-ribosyltransferase 2a, pseudogene	8.00061	7.221207	6.992896	6.900831	6.294754	6.047044
17493912	Art2b	ADP-ribosyltransferase 2b	7.844274	3.041894	7.769167	5.659835	3.167051	7.017562
17439094	Art3	ADP-ribosyltransferase 3	5.842112	5.590239	4.259695	7.084863	5.323002	5.246735
17472185	Art4	ADP-ribosyltransferase 4	21.03894	19.35333	20.96629	20.96629	20.0582	19.36152
17494020	Art5	ADP-ribosyltransferase 5	4.152931	4.513135	4.152931	5.036914	3.585116	4.152931
17361223	Adrbk1	adrenergic receptor kinase, beta 1	45.95882	47.96101	47.26171	36.99068	43.42609	52.9339
17451297	Adrbk2	adrenergic receptor kinase, beta 2	12.58218	20.03666	20.84746	18.49649	15.88144	15.1407
17301502	Adra1a	adrenergic receptor, alpha 1a	7.8738	6.979724	6.960399	6.791311	6.641274	7.475655
17261982	Adra1b	adrenergic receptor, alpha 1b	65.41955	75.25054	78.70645	75.08954	64.40898	72.11213
17391899	Adra1d	adrenergic receptor, alpha 1d	3.439908	3.729055	3.729055	4.834645	3.729055	3.729055
17360440	Adra2a	adrenergic receptor, alpha 2a	3.854223	5.351525	7.122778	5.002052	4.026454	4.164866
17375880	Adra2b	adrenergic receptor, alpha 2b	32.94094	29.41541	20.68109	29.31506	25.35591	32.85088
17436823	Adra2c	adrenergic receptor, alpha 2c	4.11836	4.710238	4.453933	6.295147	3.939056	5.538556
17360599	Adrb1	adrenergic receptor, beta 1	11.81453	12.70989	14.83775	12.70989	12.50766	14.37958
17354857	Adrb2	adrenergic receptor, beta 2	8.997037	10.21028	9.664734	8.650299	9.697548	9.281256
17508544	Adrb3	adrenergic receptor, beta 3	11.82682	12.37983	10.42684	11.71285	11.58037	9.566359
17512479	Acd	adrenocortical dysplasia	34.29437	36.80628	36.09482	35.60308	31.57454	34.29437
17481770	Adm	adrenomedullin	11.87726	16.86137	17.16395	10.55087	13.01614	13.58997
17314115	Adm2	adrenomedullin 2	4.295452	5.408318	5.617429	5.193942	6.793052	4.198314
17336618	Ager	advanced glycosylation end product-specific receptor	13.44521	16.20178	17.24194	16.79186	16.79186	15.6125
17237851	Avil	advillin	8.749254	9.273539	8.750831	8.542214	8.733319	9.540269
17247039	Aebp1	AE binding protein 1	8.381567	10.49085	10.54195	10.54195	7.577542	9.500148
17464010	Aebp2	AE binding protein 2	63.82015	61.6821	62.09693	65.33481	64.31128	63.69464
17439741	Aff1	AF4/FMR2 family, member 1	42.97751	50.29036	58.81206	53.34348	48.61973	47.22307
17535245	Aff2	AF4/FMR2 family, member 2	3.914641	3.990944	3.990944	3.990944	4.136556	3.693222
17222465	Aff3	AF4/FMR2 family, member 3	4.332711	5.583153	4.663393	4.802881	4.854087	4.3115
17249489	Aff4	AF4/FMR2 family, member 4	224.5885	265.9571	246.0197	237.2986	234.1907	228.5934
17438920	Afm	afamin	111.4058	108.273	92.6841	85.94251	128.6565	122.3525
17506661	Afg3l1	AFG3-like AAA ATPase 1	102.0378	87.07339	83.41818	82.51188	87.59703	72.46638
17355169	Afg3l2	AFG3-like AAA ATPase 2	115.6146	131.3015	132.0792	126.1241	115.3025	135.138
17260885	Aftph	aftiphilin	69.82056	70.10474	70.99916	71.27825	77.25573	73.61766
17479335	Acan	aggrecan	4.378176	4.378176	5.252412	4.319038	5.120306	5.132494
17421110	Agmat	agmatine ureohydrolase (agmatinase)	318.9287	347.014	310.4764	313.0415	269.048	304.9312
17512467	Agrp	agouti related neuropeptide	4.927719	6.16786	5.606965	4.81398	4.793602	8.470517
17433977	Agrn	agrin	29.47112	27.56107	24.42066	29.86885	25.16035	26.2059
17277609	Ahsa1	AHA1, activator of heat shock protein ATPase 1	131.4846	107.7101	102.3608	116.3494	108.0002	105.552
17261035	Ahsa2	AHA1, activator of heat shock protein ATPase 2	63.94413	50.33116	45.91425	45.78934	46.27272	46.18396
17357389	Ahnak	AHNAK nucleoprotein (desmoyokin)	20.19295	20.55646	20.29159	20.29159	15.25003	19.43022
17284222	Ahnak2	AHNAK nucleoprotein 2	12.95916	9.993638	14.48281	12.52562	12.63374	14.80928
17547719	Ahnak2	AHNAK nucleoprotein 2	2.870058	3.344406	3.272175	3.099574	4.203235	3.327941
17293695	Aaed1	AhpC/TSA antioxidant enzyme domain containing 1	297.7567	318.3809	288.1867	311.5608	292.1975	304.8129
17306396	Ajuba	ajuba LIM protein	16.39268	22.81878	19.65072	21.09311	18.03178	14.37958
17429810	Akirin1	akirin 1	12.30259	14.11229	9.008519	13.92794	11.35708	7.991022
17337404	Gm6623	akirin 1 pseudogene	4.20927	3.35325	3.052286	3.663823	3.083203	2.965468
17412645	Akirin2	akirin 2	93.20723	90.02476	85.11609	93.78271	79.35394	91.30551
17401790	Aknad1	AKNA domain containing 1	3.704452	4.447856	4.355777	3.812639	4.833439	4.085719
17223277	Gm6644	Akr1b3 pseudogene	15.02195	13.76373	16.10269	22.48078	15.10946	16.02042
17477579	Akt1s1	AKT1 substrate 1 (proline-rich)	36.02234	35.35984	31.34905	41.29313	36.95019	36.13337
17477595	Akt1s1	AKT1 substrate 1 (proline-rich)	17.50397	26.1823	23.49513	20.50409	18.83286	18.5368
17311473	Aard	alanine and arginine rich domain containing protein	7.01479	6.148097	6.24837	8.998721	7.910671	7.889479
17215885	Agxt	alanine-glyoxylate aminotransferase	204.9915	185.4942	196.3645	229.8725	257.979	318.3147
17310282	Agxt2	alanine-glyoxylate aminotransferase 2	219.0566	226.4471	228.8423	245.5989	262.6504	296.9878
17492431	Anpep	alanyl (membrane) aminopeptidase	212.2043	214.7047	250.9385	191.1432	233.3805	199.873
17337895	Aars2	alanyl-tRNA synthetase 2, mitochondrial (putative)	25.6851	21.91748	25.59269	28.16302	25.5045	25.70113
17269870	Aarsd1	alanyl-tRNA synthetase domain containing 1	17.77503	19.16298	17.6722	18.71211	23.14245	18.93824
17438886	Alb	albumin	7455.193	7834.796	7755.209	7195.254	7831.267	7575.892
17403070	Adh1	alcohol dehydrogenase 1 (class I)	2842.412	3064.275	3323.16	2762.522	2666.589	2942.262
17403114	Adh4	alcohol dehydrogenase 4 (class II), pi polypeptide	515.0576	328.2611	174.4984	342.2271	337.4898	345.2697
17403126	Adh5	alcohol dehydrogenase 5 (class III), chi polypeptide	332.0899	336.0961	306.8777	336.3688	365.3841	354.6126
17403108	Adh6-ps1	alcohol dehydrogenase 6 (class V), pseudogene 1	156.8357	70.45597	70.16648	131.5165	108.7145	83.45809
17403084	Adh6a	alcohol dehydrogenase 6A (class V)	3.783024	4.229333	3.786356	4.417561	5.550124	4.253263
17403096	Adh6b	alcohol dehydrogenase 6B (class V)	4.418725	4.207578	4.224176	4.526105	4.260964	4.273891
17403058	Adh7	alcohol dehydrogenase 7 (class IV), mu or sigma polypeptide	8.205265	8.272079	7.933294	9.524148	8.272079	6.347815
17211004	Adhfe1	alcohol dehydrogenase, iron containing, 1	216.2461	263.4545	278.5901	251.5428	240.3478	241.3579
17413674	Aldh1b1	aldehyde dehydrogenase 1 family, member B1	49.15342	57.52192	70.60116	59.06043	62.57958	66.67158
17460831	Aldh1l1	aldehyde dehydrogenase 1 family, member L1	580.2778	595.5216	583.8484	668.4473	645.5567	727.9573
17243544	Aldh1l2	aldehyde dehydrogenase 1 family, member L2	5.508731	5.67669	5.402024	5.830097	6.11304	5.22286
17490644	Aldh16a1	aldehyde dehydrogenase 16 family, member A1	26.01461	26.14748	24.91996	25.38633	24.51121	24.77392
17364521	Aldh18a1	aldehyde dehydrogenase 18 family, member A1	5.183944	5.880423	6.506988	6.100933	6.50541	5.67791
17452231	Aldh2	aldehyde dehydrogenase 2, mitochondrial	2233.251	2063.089	1990.272	1994.097	1836.814	2021.752
17361041	Aldh3b1	aldehyde dehydrogenase 3 family, member B1	16.39291	15.08727	19.99282	17.91964	19.58395	11.02915
17356028	Aldh3b2	aldehyde dehydrogenase 3 family, member B2	13.524	13.524	13.9234	13.41644	18.25829	20.30833
17356016	Aldh3b3	aldehyde dehydrogenase 3 family, member B3	5.201869	6.645787	6.47904	6.78924	6.992896	6.47904
17420777	Aldh4a1	aldehyde dehydrogenase 4 family, member A1	227.9305	199.646	188.5064	196.6355	198.6879	237.0948
17232025	Aldh8a1	aldehyde dehydrogenase 8 family, member A1	532.6891	539.6312	463.5496	440.7388	422.6911	424.7205
17219096	Aldh9a1	aldehyde dehydrogenase 9, subfamily A1	483.563	515.0183	461.1141	474.6071	422.129	463.826
17358103	Aldh1a1	aldehyde dehydrogenase family 1, subfamily A1	1856.217	1694.813	1389.317	1742.678	1511.274	1821.991
17519112	Aldh1a2	aldehyde dehydrogenase family 1, subfamily A2	4.416826	4.877561	5.400189	5.045055	6.098101	6.064758
17491941	Aldh1a3	aldehyde dehydrogenase family 1, subfamily A3	5.391886	6.90107	5.082753	5.059799	5.059799	5.972107
17363429	Aldh1a7	aldehyde dehydrogenase family 1, subfamily A7	455.2894	444.5926	347.4713	414.8138	338.2603	405.8464
17250567	Aldh3a1	aldehyde dehydrogenase family 3, subfamily A1	4.33818	3.253314	4.045029	4.043851	4.404597	5.188221
17263724	Aldh3a2	aldehyde dehydrogenase family 3, subfamily A2	668.7902	830.4503	727.4831	682.3509	477.5838	642.3296

17282518	Aldh6a1	aldehyde dehydrogenase family 6, subfamily A1	718.3285	656.0991	589.1863	683.8518	613.0751	670.345
17354434	Aldh7a1	aldehyde dehydrogenase family 7, member A1	629.9485	700.6888	660.2323	686.7732	572.5508	630.0271
17212982	Aox1	aldehyde oxidase 1	179.5547	164.1795	128.7207	153.8414	106.4181	114.4275
17213101	Aox2	aldehyde oxidase 2	5.145139	4.989828	6.287864	6.123727	6.094256	5.649213
17213021	Aox3	aldehyde oxidase 3	864.283	512.355	371.4154	630.0183	662.8736	593.3666
17213060	Aox4	aldehyde oxidase 4	2.642494	2.394625	2.470221	2.750454	2.636785	2.770257
17291361	Aldh5a1	aldhehyde dehydrogenase family 5, subfamily A1	92.43325	97.68533	90.49461	97.87239	80.62079	104.7735
17428684	Akr1a1	aldo-keto reductase family 1, member A1 (aldehyde reductase)	317.4994	347.1343	294.6299	325.4743	323.9237	319.9849
17457143	Akr1b10	aldo-keto reductase family 1, member B10 (aldose reductase)	6.930887	7.518423	8.894047	9.992738	8.382961	7.109017
17465711	Akr1b3	aldo-keto reductase family 1, member B3 (aldose reductase)	17.22151	21.90487	23.35598	13.54971	16.24935	19.82185
17457158	Akr1b7	aldo-keto reductase family 1, member B7	2.860148	2.945005	2.819658	3.444322	2.549987	2.602269
17457128	Akr1b8	aldo-keto reductase family 1, member B8	5.791994	3.695757	3.498921	3.359881	3.119057	3.630555
17290275	Akr1c12	aldo-keto reductase family 1, member C12	16.83393	20.48506	16.44925	17.21507	20.7467	22.77501
17284936	Akr1c13	aldo-keto reductase family 1, member C13	60.18992	67.76789	74.49285	74.07065	76.64873	87.85728
17284919	Akr1c14	aldo-keto reductase family 1, member C14	283.0906	276.4433	230.0877	251.9494	245.5989	304.6967
17290263	Akr1c18	aldo-keto reductase family 1, member C18	16.00144	11.42427	7.068578	13.62949	17.34755	21.99174
17284948	Akr1c19	aldo-keto reductase family 1, member C19	18.57049	17.34442	15.31836	16.01043	11.36637	12.25221
17290288	Akr1c20	aldo-keto reductase family 1, member C20	45.55918	30.87998	22.84157	39.75212	36.40084	42.42462
17284981	Akr1c21	aldo-keto reductase family 1, member C21	2.783307	2.685387	2.763583	2.763583	3.512723	2.841239
17284965	Akr1c6	aldo-keto reductase family 1, member C6	43.30255	42.91083	34.36124	59.43208	46.1446	39.43842
17223813	Akr1cl	aldo-keto reductase family 1, member C-like	4.222625	4.009688	3.248357	4.208686	3.802953	3.015673
17457330	Akr1d1	aldo-keto reductase family 1, member D1	216.5821	226.2917	258.3566	272.1371	298.4303	335.0935
17290301	Akr1e1	aldo-keto reductase family 1, member E1	65.34342	65.33028	68.2719	67.63462	60.81056	67.30912
17420604	Akr7a5	aldo-keto reductase family 7, member A5 (aflatoxin aldehyde reductase)	73.16897	76.76091	72.13762	68.81351	77.47506	74.09786
17426549	Aldoart1	aldolase 1 A, retrogene 1	2.801016	2.627881	2.542381	3.16205	2.568596	2.62224
17275567	Aldoart2	aldolase 1 A, retrogene 2	3.375778	2.177466	2.67402	3.23241	2.907683	2.218278
17496408	Aldoa	aldolase A, fructose-bisphosphate	13.30033	12.43194	12.57817	12.52623	13.7686	14.57264
17496427	Aldoa	aldolase A, fructose-bisphosphate	10.76085	9.935138	11.86179	11.48381	7.935109	10.77996
17425233	Aldob	aldolase B, fructose-bisphosphate	2156.954	2172.635	2095.095	2083.798	2026.283	2146.068
17253599	Aldoc	aldolase C, fructose-bisphosphate	33.53673	39.70008	34.20108	32.83631	40.53033	29.16993
17346401	Acer1	alkaline ceramidase 1	3.139076	2.817567	2.834865	2.935548	3.20277	3.286154
17415219	Acer2	alkaline ceramidase 2	31.85637	34.78468	36.95557	30.89454	25.29166	30.89454
17493556	Acer3	alkaline ceramidase 3	18.39255	13.81583	13.36187	12.82449	12.23159	11.15865
17215166	Akp3	alkaline phosphatase 3, intestine, not Mn requiring	3.341116	4.27475	4.795695	3.978019	4.130997	4.218354
17225210	Alpi	alkaline phosphatase, intestinal	4.995607	5.068118	5.783449	4.973219	4.981534	6.404696
17431720	Alpl	alkaline phosphatase, liver/bone/kidney	21.87711	37.11036	49.65063	35.92808	29.13639	27.6217
17225196	Alppl2	alkaline phosphatase, placental-like 2	4.563403	5.020427	4.60372	4.60372	3.290325	4.60372
17282886	Alkbh1	alkB, alkylation repair homolog 1 (E. coli)	12.40036	9.98097	14.05713	12.46154	16.22853	13.83461
17451504	Alkbh2	alkB, alkylation repair homolog 2 (E. coli)	27.73835	27.0966	32.12668	31.21008	27.53854	30.95198
17388551	Alkbh3	alkB, alkylation repair homolog 3 (E. coli)	39.086	42.48583	36.3217	39.41547	36.96121	40.46941
17443437	Alkbh4	alkB, alkylation repair homolog 4 (E. coli)	19.62866	23.38319	17.86582	19.62866	19.05041	19.62866
17250460	Alkbh5	alkB, alkylation repair homolog 5 (E. coli)	121.1105	95.24424	91.78268	81.38269	92.45183	88.13725
17476335	Alkbh6	alkB, alkylation repair homolog 6 (E. coli)	23.69449	20.45342	23.1285	21.18909	16.25363	26.47484
17338934	Alkbh7	alkB, alkylation repair homolog 7 (E. coli)	16.82915	23.03746	16.71023	25.27011	16.66917	23.24502
17514359	Alkbh8	alkB, alkylation repair homolog 8 (E. coli)	30.78509	23.27308	22.04195	21.92006	22.43797	19.46445
17275069	Agmo	alkylglycerol monooxygenase	138.6881	169.1841	166.3306	143.7083	143.4844	168.2194
17372161	Agps	alkylglycerone phosphate synthase	26.99836	24.56275	23.2918	23.73477	22.24039	20.49642
17280368	Allc	allantoicase	3.61827	3.40329	4.562675	3.953526	4.844608	4.085946
17344289	Aif1	allograft inflammatory factor 1	10.54517	9.675525	10.54517	9.131045	14.30492	10.07683
17369613	Aif1l	allograft inflammatory factor 1-like	12.313	12.313	11.80618	12.22642	10.13314	13.29931
17435963	Atraid	all-trans retinoic acid induced differentiation factor	101.4011	105.9361	118.9936	104.8352	133.455	136.145
17426237	Ambp	alpha 1 microglobulin/bikunin	1549.632	1493.157	1400.902	1247.419	1538.139	1256.896
17418881	A3galt2	alpha 1,3-galactosyltransferase 2 (isoglobotriaosylceramide synthase)	7.626336	7.545727	7.648676	8.530203	8.347649	7.767929
17319801	A4galt	alpha 1,4-galactosyltransferase	7.990911	8.859161	8.467112	10.89066	8.577754	7.422576
17518256	Aagab	alpha- and gamma-adaptin binding protein	21.78223	20.30031	18.90532	25.07087	24.76241	24.28275
17438903	Afp	alpha fetoprotein	13.56014	11.43172	12.33905	13.67378	15.03643	12.6885
17357280	Ganab	alpha glucosidase 2 alpha neutral subunit	172.2977	148.6371	149.5029	150.5553	154.7483	142.6771
17543930	Atrx	alpha thalassemia/mental retardation syndrome X-linked homolog (human)	64.03062	65.97283	60.187	64.91365	66.20233	64.03062
17344472	Atat1	alpha tubulin acetyltransferase 1	20.38604	23.62447	27.17391	26.66102	17.67257	21.70492
17520736	A4gnt	alpha-1,4-N-acetylglucosaminyltransferase	3.599758	3.160165	3.729055	3.67397	4.312995	3.729055
17317398	A1bg	alpha-1-B glycoprotein	4.740043	4.478265	4.47417	4.929803	3.876111	5.519968
17443710	Azgp1	alpha-2-glycoprotein 1, zinc	2407.366	2378.374	2305.478	2227.145	2354.557	2208.667
17324322	Ahsg	alpha-2-HS-glycoprotein	5136.928	5357.002	5735.526	5005.505	5830.969	5431.895
17462492	A2m	alpha-2-macroglobulin	3.749167	4.95646	5.728351	5.254706	4.861461	4.325869
17410204	Alpk1	alpha-kinase 1	7.793817	8.829612	8.416148	8.428524	8.852347	6.883966
17355060	Alpk2	alpha-kinase 2	3.191291	4.185326	2.99397	3.378634	3.300398	3.454795
17479628	Alpk3	alpha-kinase 3	4.055697	4.442772	4.882568	3.597538	4.230564	4.276291
17310349	Amacr	alpha-methylacyl-CoA racemase	377.3344	297.959	277.555	359.2215	291.2794	303.3081
17256502	Naglu	alpha-N-acetylglucosaminidase (Sanfilippo disease IIIB)	34.30269	38.8669	38.55511	33.32886	42.10209	31.51114
17296653	Gm3002	alpha-takusan pseudogene	2.763851	2.301317	2.621623	2.552534	2.732743	4.113938
17545078	Ammecr1	Alport syndrome, mental retardation, midface hypoplasia and elliptocytosis chrom	5.848757	5.386134	5.398318	5.386134	5.694424	5.905012
17522504	Als2cl	ALS2 C-terminal like	21.70687	15.52703	19.62866	20.8466	12.9766	18.98476
17460307	Alms1	Alstrom syndrome 1	8.314215	9.79829	11.44964	10.22083	10.51961	9.699834
17460333	Alms1-ps2	Alstrom syndrome 1, pseudogene 2	2.084325	2.117042	2.137597	2.722024	1.98923	2.727709
17259445	Aspscr1	alveolar soft part sarcoma chromosome region, candidate 1 (human)	104.7045	109.1297	117.5874	121.8017	118.9895	130.4741
17244608	Alx1	ALX homeobox 1	5.120306	3.515817	4.20927	5.852853	5.343704	5.120306
17273191	Alyref	Aly/REF export factor	4.845045	4.814113	4.798788	6.168501	5.333601	7.708927
17219374	Alyref2	Aly/REF export factor 2	19.073	28.56111	23.10559	22.10131	21.76126	22.78859
17438759	Ambn	ameloblastin	3.139337	3.854731	3.435524	3.283558	2.837873	2.860823
17546180	Amelx	amelogenin, X-linked	3.512577	3.401615	3.458269	4.215577	3.584172	3.554656
17438749	Amtn	amelotin	4.112085	3.011454	3.221405	4.236638	3.938101	3.339703
17244350	Amdhd1	amidohydrolase domain containing 1	348.2187	342.3006	340.2285	366.706	366.4341	387.357
17341712	Amdhd2	amidohydrolase domain containing 2	30.09746	26.1961	25.06791	28.71296	27.73258	26.73615
17256691	Aoc2	amine oxidase, copper containing 2 (retina-specific)	8.198671	9.187676	9.210376	8.391915	9.188249	8.866901

17256697	Aoc3	amine oxidase, copper containing 3	4.477893	5.590162	3.748569	4.238048	3.847416	3.642023
17458407	Aoc1	amine oxidase, copper-containing 1	4.460761	4.730131	4.6731	4.893749	5.767676	5.305983
17216779	Acmsd	amino carboxymuconate semialdehyde decarboxylase	129.2589	83.62776	108.105	195.9606	155.1608	186.7663
17410424	Aimp1	aminoacyl tRNA synthetase complex-interacting multifunctional protein 1	44.02314	34.00512	39.66075	37.08413	43.2081	40.29671
17454908	Aimp2	aminoacyl tRNA synthetase complex-interacting multifunctional protein 2	29.94576	29.3828	33.74051	27.08702	26.47722	29.94576
17530669	Acy1	aminoacylase 1	29.29556	33.08969	40.04333	32.96196	35.0191	38.57007
17501329	Aadat	aminoadipate aminotransferase	520.5056	365.7667	394.2372	430.4793	360.9162	438.2438
17449008	Aasdh	aminoadipate-semialdehyde dehydrogenase	14.47478	15.02403	15.23914	19.18225	15.23914	13.08562
17523695	Aasdhppt	aminoadipate-semialdehyde dehydrogenase-phosphopantetheinyl transferase	95.04178	54.83405	59.01624	79.22314	51.31078	66.90212
17465080	Aass	aminoadipate-semialdehyde synthase	640.3558	605.0288	665.7515	800.7955	622.0643	835.5834
17426206	Alad	aminolevulinate, delta-, dehydratase	151.297	150.8008	148.0233	149.8817	146.6151	145.8579
17530653	Alas1	aminolevulinic acid synthase 1	677.4121	759.3546	822.5502	421.1703	544.3007	391.4678
17538575	Alas2	aminolevulinic acid synthase 2, erythroid	86.30128	51.0603	57.72443	58.55173	49.21342	29.96279
17521661	Amt	aminomethyltransferase	96.69035	105.9111	113.5898	119.3959	133.2046	132.1397
17268493	Npepps	aminopeptidase puromycin sensitive	54.30338	52.92729	51.02775	51.25	37.29447	45.21815
17380291	Npepl1	aminopeptidase-like 1	81.16917	85.83594	83.59936	85.82702	71.71684	84.09453
17235748	Aes	amino-terminal enhancer of split	670.8145	676.68	674.8766	649.2904	606.9021	617.502
17349162	Ammecr1l	AMME chromosomal region gene 1-like	96.66824	130.7956	126.8456	131.007	113.9084	139.0481
17279088	Amn	amnionless	13.31712	13.20287	12.41283	10.77279	9.032467	9.203228
17285488	Amph	amphiphysin	4.20927	4.620343	4.20927	3.94742	4.805545	4.298013
17439029	Areg	amphiregulin	7.492982	9.856121	10.83727	10.0497	9.426486	8.35228
17245709	Os9	amplified in osteosarcoma	272.6223	258.0595	260.6139	257.8235	266.7399	268.1395
17544316	Astx	amplified spermatogenic transcripts X encoded	2.261814	2.997501	2.972754	2.979499	2.867751	2.99908
17537409	Astx1c	amplified spermatogenic transcripts X encoded 1C [Source:MGI Symbol;Acc:MGI:3	3.356971	3.513941	4.672841	4.534835	3.583278	4.387016
17537450	Astx1c	amplified spermatogenic transcripts X encoded 1C [Source:MGI Symbol;Acc:MGI:3	3.356971	3.513941	4.672841	4.534835	3.583278	4.387016
17537488	Astx1c	amplified spermatogenic transcripts X encoded 1C [Source:MGI Symbol;Acc:MGI:3	3.356971	3.513941	4.672841	4.534835	3.583278	4.387016
17544300	Astx2	amplified spermatogenic transcripts X encoded 2 [Source:MGI Symbol;Acc:MGI:37	3.464723	2.575745	2.625842	2.606909	2.625842	2.263068
17537513	Astx3	amplified spermatogenic transcripts X encoded 3 [Source:MGI Symbol;Acc:MGI:37	3.518255	4.439232	4.791708	3.801213	4.007771	4.427063
17537423	Astx4d	amplified spermatogenic transcripts X encoded 4D [Source:MGI Symbol;Acc:MGI:3	3.339518	3.639777	5.20941	4.234847	3.634911	4.13157
17537437	Astx4d	amplified spermatogenic transcripts X encoded 4D [Source:MGI Symbol;Acc:MGI:3	3.339518	3.639777	5.20941	4.234847	3.634911	4.13157
17537483	Astx4d	amplified spermatogenic transcripts X encoded 4D [Source:MGI Symbol;Acc:MGI:3	3.339518	3.639777	5.20941	4.234847	3.634911	4.13157
17537502	Astx4d	amplified spermatogenic transcripts X encoded 4D [Source:MGI Symbol;Acc:MGI:3	3.339518	3.639777	5.20941	4.234847	3.634911	4.13157
17544290	Astx6	amplified spermatogenic transcripts X encoded 6	2.739381	2.433883	3.010827	3.703322	2.73263	2.205185
17409575	Amy1	amylase 1, salivary	211.1201	170.4612	147.9371	198.6273	227.2285	257.181
17409513	Amy2a5	amylase 2a5	22.6497	17.11655	16.67633	17.37848	25.97749	21.39764
17409526	Amy2a5	amylase 2a5	20.96237	17.43398	17.93667	17.59569	26.05449	22.81183
17409540	Amy2a5	amylase 2a5	30.12146	24.15182	23.81387	24.43081	36.19077	31.22934
17409555	Amy2a5	amylase 2a5	22.40579	18.06368	17.20541	18.98003	25.40499	22.22251
17409502	Amy2b	amylase 2b	3.776344	2.420844	4.082237	2.152331	3.054508	2.725857
17409753	Agl	amylo-1,6-glucosidase, 4-alpha-glucanotransferase	59.35715	59.44279	60.02161	61.19916	63.42238	61.31465
17331669	App	amyloid beta (A4) precursor protein	113.7261	139.6702	187.6838	119.7615	105.0877	104.5863
17358240	Apba1	amyloid beta (A4) precursor protein binding, family A, member 1	3.364122	3.438454	4.133919	3.154803	3.773858	4.77202
17478889	Apba2	amyloid beta (A4) precursor protein-binding, family A, member 2	4.20962	4.360822	4.20962	4.664039	3.23483	3.867604
17235647	Apba3	amyloid beta (A4) precursor protein-binding, family A, member 3	30.12021	26.76118	26.76118	28.2653	25.46846	24.63453
17494510	Apbb1	amyloid beta (A4) precursor protein-binding, family B, member 1	8.495801	7.931699	8.54707	7.931699	8.549144	7.931699
17367533	Apbb1ip	amyloid beta (A4) precursor protein-binding, family B, member 1 interacting prote	4.619158	3.255096	3.577347	2.788772	3.255096	3.255096
17367536	Apbb1ip	amyloid beta (A4) precursor protein-binding, family B, member 1 interacting prote	12.68587	12.42454	13.36835	14.0903	14.63104	13.02553
17448415	Apbb2	amyloid beta (A4) precursor protein-binding, family B, member 2	39.74606	48.15721	50.52025	38.26358	42.17657	38.59923
17353716	Apbb3	amyloid beta (A4) precursor protein-binding, family B, member 3	9.054532	13.12422	15.8755	13.43766	14.70189	16.28758
17489052	Aplp1	amyloid beta (A4) precursor-like protein 1	11.12081	12.29347	12.62271	12.05024	11.56746	13.07936
17525263	Aplp2	amyloid beta (A4) precursor-like protein 2	349.3163	419.4747	421.2316	332.1889	369.0136	349.9609
17267155	Appbp2	amyloid beta precursor protein (cytoplasmic tail) binding protein 2	64.55245	53.81812	46.82023	47.09461	46.183	56.19695
17254532	Appbp2os	amyloid beta precursor protein (cytoplasmic tail) binding protein 2, opposite strar	15.41999	14.96614	17.69684	22.67971	13.54464	16.31797
17223532	Als2	amyotrophic lateral sclerosis 2 (juvenile)	75.88643	74.88111	72.05517	73.80415	80.5828	65.54619
17223473	Als2cr11	amyotrophic lateral sclerosis 2 (juvenile) chromosome region, candidate 11 (huma	2.576388	2.55588	2.327466	2.809571	3.782893	2.649206
17223429	Als2cr12	amyotrophic lateral sclerosis 2 (juvenile) chromosome region, candidate 12 (huma	4.995469	5.831876	5.109847	6.346099	3.729055	4.578897
17391422	Anapc1	anaphase promoting complex subunit 1	59.77706	58.1223	55.62109	56.85479	51.74294	54.97486
17502781	Anapc10	anaphase promoting complex subunit 10	11.54503	17.06656	16.42214	13.39953	20.44803	17.03441
17259422	Anapc11	anaphase promoting complex subunit 11	20.43089	18.62786	21.08522	20.58583	20.72126	25.86358
17520851	Anapc13	anaphase promoting complex subunit 13	393.4946	384.9187	357.4283	373.9107	511.7765	420.5315
17241038	Anapc16	anaphase promoting complex subunit 16	73.93113	70.82319	66.15691	73.32738	71.1578	63.41666
17367892	Anapc2	anaphase promoting complex subunit 2	45.79315	47.42777	45.79315	45.79315	42.85436	39.01021
17437425	Anapc4	anaphase promoting complex subunit 4	25.72252	21.27386	26.67451	28.23984	33.29445	30.29085
17442107	Anapc7	anaphase promoting complex subunit 7	51.67663	40.19628	34.4736	36.09332	38.08128	43.98653
17480962	Anapc15	anaphase prompoting complex C subunit 15	7.891831	7.503429	7.870689	7.870689	7.870689	9.883486
17244524	Anapc15-ps	anaphase prompoting complex C subunit 15, pseudogene [Source:MGI Symbol;Ac	13.86129	22.01462	20.32523	26.78419	13.87225	15.86274
17452477	Anapc5	anaphase-promoting complex subunit 5	117.9433	126.6137	114.6053	101.8258	108.0054	113.7064
17347082	Alk	anaplastic lymphoma kinase	7.040022	7.076176	7.955541	8.6494	6.53832	7.074019
17459888	Aup1	ancient ubiquitous protein 1	314.5422	309.6404	309.6404	300.3269	294.6912	286.2278
17292084	Adtrp	androgen dependent TFPI regulating protein	82.94252	70.31643	73.85839	75.14108	70.16793	79.22534
17536436	Ar	androgen receptor	4.20927	4.20927	3.562179	4.486063	4.403834	3.878369
17239420	Aig1	androgen-induced 1	28.95278	36.22742	31.12237	30.06119	31.00438	28.29686
17239184	Adgb	androglobin	9.682905	11.96634	11.95324	12.69158	14.09821	12.6475
17282760	Angel1	angel homolog 1 (Drosophila)	8.89454	11.34599	8.945239	10.49696	9.9412	11.78092
17220751	Angel2	angel homolog 2 (Drosophila)	14.41661	13.03059	15.61748	17.06821	17.87127	16.75907
17224256	Aamp	angio-associated migratory protein	81	80.85584	71.90499	78.94631	93.32489	83.59762
17295093	Aggf1	angiogenic factor with G patch and FHA domains 1	56.38932	51.06313	55.47055	55.78982	57.33126	57.26612
17306102	Ang2	angiogenin, ribonuclease A family, member 2	2.364377	2.403905	1.902637	2.138753	2.434204	2.005539
17306143	Ang4	angiogenin, ribonuclease A family, member 4	3.28727	2.754213	3.083866	4.636544	3.750362	3.263862
17299093	Ang5	angiogenin, ribonuclease A family, member 5	1.942857	1.950292	1.908594	1.887792	2.622277	2.129213
17305527	Ang6	angiogenin, ribonuclease A family, member 6	3.913719	3.543881	4.109264	3.630404	5.731489	3.438692
17299575	Ang	angiogenin, ribonuclease, RNase A family, 5	288.9908	323.5848	303.6804	272.2881	324.5147	305.5617
17545159	Amot	angiomotin	14.92315	21.26192	18.44682	14.90185	19.83629	18.54258
17524028	Amotl1	angiomotin-like 1	22.62789	25.2358	27.51488	24.94243	19.96803	23.16123

17520856	Amotl2	angiomotin-like 2	25.05871	21.86558	25.40305	24.9682	21.94089	25.8496
17316780	Angpt1	angiopoietin 1	4.323831	4.148644	3.438645	4.207607	4.163855	4.945958
17507799	Angpt2	angiopoietin 2	3.411935	4.338481	3.347723	3.287019	4.808244	5.051634
17377638	Angpt4	angiopoietin 4	7.258543	6.794465	7.22346	6.366277	5.908205	7.127519
17218449	Angptl1	angiopoietin-like 1	3.416171	3.667711	3.653958	4.408384	4.504853	3.315186
17370043	Angptl2	angiopoietin-like 2	12.49302	13.66932	12.17078	14.10828	13.18467	14.19666
17415728	Angptl3	angiopoietin-like 3	396.9655	397.7643	423.4506	405.9983	614.0235	480.2427
17343628	Angptl4	angiopoietin-like 4	198.5282	235.9668	274.2409	228.8772	206.5604	210.1069
17524515	Angptl6	angiopoietin-like 6	24.74611	22.93613	24.24642	24.24021	27.68457	24.20243
17432976	Angptl7	angiopoietin-like 7	9.906943	10.48409	13.43729	9.852979	12.02971	10.57686
17515335	Angptl8	angiopoietin-like 8	32.21454	34.75961	30.93247	40.43969	45.05234	36.2347
17257444	Ace	angiotensin I converting enzyme (peptidyl-dipeptidase A) 1	8.867024	7.698994	7.66736	9.127375	6.218127	9.997934
17539494	Ace2	angiotensin I converting enzyme (peptidyl-dipeptidase A) 2	3.745764	3.631486	3.540738	3.703245	3.737076	3.737076
17286271	Agtr1a	angiotensin II receptor, type 1a	122.3322	182.1824	179.825	149.2751	169.868	154.0217
17404366	Agtr1b	angiotensin II receptor, type 1b	3.263862	3.263862	3.896152	3.433952	3.77493	3.133077
17533746	Agtr2	angiotensin II receptor, type 2	3.88488	4.20927	4.20927	4.20927	4.854424	4.20927
17432895	Agtrap	angiotensin II, type I receptor-associated protein	6.947626	8.769594	9.548605	10.04433	9.118332	8.162833
17514078	Agt	angiotensinogen (serpin peptidase inhibitor, clade A, member 8)	313.7583	312.4432	378.9044	286.4283	339.9739	354.114
17524969	Anln	anillin, actin binding protein	4.029051	3.883991	4.316614	3.786513	3.533554	4.005105
17405389	Ankub1	ankrin repeat and ubiquitin domain containing 1	3.761088	4.435664	4.597979	4.336857	3.539732	5.903498
17500005	Ank1	ankyrin 1, erythroid	11.29287	8.786082	11.19234	9.384566	9.475421	8.429517
17410130	Ank2	ankyrin 2, brain	7.337456	7.337456	8.300338	7.23328	8.052741	7.337456
17234066	Ank3	ankyrin 3, epithelial	23.34853	27.32804	26.93481	25.17727	26.69819	24.78903
17224154	Ankar	ankyrin and armadillo repeat containing	3.336164	3.249573	3.309607	3.279782	3.560871	3.268254
17468765	Abtb1	ankyrin repeat and BTB (POZ) domain containing 1	29.58697	25.77767	33.20779	30.14799	34.99799	29.76098
17373754	Abtb2	ankyrin repeat and BTB (POZ) domain containing 2	41.73136	36.49811	44.60984	45.50395	37.2152	45.81697
17528255	Ankdd1a	ankyrin repeat and death domain containing 1A	5.37096	5.003439	4.192585	5.478952	6.217049	4.634199
17528271	Ankdd1a	ankyrin repeat and death domain containing 1A	7.577175	10.11911	7.088842	7.849441	5.655012	9.08093
17295196	Ankdd1b	ankyrin repeat and death domain containing 1B	6.299905	6.386653	6.263113	5.457613	5.482485	7.265741
17376798	Ankef1	ankyrin repeat and EF-hand domain containing 1	3.173313	2.399677	2.747558	2.425429	3.056817	2.704103
17376805	Ankef1	ankyrin repeat and EF-hand domain containing 1	4.280413	4.346578	4.437048	3.552021	3.952266	3.176857
17252408	Ankfy1	ankyrin repeat and FYVE domain containing 1	71.99726	72.32036	72.28879	71.99726	67.09903	64.90555
17445280	Ankib1	ankyrin repeat and IBR domain containing 1	77.63848	73.21463	72.96891	63.86307	60.9608	64.73931
17349671	Ankhd1	ankyrin repeat and KH domain containing 1	68.95819	81.10393	65.42817	70.18837	70.18837	77.21183
17349713	Ankhd1	ankyrin repeat and KH domain containing 1	62.10209	66.72798	72.18264	53.60367	75.78445	79.57206
17526743	Ankk1	ankyrin repeat and kinase domain containing 1	4.337519	4.303797	4.745598	4.907064	4.061155	6.941801
17502179	Ankle1	ankyrin repeat and LEM domain containing 1	12.57843	12.76729	15.84911	11.50086	10.32779	14.79707
17440439	Ankle2	ankyrin repeat and LEM domain containing 2	51.87868	44.31644	38.83027	45.16959	48.70777	45.0564
17225603	Ankmy1	ankyrin repeat and MYND domain containing 1	4.663328	4.890358	4.890358	4.824301	4.561632	5.127516
17275029	Ankmy2	ankyrin repeat and MYND domain containing 2	23.53131	29.30368	28.82303	31.18478	28.66583	25.3983
17335204	Anks1	ankyrin repeat and SAM domain containing 1	23.39016	19.32116	24.42827	21.16179	18.23186	22.6351
17215788	Asb1	ankyrin repeat and SOCS box-containing 1	12.93941	14.56627	15.23914	13.5529	18.36478	18.35015
17446220	Asb10	ankyrin repeat and SOCS box-containing 10	3.513089	4.537382	3.985636	3.52905	3.279214	4.060141
17539560	Asb11	ankyrin repeat and SOCS box-containing 11	4.595176	4.023581	3.965465	4.468777	3.98583	3.649933
17543315	Asb12	ankyrin repeat and SOCS box-containing 12	2.612227	2.573586	2.606909	2.693216	2.829682	2.606909
17284894	Asb13	ankyrin repeat and SOCS box-containing 13	104.8403	87.64197	106.2891	68.45481	57.66124	56.28935
17297999	Asb14	ankyrin repeat and SOCS box-containing 14	5.927279	5.74131	5.999574	6.603633	6.426129	5.74131
17456414	Asb15	ankyrin repeat and SOCS box-containing 15	3.748647	3.477595	4.221103	3.892157	4.222508	2.953284
17256924	Asb16	ankyrin repeat and SOCS box-containing 16	7.254419	6.739166	6.800072	5.564106	5.868411	5.928841
17403735	Asb17	ankyrin repeat and SOCS box-containing 17	3.860372	3.165163	3.185147	3.28588	3.374117	3.211372
17411274	Asb17os	ankyrin repeat and SOCS box-containing 17, opposite strand	3.330203	3.136314	2.907582	4.496212	3.263862	2.310668
17225379	Asb18	ankyrin repeat and SOCS box-containing 18	11.93271	9.61822	11.50086	11.7681	15.50434	12.85199
17283516	Asb2	ankyrin repeat and SOCS box-containing 2	12.42919	9.507328	12.81312	12.42919	8.940032	8.508472
17248196	Asb3	ankyrin repeat and SOCS box-containing 3	11.84782	12.49397	12.35206	13.69588	18.49649	14.54599
17455903	Asb4	ankyrin repeat and SOCS box-containing 4	4.438893	3.9518	4.304155	3.723889	4.770069	4.384614
17501170	Asb5	ankyrin repeat and SOCs box-containing 5	3.714943	2.67402	2.588058	2.789178	2.564558	2.580517
17383699	Asb6	ankyrin repeat and SOCS box-containing 6	17.09864	18.10116	17.03547	16.00621	14.96977	16.36997
17491961	Asb7	ankyrin repeat and SOCS box-containing 7	13.14899	11.56313	11.54215	14.87327	11.88673	12.24686
17321169	Asb8	ankyrin repeat and SOCS box-containing 8	39.78493	43.75482	35.06816	44.53075	37.14664	44.10346
17539574	Asb9	ankyrin repeat and SOCS box-containing 9	4.638087	4.323321	4.399593	3.572317	3.671145	3.737931
17236439	Anks1b	ankyrin repeat and sterile alpha motif domain containing 1B	2.739989	3.521793	3.114261	3.478873	2.937177	3.114261
17327842	Anks3	ankyrin repeat and sterile alpha motif domain containing 3	14.66739	13.3019	13.12422	13.49263	13.07827	10.16233
17482376	Anks4b	ankyrin repeat and sterile alpha motif domain containing 4B	27.44535	32.29435	29.64958	32.29435	37.23094	29.55991
17425123	Anks6	ankyrin repeat and sterile alpha motif domain containing 6	9.361307	9.361307	10.1025	9.361307	7.517641	8.657557
17214433	Ankzf1	ankyrin repeat and zinc finger domain containing 1	17.09864	21.49054	23.79885	26.04925	18.66275	21.8668
17364176	Ankrd1	ankyrin repeat domain 1 (cardiac muscle)	8.360852	8.761088	6.830731	6.936751	6.261551	7.752706
17507462	Ankrd10	ankyrin repeat domain 10	10.2997	10.1579	12.53708	8.162946	9.310315	8.956965
17513835	Ankrd11	ankyrin repeat domain 11	47.75533	56.37173	53.74654	53.07719	50.70092	49.66439
17346781	Ankrd12	ankyrin repeat domain 12	13.30245	14.31296	15.26864	10.97531	14.09987	12.71747
17361200	Ankrd13d	ankyrin repeat domain 13 family, member D	6.500136	6.504283	6.280503	6.481735	6.448305	6.023074
17440998	Ankrd13a	ankyrin repeat domain 13a	56.4202	44.6318	52.74455	49.28306	49.34288	49.30083
17266223	Ankrd13b	ankyrin repeat domain 13b	28.12946	28.56111	24.67794	26.51671	26.94676	26.38964
17403879	Ankrd13c	ankyrin repeat domain 13c	86.97869	95.1156	82.51989	100.1126	90.59691	100.8565
17367020	Ankrd16	ankyrin repeat domain 16	10.76025	14.27937	16.67032	13.43757	14.65783	17.54733
17449526	Ankrd17	ankyrin repeat domain 17	124.3842	159.2589	149.1738	146.3795	145.3513	146.1859
17359493	Ankrd2	ankyrin repeat domain 2 (stretch responsive muscle)	10.16607	11.04774	13.06378	9.130982	11.56321	16.33019
17364090	Ankrd22	ankyrin repeat domain 22	2.929877	3.072387	2.844372	3.037849	3.253111	2.588184
17222193	Ankrd23	ankyrin repeat domain 23	11.44424	8.422801	9.310251	11.57757	9.638108	10.21927
17235792	Ankrd24	ankyrin repeat domain 24	21.47669	22.98148	21.89197	28.02903	23.34077	23.835
17470196	Ankrd26	ankyrin repeat domain 26	14.63235	11.42174	10.1025	10.48228	8.138766	10.48228
17476819	Ankrd27	ankyrin repeat domain 27 (VPS9 domain)	36.85785	43.78061	49.16126	44.5678	43.38066	38.48782
17304817	Ankrd28	ankyrin repeat domain 28	52.14185	43.24076	48.67233	55.14507	62.88831	56.01689
17352763	Ankrd29	ankyrin repeat domain 29	8.96243	9.067092	7.738103	6.171709	7.338779	7.222258
17315124	Ankrd33	ankyrin repeat domain 33	7.544211	7.524157	7.644065	8.509769	8.220351	7.900533

17316280	Ankrd33b	ankyrin repeat domain 33B	21.95808	23.4317	26.03048	26.03048	25.07243	27.57808
17400617	Ankrd34a	ankyrin repeat domain 34A	5.123501	6.733283	6.872476	6.872476	5.171884	8.923069
17289094	Ankrd34b	ankyrin repeat domain 34B	6.624585	6.413803	6.707755	7.140871	6.971794	6.051866
17529640	Ankrd34c	ankyrin repeat domain 34C	3.905778	2.622313	3.062691	2.953407	2.886037	2.936138
17400682	Ankrd35	ankyrin repeat domain 35	3.343618	3.349115	3.592654	4.10414	2.504426	4.353181
17246980	Ankrd36	ankyrin repeat domain 36	5.916361	5.520121	5.918001	4.616278	6.719202	5.093086
17509139	Ankrd37	ankyrin repeat domain 37	8.321133	8.253351	6.365307	8.01832	10.20794	8.321133
17222205	Ankrd39	ankyrin repeat domain 39	25.05159	28.55517	24.85493	29.04142	27.02776	35.74575
17255215	Ankrd40	ankyrin repeat domain 40	69.08976	69.13144	73.88451	72.77797	69.2163	71.15879
17493312	Ankrd42	ankyrin repeat domain 42	7.869053	6.488972	7.709514	7.868289	7.027249	8.392613
17223169	Ankrd44	ankyrin repeat domain 44	13.2796	19.38193	16.10269	13.90392	17.81845	16.10269
17218694	Ankrd45	ankyrin repeat domain 45	7.465299	9.039669	8.340304	11.22311	8.583643	12.56954
17316495	Ankrd46	ankyrin repeat domain 46	88.49782	88.21793	90.09468	110.1534	76.46089	83.17799
17524087	Ankrd49	ankyrin repeat domain 49	31.24991	27.56107	20.92608	27.93554	30.09684	23.00794
17404947	Ankrd50	ankyrin repeat domain 50	21.30825	22.16129	25.29079	22.16129	19.81801	22.16129
17238449	Ankrd52	ankyrin repeat domain 52	70.45499	54.55388	55.07058	54.95504	56.80476	50.43174
17460124	Ankrd53	ankyrin repeat domain 53	6.992896	7.402692	6.024828	6.457027	7.649153	7.94832
17319109	Ankrd54	ankyrin repeat domain 54	13.74819	18.68774	14.18053	14.94675	13.27785	13.83768
17289864	Ankrd55	ankyrin repeat domain 55	4.581087	4.745631	5.10252	4.745631	4.906622	4.548308
17423756	Ankrd6	ankyrin repeat domain 6	7.522332	9.43361	6.818479	7.627974	6.955528	5.499041
17395099	Ankrd60	ankyrin repeat domain 60	5.695214	5.780603	5.649527	6.270281	6.026707	5.453491
17454897	Ankrd61	ankyrin repeat domain 61	4.27241	3.777522	3.743869	4.966674	4.109178	3.550667
17389736	Ankrd63	ankyrin repeat domain 63	9.587934	10.62418	9.454034	11.74089	10.81484	10.62418
17422691	Ankrd65	ankyrin repeat domain 65	10.88214	12.54456	12.79236	10.02069	7.947769	11.24366
17345085	Ankrd66	ankyrin repeat domain 66	3.600482	4.143012	4.764847	5.822465	4.248637	3.809311
17456293	Ankrd7	ankyrin repeat domain 7	2.839211	2.623586	2.669834	2.524154	3.408436	2.37479
17284049	Ankrd9	ankyrin repeat domain 9	8.581501	7.500777	8.06682	8.854556	9.582685	9.310251
17289396	Ankra2	ankyrin repeat, family A (RFXANK-like), 2	9.538749	11.98892	13.13296	12.90511	12.22506	14.70658
17464986	Asz1	ankyrin repeat, SAM and basic leucine zipper domain containing 1	2.927522	2.927522	2.501737	3.263862	2.972692	3.612155
17267667	Ankfn1	ankyrin-repeat and fibronectin type III domain containing 1	6.350763	4.9909	5.373939	6.300691	4.76313	4.958281
17363407	Anxa1	annexin A1	11.62541	15.3104	15.56831	13.77901	8.103696	12.81481
17509565	Anxa10	annexin A10	2.875175	3.54506	3.267891	3.086667	3.283285	2.706691
17297759	Anxa11	annexin A11	67.12973	74.19705	86.71803	82.44842	60.46926	57.2558
17317284	Anxa13	annexin A13	13.44726	12.22371	13.44362	16.48589	10.92014	13.90672
17518962	Anxa2	annexin A2	27.57387	34.24022	47.06459	28.79268	27.44821	29.38117
17439464	Anxa3	annexin A3	21.16326	23.8246	24.70138	20.41532	18.04541	19.19395
17468551	Anxa4	annexin A4	97.78627	108.6979	96.05175	84.57621	78.73428	90.70625
17404796	Anxa5	annexin A5	262.0648	318.9441	313.1608	282.7748	284.7042	271.9373
17262887	Anxa6	annexin A6	222.2439	225.9814	235.3102	216.8404	176.0669	180.8726
17303804	Anxa7	annexin A7	157.2003	166.0749	162.7327	129.9615	130.0408	146.0016
17298775	Anxa8	annexin A8	5.445823	5.688349	4.700258	4.268059	5.284401	6.683436
17407775	Anxa9	annexin A9	6.729736	4.972599	8.538307	6.708871	9.58149	8.690445
17407794	Anxa9	annexin A9	13.524	14.37958	13.64973	14.85699	11.19847	13.524
17498467	Ano1	anoctamin 1, calcium activated chloride channel	10.97508	9.297159	9.133324	8.905212	8.744161	9.891025
17532418	Ano10	anoctamin 10	40.8972	44.17934	39.46987	40.38955	40.58374	35.238
17463268	Ano2	anoctamin 2	8.286541	6.76819	5.747761	6.943774	6.851406	6.397552
17389161	Ano3	anoctamin 3	3.045629	3.533309	3.713501	3.520133	3.660447	3.445516
17244022	Ano4	anoctamin 4	4.474635	3.112859	2.795856	3.430622	4.623483	3.668881
17244054	Ano4	anoctamin 4	8.117976	8.264457	8.306784	9.128957	11.97663	9.776174
17478435	Ano5	anoctamin 5	3.348488	3.196272	3.339703	3.484424	3.404729	3.57405
17314487	Ano6	anoctamin 6	39.84481	32.55168	44.70084	33.32285	36.27063	32.72248
17215984	Ano7	anoctamin 7	4.362333	4.229274	5.298816	4.818119	3.729055	4.486561
17510318	Ano8	anoctamin 8	7.881444	9.399865	8.445366	9.322972	8.023935	10.26976
17497741	Ano9	anoctamin 9	3.248312	3.263862	3.602204	3.196648	3.83477	3.671857
17473091	Amn1	antagonist of mitotic exit network 1	24.36114	25.30622	19.53623	19.00622	24.99695	19.96194
17275015	Agr2	anterior gradient 2	2.596467	2.998581	3.28481	2.571571	3.339565	2.835592
17275003	Agr3	anterior gradient 3	1.932596	2.27198	2.23349	2.690678	2.468047	2.248184
17228860	Aph1a	anterior pharynx defective 1a homolog (C. elegans)	149.2203	160.5724	159.8129	148.0694	159.2037	137.5355
17400437	Aph1a	anterior pharynx defective 1a homolog (C. elegans)	31.20726	33.02782	33.02782	33.02782	36.13788	28.76116
17528377	Aph1b	anterior pharynx defective 1b homolog (C. elegans)	14.50502	12.41076	14.75733	11.7139	14.31296	12.81925
17528395	Aph1c	anterior pharynx defective 1c homolog (C. elegans)	30.29589	30.60658	39.54737	30.22101	35.24459	34.36124
17468573	Antxr1	anthrax toxin receptor 1	15.27161	13.72115	13.8586	11.17134	13.65247	13.46209
17449939	Antxr2	anthrax toxin receptor 2	101.9756	106.3414	111.3795	104.7212	81.27163	80.31291
17298757	Antxrl	anthrax toxin receptor-like	3.808598	4.076356	4.298967	4.076356	4.076356	4.22649
17497334	Mki67	antigen identified by monoclonal antibody Ki 67	15.94774	15.186	20.234	15.57435	20.70446	18.29021
17324695	Mfi2	antigen p97 (melanoma associated) identified by monoclonal antibodies 133.2 and	7.295971	7.710316	6.58681	8.02566	7.15896	7.380252
17366660	Kin	antigenic determinant of rec-A protein	17.73995	22.44559	18.41884	21.99372	22.27757	20.14961
17235456	Amh	anti-Mullerian hormone	8.105437	8.127378	8.391275	10.64298	10.0483	8.882095
17315422	Amhr2	anti-Mullerian hormone type 2 receptor	5.49565	7.736923	6.812952	5.333934	5.523758	5.226916
17333307	Airn	antisense Igf2r RNA	8.83206	11.64114	11.26582	10.25162	10.41543	7.18023
17233331	Asf1a	anti-silencing function 1A histone chaperone	33.59303	29.34962	29.15093	28.56388	27.95278	36.06331
17503023	Asf1b	anti-silencing function 1B histone chaperone	7.052819	7.031585	6.425684	8.106111	5.983792	6.856774
17316700	Azin1	antizyme inhibitor 1	130.4823	126.6761	133.6559	127.6792	126.6541	146.7575
17430288	Azin2	antizyme inhibitor 2	7.051597	8.661819	9.118648	9.245363	8.790468	11.59869
17460468	Aak1	AP2 associated kinase 1	24.53433	25.71717	30.14131	25.59677	24.34663	20.26081
17373721	Apip	APAF1 interacting protein	25.03683	26.62778	24.03725	27.38986	29.91693	26.62778
17543309	Amer1	APC membrane recruitment 1	14.95743	18.21178	18.21178	13.79066	16.71047	17.10457
17301026	Amer2	APC membrane recruitment 2	5.338965	5.300432	5.039777	5.743022	6.758626	5.300432
17211748	Amer3	APC membrane recruitment 3	5.407006	3.906482	4.136126	4.507852	4.231297	3.647126
17541378	Apln	apelin	3.400955	4.309738	4.02439	3.315623	3.174545	2.989678
17372725	Aplnr	apelin receptor	4.965297	6.096199	4.76564	4.544779	5.045125	5.164111
17509676	Apela	apelin receptor early endogenous ligand	3.006823	2.885757	3.107598	2.929491	3.200573	3.051207
17358664	A1cf	APOBEC1 complementation factor	676.985	563.4056	532.726	548.516	492.4283	521.2205
17516901	Apoa1	apolipoprotein A-I	2128.896	1996.448	2006.604	2021.429	2376.199	1902.829

17406656	Apoa1bp	apolipoprotein A-I binding protein	146.122	154.4033	154.8395	142.9414	172.7617	162.6503
17219242	Apoa2	apolipoprotein A-II	4335.522	4736.843	5028.039	4739.601	4805.897	4836.541
17516908	Apoa4	apolipoprotein A-IV	1507.675	1680.029	1974.498	1533.497	1691.163	1189.476
17516915	Apoa5	apolipoprotein A-V	212.6667	207.4055	195.7393	206.6965	202.2884	271.2388
17273864	Apob	apolipoprotein B	3920.304	4073.604	3842.581	3617.783	3631.601	3679.898
17470580	Apobec1	apolipoprotein B mRNA editing enzyme, catalytic polypeptide 1	102.7554	77.92585	97.61764	71.44148	76.28936	75.67002
17345797	Apobec2	apolipoprotein B mRNA editing enzyme, catalytic polypeptide 2	5.901289	6.608441	4.920892	6.171709	5.667608	5.571941
17313050	Apobec3	apolipoprotein B mRNA editing enzyme, catalytic polypeptide 3	10.42164	11.31175	12.88401	8.443727	7.889479	10.2032
17218249	Apobec4	apolipoprotein B mRNA editing enzyme, catalytic polypeptide-like 4 (putative)	2.961771	2.844963	2.627134	3.01016	2.838601	2.354744
17483048	Apobr	apolipoprotein B receptor	20.30707	19.58178	23.98233	19.2022	15.02747	16.21155
17487374	Apoc1	apolipoprotein C-I	2468.16	2477.656	2554.473	2626.271	2670.141	2654.749
17487361	Apoc2	apolipoprotein C-II	408.1088	408.5135	482.6956	436.1438	356.8663	418.7223
17526623	Apoc3	apolipoprotein C-III	83.01269	93.04779	105.2583	106.8298	115.5593	118.5301
17487369	Apoc4	apolipoprotein C-IV	665.7654	911.0649	970.9815	919.0758	1027.705	892.6433
17329759	Apod	apolipoprotein D	3.462322	2.698018	3.338801	3.823304	3.356971	3.045354
17487381	Apoe	apolipoprotein E	179.9547	195.5753	218.2957	197.9607	181.6276	197.2894
17238363	Apof	apolipoprotein F	737.0456	709.2203	696.8382	724.0071	865.914	746.1645
17257743	Apoh	apolipoprotein H	1488.527	1417.359	1273.585	1347.743	1484.036	1545.202
17312661	Apol10a	apolipoprotein L 10A	6.171709	5.068891	4.035647	4.715465	4.477396	2.953407
17318807	Apol10b	apolipoprotein L 10B	3.399518	3.823755	3.15218	3.238622	4.295541	3.1823
17312669	Apol11a	apolipoprotein L 11a	1.904589	2.029452	1.957823	2.26299	1.790509	1.994158
17318815	Apol11b	apolipoprotein L 11b	6.110244	5.103758	5.740634	3.426439	5.429277	3.285653
17312637	Apol6	apolipoprotein L 6	3.935672	5.240738	3.900016	5.076028	4.181614	4.431669
17318772	Apol7a	apolipoprotein L 7a	112.8029	109.8667	103.9445	124.3101	113.7135	137.7568
17318787	Apol7b	apolipoprotein L 7b	26.58517	21.31726	23.44421	30.04306	23.40752	36.70412
17318794	Apol7c	apolipoprotein L 7c	6.739866	4.538891	4.700649	4.617046	2.847094	5.231664
17312675	Gm8221	apolipoprotein L 7c pseudogene	3.95827	5.107616	3.95827	4.554492	3.29818	3.608227
17214009	Apol7d	apolipoprotein L 7d	3.579678	3.536421	3.497539	4.059916	3.707843	4.193021
17312679	Apol7e	apolipoprotein L 7e	12.28376	14.00636	13.39423	9.670564	14.31296	13.56841
17318822	Apol8	apolipoprotein L 8	6.429382	7.93995	5.278983	6.429382	6.610824	9.310251
17318780	Apol9a	apolipoprotein L 9a	194.8135	139.9182	166.8607	192.1195	139.8387	198.8298
17312686	Apol9b	apolipoprotein L 9b	367.7827	259.4603	303.8952	345.229	274.53	322.3089
17463727	Apold1	apolipoprotein L domain containing 1	20.17868	25.55423	17.61009	16.08975	15.00967	15.43764
17344242	Apom	apolipoprotein M	134.9246	112.6302	134.4392	145.8913	153.8382	141.67
17238359	Apon	apolipoprotein N	347.8786	510.4302	591.6786	476.9354	606.3849	466.4375
17536312	Apoo	apolipoprotein O	40.04194	27.80881	27.49448	29.49851	27.69435	21.53498
17296082	Apoo-ps	apolipoprotein O, pseudogene	96.16433	89.30258	79.60295	106.147	51.62257	79.34239
17537248	Apool	apolipoprotein O-like	70.62514	69.4932	70.29499	65.28366	66.80295	70.32667
17279198	Apopt1	apoptogenic, mitochondrial 1	18.95688	17.21256	18.94191	17.88428	21.6762	18.95688
17267021	Aatf	apoptosis antagonizing transcription factor	39.36032	38.76116	38.76116	35.6542	42.92005	40.35121
17479303	Aen	apoptosis enhancing nuclease	21.24719	19.42873	16.02842	25.68546	17.11358	19.42873
17388632	Api5	apoptosis inhibitor 5	105.37	76.84609	81.6126	80.16108	88.75152	84.43095
17282611	Arel1	apoptosis resistant E3 ubiquitin protein ligase 1	111.8576	134.6696	122.0239	120.2106	87.77511	103.2909
17374304	Aven	apoptosis, caspase activation inhibitor	18.68217	20.21802	14.59447	18.7243	16.12291	20.73419
17272945	Aatk	apoptosis-associated tyrosine kinase	8.0978	8.699787	8.949704	10.30013	8.345162	7.396435
17541417	Aifm1	apoptosis-inducing factor, mitochondrion-associated 1	113.9376	102.9878	103.4895	110.9695	110.8665	111.2375
17233751	Aifm2	apoptosis-inducing factor, mitochondrion-associated 2	31.87846	38.51798	39.76811	39.1489	39.12205	39.42339
17323479	Aifm3	apoptosis-inducing factor, mitochondrion-associated 3	9.142445	10.23685	9.432826	10.88795	9.310251	8.877233
17433029	Apitd1	apoptosis-inducing, TAF9-like domain 1	6.287864	7.355177	6.287864	6.943774	6.287864	6.287864
17306440	Acin1	apoptotic chromatin condensation inducer 1	44.08329	51.67448	52.86973	53.49121	50.74838	53.71712
17244140	Apaf1	apoptotic peptidase activating factor 1	9.576444	11.75693	13.52409	10.11848	11.50086	15.09323
17424042	Aptx	aprataxin	12.79165	17.95446	15.55137	15.55137	15.30796	17.23525
17468633	Aplf	aprataxin and PNKP like factor	7.208854	7.110544	7.208854	7.208854	7.208854	7.643535
17299542	Apex1	apurinic/apyrimidinic endonuclease 1	36.38066	35.67669	31.01125	35.14141	40.83544	38.53507
17545306	Apex2	apurinic/apyrimidinic endonuclease 2	16.61564	25.69454	18.97016	24.84201	20.02125	24.62787
17458813	Aqp1	aquaporin 1	65.88463	49.1991	69.92614	63.78388	31.33102	41.13287
17493461	Aqp11	aquaporin 11	78.01394	77.4085	96.13368	91.93103	97.15996	100.571
17215879	Aqp12	aquaporin 12	7.558395	9.761684	7.613923	7.511026	7.180927	10.89127
17314833	Aqp2	aquaporin 2	8.15763	7.384806	10.35561	10.71671	10.35561	8.420524
17424119	Aqp3	aquaporin 3	4.718669	6.999054	6.322258	5.375094	5.92995	5.773555
17352862	Aqp4	aquaporin 4	17.24636	15.61348	8.174724	13.80798	8.67606	11.64646
17314840	Aqp5	aquaporin 5	25.18163	18.16841	18.57165	23.19947	18.61586	20.27542
17314848	Aqp6	aquaporin 6	4.01461	4.56296	5.098525	4.636737	4.625919	3.542996
17424105	Aqp7	aquaporin 7	15.75535	21.99083	17.31583	17.36523	11.83649	11.48143
17482897	Aqp8	aquaporin 8	30.4329	26.06478	23.8279	25.22706	30.78232	33.29838
17528644	Aqp9	aquaporin 9	294.7708	292.544	273.3452	274.1326	254.7838	257.9379
17389522	Aqr	aquarius	36.6167	35.34098	39.67147	36.32976	36.06281	33.92296
17265193	Alox12	arachidonate 12-lipoxygenase	6.277499	6.168501	6.752445	6.541539	6.168501	6.457295
17251583	Alox12b	arachidonate 12-lipoxygenase, 12R type	4.178804	4.562992	5.760923	4.574741	4.871412	4.681043
17265229	Alox15	arachidonate 15-lipoxygenase	7.159555	8.959573	6.909781	6.476615	7.253463	6.540224
17470031	Alox5	arachidonate 5-lipoxygenase	4.955017	4.535495	4.112854	4.535495	3.72815	4.907778
17444961	Alox5ap	arachidonate 5-lipoxygenase activating protein	22.77707	36.96479	33.32147	22.73054	26.29178	27.36291
17264521	Alox8	arachidonate 8-lipoxygenase	6.024034	6.083831	6.830636	5.86341	7.026323	6.46016
17251565	Aloxe3	arachidonate lipoxygenase 3	3.751792	3.871573	3.600831	3.871573	3.653654	4.147523
17265211	Alox12e	arachidonate lipoxygenase, epidermal	5.680902	4.988133	5.617429	5.357203	4.985368	5.357203
17444137	Amz1	archaelysin family metallopeptidase 1	6.013896	6.013896	6.191504	5.862963	5.120306	7.202262
17257835	Amz2	archaelysin family metallopeptidase 2	29.88936	27.45392	26.93126	28.46959	28.12576	29.09441
17257848	Amz2	archaelysin family metallopeptidase 2	13.63195	13.07655	14.3521	19.39859	17.16526	15.71467
17526349	Arcn1	archain 1	250.9959	205.7103	185.4569	174.8083	214.3969	191.9278
17265030	Acap1	ArfGAP with coiled-coil, ankyrin repeat and PH domains 1	5.379045	7.888823	6.306283	5.808638	6.121945	6.730343
17329723	Acap2	ArfGAP with coiled-coil, ankyrin repeat and PH domains 2	52.21274	47.89058	55.46555	52.73431	44.29248	49.87448
17422785	Acap3	ArfGAP with coiled-coil, ankyrin repeat and PH domains 3	10.16607	12.13279	10.69586	12.74094	11.75791	9.901726
17454382	Adap1	ArfGAP with dual PH domains 1	6.316039	6.737204	7.4144	7.4144	8.460717	6.857867
17253885	Adap2	ArfGAP with dual PH domains 2	109.6369	115.0016	117.6894	99.45831	80.38364	81.01628

17266681	Adap2os	ArfGAP with dual PH domains 2, opposite strand	13.14097	13.35955	14.31723	14.22454	17.5019	13.9549
17214841	Agfg1	ArfGAP with FG repeats 1	69.91548	73.69475	74.18831	69.67987	65.07674	70.39739
17454121	Agfg2	ArfGAP with FG repeats 2	59.90523	59.46092	61.85893	59.21866	59.24453	59.42796
17215576	Agap1	ArfGAP with GTPase domain, ankyrin repeat and PH domain 1	27.97565	25.14849	34.01573	26.2059	27.79609	22.4666
17237915	Agap2	ArfGAP with GTPase domain, ankyrin repeat and PH domain 2	17.05059	20.54677	33.38921	24.25819	14.10026	15.70302
17435380	Agap3	ArfGAP with GTPase domain, ankyrin repeat and PH domain 3	34.87303	29.46704	35.19333	33.7611	31.98603	33.55835
17480829	Arap1	ArfGAP with RhoGAP domain, ankyrin repeat and PH domain 1	35.75542	34.11395	35.75542	38.82274	35.99763	38.63456
17448174	Arap2	ArfGAP with RhoGAP domain, ankyrin repeat and PH domain 2	43.04787	52.71677	54.80213	50.05388	51.32856	37.99822
17353881	Arap3	ArfGAP with RhoGAP domain, ankyrin repeat and PH domain 3	19.60881	16.35489	23.19256	20.80531	18.66224	17.38643
17274415	Asap2	ArfGAP with SH3 domain, ankyrin repeat and PH domain 2	25.58418	25.96111	23.05504	31.14006	27.56623	27.88196
17420036	Asap3	ArfGAP with SH3 domain, ankyrin repeat and PH domain 3	29.79999	27.10487	21.5535	29.10227	22.4305	22.61221
17317500	Asap1	ArfGAP with SH3 domain, ankyrin repeat and PH domain1	65.70585	67.52782	95.25811	61.45975	58.79738	67.42585
17239546	Arfgef3	ARFGEF family member 3	6.965756	7.990357	8.038186	6.504779	6.992896	8.093704
17276776	Arg2	arginase type II	4.512979	5.036355	4.580643	5.243645	6.790181	5.980019
17239845	Arg1	arginase, liver	811.6815	981.5242	1122.142	1107.553	1207.485	1227.587
17507333	Arglu1	arginine and glutamate rich 1	49.65889	54.06264	51.43684	62.13826	57.07947	56.34517
17421928	Rere	arginine glutamic acid dipeptide (RE) repeats	41.04743	40.96018	45.19214	45.36507	38.22382	41.80982
17391665	Avp	arginine vasopressin	12.36628	17.36644	17.77811	15.58148	15.27372	18.66832
17237762	Avpr1a	arginine vasopressin receptor 1A	39.241	19.56672	12.67163	31.65058	35.75889	22.29657
17217030	Avpr1b	arginine vasopressin receptor 1B	3.332373	2.550694	3.364752	3.633072	2.530005	2.712099
17535712	Avpr2	arginine vasopressin receptor 2	8.726058	10.14636	9.470956	11.22373	7.620153	10.1996
17364813	Avpi1	arginine vasopressin-induced 1	28.48321	30.23107	28.93533	32.63459	29.65842	30.23107
17419812	Rsrp1	arginine/serine rich protein 1	176.8583	228.9597	224.0846	213.4405	217.6626	281.4803
17398121	Rsrc1	arginine/serine-rich coiled-coil 1	15.15782	15.85537	17.05414	17.80314	15.7053	18.57693
17452689	Rsrc2	arginine/serine-rich coiled-coil 2	23.3563	27.04725	27.71798	27.96654	29.67014	28.01621
17453179	Asl	argininosuccinate lyase	438.1983	449.7408	552.561	647.2728	561.6276	565.4948
17233769	Gm5424	argininosuccinate synthase pseudogene	1009.265	1018.853	1013.029	1135.892	1003.761	1244.767
17369487	Ass1	argininosuccinate synthetase 1	21.47878	22.30044	21.40462	18.24223	17.6063	29.01908
17227278	Rnpep	arginyl aminopeptidase (aminopeptidase B)	44.97402	37.17652	39.45401	41.85651	33.73888	37.13841
17215839	Rnpepl1	arginyl aminopeptidase (aminopeptidase B)-like 1	90.41436	98.89461	103.2824	87.59278	85.06478	83.09329
17496973	Ate1	arginyltransferase 1	45.80236	37.24565	35.89572	40.43885	37.24565	35.26634
17261730	Rars	arginyl-tRNA synthetase	91.29665	76.43863	72.48045	77.59437	80.79309	69.09934
17412653	Rars2	arginyl-tRNA synthetase 2, mitochondrial	30.12355	26.15618	25.72983	30.33644	26.46713	30.45506
17430080	Ago1	argonaute RISC catalytic subunit 1	23.9345	21.20834	22.54976	19.74301	24.92584	19.42968
17317837	Ago2	argonaute RISC catalytic subunit 2	51.82046	69.20335	80.51692	70.49456	73.08381	60.82785
17430052	Ago3	argonaute RISC catalytic subunit 3	39.1028	37.42006	37.02685	28.98571	25.67887	34.7895
17430103	Ago4	argonaute RISC catalytic subunit 4	7.371049	7.370589	7.755501	5.747601	6.228738	6.587815
17531238	Arih2	ariadne homolog 2 (Drosophila)	103.6783	102.3388	103.9467	103.2609	112.8787	89.38619
17527787	Arih1	ariadne ubiquitin-conjugating enzyme E2 binding protein homolog 1 (Drosophila)	141.562	127.8936	123.2591	121.4737	111.9942	121.0064
17536254	Arx	aristaless related homeobox	18.93574	19.4507	15.18316	18.35575	19.92434	19.6578
17401578	Alx3	aristaless-like homeobox 3	12.02304	10.79364	12.62297	12.43591	13.42053	11.69768
17373585	Alx4	aristaless-like homeobox 4	12.107	10.09333	9.403449	15.93801	9.2931	10.29855
17404259	Armc1	armadillo repeat containing 1	94.96079	62.33671	58.53429	79.03556	79.79796	77.68693
17435116	Armc10	armadillo repeat containing 10	19.70009	20.39636	19.70009	17.68017	19.70009	24.68632
17335344	Armc12	armadillo repeat containing 12	3.491532	4.150773	3.256518	3.937282	3.85457	3.173841
17240502	Armc2	armadillo repeat containing 2	4.795064	5.166474	4.071979	4.659354	4.681043	4.486685
17367338	Armc3	armadillo repeat containing 3	3.354575	3.292515	3.339703	3.466308	3.292515	3.292515
17352527	Armc4	armadillo repeat containing 4	5.062325	4.4983	5.062325	6.221748	5.062325	5.062325
17483687	Armc5	armadillo repeat containing 5	21.63012	24.11836	23.46584	21.29481	17.62376	21.29481
17509985	Armc6	armadillo repeat containing 6	8.148531	8.662299	8.285392	9.259537	9.359296	10.18808
17258230	Armc7	armadillo repeat containing 7	15.29661	16.57067	20.06793	11.86031	7.806685	14.63327
17530069	Armc8	armadillo repeat containing 8	28.6045	36.25656	33.14249	35.5173	35.23214	33.43483
17215067	Armc9	armadillo repeat containing 9	11.6122	13.27601	13.83653	9.831694	12.64601	13.91491
17537742	Armcx1	armadillo repeat containing, X-linked 1	7.879095	8.171721	8.352106	6.507665	7.49506	7.918735
17544545	Armcx2	armadillo repeat containing, X-linked 2	4.759632	6.241142	5.579052	4.990416	5.036355	3.995926
17537756	Armcx3	armadillo repeat containing, X-linked 3	31.01426	28.65232	26.84702	26.84702	26.84702	26.40941
17537730	Armcx4	armadillo repeat containing, X-linked 4	8.134028	7.630037	8.442615	8.306841	7.746744	6.224595
17544535	Armcx6	armadillo repeat containing, X-linked 6	7.533081	5.888173	6.340383	5.207244	4.315806	5.949465
17323675	Arvcf	armadillo repeat gene deleted in velo-cardio-facial syndrome	14.56557	15.37559	12.0739	14.96915	15.08027	12.1243
17365384	Actr1a	ARP1 actin-related protein 1A, centractin alpha	108.168	94.48478	88.2161	105.9221	90.53477	86.66334
17222256	Actr1b	ARP1 actin-related protein 1B, centractin beta	67.29465	47.70042	56.0946	57.92483	44.68349	56.0946
17276040	Actr10	ARP10 actin-related protein 10	90.71945	68.23311	59.61072	77.48311	67.71014	68.99644
17260839	Actr2	ARP2 actin-related protein 2	310.2979	319.0193	286.754	266.9711	255.9414	294.1911
17226420	Actr3	ARP3 actin-related protein 3	237.557	195.4262	201.4224	208.2944	202.926	202.2982
17435505	Actr3b	ARP3 actin-related protein 3B	2.954348	3.132555	3.252232	3.211372	3.428007	3.476149
17378929	Actr5	ARP5 actin-related protein 5	16.3702	14.35479	13.94958	13.90036	14.1825	13.15136
17244126	Actr6	ARP6 actin-related protein 6	26.08629	28.4769	21.28817	33.69494	34.09315	36.22591
17298139	Actr8	ARP8 actin-related protein 8	66.77009	60.1991	58.43821	58.71023	57.65485	60.04158
17536548	Arr3	arrestin 3, retinal	3.432001	3.515452	3.786251	4.240605	3.223147	3.701038
17382335	Arrdc1	arrestin domain containing 1	6.093833	8.474217	7.226114	7.207006	6.659944	7.32973
17510200	Arrdc2	arrestin domain containing 2	17.69954	21.82394	20.84067	19.12543	25.91382	17.22868
17288876	Arrdc3	arrestin domain containing 3	131.6615	121.3853	354.0281	249.5889	281.7672	262.8599
17492051	Arrdc4	arrestin domain containing 4	45.09451	44.76161	54.31542	38.35333	53.59922	50.48315
17346226	Arrdc5	arrestin domain containing 5	4.89816	4.417561	4.044048	5.42262	4.86072	4.732951
17480568	Arrb1	arrestin, beta 1	31.92113	32.30174	43.09385	32.72474	26.63774	25.52095
17252013	Arrb2	arrestin, beta 2	22.4403	31.54261	38.31719	34.82521	30.55867	32.6165
17511277	Asna1	arsA arsenite transporter, ATP-binding, homolog 1 (bacterial)	60.87298	58.07317	66.03459	54.52402	49.50423	52.94601
17360084	As3mt	arsenic (+3 oxidation state) methyltransferase	92.21503	92.31736	106.7491	101.0426	100.7628	87.76112
17428996	Artn	artemin	6.621325	5.198336	5.120732	4.20927	4.768746	5.2036
17506800	Arv1	ARV1 homolog (yeast)	6.466432	11.79497	8.297059	10.19175	8.247404	9.482122
17400339	Arnt	aryl hydrocarbon receptor nuclear translocator	68.87746	83.69213	96.82448	84.18026	73.58093	70.82761
17492985	Arnt2	aryl hydrocarbon receptor nuclear translocator 2	6.078688	6.993332	7.639933	7.319576	8.978286	7.614293
17481960	Arntl	aryl hydrocarbon receptor nuclear translocator-like	62.80554	35.6856	27.37866	49.03636	29.81115	30.05369
17464331	Arntl2	aryl hydrocarbon receptor nuclear translocator-like 2	7.281169	8.828388	9.908935	9.907149	10.71426	8.594001

17265560	Aipl1	aryl hydrocarbon receptor-interacting protein-like 1	7.690461	7.437821	7.476743	7.942317	8.408859	7.89692
17397923	Aadac	arylacetamide deacetylase (esterase)	693.8548	447.7936	341.2961	481.4126	554.7216	659.2674
17397915	Aadacl2	arylacetamide deacetylase-like 2	2.39164	2.215542	2.73443	2.320957	3.160231	2.429602
17397907	Gm9696	arylacetamide deacetylase-like 2 pseudogene	2.148366	2.158816	1.933842	2.708267	2.309531	1.926684
17432565	Aadacl3	arylacetamide deacetylase-like 3	3.10734	3.263862	3.64753	3.366805	4.798355	3.07763
17258604	Aanat	arylalkylamine N-acetyltransferase	5.946291	5.120306	5.481573	5.05433	4.521213	5.334008
17258851	Afmid	arylformamidase	141.2718	167.699	166.2165	226.4063	186.9048	217.6792
17280729	Ahr	aryl-hydrocarbon receptor	89.26298	121.7704	110.2168	116.5407	128.1607	142.5209
17294352	Ahrr	aryl-hydrocarbon receptor repressor	4.955979	5.300432	5.300432	4.991384	5.300432	5.961643
17361099	Aip	aryl-hydrocarbon receptor-interacting protein	22.26491	24.23517	21.8465	23.52561	20.03124	21.8465
17320538	Arsa	arylsulfatase A	37.14948	53.42896	42.69899	46.75439	31.27331	31.15496
17289181	Arsb	arylsulfatase B	93.2932	79.08324	79.08324	72.63082	83.52985	84.48539
17257852	Arsg	arylsulfatase G	25.3278	24.09831	20.80618	24.61627	27.64564	23.42131
17350996	Arsi	arylsulfatase i	10.13841	8.76613	9.068664	10.97995	6.876145	8.683099
17402433	Arsj	arylsulfatase J	6.786793	4.381546	5.522571	6.943774	5.364486	5.776838
17294489	Arsk	arylsulfatase K	19.28664	16.87084	19.84715	15.5845	14.50482	18.35206
17399237	Ash1l	ash1 (absent, small, or homeotic)-like (Drosophila)	54.21312	79.09804	74.70744	64.34696	70.62563	85.16684
17508423	Ash2l	ash2 (absent, small, or homeotic)-like (Drosophila)	43.96123	41.39544	39.50217	40.72125	34.10497	40.72125
17251933	Asgr1	asialoglycoprotein receptor 1	300.6331	303.0559	266.862	304.2851	328.6162	304.2451
17251947	Asgr2	asialoglycoprotein receptor 2	240.8615	218.1702	206.3358	214.7419	268.6344	235.0282
17217914	Aspm	asp (abnormal spindle)-like, microcephaly associated (Drosophila)	5.023142	5.907223	4.900877	4.51949	4.917653	4.701594
17279277	Aspg	asparaginase homolog (S. cerevisiae)	85.99435	91.54632	84.66654	90.16527	84.51292	100.3301
17362544	Asrgl1	asparaginase like 1	21.96295	21.36711	19.82433	20.49955	17.10742	22.95203
17464718	Asns	asparagine synthetase	16.16868	13.34808	13.37846	16.29582	15.85197	13.82177
17222988	Asnsd1	asparagine synthetase domain containing 1	39.86249	42.75259	42.75259	42.75259	42.75259	47.94339
17322682	Alg1	asparagine-linked glycosylation 1 (beta-1,4-mannosyltransferase)	37.34362	40.05027	46.04109	46.13821	40.43576	41.58064
17314233	Alg10b	asparagine-linked glycosylation 10B (alpha-1,2-glucosyltransferase)	64.7079	54.52175	37.02788	46.6411	47.61519	45.77919
17499849	Alg11	asparagine-linked glycosylation 11 (alpha-1,2-mannosyltransferase)	65.01587	63.20689	55.14775	59.00642	52.84298	68.87746
17320189	Alg12	asparagine-linked glycosylation 12 (alpha-1,6-mannosyltransferase)	50.46461	29.82231	35.35568	28.70798	27.04725	24.57296
17538356	Alg13	asparagine-linked glycosylation 13	12.35189	12.26929	12.50489	12.50489	12.35189	11.63638
17402146	Alg14	asparagine-linked glycosylation 14	105.5052	84.98639	76.22752	81.59507	58.71145	66.94572
17425151	Alg2	asparagine-linked glycosylation 2 (alpha-1,3-mannosyltransferase)	43.97129	26.53823	32.59955	29.81239	27.747	26.86433
17329151	Alg3	asparagine-linked glycosylation 3 (alpha-1,3-mannosyltransferase)	54.56561	57.03952	63.19331	53.74021	45.57687	42.68922
17397632	Alg5	asparagine-linked glycosylation 5 (dolichyl-phosphate beta-glucosyltransferase)	70.57572	93.51397	89.86516	81.1208	92.70852	89.37132
17415778	Alg6	asparagine-linked glycosylation 6 (alpha-1,3,-glucosyltransferase)	11.87734	13.91376	10.46906	13.21404	10.6244	10.29855
17480327	Alg8	asparagine-linked glycosylation 8 (alpha-1,3,-glucosyltransferase)	45.29218	47.26204	52.66162	36.49255	48.56079	53.70334
17517151	Alg9	asparagine-linked glycosylation 9 (alpha 1,2 mannosyltransferase)	31.74396	28.85881	27.19708	29.77274	30.72136	27.71644
17355009	Nars	asparaginyl-tRNA synthetase	244.4592	201.0416	183.3403	182.6009	203.9356	162.8579
17480292	Nars2	asparaginyl-tRNA synthetase 2 (mitochondrial)(putative)	44.49373	28.95218	26.25372	31.80254	29.33416	26.39715
17496492	Asphd1	aspartate beta-hydroxylase domain containing 1	6.258296	6.954805	10.24423	5.971486	6.258296	7.824779
17451217	Asphd2	aspartate beta-hydroxylase domain containing 2	10.15811	5.863817	6.676722	6.769926	6.773399	6.773399
17477475	Aspdh	aspartate dehydrogenase domain containing	69.89222	81.88629	95.93331	93.07424	85.21113	91.66188
17423146	Asph	aspartate-beta-hydroxylase	34.58393	31.1118	29.35511	27.79339	32.8675	32.31092
17460465	Asprv1	aspartic peptidase, retroviral-like 1	4.070878	5.396448	5.717403	4.45937	3.492451	4.40762
17265748	Aspa	aspartoacylase	29.64465	22.00653	33.63497	25.71717	25.82936	30.14925
17356041	Acy3	aspartoacylase (aminoacylase) 3	165.8529	184.0278	180.2106	182.214	150.4041	184.2499
17224587	Dnpep	aspartyl aminopeptidase	64.42997	58.92059	63.27396	57.71442	63.27396	63.27396
17501148	Aga	aspartylglucosaminidase	53.03931	48.58739	54.30865	46.1782	51.99569	54.90973
17226572	Dars	aspartyl-tRNA synthetase	132.8892	119.9284	100.098	128.4123	112.0074	124.6501
17228804	Dars2	aspartyl-tRNA synthetase 2 (mitochondrial)	31.10576	30.10268	30.64819	29.21303	29.19704	29.99978
17287160	Aspn	asporin	3.313601	2.335838	2.577246	2.805912	3.130255	2.278462
17375866	Astl	astacin-like metalloendopeptidase (M12 family)	6.064253	5.765517	5.706713	5.294227	5.412631	4.756664
17521091	Aste1	asteroid homolog 1 (Drosophila)	16.79454	22.1444	18.16941	15.60136	18.60928	25.81576
17218514	Astn1	astrotactin 1	7.347788	7.701449	7.570193	7.654616	7.632258	8.826613
17426402	Astn2	astrotactin 2	14.29016	19.21206	17.30564	18.04053	16.23246	17.30564
17472847	Asun	asunder, spermatogenesis regulator	72.00374	63.61389	60.2964	62.1904	61.06484	66.3337
17230345	Ahctf1	AT hook containing transcription factor 1	42.23333	39.94713	36.20959	40.44642	37.29447	36.20959
17419500	Ahdc1	AT hook, DNA binding motif, containing 1	16.58135	20.1619	16.69947	21.56665	19.48632	23.14582
17431075	Arid1a	AT rich interactive domain 1A (SWI-like)	72.32939	78.59905	81.28572	64.65276	64.72181	76.22805
17332739	Arid1b	AT rich interactive domain 1B (SWI-like)	65.1196	69.66441	83.22702	77.57823	76.09449	69.97851
17314515	Arid2	AT rich interactive domain 2 (ARID, RFX-like)	63.43205	69.86849	70.1728	68.09562	65.91249	78.50486
17235037	Arid3a	AT rich interactive domain 3A (BRIGHT-like)	9.666476	11.09317	8.064472	9.709116	6.832152	8.561835
17527606	Arid3b	AT rich interactive domain 3B (BRIGHT-like)	10.61005	15.44417	12.17517	12.35189	12.168	12.35189
17424309	Arid3c	AT rich interactive domain 3C (BRIGHT-like)	10.34979	12.65451	11.99341	11.70319	11.50014	15.66008
17276070	Arid4a	AT rich interactive domain 4A (RBP1-like)	32.05994	37.90431	33.55603	37.06203	38.72169	35.10172
17285347	Arid4b	AT rich interactive domain 4B (RBP1-like)	31.81488	35.5683	30.18375	32.71545	30.18375	32.3636
17211795	Arid5a	AT rich interactive domain 5A (MRF1-like)	7.300777	7.337456	6.906573	9.157159	6.803702	7.337456
17241660	Arid5b	AT rich interactive domain 5B (MRF1-like)	172.4459	166.0703	175.6476	109.1728	133.7885	147.1884
17520439	Atr	ataxia telangiectasia and Rad3 related	18.07921	15.68159	13.58432	19.99143	20.68711	16.84467
17520490	Atr	ataxia telangiectasia and Rad3 related	4.8239	5.55351	4.753631	5.056819	6.899778	5.361252
17527054	Atm	ataxia telangiectasia mutated	24.67965	23.73214	16.63661	24.56121	21.26634	26.54761
17243229	Atcay	ataxia, cerebellar, Cayman type homolog (human)	6.287864	6.169699	5.594852	6.287864	6.331556	6.287864
17235597	Atcayos	ataxia, cerebellar, Cayman type homolog (human), opposite strand	7.698994	7.698994	7.698994	7.815727	6.721811	7.763461
17292174	Atxn1	ataxin 1	71.32075	80.4115	82.19963	92.20672	81.72412	76.54679
17313836	Atxn10	ataxin 10	227.1765	212.0043	223.0278	219.8781	232.4634	220.4335
17512846	Atxn1l	ataxin 1-like	38.13701	40.12335	31.02243	42.54418	42.965	44.66089
17441968	Atxn2	ataxin 2	158.3354	195.4452	216.1981	188.1644	138.2025	181.5647
17496253	Atxn2l	ataxin 2-like	80.72424	89.8253	99.23176	76.23175	87.60614	75.73299
17283422	Atxn3	ataxin 3	34.09835	42.66585	39.91115	44.4478	39.91115	41.56306
17297196	Atxn7	ataxin 7	47.0235	45.44753	48.04652	46.46266	47.07894	44.01429
17274911	Atxn7l1	ataxin 7-like 1	14.37978	15.52025	16.35239	15.66453	12.53856	15.56302
17274919	Atxn7l1	ataxin 7-like 1	13.13132	16.40291	17.47984	16.16263	14.68631	19.17441
17274940	Atxn7l1	ataxin 7-like 1	48.58032	33.64476	47.96101	59.95591	42.728	57.51475
17280677	Atxn7l1os1	ataxin 7-like 1, opposite strand 1 [Source:MGI Symbol;Acc:MGI:3779275]	2.513347	2.215097	2.481049	2.513347	2.513347	2.379799

17280682	Atxn7l1os2	ataxin 7-like 1, opposite strand 2	6.949215	8.939722	11.73609	9.298577	9.190861	9.606161
17409243	Atxn7l2	ataxin 7-like 2	5.843489	12.41481	8.636938	10.56141	9.046314	10.27616
17270118	Atxn7l3	ataxin 7-like 3	21.19585	19.98077	21.16033	20.77606	24.37178	24.05942
17244967	Atxn7l3b	ataxin 7-like 3B	150.101	167.4945	156.5869	146.1423	175.9998	169.5997
17484682	Athl1	ATH1, acid trehalase-like 1 (yeast)	35.1246	38.70532	35.48383	35.3209	34.32481	30.87913
17426277	Akna	AT-hook transcription factor	11.781	12.26079	13.90055	12.30958	12.26079	15.11868
17414756	Aknaos	AT-hook transcription factor, opposite strand	3.779722	4.129222	4.124445	4.117104	3.791976	4.147408
17275955	Atl1	atlastin GTPase 1	7.47892	8.074819	7.22366	7.148695	7.108918	7.424326
17347457	Atl2	atlastin GTPase 2	176.2256	232.0095	222.688	216.0648	190.3895	215.8672
17357054	Atl3	atlastin GTPase 3	187.6707	173.3495	156.3191	171.1384	176.1817	183.184
17505927	Atmin	ATM interactor	24.21753	21.82788	23.99547	23.96405	27.67862	23.90284
17459153	Atoh1	atonal homolog 1 (Drosophila)	7.086926	7.399511	7.870689	7.548343	5.079125	7.126185
17233886	Atoh7	atonal homolog 7 (Drosophila)	16.03564	10.96535	12.50601	17.92362	13.67311	14.07663
17467706	Atoh8	atonal homolog 8 (Drosophila)	33.08373	37.01274	40.02862	41.26281	50.17894	51.37947
17269521	Acly	ATP citrate lyase	197.7755	144.2323	169.292	152.4064	189.0259	162.1314
17221873	ATP8	ATP synthase F0 subunit 8	5482.127	5497.538	5311.082	5662.761	5373.29	5688.726
17532617	ATP8	ATP synthase F0 subunit 8	4619.497	4471.552	4461.057	4612.298	4551.385	4820.376
17417290	Atpaf1	ATP synthase mitochondrial F1 complex assembly factor 1	37.32111	39.62777	41.01927	42.45022	40.89918	42.63156
17263594	Atpaf2	ATP synthase mitochondrial F1 complex assembly factor 2	39.92526	46.41824	42.24357	48.43809	40.29391	43.38908
17238278	Atp5b	ATP synthase, H+ transporting mitochondrial F1 complex, beta subunit	1630.456	1591.201	1540.422	1603.535	1311.744	1521.839
17408863	Atp5f1	ATP synthase, H+ transporting, mitochondrial F0 complex, subunit B1	379.8353	372.8212	347.6472	354.679	334.62	400.2229
17268289	Atp5g1	ATP synthase, H+ transporting, mitochondrial F0 complex, subunit C1 (subunit 9)	17.58167	16.94644	15.76147	12.92838	17.15801	20.10915
17322280	Atp5g2	ATP synthase, H+ transporting, mitochondrial F0 complex, subunit C2 (subunit 9)	178.0847	231.5741	295.3578	215.5278	221.9837	234.9467
17340500	Atp5g2	ATP synthase, H+ transporting, mitochondrial F0 complex, subunit C2 (subunit 9)	240.5112	314.3352	396.0668	299.6927	316.2233	313.137
17386621	Atp5g3	ATP synthase, H+ transporting, mitochondrial F0 complex, subunit C3 (subunit 9)	184.4684	206.4302	204.7841	199.7761	205.2132	227.446
17271891	Atp5h	ATP synthase, H+ transporting, mitochondrial F0 complex, subunit D	9.511349	9.987198	11.00151	9.600836	12.01271	9.447417
17315713	Atp5h	ATP synthase, H+ transporting, mitochondrial F0 complex, subunit D	494.2475	615.6557	618.4229	578.4707	541.5159	610.5138
17331656	Atp5j	ATP synthase, H+ transporting, mitochondrial F0 complex, subunit F	23.29454	22.49231	20.29525	24.05542	23.50686	23.29454
17455083	Atp5j2	ATP synthase, H+ transporting, mitochondrial F0 complex, subunit F2	7.870689	5.817866	8.404896	4.840983	4.815281	7.189293
17526421	Atp5l	ATP synthase, H+ transporting, mitochondrial F0 complex, subunit G	281.065	221.6501	217.7396	220.871	195.3138	202.1286
17478797	Atp5l-ps1	ATP synthase, H+ transporting, mitochondrial F0 complex, subunit G, pseudogene	200.228	308.9608	296.5435	333.9226	347.4087	355.8265
17275929	Atp5s	ATP synthase, H+ transporting, mitochondrial F0 complex, subunit S	11.06215	13.74057	14.92263	12.82307	14.34892	14.85195
17352020	Atp5a1	ATP synthase, H+ transporting, mitochondrial F1 complex, alpha subunit 1	1167.478	1162.415	1178.054	1247.073	1182.234	1363.18
17235198	Atp5d	ATP synthase, H+ transporting, mitochondrial F1 complex, delta subunit	96.03311	100.0072	107.8214	97.24485	87.70517	87.22078
17395165	Atp5e	ATP synthase, H+ transporting, mitochondrial F1 complex, epsilon subunit	112.239	83.02362	107.9822	125.4884	142.8018	138.145
17381573	Atp5c1	ATP synthase, H+ transporting, mitochondrial F1 complex, gamma polypeptide 1	21.83408	24.8217	21.30042	24.29807	21.1615	29.07284
17294544	Atp5c1-ps	ATP synthase, H+ transporting, mitochondrial F1 complex, gamma polypeptide 1,	3.145414	2.954246	2.501355	2.54868	2.909054	2.472996
17332203	Atp5o	ATP synthase, H+ transporting, mitochondrial F1 complex, O subunit	29.17458	21.0178	25.2498	22.63338	21.11183	25.79698
17450741	Atp5k	ATP synthase, H+ transporting, mitochondrial F1F0 complex, subunit E	131.4645	153.2571	152.5015	160.6904	175.6476	190.8652
17293190	Agtpbp1	ATP/GTP binding protein 1	18.98463	17.55451	17.9856	17.82154	15.34259	14.44111
17479259	Agbl1	ATP/GTP binding protein-like 1	4.291345	2.569006	3.146592	3.56994	2.58953	3.162663
17373057	Agbl2	ATP/GTP binding protein-like 2	3.092964	3.991359	3.092964	3.092964	2.794364	3.034755
17457207	Agbl3	ATP/GTP binding protein-like 3	4.204458	4.123244	4.113909	4.963336	4.059888	4.369566
17416926	Agbl4	ATP/GTP binding protein-like 4	4.788985	4.38185	5.114524	4.661809	4.755642	4.661809
17435888	Agbl5	ATP/GTP binding protein-like 5	16.27843	21.21511	20.32213	18.62683	14.08405	19.84866
17475311	Atp5sl	ATP5S-like	19.14041	33.49445	30.99084	32.07728	31.32386	34.36124
17363983	Atad1	ATPase family, AAA domain containing 1	155.694	136.0686	131.1569	130.0786	116.3559	131.7373
17317233	Atad2	ATPase family, AAA domain containing 2	15.64034	11.83887	17.39596	10.72332	11.27707	11.40323
17273819	Atad2b	ATPase family, AAA domain containing 2B	57.42233	60.68256	46.59425	55.83199	52.81662	63.5174
17433860	Atad3a	ATPase family, AAA domain containing 3A	56.0946	63.89052	75.82228	78.68627	81.2553	77.01513
17422678	Atad3aos	ATPase family, AAA domain containing 3A, opposite strand	8.561657	8.166625	8.482209	8.846152	8.790773	8.561657
17253858	Atad5	ATPase family, AAA domain containing 5	4.001608	3.782369	3.7798	2.890722	3.288296	3.726393
17430878	Atpif1	ATPase inhibitory factor 1	72.69731	107.5907	141.2562	104.8483	83.16877	97.35006
17501692	Atp13a1	ATPase type 13A1	52.51108	46.72167	47.68257	44.51193	39.57726	45.07608
17420911	Atp13a2	ATPase type 13A2	24.70498	24.34426	28.10296	24.96647	27.07332	24.87061
17329640	Atp13a3	ATPase type 13A3	325.4225	252.6226	254.1647	245.6891	265.9369	258.1096
17329568	Atp13a4	ATPase type 13A4	4.240869	3.437501	3.358344	3.30303	3.308621	3.49066
17329533	Atp13a5	ATPase type 13A5	3.895693	3.841314	3.198133	3.501444	3.734927	3.937391
17534337	Atp1b4	ATPase, (Na+)/K+ transporting, beta 4 polypeptide	3.496133	3.8716	4.072571	4.072571	4.072571	3.896503
17448475	Atp8a1	ATPase, aminophospholipid transporter (APLT), class I, type 8A, member 1	18.76536	20.08519	20.46505	16.01787	11.04889	14.04048
17307354	Atp8a2	ATPase, aminophospholipid transporter-like, class I, type 8A, member 2	3.969315	5.535409	4.378905	5.574723	4.886969	5.396485
17496211	Atp2a1	ATPase, Ca++ transporting, cardiac muscle, fast twitch 1	7.326123	8.425078	7.193929	6.63143	4.433789	6.059505
17452405	Atp2a2	ATPase, Ca++ transporting, cardiac muscle, slow twitch 2	631.3429	415.3662	431.2229	440.8029	418.4496	347.852
17236836	Atp2b1	ATPase, Ca++ transporting, plasma membrane 1	102.3871	91.11268	92.40698	86.2104	94.53325	98.25167
17469814	Atp2b2	ATPase, Ca++ transporting, plasma membrane 2	8.974428	9.051018	10.36853	10.54078	8.877602	7.015763
17535572	Atp2b3	ATPase, Ca++ transporting, plasma membrane 3	4.121073	3.837136	4.056625	3.440456	3.760957	3.134141
17227037	Atp2b4	ATPase, Ca++ transporting, plasma membrane 4	11.55416	9.655427	12.54178	10.52188	9.22034	9.448269
17506147	Atp2c2	ATPase, Ca++ transporting, type 2C, member 2	5.465487	4.801649	5.48933	5.48933	5.857317	5.48933
17252497	Atp2a3	ATPase, Ca++ transporting, ubiquitous	6.894997	6.957071	9.070298	5.859307	7.887292	7.46354
17530457	Atp2c1	ATPase, Ca++-sequestering	70.27891	63.02796	56.11327	55.49131	63.02796	63.02796
17355026	Atp8b1	ATPase, class I, type 8B, member 1	165.9391	113.8066	107.473	109.1698	147.6349	94.4815
17407149	Atp8b2	ATPase, class I, type 8B, member 2	7.240353	7.674219	9.059868	7.07044	4.999625	7.751578
17242959	Atp8b3	ATPase, class I, type 8B, member 3	7.870689	11.42593	7.735208	8.64143	10.75719	8.590368
17391056	Atp8b4	ATPase, class I, type 8B, member 4	4.130081	3.08945	3.605952	3.224018	3.315554	3.144891
17413269	Atp8b5	ATPase, class I, type 8B, member 5	6.96508	7.086582	7.275569	6.198933	7.219305	7.638345
17394829	Atp9a	ATPase, class II, type 9A	73.25473	85.99256	83.15033	65.89011	69.12521	53.38639
17355687	Atp9b	ATPase, class II, type 9B	43.99172	47.89788	39.94159	38.96643	31.50568	37.23403
17478745	Atp10a	ATPase, class V, type 10A	4.524756	4.933461	5.168838	4.036962	5.584972	4.378449
17248561	Atp10b	ATPase, class V, type 10B	3.402511	3.686087	5.160497	3.496036	2.948089	3.096654
17438035	Atp10d	ATPase, class V, type 10D	12.45592	14.60692	12.91826	17.4445	15.78924	12.71033
17499117	Atp11a	ATPase, class VI, type 11A	56.80083	48.45219	48.92059	39.55039	56.20552	33.99451
17396892	Atp11b	ATPase, class VI, type 11B	59.40984	57.15746	43.72693	54.21312	56.32916	60.22205
17541986	Atp11c	ATPase, class VI, type 11C	498.749	474.0581	338.1689	482.164	471.77	458.8536
17537055	Atp7a	ATPase, Cu++ transporting, alpha polypeptide	33.30669	26.46438	22.16129	22.11694	26.34505	24.19438

17507955	Atp7b	ATPase, Cu++ transporting, beta polypeptide	44.72687	40.2981	44.50113	43.29324	37.38843	42.44257
17535792	Atp6ap1	ATPase, H+ transporting, lysosomal accessory protein 1	118.9491	119.7258	122.2762	94.42258	107.4879	124.0657
17294775	Atp6ap1l	ATPase, H+ transporting, lysosomal accessory protein 1-like	3.225896	4.223854	3.729055	3.280828	4.536187	3.729055
17533336	Atp6ap2	ATPase, H+ transporting, lysosomal accessory protein 2	65.80658	72.61874	68.07202	48.548	64.95506	52.62768
17256473	Atp6v0a1	ATPase, H+ transporting, lysosomal V0 subunit A1	66.11061	73.69424	90.96439	73.69424	58.6354	66.74741
17442588	Atp6v0a2	ATPase, H+ transporting, lysosomal V0 subunit A2	80.5465	56.56195	68.67104	69.24222	50.7709	55.81187
17465942	Atp6v0a4	ATPase, H+ transporting, lysosomal V0 subunit A4	2.858365	5.162644	3.092299	3.703322	3.34385	4.396361
17428946	Atp6v0b	ATPase, H+ transporting, lysosomal V0 subunit B	105.8641	110.9011	111.6895	99.93028	109.5731	96.18167
17341731	Atp6v0c	ATPase, H+ transporting, lysosomal V0 subunit C	12.90126	12.61161	12.88098	13.89689	11.31151	12.38868
17457343	Atp6v0c-ps2	ATPase, H+ transporting, lysosomal V0 subunit C, pseudogene 2	94.95355	89.16094	120.2547	89.23328	87.49751	102.2942
17512456	Atp6v0d1	ATPase, H+ transporting, lysosomal V0 subunit D1	174.0502	149.3403	129.2777	144.1773	135.2777	117.1473
17423577	Atp6v0d2	ATPase, H+ transporting, lysosomal V0 subunit D2	4.681043	5.303887	5.532839	4.24081	20.09507	12.64872
17334948	Atp6v0e	ATPase, H+ transporting, lysosomal V0 subunit E	108.6919	109.7632	119.1438	99.65084	103.5454	99.196
17458308	Atp6v0e2	ATPase, H+ transporting, lysosomal V0 subunit E2	10.66876	8.88579	10.66876	10.73135	10.66876	10.66876
17330523	Atp6v1a	ATPase, H+ transporting, lysosomal V1 subunit A	96.03111	99.56591	95.06682	90.85979	80.99439	83.29454
17460107	Atp6v1b1	ATPase, H+ transporting, lysosomal V1 subunit B1	8.731624	8.461421	9.400451	9.314253	9.206291	8.554894
17501652	Atp6v1b2	ATPase, H+ transporting, lysosomal V1 subunit B2	114.1004	133.8961	123.3557	122.1331	125.3293	128.3474
17311157	Atp6v1c1	ATPase, H+ transporting, lysosomal V1 subunit C1	88.52604	90.47203	88.69618	90.28222	76.05283	81.91808
17280148	Atp6v1c2	ATPase, H+ transporting, lysosomal V1 subunit C2	3.621808	5.007082	3.852567	4.550768	3.569421	5.755495
17282104	Atp6v1d	ATPase, H+ transporting, lysosomal V1 subunit D	66.18489	62.34103	57.10836	62.88221	59.40408	64.84035
17470445	Atp6v1e1	ATPase, H+ transporting, lysosomal V1 subunit E1	121.1684	157.0816	155.6477	151.0728	190.6681	153.4697
17347883	Atp6v1e2	ATPase, H+ transporting, lysosomal V1 subunit E2	7.712187	9.317804	8.921647	9.617287	9.481465	8.171551
17456678	Atp6v1f	ATPase, H+ transporting, lysosomal V1 subunit F	6.073171	6.566752	6.176588	6.477019	4.483887	5.034889
17414760	Atp6v1g1	ATPase, H+ transporting, lysosomal V1 subunit G1	59.92392	55.98545	57.16063	57.68283	57.21137	58.55011
17337036	Atp6v1g2	ATPase, H+ transporting, lysosomal V1 subunit G2	29.12084	27.7372	24.99903	29.70863	27.69435	30.54639
17217848	Atp6v1g3	ATPase, H+ transporting, lysosomal V1 subunit G3	3.219865	3.941214	4.005993	5.085537	4.033046	5.554011
17210887	Atp6v1h	ATPase, H+ transporting, lysosomal V1 subunit H	56.1475	44.99554	39.61267	49.26325	50.82372	54.62653
17507595	Atp4b	ATPase, H+/K+ exchanging, beta polypeptide	6.885398	6.711336	6.415893	6.652792	7.100743	7.095725
17476497	Atp4a	ATPase, H+/K+ exchanging, gastric, alpha polypeptide	7.979952	8.784012	8.530262	9.209546	7.557086	7.700382
17300735	Atp12a	ATPase, H+/K+ transporting, nongastric, alpha polypeptide	5.40472	4.709977	4.800152	4.412389	4.709977	4.886969
17408509	Atp1a1	ATPase, Na+/K+ transporting, alpha 1 polypeptide	249.7308	336.7094	379.9959	302.3341	346.4348	321.5435
17229891	Atp1a2	ATPase, Na+/K+ transporting, alpha 2 polypeptide	8.674496	7.698994	9.070738	8.658577	9.866032	11.28778
17487805	Atp1a3	ATPase, Na+/K+ transporting, alpha 3 polypeptide	6.267804	6.328089	6.328089	6.211234	11.94041	7.215053
17229865	Atp1a4	ATPase, Na+/K+ transporting, alpha 4 polypeptide	7.712508	5.440691	6.895905	7.699581	7.25467	9.342444
17229178	Atp1b1	ATPase, Na+/K+ transporting, beta 1 polypeptide	151.3191	169.541	132.6527	148.5557	189.8347	207.7378
17264792	Atp1b2	ATPase, Na+/K+ transporting, beta 2 polypeptide	4.789682	6.489152	4.502418	4.789682	4.688642	4.535872
17529793	Atp1b3	ATPase, Na+/K+ transporting, beta 3 polypeptide	63.96097	66.75065	73.66258	57.11073	66.50957	68.61585
17425301	Abca1	ATP-binding cassette, sub-family A (ABC1), member 1	219.7608	225.9469	221.9206	220.0946	240.4678	209.9119
17224015	Abca12	ATP-binding cassette, sub-family A (ABC1), member 12	4.47203	4.47203	4.47203	4.47203	3.945478	4.551493
17247241	Abca13	ATP-binding cassette, sub-family A (ABC1), member 13	3.461554	3.233283	3.190141	3.559168	3.855559	3.542014
17482381	Abca14	ATP-binding cassette, sub-family A (ABC1), member 14	3.517206	3.587826	3.070854	3.00822	3.28604	3.362397
17482416	Abca15	ATP-binding cassette, sub-family A (ABC1), member 15	3.626908	3.66395	3.729055	4.549443	3.729055	3.723321
17482450	Abca16	ATP-binding cassette, sub-family A (ABC1), member 16	2.890121	2.242332	2.26459	2.390595	2.507709	2.592547
17341803	Abca17	ATP-binding cassette, sub-family A (ABC1), member 17	7.622055	6.603038	8.50293	8.995977	6.879482	7.305637
17368009	Abca2	ATP-binding cassette, sub-family A (ABC1), member 2	52.34755	46.62785	42.02075	50.91998	42.09655	46.02673
17334205	Abca3	ATP-binding cassette, sub-family A (ABC1), member 3	89.76182	85.50459	115.1252	96.92453	87.33512	101.2635
17402222	Abca4	ATP-binding cassette, sub-family A (ABC1), member 4	5.676808	5.269149	4.82283	6.554156	4.759203	4.97284
17271486	Abca5	ATP-binding cassette, sub-family A (ABC1), member 5	17.206	19.49537	20.20373	15.56086	18.93771	20.42126
17271443	Abca6	ATP-binding cassette, sub-family A (ABC1), member 6	334.9426	349.7887	337.0736	345.0544	385.5382	401.7098
17235087	Abca7	ATP-binding cassette, sub-family A (ABC1), member 7	9.049575	9.666744	10.00494	9.985191	10.61189	8.836592
17271350	Abca8a	ATP-binding cassette, sub-family A (ABC1), member 8a	112.5615	62.64728	56.69226	81.73601	79.35889	79.0942
17271307	Abca8b	ATP-binding cassette, sub-family A (ABC1), member 8b	83.19068	80.28564	78.43741	71.8457	88.55489	74.01316
17271399	Abca9	ATP-binding cassette, sub-family A (ABC1), member 9	11.84002	12.55021	11.68957	11.65123	10.05227	10.75272
17514033	Abcb10	ATP-binding cassette, sub-family B (MDR/TAP), member 10	56.26828	41.13002	41.07048	48.07883	42.49585	40.14811
17386177	Abcb11	ATP-binding cassette, sub-family B (MDR/TAP), member 11	724.5643	542.3574	463.2573	588.8719	535.1391	478.8035
17434524	Abcb1a	ATP-binding cassette, sub-family B (MDR/TAP), member 1A	11.24904	25.0344	17.0252	14.35727	19.93056	18.24374
17434555	Abcb1b	ATP-binding cassette, sub-family B (MDR/TAP), member 1B	2.861755	4.792904	3.990225	5.167083	5.57998	4.746125
17434585	Abcb4	ATP-binding cassette, sub-family B (MDR/TAP), member 4	746.2887	792.54	706.5315	744.6146	683.5201	702.4443
17284808	Abcb5	ATP-binding cassette, sub-family B (MDR/TAP), member 5	3.416384	3.54506	3.267099	3.662402	3.68831	3.582061
17224476	Abcb6	ATP-binding cassette, sub-family B (MDR/TAP), member 6	100.6519	104.3951	100.9173	99.45693	80.27785	98.42958
17543852	Abcb7	ATP-binding cassette, sub-family B (MDR/TAP), member 7	103.1343	95.47493	94.047	90.09468	86.73245	92.07196
17435311	Abcb8	ATP-binding cassette, sub-family B (MDR/TAP), member 8	28.70161	26.89265	25.82757	30.99965	29.11761	29.20533
17452719	Abcb9	ATP-binding cassette, sub-family B (MDR/TAP), member 9	11.87627	13.20388	11.69946	11.50086	11.76435	9.951196
17323138	Abcc1	ATP-binding cassette, sub-family C (CFTR/MRP), member 1	10.81949	17.43072	15.77633	19.3138	11.95896	16.8761
17345362	Abcc10	ATP-binding cassette, sub-family C (CFTR/MRP), member 10	23.96123	23.96123	28.29451	23.96123	22.43563	18.56702
17511410	Abcc12	ATP-binding cassette, sub-family C (CFTR/MRP), member 12	4.236785	4.746848	5.15592	5.151919	3.819887	4.954021
17359636	Abcc2	ATP-binding cassette, sub-family C (CFTR/MRP), member 2	727.7908	828.0649	713.7178	763.1697	789.1523	894.2287
17267846	Abcc3	ATP-binding cassette, sub-family C (CFTR/MRP), member 3	336.8844	392.552	405.0763	343.1344	267.3322	359.351
17309420	Abcc4	ATP-binding cassette, sub-family C (CFTR/MRP), member 4	15.4233	20.06418	22.64421	18.15582	11.82215	15.43764
17329105	Abcc5	ATP-binding cassette, sub-family C (CFTR/MRP), member 5	9.027334	10.25418	9.965485	9.34089	8.149604	8.226792
17491035	Abcc6	ATP-binding cassette, sub-family C (CFTR/MRP), member 6	71.05774	74.67016	78.88997	78.85225	82.04139	85.55024
17491074	Abcc8	ATP-binding cassette, sub-family C (CFTR/MRP), member 8	7.663854	6.904323	7.274106	7.698994	7.817785	8.79638
17472536	Abcc9	ATP-binding cassette, sub-family C (CFTR/MRP), member 9	92.71045	113.4058	123.543	87.65655	95.74266	81.13036
17535627	Abcd1	ATP-binding cassette, sub-family D (ALD), member 1	45.83189	44.78364	57.43905	45.84556	35.8798	45.99834
17320652	Abcd2	ATP-binding cassette, sub-family D (ALD), member 2	46.43305	84.50589	86.58769	48.21867	114.3474	77.08296
17409920	Abcd3	ATP-binding cassette, sub-family D (ALD), member 3	1195.899	1145.118	1116.347	1230.748	985.97	1140.887
17282534	Abcd4	ATP-binding cassette, sub-family D (ALD), member 4	19.73042	19.59414	24.78079	26.28194	27.31024	27.57846
17510749	Abce1	ATP-binding cassette, sub-family E (OABP), member 1	282.0916	210.9182	208.864	216.9848	209.6937	190.5321
17344511	Abcf1	ATP-binding cassette, sub-family F (GCN20), member 1	80.97474	82.9891	82.13755	96.00782	82.88448	83.54722
17446253	Abcf2	ATP-binding cassette, sub-family F (GCN20), member 2	88.82907	81.62957	72.31886	74.12971	82.93332	88.28155
17323945	Abcf3	ATP-binding cassette, sub-family F (GCN20), member 3	66.44598	53.0724	46.01716	52.29822	50.97048	46.87575
17335770	Abcg1	ATP-binding cassette, sub-family G (WHITE), member 1	29.0042	28.94034	32.13292	26.76953	29.99687	31.28458
17459014	Abcg2	ATP-binding cassette, sub-family G (WHITE), member 2	184.8947	120.7358	77.23913	113.734	85.57396	102.9293
17450414	Abcg3	ATP-binding cassette, sub-family G (WHITE), member 3	13.25018	12.71712	13.75496	12.22311	10.16128	7.880078

17526175	Abcg4	ATP-binding cassette, sub-family G (WHITE), member 4	4.647678	4.220927	7.076765	4.339594	5.273863	4.816383
17347729	Abcg5	ATP-binding cassette, sub-family G (WHITE), member 5	113.2165	133.8803	80.07778	146.8175	134.333	159.7852
17340099	Abcg8	ATP-binding cassette, sub-family G (WHITE), member 8	126.0568	153.6922	105.8135	147.9873	156.8683	188.8203
17531265	Atrip	ATR interacting protein	12.35763	13.58695	11.06959	14.10779	10.23465	12.63059
17470831	Atn1	atrophin 1	28.83102	42.16108	49.10064	40.31878	42.51323	48.88026
17376391	Atrn	attractin	111.3409	102.9578	107.041	113.7261	110.3957	84.72759
17360695	Atrnl1	attractin like 1	65.38963	56.32057	56.20163	59.42879	54.09187	54.16128
17263004	Atox1	ATX1 (antioxidant protein 1) homolog 1 (yeast)	122.8701	178.7179	200.5016	169.0586	131.4072	148.2235
17229984	Ackr1	atypical chemokine receptor 1 (Duffy blood group)	6.812102	6.812102	8.237691	6.41785	7.33161	6.356136
17523371	Ackr2	atypical chemokine receptor 2	33.65759	31.68812	42.70432	34.71988	25.1867	32.3894
17215605	Ackr3	atypical chemokine receptor 3	11.05763	10.06922	11.48151	8.422742	7.519517	10.86081
17530339	Ackr4	atypical chemokine receptor 4	5.134344	5.008609	6.667519	4.970979	5.230359	4.367507
17292612	Auh	AU RNA binding protein/enoyl-coenzyme A hydratase	22.2958	25.64703	26.99256	25.69329	21.42911	22.99471
17395016	Aurka	aurora kinase A	7.897193	7.847592	7.698994	8.092694	6.103937	7.698994
17419792	Aunip	aurora kinase A and ninein interacting protein	5.68969	4.839876	5.167513	4.72181	4.637123	7.05365
17422722	Aurkaip1	aurora kinase A interacting protein 1	29.90283	31.46116	30.24301	32.61446	27.3413	33.53533
17251485	Aurkb	aurora kinase B	9.914362	8.953737	8.765887	7.698994	6.866286	9.41917
17473650	Aurkc	aurora kinase C	14.77606	9.07099	15.77251	10.91553	9.755461	14.88606
17453242	Auts2	autism susceptibility candidate 2	5.022376	5.644986	5.543396	5.543396	5.169053	6.940406
17511808	Amfr	autocrine motility factor receptor	415.7522	377.3004	386.2443	419.0882	444.7282	367.4566
17242401	Aire	autoimmune regulator (autoimmune polyendocrinopathy candidiasis ectodermal c	16.48555	18.0588	18.45922	17.0945	16.5241	16.62785
17294785	Atg10	autophagy related 10	21.83363	24.27619	16.8994	14.58325	22.35336	19.05067
17315187	Atg101	autophagy related 101	46.11487	46.60846	45.02767	41.3768	72.47844	60.82785
17354290	Atg12	autophagy related 12	39.25923	34.93763	38.95509	38.64018	32.65699	32.43726
17388261	Atg13	autophagy related 13	127.459	83.93671	82.05504	93.04392	83.93671	84.2947
17305813	Atg14	autophagy related 14	38.03907	37.50055	47.53967	47.53967	51.57071	46.87023
17215345	Atg16l1	autophagy related 16-like 1 (S. cerevisiae)	45.22003	39.9355	44.61138	49.45108	52.54458	33.44225
17493880	Atg16l2	autophagy related 16-like 2 (S. cerevisiae)	17.01612	20.08811	18.49649	18.49649	15.03195	12.80912
17356695	Atg2a	autophagy related 2A	50.37758	47.79309	54.2808	57.43348	49.98142	47.44782
17283784	Atg2b	autophagy related 2B	53.22455	51.08012	46.98082	48.54228	42.41228	44.02466
17325919	Atg3	autophagy related 3	193.9163	217.0058	188.645	196.536	201.5536	226.3984
17538206	Atg4a	autophagy related 4A, cysteine peptidase	43.09355	46.90631	43.4574	45.50395	33.49979	38.33446
17216070	Atg4b	autophagy related 4B, cysteine peptidase	28.61064	28.02476	28.61064	24.20964	27.69435	30.17057
17415747	Atg4c	autophagy related 4C, cysteine peptidase	42.07204	40.58374	33.74917	40.47502	48.88195	39.37178
17415768	Atg4c	autophagy related 4C, cysteine peptidase	9.049449	8.426655	9.488237	7.357165	9.810115	11.40315
17515132	Atg4d	autophagy related 4D, cysteine peptidase	21.99357	24.95644	24.27131	27.32899	25.72091	25.5707
17233061	Atg5	autophagy related 5	107.4126	106.3436	91.56092	121.0408	115.7951	111.3973
17461897	Atg7	autophagy related 7	69.82975	67.61306	67.61306	66.62307	63.60772	68.99309
17224500	Atg9a	autophagy related 9A	54.34216	57.34358	61.17711	51.40583	44.87064	63.70788
17446153	Atg9b	autophagy related 9B	4.01461	4.347211	4.133059	3.781242	3.997699	4.667662
17373417	Ambra1	autophagy/beclin 1 regulator 1	47.20704	50.11081	49.52963	40.63694	42.24738	49.52963
17332358	Erg	avian erythroblastosis virus E-26 (v-ets) oncogene related	17.23776	15.19958	20.22027	15.3121	14.1553	15.87242
17513289	Maf	avian musculoaponeurotic fibrosarcoma (v-maf) AS42 oncogene homolog	41.42357	61.75654	63.48219	53.02791	38.48248	48.6946
17487325	Relb	avian reticuloendotheliosis viral (v-rel) oncogene related B	17.69684	17.69684	19.47188	20.07962	17.69684	18.64788
17458892	Avl9	AVL9 homolog (S. cerevisiase)	54.25447	70.29548	58.65247	68.40358	57.44383	67.28766
17334869	Axin1	axin 1	34.9895	36.29905	32.05928	28.20287	31.15734	31.07107
17220439	Aida	axin interactor, dorsalization associated	23.13363	24.76447	31.926	34.12341	26.42552	24.36114
17257801	Axin2	axin2	19.99185	26.26592	22.10407	23.84029	16.80537	21.93587
17488032	Axl	AXL receptor tyrosine kinase	68.95484	79.53511	100.3836	68.95484	55.49093	68.95484
17228515	Axdnd1	axonemal dynein light chain domain containing 1	3.462322	3.098865	3.747062	2.376239	3.638919	3.261329
17325938	Btla	B and T lymphocyte associated	4.27312	4.613143	4.237167	3.520938	4.621304	4.440894
17247849	Bcl11a	B cell CLL/lymphoma 11A (zinc finger protein)	12.22337	12.34881	13.94175	12.23091	12.29577	11.17072
17247852	Bcl11a	B cell CLL/lymphoma 11A (zinc finger protein)	5.338965	4.495682	5.338965	6.831631	4.371838	5.338965
17265164	Bcl6b	B cell CLL/lymphoma 6, member B	9.67177	10.20356	9.064893	9.544635	12.94847	11.09486
17442269	Bcl7a	B cell CLL/lymphoma 7A	13.55403	12.3958	13.78436	13.91231	12.22735	12.87044
17443221	Bcl7b	B cell CLL/lymphoma 7B	40.07137	28.86481	31.21397	31.21397	22.69905	31.63062
17496742	Bcl7c	B cell CLL/lymphoma 7C	10.09907	9.905433	7.614081	12.24907	9.905501	10.09928
17408163	Bcl9	B cell CLL/lymphoma 9	11.54951	14.5465	19.16165	15.00426	14.59225	14.69038
17516621	Bcl9l	B cell CLL/lymphoma 9-like	27.21009	37.42784	34.05158	29.35939	34.05158	36.07556
17403451	Bcl10	B cell leukemia/lymphoma 10	33.49607	41.57545	34.79167	35.25021	39.7199	35.90095
17283871	Bcl11b	B cell leukemia/lymphoma 11B	4.586647	7.520351	5.543396	7.361705	4.683769	4.777911
17226043	Bcl2	B cell leukemia/lymphoma 2	10.61035	9.521111	11.77765	9.07099	9.706963	8.231127
17520162	Bcl2a1a	B cell leukemia/lymphoma 2 related protein A1a	6.992896	6.855301	6.794983	7.432616	6.992896	8.420524
17520171	Bcl2a1b	B cell leukemia/lymphoma 2 related protein A1b	5.520657	4.619126	7.07598	4.959003	7.093167	5.918699
17522726	Bcl2a1c	B cell leukemia/lymphoma 2 related protein A1c	5.876552	6.586947	6.828837	6.434732	6.045744	7.558029
17529575	Bcl2a1d	B cell leukemia/lymphoma 2 related protein A1d	11.04139	10.97524	13.58036	10.95722	10.82144	8.624495
17487457	Bcl3	B cell leukemia/lymphoma 3	64.89566	54.85571	62.63773	64.22798	50.44747	31.93087
17329433	Bcl6	B cell leukemia/lymphoma 6	39.08681	31.04287	20.42296	105.2721	93.80546	74.66602
17364565	Blnk	B cell linker	7.31126	11.43829	10.41665	9.178956	9.991076	8.535585
17280552	Bcap29	B cell receptor associated protein 29	37.13609	37.49769	36.07206	39.34886	40.35345	37.903
17542382	Bcap31	B cell receptor associated protein 31	218.2155	220.8832	213.6551	215.5278	180.1481	211.8119
17410581	Bank1	B cell scaffold protein with ankyrin repeats 1	20.57114	19.38609	26.88203	17.64797	17.83575	17.31944
17236787	Btg1	B cell translocation gene 1, anti-proliferative	26.46952	30.16116	34.72325	28.32806	31.44939	31.14546
17541134	Btg1-ps1	B cell translocation gene 1, anti-proliferative, pseudogene 1	4.496679	5.311318	3.703322	4.20927	5.185884	4.396118
17541140	Btg1-ps2	B cell translocation gene 1, anti-proliferative, pseudogene 2	4.122101	4.514731	4.814981	4.645359	5.662582	4.03713
17227089	Btg2	B cell translocation gene 2, anti-proliferative	28.61481	31.11115	43.74269	19.45137	63.97073	40.57559
17331589	Btg3	B cell translocation gene 3	30.0666	21.70191	17.25461	18.37739	22.64782	10.28023
17517200	Btg4	B cell translocation gene 4	3.288615	3.060442	2.98477	2.884058	2.961422	3.080298
17295525	Bdp1	B double prime 1, subunit of RNA polymerase III transcription initiation factor IIIB	45.48062	42.18418	38.33021	33.03698	40.58909	36.72626
17307623	Blk	B lymphoid kinase	6.922101	8.297864	8.122929	8.976232	6.930935	9.232683
17250597	B9d1	B9 protein domain 1	18.12463	16.47687	16.47687	15.77436	15.82683	17.76838
17263835	B9d1os	B9 protein domain 1, opposite strand	4.821092	4.20927	5.526362	4.804612	5.292429	4.670931
17475336	B9d2	B9 protein domain 2	10.03098	11.24366	10.09802	9.528374	11.24366	11.24366
17548324	Anp32b-ps1	Bacidic (leucine-rich) nuclear phosphoprotein 32 family, member B, pseudogene 1	148.7411	207.8185	214.3404	176.1939	236.1959	207.8185

17378808	Bpi	bactericidal permeablility increasing protein	4.987822	4.113681	4.20927	4.20927	4.148903	4.196132
17523852	Birc2	baculoviral IAP repeat-containing 2	27.16675	24.17627	24.37162	27.41968	32.39816	31.3547
17523861	Birc2	baculoviral IAP repeat-containing 2	8.839961	10.00438	8.493446	8.493446	7.366452	8.493446
17523863	Birc3	baculoviral IAP repeat-containing 3	29.23876	27.02195	29.61939	33.53208	36.7665	36.70692
17258867	Birc5	baculoviral IAP repeat-containing 5	2.567635	3.263862	3.263862	3.058809	3.396573	2.52997
17339630	Birc6	baculoviral IAP repeat-containing 6	138.5612	137.0264	131.667	134.8891	127.0348	128.111
17380879	Birc7	baculoviral IAP repeat-containing 7 (livin)	3.705633	3.784598	3.931337	3.985774	4.252526	4.217915
17259276	Bahcc1	BAH domain and coiled-coil containing 1	18.58131	17.68826	17.82487	17.82487	14.07058	17.82487
17454977	Baiap2l1	BAI1-associated protein 2-like 1	111.8468	131.1615	159.4257	132.5663	155.9868	120.7835
17319150	Baiap2l2	BAI1-associated protein 2-like 2	6.064211	7.948375	8.170192	9.450758	7.373604	8.046994
17342182	Baiap3	BAI1-associated protein 3	13.59986	13.35465	16.26434	12.43332	10.87241	14.3522
17361385	Bbs1	Bardet-Biedl syndrome 1 (human)	10.20207	12.21879	11.70489	10.93234	10.04489	12.59658
17237247	Bbs10	Bardet-Biedl syndrome 10 (human)	7.302953	7.072205	7.087172	7.865236	6.105968	8.67594
17397120	Bbs12	Bardet-Biedl syndrome 12 (human)	8.913549	7.725402	8.666864	8.095274	7.550018	6.734123
17511834	Bbs2	Bardet-Biedl syndrome 2 (human)	8.175064	10.46369	10.48228	11.50086	11.466	13.59656
17527769	Bbs4	Bardet-Biedl syndrome 4 (human)	6.216704	6.11482	7.18242	8.555546	7.345649	6.2941
17371436	Bbs5	Bardet-Biedl syndrome 5 (human)	9.032279	6.975758	6.250017	7.019898	5.75753	7.31638
17404835	Bbs7	Bardet-Biedl syndrome 7 (human)	17.07472	13.95635	15.78957	14.86884	15.28707	14.32338
17515446	Bbs9	Bardet-Biedl syndrome 9 (human)	14.2762	17.63378	17.35609	17.35609	16.25115	12.43324
17383441	Barhl1	BarH-like 1 (Drosophila)	5.276532	5.255471	4.91016	5.999199	4.903528	4.115136
17450536	Barhl2	BarH-like 2 (Drosophila)	8.114206	8.785412	7.063631	9.379883	10.52056	11.14643
17287093	Barx1	BarH-like homeobox 1	19.67661	18.71626	18.62954	18.11697	20.06432	17.82067
17525296	Barx2	BarH-like homeobox 2	4.658934	3.829059	3.557244	4.250463	3.060739	4.475845
17361552	Banf1	barrier to autointegration factor 1	31.66155	35.81396	44.55854	33.73817	35.57939	42.35352
17377019	Banf2	barrier to autointegration factor 2	5.791351	6.519268	6.171709	9.079545	7.221207	6.978902
17392366	Banf2os	barrier to autointegration factor 2, opposite strand	5.293016	5.518438	5.563374	5.046594	4.17947	4.993564
17427784	Bsnd	Bartter syndrome, infantile, with sensorineural deafness (Barttin)	6.350895	5.940853	6.953069	5.085501	5.703668	6.573993
17277251	Bbof1	basal body orientation factor 1	6.245599	5.458483	5.696793	6.087247	5.613148	6.539725
17487422	Bcam	basal cell adhesion molecule	14.78754	17.5941	16.3182	17.05733	14.74486	16.79267
17537853	Bhlhb9	basic helix-loop-helix domain containing, class B9	10.00043	14.16482	15.09438	11.28356	11.28356	9.605096
17444481	Bhlha15	basic helix-loop-helix family, member a15	8.801443	8.21887	6.564704	6.441336	9.866853	8.133013
17253170	Bhlha9	basic helix-loop-helix family, member a9	12.27388	13.76326	13.39423	16.02164	13.73162	12.10734
17396195	Bhlhe22	basic helix-loop-helix family, member e22	5.457348	6.431922	5.96371	6.204307	4.317721	8.112294
17395684	Bhlhe23	basic helix-loop-helix family, member e23	7.814535	7.814535	8.505936	7.064855	7.895004	7.995122
17461414	Bhlhe40	basic helix-loop-helix family, member e40	106.9669	154.0879	179.4614	93.79051	155.6714	134.2511
17472760	Bhlhe41	basic helix-loop-helix family, member e41	3.462322	6.043943	4.763692	6.154834	5.390765	5.629786
17213139	Bzw1	basic leucine zipper and W2 domains 1	223.0016	197.1473	159.4754	194.0857	192.326	203.1315
17280760	Bzw2	basic leucine zipper and W2 domains 2	17.60679	21.09487	21.79024	19.04605	24.65195	25.92748
17229166	Blzf1	basic leucine zipper nuclear factor 1	18.59187	15.27817	16.15882	18.52742	16.15781	15.71293
17277404	Batf	basic leucine zipper transcription factor, ATF-like	11.97455	13.59948	11.37465	16.11207	9.621421	12.42583
17356662	Batf2	basic leucine zipper transcription factor, ATF-like 2	10.48228	10.48228	10.29934	10.66218	10.48228	11.30595
17220787	Batf3	basic leucine zipper transcription factor, ATF-like 3	7.698994	8.20902	8.343831	7.545884	7.800086	6.398883
17295362	Btf3	basic transcription factor 3	82.61776	111.9562	119.3405	78.19367	89.74813	96.82784
17428116	Btf3l4	basic transcription factor 3-like 4	47.02057	58.82592	53.45155	58.82592	51.31825	61.74327
17212406	Bivm	basic, immunoglobulin-like variable motif containing	22.89562	19.13749	20.59425	20.80055	22.59748	21.15593
17234926	Bsg	basigin	523.244	652.2359	794.0302	521.6361	682.8524	555.676
17492870	Bnc1	basonuclin 1	5.41706	3.278737	3.708125	3.969122	4.376052	4.425284
17426929	Bnc2	basonuclin 2	3.193946	4.375021	3.995705	3.995705	3.263862	3.995705
17531154	Bsn	bassoon	8.969142	8.968272	8.779081	8.601868	8.601868	9.277268
17414572	Bspry	B-box and SPRY domain containing	8.37158	7.281471	8.61619	7.281471	7.281471	7.169189
17365721	Bbip1	BBSome interacting protein 1	26.72207	24.02776	25.90215	22.83777	23.67299	26.97386
17321578	Bcdin3d	BCDIN3 domain containing	11.40971	9.289043	10.03077	11.43148	12.14592	15.56539
17298823	Gm3219	B-cell CLL/lymphoma 7C pseudogene	72.02071	57.78928	47.50739	60.75916	59.96089	50.7709
17338529	Gm7334	B-cell translocation gene 3 pseudogene	23.64396	14.37799	15.29067	18.70588	23.47576	7.552635
17474097	Bbc3	BCL2 binding component 3	8.871758	13.88072	11.74781	10.57862	11.68868	13.96535
17389724	Bmf	BCL2 modifying factor	8.676721	9.573391	10.39422	12.8683	10.83217	11.43639
17407749	Bnipl	BCL2/adenovirus E1B 19kD interacting protein like	6.661283	8.092469	8.612846	11.13662	8.959635	8.415856
17334970	Bnip1	BCL2/adenovirus E1B interacting protein 1	34.82907	35.07254	33.12182	35.99189	41.44511	45.13829
17518991	Bnip2	BCL2/adenovirus E1B interacting protein 2	75.22603	69.27006	60.42945	59.49885	69.99791	69.07348
17497421	Bnip3	BCL2/adenovirus E1B interacting protein 3	99.6239	152.3938	159.7974	114.0964	181.1687	149.1955
17307923	Bnip3l	BCL2/adenovirus E1B interacting protein 3-like	206.8025	210.2395	202.3319	193.5993	201.9158	177.1306
17342676	Bak1	BCL2-antagonist/killer 1	22.04378	26.98714	37.02788	29.22781	28.29098	26.92604
17356974	Bad	BCL2-associated agonist of cell death	13.54642	13.44772	13.40268	13.49909	12.78004	11.51554
17424094	Bag1	BCL2-associated athanogene 1	58.03133	58.45968	61.94303	64.46034	65.01272	64.02707
17222020	Bag2	BCL2-associated athanogene 2	19.42873	17.13649	16.67159	15.12601	18.08134	17.93132
17483733	Bag3	BCL2-associated athanogene 3	92.94981	81.69467	75.29019	63.94723	62.51455	53.6007
17508416	Bag4	BCL2-associated athanogene 4	91.30488	86.95457	94.16767	86.33837	81.92546	93.88287
17284124	Bag5	BCL2-associated athanogene 5	21.83726	22.19357	20.13844	23.29454	17.81907	25.39302
17336987	Bag6	BCL2-associated athanogene 6	101.2908	116.5431	105.1613	86.03489	107.3732	105.1613
17231946	Bclaf1	BCL2-associated transcription factor 1	84.09103	131.7017	103.3977	105.9052	106.8646	113.9968
17490839	Bax	BCL2-associated X protein	41.66459	43.65273	41.96108	36.20056	44.91359	34.41892
17313647	Bik	BCL2-interacting killer	23.65773	25.88241	28.15345	29.10328	20.44605	29.61138
17392991	Bcl2l1	BCL2-like 1	25.08217	26.80815	24.12421	23.70435	29.81466	22.89182
17519498	Bcl2l10	Bcl2-like 10	3.581118	3.600731	3.715561	3.781766	4.039705	3.036607
17375961	Bcl2l11	BCL2-like 11 (apoptosis facilitator)	7.404231	8.420466	10.77914	9.801075	9.296322	11.2109
17490521	Bcl2l12	BCL2-like 12 (proline rich)	18.65086	17.39511	18.16261	18.3518	20.65959	19.44072
17462407	Bcl2l13	BCL2-like 13 (apoptosis facilitator)	39.45838	42.26935	43.11327	44.7914	41.20222	43.05861
17463689	Bcl2l14	BCL2-like 14 (apoptosis facilitator)	3.627436	3.06093	2.968841	4.54938	4.080823	4.255239
17300349	Bcl2l2	BCL2-like 2	19.14891	17.81166	17.3526	15.17516	13.96961	21.79779
17216063	Bok	BCL2-related ovarian killer	25.87846	25.53404	25.09295	32.06528	28.20443	28.06013
17534593	Bcorl1	BCL6 co-repressor-like 1	19.26126	27.69877	24.8863	20.86438	23.62185	26.64753
17540243	Bcor	BCL6 interacting corepressor	17.77946	24.4381	20.29525	20.29525	21.09662	20.86626
17401261	Bcl2l15	BCLl2-like 15	2.720006	2.686039	2.83744	3.114477	3.0932	2.606909
17214293	Bcs1l	BCS1-like (yeast)	32.73336	39.62777	32.02574	33.28845	23.74859	32.88232

17392322	Bfsp1	beaded filament structural protein 1, in lens-CP94	5.779361	6.551204	5.993588	8.743495	6.171709	7.336541
17530301	Bfsp2	beaded filament structural protein 2, phakinin	3.386394	3.849577	4.819488	3.273967	3.764191	4.454952
17269845	Becn1	beclin 1, autophagy related	48.56416	46.91509	44.72067	46.22784	48.56416	54.0132
17233014	Bend3	BEN domain containing 3	17.93219	21.44887	23.8279	20.56744	22.18803	22.69464
17448467	Bend4	BEN domain containing 4	14.11063	9.05428	10.43147	10.51845	7.679487	9.379688
17416955	Bend5	BEN domain containing 5	6.443256	6.337307	6.152531	4.978117	6.419273	6.273802
17222053	Bend6	BEN domain containing 6	3.550691	5.051003	6.938674	4.440186	5.297274	4.715105
17366484	Bend7	BEN domain containing 7	27.1013	32.9115	31.57082	26.36076	30.62313	32.46245
17254840	Bzrap1	benzodiazepine receptor associated protein 1	6.497524	8.37663	6.19232	5.45765	5.848108	6.822072
17357236	Bscl2	Berardinelli-Seip congenital lipodystrophy 2 homolog (seipin)	70.53219	72.6283	82.25834	81.22771	69.25758	74.44071
17362579	Best1	bestrophin 1	3.719942	3.883345	4.583215	4.256536	4.073503	4.075847
17511263	Best2	bestrophin 2	4.950348	6.438747	7.219255	5.826017	6.541539	6.7605
17237522	Best3	bestrophin 3	3.364962	3.169686	3.30697	3.205546	3.765053	3.464555
17324420	St6gal1	beta galactoside alpha 2,6 sialyltransferase 1	97.07918	93.89523	101.489	76.69763	110.1687	93.6119
17338599	St6gal2	beta galactoside alpha 2,6 sialyltransferase 2	4.18936	4.268946	4.141921	4.18936	4.518143	4.217944
17515551	B3gat1	beta-1,3-glucuronyltransferase 1 (glucuronosyltransferase P)	19.49537	19.49537	19.49537	18.21582	20.18889	22.2279
17211445	B3gat2	beta-1,3-glucuronyltransferase 2 (glucuronosyltransferase S)	5.989726	6.654268	6.603404	7.98864	7.710583	8.846949
17357306	B3gat3	beta-1,3-glucuronyltransferase 3 (glucuronosyltransferase I)	49.22775	49.22775	49.28989	61.26028	49.22775	49.22775
17356339	B4gat1	beta-1,4-glucuronyltransferase 1	25.1295	26.56436	30.22436	30.24406	22.77123	28.14936
17237937	B4galnt1	beta-1,4-N-acetyl-galactosaminyl transferase 1	68.31971	74.91538	89.699	78.17796	89.21968	68.46477
17268243	B4galnt2	beta-1,4-N-acetyl-galactosaminyl transferase 2	4.484478	4.477551	3.339703	4.020653	4.307826	3.625852
17470394	B4galnt3	beta-1,4-N-acetyl-galactosaminyl transferase 3	8.269213	6.218385	5.688113	7.340254	7.571504	7.169189
17484710	B4galnt4	beta-1,4-N-acetyl-galactosaminyl transferase 4	3.969067	5.029587	5.72855	6.036909	5.077611	5.644399
17375373	B2m	beta-2 microglobulin	3500.05	3543.968	3699.206	3444.145	3440.184	3525.641
17445044	B3glct	beta-3-glucosyltransferase	10.4413	7.61709	9.385997	11.98768	8.134367	9.777462
17505938	Bco1	beta-carotene oxygenase 1	19.93608	19.8572	22.49855	18.49649	18.49649	14.83991
17526820	Bco2	beta-carotene oxygenase 2	32.60429	24.84046	24.78422	33.0686	33.13445	44.51223
17449585	Btc	betacellulin, epidermal growth factor family member	14.06931	12.99694	12.24313	13.08081	13.10313	11.78999
17331284	Crybg3	beta-gamma crystallin domain containing 3	25.02781	24.03092	24.10031	25.02781	20.81094	16.42476
17331306	Crybg3	beta-gamma crystallin domain containing 3	32.63572	21.48027	23.8279	20.39396	16.15938	18.2416
17295005	Bhmt	betaine-homocysteine methyltransferase	11.34724	10.81559	10.74669	11.84544	10.14636	9.325622
17295017	Bhmt2	betaine-homocysteine methyltransferase 2	324.9131	309.7993	369.6894	379.5484	429.9217	398.7222
17423724	Bhmt-ps1	betaine-homocysteine methyltransferase, pseudogene 1	50.2374	36.67767	48.20865	66.32542	50.25482	71.33014
17298504	Msmb	beta-microseminoprotein	5.366829	4.180426	3.49434	6.533291	4.336918	5.488188
17516837	Bace1	beta-site APP cleaving enzyme 1	42.0945	48.01755	51.03093	39.73697	28.3951	40.56884
17327544	Bace2	beta-site APP-cleaving enzyme 2	12.20144	13.75382	13.98637	11.94504	11.32328	11.26855
17359834	Btrc	beta-transducin repeat containing protein	52.42707	47.48467	48.30197	46.11327	39.21436	52.24098
17470457	Bid	BH3 interacting domain death agonist	14.7776	13.13159	18.3147	14.55235	16.26896	14.55689
17241731	Bicc1	bicaudal C homolog 1 (Drosophila)	23.24986	27.99952	25.49443	24.02043	25.49443	21.89531
17464514	Bicd1	bicaudal D homolog 1 (Drosophila)	4.552819	4.782218	5.536714	6.865239	3.600407	6.253266
17287115	Bicd2	bicaudal D homolog 2 (Drosophila)	17.09864	17.09864	18.20963	17.09864	12.59562	18.01854
17323042	Bfar	bifunctional apoptosis regulator	61.11989	57.12103	59.96588	56.12182	61.11989	59.0977
17535558	Bgn	biglycan	400.9532	457.2058	530.0079	379.3091	233.9052	296.7841
17425221	Baat	bile acid-Coenzyme A: amino acid N-acyltransferase	278.5206	223.8657	195.0351	277.6455	266.557	232.9473
17375767	Blvra	biliverdin reductase A	34.00323	44.46012	36.8944	36.8944	37.29447	36.05606
17475580	Blvrb	biliverdin reductase B (flavin reductase (NADPH))	100.1432	120.4199	99.69851	101.4792	57.80771	67.90708
17473925	Bsph1	binder of sperm protein homolog 1	2.33689	2.420844	2.074725	2.251849	2.251849	2.139524
17486659	Bsph2	binder of sperm protein homolog 2	2.19544	3.775297	2.255364	2.625842	2.379997	2.625842
17246368	Bloc1s1	biogenesis of lysosomal organelles complex-1, subunit 1	33.72181	35.16504	36.88392	39.37806	43.12851	41.00279
17365072	Bloc1s2	biogenesis of lysosomal organelles complex-1, subunit 2	17.29916	25.45646	17.76973	15.58472	30.13128	15.57305
17370804	Bloc1s2-ps	biogenesis of lysosomal organelles complex-1, subunit 2, pseudogene	6.147629	7.334355	7.662897	13.8031	7.814535	9.646262
17487272	Bloc1s3	biogenesis of lysosomal organelles complex-1, subunit 3	38.52572	35.72619	43.00045	35.19284	38.17298	40.40607
17447506	Bloc1s4	biogenesis of lysosomal organelles complex-1, subunit 4, cappuccino	28.94154	26.89489	23.8279	23.35404	25.11104	23.8279
17291953	Bloc1s5	biogenesis of lysosomal organelles complex-1, subunit 5, muted	14.72525	13.21578	13.69711	13.29222	13.37494	15.19042
17375526	Bloc1s6	biogenesis of lysosomal organelles complex-1, subunit 6, pallidin	12.76375	14.65601	16.29943	12.55422	19.78657	16.29943
17390833	Bloc1s6os	biogenesis of lysosomal organelles complex-1, subunit 6, pallidin, opposite strand	4.019873	3.593401	2.920489	3.289846	3.289846	2.848357
17261439	Bod1	biorientation of chromosomes in cell division 1	8.009432	6.82666	6.287864	5.984456	6.119102	7.890737
17447757	Bod1l	biorientation of chromosomes in cell division 1-like	45.73003	45.03173	44.58449	47.96101	47.70902	47.96101
17298460	Btd	biotinidase	40.81705	36.21486	33.53347	40.39963	41.71516	34.53052
17286487	Bphl	biphenyl hydrolase-like (serine hydrolase, breast epithelial mucin-associated antig	322.7918	259.8473	248.0367	308.8045	290.1892	299.4892
17330577	Boc	biregional cell adhesion molecule-related/down-regulated by oncogenes (Cdon) b	4.776719	4.662003	5.618987	5.111476	5.151312	4.810811
17220533	Bpnt1	bisphosphate 3-nucleotidase 1	74.14307	83.33265	72.74318	79.3116	68.71009	77.07387
17393755	Blcap	bladder cancer associated protein homolog (human)	6.584619	7.267101	7.487012	10.08417	10.19458	7.12342
17253191	Blmh	bleomycin hydrolase	74.34274	67.09763	70.2346	69.20767	71.68404	74.48304
17463702	Borcs5	BLOC-1 related complex subunit 5	24.87458	27.76162	27.49867	24.44013	19.62866	26.53032
17251500	Borcs6	BLOC-1 related complex subunit 6	13.17645	16.99443	16.99443	15.50842	16.99443	16.56608
17360078	Borcs7	BLOC-1 related complex subunit 7	12.44315	9.364552	9.005398	11.02128	8.826736	9.896923
17501818	Borcs8	BLOC-1 related complex subunit 8	57.93888	67.07531	57.49761	55.9199	52.70288	45.7776
17318483	Bop1	block of proliferation 1	39.61487	36.46777	35.36107	36.95941	34.56165	33.10162
17464580	Bet1	blocked early in transport 1 homolog (S. cerevisiae)	89.59958	56.00447	46.70904	60.98912	60.10987	54.39492
17497687	Bet1l	blocked early in transport 1 homolog (S. cerevisiae)-like	25.37243	24.3576	24.7856	26.21172	26.08503	22.28591
17233104	Bves	blood vessel epicardial substance	3.185235	3.828263	3.337806	3.339703	3.632015	3.951335
17270189	Bloodlinc	Bloodlinc, erythroid developmental long intergenic non-protein coding transcript	8.160344	7.627393	8.451164	6.128738	4.963026	6.351336
17492619	Blm	Bloom syndrome, RecQ helicase-like	4.932264	5.12748	5.35672	5.485564	7.002548	5.470755
17367295	Bmi1	Bmi1 polycomb ring finger oncogene	7.698994	7.698994	6.017357	6.906621	7.698994	8.445483
17348105	Bambi	BMP and activin membrane-bound inhibitor	16.78534	14.56012	9.059679	13.82675	18.13997	11.51402
17390808	Bambi-ps1	BMP and activin membrane-bound inhibitor, pseudogene (Xenopus laevis)	12.14937	9.345553	15.25024	10.21098	10.72838	13.39423
17439481	Bmp2k	BMP2 inducible kinase	61.8525	82.82819	87.75929	60.63421	66.39672	60.1478
17515478	Bmper	BMP-binding endothelial regulator	5.696082	6.171709	6.171709	4.801649	9.038855	6.599423
17470150	Bms1	BMS1 homolog, ribosome assembly protein (yeast)	50.28618	40.27967	36.43543	41.14	41.7114	40.75344
17545955	Bmx	BMX non-receptor tyrosine kinase	5.036355	5.341149	5.150741	4.968327	4.911045	4.447609
17330875	Bbx	bobby sox homolog (Drosophila)	22.11909	17.91294	20.51191	16.86289	15.2116	18.57422
17223261	Boll	bol, boule-like (Drosophila)	2.576228	2.547885	2.67402	2.397681	2.782767	2.474951
17408001	Bola1	bolA-like 1 (E. coli)	17.41417	16.13286	18.59419	19.82089	15.81016	20.30003

17483075	Bola2	bolA-like 2 (E. coli)	25.27501	24.79367	26.05413	27.56107	25.22776	17.38559
17460073	Bola3	bolA-like 3 (E. coli)	34.79553	45.29438	50.58894	53.18399	45.36098	48.40687
17535012	Brs3	bombesin-like receptor 3	5.946744	4.672517	4.647678	6.089062	3.87262	3.729055
17406737	Bglap	bone gamma carboxyglutamate protein	6.992896	5.317912	5.355124	6.241964	5.016392	5.73137
17406730	Bglap2	bone gamma-carboxyglutamate protein 2	10.99747	10.99747	11.87495	9.4484	10.99747	11.86697
17406718	Bglap3	bone gamma-carboxyglutamate protein 3	13.40984	11.188	11.70133	12.61773	12.3734	12.04832
17437198	Bst1	bone marrow stromal cell antigen 1	5.406032	6.581699	6.651686	7.067647	7.513994	6.763594
17510345	Bst2	bone marrow stromal cell antigen 2	427.0615	405.0623	557.984	402.0079	274.5281	353.7583
17308361	Bmp1	bone morphogenetic protein 1	84.52464	95.80905	110.6186	100.1182	111.0696	106.4299
17460545	Bmp10	bone morphogenetic protein 10	24.77495	17.63769	12.02104	17.39246	16.8428	18.85558
17539772	Bmp15	bone morphogenetic protein 15	5.268309	4.823198	5.968713	5.304843	6.011062	5.364784
17376677	Bmp2	bone morphogenetic protein 2	25.12427	28.34181	24.93638	36.7232	14.43351	25.22688
17439535	Bmp3	bone morphogenetic protein 3	6.455326	6.919223	6.76833	6.76833	7.508892	6.76833
17305709	Bmp4	bone morphogenetic protein 4	13.41839	12.74191	19.97689	10.77003	12.40896	13.79812
17519538	Bmp5	bone morphogenetic protein 5	25.90377	32.19289	27.91619	33.3448	14.92967	35.55499
17286682	Bmp6	bone morphogenetic protein 6	9.745053	10.92476	10.92476	9.380469	11.33545	16.55666
17395041	Bmp7	bone morphogenetic protein 7	14.80918	16.49092	13.71507	16.97547	18.94874	17.4996
17429674	Bmp8a	bone morphogenetic protein 8a	9.806104	9.782749	8.713063	12.77525	11.75938	8.514548
17418141	Bmp8b	bone morphogenetic protein 8b	8.776222	6.258383	10.23784	5.477699	4.632176	4.933085
17305042	Bmpr1a	bone morphogenetic protein receptor, type 1A	61.17075	57.01699	77.59921	62.02767	58.24773	64.22798
17410729	Bmpr1b	bone morphogenetic protein receptor, type 1B	3.494946	2.75704	3.009972	3.386512	2.845732	3.212174
17213321	Bmpr2	bone morphogenetic protein receptor, type II (serine/threonine kinase)	287.5582	292.2015	319.11	280.5628	214.082	236.6301
17213336	Bmpr2	bone morphogenetic protein receptor, type II (serine/threonine kinase)	40.39794	39.64728	39.24835	48.8911	34.58993	43.9966
17218044	Brinp3	bone morphogenetic protein/retinoic acid inducible neural specific 3	4.383369	4.539206	3.442914	4.012246	3.157363	4.394777
17426434	Brinp1	bone morphogenic protein/retinoic acid inducible neural specific 1	6.068626	7.230824	6.33823	7.494592	6.625136	7.351099
17228656	Brinp2	bone morphogenic protein/retinoic acid inducible neural-specific 2	9.744715	12.54873	9.310251	10.5666	9.277139	9.744715
17302362	Bora	bora, aurora kinase A activator	3.838227	3.838227	3.292104	3.752937	3.142146	3.838227
17378125	Bpifa1	BPI fold containing family A, member 1	3.189765	2.947517	3.036354	3.03707	2.678899	3.301176
17378104	Bpifa2	BPI fold containing family A, member 2	5.007221	5.266301	4.28546	3.828104	4.339353	4.9283
17378116	Bpifa3	BPI fold containing family A, member 3	5.344148	5.137548	5.48933	5.289642	4.124817	5.983253
17378138	Bpifa5	BPI fold containing family A, member 5	3.339565	3.220512	3.400342	3.411296	3.27617	3.176967
17378092	Bpifa6	BPI fold containing family A, member 6	2.283236	2.42034	2.467431	2.521811	2.467431	2.072138
17378149	Bpifb1	BPI fold containing family B, member 1	3.70553	3.256788	3.727323	3.301679	3.090007	3.182962
17378017	Bpifb2	BPI fold containing family B, member 2	4.315088	4.801649	5.215191	5.145602	4.801516	6.998133
17378053	Bpifb3	BPI fold containing family B, member 3	3.954266	5.207244	4.759335	3.746179	5.239104	4.821493
17378072	Bpifb4	BPI fold containing family B, member 4	4.326169	4.212422	4.171077	4.067155	3.867738	4.016197
17378168	Bpifb5	BPI fold containing family B, member 5	5.597218	5.993298	5.851514	5.186603	5.703312	5.461851
17378035	Bpifb6	BPI fold containing family B, member 6	4.175329	3.99745	3.903342	4.265929	4.085747	4.251818
17378184	Bpifb9a	BPI fold containing family B, member 9A	15.21666	18.03078	14.84495	15.30637	14.7514	15.84527
17378200	Bpifb9b	BPI fold containing family B, member 9B	11.13215	13.80664	13.04179	10.55116	12.49821	13.85524
17243693	Bpifc	BPI fold containing family C	2.755702	2.49789	2.731891	2.769701	2.366295	2.486006
17473341	Brsk1	BR serine/threonine kinase 1	5.253905	5.913532	5.197039	5.798581	6.059211	5.010415
17485152	Brsk2	BR serine/threonine kinase 2	4.339263	3.19264	3.030992	3.886846	4.067268	3.589592
17333121	T	brachyury	6.506673	5.781925	5.799948	5.839197	7.479491	5.908861
17333107	T2	brachyury 2	4.275313	3.870527	3.916052	4.001109	3.707843	4.719356
17278404	Bdkrb1	bradykinin receptor, beta 1	3.270611	3.307176	3.116183	3.266283	2.782362	3.48382
17278398	Bdkrb2	bradykinin receptor, beta 2	7.20316	6.836889	6.553656	6.056272	6.111515	6.064211
17466168	Braf	Braf transforming gene	66.93273	93.41291	85.21527	69.08162	68.55167	71.55546
17316175	Basp1	brain abundant, membrane attached signal protein 1	28.9243	32.10353	27.44174	27.37335	30.56122	36.24977
17311173	Baalc	brain and acute leukemia, cytoplasmic	4.322183	3.722418	3.722418	4.486779	4.844239	4.20927
17436210	Bre	brain and reproductive organ-expressed protein	53.50938	58.31843	65.9989	58.05708	56.73865	53.74617
17374077	Bdnf	brain derived neurotrophic factor	5.484956	5.360398	4.20927	6.969717	6.56698	5.484956
17544734	Bex1	brain expressed gene 1	2.895816	2.537381	2.953407	2.953407	2.935772	2.953407
17537878	Bex4	brain expressed gene 4	3.39509	2.574853	2.441301	2.660726	4.618358	2.78134
17324745	Bex6	brain expressed gene 6	3.219842	3.074539	3.084293	2.326159	3.045354	3.632568
17368171	Bmyc	brain expressed myelocytomatosis oncogene	26.16307	34.82014	28.56447	33.90556	37.59685	32.93227
17544691	Bex2	brain expressed X-linked 2	12.3566	8.127322	12.08571	10.83998	9.59046	10.48148
17504413	Bean1	brain expressed, associated with Nedd4, 1	8.445718	7.32262	7.799437	8.512542	9.04763	8.257585
17377498	Pygb	brain glycogen phosphorylase	24.90131	28.88723	26.89806	20.70719	24.90131	18.09125
17444486	Bri3	brain protein I3	12.8014	10.43978	10.44224	11.15749	10.33646	10.53538
17516385	Bsx	brain specific homeobox	16.75593	16.94197	16.32702	20.22657	16.51334	17.75877
17283947	Begain	brain-enriched guanylate kinase-associated	9.19666	6.284552	9.385346	7.767014	6.883441	6.297023
17259235	Baiap2	brain-specific angiogenesis inhibitor 1-associated protein 2	48.54867	41.8716	42.19763	50.1612	54.77023	36.71633
17472678	Bcat1	branched chain aminotransferase 1, cytosolic	4.590241	4.737251	3.9324	4.280561	3.540149	3.95328
17477897	Bcat2	branched chain aminotransferase 2, mitochondrial	14.79502	20.87754	19.36715	24.84924	16.48783	23.86824
17487982	Bckdha	branched chain ketoacid dehydrogenase E1, alpha polypeptide	213.0193	205.4723	206.2056	226.4251	242.3822	236.3466
17519940	Bckdhb	branched chain ketoacid dehydrogenase E1, beta polypeptide	141.959	157.9627	157.1012	146.961	161.5526	166.3387
17483516	Bckdk	branched chain ketoacid dehydrogenase kinase	38.49449	32.95008	39.53531	43.72147	41.02155	37.98821
17351133	Bvht	braveheart long non-coding RNA	3.339703	3.272039	3.035912	4.20927	5.019626	4.495495
17441952	Brap	BRCA1 associated protein	221.389	305.9559	296.1902	265.9148	263.1607	302.6088
17298407	Bap1	Brca1 associated protein 1	35.16187	33.36492	31.42912	34.07567	33.80278	31.24817
17223999	Bard1	BRCA1 associated RING domain 1	4.611512	3.99709	3.703322	3.896773	5.120306	3.883991
17267209	Brip1	BRCA1 interacting protein C-terminal helicase 1	5.018444	4.352065	4.352065	4.352065	4.352065	4.00991
17254618	Brip1os	BRCA1 interacting protein C-terminal helicase 1, opposite strand	11.00136	11.45504	10.66876	11.67208	11.17978	12.50333
17535957	Brcc3	BRCA1/BRCA2-containing complex, subunit 3	46.58714	47.92977	46.92679	47.95502	50.83076	51.69311
17444117	Brat1	BRCA1-associated ATM activator 1	20.36161	20.42126	23.90748	18.5287	19.20513	21.86589
17484140	Bccip	BRCA2 and CDKN1A interacting protein	53.75846	51.52033	52.54895	60.70023	52.09453	66.76083
17234289	Bcr	breakpoint cluster region	62.49115	60.32608	61.28194	59.41972	52.44088	49.73501
17269921	Brca1	breast cancer 1, early onset	5.919642	5.362627	5.342778	6.226839	5.84357	5.48933
17445162	Brca2	breast cancer 2, early onset	6.266761	6.819755	8.479446	7.698994	7.074362	8.934209
17513158	Bcar1	breast cancer anti-estrogen resistance 1	44.95189	48.05085	51.11341	43.68815	50.25447	52.0754
17402305	Bcar3	breast cancer anti-estrogen resistance 3	109.2925	91.56219	88.42792	84.35607	67.94851	88.42792
17356344	Brms1	breast cancer metastasis-suppressor 1	27.71413	29.7779	27.96674	31.39929	31.75166	30.44345
17275576	Brms1l	breast cancer metastasis-suppressor 1-like	15.61889	14.57294	16.21481	16.38973	12.36723	15.881

17394943	Bcas1	breast carcinoma amplified sequence 1	3.396738	4.413123	3.48353	4.217681	3.968187	4.34697
17380040	Bcas1os1	breast carcinoma amplified sequence 1, opposite strand 1 [Source:MGI Symbol;Ac	3.424112	3.547444	5.120306	3.674403	3.298157	4.220606
17380031	Bcas1os2	breast carcinoma amplified sequence 1, opposite strand 2	5.986364	5.273534	5.406894	6.534741	5.113673	6.074771
17401169	Bcas2	breast carcinoma amplified sequence 2	44.1573	50.69565	55.2568	58.41634	65.05148	65.60255
17254547	Bcas3	breast carcinoma amplified sequence 3	42.27462	35.83506	38.62545	41.30373	43.80853	34.85056
17267186	Bcas3os2	breast carcinoma amplified sequence 3 opposite strand 2	7.042414	5.95095	6.952106	6.222093	7.08894	6.646432
17267174	Bcas3os1	breast carcinoma amplified sequence 3, opposite strand 1	3.758195	3.420673	4.608477	3.472441	3.694579	3.690279
17406629	Bcan	brevican	4.990831	5.848027	4.880639	5.31747	4.456249	5.427847
17284290	Brf1	BRF1 homolog, subunit of RNA polymerase III transcription initiation factor IIIB (S.	24.54011	27.34945	26.63571	31.22825	33.3448	30.66498
17508516	Brf2	BRF2, subunit of RNA polymerase III transcription initiation factor, BRF1-like	8.874218	9.398171	12.16345	11.27871	10.37997	10.06636
17442714	Bri3bp	Bri3 binding protein	64.98613	65.0321	75.55891	70.41008	54.44358	59.10876
17334266	Bricd5	BRICHOS domain containing 5	4.834813	5.615911	7.950524	5.53165	6.576363	8.630655
17461793	Brk1	BRICK1, SCAR/WAVE actin-nucleating complex subunit	81.64994	89.79418	89.968	70.74469	78.25223	90.97448
17349283	Bin1	bridging integrator 1	22.50463	22.61519	21.19556	24.36655	19.55547	22.61519
17321768	Bin2	bridging integrator 2	19.43358	15.51692	24.21401	15.32452	14.31653	12.07558
17301722	Bin3	bridging integrator 3	57.59693	56.6416	51.10668	58.82796	61.30233	67.33945
17502168	Babam1	BRISC and BRCA1 A complex member 1	141.3139	148.7401	166.8028	147.6922	113.482	149.7612
17230651	Brox	BRO1 domain and CAAX motif containing	44.511	43.60707	34.52382	39.38188	39.09846	40.90457
17374669	Bahd1	bromo adjacent homology domain containing 1	17.09864	19.36621	19.22844	19.04394	17.35164	20.75749
17281148	Baz1a	bromodomain adjacent to zinc finger domain 1A	32.28249	31.44655	28.73267	30.9151	24.48048	24.75006
17443231	Baz1b	bromodomain adjacent to zinc finger domain, 1B	68.69913	66.37969	81.041	66.86874	59.32055	61.94646
17238245	Baz2a	bromodomain adjacent to zinc finger domain, 2A	48.41023	56.80279	62.31942	62.73593	59.1723	49.74157
17385508	Baz2b	bromodomain adjacent to zinc finger domain, 2B	23.20478	24.72125	23.39568	25.60724	22.90689	25.73143
17461606	Brpf1	bromodomain and PHD finger containing, 1	19.29145	19.86174	18.08322	15.31666	24.58182	26.94751
17335398	Brpf3	bromodomain and PHD finger containing, 3	20.0243	22.8712	23.0405	18.14777	21.78284	20.19715
17332395	Brwd1	bromodomain and WD repeat domain containing 1	32.25431	33.55812	29.32563	33.28176	28.71554	34.05512
17544102	Brwd3	bromodomain and WD repeat domain containing 3	20.57956	22.51914	20.57956	21.1615	21.1615	25.15686
17320163	Brd1	bromodomain containing 1	26.55258	28.5253	22.6915	23.56396	23.44275	28.61739
17343768	Brd2	bromodomain containing 2	64.41836	65.56118	53.61965	50.96129	51.25852	58.56675
17383320	Brd3	bromodomain containing 3	44.45488	48.68988	47.81297	52.38747	43.42127	50.24437
17343344	Brd4	bromodomain containing 4	66.09366	78.2799	88.13481	73.10712	65.469	69.71465
17511509	Brd7	bromodomain containing 7	53.48899	53.09448	50.61559	50.12332	53.58621	53.09448
17353462	Brd8	bromodomain containing 8	22.52492	21.97589	19.39738	21.97589	28.52708	22.13995
17288585	Brd9	bromodomain containing 9	30.93954	32.8404	32.8404	33.13514	36.62838	33.48702
17271051	Bptf	bromodomain PHD finger transcription factor	52.18669	53.39305	42.85139	48.34819	54.60571	48.6946
17439997	Brdt	bromodomain, testis-specific	4.361154	3.815283	4.090423	4.801649	3.958462	4.654641
17512765	Lncbate1	brown adipose tissue enriched long non-coding RNA 1	6.28281	6.751462	7.210553	7.528018	8.157743	9.652951
17544491	Btk	Bruton agammaglobulinemia tyrosine kinase	5.637713	5.915582	6.317046	4.379846	10.61248	7.475292
17315960	Brix1	BRX1, biogenesis of ribosomes, homolog (S. cerevisiae)	127.7757	80.8564	90.19778	79.83614	102.3913	87.42718
17418999	Bsdc1	BSD domain containing 1	51.94129	55.44325	54.0945	55.11221	59.20635	56.6153
17358962	Btaf1	BTAF1 RNA polymerase II, B-TFIID transcription factor-associated, (Mot1 homolog,	54.15978	47.59803	39.37505	45.29438	38.18648	40.50337
17492847	Btbd1	BTB (POZ) domain containing 1	99.52175	109.9078	93.88938	89.26545	90.8113	93.44334
17495223	Btbd10	BTB (POZ) domain containing 10	6.812291	5.402136	5.373976	6.287864	6.606472	5.855125
17236102	Btbd11	BTB (POZ) domain containing 11	6.992896	8.021377	7.689235	6.992896	7.042414	6.992896
17483870	Btbd16	BTB (POZ) domain containing 16	3.835993	5.013055	4.766235	4.79636	4.854424	4.917619
17271700	Btbd17	BTB (POZ) domain containing 17	5.015801	6.992896	6.734823	6.171709	6.287864	6.250363
17372578	Btbd18	BTB (POZ) domain containing 18	10.39891	8.778716	8.536768	8.927585	8.038075	8.708957
17428752	Btbd19	BTB (POZ) domain containing 19	9.050641	7.032268	9.443686	7.109657	8.247918	9.42028
17428763	Btbd19	BTB (POZ) domain containing 19	6.379264	8.729385	6.912224	8.605088	6.605236	6.434464
17243022	Btbd2	BTB (POZ) domain containing 2	51.68773	57.26731	56.64906	65.07223	52.67038	66.64801
17376867	Btbd3	BTB (POZ) domain containing 3	7.375291	6.675195	7.508475	7.482809	7.450566	7.580011
17279427	Btbd6	BTB (POZ) domain containing 6	38.50089	31.47905	36.7479	31.60345	33.34457	37.30482
17283487	Btbd7	BTB (POZ) domain containing 7	33.00791	33.57906	33.30461	32.32257	37.39569	32.76242
17440042	Btbd8	BTB (POZ) domain containing 8	8.389297	8.447942	7.388134	8.016153	8.016153	6.632901
17343119	Btbd9	BTB (POZ) domain containing 9	27.82173	33.14938	35.93655	28.73804	29.79627	28.77711
17326895	Bach1	BTB and CNC homology 1	67.81112	58.29297	61.10083	61.77024	59.477	62.39636
17412374	Bach2	BTB and CNC homology 2	9.492184	13.05246	13.90498	15.57262	9.616621	12.4796
17412395	Bach2	BTB and CNC homology 2	3.310938	3.7126	3.714788	3.677512	3.459995	3.669338
17423741	Bach2os	BTB and CNC homology 2, opposite strand	6.112659	6.395823	5.979356	6.614536	5.271158	5.567887
17506332	Banp	BTG3 associated nuclear protein	30.04723	25.02607	26.99462	21.3452	27.87442	20.78672
17516938	Bud13	BUD13 homolog (yeast)	19.60174	21.57951	17.38306	19.18597	18.09401	23.4865
17444628	Bud31	BUD31 homolog (yeast)	26.35619	20.7237	17.28118	21.36148	19.66775	21.78087
17391376	Bub1	budding uninhibited by benzimidazoles 1 homolog (S. cerevisiae)	2.387946	2.853119	2.740274	3.168193	3.601181	3.132946
17374569	Bub1b	budding uninhibited by benzimidazoles 1 homolog, beta (S. cerevisiae)	4.978531	5.780603	5.780603	5.671892	5.649331	5.780603
17484040	Bub3	budding uninhibited by benzimidazoles 3 homolog (S. cerevisiae)	100.3614	82.90976	92.65584	80.05114	85.48267	72.62075
17389144	Bbox1	butyrobetaine (gamma), 2-oxoglutarate dioxygenase 1 (gamma-butyrobetaine hyc	331.6229	326.1993	286.613	323.0223	262.8599	325.8739
17291146	Btn1a1	butyrophilin, subfamily 1, member A1	5.364263	4.831764	5.051038	4.886969	3.879713	5.673818
17291158	Btn2a2	butyrophilin, subfamily 2, member A2	3.475836	4.908697	5.592177	4.190203	4.36058	4.712916
17336526	Btnl1	butyrophilin-like 1	3.482178	3.523307	3.590787	2.953407	4.153046	3.092514
17250149	Btnl10	butyrophilin-like 10	6.601848	6.115625	5.398805	5.566807	4.872898	6.026457
17336516	Btnl2	butyrophilin-like 2	7.539036	8.73574	7.801816	7.553682	8.151525	8.647781
17343834	Btnl4	butyrophilin-like 4	4.012941	6.106179	6.807287	6.198976	5.549124	5.80401
17343856	Btnl5-ps	butyrophilin-like 5, pseudogene	2.861755	3.219463	4.206382	2.47574	4.330669	5.442917
17343865	Btnl7-ps	butyrophilin-like 7, pseudogene	3.528634	3.54506	2.417926	2.656838	3.453191	2.606909
17262259	Btnl9	butyrophilin-like 9	18.7304	15.09888	18.2373	17.09734	15.26388	11.70019
17405908	Bche	butyrylcholinesterase	157.4184	149.1314	183.6443	162.946	193.9109	185.9731
17345696	Bysl	bystin-like	31.91892	21.29481	19.70172	16.16353	20.13286	17.60301
17247471	C1d	C1D nuclear receptor co-repressor	12.59553	11.90809	12.48358	13.26451	12.50463	11.01845
17541217	C1galt1c1	C1GALT1-specific chaperone 1	19.27087	15.93934	14.02423	13.74276	13.87946	17.35128
17258902	C1qtnf1	C1q and tumor necrosis factor related protein 1	19.35964	18.27577	18.0994	19.68957	16.34684	15.0992
17248613	C1qtnf2	C1q and tumor necrosis factor related protein 2	5.694188	5.901043	5.972438	5.901043	5.500108	7.504833
17310339	C1qtnf3	C1q and tumor necrosis factor related protein 3	2.873084	3.458676	3.881784	2.941659	2.448215	2.49239
17373105	C1qtnf4	C1q and tumor necrosis factor related protein 4	32.37392	37.14433	36.61036	31.6846	29.29556	35.37112
17516488	C1qtnf5	C1q and tumor necrosis factor related protein 5	8.134592	8.497273	9.014828	10.76339	8.807973	10.27616

17319022	C1qtnf6	C1q and tumor necrosis factor related protein 6	8.762424	9.682301	11.42324	9.372345	8.217446	8.795405
17437148	C1qtnf7	C1q and tumor necrosis factor related protein 7	3.703322	3.703322	3.352971	3.703322	3.729055	4.863989
17301055	C1qtnf9	C1q and tumor necrosis factor related protein 9	7.148844	7.584531	6.017733	7.089383	6.394848	7.639562
17381774	C1ql3	C1q-like 3	9.123011	12.53422	12.2028	12.34342	12.10952	14.31623
17332628	C2cd2	C2 calcium-dependent domain containing 2	152.1942	182.1584	164.3457	159.175	120.4024	161.7677
17526206	C2cd2l	C2 calcium-dependent domain containing 2-like	70.81534	48.48108	60.50993	53.74356	52.04256	52.17331
17480691	C2cd3	C2 calcium-dependent domain containing 3	22.22991	25.70202	28.62989	25.70202	23.83732	24.53195
17528532	C2cd4a	C2 calcium-dependent domain containing 4A	8.51685	10.45101	8.51685	7.767121	5.653013	8.51685
17518813	C2cd4b	C2 calcium-dependent domain containing 4B	8.708112	10.02243	9.75012	11.26738	14.03834	9.619621
17242624	C2cd4c	C2 calcium-dependent domain containing 4C	16.31763	16.95243	15.37197	17.91145	17.48396	17.12676
17400043	C2cd4d	C2 calcium-dependent domain containing 4D	16.84397	21.26059	16.66905	13.28052	15.51466	17.37884
17472598	C2cd5	C2 calcium-dependent domain containing 5	17.16526	15.77436	18.6537	15.14952	15.22436	14.85967
17465115	Cadps2	Ca2+-dependent activator protein for secretion 2	22.02103	17.11821	15.387	18.33439	18.4751	18.52973
17303504	Cadps	Ca2+-dependent secretion activator	4.925704	4.360217	4.422708	4.062844	4.495121	4.360338
17541776	Cxx1a	CAAX box 1A	2.287418	1.784253	2.465329	2.083357	3.353668	2.287418
17541780	Cxx1b	CAAX box 1B	15.46087	16.71081	44.00849	16.37247	13.64537	12.97893
17534746	Cxx1c	CAAX box 1C	5.145709	5.066502	6.009771	5.066502	5.066502	5.114843
17369555	Abl1	c-abl oncogene 1, non-receptor tyrosine kinase	14.37958	14.37958	16.23516	12.03697	11.7877	10.95897
17415863	Cachd1	cache domain containing 1	15.50412	14.06795	15.63774	11.85053	9.750864	14.03736
17235682	Cactin	cactin, spliceosome C complex subunit	20.60325	21.4755	21.04988	22.90245	21.76156	23.82179
17505148	Cdh1	cadherin 1	112.4407	74.51402	81.04437	94.00594	90.07844	79.76368
17310530	Cdh10	cadherin 10	3.234584	2.943617	3.062861	2.732649	3.282602	3.677282
17512153	Cdh11	cadherin 11	7.674219	9.066527	8.401343	9.142065	8.415156	7.76061
17499676	2610005L07Rik	cadherin 11 pseudogene	3.919746	5.780603	4.20927	4.27804	3.926843	3.416976
17507872	2610005L07Rik	cadherin 11 pseudogene	12.6929	16.64527	19.54313	21.33174	15.48136	18.61857
17507851	6820431F20Rik	cadherin 11 pseudogene	243.6084	277.8726	254.2229	261.1781	235.6775	271.5925
17310550	Cdh12	cadherin 12	3.184838	2.347036	2.581	2.738679	3.003428	2.55923
17506042	Cdh13	cadherin 13	9.527845	8.402275	9.1294	9.527845	10.4116	8.908978
17506460	Cdh15	cadherin 15	7.83395	8.151695	8.652458	10.61483	8.652458	12.0657
17512299	Cdh16	cadherin 16	5.677241	7.690727	5.327689	6.479085	5.177659	6.308294
17411848	Cdh17	cadherin 17	3.802531	4.004383	3.756711	4.076271	4.623675	3.98594
17310565	Cdh18	cadherin 18	7.506914	10.88546	7.849877	8.546952	9.52527	8.86389
17226127	Cdh19	cadherin 19, type 2	2.516276	2.782863	2.624695	3.042991	2.516276	2.072138
17352884	Cdh2	cadherin 2	222.1807	220.9154	214.9698	203.1287	213.1699	211.517
17216280	Cdh20	cadherin 20	4.362636	4.786032	3.836791	4.362636	4.576612	4.487121
17394366	Cdh22	cadherin 22	9.606827	10.75332	9.606827	11.58359	9.606827	10.68548
17241053	Cdh23	cadherin 23 (otocadherin)	3.367085	3.641417	3.718678	3.652261	3.617843	3.39128
17505124	Cdh3	cadherin 3	7.144832	7.619889	7.425664	7.098775	6.556382	6.927189
17380637	Cdh4	cadherin 4	4.865473	5.974591	6.225846	5.923706	5.024848	5.411655
17380659	Cdh4	cadherin 4	13.24146	12.90099	11.25169	11.66723	12.74854	15.03559
17504399	Cdh5	cadherin 5	132.1965	128.6573	169.258	127.9494	101.1561	92.61218
17316120	Cdh6	cadherin 6	24.33161	17.82636	13.2113	19.19941	17.71341	17.48057
17216498	Cdh7	cadherin 7, type 2	4.559135	5.611864	4.114508	4.755609	4.175937	5.275179
17512121	Cdh8	cadherin 8	4.039061	3.533064	4.478669	4.050472	4.050472	3.84643
17310515	Cdh9	cadherin 9	3.824046	2.879663	3.164769	3.130797	3.301336	3.402087
17320103	Celsr1	cadherin, EGF LAG seven-pass G-type receptor 1 (flamingo homolog, Drosophila)	30.54554	26.66749	22.29456	22.29456	21.87332	17.4503
17409284	Celsr2	cadherin, EGF LAG seven-pass G-type receptor 2 (flamingo homolog, Drosophila)	6.074139	6.380945	7.085109	6.215369	6.009479	4.768614
17521934	Celsr3	cadherin, EGF LAG seven-pass G-type receptor 3 (flamingo homolog, Drosophila)	3.677894	3.774956	3.766123	4.05986	4.486592	4.983537
17306426	Cdh24	cadherin-like 24	28.52134	25.9282	26.992	28.97908	28.32806	28.20482
17380612	Cdh26	cadherin-like 26	11.80626	12.47476	10.65612	8.133183	11.13755	9.475355
17456339	Cped1	cadherin-like and PC-esterase domain containing 1	127.7376	120.6781	112.1363	109.8827	121.139	122.7348
17305143	Cdhr1	cadherin-related family member 1	5.338965	5.338965	5.338965	5.301461	5.338965	5.379007
17287486	Cdhr2	cadherin-related family member 2	2.606909	2.798182	2.261171	2.319751	2.713265	2.680459
17280640	Cdhr3	cadherin-related family member 3	4.8479	5.338965	5.171167	5.386134	5.616105	5.338965
17521570	Cdhr4	cadherin-related family member 4	5.793921	5.43018	6.690157	6.785946	6.077761	7.009735
17497829	Cdhr5	cadherin-related family member 5	112.4126	105.8274	106.1971	119.2181	105.8274	89.85333
17411966	Calb1	calbindin 1	3.703322	3.353366	3.703322	3.703322	4.314669	4.547835
17512872	Calb2	calbindin 2	3.673894	2.953652	3.027653	3.094486	3.950486	2.963208
17241867	Cabin1	calcineurin binding protein 1	28.79707	30.52967	31.82768	31.73978	26.02759	26.65215
17374814	Chp1	calcineurin-like EF hand protein 1	115.7453	109.9421	107.6646	94.04504	83.65791	81.67428
17482752	Chp2	calcineurin-like EF hand protein 2	10.00563	13.35798	11.80724	11.98402	13.64102	9.696876
17328225	Cpped1	calcineurin-like phosphoesterase domain containing 1	34.49965	34.80228	31.35579	32.70683	38.47795	30.89454
17442977	Crcp	calcitonin gene-related peptide-receptor component protein	52.77307	54.81618	65.90427	75.41241	70.42667	72.17013
17464549	Calcr	calcitonin receptor	3.339703	3.25864	3.339703	3.339703	3.339703	4.02149
17387385	Calcrl	calcitonin receptor-like	63.99335	54.54708	57.76285	44.12181	27.64545	36.88826
17495328	Calca	calcitonin/calcitonin-related polypeptide, alpha	4.188286	5.300432	4.975012	4.630025	4.206849	5.01667
17482041	Calcb	calcitonin-related polypeptide, beta	6.171709	4.348085	4.858834	4.276795	4.430931	5.874557
17272798	Cant1	calcium activated nucleotidase 1	30.49879	31.7334	29.89744	20.24256	28.75578	28.97989
17492495	Cib1	calcium and integrin binding 1 (calmyrin)	37.29835	39.17035	39.30879	37.30042	39.28972	42.89894
17527261	Cib2	calcium and integrin binding family member 2	30.247	30.44451	30.40043	29.76263	26.33373	33.16478
17510484	Cib3	calcium and integrin binding family member 3	18.16312	10.65087	11.52544	12.01671	16.68824	18.22605
17446740	Cib4	calcium and integrin binding family member 4	3.977522	3.569767	4.213853	3.176548	2.864493	3.269024
17322289	Calcoco1	calcium binding and coiled coil domain 1	70.41252	83.98152	90.80501	83.98152	72.03669	84.29411
17268301	Calcoco2	calcium binding and coiled-coil domain 2	3.993545	3.92483	3.641594	3.92483	3.628567	3.729055
17451677	Cabp1	calcium binding protein 1	6.220799	5.289642	5.304953	4.886969	4.855601	4.886969
17356084	Cabp2	calcium binding protein 2	9.023143	8.573891	7.077403	8.073364	7.814535	6.446652
17214983	Cab39	calcium binding protein 39	101.3962	102.3608	99.27372	103.2516	99.85204	89.96114
17300984	Cab39l	calcium binding protein 39-like	224.167	194.9946	195.7556	206.6377	226.9656	206.1913
17361112	Cabp4	calcium binding protein 4	7.651751	5.844947	7.698994	6.074855	6.210589	7.162881
17473914	Cabp5	calcium binding protein 5	3.110098	3.299666	3.362863	3.263862	3.2428	3.754758
17260029	Cabp7	calcium binding protein 7	5.688073	6.678805	6.097974	6.556973	5.514844	6.00723
17438716	Cabs1	calcium binding protein, spermatid specific 1	4.502512	5.012464	4.782848	4.554713	4.759665	4.394412
17368456	Cacfd1	calcium channel flower domain containing 1	10.54093	10.14826	9.723799	10.29028	9.310251	9.554696
17532828	Cacna1f	calcium channel, voltage-dependent, alpha 1F subunit	6.889838	5.794965	5.794965	5.258787	6.030928	5.557092

17313106	Cacna1i	calcium channel, voltage-dependent, alpha 1I subunit	3.438859	3.896881	4.091699	4.064703	3.729055	3.654895
17521327	Cacna2d2	calcium channel, voltage-dependent, alpha 2/delta subunit 2	4.000028	5.338965	5.338965	5.338965	5.431836	6.040341
17462244	Cacna2d4	calcium channel, voltage-dependent, alpha 2/delta subunit 4	9.835988	8.895835	9.428969	8.724425	9.069418	8.880618
17434890	Cacna2d1	calcium channel, voltage-dependent, alpha2/delta subunit 1	3.904966	3.942252	3.977826	4.272439	3.785018	2.990154
17304297	Cacna2d3	calcium channel, voltage-dependent, alpha2/delta subunit 3	4.776951	4.975391	4.776951	4.776951	4.776951	4.841822
17268691	Cacnb1	calcium channel, voltage-dependent, beta 1 subunit	11.76688	10.60527	10.66876	11.29365	11.39983	11.24366
17367163	Cacnb2	calcium channel, voltage-dependent, beta 2 subunit	4.779203	4.350828	4.968946	4.155465	3.62741	5.426192
17314636	Cacnb3	calcium channel, voltage-dependent, beta 3 subunit	11.15115	11.53871	7.639615	9.980209	8.677804	11.44147
17385281	Cacnb4	calcium channel, voltage-dependent, beta 4 subunit	4.742409	5.610191	4.20927	4.20927	3.445898	4.573219
17242822	Cbarp	calcium channel, voltage-dependent, beta subunit associated regulatory protein	15.37271	13.1993	13.1993	11.30665	14.06629	13.1993
17271143	Cacng1	calcium channel, voltage-dependent, gamma subunit 1	7.050913	6.461683	6.332653	6.461683	6.467284	5.595124
17318923	Cacng2	calcium channel, voltage-dependent, gamma subunit 2	5.068785	5.003994	5.068785	4.661615	6.530756	5.068785
17482793	Cacng3	calcium channel, voltage-dependent, gamma subunit 3	4.388415	4.533766	3.974436	5.46867	3.630857	3.729055
17271151	Cacng4	calcium channel, voltage-dependent, gamma subunit 4	17.65812	15.49445	13.66639	11.7562	15.42715	12.86304
17271158	Cacng5	calcium channel, voltage-dependent, gamma subunit 5	7.979454	8.725755	8.518622	8.903669	8.276495	8.601987
17473166	Cacng6	calcium channel, voltage-dependent, gamma subunit 6	6.996144	6.58818	5.881116	4.959346	6.58818	7.304219
17473155	Cacng7	calcium channel, voltage-dependent, gamma subunit 7	28.58151	24.93914	31.43261	21.75206	19.47809	22.82828
17473161	Cacng8	calcium channel, voltage-dependent, gamma subunit 8	8.297806	6.282157	7.213503	7.852707	6.421588	6.945603
17470235	Cacna1c	calcium channel, voltage-dependent, L type, alpha 1C subunit	6.03716	6.508071	6.349179	6.08324	5.819963	6.629638
17304353	Cacna1d	calcium channel, voltage-dependent, L type, alpha 1D subunit	4.600179	3.968682	4.179905	3.565464	4.195608	4.017617
17217678	Cacna1s	calcium channel, voltage-dependent, L type, alpha 1S subunit	6.896383	7.796194	7.441327	7.983935	6.448976	7.650107
17382249	Cacna1b	calcium channel, voltage-dependent, N type, alpha 1B subunit	7.65934	7.65934	7.65934	8.355754	8.477565	8.202593
17503122	Cacna1a	calcium channel, voltage-dependent, P/Q type, alpha 1A subunit	5.780483	5.989062	5.994212	6.030928	6.030928	6.030928
17228293	Cacna1e	calcium channel, voltage-dependent, R type, alpha 1E subunit	2.79326	3.347375	2.665914	2.79326	2.856067	2.953407
17267885	Cacna1g	calcium channel, voltage-dependent, T type, alpha 1G subunit	2.754556	3.343317	4.142897	3.267393	3.37033	3.882834
17342249	Cacna1h	calcium channel, voltage-dependent, T type, alpha 1H subunit	6.992896	10.34241	7.861638	8.341692	8.687433	8.74204
17510542	Cherp	calcium homeostasis endoplasmic reticulum protein	54.96038	60.43071	76.21959	62.33152	61.61373	58.80716
17365471	Calhm1	calcium homeostasis modulator 1	7.981832	6.365086	7.502025	7.593053	6.160596	6.916825
17365461	Calhm2	calcium homeostasis modulator 2	5.039114	6.085138	9.219957	5.875656	5.093333	4.357649
17287726	Caml	calcium modulating ligand	32.76106	28.251	28.04983	30.22771	33.29445	33.38805
17327995	Carhsp1	calcium regulated heat stable protein 1	87.59035	94.25714	90.78424	83.18549	84.29061	95.01873
17463377	Cracr2a	calcium release activated channel regulator 2A	9.63577	11.53367	12.21718	12.48566	12.66961	10.58904
17484925	Cracr2b	calcium release activated channel regulator 2B	7.307764	7.702677	6.703851	7.257537	6.896622	7.07044
17213349	Carf	calcium response factor	10.12591	10.56873	10.4982	7.820495	12.69721	11.99998
17469705	Camk1	calcium/calmodulin-dependent protein kinase I	31.52949	36.02783	36.88392	34.05937	27.69435	36.80909
17231252	Camk1g	calcium/calmodulin-dependent protein kinase I gamma	5.829208	5.979356	6.540496	6.724887	5.832078	5.345334
17381357	Camk1d	calcium/calmodulin-dependent protein kinase ID	55.01374	45.81982	51.27594	46.01142	55.51286	47.46493
17351001	Camk2a	calcium/calmodulin-dependent protein kinase II alpha	6.045367	9.47818	5.764079	9.468986	4.874351	8.326961
17303916	Camk2g	calcium/calmodulin-dependent protein kinase II gamma	22.37631	24.36114	20.95525	23.29454	24.84132	26.41948
17420514	Camk2n1	calcium/calmodulin-dependent protein kinase II inhibitor 1	80.9826	89.00226	95.55702	88.33235	110.5979	100.9208
17329163	Camk2n2	calcium/calmodulin-dependent protein kinase II inhibitor 2	13.70566	12.85021	12.04431	11.18366	13.19043	14.44271
17260261	Camk2b	calcium/calmodulin-dependent protein kinase II, beta	6.992896	6.405983	6.992896	8.368796	7.998891	7.024132
17402438	Camk2d	calcium/calmodulin-dependent protein kinase II, delta	15.30944	15.088	12.5923	17.63073	17.07188	18.47804
17349341	Camk4	calcium/calmodulin-dependent protein kinase IV	4.672161	4.088382	3.992825	3.992825	3.853502	3.653223
17252539	Camkk1	calcium/calmodulin-dependent protein kinase kinase 1, alpha	6.064884	6.519675	5.695372	6.441336	6.300953	5.832442
17452456	Camkk2	calcium/calmodulin-dependent protein kinase kinase 2, beta	15.82342	19.65657	18.39842	17.81919	18.93824	18.7208
17540332	Cask	calcium/calmodulin-dependent serine protein kinase (MAGUK family)	38.46862	38.6193	38.81278	32.36315	38.81278	30.996
17348591	Cabyr	calcium-binding tyrosine-(Y)-phosphorylation regulated (fibrousheathin 2)	5.078914	5.964371	4.274661	5.074269	4.283262	4.67647
17330188	Casr	calcium-sensing receptor	5.266593	5.266593	5.359803	5.818834	5.266593	4.886969
17228743	Cacybp	calcyclin binding protein	131.2415	90.36862	78.14437	89.93682	77.07174	80.97698
17497584	Caly	calcyon neuron-specific vesicular protein	11.50086	11.50086	12.82387	9.488237	8.299302	13.15701
17310250	Capsl	calcyphosine-like	3.195895	3.263862	2.997397	4.365691	3.248582	4.508539
17237313	Caps2	calcyphosphine 2	3.422095	3.645129	3.506787	3.648011	3.072387	3.335031
17457184	Cald1	caldesmon 1	185.0832	161.8373	171.8445	155.5247	154.3348	148.7535
17413507	Ccin	calicin	3.353436	3.617568	2.953734	2.831232	3.933654	2.957934
17502921	Clgn	calmegin	6.606792	7.228368	7.935109	9.730069	7.935109	8.352048
17283725	Clmn	calmin	141.5924	162.2742	183.5081	159.1518	156.1415	174.8616
17277924	Calm1	calmodulin 1	99.49416	95.56497	98.03534	87.86886	81.69637	93.13167
17347938	Calm2	calmodulin 2	268.424	314.1196	384.0313	321.334	371.3202	354.8757
17486967	Calm3	calmodulin 3	127.9645	138.7285	146.8551	123.8251	109.3319	119.676
17284906	Calm4	calmodulin 4	9.088675	9.65188	7.567412	8.086862	10.29855	8.74695
17284910	Calm5	calmodulin 5	6.506402	4.778143	6.127973	9.026145	5.447522	7.98864
17433359	Camta1	calmodulin binding transcription activator 1	4.313234	4.356924	5.2036	4.861225	5.372226	4.910194
17265340	Camta2	calmodulin binding transcription activator 2	35.53626	50.1473	53.19873	41.59332	39.62173	44.93071
17382778	Camsap1	calmodulin regulated spectrin-associated protein 1	27.61557	34.80759	27.49638	25.27484	25.45046	27.29661
17227483	Camsap2	calmodulin regulated spectrin-associated protein family, member 2	56.55058	47.74507	44.79171	47.88228	42.73452	41.26652
17498673	Camsap3	calmodulin regulated spectrin-associated protein family, member 3	21.67214	22.2805	20.03249	24.42319	18.97516	18.6018
17290238	Calml3	calmodulin-like 3	6.171709	7.069509	6.438167	6.717573	6.171709	7.036802
17518238	Calml4	calmodulin-like 4	13.354	23.30391	17.18955	15.6717	12.08839	13.91906
17340148	Camkmt	calmodulin-lysine N-methyltransferase	9.516361	7.562745	10.40222	8.51685	7.291977	6.393607
17443047	Caln1	calneuron 1	6.261421	5.572096	7.799005	7.194328	6.614949	8.332215
17262336	Canx	calnexin	864.271	716.3645	724.9964	670.8889	726.0223	665.1519
17361890	Capn1	calpain 1	38.86232	40.48288	46.09218	42.8404	44.56163	38.02088
17215853	Capn10	calpain 10	23.05056	17.14695	22.89943	20.72859	17.96018	19.24391
17345238	Capn11	calpain 11	4.081983	3.751038	4.20927	7.532193	5.066994	3.20368
17476011	Capn12	calpain 12	10.49653	11.68001	10.65412	10.61638	10.97866	12.65943
17347114	Capn13	calpain 13	4.453193	4.028548	3.798632	3.598087	3.825054	3.860881
17342544	Capn15	calpain 15	17.57131	13.64594	16.83206	15.56895	18.46601	15.18884
17230611	Capn2	calpain 2	101.9968	99.65774	95.93331	100.7615	101.6453	101.0538
17375045	Capn3	calpain 3	3.840116	3.370284	4.20927	4.155465	4.383824	5.285207
17493526	Capn5	calpain 5	13.51941	14.7229	19.14837	13.70158	12.49449	14.90526
17545106	Capn6	calpain 6	4.345433	6.812716	4.647678	6.812716	4.912475	6.396178
17298426	Capn7	calpain 7	85.15681	80.23779	68.16126	84.99759	83.50033	85.35004
17220394	Capn8	calpain 8	3.706661	6.208092	4.243457	5.245717	5.458218	5.458218

17506775	Capn9	calpain 9	6.227918	7.072205	6.632119	6.987082	5.601526	6.460698
17488957	Capns1	calpain, small subunit 1	495.8431	480.8156	557.8719	429.4064	379.409	398.8521
17503862	Capns2	calpain, small subunit 2	4.512854	11.15068	6.645326	5.345593	4.850387	6.232366
17294421	Cast	calpastatin	137.0948	129.7607	136.6489	123.1762	135.817	133.5161
17515402	Cnn1	calponin 1	5.759366	4.763163	6.534378	5.386134	5.386134	5.386134
17235077	Cnn2	calponin 2	26.7508	26.09099	30.05202	23.91162	23.78615	20.7069
17402155	Cnn3	calponin 3, acidic	373.5403	350.5897	315.8925	341.4735	322.4899	320.0492
17511183	Calr	calreticulin	1800.161	1274.08	1160.749	1291.615	1434.398	1161.988
17510526	Calr3	calreticulin 3	12.97669	11.50086	10.95115	10.94417	10.97174	11.85702
17416814	Calr4	calreticulin 4	2.656101	2.583828	2.52876	2.729695	3.314888	3.103422
17229851	Casq1	calsequestrin 1	2.494136	3.207368	3.838759	3.482902	4.061267	4.57804
17401041	Casq2	calsequestrin 2	8.088432	6.623115	5.746765	5.532801	5.682044	6.091764
17421828	Clstn1	calsyntenin 1	14.89845	12.59658	17.87498	14.89845	15.21413	12.02038
17529930	Clstn2	calsyntenin 2	3.200462	3.599658	3.475595	3.171884	3.263862	3.486864
17470711	Clstn3	calsyntenin 3	7.870689	9.646998	9.310251	9.359166	6.533065	9.800056
17456584	Calu	calumenin	87.50418	76.49269	81.85962	78.22783	78.61649	82.05162
17521510	Camkv	CaM kinase-like vesicle-associated	7.42093	9.999251	10.36042	8.559283	8.122422	11.33349
17468461	Cml1	camello-like 1	58.63702	52.18524	49.28887	59.02565	55.09807	56.80988
17468444	Cml2	camello-like 2	629.416	575.0565	629.1281	605.0833	617.1255	504.4684
17468417	Cml3	camello-like 3	10.2209	11.38845	13.15063	15.23228	12.67022	12.84674
17468432	Cml5	camello-like 5	5.769355	3.440814	3.392362	5.950331	4.579182	4.228541
17213676	Creb1	cAMP responsive element binding protein 1	40.52668	33.49445	41.68568	36.963	36.30685	38.31374
17413369	Creb3	cAMP responsive element binding protein 3	62.54922	51.96218	49.78606	62.463	52.94234	58.06392
17388332	Creb3l1	cAMP responsive element binding protein 3-like 1	7.185955	7.170382	9.971565	10.43877	11.92246	8.913796
17465906	Creb3l2	cAMP responsive element binding protein 3-like 2	71.59466	37.2072	58.0764	48.51974	47.87067	40.60907
17243183	Creb3l3	cAMP responsive element binding protein 3-like 3	482.3277	446.4578	420.3654	438.1436	342.9133	399.4442
17407246	Creb3l4	cAMP responsive element binding protein 3-like 4	4.032431	4.144822	4.681043	4.188315	4.681043	3.369233
17458677	Creb5	cAMP responsive element binding protein 5	4.238019	4.525697	5.38117	5.603662	5.219349	4.87354
17458682	Creb5	cAMP responsive element binding protein 5	6.561656	8.652518	7.81746	7.298854	7.590316	7.048469
17463709	Crebl2	cAMP responsive element binding protein-like 2	87.60492	79.20557	88.90052	81.33024	86.11365	96.06174
17352371	Crem	cAMP responsive element modulator	19.47431	15.83615	15.76398	15.28707	15.28707	14.65143
17519384	Arpp19	cAMP-regulated phosphoprotein 19	16.99184	21.30913	20.13062	20.85368	17.23238	18.12036
17291915	Cage1	cancer antigen 1	3.158632	2.704459	2.974712	4.059888	3.425038	3.158632
17472705	Casc1	cancer susceptibility candidate 1	4.136126	4.002912	3.73459	4.136126	4.573473	3.125203
17256177	Casc3	cancer susceptibility candidate 3	28.377	25.43001	28.86761	25.70042	26.43395	24.13191
17375327	Casc4	cancer susceptibility candidate 4	38.24661	28.23808	31.22371	19.86215	18.84082	19.27274
17374692	Casc5	cancer susceptibility candidate 5	3.474439	3.292013	3.837748	4.043936	3.606726	3.837748
17535175	Ctag2	cancer/testis antigen 2	3.250631	3.428149	3.817584	3.532379	3.043244	4.219816
17265917	Ovca2	candidate tumor suppressor in ovarian cancer 2	29.27404	31.62777	31.62777	32.36606	30.76035	31.62777
17412627	Cnr1	cannabinoid receptor 1 (brain)	5.000908	4.373717	4.598617	3.706712	3.965575	4.195754
17419959	Cnr2	cannabinoid receptor 2 (macrophage)	17.48747	17.48747	19.49537	16.11207	10.41175	15.91858
17247438	Cnrip1	cannabinoid receptor interacting protein 1	13.16951	17.2239	11.53943	12.30412	10.38536	12.98892
17446567	Cnpy1	canopy 1 homolog (zebrafish)	6.136517	7.241357	6.99338	6.270846	6.270846	7.737889
17238424	Cnpy2	canopy 2 homolog (zebrafish)	199.1608	223.1608	196.7282	201.1977	241.7312	215.5308
17345570	Cnpy3	canopy 3 homolog (zebrafish)	54.05814	41.31375	47.84745	48.58234	41.64814	38.05094
17443785	Cnpy4	canopy 4 homolog (zebrafish)	12.82361	12.75747	14.7025	11.98377	10.7716	12.82361
17335579	Cmtr1	cap methyltransferase 1	88.71647	77.7074	83.42801	84.18726	70.09356	78.8058
17505501	Cmtr2	cap methyltransferase 2	9.348403	10.33338	10.16607	12.95628	8.322402	10.16607
17429613	Cap1	CAP, adenylate cyclase-associated protein 1 (yeast)	176.9086	193.1771	186.062	157.8839	153.1116	148.0622
17287005	Cap2	CAP, adenylate cyclase-associated protein, 2 (yeast)	5.338965	6.251056	5.269441	5.513238	6.129333	4.990796
17452640	Clip1	CAP-GLY domain containing linker protein 1	53.04335	64.26049	75.3706	63.87945	59.61651	72.52116
17453383	Clip2	CAP-GLY domain containing linker protein 2	15.17684	19.49186	17.78489	15.59682	9.283057	13.596
17476317	Clip3	CAP-GLY domain containing linker protein 3	6.157138	6.566843	5.437713	5.191099	6.157138	4.977219
17339529	Clip4	CAP-GLY domain containing linker protein family, member 4	11.46878	9.487908	11.03336	10.55833	13.99918	9.844309
17475193	Cic	capicua homolog (Drosophila)	23.85732	25.22776	31.07839	24.81912	26.37374	19.15979
17408793	Capza1	capping protein (actin filament) muscle Z-line, alpha 1	82.67676	73.4291	79.72443	72.3359	65.77193	65.56573
17546205	4933400A11Rik	capping protein (actin filament) muscle Z-line, alpha 1 pseudogene	2.323307	2.523979	2.260842	2.789642	3.004157	2.447621
17456204	Capza2	capping protein (actin filament) muscle Z-line, alpha 2	78.22783	76.4275	86.33718	84.22753	84.22753	99.67225
17463967	Capza3	capping protein (actin filament) muscle Z-line, alpha 3	3.253494	3.375755	2.937523	3.400814	2.645646	3.622511
17420582	Capzb	capping protein (actin filament) muscle Z-line, beta	241.4934	256.087	274.1782	208.0563	199.1581	193.9889
17459656	Capg	capping protein (actin filament), gelsolin-like	11.77202	10.41665	9.748025	9.194557	12.25077	10.41665
17537188	5730416F02Rik	capping protein (actin filament), gelsolin-like pseudogene	4.886969	3.617793	4.42467	4.886969	6.551294	5.639941
17473018	Caprin2	caprin family member 2	10.12318	9.685993	14.72382	12.21558	9.059868	11.21688
17213894	Cps1	carbamoyl-phosphate synthetase 1	2507.441	2493.178	2758.771	2954.36	2654.271	3108.472
17435978	Cad	carbamoyl-phosphate synthetase 2, aspartate transcarbamylase, and dihydroorota	24.82772	13.10804	16.23786	12.71051	15.96466	15.96466
17468895	Chst13	carbohydrate (chondroitin 4) sulfotransferase 13	30.40486	29.73871	25.7416	40.51572	28.59062	29.32949
17241045	Chst3	carbohydrate (chondroitin 6/keratan) sulfotransferase 3	3.883991	3.883991	3.718704	3.679195	4.519678	4.081021
17512868	Chst4	carbohydrate (chondroitin 6/keratan) sulfotransferase 4	3.946654	3.839903	4.532164	4.20927	3.267778	4.350647
17373521	Chst1	carbohydrate (keratan sulfate Gal-6) sulfotransferase 1	8.936377	8.254209	10.66876	9.488237	6.209512	9.662389
17374678	Chst14	carbohydrate (N-acetylgalactosamine 4-0) sulfotransferase 14	16.26828	22.31605	20.59611	15.06731	16.29096	19.26006
17489699	Chst8	carbohydrate (N-acetylgalactosamine 4-0) sulfotransferase 8	13.39275	14.06912	14.08209	10.57826	14.53068	13.60542
17352872	Chst9	carbohydrate (N-acetylgalactosamine 4-0) sulfotransferase 9	2.450218	2.64264	2.515648	2.752953	2.546049	2.885617
17497076	Chst15	carbohydrate (N-acetylgalactosamine 4-sulfate 6-O) sulfotransferase 15	19.18571	24.38682	31.30324	28.44159	20.19435	20.92245
17513181	Chst5	carbohydrate (N-acetylglucosamine 6-O) sulfotransferase 5	13.82905	12.86813	10.89958	12.86813	12.04966	15.43764
17533535	Chst7	carbohydrate (N-acetylglucosamino) sulfotransferase 7	4.681043	5.728073	3.64422	5.273716	3.536862	3.372644
17499014	Carkd	carbohydrate kinase domain containing	56.85321	60.35243	56.73669	56.51688	57.39846	55.86334
17222511	Chst10	carbohydrate sulfotransferase 10	9.181692	8.314042	9.181692	8.969764	10.48228	11.07097
17235941	Chst11	carbohydrate sulfotransferase 11	7.647298	10.52516	12.37477	9.097562	6.529805	9.142635
17444100	Chst12	carbohydrate sulfotransferase 12	7.648782	6.778096	6.980547	6.971118	7.061575	7.822989
17529708	Chst2	carbohydrate sulfotransferase 2	18.26412	22.85202	21.73397	17.84131	17.33601	18.2109
17404180	Car1	carbonic anhydrase 1	22.03248	23.46649	16.83206	29.89392	21.97452	29.98523
17255097	Car10	carbonic anhydrase 10	5.488607	5.708295	5.532456	6.744914	4.927275	5.96247
17477968	Car11	carbonic anhydrase 11	8.1985	11.39398	11.91758	12.64452	13.33578	11.24366
17396143	Car13	carbonic anhydrase 13	10.77018	10.21835	11.81829	12.18657	7.341068	10.22097

17407956	Car14	carbonic anhydrase 14	112.4274	110.4431	101.7179	128.415	105.2751	104.039
17328780	Car15	carbonic anhydrase 15	9.692508	7.306143	8.116063	8.2644	7.275619	7.1482
17396162	Car2	carbonic anhydrase 2	42.89448	49.44868	48.63861	54.01132	29.05269	45.97125
17396152	Car3	carbonic anhydrase 3	2171.521	1573.324	1223.925	1766.796	1828.43	2554.774
17254508	Car4	carbonic anhydrase 4	4.933153	5.448768	4.888765	5.523145	6.089568	6.642379
17513655	Car5a	carbonic anhydrase 5a, mitochondrial	158.0547	161.5447	173.4445	188.6032	140.3582	174.7998
17545936	Car5b	carbonic anhydrase 5b, mitochondrial	13.74876	11.77349	12.1201	10.08962	12.33187	8.097295
17433265	Car6	carbonic anhydrase 6	3.497733	3.122821	3.318681	3.55207	2.824724	3.28882
17504454	Car7	carbonic anhydrase 7	17.04386	16.34016	15.74793	18.63148	14.31296	16.70792
17423121	Car8	carbonic anhydrase 8	201.8906	152.8285	128.7037	160.1034	189.6374	194.4776
17413352	Car9	carbonic anhydrase 9	4.50248	4.191248	5.049218	4.109292	4.516546	4.316554
17518780	Car12	carbonic anyhydrase 12	6.767627	5.995583	5.425552	5.613848	4.318349	6.45743
17327255	Cbr1	carbonyl reductase 1	73.84406	124.2378	144.3603	86.48632	76.9415	71.98877
17547965	Cbr1	carbonyl reductase 1	242.0699	393.9531	482.9232	287.2175	236.9666	224.2664
17273304	Cbr2	carbonyl reductase 2	11.10933	10.49005	10.91674	10.64313	12.74271	13.71669
17327264	Cbr3	carbonyl reductase 3	8.542984	19.29787	18.73819	12.67251	10.90078	9.22392
17501427	Cbr4	carbonyl reductase 4	45.04172	42.53062	36.67385	44.21549	49.83233	45.73764
17504495	Ces2b	carboxyesterase 2B	5.201256	3.589543	2.610344	4.408109	3.558428	3.450391
17383381	Cel	carboxyl ester lipase	5.12581	5.961395	5.63912	5.416234	5.893808	7.681883
17511643	Ces1a	carboxylesterase 1A	3.984421	5.879323	5.085572	5.203419	4.529432	6.013229
17511660	Ces1b	carboxylesterase 1B	41.83765	39.49861	36.92997	44.39176	42.68153	40.09193
17511677	Ces1c	carboxylesterase 1C	1812.682	1843.677	1663.908	1638.375	2204.155	1857.427
17511693	Ces1d	carboxylesterase 1D	67.97159	95.38497	118.0071	76.99111	110.9565	116.0474
17511714	Ces1e	carboxylesterase 1E	140.7548	109.9352	97.96334	90.55485	125.0005	142.8513
17511731	Ces1f	carboxylesterase 1F	498.0787	627.2427	578.4306	440.0489	363.4694	492.5034
17511748	Ces1g	carboxylesterase 1G	234.7465	285.5362	292.753	335.3305	417.618	513.233
17511765	Ces1h	carboxylesterase 1H	2.505624	2.521059	2.67402	3.475619	2.560472	2.953407
17504477	Ces2a	carboxylesterase 2A	218.4229	132.0911	89.56232	148.7597	159.8971	153.4984
17504512	Ces2c	carboxylesterase 2C	20.19463	19.43614	21.42941	38.10346	18.49496	25.07695
17504527	Ces2d-ps	carboxylesterase 2D, pseudogene	16.35081	12.38679	18.18617	18.49649	17.79007	20.51433
17504541	Ces2e	carboxylesterase 2E	182.606	171.7183	127.5775	177.8923	167.649	178.1576
17504558	Ces2f	carboxylesterase 2F	2.815498	3.092985	3.366665	3.584073	2.877767	3.870366
17504572	Ces2g	carboxylesterase 2G	88.53463	74.15489	67.72516	89.17392	114.6601	127.7607
17504587	Ces2h	carboxylesterase 2H	3.478728	4.163538	6.348651	4.576009	4.52206	3.896638
17504601	Ces3a	carboxylesterase 3A	1342.159	729.2603	422.5974	1154.386	1079.046	894.7371
17504616	Ces3b	carboxylesterase 3B	216.8119	100.2571	87.34541	303.1966	233.0749	155.1264
17504632	Ces4a	carboxylesterase 4A	9.455475	8.817624	6.726425	8.137073	8.306611	7.810149
17511788	Ces5a	carboxylesterase 5A	3.327641	3.605027	3.514209	3.40178	4.161288	2.82085
17310816	Cmbl	carboxymethylenebutenolidase-like (Pseudomonas)	234.7937	250.6326	235.8425	279.2474	243.5375	272.4523
17456918	Cpa1	carboxypeptidase A1, pancreatic	6.149461	5.245681	4.90999	5.230033	6.335111	5.245681
17456868	Cpa2	carboxypeptidase A2, pancreatic	3.844591	3.54506	3.130059	3.363399	3.877536	3.54506
17404337	Cpa3	carboxypeptidase A3, mast cell	3.462322	3.244351	3.009972	3.025346	3.346307	3.245273
17456886	Cpa4	carboxypeptidase A4	7.154644	6.243132	6.915866	5.644047	6.35411	6.753334
17456900	Cpa5	carboxypeptidase A5	3.343664	3.397327	3.057431	3.207368	3.560229	3.336487
17221269	Cpa6	carboxypeptidase A6	2.886738	2.991523	3.044468	3.436429	2.991523	2.781417
17404350	Cpb1	carboxypeptidase B1 (tissue)	2.454706	2.49815	2.763794	2.881041	2.814503	2.862013
17301995	Cpb2	carboxypeptidase B2 (plasma)	861.7885	901.2478	1030.915	816.8288	1014.42	852.0829
17266157	Cpd	carboxypeptidase D	66.10145	66.33324	61.31083	62.65814	88.0945	69.13862
17509617	Cpe	carboxypeptidase E	5.745769	5.130218	6.030886	5.886785	5.945673	5.965074
17237547	Cpm	carboxypeptidase M	8.255983	8.511834	7.861311	5.016635	6.992896	7.613501
17364988	Cpn1	carboxypeptidase N, polypeptide 1	656.3903	668.211	760.271	534.8016	626.895	503.9651
17329627	Cpn2	carboxypeptidase N, polypeptide 2	214.7479	216.534	247.1323	215.3784	298.8049	215.7864
17310882	Cpq	carboxypeptidase Q	173.6237	182.763	157.6947	147.3926	147.9412	153.4591
17391623	Cpxm1	carboxypeptidase X 1 (M14 family)	7.151074	6.817062	6.403497	7.676667	6.776545	8.644185
17497058	Cpxm2	carboxypeptidase X 2 (M14 family)	5.979356	5.979356	5.787659	5.836081	5.544087	5.979356
17447331	Cpz	carboxypeptidase Z	4.371081	6.381166	5.816213	5.927443	4.093146	5.963172
17466938	Cpvl	carboxypeptidase, vitellogenic-like	4.99218	4.655513	4.238107	5.548278	4.308752	5.726366
17487952	Ceacam1	carcinoembryonic antigen-related cell adhesion molecule 1	792.1117	719.1505	714.9408	644.1889	660.4566	712.3685
17475081	Ceacam10	carcinoembryonic antigen-related cell adhesion molecule 10	3.560451	3.92483	4.60222	3.873023	3.382219	3.729055
17474315	Ceacam11	carcinoembryonic antigen-related cell adhesion molecule 11	11.88854	8.008822	10.2561	9.378453	8.569019	12.67549
17474329	Ceacam12	carcinoembryonic antigen-related cell adhesion molecule 12	3.756476	4.674947	4.20927	4.34468	4.073475	5.431761
17474322	Ceacam13	carcinoembryonic antigen-related cell adhesion molecule 13	2.340165	2.82355	2.659804	2.606909	2.5612	2.881919
17474292	Ceacam14	carcinoembryonic antigen-related cell adhesion molecule 14	3.982157	3.243002	4.041414	2.640864	3.488701	3.663722
17486948	Ceacam15	carcinoembryonic antigen-related cell adhesion molecule 15	3.339703	5.310913	3.473139	2.77287	3.370821	3.233889
17487471	Ceacam16	carcinoembryonic antigen-related cell adhesion molecule 16	7.98936	8.240832	8.298036	12.01263	8.093984	10.99176
17477169	Ceacam18	carcinoembryonic antigen-related cell adhesion molecule 18	2.997147	3.393491	3.289207	3.229991	2.522475	3.977026
17487479	Ceacam19	carcinoembryonic antigen-related cell adhesion molecule 19	8.083219	6.571624	5.97919	5.811538	6.030928	6.571624
17487964	Ceacam2	carcinoembryonic antigen-related cell adhesion molecule 2	15.13923	14.02103	12.75163	9.615288	14.86142	12.48955
17474694	Ceacam20	carcinoembryonic antigen-related cell adhesion molecule 20	9.204696	8.646582	7.372838	7.210404	7.291573	7.684226
17474248	Ceacam3	carcinoembryonic antigen-related cell adhesion molecule 3	7.217854	4.895207	5.969334	5.591247	5.058116	8.067323
17474278	Ceacam5	carcinoembryonic antigen-related cell adhesion molecule 5	2.741414	2.796922	2.316794	2.276851	3.308621	2.344906
17474130	Ceacam9	carcinoembryonic antigen-related cell adhesion molecule 9	3.336464	3.992134	4.04416	3.108072	4.402582	3.97529
17486944	Ceacam-ps1	carcinoembryonic antigen-related cell adhesion molecule pseudogene 1	12.13019	7.074117	6.992896	8.422917	8.838981	6.771944
17376649	Crls1	cardiolipin synthase 1	149.5631	161.5739	150.022	150.0584	167.9165	128.5967
17294959	Cmya5	cardiomyopathy associated 5	8.995915	9.656297	9.717936	10.54064	10.16029	11.99765
17483420	Ctf1	cardiotrophin 1	44.10621	42.2105	41.9198	39.62777	41.88785	40.79018
17496757	Ctf2	cardiotrophin 2	3.116939	3.490056	3.82638	3.403479	3.630932	3.449985
17356194	Clcf1	cardiotrophin-like cytokine factor 1	9.608891	11.57588	9.825767	11.36275	8.559224	10.06399
17383633	Crat	carnitine acetyltransferase	102.9279	143.2747	173.9333	106.0595	75.333	92.3961
17530291	Cdv3	carnitine deficiency-associated gene expressed in ventricle 3	62.84343	65.75096	61.00688	61.25306	64.02263	74.92421
17421390	Gm13238	carnitine deficiency-associated gene expressed in ventricle 3 pseudogene	4.63436	4.094451	3.480754	4.178456	5.964082	5.806988
17445543	Crot	carnitine O-octanoyltransferase	1925.891	1991.597	1515.565	1562.619	1404.051	1537.755
17355915	Cpt1a	carnitine palmitoyltransferase 1a, liver	610.7169	760.055	820.944	727.4327	696.7996	729.2906
17490481	Cpt1c	carnitine palmitoyltransferase 1c	5.393755	7.814535	6.935981	7.232829	8.025215	7.846613

17427961	Cpt2	carnitine palmitoyltransferase 2	356.6512	379.7852	331.8276	344.7078	268.8803	332.4307
17355810	Cndp1	carnosine dipeptidase 1 (metallopeptidase M20 family)	8.968831	9.313091	8.746283	12.68077	8.184362	9.232747
17358032	Carnmt1	carnosine N-methyltransferase 1	73.43368	59.84007	56.2636	66.01903	67.52594	64.76399
17361141	Carns1	carnosine synthase 1	13.9519	17.48687	15.84494	17.48687	17.09485	17.33998
17295496	Cartpt	CART prepropeptide	10.83164	10.85818	11.25427	11.88805	12.01063	11.4314
17364828	Crtac1	cartilage acidic protein 1	6.992896	5.821738	5.38815	6.632303	5.484994	6.694888
17531877	Crtap	cartilage associated protein	24.98153	29.69422	36.52722	29.88874	22.1401	31.87095
17509879	Cilp2	cartilage intermediate layer protein 2	15.58871	10.03021	14.21527	12.4291	13.89111	17.17526
17518512	Cilp	cartilage intermediate layer protein, nucleotide pyrophosphohydrolase	5.70098	6.989019	5.209374	6.185756	10.11567	5.110661
17501919	Comp	cartilage oligomeric matrix protein	5.324367	6.029089	6.611786	6.171709	6.992896	5.880709
17380119	Cass4	Cas scaffolding protein family member 4	3.954348	3.801556	3.659585	3.751636	4.950485	4.585376
17455855	Casd1	CAS1 domain containing 1	82.38673	68.91327	67.42445	70.77461	75.91905	72.80169
17438631	Csn1s1	casein alpha s1	2.133127	2.029395	1.767181	1.85648	2.10003	2.229855
17438649	Csn1s2a	casein alpha s2-like A	3.375404	2.959883	3.124488	2.922757	3.31606	3.729055
17438661	Csn1s2b	casein alpha s2-like B	2.633753	2.395721	2.441588	2.65187	2.71782	3.203835
17449429	Csn2	casein beta	3.224153	3.009888	2.935447	3.476655	2.758798	2.766151
17438697	Csn3	casein kappa	3.576975	3.154847	3.426605	3.42117	3.898773	3.432239
17351111	Csnk1a1	casein kinase 1, alpha 1	152.2069	153.4622	153.5772	152.3103	154.7022	155.9749
17273420	Csnk1d	casein kinase 1, delta	62.80076	72.93705	63.58083	67.34458	62.73506	62.7168
17319207	Csnk1e	casein kinase 1, epsilon	12.1788	13.11413	18.12489	14.12413	10.85547	15.18232
17518644	Csnk1g1	casein kinase 1, gamma 1	20.03416	20.22854	18.96135	19.41258	23.80001	21.1615
17235379	Csnk1g2	casein kinase 1, gamma 2	99.26065	104.624	115.0933	98.98788	106.2184	100.7733
17350656	Csnk1g3	casein kinase 1, gamma 3	96.08704	106.3473	93.84448	103.1614	127.774	100.2515
17377675	Csnk2a1	casein kinase 2, alpha 1 polypeptide	159.896	155.9889	156.8824	164.8626	161.3142	169.3695
17331403	Csnka2ip	casein kinase 2, alpha prime interacting protein	3.423804	4.128592	3.194588	3.895531	3.291967	3.470564
17512009	Csnk2a2	casein kinase 2, alpha prime polypeptide	72.52015	66.88218	64.81654	69.29743	76.19001	63.02796
17344221	Csnk2b	casein kinase 2, beta polypeptide	262.0957	247.3414	229.298	206.9101	225.07	239.8419
17518526	Clpx	caseinolytic mitochondrial matrix peptidase chaperone subunit	321.1737	233.4274	193.9526	315.6561	230.573	252.0419
17338925	Clpp	caseinolytic mitochondrial matrix peptidase proteolytic subunit	67.67401	67.98761	58.77416	64.25781	63.35735	64.22798
17526130	Cbl	Casitas B-lineage lymphoma	25.1403	27.70453	29.11721	25.25435	26.34633	22.40253
17326208	Cblb	Casitas B-lineage lymphoma b	41.77911	46.85204	54.75353	37.64405	31.36862	41.18337
17487440	Cblc	Casitas B-lineage lymphoma c	20.30636	18.0727	24.15534	25.18373	17.86223	20.39551
17280515	Cbll1	Casitas B-lineage lymphoma-like 1	46.70386	40.95081	41.11207	33.85718	36.58778	37.22887
17334275	Caskin1	CASK interacting protein 1	11.70433	7.286318	9.310251	7.996674	7.824237	8.272825
17272019	Caskin2	CASK-interacting protein 2	16.93422	10.72436	13.86264	13.86264	9.812495	10.75481
17244476	Cradd	CASP2 and RIPK1 domain containing adaptor with death domain	30.56355	29.42499	30.84148	33.40125	31.64159	35.1609
17213192	Cflar	CASP8 and FADD-like apoptosis regulator	104.6719	78.10105	74.29998	66.36129	77.34092	62.83559
17514424	Casp1	caspase 1	12.0004	12.04306	11.15903	10.97212	12.03371	9.69009
17514447	Casp12	caspase 12	5.2423	5.036355	4.247842	3.973444	5.036355	4.936197
17242548	Casp14	caspase 14	5.778159	5.435716	5.398692	5.372747	5.707623	5.770115
17341484	Casp16-ps	caspase 16, apoptosis-related cysteine peptidase, pseudogene	23.93766	24.36114	21.1615	20.2982	21.1615	17.33397
17457892	Casp2	caspase 2	16.33087	16.41008	18.49649	16.33087	14.8574	15.90744
17501041	Casp3	caspase 3	21.02349	15.07682	22.11526	15.53952	19.46513	16.75593
17514435	Casp4	caspase 4, apoptosis-related cysteine peptidase	6.022865	6.287864	6.287864	5.415896	5.570512	5.300616
17402595	Casp6	caspase 6	25.53086	35.629	35.5301	30.06702	34.17075	43.9524
17360561	Casp7	caspase 7	57.93768	56.08916	61.75782	75.00475	62.8443	63.74057
17213213	Casp8	caspase 8	152.2227	125.4032	131.6469	123.1882	86.78898	88.01576
17412404	Casp8ap2	caspase 8 associated protein 2	16.99679	15.44941	12.28946	14.60621	14.02657	18.76666
17213189	Gm20257	caspase 8 pseudogene	3.878288	3.013876	4.945992	4.046992	5.821214	3.094444
17421120	Casp9	caspase 9	20.58155	23.67397	25.46564	26.26264	25.17553	26.26264
17427222	Caap1	caspase activity and apoptosis inhibitor 1	5.831997	5.139935	7.512328	5.386134	9.49758	6.259858
17427229	Caap1	caspase activity and apoptosis inhibitor 1	16.38189	18.47599	16.84875	13.90228	17.378	17.60142
17319063	Card10	caspase recruitment domain family, member 10	10.93181	13.91511	13.1407	14.31296	12.6468	10.29855
17454627	Card11	caspase recruitment domain family, member 11	8.317673	7.249744	8.05732	8.840758	6.660221	7.106405
17259026	Card14	caspase recruitment domain family, member 14	5.202554	5.06738	6.238893	5.031784	5.131	5.864182
17315705	Card6	caspase recruitment domain family, member 6	6.992896	5.990722	8.380405	8.688096	8.231983	10.39797
17382920	Card9	caspase recruitment domain family, member 9	8.10246	8.322979	7.628768	7.952453	7.126037	8.422859
17421735	Casz1	castor zinc finger 1	10.09158	11.94877	10.19097	8.772694	7.201912	7.938795
17462373	Cecr2	cat eye syndrome chromosome region, candidate 2	27.12705	27.02308	26.87662	32.65858	29.23065	31.66155
17470435	Cecr5	cat eye syndrome chromosome region, candidate 5	56.67341	47.44815	42.55126	52.55915	40.19182	51.20739
17470433	Cecr6	cat eye syndrome chromosome region, candidate 6	5.95904	5.617429	5.617429	5.617429	5.617429	5.617429
17388803	Cat	catalase	4632.066	4502.467	4005.01	4066.156	3393.645	3674.214
17328937	Comt	catechol-O-methyltransferase	648.8989	648.3999	645.4269	727.4781	615.2419	518.6976
17303979	Comtd1	catechol-O-methyltransferase domain containing 1	13.86177	16.27336	17.25497	11.49066	14.21094	13.21615
17349520	Ctnna1	catenin (cadherin associated protein), alpha 1	141.6081	147.0018	164.6148	139.2892	147.0018	143.1129
17467938	Ctnna2	catenin (cadherin associated protein), alpha 2	3.286701	2.914725	3.869052	3.457694	3.250654	3.219976
17233930	Ctnna3	catenin (cadherin associated protein), alpha 3	4.098795	4.929085	4.694918	4.495838	4.273387	4.31458
17425466	Ctnnal1	catenin (cadherin associated protein), alpha-like 1	10.29256	8.827898	10.03216	11.48079	10.35345	10.45514
17523257	Ctnnb1	catenin (cadherin associated protein), beta 1	204.5997	187.5511	191.1882	194.0709	179.7864	169.494
17387432	Ctnnd1	catenin (cadherin associated protein), delta 1	90.12279	91.69429	91.69429	94.65324	99.69091	95.87349
17310772	Ctnnd2	catenin (cadherin associated protein), delta 2	7.028564	8.148136	7.028564	6.868952	7.028564	6.206242
17421821	Ctnnbip1	catenin beta interacting protein 1	23.2805	22.62789	21.58145	24.07026	21.95397	25.15355
17378754	Ctnnbl1	catenin, beta like 1	45.11296	43.21889	36.83486	43.72783	29.86161	38.59307
17531482	Camp	cathelicidin antimicrobial peptide	3.714557	3.313119	3.331149	3.169642	4.599031	3.458508
17293454	Cts3	cathepsin 3	4.791509	4.10414	5.107085	5.500299	5.499536	4.526607
17293408	Cts6	cathepsin 6	2.406006	2.420844	1.985304	2.192733	2.458673	4.395295
17293434	Cts7	cathepsin 7	2.568222	2.865963	3.031622	2.968677	3.651831	2.432483
17293418	Cts8	cathepsin 8	2.585548	3.192862	2.67402	2.703578	2.871232	3.102282
17293428	Cts8-ps	cathepsin 8, pseudogene	4.198838	3.808862	4.268562	4.063154	4.424241	4.320385
17379560	Ctsa	cathepsin A	172.213	201.4224	228.3432	164.858	181.2328	157.9255
17301213	Ctsb	cathepsin B	1330.839	1416.24	1582.818	1172.962	1086.566	1076.453
17480018	Ctsc	cathepsin C	283.4656	277.9728	275.7792	201.4391	257.0259	193.09
17498099	Ctsd	cathepsin D	439.6343	569.1046	638.1315	479.92	502.9531	519.5072
17217035	Ctse	cathepsin E	9.643254	12.46309	14.31256	8.679067	8.185497	7.486493

17356288	Ctsf	cathepsin F	29.77274	29.49053	31.01813	31.85372	30.55571	30.88276
17306975	Ctsg	cathepsin G	7.972101	4.886529	4.801649	4.886529	4.620215	4.801649
17520233	Ctsh	cathepsin H	235.7918	241.1187	247.2283	219.3788	224.5838	223.6362
17293376	Ctsj	cathepsin J	5.868452	5.663799	5.964991	7.436532	5.338965	5.127551
17400365	Ctsk	cathepsin K	4.82283	4.367053	5.537712	5.993339	5.480775	5.865036
17293706	Ctsl	cathepsin L	447.3561	519.972	613.832	552.856	655.3719	608.5365
17293328	Ctsll3	cathepsin L-like 3	4.756334	4.446561	3.765862	3.758325	4.196335	4.16048
17293444	Ctsm	cathepsin M	1.989781	2.00233	2.19217	2.195105	2.019629	2.491751
17398430	Ctso	cathepsin O	117.8624	118.9161	143.9716	116.7946	113.1239	136.665
17293386	Ctsq	cathepsin Q	4.025143	2.581608	4.686465	3.874553	3.730606	3.869186
17293397	Ctsr	cathepsin R	3.461506	3.30445	3.276624	3.475716	4.285341	3.323838
17400375	Ctss	cathepsin S	179.4838	207.2732	232.3217	144.9478	196.8646	166.2108
17361605	Ctsw	cathepsin W	6.805589	6.514119	5.874394	7.544734	5.935049	5.374721
17395155	Ctsz	cathepsin Z	436.4462	471.3223	494.0455	422.0236	460.8297	405.9927
17356389	Catsper1	cation channel, sperm associated 1	3.829856	3.846243	3.495454	3.371428	3.836897	4.03218
17390561	Catsper2	cation channel, sperm associated 2	7.240905	10.63767	7.76906	7.698994	7.834656	6.616967
17287777	Catsper3	cation channel, sperm associated 3	3.262437	2.916746	3.262437	3.262437	3.780403	2.769681
17431216	Catsper4	cation channel, sperm associated 4	6.929878	6.543851	6.543851	7.115672	5.767316	6.217437
17277978	Catsperb	catsper channel auxiliary subunit beta	3.245701	2.78626	3.102863	2.685275	3.785516	2.901925
17338826	Catsperd	catsper channel auxiliary subunit delta	3.346447	3.136705	3.474704	3.109624	3.181352	3.649124
17488752	Catsperg1	catsper channel auxiliary subunit gamma 1	10.98254	13.75601	11.39485	11.39485	9.850248	10.23096
17488850	Catsperg2	catsper channel auxiliary subunit gamma 2	3.430075	5.160425	4.032851	3.57197	6.021697	4.595239
17354698	Cdx1	caudal type homeobox 1	13.524	11.23548	11.21168	11.96966	11.69241	10.64335
17455307	Cdx2	caudal type homeobox 2	7.140277	6.55134	6.55134	6.078014	7.739981	8.764914
17536954	Cdx4	caudal type homeobox 4	3.883991	3.883991	3.68831	3.756554	4.015724	3.37234
17456161	Cav1	caveolin 1, caveolae protein	12.03521	9.481925	10.18299	8.097968	10.93529	8.420524
17456152	Cav2	caveolin 2	16.79675	15.37101	15.31581	16.75442	15.23239	12.2772
17461489	Cav3	caveolin 3	4.292565	4.762008	3.310181	3.756841	4.704691	4.258632
17543664	Cited1	Cbp/p300-interacting transactivator with Glu/Asp-rich carboxy-terminal domain 1	18.81876	25.31324	20.93914	22.53866	17.69905	25.96345
17231784	Cited2	Cbp/p300-interacting transactivator, with Glu/Asp-rich carboxy-terminal domain, 2	91.78459	68.91136	88.03773	84.07121	94.44614	94.31269
17418035	Cited4	Cbp/p300-interacting transactivator, with Glu/Asp-rich carboxy-terminal domain, 4	10.34226	11.39461	16.46899	12.2171	11.16855	14.57961
17355463	Ctif	CBP80/20-dependent translation initiation factor	60.02202	64.24267	68.05834	57.916	63.01223	47.0685
17434087	LOC100043918	C-C motif chemokine 19-like	19.0289	19.85843	23.87916	18.02116	19.62866	19.42119
17412973	LOC100043921	C-C motif chemokine 19-like	16.63881	15.6603	22.7515	16.56631	15.89829	16.86406
17413084	LOC100043921	C-C motif chemokine 19-like	16.63881	15.6603	22.7515	16.56631	15.89829	16.86406
17434141	LOC100043921	C-C motif chemokine 19-like	18.60811	18.17571	26.30381	19.48484	20.36599	20.01001
17434105	LOC100041504	C-C motif chemokine 21c	15.01945	13.95219	17.6711	14.8325	13.61401	16.51426
17476728	Cebpa	CCAAT/enhancer binding protein (C/EBP), alpha	175.1164	208.9683	230.2728	238.3834	214.4281	276.5257
17379938	Cebpb	CCAAT/enhancer binding protein (C/EBP), beta	111.5642	125.8813	128.5557	118.3782	125.082	120.8799
17323297	Cebpd	CCAAT/enhancer binding protein (C/EBP), delta	9.168972	9.622155	7.011436	8.353727	10.1236	7.175603
17306472	Cebpe	CCAAT/enhancer binding protein (C/EBP), epsilon	27.80476	26.61966	24.42777	21.44248	23.67102	24.42777
17489707	Cebpg	CCAAT/enhancer binding protein (C/EBP), gamma	80.8295	73.10915	92.06175	71.59069	80.09443	69.53802
17339858	Cebpzos	CCAAT/enhancer binding protein (C/EBP), zeta, opposite strand	25.45205	31.79394	26.97779	37.10238	41.52016	33.01867
17347387	Cebpz	CCAAT/enhancer binding protein zeta	52.61091	45.14487	37.89433	43.60676	49.9551	43.08489
17504861	Ctcf	CCCTC-binding factor	60.00081	47.52023	48.43742	48.04019	53.63527	45.53803
17395063	Ctcfl	CCCTC-binding factor (zinc finger protein)-like	7.144534	7.431122	7.783991	8.246489	7.256128	7.621844
17380211	Ctcflos	CCCTC-binding factor (zinc finger protein)-like, opposite strand	23.74118	39.40837	46.22816	44.65872	30.52205	32.50748
17512032	Cnot1	CCR4-NOT transcription complex, subunit 1	189.3406	189.1766	179.4938	179.4938	170.0789	175.6829
17531900	Cnot10	CCR4-NOT transcription complex, subunit 10	29.995	29.88563	29.24058	28.31628	29.61569	29.4101
17212123	Cnot11	CCR4-NOT transcription complex, subunit 11	31.67033	29.67755	30.8984	36.5746	36.24098	31.72944
17245095	Cnot2	CCR4-NOT transcription complex, subunit 2	85.08955	85.07716	81.22264	86.84976	87.37024	87.05891
17473193	Cnot3	CCR4-NOT transcription complex, subunit 3	40.24172	45.15895	40.67386	42.30306	39.35623	39.96707
17465788	Cnot4	CCR4-NOT transcription complex, subunit 4	61.85464	81.42162	65.60437	62.6716	58.95491	56.20202
17262277	Cnot6	CCR4-NOT transcription complex, subunit 6	72.23519	73.63399	72.23519	72.13312	73.01849	72.8133
17449899	Cnot6l	CCR4-NOT transcription complex, subunit 6-like	136.3216	146.116	122.1652	139.8455	126.6901	127.0163
17508868	Cnot7	CCR4-NOT transcription complex, subunit 7	22.22867	23.68333	21.38771	21.37126	22.03599	23.96405
17249950	Cnot8	CCR4-NOT transcription complex, subunit 8	48.71385	40.94683	36.30912	43.57866	36.61417	44.07199
17454919	Ccz1	CCZ1 vacuolar protein trafficking and biogenesis associated	62.9773	71.45683	68.157	71.83575	71.73822	74.10659
17408470	Cd101	CD101 antigen	6.739072	7.093462	8.303215	6.91117	7.935109	6.155645
17519738	Cd109	CD109 antigen	2.67402	3.427413	2.692731	2.420978	3.138054	3.376293
17353747	Cd14	CD14 antigen	14.57496	19.10767	29.57139	21.01037	15.75983	17.69684
17484940	Cd151	CD151 antigen	93.06134	89.61572	92.49862	98.68302	72.89056	72.93048
17408123	Cd160	CD160 antigen	3.282784	3.834398	4.20927	4.592787	4.20927	4.20927
17462843	Cd163	CD163 antigen	38.32623	47.69017	56.75675	44.54804	42.07729	35.36107
17484513	Cd163l1	CD163 molecule-like 1	5.414207	5.974343	4.369596	4.224205	5.774956	4.102149
17232892	Cd164	CD164 antigen	484.7644	475.0317	447.837	410.9617	441.0016	436.1408
17419543	Cd164l2	CD164 sialomucin-like 2	14.30205	13.6226	15.27627	12.91763	9.591191	13.47441
17487759	Cd177	CD177 antigen	6.171709	7.690514	7.334761	8.101112	7.334761	7.334761
17289527	Cd180	CD180 antigen	6.487893	7.425304	10.19507	6.568573	5.354493	6.568573
17496193	Cd19	CD19 antigen	6.903414	7.250096	6.939877	7.931644	7.377848	7.154545
17406482	Cd1d1	CD1d1 antigen	76.78007	79.24841	52.96106	77.67401	65.73865	82.21616
17398723	Cd1d2	CD1d2 antigen	2.923345	3.773858	3.290644	3.703322	2.885977	2.958693
17408497	Cd2	CD2 antigen	6.791875	8.5632	7.642793	8.51685	8.108191	9.152336
17496605	Cd2bp2	CD2 antigen (cytoplasmic tail) binding protein 2	40.25399	42.43139	44.16005	38.64768	40.53763	42.34207
17330625	Cd200	CD200 antigen	5.186136	5.530462	5.596132	5.439409	7.520143	5.439409
17325861	Cd200r1	CD200 receptor 1	4.70267	4.594284	4.383095	4.240575	4.378844	5.266301
17325884	Cd200r2	Cd200 receptor 2	3.854918	3.993379	5.366792	3.834796	4.789516	3.854918
17325892	Cd200r3	CD200 receptor 3	3.347862	4.188257	3.746205	3.263862	3.768473	3.697653
17325874	Cd200r4	CD200 receptor 4	2.406006	4.33178	3.40657	4.038949	3.498169	2.929917
17468222	Cd207	CD207 antigen	11.97613	9.9503	13.20397	17.62975	8.895465	14.171
17507161	Cd209a	CD209a antigen	4.79038	6.474371	6.474371	7.024327	6.672882	5.948393
17507194	Cd209b	CD209b antigen	4.20927	4.411319	4.20927	4.379785	3.581391	3.359881
17507209	Cd209c	CD209c antigen	3.885607	4.111885	4.025199	3.557787	5.174072	4.681952
17507184	Cd209d	CD209d antigen	3.555987	2.450184	3.555987	2.865188	4.491726	2.936016

17507175	Cd209e	CD209e antigen	2.595442	2.197251	2.541218	3.585315	3.129907	2.801928
17507221	Cd209f	CD209f antigen	3.310892	3.476679	3.617242	2.765921	3.47774	3.838599
17498750	Cd209g	CD209g antigen	7.681138	3.881462	5.570474	6.022865	4.892053	5.155527
17489320	Cd22	CD22 antigen	7.225813	11.50884	8.727449	8.676661	7.100497	8.436415
17352330	Cd226	CD226 antigen	6.068374	4.20927	3.844324	4.560747	3.823225	4.832233
17219382	Cd244	CD244 natural killer cell receptor 2B4	5.134736	3.706455	4.20927	3.645609	6.086108	4.130167
17219011	Cd247	CD247 antigen	7.148894	6.337835	7.214353	7.844438	7.610335	8.783464
17356369	Cd248	CD248 antigen, endosialin	11.82419	13.44064	13.5911	14.42301	10.31127	13.31813
17233022	Cd24a	CD24a antigen	19.47188	27.29604	18.17798	21.06623	18.50214	20.46391
17471073	Cd27	CD27 antigen	4.681043	3.515037	4.799087	4.93182	4.163769	3.339703
17358544	Cd274	CD274 antigen	16.76453	15.01716	15.01716	13.39423	15.01716	13.98376
17527719	Cd276	CD276 antigen	32.37145	32.13448	31.49564	32.97498	26.80221	32.13448
17213462	Cd28	CD28 antigen	4.047273	5.007845	3.960136	4.634295	4.528584	6.491402
17345038	Cd2ap	CD2-associated protein	134.6136	134.6593	146.3085	128.1012	108.7808	132.7649
17516691	Cd3d	CD3 antigen, delta polypeptide	7.870689	5.821738	5.700071	6.935789	5.09118	8.405712
17526464	Cd3e	CD3 antigen, epsilon polypeptide	10.29855	13.46703	10.26798	8.51685	10.85088	10.24601
17526456	Cd3g	CD3 antigen, gamma polypeptide	8.151356	6.308732	5.935378	5.281618	6.033228	6.333048
17271710	Cd300lb	CD300 antigen like family member B	6.477827	6.517732	7.860004	6.916442	4.830659	5.56106
17271776	Cd300lf	CD300 antigen like family member F	4.739977	5.337152	5.337485	5.386134	6.588545	5.673189
17256870	Cd300lg	CD300 antigen like family member G	36.02783	39.85752	40.00754	44.25719	23.92356	29.96113
17271763	Cd300lh	CD300 antigen like family member H	20.39933	43.99904	26.43395	29.48358	29.85747	24.12221
17271724	Cd300ld	CD300 molecule-like family member d	36.75478	36.36781	61.29298	32.29435	25.18705	30.41413
17258107	Cd300a	CD300A antigen	11.06944	10.25873	12.31786	8.70781	8.415098	6.639203
17271719	Cd300c	CD300C antigen	17.09864	15.84867	15.6333	17.09864	16.4136	17.64638
17271769	Cd300e	CD300e antigen	16.14494	22.64703	22.22806	22.1702	20.62826	14.24082
17385564	Cd302	CD302 antigen	232.2331	221.7223	223.7199	181.2026	256.9671	240.2845
17336199	Cd320	CD320 antigen	9.290395	10.10061	11.6963	9.992738	6.118551	11.88179
17490149	Cd33	CD33 antigen	11.08095	11.9304	11.06123	12.35189	9.835715	9.986644
17221014	Cd34	CD34 antigen	8.888069	9.89754	13.0829	8.707871	8.534283	10.80555
17445715	Cd36	CD36 antigen	632.3458	660.9421	708.4342	581.3326	554.5563	616.7534
17490702	Cd37	CD37 antigen	14.80036	16.42931	16.81737	17.80561	12.69721	19.13802
17437213	Cd38	CD38 antigen	44.22162	46.56745	64.19371	45.3217	39.76921	32.52461
17487218	Cd3eap	CD3E antigen, epsilon polypeptide associated protein	13.14626	12.88303	13.14626	11.80225	15.60861	10.18299
17470960	Cd4	CD4 antigen	5.431083	5.33804	7.30594	5.37953	4.932811	6.083198
17379659	Cd40	CD40 antigen	7.732045	8.939536	6.992799	12.20314	8.818235	7.372531
17535048	Cd40lg	CD40 ligand	3.14849	3.549707	3.03465	3.485776	3.263862	3.776841
17388733	Cd44	CD44 antigen	11.8878	16.17832	25.2512	14.09323	12.38807	10.52961
17231287	Cd46	CD46 antigen, complement regulatory protein	5.139329	5.749833	5.673465	5.546048	5.02262	6.992556
17326166	Cd47	CD47 antigen (Rh-related antigen, integrin-associated signal transducer)	206.0471	186.2053	190.2708	191.4322	152.9218	182.6275
17219397	Cd48	CD48 antigen	9.829445	13.76268	11.56746	9.829445	9.829445	9.718004
17362753	Cd5	CD5 antigen	9.362605	9.915393	8.283727	8.732774	7.824833	9.860631
17398734	Cd5l	CD5 antigen-like	150.8541	220.7424	246.725	164.0748	154.8707	162.6402
17431174	Cd52	CD52 antigen	17.29904	19.70842	23.70172	19.2956	19.30456	17.59837
17408960	Cd53	CD53 antigen	37.29447	38.38152	51.44646	36.97684	31.58987	36.97684
17226618	Cd55	CD55 molecule, decay accelerating factor for complement	17.68777	23.54012	22.29673	22.94297	20.76296	13.07193
17226622	Cd55	CD55 molecule, decay accelerating factor for complement	27.52613	34.26776	33.2101	28.24258	17.1699	22.10606
17226605	Cd55b	CD55 molecule, decay accelerating factor for complement B	4.879421	4.091018	3.366058	5.14193	4.890595	4.284569
17373825	Cd59a	CD59a antigen	200.6379	234.8165	181.1612	171.6671	133.4356	146.6365
17373817	Cd59b	CD59b antigen	21.66148	21.40847	12.0739	22.4277	11.53695	18.80286
17362765	Cd6	CD6 antigen	7.073381	9.348987	8.321768	10.36013	8.497686	9.280548
17238594	Cd63	CD63 antigen	22.44543	43.96123	51.10456	25.33131	17.52837	19.68139
17264835	Cd68	CD68 antigen	45.06108	69.65234	118.1847	57.61011	61.17923	50.20538
17471502	Cd69	CD69 antigen	4.462277	3.666059	3.073069	3.542137	3.418563	3.381398
17273440	Cd7	CD7 antigen	6.900304	7.050571	5.243572	7.689128	7.655412	6.317834
17346516	Cd70	CD70 antigen	2.67402	3.198333	4.142725	2.943842	3.542432	2.911898
17424608	Cd72	CD72 antigen	13.31758	16.03742	17.37463	14.55305	13.61797	11.81797
17350982	Cd74	CD74 antigen (invariant polypeptide of major histocompatibility complex, class II a	156.9009	155.2328	149.9689	96.96216	121.7114	108.1478
17475127	Cd79a	CD79A antigen (immunoglobulin-associated alpha)	6.516422	5.351747	5.226554	5.09065	5.197796	4.348537
17270837	Cd79b	CD79B antigen	6.449959	6.192921	6.674177	6.990473	7.187002	7.642158
17459482	Cd8a	CD8 antigen, alpha chain	4.596068	4.37393	5.074761	4.493937	6.370029	4.16553
17459474	Cd8b1	CD8 antigen, beta chain 1	8.138766	7.674219	8.042979	9.508053	6.102202	6.804598
17325608	Cd80	CD80 antigen	7.488309	9.245876	6.999782	6.885923	6.629684	6.91208
17485297	Cd81	CD81 antigen	1047.322	1229.069	1279.274	913.5374	938.869	940.9733
17388454	Cd82	CD82 antigen	40.33918	41.13373	43.66968	40.89181	39.86194	32.38065
17286905	Cd83	CD83 antigen	13.59326	12.63514	15.06616	14.34057	13.36224	15.16097
17219418	Cd84	CD84 antigen	12.80877	17.89519	22.96126	16.75384	14.11963	13.23054
17330203	Cd86	CD86 antigen	9.460392	9.623355	13.20388	7.87118	11.13161	8.29131
17471127	Cd9	CD9 antigen	59.18419	55.52325	71.92643	50.64683	50.0407	34.32434
17392572	Cd93	CD93 antigen	25.44253	21.9421	35.22774	24.96768	19.62866	23.29454
17330751	Cd96	CD96 antigen	3.655275	3.263862	5.023699	3.300764	2.973455	3.128996
17542180	Cd99l2	CD99 antigen-like 2	29.04202	32.97521	37.38558	25.90592	33.47286	31.71932
17249282	Clk4	CDC like kinase 4	73.7796	79.89703	73.13398	74.64739	69.7219	74.71883
17409668	Cdc14a	CDC14 cell division cycle 14A	7.357777	8.679067	9.411013	7.299613	7.850094	7.542224
17293675	Cdc14b	CDC14 cell division cycle 14B	62.90008	70.5674	63.47559	57.95053	54.86256	72.96132
17499353	Cdc16	CDC16 cell division cycle 16	36.44098	33.94836	32.03484	34.82159	33.13445	33.24833
17353488	Cdc23	CDC23 cell division cycle 23	28.7502	26.13317	23.50882	26.13317	25.80949	28.76116
17407116	Cks1b	CDC28 protein kinase 1b	26.06587	25.62713	26.78568	21.07309	33.39754	30.12
17503424	Cks1brt	CDC28 protein kinase 1b, retrogene	5.818027	4.679226	6.02512	5.553049	7.367014	6.857297
17287330	Cks2	CDC28 protein kinase regulatory subunit 2	17.47221	20.07489	22.30105	26.60896	20.37263	24.36114
17547744	Cks2	CDC28 protein kinase regulatory subunit 2	45.60247	51.10031	61.20001	68.6839	54.31919	62.49202
17548955	Cks2	CDC28 protein kinase regulatory subunit 2	45.60247	51.10031	61.20001	68.6839	54.31919	62.49202
17220059	Cdc42bpa	CDC42 binding protein kinase alpha	49.62792	49.62792	49.62792	48.15454	46.72815	49.62792
17284065	Cdc42bpb	CDC42 binding protein kinase beta	108.8947	89.73818	108.8947	108.8947	101.9685	108.8947
17356754	Cdc42bpg	CDC42 binding protein kinase gamma (DMPK-like)	31.81753	30.203	37.02275	31.27591	29.1051	35.82165

17312774	Cdc42ep1	CDC42 effector protein (Rho GTPase binding) 1	20.91245	25.42913	31.62273	24.53042	25.95967	23.29454
17361850	Cdc42ep2	CDC42 effector protein (Rho GTPase binding) 2	8.013375	12.06344	13.07329	7.920162	10.50759	8.630774
17347435	Cdc42ep3	CDC42 effector protein (Rho GTPase binding) 3	16.01354	14.49748	13.76965	14.33828	14.66312	16.39564
17271622	Cdc42ep4	CDC42 effector protein (Rho GTPase binding) 4	24.88975	24.58728	23.87055	27.22972	18.52036	23.87055
17485706	Cdc42ep5	CDC42 effector protein (Rho GTPase binding) 5	20.15897	11.52504	17.61106	9.193792	9.115046	10.72644
17543290	Arhgef9	CDC42 guanine nucleotide exchange factor (GEF) 9	6.58398	6.979821	6.987227	6.131032	6.779975	6.950227
17400281	Cdc42se1	CDC42 small effector 1	30.10164	28.71176	28.75359	28.71176	22.40642	25.45293
17262836	Cdc42se2	CDC42 small effector 2	17.69562	11.22349	12.42712	12.35189	10.88976	11.87528
17223353	Clk1	CDC-like kinase 1	51.07375	73.38992	61.38014	58.65735	59.7638	65.56618
17399285	Clk2	CDC-like kinase 2	26.0386	36.33908	38.76385	43.16081	36.13162	29.99583
17527590	Clk3	CDC-like kinase 3	54.51382	50.84239	49.59319	45.99771	44.0027	41.5766
17241789	Cisd1	CDGSH iron sulfur domain 1	72.06815	81.68957	88.33725	87.67235	100.0613	113.0079
17410529	Cisd2	CDGSH iron sulfur domain 2	29.99437	28.03855	26.0074	31.71339	32.29547	35.44819
17255853	Cisd3	CDGSH iron sulfur domain 3	56.06311	87.16095	87.96087	91.32703	93.63656	103.651
17548228	Cisd3b	CDGSH iron sulfur domain 3B	70.18155	83.81578	120.8413	90.00792	89.2933	95.39488
17262193	Cdk2ap1	CDK2 (cyclin-dependent kinase 2)-associated protein 1	19.8864	35.1865	27.042	42.62358	52.5362	32.04262
17452808	Cdk2ap1	CDK2 (cyclin-dependent kinase 2)-associated protein 1	6.703339	8.311795	7.921919	8.579003	8.919173	7.169189
17366081	Cacul1	CDK2 associated, cullin domain 1	105.1912	101.6242	88.01942	91.43914	92.03942	99.63564
17548127	Cacul1	CDK2 associated, cullin domain 1	79.12107	74.82923	65.46083	78.86427	65.97466	68.97444
17356099	Cdk2ap2	CDK2-associated protein 2	74.46962	61.81178	69.73737	64.80126	65.95819	66.41697
17348435	Cables1	CDK5 and Abl enzyme substrate 1	12.1739	15.42512	16.62612	14.97786	16.73017	18.29985
17395601	Cables2	CDK5 and Abl enzyme substrate 2	39.47753	26.67211	38.0251	41.63803	38.24502	29.69175
17393110	Cdk5rap1	CDK5 regulatory subunit associated protein 1	24.2518	26.82209	25.47694	24.41389	27.72201	21.21525
17291534	Cdkal1	CDK5 regulatory subunit associated protein 1-like 1	46.63464	41.78317	36.68911	33.8077	38.81978	37.56403
17426447	Cdk5rap2	CDK5 regulatory subunit associated protein 2	15.43464	13.53901	13.14589	12.57006	16.32284	14.72566
17268380	Cdk5rap3	CDK5 regulatory subunit associated protein 3	61.5608	51.32074	53.32499	56.05495	65.01407	56.59058
17369789	Ciz1	CDKN1A interacting zinc finger protein 1	25.55954	23.62283	30.31647	25.92424	25.44182	30.99299
17509282	Cdkn2aip	CDKN2A interacting protein	23.42602	32.0655	29.82872	32.55642	26.31895	35.64308
17249375	Cdkn2aipnl	CDKN2A interacting protein N-terminal like	55.05379	55.30431	57.46812	52.74966	52.74966	53.56468
17286052	9330162012Rik	cDNA RIKEN 9330162012 gene	14.09792	16.93106	13.59864	19.27301	23.17793	20.35089
17450950	AB010352	cDNA sequence AB010352	5.249645	3.620227	4.20927	4.313085	4.352065	3.179214
17515238	AB124611	cDNA sequence AB124611	20.68252	30.82694	25.35643	21.66493	21.66493	16.65773
17290199	AF067061	cDNA sequence AF067061	2.18339	3.191114	2.396601	2.894311	2.959842	2.57273
17296545	AF067063	cDNA sequence AF067063	3.280919	3.263862	3.729055	3.183668	3.068556	3.561364
17271733	AF251705	cDNA sequence AF251705	2.847528	5.978775	4.72682	4.759764	5.203672	3.623239
17507665	AF366264	cDNA sequence AF366264	2.948477	2.438274	2.690119	2.353308	2.960273	2.314964
17529625	AF529169	cDNA sequence AF529169	4.561853	4.888223	4.20927	3.502294	4.113254	4.980187
17285668	AK157302	cDNA sequence AK157302 [Source:MGI Symbol;Acc:MGI:3574096]	61.78009	71.16125	65.04427	84.07471	69.25278	65.89102
17527308	AY074887	cDNA sequence AY074887	6.910547	6.468315	5.617429	6.628581	5.655443	5.168731
17299627	AY358078	cDNA sequence AY358078	15.74684	30.16158	32.55664	27.84507	21.78827	23.56674
17469249	AY512915	cDNA sequence AY512915	3.604677	3.836684	4.060845	3.36984	4.587824	3.687492
17509025	AY512931	cDNA sequence AY512931	2.74398	3.041156	2.98626	3.703322	3.042991	3.091506
17394916	AY702102	cDNA sequence AY702102	2.742212	2.456867	2.797969	2.640589	2.076408	2.692283
17338517	AY702103	cDNA sequence AY702103	3.459827	2.67402	2.67402	2.560881	2.812825	2.438054
17507933	AY761184	cDNA sequence AY761184	4.807211	4.352065	3.670687	4.704072	4.352065	3.725205
17507894	AY761185	cDNA sequence AY761185	2.618717	2.406006	2.406006	2.406006	2.287022	2.852269
17333630	BC002059	cDNA sequence BC002059	7.175105	10.01548	11.95084	11.4417	6.662021	11.39098
17227872	BC003331	cDNA sequence BC003331	89.63374	75.47359	76.34491	76.34491	72.15063	71.23479
17334599	BC003965	cDNA sequence BC003965	23.34594	27.64066	28.09127	26.07599	26.43835	28.05119
17335493	BC004004	cDNA sequence BC004004	228.6647	220.8985	201.6081	200.4571	221.4811	213.787
17309374	BC005512	cDNA sequence BC005512	4.01461	4.012969	3.287521	2.76853	4.32479	3.789953
17285998	BC005537	cDNA sequence BC005537	354.7896	377.9601	418.6788	361.5923	378.3821	360.6886
17439861	BC005561	cDNA sequence BC005561	31.96497	39.10849	28.48163	40.21523	28.89644	33.60374
17383733	BC005624	cDNA sequence BC005624	41.62793	43.48392	50.7206	42.00299	34.33314	44.09521
17271539	BC006965	cDNA sequence BC006965	4.09641	4.185355	4.184572	4.062929	4.362484	4.392341
17388798	BC016548	cDNA sequence BC016548	2.865149	2.659804	2.495312	2.526745	3.982957	2.884278
17496867	BC017158	cDNA sequence BC017158	17.62865	15.03528	18.88436	16.33597	12.66768	14.55568
17273498	BC017643	cDNA sequence BC017643	15.86032	14.26878	16.45792	13.93673	14.16099	15.43582
17258644	BC018473	cDNA sequence BC018473	3.535122	3.571599	3.286336	3.347212	2.75897	3.263613
17239070	BC020402	cDNA sequence BC020402	9.525864	7.826406	13.22825	8.645084	4.124416	9.760263
17361090	BC021614	cDNA sequence BC021614	49.06866	73.72285	102.3608	72.14612	78.08264	71.75712
17240309	BC021785	cDNA sequence BC021785	8.598589	6.014855	5.305909	6.605098	6.454431	7.169189
17506904	BC021891	cDNA sequence BC021891	11.75791	11.37213	11.55472	13.33532	12.28197	15.14133
17279417	BC022687	cDNA sequence BC022687	6.180699	10.95828	8.265832	8.265832	5.506059	10.29392
17539682	BC022960	cDNA sequence BC022960	3.355552	2.769451	2.835907	2.654702	3.209614	3.167446
17350932	BC023105	cDNA sequence BC023105	25.54254	24.10215	19.49537	22.24316	25.66642	21.9067
17548055	BC023105	cDNA sequence BC023105	56.00835	30.06765	43.4303	34.66721	38.72759	40.21021
17484667	BC024386	cDNA sequence BC023486	244.6559	497.5611	457.6053	493.2824	496.6962	527.6948
17337373	BC023719	cDNA sequence BC023719 [Source:MGI Symbol;Acc:MGI:3574836]	3.476053	3.883991	3.779538	4.20927	3.149276	4.524286
17542167	BC023829	cDNA sequence BC023829	38.35386	35.56608	35.71603	28.01019	33.42695	28.50889
17318326	BC024139	cDNA sequence BC024139	8.951193	10.17425	11.0904	9.996479	10.17425	10.77309
17475523	BC024978	cDNA sequence BC024978	23.51517	19.74547	18.45462	18.90112	18.84317	14.8138
17312223	BC025446	cDNA sequence BC025446	169.2157	157.6302	136.5599	198.3425	200.2946	163.9611
17218465	BC026585	cDNA sequence BC026585	49.14423	47.54989	47.36764	47.84247	42.84782	47.91515
17347077	BC027072	cDNA sequence BC027072	4.336978	5.877041	5.433192	5.549008	5.148706	5.455911
17407947	BC028528	cDNA sequence BC028528	6.235477	6.86919	5.576926	6.943774	7.952729	6.280024
17334200	BC028777	cDNA sequence BC028777	8.740222	9.873626	8.509415	11.97264	10.92105	10.19627
17382574	BC029214	cDNA sequence BC029214	11.24366	9.797475	7.056242	13.12149	10.25205	8.899905
17393353	BC029722	cDNA sequence BC029722	19.28985	20.22685	18.57628	17.32316	14.11943	14.01529
17482503	BC030336	cDNA sequence BC030336	39.41028	39.18148	39.57973	36.02259	35.31551	43.06787
17253555	BC030499	cDNA sequence BC030499	4.881552	4.414194	4.165703	6.25045	4.882703	5.84596
17501317	BC030500	cDNA sequence BC030500	4.392493	4.910977	4.392493	4.282253	3.482902	4.449675
17256910	BC030867	cDNA sequence BC030867	6.55661	6.937424	7.754319	7.776063	6.834094	7.854449
17509682	BC030870	cDNA sequence BC030870	3.703322	3.703322	3.528023	4.035535	3.831077	3.834398

17351837	BC031181	cDNA sequence BC031181	76.9191	64.54171	62.53361	61.14362	56.83036	73.1426
17330321	BC031361	cDNA sequence BC031361	2.769758	3.883991	3.339703	3.339703	3.216073	2.774081
17228362	BC034090	cDNA sequence BC034090	2.827055	3.54506	3.54506	3.229677	3.54506	3.456088
17548985	BC034090	cDNA sequence BC034090	4.1775	5.6556	4.074943	5.190703	3.729055	4.247253
17471473	BC035044	cDNA sequence BC035044	3.573753	3.263862	3.242845	3.263862	3.263862	3.722727
17224724	BC035947	cDNA sequence BC035947	8.5915	9.984706	11.17893	10.12725	12.51937	9.912507
17315677	BC037032	cDNA Sequence BC037032	4.278574	4.280858	3.835461	4.730819	4.095955	3.421147
17454283	BC037034	cDNA sequence BC037034	26.01461	27.53911	29.63212	26.70319	27.70549	27.70549
17359606	BC037704	cDNA sequence BC037704	7.18237	7.688062	5.472993	7.499165	5.721328	5.960817
17377170	BC039771	cDNA sequence BC039771	14.21163	12.441	12.97174	17.65898	17.97949	14.8873
17433537	BC039966	cDNA sequence BC039966	7.588528	5.8602	7.098037	6.047002	5.675471	4.556418
17529803	BC043934	cDNA sequence BC043934	2.770814	2.6283	2.392419	3.404517	2.951647	2.514846
17548767	BC043934	cDNA sequence BC043934 [Source:MGI Symbol;Acc:MGI:2679715]	3.051334	3.811186	4.161028	3.889973	4.735281	3.497563
17478910	BC046251	cDNA sequence BC046251	4.86409	6.12186	5.499308	5.332973	5.238813	5.579864
17379713	BC046401	cDNA sequence BC046401	4.01461	4.233967	3.608052	4.058875	4.01461	2.832488
17237751	BC048403	cDNA sequence BC048403	20.52912	16.93235	16.35341	15.31624	15.34961	16.11341
17322418	BC048502	cDNA sequence BC048502	2.3059	2.1492	2.401124	1.946632	2.765499	2.381796
17288354	BC048507	cDNA sequence BC048507	22.54741	16.07838	16.01387	16.75477	19.89522	21.46434
17471394	BC048546	cDNA sequence BC048546	4.589796	4.833204	5.617429	4.833204	5.391363	4.86753
17521716	BC048562	cDNA sequence BC048562	3.435428	2.801229	3.203969	2.999475	2.804551	3.013437
17373996	BC048594	cDNA sequence BC048594	2.393646	3.034944	2.556961	2.988269	2.732081	2.327192
17316428	BC048602	cDNA sequence BC048602	7.744167	10.7443	7.314453	7.814535	9.681429	8.389472
17356616	BC048609	cDNA sequence BC048609	7.08894	7.873854	7.414349	7.446074	8.462536	7.972156
17506329	BC048644	cDNA sequence BC048644	3.061715	3.742286	3.556948	4.486965	3.648542	3.094572
17460775	BC048671	cDNA sequence BC048671	4.250198	3.703322	3.703322	3.274989	4.766598	3.666161
17492803	BC048679	cDNA sequence BC048679	9.107784	9.485212	7.860167	8.441504	10.50162	10.18067
17516752	BC049352	cDNA sequence BC049352	4.165299	4.072402	3.274149	3.60178	3.622737	5.443558
17540460	BC049702	cDNA sequence BC049702	3.536372	4.432283	4.623258	3.750804	3.231312	3.74335
17463837	BC049715	cDNA sequence BC049715	4.081983	4.667209	4.20927	3.749349	3.482661	5.037682
17475070	BC049730	cDNA sequence BC049730	2.274123	2.603135	2.208734	2.533058	2.921319	2.442265
17262471	BC049762	cDNA sequence BC049762	8.424027	10.66218	8.125407	9.372604	9.089557	9.239149
17528388	BC050972	cDNA sequence BC050972 [Source:MGI Symbol;Acc:MGI:3039596]	7.424223	4.854794	7.083979	7.165215	8.15322	6.283159
17494917	BC051019	cDNA sequence BC051019	5.011769	5.530385	5.486933	5.515455	5.897649	6.266761
17438706	BC051076	cDNA sequence BC051076	2.418462	2.306524	2.488317	2.952117	2.207265	2.542962
17451862	BC051077	cDNA sequence BC051077	3.768264	4.198052	3.542677	4.265308	3.543954	4.428843
17336536	BC051142	cDNA sequence BC051142	3.416857	3.310364	3.899394	4.006632	4.332981	3.86546
17343668	BC051226	cDNA sequence BC051226	13.62392	17.63488	16.10269	12.14643	16.04242	16.04242
17343762	BC051537	cDNA sequence BC051537	4.260374	4.666432	4.804246	6.270715	5.851311	5.147136
17380963	BC051628	cDNA sequence BC051628	5.261521	5.3493	7.467938	5.45958	7.02457	5.900511
17293318	BC051665	cDNA sequence BC051665	4.053533	3.103121	2.847232	4.009799	2.454876	3.823516
17374406	BC052040	cDNA sequence BC052040	15.74083	18.64491	13.38226	17.04847	15.37058	17.04847
17288030	BC052688	cDNA sequence BC052688	2.621205	2.239691	3.009972	2.304206	3.263862	2.262692
17248839	BC053393	cDNA sequence BC053393	3.462322	2.588776	2.79295	3.009972	3.112082	3.009972
17229128	BC055324	cDNA sequence BC055324	4.801649	4.801649	4.622746	4.801649	5.289605	4.801649
17223300	BC055402	cDNA sequence BC055402	6.559974	5.532916	5.532916	6.017315	5.532916	6.570622
17536247	BC061195	cDNA sequence BC061195	6.11465	5.162608	5.579593	5.230939	6.194638	5.640488
17299145	BC061237	cDNA sequence BC061237	2.685071	2.451424	3.152421	2.450897	2.584527	2.971662
17463478	BC064078	cDNA sequence BC064078	15.74236	16.95948	15.9016	12.88866	12.74068	13.64433
17537942	BC065397	cDNA sequence BC065397	8.087815	7.337456	7.337456	7.305586	6.132307	7.650426
17528746	BC065403	cDNA sequence BC065403	4.431576	4.431576	4.591864	4.801649	4.431576	4.433973
17290016	BC067074	cDNA sequence BC067074	3.339703	3.339703	3.098672	3.098887	2.763985	3.641745
17273812	BC068281	cDNA sequence BC068281	13.45938	13.45938	12.53708	14.21212	15.38231	13.45938
17421215	BC080695	cDNA sequence BC080695	11.51769	8.139048	8.116738	6.932905	8.949518	10.65339
17449862	BC080696	cDNA sequence BC080696	4.13624	3.536053	3.536053	3.437477	3.803137	4.106303
17455643	BC080696	cDNA sequence BC080696	4.878981	3.735186	4.276736	3.721386	4.629223	4.20927
17488370	BC089491	cDNA sequence BC089491	3.803823	2.947312	4.064957	3.798132	3.874338	4.08705
17238191	BC089597	cDNA sequence BC089597	60.66237	149.4853	141.8606	118.517	163.6115	137.0359
17230014	BC094916	cDNA sequence BC094916	3.462322	2.537557	2.839329	3.157494	2.977951	3.787485
17272780	BC100451	cDNA sequence BC100451	4.940373	5.704221	4.940373	5.823514	9.835443	5.56106
17330168	BC100530	cDNA sequence BC100530	2.145286	1.834784	1.881926	2.503784	2.596341	1.991341
17547678	BC106175	cDNA sequence BC106175	4.420072	4.301113	3.700422	5.008366	3.444059	4.384766
17329395	BC106179	cDNA sequence BC106179	5.166331	7.30822	5.562371	6.499866	6.030928	6.443569
17408054	BC107364	cDNA sequence BC107364	5.57824	4.682925	5.009199	5.331236	4.479849	4.687114
17330162	BC117090	cDNA sequence BC1179090	4.780296	4.994326	5.205945	6.953021	5.8905	4.014193
17290205	BC147527	cDNA sequence BC147527	4.821928	3.883991	3.801661	3.514574	3.760671	3.853288
17337963	BE949265	cDNA sequence BE949265	3.042548	2.945148	3.201238	3.465492	2.799502	3.229252
17430237	CK137956	cDNA sequence CK137956	9.367603	8.623001	7.266698	12.78154	11.67297	11.14558
17314242	CN725425	cDNA sequence CN725425	2.676968	2.740027	2.625842	2.761094	2.882259	3.162598
17449649	U90926	cDNA sequence U90926	3.067961	3.21507	3.173665	2.963989	3.251961	2.936586
17330654	BC016579	cDNA sequence, BC016579	3.456088	3.240441	3.806513	3.545134	3.294387	3.832989
17376563	Cds2	CDP-diacylglycerol synthase (phosphatidate cytidylyltransferase) 2	102.134	124.9338	124.1939	116.7485	110.7628	119.2992
17439646	Cds1	CDP-diacylglycerol synthase 1	3.904425	3.89437	3.89437	4.177153	3.909055	4.621048
17483220	Cdipt	CDP-diacylglycerol--inositol 3-phosphatidyltransferase (phosphatidylinositol syntha	94.51491	104.0765	124.8559	106.2898	91.3131	86.48991
17516960	Cadm1	cell adhesion molecule 1	39.97067	41.48765	48.82676	38.98318	38.964	37.98979
17331447	Cadm2	cell adhesion molecule 2	3.511458	3.274989	3.781346	3.710285	4.135094	3.274989
17229988	Cadm3	cell adhesion molecule 3	17.49257	23.75007	17.64002	27.05719	10.84825	19.53555
17474983	Cadm4	cell adhesion molecule 4	11.7282	11.23758	11.80119	11.17041	11.71618	10.49238
17461205	Chl1	cell adhesion molecule with homology to L1CAM	2.96477	2.624586	2.829388	3.450654	3.131405	3.135727
17515921	Cdon	cell adhesion molecule-related/down-regulated by oncogenes	5.811618	8.833346	6.171709	6.943774	6.171709	5.589542
17515950	Cdon	cell adhesion molecule-related/down-regulated by oncogenes	2.932112	2.558414	2.642823	2.454978	2.696784	3.060654
17308209	Ccar2	cell cycle activator and apoptosis regulator 2	32.51785	32.51785	32.50793	31.51158	31.24038	33.7494
17388854	Caprin1	cell cycle associated protein 1	71.56886	70.12563	62.39896	75.83069	58.31035	67.33245
17497895	Cend1	cell cycle exit and neuronal differentiation 1	11.97937	9.219446	11.81346	12.41541	9.244146	13.18632
17519259	Ccpg1	cell cycle progression 1	77.42084	86.12081	86.72223	80.02673	73.50447	72.24321

17528775	Ccpg1os	cell cycle progression 1, opposite strand	80.69067	89.41717	98.20877	98.24486	90.50151	88.27971
17327815	Cdip1	cell death inducing Trp53 target 1	30.52417	44.92697	36.44629	41.88814	42.03182	38.75445
17469754	Cidec	cell death-inducing DFFA-like effector c	35.7143	79.67968	127.7607	62.91927	40.46773	49.4264
17351457	Cidea	cell death-inducing DNA fragmentation factor, alpha subunit-like effector A	19.36366	16.43273	15.51079	8.51685	14.83836	17.69684
17306867	Cideb	cell death-inducing DNA fragmentation factor, alpha subunit-like effector B	522.53	571.6705	699.6017	579.9682	526.2119	581.3568
17381376	Cdc123	cell division cycle 123	114.1621	111.0365	109.2849	109.4881	110.2955	113.4537
17429177	Cdc20	cell division cycle 20	15.83637	15.82364	19.07604	15.89807	13.96748	16.87798
17289986	Cdc20b	cell division cycle 20B	4.352065	4.577024	4.352065	4.446315	4.352065	4.730524
17522235	Cdc25a	cell division cycle 25A	5.831997	5.718552	4.33184	5.533913	5.591479	4.47172
17376446	Cdc25b	cell division cycle 25B	6.456579	4.800784	5.641896	5.239757	6.112193	5.291035
17353520	Cdc25c	cell division cycle 25C	4.591514	3.902314	3.703322	3.491411	4.337218	3.703322
17426166	Cdc26	cell division cycle 26	49.90284	39.81803	42.92719	41.33036	35.38951	47.37552
17270648	Cdc27	cell division cycle 27	86.68978	75.43123	82.65499	78.69009	74.79189	65.3996
17234910	Cdc34	cell division cycle 34	60.31312	48.7206	42.72386	40.1303	38.63777	48.45656
17255254	Cdc34-ps	cell division cycle 34 homolog, pseudogene (S. cerevisiae)	3.152333	2.863918	3.046431	2.965078	2.646967	2.953407
17524648	Cdc37	cell division cycle 37	72.84611	77.85188	99.4018	89.52136	90.90325	83.41067
17358486	Cdc37l1	cell division cycle 37-like 1	42.85525	41.93114	35.21895	44.28327	31.54851	40.62906
17240379	Cdc40	cell division cycle 40	71.68304	71.21701	71.43306	67.62431	71.43306	72.47442
17431672	Cdc42	cell division cycle 42	386.9303	434.9302	427.6984	366.6272	343.4485	379.4142
17328978	Cdc45	cell division cycle 45	6.992896	8.855907	6.992896	7.030757	6.870714	7.041535
17345152	Cdc5l	cell division cycle 5-like (S. pombe)	20.8583	19.62866	19.46756	19.97578	29.7779	21.1549
17256225	Cdc6	cell division cycle 6	6.356577	6.221446	4.835517	5.461094	7.935109	6.914285
17439978	Cdc7	cell division cycle 7 (S. cerevisiae)	3.478222	3.84563	4.053758	4.726689	3.476053	3.977219
17227729	Cdc73	cell division cycle 73, Paf1/RNA polymerase II complex component	48.81017	47.45276	44.68287	46.0794	46.0794	44.9195
17241483	Ccar1	cell division cycle and apoptosis regulator 1	67.25595	81.66862	74.20888	73.61256	78.97751	68.91566
17307969	Cdca2	cell division cycle associated 2	5.826583	6.219592	5.904439	5.686851	5.686851	6.214723
17462958	Cdca3	cell division cycle associated 3	8.154407	6.3128	7.708018	6.563112	7.231225	6.637362
17284243	Cdca4	cell division cycle associated 4	16.03631	17.77207	14.51538	16.8183	17.34839	19.27795
17356622	Cdca5	cell division cycle associated 5	7.56972	7.522593	6.977257	6.943774	6.886734	6.923541
17371941	Cdca7	cell division cycle associated 7	5.048273	5.897772	6.183827	5.278398	5.680154	5.721764
17279701	Cdca7l	cell division cycle associated 7 like	5.338965	5.153562	5.338965	5.217722	6.282244	5.715778
17429896	Cdca8	cell division cycle associated 8	4.498612	4.75429	4.498612	5.1481	4.038893	4.410891
17446767	Cgref1	cell growth regulator with EF hand domain 1	11.62155	11.50277	11.41564	8.759995	12.75791	7.988585
17299279	Cgrrf1	cell growth regulator with ring finger domain 1	54.02143	43.47126	36.83001	54.07163	50.94752	50.90128
17492947	Cemip	cell migration inducing protein, hyaluronan binding	4.603943	4.896836	5.464956	4.851665	4.494934	5.983833
17438113	Cwh43	cell wall biogenesis 43 C-terminal homolog (S. cerevisiae)	4.536878	4.63452	4.50857	4.801649	4.708149	5.387702
17468690	Cnbp	cellular nucleic acid binding protein	392.8351	360.3263	334.416	358.2915	334.0106	359.117
17219005	Creg1	cellular repressor of E1A-stimulated genes 1	1194.888	1245.656	1107.53	1000.621	830.4618	967.2332
17222568	Creg2	cellular repressor of E1A-stimulated genes 2	7.289147	7.750074	7.698994	7.698994	9.158428	7.892597
17517416	Crabp1	cellular retinoic acid binding protein I	4.809244	3.621733	4.809244	4.809244	4.364571	4.965882
17398895	Crabp2	cellular retinoic acid binding protein II	6.446294	8.303042	5.274119	7.901738	5.635877	7.003034
17415076	Cntln	centlein, centrosomal protein	6.436204	5.767276	6.538728	6.187086	8.32246	6.9105
17352593	Cetn1	centrin 1	2.942658	2.601241	2.830467	2.841869	2.616884	2.788521
17542239	Cetn2	centrin 2	7.313439	7.210653	8.110608	8.864873	7.935109	9.27084
17288908	Cetn3	centrin 3	24.0963	24.53552	19.95862	23.21025	30.0491	25.75552
17404896	Cetn4	centrin 4	7.13523	6.176331	7.511286	7.756523	6.771568	7.808362
17482160	Ccp110	centriolar coiled coil protein 110	6.725679	8.937678	7.56652	5.564993	8.320441	8.614219
17513986	Ccsap	centriole, cilia and spindle associated protein	2.669279	2.788695	2.898487	2.856879	3.125182	3.052603
17513989	Ccsap	centriole, cilia and spindle associated protein	9.107026	8.766676	9.459802	9.181055	7.366095	9.477851
17370145	Cntrl	centriolin	17.78452	18.36555	17.43519	17.36523	18.9402	19.57798
17264562	Cntrob	centrobin, centrosomal BRCA2 interacting protein	15.77557	17.69684	17.72029	19.00056	17.69684	19.09893
17251604	Cntrobos	centrobin, centrosomal BRCA2 interacting protein, opposite strand [Source:MGI Sy	9.865417	9.620154	10.1335	11.06192	11.38482	9.310251
17435825	Cenpa	centromere protein A	6.794371	6.863193	6.976532	6.501759	7.469698	7.286217
17391883	Cenpb	centromere protein B	12.59955	19.10357	23.40622	18.28235	20.9197	18.70355
17449155	Cenpc1	centromere protein C1	7.073724	6.397685	5.930032	6.565159	6.664146	6.565159
17402797	Cenpe	centromere protein E	5.321083	5.155885	6.237509	5.434548	5.09238	5.06264
17230918	Cenpf	centromere protein F	4.301411	6.180228	5.267287	4.851867	5.32101	5.028576
17295745	Cenph	centromere protein H	2.998249	3.036038	3.290325	3.290325	3.033998	3.857457
17537648	Cenpi	centromere protein I	4.757785	3.69335	3.258053	4.813946	3.832033	3.866987
17307046	Cenpj	centromere protein J	9.062757	10.28087	12.19223	11.47514	11.0954	13.75029
17289584	Cenpk	centromere protein K	2.987399	3.670203	4.194154	2.754098	3.62741	3.313188
17218682	Cenpl	centromere protein L	5.878712	6.333531	6.920278	7.005607	7.035437	7.478091
17319596	Cenpm	centromere protein M	28.17922	28.43331	43.16111	36.34462	43.92925	31.5166
17505913	Cenpn	centromere protein N	6.492437	6.35455	6.081385	6.512313	6.123175	6.425862
17279789	Cenpo	centromere protein O	11.21401	12.2624	12.2624	9.759519	12.66364	13.50873
17292477	Cenpp	centromere protein P	3.683992	3.572317	3.416123	3.546362	3.363842	3.642528
17344990	Cenpq	centromere protein Q	6.784394	5.449712	4.197354	4.347542	6.004483	5.730853
17512529	Cenpt	centromere protein T	6.16987	9.348468	10.9747	10.47799	8.685386	9.322972
17501025	Cenpu	centromere protein U	4.572712	4.410188	3.899367	3.482178	4.352065	3.213087
17264021	Cenpv	centromere protein V	55.97458	51.55141	58.76601	67.13951	54.59512	60.97771
17240022	Cenpw	centromere protein W	3.003178	2.582664	2.723685	2.953407	2.579283	3.867497
17422276	Cep104	centrosomal protein 104	21.83499	23.29454	17.41393	23.29454	23.29454	21.07791
17257760	Cep112	centrosomal protein 112	7.095085	6.357899	7.31638	6.794889	6.794889	6.794889
17271194	Cep112os1	centrosomal protein 112, opposite strand 1 [Source:MGI Symbol;Acc:MGI:3652256]	3.668804	3.813538	3.921702	3.479307	4.757785	3.632317
17257798	Cep112os2	centrosomal protein 112, opposite strand 2	3.503435	3.523966	3.087716	3.503435	3.104003	3.503435
17354386	Cep120	centrosomal protein 120	29.07687	33.38759	31.14028	33.22737	29.39258	37.52552
17523908	Cep126	centrosomal protein 126	2.406006	2.748663	2.72926	2.426186	2.537469	2.893088
17282980	Cep128	centrosomal protein 128	6.735663	7.413527	7.413527	7.698994	5.573679	7.344835
17272971	Cep131	centrosomal protein 131	19.91923	22.42522	20.45356	21.16898	19.27622	17.18371
17438361	Cep135	centrosomal protein 135	17.04102	17.93381	20.30144	17.07318	22.79539	21.25028
17390950	Cep152	centrosomal protein 152	6.287864	6.721345	7.439419	7.773477	6.546664	6.071151
17529449	Cep162	centrosomal protein 162	12.14045	13.75448	12.14045	12.96347	12.14045	11.64493
17526514	Cep164	centrosomal protein 164	16.09711	19.11867	15.23914	18.49649	16.5083	13.57095
17230195	Cep170	centrosomal protein 170	26.30545	24.5883	25.80824	18.47036	18.17433	21.05382

17279381	Cep170b	centrosomal protein 170B	47.65415	46.77222	51.24715	48.24809	48.99457	44.20722
17324733	Cep19	centrosomal protein 19	12.44807	11.40243	9.965278	11.40243	12.84424	8.563081
17351511	Cep192	centrosomal protein 192	8.995167	12.90564	12.00231	12.08118	15.09156	13.68108
17378468	Cep250	centrosomal protein 250	19.42873	20.99131	19.10807	18.2085	17.96927	18.03128
17236934	Cep290	centrosomal protein 290	11.13338	9.909004	9.497844	10.61344	9.762563	11.54071
17524170	Cep295	centrosomal protein 295	14.41791	14.99043	16.04909	13.43356	12.96356	16.27573
17228426	Cep350	centrosomal protein 350	63.79449	75.10463	79.08707	69.26142	70.27015	70.07316
17465535	Cep41	centrosomal protein 41	5.264513	5.795849	6.401633	5.599275	5.890255	5.338928
17509435	Cep44	centrosomal protein 44	6.6312	7.526035	7.314656	6.155517	10.23628	6.635108
17359098	Cep55	centrosomal protein 55	4.008521	4.358434	3.431668	3.227887	3.057177	3.278396
17523994	Cep57	centrosomal protein 57	17.24516	17.91083	18.49649	17.40572	15.03216	21.84544
17240484	Cep57l1	centrosomal protein 57-like 1	11.15687	12.60295	12.60295	14.29987	11.15772	10.9576
17530203	Cep63	centrosomal protein 63	16.3769	18.49649	21.18704	18.49649	19.14147	19.04935
17260855	Cep68	centrosomal protein 68	14.60398	16.10559	17.22951	14.53632	11.54647	15.0002
17520687	Cep70	centrosomal protein 70	3.549387	4.959381	5.094745	4.589796	5.464085	4.788122
17294324	Cep72	centrosomal protein 72	3.26916	5.187466	5.089274	4.470078	3.81724	4.031146
17355213	Cep76	centrosomal protein 76	18.16312	13.78436	13.09333	11.11018	13.78436	9.981938
17363220	Cep78	centrosomal protein 78	11.56369	9.972049	10.21084	9.922062	11.51298	11.60456
17236708	Cep83	centrosomal protein 83	22.70172	22.70172	22.70172	21.3643	22.70172	18.64646
17244435	Cep83os	centrosomal protein 83, opposite strand	8.441913	7.892925	8.441913	7.613765	9.310251	7.149389
17431187	Cep85	centrosomal protein 85	29.78533	32.48654	38.02694	43.15782	47.80534	41.49139
17240819	Cep85l	centrosomal protein 85-like	43.44084	38.42225	47.40772	57.40442	56.61294	56.70445
17476773	Cep89	centrosomal protein 89	6.760079	7.44076	9.24876	10.20766	7.201014	8.648021
17257612	Cep95	centrosomal protein 95	10.06085	11.01639	10.72414	11.10217	11.25817	11.57949
17330998	Cep97	centrosomal protein 97	12.12372	11.59451	9.961963	12.90859	11.59451	12.37666
17211090	Cspp1	centrosome and spindle pole associated protein 1	34.85708	30.75331	28.60569	34.22456	39.04024	35.54661
17320143	Cerk	ceramide kinase	14.64585	24.01061	30.74564	22.67593	23.80232	20.00405
17387246	Cerkl	ceramide kinase-like	10.934	12.80762	18.08686	14.16315	13.39423	15.1191
17400317	Cers2	ceramide synthase 2	192.9402	190.8466	189.9716	190.9102	174.8483	189.0468
17479027	Cers3	ceramide synthase 3	4.717688	3.646368	3.834876	3.570312	4.352065	6.029841
17498847	Cers4	ceramide synthase 4	9.047881	9.821817	11.15749	9.360074	8.433491	10.7376
17321630	Cers5	ceramide synthase 5	8.467112	9.023268	8.467112	7.423091	8.467112	7.538356
17371374	Cers6	ceramide synthase 6	59.21127	72.56591	73.58501	61.98082	72.77848	58.90875
17433909	Cptp	ceramide-1-phosphate transfer protein	25.76124	21.85483	23.16123	25.16872	16.638	22.64138
17426803	Cer1	cerberus 1 homolog (Xenopus laevis)	5.146137	4.790911	5.953054	4.582262	4.542354	7.328663
17542034	Cdr1	cerebellar degeneration related antigen 1	2.672759	2.713171	2.937869	2.570555	2.881999	2.716596
17495821	Cdr2	cerebellar degeneration-related 2	10.17756	11.19498	10.7208	12.76976	9.015516	10.8396
17258178	Cdr2l	cerebellar degeneration-related protein 2-like	22.21789	22.50292	23.02246	20.26193	14.82561	24.36706
17511474	Cbln1	cerebellin 1 precursor protein	6.606747	5.888907	5.453907	5.917591	6.030928	5.787539
17352265	Cbln2	cerebellin 2 precursor protein	6.570804	7.698994	6.788675	5.482409	6.179928	5.783168
17306921	Cbln3	cerebellin 3 precursor protein	5.984829	6.123472	5.672757	4.915608	5.147065	6.237509
17395009	Cbln4	cerebellin 4 precursor protein	3.892616	3.988566	3.988566	3.585191	4.055585	3.62555
17216683	Celrr	cerebellum expressed regulatory RNA	6.247028	6.156242	5.617429	6.247028	5.567038	6.776451
17469556	Crbn	cereblon	61.89496	75.81966	77.14389	78.70154	66.92438	84.67769
17247158	Ccm2	cerebral cavernous malformation 2	17.36523	17.36523	23.25711	17.36523	15.77808	15.26293
17377857	Ccm2l	cerebral cavernous malformation 2-like	12.2898	15.55903	14.76634	14.14353	12.43436	14.26611
17366428	Cdnf	cerebral dopamine neurotrophic factor	6.559837	4.801649	4.482924	5.765637	5.882625	6.004233
17368930	Cercam	cerebral endothelial cell adhesion molecule	3.498363	4.597342	3.676034	4.37393	4.280087	5.253103
17496310	Cln3	ceroid lipofuscinosis, neuronal 3, juvenile (Batten, Spielmeyer-Vogt disease)	30.58368	27.49905	29.01969	21.28197	28.82982	22.35848
17302483	Cln5	ceroid-lipofuscinosis, neuronal 5	72.96183	61.78137	64.41211	61.53861	77.20113	60.18366
17518227	Cln6	ceroid-lipofuscinosis, neuronal 6	19.03206	20.51148	27.70511	20.58797	16.92718	18.23161
17499436	Cln8	ceroid-lipofuscinosis, neuronal 8	21.83847	23.45494	20.88652	18.49649	19.56184	18.62954
17396260	Cp	ceruloplasmin	1216.466	1177.091	1082.394	998.5079	1186.774	885.8017
17539536	Figf	c-fos induced growth factor	9.151131	8.134987	9.64673	8.105212	7.027639	8.124055
17326527	Cggbp1	CGG triplet repeat binding protein 1	103.6618	89.67476	83.51596	81.04998	94.92393	93.36112
17374807	Chac1	ChaC, cation transport regulator 1	18.88724	18.34926	21.91338	18.55376	22.18942	18.19979
17261417	Chac2	ChaC, cation transport regulator 2	63.71407	51.07339	45.07076	59.91436	70.31448	72.57899
17245171	Cct2	chaperonin containing Tcp1, subunit 2 (beta)	308.6205	270.5272	249.9005	272.5033	251.8394	251.0359
17399012	Cct3	chaperonin containing Tcp1, subunit 3 (gamma)	128.0515	109.5602	108.2325	109.5617	86.68858	92.32887
17247656	Cct4	chaperonin containing Tcp1, subunit 4 (delta)	100.9684	108.6677	102.4276	107.0625	103.969	102.4276
17316328	Cct5	chaperonin containing Tcp1, subunit 5 (epsilon)	118.5104	104.8483	94.91538	97.17815	100.5988	107.6482
17442925	Cct6a	chaperonin containing Tcp1, subunit 6a (zeta)	293.8997	224.4344	216.6271	230.5427	202.8782	225.6542
17266773	Cct6b	chaperonin containing Tcp1, subunit 6b (zeta)	3.945068	4.346608	4.171829	4.375749	4.61001	4.429857
17460293	Cct7	chaperonin containing Tcp1, subunit 7 (eta)	91.44929	92.21056	88.98005	88.31215	87.16095	91.5533
17331774	Cct8	chaperonin containing Tcp1, subunit 8 (theta)	205.0696	179.7615	166.4067	179.0154	191.3049	179.7615
17435499	Cct8l1	chaperonin containing TCP1, subunit 8 (theta)-like 1	3.675626	3.861603	3.339703	2.953407	3.339703	3.339703
17513871	Chmp1a	charged multivesicular body protein 1A	47.30596	52.78368	59.05757	64.30683	52.06024	54.27403
17351444	Chmp1b	charged multivesicular body protein 1B	59.90523	66.77471	50.53006	69.96056	75.90326	60.05948
17486611	Chmp2a	charged multivesicular body protein 2A	151.2278	207.6385	187.3173	190.0203	200.496	208.3911
17331438	Chmp2b	charged multivesicular body protein 2B	59.44568	63.02796	74.13023	78.26362	71.25454	71.04986
17459504	Chmp3	charged multivesicular body protein 3	37.35242	43.43482	39.36714	35.1531	40.37695	40.99881
17378260	Chmp4b	charged multivesicular body protein 4B	68.29273	66.34473	65.00415	69.25998	81.11462	66.20737
17396069	Chmp4c	charged multivesicular body protein 4C	10.61542	10.82504	9.952852	11.47252	9.941958	11.54879
17412801	Chmp5	charged multivesicular body protein 5	157.7537	145.0483	141.1886	142.875	140.4955	157.1295
17259220	Chmp6	charged multivesicular body protein 6	24.52617	23.70698	23.70698	27.05794	20.40994	23.26163
17308182	Chmp7	charged multivesicular body protein 7	62.53188	62.47772	61.89453	68.48851	57.62328	58.74931
17525503	Chek1	checkpoint kinase 1	4.387928	5.172709	4.352065	4.57287	4.352065	4.459772
17440575	Chek2	checkpoint kinase 2	11.6396	11.06652	8.375295	9.822362	9.946783	9.224687
17440387	Chfr	checkpoint with forkhead and ring finger domains	34.51281	35.29618	28.78988	35.29618	32.50388	40.01475
17229187	Xcl1	chemokine (C motif) ligand 1	7.513734	7.814535	7.814535	8.51685	9.120102	7.814535
17532564	Xcr1	chemokine (C motif) receptor 1	5.143178	4.987269	5.143178	4.848303	4.635195	6.878719
17266754	Ccl1	chemokine (C-C motif) ligand 1	6.055474	6.384749	5.445445	7.100792	7.063631	6.244257
17254053	Ccl11	chemokine (C-C motif) ligand 11	9.777055	9.335388	8.597219	9.335388	11.33655	11.24366
17254059	Ccl12	chemokine (C-C motif) ligand 12	3.442269	3.558527	3.762522	3.270611	3.178465	2.874199

17504138	Ccl17	chemokine (C-C motif) ligand 17	4.806278	4.125674	3.175008	3.781163	3.702552	4.096012
17434280	Ccl19	chemokine (C-C motif) ligand 19	14.57264	14.91581	19.39819	14.21419	14.30641	17.29988
17254041	Ccl2	chemokine (C-C motif) ligand 2	12.07298	17.44994	14.62769	16.70734	12.68578	9.018891
17214857	Ccl20	chemokine (C-C motif) ligand 20	5.600944	6.667426	7.742825	6.402743	7.013721	8.752772
17504122	Ccl22	chemokine (C-C motif) ligand 22	4.739977	6.143965	5.021749	5.774436	6.820795	5.426154
17453611	Ccl24	chemokine (C-C motif) ligand 24	11.74651	14.33848	20.87479	16.23595	14.43961	9.415254
17498830	Ccl25	chemokine (C-C motif) ligand 25	34.23096	33.9759	36.78842	35.60135	24.62855	30.9522
17453607	Ccl26	chemokine (C-C motif) ligand 26	4.381425	3.939848	3.54506	3.292378	2.84591	4.01862
17424331	Ccl27a	chemokine (C-C motif) ligand 27A	13.40398	13.72429	13.02228	13.31712	11.56746	13.39953
17290163	Ccl28	chemokine (C-C motif) ligand 28	5.727518	5.696122	4.760292	6.255261	5.595705	5.79822
17266967	Ccl3	chemokine (C-C motif) ligand 3	5.338965	5.378709	7.698994	5.338965	7.261311	5.597839
17254283	Ccl4	chemokine (C-C motif) ligand 4	12.29654	8.637836	8.429225	8.429225	8.978286	11.60352
17266946	Ccl5	chemokine (C-C motif) ligand 5	30.51274	38.3919	33.36469	28.54745	29.4654	32.33176
17266960	Ccl6	chemokine (C-C motif) ligand 6	21.12999	23.93915	43.24825	26.79831	25.7682	21.79205
17254047	Ccl7	chemokine (C-C motif) ligand 7	18.29896	26.76118	24.62599	16.66582	21.36148	21.24498
17254065	Ccl8	chemokine (C-C motif) ligand 8	4.083907	3.640054	3.140687	4.459679	4.581976	3.872218
17266952	Ccl9	chemokine (C-C motif) ligand 9	210.9621	223.9946	231.8535	241.9475	327.1821	283.9691
17532569	Ccr1	chemokine (C-C motif) receptor 1	3.564328	3.098607	4.008799	4.545125	2.920732	2.783635
17269806	Ccr10	chemokine (C-C motif) receptor 10	6.747813	6.017357	7.468042	7.436377	5.467912	6.848699
17532574	Ccr1l1	chemokine (C-C motif) receptor 1-like 1	4.571508	2.861318	2.481049	2.789526	2.769125	2.861318
17523650	Ccr2	chemokine (C-C motif) receptor 2	4.548151	4.346488	4.827747	4.355354	4.351642	4.911828
17523646	Ccr3	chemokine (C-C motif) receptor 3	2.81911	2.81911	2.741832	2.81911	2.81911	2.81911
17531887	Ccr4	chemokine (C-C motif) receptor 4	4.620759	3.651097	4.137244	3.309997	3.940941	3.709154
17523659	Ccr5	chemokine (C-C motif) receptor 5	9.116689	9.237036	10.66876	9.194238	9.194238	5.79103
17333066	Ccr6	chemokine (C-C motif) receptor 6	4.537948	4.537948	4.244928	4.708084	4.774998	4.537948
17268972	Ccr7	chemokine (C-C motif) receptor 7	6.169185	10.98201	9.389641	10.24771	7.066227	8.140966
17523158	Ccr8	chemokine (C-C motif) receptor 8	4.327789	4.619927	4.385222	4.091302	3.463018	6.71115
17523633	Ccr9	chemokine (C-C motif) receptor 9	2.406006	3.685295	3.685295	3.114499	3.601755	3.685295
17531705	Ccrl2	chemokine (C-C motif) receptor-like 2	7.891557	7.938245	6.295278	6.173677	5.990473	7.667467
17504130	Cx3cl1	chemokine (C-X3-C motif) ligand 1	12.46914	11.78966	10.70995	13.86725	13.25395	12.19561
17532268	Cx3cr1	chemokine (C-X3-C motif) receptor 1	13.23898	16.36612	15.23914	18.93574	14.93174	14.84063
17438987	Cxcl1	chemokine (C-X-C motif) ligand 1	23.19384	21.50559	33.35058	31.25684	31.5833	22.37941
17449718	Cxcl10	chemokine (C-X-C motif) ligand 10	18.73845	14.99968	18.52305	13.52953	14.99968	14.30998
17449725	Cxcl11	chemokine (C-X-C motif) ligand 11	7.319728	12.51	12.53708	16.80571	12.43082	7.730437
17462149	Cxcl12	chemokine (C-X-C motif) ligand 12	245.9344	223.201	249.4799	209.1262	230.8385	216.6992
17439367	Cxcl13	chemokine (C-X-C motif) ligand 13	28.07103	23.42781	16.74583	17.49778	33.94954	25.27799
17293006	Cxcl14	chemokine (C-X-C motif) ligand 14	32.20717	25.08686	23.75962	31.53277	61.57616	30.63332
17438980	Cxcl15	chemokine (C-X-C motif) ligand 15	4.436402	4.362182	4.029218	3.832989	4.094508	3.812111
17265268	Cxcl16	chemokine (C-X-C motif) ligand 16	33.20204	45.92411	45.03454	34.34004	32.44648	30.96078
17487945	Cxcl17	chemokine (C-X-C motif) ligand 17	7.694086	8.217389	9.563707	8.774397	6.795125	9.074575
17438995	Cxcl2	chemokine (C-X-C motif) ligand 2	10.85961	9.945404	10.62219	8.778534	9.491065	10.83938
17438975	Cxcl3	chemokine (C-X-C motif) ligand 3	2.216003	2.817372	2.728882	2.961319	2.458656	2.652844
17438955	Cxcl5	chemokine (C-X-C motif) ligand 5	4.392189	5.379268	4.771027	4.583088	4.655416	4.927241
17449710	Cxcl9	chemokine (C-X-C motif) ligand 9	129.884	85.88415	85.48385	71.79542	111.1427	86.38806
17224251	Cxcr1	chemokine (C-X-C motif) receptor 1	8.120508	10.72644	8.184873	8.798576	8.798576	9.342897
17214142	Cxcr2	chemokine (C-X-C motif) receptor 2	6.424481	7.937639	5.79372	6.951913	6.979047	7.599529
17543625	Cxcr3	chemokine (C-X-C motif) receptor 3	5.94761	6.287864	7.277082	7.556457	5.582417	5.644829
17226593	Cxcr4	chemokine (C-X-C motif) receptor 4	16.90537	14.12736	13.99171	12.00797	13.60571	13.54633
17526310	Cxcr5	chemokine (C-X-C motif) receptor 5	9.280741	6.750339	7.302245	7.200115	6.310569	7.179782
17523642	Cxcr6	chemokine (C-X-C motif) receptor 6	3.439789	3.579554	4.469303	3.729934	4.256094	3.175558
17504422	Cklf	chemokine-like factor	4.343446	5.504723	4.184108	4.149622	3.671934	4.421298
17451390	Cmklr1	chemokine-like receptor 1	8.425019	8.012709	12.07173	8.49221	7.852217	7.157571
17313008	Cby1	chibby homolog 1 (Drosophila)	20.72054	20.86727	23.08078	23.46177	17.30588	22.0583
17249172	Cby3	chibby homolog 3 (Drosophila)	8.9633	9.910515	7.544629	8.933652	10.65863	10.76995
17386569	Chn1	chimerin 1	3.796184	3.569965	3.540222	3.569965	3.526947	4.750601
17372021	Chn1os1	chimerin 1, opposite strand 1 [Source:MGI Symbol;Acc:MGI:3702041]	3.207924	3.306787	3.271019	3.561191	4.039313	5.160461
17372013	Chn1os3	chimerin 1, opposite strand 3	6.273324	5.56106	5.120306	7.03451	5.482333	6.079109
17458692	Chn2	chimerin 2	51.44397	61.84521	64.70925	52.14945	55.34496	60.62328
17217383	Chit1	chitinase 1 (chitotriosidase)	5.889438	6.504779	5.979356	6.707151	6.564659	5.889438
17497957	Chid1	chitinase domain containing 1	34.75094	18.03853	23.07534	26.7217	23.54045	22.48062
17401465	Chia1	chitinase, acidic 1	3.402063	3.263862	3.222119	3.263862	3.715973	3.522907
17217399	Chil1	chitinase-like 1	10.28685	8.322114	9.880198	11.37757	7.169736	8.914476
17408897	Chil3	chitinase-like 3	2.747596	4.20927	2.43277	2.953407	3.015819	3.69481
17408911	Chil4	chitinase-like 4	5.610658	3.669389	4.038473	4.080455	4.806844	4.117418
17408885	Chil5	chitinase-like 5	2.338754	2.43277	2.524487	2.588991	2.458673	2.369643
17408926	Chil6	chitinase-like 6	3.065452	3.557441	4.248872	3.92483	3.019689	4.864731
17403533	Ctbs	chitobiase, di-N-acetyl-	20.05222	20.44846	22.57134	15.41732	24.11468	21.73563
17539797	Clcn5	chloride channel 5	21.79885	22.25396	25.66215	19.61329	21.19379	17.92002
17410939	Clca1	chloride channel accessory 1	6.116982	5.821537	5.713441	6.229904	5.973556	5.716928
17410956	Clca2	chloride channel accessory 2	2.952322	3.751064	3.777679	3.048818	2.726972	3.887843
17410845	Clca3a1	chloride channel accessory 3A1	15.1491	17.11323	14.96717	16.58986	13.7855	9.310251
17410863	Clca3a2	chloride channel accessory 3A2	3.274263	2.545378	2.60364	2.813371	2.787361	2.249213
17410880	Clca3b	chloride channel accessory 3B	3.049558	2.763717	2.845969	2.911737	3.365101	3.20004
17410921	Clca4a	chloride channel accessory 4A	5.576152	6.241834	4.604358	6.897148	7.945896	6.958662
17410905	Clca4b	chloride channel accessory 4B	3.462322	2.298257	1.873414	2.507483	2.625842	2.254941
17410898	Clca4c-ps	chloride channel accessory 4C, pseudogene	2.985164	4.18492	2.610489	3.281897	3.093071	4.507664
17401768	Clcc1	chloride channel CLIC-like 1	22.2085	24.65913	23.45267	25.51016	22.40611	25.72519
17480407	Clns1a	chloride channel, nucleotide-sensitive, 1A	58.36979	55.86218	47.99626	53.51643	51.12333	55.20397
17457913	Clcn1	chloride channel, voltage-sensitive 1	5.885194	4.678513	6.992896	6.164483	4.994049	5.640527
17329167	Clcn2	chloride channel, voltage-sensitive 2	61.68766	63.58127	52.79063	66.62446	65.71679	69.23742
17509506	Clcn3	chloride channel, voltage-sensitive 3	27.66653	26.36112	24.36908	33.28938	30.10372	23.67496
17486173	Clcn4	chloride channel, voltage-sensitive 4	61.42781	48.59379	54.48926	48.20965	60.76716	45.08139
17432865	Clcn6	chloride channel, voltage-sensitive 6	6.76125	8.449992	10.01049	7.243766	11.2109	8.420524
17334545	Clcn7	chloride channel, voltage-sensitive 7	39.56108	36.77007	38.01218	36.93739	36.7507	32.08217

17432148	Clcnka	chloride channel, voltage-sensitive Ka	5.067029	7.165861	6.443748	9.048759	7.610282	6.46845
17432173	Clcnkb	chloride channel, voltage-sensitive Kb	11.81354	12.04498	10.76264	11.44488	13.66345	14.22533
17336896	Clic1	chloride intracellular channel 1	27.75354	29.29698	36.76625	35.29446	27.88718	29.29698
17368069	Clic3	chloride intracellular channel 3	13.29968	10.07243	12.14744	12.11347	9.498897	11.36338
17431394	Clic4	chloride intracellular channel 4 (mitochondrial)	179.7739	153.9566	190.6893	161.4944	153.6869	132.6242
17337852	Clic5	chloride intracellular channel 5	11.90735	10.94159	11.14975	14.36255	14.22138	12.0373
17327236	Clic6	chloride intracellular channel 6	3.54506	2.983343	3.689768	4.098256	3.958352	3.9518
17532334	Cck	cholecystokinin	4.489859	5.308374	4.982121	4.433297	7.199666	4.999972
17448135	Cckar	cholecystokinin A receptor	3.748465	3.574719	2.533743	3.077865	2.996046	3.809205
17481378	Cckbr	cholecystokinin B receptor	4.348507	6.092608	4.860012	4.407498	5.728709	4.742244
17364111	Ch25h	cholesterol 25-hydroxylase	3.949828	3.657429	4.552692	3.713321	4.077232	4.960722
17304880	Chat	choline acetyltransferase	5.528431	5.032307	3.634684	4.681043	3.73296	5.305284
17298155	Chdh	choline dehydrogenase	128.0275	128.0275	140.7675	122.7177	129.6474	114.0545
17355984	Chka	choline kinase alpha	105.3926	78.77249	60.0241	81.41372	87.19358	72.23069
17320499	Chkb	choline kinase beta	20.62268	32.29995	37.08927	26.76619	18.94821	27.91425
17243888	Chpt1	choline phosphotransferase 1	110.0382	127.011	93.80611	120.0074	105.7197	117.6934
17408945	Cept1	choline/ethanolaminephosphotransferase 1	154.3466	136.1922	151.143	126.3656	120.1789	121.6717
17357120	Chrm1	cholinergic receptor, muscarinic 1, CNS	8.561657	10.30905	8.561657	8.083724	7.91654	10.33732
17457310	Chrm2	cholinergic receptor, muscarinic 2, cardiac	4.257127	3.07842	3.686725	5.259589	4.745565	3.829272
17290447	Chrm3	cholinergic receptor, muscarinic 3, cardiac	12.1217	5.898058	6.287864	5.752145	8.381451	4.836824
17373443	Chrm4	cholinergic receptor, muscarinic 4	3.916541	4.165703	5.993796	4.514387	4.739813	4.680848
17389335	Chrm5	cholinergic receptor, muscarinic 5	2.939743	3.639146	3.074944	2.912625	3.112082	3.397586
17386557	Chrna1	cholinergic receptor, nicotinic, alpha polypeptide 1 (muscle)	2.953407	3.478439	2.472808	2.953407	2.828015	3.20004
17494033	Chrna10	cholinergic receptor, nicotinic, alpha polypeptide 10	4.261585	5.886663	3.644498	3.868435	4.829219	4.159067
17301468	Chrna2	cholinergic receptor, nicotinic, alpha polypeptide 2 (neuronal)	34.6689	32.43366	33.26401	30.63056	23.90897	27.23028
17527312	Chrna3	cholinergic receptor, nicotinic, alpha polypeptide 3	7.628397	5.968507	6.606976	5.519548	6.562793	7.676454
17395718	Chrna4	cholinergic receptor, nicotinic, alpha polypeptide 4	5.567501	5.16565	5.589348	7.514932	5.906895	5.748478
17517469	Chrna5	cholinergic receptor, nicotinic, alpha polypeptide 5	3.530029	3.713681	4.050865	3.713681	3.713681	3.906455
17508554	Chrna6	cholinergic receptor, nicotinic, alpha polypeptide 6	4.793136	6.187343	6.192749	5.022689	6.684316	5.29397
17491763	Chrna7	cholinergic receptor, nicotinic, alpha polypeptide 7	14.16089	15.36771	13.48141	12.49068	12.06236	10.51787
17437836	Chrna9	cholinergic receptor, nicotinic, alpha polypeptide 9	5.839278	5.223837	5.733953	6.185542	5.449788	5.978029
17264946	Chrnb1	cholinergic receptor, nicotinic, beta polypeptide 1 (muscle)	4.031705	3.753249	4.808677	3.919501	4.28347	3.899935
17407124	Chrnb2	cholinergic receptor, nicotinic, beta polypeptide 2 (neuronal)	4.990658	4.800118	6.747345	3.638036	4.867024	4.517204
17500340	Chrnb3	cholinergic receptor, nicotinic, beta polypeptide 3	3.703322	4.785733	3.703322	4.469118	3.89097	3.339703
17527323	Chrnb4	cholinergic receptor, nicotinic, beta polypeptide 4	8.34279	9.235179	8.802541	10.11167	8.51685	9.899324
17215192	Chrnd	cholinergic receptor, nicotinic, delta polypeptide	6.992896	6.328967	7.799816	5.752863	5.11988	8.652758
17265285	Chrne	cholinergic receptor, nicotinic, epsilon polypeptide	5.073601	7.408852	6.016982	6.091848	5.267104	6.434553
17215208	Chrng	cholinergic receptor, nicotinic, gamma polypeptide	6.943774	7.413938	6.49928	6.943774	6.943774	6.283333
17255227	Chad	chondroadherin	7.040608	6.139708	7.698994	6.867	7.433491	5.532226
17319494	Chadl	chondroadherin-like	7.76061	8.764246	8.992486	7.76061	5.988398	6.015606
17224620	Chpf	chondroitin polymerizing factor	17.26454	16.13498	18.55724	17.30564	19.22071	15.31443
17435403	Chpf2	chondroitin polymerizing factor 2	9.888488	13.27426	11.56746	11.99175	11.52025	14.14196
17509758	Csgalnact1	chondroitin sulfate N-acetylgalactosaminyltransferase 1	4.998932	4.752972	4.479352	4.632529	3.912254	4.09255
17470105	Csgalnact2	chondroitin sulfate N-acetylgalactosaminyltransferase 2	15.5669	10.91326	12.08671	12.2489	12.6625	17.05307
17517592	Cspg4	chondroitin sulfate proteoglycan 4	8.420524	12.96131	8.420524	8.420524	7.151173	10.00355
17522319	Cspg5	chondroitin sulfate proteoglycan 5	7.401615	8.598232	5.708889	6.267977	7.681936	6.926661
17479006	Chsy1	chondroitin sulfate synthase 1	32.40535	40.4927	41.67586	36.89875	40.86602	40.78085
17350909	Chsy3	chondroitin sulfate synthase 3	22.2045	18.7317	18.38338	15.44438	15.5628	23.68645
17326735	Chodl	chondrolectin	3.862353	3.795395	4.000083	3.580125	3.79908	3.446185
17324135	Chrd	chordin	7.373962	6.400879	7.893417	7.123025	6.394848	8.331233
17545087	Chrdl1	chordin-like 1	3.754472	4.801649	3.104885	4.422616	3.279464	3.729055
17480620	Chrdl2	chordin-like 2	6.440488	7.910835	6.440488	5.932006	6.719528	6.62284
17230174	Chml	choroideremia-like	9.266728	10.18815	9.123011	9.123011	8.642628	8.633587
17544244	Chm	choroidermia (RAB escort protein 1)	47.95934	46.70516	45.87798	48.55271	51.26847	44.22438
17312089	Chrac1	chromatin accessibility complex 1	30.08036	29.49033	29.06236	28.47413	29.28967	33.08235
17338701	Chaf1a	chromatin assembly factor 1, subunit A (p150)	6.339109	8.40146	6.575087	6.465446	8.116006	9.569543
17327331	Chaf1b	chromatin assembly factor 1, subunit B (p60)	10.49071	10.38004	11.29851	8.882772	10.27894	10.54955
17506418	Cdt1	chromatin licensing and DNA replication factor 1	13.06803	17.11797	17.74462	11.38246	14.99469	16.19549
17407341	Chtop	chromatin target of PRMT1	28.86281	32.65654	25.20661	31.03749	31.03749	35.46146
17255653	Cbx1	chromobox 1	31.01555	27.42691	30.19756	34.99677	33.20457	32.28764
17258947	Cbx2	chromobox 2	35.96296	34.12318	34.12318	30.87763	36.00187	38.47688
17458561	Cbx3	chromobox 3	53.7026	77.37577	75.66216	80.33852	70.04014	91.34476
17288221	Cbx3-ps2	chromobox 3, pseudogene 2 [Source:MGI Symbol;Acc:MGI:1890539]	2.535482	2.67402	2.67402	2.459219	2.67402	3.339703
17308622	Cbx3-ps6	chromobox 3, pseudogene 6 [Source:MGI Symbol;Acc:MGI:3646710]	151.1796	191.2452	213.0015	189.7755	221.8084	230.9809
17213185	Cbx3-ps7	chromobox 3, pseudogene 7 [Source:MGI Symbol;Acc:MGI:3704187]	76.53883	76.19424	75.07653	71.18888	78.71027	85.33407
17548912	Cbx3-ps7	chromobox 3, pseudogene 7 [Source:MGI Symbol;Acc:MGI:3704187]	85.00053	94.26433	107.3568	94.51622	100.2209	112.9539
17272857	Cbx4	chromobox 4	31.56907	31.66594	27.52747	35.39614	29.82975	39.77362
17322327	Cbx5	chromobox 5	23.89919	23.18806	22.40626	23.22699	23.89919	25.36118
17319366	Cbx7	chromobox 7	14.42761	17.55634	15.36558	17.91381	15.91362	17.42697
17272845	Cbx8	chromobox 8	7.664172	9.003525	8.041809	9.568084	8.03039	9.497778
17333589	Chd1	chromodomain helicase DNA binding protein 1	74.81782	71.78647	61.81393	71.02377	60.74821	62.12405
17408184	Chd1l	chromodomain helicase DNA binding protein 1-like	12.20474	14.93929	16.83206	14.73137	16.83206	14.97444
17492114	Chd2	chromodomain helicase DNA binding protein 2	22.04455	29.70883	32.78264	26.27229	29.7041	29.70101
17264588	Chd3	chromodomain helicase DNA binding protein 3	64.10833	76.74921	95.0576	86.12797	72.31485	75.333
17251634	Chd3os	chromodomain helicase DNA binding protein 3, opposite strand	4.063154	3.751922	4.151953	4.172754	5.159531	4.134606
17463056	Chd4	chromodomain helicase DNA binding protein 4	96.33713	100.0086	93.16719	101.4904	89.67911	91.44865
17422170	Chd5	chromodomain helicase DNA binding protein 5	12.53961	12.88115	11.2834	11.86212	15.32909	12.65346
17393910	Chd6	chromodomain helicase DNA binding protein 6	17.49717	17.98958	19.05846	18.04553	18.3067	20.79219
17411647	Chd7	chromodomain helicase DNA binding protein 7	17.29556	25.96111	29.57672	27.5164	28.7253	31.22631
17306230	Chd8	chromodomain helicase DNA binding protein 8	43.73875	41.93724	43.96123	40.95478	47.87	45.47904
17503708	Chd9	chromodomain helicase DNA binding protein 9	34.38435	33.50815	33.71994	30.28035	33.3707	26.49246
17286553	Cdyl	chromodomain protein, Y chromosome-like	8.504403	9.419758	10.63936	9.436553	9.69412	9.176856
17513297	Cdyl2	chromodomain protein, Y chromosome-like 2	12.98739	15.6282	12.62219	12.98739	14.28055	13.27068
17278090	Chga	chromogranin A	6.286949	10.10565	8.088712	7.161193	7.35059	7.299006

17376618	Chgb	chromogranin B	4.699476	4.641915	4.687829	5.551778	4.971978	4.787359
17499387	Champ1	chromosome alignment maintaining phosphoprotein 1	15.88816	20.76972	22.14808	20.71179	20.7536	18.66664
17379808	Cse1l	chromosome segregation 1-like (S. cerevisiae)	128.4684	120.8924	98.91106	118.0701	104.3604	103.7646
17276614	Churc1	churchill domain containing 1	59.28437	56.08449	42.43492	54.01132	55.27557	58.91079
17306937	Cma1	chymase 1, mast cell	2.885157	2.529408	2.491544	2.722421	2.81074	3.259114
17300707	Cma2	chymase 2, mast cell	12.29995	9.683375	11.22738	14.69171	16.04353	14.20336
17408974	Cym	chymosin	12.02954	16.43273	16.43273	15.75382	18.68126	16.43273
17432330	Ctrc	chymotrypsin C (caldecrin)	6.669506	9.550326	6.992896	8.324306	5.712095	7.399357
17421138	Ctrcos	chymotrypsin C (caldecrin), opposite strand	9.762834	12.89715	13.41365	11.39509	11.56746	8.566228
17512544	Ctrl	chymotrypsin-like	7.127964	7.121198	6.663037	6.799271	6.992896	10.02965
17321780	Cela1	chymotrypsin-like elastase family, member 1	10.70951	9.515174	10.30755	10.80622	9.515174	11.45766
17432320	Cela2a	chymotrypsin-like elastase family, member 2A	6.781009	10.11848	10.11848	12.06988	8.156329	10.11848
17431683	Cela3a	chymotrypsin-like elastase family, member 3A	4.429335	3.420886	3.984034	2.832763	3.468423	2.868666
17431693	Cela3b	chymotrypsin-like elastase family, member 3B	4.022521	3.73252	3.59116	4.034193	5.05377	3.600083
17513149	Ctrb1	chymotrypsinogen B1	16.31266	17.77897	14.50532	11.74415	13.67321	12.57128
17468900	Cfap100	cilia and flagella associated protein 100	3.083161	3.508587	3.560476	3.551036	3.317623	3.670687
17219208	Cfap126	cilia and flagella associated protein 126	6.840254	9.0837	7.992961	6.363763	6.732816	7.992961
17384008	Cfap157	cilia and flagella associated protein 157	17.43893	20.1619	20.42055	22.68411	18.02778	20.68639
17492930	Cfap161	cilia and flagella associated protein 161	4.681043	4.714321	4.494311	5.062711	4.681043	5.863451
17512001	Cfap20	cilia and flagella associated protein 20	36.30358	40.34142	38.12618	41.51296	39.82742	51.31931
17423867	Cfap206	cilia and flagella associated protein 206	5.673779	4.43044	3.819967	4.33181	4.603209	4.396727
17226269	Cfap221	cilia and flagella associated protein 221	5.48933	5.528162	5.331125	5.48933	5.969789	6.117024
17261250	Cfap36	cilia and flagella associated protein 36	28.29843	28.7261	29.64342	34.60407	33.69844	29.92792
17365571	Cfap43	cilia and flagella associated protein 43	4.427554	3.734875	3.942963	3.703322	3.563661	3.863531
17325810	Cfap44	cilia and flagella associated protein 44	3.757804	4.51758	5.192359	4.647678	4.83669	4.336617
17219591	Cfap45	cilia and flagella associated protein 45	8.832672	7.953225	8.328519	10.02402	7.116806	11.45948
17497458	Cfap46	cilia and flagella associated protein 46	7.282531	7.343206	7.282531	6.384705	9.78336	6.948926
17497477	Cfap46	cilia and flagella associated protein 46	4.547425	4.360459	4.076978	3.782998	3.94258	4.266432
17264382	Cfap52	cilia and flagella associated protein 52	5.114595	6.817959	5.491499	5.295218	6.030928	6.386786
17351747	Cfap53	cilia and flagella associated protein 53	4.095671	4.205158	3.583725	4.275224	3.97529	3.828449
17244263	Cfap54	cilia and flagella associated protein 54	3.873103	3.946791	3.960026	3.856067	3.722444	4.037662
17429245	Cfap57	cilia and flagella associated protein 57	5.60141	5.450694	5.450694	6.152403	6.089315	6.519901
17360240	Cfap58	cilia and flagella associated protein 58	7.874455	5.918247	7.192633	6.733189	9.746134	7.577962
17377210	Cfap61	cilia and flagella associated protein 61	6.428	6.382183	7.116362	6.958276	6.516693	6.347815
17445394	Cfap69	cilia and flagella associated protein 69	4.01461	5.062711	4.242604	3.831289	5.22909	4.681043
17303769	Cfap70	cilia and flagella associated protein 70	4.781986	5.651955	5.552279	4.935102	4.511634	4.935102
17452030	Cfap73	cilia and flagella associated protein 73	8.510713	10.57085	8.867024	9.732834	9.732834	9.428511
17422521	Cfap74	cilia and flagella associated protein 74	5.562024	4.571349	5.160318	4.905601	4.193399	4.886969
17422546	Cfap74	cilia and flagella associated protein 74	4.01461	4.826408	6.473518	4.451063	4.665753	5.265243
17383452	Cfap77	cilia and flagella associated protein 77	7.005461	6.461817	6.759891	6.620591	5.628927	7.046222
17500975	Cfap97	cilia and flagella associated protein 97	38.42491	33.77233	39.77583	40.64652	37.46314	40.90315
17436558	Cfap99	cilia and flagella associated protein 99	6.555746	8.906879	8.28769	10.2112	9.092897	9.087919
17424279	Cntfr	ciliary neurotrophic factor receptor	7.861747	11.34488	14.55628	11.91403	10.12437	11.19242
17432082	Crocc	ciliary rootlet coiled-coil, rootletin	11.56834	12.2624	13.39423	12.81081	13.78875	14.2003
17215899	Crocc2	ciliary rootlet coiled-coil, rootletin family member 2	14.22799	16.23595	13.35835	15.7053	14.48362	14.48362
17214184	Catip	ciliogenesis associated TTC17 interacting protein	5.099055	5.099055	5.099055	5.517673	4.196481	5.099055
17407594	Cgn	cingulin	40.87736	45.02486	45.02486	32.37504	38.18621	49.04044
17407621	BC021767	cingulin pseudogene	17.62975	11.807	21.19761	21.77287	20.87769	11.83682
17528692	Cgnl1	cingulin-like 1	82.11136	77.33609	89.78111	74.91642	84.00655	75.88538
17407934	Ciart	circadian associated repressor of transcription	12.10054	16.94479	14.65397	15.08497	15.63535	19.3067
17448960	Clock	circadian locomotor output cycles kaput	268.748	231.6945	198.9276	256.0639	180.5557	205.0241
17505197	Cirh1a	cirrhosis, autosomal recessive 1A (human)	109.5093	82.50102	83.34883	89.69154	66.23216	69.7161
17302869	Clybl	citrate lyase beta like	145.4601	180.0658	186.1782	161.3724	136.1554	150.7924
17238433	Cs	citrate synthase	169.6926	177.3628	174.1033	183.5654	156.752	169.5645
17244563	Csl	citrate synthase like	6.310175	7.423554	6.595262	6.569119	5.380052	4.20927
17441275	Cit	citron	5.515608	5.882625	5.909393	5.831189	5.974633	5.909393
17512209	Cmtm1	CKLF-like MARVEL transmembrane domain containing 1	2.996275	2.0225	2.167272	2.182528	2.283869	3.555223
17512193	Cmtm2a	CKLF-like MARVEL transmembrane domain containing 2A	3.255818	2.692507	2.568365	2.854227	2.876531	2.927684
17504437	Cmtm2b	CKLF-like MARVEL transmembrane domain containing 2B	3.194633	2.783075	2.478436	3.086132	3.129192	2.670278
17504444	Cmtm3	CKLF-like MARVEL transmembrane domain containing 3	19.49537	19.86215	29.07163	20.84934	18.66987	15.43764
17512221	Cmtm4	CKLF-like MARVEL transmembrane domain containing 4	62.27063	67.1665	69.32818	60.59513	61.29766	65.70494
17300381	Cmtm5	CKLF-like MARVEL transmembrane domain containing 5	10.58515	10.22657	10.55665	8.325114	8.672993	9.947059
17522779	Cmtm6	CKLF-like MARVEL transmembrane domain containing 6	163.8305	145.5701	123.82	136.0789	126.1066	108.1005
17531923	Cmtm7	CKLF-like MARVEL transmembrane domain containing 7	14.66861	18.8752	24.05575	21.05426	19.22831	12.11028
17531932	Cmtm8	CKLF-like MARVEL transmembrane domain containing 8	91.83677	94.03527	97.80118	85.96098	111.028	117.7073
17405446	Clrn1	clarin 1	3.633198	4.750305	3.843258	5.923993	4.911794	4.385344
17437241	Clrn2	clarin 2	5.805619	7.648729	5.7272	7.039046	6.776076	5.00552
17497320	Clrn3	clarin 3	4.952133	5.404046	5.063939	5.598809	5.422319	6.292398
17418592	Clspn	claspin	7.417125	5.913655	6.008896	6.160468	5.123324	6.680148
17322842	Ciita	class II transactivator	7.056242	8.310354	7.378308	7.188895	6.996823	7.199017
17248086	Clhc1	clathrin heavy chain linker domain containing 1	3.017471	2.800453	2.963783	3.072621	2.915534	3.108158
17248728	Clint1	clathrin interactor 1	125.318	109.9802	132.4276	104.4849	110.6462	96.93932
17267353	Cltc	clathrin, heavy polypeptide (Hc)	316.1685	272.7981	269.8979	241.8737	241.5018	254.318
17413511	Clta	clathrin, light polypeptide (Lca)	69.94456	86.01343	84.22753	74.21609	75.03543	77.3377
17292722	Cltb	clathrin, light polypeptide (Lcb)	87.97185	79.68907	89.02015	94.10569	85.40212	71.50043
17329479	Cldn1	claudin 1	223.4085	134.8629	137.3145	167.4191	189.2789	176.5166
17302711	Cldn10	claudin 10	3.946845	3.946845	4.056794	3.379009	4.162499	3.987432
17396652	Cldn11	claudin 11	12.71897	11.5673	11.64824	16.00233	12.07943	10.33273
17445370	Cldn12	claudin 12	54.12788	53.71935	47.0623	52.99521	52.1574	61.96106
17453492	Cldn13	claudin 13	8.28993	7.202561	7.003373	9.698422	11.59853	7.799545
17332290	Cldn14	claudin 14	8.473159	9.551187	8.406178	8.406178	8.879694	8.207142
17443486	Cldn15	claudin 15	6.130607	5.275106	5.233478	5.889112	4.159874	5.073988
17324513	Cldn16	claudin 16	3.517499	3.42872	3.651983	3.861095	3.703887	3.735341
17331824	Cldn17	claudin 17	2.67402	2.421784	2.67402	3.054508	2.67402	2.636602

17530094	Cldn18	claudin 18	5.618325	5.29452	5.710432	3.967609	4.989413	4.681887
17417898	Cldn19	claudin 19	12.81774	17.71488	15.7992	15.33621	15.80369	15.84801
17538134	Cldn2	claudin 2	8.350601	9.689888	9.612089	8.290965	8.71107	6.500767
17332728	Cldn20	claudin 20	4.558376	4.269124	4.805911	7.505926	4.556702	4.566535
17501098	Cldn22	claudin 22	4.322782	3.83836	4.205041	4.651706	3.654135	3.927551
17508779	Cldn23	claudin 23	10.38069	11.6201	12.75243	10.01305	15.00311	10.48337
17501096	Cldn24	claudin 24	3.533824	3.843951	3.447331	3.516499	3.613257	4.1718
17443148	Cldn3	claudin 3	115.9613	118.563	123.2744	103.2623	118.7373	120.0158
17542897	Cldn34-ps	claudin 34, pseudogene	2.331874	2.095072	2.403372	2.416317	2.584294	2.321295
17538958	Cldn34a	claudin 34A	2.794713	3.410729	3.051249	3.123189	3.1982	3.216206
17545543	Cldn34b1	claudin 34B1	2.496903	2.677469	2.677469	2.304206	3.752547	2.937889
17545588	Cldn34b2	claudin 34B2	2.665193	2.95777	2.291401	2.5601	2.037881	2.391126
17535991	Cldn34b3	claudin 34B3	2.074509	1.968504	2.182467	1.831163	1.876989	2.361691
17535997	Cldn34b4	claudin 34B4	2.989118	2.661944	2.416602	2.624095	2.701012	2.919315
17537379	Cldn34c1	claudin 34C1	3.338199	3.43631	3.429718	3.208258	3.431787	3.463162
17537394	Cldn34c2	claudin 34C2	4.989655	5.061307	4.271936	5.393755	5.544395	5.487466
17537397	Cldn34c3	claudin 34C3	2.564433	1.965001	2.527218	2.309675	2.133112	2.967422
17544352	Cldn34c4	claudin 34C4	2.675707	2.515142	2.220585	2.263758	2.374329	2.469844
17542888	Cldn34d	claudin 34D	2.438324	2.113553	2.141839	1.95381	2.147131	2.381681
17453496	Cldn4	claudin 4	7.748032	8.275463	6.368881	6.60551	7.580694	5.382737
17323755	Cldn5	claudin 5	7.818598	8.598887	7.392386	8.015875	7.150777	7.920492
17334097	Cldn6	claudin 6	3.524161	3.525921	3.17602	3.714711	3.512967	4.279642
17251829	Cldn7	claudin 7	5.022063	4.501263	3.877616	3.856788	7.091545	4.482209
17331828	Cldn8	claudin 8	3.137836	2.718103	3.01699	3.288204	3.06845	3.397421
17341547	Cldn9	claudin 9	9.380339	9.50186	9.448531	9.251453	8.797905	10.72689
17326438	Cldnd1	claudin domain containing 1	87.90784	63.75029	79.46182	75.23906	76.11137	76.4858
17477109	Cldnd2	claudin domain containing 2	4.326709	4.089657	4.792007	4.507727	5.270756	3.674251
17411697	Clvs1	clavesin 1	6.595765	5.653718	4.485815	4.813179	4.949319	6.607067
17240123	Clvs2	clavesin 2	2.609548	2.34907	2.431641	2.677506	2.398463	2.990009
17493331	Pcf11	cleavage and polyadenylation factor subunit homolog (S. cerevisiae)	39.23991	52.1527	43.97282	41.21735	40.33136	55.01831
17318548	Cpsf1	cleavage and polyadenylation specific factor 1	77.18775	71.52918	65.50259	75.67894	62.20333	67.88496
17278012	Cpsf2	cleavage and polyadenylation specific factor 2	36.62533	32.61062	28.68292	43.66726	32.93295	24.61695
17422764	Cpsf3l	cleavage and polyadenylation specific factor 3-like	28.31314	22.24285	21.51901	23.02405	21.73458	22.97049
17444640	Cpsf4	cleavage and polyadenylation specific factor 4	38.89573	35.66532	37.36848	37.28853	40.46605	31.19969
17271607	Cpsf4l	cleavage and polyadenylation specific factor 4-like	16.9836	16.27708	14.68814	24.51393	14.8289	15.94619
17245237	Cpsf6	cleavage and polyadenylation specific factor 6	69.3104	78.13137	72.37051	69.16115	67.62946	64.65141
17357502	Cpsf7	cleavage and polyadenylation specific factor 7	33.12687	35.95075	28.13239	28.30412	19.75355	28.30412
17274448	Cpsf3	cleavage and polyadenylation specificity factor 3	74.14975	61.64791	64.44292	57.02015	64.78824	64.12922
17537607	Cstf2	cleavage stimulation factor, 3 pre-RNA subunit 2	19.42873	17.58837	19.40666	16.80513	18.43416	19.09761
17537630	Cstf2	cleavage stimulation factor, 3 pre-RNA subunit 2	3.062691	3.062691	2.630578	2.841849	3.298637	3.286359
17358660	Cstf2t	cleavage stimulation factor, 3 pre-RNA subunit 2, tau	17.3306	20.77663	13.59251	18.49649	20.24495	18.9628
17380108	Cstf1	cleavage stimulation factor, 3 pre-RNA, subunit 1	33.36099	35.13653	32.7091	50.93657	34.7572	28.27823
17373857	Cstf3	cleavage stimulation factor, 3 pre-RNA, subunit 3	29.38382	20.22854	20.22854	17.36523	20.22854	18.87625
17487346	Clptm1	cleft lip and palate associated transmembrane protein 1	213.4124	217.8045	211.7312	209.1987	198.6135	210.3036
17216574	Clasp1	CLIP associating protein 1	49.07342	40.09249	49.57325	41.43649	37.7436	34.33766
17522651	Clasp2	CLIP associating protein 2	28.45657	28.90846	30.98719	30.61231	27.65886	30.52776
17487301	Clasrp	CLK4-associating serine/arginine rich protein	16.84735	20.61896	22.16129	24.22559	28.82143	23.67644
17277552	Cipc	CLOCK interacting protein, circadian	30.03911	34.80976	34.36957	35.46097	27.57617	35.36328
17387504	Clp1	CLP1, cleavage and polyadenylation factor I subunit	19.42873	19.06719	19.06719	18.18466	21.38593	19.06719
17480928	Clpb	ClpB caseinolytic peptidase B	56.47811	55.86412	61.99113	55.02785	55.63497	56.07089
17288501	Clptm1l	CLPTM1-like	159.6889	151.0477	136.9182	140.3728	135.9347	128.969
17252767	Cluh	clustered mitochondria (cluA/CLU1) homolog	308.3211	311.7509	286.8137	273.3129	258.5644	273.4134
17301452	Clu	clusterin	767.7485	749.9705	776.8488	705.7679	779.5998	722.438
17322488	Cluap1	clusterin associated protein 1	12.60383	10.07208	11.20554	12.63646	10.05722	11.91164
17505967	Cmip	c-Maf inducing protein	89.8197	98.40707	132.3368	89.88821	112.1908	85.56625
17375997	Mertk	c-mer proto-oncogene tyrosine kinase	55.26484	49.0299	59.82929	57.08936	47.76957	59.27123
17331148	Cmss1	cms small ribosomal subunit 1	11.26722	18.49649	21.97315	21.67305	17.25306	20.29595
17250824	Cdrt4	CMT1A duplicated region transcript 4	3.839238	4.580547	4.60918	4.271906	5.343778	5.17443
17264097	Cdrt4os1	CMT1A duplicated region transcript 4, opposite strand 1	4.29998	3.907592	4.386803	4.370263	4.506977	3.89861
17264094	Cdrt4os2	CMT1A duplicated region transcript 4, opposite strand 2	4.595048	3.073537	3.615287	4.001192	3.949828	4.79028
17418241	Mycbp	c-myc binding protein	61.26198	53.47194	48.0815	54.00159	64.05104	51.95245
17355825	Cndp2	CNDP dipeptidase 2 (metallopeptidase M20 family)	61.62142	54.22589	57.08105	62.50631	82.25207	62.44006
17239023	Cnksr3	Cnksr family member 3	28.64676	31.07689	29.00581	19.30777	15.86593	26.3858
17515248	Carm1	coactivator-associated arginine methyltransferase 1	44.77092	53.10295	58.62605	48.32507	39.96319	50.55178
17513491	Cotl1	coactosin-like 1 (Dictyostelium)	26.31257	26.72615	30.68348	27.51888	27.12968	28.95398
17275378	Coch	coagulation factor C homolog (Limulus polyphemus)	4.581373	4.581373	5.13317	4.882635	4.041974	4.581373
17388234	F2	coagulation factor II	1430.814	1330.959	1394.943	1292.833	1661.615	1533.614
17295130	F2r	coagulation factor II (thrombin) receptor	104.9916	121.0719	129.0933	108.2933	95.90606	75.19371
17295125	F2rl1	coagulation factor II (thrombin) receptor-like 1	4.03657	4.415693	4.792638	4.024418	4.708867	4.453563
17289281	F2rl2	coagulation factor II (thrombin) receptor-like 2	4.522373	4.51783	7.783883	5.143641	2.796437	4.370838
17502521	F2rl3	coagulation factor II (thrombin) receptor-like 3	6.566707	5.482637	7.229771	5.250555	6.301521	10.31856
17402181	F3	coagulation factor III	10.82519	9.234667	9.32782	11.50086	9.523224	11.08172
17535098	F9	coagulation factor IX	695.2847	614.0618	571.0368	620.2689	699.0733	601.9542
17218861	F5	coagulation factor V	720.4278	667.9147	608.41	583.4964	747.7437	690.1805
17499212	F7	coagulation factor VII	211.1523	196.3998	216.2431	207.9467	206.433	183.1231
17542781	F8	coagulation factor VIII	89.35397	81.49107	115.7253	81.46622	51.8298	60.39428
17499224	F10	coagulation factor X	447.7843	380.7288	405.9618	377.6589	475.1239	379.7773
17509035	F11	coagulation factor XI	108.6587	51.24005	45.67934	102.0965	173.3015	99.60526
17292855	F12	coagulation factor XII (Hageman factor)	695.4245	676.0518	626.8081	658.345	745.8905	626.2262
17291881	F13a1	coagulation factor XIII, A1 subunit	6.275107	6.174747	5.979356	5.407269	6.431254	5.56106
17217946	F13b	coagulation factor XIII, beta subunit	271.8619	223.8875	212.6136	259.6456	320.8844	300.9834
17219448	Copa	coatomer protein complex subunit alpha	315.4003	267.6975	255.3124	227.58	237.2707	215.6428
17495271	Copb1	coatomer protein complex, subunit beta 1	238.5636	206.3	160.1045	178.0946	193.378	215.3889
17520638	Copb2	coatomer protein complex, subunit beta 2 (beta prime)	233.2349	213.4997	209.4831	192.302	212.7109	198.4264

17501891	Cope	coatomer protein complex, subunit epsilon	105.6471	98.71723	97.85136	100.1265	117.6706	104.3611
17460572	Copg1	coatomer protein complex, subunit gamma 1	80.33351	74.02753	79.44861	66.87245	62.44266	56.63688
17465557	Copg2	coatomer protein complex, subunit gamma 2	78.20397	61.62014	67.32918	70.75744	61.39503	74.74888
17315558	Copz1	coatomer protein complex, subunit zeta 1	275.5194	281.0221	291.2754	274.0281	293.6492	274.5395
17255663	Copz2	coatomer protein complex, subunit zeta 2	60.97348	72.66355	93.50101	76.46619	68.79062	65.62666
17385879	Cobll1	Cobl-like 1	89.31682	96.03244	95.7805	88.37461	101.2944	100.6798
17363644	Cbwd1	COBW domain containing 1	19.92793	20.89578	18.84774	21.85119	22.36886	23.20349
17256522	Coasy	Coenzyme A synthase	57.96178	56.502	57.74804	63.04412	62.23999	63.91222
17246100	Coq10a	coenzyme Q10 homolog A (yeast)	30.14548	32.69436	33.56487	34.69438	38.60083	40.5753
17212874	Coq10b	coenzyme Q10 homolog B (S. cerevisiae)	64.82148	98.59072	95.37836	65.27642	85.3601	88.7423
17450131	Coq2	coenzyme Q2 homolog, prenyltransferase (yeast)	37.95558	33.08373	37.94637	36.94021	37.96821	41.6594
17412191	Coq3	coenzyme Q3 homolog, methyltransferase (yeast)	50.54302	49.12686	51.37484	55.67316	55.17183	64.23599
17368896	Coq4	coenzyme Q4 homolog (yeast)	41.36016	43.37825	44.41423	45.2216	37.3281	43.41946
17441105	Coq5	coenzyme Q5 homolog, methyltransferase (yeast)	211.0235	169.8856	176.3099	173.5154	116.7938	131.3898
17277232	Coq6	coenzyme Q6 homolog (yeast)	20.15785	19.42873	19.42873	19.42873	19.39362	19.42873
17504146	Coq9	coenzyme Q9 homolog (yeast)	59.65082	66.97682	64.59721	62.95242	57.74924	62.71028
17356472	Cfl1	cofilin 1, non-muscle	117.5556	174.6896	187.7267	130.9589	150.3499	137.5002
17281142	Cfl2	cofilin 2, muscle	244.5338	188.2088	198.4071	203.5614	207.1842	237.6064
17353054	Ccdc178	coiled coil domain containing 178	3.187179	3.334961	3.417923	3.703322	3.417923	3.102626
17430475	Ccdc28b	coiled coil domain containing 28B	11.80888	9.628159	11.75213	12.86483	11.65696	10.50701
17248019	Ccdc88a	coiled coil domain containing 88A	15.10569	12.89956	12.99919	12.99919	12.99919	14.41671
17337169	Cchcr1	coiled-coil alpha-helical rod protein 1	8.917319	12.27371	12.35557	11.11387	8.297519	10.61226
17511038	Cc2d1a	coiled-coil and C2 domain containing 1A	26.36734	25.1661	24.22458	27.5206	30.14987	26.21644
17416714	Cc2d1b	coiled-coil and C2 domain containing 1B	23.43577	24.79161	27.81151	22.47953	28.13239	23.75781
17437156	Cc2d2a	coiled-coil and C2 domain containing 2A	6.657036	9.231531	8.818663	7.917583	7.294606	11.66052
17359368	Cc2d2b	coiled-coil and C2 domain containing 2B	5.507547	4.72456	6.055936	6.171709	6.227875	5.635525
17511912	Ccdc102a	coiled-coil domain containing 102A	10.99031	13.58017	13.64377	13.86908	16.98112	13.48001
17242558	Ccdc105	coiled-coil domain containing 105	4.25288	4.399013	4.424824	4.755543	4.690267	4.157165
17473463	Ccdc106	coiled-coil domain containing 106	8.963921	9.019641	7.162186	9.322779	8.83702	7.7882
17413339	Ccdc107	coiled-coil domain containing 107	38.48142	36.10358	40.04638	41.50347	47.4567	47.15798
17224397	Ccdc108	coiled-coil domain containing 108	7.152909	7.042463	7.584741	5.677871	6.741876	6.349663
17410332	Ccdc109b	coiled-coil domain containing 109B	7.682575	7.483743	7.815835	8.019098	8.355812	8.135381
17500932	Ccdc110	coiled-coil domain containing 110	2.784735	2.842243	2.6341	3.124099	2.792021	2.775678
17354244	Ccdc112	coiled-coil domain containing 112	5.928101	5.237506	4.72515	5.198985	3.772916	4.757917
17504309	Ccdc113	coiled-coil domain containing 113	5.191747	4.801449	5.582379	5.491651	5.234276	4.722268
17478058	Ccdc114	coiled-coil domain containing 114	5.046664	6.164184	7.118681	6.295801	5.957512	7.106061
17222072	Ccdc115	coiled-coil domain containing 115	20.93101	14.8708	13.74305	15.22732	12.84914	13.9291
17328631	Ccdc116	coiled-coil domain containing 116	6.171709	5.197832	6.171709	6.772789	6.171709	7.170233
17260167	Ccdc117	coiled-coil domain containing 117	81.1568	54.12375	53.82782	53.82782	53.82782	64.33313
17522430	Ccdc12	coiled-coil domain containing 12	15.97451	23.9093	17.49535	19.29988	22.18404	19.60242
17539898	Ccdc120	coiled-coil domain containing 120	10.38385	11.23237	10.88749	9.960858	10.77585	8.855722
17230546	Ccdc121	coiled-coil domain containing 121	7.375496	5.690124	5.73431	4.618614	6.435401	4.461535
17302102	Ccdc122	coiled-coil domain containing 122	14.24032	14.24032	13.39423	13.37697	16.38325	12.09526
17510223	Ccdc124	coiled-coil domain containing 124	47.62773	45.62776	46.167	48.16122	48.215	43.57564
17289497	Ccdc125	coiled-coil domain containing 125	29.73706	31.31973	37.09596	33.61889	31.89437	23.49432
17458465	Ccdc126	coiled-coil domain containing 126	8.827959	8.371174	8.468521	7.815131	8.513309	8.78456
17288641	Ccdc127	coiled-coil domain containing 127	37.1706	36.184	32.93432	36.184	38.89573	35.36107
17458866	Ccdc129	coiled-coil domain containing 129	4.424088	3.271291	3.454364	3.703322	2.911999	3.674047
17532382	Ccdc13	coiled-coil domain containing 13	14.2861	14.68855	14.68855	18.91803	14.68855	13.51622
17511112	Ccdc130	coiled-coil domain containing 130	24.60381	22.22436	28.17942	27.44821	25.1403	24.68769
17313495	Ccdc134	coiled-coil domain containing 134	11.6036	10.98178	9.325041	11.01372	11.56746	10.98178
17456604	Ccdc136	coiled-coil domain containing 136	6.451122	6.478546	6.331205	7.044074	6.053082	6.369632
17259331	Ccdc137	coiled-coil domain containing 137	22.10315	23.03443	24.62787	28.09361	17.15753	23.44389
17233489	Ccdc138	coiled-coil domain containing 138	7.44912	7.435347	7.437151	8.3911	9.738638	9.409643
17325144	Ccdc14	coiled-coil domain containing 14	5.829532	8.477272	6.675704	8.847194	8.247804	8.696712
17387131	Ccdc141	coiled-coil domain containing 141	23.26404	32.36943	41.77188	32.84154	33.99475	30.48125
17459936	Ccdc142	coiled-coil domain containing 142	13.07093	10.58332	10.83247	11.78043	11.7378	15.5462
17468095	Ccdc142os	coiled-coil domain containing 142, opposite strand	6.063034	7.76061	7.479439	6.966094	9.310251	8.242831
17404772	Ccdc144b	coiled-coil domain containing 144B	2.261892	2.488903	2.893288	2.734733	2.3797	2.813001
17445809	Ccdc146	coiled-coil domain containing 146	4.186574	3.553424	3.855799	3.639146	4.43591	4.045197
17385455	Ccdc148	coiled-coil domain containing 148	4.378965	5.142322	4.702442	5.080428	5.573216	5.008088
17448050	Ccdc149	coiled-coil domain containing 149	13.13533	17.33481	12.54717	14.31296	14.31296	11.00548
17525599	Ccdc15	coiled-coil domain containing 15	19.77026	25.41732	23.12498	21.58594	26.58683	32.87525
17212840	Ccdc150	coiled-coil domain containing 150	4.417346	3.928068	4.042506	4.901794	3.642477	4.683607
17524879	Ccdc151	coiled-coil domain containing 151	4.40759	3.960493	4.095501	4.265811	3.714685	4.479103
17315627	Ccdc152	coiled-coil domain containing 152	8.737799	8.622941	5.634509	6.446294	7.138693	7.635962
17516538	Ccdc153	coiled-coil domain containing 153	21.51171	22.62789	19.62077	27.42786	14.36693	21.38282
17334576	Ccdc154	coiled-coil domain containing 154	4.626785	5.459126	5.663995	6.208264	6.015898	5.663485
17490673	Ccdc155	coiled-coil domain containing 155	6.82666	7.400794	6.82666	7.541544	5.767636	6.842673
17259956	Ccdc157	coiled-coil domain containing 157	10.77174	12.53213	12.42445	13.22724	9.71127	10.21141
17449763	Ccdc158	coiled-coil domain containing 158	4.452791	3.698474	3.629523	3.816182	3.31898	3.818537
17515344	Ccdc159	coiled-coil domain containing 159	18.87036	23.36958	36.02409	21.8941	18.88606	17.74782
17534702	Ccdc160	coiled-coil domain containing 160	5.805297	5.791953	7.594474	6.513577	9.462819	6.397685
17240444	Ccdc162	coiled-coil domain containing 162	7.486181	7.252558	7.346057	8.92109	6.96677	9.011892
17240467	Ccdc162	coiled-coil domain containing 162	4.058059	3.070109	2.726424	3.62648	4.452051	4.110945
17417492	Ccdc163	coiled-coil domain containing 163	5.336597	7.460953	6.1315	8.198671	7.536058	7.622953
17318245	Ccdc166	coiled-coil domain containing 166	6.603313	9.69318	7.317039	9.179019	9.908592	6.947626
17343079	Ccdc167	coiled-coil domain containing 167	19.52946	25.31271	24.92169	26.36112	20.48279	27.69435
17222677	Ccdc168	coiled-coil domain containing 168	2.840707	2.461589	2.392833	3.077908	2.802336	2.425328
17397679	Ccdc169	coiled-coil domain containing 169	4.35629	4.489796	4.134778	4.347723	4.506102	4.015585
17417456	Ccdc17	coiled-coil domain containing 17	16.97806	16.3268	18.51612	18.3532	13.94659	13.11549
17231443	Ccdc170	coiled-coil domain containing 170	9.179337	12.93538	10.95236	11.26363	10.50046	9.42544
17415035	Ccdc171	coiled-coil domain containing 171	17.40452	17.05485	16.92894	14.96531	13.73276	19.14134
17360735	Ccdc172	coiled-coil domain containing 172	3.117652	2.846048	2.602179	3.12109	3.031055	3.742131

17386315	Ccdc173	coiled-coil domain containing 173	2.499605	2.679734	2.919234	3.27272	2.882998	2.962202
17460958	Ccdc174	coiled-coil domain containing 174	15.88761	16.83206	16.83206	13.96981	17.12414	17.83612
17281801	Ccdc175	coiled-coil domain containing 175	2.521094	2.420223	2.610362	2.610362	3.06023	2.646068
17282284	Ccdc177	coiled-coil domain containing 177	5.39742	6.397419	5.578975	5.723906	5.191171	5.234711
17440164	Ccdc18	coiled-coil domain containing 18	2.960088	3.309492	3.546092	3.071152	3.538284	3.212241
17413706	Ccdc180	coiled-coil domain containing 180	4.666206	5.086418	4.847329	4.726132	4.956735	5.241755
17218899	Ccdc181	coiled-coil domain containing 181	12.72117	13.4633	11.91949	12.72779	11.53607	13.89294
17254970	Ccdc182	coiled-coil domain containing 182	6.23025	4.7664	5.120306	4.681595	5.922105	4.886969
17382709	Ccdc183	coiled-coil domain containing 183	7.996119	8.283382	7.816539	8.467112	10.64298	9.830399
17314604	Ccdc184	coiled-coil domain containing 184	8.380986	8.955786	8.478271	9.925158	8.647242	9.992391
17230636	Ccdc185	coiled-coil domain containing 185	6.992896	6.29663	4.883888	6.76758	5.9955	6.152446
17365863	Ccdc186	coiled-coil domain containing 186	38.61689	30.75182	28.12361	41.63197	32.68009	25.6076
17382885	Ccdc187	coiled-coil domain containing 187	3.550372	3.234359	3.23649	3.264586	4.005466	3.860506
17382900	Ccdc187	coiled-coil domain containing 187	5.260427	4.979221	4.998274	6.753007	5.530309	6.039671
17496715	Ccdc189	coiled-coil domain containing 189	8.854249	8.438345	6.62408	6.898439	6.153469	7.66226
17219148	Ccdc190	coiled-coil domain containing 190	5.777879	5.754378	5.421041	5.329869	7.005558	5.582456
17325736	Ccdc191	coiled-coil domain containing 191	15.2893	15.95824	16.08507	17.20064	15.95824	15.95824
17539854	Ccdc22	coiled-coil domain containing 22	20.75504	26.77361	24.4603	32.86705	26.36112	32.35664
17428910	Ccdc24	coiled-coil domain containing 24	9.241583	8.316981	8.674677	8.291482	7.697767	11.49194
17301440	Ccdc25	coiled-coil domain containing 25	51.31754	44.82712	28.56685	47.35779	45.58193	40.47502
17433567	Ccdc27	coiled-coil domain containing 27	5.6489	4.316195	5.680114	6.106095	6.810969	5.680114
17239528	Ccdc28a	coiled-coil domain containing 28A	17.67073	26.94956	25.61772	26.85912	23.93583	27.98846
17366536	Ccdc3	coiled-coil domain containing 3	15.83495	22.80455	21.99235	16.23595	14.21981	16.56608
17429383	Ccdc30	coiled-coil domain containing 30	5.758647	5.327726	5.758089	5.679957	5.669769	5.032726
17389795	Ccdc32	coiled-coil domain containing 32	21.27386	24.34275	24.58046	21.28979	32.94734	29.11317
17527630	Ccdc33	coiled-coil domain containing 33	9.800056	9.702456	8.715963	11.05242	9.71504	10.78183
17374121	Ccdc34	coiled-coil domain containing 34	13.4371	18.20837	13.29793	15.63991	18.92944	14.85565
17389138	Ccdc34os	coiled-coil domain containing 34, opposite strand	2.603694	2.529777	2.912625	2.701424	2.656304	2.361331
17531183	Ccdc36	coiled-coil domain containing 36	5.903661	5.293897	5.644556	6.703061	6.030928	6.568391
17236577	Ccdc38	coiled-coil domain containing 38	4.218383	4.034976	4.379937	4.334363	5.061939	4.699151
17404654	Ccdc39	coiled-coil domain containing 39	4.52736	4.075198	3.86072	5.013889	3.292264	4.172668
17258964	Ccdc40	coiled-coil domain containing 40	10.98125	11.47124	11.74032	10.76794	9.696943	13.86629
17251358	Ccdc42	coiled-coil domain containing 42	6.853496	10.65316	10.41167	7.455577	8.437409	8.956096
17264421	Ccdc42os	coiled-coil domain containing 42, opposite strand [Source:MGI Symbol;Acc:MGI:19	3.015715	2.963824	2.963824	3.242598	2.963824	3.161042
17270305	Ccdc43	coiled-coil domain containing 43	25.49178	19.6239	25.11661	23.68743	21.18968	28.62969
17270761	Ccdc47	coiled-coil domain containing 47	62.7068	55.58524	51.60969	47.60397	46.46621	47.16517
17324553	Ccdc50	coiled-coil domain containing 50	66.89563	79.0942	73.12993	62.55442	76.45241	73.39043
17522161	Ccdc51	coiled-coil domain containing 51	6.893181	7.184212	6.265067	8.410549	5.60009	6.473743
17236327	Ccdc53	coiled-coil domain containing 53	58.6545	56.07322	58.30469	57.78648	57.78648	47.93741
17330901	Ccdc54	coiled-coil domain containing 54	5.755535	7.910616	6.220411	6.171709	4.617174	5.306203
17273394	Ccdc57	coiled-coil domain containing 57	8.331175	9.732834	9.650676	9.84622	9.628226	10.38357
17325312	Ccdc58	coiled-coil domain containing 58	34.25517	46.18076	41.68857	44.49619	45.93685	49.89454
17237084	Ccdc59	coiled-coil domain containing 59	20.79335	18.26007	19.605	19.605	19.48389	20.46576
17234116	Ccdc6	coiled-coil domain containing 6	70.95439	77.3747	76.91857	78.79379	72.16813	63.9725
17451791	Ccdc60	coiled-coil domain containing 60	5.326545	5.752942	5.169125	5.927854	6.346935	5.774756
17487120	Ccdc61	coiled-coil domain containing 61	14.84577	15.14416	14.84577	13.97213	14.23924	17.26681
17442413	Ccdc62	coiled-coil domain containing 62	4.650868	4.100216	4.590846	4.524568	4.743954	4.185355
17452343	Ccdc63	coiled-coil domain containing 63	8.122816	7.889916	8.531149	8.467112	8.467112	8.467112
17451748	Ccdc64	coiled-coil domain containing 64	6.979772	7.219856	7.698994	7.331813	8.238148	9.297482
17334080	Ccdc64b	coiled-coil domain containing 64B	10.36336	11.11842	9.118332	10.82871	9.723731	11.24966
17314653	Ccdc65	coiled-coil domain containing 65	3.33473	4.871344	4.921199	4.093429	4.315597	3.626455
17304273	Ccdc66	coiled-coil domain containing 66	22.03416	18.29009	23.04098	25.64454	18.15733	21.36267
17524211	Ccdc67	coiled-coil domain containing 67	4.098369	6.975129	7.370896	5.890785	4.660646	8.116569
17351634	Ccdc68	coiled-coil domain containing 68	5.63615	4.44576	5.044391	5.685944	5.159031	5.319719
17262915	Ccdc69	coiled-coil domain containing 69	11.36858	10.21084	10.72548	10.72548	12.02979	11.07358
17499843	Ccdc70	coiled-coil domain containing 70	5.55817	5.06415	3.811001	4.555344	4.682633	5.56106
17521722	Ccdc71	coiled-coil domain containing 71	21.44189	22.25643	25.50185	22.15991	20.23905	22.4361
17274868	Ccdc71l	coiled-coil domain containing 71 like	22.98722	21.4764	22.23299	21.91581	22.98722	23.43398
17373887	Ccdc73	coiled-coil domain containing 73	3.850832	4.172378	3.291443	4.172378	4.795961	4.246546
17323576	Ccdc74a	coiled-coil domain containing 74A	3.767506	4.733673	4.03783	4.049209	4.479041	4.296584
17470417	Ccdc77	coiled-coil domain containing 77	15.37101	14.60287	15.44802	15.37101	14.46897	15.37101
17334764	Ccdc78	coiled-coil domain containing 78	10.41665	11.93685	10.41665	10.41665	10.41665	10.52932
17512245	Ccdc79	coiled-coil domain containing 79	2.769528	2.719271	2.719271	2.733994	3.263862	2.854741
17514300	Ccdc7a	coiled-coil domain containing 7A	2.411482	2.227399	3.152158	2.968348	2.996794	3.623666
17507038	Ccdc7b	coiled-coil domain containing 7B	3.243564	2.79859	1.989202	2.615216	3.636549	2.624222
17474231	Ccdc8	coiled-coil domain containing 8	8.200944	7.566363	9.926396	9.250235	8.24386	7.192085
17325904	Ccdc80	coiled-coil domain containing 80	26.9016	33.84029	32.25051	29.61795	28.18782	27.69551
17493220	Ccdc81	coiled-coil domain containing 81	4.351009	5.035657	3.986907	3.791661	4.034305	3.576529
17514666	Ccdc82	coiled-coil domain containing 82	5.014897	5.203383	5.338891	5.522456	5.048903	5.926786
17493275	Ccdc83	coiled-coil domain containing 83	5.706911	5.512512	5.918986	6.512539	6.283638	5.905994
17526274	Ccdc84	coiled-coil domain containing 84	19.21339	22.46785	21.82228	16.37531	20.69485	20.70533
17261233	Ccdc85a	coiled-coil domain containing 85A	4.801649	5.227749	4.421972	4.801649	4.801649	4.938661
17361600	Ccdc85b	coiled-coil domain containing 85B	11.45178	12.84638	10.92234	10.00272	11.72755	10.33302
17283897	Ccdc85c	coiled-coil domain containing 85C	20.88029	18.58324	24.02492	26.89787	18.24362	24.57637
17362831	Ccdc86	coiled-coil domain containing 86	11.37228	9.787633	8.534875	9.939891	11.33341	10.90199
17356284	Ccdc87	coiled-coil domain containing 87	4.608221	4.047244	3.630228	3.708819	3.836525	3.263636
17362070	Ccdc88b	coiled-coil domain containing 88B	17.87176	14.00937	14.27768	12.78526	16.09845	16.6039
17283303	Ccdc88c	coiled-coil domain containing 88C	15.65238	18.96516	22.05494	15.38359	14.8098	18.74872
17480127	Ccdc89	coiled-coil domain containing 89	3.174809	4.176198	3.592007	3.194765	2.618626	3.195718
17486874	Ccdc9	coiled-coil domain containing 9	11.03611	10.09823	10.04399	10.78347	9.698422	9.289751
17480192	Ccdc90b	coiled-coil domain containing 90B	13.04712	16.05188	17.35609	16.56504	17.05993	19.86725
17464429	Ccdc91	coiled-coil domain containing 91	27.52728	34.74395	30.16701	32.28406	20.76022	29.16043
17452889	Ccdc92	coiled-coil domain containing 92	11.65729	11.69946	12.77348	11.51626	10.97394	11.45361
17216690	Ccdc93	coiled-coil domain containing 93	26.76564	24.24625	22.42662	18.96306	18.04241	20.04069

17338650	Ccdc94	coiled-coil domain containing 94	48.1442	47.76129	46.50681	49.20728	55.17336	50.01296
17436938	Ccdc96	coiled-coil domain containing 96	14.4789	9.821545	7.667254	9.821545	8.42537	9.821545
17488001	Ccdc97	coiled-coil domain containing 97	35.2485	29.5468	28.78908	29.1382	29.60625	24.80278
17236832	Ccer1	coiled-coil glutamate-rich protein 1	9.285696	9.366954	9.422762	11.37149	12.68587	9.488237
17475916	Ccer2	coiled-coil glutamate-rich protein 2	6.362395	5.172637	10.66757	10.31263	8.956903	7.660932
17459108	Ccser1	coiled-coil serine rich 1	5.300212	5.377032	4.979773	4.719225	4.990035	5.891561
17305116	Ccser2	coiled-coil serine rich 2	136.4331	147.1088	175.973	134.4765	127.8333	124.8074
17297497	Chchd1	coiled-coil-helix-coiled-coil-helix domain containing 1	31.19537	29.33884	28.1929	36.06206	36.46347	44.52982
17234436	Chchd10	coiled-coil-helix-coiled-coil-helix domain containing 10	1070.612	1145.538	1289.736	1262.38	1027.79	1054.315
17453154	Chchd2	coiled-coil-helix-coiled-coil-helix domain containing 2	33.38296	40.4927	38.78186	42.06971	32.46223	39.95378
17465671	Chchd3	coiled-coil-helix-coiled-coil-helix domain containing 3	96.4915	90.76096	91.44485	90.76096	76.52186	101.4743
17469030	Chchd4	coiled-coil-helix-coiled-coil-helix domain containing 4	21.50082	26.00397	25.15913	22.7758	22.2279	22.2279
17376119	Chchd5	coiled-coil-helix-coiled-coil-helix domain containing 5	21.09487	21.09487	20.65974	25.59287	17.51161	19.62023
17468865	Chchd6	coiled-coil-helix-coiled-coil-helix domain containing 6	14.57749	16.92789	19.3732	17.32004	13.6075	14.77903
17411547	Chchd7	coiled-coil-helix-coiled-coil-helix domain containing 7	15.40333	14.77873	14.86111	16.81807	17.84008	15.05457
17255008	Coil	coilin	5.53564	7.364155	5.909393	10.27403	9.224943	7.951075
17235227	Cirbp	cold inducible RNA binding protein	11.47037	14.41381	16.72437	16.35273	16.82728	16.58503
17313418	Csdc2	cold shock domain containing C2, RNA binding	9.720699	11.53415	13.11331	11.72503	11.52081	11.72471
17401094	Csde1	cold shock domain containing E1, RNA binding	417.5919	426.9017	388.6533	399.5715	403.2163	419.6637
17342890	Clps	colipase, pancreatic	5.658462	4.72705	4.808277	5.56106	5.56106	5.56106
17335352	Clpsl2	colipase-like 2	5.767915	6.904227	6.677092	8.309433	6.701899	6.867333
17268177	LOC102640586	collagen alpha-1(II) chain-like	11.36385	9.341084	7.08953	7.204608	9.748904	8.854188
17355113	Ccbe1	collagen and calcium binding EGF domains 1	7.541544	8.402275	11.56746	9.026145	8.608668	9.052649
17502280	Colgalt1	collagen beta(1-O)galactosyltransferase 1	83.76932	98.03806	88.91285	69.91306	84.4368	84.41983
17218233	Colgalt2	collagen beta(1-O)galactosyltransferase 2	5.177731	5.46064	5.061447	5.985119	4.718734	5.846852
17311191	Cthrc1	collagen triple helix repeat containing 1	11.29005	12.32845	10.27104	13.52709	13.3986	8.472748
17255260	Col1a1	collagen, type I, alpha 1	29.73355	46.33531	49.74777	36.69089	33.58348	34.38601
17455801	Col1a2	collagen, type I, alpha 2	24.52396	30.33833	34.29461	27.52213	23.35387	27.19972
17321094	Col2a1	collagen, type II, alpha 1	11.86319	12.63006	11.6505	12.16589	12.91701	11.56521
17212461	Col3a1	collagen, type III, alpha 1	32.14071	39.7921	45.26864	37.22784	37.30249	41.22193
17507377	Col4a1	collagen, type IV, alpha 1	36.15066	36.60604	37.69653	37.12477	34.74877	35.28761
17498962	Col4a2	collagen, type IV, alpha 2	28.18841	28.84341	32.52957	26.36112	33.28592	27.39081
17214771	Col4a3	collagen, type IV, alpha 3	5.746446	6.418917	6.759797	7.921315	6.747813	6.307857
17289304	Col4a3bp	collagen, type IV, alpha 3 (Goodpasture antigen) binding protein	94.02419	95.2766	94.93841	103.1235	72.0267	98.89255
17224882	Col4a4	collagen, type IV, alpha 4	5.088075	5.592642	5.703628	4.452174	5.703628	5.703628
17538222	Col4a5	collagen, type IV, alpha 5	5.76292	6.777579	7.166059	5.549432	6.268803	7.247885
17544969	Col4a6	collagen, type IV, alpha 6	6.750433	7.651698	7.667042	6.992896	6.992896	7.513057
17211458	Col9a1	collagen, type IX, alpha 1	7.904477	7.739498	7.230573	6.765375	8.52902	7.945566
17418059	Col9a2	collagen, type IX, alpha 2	44.16097	44.69	43.17966	46.13405	51.24715	44.16097
17380813	Col9a3	collagen, type IX, alpha 3	13.76402	11.46234	14.68611	14.375	14.84454	13.55647
17368569	Col5a1	collagen, type V, alpha 1	21.77499	22.45819	19.03206	21.48161	20.08574	20.0053
17222719	Col5a2	collagen, type V, alpha 2	11.58238	12.3692	11.23969	12.24016	9.916356	11.56746
17524451	Col5a3	collagen, type V, alpha 3	24.00928	22.08033	22.89229	29.05511	31.00373	27.57617
17242177	Col6a1	collagen, type VI, alpha 1	28.79607	33.47356	40.39262	34.53961	25.52325	20.84197
17242150	Col6a2	collagen, type VI, alpha 2	10.7312	16.19089	20.77591	18.76432	18.11019	17.70666
17225413	Col6a3	collagen, type VI, alpha 3	14.5688	18.35448	17.11169	16.63546	13.77366	13.03646
17530579	Col6a4	collagen, type VI, alpha 4	5.991179	6.100722	5.347298	5.360955	6.738278	6.476211
17530540	Col6a5	collagen, type VI, alpha 5	5.338965	6.095269	7.800789	6.53379	6.051866	6.53379
17530503	Col6a6	collagen, type VI, alpha 6	14.41561	13.80501	13.58855	14.32298	13.73648	13.23613
17522013	Col7a1	collagen, type VII, alpha 1	13.20196	12.21879	11.44289	12.97327	12.6333	12.21879
17331162	Col8a1	collagen, type VIII, alpha 1	12.70743	12.21879	13.77347	14.88885	7.422525	9.856736
17418581	Col8a2	collagen, type VIII, alpha 2	11.52664	9.744377	12.52015	8.978037	11.20857	12.96581
17232529	Col10a1	collagen, type X, alpha 1	4.49643	4.484105	4.795994	4.092975	3.624997	5.329388
17401902	Col11a1	collagen, type XI, alpha 1	4.973391	4.964815	5.056468	4.909956	5.091215	5.041909
17336338	Col11a2	collagen, type XI, alpha 2	16.08496	15.26854	16.20975	16.08496	16.08496	15.92068
17529099	Col12a1	collagen, type XII, alpha 1	7.458782	8.183852	8.284301	7.760018	7.706202	7.308473
17241257	Col13a1	collagen, type XIII, alpha 1	21.3797	18.00892	17.73872	22.22883	17.38173	17.91145
17311551	Col14a1	collagen, type XIV, alpha 1	63.50023	66.6725	56.13739	48.16723	50.18172	54.07313
17221817	Col19a1	collagen, type XIX, alpha 1	6.808892	7.592474	8.081651	8.350774	7.567097	6.918599
17413866	Col15a1	collagen, type XV, alpha 1	17.09864	14.31296	14.31296	14.31296	14.31296	14.2005
17419131	Col16a1	collagen, type XVI, alpha 1	11.78802	13.06568	11.49026	14.74087	12.67734	14.33401
17365511	Col17a1	collagen, type XVII, alpha 1	6.103556	5.684526	6.111049	7.144039	7.011679	7.456094
17242232	Col18a1	collagen, type XVIII, alpha 1	159.8395	153.8307	149.6657	164.7084	212.0734	171.5292
17380911	Col20a1	collagen, type XX, alpha 1	6.171709	7.372838	6.799318	6.171709	5.750152	6.016857
17380915	Col20a1	collagen, type XX, alpha 1	12.35189	13.7921	12.35189	12.65635	12.83632	10.9259
17317739	Col22a1	collagen, type XXII, alpha 1	6.868333	7.703905	7.909574	7.933404	7.527652	7.707484
17249301	Col23a1	collagen, type XXIII, alpha 1	18.76094	22.11112	19.51322	21.8474	20.61482	24.94364
17403363	Col24a1	collagen, type XXIV, alpha 1	10.28115	10.00944	11.44258	12.13061	10.65723	11.65123
17402607	Col25a1	collagen, type XXV, alpha 1	8.099203	8.715721	8.601212	6.502209	9.365331	7.798032
17453776	Col26a1	collagen, type XXVI, alpha 1	13.30522	17.08253	15.13357	15.14553	14.52353	14.17267
17414672	Col27a1	collagen, type XXVII, alpha 1	16.47367	19.42873	19.42873	19.42873	19.51254	19.42873
17464738	Col28a1	collagen, type XXVIII, alpha 1	5.109068	5.724581	5.449032	5.820044	4.352065	6.33801
17304768	Colq	collagen-like tail subunit (single strand of homotrimer) of asymmetric acetylcholin	15.98038	16.29221	13.50339	15.80227	14.46516	14.44802
17436999	Crmp1	collapsin response mediator protein 1	7.118583	7.911548	7.49132	7.117497	7.054432	8.027329
17311504	Colec10	collectin sub-family member 10	37.84314	46.39733	35.37897	37.16802	32.9414	29.58574
17280383	Colec11	collectin sub-family member 11	49.9596	59.17148	58.34269	58.89283	57.83617	60.81604
17348282	Colec12	collectin sub-family member 12	134.7536	94.49198	91.96991	103.1457	114.0537	113.4828
17333186	Cahm	colon adenocarcinoma hypermethylated RNA	6.343284	4.775196	4.746783	5.648391	4.281719	5.720138
17409075	Csf1	colony stimulating factor 1 (macrophage)	19.28838	19.37964	17.10623	19.28838	17.80117	18.6568
17351053	Csf1r	colony stimulating factor 1 receptor	105.7849	126.4392	132.5571	93.35724	101.4904	91.29095
17262816	Csf2	colony stimulating factor 2 (granulocyte-macrophage)	2.784233	2.554003	2.742763	2.901361	2.497959	2.87167
17366170	Csf2ra	colony stimulating factor 2 receptor, alpha, low-affinity (granulocyte-macrophage)	31.62777	35.28272	45.24354	35.28272	31.93596	36.41573
17318950	Csf2rb2	colony stimulating factor 2 receptor, beta 2, low-affinity (granulocyte-macrophage	12.18091	18.49009	23.94048	11.67985	14.86873	11.4792
17312716	Csf2rb	colony stimulating factor 2 receptor, beta, low-affinity (granulocyte-macrophage)	14.37938	14.43811	16.56401	8.592274	11.72487	11.61124

17256129	Csf3	colony stimulating factor 3 (granulocyte)	6.129927	6.287864	6.608166	5.854679	5.944725	6.287864
17418485	Csf3r	colony stimulating factor 3 receptor (granulocyte)	11.56866	11.63138	14.55457	11.56866	11.84273	11.52288
17511630	Crnde	colorectal neoplasia differentially expressed (non-protein coding)	6.326116	7.653661	6.035193	7.013721	6.624493	6.76486
17261016	Commd1	COMM domain containing 1	19.40801	21.66298	22.27819	22.73621	22.27819	23.95193
17350408	Commd10	COMM domain containing 10	14.57749	16.62635	17.90239	20.26994	14.21242	16.49492
17405380	Commd2	COMM domain containing 2	30.72881	31.25576	30.12293	31.12388	37.11988	32.02774
17367278	Commd3	COMM domain containing 3	38.12565	35.8619	36.57941	42.42786	42.42786	45.71767
17527495	Commd4	COMM domain containing 4	54.5312	60.51874	56.067	64.26895	59.6777	54.73835
17312615	Commd5	COMM domain containing 5	6.310087	9.042615	9.174503	8.718803	14.16933	10.18003
17309143	Commd6	COMM domain containing 6	48.1896	42.98406	48.46328	49.26599	49.56947	48.91754
17393076	Commd7	COMM domain containing 7	14.28679	14.80354	13.66165	11.69962	13.55037	12.21312
17448607	Commd8	COMM domain containing 8	31.66726	31.94725	29.70142	30.18814	29.94473	31.21289
17373661	Commd9	COMM domain containing 9	29.18631	26.02308	27.95375	29.68989	28.41538	28.19036
17231301	Cr1l	complement component (3b/4b) receptor 1-like	59.44897	56.7898	62.58261	60.26924	60.26924	60.82785
17265422	C1qbp	complement component 1, q subcomponent binding protein	139.83	145.8044	144.8303	154.3969	134.2343	144.3783
17431619	C1qa	complement component 1, q subcomponent, alpha polypeptide	71.99226	96.92991	95.88811	74.66913	94.4396	75.49086
17431607	C1qb	complement component 1, q subcomponent, beta polypeptide	172.318	223.3527	279.7046	175.8023	187.3614	158.1511
17431612	C1qc	complement component 1, q subcomponent, C chain	47.96101	53.56096	56.5647	47.96101	47.96101	45.92443
17270388	C1ql1	complement component 1, q subcomponent-like 1	9.038542	9.256136	8.378895	11.23673	6.158504	8.951752
17216671	C1ql2	complement component 1, q subcomponent-like 2	5.14967	5.454814	5.837295	6.352833	6.311443	6.49901
17321484	C1ql4	complement component 1, q subcomponent-like 4	3.405484	3.092128	3.141144	2.724006	2.7346	2.571856
17462872	C1ra	complement component 1, r subcomponent A	21.50202	25.47005	26.83679	25.13438	24.70601	21.97011
17462893	C1rb	complement component 1, r subcomponent B	16.85997	22.39788	19.57337	15.57543	27.904	18.23616
17462863	C1rl	complement component 1, r subcomponent-like	241.1722	207.9035	232.8392	214.1859	255.2381	235.5484
17470740	C1s1	complement component 1, s subcomponent 1	7.600372	9.244146	8.039969	8.571395	9.115868	7.844764
17470764	C1s2	complement component 1, s subcomponent 2	3.084186	2.840412	3.448957	2.485231	3.143169	2.948825
17344086	C2	complement component 2 (within H-2S)	94.42782	75.30637	90.89569	85.6707	99.02219	94.42782
17346528	C3	complement component 3	4035.691	4217.376	4339.19	3587.915	4350.363	3672.101
17470616	C3ar1	complement component 3a receptor 1	14.01325	15.50434	17.21399	15.19379	15.50434	10.59587
17226655	C4bp	complement component 4 binding protein	660.6077	514.8684	417.8062	503.0821	701.7921	523.9299
17226669	C4bp-ps1	complement component 4 binding protein, pseudogene 1	2.7808	4.045281	3.253675	3.244666	5.316401	3.589667
17343976	C4a	complement component 4A (Rodgers blood group)	11.05387	11.08502	12.44738	11.87742	13.41635	7.965362
17343918	C4b	complement component 4B (Chido blood group)	1025.641	999.0479	1064.042	883.4961	1177.189	850.3188
17486864	C5ar1	complement component 5a receptor 1	6.73669	7.4485	8.392729	12.73308	6.992896	5.180675
17486859	C5ar2	complement component 5a receptor 2	28.80086	22.11863	33.4156	19.10926	19.56591	18.51586
17309832	C6	complement component 6	268.2343	88.65807	68.49705	181.1122	210.9811	85.35182
17315686	C7	complement component 7	9.606827	12.53248	12.31701	9.606827	7.810311	8.821476
17427729	C8a	complement component 8, alpha polypeptide	671.9826	473.6639	416.8198	426.6059	654.2553	307.0798
17416196	C8b	complement component 8, beta polypeptide	668.35	406.9845	327.0483	558.5141	708.4096	356.7377
17382613	C8g	complement component 8, gamma polypeptide	301.2317	241.691	232.3281	314.2807	299.8277	302.6633
17309965	C9	complement component 9	782.8814	431.2289	381.3759	572.7096	657.474	473.0373
17227696	Cfh	complement component factor h	1679.668	1721.738	1755.663	1282.151	1796.235	1153.027
17402572	Cfi	complement component factor i	994.1984	1001.1	904.5397	799.2926	1194.781	894.9728
17344064	Cfb	complement factor B	739.8403	670.768	632.451	598.132	862.7089	698.8602
17235018	Cfd	complement factor D (adipsin)	13.35039	12.08261	12.52918	14.25504	17.294	15.24252
17227636	Cfhr1	complement factor H-related 1	372.5654	297.9776	248.3395	280.7632	314.1784	330.9042
17227675	Cfhr2	complement factor H-related 2	132.4579	98.61191	90.60571	108.2212	101.3822	106.63
17540521	Cfp	complement factor properdin	63.6655	77.80117	64.39381	61.49768	58.2756	56.17514
17231314	Cr2	complement receptor 2	5.343074	3.443654	3.727117	3.580398	5.302711	3.418136
17450763	Cplx1	complexin 1	16.34684	17.56705	12.10432	18.39638	22.21312	15.27838
17287432	Cplx2	complexin 2	13.15838	14.62313	12.46689	14.84474	14.86595	15.70432
17527542	Cplx3	complexin 3	7.834113	7.820116	6.659851	7.198419	6.30895	5.887071
17355086	Cplx4	complexin 4	4.183238	3.532477	4.183238	4.183238	5.094004	4.691372
17257969	Cog1	component of oligomeric golgi complex 1	20.60054	19.06811	20.02375	19.73767	17.40331	22.95283
17506754	Cog2	component of oligomeric golgi complex 2	30.39285	24.66204	30.39285	31.67143	33.89898	32.48001
17308686	Cog3	component of oligomeric golgi complex 3	53.15413	37.09621	29.69072	40.15339	42.94862	31.70415
17505648	Cog4	component of oligomeric golgi complex 4	105.3963	100.1452	102.4162	113.295	78.12758	104.482
17274841	Cog5	component of oligomeric golgi complex 5	48.86434	45.88498	45.76333	47.27973	43.80125	46.79168
17405174	Cog6	component of oligomeric golgi complex 6	50.77794	43.52583	33.46474	42.66585	38.62786	42.6032
17495869	Cog7	component of oligomeric golgi complex 7	25.22321	31.22154	31.22154	32.29435	27.29832	28.47433
17512696	Cog8	component of oligomeric golgi complex 8	24.89079	20.08491	16.77476	21.85634	16.29537	19.88543
17366278	Csprs	component of Sp100-rs	13.66175	16.23595	21.93252	19.6046	12.49683	20.98025
17366355	Csprs	component of Sp100-rs	4.953334	4.873506	9.13896	6.554837	4.80378	8.336258
17548926	LOC101055758	component of Sp100-rs	6.451524	6.992896	11.1571	9.682436	7.387058	7.076618
17366197	LOC101055663	component of Sp100-rs-like	3.828316	3.384775	4.647678	3.588523	2.541112	4.085011
17366275	LOC102636558	component of Sp100-rs-like	12.1138	7.528122	8.63622	12.06202	8.677022	5.651759
17486771	Crx	cone-rod homeobox	4.16348	4.006993	4.38109	3.776946	3.160998	5.480357
17473999	Crxos	cone-rod homeobox, opposite strand	2.454876	2.588596	3.924149	3.644599	2.910385	2.805251
17390263	Cdan1	congenital dyserythropoietic anemia, type I (human)	12.28529	11.60343	12.28529	13.65502	12.33666	12.28529
17232235	Ctgf	connective tissue growth factor	20.73003	21.93405	26.64624	21.93055	20.56344	20.21592
17431234	Cnksr1	connector enhancer of kinase suppressor of Ras 1	4.478638	4.801649	4.739911	5.726486	6.374976	5.012881
17545712	Cnksr2	connector enhancer of kinase suppressor of Ras 2	2.956807	3.92124	2.928313	4.086285	3.447499	3.066175
17365030	Chuk	conserved helix-loop-helix ubiquitous kinase	154.1093	142.4794	133.9425	161.4888	164.4859	169.7126
17220018	Cnst	consortin, connexin sorting protein	16.57572	19.47485	22.46084	14.96935	15.43646	13.18705
17314387	Cntn1	contactin 1	5.97012	5.384193	4.898058	3.610253	5.200967	5.447786
17226891	Cntn2	contactin 2	6.555246	7.181424	8.42794	7.535379	6.992896	8.48509
17469506	Cntn3	contactin 3	3.198754	3.123449	3.065686	3.12016	3.353482	3.332881
17461271	Cntn4	contactin 4	4.308035	3.920832	4.220167	4.545598	4.172552	3.961756
17523952	Cntn5	contactin 5	3.911224	3.209481	3.987018	3.183425	3.378025	3.961893
17461240	Cntn6	contactin 6	3.043497	2.888639	3.620377	3.229968	3.992715	3.145785
17256579	Cntnap1	contactin associated protein-like 1	18.06957	20.58769	21.57741	23.3712	17.19837	18.62954
17458043	Cntnap2	contactin associated protein-like 2	5.8713	4.97153	5.457007	6.051153	6.890172	6.571032
17293721	Cntnap3	contactin associated protein-like 3	7.851128	8.288494	8.237862	7.851128	8.238776	8.286886
17505790	Cntnap4	contactin associated protein-like 4	3.762991	3.786172	4.116106	3.762991	3.729055	3.887008

17216527	Cntnap5a	contactin associated protein-like 5A	2.676133	3.499745	2.905145	3.990308	3.067706	3.724715
17216246	Cntnap5b	contactin associated protein-like 5B	3.190517	4.043291	3.350253	2.954082	4.011968	3.409074
17339041	Cntnap5c	contactin associated protein-like 5C	8.224227	7.661623	6.782701	6.708034	6.200566	8.013653
17507668	Coprs	coordinator of PRMT5, differentiation stimulator	13.79755	19.41904	18.84226	15.61813	16.64815	13.631
17391018	Cops2	COP9 (constitutive photomorphogenic) homolog, subunit 2 (Arabidopsis thaliana)	175.012	155.4277	136.4823	148.2009	153.7327	156.3613
17263482	Cops3	COP9 (constitutive photomorphogenic) homolog, subunit 3 (Arabidopsis thaliana)	46.01206	37.24049	36.13738	40.93832	42.66437	44.04145
17439590	Cops4	COP9 (constitutive photomorphogenic) homolog, subunit 4 (Arabidopsis thaliana)	48.78109	48.19194	41.04402	41.80953	40.94598	49.08329
17221215	Cops5	COP9 (constitutive photomorphogenic) homolog, subunit 5 (Arabidopsis thaliana)	106.4727	92.423	80.83398	93.81716	89.29268	87.46537
17443752	Cops6	COP9 (constitutive photomorphogenic) homolog, subunit 6 (Arabidopsis thaliana)	215.7879	226.931	192.5421	199.1001	181.6352	192.5421
17470994	Cops7a	COP9 (constitutive photomorphogenic) homolog, subunit 7a (Arabidopsis thaliana	43.98135	51.95858	49.32681	42.87575	42.50469	44.36623
17215123	Cops7b	COP9 (constitutive photomorphogenic) homolog, subunit 7b (Arabidopsis thaliana	17.63415	27.14266	30.29484	34.97543	29.75561	28.35321
17215612	Cops8	COP9 (constitutive photomorphogenic) homolog, subunit 8 (Arabidopsis thaliana)	40.47474	46.12765	47.58055	38.56473	39.02861	44.85199
17461581	Cpne9	copine family member IX	4.198343	4.948564	5.553895	6.024452	5.911073	6.347815
17504074	Cpne2	copine II	65.72772	58.86263	63.50067	41.58583	39.1489	38.78428
17423525	Cpne3	copine III	125.351	104.889	103.4321	103.0285	95.24424	116.3922
17521043	Cpne4	copine IV	3.650388	3.609252	3.481189	3.62726	3.978956	3.715509
17342999	Cpne5	copine V	4.065887	4.252909	4.744908	4.542605	7.142752	4.216483
17300484	Cpne6	copine VI	12.61711	14.15667	16.77708	17.46543	15.50434	17.86966
17506512	Cpne7	copine VII	17.66081	12.85796	10.78258	12.71359	13.5091	14.81647
17320583	Cpne8	copine VIII	14.52554	15.69203	15.43839	12.19967	14.7328	12.96419
17361352	Ccs	copper chaperone for superoxide dismutase	203.2103	262.1393	241.2374	247.9216	257.727	210.5166
17326428	Cpox	coproporphyrinogen oxidase	467.5932	415.9914	373.5325	427.3783	362.8753	419.5794
17260699	Cobl	cordon-bleu WH2 repeat	23.29454	24.22677	21.23938	24.01893	24.43302	20.32847
17455962	C1galt1	core 1 synthase, glycoprotein-N-acetylgalactosamine 3-beta-galactosyltransferase,	156.2822	144.9941	135.2852	115.6988	137.4116	136.1535
17504649	Cbfb	core binding factor beta	23.24406	27.71605	31.86498	27.70895	31.12863	24.3387
17378216	Cbfa2t2	core-binding factor, runt domain, alpha subunit 2, translocated to, 2 (human)	13.13787	22.99662	19.61302	24.77855	17.795	20.13565
17513806	Cbfa2t3	core-binding factor, runt domain, alpha subunit 2, translocated to, 3 (human)	15.16097	11.22552	13.85601	14.6282	14.50864	13.99112
17386499	Cir1	corepressor interacting with RBPJ, 1	16.82576	18.90636	16.23336	19.49537	16.62693	24.36114
17448614	Corin	corin	18.64517	22.10177	16.70688	19.47957	24.65879	18.81668
17337197	Cdsn	corneodesmosin	7.018487	5.872277	7.417639	5.901616	4.383399	6.925556
17305719	Cnih1	cornichon homolog 1 (Drosophila)	48.43507	44.15118	43.3494	48.43507	44.76316	56.03204
17361454	Cnih2	cornichon homolog 2 (Drosophila)	6.517642	5.403185	5.213709	4.780826	7.547401	4.382275
17220268	Cnih3	cornichon homolog 3 (Drosophila)	3.388578	4.59126	4.277447	4.587029	6.403719	4.561916
17220255	Cnih4	cornichon homolog 4 (Drosophila)	28.39844	34.57099	33.4441	35.90991	31.64839	34.09102
17487919	Cnfn	cornifelin	13.50976	15.54717	15.38999	19.36259	11.1178	12.28529
17399936	Crnn	cornulin	3.610378	3.729985	3.328749	4.028911	3.419511	5.175901
17253258	Coro6	coronin 6	9.232555	9.948852	9.569277	10.30212	11.42276	10.71203
17327765	Coro7	coronin 7	28.75418	47.7282	41.06707	35.55474	41.11805	40.39039
17496376	Coro1a	coronin, actin binding protein 1A	17.54831	23.67758	17.11857	16.25881	14.10476	17.59923
17356160	Coro1b	coronin, actin binding protein 1B	86.34436	99.81052	84.28944	97.98575	93.29449	104.5624
17451443	Coro1c	coronin, actin binding protein 1C	84.58442	88.54813	80.16552	68.99883	71.65473	70.59235
17425058	Coro2a	coronin, actin binding protein 2A	10.1503	11.16383	13.842	10.9881	13.19482	11.50086
17527996	Coro2b	coronin, actin binding protein, 2B	6.563476	7.629614	12.75685	11.18939	8.625033	9.158872
17498398	Cttn	cortactin	64.57796	62.53968	62.57784	55.62803	57.29431	60.7461
17465006	Cttnbp2	cortactin binding protein 2	5.05356	4.491944	4.389571	4.837796	5.150313	4.285044
17507258	Ctxn1	cortexin 1	12.7307	13.04486	12.72726	12.94955	13.88832	14.95888
17375603	Ctxn2	cortexin 2	4.012886	3.819066	3.183845	3.821026	3.269931	3.974794
17350806	Ctxn3	cortexin 3	5.663328	6.22835	6.550568	5.821133	5.487998	5.995084
17404296	Crh	corticotropin releasing hormone	11.70903	14.55588	13.34262	13.29471	11.83403	10.01257
17295112	Crhbp	corticotropin releasing hormone binding protein	4.774634	5.59524	5.067731	5.302453	5.839926	5.996497
17257175	Crhr1	corticotropin releasing hormone receptor 1	8.269901	9.266214	8.991925	8.246489	8.805714	6.452597
17467040	Crhr2	corticotropin releasing hormone receptor 2	7.261663	7.872763	7.261663	8.571514	7.603955	8.138822
17433023	Cort	cortistatin	5.051669	6.332741	3.761088	4.226871	4.717524	5.120696
17532030	Cmc1	COX assembly mitochondrial protein 1	39.05702	34.54057	31.98426	39.18392	39.87659	40.63948
17513311	Cmc2	COX assembly mitochondrial protein 2	27.73835	22.57087	27.64008	32.41164	20.89839	22.80408
17219978	Cox20	COX20 Cox2 chaperone	67.32265	119.1901	110.0969	101.1737	94.54767	102.4077
17326711	Cxadr	coxsackie virus and adenovirus receptor	86.99135	68.92522	75.18432	62.567	57.71562	59.75759
17537338	Cpxcr1	CPX chromosome region, candidate 1	3.476053	3.255728	2.739628	2.831644	2.696821	2.70791
17513166	Cfdp1	craniofacial development protein 1	53.89838	52.42453	49.10881	48.47907	62.1275	58.42484
17219623	Crp	C-reactive protein, pentraxin-related	962.8852	878.1539	930.3889	877.0711	907.9066	843.5443
17284114	Ckb	creatine kinase, brain	36.02783	36.09432	38.01508	35.65271	30.15991	26.17468
17375257	Ckmt1	creatine kinase, mitochondrial 1, ubiquitous	3.976475	5.883563	5.079512	5.676848	5.03625	4.911794
17294816	Ckmt2	creatine kinase, mitochondrial 2	3.834876	3.275647	3.381258	3.202081	3.102153	3.440266
17474621	Ckm	creatine kinase, muscle	15.11763	15.88728	12.76198	14.1883	13.76602	14.44612
17327681	Crebbp	CREB binding protein	167.4945	198.0636	194.8162	179.0576	191.4866	176.9036
17510040	Crtc1	CREB regulated transcription coactivator 1	15.76038	24.28629	22.7515	21.29481	21.29481	18.32435
17399679	Crtc2	CREB regulated transcription coactivator 2	29.05833	43.44806	43.08041	39.07435	36.30081	44.53785
17492644	Crtc3	CREB regulated transcription coactivator 3	35.90095	43.09444	44.63582	36.84278	42.91143	29.90449
17480131	Crebzf	CREB/ATF bZIP transcription factor	46.02099	46.85984	31.86035	43.86505	43.86505	52.10392
17334956	Crebrf	CREB3 regulatory factor	34.61439	39.81196	31.71536	35.79634	36.28497	31.91295
17211739	Cfc1	cripto, FRL-1, cryptic family 1	8.122929	7.230874	8.937987	10.34965	9.310251	8.51685
17342109	Cramp1l	Crm, cramped-like (Drosophila)	24.38835	23.69942	22.96126	26.16615	23.69942	23.69942
17392452	Crnkl1	Crn, crooked neck-like 1 (Drosophila)	23.0504	24.62633	18.91239	23.0891	20.43259	20.94131
17227616	Crb1	crumbs family member 1, photoreceptor morphogenesis associated	4.024139	4.657933	4.865877	3.648415	6.863764	4.886969
17370420	Crb2	crumbs family member 2	6.886639	8.098137	6.71236	5.952353	7.235837	7.575652
17338946	Crb3	crumbs family member 3	26.94284	30.02537	29.53595	28.69067	25.01203	30.41329
17243644	Cry1	cryptochrome 1 (photolyase-like)	32.12557	15.18442	16.66431	25.57584	16.34265	13.77843
17388389	Cry2	cryptochrome 2 (photolyase-like)	20.20457	29.48236	43.76331	34.5353	35.19381	29.394
17335893	Cryaa	crystallin, alpha A	6.746129	8.164984	8.427356	8.454855	8.675759	10.99069
17517132	Cryab	crystallin, alpha B	13.34604	11.20663	14.68163	11.52944	13.19482	14.65966
17266289	Cryba1	crystallin, beta A1	4.389602	3.689128	4.545409	4.851127	3.397421	4.505571
17224386	Cryba2	crystallin, beta A2	5.821819	7.02535	5.625534	6.030928	5.989851	7.019752
17451195	Cryba4	crystallin, beta A4	9.196341	6.464505	8.276495	7.439883	9.232619	6.967398
17440658	Crybb1	crystallin, beta B1	13.8736	14.51849	15.43742	12.79244	15.66073	14.11004

17451323	Crybb2	crystallin, beta B2	29.30754	33.73256	34.02162	31.42694	30.2793	28.13317
17451334	Crybb3	crystallin, beta B3	17.5746	26.37629	21.84589	19.42873	19.42873	20.05834
17223848	Cryga	crystallin, gamma A	3.290507	3.707303	3.350392	4.146689	3.544642	4.886969
17223844	Crygb	crystallin, gamma B	2.834688	2.953407	2.236186	2.859732	2.487954	2.67402
17223839	Crygc	crystallin, gamma C	3.168785	3.45264	3.181352	3.591882	3.758013	3.542358
17223834	Crygd	crystallin, gamma D	2.406006	2.202222	2.135864	2.054176	2.377771	2.285991
17223828	Cryge	crystallin, gamma E	3.291602	2.708699	2.431506	3.082819	2.87677	2.183511
17213765	Crygf	crystallin, gamma F	2.074236	1.898013	2.255364	2.956397	2.652587	2.397549
17446300	Crygn	crystallin, gamma N	12.2006	10.03355	10.19246	10.27937	10.92734	14.62363
17329345	Crygs	crystallin, gamma S	5.545894	6.171709	6.571078	5.425703	6.176588	6.138516
17307134	Cryl1	crystallin, lambda 1	32.46313	33.0308	26.47264	36.02084	23.49741	26.44586
17495790	Crym	crystallin, mu	11.22357	13.2613	14.09127	15.43475	12.43375	12.31633
17403775	Cryz	crystallin, zeta	99.86934	121.1819	115.3984	106.5931	108.974	100.9873
17332181	Cryzl1	crystallin, zeta (quinone reductase)-like 1	16.36612	18.17269	16.10269	15.96444	16.92132	16.36612
17369256	Cstad	CSA-conditional, T cell activation-dependent protein	11.46123	8.152938	8.615891	8.189753	8.361142	8.842719
17527565	Csk	c-src tyrosine kinase	66.15233	57.77206	73.46932	67.44502	64.55827	68.23831
17275724	Ctage5	CTAGE family, member 5	354.362	428.0009	388.912	379.6773	376.1984	406.9224
17355646	Ctdp1	CTD (carboxy-terminal domain, RNA polymerase II, polypeptide A) phosphatase, s	17.17538	20.80877	21.48027	17.4184	16.49801	17.89147
17214220	Ctdsp1	CTD (carboxy-terminal domain, RNA polymerase II, polypeptide A) small phosphat	161.3724	148.3879	129.7284	163.4596	191.6699	169.9481
17237835	Ctdsp2	CTD (carboxy-terminal domain, RNA polymerase II, polypeptide A) small phosphat	40.25595	49.94056	48.33881	49.32339	44.44626	49.46239
17375341	Ctdspl2	CTD (carboxy-terminal domain, RNA polymerase II, polypeptide A) small phosphat	36.35142	47.52353	36.79582	37.96847	38.0636	44.86163
17522958	Ctdspl	CTD (carboxy-terminal domain, RNA polymerase II, polypeptide A) small phosphat	24.34089	23.68218	23.8279	23.49497	19.05886	26.77306
17251840	Ctdnep1	CTD nuclear envelope phosphatase 1	21.96904	23.16669	27.3779	20.87537	21.23335	25.41344
17503559	Cnep1r1	CTD nuclear envelope phosphatase 1 regulatory subunit 1	21.37304	21.37304	19.42887	21.12486	18.49649	21.25529
17447089	Ctbp1	C-terminal binding protein 1	92.87832	80.02784	89.62753	81.01291	71.9364	81.87551
17497153	Ctbp2	C-terminal binding protein 2	8.127096	7.645919	7.987256	10.0976	8.770141	8.042812
17342308	Chtf18	CTF18, chromosome transmission fidelity factor 18	7.923347	6.208221	8.448235	7.17247	6.992896	6.379529
17512680	Chtf8	CTF8, chromosome transmission fidelity factor 8	90.28159	102.9001	119.233	98.89667	104.9734	96.83925
17481801	Ctr9	Ctr9, Paf1/RNA polymerase II complex component, homolog (S. cerevisiae)	49.23628	42.55952	42.12136	49.90284	42.2026	37.85652
17251454	Ctc1	CTS telomere maintenance complex component 1	47.02448	36.74688	36.5376	38.96265	29.16103	30.9698
17408813	Cttnbp2nl	CTTNBP2 N-terminal like	10.00292	13.09605	21.02553	10.18116	12.72876	12.57625
17471528	Clec1a	C-type lectin domain family 1, member a	8.473688	7.248337	7.774608	6.800166	7.566311	6.59828
17463520	Clec1b	C-type lectin domain family 1, member b	36.28798	38.33021	47.47612	36.33656	38.81628	38.62786
17251978	Clec10a	C-type lectin domain family 10, member A	7.627657	4.801649	6.287864	5.48933	6.77617	5.48933
17490211	Clec11a	C-type lectin domain family 11, member a	5.138688	5.803809	4.688837	4.629704	5.535716	4.722596
17463509	Clec12a	C-type lectin domain family 12, member a	25.20137	21.069	23.2476	24.90787	24.25079	23.33235
17471518	Clec12b	C-type lectin domain family 12, member B	3.067642	2.855612	2.722307	2.656267	2.79088	2.423631
17281350	Clec14a	C-type lectin domain family 14, member a	28.70142	25.48065	32.4584	32.18485	19.77424	28.36952
17322874	Clec16a	C-type lectin domain family 16, member A	20.44576	21.7777	22.42786	22.42786	19.39308	22.42786
17513010	Clec18a	C-type lectin domain family 18, member A	12.53231	16.48292	16.52937	17.58228	16.69403	16.70491
17463501	Clec2d	C-type lectin domain family 2, member d	161.6949	268.1785	355.8289	282.914	308.9201	373.4574
17471484	Clec2e	C-type lectin domain family 2, member e	3.80435	4.068085	3.241137	3.164922	4.558597	3.119338
17463465	Clec2g	C-type lectin domain family 2, member g	2.755664	2.96843	2.337943	3.403479	2.352313	3.509292
17463443	Clec2h	C-type lectin domain family 2, member h	18.60102	10.02555	7.199317	9.740528	15.48597	12.12767
17463454	Clec2i	C-type lectin domain family 2, member i	7.889096	10.29356	8.966718	13.26691	10.96429	14.63986
17471440	Clec2j	C-type lectin domain family 2, member J	2.739818	3.512042	3.01089	2.938052	3.084443	3.464963
17457465	Clec2l	C-type lectin domain family 2, member L	3.680113	4.149622	4.497147	5.754498	5.189552	3.004719
17505877	Clec3a	C-type lectin domain family 3, member a	6.030928	5.508655	5.563335	6.700599	6.030928	6.700599
17523566	Clec3b	C-type lectin domain family 3, member b	9.315931	7.086337	9.053716	8.717896	7.429062	7.703105
17462729	Clec4a1	C-type lectin domain family 4, member a1	11.92081	10.99846	12.53708	12.57329	12.52901	9.622488
17462764	Clec4a2	C-type lectin domain family 4, member a2	4.357015	3.749063	3.983592	3.516231	4.227457	3.729055
17462738	Clec4a3	C-type lectin domain family 4, member a3	13.57274	13.69009	16.77336	9.388209	17.00103	14.69935
17462748	Clec4a4	C-type lectin domain family 4, member a4	2.756829	2.323484	2.536167	2.466901	2.845634	2.539246
17462756	Clec4b1	C-type lectin domain family 4, member b1	2.829957	2.540742	2.329451	2.452206	2.709563	2.519522
17462778	Clec4b2	C-type lectin domain family 4, member b2	7.613554	5.547047	4.364632	5.276386	6.574084	5.850541
17462796	Clec4d	C-type lectin domain family 4, member d	4.599063	4.003939	4.778408	3.703322	4.910534	5.173892
17470627	Clec4e	C-type lectin domain family 4, member e	2.681983	3.416171	3.160078	2.622986	2.965592	4.114907
17468209	Clec4f	C-type lectin domain family 4, member f	145.1147	185.1897	200.1767	173.1442	194.4048	172.3503
17507148	Clec4g	C-type lectin domain family 4, member g	186.0724	123.7187	161.2583	149.1386	58.36251	91.82149
17462788	Clec4n	C-type lectin domain family 4, member n	12.09048	21.82788	22.28977	14.31296	17.4445	10.29128
17466228	Clec5a	C-type lectin domain family 5, member a	3.232947	2.968265	3.078569	3.035218	3.262188	3.199441
17471541	Clec7a	C-type lectin domain family 7, member a	19.39577	26.82228	22.09886	19.14519	16.90408	19.8334
17463530	Clec9a	C-type lectin domain family 9, member a	6.817062	7.101383	7.794843	8.575852	7.524157	9.615221
17507708	Csmd1	CUB and Sushi multiple domains 1	4.927036	4.935376	4.131742	4.870399	4.981845	5.496792
17418767	Csmd2	CUB and Sushi multiple domains 2	6.904132	7.008764	7.137258	7.145376	6.977112	7.008764
17430247	Csmd2os	CUB and Sushi multiple domains 2, opposite strand	2.751389	2.536765	2.456152	2.490163	2.879384	2.708117
17316878	Csmd3	CUB and Sushi multiple domains 3	3.094765	3.263862	3.094765	3.183889	3.003594	3.094765
17497017	Cuzd1	CUB and zona pellucida-like domains 1	4.265456	4.649353	5.356498	5.854516	4.234554	4.852641
17532472	Cdcp1	CUB domain containing protein 1	12.61921	14.91301	14.56264	16.58768	13.31675	13.5004
17416415	Cdcp2	CUB domain containing protein 2	5.867557	5.257985	5.260245	5.810289	4.590528	5.309257
17381791	Cubn	cubilin (intrinsic factor-cobalamin receptor)	3.250654	3.274898	3.274898	3.33732	3.335308	3.443272
17254948	Cuedc1	CUE domain containing 1	5.611942	6.609128	6.819377	6.995223	4.822228	6.15987
17365369	Cuedc2	CUE domain containing 2	28.11543	24.96439	28.14916	26.09298	18.55544	24.09714
17373120	Celf1	CUGBP, Elav-like family member 1	94.31792	108.5074	112.2086	110.3452	102.5348	85.96277
17381475	Celf2	CUGBP, Elav-like family member 2	15.83637	14.23164	13.90402	12.26223	10.44333	13.31869
17400103	Celf3	CUGBP, Elav-like family member 3	6.887546	7.626547	8.196057	6.769034	6.62385	5.278361
17353183	Celf4	CUGBP, Elav-like family member 4	7.403975	7.246277	7.74626	7.174508	8.092862	8.317327
17243344	Celf5	CUGBP, Elav-like family member 5	7.069705	8.750467	7.608225	8.857564	7.613079	10.36164
17517964	Celf6	CUGBP, Elav-like family member 6	10.53538	12.42479	11.42071	8.037239	14.68	16.32454
17458074	Cul1	cullin 1	102.2665	104.9938	99.5949	98.10876	102.5654	107.888
17348079	Cul2	cullin 2	85.37726	61.56634	66.82704	92.01199	65.36108	63.35779
17224791	Cul3	cullin 3	200.7283	192.6983	161.8474	188.9643	192.0236	198.4786
17499255	Cul4a	cullin 4A	98.73365	93.49453	96.93528	105.5915	104.0303	95.25481
17541190	Cul4b	cullin 4B	88.089	71.16125	79.79022	86.57749	74.77582	80.87377

17527139	Cul5	cullin 5	45.44911	41.17709	31.32516	36.2739	36.5214	36.72015
17338136	Cul7	cullin 7	13.524	13.27905	13.30171	11.99324	12.45515	13.85467
17345422	Cul9	cullin 9	14.82633	12.98766	15.77393	15.10998	15.10998	15.30775
17245342	Cand1	cullin associated and neddylation disassociated 1	90.31164	71.16125	69.44024	72.88096	67.42772	58.16382
17461978	Cand2	cullin-associated and neddylation-dissociated 2 (putative)	9.70777	14.41211	9.70777	8.532509	9.310251	8.343427
17342664	Cuta	cutA divalent cation tolerance homolog (E. coli)	76.52239	101.4714	105.8018	98.76103	101.7997	98.76103
17370124	Cutal	cutA divalent cation tolerance homolog-like	23.14582	28.41361	30.22143	31.35883	27.92354	33.57929
17359623	Cutc	cutC copper transporter homolog (E.coli)	21.21731	19.00715	21.21731	22.09564	22.02469	24.16723
17453726	Cux1	cut-like homeobox 1	30.0002	30.7254	29.22113	27.16581	25.23913	20.56829
17452311	Cux2	cut-like homeobox 2	12.85556	11.59684	11.30368	11.50604	15.06073	10.43204
17514745	Cwc15	CWC15 homolog (S. cerevisiae)	98.47393	89.20978	86.55348	89.22091	86.08262	90.1609
17387210	Cwc22	CWC22 spliceosome-associated protein homolog (S. cerevisiae)	4.189912	5.470073	6.608304	5.427207	4.399196	5.811417
17268611	Cwc25	CWC25 spliceosome-associated protein homolog (S. cerevisiae)	12.44677	12.85493	13.20617	12.71827	11.88508	11.74488
17295960	Cwc27	CWC27 spliceosome-associated protein homolog (S. cerevisiae)	21.36134	21.318	17.75779	21.83635	20.24004	18.62954
17365054	Cwf19l1	CWF19-like 1, cell cycle control (S. pombe)	25.56114	24.54028	27.86515	28.33769	21.16575	34.28201
17514379	Cwf19l2	CWF19-like 2, cell cycle control (S. pombe)	27.77317	26.82711	27.49448	29.48481	27.94271	32.24783
17516352	Clmp	CXADR-like membrane protein	11.39066	14.05625	12.81001	13.49235	10.49653	11.5023
17351708	Cxxc1	CXXC finger 1 (PHD domain)	44.38899	49.15785	47.76096	41.53887	43.64728	43.63972
17402778	Cxxc4	CXXC finger 4	5.248154	5.189336	5.459883	6.646432	5.313307	5.242337
17349601	Cxxc5	CXXC finger 5	73.82103	88.14031	69.6258	80.73151	86.64352	100.1258
17531751	Arpp21	cyclic AMP-regulated phosphoprotein, 21	3.765053	3.777653	3.749349	3.749349	3.535146	3.700346
17423511	Cnbd1	cyclic nucleotide binding domain containing 1	2.207372	2.406006	2.506527	2.304206	2.171648	2.333944
17378575	Cnbd2	cyclic nucleotide binding domain containing 2	3.767298	3.126807	3.587776	3.653679	3.41009	3.939848
17448682	Cnga1	cyclic nucleotide gated channel alpha 1	3.669593	3.21095	3.289937	3.195829	2.983054	3.295666
17535405	Cnga2	cyclic nucleotide gated channel alpha 2	3.949883	3.136097	3.339703	3.339703	4.062534	3.369326
17211927	Cnga3	cyclic nucleotide gated channel alpha 3	5.770835	5.780603	5.671381	6.171709	7.223259	4.801982
17481369	Cnga4	cyclic nucleotide gated channel alpha 4	3.87888	4.338872	4.320206	4.445667	4.35774	3.562525
17511953	Cngb1	cyclic nucleotide gated channel beta 1	11.40267	13.06568	12.22727	11.11657	10.42106	13.39423
17412047	Cngb3	cyclic nucleotide gated channel beta 3	2.715259	2.953407	3.063031	2.953407	2.749444	3.028514
17405274	Ccna1	cyclin A1	4.688642	6.826329	5.033493	6.11232	3.996397	4.839909
17404821	Ccna2	cyclin A2	4.650159	4.846556	7.351609	5.181249	6.40048	5.56106
17295757	Ccnb1	cyclin B1	4.428291	4.447609	4.447609	5.025893	4.854356	5.025893
17305980	Ccnb1ip1	cyclin B1 interacting protein 1	3.103853	3.103853	3.103853	4.083171	3.430098	2.992331
17528586	Ccnb2	cyclin B2	11.4337	9.272768	11.57749	9.578834	7.81551	8.556139
17539781	Ccnb3	cyclin B3	3.429195	2.727218	2.706935	2.917737	3.150258	3.194345
17412123	Ccnc	cyclin C	26.72226	27.39708	25.30973	27.61232	26.40813	24.32402
17445568	Dmtf1	cyclin D binding myb-like transcription factor 1	27.27959	25.72163	27.92954	28.52945	29.16124	35.69673
17498502	Ccnd1	cyclin D1	64.24846	70.78835	81.57301	72.10213	72.78453	70.19664
17471222	Ccnd2	cyclin D2	44.21089	40.23251	49.62379	34.98368	33.76742	19.12927
17338269	Ccnd3	cyclin D3	33.0537	38.16108	44.74517	32.922	35.04799	32.25252
17375185	Ccndbp1	cyclin D-type binding-protein 1	33.90979	38.29861	36.49457	37.49016	35.63196	32.86637
17489886	Ccne1	cyclin E1	9.775158	14.40173	15.15624	12.72682	13.34576	15.97518
17411761	Ccne2	cyclin E2	5.214106	5.606304	7.419233	5.888499	6.151209	6.565842
17341781	Ccnf	cyclin F	13.09396	14.51407	14.53481	17.51574	13.79267	15.25669
17450770	Gak	cyclin G associated kinase	101.3512	109.7517	98.57022	93.5049	93.66122	89.45685
17261865	Ccng1	cyclin G1	199.3307	189.056	162.3102	159.6203	221.3768	239.5528
17439184	Ccng2	cyclin G2	29.56647	28.00107	34.72493	31.44503	28.5522	35.97019
17288970	Ccnh	cyclin H	40.16119	39.94409	40.16119	43.59528	38.63777	43.55299
17449804	Ccni	cyclin I	256.8638	249.4955	267.7179	262.6195	257.3183	251.6527
17359394	Ccnj	cyclin J	15.25933	12.83045	12.1301	15.92134	13.20534	13.39423
17248621	Ccnjl	cyclin J-like	5.145531	4.323741	4.976047	5.094569	4.291762	4.000665
17278512	Ccnk	cyclin K	85.56032	89.16094	77.15191	82.477	88.55304	96.05308
17405688	Ccnl1	cyclin L1	48.1055	85.41455	60.03658	60.2279	73.10307	82.88735
17422704	Ccnl2	cyclin L2	71.0538	100.7203	90.9001	101.4525	91.04261	93.65019
17359583	Cnnm1	cyclin M1	7.665022	9.971151	10.30648	7.002936	8.727086	7.169189
17360097	Cnnm2	cyclin M2	18.09539	14.98752	17.37812	16.79046	15.72535	14.15265
17211832	Cnnm3	cyclin M3	14.87162	14.6208	18.9217	19.33134	16.44401	19.10383
17211822	Cnnm4	cyclin M4	14.24003	8.470223	13.33661	10.45767	11.47936	9.226286
17256655	Cntd1	cyclin N-terminal domain containing 1	3.729055	3.663493	4.288193	3.729055	3.658976	3.729055
17289971	Ccno	cyclin O	4.990692	4.298162	3.302206	3.718653	3.710902	5.16318
17224462	Cnppd1	cyclin Pas1/PHO80 domain containing 1	42.71349	61.74969	53.97764	56.36118	58.41067	56.36118
17321248	Ccnt1	cyclin T1	30.56122	31.68965	33.02943	27.88795	27.62897	25.52502
17216792	Ccnt2	cyclin T2	24.17493	25.22968	20.02874	21.12999	30.36253	24.94606
17352580	Ccny	cyclin Y	151.3579	142.0948	141.1544	143.9616	148.6381	146.9335
17213687	Ccnyl1	cyclin Y-like 1	7.823694	9.699296	9.578103	10.00001	10.63052	13.23815
17241692	Cdk1	cyclin-dependent kinase 1	9.766624	7.88128	8.467112	7.821363	9.530487	8.467112
17506551	Cdk10	cyclin-dependent kinase 10	30.67221	34.08063	37.84288	37.73444	41.92707	39.10931
17422629	Cdk11b	cyclin-dependent kinase 11B	51.10208	44.97683	53.85394	49.5736	61.69835	47.64589
17255918	Cdk12	cyclin-dependent kinase 12	29.7006	30.08516	28.31766	30.08516	28.11211	29.08594
17290797	Cdk13	cyclin-dependent kinase 13	124.9026	131.6579	125.8665	120.8933	110.19	127.2146
17445329	Cdk14	cyclin-dependent kinase 14	4.384584	6.470423	6.344516	5.576771	5.943901	6.200351
17213246	Cdk15	cyclin-dependent kinase 15	6.846611	6.345791	7.563846	6.815361	6.992896	7.618463
17533640	Cdk16	cyclin-dependent kinase 16	46.91639	45.72528	55.42481	46.41116	48.02787	49.21649
17236502	Cdk17	cyclin-dependent kinase 17	47.82457	46.01269	41.56104	41.47845	34.21886	41.56104
17226847	Cdk18	cyclin-dependent kinase 18	58.71674	63.44392	74.04703	59.48112	60.29264	55.20473
17232734	Cdk19	cyclin-dependent kinase 19	20.80964	25.24752	20.36288	20.71265	15.10736	29.50975
17240376	Cdk19os	cyclin-dependent kinase 19, opposite strand [Source:MGI Symbol;Acc:MGI:1914837	6.711103	5.569663	5.926827	5.918452	5.918083	7.631571
17246299	Cdk2	cyclin-dependent kinase 2	24.27265	24.82772	24.3156	22.09105	24.3848	23.37979
17284039	Cinp	cyclin-dependent kinase 2 interacting protein	35.67867	42.15582	42.29455	41.16767	41.23336	46.59651
17288160	Cdk20	cyclin-dependent kinase 20	14.87275	14.28729	15.88695	14.23697	15.14206	14.96303
17258555	Cdk3-ps	cyclin-dependent kinase 3, pseudogene	6.869523	6.340427	6.402609	6.638098	8.619117	11.54631
17237899	Cdk4	cyclin-dependent kinase 4	56.19656	52.77307	55.23765	50.53707	47.06132	52.45833
17446170	Cdk5	cyclin-dependent kinase 5	45.68093	52.91446	53.21717	54.22777	56.64513	54.22777
17253991	Cdk5r1	cyclin-dependent kinase 5, regulatory subunit 1 (p35)	11.3752	10.38119	7.771052	12.00447	10.14896	10.04795

17214400	Cdk5r2	cyclin-dependent kinase 5, regulatory subunit 2 (p39)	7.870689	10.29513	6.878052	9.503705	8.917937	11.5011
17434297	Cdk6	cyclin-dependent kinase 6	59.05306	69.6147	74.06089	54.90402	47.65481	52.60726
17295718	Cdk7	cyclin-dependent kinase 7	26.02489	19.41944	22.77296	25.39355	24.63077	24.66716
17444713	Cdk8	cyclin-dependent kinase 8	100.2126	112.0959	101.9219	95.1611	83.36847	102.2942
17383965	Cdk9	cyclin-dependent kinase 9 (CDC2-related kinase)	24.4854	29.33152	24.20998	31.86763	27.56107	27.56107
17335467	Cdkn1a	cyclin-dependent kinase inhibitor 1A (P21)	113.0048	86.80282	90.62643	90.60759	160.4656	95.76656
17463718	Cdkn1b	cyclin-dependent kinase inhibitor 1B	29.07586	28.03097	28.73326	23.18179	30.73904	30.86415
17498245	Cdkn1c	cyclin-dependent kinase inhibitor 1C (P57)	6.887259	8.809866	12.16108	9.930181	9.571201	9.749377
17427147	Cdkn2a	cyclin-dependent kinase inhibitor 2A	11.83855	14.27353	14.90764	16.49995	14.27353	15.02799
17427155	Cdkn2b	cyclin-dependent kinase inhibitor 2B (p15, inhibits CDK4)	12.26623	12.35189	13.52597	15.4702	13.01596	10.40814
17428217	Cdkn2c	cyclin-dependent kinase inhibitor 2C (p18, inhibits CDK4)	15.32059	14.47378	15.53877	14.73485	12.37143	17.27256
17524698	Cdkn2d	cyclin-dependent kinase inhibitor 2D (p19, inhibits CDK4)	7.935659	10.16995	11.12852	11.12359	8.067826	10.7283
17299268	Cdkn3	cyclin-dependent kinase inhibitor 3	5.056783	4.785899	4.844138	5.87187	4.402521	5.56106
17281622	Cdkl1	cyclin-dependent kinase-like 1 (CDC2-related kinase)	5.410268	5.754657	5.668944	5.123359	5.259844	4.551935
17449609	Cdkl2	cyclin-dependent kinase-like 2 (CDC2-related kinase)	7.844764	7.698994	7.480476	7.524157	8.421516	8.206801
17249379	Cdkl3	cyclin-dependent kinase-like 3	6.627524	4.801649	5.016009	4.523502	5.468746	4.523502
17249382	Cdkl3	cyclin-dependent kinase-like 3	3.583005	3.773491	4.741488	3.837083	4.165414	3.965108
17347558	Cdkl4	cyclin-dependent kinase-like 4	2.929877	2.768952	2.788327	2.606909	2.706184	4.20927
17545785	Cdkl5	cyclin-dependent kinase-like 5	12.6697	11.50086	11.59917	9.89802	12.48116	12.75782
17500624	LOC102641693	cyclin-related protein FAM58A-like	4.14373	4.460174	2.513347	4.106388	2.458673	6.229213
17537212	Cylc1	cylicin, basic protein of sperm head cytoskeleton 1	2.253442	2.243763	2.553843	2.186889	2.438409	2.438409
17414099	Cylc2	cylicin, basic protein of sperm head cytoskeleton 2	2.67402	2.820127	3.068577	2.93386	2.67402	2.953407
17503681	Cyld	cylindromatosis (turban tumor syndrome)	23.92057	17.20886	21.03456	18.56444	20.19421	25.01289
17411420	Cth	cystathionase (cystathionine gamma-lyase)	767.4559	850.2186	1076.946	1074.858	786.2368	1032.31
17343227	Cbs	cystathionine beta-synthase	560.8146	399.674	360.2289	388.3248	494.2544	491.77
17377425	Cst10	cystatin 10 (chondrocytes)	2.916969	2.366705	2.584795	2.461606	2.756428	2.356589
17392594	Cst11	cystatin 11	2.884897	3.122626	2.880162	3.083161	4.305408	3.201549
17377388	Cst12	cystatin 12	3.109667	3.033283	3.033283	2.682448	3.972838	2.682448
17377405	Cst13	cystatin 13	2.963249	2.875454	3.171972	3.491048	3.503993	3.119533
17377395	Cst8	cystatin 8 (cystatin-related epididymal spermatogenic)	4.01461	3.878261	4.009549	4.132859	4.203468	4.735445
17377412	Cst9	cystatin 9	4.141978	4.122216	3.664662	3.334337	3.616339	3.614309
17330141	Csta1	cystatin A1	4.256359	2.821065	2.961073	3.251826	2.723629	3.409216
17234803	Cstb	cystatin B	36.31038	45.73035	44.34256	47.47052	47.43894	40.2752
17392609	Cst3	cystatin C	117.3977	119.8412	139.1705	119.0943	139.323	135.7266
17361546	Cst6	cystatin E/M	3.254396	2.771525	3.146112	3.190295	3.243969	3.651907
17377464	Cst7	cystatin F (leukocystatin)	6.689276	8.811759	7.611654	6.671865	7.01304	8.806141
17377372	Cstl1	cystatin-like 1	7.502649	7.826569	8.038799	8.794247	7.439522	7.316786
17217610	Csrp1	cysteine and glycine-rich protein 1	20.92071	25.66055	36.59717	24.8291	18.93968	24.44894
17237208	Csrp2	cysteine and glycine-rich protein 2	25.66304	31.31539	35.55129	34.78902	27.16355	36.42785
17491368	Csrp3	cysteine and glycine-rich protein 3	38.75821	54.17217	58.82796	76.62376	96.88155	42.05805
17318650	Cyhr1	cysteine and histidine rich 1	45.00427	44.67637	47.2604	40.23447	47.53209	42.91173
17514871	Chordc1	cysteine and histidine-rich domain (CHORD)-containing, zinc-binding protein 1	125.4527	102.8765	92.98718	89.01274	63.25467	60.10654
17331692	Cyyr1	cysteine and tyrosine-rich protein 1	31.72218	34.26562	41.0133	36.28144	21.49769	27.86534
17331701	Cyyr1	cysteine and tyrosine-rich protein 1	6.155304	4.081671	6.492572	5.521346	6.045158	5.145531
17383588	Ccbl1	cysteine conjugate-beta lyase 1	105.0484	107.8767	97.40879	110.5925	111.1058	73.22782
17403282	Ccbl2	cysteine conjugate-beta lyase 2	467.5867	325.2082	336.7117	323.9798	346.3387	378.2457
17354282	Cdo1	cysteine dioxygenase 1, cytosolic	1309.055	1194.921	1103.675	1510.698	1726.112	1638.602
17279499	Crip2	cysteine rich protein 2	229.8597	173.5659	187.5524	187.8894	172.0304	165.5795
17410974	Cyr61	cysteine rich protein 61	50.2287	54.68677	46.82769	51.71712	35.99638	45.29438
17382453	Cysrt1	cysteine rich tail 1	6.451435	11.30164	8.055309	8.249119	10.03452	8.249119
17339801	Crim1	cysteine rich transmembrane BMP regulator 1 (chordin like)	57.28359	57.16103	57.16103	50.58753	51.93409	49.25506
17320090	Cdpf1	cysteine rich, DPF motif domain containing 1	15.34535	14.94717	11.66238	15.83121	11.72251	14.87399
17322146	Csad	cysteine sulfinic acid decarboxylase	70.46427	77.50621	59.45062	61.47551	66.37923	28.3051
17407496	Crct1	cysteine-rich C-terminal 1	2.997625	3.197956	2.828035	2.883098	2.784407	3.283353
17536961	Chic1	cysteine-rich hydrophobic domain 1	4.176024	3.418753	3.25496	4.501201	4.352065	4.398495
17448905	Chic2	cysteine-rich hydrophobic domain 2	68.0489	69.08353	53.43674	66.92299	70.90277	68.81447
17340232	Cript	cysteine-rich PDZ-binding protein	114.4814	98.35115	90.499	94.25126	92.41403	96.69437
17515517	Cypt10	cysteine-rich perinuclear theca 10	4.25008	4.489174	5.516947	3.049832	4.855938	4.686497
17396192	Cypt12	cysteine-rich perinuclear theca 12	4.223796	4.13538	4.193631	4.223796	4.038473	4.817618
17541229	Cypt14	cysteine-rich perinuclear theca 14	2.637315	2.67402	2.202115	2.912867	2.467449	2.822963
17534372	Cypt15	cysteine-rich perinuclear theca 15	2.173274	2.137701	2.188405	2.072842	2.955967	2.964235
17537034	Cypt2	cysteine-rich perinuclear theca 2	3.310295	2.68031	2.90984	3.463378	3.158282	2.388757
17539023	Cypt3	cysteine-rich perinuclear theca 3	5.195239	6.054173	5.769755	6.180228	4.769077	6.624814
17533469	Cypt8	cysteine-rich perinuclear theca 8	26.84386	19.08252	20.62811	17.54101	17.71034	24.7043
17279509	Crip1	cysteine-rich protein 1 (intestinal)	19.05622	23.42781	26.77584	22.87104	22.87104	23.29406
17338103	Crip3	cysteine-rich protein 3	8.265545	7.118583	7.069166	7.200015	6.632349	7.964148
17344953	Crisp1	cysteine-rich secretory protein 1	4.39365	3.233239	2.6055	3.748673	3.380648	4.832065
17344963	Crisp2	cysteine-rich secretory protein 2	2.972754	2.700469	5.164934	3.742753	3.863665	3.52553
17344943	Crisp3	cysteine-rich secretory protein 3	4.56663	2.717123	2.710746	3.677257	3.161173	2.659804
17221520	Crisp4	cysteine-rich secretory protein 4	3.005094	2.945964	3.005094	3.263862	3.327019	3.339703
17211313	Crispld1	cysteine-rich secretory protein LCCL domain containing 1	4.420716	4.420716	3.977191	5.330349	3.757414	4.476558
17506206	Crispld2	cysteine-rich secretory protein LCCL domain containing 2	11.64009	11.80266	12.10272	10.6289	11.12775	11.30446
17349634	Cystm1	cysteine-rich transmembrane module containing 1	22.0166	20.70533	19.21272	23.21797	20.24818	25.93485
17461725	Creld1	cysteine-rich with EGF-like domains 1	60.34992	53.24632	46.27336	69.62483	46.11774	54.77289
17313998	Creld2	cysteine-rich with EGF-like domains 2	303.3144	64.18259	80.78133	84.79398	85.47022	45.39527
17532257	Csrnp1	cysteine-serine-rich nuclear protein 1	10.3037	8.290965	9.631096	10.76368	9.218104	11.46433
17321714	Csrnp2	cysteine-serine-rich nuclear protein 2	17.52874	18.46767	13.79497	13.01019	14.52011	14.67746
17371254	Csrnp3	cysteine-serine-rich nuclear protein 3	6.082186	6.759704	6.722883	11.72389	5.944725	6.486904
17544061	Cysltr1	cysteinyl leukotriene receptor 1	3.371943	3.371943	4.152471	3.287338	3.371943	3.852567
17308556	Cysltr2	cysteinyl leukotriene receptor 2	5.350783	3.480006	3.095237	3.300581	4.911215	3.261759
17498271	Cars	cysteinyl-tRNA synthetase	38.36769	31.88619	27.24765	35.75715	42.61265	31.88619
17507440	Cars2	cysteinyl-tRNA synthetase 2 (mitochondrial)(putative)	32.76106	28.11699	30.11186	30.6501	30.21012	33.58325
17456247	Cftr	cystic fibrosis transmembrane conductance regulator	3.009972	3.009972	2.48961	3.317416	4.30705	2.884817
17280296	Cys1	cystin 1	8.684965	6.524422	7.345649	5.528431	5.455457	8.949642

17265733	Ctns	cystinosis, nephropathic	47.9022	51.82154	47.07862	39.81444	50.53952	59.68597
17429454	Ctps	cytidine 5-triphosphate synthase	13.75191	14.37958	14.64057	16.23595	13.38885	14.45563
17539434	Ctps2	cytidine 5-triphosphate synthase 2	18.78176	20.23779	20.91549	20.19659	19.26687	20.28217
17307337	Cdadc1	cytidine and dCMP deaminase domain containing 1	45.46423	46.31508	42.57044	46.31508	41.22764	49.56398
17431761	Cda	cytidine deaminase	39.95433	49.50595	49.14048	46.22784	42.28517	30.48315
17428430	Cmpk1	cytidine monophosphate (UMP-CMP) kinase 1	53.61445	56.28232	51.34244	48.28289	52.12921	53.62709
17274620	Cmpk2	cytidine monophosphate (UMP-CMP) kinase 2, mitochondrial	40.03001	23.15401	29.04605	35.88876	24.39781	26.36935
17285944	Cmah	cytidine monophospho-N-acetylneuraminic acid hydroxylase	278.2716	177.0288	162.3091	247.1615	234.9419	189.4811
17464146	Cmas	cytidine monophospho-N-acetylneuraminic acid synthetase	102.3169	78.08264	74.25571	90.75719	83.62486	89.43143
17532633	CYTB	cytochrome b	6258.365	6467.399	6484.502	6537.802	6656.37	6761.042
17371708	Cybrd1	cytochrome b reductase 1	8.863522	12.9009	11.70433	10.59653	11.47093	9.672307
17513672	Cyba	cytochrome b-245, alpha polypeptide	34.1717	30.97366	38.59468	26.36112	36.25053	26.31147
17540154	Cybb	cytochrome b-245, beta polypeptide	68.30835	76.53194	85.61134	61.01703	61.01703	59.43291
17264636	Cyb5d1	cytochrome b5 domain containing 1	45.09451	40.75994	45.89452	42.15465	46.45204	40.84847
17265710	Cyb5d2	cytochrome b5 domain containing 2	38.35838	36.55837	31.38297	39.00454	37.01017	34.341
17217440	Cyb5r1	cytochrome b5 reductase 1	18.76237	21.41797	21.41797	22.85107	17.22927	20.12867
17494739	Cyb5r2	cytochrome b5 reductase 2	4.694235	4.801649	4.801649	4.502793	4.86136	5.675195
17319783	Cyb5r3	cytochrome b5 reductase 3	635.8488	688.2935	778.3634	561.0945	560.7835	536.543
17520043	Cyb5r4	cytochrome b5 reductase 4	46.25156	36.9994	32.23911	40.13252	34.67803	42.6493
17416397	Cyb5rl	cytochrome b5 reductase-like	16.16935	16.16935	16.47367	15.71129	14.23184	16.16935
17352229	Cyb5a	cytochrome b5 type A (microsomal)	320.1979	359.9892	342.1962	369.0596	384.916	404.9556
17505252	Cyb5b	cytochrome b5 type B	315.7918	312.0211	292.822	283.2751	217.6792	244.1223
17270703	Cyb561	cytochrome b-561	15.18642	15.46859	16.88898	19.55072	16.23584	13.79717
17409234	Cyb561d1	cytochrome b-561 domain containing 1	6.23725	8.467112	9.01083	7.898014	10.95684	8.355522
17530909	Cyb561d2	cytochrome b-561 domain containing 2	13.333	10.33467	11.08933	12.35189	12.5113	12.35189
17357514	Cyb561a3	cytochrome b561 family, member A3	20.04333	17.37282	21.80837	27.09341	20.38915	20.38915
17269839	Coa3	cytochrome C oxidase assembly factor 3	113.4946	90.99655	98.72407	97.68668	92.40634	99.27579
17480757	Coa4	cytochrome c oxidase assembly factor 4	14.92191	12.54708	15.66497	15.72197	18.84265	14.20995
17222326	Coa5	cytochrome C oxidase assembly factor 5	140.2007	139.467	136.5618	143.0445	131.9018	144.9016
17506933	Coa6	cytochrome c oxidase assembly factor 6	56.0946	44.04908	47.68752	51.67949	57.69562	46.2429
17416633	Coa7	cytochrome c oxidase assembly factor 7	23.69449	21.30987	22.46209	25.36118	21.81381	24.41118
17264107	Cox10	cytochrome c oxidase assembly protein 10	26.13407	23.54877	30.02787	29.86389	29.25477	25.99838
17255081	Cox11	cytochrome c oxidase assembly protein 11	9.860631	12.43832	11.1842	11.14805	7.822176	14.03804
17314900	Cox14	cytochrome c oxidase assembly protein 14	28.59102	35.36107	30.67114	32.69391	26.62261	36.75223
17364954	Cox15	cytochrome c oxidase assembly protein 15	65.71132	72.93958	67.74911	77.53146	42.74963	53.69143
17282320	Cox16	cytochrome c oxidase assembly protein 16	30.66094	32.41995	42.12924	30.72498	27.97565	37.73863
17325581	Cox17	cytochrome c oxidase assembly protein 17	28.34515	45.27774	36.89619	42.05397	40.60062	36.79404
17449516	Cox18	cytochrome c oxidase assembly protein 18	38.76519	37.7164	43.65576	32.32593	33.48911	35.82438
17454398	Cox19	cytochrome c oxidase assembly protein 19	23.23343	20.43981	25.6044	19.54449	18.66896	21.8126
17290965	LOC102641600	cytochrome c oxidase subunit 5B, mitochondrial	327.0551	336.0681	399.8874	368.9624	451.9283	361.8405
17532609	COX1	cytochrome c oxidase subunit I	6827.634	6768.92	6558.454	7047.325	6533.181	6893.448
17532613	COX2	cytochrome c oxidase subunit II	4283.784	4612.33	4584.949	4688.465	4525.966	4769.955
17506273	Cox4i1	cytochrome c oxidase subunit IV isoform 1	266.6937	264.142	244.2627	285.6273	304.4117	288.5325
17377785	Cox4i2	cytochrome c oxidase subunit IV isoform 2	6.224725	5.979936	6.260249	5.278983	8.818174	4.948599
17517727	Cox5a	cytochrome c oxidase subunit Va	254.1101	221.3246	238.6959	265.116	259.1458	261.5984
17211850	Cox5b	cytochrome c oxidase subunit Vb	5.257292	8.467112	6.115922	6.287864	7.603744	7.438955
17451726	Cox6a1	cytochrome c oxidase subunit VIa polypeptide 1	1277.874	1726.542	2069.462	1630.931	1585.135	1675.76
17496857	Cox6a2	cytochrome c oxidase subunit VIa polypeptide 2	8.522106	8.522106	8.696953	8.522106	6.748	8.60163
17485904	Cox6b2	cytochrome c oxidase subunit VIb polypeptide 2	10.24658	10.71916	11.28911	10.47501	7.007355	8.149322
17316436	Cox6c	cytochrome c oxidase subunit VIc	14.45363	15.83934	16.10269	16.24102	16.15053	16.9117
17547698	Cox6c	cytochrome c oxidase subunit VIc	520.0945	556.2964	508.6749	549.6426	549.6426	578.182
17476288	Cox7a1	cytochrome c oxidase subunit VIIa 1	8.488561	12.36594	14.10525	13.32875	10.79948	12.70602
17529169	Cox7a2	cytochrome c oxidase subunit VIIa 2	247.7549	226.92	241.5219	273.3812	279.1003	296.1019
17347638	Cox7a2l	cytochrome c oxidase subunit VIIa polypeptide 2-like	47.22766	53.2526	49.71847	55.2342	60.40851	54.49833
17537048	Cox7b	cytochrome c oxidase subunit VIIb	4.649933	5.922146	6.352216	3.808572	7.549808	5.439372
17448589	Cox7b2	cytochrome c oxidase subunit VIIb2	2.575281	2.769777	2.529899	2.304206	2.720421	2.708962
17294723	Cox7c	cytochrome c oxidase subunit VIIc	8.572999	7.188995	8.479563	7.458937	8.768074	11.24304
17362274	Cox8a	cytochrome c oxidase subunit VIIIa	228.5079	321.0958	359.9868	299.0432	336.553	324.6519
17497704	Cox8b	cytochrome c oxidase subunit VIIIb	3.643184	4.033633	3.009972	3.16251	3.394102	3.45264
17278124	Cox8c	cytochrome c oxidase subunit VIIIc	3.566626	3.826406	3.243025	3.357716	3.826406	4.277299
17489223	Cox6b1	cytochrome c oxidase, subunit VIb polypeptide 1	210.9358	240.6195	203.5571	225.187	216.7533	232.7762
17342507	Gm10012	cytochrome c oxidase, subunit VIIc pseudogene	76.01488	85.33466	79.44971	90.66979	77.57716	91.71654
17466723	Cycs	cytochrome c, somatic	15.67594	13.98986	13.50864	14.89463	14.40782	16.27121
17548541	Cycs	cytochrome c, somatic	157.9769	144.2293	125.8263	128.5014	117.2651	152.8847
17386751	Cyct	cytochrome c, testis	3.738372	3.46727	3.856842	3.704888	4.875094	3.788351
17312396	Cyc1	cytochrome c-1	109.6886	109.3099	108.9143	115.9903	111.7553	123.7633
17359299	LOC105247429	cytochrome P450 2C38-like	4.217944	10.22685	8.28746	4.378753	7.423194	7.19797
17517770	Cyp1a1	cytochrome P450, family 1, subfamily a, polypeptide 1	4.158289	5.605682	6.992896	5.229127	4.47721	6.182713
17527581	Cyp1a2	cytochrome P450, family 1, subfamily a, polypeptide 2	539.6985	558.4174	427.8556	594.2722	514.6758	703.7016
17347448	Cyp1b1	cytochrome P450, family 1, subfamily b, polypeptide 1	4.158692	4.72289	4.265397	4.010995	3.824391	3.892967
17517828	Cyp11a1	cytochrome P450, family 11, subfamily a, polypeptide 1	4.923758	4.958212	4.103315	3.660067	4.204808	4.20927
17517831	Cyp11a1	cytochrome P450, family 11, subfamily a, polypeptide 1	5.466813	4.857116	4.647678	5.24819	6.047714	5.124531
17318045	Cyp11b1	cytochrome P450, family 11, subfamily b, polypeptide 1	3.276805	2.922291	2.85779	2.69598	3.138119	3.441697
17318058	Cyp11b2	cytochrome P450, family 11, subfamily b, polypeptide 2	4.801649	4.20927	4.397428	4.801649	4.801649	4.801649
17365410	Cyp17a1	cytochrome P450, family 17, subfamily a, polypeptide 1	85.4264	114.9617	556.1383	233.3206	360.2039	373.2685
17527193	Cyp19a1	cytochrome P450, family 19, subfamily a, polypeptide 1	3.083246	3.245138	3.221941	3.85206	2.878905	3.171906
17475487	Cyp2a12	cytochrome P450, family 2, subfamily a, polypeptide 12	2062.002	2024.837	1994.954	2033.769	2140.037	2221.287
17488105	Cyp2a22	cytochrome P450, family 2, subfamily a, polypeptide 22	31.49105	75.09162	103.1722	60.52587	62.9904	89.6847
17475399	Cyp2a4	cytochrome P450, family 2, subfamily a, polypeptide 4	237.6328	657.597	1202.374	349.6336	239.1646	348.2525
17475469	Cyp2a5	cytochrome P450, family 2, subfamily a, polypeptide 5	21.93496	36.19077	29.85643	31.58221	15.52423	33.41699
17329093	Cyp2ab1	cytochrome P450, family 2, subfamily ab, polypeptide 1	4.341429	3.447331	3.703322	3.703322	3.670432	4.187677
17475360	Cyp2b10	cytochrome P450, family 2, subfamily b, polypeptide 10	11.56048	4.870399	6.479803	5.254524	6.041848	6.358031
17475373	Cyp2b13	cytochrome P450, family 2, subfamily b, polypeptide 13	13.80539	29.41887	67.10461	16.33472	15.06564	92.12686
17475447	Cyp2b19	cytochrome P450, family 2, subfamily b, polypeptide 19	2.406006	2.837814	2.64145	2.822728	2.644491	3.19191

17488094	Cyp2b23	cytochrome P450, family 2, subfamily b, polypeptide 23	3.92894	3.382102	3.509316	3.099166	2.807449	3.529001
17475385	Cyp2b9	cytochrome P450, family 2, subfamily b, polypeptide 9	324.3303	1606.55	2085.301	697.8823	547.4942	1220.418
17359287	Cyp2c29	cytochrome P450, family 2, subfamily c, polypeptide 29	533.539	328.227	493.4602	340.3912	712.097	779.5242
17364392	Cyp2c38	cytochrome P450, family 2, subfamily c, polypeptide 38	180.5745	546.7282	495.582	291.9788	357.146	315.84
17359304	Cyp2c39	cytochrome P450, family 2, subfamily c, polypeptide 39	18.90204	31.39211	31.36188	26.40428	16.41884	29.72778
17364426	Cyp2c40	cytochrome P450, family 2, subfamily c, polypeptide 40	7.902121	7.868944	6.870285	9.018141	9.173231	6.334453
17365001	Cyp2c44	cytochrome P450, family 2, subfamily c, polypeptide 44	852.5733	723.1895	607.4619	659.0253	765.262	667.9008
17359327	Cyp2c50	cytochrome P450, family 2, subfamily c, polypeptide 50	773.7158	720.7424	567.4423	949.6157	870.886	1101.207
17359274	Cyp2c53-ps	cytochrome P450, family 2, subfamily c, polypeptide 53-ps	2.64726	2.046672	2.370186	2.752819	2.390662	2.094869
17364452	Cyp2c54	cytochrome P450, family 2, subfamily c, polypeptide 54	416.2395	412.2541	328.1292	517.283	580.2577	574.3952
17359239	Cyp2c55	cytochrome P450, family 2, subfamily c, polypeptide 55	6.076034	5.493479	7.238697	5.697859	4.016197	5.945673
17359251	Cyp2c65	cytochrome P450, family 2, subfamily c, polypeptide 65	1.692822	1.976188	1.87561	2.609693	2.8288	2.101748
17359262	Cyp2c66	cytochrome P450, family 2, subfamily c, polypeptide 66	2.432315	1.988403	2.50243	2.259886	2.141141	3.359904
17364403	Cyp2c67	cytochrome P450, family 2, subfamily c, polypeptide 67	472.2477	427.405	416.999	458.094	489.909	310.5173
17364414	Cyp2c68	cytochrome P450, family 2, subfamily c, polypeptide 68	121.2382	147.117	142.4212	118.457	144.3453	119.6669
17364440	Cyp2c69	cytochrome P450, family 2, subfamily c, polypeptide 69	19.0902	18.1897	17.96118	20.82667	15.8423	15.00103
17364463	Cyp2c70	cytochrome P450, family 2, subfamily c, polypeptide 70	279.6154	176.9564	318.5774	276.9881	454.7059	280.4714
17319700	Cyp2d41-ps	cytochrome P450, family 2, subfamily d, member 41, pseudogene	19.01861	15.55504	11.05472	27.96034	23.15898	19.37011
17319649	Cyp2d10	cytochrome P450, family 2, subfamily d, polypeptide 10	239.0883	257.3611	260.4947	327.3772	255.4346	281.1527
17319638	Cyp2d11	cytochrome P450, family 2, subfamily d, polypeptide 11	2.753354	2.527603	2.693179	2.530618	3.061185	1.969924
17313599	Cyp2d12	cytochrome P450, family 2, subfamily d, polypeptide 12	2.391225	2.099215	2.255364	2.304206	2.251974	2.100889
17319674	Cyp2d13	cytochrome P450, family 2, subfamily d, polypeptide 13	322.0007	371.4283	394.0514	413.6251	349.3259	429.1238
17319625	Cyp2d22	cytochrome P450, family 2, subfamily d, polypeptide 22	211.265	220.377	202.628	246.5216	255.5497	236.0846
17319707	Cyp2d26	cytochrome P450, family 2, subfamily d, polypeptide 26	1078.298	1101.52	1010.918	929.9955	987.8307	1077.021
17319661	Cyp2d34	cytochrome P450, family 2, subfamily d, polypeptide 34	4.253882	5.300432	3.801688	6.657267	4.180223	4.56508
17319684	Cyp2d37-ps	cytochrome P450, family 2, subfamily d, polypeptide 37, pseudogene	96.161	128.718	159.3417	179.357	167.2057	171.566
17319691	Cyp2d40	cytochrome P450, family 2, subfamily d, polypeptide 40	60.25086	42.40522	51.02775	35.14092	47.32236	40.32437
17313583	Cyp2d9	cytochrome P450, family 2, subfamily d, polypeptide 9	438.4109	324.6969	198.9276	422.9578	324.4765	334.5968
17484587	Cyp2e1	cytochrome P450, family 2, subfamily e, polypeptide 1	4097.335	4526.091	4050.825	4467.618	4141.248	4874.201
17475498	Cyp2f2	cytochrome P450, family 2, subfamily f, polypeptide 2	1809.932	1547.882	1634.666	1542.57	1275.9	1197.608
17475458	Cyp2g1	cytochrome P450, family 2, subfamily g, polypeptide 1	4.818219	3.821926	3.595968	4.400996	6.754598	5.10974
17427367	Cyp2j11	cytochrome P450, family 2, subfamily j, polypeptide 11	4.941503	3.883991	3.339703	3.96063	2.734032	2.606909
17427345	Cyp2j12	cytochrome P450, family 2, subfamily j, polypeptide 12	5.137548	5.001878	4.536155	4.283678	4.659645	4.169198
17427331	Cyp2j13	cytochrome P450, family 2, subfamily j, polypeptide 13	3.80095	4.728229	4.065013	5.14464	5.078456	5.232353
17427414	Cyp2j5	cytochrome P450, family 2, subfamily j, polypeptide 5	1539.931	1303.152	1228.013	1335.542	1256.069	1345.914
17427389	Cyp2j6	cytochrome P450, family 2, subfamily j, polypeptide 6	83.4957	67.71812	74.07989	81.53683	71.06907	75.23854
17427357	Cyp2j7	cytochrome P450, family 2, subfamily j, polypeptide 7	1.840924	2.497284	2.122949	3.033661	2.780415	2.08603
17427379	Cyp2j8	cytochrome P450, family 2, subfamily j, polypeptide 8	4.079861	4.226431	3.653857	4.051707	6.129842	5.018304
17427401	Cyp2j9	cytochrome P450, family 2, subfamily j, polypeptide 9	21.10701	22.10131	16.86558	15.30128	17.45599	21.04769
17495320	Cyp2r1	cytochrome P450, family 2, subfamily r, polypeptide 1	89.2104	39.49888	38.16055	49.78123	53.77561	38.28507
17488057	Cyp2s1	cytochrome P450, family 2, subfamily s, polypeptide 1	5.315406	6.196184	5.879445	6.546392	4.897923	5.705764
17475512	Cyp2t4	cytochrome P450, family 2, subfamily t, polypeptide 4	4.65519	4.38185	4.049574	4.220664	4.820925	4.225728
17410400	Cyp2u1	cytochrome P450, family 2, subfamily u, polypeptide 1	147.2016	49.58012	37.65449	80.80373	73.19281	74.85361
17443985	Cyp2w1	cytochrome P450, family 2, subfamily w, polypeptide 1	6.973969	7.740195	8.471515	7.310196	6.890936	7.105962
17359316	Cyp2c37	cytochrome P450, family 2. subfamily c, polypeptide 37	670.8284	637.791	745.5855	679.486	729.3108	834.8366
17213428	Cyp20a1	cytochrome P450, family 20, subfamily a, polypeptide 1	39.43186	30.40064	31.03684	25.40235	35.58235	32.08573
17343962	Cyp21a1	cytochrome P450, family 21, subfamily a, polypeptide 1	6.992896	6.801156	5.629122	7.599371	8.35089	6.853163
17394965	Cyp24a1	cytochrome P450, family 24, subfamily a, polypeptide 1	6.889885	7.265691	8.275635	7.164768	7.407209	7.866326
17359088	Cyp26a1	cytochrome P450, family 26, subfamily a, polypeptide 1	6.187557	7.260455	5.804453	7.629931	5.664388	7.928127
17468286	Cyp26b1	cytochrome P450, family 26, subfamily b, polypeptide 1	22.74457	19.53839	20.19813	31.11784	15.69747	18.33629
17359079	Cyp26c1	cytochrome P450, family 26, subfamily c, polypeptide 1	4.274957	4.400416	4.145454	3.77815	4.464876	4.294738
17214368	Cyp27a1	cytochrome P450, family 27, subfamily a, polypeptide 1	454.5074	366.9348	330.1482	452.7089	410.1449	373.3953
17237887	Cyp27b1	cytochrome P450, family 27, subfamily b, polypeptide 1	4.538608	4.538608	5.108855	3.985691	4.723185	5.087546
17455175	Cyp3a11	cytochrome P450, family 3, subfamily a, polypeptide 11	399.4746	581.5744	458.3291	344.5262	302.9341	415.8156
17454193	Cyp3a13	cytochrome P450, family 3, subfamily a, polypeptide 13	246.2688	316.2979	368.8371	256.7464	336.5274	219.2708
17455111	Cyp3a16	cytochrome P450, family 3, subfamily a, polypeptide 16	3.199552	3.277555	3.316565	3.066281	3.250091	3.310295
17455193	Cyp3a25	cytochrome P450, family 3, subfamily a, polypeptide 25	55.39946	102.0074	138.2629	197.9401	154.9716	149.81
17455143	Cyp3a41a	cytochrome P450, family 3, subfamily a, polypeptide 41A	287.3211	375.6773	291.1644	239.5478	189.8663	276.361
17455127	Cyp3a41b	cytochrome P450, family 3, subfamily a, polypeptide 41B	552.6836	701.8894	549.555	467.8331	364.9538	514.9898
17455159	Cyp3a44	cytochrome P450, family 3, subfamily a, polypeptide 44	8.194807	8.911881	7.6943	6.098101	6.87219	6.740053
17444682	Cyp3a57	cytochrome P450, family 3, subfamily a, polypeptide 57	2.782767	2.37173	3.306695	2.85136	2.622659	3.339703
17444697	Cyp3a59	cytochrome P450, family 3, subfamily a, polypeptide 59	25.04655	27.69762	40.03389	27.66155	27.40657	28.88823
17337816	Cyp39a1	cytochrome P450, family 39, subfamily a, polypeptide 1	67.29465	99.39973	133.4245	91.51396	44.32227	91.09879
17417223	Cyp4a10	cytochrome P450, family 4, subfamily a, polypeptide 10	2544.982	3234.395	3431.705	3162.785	2288.886	2599.16
17417180	Cyp4a12a	cytochrome P450, family 4, subfamily a, polypeptide 12a	206.695	231.1171	55.82154	158.8102	146.0947	87.68086
17417195	Cyp4a12b	cytochrome P450, family 4, subfamily a, polypeptide 12B	349.1371	175.2985	65.69583	246.6976	119.6296	137.6919
17428457	Cyp4a14	cytochrome P450, family 4, subfamily a, polypeptide 14	2728.023	3864.698	3827.297	3064.19	2385.821	2580.509
17417167	Cyp4a29	cytochrome P450, family 4, subfamily a, polypeptide 29	3.405886	2.602972	3.532697	2.671685	2.785932	2.865983
17417210	Cyp4a30b	cytochrome P450, family 4, subfamily a, polypeptide 30b	3.319601	3.369303	2.690175	4.380453	3.580175	3.47909
17417240	Cyp4a31	cytochrome P450, family 4, subfamily a, polypeptide 31	251.9109	499.9292	603.6088	403.6273	359.5304	333.0119
17417255	Cyp4a32	cytochrome P450, family 4, subfamily a, polypeptide 32	78.51737	141.6464	152.0614	117.5059	77.70255	132.7502
17428477	Cyp4b1	cytochrome P450, family 4, subfamily b, polypeptide 1	45.00365	38.13886	52.04941	43.16111	19.62866	26.87159
17428473	Cyp4b1-ps2	cytochrome P450, family 4, subfamily b, polypeptide 1, pseudogene 2	1.830135	2.67402	2.669871	2.879563	3.632166	2.719271
17343505	Cyp4f13	cytochrome P450, family 4, subfamily f, polypeptide 13	76.76996	78.14816	69.5626	66.82148	65.74731	70.08919
17343488	Cyp4f14	cytochrome P450, family 4, subfamily f, polypeptide 14	154.0303	72.78655	58.58665	108.8947	124.4558	88.85186
17336013	Cyp4f15	cytochrome P450, family 4, subfamily f, polypeptide 15	163.5502	201.5648	177.1503	223.0201	171.7981	201.5648
17335966	Cyp4f16	cytochrome P450, family 4, subfamily f, polypeptide 16	6.992896	7.552059	7.666988	7.252408	7.727223	10.1135
17335952	Cyp4f17	cytochrome P450, family 4, subfamily f, polypeptide 17	31.91892	37.25572	35.18894	32.83745	21.42465	27.64449
17510462	Cyp4f18	cytochrome P450, family 4, subfamily f, polypeptide 18	6.591423	7.714914	6.054383	6.014313	8.4294	5.859104
17335983	Cyp4f37	cytochrome P450, family 4, subfamily f, polypeptide 37	4.3015	5.694227	5.479636	8.731321	5.543626	4.008604
17335936	Cyp4f39	cytochrome P450, family 4, subfamily f, polypeptide 39	4.740864	3.959395	5.2659	4.20927	3.836471	4.340286
17335998	Cyp4f40	cytochrome P450, family 4, subfamily f, polypeptide 40	3.141514	2.67402	2.569522	2.826115	2.782767	2.67402
17343521	Cyp4f41-ps	cytochrome P450, family 4, subfamily f, polypeptide 41 pseudogene	4.405482	3.866934	3.869025	3.520426	3.250631	4.189825
17509069	Cyp4v3	cytochrome P450, family 4, subfamily v, polypeptide 3	725.2879	693.9125	556.416	693.5038	482.5685	672.7376

17428441	Cyp4x1	cytochrome P450, family 4, subfamily x, polypeptide 1	6.702596	7.629984	7.868726	8.64107	7.06701	9.19768
17417162	Cyp4x1os	cytochrome P450, family 4, subfamily x, polypeptide 1, opposite strand	5.14079	4.579436	4.117218	4.527988	4.527988	4.794997
17278543	Cyp46a1	cytochrome P450, family 46, subfamily a, polypeptide 1	11.18955	11.99773	14.43741	13.77204	12.35489	12.35489
17445308	Cyp51	cytochrome P450, family 51	111.2946	78.20018	117.3293	90.43191	135.0163	64.61736
17423051	Cyp7a1	cytochrome P450, family 7, subfamily a, polypeptide 1	32.03995	198.9083	251.7626	157.8664	254.237	487.6323
17404245	Cyp7b1	cytochrome P450, family 7, subfamily b, polypeptide 1	365.0398	102.9093	59.74226	325.454	116.9331	98.51695
17532409	Cyp8b1	cytochrome P450, family 8, subfamily b, polypeptide 1	296.4777	403.5126	406.8773	428.8978	268.8225	326.559
17272461	Cygb	cytoglobin	47.60034	51.95317	55.62764	45.33866	35.4676	35.4676
17272722	Cyth1	cytohesin 1	26.76322	32.91492	33.36122	29.42621	26.59844	31.9488
17385405	Cytip	cytohesin 1 interacting protein	3.935345	4.20927	3.828661	5.000457	5.231991	4.226871
17490960	Cyth2	cytohesin 2	50.55599	49.07172	53.64382	51.88336	52.23446	45.00926
17444372	Cyth3	cytohesin 3	9.043743	12.13481	15.91185	11.86385	10.00743	10.18603
17312759	Cyth4	cytohesin 4	34.5365	36.02983	44.95719	38.9192	39.55039	36.24525
17511887	Ciapin1	cytokine induced apoptosis inhibitor 1	38.15367	46.17596	49.66543	50.97755	36.10758	38.13622
17521300	Cish	cytokine inducible SH2-containing protein	15.95404	9.884445	11.97895	12.82121	13.03592	8.585725
17501940	Crlf1	cytokine receptor-like factor 1	7.744918	8.633467	9.092078	10.32235	7.446074	8.871204
17450918	Crlf2	cytokine receptor-like factor 2	18.16312	18.90859	18.55582	22.93979	19.49537	18.71405
17266659	Crlf3	cytokine receptor-like factor 3	19.08649	17.66963	17.69733	21.75432	16.74931	19.74438
17447698	Clnk	cytokine-dependent hematopoietic cell linker	4.01823	4.596195	4.074915	3.851739	4.59594	3.319624
17437117	Clnkos	cytokine-dependent hematopoietic cell linker, opposite strand	6.631982	4.801649	5.050933	5.895401	5.886132	4.802015
17437043	Cytl1	cytokine-like 1	6.171709	8.767223	9.268205	6.431789	11.01907	7.865726
17478549	Cyfip1	cytoplasmic FMR1 interacting protein 1	78.10321	75.03647	77.87941	68.29462	59.12884	78.52717
17262065	Cyfip2	cytoplasmic FMR1 interacting protein 2	13.45882	10.47073	9.722788	8.261192	8.439397	8.398316
17297777	Cphx2	cytoplasmic polyadenylated homeobox 2	5.681178	4.620503	5.466927	6.124067	4.55667	5.170307
17297810	Cphx2	cytoplasmic polyadenylated homeobox 2	5.138438	4.257864	4.862203	5.111051	4.404383	4.843534
17297842	Cphx2	cytoplasmic polyadenylated homeobox 2	5.138438	4.257864	4.862203	5.111051	4.404383	4.843534
17492754	Cpeb1	cytoplasmic polyadenylation element binding protein 1	7.98864	8.870467	8.6984	9.114288	8.597815	8.470458
17437129	Cpeb2	cytoplasmic polyadenylation element binding protein 2	114.3625	103.4722	101.7772	125.3962	98.0734	126.8509
17364218	Cpeb3	cytoplasmic polyadenylation element binding protein 3	7.666723	9.383655	9.196405	9.020641	9.85216	9.160396
17248213	Cpeb4	cytoplasmic polyadenylation element binding protein 4	146.1079	124.0287	111.4544	116.8319	82.77539	87.89688
17508025	Ckap2	cytoskeleton associated protein 2	3.832617	4.08722	3.913312	3.598286	4.707562	4.600817
17391544	Ckap2l	cytoskeleton associated protein 2-like	3.953417	5.429653	3.692019	5.507853	5.291145	6.286426
17373328	Ckap5	cytoskeleton associated protein 5	31.33841	30.59937	31.31235	30.59937	26.88985	32.04039
17243617	Ckap4	cytoskeleton-associated protein 4	7.996618	12.82476	11.54327	11.4784	13.21936	11.10402
17391247	Ciao1	cytosolic iron-sulfur protein assembly 1	75.88064	73.11269	71.3163	76.22752	72.72855	79.01748
17477178	Ctu1	cytosolic thiouridylase subunit 1 homolog (S. pombe)	9.016765	10.46108	8.30759	5.828966	6.538139	8.745434
17506400	Ctu2	cytosolic thiouridylase subunit 2 homolog (S. pombe)	23.4192	21.92158	25.50061	22.21019	24.91012	22.65143
17525856	Crtam	cytotoxic and regulatory T cell molecule	4.377873	5.01368	4.418633	4.399776	4.467972	4.126132
17460405	Tia1	cytotoxic granule-associated RNA binding protein 1	10.87113	14.31296	14.31296	13.93653	14.49095	14.31296
17293362	Ctla2a	cytotoxic T lymphocyte-associated protein 2 alpha	7.823315	6.584391	8.264457	6.73515	7.370079	8.476038
17293348	Ctla2b	cytotoxic T lymphocyte-associated protein 2 beta	4.896599	4.114708	6.41736	5.872684	6.066944	9.003463
17213478	Ctla4	cytotoxic T-lymphocyte-associated protein 4	3.027506	2.577138	2.578193	2.690138	2.87159	2.536941
17477979	Dbp	D site albumin promoter binding protein	42.13829	105.4723	145.167	62.97817	87.39265	117.7571
17216096	D2hgdh	D-2-hydroxyglutarate dehydrogenase	30.91146	33.89804	31.69097	30.91146	31.61331	23.62922
17476125	Dpf1	D4, zinc and double PHD fingers family 1	9.060307	12.71589	10.67704	10.67379	9.860221	11.49839
17361833	Dpf2	D4, zinc and double PHD fingers family 2	35.5572	37.47873	39.15596	35.69277	34.66842	37.74124
17282403	Dpf3	D4, zinc and double PHD fingers, family 3	6.171709	8.467112	8.467112	9.812427	8.467112	8.669748
17309041	Dach1	dachshund 1 (Drosophila)	2.980822	3.426581	3.224532	3.42416	2.734961	3.213644
17537283	Dach2	dachshund 2 (Drosophila)	3.046431	2.78215	3.505451	3.361139	3.048374	2.810779
17494610	Dchs1	dachsous 1 (Drosophila)	9.756948	12.81214	11.12281	11.50086	13.92273	10.05109
17398528	Dchs2	dachsous 2 (Drosophila) [Source:MGI Symbol;Acc:MGI:2685263]	3.927932	3.883991	3.553572	3.514453	4.004355	3.927932
17521858	Dalrd3	DALR anticodon binding domain containing 3	44.08543	48.22168	46.63464	46.19677	43.90977	42.10559
17357529	Ddb1	damage specific DNA binding protein 1	228.1454	212.5222	198.8298	204.2228	202.5747	183.259
17388196	Ddb2	damage specific DNA binding protein 2	16.52055	21.44709	21.88029	16.02753	19.26219	18.85781
17440775	Dao	D-amino acid oxidase	6.554519	5.292979	5.15996	5.376622	5.286196	5.852812
17511161	Dand5	DAN domain family, member 5	5.338965	4.953781	6.408426	4.705376	5.193906	5.376063
17276139	Dact1	dapper homolog 1, antagonist of beta-catenin (xenopus)	11.00266	10.1498	7.97586	10.52268	9.056603	11.57251
17341069	Dact2	dapper homolog 2, antagonist of beta-catenin (xenopus)	31.10641	27.61366	31.45288	32.63821	28.36009	23.67824
17474205	Dact3	dapper homolog 3, antagonist of beta-catenin (xenopus)	7.982164	8.536827	13.91858	8.793759	11.13593	8.551218
17232763	Ddo	D-aspartate oxidase	14.28768	14.77955	18.18668	21.82985	13.30005	16.43159
17235278	Dazap1	DAZ associated protein 1	86.79259	119.4324	118.5614	113.379	104.4277	105.6281
17315045	Dazap2	DAZ associated protein 2	168.8701	197.9813	186.3499	160.1944	195.2908	171.8803
17309457	Dzip1	DAZ interacting protein 1	4.816383	5.856749	5.565649	4.832769	5.30091	5.525098
17438680	Gm7337	DAZ interacting protein 1 pseudogene	12.21939	12.19045	10.66876	7.975749	10.82196	10.66876
17520742	Dzip1l	DAZ interacting protein 1-like	6.225458	7.274207	6.273672	7.097692	6.030928	7.562745
17330815	Dzip3	DAZ interacting protein 3, zinc finger	7.698994	8.672993	7.698994	7.698994	5.957677	6.465626
17445507	Dbf4	DBF4 homolog (S. cerevisiae)	8.58281	7.902121	7.922084	7.922084	6.981998	10.29855
17501104	Dctd	dCMP deaminase	10.04997	11.71918	9.732834	11.41802	10.4806	11.24366
17404731	Dcun1d1	DCN1, defective in cullin neddylation 1, domain containing 1 (S. cerevisiae)	12.44962	13.77108	10.86021	12.36508	12.25527	12.44574
17507584	Dcun1d2	DCN1, defective in cullin neddylation 1, domain containing 2 (S. cerevisiae)	35.34171	26.44293	29.75953	28.24141	25.95463	32.17928
17495692	Dcun1d3	DCN1, defective in cullin neddylation 1, domain containing 3 (S. cerevisiae)	16.64792	19.67729	18.65939	15.77972	15.24908	11.72991
17438134	Dcun1d4	DCN1, defective in cullin neddylation 1, domain containing 4 (S. cerevisiae)	56.50513	53.94473	53.34755	50.56825	49.15513	53.01615
17514471	Dcun1d5	DCN1, defective in cullin neddylation 1, domain containing 5 (S. cerevisiae)	48.6612	49.19978	39.99894	53.95557	53.93837	48.6612
17298171	Dcp1a	DCP1 decapping enzyme homolog A (S. cerevisiae)	16.8685	18.06619	19.9459	18.67259	11.08164	14.32368
17462231	Dcp1b	DCP1 decapping enzyme homolog B (S. cerevisiae)	12.38378	12.50601	12.37451	12.74483	14.03532	13.8587
17350281	Dcp2	DCP2 decapping enzyme homolog (S. cerevisiae)	26.0693	23.89903	25.96111	26.55515	32.45953	27.71144
17407049	Dcst1	DC-STAMP domain containing 1	4.530122	5.291035	6.136644	5.185812	6.452776	5.252885
17399411	Dcst2	DC-STAMP domain containing 2	6.803325	9.879171	6.82666	6.107619	5.993007	7.383475
17496645	Dctpp1	dCTP pyrophosphatase 1	29.96923	32.15341	37.5518	41.44454	37.29447	41.27968
17413649	Dcaf10	DDB1 and CUL4 associated factor 10	84.33853	56.0946	49.95164	56.0946	54.66365	46.43498
17300519	Dcaf11	DDB1 and CUL4 associated factor 11	236.9272	232.1704	240.1347	253.9763	224.6772	257.8503
17424203	Dcaf12	DDB1 and CUL4 associated factor 12	54.23153	58.00519	50.22452	53.3261	53.92379	50.05839
17541328	Dcaf12l1	DDB1 and CUL4 associated factor 12-like 1	5.174107	6.329274	6.372502	5.774196	5.548701	4.862405
17541322	Dcaf12l2	DDB1 and CUL4 associated factor 12-like 2	3.827971	4.886969	4.886969	4.886969	5.299183	4.367901

17311199	Dcaf13	DDB1 and CUL4 associated factor 13	52.69704	63.27352	69.13144	74.2166	84.98345	76.8184
17511023	Dcaf15	DDB1 and CUL4 associated factor 15	10.32457	12.43186	10.64925	11.57099	10.63664	15.4747
17371684	Dcaf17	DDB1 and CUL4 associated factor 17	26.75803	28.84121	26.58038	28.62592	30.07828	25.02521
17277043	Dcaf4	DDB1 and CUL4 associated factor 4	9.908592	13.11386	10.87218	13.11386	11.56746	16.03286
17282253	Dcaf5	DDB1 and CUL4 associated factor 5	53.05916	52.30076	50.32139	54.31467	45.80712	46.82931
17281481	Gm17821	DDB1 and CUL4 associated factor 5 pseudogene	2.897101	3.973031	3.338801	3.414206	5.285903	3.559094
17229233	Dcaf6	DDB1 and CUL4 associated factor 6	121.1877	155.292	157.6553	148.3252	152.9886	136.7029
17257492	Dcaf7	DDB1 and CUL4 associated factor 7	80.88611	81.846	99.86658	83.44999	91.52348	89.04113
17219499	Dcaf8	DDB1 and CUL4 associated factor 8	186.7921	183.9513	158.0766	167.9782	168.0609	160.9122
17305685	Ddhd1	DDHD domain containing 1	67.60462	53.06909	51.8528	51.08685	50.1939	44.23419
17508396	Ddhd2	DDHD domain containing 2	36.93944	53.65683	49.68953	45.49765	34.47432	44.74765
17523730	Ddi1	DDI1, DNA-damage inducible 1, homolog 1 (S. cerevisiae)	2.850154	3.056202	2.72378	2.686616	3.081152	2.41739
17241912	Ddt	D-dopachrome tautomerase	573.1901	533.7795	531.2768	606.0067	553.1857	581.3971
17391693	Ddrgk1	DDRGK domain containing 1	114.5307	106.4875	118.9779	109.6286	137.2945	150.989
17280009	Ddx1	DEAD (Asp-Glu-Ala-Asp) box polypeptide 1	179.9809	172.6121	177.5203	180.4281	169.013	168.0283
17527032	Ddx10	DEAD (Asp-Glu-Ala-Asp) box polypeptide 10	38.05806	27.79204	28.13434	29.19117	23.80463	26.75265
17319230	Ddx17	DEAD (Asp-Glu-Ala-Asp) box polypeptide 17	124.4369	127.9716	121.5613	121.5613	111.3247	127.4537
17226369	Ddx18	DEAD (Asp-Glu-Ala-Asp) box polypeptide 18	59.50752	51.46608	52.35916	55.68782	54.51873	42.70668
17512975	Ddx19a	DEAD (Asp-Glu-Ala-Asp) box polypeptide 19a	60.81815	89.56977	71.26343	73.25016	88.8574	93.38572
17512990	Ddx19b	DEAD (Asp-Glu-Ala-Asp) box polypeptide 19b	21.33825	19.34662	21.4974	19.87276	19.54896	17.90723
17408829	Ddx20	DEAD (Asp-Glu-Ala-Asp) box polypeptide 20	24.82772	20.54264	23.26566	24.52906	23.27743	26.76137
17241436	Ddx21	DEAD (Asp-Glu-Ala-Asp) box polypeptide 21	53.95482	49.16126	47.36075	49.81851	46.65113	39.87769
17321287	Ddx23	DEAD (Asp-Glu-Ala-Asp) box polypeptide 23	64.48135	58.02771	60.67246	67.2732	62.76116	64.34829
17283536	Ddx24	DEAD (Asp-Glu-Ala-Asp) box polypeptide 24	52.34646	47.75236	57.282	57.36942	56.39596	54.85267
17525405	Ddx25	DEAD (Asp-Glu-Ala-Asp) box polypeptide 25	3.477113	4.225172	3.005552	3.60203	4.096154	4.096154
17379841	Ddx27	DEAD (Asp-Glu-Ala-Asp) box polypeptide 27	33.01981	28.63703	26.29306	31.84401	36.52443	32.96881
17512630	Ddx28	DEAD (Asp-Glu-Ala-Asp) box polypeptide 28	17.25114	20.28414	22.10683	18.69383	23.99547	18.7417
17546834	Ddx3y	DEAD (Asp-Glu-Ala-Asp) box polypeptide 3, Y-linked	151.2121	142.434	136.5126	147.0456	158.2564	150.5032
17502976	Ddx39	DEAD (Asp-Glu-Ala-Asp) box polypeptide 39	18.47343	18.93955	29.71007	16.73191	17.56912	15.90998
17337046	Ddx39b	DEAD (Asp-Glu-Ala-Asp) box polypeptide 39B	35.94402	50.80329	60.47219	52.13174	48.99219	57.70682
17296225	Ddx4	DEAD (Asp-Glu-Ala-Asp) box polypeptide 4	3.323515	3.258188	3.305045	3.409263	3.364075	3.642831
17292922	Ddx41	DEAD (Asp-Glu-Ala-Asp) box polypeptide 41	29.71316	24.42489	26.93593	29.09663	28.14975	30.90911
17257534	Ddx42	DEAD (Asp-Glu-Ala-Asp) box polypeptide 42	38.43876	35.37308	37.83055	40.8989	41.12689	33.80934
17519699	Ddx43	DEAD (Asp-Glu-Ala-Asp) box polypeptide 43	2.837027	2.837027	2.925088	2.62384	2.459525	2.796883
17287733	Ddx46	DEAD (Asp-Glu-Ala-Asp) box polypeptide 46	78.83585	74.84272	74.68414	69.85348	78.00799	62.33282
17463731	Ddx47	DEAD (Asp-Glu-Ala-Asp) box polypeptide 47	58.86303	47.3509	43.69511	46.28042	50.36397	50.28862
17509997	Ddx49	DEAD (Asp-Glu-Ala-Asp) box polypeptide 49	50.4905	41.33437	37.30766	40.95024	39.50655	48.50461
17270974	Ddx5	DEAD (Asp-Glu-Ala-Asp) box polypeptide 5	266.0475	334.3071	326.9146	329.4144	303.6131	325.1158
17270999	Ddx5	DEAD (Asp-Glu-Ala-Asp) box polypeptide 5	585.4613	882.4373	942.2591	1003.079	845.0952	994.2398
17241454	Ddx50	DEAD (Asp-Glu-Ala-Asp) box polypeptide 50	82.76105	72.57546	66.16884	69.89464	64.54529	74.04447
17440553	Ddx51	DEAD (Asp-Glu-Ala-Asp) box polypeptide 51	15.53252	18.76315	18.25893	23.03395	21.32036	20.31988
17254347	Ddx52	DEAD (Asp-Glu-Ala-Asp) box polypeptide 52	24.21468	24.56121	22.60328	24.56121	24.56121	18.18353
17441701	Ddx54	DEAD (Asp-Glu-Ala-Asp) box polypeptide 54	101.6369	78.93428	77.31626	84.40637	86.5133	84.40637
17442534	Ddx55	DEAD (Asp-Glu-Ala-Asp) box polypeptide 55	16.94714	15.41475	15.03601	16.09566	13.73162	15.43357
17260331	Ddx56	DEAD (Asp-Glu-Ala-Asp) box polypeptide 56	34.2547	39.49012	34.08299	31.71624	30.63078	25.3756
17423987	Ddx58	DEAD (Asp-Glu-Ala-Asp) box polypeptide 58	56.70563	46.18428	50.8981	55.58755	49.03568	57.46175
17217777	Ddx59	DEAD (Asp-Glu-Ala-Asp) box polypeptide 59	12.95081	14.82335	13.39841	15.16391	9.279069	14.93049
17516633	Ddx6	DEAD (Asp-Glu-Ala-Asp) box polypeptide 6	299.1925	253.7387	210.4947	266.8083	249.6079	242.3805
17501440	Ddx60	DEAD (Asp-Glu-Ala-Asp) box polypeptide 60	8.297519	10.19677	11.05081	14.85637	10.59925	11.54319
17353758	Dnd1	dead end homolog 1 (zebrafish)	3.864602	4.124216	4.709259	4.651706	5.38688	4.20927
17339187	Ddx11	DEAD/H (Asp-Glu-Ala-Asp/His) box helicase 11	15.13818	12.09669	10.37716	12.40208	7.968234	10.1189
17534909	Ddx26b	DEAD/H (Asp-Glu-Ala-Asp/His) box polypeptide 26B	7.035583	8.396162	9.105764	7.945401	9.636906	10.12781
17533416	Ddx3x	DEAD/H (Asp-Glu-Ala-Asp/His) box polypeptide 3, X-linked	203.4894	215.6697	175.2852	199.1208	228.2577	198.458
17368762	Ddx31	DEAD/H (Asp-Glu-Ala-Asp/His) box polypeptide 31	12.86197	11.58423	10.24736	10.33302	8.80663	10.7292
17466670	Dfna5	deafness, autosomal dominant 5 (human)	6.359353	6.29266	6.214852	5.824645	4.920313	6.275498
17372233	Dfnb59	deafness, autosomal recessive 59 (human)	3.703322	3.90567	3.188814	3.557244	3.729055	4.120902
17347505	Dhx57	DEAH (Asp-Glu-Ala-Asp/His) box polypeptide 57	27.85916	26.12773	25.02156	33.03103	23.83814	30.44809
17448021	Dhx15	DEAH (Asp-Glu-Ala-His) box polypeptide 15	118.3183	116.3897	110.6063	120.8966	113.361	112.0874
17337285	Dhx16	DEAH (Asp-Glu-Ala-His) box polypeptide 16	38.8031	35.50229	37.76113	38.20872	35.88826	37.76113
17289941	Dhx29	DEAH (Asp-Glu-Ala-His) box polypeptide 29	40.98517	31.80651	35.62554	41.18223	31.82569	30.49583
17531488	Dhx30	DEAH (Asp-Glu-Ala-His) box polypeptide 30	16.49081	15.67529	16.67726	18.33489	15.10464	16.92812
17497223	Dhx32	DEAH (Asp-Glu-Ala-His) box polypeptide 32	49.07478	51.75047	45.51121	51.61362	43.16679	45.10796
17265432	Dhx33	DEAH (Asp-Glu-Ala-His) box polypeptide 33	43.7248	39.12069	41.08671	40.80234	41.1266	41.6721
17486834	Dhx34	DEAH (Asp-Glu-Ala-His) box polypeptide 34	16.26276	15.76256	16.38291	17.7279	16.20054	16.38291
17378964	Dhx35	DEAH (Asp-Glu-Ala-His) box polypeptide 35	17.77663	20.40315	25.69294	22.77643	20.67277	21.11037
17405513	Dhx36	DEAH (Asp-Glu-Ala-His) box polypeptide 36	107.1368	82.72492	78.60341	92.65841	85.07716	93.34624
17452989	Dhx37	DEAH (Asp-Glu-Ala-His) box polypeptide 37	24.88975	23.63069	24.4364	25.25978	23.63069	21.37244
17512780	Dhx38	DEAH (Asp-Glu-Ala-His) box polypeptide 38	30.12439	27.69589	27.98244	26.21117	31.00975	25.54519
17267393	Dhx40	DEAH (Asp-Glu-Ala-His) box polypeptide 40	78.50867	71.80389	75.05467	72.21567	82.62807	74.24541
17256828	Dhx8	DEAH (Asp-Glu-Ala-His) box polypeptide 8	55.47131	44.95252	42.64457	46.91671	55.21813	41.86086
17228204	Dhx9	DEAH (Asp-Glu-Ala-His) box polypeptide 9	170.1178	159.4953	133.9824	129.8543	149.3538	143.3015
17459903	Dqx1	DEAQ RNA-dependent ATPase	9.269683	13.22797	18.3354	19.58314	16.25329	18.01179
17406867	Dap3	death associated protein 3	30.3211	33.69868	32.26325	35.77699	33.90627	33.90627
17287984	Dapk1	death associated protein kinase 1	140.4118	157.1959	154.3134	176.4249	158.4232	117.7718
17371011	Dapl1	death associated protein-like 1	3.421408	3.98017	4.235816	4.535369	4.141777	4.034193
17437574	Dthd1	death domain containing 1	5.008331	4.097006	3.682614	4.260728	3.816526	3.932345
17219286	Dedd	death effector domain-containing	39.36932	39.36932	45.1139	43.00463	39.36932	39.36932
17487876	Dedd2	death effector domain-containing DNA binding protein 2	32.59797	35.86091	30.37221	36.97479	25.98865	35.92559
17395658	Dido1	death inducer-obliterator 1	52.89429	62.57958	54.88652	52.49762	54.42132	55.25488
17310808	Dap	death-associated protein	402.3201	508.8159	695.7764	450.5521	553.2777	503.2181
17518680	Dapk2	death-associated protein kinase 2	22.41729	19.30189	25.30271	19.98132	15.79877	19.09933
17235584	Dapk3	death-associated protein kinase 3	41.16111	35.65197	44.16771	39.57342	52.21962	40.42007
17520722	Dbr1	debranching enzyme homolog 1 (S. cerevisiae)	4.478079	4.914178	5.277007	5.567077	5.235764	5.876634
17525342	Dcps	decapping enzyme, scavenger	17.8313	20.78902	20.21438	15.81312	18.50111	23.17874

17336725	Dxo	decapping exoribonuclease	25.21744	18.54541	27.38056	26.11234	26.70115	20.74239
17236800	Dcn	decorin	363.6559	469.3792	432.735	359.0946	255.9556	330.6268
17484167	Dock1	dedicator of cytokinesis 1	32.2094	42.01318	49.08363	41.02297	40.09777	34.22242
17224813	Dock10	dedicator of cytokinesis 10	23.56249	26.89079	30.92818	21.11491	23.85798	22.27325
17533994	Dock11	dedicator of cytokinesis 11	7.534595	6.992896	8.713909	6.831489	7.751578	6.712732
17261650	Dock2	dedicator of cyto-kinesis 2	15.40311	13.63989	14.31296	15.72818	13.39888	13.59901
17530805	Dock3	dedicator of cyto-kinesis 3	4.936573	4.684808	4.034556	4.772384	4.443696	4.490263
17275168	Dock4	dedicator of cytokinesis 4	72.85217	71.67808	70.97259	71.24911	73.48715	67.05625
17307991	Dock5	dedicator of cytokinesis 5	44.33088	47.33449	46.62785	46.62785	47.00036	44.90549
17524775	Dock6	dedicator of cytokinesis 6	17.61216	21.43193	25.61346	18.44528	17.93642	16.47778
17427494	Dock7	dedicator of cytokinesis 7	35.02784	32.87844	37.77867	26.82619	36.03308	33.32863
17358274	Dock8	dedicator of cytokinesis 8	65.77056	70.94308	77.49761	71.49002	72.16113	61.1237
17309580	Dock9	dedicator of cytokinesis 9	30.55825	33.4557	30.35264	26.09154	24.98777	26.79942
17492289	Det1	de-etiolated homolog 1 (Arabidopsis)	13.90305	11.56906	16.73759	10.95274	10.50431	7.781995
17317115	Dscc1	defective in sister chromatid cohesion 1 homolog (S. cerevisiae)	3.119079	3.232006	3.483192	2.988725	3.309355	3.559809
17306333	Dad1	defender against cell death 1	435.7238	510.6532	507.1856	392.0055	411.5517	404.9697
17499808	Defb1	defensin beta 1	4.269331	6.282288	6.639249	5.826987	7.636174	5.971527
17499823	Defb10	defensin beta 10	2.959944	2.67402	2.481049	2.990942	3.024738	3.207124
17507945	Defb11	defensin beta 11	1.996357	2.802841	2.120773	2.358468	2.388508	2.665489
17507842	Defb12	defensin beta 12	2.406006	2.281385	2.304206	3.017367	2.041996	2.268486
17499831	Defb13	defensin beta 13	4.335024	3.580075	3.602254	5.223222	4.392249	3.02927
17499591	Defb14	defensin beta 14	3.912688	3.724586	3.376082	4.075763	3.920343	4.125188
17507950	Defb15	defensin beta 15	3.714299	4.464473	5.92843	3.857484	5.366866	3.255479
17221533	Defb18	defensin beta 18	4.28163	3.285129	3.299437	3.908432	3.521744	4.523972
17392968	Defb19	defensin beta 19	6.042644	4.350587	4.919597	5.433644	7.325057	4.864158
17499818	Defb2	defensin beta 2	4.791542	3.454196	6.903701	4.038669	6.819425	2.752476
17377723	Defb20	defensin beta 20	3.045481	3.039239	2.923872	2.614998	3.403172	2.54875
17377734	Defb21	defensin beta 21	5.382178	2.847785	2.778218	3.38311	4.924953	3.832299
17392951	Defb22	defensin beta 22	2.662442	3.042358	3.037617	3.049431	2.866241	3.031874
17392947	Defb23	defensin beta 23	6.106476	6.895236	6.445222	6.450227	5.567656	5.68965
17392980	Defb25	defensin beta 25	7.409006	7.362011	7.00085	7.153107	6.900926	8.552641
17392956	Defb26	defensin beta 26	3.219753	2.914907	2.392717	2.935141	2.586839	2.70883
17377728	Defb28	defensin beta 28	3.703322	4.397032	3.524503	4.19555	3.263862	3.703322
17392959	Defb29	defensin beta 29	9.123011	7.817948	7.858043	9.019766	9.310251	8.407576
17499613	Defb3	defensin beta 3	3.959011	3.335377	3.345009	3.806038	3.882296	2.874737
17307580	Defb30	defensin beta 30	4.473084	4.392493	4.392493	4.661874	4.392493	4.415908
17499706	Defb33	defensin beta 33	7.386034	5.628927	4.926763	5.496335	5.768115	6.331336
17499576	Defb34	defensin beta 34	2.782767	2.582109	2.67402	2.582109	4.422463	2.847429
17377738	Defb36	defensin beta 36	10.87369	12.54187	14.10515	11.2305	13.92601	13.38876
17507829	Defb37	defensin beta 37	2.482855	2.168744	2.168744	3.119706	2.234172	2.285991
17507834	Defb38	defensin beta 38	2.204269	1.804451	1.941296	2.088417	2.076509	2.598862
17507839	Defb39	defensin beta 39	2.52251	2.580875	2.373128	2.474145	2.419837	3.437691
17499594	Defb4	defensin beta 4	21.65953	25.22776	25.23755	18.0722	25.22776	29.2104
17507825	Defb40	defensin beta 40	3.034292	2.458469	1.974682	2.393646	2.996836	2.606909
17301200	Defb42	defensin beta 42	2.592385	1.972493	2.131354	2.458111	2.827486	2.620387
17301195	Defb43	defensin beta 43	4.743691	4.972185	5.196787	7.122482	5.642091	6.575133
17221531	Defb44-ps	defensin beta 44, pseudogene	2.241524	2.234032	2.045509	2.45139	2.53211	2.372108
17392973	Defb45	defensin beta 45	5.48933	5.48933	4.751819	4.52667	6.066313	6.347815
17499607	Defb46	defensin beta 46	2.544267	2.590553	2.475757	3.01584	2.82044	3.293633
17301192	Defb47	defensin beta 47	10.05311	7.566573	9.256264	12.46378	10.31449	9.732834
17307575	Defb48	defensin beta 48	4.792572	4.801649	5.160712	5.19812	4.948873	4.918574
17499703	Gm21944	defensin beta 48 pseudogene	2.406006	2.657408	2.336842	2.710295	2.406006	2.464013
17499610	Defb5	defensin beta 5	4.385496	4.598043	4.404872	5.179705	5.77872	4.034333
17499813	Defb50	defensin beta 50	2.337814	2.483974	2.937523	3.475065	2.346987	2.608843
17499602	Defb6	defensin beta 6	2.90728	3.999861	3.374936	3.448001	4.006521	3.26038
17499622	Defb7	defensin beta 7	18.01317	24.21585	20.29525	23.35565	21.57891	25.29201
17507847	Defb8	defensin beta 8	7.23323	6.716828	6.685243	5.751347	7.660189	7.19792
17507940	Defb9	defensin beta 9	6.40625	5.458294	4.945032	6.381121	8.596146	6.261507
17499792	Defa17	defensin, alpha, 17	3.29642	2.639638	4.342753	3.692812	3.907484	5.1169
17499768	Defa20	defensin, alpha, 20	8.61822	13.32257	10.79873	10.37256	9.027209	11.48389
17499710	Defa21	defensin, alpha, 21	15.51563	11.35133	9.077909	10.87196	13.88206	9.347496
17499739	Defa22	defensin, alpha, 22	5.121868	3.950184	5.208652	5.046174	6.449199	4.233409
17499728	Defa23	defensin, alpha, 23	7.786905	8.166738	7.160151	8.401052	7.088891	7.786905
17499744	Defa23	defensin, alpha, 23	7.870689	8.521752	7.237192	8.491445	7.083488	7.760502
17499803	Defa24	defensin, alpha, 24	12.85814	13.37262	10.20844	10.48954	13.86398	10.90033
17499736	Defa25	defensin, alpha, 25	6.790416	7.77283	7.810094	7.676454	7.444732	7.818436
17499788	Defa26	defensin, alpha, 26	7.734082	8.193671	10.02027	6.860435	9.197871	9.989621
17499757	Defa3	defensin, alpha, 3	12.92973	25.78482	28.61124	19.0347	19.62866	19.49537
17499761	Defa5	defensin, alpha, 5	4.790346	5.65411	4.681043	4.642011	5.694188	4.681043
17499800	Defa-ps1	defensin, alpha, pseudogene 1	3.741716	3.636171	3.703322	3.703322	4.308095	3.89464
17499599	Defa-ps12	defensin, alpha, pseudogene 12	2.544179	2.907965	2.301621	3.312155	2.953407	3.41378
17499617	Defa-ps13	defensin, alpha, pseudogene 13	1.833576	2.190515	2.133526	2.261218	2.068307	2.778815
17507910	Defa-rs1	defensin, alpha, related sequence 1	10.27909	13.52391	11.24366	11.50086	8.718259	8.420524
17497854	Deaf1	deformed epidermal autoregulatory factor 1 (Drosophila)	38.70613	34.64055	36.96249	35.66211	32.89166	35.04872
17230595	Degs1	degenerative spermatocyte homolog 1 (Drosophila)	110.9203	105.0943	100.3454	102.3608	89.39362	109.039
17283915	Degs2	degenerative spermatocyte homolog 2 (Drosophila), lipid desaturase	6.801345	6.646892	6.801345	7.007501	8.152994	7.377541
17431152	Dhdds	dehydrodolichyl diphosphate synthase	62.05132	55.33499	47.57033	47.79938	44.03931	47.79739
17381424	Dhtkd1	dehydrogenase E1 and transketolase domain containing 1	47.05545	69.68856	76.30047	76.5139	66.68082	93.75281
17306856	Dhrs1	dehydrogenase/reductase (SDR family) member 1	212.9041	252.6611	237.1671	220.8159	185.7618	202.6407
17267066	Dhrs11	dehydrogenase/reductase (SDR family) member 11	93.82757	89.78982	88.3446	89.78982	79.5875	74.05935
17253422	Dhrs13	dehydrogenase/reductase (SDR family) member 13	5.239685	6.287864	6.251143	5.759486	6.287864	8.371522
17266319	Dhrs13os	dehydrogenase/reductase (SDR family) member 13, opposite strand	4.657514	4.221103	4.193137	4.635034	3.398976	5.145032
17421312	Dhrs3	dehydrogenase/reductase (SDR family) member 3	193.3619	183.964	182.8631	198.7113	181.5068	220.4366
17300429	Dhrs4	dehydrogenase/reductase (SDR family) member 4	283.8195	291.6572	285.6312	238.1274	250.3374	256.412

17281843	Dhrs7	dehydrogenase/reductase (SDR family) member 7	35.88328	42.73985	51.96434	36.63219	45.02049	44.44133
17250519	Dhrs7b	dehydrogenase/reductase (SDR family) member 7B	54.16954	43.09444	45.31039	43.16111	51.53783	42.27843
17251264	Dhrs7c	dehydrogenase/reductase (SDR family) member 7C	6.595811	4.126933	3.703322	5.031435	3.729055	4.498519
17371427	Dhrs9	dehydrogenase/reductase (SDR family) member 9	53.41007	31.70723	36.04807	28.32256	20.87986	17.50494
17434073	Dhrsx	dehydrogenase/reductase (SDR family) X chromosome	29.29414	30.42447	26.73912	32.53589	31.11655	36.24525
17300416	Dhrs2	dehydrogenase/reductase member 2	5.48933	5.48933	5.574838	5.213998	5.047259	5.333971
17278890	Dio3	deiodinase, iodothyronine type Ⅲ	3.563364	3.788036	3.471069	3.430931	3.13912	2.812572
17283976	Dio3os	deiodinase, iodothyronine type Ⅲ, opposite strand	11.65858	17.18324	14.06161	12.17424	11.60078	17.84453
17427928	Dio1	deiodinase, iodothyronine, type I	230.0032	180.1906	261.7199	285.0755	267.4953	253.8021
17282970	Dio2	deiodinase, iodothyronine, type Ⅱ	4.414928	5.481193	5.020531	4.206936	3.261261	5.423973
17292276	Dek	DEK oncogene (DNA binding)	50.56124	54.63146	51.43042	46.39894	55.8436	52.56498
17345880	Dazl	deleted in azoospermia-like	3.457837	4.01475	4.080483	4.674623	4.444312	3.326166
17355332	Dcc	deleted in colorectal carcinoma	4.282965	4.172349	4.20927	4.180223	4.113852	5.516296
17508804	Dlc1	deleted in liver cancer 1	66.92902	72.16113	76.85248	72.14412	78.21861	59.09032
17522998	Dlec1	deleted in lung and esophageal cancer 1	3.995428	7.349367	6.287864	6.794889	5.989934	7.332016
17307502	Dleu2	deleted in lymphocytic leukemia, 2	18.81146	15.66399	22.16129	22.98833	19.53162	29.25599
17547825	Dleu2	deleted in lymphocytic leukemia, 2 [Source:MGI Symbol;Acc:MGI:1934030]	14.57749	23.20735	19.28129	19.44396	23.64937	23.41401
17307522	Dleu7	deleted in lymphocytic leukemia, 7	4.20927	5.539478	3.905507	4.829822	3.405791	4.351944
17483925	Dmbt1	deleted in malignant brain tumors 1	2.259463	3.049579	3.049579	2.938643	3.049579	2.996545
17359858	Dpcd	deleted in primary ciliary dyskinesia	24.50697	19.42873	17.18645	17.53202	21.08449	22.43983
17224977	Dner	delta/notch-like EGF repeat containing	4.94517	3.460331	4.20927	4.20927	3.508879	5.010692
17341179	Dll1	delta-like 1 (Drosophila)	10.09193	10.01444	8.112013	10.70298	9.948921	10.86458
17278647	Dlk1	delta-like 1 homolog (Drosophila)	5.009581	5.069347	5.775276	5.54305	5.069347	4.191946
17338071	Dlk2	delta-like 2 homolog (Drosophila)	9.843627	10.11988	9.17215	7.31709	7.364206	8.947037
17488379	Dll3	delta-like 3 (Drosophila)	6.557155	5.117574	5.511748	6.1597	5.451526	4.577056
17374792	Dll4	delta-like 4 (Drosophila)	12.15544	12.76481	16.59803	12.82138	12.59143	11.4999
17452038	Dtx1	deltex 1 homolog (Drosophila)	22.32085	13.82119	15.37101	15.37101	11.91858	15.13388
17443363	Dtx2	deltex 2 homolog (Drosophila)	13.48684	13.78283	15.27425	14.20218	12.84976	13.47945
17245772	Dtx3	deltex 3 homolog (Drosophila)	10.73024	12.59029	16.2525	11.12991	9.310251	11.24366
17330119	Dtx3l	deltex 3-like (Drosophila)	78.33526	72.54428	74.63653	85.98601	62.81077	73.2131
17363086	Dtx4	deltex 4 homolog (Drosophila)	32.76106	29.13134	25.83993	31.63435	24.93534	25.60884
17308413	Dmtn	dematin actin binding protein	7.919119	7.406644	6.992896	6.992896	7.277283	6.702132
17495553	Coq7	demethyl-Q 7	30.18668	31.63391	30.23086	37.03378	45.78173	41.76869
17334166	Dcpp1	demilune cell and parotid protein 1	6.47666	5.871219	6.110795	5.402548	4.494373	3.792003
17334172	Dcpp2	demilune cell and parotid protein 2	18.16312	9.903716	15.23914	19.69326	16.54152	15.31008
17334178	Dcpp3	demilune cell and parotid protein 3	8.060727	10.67349	11.55704	15.98825	11.08249	11.15694
17321346	Ddn	dendrin	4.115934	7.181424	4.638859	7.14607	5.203924	5.550547
17384659	Dennd1a	DENN/MADD domain containing 1A	47.33056	56.81776	59.1481	49.50938	37.80775	41.46005
17217854	Dennd1b	DENN/MADD domain containing 1B	34.84211	31.15172	29.60153	31.29434	30.56122	32.31988
17327445	Dennd1b	DENN/MADD domain containing 1B	3.462322	3.462322	3.462322	3.260312	3.462322	3.630202
17346477	Dennd1c	DENN/MADD domain containing 1C	9.108289	12.50133	10.05004	8.51685	6.301783	8.372799
17466142	Dennd2a	DENN/MADD domain containing 2A	8.274947	7.698994	9.290911	9.488237	11.05364	9.290911
17401144	Dennd2c	DENN/MADD domain containing 2C	3.432715	4.20927	4.20927	4.509633	4.834075	4.33809
17401480	Dennd2d	DENN/MADD domain containing 2D	13.32358	13.4717	16.29808	12.66127	14.60186	14.13118
17312097	Dennd3	DENN/MADD domain containing 3	14.83847	17.09864	16.76302	14.20296	15.28919	16.82681
17518378	Dennd4a	DENN/MADD domain containing 4A	100.001	107.7661	107.687	91.32956	103.3855	97.62779
17399702	Dennd4b	DENN/MADD domain containing 4B	13.75973	12.84683	13.524	13.25339	13.524	14.39943
17415181	Dennd4c	DENN/MADD domain containing 4C	81.45267	91.15753	79.73217	77.07174	76.58606	79.69239
17494978	Dennd5a	DENN/MADD domain containing 5A	83.41818	75.89064	81.94136	67.40903	58.47386	58.67199
17473061	Dennd5b	DENN/MADD domain containing 5B	104.4175	132.1562	131.096	108.2002	148.0038	126.6365
17297889	Dennd6a	DENN/MADD domain containing 6A	80.8648	82.1609	78.50268	82.1609	80.29677	75.73824
17320389	Dennd6b	DENN/MADD domain containing 6B	14.98451	12.56693	16.16924	14.97184	14.18211	14.34603
17442401	Denr	density-regulated protein	102.1539	87.03417	89.72947	84.67123	104.6878	80.89452
17231066	Dtl	denticleless homolog (Drosophila)	3.439908	3.664408	3.885742	3.622335	4.350647	3.622335
17439805	Dmp1	dentin matrix protein 1	5.36709	4.24231	4.293666	3.699858	4.867463	4.137531
17439797	Dspp	dentin sialophosphoprotein	5.595318	5.795809	5.626899	5.120306	3.940121	5.62881
17311251	Dcstamp	dentrocyte expressed seven transmembrane protein	5.958173	6.189187	4.846825	5.392185	5.972066	5.820044
17438823	Dck	deoxycytidine kinase	16.26264	15.20686	15.35972	15.17737	15.43464	14.19716
17468172	Dguok	deoxyguanosine kinase	29.07385	31.33168	27.05831	28.12907	28.40041	23.7226
17235714	Dohh	deoxyhypusine hydroxylase/monooxygenase	13.63998	13.39423	13.39423	16.08942	13.39423	13.29766
17503377	Dhps	deoxyhypusine synthase	8.001886	9.351061	9.668888	12.08345	11.50939	10.78213
17359421	Dntt	deoxynucleotidyltransferase, terminal	4.69121	5.229453	5.235473	4.620824	4.704952	5.388748
17379503	Dnttip1	deoxynucleotidyltransferase, terminal, interacting protein 1	48.70946	51.393	53.98213	60.63337	56.43389	49.82266
17402296	Dnttip2	deoxynucleotidyltransferase, terminal, interacting protein 2	30.73797	33.04477	33.55952	35.45482	25.51493	27.32955
17542642	Dnase1l1	deoxyribonuclease 1-like 1	13.30319	14.03007	15.23914	15.06815	11.56401	13.0165
17341843	Dnase1l2	deoxyribonuclease 1-like 2	6.327125	6.644037	6.402609	7.918735	7.596106	6.964936
17303386	Dnase1l3	deoxyribonuclease 1-like 3	126.7577	105.8274	161.4944	116.827	65.48942	86.55348
17322507	Dnase1	deoxyribonuclease I	7.707484	8.781394	7.840904	7.29678	6.916777	7.29678
17503260	Dnase2a	deoxyribonuclease Ⅱ alpha	30.7644	30.83507	28.45361	24.63794	20.84429	18.28197
17411020	Dnase2b	deoxyribonuclease Ⅱ beta	69.42291	67.9099	68.34387	48.48646	82.62807	69.42291
17225785	Dtymk	deoxythymidylate kinase	22.28715	20.10065	21.98747	21.81124	22.35553	22.65315
17375641	Dut	deoxyuridine triphosphatase	28.55715	33.37972	28.35674	33.06654	26.9406	29.20857
17375651	Dut	deoxyuridine triphosphatase	2.76759	2.406006	2.223404	2.458111	2.493773	2.607523
17403919	Depdc1a	DEP domain containing 1a	2.640608	2.557457	3.146788	2.860565	3.181308	2.953407
17289728	Depdc1b	DEP domain containing 1B	4.184311	4.526638	4.248431	3.263862	4.454736	3.568555
17436345	Depdc5	DEP domain containing 5	30.56694	24.77426	28.00534	28.94495	20.39665	27.58343
17388974	Depdc7	DEP domain containing 7	59.79819	55.16113	45.1887	54.23228	60.8152	58.56107
17311533	Deptor	DEP domain containing MTOR-interacting protein	260.4062	268.0354	290.1248	272.3164	236.2647	239.1546
17547877	Deptor	DEP domain containing MTOR-interacting protein	128.6395	165.0913	145.4581	164.9769	170.7616	163.9725
17270398	Dcakd	dephospho-CoA kinase domain containing	58.90222	58.90222	63.37096	58.90222	49.5444	56.69855
17317203	Derl1	Der1-like domain family, member 1	254.5138	217.6219	191.9744	183.1612	221.3415	157.2722
17265451	Derl2	Der1-like domain family, member 2	62.38987	43.94813	39.84371	47.24959	47.24959	52.41726
17234423	Derl3	Der1-like domain family, member 3	14.70587	14.2274	8.702139	8.367287	6.794041	7.134093
17240190	Dse	dermatan sulfate epimerase	21.03179	24.70943	28.29451	19.60446	17.67453	22.07345

17226142	Dsel	dermatan sulfate epimerase-like	7.182719	6.091257	5.617429	4.924441	3.681822	5.585707
17218927	Dpt	dermatopontin	21.37837	17.67955	25.69775	26.73893	14.75948	19.93815
17476535	Dmkn	dermokine	5.080604	4.54585	5.222715	4.54585	4.549632	4.587697
17321434	Dhh	desert hedgehog	5.792355	5.792355	6.834094	6.279066	5.464881	5.159531
17214476	Des	desmin	7.244871	12.35189	13.78054	10.70045	8.283899	9.865759
17352957	Dsc1	desmocollin 1	4.191539	3.199619	3.263862	3.686419	3.148686	2.944433
17352933	Dsc2	desmocollin 2	81.14442	79.76755	81.82615	78.28533	93.49064	97.18825
17352913	Dsc3	desmocollin 3	3.302274	2.968821	3.298957	3.753353	3.753353	4.421635
17348726	Dsg1a	desmoglein 1 alpha	6.293532	6.227961	4.789583	7.807063	5.849162	4.705474
17348745	Dsg1b	desmoglein 1 beta	3.155897	4.151981	3.708228	3.20499	3.268639	2.865884
17348707	Dsg1c	desmoglein 1 gamma	2.911071	3.25977	2.344808	3.375193	6.57992	4.886969
17348812	Dsg2	desmoglein 2	180.5119	198.7499	192.2833	189.0796	229.855	185.8945
17348791	Dsg3	desmoglein 3	3.662072	3.808044	3.98246	3.97226	4.87844	4.280502
17348765	Dsg4	desmoglein 4	3.830148	3.731097	5.283339	3.345774	7.082605	3.84779
17286641	Dsp	desmoplakin	91.14743	97.43377	85.6333	81.6941	92.90923	77.91828
17377013	Dstn	destrin	344.9611	358.9303	384.5534	361.4444	370.2433	364.3421
17319565	Desi1	desumoylating isopeptidase 1	70.24677	60.0266	61.95677	58.57568	61.14828	63.92817
17219963	Desi2	desumoylating isopeptidase 2	13.09442	12.70188	12.86206	10.36336	10.52217	9.228461
17219974	Desi2	desumoylating isopeptidase 2	5.100399	5.100399	5.196391	7.073038	5.100399	4.437171
17502196	Dda1	DET1 and DDB1 associated 1	26.29178	27.79725	23.99513	25.86645	20.49287	26.59181
17491396	Dbx1	developing brain homeobox 1	6.619811	6.44835	7.294302	7.274459	6.992896	6.289608
17320844	Dbx2	developing brain homeobox 2	3.506423	4.003911	3.908513	3.692659	4.108694	3.540149
17262127	Dppa1	developmental pluripotency associated 1	3.03158	3.445683	2.751503	4.910773	3.736817	3.445683
17326021	Dppa2	developmental pluripotency associated 2	4.697034	3.851766	4.723349	4.553765	3.950869	4.857756
17326011	Dppa4	developmental pluripotency associated 4	6.321557	6.971939	8.119776	4.748889	5.690084	5.992218
17529029	Dppa5a	developmental pluripotency associated 5A	5.178162	6.398217	5.481725	5.564916	5.331753	5.409968
17462686	Dppa3	developmental pluripotency-associated 3	9.158365	8.160797	5.71257	8.467934	8.710104	9.257355
17259748	Drg1	developmentally regulated GTP binding protein 1	70.89146	73.48613	67.01165	76.17945	88.36542	75.72827
17250374	Drg2	developmentally regulated GTP binding protein 2	76.99538	75.27923	73.7433	76.31792	74.52177	78.3521
17540376	Drr1	developmentally regulated repeat element-containing transcript 1 [Source:MGI Syı	4.426204	3.601006	4.607806	2.75074	3.960218	3.355691
17328093	Dexi	dexamethasone-induced transcript	84.97403	58.28691	43.7891	73.80261	55.62841	54.34178
17269595	Dhx58	DEXH (Asp-Glu-X-His) box polypeptide 58	23.69236	25.96111	23.2684	30.06494	23.79044	30.66413
17452615	Diablo	diablo homolog (Drosophila)	29.11156	30.42172	27.43584	28.68769	30.36211	27.47676
17532755	Dgkk	diacylglycerol kinase kappa	4.20927	4.20927	4.20927	4.451156	5.546432	4.649095
17388293	Dgkz	diacylglycerol kinase zeta	31.29434	29.78843	35.29349	31.71031	36.14314	28.90886
17246310	Dgka	diacylglycerol kinase, alpha	29.59907	36.81751	45.97922	48.78379	24.62907	35.47326
17275090	Dgkb	diacylglycerol kinase, beta	2.953407	2.646306	2.913837	3.263862	3.44683	3.040145
17215395	Dgkd	diacylglycerol kinase, delta	28.65133	34.7129	31.4701	27.74085	32.0973	32.38155
17267632	Dgke	diacylglycerol kinase, epsilon	4.343476	5.700467	5.700467	5.503846	5.436507	5.137975
17255042	Dgkeos	diacylglycerol kinase, epsilon, opposite strand	13.12622	12.9694	13.3553	16.26242	13.78178	13.33337
17308842	Dgkh	diacylglycerol kinase, eta	21.8762	26.95629	40.45483	22.87675	22.15853	21.3078
17329316	Dgkg	diacylglycerol kinase, gamma	3.696116	4.153075	3.486695	4.517016	4.189157	4.57287
17465865	Dgki	diacylglycerol kinase, iota	7.102466	7.238295	6.121775	7.587423	7.258342	7.587423
17450802	Dgkq	diacylglycerol kinase, theta	12.65916	12.10566	13.32801	13.8097	12.83383	13.09442
17362646	Dagla	diacylglycerol lipase, alpha	8.339553	9.915462	11.4158	9.196532	9.201825	10.05325
17444346	Daglb	diacylglycerol lipase, beta	63.11846	56.70799	53.45044	58.95	53.07866	59.66984
17318502	Dgat1	diacylglycerol O-acyltransferase 1	24.84563	22.92119	22.37305	25.49443	21.20364	32.10776
17493632	Dgat2	diacylglycerol O-acyltransferase 2	347.7604	373.3332	416.7909	411.1469	502.4548	479.5675
17536527	Dgat2l6	diacylglycerol O-acyltransferase 2-like 6	3.462322	2.768741	3.907105	2.988145	3.691866	3.171554
17458424	Doxl2	diamine oxidase-like protein 2	3.611179	4.05373	4.025171	4.287718	4.05373	4.05373
17353806	Diaph1	diaphanous related formin 1	67.03627	68.42777	78.98463	58.94878	65.85997	66.26614
17537537	Diaph2	diaphanous related formin 2	67.76695	59.16081	66.98982	69.24942	62.6004	69.17218
17308965	Diaph3	diaphanous related formin 3	5.125917	6.011021	5.402286	5.76268	5.73141	6.506583
17226302	Dbi	diazepam binding inhibitor	57.42432	58.20293	60.95658	50.38212	57.49003	61.0496
17253145	Dbil5	diazepam binding inhibitor-like 5	5.559519	5.300432	5.74565	5.970907	6.103091	5.423597
17273292	Dcxr	dicarbonyl L-xylulose reductase	54.88614	65.58755	65.53892	76.27086	85.08306	89.26174
17283691	Dicer1	dicer 1, ribonuclease type III	44.46105	55.37028	49.15002	58.46373	51.75513	53.97876
17363895	Dkk1	dickkopf homolog 1 (Xenopus laevis)	7.155041	7.890846	5.871219	7.416919	5.070894	6.495903
17402698	Dkk2	dickkopf homolog 2 (Xenopus laevis)	5.191675	5.48933	7.655783	5.48933	4.793967	6.435758
17495191	Dkk3	dickkopf homolog 3 (Xenopus laevis)	17.56766	16.09968	14.08698	15.4995	14.84628	12.07173
17499949	Dkk4	dickkopf homolog 4 (Xenopus laevis)	6.843005	6.423235	6.992896	6.843005	6.030928	6.843005
17490695	Dkkl1	dickkopf-like 1	5.173318	3.936436	4.379997	4.379997	4.197703	4.452668
17428501	Dmbx1	diencephalon/mesencephalon homeobox 1	3.902909	4.405147	4.569005	4.006327	5.148171	5.974011
17523326	Deb1	differentially expressed in B16F10 1	75.86381	58.02771	52.48343	62.93846	88.26625	84.13826
17335281	Def6	differentially expressed in FDCP 6	6.704548	6.830684	5.048903	5.963214	5.562333	6.557019
17506639	Def8	differentially expressed in FDCP 8	53.72308	49.7133	54.38813	53.03637	44.51563	53.57062
17438180	Dancr	differentiation antagonizing non-protein coding RNA	6.212397	6.992896	6.992896	7.030757	6.992896	7.127173
17344349	Dpcr1	diffuse panbronchiolitis critical region 1 (human)	6.349399	5.552471	5.983958	6.943774	6.019068	5.601332
17328810	Dgcr14	DiGeorge syndrome critical region gene 14	20.8388	23.80001	27.35192	24.64478	18.8322	26.29342
17328792	Dgcr2	DiGeorge syndrome critical region gene 2	55.1856	51.9701	60.1032	55.25641	51.71318	53.91781
17323632	Dgcr6	DiGeorge syndrome critical region gene 6	29.70513	33.18156	32.63776	28.45598	38.34828	31.06461
17328897	Dgcr8	DiGeorge syndrome critical region gene 8	18.51907	18.20307	22.12553	18.69188	19.21951	17.8376
17231188	Diexf	digestive organ expansion factor homolog (zebrafish)	23.94446	19.38623	24.05208	23.07502	24.78525	24.31088
17490848	Dhdh	dihydrodiol dehydrogenase (dimeric)	392.718	363.6886	307.8961	329.152	260.9645	289.47
17289085	Dhfr	dihydrofolate reductase	62.34708	54.0293	38.30551	50.1612	67.34178	61.0183
17402025	Dbt	dihydrolipoamide branched chain transacylase E2	214.9906	210.9606	209.4889	240.4228	237.814	272.2277
17280498	Dld	dihydrolipoamide dehydrogenase	341.1329	302.6256	275.6435	301.8044	287.5024	329.5469
17526861	Dlat	dihydrolipoamide S-acetyltransferase (E2 component of pyruvate dehydrogenase c	56.0946	46.19068	45.49859	53.26736	48.80848	62.34535
17277352	Dlst	dihydrolipoamide S-succinyltransferase (E2 component of 2-oxo-glutarate comple:	284.1601	304.2915	294.6279	320.6532	214.7389	275.2598
17512816	Dhodh	dihydroorotate dehydrogenase	16.59757	15.34791	15.2058	17.32904	16.74119	20.24467
17316739	Dpys	dihydropyrimidinase	245.1702	272.5808	272.5808	295.1613	316.9299	286.925
17307905	Dpysl2	dihydropyrimidinase-like 2	29.59907	32.17615	39.16112	32.53859	20.1019	30.34696
17354101	Dpysl3	dihydropyrimidinase-like 3	9.712482	8.304366	11.15741	9.620087	9.807259	7.487012
17548074	Dpysl3	dihydropyrimidinase-like 3	24.29454	24.42675	27.60504	23.02757	25.22776	24.95436

17484331	Dpysl4	dihydropyrimidinase-like 4	12.09769	10.53749	10.36307	10.15635	7.091938	10.14671
17435834	Dpysl5	dihydropyrimidinase-like 5	8.070343	7.698994	9.100653	7.68689	8.763942	9.102861
17402113	Dpyd	dihydropyrimidine dehydrogenase	695.2076	718.0945	716.2205	709.6186	811.6084	823.035
17273328	Dus1l	dihydrouridine synthase 1-like (S. cerevisiae)	85.01526	82.37759	75.08381	78.37328	72.89157	76.37561
17505013	Dus2	dihydrouridine synthase 2	19.8466	20.1619	20.1619	19.52648	20.1619	20.13942
17338882	Dus3l	dihydrouridine synthase 3-like (S. cerevisiae)	20.31326	20.70719	20.88406	17.41115	18.90348	20.14025
17280561	Dus4l	dihydrouridine synthase 4-like (S. cerevisiae)	10.67053	9.79829	8.836224	9.79829	9.153922	10.38026
17289660	Dimt1	DIM1 dimethyladenosine transferase 1-like (S. cerevisiae)	15.22151	16.26445	15.14427	18.29275	17.16526	15.94442
17403439	Ddah1	dimethylarginine dimethylaminohydrolase 1	335.7352	393.9449	386.2524	325.7091	285.1842	366.9348
17336906	Ddah2	dimethylarginine dimethylaminohydrolase 2	12.97974	10.03167	11.3711	11.75343	10.98163	13.01813
17289163	Dmgdh	dimethylglycine dehydrogenase precursor	437.0305	364.8349	406.5503	436.9578	556.1653	546.2736
17242046	Dip2a	DIP2 disco-interacting protein 2 homolog A (Drosophila)	9.999181	10.41665	10.99579	13.21899	9.46131	10.41665
17314929	Dip2b	DIP2 disco-interacting protein 2 homolog B (Drosophila)	18.06543	23.43398	20.83432	19.50457	22.53866	26.37447
17285097	Dip2c	DIP2 disco-interacting protein 2 homolog C (Drosophila)	39.78658	30.99041	39.70779	33.60304	35.67026	36.32976
17506532	Dpep1	dipeptidase 1 (renal)	7.771591	5.300432	5.300432	5.656149	4.408537	5.240484
17512611	Dpep2	dipeptidase 2	8.531799	8.162098	6.423858	6.749778	6.749778	6.087036
17512628	Dpep2	dipeptidase 2	9.658372	12.95449	11.60311	11.95473	24.31728	18.58775
17512598	Dpep3	dipeptidase 3	6.546437	6.288213	5.990556	5.882625	5.981387	5.751068
17226387	Dpp10	dipeptidylpeptidase 10	2.922514	3.103401	3.324736	3.080981	3.037722	3.348303
17361404	Dpp3	dipeptidylpeptidase 3	117.5035	101.1835	110.9249	103.5167	92.64813	87.44718
17385719	Dpp4	dipeptidylpeptidase 4	168.2439	180.9855	174.1661	172.5834	158.862	211.164
17435528	Dpp6	dipeptidylpeptidase 6	3.551578	4.296435	3.540124	3.173225	3.212063	3.670839
17382533	Dpp7	dipeptidylpeptidase 7	17.38764	20.29525	23.11857	20.29525	16.43409	15.75437
17518434	Dpp8	dipeptidylpeptidase 8	115.0726	116.1608	110.4141	105.5974	92.7278	111.142
17346185	Dpp9	dipeptidylpeptidase 9	71.69	67.20189	67.06881	70.6506	76.9207	61.78523
17390490	Ppip5k1	diphosphoinositol pentakisphosphate kinase 1	10.13933	11.71122	9.309218	8.948029	10.04176	11.35086
17225893	Ppip5k2	diphosphoinositol pentakisphosphate kinase 2	99.12314	72.98864	72.31685	89.91999	94.37809	75.05363
17367750	Dph7	diphthamine biosynethesis 7	6.380414	7.17252	7.161491	7.715288	7.261965	7.349876
17304850	Dph3	diphthamine biosynthesis 3	28.3314	24.51104	21.23467	26.27174	21.10086	29.01124
17389578	Dph6	diphthamine biosynthesis 6	29.54496	33.06676	28.86001	38.16452	27.05831	37.26734
17243157	Diras1	DIRAS family, GTP-binding RAS-like 1	23.52283	25.96111	22.9775	26.46897	19.64159	25.96111
17292598	Diras2	DIRAS family, GTP-binding RAS-like 2	6.82666	6.82666	6.968509	6.82666	6.735617	7.622583
17309070	Dis3	DIS3 mitotic control homolog (S. cerevisiae)	44.46166	42.19587	43.1647	36.95275	36.55609	34.08819
17528175	Dis3l	DIS3 mitotic control homolog (S. cerevisiae)-like	28.03583	28.03583	26.73523	28.21245	26.49448	28.03583
17215137	Dis3l2	DIS3 mitotic control homolog (S. cerevisiae)-like 2	27.09454	32.32929	27.68764	25.94815	24.95834	25.93197
17416162	Dab1	disabled 1	4.93962	5.300432	4.779832	6.440845	3.912742	4.693226
17370234	Dab2ip	disabled 2 interacting protein	15.33366	15.07567	15.85043	13.69417	16.81504	14.37958
17309935	Dab2	disabled 2, mitogen-responsive phosphoprotein	37.71143	45.59046	50.87588	31.50699	25.35995	30.08078
17344405	Ddr1	discoidin domain receptor family, member 1	28.29451	26.70245	35.54291	24.16254	19.85706	16.82355
17229481	Ddr2	discoidin domain receptor family, member 2	15.97562	12.58192	16.97595	12.08244	9.199338	8.759388
17233273	Dcbld1	discoidin, CUB and LCCL domain containing 1	14.39544	15.37101	16.93657	17.36439	13.44297	17.1774
17326405	Dcbld2	discoidin, CUB and LCCL domain containing 2	9.566956	8.516791	8.026828	8.516791	8.82747	8.516791
17339357	Dlgap1	discs, large (Drosophila) homolog-associated protein 1	6.171709	5.013958	6.760172	6.096453	7.030854	6.129545
17499412	Dlgap2	discs, large (Drosophila) homolog-associated protein 2	3.062691	3.362397	3.030425	3.18422	3.362397	3.291146
17418732	Dlgap3	discs, large (Drosophila) homolog-associated protein 3	6.470871	6.852735	6.122114	6.235391	6.235391	5.524026
17305789	Dlgap5	discs, large (Drosophila) homolog-associated protein 5	5.359803	6.145967	6.145967	6.145967	6.145967	7.180976
17324661	Dlg1	discs, large homolog 1 (Drosophila)	96.7232	99.76141	101.6538	103.2695	119.9243	111.4266
17480143	Dlg2	discs, large homolog 2 (Drosophila)	3.085427	3.143322	3.18219	3.279964	3.21302	3.18219
17536624	Dlg3	discs, large homolog 3 (Drosophila)	11.77292	13.5845	13.3933	14.28966	15.25775	16.05789
17251900	Dlg4	discs, large homolog 4 (Drosophila)	4.937224	5.392521	4.650417	5.390204	4.311172	5.427659
17304049	Dlg5	discs, large homolog 5 (Drosophila)	21.39497	22.20065	22.27386	22.20065	19.61438	22.2279
17378640	Dlgap4	discs, large homolog-associated protein 4 (Drosophila)	16.08618	16.90467	16.45792	15.41796	16.45792	18.62954
17251875	Dvl2	dishevelled 2, dsh homolog (Drosophila)	9.351709	9.670028	9.320065	8.647481	8.339784	9.277782
17323909	Dvl3	dishevelled 3, dsh homolog (Drosophila)	85.04061	67.83745	66.97543	68.05598	61.45038	54.79415
17276153	Daam1	dishevelled associated activator of morphogenesis 1	72.56641	67.49366	67.9212	68.24824	58.45927	63.63286
17345830	Daam2	dishevelled associated activator of morphogenesis 2	4.842527	7.121741	5.499346	6.044529	7.046467	6.662252
17422745	Dvl1	dishevelled, dsh homolog 1 (Drosophila)	59.41355	56.87844	62.52841	55.6585	56.96998	57.08976
17305514	LOC102634304	disks large homolog 5	5.519088	5.801959	5.93472	5.4399	6.985435	6.585486
17305456	LOC102633225	disks large homolog 5-like	49.05098	55.62031	48.309	56.14984	37.12091	54.86408
17305266	LOC102635554	disks large homolog 5-like	2.706428	2.953407	3.04259	2.953407	3.72877	3.688643
17299158	LOC102636873	disks large homolog 5-like	3.260063	2.313986	2.933311	2.933311	2.846896	4.071612
17305321	LOC102637484	disks large homolog 5-like	4.870264	5.346668	5.604866	7.090513	5.386395	6.499055
17305291	LOC102639794	disks large homolog 5-like	13.03573	11.4633	11.99549	12.19257	11.91692	10.79177
17305508	LOC102641378	disks large homolog 5-like	2.731475	3.229386	4.150744	2.955495	3.017618	3.729055
17305401	LOC105245735	disks large homolog 5-like	5.021506	4.844138	5.238268	4.991696	5.083951	6.130055
17230638	Disp1	dispatched homolog 1 (Drosophila)	8.265488	7.332372	8.87631	10.68133	5.480889	7.348501
17374618	Disp2	dispatched homolog 2 (Drosophila)	6.547254	8.469754	14.3078	10.11833	7.163775	10.48228
17330080	Dirc2	disrupted in renal carcinoma 2 (human)	87.49993	87.95782	81.69467	88.07435	74.5445	77.96907
17506862	Disc1	disrupted in schizophrenia 1	9.800396	8.427531	10.11763	9.903716	9.961065	9.20527
17371777	Dlx1	distal-less homeobox 1	4.824201	4.990692	6.085518	4.723971	4.34712	5.022515
17386426	Dlx1as	distal-less homeobox 1, antisense	4.801982	5.70608	4.723316	3.691277	4.256566	4.207753
17386433	Dlx2	distal-less homeobox 2	11.04629	10.15705	8.467112	6.640169	8.467112	7.238496
17255352	Dlx3	distal-less homeobox 3	6.559064	7.725402	7.122729	7.699795	7.456611	8.907743
17268169	Dlx4	distal-less homeobox 4	4.72325	7.872981	4.577976	5.840776	5.258386	6.067491
17464706	Dlx5	distal-less homeobox 5	3.159618	3.148621	2.926447	3.082947	3.168588	3.604402
17455936	Dlx6	distal-less homeobox 6	8.197477	8.733379	6.889074	10.45753	8.596623	7.698994
17464698	Dlx6os1	distal-less homeobox 6, opposite strand 1	3.920751	4.048339	3.928695	3.966427	3.069067	3.520377
17464704	Dlx6os2	distal-less homeobox 6, opposite strand 2	4.600562	6.915914	4.946129	6.209814	6.890554	5.467154
17526881	Dixdc1	DIX domain containing 1	6.306764	6.577503	7.419438	7.160349	8.029555	7.841502
17319248	Dmc1	DMC1 dosage suppressor of mck1 homolog, meiosis-specific homologous recomb	4.91721	5.66321	5.081766	5.650584	6.057322	5.68295
17427939	Dmrtb1	DMRT-like family B with proline-rich C-terminal, 1	7.36681	6.944544	6.958035	7.18018	6.944544	6.944544
17543753	Dmrtc1a	DMRT-like family C1a	8.212264	6.923493	9.326204	8.405828	7.986647	5.160425
17536895	Dmrtc1b	DMRT-like family C1b	2.563562	2.787941	2.737673	2.502691	3.069535	2.38831
17536933	Dmrtc1c2	DMRT-like family C1c2	12.23888	14.674	12.78473	13.59289	11.17862	13.90884

17543732	Dmrtc1c2	DMRT-like family C1c2	12.23888	14.674	12.78473	13.59289	11.17862	13.90884
17536922	Gm5166	DMRT-like family pseudogene	3.382805	3.470299	2.546455	3.502148	4.119788	3.372621
17350447	Dmxl1	Dmx-like 1	64.07413	58.14366	49.81989	59.74972	61.93745	52.14185
17527210	Dmxl2	Dmx-like 2	59.13376	55.83663	58.3965	71.77751	50.82795	58.51928
17234763	Dnmt3l	DNA (cytosine-5-)-methyltransferase 3-like	6.484476	6.110541	6.796632	6.439238	5.383521	6.296194
17276382	Dbpht2	DNA binding protein with his-thr domain	2.971415	3.095323	3.267167	2.940028	4.938079	4.775229
17365848	Dclre1a	DNA cross-link repair 1A, PSO2 homolog (S. cerevisiae)	29.604	33.49027	36.64209	29.34433	31.7279	33.95071
17408684	Dclre1b	DNA cross-link repair 1B, PSO2 homolog (S. cerevisiae)	16.99572	18.96977	13.50564	13.75696	16.16935	13.04342
17366399	Dclre1c	DNA cross-link repair 1C, PSO2 homolog (S. cerevisiae)	18.96227	17.75668	18.8437	18.8437	17.1188	10.81994
17366425	Dclre1c	DNA cross-link repair 1C, PSO2 homolog (S. cerevisiae)	2.924703	3.243901	3.243901	2.922919	3.208235	3.768029
17493358	Ddias	DNA damage-induced apoptosis suppressor	5.833089	5.779161	7.579328	6.762234	7.0198	9.15259
17421774	Dffa	DNA fragmentation factor, alpha subunit	28.27862	31.58855	31.39798	32.63663	22.98499	25.56893
17433543	Dffb	DNA fragmentation factor, beta subunit	6.042896	5.433644	5.433644	3.856788	6.404873	5.433644
17524535	Dnmt1	DNA methyltransferase (cytosine-5) 1	30.10477	35.2126	39.65498	32.31316	35.2126	31.62514
17428885	Dmap1	DNA methyltransferase 1-associated protein 1	10.48228	10.22529	10.73522	10.2311	12.05291	11.24366
17273652	Dnmt3a	DNA methyltransferase 3A	37.29447	37.29447	33.77655	32.23777	37.29447	35.97842
17279759	Dnmt3aos	DNA methyltransferase 3A, opposite strand	4.036402	4.843702	4.555976	4.288877	3.827812	3.904804
17377963	Dnmt3b	DNA methyltransferase 3B	8.607176	7.580589	7.837046	7.459661	7.153008	7.365482
17393092	Dnmt3bos	DNA methyltransferase 3B, opposite strand [Source:MGI Symbol;Acc:MGI:2685409]	14.38527	13.39423	13.39423	14.20099	17.16526	13.39423
17447185	Poln	DNA polymerase N	10.84035	8.388425	8.631612	9.500016	11.19715	9.697279
17238210	Prim1	DNA primase, p49 subunit	6.857012	9.768452	11.42601	8.468521	7.516859	7.222358
17222001	Prim2	DNA primase, p58 subunit	15.63752	14.67573	18.3321	15.84658	14.20168	15.39842
17233811	Dna2	DNA replication helicase 2 homolog (yeast)	18.68346	17.87548	18.2982	18.77642	20.2899	21.75176
17225888	D1Ertd622e	DNA segment, Chr 1, ERATO Doi 622, expressed	27.44707	26.49613	24.62787	19.84578	25.75392	24.62787
17220600	D1Pas1	DNA segment, Chr 1, Pasteur Institute 1	4.563403	6.330283	4.160826	4.299891	7.324956	4.648419
17242416	D10Jhu81e	DNA segment, Chr 10, Johns Hopkins University 81 expressed	107.9096	108.4616	93.58335	116.721	109.5207	118.8724
17235947	D10Wsu102e	DNA segment, Chr 10, Wayne State University 102, expressed	28.27627	22.13396	20.41716	23.652	19.44369	27.17221
17257994	D11Wsu47e	DNA segment, Chr 11, Wayne State University 47, expressed	8.27684	10.54714	10.07145	9.070047	15.86054	9.035034
17290190	D13Ertd608e	DNA segment, Chr 13, ERATO Doi 608, expressed	2.565109	2.180774	1.87309	2.423631	2.099928	2.305804
17311706	D15Ertd621e	DNA segment, Chr 15, ERATO Doi 621, expressed	130.0444	117.5117	99.97878	100.897	129.1461	120.7199
17331598	D16Ertd472e	DNA segment, Chr 16, ERATO Doi 472, expressed	37.07462	30.16283	25.87219	31.17354	28.04749	29.05874
17326588	D16Ertd519e	DNA segment, Chr 16, ERATO Doi 519, expressed	2.782767	2.886538	2.592259	2.592259	3.107211	2.898125
17340784	D17Ertd648e	DNA segment, Chr 17, ERATO Doi 648, expressed	4.985299	3.957831	3.951335	4.037941	3.907592	4.071047
17336980	D17H6S53E	DNA segment, Chr 17, human D6S53E	15.4907	16.40246	16.89062	16.67668	15.36632	18.4806
17344140	D17H6S56E-5	DNA segment, Chr 17, human D6S56E 5	24.68992	26.05973	42.11902	19.92641	24.1197	24.57842
17342785	D17Wsu92e	DNA segment, Chr 17, Wayne State University 92, expressed	254.3709	260.3755	273.6655	238.1786	233.7982	245.0173
17383571	D2Wsu81e	DNA segment, Chr 2, Wayne State University 81, expressed	40.92357	30.67263	34.18354	28.9271	43.5986	40.08332
17396966	D3Ertd254e	DNA segment, Chr 3, ERATO Doi 254, expressed	11.00578	11.64162	8.895835	12.0348	10.85073	11.64162
17397360	D3Ertd751e	DNA segment, Chr 3, ERATO Doi 751, expressed	9.43518	9.372085	9.043868	10.04747	8.938793	10.72243
17417808	D4Ertd617e	DNA segment, Chr 4, ERATO Doi 617, expressed	7.028759	6.622886	8.287	9.518868	6.88139	8.666083
17439319	D5Ertd577e	DNA segment, Chr 5, ERATO Doi 577, expressed	2.084816	2.483681	2.903776	2.527148	2.297333	3.517231
17447487	D5Ertd579e	DNA segment, Chr 5, ERATO Doi 579, expressed	23.24518	32.69436	28.96623	35.61739	33.06929	35.17674
17444851	D5Ertd605e	DNA segment, Chr 5, ERATO Doi 605, expressed	5.896219	5.614159	6.97866	8.134987	4.743822	5.639315
17447900	D5Ertd615e	DNA segment, Chr 5, ERATO Doi 615, expressed	5.995791	6.066902	6.679268	7.138545	7.661517	7.43545
17464175	D6Ertd474e	DNA segment, Chr 6, ERATO Doi 474, expressed	4.202565	3.837775	3.804297	3.607001	3.882511	3.911549
17460533	D6Ertd527e	DNA segment, Chr 6, ERATO Doi 527, expressed	5.656972	6.086277	6.45054	5.928799	6.362969	6.297503
17463316	D6Wsu163e	DNA segment, Chr 6, Wayne State University 163, expressed	33.09864	29.17134	26.52921	33.65689	26.81633	30.49984
17473113	D7Ertd143e	DNA segment, Chr 7, ERATO Doi 143, expressed	12.12691	12.00156	13.94427	11.11126	10.4306	11.83871
17497283	D7Ertd443e	DNA segment, Chr 7, ERATO Doi 443, expressed	5.402136	4.704104	5.029308	4.668729	5.156242	4.723218
17511096	D8Ertd738e	DNA segment, Chr 8, ERATO Doi 738, expressed	111.2499	99.05171	112.4944	115.7951	136.012	123.6047
17500595	D8Ertd82e	DNA segment, Chr 8, ERATO Doi 82, expressed	11.15718	10.03466	10.00355	11.24771	13.41161	9.310251
17542292	DXBay18	DNA segment, Chr X, Baylor 18	10.89519	10.48228	10.52757	10.80585	10.28436	12.87839
17318143	Top1mt	DNA topoisomerase 1, mitochondrial	22.69559	31.24796	32.7463	29.39849	22.73054	27.77105
17432287	Ddi2	DNA-damage inducible protein 2	210.6129	244.8747	224.6943	225.451	204.2638	227.323
17237978	Ddit3	DNA-damage inducible transcript 3	35.11949	24.25281	26.30819	23.16701	23.8213	23.28776
17243868	Dram1	DNA-damage regulated autophagy modulator 1	10.05625	9.65556	10.76122	8.231412	9.732834	9.732834
17401500	Dram2	DNA-damage regulated autophagy modulator 2	168.3827	201.6738	179.9834	185.9138	180.3793	169.5468
17241032	Ddit4	DNA-damage-inducible transcript 4	21.49471	26.7043	22.70708	19.96056	30.47554	38.72518
17402996	Ddit4l	DNA-damage-inducible transcript 4-like	8.751134	7.009541	6.022782	7.13711	6.707848	8.454386
17412774	Dnaja1	DnaJ (Hsp40) homolog, subfamily A, member 1	49.16296	51.13716	44.4481	45.49102	37.29447	41.34211
17511366	Dnaja2	DnaJ (Hsp40) homolog, subfamily A, member 2	232.3603	214.5084	198.2188	226.5334	189.9519	204.3318
17322541	Dnaja3	DnaJ (Hsp40) homolog, subfamily A, member 3	7.011776	7.929226	6.893898	8.106392	8.258845	8.842412
17322543	Dnaja3	DnaJ (Hsp40) homolog, subfamily A, member 3	6.96397	6.970296	5.954127	5.697899	6.906764	7.488724
17322545	Dnaja3	DnaJ (Hsp40) homolog, subfamily A, member 3	171.5886	183.5615	172.2894	193.7322	197.4878	175.3654
17517404	Dnaja4	DnaJ (Hsp40) homolog, subfamily A, member 4	11.7859	11.7859	12.52588	10.66491	11.7859	11.7859
17502954	Dnajb1	DnaJ (Hsp40) homolog, subfamily B, member 1	38.27631	25.18914	21.77484	20.49784	20.12811	20.97632
17324305	Dnajb11	DnaJ (Hsp40) homolog, subfamily B, member 11	173.8646	82.99601	84.19835	92.69245	114.6577	79.6946
17233586	Dnajb12	DnaJ (Hsp40) homolog, subfamily B, member 12	161.4261	144.7671	159.8517	145.2778	153.6112	144.98
17403013	Dnajb14	DnaJ (Hsp40) homolog, subfamily B, member 14	28.45756	27.16355	32.12178	27.16355	22.84205	26.14748
17214462	Dnajb2	DnaJ (Hsp40) homolog, subfamily B, member 2	33.27784	29.52858	29.27404	29.27404	27.96053	30.06973
17225325	Dnajb3	DnaJ (Hsp40) homolog, subfamily B, member 3	3.062691	4.563023	2.777698	3.918958	2.873343	2.559213
17411188	Dnajb4	DnaJ (Hsp40) homolog, subfamily B, member 4	18.68424	14.13284	12.90332	12.05834	12.95682	12.67874
17413191	Dnajb5	DnaJ (Hsp40) homolog, subfamily B, member 5	13.15993	13.51706	16.6515	14.55709	13.57039	15.25986
17435702	Dnajb6	DnaJ (Hsp40) homolog, subfamily B, member 6	18.63832	18.04754	18.49649	20.98592	19.09827	19.6514
17319484	Dnajb7	DnaJ (Hsp40) homolog, subfamily B, member 7	4.777911	3.043202	3.53983	3.627134	3.206257	3.399188
17460643	Dnajb8	DnaJ (Hsp40) homolog, subfamily B, member 8	6.418784	9.187103	8.871389	8.022156	8.022156	8.562428
17280893	Dnajb9	DnaJ (Hsp40) homolog, subfamily B, member 9	488.0212	192.6315	198.6452	183.5488	211.8369	132.7152
17539107	Gm6568	DnaJ (Hsp40) homolog, subfamily B, member 9 pseudogene	6.393208	5.089663	5.327394	5.593999	6.766032	3.999002
17382013	Dnajc1	DnaJ (Hsp40) homolog, subfamily C, member 1	32.49081	27.12216	29.99604	31.19775	30.90482	33.36862
17372392	Dnajc10	DnaJ (Hsp40) homolog, subfamily C, member 10	81.07302	57.76085	59.75966	48.05351	56.9842	48.51436
17422015	Dnajc11	DnaJ (Hsp40) homolog, subfamily C, member 11	73.14767	66.53216	72.49452	79.47284	71.95934	64.53366
17233920	Dnajc12	DnaJ (Hsp40) homolog, subfamily C, member 12	66.26155	41.81533	48.62782	38.31188	58.15092	41.98232
17530348	Dnajc13	DnaJ (Hsp40) homolog, subfamily C, member 13	67.53671	76.68434	71.4811	74.29432	71.25404	74.42317
17238571	Dnajc14	DnaJ (Hsp40) homolog, subfamily C, member 14	53.82782	53.56096	53.82782	53.82782	46.2256	49.20797

17308800	Dnajc15	DnaJ (Hsp40) homolog, subfamily C, member 15	60.90547	67.39829	59.20225	70.75254	82.58169	72.59307
17432299	Dnajc16	DnaJ (Hsp40) homolog, subfamily C, member 16	29.29454	26.00307	26.46548	22.56133	25.91005	23.40022
17389820	Dnajc17	DnaJ (Hsp40) homolog, subfamily C, member 17	6.764907	7.159555	7.206856	7.025009	6.939684	7.15276
17353639	Dnajc18	DnaJ (Hsp40) homolog, subfamily C, member 18	15.72633	16.38712	19.48605	22.21712	12.99757	13.79975
17404682	Dnajc19	DnaJ (Hsp40) homolog, subfamily C, member 19	24.33718	18.36326	23.66298	20.67062	24.90908	33.1611
17548620	Dnajc19-ps	DnaJ (Hsp40) homolog, subfamily C, member 19, pseudogene	230.7233	234.7758	231.5115	233.4403	223.3063	278.4376
17445869	Dnajc2	DnaJ (Hsp40) homolog, subfamily C, member 2	40.60231	38.73216	39.61926	49.72054	45.69454	43.83071
17315946	Dnajc21	DnaJ (Hsp40) homolog, subfamily C, member 21	46.83159	52.74381	47.69447	55.95441	59.41396	55.50901
17314729	Dnajc22	DnaJ (Hsp40) homolog, subfamily C, member 22	73.76323	62.78726	67.18932	69.55007	66.45197	71.7805
17389066	Dnajc24	DnaJ (Hsp40) homolog, subfamily C, member 24	19.22938	19.1184	18.13305	24.83323	14.94116	19.09628
17414416	Dnajc25	DnaJ (Hsp40) homolog, subfamily C, member 25	34.9781	37.82767	30.34633	36.00362	38.32224	31.52141
17273700	Dnajc27	DnaJ (Hsp40) homolog, subfamily C, member 27	6.006398	5.298889	7.133153	6.033144	6.293489	6.428847
17332133	Dnajc28	DnaJ (Hsp40) homolog, subfamily C, member 28	18.51715	17.2807	17.24684	17.92188	20.16107	17.8407
17302724	Dnajc3	DnaJ (Hsp40) homolog, subfamily C, member 3	610.2176	388.8042	348.6051	416.9961	429.7608	347.7845
17443181	Dnajc30	DnaJ (Hsp40) homolog, subfamily C, member 30	40.98204	55.42673	59.91187	57.54785	69.09742	48.93518
17362207	Dnajc4	DnaJ (Hsp40) homolog, subfamily C, member 4	40.06554	40.28414	39.60965	39.09386	37.66493	42.77838
17381058	Dnajc5	DnaJ (Hsp40) homolog, subfamily C, member 5	43.36532	42.10034	44.16097	37.16107	44.16097	44.87189
17396228	Dnajc5b	DnaJ (Hsp40) homolog, subfamily C, member 5 beta	7.29764	6.992896	5.919068	7.734189	7.723849	7.673102
17436035	Dnajc5g	DnaJ (Hsp40) homolog, subfamily C, member 5 gamma	4.036402	3.873828	3.089557	3.029543	3.67201	3.955143
17415943	Dnajc6	DnaJ (Hsp40) homolog, subfamily C, member 6	5.060956	5.193114	4.612823	5.466851	5.418937	5.933322
17269556	Dnajc7	DnaJ (Hsp40) homolog, subfamily C, member 7	55.07135	52.58648	64.2498	59.33371	59.5203	61.71546
17419423	Dnajc8	DnaJ (Hsp40) homolog, subfamily C, member 8	20.28301	17.15349	24.21065	18.93522	17.53311	19.35172
17303757	Dnajc9	DnaJ (Hsp40) homolog, subfamily C, member 9	16.70248	16.97159	20.69801	17.70249	16.97159	25.32903
17493772	Dnajb13	DnaJ (Hsp40) related, subfamily B, member 13	3.792712	4.77864	4.604677	4.556355	3.888059	5.920217
17382914	Dnlz	DNL-type zinc finger	45.91711	46.49263	43.49296	47.02774	52.96069	47.55384
17468067	Dok1	docking protein 1	5.970617	8.190491	7.770729	7.763838	7.198768	8.423735
17301802	Dok2	docking protein 2	6.069973	6.899587	9.384566	5.450921	8.350717	7.771376
17292915	Dok3	docking protein 3	11.41991	12.77375	10.73039	11.42712	9.473713	11.41991
17511900	Dok4	docking protein 4	7.371305	6.287864	6.287864	6.023408	7.44721	6.130055
17380060	Dok5	docking protein 5	4.034305	4.188722	4.601168	4.036011	4.22084	5.210132
17355892	Dok6	docking protein 6	3.505354	3.429053	3.009972	3.116961	3.263862	3.019668
17436810	Dok7	docking protein 7	4.97715	5.992508	5.093368	5.025022	5.153276	5.372896
17383612	Dolk	dolichol kinase	28.5083	24.62343	21.47491	22.36282	22.73779	25.44641
17394782	Dpm1	dolichol-phosphate (beta-D) mannosyltransferase 1	75.5343	68.26196	72.69328	68.4401	75.5343	77.49439
17369860	Dpm2	dolichol-phosphate (beta-D) mannosyltransferase 2	43.69027	45.71767	39.04673	36.29151	45.05702	42.5551
17369218	Dolpp1	dolichyl pyrophosphate phosphatase 1	37.64353	39.79182	39.10741	36.74841	36.02234	37.56117
17548049	LOC102632739	dolichyl-diphosphooligosaccharide--protein glycosyltransferase subunit 4-like	618.6673	565.8712	573.9455	528.8446	530.4526	478.3391
17420486	Ddost	dolichyl-di-phosphooligosaccharide-protein glycotransferase	326.023	228.8344	235.604	207.0134	267.3285	215.1263
17516546	Dpagt1	dolichyl-phosphate (UDP-N-acetylglucosamine) acetylglucosaminephosphotransfer	79.33579	72.35897	86.34316	63.02359	74.75924	63.19594
17399396	Dpm3	dolichyl-phosphate mannosyltransferase polypeptide 3	7.553786	7.80279	7.318003	7.970609	7.319931	8.103977
17260644	Ddc	dopa decarboxylase	89.66854	87.16095	77.21237	103.7121	72.23319	76.74921
17309385	Dct	dopachrome tautomerase	6.247331	5.691189	5.61307	5.312276	5.266192	5.879078
17368499	Dbh	dopamine beta hydroxylase	3.356412	4.39298	4.184224	4.20927	5.016496	4.120045
17383246	Dbhos	dopamine beta hydroxylase, opposite strand	5.461473	6.746691	5.592642	4.903834	5.48933	4.886969
17292677	Drd1	dopamine receptor D1	3.062691	3.450415	3.383039	2.969397	2.535869	2.953407
17517073	Drd2	dopamine receptor D2	3.910871	3.714402	3.760879	3.339148	5.369025	4.050051
17325725	Drd3	dopamine receptor D3	5.79617	7.916046	8.905891	6.795784	7.024084	7.376825
17484832	Drd4	dopamine receptor D4	10.4806	9.488237	9.488237	9.488237	11.05977	9.986852
17437107	Drd5	dopamine receptor D5	13.39423	15.49993	15.49993	14.35121	15.65194	15.69801
17519972	Dopey1	dopey family member 1	37.29447	42.71024	45.71387	42.92214	47.80071	45.07639
17327270	Dopey2	dopey family member 2	42.98466	52.56024	66.43355	43.56266	53.62114	55.27059
17432904	Draxin	dorsal inhibitory axon guidance protein	6.783078	6.44281	5.907305	6.454789	7.1675	8.536058
17235408	Dot1l	DOT1-like, histone H3 methyltransferase (S. cerevisiae)	42.91738	47.96101	50.46286	43.16111	32.77264	37.65005
17483146	Doc2a	double C2, alpha	9.66038	7.32054	6.992896	7.723582	7.723582	8.166399
17266010	Doc2b	double C2, beta	7.070097	6.505275	6.408204	6.497299	5.642834	6.645326
17356070	Doc2g	double C2, gamma	13.524	16.30124	16.99714	15.37101	14.85987	16.88792
17240963	Dux	double homeobox	2.116133	2.461504	2.465312	2.205843	2.499969	2.107219
17297784	Duxbl1	double homeobox B-like 1	10.12423	9.387884	8.615294	9.038354	11.47037	9.252736
17297817	Duxbl2	double homeobox B-like 2	5.442049	5.241465	4.864562	5.167226	5.350301	4.532415
17297849	Duxbl2	double homeobox B-like 2	5.570512	5.286489	5.120306	5.09411	5.919478	4.965366
17240957	LOC100504180	double homeobox protein 4-like	3.694938	3.114045	3.890161	3.538603	4.352065	3.004802
17392414	Dzank1	double zinc ribbon and ankyrin repeat domains 1	4.674202	3.77514	3.732727	4.297716	3.548133	3.732727
17545121	Dcx	doublecortin	3.599758	3.321581	3.977715	3.599758	3.735652	3.909624
17286087	Dcdc2a	doublecortin domain containing 2a	7.269368	7.766098	7.03334	9.661385	6.803938	8.5689
17430449	Dcdc2b	doublecortin domain containing 2b	17.52922	17.52922	12.53708	15.42213	16.12324	17.61448
17430451	Dcdc2b	doublecortin domain containing 2b	10.14291	9.182392	8.963114	11.95067	9.716993	9.027772
17280356	Dcdc2c	doublecortin domain containing 2C	4.06614	3.189588	3.086026	2.391059	2.815322	2.75964
17374000	Dcdc5	doublecortin domain containing 5	5.741191	7.077108	4.514418	5.386134	4.857655	5.544472
17397708	Dclk1	doublecortin-like kinase 1	4.2701	4.273891	4.246429	4.441047	4.149335	4.2701
17406460	Dclk2	doublecortin-like kinase 2	4.439662	4.828717	5.175973	5.144747	4.775097	5.059658
17522626	Dclk3	doublecortin-like kinase 3	12.75764	11.87833	12.96922	15.738	14.12256	14.94468
17358350	Dmrt1	doublesex and mab-3 related transcription factor 1	5.398692	4.638473	5.284987	6.449065	5.654698	5.615405
17358368	Dmrt2	doublesex and mab-3 related transcription factor 2	4.886969	5.16565	5.627991	4.992803	4.429427	4.20927
17358363	Dmrt3	doublesex and mab-3 related transcription factor 3	3.427698	3.163738	3.234718	3.339703	2.882898	4.19337
17415401	Dmrta1	doublesex and mab-3 related transcription factor like family A1	3.263862	3.263862	3.207679	3.106435	3.263862	4.17099
17416921	Dmrta2	doublesex and mab-3 related transcription factor like family A2	14.16806	12.33982	12.54569	13.55929	11.86031	12.21261
17428223	Dmrta2os	doublesex and mab-3 related transcription factor like family A2, opposite strand	11.13068	10.92681	10.49296	8.70039	13.80903	11.72341
17475101	Dmrtc2	doublesex and mab-3 related transcription factor like family C2	7.037632	9.20208	9.064202	10.04426	9.535047	10.3142
17536906	LOC102632256	doublesex- and mab-3-related transcription factor C1-like	2.626934	2.549104	2.775908	2.225994	3.37641	2.0661
17332495	Dscam	Down syndrome cell adhesion molecule	5.293456	5.324146	5.324146	5.354196	7.870471	5.775877
17516795	Dscaml1	Down syndrome cell adhesion molecule like 1	5.32112	5.796452	6.171709	6.171709	6.171709	7.716518
17332333	Dscr3	Down syndrome critical region gene 3	30.99342	29.48808	33.38898	32.58373	25.0018	30.92711
17440194	Dr1	down-regulator of transcription 1	65.3706	60.12445	59.09442	59.6533	59.71287	59.6533
17332166	Donson	downstream neighbor of SON	6.591789	7.698994	9.310251	8.461128	6.639893	8.451339

17428957	Dph2	DPH2 homolog (S. cerevisiae)	12.35771	10.521	10.60388	10.02444	10.36875	10.13869
17296009	Dph3b-ps	DPH3B, KTI11 homolog B (S. cerevisiae), pseudogene	4.288193	2.939845	3.709256	3.263862	3.429243	4.20927
17401987	Dph5	DPH5 homolog (S. cerevisiae)	18.85271	18.22302	18.22302	18.22302	18.98568	15.00582
17330905	Dubr	Dppa2 upstream binding RNA	5.338965	6.73529	6.327607	4.518237	6.145413	4.505259
17525007	Dpy19l1	dpy-19-like 1 (C. elegans)	125.7531	120.9536	101.9713	115.0351	110.5105	81.14667
17525036	Dpy19l2	dpy-19-like 2 (C. elegans)	5.043621	4.735117	4.988617	5.073706	5.222751	5.325068
17489824	Dpy19l3	dpy-19-like 3 (C. elegans)	20.66203	10.54794	12.22998	14.28679	14.31296	12.34213
17423265	Dpy19l4	dpy-19-like 4 (C. elegans)	27.55362	19.3693	18.86565	21.6995	21.84377	24.76619
17298946	Dydc1	DPY30 domain containing 1	5.64436	5.197436	4.981396	5.724977	6.888883	5.867191
17305235	Dydc2	DPY30 domain containing 2	5.008158	3.54506	3.38839	4.640017	2.749101	2.814893
17347222	Dpy30	dpy-30 homolog (C. elegans)	43.56235	48.93653	45.99611	46.27946	55.63497	46.47845
17361589	Drap1	Dr1 associated protein 1 (negative cofactor 2 alpha)	62.42059	63.37491	61.09829	69.92857	70.99768	74.54192
17292871	Dbn1	drebrin 1	18.00306	18.28147	15.72491	17.36523	18.58401	20.63097
17247023	Dbnl	drebrin-like	39.691	38.27552	36.97991	36.44932	29.16103	38.47235
17310466	Drosha	drosha, ribonuclease type III	22.59732	27.60294	26.12991	26.38855	26.82154	23.16364
17393620	Dsn1	DSN1, MIND kinetochore complex component, homolog (S. cerevisiae)	4.693682	4.590273	3.687288	2.842874	4.193195	4.094309
17375694	Dtwd1	DTW domain containing 1	11.95647	6.947866	7.588738	8.914476	9.940166	9.738435
17354327	Dtwd2	DTW domain containing 2	8.546063	7.118879	6.733469	8.546952	10.39891	8.695446
17377126	Dtd1	D-tyrosyl-tRNA deacylase 1	22.10775	19.34528	21.66539	19.64677	19.64677	20.49685
17281063	Dtd2	D-tyrosyl-tRNA deacylase 2	38.95806	38.26438	41.69233	41.21393	48.18593	45.97189
17410617	Dapp1	dual adaptor for phosphotyrosine and 3-phosphoinositides 1	5.425327	4.509758	4.675044	4.402613	4.923485	4.257186
17406397	Dear1	dual endothelin 1/angiotensin II receptor 1	3.413496	3.566774	4.690982	3.647151	3.738501	3.798579
17375418	Duox1	dual oxidase 1	6.002777	5.587876	5.206558	4.568815	5.908042	4.54355
17390738	Duox2	dual oxidase 2	8.714271	7.566048	7.635698	5.835394	5.142928	8.36578
17390778	Duoxa1	dual oxidase maturation factor 1	5.238377	4.9963	5.830704	4.937053	3.829404	4.112826
17375409	Duoxa2	dual oxidase maturation factor 2	4.61855	5.439409	5.338965	5.338965	6.093495	5.930978
17217197	Dstyk	dual serine/threonine and tyrosine protein kinase	18.89837	20.29722	18.89837	18.89837	18.89837	20.81786
17342642	Dusp1	dual specificity phosphatase 1	53.87373	62.20894	30.89454	63.21697	47.06687	69.31905
17220475	Dusp10	dual specificity phosphatase 10	13.26966	14.1031	15.57748	11.3808	11.61043	9.924745
17468469	Dusp11	dual specificity phosphatase 11 (RNA/RNP complex 1-interacting)	76.57226	84.20418	77.96312	84.63251	69.16211	75.18172
17229578	Dusp12	dual specificity phosphatase 12	30.96314	33.49445	27.71087	32.99922	30.96314	35.36426
17303964	Dusp13	dual specificity phosphatase 13	6.224207	5.416159	6.04805	6.035068	4.66042	5.483587
17266978	Dusp14	dual specificity phosphatase 14	21.1615	21.1615	23.87022	20.27303	21.1615	25.0226
17472030	Dusp16	dual specificity phosphatase 16	64.87542	58.89854	53.93164	53.93164	54.52969	61.23608
17246674	Dusp18	dual specificity phosphatase 18	11.80626	10.25112	7.87369	8.33285	8.216762	12.65723
17372430	Dusp19	dual specificity phosphatase 19	17.42939	17.71402	16.10269	16.75361	11.66327	17.71402
17375859	Dusp2	dual specificity phosphatase 2	16.93223	19.55723	16.43922	17.47911	13.35992	17.39644
17533494	Dusp21	dual specificity phosphatase 21	2.52876	2.762377	2.754652	3.057452	2.839585	2.596701
17286283	Dusp22	dual specificity phosphatase 22	33.73701	29.75066	30.20154	32.09463	37.55075	39.5071
17229948	Dusp23	dual specificity phosphatase 23	37.28078	26.85912	31.85814	33.29445	33.40217	27.99525
17500380	Dusp26	dual specificity phosphatase 26 (putative)	4.406093	4.801649	4.559925	5.036355	5.986903	5.036355
17229307	Dusp27	dual specificity phosphatase 27 (putative)	6.41007	7.698994	6.287864	7.698994	6.992896	8.015042
17215834	Dusp28	dual specificity phosphatase 28	17.16526	16.07749	14.44001	14.97745	17.16526	18.94388
17269985	Dusp3	dual specificity phosphatase 3 (vaccinia virus phosphatase VH1-related)	51.76661	55.91835	64.74918	50.84204	49.58562	40.41251
17500535	Dusp4	dual specificity phosphatase 4	12.78287	8.610816	7.425509	10.51517	7.954438	10.00195
17360348	Dusp5	dual specificity phosphatase 5	16.24834	16.71023	20.14989	16.8491	16.60401	14.46596
17236882	Dusp6	dual specificity phosphatase 6	119.1793	151.7592	133.4893	91.61805	95.08527	99.74551
17521194	Dusp7	dual specificity phosphatase 7	15.36089	20.32199	27.42672	23.8284	19.45528	18.76562
17498041	Dusp8	dual specificity phosphatase 8	13.17864	12.07014	8.118032	9.08836	9.71861	9.286339
17535600	Dusp9	dual specificity phosphatase 9	5.462949	6.12517	6.217911	6.513487	8.440099	5.523987
17303958	Dupd1	dual specificity phosphatase and pro isomerase domain containing 1	4.017199	4.1525	3.551725	3.191733	3.780665	3.918577
17393008	Dusp15	dual specificity phosphatase-like 15	3.698884	3.811503	4.270663	4.112113	4.144362	4.56834
17327428	Dyrk1a	dual-specificity tyrosine-(Y)-phosphorylation regulated kinase 1a	88.53831	84.48129	76.62217	74.96992	73.28825	76.86953
17475787	Dyrk1b	dual-specificity tyrosine-(Y)-phosphorylation regulated kinase 1b	47.52682	63.64609	65.46355	62.90401	62.8382	80.64315
17245334	Dyrk2	dual-specificity tyrosine-(Y)-phosphorylation regulated kinase 2	98.24009	113.098	99.8825	106.7861	93.14652	126.6365
17226752	Dyrk3	dual-specificity tyrosine-(Y)-phosphorylation regulated kinase 3	6.938289	8.307475	6.938289	8.51685	6.543715	5.415933
17471179	Dyrk4	dual-specificity tyrosine-(Y)-phosphorylation regulated kinase 4	2.879004	2.974588	2.650547	3.47143	3.948131	2.958221
17351842	Dym	dymeclin	50.26353	40.70827	50.2698	47.94771	40.80913	51.02775
17459989	Dctn1	dynactin 1	44.34686	48.24709	46.29389	45.98687	46.29389	44.27683
17237958	Dctn2	dynactin 2	156.2313	154.9609	154.9609	149.5703	163.1121	142.2564
17424298	Dctn3	dynactin 3	75.96063	95.22906	88.11221	92.95625	77.88697	84.2947
17350936	Dctn4	dynactin 4	105.2802	102.9471	102.9471	93.59049	93.94145	97.1539
17482730	Dctn5	dynactin 5	109.7974	130.899	127.5112	114.0901	130.2166	121.0744
17508706	Dctn6	dynactin 6	21.48905	18.1674	19.92586	16.37667	15.70182	17.91008
17355298	Dynap	dynactin associated protein	3.827547	3.64119	3.130602	3.621381	3.038375	3.339703
17383858	Dnm1	dynamin 1	14.34733	12.78092	10.99122	14.05674	17.28981	12.32871
17328484	Dnm1l	dynamin 1-like	168.8596	128.3207	117.1051	127.3981	120.2047	121.4627
17515208	Dnm2	dynamin 2	65.15256	67.51331	71.63635	54.59285	63.56409	52.39982
17228906	Dnm3	dynamin 3	4.35928	4.762437	5.175722	4.741949	4.863011	4.795031
17218727	Dnm3os	dynamin 3, opposite strand	5.94007	6.097636	5.387851	5.161642	5.859591	5.907632
17364968	Dnmbp	dynamin binding protein	43.83435	58.03776	67.70545	56.4695	49.03534	52.99153
17214865	Daw1	dynein assembly factor with WDR repeat domains 1	2.411415	2.399028	2.367854	2.557741	2.678917	2.525502
17278918	Dync1h1	dynein cytoplasmic 1 heavy chain 1	159.9736	153.6272	157.7909	146.9183	144.5806	140.8963
17455913	Dync1i1	dynein cytoplasmic 1 intermediate chain 1	2.787497	3.08885	2.978157	2.723893	3.10495	2.479467
17371716	Dync1i2	dynein cytoplasmic 1 intermediate chain 2	62.66422	68.21466	60.16114	75.64275	65.36108	60.54475
17522761	Dync1li1	dynein cytoplasmic 1 light intermediate chain 1	65.17198	65.17198	61.39673	58.16099	60.48267	65.29859
17523736	Dync2h1	dynein cytoplasmic 2 heavy chain 1	6.786087	6.987808	6.959096	6.687189	7.018146	6.183741
17340084	Dync2li1	dynein cytoplasmic 2 light intermediate chain 1	3.751714	3.956706	3.643841	4.851766	3.729055	5.131747
17451713	Dynll1	dynein light chain LC8-type 1	7.293595	5.444955	5.347817	5.226373	5.683935	5.023629
17267546	Dynll2	dynein light chain LC8-type 2	56.33619	44.21702	50.09796	55.41213	49.03772	53.07093
17378329	Dynlrb1	dynein light chain roadblock-type 1	344.383	341.5611	376.0264	321.363	289.091	343.5128
17505907	Dynlrb2	dynein light chain roadblock-type 2	5.655247	4.844138	5.655247	5.216492	4.774667	4.329619
17340524	Dynlt1a	dynein light chain Tctex-type 1A	109.9154	124.772	132.9611	131.0778	121.8	140.5179
17332915	Dynlt1b	dynein light chain Tctex-type 1B	93.82757	109.3773	113.368	117.8281	117.8281	126.0612

17332923	Dynlt1c	dynein light chain Tctex-type 1C	57.31497	57.94049	58.02771	58.02771	51.49141	62.11544
17340599	Dynlt1f	dynein light chain Tctex-type 1F	7.628345	5.616105	9.1684	7.101038	8.271793	7.628345
17540178	Dynlt3	dynein light chain Tctex-type 3	77.5046	68.49943	64.79542	57.48167	53.79126	62.62297
17435786	Drc1	dynein regulatory complex subunit 1	9.87965	9.87965	9.87965	9.768452	10.85795	11.34732
17504222	Drc7	dynein regulatory complex subunit 7	9.361307	8.626109	11.65115	8.69406	7.224812	11.69168
17506112	Dnaaf1	dynein, axonemal assembly factor 1	10.3876	8.449055	8.076387	7.614768	7.601373	11.16832
17281497	Dnaaf2	dynein, axonemal assembly factor 2	15.7187	15.59736	16.60574	17.32952	17.42178	19.17002
17485801	Dnaaf3	dynein, axonemal assembly factor 3	6.026415	4.641046	4.334603	4.757224	3.906942	4.453038
17443924	Dnaaf5	dynein, axonemal, assembly factor 5	55.71369	43.51859	40.42792	40.77774	35.67669	31.75606
17304658	Dnah1	dynein, axonemal, heavy chain 1	5.483435	5.737968	6.18267	5.811417	5.160211	6.685845
17442609	Dnah10	dynein, axonemal, heavy chain 10	4.713471	6.306677	5.837295	5.098525	5.168946	4.573029
17284710	Dnah11	dynein, axonemal, heavy chain 11	4.747342	4.647678	4.647678	5.165292	4.596099	4.755675
17297923	Dnah12	dynein, axonemal, heavy chain 12	3.943127	4.430962	4.430962	4.430962	4.430962	4.263269
17220277	Dnah14	dynein, axonemal, heavy chain 14	3.831077	3.785674	3.541744	3.831077	3.831077	4.71589
17272626	Dnah17	dynein, axonemal, heavy chain 17	3.729055	3.729055	3.729055	3.729055	3.5136	4.113909
17264676	Dnah2	dynein, axonemal, heavy chain 2	4.785966	4.08252	3.634231	4.280472	4.08252	4.110033
17251656	Dnah2os	dynein, axonemal, heavy chain 2, opposite strand [Source:MGI Symbol;Acc:MGI:19	6.317352	7.333999	6.060219	6.802146	6.555701	6.785617
17495702	Dnah3	dynein, axonemal, heavy chain 3	3.840595	3.745167	3.840595	3.804957	3.729727	4.335325
17310688	Dnah5	dynein, axonemal, heavy chain 5	4.294827	4.294827	4.489859	4.294827	4.493906	4.294827
17467840	Dnah6	dynein, axonemal, heavy chain 6	3.668677	3.668677	3.668677	3.668677	3.560599	3.668677
17223002	Dnah7a	dynein, axonemal, heavy chain 7A	2.717594	3.350183	2.859811	2.409694	5.384342	2.353275
17212548	Dnah7b	dynein, axonemal, heavy chain 7B	3.287247	3.132186	2.85304	3.213889	3.843871	3.313004
17212619	Dnah7c	dynein, axonemal, heavy chain 7C	2.772197	2.279046	2.67402	2.810779	2.999205	1.82959
17335628	Dnah8	dynein, axonemal, heavy chain 8	4.01461	4.01461	3.941651	4.20927	3.784572	4.132486
17264208	Dnah9	dynein, axonemal, heavy chain 9	5.421906	5.871829	5.48933	6.086994	5.364672	5.393119
17412883	Dnaic1	dynein, axonemal, intermediate chain 1	10.41665	10.41665	11.3879	10.41665	11.44448	10.41665
17258041	Dnaic2	dynein, axonemal, intermediate chain 2	4.81872	5.239394	4.63224	5.595783	5.433267	5.010623
17277177	Dnal1	dynein, axonemal, light chain 1	4.391367	4.746717	4.650062	3.750544	3.913203	3.832246
17319324	Dnal4	dynein, axonemal, light chain 4	5.857886	4.906962	5.830825	5.386134	5.119774	5.386134
17429921	Dnali1	dynein, axonemal, light intermediate polypeptide 1	10.48976	11.40923	10.52188	12.8301	10.20815	10.40922
17512229	Dync1li2	dynein, cytoplasmic 1 light intermediate chain 2	28.71554	32.21231	35.99713	36.56446	34.09764	29.80598
17513965	Dbndd1	dysbindin (dystrobrevin binding protein 1) domain containing 1	15.86615	19.09046	16.975	16.51094	19.30349	20.4306
17379427	Dbndd2	dysbindin (dystrobrevin binding protein 1) domain containing 2	7.29137	7.744703	7.219455	6.698927	8.078234	7.07446
17460185	Dysf	dysferlin	14.22572	13.9262	20.89245	13.36261	13.64528	13.9262
17535930	Dkc1	dyskeratosis congenita 1, dyskerin	28.83242	24.05275	26.16507	22.9869	22.61095	23.39324
17519245	Dyx1c1	dyslexia susceptibility 1 candidate 1 homolog (human)	4.040293	4.527172	4.236198	4.236198	3.977054	4.024892
17211587	Dst	dystonin	32.19579	34.68548	42.08167	37.38973	39.4904	36.13913
17348899	Dtna	dystrobrevin alpha	5.386134	5.579593	5.386134	5.386134	5.263272	5.386134
17348902	Dtna	dystrobrevin alpha	7.759749	6.190989	7.618358	7.618358	8.589594	7.566783
17292157	Dtnbp1	dystrobrevin binding protein 1	65.3004	60.77306	56.42764	57.16103	63.9561	65.3004
17273620	Dtnb	dystrobrevin, beta	46.65986	46.54002	52.41544	32.19869	47.69447	39.17768
17279755	Dtnbos	dystrobrevin, beta, opposite strand [Source:MGI Symbol;Acc:MGI:5141913]	2.782767	2.651502	3.143496	2.782767	2.782767	2.737104
17531168	Dag1	dystroglycan 1	144.0844	134.7088	140.1531	129.3028	100.8201	116.653
17474444	Dmwd	dystrophia myotonica-containing WD repeat motif	15.12748	17.36523	21.1706	17.36523	20.68338	17.69684
17474454	Dmpk	dystrophia myotonica-protein kinase	25.3661	56.67223	57.73043	41.99018	32.33019	50.91151
17537677	Drp2	dystrophin related protein 2	3.936109	5.192646	4.280265	4.435725	4.527831	4.290542
17536078	Dmd	dystrophin, muscular dystrophy	7.169189	7.169189	7.169189	7.169189	8.012209	7.169189
17223730	Dytn	dystrotelin	5.365416	6.049266	4.683932	4.274957	5.395251	6.246249
17313284	Ep300	E1A binding protein p300	62.67204	71.82429	73.93215	65.55255	64.7236	67.42632
17451061	Ep400	E1A binding protein p400	54.02293	52.85544	60.13321	50.4842	53.77449	52.83346
17451126	Ep400	E1A binding protein p400	146.3694	131.1006	139.4206	144.4363	191.0213	165.6047
17515819	Ets1	E26 avian leukemia oncogene 1, 5 domain	27.93671	28.88663	34.36124	26.6673	22.36127	19.00082
17327465	Ets2	E26 avian leukemia oncogene 2, 3 domain	52.96656	71.20467	74.25107	53.773	59.77954	39.97455
17393160	E2f1	E2F transcription factor 1	14.85545	13.32765	14.83466	13.53572	13.8837	14.63235
17420024	E2f2	E2F transcription factor 2	17.21388	10.25333	12.16437	11.44678	9.891093	14.79471
17291559	E2f3	E2F transcription factor 3	18.22403	14.45243	13.49104	13.56183	10.64556	10.04259
17504715	E2f4	E2F transcription factor 4	39.5123	34.78878	44.67017	30.01892	35.89448	29.32441
17396132	E2f5	E2F transcription factor 5	19.76451	16.09053	19.05028	14.67379	23.45608	18.96109
17274202	E2f6	E2F transcription factor 6	27.04388	24.69608	25.56114	24.69608	24.23466	26.61615
17237186	E2f7	E2F transcription factor 7	7.592105	8.636459	7.126185	7.442411	6.437542	6.461638
17491378	E2f8	E2F transcription factor 8	16.97995	23.24937	29.79503	12.58445	14.75876	11.24366
17281108	Eapp	E2F-associated phosphoprotein	32.80332	36.45336	32.80741	32.19088	25.47323	28.95138
17341856	E4f1	E4F transcription factor 1	16.15009	13.9823	18.05667	14.19037	16.78278	20.36782
17302238	Elf1	E74-like factor 1	71.25849	82.93907	83.42801	76.49746	69.28495	70.72018
17405099	Elf2	E74-like factor 2	35.76434	43.32086	40.4509	34.28724	37.32344	37.7936
17227266	Elf3	E74-like factor 3	4.194678	4.072797	4.167436	2.908348	3.179743	3.703322
17541404	Elf4	E74-like factor 4 (ets domain transcription factor)	13.54755	13.83528	13.72363	13.19949	10.90532	14.64443
17373739	Elf5	E74-like factor 5	4.376234	4.34185	3.995372	4.319337	4.857217	4.577373
17248691	Ebf1	early B cell factor 1	4.543865	3.946161	4.567865	3.975207	3.940367	4.196772
17301536	Ebf2	early B cell factor 2	4.873134	4.587601	4.391671	4.07938	4.622361	3.68532
17497366	Ebf3	early B cell factor 3	12.79537	11.50086	12.36843	11.32202	13.79296	12.33033
17376278	Ebf4	early B cell factor 4	8.922698	10.73128	9.556948	10.62934	10.01389	9.361307
17236754	Eea1	early endosome antigen 1	56.73039	57.97825	49.80228	50.93304	60.03492	55.39485
17349514	Egr1	early growth response 1	21.79991	28.55319	50.35664	26.38745	53.68771	34.79698
17233993	Egr2	early growth response 2	8.322114	6.368131	7.916595	8.620072	7.145475	7.990246
17301718	Egr3	early growth response 3	6.998715	8.094096	6.992896	7.440399	6.992896	7.786851
17468405	Egr4	early growth response 4	6.343196	8.986753	7.324448	7.511026	9.554829	8.71258
17417791	Ebna1bp2	EBNA1 binding protein 2	24.26912	28.15931	24.08094	27.32084	34.00088	27.71279
17303738	Ecd	ecdysoneless homolog (Drosophila)	65.82255	54.361	56.29754	54.89832	42.94386	46.43015
17278561	Eml1	echinoderm microtubule associated protein like 1	7.584373	9.488237	11.28465	9.488237	7.871508	8.581501
17474497	Eml2	echinoderm microtubule associated protein like 2	14.70342	17.45091	20.44208	17.61741	16.64261	22.65786
17357314	Eml3	echinoderm microtubule associated protein like 3	43.55481	31.52447	37.35657	33.96978	30.96786	36.7999
17339991	Eml4	echinoderm microtubule associated protein like 4	35.06379	36.35924	35.69104	29.00842	34.49439	35.55474
17283155	Eml5	echinoderm microtubule associated protein like 5	13.43691	15.53026	12.94605	14.02569	12.24627	13.83029

17261289	Eml6	echinoderm microtubule associated protein like 6	11.75905	10.69007	11.06253	10.89127	9.559201	10.30398
17266590	Evi2a	ecotropic viral integration site 2a	13.10377	14.60986	13.93026	13.10377	13.19482	10.98071
17450690	Evi5	ecotropic viral integration site 5	110.0061	114.2555	94.89433	104.706	88.68695	95.49412
17498760	Evi5l	ecotropic viral integration site 5 like	11.46918	11.59861	11.99233	13.4774	12.03438	11.98635
17524914	Ecsit	ECSIT homolog (Drosophila)	61.89667	53.53127	55.00649	56.7839	46.64886	53.58919
17404402	Ect2	ect2 oncogene	5.8242	6.166577	8.761391	6.253743	6.131245	4.76082
17289372	Enc1	ectodermal-neural cortex 1	54.18569	56.96682	57.12182	55.16113	53.01137	57.58336
17543396	Eda2r	ectodysplasin A2 receptor	11.80323	9.762157	6.627708	7.314859	9.175584	11.78116
17536501	Eda	ectodysplasin-A	6.060849	10.47617	10.79013	9.254275	8.703647	10.08249
17240971	Edar	ectodysplasin-A receptor	9.063888	9.9222	10.30884	11.74048	8.091348	9.728787
17302113	Enox1	ecto-NOX disulfide-thiol exchanger 1	6.553157	6.470692	5.969003	8.014986	6.430228	8.030167
17541493	Enox2	ecto-NOX disulfide-thiol exchanger 2	25.15285	31.87537	38.21402	31.14352	34.9575	36.46423
17359344	Entpd1	ectonucleoside triphosphate diphosphohydrolase 1	16.13118	19.47714	26.44696	17.44244	16.81527	17.52801
17367963	Entpd2	ectonucleoside triphosphate diphosphohydrolase 2	25.96111	22.66729	22.22482	18.68916	21.1092	18.09012
17523231	Entpd3	ectonucleoside triphosphate diphosphohydrolase 3	3.45858	4.172812	4.260728	4.18202	4.211108	3.940394
17301615	Entpd4	ectonucleoside triphosphate diphosphohydrolase 4	78.19096	112.0198	91.38212	91.57488	81.20688	85.03943
17301647	Entpd4	ectonucleoside triphosphate diphosphohydrolase 4	64.47598	92.41339	75.38262	76.5712	64.31084	65.32983
17282498	Entpd5	ectonucleoside triphosphate diphosphohydrolase 5	425.0533	511.5956	439.808	407.6904	309.3015	394.7595
17377478	Entpd6	ectonucleoside triphosphate diphosphohydrolase 6	23.99513	16.85448	20.92985	19.01769	19.62621	20.08268
17359608	Entpd7	ectonucleoside triphosphate diphosphohydrolase 7	34.09339	20.47398	14.29521	21.03602	20.33552	21.03602
17367834	Entpd8	ectonucleoside triphosphate diphosphohydrolase 8	68.91853	64.96542	62.66031	81.47186	71.1578	74.07937
17239787	Enpp1	ectonucleotide pyrophosphatase/phosphodiesterase 1	66.66049	46.21887	49.91149	45.41352	53.02203	45.7849
17317056	Enpp2	ectonucleotide pyrophosphatase/phosphodiesterase 2	142.8067	121.7055	119.2644	127.9361	196.1876	170.9701
17239817	Enpp3	ectonucleotide pyrophosphatase/phosphodiesterase 3	244.2949	166.7716	108.3796	150.5188	166.7751	178.312
17345124	Enpp4	ectonucleotide pyrophosphatase/phosphodiesterase 4	66.89006	77.09257	61.07288	68.29462	69.80459	66.31209
17337844	Enpp5	ectonucleotide pyrophosphatase/phosphodiesterase 5	75.57462	62.45651	69.13767	65.57164	45.58003	64.9222
17501061	Enpp6	ectonucleotide pyrophosphatase/phosphodiesterase 6	6.905759	5.346594	6.042519	6.401766	4.998586	7.343002
17258935	Enpp7	ectonucleotide pyrophosphatase/phosphodiesterase 7	3.348721	3.511092	3.867925	3.511092	3.511092	3.511092
17497003	Etos1	ectopic ossification 1	6.499686	6.645326	6.454745	7.545937	8.241175	6.753943
17352056	Epg5	ectopic P-granules autophagy protein 5 homolog (C. elegans)	39.92554	40.26236	41.2834	41.16111	32.7218	36.86603
17290646	Edaradd	EDAR (ectodysplasin-A receptor)-associated death domain	3.502585	3.47627	3.729055	4.696415	3.707072	4.886969
17460558	Efcc1	EF hand and coiled-coil domain containing 1	8.705336	10.61535	11.09486	9.535575	13.22311	9.539873
17323198	Efcab1	EF hand calcium binding domain 1	2.919234	2.302801	2.606909	2.606909	2.546243	3.121133
17215240	Efhd1	EF hand domain containing 1	6.776921	8.296196	4.872324	7.396691	8.304193	8.293551
17225248	Efhd1os	EF hand domain containing 1, opposite strand [Source:MGI Symbol;Acc:MGI:44134	3.97868	4.20927	4.992838	4.20927	5.056153	4.182049
17432341	Efhd2	EF hand domain containing 2	50.01053	61.89667	70.85855	62.07799	70.52486	68.23547
17345955	Efhb	EF hand domain family, member B	3.081708	3.009972	3.009972	3.128845	3.16251	3.302755
17274942	Efcab10	EF-hand calcium binding domain 10	2.902166	2.47538	2.242394	2.430798	2.754805	3.041662
17283216	Efcab11	EF-hand calcium binding domain 11	6.378733	6.287864	6.972471	9.040359	5.523145	7.632364
17469941	Efcab12	EF-hand calcium binding domain 12	3.384236	5.243281	5.387515	4.06031	4.947844	4.471751
17257254	Efcab13	EF-hand calcium binding domain 13	3.90318	3.229632	2.537785	2.304206	2.55868	2.95355
17417269	Efcab14	EF-hand calcium binding domain 14	67.98997	67.9019	68.42208	62.64685	76.02595	59.48112
17219988	Efcab2	EF-hand calcium binding domain 2	4.10286	4.665042	3.960246	5.027043	4.640982	6.627202
17257283	Efcab3	EF-hand calcium binding domain 3	3.090157	3.160888	3.090157	2.686895	3.216385	3.332327
17266198	Efcab5	EF-hand calcium binding domain 5	3.76367	3.775244	3.76367	3.780219	4.250846	3.76367
17319913	Efcab6	EF-hand calcium binding domain 6	4.876513	5.127622	4.876513	5.078914	4.764781	5.622181
17415798	Efcab7	EF-hand calcium binding domain 7	3.610929	2.934775	3.339703	3.263862	3.263862	3.36291
17378000	Efcab8	EF-hand calcium binding domain 8	3.496254	3.618395	3.197047	3.240238	3.091206	3.210393
17261517	Efcab9	EF-hand calcium binding domain 9	3.504601	3.820549	3.198976	2.732535	3.634684	3.370307
17211382	Efhc1	EF-hand domain (C-terminal) containing 1	4.765409	5.182902	4.970221	5.121939	4.36669	5.121939
17540402	Efhc2	EF-hand domain (C-terminal) containing 2	3.038923	3.286906	3.467582	2.86499	3.922926	3.647429
17311909	Efr3a	EFR3 homolog A (S. cerevisiae)	81.78078	82.70829	64.74379	68.83737	57.13924	65.42772
17279764	Efr3b	EFR3 homolog B (S. cerevisiae)	6.617058	9.182646	8.51685	8.51685	8.51685	9.11808
17469289	Eogt	EGF domain-specific O-linked N-acetylglucosamine (GlcNAc) transferase	24.35034	16.14841	17.69684	16.23595	17.05083	10.51327
17396521	Egfem1	EGF-like and EMI domain containing 1	3.443152	4.490294	4.567232	3.768055	4.270189	4.982812
17368330	Egfl7	EGF-like domain 7	33.62401	41.39573	36.03907	38.48328	37.7049	34.74564
17343882	Egfl8	EGF-like domain 8	12.2149	15.71957	15.23914	12.49995	11.73381	15.59736
17288992	Edil3	EGF-like repeats and discoidin I-like domains 3	3.359904	3.228424	3.349069	3.139402	3.744959	3.540198
17315763	Egflam	EGF-like, fibronectin type III and laminin G domains	4.249609	3.703322	3.56376	3.703322	5.002503	4.279968
17546055	Egfl6	EGF-like-domain, multiple 6	5.758687	7.028077	7.579854	5.233188	5.044705	6.793147
17260747	Eldr	Egfr long non-coding downstream RNA	2.937258	3.603028	3.043666	3.163387	3.592256	3.173027
17514135	Egln1	egl-9 family hypoxia-inducible factor 1	58.08364	63.96097	61.20086	63.96097	83.70837	63.96097
17488122	Egln2	egl-9 family hypoxia-inducible factor 2	53.53832	58.51887	58.34957	57.3106	62.19945	58.19729
17281084	Egln3	egl-9 family hypoxia-inducible factor 3	77.47613	49.87206	51.24041	47.94339	57.40999	64.52293
17260968	Ehbp1	EH domain binding protein 1	11.34748	11.67839	13.62836	14.18044	18.30226	15.02018
17361741	Ehbp1l1	EH domain binding protein 1-like 1	18.49649	17.44595	25.06027	18.49649	19.02903	16.56608
17356743	Ehd1	EH-domain containing 1	83.04722	50.3947	58.36413	65.64485	47.20834	52.46815
17486807	Ehd2	EH-domain containing 2	7.967516	11.29506	11.99324	7.085502	9.629227	10.54955
17339574	Ehd3	EH-domain containing 3	264.6864	163.8123	179.7939	188.5666	83.69677	83.97454
17390066	Ehd4	EH-domain containing 4	16.74432	14.77289	14.77289	14.77289	14.77289	14.69578
17355386	Elac1	elaC homolog 1 (E. coli)	17.79401	22.90975	22.54632	26.65215	22.87342	24.788
17250876	Elac2	elaC homolog 2 (E. coli)	45.19621	42.56218	43.48181	35.88154	38.91596	37.52838
17235011	Elane	elastase, neutrophil expressed	5.3254	5.134166	5.617429	6.439238	4.990519	5.87069
17453454	Eln	elastin	12.60295	10.85095	18.27311	12.60295	6.905951	11.86615
17435920	Emilin1	elastin microfibril interfacer 1	32.7023	33.77327	38.91164	23.29454	25.96345	30.07223
17346975	Emilin2	elastin microfibril interfacer 2	11.4691	12.22235	10.66876	10.66876	10.66876	11.17583
17393898	Emilin3	elastin microfibril interfacer 3	6.992896	9.272639	7.64433	8.98034	6.992896	6.347815
17507277	Elavl1	ELAV (embryonic lethal, abnormal vision)-like 1 (Hu antigen R)	92.14922	90.99403	103.4522	98.53402	88.89929	82.35589
17427164	Elavl2	ELAV (embryonic lethal, abnormal vision, Drosophila)-like 2 (Hu antigen B)	6.676954	5.918452	6.234483	5.925348	6.447769	6.295365
17524895	Elavl3	ELAV (embryonic lethal, abnormal vision, Drosophila)-like 3 (Hu antigen C)	9.540005	9.727102	10.05534	9.488237	11.46481	8.436941
17428228	Elavl4	ELAV (embryonic lethal, abnormal vision, Drosophila)-like 4 (Hu antigen D)	3.216385	3.888193	4.07387	3.653299	3.594223	3.476294
17527353	Etfa	electron transferring flavoprotein, alpha polypeptide	505.2382	502.5036	452.6054	540.6458	604.2367	635.6417
17477116	Etfb	electron transferring flavoprotein, beta polypeptide	373.5196	355.2153	346.4852	365.3436	371.0655	416.9036
17406091	Etfdh	electron transferring flavoprotein, dehydrogenase	792.3203	826.4307	762.9422	720.0883	713.2233	731.8885

17540535	Elk1	ELK1, member of ETS oncogene family	15.29258	19.43803	18.90663	20.05931	18.55621	16.40143
17244341	Elk3	ELK3, member of ETS oncogene family	20.35258	21.98732	37.40243	22.7381	18.63419	16.70734
17217145	Elk4	ELK4, member of ETS oncogene family	25.24245	38.69432	43.28424	38.58023	43.71359	43.16889
17470323	Erc1	ELKS/RAB6-interacting/CAST family member 1	53.72717	51.47464	54.38738	52.47761	39.54984	47.88162
17298090	Erc2	ELKS/RAB6-interacting/CAST family member 2	4.975564	5.074761	3.85254	4.193108	4.424824	4.571286
17298451	Eaf1	ELL associated factor 1	75.35389	78.95616	76.14936	71.85218	71.27726	65.59391
17330246	Eaf2	ELL associated factor 2	3.339703	3.339703	3.370073	3.339703	3.339703	3.339703
17447560	Evc	Ellis van Creveld gene syndrome	15.074	16.27324	15.97761	16.27324	15.24696	16.38893
17437019	Evc2	Ellis van Creveld syndrome 2	11.16267	11.51594	12.0744	11.34363	12.29466	11.72885
17282465	Elmsan1	ELM2 and Myb/SANT-like domain containing 1	13.524	14.51206	14.79307	14.79307	17.40597	15.35514
17527173	Elmod1	ELMO/CED-12 domain containing 1	4.432682	5.789987	4.773045	4.801649	5.55351	4.711283
17510885	Elmod2	ELMO/CED-12 domain containing 2	32.76106	27.08496	30.89454	30.89454	34.37291	34.21151
17467782	Elmod3	ELMO/CED-12 domain containing 3	52.09886	47.90054	69.12281	55.41329	41.5429	40.74017
17524925	Elof1	elongation factor 1 homolog (ELF1, S. cerevisiae)	17.08905	20.4391	19.62458	18.14789	18.30531	14.47298
17532692	LOC100861738	elongation factor 2 pseudogene	5.298192	7.027931	7.684546	9.10892	6.992896	8.158761
17501962	Ell	elongation factor RNA polymerase II	38.67046	43.77302	50.60402	36.34059	50.87377	40.29028
17288699	Ell2	elongation factor RNA polymerase II 2	126.4339	130.2275	101.9558	131.3443	151.0131	149.6927
17390580	Ell3	elongation factor RNA polymerase II-like 3	5.205367	7.337456	7.264231	8.172968	7.331051	6.957891
17479804	Eftud1	elongation factor Tu GTP binding domain containing 1	27.8196	27.18257	25.41679	24.34291	17.49014	21.76865
17270322	Eftud2	elongation factor Tu GTP binding domain containing 2	35.10489	36.49533	37.93164	36.10934	33.56812	30.89454
17417767	Elovl1	elongation of very long chain fatty acids (FEN1/Elo2, SUR4/Elo3, yeast)-like 1	47.46657	64.07635	58.211	57.16103	46.51261	45.28841
17292058	Elovl2	elongation of very long chain fatty acids (FEN1/Elo2, SUR4/Elo3, yeast)-like 2	1028.895	1010.582	1003.657	851.6401	734.8113	789.3493
17359918	Elovl3	elongation of very long chain fatty acids (FEN1/Elo2, SUR4/Elo3, yeast)-like 3	1695.271	186.8362	36.64819	481.2324	264.5378	143.3512
17529307	Elovl4	elongation of very long chain fatty acids (FEN1/Elo2, SUR4/Elo3, yeast)-like 4	3.775637	4.754158	4.855601	4.801649	5.982423	4.822529
17389051	Elp4	elongation protein 4 homolog (S. cerevisiae)	37.94663	31.62777	29.36712	31.77169	34.6588	38.72545
17348990	Elp2	elongator acetyltransferase complex subunit 2	48.2946	38.08682	42.8214	50.60261	46.79687	45.50048
17307755	Elp3	elongator acetyltransferase complex subunit 3	57.79449	43.61855	43.94204	49.30356	56.02776	51.46965
17265111	Elp5	elongator acetyltransferase complex subunit 5	36.63422	26.88296	28.29451	24.62787	28.38762	30.61231
17522328	Elp6	elongator acetyltransferase complex subunit 6	8.325287	10.6116	10.39682	7.783235	9.077217	9.296773
17519598	Elovl5	ELOVL family member 5, elongation of long chain fatty acids (yeast)	1279.106	1437.006	1415.19	1226.856	1304.797	1360.725
17402558	Elovl6	ELOVL family member 6, elongation of long chain fatty acids (yeast)	169.3918	195.2015	258.3369	156.6999	121.6051	127.7846
17289717	Elovl7	ELOVL family member 7, elongation of long chain fatty acids (yeast)	6.25873	6.437632	6.762797	6.79748	5.211685	6.655514
17290083	Emb	embigin	3.328841	5.094427	4.656997	4.20927	4.70003	4.880605
17306524	Efs	embryonal Fyn-associated substrate	5.460337	5.306866	5.961684	5.386134	4.465588	6.584619
17493250	Eed	embryonic ectoderm development	47.70372	41.49168	36.19654	38.93188	40.93747	47.79375
17534741	Etd	embryonic testis differentiation	3.245903	2.549651	2.411549	2.324419	2.477886	2.631071
17535752	Emd	emerin	17.11679	16.38871	14.67379	15.49606	22.06549	14.3749
17470779	Emg1	EMG1 nucleolar protein homolog (S. cerevisiae)	47.29448	36.62026	42.74637	48.70676	40.84422	45.96201
17260119	Emid1	EMI domain containing 1	9.791433	9.792655	8.518326	11.76101	9.733576	10.01396
17307463	Ebpl	emopamil binding protein-like	72.00923	84.92162	73.23239	75.11869	85.87522	79.86436
17460261	Emx1	empty spiracles homeobox 1	8.292459	7.577122	8.039245	8.119495	9.037289	9.659711
17360845	Emx2	empty spiracles homeobox 2	4.01461	4.176082	4.604294	4.184224	4.037298	4.20927
17493583	Emsy	EMSY, BRCA2-interacting transcriptional repressor	34.50324	41.49254	40.65977	38.31109	31.66572	37.11267
17366047	Emx2os	Emx2 opposite strand/antisense transcript (non-protein coding)	3.81145	3.503945	3.640105	4.412725	3.263862	3.400342
17230573	Enah	enabled homolog (Drosophila)	4.078391	4.048956	3.658875	4.269301	3.603828	4.919256
17438772	Enam	enamelin	3.897422	3.138228	3.026059	3.002054	2.988145	3.007574
17278593	Evl	Ena-vasodilator stimulated phosphoprotein	30.56122	31.58505	36.30333	32.62735	30.62653	33.7328
17523095	Exog	endo/exonuclease (5-3), endonuclease G-like	5.94728	4.544558	5.692491	6.040048	5.970493	4.400294
17258912	Engase	endo-beta-N-acetylglucosaminidase	26.0083	32.71975	33.57743	28.9259	37.18168	29.61795
17391915	Erv3	endogenous retroviral sequence 3	4.982915	5.419313	6.631292	4.982915	4.982915	5.56106
17369925	Eng	endoglin	168.5392	150.3801	175.5417	144.984	75.39046	87.76112
17402981	Emcn	endomucin	15.82485	13.77003	15.23914	12.63068	12.77782	12.88446
17524014	Endod1	endonuclease domain containing 1	15.81049	16.91686	15.44813	14.69283	17.25712	17.58801
17369120	Endog	endonuclease G	45.43493	35.15846	29.31485	42.45728	46.26085	33.77023
17259157	Endov	endonuclease V	13.09823	12.2033	13.14161	13.81209	11.99042	11.51953
17320993	Endou	endonuclease, polyU-specific	4.229127	4.433727	3.230013	4.411563	3.665729	3.7356
17515539	Eepd1	endonuclease/exonuclease/phosphatase family domain containing 1	32.52484	32.52484	38.64366	32.52484	29.27648	30.21221
17270888	Ern1	endoplasmic reticulum (ER) to nucleus signalling 1	122.3118	116.5633	111.4096	116.2221	105.8641	88.13908
17495958	Ern2	endoplasmic reticulum (ER) to nucleus signalling 2	5.070858	5.317765	5.317765	5.317765	5.317765	5.38998
17288654	Erap1	endoplasmic reticulum aminopeptidase 1	160.4333	136.5381	122.882	136.1761	137.053	143.5779
17353585	Sil1	endoplasmic reticulum chaperone SIL1 homolog (S. cerevisiae)	162.8466	150.5052	135.9583	132.1397	182.8111	150.7611
17261399	Erlec1	endoplasmic reticulum lectin 1	65.40187	72.14362	69.525	62.74071	75.54006	75.59453
17363828	Ermp1	endoplasmic reticulum metallopeptidase 1	73.84201	79.10626	76.38619	66.50035	71.28813	67.94097
17472199	Erp27	endoplasmic reticulum protein 27	12.42479	13.5845	12.48748	14.32437	13.01587	11.25317
17452194	Erp29	endoplasmic reticulum protein 29	39.97455	24.66426	22.01615	27.42786	30.35011	19.17427
17425160	Erp44	endoplasmic reticulum protein 44	75.78603	68.54502	61.50919	72.36248	73.93676	72.36248
17334932	Ergic1	endoplasmic reticulum-golgi intermediate compartment (ERGIC) 1	160.6325	136.9466	165.7563	115.9613	123.8843	119.9484
17400412	Ensa	endosulfine alpha	24.38885	24.07393	22.72251	22.6351	22.98101	17.69402
17353650	Ecscr	endothelial cell surface expressed chemotaxis and apoptosis regulator	10.52333	17.1548	15.64067	13.42732	11.72503	14.32884
17516074	Esam	endothelial cell-specific adhesion molecule	13.22458	14.60641	14.31296	11.61913	10.99206	13.38152
17290010	Esm1	endothelial cell-specific molecule 1	6.516874	7.263727	5.474549	5.084373	7.535274	7.94441
17368096	Edf1	endothelial differentiation-related factor 1	6.484611	6.979724	7.140623	5.299	7.804575	6.759376
17340197	Epas1	endothelial PAS domain protein 1	202.7798	262.9838	277.3704	196.9957	256.5133	189.3012
17415469	Tek	endothelial-specific receptor tyrosine kinase	33.08029	23.84856	32.59977	27.42405	24.66375	18.58659
17286830	Edn1	endothelin 1	6.469436	6.24032	7.698994	5.499803	5.843043	5.525404
17417976	Edn2	endothelin 2	11.24896	12.85333	12.49778	12.96922	10.83149	13.26948
17380377	Edn3	endothelin 3	22.36669	19.17148	18.25753	22.86042	17.5924	20.9023
17420347	Ece1	endothelin converting enzyme 1	284.8029	263.5385	249.0549	224.4686	226.6575	200.6518
17324001	Ece2	endothelin converting enzyme 2	9.664667	11.39177	11.78557	11.40908	9.704138	12.12346
17225224	Ecel1	endothelin converting enzyme-like 1	6.949841	7.484988	6.596497	7.374729	6.651086	8.03412
17510685	Ednra	endothelin receptor type A	23.57621	24.02776	25.86914	20.29525	18.75912	20.25856
17309268	Ednrb	endothelin receptor type B	66.69469	62.53015	65.65213	55.02785	27.74296	36.06231
17424276	Enho	energy homeostasis associated	21.5828	22.64201	24.43335	26.87774	23.10319	27.18728
17216676	En1	engrailed 1	3.462322	3.93071	3.393585	3.412006	3.792082	3.437763

17435592	En2	engrailed 2	5.892501	4.874959	6.1638	4.388537	4.113368	5.92075
17285559	Elmo1	engulfment and cell motility 1	16.56699	15.9867	16.38768	13.95016	14.01792	12.25595
17394405	Elmo2	engulfment and cell motility 2	23.17103	21.23026	22.92278	19.08504	17.31643	20.32073
17504727	Elmo3	engulfment and cell motility 3	28.99536	29.68084	26.25299	27.46876	29.55725	28.73247
17517780	Edc3	enhancer of mRNA decapping 3 homolog (S. cerevisiae)	25.51529	39.56547	31.82768	39.22768	27.94833	33.77608
17504964	Edc4	enhancer of mRNA decapping 4	20.44874	20.09786	20.21298	23.98034	18.5768	16.3414
17352493	Epc1	enhancer of polycomb homolog 1 (Drosophila)	26.9963	31.97849	32.85133	28.1718	36.1855	32.528
17370641	Epc2	enhancer of polycomb homolog 2 (Drosophila)	30.92411	36.16219	28.83362	31.42694	30.34675	35.14725
17282274	Erh	enhancer of rudimentary homolog (Drosophila)	11.20826	9.697481	7.899547	8.403673	11.43069	12.28427
17405069	1700018B24Rik	enhancer of rudimentary homolog pseudogene	4.063323	4.20927	3.844324	4.194416	4.596928	3.279259
17311343	Eny2	enhancer of yellow 2 homolog (Drosophila)	82.93677	102.2821	86.44736	83.67009	121.4695	127.1097
17269809	Ezh1	enhancer of zeste 1 polycomb repressive complex 2 subunit	48.2869	60.14863	63.16135	63.16135	63.16135	64.06258
17466507	Ezh2	enhancer of zeste 2 polycomb repressive complex 2 subunit	21.41144	22.83825	21.41144	24.29454	21.14097	23.20735
17367390	Etl4	enhancer trap locus 4	20.42353	15.89367	16.87926	13.35039	13.94659	14.83826
17512494	Enkd1	enkurin domain containing 1	12.05074	12.42436	12.42436	11.24265	13.48609	12.58619
17382139	Enkur	enkurin, TRPC channel interacting protein	2.582915	2.205078	2.500541	2.636876	3.049198	2.296059
17421908	Eno1	enolase 1, alpha non-neuron	6.734543	8.886344	7.603059	7.936759	8.993546	8.637417
17350437	Eno1b	enolase 1B, retrotransposed	2.193645	1.903283	2.735529	1.899474	2.317437	2.498133
17470846	Eno2	enolase 2, gamma neuronal	8.225139	9.928598	10.71701	10.78803	8.068833	7.470164
17252183	Eno3	enolase 3, beta muscle	23.84013	22.29379	22.62789	22.97973	20.97545	22.62789
17360800	Eno4	enolase 4	4.00941	4.20927	4.325179	4.099307	4.20927	3.887466
17439563	Enoph1	enolase-phosphatase 1	25.23843	22.16452	26.6808	30.36947	24.63231	26.46236
17475975	Ech1	enoyl coenzyme A hydratase 1, peroxisomal	935.8412	950.0963	933.6513	852.077	745.3995	774.9933
17232426	Echdc1	enoyl Coenzyme A hydratase domain containing 1	96.161	88.7546	90.76474	96.161	106.7136	106.6773
17416616	Echdc2	enoyl Coenzyme A hydratase domain containing 2	141.1798	174.2518	188.7928	179.8637	173.0962	161.2986
17381457	Echdc3	enoyl Coenzyme A hydratase domain containing 3	40.56124	42.29455	42.59375	48.78886	47.22831	42.29455
17497612	Echs1	enoyl Coenzyme A hydratase, short chain, 1, mitochondrial	238.0218	275.0328	270.5854	285.8194	289.1371	308.7938
17334253	Eci1	enoyl-Coenzyme A delta isomerase 1	245.868	270.6923	258.7724	260.659	245.2518	244.0258
17291833	Eci2	enoyl-Coenzyme A delta isomerase 2	192.2727	276.3954	218.7259	197.5727	214.1043	190.3248
17291821	Eci3	enoyl-Coenzyme A delta isomerase 3	7.160399	5.833129	6.77927	4.557492	5.785092	4.781655
17329220	Ehhadh	enoyl-Coenzyme A, hydratase/3-hydroxyacyl Coenzyme A dehydrogenase	1168.013	1708.317	1644.793	1324.186	755.9987	865.0921
17319412	Enthd1	ENTH domain containing 1	9.177365	8.727691	8.448587	8.698098	8.893245	11.47864
17273003	Enthd2	ENTH domain containing 2	19.86367	14.26215	17.07555	16.79826	15.66204	17.92026
17272262	Evpl	envoplakin	7.875328	9.441002	9.361307	7.698994	10.01389	7.926258
17522876	Eomes	eomesodermin homolog (Xenopus laevis)	3.81108	6.901835	5.257147	4.20927	5.120306	3.506836
17267520	Epx	eosinophil peroxidase	3.767924	4.468653	3.918442	5.108997	5.886092	5.112291
17305520	Ear1	eosinophil-associated, ribonuclease A family, member 1	7.710156	2.645353	3.246578	3.316588	3.609828	3.812851
17305524	Ear10	eosinophil-associated, ribonuclease A family, member 10	2.072138	1.920657	2.282951	2.442519	1.996496	2.072138
17299593	Ear14	eosinophil-associated, ribonuclease A family, member 14	3.122497	3.393679	4.280265	3.10495	3.276624	3.415342
17299101	Ear2	eosinophil-associated, ribonuclease A family, member 2	38.47502	40.60344	53.01909	35.94676	32.10642	40.72295
17299638	Ear6	eosinophil-associated, ribonuclease A family, member 6	2.738527	3.133163	2.867453	3.10523	6.11677	2.97824
17375661	Eid1	EP300 interacting inhibitor of differentiation 1	7.512953	7.351405	7.749375	8.483679	10.29142	7.487531
17475801	Eid2	EP300 interacting inhibitor of differentiation 2	6.019568	6.183699	5.736457	6.970441	5.490167	6.171709
17475805	Eid2b	EP300 interacting inhibitor of differentiation 2B	10.16607	8.840635	10.09704	9.759722	11.76207	9.734454
17235937	Eid3	EP300 interacting inhibitor of differentiation 3	11.81698	11.40307	16.68894	14.87915	13.24752	10.30884
17290865	Epdr1	ependymin related protein 1 (zebrafish)	14.31177	18.88109	17.43193	17.10433	13.46619	16.79943
17466397	Epha1	Eph receptor A1	52.96583	59.76132	61.02676	59.10507	57.96017	59.76132
17418367	Epha10	Eph receptor A10	10.01007	11.50086	10.9119	8.671791	9.984914	10.16219
17421033	Epha2	Eph receptor A2	21.83333	18.49649	21.39156	26.73134	26.6084	24.15031
17331380	Epha3	Eph receptor A3	6.871285	7.108573	6.627938	6.652193	6.627938	6.627938
17224661	Epha4	Eph receptor A4	7.157918	7.758512	8.089385	6.850124	5.935543	6.860292
17449129	Epha5	Eph receptor A5	4.066507	4.142983	3.772236	3.929103	4.312397	3.751012
17331326	Epha6	Eph receptor A6	3.062691	3.557367	3.557367	3.703322	3.703322	3.86755
17412296	Epha7	Eph receptor A7	9.167002	9.144409	8.827592	9.503968	8.641789	8.540497
17431624	Epha8	Eph receptor A8	8.93681	8.367055	8.044931	8.554598	7.354973	7.922249
17530183	Ephb1	Eph receptor B1	4.178978	4.054489	4.291197	4.560905	3.524968	3.927279
17431588	Ephb2	Eph receptor B2	6.033186	8.025549	6.645234	6.476031	6.549796	5.880749
17324163	Ephb3	Eph receptor B3	11.07658	10.27987	10.02368	10.05276	7.705134	10.9932
17443539	Ephb4	Eph receptor B4	30.66264	37.29344	40.10638	32.61333	29.94327	31.48821
17457807	Ephb6	Eph receptor B6	5.285097	4.66695	5.235002	4.909173	5.322559	4.909173
17406990	Efna1	ephrin A1	78.00691	60.43574	87.65412	73.22072	71.97131	63.23099
17235243	Efna2	ephrin A2	6.522975	6.750152	8.733258	6.561838	7.935109	5.846527
17407001	Efna3	ephrin A3	6.111261	5.252084	5.516679	4.939277	5.220942	4.197208
17407010	Efna4	ephrin A4	3.679093	3.679093	4.910058	3.170279	3.679093	3.818352
17346637	Efna5	ephrin A5	14.89463	15.19116	14.87461	18.60992	16.70491	18.73728
17536483	Efnb1	ephrin B1	45.6246	43.77818	43.77818	42.91738	40.39458	32.49599
17507321	Efnb2	ephrin B2	11.79178	12.64268	13.44959	11.648	11.74057	12.19553
17264769	Efnb3	ephrin B3	16.26411	16.83603	15.30435	18.22062	15.9578	15.53295
17410275	Egf	epidermal growth factor	3.15253	4.20927	4.20927	3.676671	4.359371	4.890595
17247389	Egfr	epidermal growth factor receptor	657.6791	648.6651	517.2041	756.9478	721.7023	363.7416
17416830	Eps15	epidermal growth factor receptor pathway substrate 15	104.9414	105.2926	90.32103	96.161	101.2944	81.94364
17510498	Eps15l1	epidermal growth factor receptor pathway substrate 15-like 1	29.12467	39.58083	38.82651	23.29454	38.3919	33.79458
17472235	Eps8	epidermal growth factor receptor pathway substrate 8	9.278104	9.278104	11.09725	9.544436	9.011954	7.169189
17260744	Egfros	epidermal growth factor receptor, opposite strand [Source:MGI Symbol;Acc:MGI:2	16.09622	13.9824	12.34949	10.68193	8.71258	10.13097
17247948	Efemp1	epidermal growth factor-containing fibulin-like extracellular matrix protein 1	14.45283	18.49649	18.91987	26.18393	19.96471	18.7134
17356447	Efemp2	epidermal growth factor-containing fibulin-like extracellular matrix protein 2	12.86643	17.45139	14.20592	14.31504	14.27551	14.20592
17394205	Eppin	epididymal peptidase inhibitor	3.134836	2.42945	3.166217	3.283877	2.855909	3.53853
17299582	Eddm3b	epididymal protein 3B	3.339703	3.613683	3.339703	2.877967	3.070748	3.339703
17231669	Epm2a	epilepsy, progressive myoclonic epilepsy, type 2 gene alpha	10.84208	7.733599	10.39811	8.411599	10.84208	14.37958
17236821	Epyc	epiphycan	3.803295	2.993223	2.950072	3.068024	3.183315	3.893291
17439021	Ereg	epiregulin	6.447009	8.689059	8.457962	9.54146	7.183366	10.28892
17340272	Epcam	epithelial cell adhesion molecule	6.171709	5.58323	5.934967	5.381506	5.062711	5.385499
17239506	Ect2l	epithelial cell transforming sequence 2 oncogene-like	4.070088	4.226197	4.193602	4.171019	4.20927	4.422769
17463781	Emp1	epithelial membrane protein 1	13.26645	9.441068	13.2567	11.70189	8.804127	11.80299

17328062	Emp2	epithelial membrane protein 2	18.4614	25.22776	28.61639	16.23595	17.4091	20.69485
17491026	Emp3	epithelial membrane protein 3	22.00592	27.43071	40.56574	25.97605	26.25263	26.25263
17439013	Epgn	epithelial mitogen	4.778507	3.143823	4.084473	4.506539	4.02785	4.119331
17423291	Esrp1	epithelial splicing regulatory protein 1	3.223259	3.715818	3.362397	3.362397	3.252232	3.421835
17512634	Esrp2	epithelial splicing regulatory protein 2	97.67043	93.8614	91.85778	100.4546	90.36862	85.10429
17302141	Epsti1	epithelial stromal interaction 1 (breast)	10.36573	12.04632	11.71569	8.107629	12.50142	20.3578
17522598	Epm2aip1	EPM2A (laforin) interacting protein 1	29.44049	27.74258	24.66255	23.86228	19.42725	22.25473
17230484	Ephx1	epoxide hydrolase 1, microsomal	501.637	600.0172	599.4435	502.4026	449.3233	475.2194
17307837	Ephx2	epoxide hydrolase 2, cytoplasmic	1224.944	1156.1	992.7248	1112.292	868.2339	990.9579
17343334	Ephx3	epoxide hydrolase 3	4.988168	4.831128	4.239282	4.801649	5.357909	5.203852
17440024	Ephx4	epoxide hydrolase 4	2.751217	2.643941	2.751217	2.679344	2.751217	2.86114
17473310	Eps8l1	EPS8-like 1	5.051213	6.710638	6.061438	6.385502	6.607846	6.347815
17484852	Eps8l2	EPS8-like 2	112.5373	122.0679	123.348	131.5129	115.4336	123.4155
17401590	Eps8l3	EPS8-like 3	4.553545	4.553545	4.553545	5.519662	4.738728	4.553545
17232634	Tube1	epsilon-tubulin 1	5.586404	6.332346	5.120306	5.120306	4.825739	5.655325
17473495	Epn1	epsin 1	97.46957	98.26188	111.7445	89.31682	95.57358	97.13976
17263837	Epn2	epsin 2	12.27065	16.77383	16.79116	15.94752	14.6969	15.22563
17267954	Epn3	epsin 3	14.45834	14.95929	10.38126	11.90297	10.92984	12.28529
17338642	Ebi3	Epstein-Barr virus induced gene 3	7.909135	8.861187	11.37142	8.436356	8.436356	8.329501
17427270	Eqtn	equatorin, sperm acrosome associated	3.062691	2.871551	3.143823	2.49545	3.485631	3.507857
17461433	Edem1	ER degradation enhancer, mannosidase alpha-like 1	304.4244	225.2916	223.1717	223.9325	256.7713	199.9021
17393336	Edem2	ER degradation enhancer, mannosidase alpha-like 2	118.2323	79.88873	77.4042	72.2948	85.28972	66.36037
17218209	Edem3	ER degradation enhancer, mannosidase alpha-like 3	113.1553	64.96091	68.08618	73.71825	74.43452	66.36589
17365014	Erlin1	ER lipid raft associated 1	60.52042	69.98433	64.20394	47.18152	62.72941	58.28651
17500275	Erlin2	ER lipid raft associated 2	46.29197	35.9864	39.86885	35.50647	33.1294	42.38377
17333521	Ermard	ER membrane associated RNA degradation	11.51602	12.50757	16.12727	17.93903	13.31933	18.53397
17420619	Emc1	ER membrane protein complex subunit 1	72.94868	77.32269	78.64646	76.49322	54.60874	69.91548
17490233	Emc10	ER membrane protein complex subunit 10	130.4976	116.2519	111.4621	119.6179	109.7221	108.4623
17311315	Emc2	ER membrane protein complex subunit 2	135.4203	111.2645	115.051	125.4084	112.6294	125.4632
17469775	Emc3	ER membrane protein complex subunit 3	116.5052	103.3396	104.6088	120.0274	109.1827	115.1963
17389324	Emc4	ER membrane protein complex subunit 4	86.36112	75.42757	63.52004	75.42757	61.17414	73.09597
17265727	Emc6	ER membrane protein complex subunit 6	69.50958	54.38776	59.22195	53.42859	52.92729	59.65164
17374295	Emc7	ER membrane protein complex subunit 7	165.428	138.3444	134.4196	136.8774	137.9183	149.1996
17513540	Emc8	ER membrane protein complex subunit 8	54.96533	83.25183	83.46619	75.19527	81.36125	85.06301
17306696	Emc9	ER membrane protein complex subunit 9	30.69943	35.81197	33.36654	36.60072	31.1595	41.81243
17266322	Eral1	Era (G-protein)-like 1 (E. coli)	36.71073	39.84978	38.90382	42.41375	35.14871	37.03327
17421972	Errfi1	ERBB receptor feedback inhibitor 1	1211.106	1205.587	909.7649	856.8388	882.0459	937.2564
17295877	Erbb2ip	Erbb2 interacting protein	341.5611	379.4142	299.3107	324.9604	311.7228	318.9287
17472927	Ergic2	ERGIC and golgi 2	19.01505	16.28125	14.31296	16.38303	19.04249	17.44232
17378507	Ergic3	ERGIC and golgi 3	120.7316	127.9193	137.0615	122.1305	118.1716	114.3823
17385398	Ermn	ermin, ERM-like protein	2.505051	2.447502	2.18792	2.53204	2.623786	2.714883
17305636	Ero1l	ERO1-like (S. cerevisiae)	103.0978	73.45353	74.41234	70.978	82.69969	59.93472
17285204	Ero1lb	ERO1-like beta (S. cerevisiae)	283.5659	107.6019	85.38022	120.2923	117.0596	81.76265
17429316	Ermap	erythroblast membrane-associated protein	5.698965	4.945684	5.617429	4.733476	7.09907	5.698965
17430729	Epb41	erythrocyte membrane protein band 4.1	82.74613	98.36956	105.8428	92.21886	90.02352	100.3113
17378600	Epb41l1	erythrocyte membrane protein band 4.1 like 1	21.43847	23.84856	17.66253	18.19676	18.22782	18.29985
17232283	Epb41l2	erythrocyte membrane protein band 4.1 like 2	35.5683	24.47505	29.86865	25.1867	24.19572	25.00076
17339313	Epb41l3	erythrocyte membrane protein band 4.1 like 3	11.45146	12.73909	14.45924	11.21074	9.57432	9.500214
17353378	Epb41l4a	erythrocyte membrane protein band 4.1 like 4a	4.988686	5.141894	5.544395	4.647678	4.542322	5.077647
17349361	Epb41l4aos	erythrocyte membrane protein band 4.1 like 4a, opposite strand	21.96295	22.35879	22.70865	28.18333	30.08954	29.05974
17425533	Epb41l4b	erythrocyte membrane protein band 4.1 like 4b	39.35268	36.33001	35.38657	35.38657	31.97162	29.96113
17226203	Epb41l5	erythrocyte membrane protein band 4.1 like 5	82.07495	83.33323	71.89502	68.3941	75.86118	63.52225
17390371	Epb42	erythrocyte membrane protein band 4.2	4.681043	5.732164	5.323297	6.357105	5.323297	4.839842
17546774	Erdr1	erythroid differentiation regulator 1	303.9542	566.1341	245.0479	346.5693	87.7891	397.7615
17484112	Edrf1	erythroid differentiation regulatory factor 1	35.2441	25.58471	23.56592	27.42786	26.49871	30.37853
17454033	Epo	erythropoietin	6.837268	5.164505	6.345835	5.853461	5.664584	5.561484
17524847	Epor	erythropoietin receptor	7.765183	8.682798	8.743495	9.357869	6.441292	8.743495
17539927	Eras	ES cell-expressed Ras	3.476366	3.717802	4.177414	4.98105	4.348416	4.739254
17392209	Esf1	ESF1, nucleolar pre-rRNA processing protein, homolog (S. cerevisiae)	24.26845	21.83484	18.09225	21.9631	21.65683	21.00004
17433436	Espn	espin	11.77545	12.73	12.53708	12.65916	11.80847	12.49484
17215738	Espnl	espin-like	15.18947	11.32626	12.04147	11.75734	14.05615	11.31136
17268031	Eme1	essential meiotic endonuclease 1 homolog 1 (S. pombe)	3.774407	4.631598	3.648415	4.20927	3.791398	4.631598
17342051	Eme2	essential meiotic endonuclease 1 homolog 2 (S. pombe)	23.23745	20.96862	19.9737	21.4303	20.69141	20.70346
17464832	AA545190	EST AA545190	2.355233	2.953407	3.351089	2.544972	2.51809	3.439622
17352662	Esco1	establishment of cohesion 1 homolog 1 (S. cerevisiae)	38.07521	39.77776	36.74052	32.81014	38.34164	45.27648
17307774	Esco2	establishment of cohesion 1 homolog 2 (S. cerevisiae)	7.913193	6.496758	6.848937	6.861148	6.216015	7.354565
17301932	Esd	esterase D/formylglutathione hydrolase	14.19401	12.75552	16.03797	20.59697	13.7145	21.04857
17231461	Esr1	estrogen receptor 1 (alpha)	43.82281	43.50532	47.91548	46.76541	63.32925	37.86597
17281979	Esr2	estrogen receptor 2 (beta)	7.703585	9.020454	8.472161	6.980159	9.960375	8.273054
17311428	Ebag9	estrogen receptor-binding fragment-associated gene 9	48.2946	48.95112	49.48708	56.45072	50.88646	55.28936
17362111	Esrra	estrogen related receptor, alpha	28.81584	32.80469	40.20324	40.34338	28.44356	39.04051
17277504	Esrrb	estrogen related receptor, beta	6.845662	6.452508	7.262821	6.992896	7.605853	8.30759
17220627	Esrrg	estrogen-related receptor gamma	17.33193	18.48253	15.96577	16.90209	15.50434	17.55111
17287969	Etohd2	ethanol decreased 2	4.506571	4.970428	4.250345	4.609755	4.11217	5.134131
17380578	Etohi1	ethanol induced 1	14.31296	13.98153	16.73562	10.86405	13.23256	12.71544
17464165	Etnk1	ethanolamine kinase 1	156.6651	110.3008	106.7136	104.8578	94.1083	96.58518
17217350	Etnk2	ethanolamine kinase 2	173.7176	223.7137	206.3787	250.105	249.0739	278.4839
17402642	Etnppl	ethanolamine phosphate phospholyase	340.9958	246.5181	209.5819	406.0434	262.574	383.1432
17435771	Ept1	ethanolaminephosphotransferase 1 (CDP-ethanolamine-specific)	138.2083	118.2396	118.0079	125.9773	103.7876	104.6421
17475026	Ethe1	ethylmalonic encephalopathy 1	160.069	176.2305	158.6947	192.9777	158.6947	184.9806
17525548	Ei24	etoposide induced 2.4 mRNA	386.3889	414.2822	406.835	413.9263	412.4084	450.2836
17388774	Ehf	ets homologous factor	4.556828	3.661919	3.97405	4.235405	4.135638	4.217096
17275123	Etv1	ets variant 1	6.715664	8.51685	10.50053	8.94896	8.634365	9.083574
17489229	Etv2	ets variant 2	7.863382	8.032338	6.866762	7.447571	6.014272	7.452529

17398769	Etv3	ets variant 3	20.46122	17.58971	15.88177	16.87166	13.7645	15.81783
17398779	Etv3l	ets variant 3-like	11.23945	9.950438	11.72463	12.04081	11.72463	11.88385
17269954	Etv4	ets variant 4	8.398141	8.467112	7.796032	8.467112	9.402536	8.669748
17329298	Etv5	ets variant 5	11.99516	9.79829	10.3676	12.35189	9.79829	11.12752
17463673	Etv6	ets variant 6	58.09814	48.87653	64.91365	49.86999	45.82363	53.13461
17487898	Erf	Ets2 repressor factor	10.36753	13.74467	13.74467	11.22536	14.49557	18.54502
17336758	Ehmt2	euchromatic histone lysine N-methyltransferase 2	61.82764	71.46228	65.37196	62.11845	66.00484	72.01672
17382303	Ehmt1	euchromatic histone methyltransferase 1	39.48876	34.77937	35.25901	36.93381	37.68164	32.42242
17327961	Eef2kmt	eukaryotic elongation factor 2 lysine methyltransferase	21.87589	25.69009	22.42786	22.93613	18.49649	18.71898
17468719	Eefsec	eukaryotic elongation factor, selenocysteine-tRNA-specific	71.33113	75.80652	79.89039	55.34611	58.36655	66.91093
17482560	Eef2k	eukaryotic elongation factor-2 kinase	13.65588	14.95235	12.97156	13.17709	16.00366	9.829786
17529062	Eef1a1	eukaryotic translation elongation factor 1 alpha 1	30.64161	35.0157	38.81547	36.77364	37.81797	35.97443
17347353	Gm6548	eukaryotic translation elongation factor 1 alpha 1 pseudogene	92.75287	115.9895	147.6349	141.9373	102.564	142.4597
17395766	Eef1a2	eukaryotic translation elongation factor 1 alpha 2	23.0496	23.29293	22.88277	22.2279	22.2279	23.83418
17213580	Eef1b2	eukaryotic translation elongation factor 1 beta 2	251.2344	273.9578	252.2639	238.6678	272.3013	237.8091
17318210	Eef1d	eukaryotic translation elongation factor 1 delta (guanine nucleotide exchange pro	75.23281	77.38274	72.32337	61.45251	75.64905	77.86807
17291964	Eef1e1	eukaryotic translation elongation factor 1 epsilon 1	58.39771	59.14277	59.33946	50.64577	47.96898	53.62523
17357374	Eef1g	eukaryotic translation elongation factor 1 gamma	173.2774	157.7482	159.628	158.3606	159.628	139.1021
17235566	Eef2	eukaryotic translation elongation factor 2	1487.547	1384.894	1371.14	1376.387	1498.933	1345.251
17256345	Eif1	eukaryotic translation initiation factor 1	15.37112	17.26993	19.08609	19.82034	19.03668	20.15534
17548207	Eif1-ps3	eukaryotic translation initiation factor 1, pseudogene 3	78.77468	86.7541	77.89614	86.06889	85.36484	78.96711
17350356	Eif1a	eukaryotic translation initiation factor 1A	106.681	70.56496	61.06018	72.17964	102.5682	57.7993
17356401	Eif1ad	eukaryotic translation initiation factor 1A domain containing	35.52001	33.12526	32.93021	34.41105	33.39199	35.72594
17282958	Gm5039	eukaryotic translation initiation factor 1A pseudogene	2.044035	2.429535	2.481049	2.563171	4.015529	3.389706
17539158	Eif1ax	eukaryotic translation initiation factor 1A, X-linked	83.94718	88.0945	109.6947	99.05446	76.65723	76.19741
17523224	Eif1b	eukaryotic translation initiation factor 1B	47.08808	42.60675	47.28006	52.26162	60.30727	49.02175
17444398	Eif2ak1	eukaryotic translation initiation factor 2 alpha kinase 1	85.5117	80.81157	68.41117	82.1609	72.05916	81.97318
17459425	Eif2ak3	eukaryotic translation initiation factor 2 alpha kinase 3	45.97284	23.97153	27.66692	22.51477	31.37405	20.79335
17374519	Eif2ak4	eukaryotic translation initiation factor 2 alpha kinase 4	11.6856	11.64929	11.55592	11.41105	11.58005	11.76704
17276732	Eif2s1	eukaryotic translation initiation factor 2, subunit 1 alpha	75.43541	54.87891	46.85431	66.50819	59.8633	58.32449
17393192	Eif2s2	eukaryotic translation initiation factor 2, subunit 2 (beta)	24.1612	27.07595	25.16663	23.53474	24.57603	18.78709
17533959	Eif2s3x	eukaryotic translation initiation factor 2, subunit 3, structural gene X-linked	3.15277	4.514481	3.617919	2.853001	3.337251	3.2776
17543229	Eif2s3x	eukaryotic translation initiation factor 2, subunit 3, structural gene X-linked	22.93104	26.35784	21.58429	25.5544	21.94804	17.97139
17546316	Eif2s3y	eukaryotic translation initiation factor 2, subunit 3, structural gene Y-linked	63.20426	71.77104	64.29256	60.45124	77.02634	80.72256
17397783	Eif2a	eukaryotic translation initiation factor 2A	58.86426	73.0276	65.56209	59.62064	70.58355	70.2307
17347355	Eif2ak2	eukaryotic translation initiation factor 2-alpha kinase 2	47.51134	31.75782	33.81426	40.0561	30.37242	34.82786
17452873	Eif2b1	eukaryotic translation initiation factor 2B, subunit 1 (alpha)	26.17359	24.37263	21.62338	21.06155	21.43713	26.84423
17277370	Eif2b2	eukaryotic translation initiation factor 2B, subunit 2 beta	26.50844	31.08313	23.24647	27.0782	34.49439	24.39071
17417598	Eif2b3	eukaryotic translation initiation factor 2B, subunit 3	22.62538	16.14796	18.7147	17.32136	14.68376	14.21153
17446881	Eif2b4	eukaryotic translation initiation factor 2B, subunit 4 delta	21.49471	13.99762	15.30775	18.46447	20.57998	17.0733
17323888	Eif2b5	eukaryotic translation initiation factor 2B, subunit 5 epsilon	38.35519	37.93059	34.95944	37.4886	41.2917	41.37536
17538188	Eif2c5	eukaryotic translation initiation factor 2C, 5	3.979204	4.681919	4.428843	4.510071	4.1435	2.731664
17216998	Eif2d	eukaryotic translation initiation factor 2D	32.41636	39.08871	35.43394	39.05621	35.89448	33.89804
17366093	Eif3a	eukaryotic translation initiation factor 3, subunit A	327.0755	324.4337	324.2539	306.2105	335.0169	313.4236
17444080	Eif3b	eukaryotic translation initiation factor 3, subunit B	161.7251	159.9392	155.7415	151.3841	157.8259	151.9939
17496283	Eif3c	eukaryotic translation initiation factor 3, subunit C	240.8164	215.5606	223.8021	208.7077	211.8281	212.2116
17318907	Eif3d	eukaryotic translation initiation factor 3, subunit D	176.5313	140.4692	144.732	138.3261	148.7102	147.9976
17316804	Eif3e	eukaryotic translation initiation factor 3, subunit E	80.7248	65.64076	64.74065	70.55371	71.38653	68.1759
17481640	Eif3f	eukaryotic translation initiation factor 3, subunit F	93.60736	89.46739	85.21881	93.07746	106.8846	102.4794
17524523	Eif3g	eukaryotic translation initiation factor 3, subunit G	156.6445	142.6089	126.2728	136.2564	156.7835	129.8012
17316986	Eif3h	eukaryotic translation initiation factor 3, subunit H	142.8542	141.6238	141.5826	119.1017	144.8635	131.1078
17430436	Eif3i	eukaryotic translation initiation factor 3, subunit I	122.2957	138.2677	142.4775	131.1915	144.1533	135.3705
17237807	4930503E24Rik	eukaryotic translation initiation factor 3, subunit I pseudogene	2.666912	2.858285	2.154271	2.774023	3.573629	2.804046
17314512	Gm4371	eukaryotic translation initiation factor 3, subunit I pseudogene	5.599585	6.702782	5.882421	7.108228	6.506808	7.382452
17375360	Eif3j1	eukaryotic translation initiation factor 3, subunit J1	17.31079	18.77095	19.38246	19.72523	19.35279	18.65047
17354128	Eif3j2	eukaryotic translation initiation factor 3, subunit J2	42.53003	44.8551	33.52092	40.79414	47.33023	44.16097
17488595	Eif3k	eukaryotic translation initiation factor 3, subunit K	73.57175	84.81221	93.67291	84.81221	69.29455	69.82007
17312905	Eif3l	eukaryotic translation initiation factor 3, subunit L	123.3274	97.23205	80.21499	96.26303	95.92268	96.86207
17389009	Eif3m	eukaryotic translation initiation factor 3, subunit M	56.0946	57.90034	38.88926	55.97963	47.57033	46.50745
17420373	Eif4g3	eukaryotic translation initiation factor 4 gamma, 3	79.89426	79.89426	87.48962	79.0942	71.87061	74.53003
17324064	Eif4g1	eukaryotic translation initiation factor 4, gamma 1	358.5151	338.9997	334.0175	333.1458	338.0306	346.6005
17495135	Eif4g2	eukaryotic translation initiation factor 4, gamma 2	881.8442	741.37	706.1153	686.5971	666.3517	803.5589
17264844	Eif4a1	eukaryotic translation initiation factor 4A1	42.94535	41.05824	43.38848	41.16054	40.31654	43.72147
17264865	Eif4a1	eukaryotic translation initiation factor 4A1	6077.406	5839.504	5983.069	5738.866	6362.288	6268.176
17324378	Eif4a2	eukaryotic translation initiation factor 4A2	104.5095	113.615	104.07	126.1687	97.79779	128.3607
17324396	Eif4a2	eukaryotic translation initiation factor 4A2	130.7512	79.86049	72.87995	106.2066	137.6509	122.7135
17324400	Eif4a2	eukaryotic translation initiation factor 4A2	13.01903	11.05334	10.27852	13.06504	15.40108	14.20493
17272895	Eif4a3	eukaryotic translation initiation factor 4A3	18.16312	13.87042	13.54154	13.54154	11.37031	13.54154
17315255	Eif4b	eukaryotic translation initiation factor 4B	239.7405	219.4502	219.7912	210.7604	249.7516	216.1816
17403138	Eif4e	eukaryotic translation initiation factor 4E	87.2359	98.43367	87.2359	99.44108	81.4092	82.02035
17500327	Eif4ebp1	eukaryotic translation initiation factor 4E binding protein 1	87.26674	92.35704	96.11102	86.04623	92.00817	90.02664
17241228	Eif4ebp2	eukaryotic translation initiation factor 4E binding protein 2	152.1035	178.165	196.8619	178.2527	204.5387	207.1655
17349715	Eif4ebp3	eukaryotic translation initiation factor 4E binding protein 3	47.22766	37.93454	54.92762	84.68356	55.24033	43.81339
17287520	Eif4e1b	eukaryotic translation initiation factor 4E family member 1B	2.517724	3.268979	3.44468	3.619449	2.782767	2.65623
17215222	Eif4e2	eukaryotic translation initiation factor 4E member 2	30.61868	36.34664	33.54812	32.34857	31.89437	32.8675
17469446	Eif4e3	eukaryotic translation initiation factor 4E member 3	19.93871	25.96165	25.19875	29.54311	27.37828	24.03009
17246520	Eif4enif1	eukaryotic translation initiation factor 4E nuclear import factor 1	25.96111	36.04957	39.9106	34.88561	36.67436	28.33179
17453421	Eif4h	eukaryotic translation initiation factor 4H	178.6609	179.306	152.3948	183.9895	169.5092	168.7695
17279148	Eif5	eukaryotic translation initiation factor 5	352.9206	409.7954	357.0816	332.9473	334.1843	331.1245
17265082	Eif5a	eukaryotic translation initiation factor 5A	66.05473	59.55497	63.08215	61.79593	58.79616	62.23137
17396492	Eif5a2	eukaryotic translation initiation factor 5A2	6.171709	7.806144	9.079293	7.045929	8.558097	7.396332
17212018	Eif5b	eukaryotic translation initiation factor 5B	56.75557	60.19159	53.55911	58.0933	64.75771	61.34399
17393357	Eif6	eukaryotic translation initiation factor 6	85.46371	88.97203	101.1218	66.59953	81.06234	64.77477
17353541	Etf1	eukaryotic translation termination factor 1	220.0275	241.0703	234.5644	226.909	236.3285	261.3356

17459826	Eva1a	eva-1 homolog A (C. elegans)	32.57673	32.73155	32.57673	33.43553	30.84126	36.2651
17418557	Eva1b	eva-1 homolog B (C. elegans)	6.571533	7.003616	6.220541	6.739446	7.319931	6.820417
17418560	Eva1b	eva-1 homolog B (C. elegans)	11.36803	12.48029	11.34874	13.11358	16.09566	14.84587
17326996	Eva1c	eva-1 homolog C (C. elegans)	5.617429	5.617429	5.617429	7.125099	5.617429	5.617429
17458630	Evx1	even skipped homeotic gene 1 homolog	4.857049	4.577754	5.617429	5.158709	5.054541	5.070507
17386664	Evx2	even skipped homeotic gene 2 homolog	9.123011	9.679282	9.123011	9.123011	8.855661	10.32157
17260089	Ewsr1	Ewing sarcoma breakpoint region 1	57.39607	75.02295	87.21413	75.81808	70.13584	75.42757
17260786	Etaa1	Ewing tumor-associated antigen 1	16.21189	10.53377	12.72717	15.65889	15.03299	19.71703
17247485	Etaa1os	Ewing tumor-associated antigen 1, opposite strand	3.54506	3.053428	3.54506	3.859891	3.14969	4.219991
17543648	Ercc6l	excision repair cross-complementing rodent repair deficiency complementation gr	5.014202	4.066056	3.810024	4.378601	4.077147	5.56106
17474558	Ercc1	excision repair cross-complementing rodent repair deficiency, complementation gı	10.16607	11.98984	11.73446	12.95844	10.81469	10.15684
17474593	Ercc2	excision repair cross-complementing rodent repair deficiency, complementation gı	34.42656	30.26587	33.11034	32.88893	33.65619	32.23375
17349262	Ercc3	excision repair cross-complementing rodent repair deficiency, complementation gı	67.06137	60.60395	58.22795	54.50588	56.64631	46.78746
17322997	Ercc4	excision repair cross-complementing rodent repair deficiency, complementation gı	19.54083	13.49899	17.83217	14.77832	19.54015	17.57375
17212423	Ercc5	excision repair cross-complementing rodent repair deficiency, complementation gı	26.14114	29.92439	33.12067	35.9119	41.8745	45.51846
17298585	Ercc6	excision repair cross-complementing rodent repair deficiency, complementation gı	22.30322	24.69762	23.1346	25.31815	17.31932	21.26944
17288118	Ercc6l2	excision repair cross-complementing rodent repair deficiency, complementation gı	23.99148	23.99148	21.47535	25.80198	24.36114	24.36114
17289699	Ercc8	excision repaiross-complementing rodent repair deficiency, complementation grou	5.916525	6.492392	5.500604	6.302831	8.002163	4.118217
17337701	Esp1	exocrine gland secreted peptide 1	2.232654	2.661113	2.49609	3.256924	2.782767	2.401058
17337667	Esp15	exocrine gland secreted peptide 15	2.611249	2.493963	2.409226	3.301428	2.782767	3.41378
17337663	Esp16	exocrine gland secreted peptide 16	2.361429	1.810201	2.345101	2.304206	1.907747	1.741463
17337659	Esp18	exocrine gland secreted peptide 18	2.959678	3.263862	3.263862	3.125745	3.059085	3.536396
17344933	Esp23	exocrine gland secreted peptide 23	3.001929	3.712343	3.123536	3.722934	4.452143	4.671028
17337655	Esp24	exocrine gland secreted peptide 24	2.415815	2.582306	2.473751	2.065985	2.246875	3.120138
17337697	Esp3	exocrine gland secreted peptide 3	2.82929	2.17237	2.302131	2.495052	2.505329	2.332117
17337650	Esp31	exocrine gland secreted peptide 31	3.074262	2.030774	1.969078	2.218124	2.107146	2.264135
17337645	Esp34	exocrine gland secreted peptide 34	2.075804	2.268581	2.714921	2.362002	2.717575	2.494222
17344927	Esp36	exocrine gland secreted peptide 36	3.447475	2.449708	3.009972	3.009972	2.876192	3.364658
17337673	Esp38	exocrine gland secreted peptide 38	3.15861	3.641215	3.837695	2.953407	2.634465	3.016467
17337693	Esp4	exocrine gland secreted peptide 4	2.149394	2.139183	2.047538	2.248246	1.821857	2.177556
17337684	Esp6	exocrine gland secreted peptide 6	2.602179	2.945821	2.978323	2.64286	2.561023	3.250451
17337679	Esp8	exocrine gland secreted peptide 8	2.480447	2.390446	2.406322	2.956028	3.096568	2.659804
17438338	Exoc1	exocyst complex component 1	36.54241	29.30592	30.30324	35.01157	33.29445	34.02445
17291576	Exoc2	exocyst complex component 2	29.8701	30.33077	30.40844	30.81114	28.87462	30.99063
17294338	Exoc3	exocyst complex component 3	55.84979	47.31055	46.26117	45.49134	48.45353	47.83584
17512361	Exoc3l	exocyst complex component 3-like	10.21743	10.6116	11.86862	12.24686	10.09424	12.94614
17474633	Exoc3l2	exocyst complex component 3-like 2	18.92262	16.93845	19.68016	20.05444	17.35224	16.56608
17279113	Exoc3l4	exocyst complex component 3-like 4	7.984876	8.855784	8.332157	7.473427	8.438345	7.873854
17457063	Exoc4	exocyst complex component 4	57.0589	68.96871	65.66624	63.22793	63.22793	70.03383
17305856	Exoc5	exocyst complex component 5	57.9662	55.09502	44.39761	54.4802	55.49439	55.03967
17359055	Exoc6	exocyst complex component 6	67.79608	66.34289	51.92869	60.19451	67.99939	61.05849
17468298	Exoc6b	exocyst complex component 6B	32.41254	28.07667	31.1487	24.4237	27.73854	31.45898
17272307	Exoc7	exocyst complex component 7	28.48519	28.48519	29.58061	28.48519	24.98794	26.32114
17514131	Exoc8	exocyst complex component 8	23.5919	23.2228	28.83602	22.29456	23.78846	21.30013
17219848	Exo1	exonuclease 1	5.289935	6.622978	5.120306	5.289935	4.108267	4.637316
17389901	Exd1	exonuclease 3-5 domain containing 1	6.918743	7.741644	7.84645	7.698994	5.013576	6.600613
17276819	Exd2	exonuclease 3-5 domain containing 2	20.41008	24.06659	30.25182	23.97336	23.69449	25.15669
17429516	Exo5	exonuclease 5	10.24516	8.461187	8.034733	10.89489	6.183056	9.987198
17517289	Exph5	exophilin 5	8.797722	7.879532	7.228619	8.503165	9.897403	7.923896
17508766	Eri1	exoribonuclease 1	19.28383	19.31594	16.35625	20.53567	19.22458	22.01203
17495677	Eri2	exoribonuclease 2	11.08072	15.10307	12.53708	14.69792	11.28974	13.39423
17417679	Eri3	exoribonuclease 3	39.01157	37.07616	35.07424	36.22591	32.29144	33.96978
17364762	Exosc1	exosome component 1	65.58118	56.08644	65.66715	60.68592	60.60521	51.29549
17421665	Exosc10	exosome component 10	61.75354	49.14865	56.88948	46.62817	48.50461	54.15865
17369540	Exosc2	exosome component 2	34.55206	32.69436	36.49255	27.34452	30.95756	27.83793
17424937	Exosc3	exosome component 3	41.23793	32.92474	37.05099	37.5695	41.94829	47.21031
17312367	Exosc4	exosome component 4	12.36611	15.25447	14.72382	17.18586	15.3758	14.48422
17475327	Exosc5	exosome component 5	22.21574	24.78749	24.78749	25.31113	23.14791	21.74347
17505689	Exosc6	exosome component 6	118.1913	95.39092	89.60951	94.91407	94.87065	98.44527
17523555	Exosc7	exosome component 7	28.29451	33.23658	40.35596	34.96501	39.89456	38.38578
17405247	Exosc8	exosome component 8	34.11348	41.56709	33.74028	37.93506	36.54444	42.42492
17396978	Exosc9	exosome component 9	37.39724	38.27631	30.48695	43.0622	42.39435	35.25828
17317015	Ext1	exostoses (multiple) 1	115.3289	79.69349	94.04439	98.44595	89.41965	84.28535
17388476	Ext2	exostoses (multiple) 2	46.76541	41.35214	38.79907	38.69754	35.91215	39.94104
17431282	Extl1	exostoses (multiple)-like 1	12.60295	10.54794	11.31536	10.09914	8.781029	11.65551
17402001	Extl2	exostoses (multiple)-like 2	23.93683	27.95007	22.65802	23.64347	20.15184	27.06206
17307726	Extl3	exostoses (multiple)-like 3	49.86515	37.72137	40.95223	38.3653	40.88472	32.8081
17247695	Xpo1	exportin 1, CRM1 homolog (yeast)	47.65283	42.27609	55.34611	46.73333	51.06667	44.77495
17307157	Xpo4	exportin 4	35.69896	22.62365	26.98863	27.7674	35.58038	33.04912
17338006	Xpo5	exportin 5	36.38041	28.83122	25.27554	31.85372	25.93233	24.11736
17496111	Xpo6	exportin 6	72.2392	89.16094	89.72761	80.11331	77.54866	72.83399
17308454	Xpo7	exportin 7	106.6973	85.93119	77.00712	87.78424	75.1239	82.70256
17245511	Xpot	exportin, tRNA (nuclear export receptor for tRNAs)	94.42127	85.83297	90.29912	97.70903	97.83101	81.33532
17501089	AA386476	expressed sequence AA386476	6.274063	6.773117	6.941608	6.941608	6.879005	6.358031
17392903	AA387200	expressed sequence AA387200	5.830461	7.060596	8.009988	8.296369	8.420874	8.219496
17360305	AA387883	expressed sequence AA387883	3.762287	3.34144	3.371218	3.798448	2.729128	3.216362
17336316	AA388235	expressed sequence AA388235	3.787485	3.787485	4.626432	4.20927	3.787485	3.354552
17547757	AA414992	expressed sequence AA414992	3.611354	2.786801	4.189448	3.504212	2.821143	3.355947
17429430	AA415398	expressed sequence AA415398	12.27763	17.58898	13.65853	14.92046	13.13496	14.81575
17375503	AA467197	expressed sequence AA467197	4.729442	3.926734	4.214058	4.204488	4.23517	4.128392
17240291	AA474331	expressed sequence AA474331	19.81004	19.14904	17.58459	18.89994	19.58544	24.47285
17495024	AA474408	expressed sequence AA474408 [Source:MGI Symbol;Acc:MGI:3034216]	9.929148	5.412668	6.568755	7.069754	5.594581	5.042817
17302928	AA536875	expressed sequence AA536875	2.337943	2.471557	2.030169	3.105682	2.178885	3.214357
17382530	AA543186	expressed sequence AA543186	10.06155	8.713486	9.310251	10.66905	6.030928	11.9602

17268084	AA623943	expressed sequence AA623943	3.124445	2.640754	2.956643	2.839644	2.482287	4.057441
17368518	AA645442	expressed sequence AA645442 [Source:MGI Symbol;Acc:MGI:3035071]	2.933718	2.701461	2.659196	2.131472	3.131991	2.949295
17503444	AA672651	expressed sequence AA672651 [Source:MGI Symbol;Acc:MGI:2142438]	12.51737	12.21879	11.85144	13.34049	15.73058	16.49183
17352108	AA684185	expressed sequence AA684185	22.71699	23.63823	22.4895	25.8799	16.78755	22.58574
17335926	AA692955	expressed sequence AA692955	4.681043	5.241356	5.580522	5.6292	4.694235	4.349773
17455656	AA792892	expressed sequence AA792892	2.960129	2.744475	2.589583	2.26715	2.488748	2.868805
17439239	AA792892	expressed sequence AA792892 [Source:MGI Symbol;Acc:MGI:2140789]	3.465564	3.081387	3.17006	2.945556	4.902202	3.749349
17216942	AA986860	expressed sequence AA986860	10.18088	10.89149	13.21514	12.55839	16.53957	16.13901
17403701	AI115009	expressed sequence AI115009	4.592087	4.364208	3.687569	3.406334	3.309424	4.891782
17384422	AI182371	expressed sequence AI182371	1101.238	940.5169	886.1333	894.5324	936.0878	931.3502
17289685	AI197445	expressed sequence AI197445	5.849973	6.015772	6.857962	5.323149	5.043132	5.676139
17425708	AI314180	expressed sequence AI314180	121.8118	116.1391	116.1745	121.1323	114.8654	121.6076
17480041	AI314278	expressed sequence AI314278	18.32371	22.03385	19.54015	21.54095	21.47461	21.8117
17240323	AI317395	expressed sequence AI317395	86.41681	93.27315	81.36464	92.54416	96.11302	105.2926
17342740	AI413582	expressed sequence AI413582	6.932761	9.945197	10.83825	10.48228	11.56746	8.420524
17515641	AI414108	expressed sequence AI414108	3.878342	4.127534	3.784572	2.871909	3.576727	3.330549
17425364	AI427809	expressed sequence AI427809	4.516421	4.595845	5.091497	3.913447	4.352065	3.749661
17500620	AI429214	expressed sequence AI429214	3.672239	3.802979	3.13962	3.756554	3.542947	3.331242
17282021	AI463170	expressed sequence AI463170	13.3933	15.09543	17.97949	16.31582	24.64187	20.43627
17291901	AI463229	expressed sequence AI463229	9.295871	8.757748	8.073924	10.9766	10.09529	8.646043
17424243	AI464131	expressed sequence AI464131	35.5572	35.72074	37.62788	32.43771	41.63226	35.39123
17483235	AI467606	expressed sequence AI467606	5.381693	6.048385	4.998135	5.692964	7.442256	4.905431
17452562	AI480526	expressed sequence AI480526 [Source:MGI Symbol;Acc:MGI:2140910]	21.60225	30.41603	28.93773	25.24682	20.72169	28.27706
17425784	AI481877	expressed sequence AI481877	3.156094	3.0739	3.137445	3.137445	3.137445	3.137445
17401530	AI504432	expressed sequence AI504432	3.542996	3.184904	2.62748	3.147901	2.989118	3.308208
17446060	AI506816	expressed sequence AI506816	3.534803	3.510752	6.913757	4.026817	3.545625	5.223367
17421105	AI507597	expressed sequence AI507597	3.598162	3.726006	3.957968	4.650481	3.806724	3.540149
17527027	AI593442	expressed sequence AI593442	4.008521	3.557318	4.445051	3.128281	3.136727	4.269893
17212346	AI597479	expressed sequence AI597479	13.66932	15.92013	14.31296	16.31277	15.76234	19.55763
17403771	AI606473	expressed sequence AI606473	9.636438	8.597517	8.211638	8.993047	9.495606	8.068497
17230034	AI607873	expressed sequence AI607873	8.905089	13.25174	16.96301	13.15382	16.34605	15.18053
17392546	AI646519	expressed sequence AI646519	14.62515	15.30531	13.40649	15.5723	13.93992	13.49076
17338258	AI661453	expressed sequence AI661453	26.89582	24.17141	28.51423	27.75739	32.93912	26.89451
17254194	AI662270	expressed sequence AI662270	13.32635	15.50369	16.65381	12.38584	17.90053	15.49843
17356422	AI837181	expressed sequence AI837181	27.22254	33.15903	31.32147	31.71866	27.45962	29.49728
17447013	AI839979	expressed sequence AI839979	3.414703	3.592704	3.310112	4.825539	5.4248	2.942413
17362320	AI846148	expressed sequence AI846148	9.886158	11.14473	10.08053	9.039419	9.641115	7.60464
17391521	AI847159	expressed sequence AI847159	4.003467	3.752417	3.601031	3.680419	3.701757	3.730632
17479415	AI854517	expressed sequence AI854517	4.892901	4.306901	4.738104	4.649095	4.836422	4.20927
17458338	AI854703	expressed sequence AI854703	6.575634	6.204608	6.360191	5.345593	5.73443	6.194681
17489989	AI987944	expressed sequence AI987944	30.36211	24.15801	19.62336	15.23767	18.35231	18.06556
17376210	AU015228	expressed sequence AU015228	2.659804	2.659804	2.742763	3.184397	2.659804	3.264971
17329475	AU015336	expressed sequence AU015336 [Source:MGI Symbol;Acc:MGI:2146536]	2.770449	2.720251	2.70883	3.309492	3.545625	2.970859
17536278	AU015836	expressed sequence AU015836	2.29334	2.656856	2.352998	2.936566	3.282762	2.539211
17346694	AU016765	expressed sequence AU016765	2.934958	4.604869	3.703322	5.019592	4.926285	4.20927
17485480	AU018091	expressed sequence AU018091	4.684743	4.20927	4.886969	5.104183	4.886969	4.953334
17526850	AU019823	expressed sequence AU019823	9.237932	6.399237	6.296674	7.933294	7.672783	8.733743
17376599	AU019990	expressed sequence AU019990	3.16738	3.904858	4.099989	3.575463	3.485172	3.605652
17492239	AU020206	expressed sequence AU020206 [Source:MGI Symbol;Acc:MGI:2142134]	16.07247	16.82401	17.10552	15.30435	11.88566	15.30435
17315163	AU021063	expressed sequence AU021063	9.406513	7.22917	6.657544	8.811026	7.935109	7.935109
17327950	AU021092	expressed sequence AU021092	11.38459	10.24274	10.69808	7.227166	8.573593	10.04232
17429341	AU022252	expressed sequence AU022252	56.86937	52.40709	57.43905	60.32232	57.46255	61.70776
17539762	AU022751	expressed sequence AU022751	8.486325	8.486325	9.310251	7.958133	8.486325	7.943088
17313852	AU022754	expressed sequence AU022754	4.582135	3.330203	3.723192	3.628115	4.279879	3.649098
17311263	AU022793	expressed sequence AU022793	3.368462	4.121787	3.884988	4.573536	5.303997	4.770796
17531804	AU023762	expressed sequence AU023762	3.62741	3.263274	3.114715	3.918224	3.263862	3.263862
17418640	AU040320	expressed sequence AU040320	30.35558	25.57035	31.15626	28.21871	29.02793	29.02793
17266581	AU040972	expressed sequence AU040972	5.180998	6.422122	5.065203	3.599159	5.325031	4.330279
17235843	AU041133	expressed sequence AU041133	4.561347	5.319203	4.212247	5.173426	5.728113	4.57547
17375154	AV039307	expressed sequence AV039307	4.732295	4.274809	4.436002	4.138535	5.352972	3.729055
17428698	AV051173	expressed sequence AV051173	5.674054	5.566923	4.687699	4.489423	4.614358	5.124851
17523847	AV064505	expressed sequence AV064505	2.785468	2.824607	2.988103	2.713735	2.824607	3.191556
17537801	AV320801	expressed sequence AV320801	2.946393	2.193538	2.428407	2.532847	3.051143	2.550818
17422904	AW011738	expressed sequence AW011738	11.50086	11.50086	12.26385	14.31296	10.84193	11.06997
17501303	AW046200	expressed sequence AW046200	7.714646	8.466819	7.821688	11.02594	6.99629	7.291421
17398989	AW047730	expressed sequence AW047730	3.07953	5.819802	5.063553	4.799453	3.670076	4.531253
17362869	AW112010	expressed sequence AW112010	137.7836	152.5058	156.1328	153.0978	199.2464	191.8892
17313616	AW121686	expressed sequence AW121686	4.040937	4.656836	3.973692	3.676696	4.040937	3.74335
17490005	AW146154	expressed sequence AW146154	18.10128	15.93835	17.28849	19.39187	18.14588	31.77544
17285398	AW209491	expressed sequence AW209491	23.71881	16.92519	18.08786	18.77317	22.00486	20.00654
17295027	AW495222	expressed sequence AW495222	4.173738	4.235523	3.673563	5.115765	5.503273	4.485473
17451930	AW549542	expressed sequence AW549542	17.54758	25.98505	21.1615	25.47959	20.86958	28.38093
17315669	AW549877	expressed sequence AW549877	72.50256	74.42782	60.16114	74.64636	75.13431	80.59285
17525746	AW551984	expressed sequence AW551984	12.83205	9.783021	8.544582	11.23143	8.720313	9.783021
17349067	AW554918	expressed sequence AW554918	13.77137	16.03064	16.13722	14.58972	19.31085	18.14035
17541784	AW822252	expressed sequence AW822252	3.826618	4.603305	5.35698	3.417734	3.354459	3.460499
17507701	BB014433	expressed sequence BB014433	3.706275	4.554365	3.995345	3.706275	3.741042	3.459468
17233507	BB019430	expressed sequence BB019430	4.081983	3.834796	4.732984	4.20927	4.134635	4.186603
17427650	BB031773	expressed sequence BB031773	4.05359	3.369256	3.549707	4.163105	4.363906	2.913675
17292608	BB123696	expressed sequence BB123696	3.245071	3.186737	3.545084	2.485627	3.520816	3.106758
17456830	BB283400	expressed sequence BB283400	5.998493	5.355941	4.918676	5.752105	5.467192	5.622142
17277659	BB287469	expressed sequence BB287469	2.830997	2.924095	2.233289	2.606909	2.393646	2.803813
17385389	BB557941	expressed sequence BB557941	2.732459	2.953407	2.630414	3.174743	2.978777	3.049579
17362966	BE692007	expressed sequence BE692007	2.406006	2.473048	2.734657	2.22251	2.827486	2.269461

17430339	C77080	expressed sequence C77080	40.02834	53.76107	52.9919	54.00758	51.17403	48.84199
17543840	C77370	expressed sequence C77370	4.01461	4.086172	4.878407	4.428813	4.09465	3.667126
17264402	C78197	expressed sequence C78197 [Source:MGI Symbol;Acc:MGI:2144636]	3.768342	5.013541	6.409403	3.837429	4.687212	3.940394
17370076	C79798	expressed sequence C79798 [Source:MGI Symbol;Acc:MGI:2139494]	2.409911	2.912282	2.860426	2.606909	2.435605	2.640992
17491273	C86187	expressed sequence C86187	6.171709	6.377496	8.308454	5.409293	7.629931	8.532745
17275596	C87198	expressed sequence C87198	3.296214	3.695732	3.470347	3.909272	3.341695	3.515549
17449827	C87414	expressed sequence C87414	6.514119	7.530418	9.769197	8.433608	8.149322	13.5927
17455691	C87414	expressed sequence C87414	2.065498	2.255364	1.979945	1.974764	2.283426	3.097748
17455717	C87414	expressed sequence C87414	3.662808	4.661486	3.948186	4.05477	4.044777	3.990916
17460433	C87436	expressed sequence C87436	35.67793	26.53142	24.25954	20.29525	26.31877	25.49443
17427081	C87499	expressed sequence C87499	3.741327	3.001971	3.250046	4.132228	4.267822	4.798222
17432521	C87977	expressed sequence C87977	2.872924	2.146431	2.422455	2.942026	2.82265	2.355952
17477055	EU599041	expressed sequence EU599041	2.204926	1.85778	2.110508	2.255364	4.036962	2.09872
17498199	R74862	expressed sequence R74862	3.799501	4.372869	5.660737	4.790313	4.681043	4.171453
17246163	Esyt1	extended synaptotagmin-like protein 1	30.30555	30.88084	38.8397	28.67795	29.01788	32.7159
17279584	Esyt2	extended synaptotagmin-like protein 2	69.03997	76.77049	69.84379	69.93487	76.42962	68.37893
17530033	Esyt3	extended synaptotagmin-like protein 3	12.93798	13.55441	12.45825	12.2204	10.86059	14.72453
17315352	Espl1	extra spindle pole bodies 1, separase	5.613731	8.386332	8.22645	9.664332	7.935109	7.500517
17407850	Ecm1	extracellular matrix protein 1	101.9947	146.5338	137.1052	127.2216	105.4525	134.2474
17287148	Ecm2	extracellular matrix protein 2, female organ and adipocyte specific	4.110774	4.110774	4.050388	4.42645	3.838147	3.673232
17544816	Esx1	extraembryonic, spermatogenesis, homeobox 1	5.99471	5.962263	5.768955	6.000572	5.48933	6.73968
17221375	Eya1	eyes absent 1 homolog (Drosophila)	5.48933	5.216058	5.871381	6.130182	5.779802	4.47448
17379692	Eya2	eyes absent 2 homolog (Drosophila)	8.458255	7.187649	5.940647	9.019016	7.617935	6.83978
17419443	Eya3	eyes absent 3 homolog (Drosophila)	43.12552	54.01432	54.31919	51.69132	43.68603	54.42019
17239719	Eya4	eyes absent 4 homolog (Drosophila)	4.438893	3.79358	4.596131	3.480875	5.063307	5.075219
17340609	Ezr	ezrin	14.97278	16.05866	20.18736	16.88523	16.78755	15.78246
17219362	F11r	F11 receptor	109.5792	118.8362	133.2951	124.1818	171.1978	137.7167
17535529	F8a	factor 8-associated gene A	5.740236	5.610619	5.405994	4.61711	4.988617	5.126236
17525363	Foxred1	FAD-dependent oxidoreductase domain containing 1	41.9355	41.23593	56.18021	41.00875	43.73329	40.72859
17318895	Foxred2	FAD-dependent oxidoreductase domain containing 2	4.681043	4.304274	4.188838	4.681043	4.681043	5.357834
17412202	Faxc	failed axon connections homolog (Drosophila)	3.580547	2.857097	3.339703	3.339703	3.087138	3.656821
17500332	Gm9731	Fam58a pseudogene	5.664977	4.301292	4.647678	4.801649	4.273387	5.521461
17442701	Fam101a	family with sequence similarity 101, member A	9.750323	12.70884	13.85284	12.37511	12.71738	12.04181
17266038	Fam101b	family with sequence similarity 101, member B	6.311837	7.525879	6.949167	6.949167	4.625182	7.838567
17369841	Fam102a	family with sequence similarity 102, member A	85.63271	99.96215	103.9078	83.75538	69.40222	78.82766
17409463	Fam102b	family with sequence similarity 102, member B	9.049073	8.911511	13.51173	11.27715	11.95258	9.568349
17479728	Fam103a1	family with sequence similarity 103, member A1	11.95987	9.943267	11.63388	9.762428	11.69768	13.36493
17271600	Fam104a	family with sequence similarity 104, member A	20.98185	21.58998	21.70747	19.16763	17.81363	22.49371
17316197	Fam105a	family with sequence similarity 105, member A	4.980878	8.063186	9.404818	7.817189	8.18215	5.950001
17303433	Fam107a	family with sequence similarity 107, member A	6.23872	6.992896	7.157422	5.802481	6.257689	7.294353
17366437	Fam107b	family with sequence similarity 107, member B	25.43847	30.39222	31.67516	24.29454	26.53124	32.74312
17441999	Fam109a	family with sequence similarity 109, member A	10.4745	11.86525	11.90792	12.69695	11.9366	13.8993
17313560	Fam109b	family with sequence similarity 109, member B	6.287864	6.988293	5.984248	6.287864	6.25873	6.942715
17392887	Fam110a	family with sequence similarity 110, member A	5.028716	6.294579	4.828751	5.663445	4.471658	5.681453
17392894	Fam110a	family with sequence similarity 110, member A	11.1828	12.27584	10.27937	12.54073	9.077972	13.69901
17411575	Fam110b	family with sequence similarity 110, member B	6.370868	6.855586	8.274488	6.433974	7.218304	5.878426
17274766	Fam110c	family with sequence similarity 110, member C	4.616566	3.416289	5.045999	4.978462	4.839842	5.174574
17357815	Fam111a	family with sequence similarity 111, member A	5.481193	7.224862	10.25852	9.369162	7.337456	5.961271
17437686	Fam114a1	family with sequence similarity 114, member A1	71.09864	54.55993	62.8099	67.09624	76.54944	63.98226
17263033	Fam114a2	family with sequence similarity 114, member A2	36.26535	33.95236	31.96652	29.01888	29.46826	34.91778
17255372	Fam117a	family with sequence similarity 117, member A	6.757127	6.394715	6.834804	5.963627	4.868273	6.927477
17213338	Fam117b	family with sequence similarity 117, member B	23.96389	31.90078	36.88034	31.89481	33.39731	34.01408
17313788	Fam118a	family with sequence similarity 118, member A	17.09864	19.23391	19.06613	14.66891	19.18039	14.69456
17313799	Fam118a	family with sequence similarity 118, member A	8.453566	13.22916	23.07134	9.418975	13.9579	13.75763
17525379	Fam118b	family with sequence similarity 118, member B	16.46568	14.1825	15.6691	13.95016	12.49397	16.51609
17292370	Fam120a	family with sequence similarity 120, member A	223.675	252.1276	258.1203	234.0025	227.5264	228.2672
17333553	Fam120b	family with sequence similarity 120, member B	30.90182	30.41266	31.13769	27.51183	26.61154	34.33148
17538683	Fam120c	family with sequence similarity 120, member C	18.521	15.89422	13.67501	18.99608	17.58142	16.38723
17287102	Fam120aos	family with sequence similarity 120A, opposite strand	19.00794	23.75336	20.70131	22.10974	19.74205	23.75221
17363624	Fam122a	family with sequence similarity 122, member A	15.63156	19.52201	19.84082	19.62336	22.14701	24.34511
17541745	Fam122b	family with sequence similarity 122, member B	6.171709	7.445042	6.076792	6.124067	6.850978	6.005981
17534730	Fam122c	family with sequence similarity 122, member C	3.824364	3.502294	4.202914	3.575488	3.627234	3.445253
17301147	Fam124a	family with sequence similarity 124, member A	21.91672	17.36475	21.04448	18.29364	16.55506	14.88833
17224783	Fam124b	family with sequence similarity 124, member B	6.560065	9.382615	5.463744	5.386134	4.122787	5.494355
17446100	Fam126a	family with sequence similarity 126, member A	13.14926	18.49611	20.68481	15.8711	17.47524	17.92076
17223412	Fam126b	family with sequence similarity 126, member B	30.66413	26.89955	23.82427	27.86843	20.13649	29.30246
17218186	Fam129a	family with sequence similarity 129, member A	7.539088	10.4476	10.38205	10.67379	8.766373	8.091011
17370002	Fam129b	family with sequence similarity 129, member B	17.03854	15.3561	19.65957	17.36523	17.84169	14.87997
17502260	Fam129c	family with sequence similarity 129, member C	8.932166	7.509464	8.943627	7.690141	6.707337	7.792196
17467209	Fam13a	family with sequence similarity 13, member A	49.26257	53.86439	63.08303	57.49801	57.29431	93.30872
17353420	Fam13b	family with sequence similarity 13, member B	66.80388	69.46864	54.76491	59.79363	62.33887	54.08663
17234146	Fam13c	family with sequence similarity 13, member C	7.200914	7.104041	7.698994	7.946007	7.104041	6.610594
17324112	Fam131a	family with sequence similarity 131, member A	8.144409	6.992896	6.9431	5.532302	7.320387	6.309038
17466386	Fam131b	family with sequence similarity 131, member B	9.890202	9.999667	9.803725	12.00223	9.155762	10.29855
17421060	Fam131c	family with sequence similarity 131, member C	17.21185	22.63102	19.58911	16.68824	12.73529	18.84278
17422832	Fam132a	family with sequence similarity 132, member A	5.259334	5.587876	6.591012	5.051914	6.171709	6.171709
17215762	Fam132b	family with sequence similarity 132, member B	7.680446	9.340631	6.981514	8.832489	9.518076	8.51685
17434310	Fam133b	family with sequence similarity 133, member B	30.89176	30.96121	30.96121	28.99073	30.96121	29.0531
17214409	Fam134a	family with sequence similarity 134, member A	149.088	164.9266	167.8699	147.3272	162.236	178.228
17310637	Fam134b	family with sequence similarity 134, member B	107.5877	129.9056	124.1663	126.1809	206.1928	208.8698
17269769	Fam134c	family with sequence similarity 134, member C	78.96	94.41604	89.93183	84.79163	93.86009	80.55599
17221792	Fam135a	family with sequence similarity 135, member A	66.50819	66.8604	54.24619	63.69243	51.01996	51.34956
17317717	Fam135b	family with sequence similarity 135, member B	2.659196	2.955414	3.202192	3.662325	2.780029	2.565091
17460393	Fam136a	family with sequence similarity 136, member A	53.89427	45.62776	56.56979	77.61857	88.11587	63.19507

17310814	Fam136b-ps	family with sequence similarity 136, member B, pseudogene [Source:MGI Symbol;A	9.648536	4.775428	6.059799	9.605296	5.803728	5.724898
17509083	Fam149a	family with sequence similarity 149, member A	33.65432	29.36102	34.74949	31.28523	29.1378	28.73346
17297425	Fam149b	family with sequence similarity 149, member B	48.60625	43.5841	47.46394	50.02093	43.9969	39.76783
17210947	Fam150a	family with sequence similarity 150, member A	2.623349	2.688013	2.816357	3.107146	3.424278	2.657537
17274744	Fam150b	family with sequence similarity 150, member B	7.885051	7.708072	6.397863	7.41476	5.6556	6.544849
17294899	Fam151b	family with sequence similarity 151, member B	19.30122	21.49471	19.49997	19.38623	22.33787	21.06301
17507347	Fam155a	family with sequence similarity 155, member A	3.767428	3.777129	3.997367	3.864763	3.947447	4.430379
17428050	Fam159a	family with sequence similarity 159, member A	9.279004	8.891951	9.206227	8.158761	7.510505	8.420524
17295981	Fam159b	family with sequence similarity 159, member B	4.161143	6.595536	7.455887	6.052957	7.13622	8.103527
17406403	Fam160a1	family with sequence similarity 160, member A1	13.30992	10.75697	10.93067	12.18083	11.56762	8.231469
17406421	Fam160a1	family with sequence similarity 160, member A1	12.81712	8.771053	6.903414	8.703224	6.181556	6.391391
17494482	Fam160a2	family with sequence similarity 160, member A2	33.10782	34.41844	28.60588	34.41844	34.41844	34.46285
17360655	Fam160b1	family with sequence similarity 160, member B1	133.5522	145.3412	133.1529	128.8832	135.318	134.185
17308395	Fam160b2	family with sequence similarity 160, member B2	15.74443	17.93629	17.17978	21.52677	15.5009	18.72573
17247675	Fam161a	family with sequence similarity 161, member A	5.40326	4.889104	5.588147	5.997578	5.001081	4.755114
17282486	Fam161b	family with sequence similarity 161, member B	5.357983	5.04488	5.948888	6.37975	6.239801	5.948888
17330135	Fam162a	family with sequence similarity 162, member A	114.1874	133.9611	142.6791	132.3652	167.3738	155.8593
17240729	Fam162b	family with sequence similarity 162, member B	4.273594	4.768746	4.006021	4.537602	4.20927	3.976861
17228490	Fam163a	family with sequence similarity 163, member A	8.704733	10.55504	8.454093	8.46858	8.45931	8.12169
17383241	Fam163b	family with sequence similarity 163, member B	8.242774	7.172768	5.363111	7.513838	7.488413	6.01865
17367858	Fam166a	family with sequence similarity 166, member A	6.216963	7.698994	6.604869	7.035924	7.649206	8.829183
17424596	Fam166b	family with sequence similarity 166, member B	3.749946	4.430747	4.092578	4.168303	4.379542	4.76313
17301225	Fam167a	family with sequence similarity 167, member A	9.405796	8.503165	9.915874	8.536709	11.14736	10.18539
17430430	Fam167b	family with sequence similarity 167, member B	14.76941	12.51009	17.60179	11.23642	11.56746	8.786143
17480777	Fam168a	family with sequence similarity 168, member A	57.3905	53.69553	56.90881	52.35009	43.2117	52.37694
17222096	Fam168b	family with sequence similarity 168, member B	53.18362	58.71226	66.27717	57.24231	62.45867	64.04038
17289329	Fam169a	family with sequence similarity 169, member A	3.322018	3.84001	3.541793	3.720251	4.001137	4.559703
17479125	Fam169b	family with sequence similarity 169, member B	25.86788	29.33355	31.62426	33.09245	22.30971	28.45795
17350545	Fam170a	family with sequence similarity 170, member A	4.834846	4.566789	4.204429	4.555439	5.131818	4.571508
17298626	Fam170b	family with sequence similarity 170, member B	26.31439	21.4694	19.98367	28.07687	16.52135	24.85424
17366359	Fam171a1	family with sequence similarity 171, member A1	12.8454	10.50329	14.55013	9.376373	10.70275	13.9292
17270211	Fam171a2	family with sequence similarity 171, member A2	13.33402	13.57246	14.06415	11.73715	8.893924	11.4968
17372552	Fam171b	family with sequence similarity 171, member B	7.669699	11.29326	6.63543	7.669699	7.508892	8.280857
17288838	Fam172a	family with sequence similarity 172, member A	21.26811	26.87327	21.99754	22.02317	28.38801	18.25424
17342376	Fam173a	family with sequence similarity 173, member A	48.47671	38.88145	38.66537	37.04046	39.96098	41.35099
17310827	Fam173b	family with sequence similarity 173, member B	34.18473	34.31054	38.2021	35.84252	43.27704	35.20333
17216158	Fam174a	family with sequence similarity 174, member A	25.84924	28.82123	27.14868	28.91187	30.40655	34.15749
17479183	Fam174b	family with sequence similarity 174, member B	16.23708	15.50434	15.50434	14.15971	15.50434	13.97029
17450180	Fam175a	family with sequence similarity 175, member A	22.12047	26.32114	32.73019	30.37895	24.51393	28.04341
17450196	Fam175a	family with sequence similarity 175, member A	7.95703	9.441198	8.200546	7.698994	9.382094	6.788016
17484080	Fam175b	family with sequence similarity 175, member B	71.11638	71.48408	73.91114	65.55482	66.71087	67.49693
17359742	Fam178a	family with sequence similarity 178, member A	29.80722	29.54516	29.03055	28.98089	28.15248	31.2144
17222232	Fam178b	family with sequence similarity 178, member B	5.48431	4.952854	5.101318	4.311769	5.101318	5.091109
17339503	Fam179a	family with sequence similarity 179, member A	9.123011	9.143839	8.528488	9.407165	8.26503	11.16189
17275785	Fam179b	family with sequence similarity 179, member B	39.71054	25.73589	28.71654	34.26562	27.26787	24.59631
17465824	Fam180a	family with sequence similarity 180, member A	4.221366	8.050509	5.801114	6.365262	6.152616	7.664119
17278180	Fam181a	family with sequence similarity 181, member A	10.41665	9.908248	13.86254	10.41665	9.755731	11.37567
17480233	Fam181b	family with sequence similarity 181, member B	6.531933	6.996678	5.691978	5.442955	5.145923	6.338537
17263193	Fam183b	family with sequence similarity 183, member B	3.456447	3.660067	4.101097	4.192527	4.823933	4.22608
17240858	Fam184a	family with sequence similarity 184, member A	6.779599	5.48933	6.076329	4.94493	4.352065	4.86045
17447921	Fam184b	family with sequence similarity 184, member B	8.473042	9.955819	8.2301	9.18895	8.661639	9.307024
17435095	Fam185a	family with sequence similarity 185, member A	25.44782	26.00776	20.06042	27.05025	22.04149	24.10733
17321513	Fam186b	family with sequence similarity 186, member B	4.297775	4.025394	4.632047	4.515826	3.986465	4.297775
17257038	Fam187a	family with sequence similarity 187, member A	3.272311	4.205887	4.238224	4.739156	2.973269	4.15656
17476571	Fam187b	family with sequence similarity 187, member B	6.171709	7.541806	8.489326	8.489326	8.489326	10.68252
17381752	Fam188a	family with sequence similarity 188, member A	100.9747	77.20059	70.26041	79.20063	73.69577	66.21426
17458790	Fam188b	family with sequence similarity 188, member B	7.021796	6.818007	6.528538	6.805447	7.030221	6.718038
17405443	Fam188b2-ps	family with sequence similarity 188, member B2, pseudogene	3.211372	4.107071	4.002468	2.735453	2.964626	3.211372
17491831	Fam189a1	family with sequence similarity 189, member A1	19.91951	19.37091	19.96623	20.10984	21.41515	21.76473
17363559	Fam189a2	family with sequence similarity 189, member A2	8.863583	7.821905	7.056535	6.992896	5.741668	6.195153
17399314	Fam189b	family with sequence similarity 189, member B	6.188115	7.397409	7.216103	5.495688	4.898534	5.958503
17461114	Fam19a1	family with sequence similarity 19, member A1	4.386408	4.855063	5.161677	4.319906	3.732856	3.791792
17237787	Fam19a2	family with sequence similarity 19, member A2	5.431573	3.896962	4.227691	5.295915	4.610426	4.046094
17408755	Fam19a3	family with sequence similarity 19, member A3	7.316684	10.04211	8.222004	8.51685	5.494697	9.744918
17469282	Fam19a4	family with sequence similarity 19, member A4	5.628264	6.962522	6.512403	6.446205	6.437899	7.612868
17313976	Fam19a5	family with sequence similarity 19, member A5	8.167191	7.987865	8.181696	7.104041	8.422217	7.449843
17511868	Fam192a	family with sequence similarity 192, member A	89.09422	74.15541	83.66777	91.5165	94.3722	100.028
17436583	Fam193a	family with sequence similarity 193, member A	46.74208	49.46547	49.46547	48.75607	39.81665	45.56108
17292941	Fam193b	family with sequence similarity 193, member B	26.11669	25.34958	24.62787	35.29446	22.71274	22.29534
17342483	Fam195a	family with sequence similarity 195, member A	50.41915	56.78469	50.29454	57.93125	43.96215	63.31827
17273152	Fam195b	family with sequence similarity 195, member B	32.28853	38.01245	41.42788	46.95478	35.70217	38.62786
17497303	Fam196a	family with sequence similarity 196, member A	7.387263	8.799979	7.882428	7.692646	8.628142	7.692646
17248412	Fam196b	family with sequence similarity 196, member B	4.681043	4.681043	4.681043	4.294619	5.775637	4.681043
17523387	Fam198a	family with sequence similarity 198, member A	16.28182	10.86382	13.04215	20.78095	23.83038	15.98548
17398406	Fam198b	family with sequence similarity 198, member B	5.275727	3.545306	4.141548	4.887714	4.352065	4.521495
17537993	Fam199x	family with sequence similarity 199, X-linked	16.33404	15.71946	18.17017	16.81574	15.50659	19.09099
17271281	Fam20a	family with sequence similarity 20, member A	77.29001	86.44257	83.94951	76.59774	104.6363	94.10112
17228576	Fam20b	family with sequence similarity 20, member B	39.84951	34.838	40.3929	37.02454	37.57705	36.12235
17443901	Fam20c	family with sequence similarity 20, member C	52.65505	36.72168	39.77252	30.08266	38.62786	34.02469
17366067	Fam204a	family with sequence similarity 204, member A	14.62931	22.89372	19.99046	21.37689	17.54794	20.37432
17424468	Fam205a1	family with sequence similarity 205, member A1	7.709889	7.850366	8.493034	10.37292	11.60118	7.308017
17424476	Fam205a1	family with sequence similarity 205, member A1	3.791135	4.825706	3.085598	3.984669	4.035843	3.439837
17412987	Fam205a2	family with sequence similarity 205, member A2	9.230956	8.014042	10.13792	9.057482	7.637497	8.467053
17413094	Fam205a2	family with sequence similarity 205, member A2	11.12513	11.47952	15.03424	13.46386	12.43651	12.16437

17424415	Fam205a2	family with sequence similarity 205, member A2	11.12513	11.47952	15.03424	13.46386	12.43651	12.16437
17434129	Fam205a2	family with sequence similarity 205, member A2	9.230956	8.014042	10.13792	9.057482	7.637497	8.467053
17424483	Fam205c	family with sequence similarity 205, member C	3.591061	3.211372	2.673853	3.422309	3.47868	3.476149
17414331	Fam206a	family with sequence similarity 206, member A	9.446959	8.24809	10.75034	8.670709	7.10414	8.24809
17242296	Fam207a	family with sequence similarity 207, member A	57.10242	55.34918	51.42115	56.43076	65.53801	56.3733
17298057	Fam208a	family with sequence similarity 208, member A	73.78215	75.16244	69.68566	85.95979	83.59357	92.46529
17290217	Fam208b	family with sequence similarity 208, member B	30.41477	27.56107	25.13507	28.74283	29.5425	24.70584
17380147	Fam209	family with sequence similarity 209	7.268915	7.793979	7.517016	8.078122	6.717666	7.870635
17462059	Fam21	family with sequence similarity 21	84.20477	105.3495	100.9572	86.56068	111.8623	122.3432
17355256	Fam210a	family with sequence similarity 210, member A	96.38923	77.81466	68.48851	86.67476	79.81234	82.1609
17380102	Fam210b	family with sequence similarity 210, member B	66.32358	65.57027	60.88901	65.41955	66.49436	48.06551
17531075	Fam212a	family with sequence similarity 212, member A	5.48815	5.530002	5.560598	7.801329	5.319166	5.480927
17401408	Fam212b	family with sequence similarity 212, member B	5.287296	5.528354	6.287864	5.701336	5.810612	5.334858
17305221	Fam213a	family with sequence similarity 213, member A	130.6189	140.1755	136.5069	118.27	81.82104	85.49452
17433672	Fam213b	family with sequence similarity 213, member B	18.9624	29.48972	23.8279	25.8695	18.63238	19.77506
17519364	Fam214a	family with sequence similarity 214, member A	81.60694	78.40806	94.88447	109.1963	106.4329	119.4324
17424573	Fam214b	family with sequence similarity 214, member B	12.99343	13.5608	14.97319	14.951	14.08707	14.28699
17452396	Fam216a	family with sequence similarity 216, member A	9.225774	11.95871	12.95152	11.37031	12.44091	12.54334
17308808	Fam216b	family with sequence similarity 216, member B	8.585487	10.92991	11.86977	10.70231	9.310251	12.28529
17291812	Fam217a	family with sequence similarity 217, member A	3.797053	3.432501	3.716282	3.128107	3.416147	2.998062
17380604	Fam217b	family with sequence similarity 217, member B	4.319158	5.880505	3.611705	4.366387	5.031993	4.168678
17424263	Fam219a	family with sequence similarity 219, member A	11.16197	9.627625	10.94674	10.71129	10.52771	10.37428
17412875	Fam219aos	family with sequence similarity 219, member A, opposite strand	6.396089	5.802763	7.158464	7.982884	5.68358	7.453097
17517733	Fam219b	family with sequence similarity 219, member B	7.26554	8.238376	6.704919	7.698994	7.008472	8.327884
17444364	Fam220a	family with sequence similarity 220, member A	28.40317	24.87837	27.65139	27.25294	23.45429	26.56104
17458472	Fam221a	family with sequence similarity 221, member A	3.462322	3.894235	3.570287	3.056117	3.296991	3.894235
17424792	Fam221b	family with sequence similarity 221, member B	8.278217	11.01403	8.278217	11.20034	7.521602	6.347815
17440974	Fam222a	family with sequence similarity 222, member A	14.09459	12.23787	12.36577	13.49731	13.21578	9.037602
17253452	Fam222b	family with sequence similarity 222, member B	34.27726	40.10305	39.73642	39.33332	36.03008	37.56117
17319265	Fam227a	family with sequence similarity 227, member A	4.655125	5.03454	5.72986	5.56106	5.56106	5.56106
17391036	Fam227b	family with sequence similarity 227, member B	3.362793	2.560064	2.828368	3.028913	4.017895	3.292789
17279837	Fam228a	family with sequence similarity 228, member A	6.079742	5.525749	5.455646	7.186902	6.026874	6.018233
17279849	Fam228b	family with sequence similarity 228, member B	7.099365	5.322116	5.88344	5.979356	5.84357	6.589276
17419015	Fam229a	family with sequence similarity 229, member A	11.68455	13.75315	8.765157	12.09493	11.63234	10.38148
17240239	Fam229b	family with sequence similarity 229, member B	3.720174	3.944575	3.717364	3.681924	3.565464	4.387898
17342625	Fam234a	family with sequence similarity 234, member A	61.34611	61.4802	52.97538	52.20189	51.66338	49.5705
17463761	Fam234b	family with sequence similarity 234, member B	34.71073	33.39847	36.96966	31.53955	24.06492	27.8443
17484000	Fam24a	family with sequence similarity 24, member A	2.632676	2.375482	2.570858	2.671907	5.128262	2.420659
17305028	Fam25c	family with sequence similarity 25, member C	39.35213	30.69113	34.39126	28.9249	41.76377	27.22349
17240176	Fam26d	family with sequence similarity 26, member D	3.95838	4.739221	4.836153	3.915129	4.893783	4.03741
17240181	Fam26e	family with sequence similarity 26, member E	4.191016	6.218946	5.630644	5.050303	5.859591	5.627718
17240186	Fam26f	family with sequence similarity 26, member F	8.050621	5.999532	6.078688	6.399326	7.143197	7.122827
17542678	Fam3a	family with sequence similarity 3, member A	22.73921	20.02708	29.16589	20.78009	22.29224	21.38949
17332554	Fam3b	family with sequence similarity 3, member B	3.023438	2.691704	2.956233	3.26038	2.946904	2.826918
17465062	Fam3c	family with sequence similarity 3, member C	86.78237	89.31063	82.8368	82.72893	91.96226	86.10529
17502414	Fam32a	family with sequence similarity 32, member A	104.1912	105.6369	129.2813	114.2777	156.964	142.0662
17305015	Fam35a	family with sequence similarity 35, member A	40.49551	32.20427	26.36533	40.9196	47.58319	40.64427
17324637	Fam43a	family with sequence similarity 43, member A	25.41327	27.19576	25.074	23.11921	13.84986	24.01377
17431768	Fam43b	family with sequence similarity 43, member B	9.409708	12.9774	14.30483	15.68333	13.46582	14.66942
17360877	Fam45a	family with sequence similarity 45, member A	73.30552	70.48918	72.57446	68.19197	68.19197	64.22798
17529316	Fam46a	family with sequence similarity 46, member A	23.90748	15.81926	22.68631	23.39389	19.49537	20.05
17419608	Fam46b	family with sequence similarity 46, member B	11.50086	11.50086	9.176856	14.07946	10.40554	14.14784
17408414	Fam46c	family with sequence similarity 46, member C	22.07146	24.85855	29.98336	28.28588	21.54214	20.82811
17537163	Fam46d	family with sequence similarity 46, member D	2.179746	2.036949	2.210435	2.169616	2.19512	2.20436
17536065	Fam47c	family with sequence similarity 47, member C	3.200972	2.684382	3.200972	3.061927	3.373205	3.432096
17439116	Fam47e	family with sequence similarity 47, member E	15.73974	15.38999	12.40234	17.94848	18.52678	17.69684
17274099	Fam49a	family with sequence similarity 49, member A	23.75238	29.41112	26.88109	21.54095	19.44355	18.91842
17317486	Fam49b	family with sequence similarity 49, member B	26.50128	23.24696	32.36068	24.67999	31.49149	24.56837
17535826	Fam50a	family with sequence similarity 50, member A	56.20358	46.87998	50.29454	39.54847	60.49693	51.68307
17286510	Fam50b	family with sequence similarity 50, member B	2.813391	2.785662	2.601115	2.891424	2.729525	2.915231
17447113	Fam53a	family with sequence similarity 53, member A	15.46302	16.63823	16.39893	17.61021	16.15759	15.88034
17497113	Fam53b	family with sequence similarity 53, member B	17.00739	19.12914	21.1615	19.95862	12.50021	21.24689
17349465	Fam53c	family with sequence similarity 53, member C	45.52793	42.16108	40.93946	40.33471	45.50143	40.93946
17253133	Fam57a	family with sequence similarity 57, member A	32.9679	24.6458	31.95213	28.56111	28.12381	23.51941
17483133	Fam57b	family with sequence similarity 57, member B	6.865953	6.316871	6.004608	6.628259	6.088091	6.239801
17266516	Fam58b	family with sequence similarity 58, member B	51.68917	48.06717	46.72588	51.51533	51.87113	59.02155
17473047	Fam60a	family with sequence similarity 60, member A	19.36608	17.42637	20.29525	17.42637	15.15719	19.66461
17400291	Fam63a	family with sequence similarity 63, member A	56.20046	67.03674	66.60045	74.71831	63.40523	57.3901
17528617	Fam63b	family with sequence similarity 63, member B	21.73367	20.40202	27.22047	28.62254	32.14762	27.5248
17252310	Fam64a	family with sequence similarity 64, member A	9.361307	9.361307	9.241199	10.15044	9.361307	9.361307
17504837	Fam65a	family with sequence similarity 65, member A	15.8083	20.22854	19.70432	17.04315	16.975	18.10128
17285964	Fam65b	family with sequence similarity 65, member B	9.776649	14.76572	12.53708	12.30148	11.59065	12.19367
17394740	Fam65c	family with sequence similarity 65, member C	6.727078	7.312932	7.698994	7.709568	8.073308	8.044429
17450718	Fam69a	family with sequence similarity 69, member A	25.4457	23.84079	22.18465	29.97442	16.81888	25.29324
17368349	Fam69b	family with sequence similarity 69, member B	6.009646	6.764578	6.320111	6.711057	5.881891	6.070856
17352216	Fam69c	family with sequence similarity 69, member C	4.647678	4.60771	4.687017	5.783689	5.735622	6.347815
17231029	Fam71a	family with sequence similarity 71, member A	3.689947	3.606151	3.410374	3.203014	3.656694	4.593583
17248793	Fam71b	family with sequence similarity 71, member B	6.3142	5.386134	5.291182	5.386134	5.102945	5.830178
17276702	Fam71d	family with sequence similarity 71, member D	4.278633	2.927481	3.027737	3.61985	3.5108	3.22829
17477498	Fam71e1	family with sequence similarity 71, member E1	4.968085	5.096935	5.051388	5.48933	5.697978	5.776798
17485910	Fam71e2	family with sequence similarity 71, member E2	3.122129	2.768971	3.015025	3.363656	3.587702	3.898043
17456569	Fam71f1	family with sequence similarity 71, member F1	6.675195	7.097987	5.811578	6.711336	6.20749	6.675195
17456560	Fam71f2	family with sequence similarity 71, member F2	6.088935	7.014645	6.95982	7.020336	6.194552	6.295976
17217022	Fam72a	family with sequence similarity 72, member A	4.295184	4.524035	4.39819	4.24793	4.604964	3.162992

17411216	Fam73a	family with sequence similarity 73, member A	13.6277	14.10456	14.31296	11.79726	14.10456	13.68203
17369196	Fam73b	family with sequence similarity 73, member B	61.76296	58.22876	68.62061	59.29999	53.2039	62.86913
17430945	Fam76a	family with sequence similarity 76, member A	20.03096	28.86541	27.74143	24.38294	29.5802	26.26045
17514719	Fam76b	family with sequence similarity 76, member B	25.77356	28.62969	24.1818	25.33166	19.87854	22.07544
17383819	Fam78a	family with sequence similarity 78, member A	11.59676	11.09333	11.25754	12.11884	10.26719	11.83264
17383825	Fam78a	family with sequence similarity 78, member A	5.084585	7.225262	6.287864	6.287864	6.287864	6.287864
17219079	Fam78b	family with sequence similarity 78, member B	8.63227	7.577595	6.55511	6.459578	8.31848	7.303004
17287024	Fam8a1	family with sequence similarity 8, member A1	138.1546	106.5731	104.4269	111.1227	111.4552	119.9168
17528568	Fam81a	family with sequence similarity 81, member A	11.02969	11.49799	13.11422	15.78859	15.83582	12.04047
17294501	Fam81b	family with sequence similarity 81, member B	3.462658	3.566156	3.538284	2.832135	3.893587	3.489209
17311678	Fam83a	family with sequence similarity 83, member A	8.614279	8.150452	11.24818	7.451909	6.851786	5.880546
17528924	Fam83b	family with sequence similarity 83, member B	3.208035	2.550111	3.938019	3.45513	3.009972	2.617882
17393369	Fam83c	family with sequence similarity 83, member C	5.555551	5.934103	5.171382	5.545509	5.002364	4.738564
17378958	Fam83d	family with sequence similarity 83, member D	5.45145	5.579632	5.093439	6.833809	3.864522	5.021645
17478001	Fam83e	family with sequence similarity 83, member E	10.613	11.54983	10.34269	9.421652	10.10586	9.226414
17313158	Fam83f	family with sequence similarity 83, member F	4.915506	6.992896	4.995122	4.996369	5.837578	4.973736
17250624	Fam83g	family with sequence similarity 83, member G	8.622044	13.91945	7.273451	8.479505	11.94148	11.85234
17318249	Fam83h	family with sequence similarity 83, member H	24.4642	32.69436	34.55159	33.24833	39.02536	32.02774
17280041	Fam84a	family with sequence similarity 84, member A	13.26681	13.76268	11.56746	16.12894	12.054	12.28529
17317393	Fam84b	family with sequence similarity 84, member B	15.94089	23.60074	24.95038	20.49642	29.70821	17.51246
17514118	Fam89a	family with sequence similarity 89, member A	13.05472	15.87121	12.35189	16.61299	12.35189	12.35189
17361764	Fam89b	family with sequence similarity 89, member B	32.99922	46.71066	46.37804	46.0794	47.50278	45.93908
17499835	Fam90a1a	family with sequence similarity 90, member A1A	6.171709	7.47032	7.304016	6.180099	9.033468	9.140671
17543246	Fam90a1b	family with sequence similarity 90, member A1B	4.878238	4.908424	4.142868	4.22485	4.421819	4.142868
17423373	Fam92a	family with sequence similarity 92, member A	34.92262	45.73701	51.35775	49.4617	48.90161	38.83189
17513514	Fam92b	family with sequence similarity 92, member B	10.56155	9.974538	10.49565	11.65995	11.25668	9.368902
17518672	Fam96a	family with sequence similarity 96, member A	90.90892	82.70485	80.42154	76.44287	76.15833	69.86849
17512329	Fam96b	family with sequence similarity 96, member B	32.02907	31.97916	30.65881	32.22057	29.79338	31.0868
17347252	Fam98a	family with sequence similarity 98, member A	84.8075	58.85365	62.10597	67.65478	70.16502	60.82785
17374470	Fam98b	family with sequence similarity 98, member B	24.24844	16.98748	15.72175	19.2058	22.05402	18.49637
17488719	Fam98c	family with sequence similarity 98, member C	30.69454	32.39973	32.73178	31.03512	24.3392	35.89746
17416353	Fam151a	family with sequence simliarity 151, member A	6.652147	6.332697	7.1068	6.965708	7.662632	8.21215
17469788	Fancd2os	Fancd2 opposite strand	6.992896	6.592566	7.217604	6.575862	7.727973	6.992896
17491800	Fan1	FANCD2/FANCI-associated nuclease 1	14.25474	11.73162	10.63199	9.293165	13.69749	12.094
17273073	Faap100	Fanconi anemia core complex associated protein 100	12.19476	19.15302	18.32854	17.81549	13.65578	15.43764
17422510	Faap20	Fanconi anemia core complex associated protein 20	12.13237	16.4374	16.36612	14.83929	19.22418	17.71537
17489759	Faap24	Fanconi anemia core complex associated protein 24	12.0474	10.86653	10.49223	11.33176	13.1275	11.22202
17513908	Fanca	Fanconi anemia, complementation group A	5.721645	8.023435	6.179842	6.179842	6.179842	7.2396
17539586	Fancb	Fanconi anemia, complementation group B	2.611611	2.622713	2.639638	2.531548	3.13388	2.680923
17293573	Fancc	Fanconi anemia, complementation group C	4.648999	4.288491	4.20927	4.20927	3.301359	4.20927
17461739	Fancd2	Fanconi anemia, complementation group D2	4.720174	4.832936	4.832936	4.918403	4.093628	4.832936
17335312	Fance	Fanconi anemia, complementation group E	15.16696	13.5862	13.30097	14.26769	18.51638	12.51087
17491417	Fancf	Fanconi anemia, complementation group F	12.60339	16.71591	13.32922	13.95529	9.95782	12.91486
17424515	Fancg	Fanconi anemia, complementation group G	8.473629	8.205777	8.473629	9.187995	8.473629	9.267756
17479371	Fanci	Fanconi anemia, complementation group I	3.700269	3.642452	3.642452	3.630781	3.825664	3.19264
17247898	Fancl	Fanconi anemia, complementation group L	7.775632	6.959241	6.118042	7.269923	8.117525	6.581653
17275832	Fancm	Fanconi anemia, complementation group M	22.62789	13.59063	17.21984	22.26229	17.90164	20.82104
17403625	Fubp1	far upstream element (FUSE) binding protein 1	83.92915	81.62788	93.6132	89.39672	81.84033	81.9942
17369507	Fubp3	far upstream element (FUSE) binding protein 3	18.85898	23.39097	22.38065	23.21009	18.12539	17.44413
17307588	Fdft1	farnesyl diphosphate farnesyl transferase 1	139.6556	109.3508	148.0161	104.6487	157.9495	104.0188
17406908	Fdps	farnesyl diphosphate synthetase	87.41082	66.39718	90.53979	70.22778	97.91378	49.08363
17508471	Fnta	farnesyltransferase, CAAX box, alpha	46.98245	48.55742	48.17224	45.70753	47.87067	53.05585
17276622	Fntb	farnesyltransferase, CAAX box, beta	6.339548	5.976124	6.280546	5.976124	4.308961	6.88788
17358797	Fas	Fas (TNF receptor superfamily member 6)	87.86094	82.3051	74.2763	71.95235	71.22442	61.99844
17272198	Fbf1	Fas (TNFRSF6) binding factor 1	12.11741	13.51388	10.04245	10.71025	13.7791	12.12851
17498461	Fadd	Fas (TNFRSF6)-associated via death domain	15.30021	14.32566	17.00303	16.8253	23.91179	16.94632
17485421	Faddos	Fas (TNFRSF6)-associated via death domain, opposite strand [Source:MGI Symbol;/	5.592681	6.287864	5.566768	6.171709	6.07094	5.687324
17520679	Faim	Fas apoptotic inhibitory molecule	29.72717	19.17401	30.14883	26.36112	33.29445	30.64819
17321582	Faim2	Fas apoptotic inhibitory molecule 2	9.668419	10.63302	9.668419	9.379818	9.381379	10.52618
17530027	Faiml	Fas apoptotic inhibitory molecule like	2.597421	2.521391	2.326837	1.898013	2.195653	2.566371
17287471	Faf2	Fas associated factor family member 2	175.2949	156.3516	151.4943	145.0282	133.9304	151.4943
17336213	Daxx	Fas death domain-associated protein	14.37659	20.3203	18.99898	19.62866	18.52742	17.41985
17228864	Fasl	Fas ligand (TNF superfamily, member 6)	3.937528	4.21733	3.806698	4.045085	3.806698	3.791319
17446185	Fastk	Fas-activated serine/threonine kinase	48.78853	59.39172	63.59934	64.18748	58.10257	57.41716
17416897	Faf1	Fas-associated factor 1	51.37626	52.81039	52.99962	52.85874	48.77804	52.99962
17516010	Fez1	fasciculation and elongation protein zeta 1 (zygin I)	3.254802	4.251465	4.894868	4.526136	3.577223	3.753821
17347267	Fez2	fasciculation and elongation protein zeta 2 (zygin II)	40.3294	42.06126	43.5373	41.0944	38.83216	39.02265
17444267	Fscn1	fascin homolog 1, actin bundling protein (Strongylocentrotus purpuratus)	19.63016	18.84722	22.13504	21.39438	18.8433	18.62954
17259314	Fscn2	fascin homolog 2, actin-bundling protein, retinal (Strongylocentrotus purpuratus)	3.274648	4.284301	3.050488	3.703322	3.488169	2.997438
17456495	Fscn3	fascin homolog 3, actin-bundling protein, testicular (Strongylocentrotus purpuratu	5.571169	5.571169	5.564608	5.714669	5.715065	5.571169
17386297	Fastkd1	FAST kinase domains 1	43.02341	33.18064	32.88049	39.25515	33.3023	47.01242
17213645	Fastkd2	FAST kinase domains 2	22.08202	18.55737	18.35041	23.66626	22.27911	23.26872
17288370	Fastkd3	FAST kinase domains 3	10.28985	8.316981	10.48366	10.88206	10.50584	10.59998
17391670	Fastkd5	FAST kinase domains 5	18.49124	17.85628	16.03631	18.27931	18.62954	17.87573
17503784	Fto	fat mass and obesity associated	25.67781	26.18176	31.11008	26.70911	30.05785	26.805
17300544	Fitm1	fat storage-inducing transmembrane protein 1	164.8648	182.5123	121.9267	144.7109	138.5142	148.3849
17394036	Fitm2	fat storage-inducing transmembrane protein 2	85.62677	92.34104	103.5576	95.77253	73.42503	80.06779
17500832	Fat1	FAT tumor suppressor homolog 1 (Drosophila)	57.63567	51.71353	58.97453	52.09453	53.13166	53.72717
17262964	Fat2	FAT tumor suppressor homolog 2 (Drosophila)	4.759203	4.20927	3.987515	3.887466	4.813746	3.164132
17524233	Fat3	FAT tumor suppressor homolog 3 (Drosophila)	4.850287	4.136011	4.249196	4.715661	3.237881	4.524819
17397185	Fat4	FAT tumor suppressor homolog 4 (Drosophila)	8.920719	8.127885	11.26301	9.143332	6.510417	7.198169
17513092	Fa2h	fatty acid 2-hydroxylase	7.588475	7.74422	8.37843	7.73167	8.953924	7.683001
17428509	Faah	fatty acid amide hydrolase	85.06596	72.01323	86.01343	83.3032	83.90181	96.50622
17459455	Fabp1	fatty acid binding protein 1, liver	2411.843	2642.467	2290.854	2267.977	2405.698	2267.898

17404098	Fabp12	fatty acid binding protein 12	5.602109	5.129933	4.762041	4.990519	6.185199	5.216456
17402350	Fabp2	fatty acid binding protein 2, intestinal	392.0625	322.7605	305.3542	347.6954	335.3305	358.3735
17419215	Fabp3	fatty acid binding protein 3, muscle and heart	104.44	99.28405	107.8035	108.0856	89.87637	93.71707
17243761	Fabp3-ps1	fatty acid binding protein 3, muscle and heart, pseudogene 1 [Source:MGI Symbol	5.991304	23.45104	19.44005	9.137123	7.150727	4.797125
17404091	Fabp4	fatty acid binding protein 4, adipocyte	78.22783	105.3692	182.763	81.60355	141.6336	93.93299
17396056	Fabp5	fatty acid binding protein 5, epidermal	27.26012	25.97425	44.16097	23.29454	33.34087	20.61325
17548238	Fabp5	fatty acid binding protein 5, epidermal	12.11548	12.62884	17.26346	6.776592	13.33014	5.126236
17548717	Fabp5	fatty acid binding protein 5, epidermal	11.55151	11.24912	15.34025	6.624493	11.88401	4.147006
17261963	Fabp6	fatty acid binding protein 6, ileal (gastrotropin)	6.14141	5.361735	5.120306	5.099055	4.266757	4.620343
17233384	Fabp7	fatty acid binding protein 7, brain	33.72111	36.30383	42.7357	40.6561	24.42269	35.47572
17404084	Fabp9	fatty acid binding protein 9, testis	3.026877	3.060506	3.026877	2.932294	3.505135	2.66115
17357460	Fads1	fatty acid desaturase 1	676.6847	698.8795	927.3435	649.6505	769.2294	784.5274
17362595	Fads2	fatty acid desaturase 2	1197.791	1415.965	2034.389	1362.594	1197.749	1359.302
17357444	Fads3	fatty acid desaturase 3	22.53023	20.70863	22.77201	19.53081	19.15647	17.69046
17271842	Fads6	fatty acid desaturase domain family, member 6	34.12128	45.54719	48.32139	47.47579	42.34295	45.06108
17273348	Fasn	fatty acid synthase	296.0137	144.6046	194.4547	175.1649	238.5553	150.2947
17481982	Far1	fatty acyl CoA reductase 1	10.39365	10.83157	10.56002	9.361307	9.276882	9.361307
17495250	Far1os	fatty acyl CoA reductase 1, opposite strand	2.887798	3.149166	2.818622	2.501182	3.078057	2.917616
17464455	Far2	fatty acyl CoA reductase 2	3.887385	3.787485	4.211751	3.787485	5.328687	4.293874
17472924	Far2os1	fatty acyl CoA reductase 2, opposite strand 1 [Source:MGI Symbol;Acc:MGI:441500	4.729803	3.553129	4.50907	3.22565	3.494098	4.552503
17472919	Far2os2	fatty acyl CoA reductase 2, opposite strand 2 [Source:MGI Symbol;Acc:MGI:192509	6.057741	5.69478	6.992896	6.171709	6.360984	6.171709
17277387	Fos	FBJ osteosarcoma oncogene	10.89678	10.05701	9.431257	9.326333	9.145297	9.11511
17487211	Fosb	FBJ osteosarcoma oncogene B	9.940786	9.258125	9.009331	9.076525	8.980838	9.737085
17524426	Fbxl12	F-box and leucine-rich repeat protein 12	11.18893	11.13817	11.321	10.37443	13.66326	9.903373
17514998	Fbxl12os	F-box and leucine-rich repeat protein 12, opposite strand	4.71406	3.703322	3.703322	3.659686	3.594223	3.518596
17445833	Fbxl13	F-box and leucine-rich repeat protein 13	3.714145	4.522154	3.81293	3.754732	3.738786	3.754732
17462285	Fbxl14	F-box and leucine-rich repeat protein 14	26.85744	25.83491	30.43902	28.58071	29.36244	20.39198
17359994	Fbxl15	F-box and leucine-rich repeat protein 15	12.54934	14.43811	16.04943	16.87692	14.57749	16.07147
17334782	Fbxl16	F-box and leucine-rich repeat protein 16	5.549778	7.225162	7.070146	7.173166	6.344735	3.974077
17346647	Fbxl17	F-box and leucine-rich repeat protein 17	47.42875	51.58573	47.81926	51.17829	51.47964	38.91974
17454740	Fbxl18	F-box and leucine-rich repeat protein 18	14.56971	15.47256	15.00561	15.83593	12.51763	13.7107
17483424	Fbxl19	F-box and leucine-rich repeat protein 19	13.43412	14.78928	11.00632	14.37909	10.79738	15.17663
17531834	Fbxl2	F-box and leucine-rich repeat protein 2	4.252585	4.965538	5.089028	6.180528	5.42811	5.139258
17268729	Fbxl20	F-box and leucine-rich repeat protein 20	28.26589	27.92896	33.29445	30.84789	33.01249	35.97244
17287814	Fbxl21	F-box and leucine-rich repeat protein 21	6.612794	6.117363	8.241575	5.962552	6.17295	8.548315
17528350	Fbxl22	F-box and leucine-rich repeat protein 22	14.57819	23.40573	13.63724	17.09461	14.11581	21.17353
17309161	Fbxl3	F-box and leucine-rich repeat protein 3	47.51628	51.62543	46.89493	51.30936	59.48607	58.56107
17412213	Fbxl4	F-box and leucine-rich repeat protein 4	15.88397	16.01342	16.10269	16.23595	15.91395	16.23595
17447803	Fbxl5	F-box and leucine-rich repeat protein 5	43.48452	36.01959	34.02681	36.24776	29.29718	34.40866
17318536	Fbxl6	F-box and leucine-rich repeat protein 6	14.65712	17.5634	19.78973	17.89035	18.81889	17.33805
17316179	Fbxl7	F-box and leucine-rich repeat protein 7	11.76297	10.97196	12.33298	11.2481	8.818052	12.07281
17504682	Fbxl8	F-box and leucine-rich repeat protein 8	9.571666	8.617504	9.60716	10.2129	7.337761	10.89421
17250790	Fbxw10	F-box and WD-40 domain protein 10	5.338965	4.929495	5.338965	5.933527	4.468281	6.447054
17248331	Fbxw11	F-box and WD-40 domain protein 11	57.81733	53.16113	53.80208	53.16113	52.06962	52.04039
17531302	Fbxw13	F-box and WD-40 domain protein 13	4.118389	3.417118	2.899411	3.686623	4.771259	3.437167
17531327	Fbxw14	F-box and WD-40 domain protein 14	3.790741	3.175052	2.255364	2.980284	2.482322	3.339703
17531405	Fbxw15	F-box and WD-40 domain protein 15	3.20095	2.950153	2.90108	3.086753	3.069301	3.204191
17531370	Fbxw16	F-box and WD-40 domain protein 16	3.118106	2.822884	2.827976	2.804299	3.263862	3.703245
17287260	Fbxw17	F-box and WD-40 domain protein 17	5.701533	6.198203	6.479983	6.119951	6.742577	6.158931
17531442	Fbxw18	F-box and WD-40 domain protein 18	3.107426	2.541817	3.061269	2.304206	2.407173	2.844253
17531383	Fbxw19	F-box and WD-40 domain protein 19	2.673464	3.703322	3.436071	2.826154	2.477371	3.333136
17384301	Fbxw2	F-box and WD-40 domain protein 2	46.26855	44.12609	42.56395	44.18608	44.59531	46.82802
17531315	Fbxw20	F-box and WD-40 domain protein 20	3.406075	4.794798	4.394168	3.104756	3.3867	3.765627
17531288	Fbxw21	F-box and WD-40 domain protein 21	2.622895	2.02525	2.458673	2.458673	2.458673	2.033127
17531357	Fbxw22	F-box and WD-40 domain protein 22	4.289799	2.373441	2.187966	2.45015	2.753945	2.480378
17531395	Fbxw23	F-box and WD-40 domain protein 23	3.769335	3.527827	3.222231	3.703322	3.466741	2.921785
17531418	Fbxw24	F-box and WD-40 domain protein 24	2.468526	2.965386	2.492166	1.934942	2.787651	1.93584
17531431	Fbxw25	F-box and WD-40 domain protein 25	2.411733	2.288116	2.542821	2.288116	2.638815	2.70534
17531455	Fbxw26	F-box and WD-40 domain protein 26	3.011683	2.49379	3.145218	2.879863	2.658219	3.256269
17531468	Fbxw27	F-box and WD-40 domain protein 27	2.406006	2.415229	2.625842	3.118517	3.028325	3.346539
17531342	Fbxw28	F-box and WD-40 domain protein 28	2.350259	1.837698	2.255442	1.688849	4.27979	2.193584
17365211	Fbxw4	F-box and WD-40 domain protein 4	27.40809	23.88859	26.04311	33.26654	28.84161	28.93172
17368079	Fbxw5	F-box and WD-40 domain protein 5	112.7138	112.061	127.0885	103.1614	87.33875	83.83728
17398581	Fbxw7	F-box and WD-40 domain protein 7	17.57801	17.41779	17.53651	18.48381	17.4687	14.43241
17451886	Fbxw8	F-box and WD-40 domain protein 8	59.45969	53.6825	57.39885	53.77859	47.49389	49.75329
17503362	Fbxw9	F-box and WD-40 domain protein 9	22.77044	22.15346	18.37586	22.17958	22.84062	22.3142
17424913	Fbxo10	F-box protein 10	6.294798	6.40292	6.860435	9.608891	7.262771	7.103697
17347953	Fbxo11	F-box protein 11	82.44214	83.46156	72.33791	83.16416	84.08287	92.0037
17352241	Fbxo15	F-box protein 15	2.890221	2.998249	3.409287	3.395914	3.107426	3.044826
17301365	Fbxo16	F-box protein 16	3.915808	4.464411	3.777103	4.040685	4.405605	3.787905
17475886	Fbxo17	F-box protein 17	6.270846	6.659713	6.722044	7.554467	6.193865	7.068137
17381683	Fbxo18	F-box protein 18	37.93191	33.69494	36.90642	38.17245	41.12033	36.99914
17421588	Fbxo2	F-box protein 2	6.682093	7.605589	6.631384	8.530557	7.425406	7.019508
17441490	Fbxo21	F-box protein 21	149.0311	190.4528	145.0051	176.7063	145.0282	203.8946
17517500	Fbxo22	F-box protein 22	60.54433	57.28716	54.37306	55.02785	59.24781	44.79668
17454099	Fbxo24	F-box protein 24	4.573441	3.963157	3.500255	3.167973	2.976981	3.549117
17499396	Fbxo25	F-box protein 25	26.62519	23.89588	25.17727	23.11825	23.56118	23.48178
17475874	Fbxo27	F-box protein 27	7.724706	6.992896	7.031634	6.861433	6.929878	7.129545
17230602	Fbxo28	F-box protein 28	33.03263	30.66498	29.70348	29.70348	30.66328	26.09986
17373794	Fbxo3	F-box protein 3	197.273	209.4178	194.7771	210.5399	178.4208	194.7771
17231662	Fbxo30	F-box protein 30	27.49448	39.08004	27.49448	29.22781	27.49448	27.49448
17513590	Fbxo31	F-box protein 31	61.88981	62.36566	74.76079	77.59383	77.90262	77.69124
17317266	Fbxo32	F-box protein 32	12.32572	10.06629	13.29618	15.7794	13.29618	15.43764
17281388	Fbxo33	F-box protein 33	27.75951	16.14986	16.13946	19.33255	14.31296	17.02508

17299341	Fbxo34	F-box protein 34	28.74063	33.09199	33.12411	32.29435	32.29435	32.29435
17214883	Fbxo36	F-box protein 36	29.12366	29.96113	29.57672	30.14131	33.01913	25.18268
17354861	Fbxo38	F-box protein 38	72.21767	57.67203	54.69814	57.45976	60.3056	55.61607
17252351	Fbxo39	F-box protein 39	3.84723	3.203769	3.923306	3.684452	3.729055	3.729055
17315658	Fbxo4	F-box protein 4	20.48946	20.31706	22.97511	21.29481	23.09694	16.8077
17330264	Fbxo40	F-box protein 40	9.812699	8.286311	7.791656	10.41665	9.123011	8.256384
17468387	Fbxo41	F-box protein 41	7.582744	7.066227	6.26624	6.992896	7.042414	6.347815
17420984	Fbxo42	F-box protein 42	34.08961	34.08961	37.05766	33.43182	36.53608	35.69401
17316472	Fbxo43	F-box protein 43	4.364844	5.198192	5.470035	5.129577	4.352065	5.122649
17432927	Fbxo44	F-box protein 44	11.24366	12.43582	12.97264	12.43582	11.38261	12.43582
17329833	Fbxo45	F-box protein 45	21.49352	25.37243	23.15802	26.97891	20.92245	23.62529
17474485	Fbxo46	F-box protein 46	12.18023	12.69906	12.69396	17.94488	18.48791	15.2999
17268647	Fbxo47	F-box protein 47	4.12948	4.594284	4.627073	4.993911	3.919012	4.801449
17247428	Fbxo48	F-box protein 48	5.276642	4.018592	3.767271	3.957941	3.796132	3.878288
17238974	Fbxo5	F-box protein 5	7.776926	9.182646	9.791026	8.850629	7.685345	12.28529
17432912	Fbxo6	F-box protein 6	66.94665	62.79379	70.9962	76.31263	76.81893	72.43575
17236155	Fbxo7	F-box protein 7	28.68968	22.50681	16.34526	23.44503	22.48966	22.61675
17501260	Fbxo8	F-box protein 8	190.1785	201.1503	157.6947	194.4991	181.2139	181.0244
17528983	Fbxo9	f-box protein 9	66.24961	66.94572	65.86591	68.95915	68.53457	62.58175
17475734	Fcgbp	Fc fragment of IgG binding protein	6.288038	8.458313	7.965804	7.759319	9.355988	7.240002
17216976	Fcmr	Fc fragment of IgM receptor	5.881279	5.667255	6.075992	7.207655	4.6943	5.532187
17216949	Fcamr	Fc receptor, IgA, IgM, high affinity	5.248008	4.459834	4.316045	7.183017	5.818188	5.373716
17229974	Fcer1a	Fc receptor, IgE, high affinity I, alpha polypeptide	3.320315	2.738508	2.443485	2.3715	2.236651	1.939776
17229658	Fcer1g	Fc receptor, IgE, high affinity I, gamma polypeptide	64.30326	95.47162	106.7417	68.07013	69.40078	62.87306
17507131	Fcer2a	Fc receptor, IgE, low affinity II, alpha polypeptide	4.721974	4.064647	4.783578	4.506165	4.716969	5.573525
17490589	Fcgrt	Fc receptor, IgG, alpha chain transporter	493.067	515.0826	480.7223	418.6324	429.6059	402.7302
17408024	Fcgr1	Fc receptor, IgG, high affinity I	40.99	38.64233	50.97861	33.88254	41.68019	32.8404
17229607	Fcgr2b	Fc receptor, IgG, low affinity IIb	148.8391	141.1094	183.6481	129.9552	104.0974	90.9417
17229620	Fcgr3	Fc receptor, IgG, low affinity III	14.1029	25.98127	27.34964	20.58184	21.84937	24.307
17219199	Fcgr4	Fc receptor, IgG, low affinity IV	18.45743	18.8099	21.33707	18.36797	16.53682	15.48082
17398742	Fcrl1	Fc receptor-like 1	4.518707	5.367722	4.57062	4.742442	5.023281	4.173073
17398755	Fcrl5	Fc receptor-like 5	4.553197	5.914393	5.642052	5.463782	5.918904	6.860387
17229939	Fcrl6	Fc receptor-like 6	4.097943	4.678156	4.365691	4.20927	4.113111	4.53006
17229596	Fcrla	Fc receptor-like A	5.1425	5.117113	4.050332	4.066281	6.300341	3.83836
17229590	Fcrlb	Fc receptor-like B	4.801117	5.05398	4.553008	5.004618	5.772075	5.303188
17406514	Fcrls	Fc receptor-like S, scavenger receptor	5.36285	5.052509	4.613814	5.36285	7.406439	5.563528
17277307	Fcf1	FCF1 small subunit (SSU) processome component homolog (S. cerevisiae)	27.31213	29.32359	23.46356	28.38369	28.05994	30.44978
17353857	Fchsd1	FCH and double SH3 domains 1	9.79829	9.79829	9.79829	11.74977	11.14883	10.05471
17480788	Fchsd2	FCH and double SH3 domains 2	48.2956	43.72511	51.86035	50.1612	32.53995	46.75861
17510428	Fcho1	FCH domain only 1	6.201039	7.118583	7.076225	7.033438	7.118583	6.841298
17295394	Fcho2	FCH domain only 2	60.62496	66.734	66.65725	64.90735	60.29849	66.00256
17492575	Fes	feline sarcoma oncogene	12.05634	14.75539	14.62962	13.51388	16.23066	16.48589
17354263	Fem1c	fem-1 homolog c (C.elegans)	45.50332	42.4561	39.58577	48.64366	45.70595	58.35846
17338743	Fem1a	feminization 1 homolog a (C. elegans)	24.21115	14.31653	17.13626	22.14317	18.70329	13.66459
17528014	Fem1b	feminization 1 homolog b (C. elegans)	41.06849	38.04988	34.74997	34.74997	34.74997	42.9793
17339081	Fer	fer (fms/fps related) protein kinase	25.58808	32.67375	30.06098	32.87799	28.57972	28.96121
17393407	Fer1l4	fer-1-like 4 (C. elegans)	5.649997	5.780603	5.780603	6.513577	5.691425	5.780603
17211809	Fer1l5	fer-1-like 5 (C. elegans)	5.894502	5.300432	5.259297	5.866947	5.518553	4.107384
17311733	Fer1l6	fer-1-like 6 (C. elegans)	3.604477	3.263862	3.263862	3.167929	3.263862	3.502366
17274955	Ferd3l	Fer3-like (Drosophila)	3.417497	3.54506	3.46333	3.517718	3.322939	3.258843
17413615	Frmpd1	FERM and PDZ domain containing 1	5.570551	6.171709	6.325108	7.332626	5.756133	6.171709
17424933	Frmpd1os	FERM and PDZ domain containing 1, opposite strand	4.762668	6.046164	3.451157	3.554188	4.040881	3.651148
17538152	Frmpd3	FERM and PDZ domain containing 3	3.680674	4.349531	4.334784	4.226988	3.376737	4.854827
17546133	Frmpd4	FERM and PDZ domain containing 4	3.594298	3.550962	4.046094	3.473813	3.825133	3.910139
17414913	Frmd3	FERM domain containing 3	2.841751	3.129127	2.902005	3.303694	3.130927	3.147486
17366451	Frmd4a	FERM domain containing 4A	18.58053	19.87179	26.15981	19.4185	19.24711	20.71279
17469365	Frmd4b	FERM domain containing 4B	53.48862	48.0292	68.43678	53.17403	65.92437	61.72059
17390637	Frmd5	FERM domain containing 5	27.56546	21.82198	20.17784	38.68118	11.23135	15.75939
17276020	Frmd6	FERM domain containing 6	15.11239	21.09487	19.03615	18.25551	16.26839	16.99914
17541597	Frmd7	FERM domain containing 7	4.653512	3.808651	4.045281	3.731382	3.94422	3.489112
17361814	Frmd8	FERM domain containing 8	33.49445	36.29729	33.49445	34.4595	29.69751	30.49054
17356559	Frmd8os	FERM domain containing 8, opposite strand	7.225313	8.00971	9.225135	8.320787	7.828902	9.702591
17302802	Farp1	FERM, RhoGEF (Arhgef) and pleckstrin domain protein 1 (chondrocyte-derived)	50.62682	34.8948	41.10494	40.41615	41.19108	28.26785
17216031	Farp2	FERM, RhoGEF and pleckstrin domain protein 2	51.82765	39.83929	41.26624	45.34244	37.94427	37.32163
17392056	Fermt1	fermitin family homolog 1 (Drosophila)	5.751467	5.518515	6.499866	5.490928	4.407682	5.490928
17305662	Fermt2	fermitin family homolog 2 (Drosophila)	168.938	102.4829	97.64539	106.6219	102.4829	126.4734
17362223	Fermt3	fermitin family homolog 3 (Drosophila)	10.96178	20.54877	21.20173	11.11303	15.54167	16.65311
17527004	Fdx1	ferredoxin 1	56.26438	55.24914	50.99133	67.58494	58.56432	68.18961
17524597	Fdx1l	ferredoxin 1-like	44.3444	43.4574	42.55657	40.10082	43.5373	38.46195
17271823	Fdxr	ferredoxin reductase	24.39629	24.66443	23.05248	26.72707	26.6407	25.80216
17517142	Fdxacb1	ferredoxin-fold anticodon binding domain containing 1	10.15938	9.882733	8.85959	9.624089	11.11904	9.442376
17402072	Frrs1	ferric-chelate reductase 1	92.26042	90.22779	93.13877	82.03286	76.29148	66.25053
17425525	Frrs1l	ferric-chelate reductase 1 like	3.136814	3.879633	3.289709	4.379238	4.942291	3.729055
17357418	Fth1	ferritin heavy polypeptide 1	3223.473	3352.427	3448.588	3147.346	3015.351	3168.886
17490830	Ftl1	ferritin light polypeptide 1	33.82786	47.05317	34.58897	31.62492	17.56997	22.66886
17350559	Ftmt	ferritin mitochondrial	7.340356	10.20539	9.369876	8.342733	8.342733	9.416102
17536165	Fthl17a	ferritin, heavy polypeptide-like 17, member A	5.742146	8.448118	11.80258	9.269426	9.603897	8.789554
17540136	Fthl17b	ferritin, heavy polypeptide-like 17, member B	7.901847	7.698994	6.358516	6.251403	7.254167	7.698994
17540140	Fthl17c	ferritin, heavy polypeptide-like 17, member C	4.505696	5.924527	5.697622	4.340978	4.918574	5.206847
17540144	Fthl17d	ferritin, heavy polypeptide-like 17, member D	4.227105	5.760604	5.215661	4.635388	6.322346	4.20927
17533187	Fthl17e	ferritin, heavy polypeptide-like 17, member E	3.626254	4.20927	3.735419	3.347467	6.279632	4.42826
17533191	Fthl17f	ferritin, heavy polypeptide-like 17, member F	17.98223	19.82364	20.35921	16.50441	13.96555	20.43811
17354995	Fech	ferrochelatase	234.0625	220.432	205.209	191.1154	172.6552	201.9298
17535395	Fate1	fetal and adult testis expressed 1	3.508635	3.770981	4.042674	4.34914	4.739648	3.664992

17324332	Fetub	fetuin beta	785.5558	886.5573	884.869	782.7946	1224.477	790.8882
17224379	Fev	FEV (ETS oncogene family)	5.902393	6.71734	7.149042	6.233878	5.951074	6.622106
17465108	Fezf1	Fez family zinc finger 1	4.600912	4.69082	5.258459	4.200555	5.510908	4.801649
17303496	Fezf2	Fez family zinc finger 2	3.062691	3.935754	2.862469	3.107146	3.958544	2.930466
17333050	Fgfr1op	Fgfr1 oncogene partner	12.48176	13.24137	11.9218	12.79342	12.60295	10.36645
17464291	Fgfr1op2	FGFR1 oncogene partner 2	64.29078	64.68234	62.87044	64.00177	63.19507	66.57506
17328411	Fopnl	Fgfr1op N-terminal like	47.6981	46.80173	37.89433	47.88859	46.60943	46.8946
17415544	Fggy	FGGY carbohydrate kinase domain containing	133.2221	136.5608	141.5541	146.61	126.7744	143.799
17406363	Fhdc1	FH2 domain containing 1	5.338965	6.467015	6.903414	5.244117	4.985333	5.665134
17475777	Fbl	fibrillarin	65.61847	55.3937	71.11638	66.82102	71.38703	57.47609
17261726	Fbll1	fibrillarin-like 1	8.834815	8.542924	6.852071	8.84652	8.362069	8.834815
17390879	Fbn1	fibrillin 1	11.69768	14.71066	13.67131	11.69768	10.23763	11.31394
17354513	Fbn2	fibrillin 2	4.133432	4.133432	4.295095	4.715138	4.779402	3.980749
17398499	Fga	fibrinogen alpha chain	1960.37	1667.141	1668.366	1562.976	2065.507	1548.998
17406252	Fgb	fibrinogen beta chain	3689.757	3458.738	3288.423	3044.585	3963.117	3136.761
17383798	Fibcd1	fibrinogen C domain containing 1	10.29855	13.54905	10.98589	12.62289	6.747766	10.29855
17398485	Fgg	fibrinogen gamma chain	3547.409	3220.971	3023.974	2835.878	3688.632	2852.715
17508938	Fgl1	fibrinogen-like protein 1	1188.1	1558.973	1659.025	1016.215	1800.772	1049.925
17435089	Fgl2	fibrinogen-like protein 2	26.05467	27.16166	31.2976	29.27262	26.80555	25.84476
17385767	Fap	fibroblast activation protein	5.142287	5.020392	5.271633	6.993671	5.378038	5.503312
17356434	Fibp	fibroblast growth factor (acidic) intracellular binding protein	25.98343	34.25565	32.86591	29.89723	37.65423	35.86811
17353962	Fgf1	fibroblast growth factor 1	161.6893	163.6183	243.0029	187.325	181.9464	175.6561
17290111	Fgf10	fibroblast growth factor 10	3.662452	3.349138	3.215203	3.144368	3.189853	2.676041
17264960	Fgf11	fibroblast growth factor 11	7.76061	7.795059	7.770514	7.698994	7.35115	9.41545
17329516	Fgf12	fibroblast growth factor 12	7.182519	7.879368	6.0805	7.849659	6.030928	8.512483
17541926	Fgf13	fibroblast growth factor 13	5.761083	5.48933	5.48933	5.201256	5.172135	5.56106
17309774	Fgf14	fibroblast growth factor 14	5.531305	6.044823	4.829353	6.460295	6.491447	6.444373
17485443	Fgf15	fibroblast growth factor 15	7.698994	8.018876	9.788651	10.08186	5.899407	7.591421
17537038	Fgf16	fibroblast growth factor 16	7.023548	6.684177	6.992896	8.533633	5.754298	7.561487
17308435	Fgf17	fibroblast growth factor 17	19.31795	17.47826	15.26981	15.73887	15.50434	15.05541
17261542	Fgf18	fibroblast growth factor 18	13.80769	14.98918	14.34723	16.8197	14.31296	20.89607
17397129	Fgf2	fibroblast growth factor 2	5.423071	4.80105	4.699411	4.057469	5.177946	5.251392
17404906	Fgf2os	fibroblast growth factor 2, opposite strand	2.742915	2.545131	2.742915	3.05508	2.777025	2.921501
17508861	Fgf20	fibroblast growth factor 20	6.436873	7.341017	7.321605	7.631835	5.918535	8.012875
17490899	Fgf21	fibroblast growth factor 21	66.56353	79.5511	110.4462	73.83229	29.41744	29.05592
17234948	Fgf22	fibroblast growth factor 22	37.69444	36.79531	39.75323	37.69444	35.63567	43.76331
17463338	Fgf23	fibroblast growth factor 23	4.709193	6.845472	41.80258	5.810612	6.044906	3.821503
17485430	Fgf3	fibroblast growth factor 3	3.666262	3.929294	5.106094	3.883372	4.212072	4.990969
17485437	Fgf4	fibroblast growth factor 4	6.855206	8.472983	7.719942	8.701777	7.570717	7.894621
17439517	Fgf5	fibroblast growth factor 5	4.58652	4.668471	4.61196	4.573821	5.158101	4.865304
17463332	Fgf6	fibroblast growth factor 6	9.892259	8.366128	9.172214	8.943441	12.48168	7.782049
17375685	Fgf7	fibroblast growth factor 7	4.765706	4.550736	4.779998	5.131107	4.511446	4.329949
17365233	Fgf8	fibroblast growth factor 8	9.123011	8.253294	9.123011	9.123011	10.13799	9.8752
17300950	Fgf9	fibroblast growth factor 9	4.293339	4.447948	4.574233	5.295768	4.062027	4.824736
17447831	Fgfbp1	fibroblast growth factor binding protein 1	2.96987	2.811363	3.498169	3.208235	4.069129	3.260244
17364213	Fgfbp3	fibroblast growth factor binding protein 3	13.57293	19.08265	19.32491	16.92589	13.20736	21.12882
17500108	Fgfr1	fibroblast growth factor receptor 1	17.20576	22.46193	22.45804	22.72755	25.52484	10.48911
17496947	Fgfr2	fibroblast growth factor receptor 2	67.34458	55.62803	49.23492	53.99336	56.7654	53.44785
17436481	Fgfr3	fibroblast growth factor receptor 3	16.41667	17.68188	15.14374	14.11268	16.421	10.53896
17287590	Fgfr4	fibroblast growth factor receptor 4	45.88466	34.2699	31.49607	33.30623	42.06738	30.79107
17245188	Frs2	fibroblast growth factor receptor substrate 2	57.16103	51.47964	55.39216	60.01329	44.33672	56.61294
17338305	Frs3	fibroblast growth factor receptor substrate 3	6.69967	7.740678	9.235884	7.605431	6.198676	9.49396
17345722	Frs3os	fibroblast growth factor receptor substrate 3, opposite strand	9.673514	5.89393	10.01659	7.718658	7.65934	11.79154
17440312	Fgfrl1	fibroblast growth factor receptor-like 1	36.87932	31.22934	31.49607	32.8773	31.54589	28.87562
17217373	Fmod	fibromodulin	10.68726	12.08462	10.24019	10.24019	11.56746	9.188631
17224071	Fn1	fibronectin 1	1812.029	1943.485	2039.189	1569.327	2077.237	1605.214
17362256	Flrt1	fibronectin leucine rich transmembrane protein 1	10.52764	11.50086	14.36394	10.05046	9.938444	9.04738
17277788	Flrt2	fibronectin leucine rich transmembrane protein 2	5.498812	5.768835	3.830944	5.30396	3.732701	6.685104
17392255	Flrt3	fibronectin leucine rich transmembrane protein 3	2.67402	2.67402	2.67402	2.67402	2.732554	2.512459
17484151	Fank1	fibronectin type 3 and ankyrin repeat domains 1	3.679654	3.310915	3.863183	4.092578	4.127562	3.915645
17338670	Fsd1	fibronectin type 3 and SPRY domain-containing protein	4.112883	5.056783	5.015383	4.908833	5.056363	4.233673
17414212	Fsd1l	fibronectin type III and SPRY domain containing 1-like	3.381516	2.735643	2.980615	2.545907	3.819543	3.678073
17492810	Fsd2	fibronectin type III and SPRY domain containing 2	3.980639	5.386134	5.386134	5.386134	6.6352	5.890867
17340673	Fndc1	fibronectin type III domain containing 1	10.42677	8.15226	6.779975	7.551221	5.624676	8.878402
17308525	Fndc3a	fibronectin type III domain containing 3A	213.2615	158.7508	164.1476	145.0624	167.3889	158.4946
17404432	Fndc3b	fibronectin type III domain containing 3B	97.26777	80.63811	105.0243	71.88406	100.1897	46.79784
17544025	Fndc3c1	fibronectin type III domain containing 3C1	3.134032	2.985722	2.940517	2.394526	3.471213	3.437858
17544002	Fnd3c2	fibronectin type III domain containing 3C2	2.596305	3.523819	3.260312	3.603528	4.351944	3.245318
17446981	Fndc4	fibronectin type III domain containing 4	46.08387	34.28344	40.00698	53.25703	44.58603	46.54002
17418945	Fndc5	fibronectin type III domain containing 5	15.16854	14.00257	16.83206	14.5022	12.35035	15.91439
17409428	Fndc7	fibronectin type III domain containing 7	3.729055	3.870929	3.729055	3.729055	4.482551	3.697474
17254121	Fndc8	fibronectin type III domain containing 8	3.28841	4.061098	3.6769	2.943372	4.637766	3.683073
17248786	Fndc9	fibronectin type III domain containing 9	4.081105	5.361104	5.623039	5.303887	5.712372	5.806585
17483325	Fbrs	fibrosin	39.68797	58.91855	63.62404	47.14654	46.00153	46.81795
17451020	Fbrsl1	fibrosin-like 1	35.90319	41.64121	42.50203	36.93457	37.49432	42.43227
17281408	Fscb	fibrous sheath CABYR binding protein	2.313729	2.030943	2.390065	2.107321	2.591648	2.446315
17389689	Fsip1	fibrous sheath-interacting protein 1	8.215111	6.316258	5.365081	6.043608	6.334585	6.043608
17372470	Fsip2	fibrous sheath-interacting protein 2	2.568026	2.67402	2.659693	2.533339	2.845594	2.583094
17313811	Fbln1	fibulin 1	11.92072	9.975437	11.71398	8.482444	9.532536	8.880187
17460891	Fbln2	fibulin 2	13.10931	15.92631	17.27232	14.67847	16.95102	16.28001
17283380	Fbln5	fibulin 5	18.65784	24.10132	22.26028	20.2162	22.68206	23.4979
17376051	Fbln7	fibulin 7	21.04536	16.12402	20.32721	18.09827	16.47047	19.3477
17440748	Ficd	FIC domain containing	21.42465	11.707	10.22643	11.52896	12.55282	11.90322
17382737	Fcna	ficolin A	92.37817	108.3481	137.7606	93.74436	70.95242	70.46476

17383353	Fcnb	ficolin B	3.536298	4.294976	5.076485	4.138936	3.610904	3.774172
17385840	Fign	fidgetin	5.212047	6.318535	7.322519	4.522906	4.453501	6.602855
17260633	Fignl1	fidgetin-like 1	3.009972	2.85049	3.190008	2.619806	2.966805	3.206679
17321811	Fignl2	fidgetin-like 2	13.00685	11.86278	12.75932	17.09947	14.81062	14.37958
17240405	Fig4	FIG4 homolog (S. cerevisiae)	35.69599	32.78786	28.48005	36.30836	25.65948	26.67987
17399952	Flg	filaggrin	7.558553	7.80398	5.515111	6.924021	5.433606	6.776921
17399940	Flg2	filaggrin family member 2	3.234202	3.811714	3.234202	3.263862	2.834609	2.46048
17529185	Filip1	filamin A interacting protein 1	5.051704	5.696754	5.19387	4.172697	4.825104	4.574582
17326394	Filip1l	filamin A interacting protein 1-like	7.271031	9.527251	7.852217	7.311665	7.985429	7.219355
17432227	Fblim1	filamin binding LIM protein 1	9.034909	11.0551	14.16786	13.53929	9.203164	10.94015
17456627	Flnc	filamin C, gamma	7.076274	8.866963	10.11427	8.833713	8.109989	9.703263
17542588	Flna	filamin, alpha	28.45223	29.62986	30.64862	26.38343	27.5248	27.2084
17297010	Flnb	filamin, beta	120.3665	118.6403	142.0318	107.8244	98.71039	105.4474
17389157	Fibin	fin bud initiation factor homolog (zebrafish)	4.635227	4.271285	4.597501	4.615926	4.380362	4.53864
17331573	Fau	Finkel-Biskis-Reilly murine sarcoma virus (FBR-MuSV) ubiquitously expressed (fox c	283.9317	303.4027	297.4782	291.4471	319.9605	323.3942
17356604	Fau	Finkel-Biskis-Reilly murine sarcoma virus (FBR-MuSV) ubiquitously expressed (fox c	11.38743	14.03921	15.08246	14.15952	18.36567	16.48955
17438196	Fip1l1	FIP1 like 1 (S. cerevisiae)	93.42392	80.55264	82.01751	86.94614	81.36125	82.01751
17443474	Fis1	fission 1 (mitochondrial outer membrane) homolog (yeast)	25.94042	24.00528	23.8279	27.12535	26.07292	26.68228
17243312	Fzr1	fizzy/cell division cycle 20 related 1 (Drosophila)	39.76949	35.12874	35.12874	30.40444	35.12874	44.0088
17256365	Fkbp10	FK506 binding protein 10	16.18516	19.58775	19.3955	19.08226	17.01435	21.19233
17321318	Fkbp11	FK506 binding protein 11	68.88796	47.12073	49.02515	43.20271	36.61721	29.33559
17466996	Fkbp14	FK506 binding protein 14	12.29543	14.22217	12.95673	12.2127	10.28614	11.07788
17426126	Fkbp15	FK506 binding protein 15	30.61804	38.71525	37.061	36.60731	41.60947	33.73888
17377597	Fkbp1a	FK506 binding protein 1a	40.92584	49.02073	44.74765	40.77152	37.83711	37.27561
17279858	Fkbp1b	FK506 binding protein 1b	3.757883	3.360346	3.323423	3.703656	3.009429	3.623792
17362190	Fkbp2	FK506 binding protein 2	266.448	201.7339	186.8919	204.1096	233.9911	212.7256
17281419	Fkbp3	FK506 binding protein 3	55.73957	59.21497	54.10762	61.76082	58.93203	65.30086
17471342	Fkbp4	FK506 binding protein 4	214.9176	175.795	160.8631	182.1067	145.6993	193.5644
17342868	Fkbp5	FK506 binding protein 5	19.42294	40.23642	41.56162	40.23642	52.79209	37.16287
17453545	Fkbp6	FK506 binding protein 6	6.933386	6.957746	6.928437	6.894472	6.665024	8.151864
17386770	Fkbp7	FK506 binding protein 7	9.01639	14.63357	10.97607	10.15628	10.08438	13.51191
17501950	Fkbp8	FK506 binding protein 8	188.7339	185.6344	222.5136	185.83	186.8478	173.838
17458911	Fkbp9	FK506 binding protein 9	59.4119	70.95931	74.68466	65.52756	53.82744	58.46171
17336655	Fkbpl	FK506 binding protein-like	7.754104	9.40723	10.1025	9.73776	10.81349	10.81349
17362609	Fen1	flap structure specific endonuclease 1	8.850322	7.238898	4.845918	5.06422	6.992896	6.597777
17229020	Fmo1	flavin containing monooxygenase 1	327.5497	374.3282	372.5138	403.6077	316.1335	312.8159
17229036	Fmo2	flavin containing monooxygenase 2	30.84447	52.17801	75.98486	58.84549	90.06096	49.15138
17229059	Fmo3	flavin containing monooxygenase 3	9.186912	16.41542	31.17246	16.96077	53.12798	18.7243
17229007	Fmo4	flavin containing monooxygenase 4	14.06122	16.8857	18.12338	19.91633	19.12529	16.25712
17400773	Fmo5	flavin containing monooxygenase 5	749.1236	763.1855	719.3051	796.6212	817.945	736.5298
17229050	Fmo6	flavin containing monooxygenase 6	3.428553	3.113376	3.762209	4.277862	3.391116	4.385435
17229370	Fmo9	flavin containing monooxygenase 9	4.446222	5.331901	4.524536	4.789915	4.257569	4.445113
17263609	Flii	flightless I homolog (Drosophila)	94.16311	101.2852	103.9741	101.5411	96.28906	98.91655
17328222	LOC105246072	flocculation protein FLO10-like	4.421022	3.328472	3.420412	3.361768	3.757648	3.218749
17337232	Flot1	flotillin 1	41.87972	44.71912	50.779	42.61384	37.51537	32.38895
17253431	Flot2	flotillin 2	54.71635	56.24138	59.9343	49.69986	48.15154	53.45711
17485985	Fiz1	Flt3 interacting zinc finger protein 1	41.94044	39.41164	38.0193	38.84993	44.6742	47.50014
17341619	Flywch2	FLYWCH family member 2	6.19232	4.845952	5.29933	5.010936	5.220435	4.312158
17341608	Flywch1	FLYWCH-type zinc finger 1	33.8084	30.67306	30.27028	29.13275	27.19614	33.14203
17455346	Flt1	FMS-like tyrosine kinase 1	26.46585	28.28372	48.1502	26.79181	26.22244	15.96311
17455319	Flt3	FMS-like tyrosine kinase 3	5.558787	7.698994	5.701889	5.558787	4.578072	5.558787
17490628	Flt3l	FMS-like tyrosine kinase 3 ligand	15.79176	24.03592	28.54607	21.18748	17.29664	17.90201
17248992	Flt4	FMS-like tyrosine kinase 4	65.43769	66.49436	74.96992	60.84134	45.99643	45.85191
17415239	Focad	focadhesin	15.91891	14.31296	14.31296	15.90832	14.24131	14.13275
17493145	Folh1	folate hydrolase 1	3.912525	3.471069	3.653401	2.973393	3.145937	3.397868
17493956	Folr1	folate receptor 1 (adult)	4.61065	5.481725	4.699411	4.886969	5.999491	5.212155
17493949	Folr2	folate receptor 2 (fetal)	85.19696	95.96125	100.8201	82.6888	74.39532	69.006
17389105	Fshb	follicle stimulating hormone beta	4.20927	4.360459	4.274839	4.941161	3.729055	4.332891
17348003	Fshr	follicle stimulating hormone receptor	3.128476	3.883991	3.907295	3.380859	3.380859	3.061948
17263463	Flcn	folliculin	33.82786	52.53912	43.24226	40.29447	35.89448	45.88053
17249702	Fnip1	folliculin interacting protein 1	57.89633	68.40595	69.93923	59.26712	53.38306	68.40595
17406066	Fnip2	folliculin interacting protein 2	33.92155	48.43809	52.96106	43.45349	61.69835	46.46524
17460360	Figla	folliculogenesis specific basic helix-loop-helix	5.291255	5.655286	4.915847	5.412631	4.931273	5.074586
17296344	Fst	follistatin	67.55029	53.33202	45.01956	44.09796	41.07789	33.84827
17325514	Fstl1	follistatin-like 1	18.49649	20.22854	24.18013	17.36523	14.74189	18.49649
17234955	Fstl3	follistatin-like 3	17.19062	19.36259	20.90056	21.17206	17.46555	19.00003
17249461	Fstl4	follistatin-like 4	7.742771	6.398838	7.097053	6.834094	6.992896	6.088345
17398338	Fstl5	follistatin-like 5	3.084849	2.88954	2.874239	3.058618	2.921137	2.950133
17383934	Fpgs	folylpolyglutamyl synthetase	114.89	93.0994	104.3322	110.6968	111.6593	85.39324
17281337	Foxa1	forkhead box A1	56.79256	42.56778	49.4617	42.76771	35.88502	41.02723
17392561	Foxa2	forkhead box A2	61.67398	56.70956	70.26528	56.47028	62.32331	61.7848
17487149	Foxa3	forkhead box A3	70.53513	73.95932	60.95151	79.99955	110.3781	87.28065
17528557	Foxb1	forkhead box B1	6.44089	5.915213	5.914885	6.144859	5.101283	7.311108
17363316	Foxb2	forkhead box B2	6.413225	10.54816	10.46137	8.225139	7.749859	11.23727
17286340	Foxc1	forkhead box C1	4.826542	5.068082	6.663869	5.644164	5.04135	5.345556
17506296	Foxc2	forkhead box C2	6.894567	6.811346	5.619026	6.563567	7.936759	6.252833
17289422	Foxd1	forkhead box D1	5.030947	5.387702	4.392493	4.366508	4.315716	5.083316
17428424	Foxd2	forkhead box D2	5.802481	5.870446	5.736179	6.489062	6.740567	6.783736
17417107	Foxd2os	forkhead box D2, opposite strand	3.703322	3.646317	3.703322	3.641064	3.895558	3.52729
17415774	Foxd3	forkhead box D3	20.50708	18.93784	15.57921	17.50846	17.96952	16.56596
17363640	Foxd4	forkhead box D4	4.112712	4.035311	3.81679	3.816156	4.992768	3.467702
17413817	Foxe1	forkhead box E1	11.96866	11.04055	12.33281	12.36243	10.82226	12.67892
17428427	Foxe3	forkhead box E3	12.69836	10.37119	9.530025	12.17424	10.2338	11.52296
17506291	Foxf1	forkhead box F1	26.21953	21.76759	22.34918	23.2389	19.63846	20.77433

17286332	Foxf2	forkhead box F2	5.555358	5.759885	6.561383	6.441247	7.974146	6.040299
17275306	Foxg1	forkhead box G1	3.464003	3.440886	3.870205	3.616389	4.478762	3.840888
17318665	Foxh1	forkhead box H1	6.524648	6.714547	5.238595	5.525787	4.644876	8.923378
17261645	Foxi1	forkhead box I1	27.25464	30.60807	31.85328	24.40576	25.21028	20.88724
17484229	Foxi2	forkhead box I2	8.63227	7.294606	6.992896	6.992896	6.992896	8.790773
17459449	Foxi3	forkhead box I3	7.519934	10.23515	8.398781	9.756272	9.393482	6.589779
17272339	Foxj1	forkhead box J1	7.399101	6.860482	8.741434	6.755909	5.996954	8.43466
17462705	Foxj2	forkhead box J2	26.01425	34.82472	45.02767	32.35664	31.77698	37.60467
17417926	Foxj3	forkhead box J3	29.21161	28.21362	29.41867	26.9044	28.84281	28.93212
17444202	Foxk1	forkhead box K1	38.72974	39.4945	49.33946	33.90768	45.07514	46.8946
17259600	Foxk2	forkhead box K2	30.81199	33.5114	31.19926	27.69378	35.68387	22.83081
17506300	Foxl1	forkhead box L1	8.158025	9.79829	11.35314	8.51685	7.972764	7.818815
17520675	Foxl2	forkhead box L2	8.135945	8.093199	7.451289	7.753512	7.951682	7.624063
17529981	Foxl2os	forkhead box L2, opposite strand	3.865996	4.121016	3.729055	3.745297	3.729055	3.729055
17463401	Foxm1	forkhead box M1	4.802648	5.91427	6.250146	5.956686	6.535919	5.726882
17266424	Foxn1	forkhead box N1	4.514981	3.913122	3.469024	4.141232	4.20408	4.008604
17340323	Foxn2	forkhead box N2	47.72952	38.82704	44.53198	41.05454	39.78713	32.23732
17283203	Foxn3	forkhead box N3	84.81926	84.81926	64.99649	76.10134	56.99407	75.1265
17451512	Foxn4	forkhead box N4	6.217351	9.747485	7.517849	6.779693	6.820417	7.050668
17397497	Foxo1	forkhead box O1	46.167	72.77444	76.21273	82.46614	82.62807	98.86514
17240529	Foxo3	forkhead box O3	29.9549	32.27779	36.54343	37.84209	38.54842	47.22176
17536657	Foxo4	forkhead box O4	20.91593	25.27396	26.17758	28.18606	28.83342	24.04908
17429449	Foxo6	forkhead box O6	14.77996	16.59964	14.81031	16.11978	17.57375	13.39098
17417985	Foxo6os	forkhead box O6, opposite strand	4.01461	4.20927	3.448598	4.793303	4.809244	4.521464
17469414	Foxp1	forkhead box P1	36.74765	45.62776	45.02767	39.14022	55.78479	46.561
17456084	Foxp2	forkhead box P2	9.162746	14.4805	17.27208	14.59305	15.99301	14.5334
17532811	Foxp3	forkhead box P3	6.450943	7.129001	6.924117	5.685393	7.271686	7.302093
17345740	Foxp4	forkhead box P4	28.58884	26.22771	27.07482	29.19137	23.14358	24.53875
17286320	Foxq1	forkhead box Q1	114.2516	23.77939	41.31289	30.56037	55.479	38.46915
17526287	Foxr1	forkhead box R1	5.28583	6.355519	4.651223	5.099126	4.908152	4.883278
17538983	Foxr2	forkhead box R2	3.899583	3.861523	3.604752	3.617267	3.635264	4.764319
17393004	Foxs1	forkhead box S1	8.406994	8.659418	8.743495	8.743495	12.63917	9.020891
17432347	Fhad1	forkhead-associated (FHA) phosphopeptide binding domain 1	4.28644	4.20927	4.967121	5.239648	3.772053	4.46695
17421145	Fhad1os1	forkhead-associated (FHA) phosphopeptide binding domain 1, opposite strand 1	3.938619	5.206053	4.20927	4.20927	4.227457	4.630731
17421142	Fhad1os2	forkhead-associated (FHA) phosphopeptide binding domain 1, opposite strand 2	12.28452	11.78941	12.28452	12.28452	10.48228	14.69364
17234588	Ftcd	formiminotransferase cyclodeaminase	233.6945	242.9928	253.6244	300.1105	268.4352	287.4147
17374332	Fmn1	formin 1	10.43313	14.62718	15.23914	16.65531	18.81485	14.62718
17219766	Fmn2	formin 2	3.654388	2.664546	2.905326	3.840808	3.025828	3.186141
17383748	Fnbp1	formin binding protein 1	40.16119	38.36211	48.28222	37.66154	37.02608	31.97273
17409963	Fnbp1l	formin binding protein 1-like	49.80884	46.29807	48.62715	47.3903	36.4329	48.6946
17373028	Fnbp4	formin binding protein 4	23.0397	34.7206	31.90587	24.86596	23.89605	24.97184
17512388	Fhod1	formin homology 2 domain containing 1	13.18029	12.5597	13.28494	14.91622	11.71585	13.18029
17349034	Fhod3	formin homology 2 domain containing 3	14.54025	15.255	13.0578	11.9337	17.39391	16.41576
17257100	Fmnl1	formin-like 1	7.459867	7.668211	7.942593	7.545257	7.935109	6.79244
17370807	Fmnl2	formin-like 2	18.37917	18.95833	18.64	19.70487	17.44413	15.3561
17321524	Fmnl3	formin-like 3	11.08088	13.43813	15.87506	16.92273	10.55745	13.30734
17341276	Fpr1	formyl peptide receptor 1	14.10574	17.60533	14.29273	15.008	18.22517	14.61158
17333731	Fpr2	formyl peptide receptor 2	5.577583	3.938128	4.059494	4.345554	4.352065	4.453193
17333739	Fpr3	formyl peptide receptor 3	3.511506	3.593476	3.115622	4.16348	3.758664	3.586956
17341340	Fpr-rs3	formyl peptide receptor, related sequence 3	3.735289	2.805621	3.470876	4.184079	3.23232	3.149821
17333742	Fpr-rs4	formyl peptide receptor, related sequence 4	2.586498	2.918041	2.606909	2.606909	2.188921	2.557865
17341303	Fpr-rs6	formyl peptide receptor, related sequence 6	1.94813	2.275731	2.063037	2.236713	2.064854	2.779085
17341300	Fpr-rs7	formyl peptide receptor, related sequence 7	1.941417	2.336583	1.982884	1.902821	1.884419	2.152794
17324916	Fyttd1	forty-two-three domain containing 1	22.65645	26.22753	19.29373	20.94741	21.71906	22.53444
17356427	Fosl1	fos-like antigen 1	4.18521	3.675702	3.480851	5.007012	6.051111	4.736364
17436237	Fosl2	fos-like antigen 2	39.5652	42.50586	49.60212	40.24507	37.57419	42.3362
17534960	Fhl1	four and a half LIM domains 1	30.13253	33.98909	34.26895	21.58774	29.93082	32.35148
17222642	Fhl2	four and a half LIM domains 2	13.88745	21.87347	17.67845	17.67845	18.39217	17.96267
17418271	Fhl3	four and a half LIM domains 3	16.93599	17.29328	14.96697	15.82123	14.87791	14.63834
17243633	Fhl4	four and a half LIM domains 4	3.011266	2.774523	3.011266	3.011266	3.553745	3.366105
17423665	Fhl5	four and a half LIM domains 5	3.437596	3.014336	3.010222	3.061312	3.162072	3.339333
17388725	Fjx1	four jointed box 1 (Drosophila)	5.457537	4.90261	6.478995	4.859036	6.58608	5.296135
17513550	Fendrr	Foxf1 adjacent non-coding developmental regulatory RNA	7.988585	7.631147	9.163	10.90305	12.4075	9.221619
17507128	Fcor	Foxo1 corepressor	5.717958	4.647388	5.141039	5.191927	4.899383	5.447144
17364350	Fra10ac1	FRA10AC1 homolog (human)	31.48843	23.79506	27.17579	33.69938	36.6261	30.89454
17303474	Fhit	fragile histidine triad gene	10.09907	11.58005	9.200613	10.1335	13.79688	10.19387
17297116	Fhitos	fragile histidine triad gene, opposite strand	3.723218	4.408507	3.873667	4.620279	3.183359	4.814347
17535233	Fmr1nb	fragile X mental retardation 1 neighbor	2.700263	3.688643	2.933331	3.378587	3.772341	3.339703
17396823	Fxr1	fragile X mental retardation gene 1, autosomal homolog	129.9714	120.1339	115.828	115.828	131.1387	127.9565
17535208	Fmr1	fragile X mental retardation syndrome 1	47.62311	37.13043	44.78551	48.57089	43.43602	53.33276
17542121	Fmr1os	fragile X mental retardation syndrome 1, opposite strand	3.143736	3.5442	3.367038	3.29674	3.459611	3.29674
17251687	Fxr2	fragile X mental retardation, autosomal homolog 2	40.54129	41.64929	44.23235	41.0833	37.51485	43.22308
17426808	Frem1	Fras1 related extracellular matrix protein 1	5.48933	6.002485	5.48933	5.48933	4.656416	5.48933
17405208	Frem2	Fras1 related extracellular matrix protein 2	5.270026	5.050303	6.425728	5.705329	6.665439	5.839804
17502799	Frem3	Fras1 related extracellular matrix protein 3	3.00399	2.823589	2.326321	2.772716	2.818954	3.042421
17439388	Fras1	Fraser syndrome 1 homolog (human)	4.608062	4.528207	4.091302	3.921838	4.1864	4.886969
17363598	Fxn	frataxin	61.8315	56.50435	54.18344	58.73669	59.86496	63.76708
17489318	Ffar1	free fatty acid receptor 1	6.205081	8.642088	7.186005	11.50963	7.057514	6.462803
17489304	Ffar2	free fatty acid receptor 2	3.523429	3.278896	4.194038	3.595344	3.61817	3.979728
17489314	Ffar3	free fatty acid receptor 3	2.878007	3.263862	3.263862	3.703322	3.085747	3.118906
17359113	Ffar4	free fatty acid receptor 4	6.790604	7.515036	7.698994	7.278595	15.2787	7.559129
17359464	Frat1	frequently rearranged in advanced T cell lymphomas	24.1612	24.23517	24.7856	27.42025	26.64642	23.45348
17364721	Frat2	frequently rearranged in advanced T cell lymphomas 2	5.919355	10.57532	7.63755	7.299158	7.287884	7.736977
17525310	Fli1	Friend leukemia integration 1	48.38339	57.1135	54.90935	45.18087	39.89456	39.64728

17421534	Fv1	Friend virus susceptibility 1	12.0469	9.20712	9.277139	10.16607	11.6151	12.58776
17445325	Fzd1	frizzled homolog 1 (Drosophila)	21.43134	25.46899	23.05552	25.83599	25.94815	23.76852
17442796	Fzd10	frizzled homolog 10 (Drosophila)	3.555839	3.213733	3.19523	2.998664	2.717839	3.000099
17256975	Fzd2	frizzled homolog 2 (Drosophila)	4.070342	4.987822	3.793843	4.144764	4.210174	4.558724
17307738	Fzd3	frizzled homolog 3 (Drosophila)	5.082647	4.350707	4.461318	4.318918	3.611955	4.740174
17480036	Fzd4	frizzled homolog 4 (Drosophila)	49.4641	58.85406	76.7971	55.86915	51.40405	69.38587
17223793	Fzd5	frizzled homolog 5 (Drosophila)	25.22269	16.30768	19.96277	24.75109	15.05812	22.0984
17311179	Fzd6	frizzled homolog 6 (Drosophila)	12.94919	13.35807	12.17466	12.211	11.40694	9.527251
17213265	Fzd7	frizzled homolog 7 (Drosophila)	24.36773	24.78216	22.05387	30.3899	23.69006	28.29941
17348276	Fzd8	frizzled homolog 8 (Drosophila)	8.038743	6.992896	7.698994	8.306784	9.997241	19.32585
17453541	Fzd9	frizzled homolog 9 (Drosophila)	9.219893	7.120359	6.292049	6.746457	5.516144	6.958325
17387305	Frzb	frizzled-related protein	20.64585	17.4225	29.27039	21.17779	15.09386	20.31861
17259623	Fn3k	fructosamine 3 kinase	6.901357	9.646463	9.237036	9.488039	4.839809	8.360273
17259613	Fn3krp	fructosamine 3 kinase related protein	20.59768	23.31716	21.08829	30.63354	23.30068	29.79194
17293557	Fbp1	fructose bisphosphatase 1	1300.427	1471.345	1445.427	1458.43	1465.178	1470.081
17293547	Fbp2	fructose bisphosphatase 2	6.482319	8.396395	16.84245	8.343716	8.599603	11.24366
17508968	Frg1	FSHD region gene 1	28.03583	31.54436	31.87515	32.59797	36.27491	36.60731
17540075	Ftsj1	FtsJ homolog 1 (E. coli)	63.93792	56.62825	52.37694	46.40763	45.13079	50.81773
17454546	Ftsj2	FtsJ homolog 2 (E. coli)	24.82772	22.04012	18.12727	20.74009	22.04012	25.53174
17270783	Ftsj3	FtsJ homolog 3 (E. coli)	60.42108	45.51247	48.46966	51.86826	48.98065	51.05003
17543795	Ftx	Ftx transcript, Xist regulator (non-protein coding)	13.32173	15.17464	12.49943	12.53708	11.52744	12.53708
17512944	Fuk	fucokinase	19.42873	28.57536	24.06209	24.27619	24.98499	27.52632
17497595	Fuom	fucose mutarotase	14.72433	13.88158	15.92377	14.5462	15.26716	14.94333
17411392	Fpgt	fucose-1-phosphate guanylyltransferase	21.09487	21.09487	23.10719	23.88909	25.46952	19.96194
17419965	Fuca1	fucosidase, alpha-L- 1, tissue	107.8371	83.35634	94.30288	98.1108	92.85193	90.67607
17231717	Fuca2	fucosidase, alpha-L- 2, plasma	16.16722	16.16722	13.89255	15.43678	16.16722	16.16722
17477914	Fut1	fucosyltransferase 1	4.690755	3.757883	4.742113	4.569765	3.248086	3.699243
17500412	Fut10	fucosyltransferase 10	6.531933	6.671448	6.2989	6.486049	6.960978	4.789151
17297490	Fut11	fucosyltransferase 11	14.50914	19.79782	14.75917	16.23595	15.8656	12.57085
17490912	Fut2	fucosyltransferase 2	5.638377	7.794951	6.283289	6.030594	5.246626	5.13883
17524077	Fut4	fucosyltransferase 4	4.901149	4.136126	3.169906	3.892913	3.263862	3.396432
17338879	Fut4-ps1	fucosyltransferase 4, pseudogene 1	3.362397	3.362397	3.362397	5.075078	3.332073	3.56858
17367999	Fut7	fucosyltransferase 7	4.513229	6.1409	5.490281	4.906179	6.54848	5.412969
17276642	Fut8	fucosyltransferase 8	20.47242	22.17758	27.15733	22.24054	25.79519	21.34964
17423701	Fut9	fucosyltransferase 9	4.350617	5.013785	3.992189	3.992189	4.269716	4.097972
17414228	Fktn	fukutin	12.01379	10.65324	13.22962	13.8888	13.39906	11.46838
17486959	Fkrp	fukutin related protein	16.64515	15.25204	12.94399	15.28368	16.23078	12.47087
17230153	Fh1	fumarate hydratase 1	350.41	419.3323	546.7547	439.4119	418.3945	474.2914
17493005	Fah	fumarylacetoacetate hydrolase	520.6392	574.8812	600.3125	560.6785	599.6097	578.948
17342038	Fahd1	fumarylacetoacetate hydrolase domain containing 1	31.28892	27.3305	26.81689	32.29861	37.3848	35.47793
17391259	Fahd2a	fumarylacetoacetate hydrolase domain containing 2A	11.46798	12.9605	10.67105	12.41842	11.01746	11.15486
17540420	Fundc1	FUN14 domain containing 1	5.338965	5.166402	5.431083	5.848878	5.338965	5.914639
17535951	Fundc2	FUN14 domain containing 2	7.836666	16.23595	16.62889	19.81581	10.50868	15.43764
17405001	1700034I23Rik	FUN14 domain containing 2 pseudogene	3.474198	2.374132	2.482373	2.780974	3.344476	2.533005
17404993	Gm10731	FUN14 domain containing 2 pseudogene	4.944141	4.657901	3.915509	4.339173	3.429124	3.828529
17541567	Firre	functional intergenic repeating RNA element	14.00073	17.51659	21.0012	17.87882	14.27976	17.69488
17492598	Furin	furin (paired basic amino acid cleaving enzyme)	251.5341	260.334	270.8894	250.0998	273.3679	258.3924
17445089	Fry	furry homolog (Drosophila)	5.669769	5.681808	5.500489	5.669769	6.201856	5.538748
17445160	Fry	furry homolog (Drosophila)	2.256755	2.028509	2.045509	2.083357	2.083357	2.408091
17448740	Fryl	furry homolog-like (Drosophila)	28.29294	29.6648	29.6648	31.29434	29.48379	27.02945
17483546	Fus	fused in sarcoma	44.89367	75.07757	74.15078	67.86379	93.39219	61.56677
17477619	Fuz	fuzzy homolog (Drosophila)	24.09898	24.57007	36.48825	30.06264	29.41459	33.2025
17489420	Fxyd1	FXYD domain-containing ion transport regulator 1	158.8444	215.1069	294.0382	226.5114	239.3155	213.4065
17516784	Fxyd2	FXYD domain-containing ion transport regulator 2	3.462322	3.568604	3.972122	3.703322	3.703322	3.703322
17489431	Fxyd3	FXYD domain-containing ion transport regulator 3	4.698206	5.789224	5.828239	7.194428	6.647537	5.924773
17470095	Fxyd4	FXYD domain-containing ion transport regulator 4	3.535857	3.406122	3.729055	3.729055	3.342204	3.729055
17489399	Fxyd5	FXYD domain-containing ion transport regulator 5	12.5937	16.44937	25.43565	13.77901	15.71336	12.25722
17516777	Fxyd6	FXYD domain-containing ion transport regulator 6	5.67197	9.421782	10.64202	7.698994	7.761201	6.937231
17489414	Fxyd7	FXYD domain-containing ion transport regulator 7	15.09397	13.12914	16.21537	15.2697	20.12867	14.34106
17309981	Fyb	FYN binding protein	8.501338	15.15456	10.55438	11.15416	14.80805	11.53575
17232649	Fyn	Fyn proto-oncogene	18.0593	14.38626	15.60958	14.02948	11.6417	12.41343
17232534	Frk	fyn-related kinase	7.850094	9.157793	11.93428	11.21891	10.7828	10.29855
17532540	Fyco1	FYVE and coiled-coil domain containing 1	92.11665	94.34407	101.9586	101.9692	87.4393	89.53067
17538628	Fgd1	FYVE, RhoGEF and PH domain containing 1	7.128359	10.95183	14.2421	9.310251	9.310251	9.599239
17335519	Fgd2	FYVE, RhoGEF and PH domain containing 2	9.189077	14.16442	12.22489	10.02833	13.10877	10.23125
17292450	Fgd3	FYVE, RhoGEF and PH domain containing 3	5.917304	7.912042	9.810115	6.949889	6.090286	6.266066
17328508	Fgd4	FYVE, RhoGEF and PH domain containing 4	34.50683	34.01549	33.33417	31.27093	37.09801	37.83475
17460994	Fgd5	FYVE, RhoGEF and PH domain containing 5	11.13686	13.38022	13.20242	14.72208	12.13767	10.51801
17236638	Fgd6	FYVE, RhoGEF and PH domain containing 6	75.48196	64.51667	66.88218	62.19212	61.36908	71.11392
17398152	Gfm1	G elongation factor, mitochondrial 1	168.5941	161.8664	138.7131	150.0282	123.681	136.3178
17289344	Gfm2	G elongation factor, mitochondrial 2	117.6257	95.91403	100.5954	104.9305	91.05965	101.2277
17293103	Gkap1	G kinase anchoring protein 1	29.21769	34.776	38.19972	33.31569	32.80605	34.53028
17336970	Gpank1	G patch domain and ankyrin repeats 1	8.069728	5.825977	6.522161	4.370414	5.48933	4.936471
17532908	Gpkow	G patch domain and KOW motifs	29.4556	28.58131	27.09923	33.33255	26.63904	23.29454
17489738	Gpatch1	G patch domain containing 1	33.0102	26.98078	26.04185	28.40829	26.77918	27.3724
17339840	Gpatch11	G patch domain containing 11	21.5044	23.63151	24.29656	22.52351	28.25022	22.54007
17220608	Gpatch2	G patch domain containing 2	10.41665	10.38033	10.41665	10.41665	10.84862	9.61762
17548948	Gpatch2	G patch domain containing 2	8.50741	7.975915	11.09963	8.426304	7.885542	10.37443
17277490	Gpatch2l	G patch domain containing 2 like	23.65363	26.99069	25.09208	25.25015	18.66689	23.62774
17419626	Gpatch3	G patch domain containing 3	13.34558	14.50843	13.51566	16.17585	13.47889	14.11728
17398915	Gpatch4	G patch domain containing 4	18.62954	21.8721	24.71766	25.16593	28.15541	23.05615
17270269	Gpatch8	G patch domain containing 8	29.60194	29.45315	28.79507	32.27891	26.91671	36.38571
17259512	Gps1	G protein pathway suppressor 1	85.91928	78.21753	76.34702	85.09427	79.27697	78.0491
17251799	Gps2	G protein pathway suppressor 2	13.49572	19.62962	23.53246	19.62771	17.40971	17.71206

17305012	Gprin2	G protein regulated inducer of neurite outgrowth 2	18.592	20.86626	21.18469	15.66063	14.84803	18.49649
17214162	Gpbar1	G protein-coupled bile acid receptor 1	5.7136	7.466386	6.992896	6.322039	5.660934	8.123098
17444016	Gper1	G protein-coupled estrogen receptor 1	10.00591	10.43783	10.43783	12.99937	12.13465	11.37236
17223710	Gpr1	G protein-coupled receptor 1	3.300901	3.026709	3.462826	2.840943	3.519572	3.337065
17541919	Gpr101	G protein-coupled receptor 101	13.55676	16.94761	12.742	14.03075	13.12713	12.91531
17369340	Gpr107	G protein-coupled receptor 107	17.10149	30.19651	26.03553	20.86626	18.78709	22.3746
17346572	Gpr108	G protein-coupled receptor 108	43.75634	33.80583	33.71643	41.81069	37.38895	42.17511
17284247	Gpr132	G protein-coupled receptor 132	10.71597	12.21879	9.520913	9.59538	10.48228	5.731171
17281789	Gpr135	G protein-coupled receptor 135	11.30336	10.29855	10.29855	9.919862	9.485475	10.29855
17362138	Gpr137	G protein-coupled receptor 137	19.91923	22.17481	19.06851	21.38534	18.99147	20.27233
17290695	Gpr137b	G protein-coupled receptor 137B	4.216658	6.36897	5.552972	3.984448	4.119788	3.570757
17290655	Gpr137b-ps	G protein-coupled receptor 137B, pseudogene	4.67994	6.451032	5.957388	6.171709	8.442791	7.072156
17299185	Gpr137c	G protein-coupled receptor 137C	3.595943	2.953407	3.863585	3.09586	3.465251	3.267552
17495617	Gpr139	G protein-coupled receptor 139	3.161655	4.531316	4.938661	4.499485	3.317899	3.997866
17290894	Gpr141	G protein-coupled receptor 141	2.600466	2.606909	3.209614	2.894732	3.156859	2.606909
17258089	Gpr142	G protein-coupled receptor 142	4.828617	4.636673	4.636673	4.636673	2.924257	4.280947
17538960	Gpr143	G protein-coupled receptor 143	3.476872	3.160779	3.525334	3.371849	3.56292	3.805774
17444002	Gpr146	G protein-coupled receptor 146	57.66124	70.82319	83.8152	74.5228	76.79338	80.28842
17405541	Gpr149	G protein-coupled receptor 149	5.478876	5.168301	4.132773	7.025642	5.535524	5.213131
17331212	Gpr15	G protein-coupled receptor 15	3.078505	3.783155	2.348322	3.260583	3.442961	3.103143
17294485	Gpr150	G protein-coupled receptor 150	5.235365	4.536344	4.545598	4.538136	5.145816	5.456289
17354074	Gpr151	G protein-coupled receptor 151	4.20927	3.720045	3.877052	4.651706	4.20927	4.20927
17356153	Gpr152	G protein-coupled receptor 152	4.573314	5.210168	5.453793	5.307712	4.735412	4.620023
17422138	Gpr153	G protein-coupled receptor 153	9.056728	9.583881	10.01028	9.123517	8.266577	7.993459
17386514	Gpr155	G protein-coupled receptor 155	56.66948	46.8998	41.93579	52.28445	52.3334	42.7603
17325540	Gpr156	G protein-coupled receptor 156	5.666076	7.097249	5.710591	8.230557	5.369694	7.175056
17421867	Gpr157	G protein-coupled receptor 157	9.485015	10.58119	14.22957	13.4731	9.259152	9.21772
17367458	Gpr158	G protein-coupled receptor 158	3.744051	3.220132	4.20927	5.03283	3.235099	3.590065
17536428	Pgr15l	G protein-coupled receptor 15-like	4.754093	4.638827	5.715065	5.713758	4.614774	4.886021
17396604	Gpr160	G protein-coupled receptor 160	6.992896	7.105273	7.685079	7.790522	6.515248	6.992896
17218942	Gpr161	G protein-coupled receptor 161	4.660937	5.797416	6.57805	5.932129	6.895332	5.941882
17470948	Gpr162	G protein-coupled receptor 162	4.80125	5.101318	3.784388	4.670315	5.481687	4.730229
17536420	Gpr165	G protein-coupled receptor 165	2.95652	3.106177	2.871511	2.750149	2.938684	2.638193
17353267	Gpr17	G protein-coupled receptor 17	8.999345	8.875079	8.093143	8.68015	8.50517	9.699296
17405458	Gpr171	G protein-coupled receptor 171	7.572082	7.759641	6.890459	7.307814	6.847513	8.614936
17537134	Gpr174	G protein-coupled receptor 174	3.59694	2.908792	3.346887	2.890883	3.08032	3.08032
17389709	Gpr176	G protein-coupled receptor 176	8.742283	8.51685	11.12798	9.488237	8.913858	8.3031
17268523	Gpr179	G protein-coupled receptor 179	7.212953	6.081849	6.13116	6.958662	9.15773	7.467317
17309644	Gpr18	G protein-coupled receptor 18	5.069523	4.43877	4.886969	5.003716	4.886969	3.914288
17302675	Gpr180	G protein-coupled receptor 180	54.61631	32.88186	36.11434	37.41928	39.07462	36.32397
17246038	Gpr182	G protein-coupled receptor 182	63.0909	47.33581	81.68108	54.58339	33.6842	47.37421
17309649	Gpr183	G protein-coupled receptor 183	11.69768	12.55822	13.46666	11.75971	10.21587	12.6075
17472043	Gpr19	G protein-coupled receptor 19	4.120416	3.321189	3.980722	4.726951	4.185326	4.191597
17317921	Gpr20	G protein-coupled receptor 20	6.836605	5.721923	5.433644	5.889928	3.499843	5.433644
17370412	Gpr21	G protein-coupled receptor 21	6.35856	6.280807	6.449467	6.7851	7.719353	5.620818
17280571	Gpr22	G protein-coupled receptor 22	4.185645	5.334119	4.144592	4.469241	4.49101	5.274448
17227480	Gpr25	G protein-coupled receptor 25	7.184412	7.600951	7.49454	7.393308	6.348035	6.236774
17484049	Gpr26	G protein-coupled receptor 26	7.936209	8.422859	7.339491	6.411847	7.045734	7.923182
17461155	Gpr27	G protein-coupled receptor 27	13.82819	13.06568	16.88558	9.788176	11.91891	17.0733
17340998	Gpr31b	G protein-coupled receptor 31, D17Leh66b region	9.902206	9.202016	8.9352	13.62279	15.04842	9.637975
17281071	Gpr33	G protein-coupled receptor 33	4.690072	3.898773	3.944931	5.477889	3.75882	4.20927
17533446	Gpr34	G protein-coupled receptor 34	3.892454	3.971351	3.884691	4.281125	4.857453	4.066704
17215873	Gpr35	G protein-coupled receptor 35	10.3012	9.359945	10.34735	8.551574	10.22444	9.310251
17465238	Gpr37	G protein-coupled receptor 37	3.815362	4.063323	4.063323	4.691893	4.086824	4.175214
17227261	Gpr37l1	G protein-coupled receptor 37-like 1	10.24345	9.980901	8.422333	8.248547	8.597576	8.685386
17216745	Gpr39	G protein-coupled receptor 39	9.083763	10.72867	10.98452	10.57745	11.4905	10.121
17474529	Gpr4	G protein-coupled receptor 4	10.70802	11.67653	10.23004	9.404362	8.682196	11.48772
17212334	Gpr45	G protein-coupled receptor 45	2.888439	3.49783	3.434405	4.304543	2.919781	3.729055
17228790	Gpr52	G protein-coupled receptor 52	9.634969	13.44856	11.91734	10.63951	6.845899	9.903305
17225134	Gpr55	G protein-coupled receptor 55	5.504914	5.809323	5.779321	4.664621	4.950211	4.741883
17240401	Gpr6	G protein-coupled receptor 6	7.246478	6.514977	7.095577	8.09376	7.031195	9.853799
17409229	Gpr61	G protein-coupled receptor 61	4.864293	5.246735	4.199391	3.999224	5.363594	5.551625
17530699	Gpr62	G protein-coupled receptor 62	5.248227	4.836086	5.087123	5.087123	5.087123	4.725674
17412282	Gpr63	G protein-coupled receptor 63	6.171709	6.171709	6.171709	6.207963	6.962184	6.274759
17283293	Gpr68	G protein-coupled receptor 68	14.28669	13.50377	13.38439	12.31086	13.13614	13.39423
17248191	Gpr75	G protein-coupled receptor 75	6.735056	8.450168	9.480808	9.391333	9.256521	10.29855
17533456	Gpr82	G protein-coupled receptor 82	2.533251	2.446976	2.46403	2.515404	2.417926	3.056075
17514789	Gpr83	G protein-coupled receptor 83	3.573877	4.639952	5.428298	3.286313	5.902884	3.729055
17322355	Gpr84	G protein-coupled receptor 84	5.664231	3.916704	4.126847	4.461937	4.537665	4.754422
17464922	Gpr85	G protein-coupled receptor 85	2.745026	3.829988	2.950031	3.044995	3.267574	3.059912
17405471	Gpr87	G protein-coupled receptor 87	2.527673	2.820244	2.200849	2.715222	2.308827	2.745407
17408135	Gpr89	G protein-coupled receptor 89	53.85917	39.48821	45.30788	39.57178	46.61977	42.77868
17537815	Gprasp1	G protein-coupled receptor associated sorting protein 1	11.56642	11.56642	11.53415	12.89169	10.40936	11.56642
17537839	Gprasp2	G protein-coupled receptor associated sorting protein 2	3.381258	3.588622	4.653738	4.20927	3.117112	3.325382
17499325	Grk1	G protein-coupled receptor kinase 1	3.966729	3.616339	4.408598	5.592565	3.875655	3.508465
17436659	Grk4	G protein-coupled receptor kinase 4	3.062691	3.577124	3.577124	3.577124	3.577124	3.577124
17360890	Grk5	G protein-coupled receptor kinase 5	17.09888	14.24388	13.02381	17.64846	9.157222	17.89085
17287682	Grk6	G protein-coupled receptor kinase 6	31.06913	25.3185	28.73227	38.91461	20.13523	25.00925
17253276	Git1	G protein-coupled receptor kinase-interactor 1	25.27116	26.45393	39.69815	29.53226	28.08932	27.96363
17451578	Git2	G protein-coupled receptor kinase-interactor 2	33.279	29.9495	31.00416	30.71454	22.13887	28.02262
17463747	Gprc5a	G protein-coupled receptor, family C, group 5, member A	5.777158	5.8377	5.485298	4.794266	5.174466	5.906977
17495610	Gprc5b	G protein-coupled receptor, family C, group 5, member B	19.78027	22.12384	19.37897	11.96468	16.94409	19.96125
17258093	Gprc5c	G protein-coupled receptor, family C, group 5, member C	28.34102	31.00309	29.40317	30.10268	30.47174	31.00309
17472056	Gprc5d	G protein-coupled receptor, family C, group 5, member D	3.462322	3.462322	3.462322	4.341429	3.462322	3.306626

17240737	Gprc6a	G protein-coupled receptor, family C, group 6, member A	4.93695	4.493564	4.527831	5.546663	5.04502	5.383446
17292753	Gprin1	G protein-regulated inducer of neurite outgrowth 1	15.94663	11.91329	12.02963	12.13237	10.06427	13.08925
17313895	Gtse1	G two S phase expressed protein 1	10.02277	11.57331	10.50817	10.70772	9.506801	11.26129
17231248	G0s2	G0/G1 switch gene 2	35.32457	73.44641	115.5329	97.48511	64.67248	75.37374
17328201	Gspt1	G1 to S phase transition 1	105.5513	111.5804	102.6443	111.2514	98.25099	102.6443
17536329	Gspt2	G1 to S phase transition 2	3.853368	5.490091	5.03859	5.080287	3.968297	3.7169
17222354	4930594C11Rik	G1 to S phase transition pseudogene	3.739797	3.063243	3.383297	3.379102	2.829309	3.015673
17275332	G2e3	G2/M-phase specific E3 ubiquitin ligase	13.7964	13.06568	14.22523	13.06568	12.76764	13.66014
17336918	G6bos	G6b opposite strand	9.438123	13.023	10.65966	13.05192	15.43849	11.24366
17326816	Gabpa	GA repeat binding protein, alpha	72.40312	80.96127	80.96127	81.88742	78.09563	82.67848
17391112	Gabpb1	GA repeat binding protein, beta 1	18.75925	18.04929	17.85591	15.91593	17.85591	15.39916
17407721	Gabpb2	GA repeat binding protein, beta 2	19.8224	18.65357	20.42211	18.5201	19.16803	19.1082
17272084	Galk1	galactokinase 1	69.86171	90.21028	77.39884	73.79289	68.69152	58.55742
17375665	Galk2	galactokinase 2	19.06507	20.65573	24.28612	20.18819	19.23404	21.65022
17513779	Galns	galactosamine (N-acetyl)-6-sulfate sulfatase	14.96987	20.52172	20.63226	17.58459	14.54751	15.21392
17339910	Galm	galactose mutarotase	106.7957	135.5555	143.9925	132.1717	117.4359	140.056
17412932	Galt	galactose-1-phosphate uridyl transferase	29.29454	30.55486	26.56104	24.62036	25.69134	27.01989
17246700	Gal3st1	galactose-3-O-sulfotransferase 1	15.06501	23.29454	24.58455	23.29454	13.91704	23.29454
17216113	Gal3st2	galactose-3-O-sulfotransferase 2	13.76955	8.976543	14.11424	10.41665	9.408339	5.655835
17356383	Gal3st3	galactose-3-O-sulfotransferase 3	17.9445	20.3917	15.77743	18.06456	17.81018	15.6359
17454301	Gal3st4	galactose-3-O-sulfotransferase 4	3.86147	3.34019	3.480851	3.121436	3.051355	3.480851
17419995	Gale	galactose-4-epimerase, UDP	19.64786	17.08822	16.95431	16.79826	20.97691	18.1318
17544517	Gla	galactosidase, alpha	16.06902	16.49835	17.45683	14.78703	11.28974	16.49835
17525118	Glb1l3	galactosidase, beta 1 like 3	2.688199	3.07309	2.687454	2.837696	2.609168	2.837696
17224520	Glb1l	galactosidase, beta 1-like	5.877978	7.76061	6.379795	7.975473	7.82581	6.976
17525090	Glb1l2	galactosidase, beta 1-like 2	2.789487	3.257579	3.459899	2.581465	2.581733	2.949417
17283096	Galc	galactosylceramidase	12.09878	10.6286	12.55761	10.38083	9.310251	15.02382
17360942	Gal	galanin	3.412479	4.30708	4.30708	4.20927	3.519401	4.20927
17355732	Galr1	galanin receptor 1	6.58786	7.718069	6.851026	7.000025	7.979896	6.563885
17258563	Galr2	galanin receptor 2	4.452514	4.806744	3.91323	4.290423	4.320805	4.320805
17312899	Galr3	galanin receptor 3	6.46464	12.73511	10.51779	11.56241	6.15987	8.613025
17473554	Galp	galanin-like peptide	9.482188	10.48228	10.64018	9.994677	10.72369	10.48228
17454568	Grifin	galectin-related inter-fiber protein	20.9023	27.38777	25.40129	27.62361	20.42721	20.78456
17322402	Gtsf1	gametocyte specific factor 1	3.540885	3.657505	3.653502	3.438382	4.21248	3.876407
17394008	Gtsf1l	gametocyte specific factor 1-like	2.94172	2.895013	3.108482	2.736003	3.354249	3.623566
17476097	Ggn	gametogenetin	16.41201	18.78853	14.91674	12.35189	14.24654	13.49544
17335046	Ggnbp1	gametogenetin binding protein 1	12.82023	10.79432	13.57999	15.51046	11.59588	12.98442
17267076	Ggnbp2	gametogenetin binding protein 2	119.5856	147.4978	133.6772	148.533	159.0945	162.0281
17254470	Ggnbp2os	gametogenetin binding protein 2, opposite strand	10.51356	14.89091	17.21411	16.78848	13.99743	17.2319
17261608	Gabrp	gamma-aminobutyric acid (GABA) A receptor, pi	5.754218	5.967142	3.921512	4.20927	4.618678	4.618678
17261896	Gabra1	gamma-aminobutyric acid (GABA) A receptor, subunit alpha 1	5.006318	4.669085	4.070822	4.8435	6.193393	4.221249
17448577	Gabra2	gamma-aminobutyric acid (GABA) A receptor, subunit alpha 2	3.607126	3.54506	4.221747	3.497151	3.54506	3.030635
17542220	Gabra3	gamma-aminobutyric acid (GABA) A receptor, subunit alpha 3	7.151372	11.99058	11.11865	9.472137	9.220724	10.09095
17448595	Gabra4	gamma-aminobutyric acid (GABA) A receptor, subunit alpha 4	2.363771	2.46275	2.695924	3.12555	3.935317	2.599763
17491480	Gabra5	gamma-aminobutyric acid (GABA) A receptor, subunit alpha 5	4.997373	5.310877	5.386134	5.386134	5.53422	5.63123
17261913	Gabra6	gamma-aminobutyric acid (GABA) A receptor, subunit alpha 6	2.710333	2.348777	2.67402	2.688497	2.708774	2.655972
17438022	Gabrb1	gamma-aminobutyric acid (GABA) A receptor, subunit beta 1	2.963989	2.903917	2.944392	2.576192	2.676003	2.734638
17248539	Gabrb2	gamma-aminobutyric acid (GABA) A receptor, subunit beta 2	3.881408	3.406925	2.866559	4.137301	3.406925	2.902769
17478730	Gabrb3	gamma-aminobutyric acid (GABA) A receptor, subunit beta 3	18.8841	20.93623	16.3225	14.54509	13.82723	18.8841
17433787	Gabrd	gamma-aminobutyric acid (GABA) A receptor, subunit delta	5.275983	4.778275	4.647678	4.620792	4.662423	5.012812
17542194	Gabre	gamma-aminobutyric acid (GABA) A receptor, subunit epsilon	3.651426	4.047946	2.847192	3.582086	4.33175	3.482202
17448565	Gabrg1	gamma-aminobutyric acid (GABA) A receptor, subunit gamma 1	3.927578	3.00526	3.099037	3.150367	3.439717	2.750226
17261878	Gabrg2	gamma-aminobutyric acid (GABA) A receptor, subunit gamma 2	3.839717	3.48184	3.386887	3.107275	4.239929	3.272493
17491463	Gabrg3	gamma-aminobutyric acid (GABA) A receptor, subunit gamma 3	4.20927	3.942498	4.436586	4.20927	4.352065	3.931064
17535420	Gabrq	gamma-aminobutyric acid (GABA) A receptor, subunit theta	3.062691	3.062691	2.85684	2.951237	2.790183	4.20927
17463550	Gabarapl1	gamma-aminobutyric acid (GABA) A receptor-associated protein-like 1	374.9618	470.2552	461.7026	401.8296	400.4893	470.102
17505774	Gabarapl2	gamma-aminobutyric acid (GABA) A receptor-associated protein-like 2	61.89925	48.35992	46.30192	54.5936	58.66996	49.59387
17337513	Gabbr1	gamma-aminobutyric acid (GABA) B receptor, 1	3.923986	6.669368	8.841799	6.337263	6.528674	8.81738
17425095	Gabbr2	gamma-aminobutyric acid (GABA) B receptor, 2	56.51297	53.0485	69.4749	50.69284	72.82894	66.75667
17412579	Gabrr1	gamma-aminobutyric acid (GABA) C receptor, subunit rho 1	3.030845	3.372761	3.956542	3.652059	3.238779	3.372761
17412563	Gabrr2	gamma-aminobutyric acid (GABA) C receptor, subunit rho 2	5.724303	6.813188	5.724303	5.755256	6.046918	5.724303
17326453	Gabrr3	gamma-aminobutyric acid (GABA) receptor, rho 3	3.480996	3.146788	3.603678	3.014189	3.380766	3.425798
17251855	Gabarap	gamma-aminobutyric acid receptor associated protein	536.1749	605.6666	648.2336	523.5197	611.7931	573.0471
17459634	Ggcx	gamma-glutamyl carboxylase	188.1605	198.1364	203.8325	178.2799	155.1554	141.9609
17467031	Ggct	gamma-glutamyl cyclotransferase	12.41722	17.46264	18.66133	17.88378	18.06506	18.38886
17412102	Ggh	gamma-glutamyl hydrolase	18.31673	25.26083	20.9615	18.63341	18.38542	17.69684
17309688	Ggact	gamma-glutamylamine cyclotransferase	190.5017	152.3843	119.1554	141.1387	155.0318	115.7293
17234373	Ggt1	gamma-glutamyltransferase 1	5.06587	5.587218	5.617429	5.06587	5.159639	4.686887
17234403	Ggt5	gamma-glutamyltransferase 5	5.172099	6.65436	6.827086	7.733224	5.995832	6.943341
17252359	Ggt6	gamma-glutamyltransferase 6	25.81558	27.48266	38.14019	30.31164	34.48674	29.76118
17393264	Ggt7	gamma-glutamyltransferase 7	6.056902	6.763687	5.267798	6.460877	5.304843	5.883767
17435055	Gsap	gamma-secretase activating protein	36.43821	39.44006	36.65073	43.19463	50.54617	46.67118
17379247	Gdap1l1	ganglioside-induced differentiation-associated protein 1-like 1	11.34001	11.42443	10.34793	10.81236	9.137947	10.57063
17211294	Gdap1	ganglioside-induced differentiation-associated-protein 1	3.22867	3.009972	3.197823	2.832979	2.647077	3.533334
17274889	Gdap10	ganglioside-induced differentiation-associated-protein 10	21.18366	25.76981	24.27131	18.08912	26.46291	20.79291
17400966	Gdap2	ganglioside-induced differentiation-associated-protein 2	89.76369	59.56777	68.28468	70.86985	75.62703	52.05446
17233347	Gja1	gap junction protein, alpha 1	19.04262	20.45994	14.73965	19.5636	9.193091	13.85361
17423750	Gja10	gap junction protein, alpha 10	2.539756	2.580105	2.330582	2.84968	2.561751	2.823002
17307113	Gja3	gap junction protein, alpha 3	4.333912	5.745052	4.952957	5.006526	5.184015	4.735412
17430211	Gja4	gap junction protein, alpha 4	11.92916	13.25477	12.93824	12.32264	12.75517	15.43764
17400749	Gja5	gap junction protein, alpha 5	5.338965	5.338965	5.338965	5.338965	6.458683	4.870804
17545809	Gja6	gap junction protein, alpha 6	3.15745	3.193415	3.801582	3.149035	3.194987	2.791809
17408156	Gja8	gap junction protein, alpha 8	2.907219	4.513041	4.641625	4.252408	3.387099	4.15999
17536736	Gjb1	gap junction protein, beta 1	129.5037	122.9127	116.8521	127.7332	144.0884	126.1564

17307117	Gjb2	gap junction protein, beta 2	214.3924	233.188	252.9643	208.4056	242.3738	200.6754
17430216	Gjb3	gap junction protein, beta 3	3.120052	4.040069	3.798974	4.217096	4.203118	4.293101
17430222	Gjb4	gap junction protein, beta 4	7.579906	7.229871	6.8385	7.674432	7.09784	9.20259
17430227	Gjb5	gap junction protein, beta 5	4.32461	5.758368	3.801714	4.287837	4.402796	4.251317
17307125	Gjb6	gap junction protein, beta 6	4.50529	3.883991	3.822377	3.4291	4.353694	3.702886
17389505	Gjd2	gap junction protein, delta 2	4.681043	4.460236	4.681043	4.681043	5.323297	5.226807
17268907	Gjd3	gap junction protein, delta 3	17.58362	24.25769	20.80358	27.25256	24.14379	21.87544
17352575	Gjd4	gap junction protein, delta 4	3.062691	3.904912	3.703322	3.930438	3.703322	3.703322
17239474	Gje1	gap junction protein, epsilon 1	2.839329	2.753373	3.562278	3.488967	2.98974	3.562031
17270315	Gjc1	gap junction protein, gamma 1	4.552819	4.296435	4.296435	4.296435	4.571634	4.65519
17263355	Gjc2	gap junction protein, gamma 2	6.63189	6.672743	6.970683	6.672743	5.758807	6.672743
17454209	Gjc3	gap junction protein, gamma 3	3.748387	4.866518	7.567937	4.92021	5.561947	5.235764
17410323	Gar1	GAR1 ribonucleoprotein homolog (yeast)	24.23836	19.23911	23.53751	26.44036	21.96052	20.96339
17419483	Fgr	Gardner-Rasheed feline sarcoma viral (Fgr) oncogene homolog	7.856191	7.367065	8.959076	6.845519	8.201854	7.000122
17256101	Gsdma	gasdermin A	5.782206	5.648744	9.667682	7.496307	4.009744	5.133455
17256082	Gsdma2	gasdermin A2	4.299116	2.999891	4.993288	4.515701	3.230998	3.385878
17256069	Gsdma3	gasdermin A3	2.489541	2.737464	2.661372	2.304206	2.49322	3.477547
17317428	Gsdmc	gasdermin C	2.746378	2.787651	3.084913	2.225917	3.153032	2.620678
17317442	Gsdmc2	gasdermin C2	2.133852	2.342875	2.365524	2.041925	2.120214	2.016929
17317458	Gsdmc3	gasdermin C3	2.622731	2.328515	2.603821	3.61153	1.794535	3.143496
17317472	Gsdmc4	gasdermin C4	2.677506	1.857253	2.481049	3.286678	3.263862	2.003274
17311878	Gsdmcl1	gasdermin C-like 1	13.16632	16.68604	13.32118	13.54746	16.55081	9.783835
17311885	Gsdmcl2	gasdermin C-like 2	5.506631	10.54794	8.469225	6.070057	11.9175	9.633767
17311893	Gsdmcl-ps	gasdermin C-like, pseudogene	3.978873	3.424801	3.98373	4.033633	2.645518	2.877109
17312280	Gsdmd	gasdermin D	33.22414	35.79336	34.5468	38.5674	29.64034	28.73844
17311874	1700065I16Rik	gasdermin pseudogene	2.572141	2.10643	2.528567	2.304206	2.467517	2.96403
17255487	Gip	gastric inhibitory polypeptide	22.62789	31.71361	26.49448	27.95472	26.49448	35.01133
17487170	Gipr	gastric inhibitory polypeptide receptor	9.055285	9.018828	7.94843	9.574983	10.2556	10.29313
17357729	Gif	gastric intrinsic factor	5.065027	6.13405	5.437411	6.000114	5.996497	6.093875
17256357	Gast	gastrin	21.74919	22.29456	27.29283	22.29456	22.29456	23.65691
17351396	Grp	gastrin releasing peptide	7.088498	8.016319	6.997939	8.538603	6.793335	7.450411
17545910	Grpr	gastrin releasing peptide receptor	3.434095	4.141978	3.54956	3.498121	3.248334	3.813379
17468594	Gkn1	gastrokine 1	8.195659	10.51728	8.38796	8.846274	9.504034	8.120058
17460538	Gkn2	gastrokine 2	4.953678	4.938216	5.777919	4.722497	4.419153	5.308484
17468602	Gkn3	gastrokine 3	3.805853	4.20927	3.990418	4.20927	4.844608	4.688154
17446213	Gbx1	gastrulation brain homeobox 1	4.457114	3.724689	4.20927	3.460331	4.116191	3.572614
17225373	Gbx2	gastrulation brain homeobox 2	9.361307	8.559165	11.19397	11.37678	9.141241	8.863829
17539966	Gata1	GATA binding protein 1	7.808687	6.898965	6.182713	6.592977	6.319673	6.314156
17460634	Gata2	GATA binding protein 2	5.65556	5.255981	6.484521	6.945459	6.537867	5.56106
17381533	Gata3	GATA binding protein 3	16.56447	12.2343	12.53708	14.67278	12.53708	11.72715
17307609	Gata4	GATA binding protein 4	35.87135	34.66697	30.11541	33.58558	25.86609	31.46552
17395629	Gata5	GATA binding protein 5	4.56043	5.451261	6.114735	5.061377	5.719781	5.145281
17380749	Gata5os	GATA binding protein 5, opposite strand	7.137308	8.785169	8.623479	8.567237	7.798086	9.801619
17348400	Gata6	GATA binding protein 6	29.34332	36.94891	32.39771	32.39771	30.16555	36.41927
17445268	Gatad1	GATA zinc finger domain containing 1	166.6722	155.8722	150.7224	149.8162	159.4323	158.4792
17509907	Gatad2a	GATA zinc finger domain containing 2A	96.72521	91.60154	89.42151	92.47875	73.48154	79.88928
17399737	Gatad2b	GATA zinc finger domain containing 2B	106.2059	125.4353	135.139	105.6149	115.4657	121.8202
17443069	Gatsl2	GATS protein-like 2	18.06218	19.29533	23.8279	19.29533	18.76731	15.89995
17246790	Gatsl3	GATS protein-like 3	9.340437	9.472925	10.27317	10.13996	8.260734	10.59873
17441202	Gcn1l1	GCN1 general control of amino-acid synthesis 1-like 1 (yeast)	42.56041	38.19521	41.98203	44.45396	44.45396	41.43305
17296142	Gpbp1	GC-rich promoter binding protein 1	115.3281	102.0491	89.48103	93.25635	91.52348	104.6704
17417437	Gpbp1l1	GC-rich promoter binding protein 1-like 1	75.42286	91.72417	83.50843	87.97795	91.11268	94.4874
17459801	Gcfc2	GC-rich sequence DNA binding factor 2	8.547604	8.009654	7.491995	9.597975	11.99266	8.56694
17528902	Gfral	GDNF family receptor alpha like	3.053132	2.682039	2.470974	3.339611	2.905004	3.215226
17377360	Gzf1	GDNF-inducible zinc finger protein 1	39.56108	45.08045	42.81249	37.80984	44.89802	53.36049
17479516	Gdpgp1	GDP-D-glucose phosphorylase 1	13.91839	11.80233	12.23193	16.44697	11.61462	11.79791
17291653	Gmds	GDP-mannose 4, 6-dehydratase	10.6005	10.3868	12.74863	10.02041	7.855374	9.674586
17214543	Gmppa	GDP-mannose pyrophosphorylase A	69.92323	57.40323	59.72612	57.09371	52.74199	45.99069
17521603	Gmppb	GDP-mannose pyrophosphorylase B	21.35645	10.07508	11.59604	15.74269	14.20542	12.58166
17370209	Gsn	gelsolin	37.56169	35.88179	33.19836	33.35659	30.56122	34.55853
17275689	Gemin2	gem (nuclear organelle) associated protein 2	10.65486	9.263453	9.094095	9.736478	9.840625	11.08295
17266074	Gemin4	gem (nuclear organelle) associated protein 4	28.01699	27.18672	28.57477	28.59875	28.70341	28.70301
17263089	Gemin5	gem (nuclear organelle) associated protein 5	35.30572	29.78492	29.74963	34.47216	27.33145	32.74131
17339928	Gemin6	gem (nuclear organelle) associated protein 6	5.930649	5.031121	6.453447	8.623539	5.979729	6.672003
17487291	Gemin7	gem (nuclear organelle) associated protein 7	17.09864	15.17001	17.49172	15.25775	18.6453	14.5692
17539600	Gemin8	gem (nuclear organelle) associated protein 8	2.699646	2.661335	2.661335	2.475552	2.906595	3.148839
17539099	4930503H13Rik	gem (nuclear organelle) associated protein 8 pseudogene	6.010729	3.613633	4.185471	3.458101	3.743584	3.724586
17316185	LOC105245899	gem-associated protein 4-like	3.836923	3.347212	4.247135	3.706686	3.697295	3.677384
17291343	Gmnn	geminin	10.50162	9.15481	6.855301	9.951679	9.924126	10.35848
17329500	Gmnc	geminin coiled-coil domain containing	6.037076	4.893172	5.608403	5.079372	5.783008	6.550704
17501721	Gmip	Gem-interacting protein	7.922908	9.894385	8.523051	8.523051	7.980394	9.310251
17279962	Gen1	Gen homolog 1, endonuclease (Drosophila)	4.016392	3.721773	4.172176	4.088354	3.892319	4.880808
17280086	Greb1	gene regulated by estrogen in breast cancer protein	4.20927	4.20927	4.670348	4.068678	4.349381	4.046768
17470820	Grcc10	gene rich cluster, C10 gene	46.75958	49.93536	49.36101	46.93851	50.08407	41.85796
17469684	Gt(ROSA)26Sor	gene trap ROSA 26, Philippe Soriano	18.84657	17.64833	18.87441	20.56701	16.68708	19.8334
17283014	Gtf2a1	general transcription factor II A, 1	80.29398	77.51803	74.74421	81.54079	84.90808	86.98049
17519009	Gtf2a2	general transcription factor II A, 2	40.65836	45.62776	52.09453	49.46067	57.14598	55.27135
17330306	Gtf2e1	general transcription factor II E, polypeptide 1 (alpha subunit)	29.6313	26.45448	21.51051	32.60949	23.00762	30.9801
17500496	Gtf2e2	general transcription factor II E, polypeptide 2 (beta subunit)	30.40781	31.08529	27.79378	25.68742	34.35147	26.66342
17478201	Gtf2h1	general transcription factor II H, polypeptide 1	71.89203	69.49465	69.49465	67.08229	67.07624	61.36482
17295646	Gtf2h2	general transcription factor II H, polypeptide 2	6.171709	5.69478	6.004733	6.27763	6.214636	6.543851
17295649	Gtf2h2	general transcription factor II H, polypeptide 2	22.53976	19.61316	20.20555	24.25029	22.93009	25.42825
17344387	Gtf2h4	general transcription factor II H, polypeptide 4	19.42873	20.17505	19.42873	19.42873	24.17259	17.05615
17453304	Gtf2i	general transcription factor II I	102.5803	107.8491	111.5425	100.2348	95.51994	90.55359

17453347	Gtf2ird1	general transcription factor II I repeat domain-containing 1	34.24663	33.70428	30.12439	32.10264	25.73126	26.37648
17340366	Gtf2a1l	general transcription factor IIA, 1-like	5.338965	6.231373	5.690321	6.048553	4.829621	3.978294
17403301	Gtf2b	general transcription factor IIB	64.86058	64.74694	60.16906	62.67986	73.45252	57.66524
17346412	Gtf2f1	general transcription factor IIF, polypeptide 1	77.49385	56.93919	65.01001	52.85324	53.96417	42.07088
17308734	Gtf2f2	general transcription factor IIF, polypeptide 2	39.45866	21.2603	24.12555	28.4923	32.53205	23.29454
17442553	Gtf2h3	general transcription factor IIH, polypeptide 3	37.01146	28.24845	31.90034	34.72277	40.23781	28.66285
17332851	Gtf2h5	general transcription factor IIH, polypeptide 5	72.98054	77.35271	65.02849	67.70263	71.95435	71.45485
17444813	Gtf3a	general transcription factor III A	40.32129	37.99032	42.29455	42.26408	48.97997	43.25275
17496063	Gtf3c1	general transcription factor III C 1	80.96632	76.8722	80.69459	83.87913	84.8969	70.5718
17446853	Gtf3c2	general transcription factor IIIC, polypeptide 2, beta	64.06169	79.20832	69.37192	66.11474	72.5649	68.40501
17223117	Gtf3c3	general transcription factor IIIC, polypeptide 3	39.89456	31.09455	30.68603	35.43541	31.50044	37.70856
17383430	Gtf3c4	general transcription factor IIIC, polypeptide 4	27.94387	28.32472	28.21851	28.21851	31.27049	29.50321
17383395	Gtf3c5	general transcription factor IIIC, polypeptide 5	8.302351	10.42915	10.29392	10.92211	10.52049	10.92181
17240357	Gtf3c6	general transcription factor IIIC, polypeptide 6, alpha	45.53424	36.20809	29.22599	34.13098	36.20809	36.20809
17506249	Gse1	genetic suppressor element 1	23.68087	32.33512	32.33512	34.38578	31.96099	31.40081
17276674	Gphn	gephyrin	192.0143	159.366	144.9217	203.5952	154.0847	194.5275
17290729	Ggps1	geranylgeranyl diphosphate synthase 1	18.67	27.86283	21.24807	24.82032	20.55033	22.17066
17468532	Gmcl1	germ cell-less homolog 1 (Drosophila)	27.17974	28.12751	22.73054	27.65196	28.16614	25.32675
17533939	Gmcl1l	germ cell-less homolog 1 (Drosophila)-like	32.23844	32.49014	26.52517	26.99013	35.97867	43.53096
17532718	Gm5925	germ cell-less homolog 1 family pseudogene	5.690873	5.593417	4.486001	5.735543	6.478636	5.469542
17472073	Gsg1	germ cell-specific gene 1	5.82307	4.844138	4.844138	5.455192	5.781485	4.844138
17265724	Gsg2	germ cell-specific gene 2	4.040461	3.237006	4.647678	4.389236	3.729055	3.192043
17325979	Gcsam	germinal center associated, signaling and motility	6.918216	5.101212	4.937669	4.801649	4.811144	3.579058
17507559	Grtp1	GH regulated TBC protein 1	35.83382	34.56907	31.54764	31.6109	34.31815	39.5844
17269679	Ghdc	GH3 domain containing	37.46548	34.62783	34.62783	38.0789	31.88111	33.75385
17469797	Ghrl	ghrelin	5.611825	7.698994	6.532567	6.921429	6.371796	8.866963
17505953	Gan	giant axonal neuropathy	12.70117	18.54065	14.54539	17.14933	14.68692	13.65891
17548650	Gan	giant axonal neuropathy	7.791548	10.04371	12.41223	11.27957	9.403579	10.05841
17250365	Gid4	GID complex subunit 4, VID24 homolog (S. cerevisiae)	60.99377	43.40713	41.41496	53.70223	50.80082	61.82421
17380840	Gid8	GID complex subunit 8 homolog (S. cerevisiae)	32.18909	40.07332	41.94625	37.45977	43.42277	46.49456
17402705	Gimd1	GIMAP family P-loop NTPase domain containing 1	9.64312	9.078349	9.259601	6.270628	8.061454	9.243121
17377525	Gins1	GINS complex subunit 1 (Psf1 homolog)	3.462322	4.31829	4.31829	4.220225	5.222027	4.077769
17513525	Gins2	GINS complex subunit 2 (Psf2 homolog)	9.065019	10.38162	6.818716	9.461638	9.286404	9.286404
17504321	Gins3	GINS complex subunit 3 (Psf3 homolog)	11.80315	11.67945	11.87273	8.833223	9.293229	11.84577
17508151	Gins4	GINS complex subunit 4 (Sld5 homolog)	35.45581	36.02783	31.38232	39.04403	36.8318	39.71632
17502962	Gipc1	GIPC PDZ domain containing family, member 1	35.15237	30.21996	41.37794	44.03412	40.16119	47.52616
17411174	Gipc2	GIPC PDZ domain containing family, member 2	5.599197	5.901329	6.204694	6.687097	5.705012	5.191639
17243278	Gipc3	GIPC PDZ domain containing family, member 3	6.378026	7.212803	10.00778	7.802249	8.631014	8.631014
17368981	Gle1	GLE1 RNA export mediator (yeast)	92.76058	99.57972	107.9081	96.49752	94.87	93.80936
17537144	4933401B06Rik	GLE1 RNA export mediator pseudogene	2.324435	2.711535	2.969891	3.076031	4.008271	3.002096
17244949	Glipr1	GLI pathogenesis-related 1 (glioma)	5.780603	5.780603	5.780603	5.859794	6.954998	5.368355
17237298	Glipr1l1	GLI pathogenesis-related 1 like 1	3.091142	3.973416	4.725674	3.652439	3.245026	4.138879
17237305	Glipr1l2	GLI pathogenesis-related 1 like 2	2.671389	2.803307	3.110378	2.375646	2.803307	2.750626
17244961	Glipr1l3	GLI pathogenesis-related 1 like 3	2.350732	2.341106	2.056327	1.980878	1.998199	2.547037
17413500	Glipr2	GLI pathogenesis-related 2	6.6278	5.337041	4.372566	4.82942	3.996896	5.609919
17305727	Gmfb	glia maturation factor, beta	69.81766	69.54573	68.11544	65.29542	51.94849	59.59296
17475839	Gmfg	glia maturation factor, gamma	12.18074	10.54794	9.731148	13.05509	7.553839	7.943418
17310072	Gdnf	glial cell line derived neurotrophic factor	4.955017	5.306351	4.770201	4.370142	5.527434	3.883318
17365960	Gfra1	glial cell line derived neurotrophic factor family receptor alpha 1	229.4554	226.535	237.7432	245.5563	276.3265	204.787
17301823	Gfra2	glial cell line derived neurotrophic factor family receptor alpha 2	15.28622	16.61506	24.78714	12.46439	8.673414	12.47052
17353509	Gfra3	glial cell line derived neurotrophic factor family receptor alpha 3	5.061623	4.977806	5.220182	4.897549	4.450724	4.987338
17391792	Gfra4	glial cell line derived neurotrophic factor family receptor alpha 4	8.329327	7.785556	9.78241	8.152034	7.839491	8.498098
17519610	Gcm1	glial cells missing homolog 1 (Drosophila)	4.144419	3.732184	4.312098	4.123044	3.476535	4.230652
17292050	Gcm2	glial cells missing homolog 2 (Drosophila)	8.123323	6.11554	5.617429	7.337202	5.370029	6.020069
17270354	Gfap	glial fibrillary acidic protein	11.6463	12.43591	10.27139	8.827837	9.741473	11.49975
17245874	Gli1	GLI-Kruppel family member GLI1	7.356655	9.838716	9.389055	8.418423	8.606818	8.328577
17226170	Gli2	GLI-Kruppel family member GLI2	4.395204	6.287864	5.48933	5.621636	8.004049	5.52958
17285414	Gli3	GLI-Kruppel family member GLI3	3.755825	4.079324	3.339703	3.703322	5.120306	4.959381
17442909	Gbas	glioblastoma amplified sequence	49.72502	42.87932	51.84777	47.09461	35.50598	47.56307
17486818	Gltscr1	glioma tumor suppressor candidate region gene 1	11.21859	11.95656	11.56249	12.96302	7.659393	8.515257
17486790	Gltscr2	glioma tumor suppressor candidate region gene 2	65.29542	68.31971	66.39902	65.91066	77.13587	66.32634
17517368	Gldn	gliomedin	12.84994	10.16064	9.101157	13.40686	11.12297	8.58162
17527206	Gldnos	gliomedin, opposite strand	3.37854	3.974601	3.708408	3.73459	3.192839	3.777731
17416506	Glis1	GLIS family zinc finger 1	6.231805	8.582572	6.027042	6.887212	9.270647	8.331291
17322525	Glis2	GLIS family zinc finger 2	10.4974	12.02596	12.21439	14.07829	12.39959	11.58969
17363727	Glis3	GLIS family zinc finger 3	4.488552	4.972116	4.309708	6.486994	4.34188	4.86861
17368674	Gbgt1	globoside alpha-1,3-N-acetylgalactosaminyltransferase 1	15.63124	15.62073	14.6744	12.63943	13.91395	19.18185
17450645	Glmn	glomulin, FKBP associated protein	5.669769	5.669769	5.77836	5.636033	6.234354	5.709206
17345593	Gltscr1l	GLTSCR1-like	33.15903	41.58784	45.89452	34.35909	37.06203	39.62777
17385757	Gcg	glucagon	4.320625	6.116007	7.47892	4.903086	6.017107	6.041807
17259401	Gcgr	glucagon receptor	108.7597	116.81	123.1276	131.5147	136.7607	130.5004
17335726	Glp1r	glucagon-like peptide 1 receptor	8.673114	9.647265	10.28472	9.651613	9.647265	8.978348
17264342	Glp2r	glucagon-like peptide 2 receptor	3.080255	2.914907	3.080255	3.753327	3.052476	3.35497
17326567	Gbe1	glucan (1,4-alpha-), branching enzyme 1	185.9189	171.1657	149.2906	153.6773	173.973	173.2474
17456001	Glcci1	glucocorticoid induced transcript 1	17.93406	21.66524	19.58653	19.56008	22.19573	18.33947
17430777	Gmeb1	glucocorticoid modulatory element binding protein 1	9.401624	12.99919	13.31573	13.5765	13.73029	10.06162
17395830	Gmeb2	glucocorticoid modulatory element binding protein 2	28.55517	40.78142	38.61046	41.12746	40.52134	38.78481
17260244	Gck	glucokinase	35.7242	32.69572	28.87182	31.09973	37.68974	38.73619
17436107	Gckr	glucokinase regulatory protein	149.3289	210.088	192.41	202.1959	241.3579	259.5089
17237715	Gns	glucosamine (N-acetyl)-6-sulfatase	350.7793	352.7715	352.505	304.5257	343.5342	311.3126
17424822	Gne	glucosamine (UDP-N-acetyl)-2-epimerase/N-acetylmannosamine kinase	183.4165	202.1973	198.0869	224.1561	213.6462	203.7251
17353948	Gnpda1	glucosamine-6-phosphate deaminase 1	43.3536	32.58396	38.10425	36.45943	23.9161	39.6888
17355665	Gm2176	glucosamine-6-phosphate deaminase 1 pseudogene	4.576929	4.898873	4.223708	4.651706	4.576929	4.20927
17455492	Gm8615	glucosamine-6-phosphate deaminase 1 pseudogene	4.212539	5.108359	5.005347	4.106844	3.263862	4.392493

17448543	Gnpda2	glucosamine-6-phosphate deaminase 2	4.163798	5.300432	4.438032	5.803527	5.300432	5.480054
17305653	Gnpnat1	glucosamine-phosphate N-acetyltransferase 1	29.29454	36.52899	32.05706	36.49457	39.8023	43.34068
17363320	Gcnt1	glucosaminyl (N-acetyl) transferase 1, core 2	5.338965	5.349893	5.626041	3.947475	6.556791	5.787659
17286707	Gcnt2	glucosaminyl (N-acetyl) transferase 2, I-branching enzyme	24.74748	33.49445	32.55709	33.30692	30.51147	30.5627
17528562	Gcnt3	glucosaminyl (N-acetyl) transferase 3, mucin type	5.034784	4.237138	4.249255	4.184195	4.837226	3.160231
17289324	Gcnt4	glucosaminyl (N-acetyl) transferase 4, core 2 (beta-1,6-N-acetylglucosaminyltransf	21.20923	19.89177	17.20541	19.43197	15.76354	17.12296
17395032	Gcnt7	glucosaminyl (N-acetyl) transferase family member 7	12.87848	10.7766	12.28665	11.91569	11.78753	11.60408
17256896	G6pc3	glucose 6 phosphatase, catalytic, 3	8.497391	15.64392	16.69055	13.53375	14.57324	17.18217
17489620	Gpi1	glucose phosphate isomerase 1	438.3532	414.9403	520.3577	392.3507	461.453	410.2644
17256707	G6pc	glucose-6-phosphatase, catalytic	1026.516	1090.875	648.764	927.2535	757.2574	914.2406
17371410	G6pc2	glucose-6-phosphatase, catalytic, 2	3.380766	3.787485	3.846457	3.886361	3.729055	2.897282
17437570	G6pd2	glucose-6-phosphate dehydrogenase 2	3.229364	3.99903	3.741508	3.611204	3.635264	4.942497
17542695	G6pdx	glucose-6-phosphate dehydrogenase X-linked	10.57342	16.07314	12.53708	11.50086	10.66299	15.96732
17292117	Gfod1	glucose-fructose oxidoreductase domain containing 1	10.77384	7.728026	7.541544	7.876747	6.515564	7.48312
17512502	Gfod2	glucose-fructose oxidoreductase domain containing 2	11.26878	11.58688	13.09505	12.17762	9.405144	12.2904
17424742	Gba2	glucosidase beta 2	14.63763	18.40901	15.25288	18.68813	17.50166	21.79069
17258994	Gaa	glucosidase, alpha, acid	104.4277	121.985	117.8477	102.38	93.44205	94.50901
17375006	Ganc	glucosidase, alpha; neutral C	30.49329	32.01797	37.95374	39.19207	35.05212	43.44355
17399333	Gba	glucosidase, beta, acid	52.99521	47.60727	50.95317	44.78364	42.69455	38.65063
17320693	Gxylt1	glucoside xylosyltransferase 1	12.78624	13.78206	10.07432	15.43239	15.86186	17.83835
17461166	Gxylt2	glucoside xylosyltransferase 2	13.33328	10.46398	10.25532	11.60987	9.821273	10.33209
17453160	Gusb	glucuronidase, beta	47.69546	43.89851	46.38704	42.65284	45.04047	39.01643
17527977	Glce	glucuronyl C5-epimerase	63.96097	69.45131	62.16971	61.51004	63.96097	56.21565
17371636	Gad1	glutamate decarboxylase 1	9.593385	11.44361	6.287864	9.299544	11.17211	13.13715
17386340	Gad1os	glutamate decarboxylase 1, opposite strand	14.51658	13.15884	12.75526	14.82253	11.51115	19.45555
17236892	Gad1-ps	glutamate decarboxylase 1, pseudogene [Source:MGI Symbol;Acc:MGI:95633]	3.661767	3.094551	3.300123	3.330687	2.672407	3.630731
17522818	Gadl1	glutamate decarboxylase-like 1	11.53311	12.73812	9.341861	13.88437	10.0266	11.24366
17298805	Glud1	glutamate dehydrogenase 1	1592.602	1464.152	1297.051	1239.326	1459.795	1399.921
17237608	Grip1	glutamate receptor interacting protein 1	12.11162	12.03955	12.11162	13.00694	12.45083	12.17838
17245374	Grip1os1	glutamate receptor interacting protein 1, opposite strand 1	4.360822	5.212878	4.610618	4.816349	4.172552	5.785614
17245370	Grip1os2	glutamate receptor interacting protein 1, opposite strand 2	6.801203	7.885925	7.667945	7.237693	7.935109	8.262567
17469059	Grip2	glutamate receptor interacting protein 2	9.659711	9.053025	10.1567	9.606827	10.30448	9.905227
17425255	Grin3a	glutamate receptor ionotropic, NMDA3A	3.733555	3.720664	3.720664	3.397162	5.467268	3.578562
17249863	Gria1	glutamate receptor, ionotropic, AMPA1 (alpha 1)	4.872155	5.173892	5.091639	5.747721	5.72589	6.347815
17406145	Gria2	glutamate receptor, ionotropic, AMPA2 (alpha 2)	4.081615	3.556332	3.703322	3.723373	3.870634	3.729055
17534385	Gria3	glutamate receptor, ionotropic, AMPA3 (alpha 3)	6.345835	7.445558	8.642867	6.577002	8.702923	7.774662
17523705	Gria4	glutamate receptor, ionotropic, AMPA4 (alpha 4)	4.420992	3.843392	3.913908	3.849337	3.843392	3.706275
17298874	Grid1	glutamate receptor, ionotropic, delta 1	4.408995	3.939848	4.522906	4.20927	5.652817	3.626631
17459126	Grid2	glutamate receptor, ionotropic, delta 2	3.071748	2.908691	2.774043	2.924804	3.260922	3.586533
17444309	Grid2ip	glutamate receptor, ionotropic, delta 2 (Grid2) interacting protein 1	4.75462	6.7788	5.404084	6.510959	5.565649	5.638377
17331802	Grik1	glutamate receptor, ionotropic, kainate 1	3.585017	3.382336	3.083716	3.662884	2.981276	3.1065
17240693	Grik2	glutamate receptor, ionotropic, kainate 2 (beta 2)	4.45094	4.985092	4.266846	4.520712	4.378662	4.514136
17418465	Grik3	glutamate receptor, ionotropic, kainate 3	5.549008	5.647295	4.691405	4.424026	5.115694	5.66643
17526008	Grik4	glutamate receptor, ionotropic, kainate 4	6.639893	6.261985	6.31385	6.200566	5.976704	6.678712
17487833	Grik5	glutamate receptor, ionotropic, kainate 5 (gamma 2)	17.49135	20.10399	22.2663	19.52066	19.62866	21.44204
17382496	Grin1	glutamate receptor, ionotropic, NMDA1 (zeta 1)	7.975251	8.700872	10.03251	11.03313	9.715175	11.88047
17367921	Grin1os	glutamate receptor, ionotropic, NMDA1 (zeta 1), opposite strand	8.524292	7.935109	7.990579	7.935109	7.935109	9.310251
17328046	Grin2a	glutamate receptor, ionotropic, NMDA2A (epsilon 1)	4.509289	5.163359	5.486401	5.667097	5.44688	5.466775
17472089	Grin2b	glutamate receptor, ionotropic, NMDA2B (epsilon 2)	6.218428	4.555912	4.471132	4.255003	4.067719	4.44308
17271803	Grin2c	glutamate receptor, ionotropic, NMDA2C (epsilon 3)	8.53109	7.199466	7.325717	6.707755	5.976786	6.378115
17490995	Grin2d	glutamate receptor, ionotropic, NMDA2D (epsilon 4)	4.432621	6.799459	6.799459	6.439149	6.318578	8.125069
17235065	Grin3b	glutamate receptor, ionotropic, NMDA3B	8.438637	10.50402	12.04389	11.00029	6.743091	9.649405
17312341	Grina	glutamate receptor, ionotropic, N-methyl D-aspartate-associated protein 1 (glutan	274.3607	322.0029	360.7236	297.1299	204.8338	200.0033
17239234	Grm1	glutamate receptor, metabotropic 1	5.613848	4.801649	4.006715	4.579944	4.96337	5.144568
17530733	Grm2	glutamate receptor, metabotropic 2	6.091173	6.070393	5.837983	5.51022	5.335376	6.347815
17445596	Grm3	glutamate receptor, metabotropic 3	3.432287	4.06214	3.064072	3.39469	2.95046	2.809941
17342719	Grm4	glutamate receptor, metabotropic 4	9.364682	7.232528	8.78663	7.212953	5.345889	5.803246
17480002	Grm5	glutamate receptor, metabotropic 5	2.807722	4.015139	3.287475	2.91317	3.54091	3.143583
17249234	Grm6	glutamate receptor, metabotropic 6	7.690887	7.438285	7.225262	6.043733	7.082752	7.672092
17461448	Grm7	glutamate receptor, metabotropic 7	4.230241	4.23382	4.0443	3.523282	4.664718	3.656542
17465272	Grm8	glutamate receptor, metabotropic 8	3.05989	2.946618	2.989781	3.255412	2.989781	3.104412
17507682	Erich1	glutamate rich 1	12.77667	15.13273	15.37016	18.26171	13.54623	16.19493
17371621	Erich2	glutamate rich 2	3.520011	3.54506	3.520011	4.522875	4.501544	3.431692
17386344	Erich2os	glutamate rich 2, opposite strand [Source:MGI Symbol;Acc:MGI:3826593]	3.781897	3.274217	3.140643	3.274739	3.140643	4.306721
17403790	Erich3	glutamate rich 3	5.149385	5.723152	6.958084	5.515685	5.247317	5.477699
17403806	Erich3	glutamate rich 3	3.364962	3.479814	3.708305	3.908811	3.863799	4.209941
17487977	Erich4	glutamate rich 4	3.951965	3.506253	4.088014	5.069839	4.352065	3.76923
17310944	Erich5	glutamate rich 5	14.66576	16.80874	15.70988	17.07898	17.05213	16.80874
17405420	Erich6	glutamate rich 6	2.780723	3.362397	3.362397	3.362397	3.19326	4.245457
17302035	Erich6b	glutamate rich 6B	4.886969	4.886969	4.886969	4.23162	6.543216	5.304328
17218349	Glul	glutamate-ammonia ligase (glutamine synthetase)	1171.645	1186.659	1187.63	1329.336	1000.996	984.6656
17519579	Gclc	glutamate-cysteine ligase, catalytic subunit	762.5246	681.6371	467.7844	541.6436	468.4723	648.7101
17402283	Gclm	glutamate-cysteine ligase, modifier subunit	490.7961	461.789	401.5679	390.2054	360.4861	322.2843
17490984	Grwd1	glutamate-rich WD repeat containing 1	18.37	20.17714	18.592	19.14545	19.75396	17.29652
17512103	Got2	glutamatic-oxaloacetic transaminase 2, mitochondrial	1131.86	1140.183	1051.272	1075.32	1054.717	1095.33
17367510	Gad2	glutamic acid decarboxylase 2	5.313012	5.933363	5.768555	7.404385	6.385148	6.385148
17503446	Gpt2	glutamic pyruvate transaminase (alanine aminotransferase) 2	311.181	470.3041	521.0002	471.0806	512.7565	474.0154
17312567	Gpt	glutamic pyruvic transaminase, soluble	140.7323	160.7885	159.4566	187.3367	194.8351	251.1786
17364932	Got1	glutamic-oxaloacetic transaminase 1, soluble	288.3905	554.9138	645.7357	543.7426	635.3202	749.7106
17508532	Got1l1	glutamic-oxaloacetic transaminase 1-like 1	3.887439	4.129823	3.434095	3.692838	3.492597	3.355715
17222880	Gls	glutaminase	14.49567	16.18595	14.40912	11.67273	17.52084	15.70509
17238295	Gls2	glutaminase 2 (liver, mitochondrial)	322.8343	371.8482	445.691	443.5491	428.5115	501.6614
17388985	Qser1	glutamine and serine rich 1	48.96809	59.58676	53.73797	52.74199	56.47263	49.89073
17460506	Gfpt1	glutamine fructose-6-phosphate transaminase 1	78.87138	36.58119	45.00614	34.1653	45.04984	39.56766

17249036	Gfpt2	glutamine fructose-6-phosphate transaminase 2	4.92369	4.449675	5.10054	6.25435	5.10054	5.042328
17225356	Glrp1	glutamine repeat protein 1	2.674242	3.444561	2.567065	2.851043	3.918197	2.772121
17272375	Qrich2	glutamine rich 2	4.970324	5.301057	5.301057	5.627679	6.56434	5.301057
17521825	Qrich1	glutamine-rich 1	84.72113	89.96114	88.83461	85.62024	81.8948	80.17441
17339876	Qpct	glutaminyl-peptide cyclotransferase (glutaminyl cyclase)	33.34203	36.48749	41.95439	30.98397	27.70146	23.19336
17487161	Qpctl	glutaminyl-peptide cyclotransferase-like	26.55387	25.86053	25.86053	19.76382	20.56644	20.77203
17240608	Qrsl1	glutaminyl-tRNA synthase (glutamine-hydrolyzing)-like 1	12.23261	15.12769	11.86138	12.91065	13.51838	9.91938
17521794	Qars	glutaminyl-tRNA synthetase	68.72485	60.91772	52.89099	53.71191	55.09311	62.80772
17410251	Enpep	glutamyl aminopeptidase	150.9221	140.4731	142.9047	115.9565	135.7774	141.5276
17220548	Eprs	glutamyl-prolyl-tRNA synthetase	169.102	133.6726	134.7041	135.2064	168.3897	158.4013
17495909	Ears2	glutamyl-tRNA synthetase 2 (mitochondrial)(putative)	17.94239	21.13394	23.89472	19.24991	18.44503	21.73458
17398611	Gatb	glutamyl-tRNA(Gln) amidotransferase, subunit B	48.843	48.55305	46.59005	44.94504	43.90551	45.45415
17451721	Gatc	glutamyl-tRNA(Gln) amidotransferase, subunit C	194.7825	187.909	201.2633	177.0766	120.7308	117.3448
17288716	Glrx	glutaredoxin	104.3618	150.2238	208.5732	182.3088	118.1946	110.6961
17218013	Glrx2	glutaredoxin 2 (thioltransferase)	11.44266	10.29149	11.6317	11.50086	8.785291	10.94098
17484282	Glrx3	glutaredoxin 3	29.03478	33.24211	38.58264	35.74352	34.00583	49.52654
17427181	Gm12669	glutaredoxin 3 pseudogene	15.44374	10.79821	10.84968	10.45456	6.992896	8.092021
17278345	Glrx5	glutaredoxin 5 homolog (S. cerevisiae)	150.2801	179.6531	167.5851	185.2988	156.7314	157.9309
17437989	Grxcr1	glutaredoxin, cysteine rich 1	2.571161	2.611394	2.799676	2.588614	3.26047	2.770008
17354023	Grxcr2	glutaredoxin, cysteine rich 2	6.185971	6.419051	6.093242	6.169827	5.557014	7.238396
17511196	Gcdh	glutaryl-Coenzyme A dehydrogenase	746.8993	801.5452	783.8968	757.3572	718.5626	839.8803
17521681	Gpx1	glutathione peroxidase 1	1669.534	1801.309	1452.769	1740.927	1476.945	1589.944
17282069	Gpx2	glutathione peroxidase 2	4.089799	3.170829	4.085209	3.895558	3.765522	3.573406
17480673	Gpx2-ps1	glutathione peroxidase 2, pseudogene 1	3.000348	4.007937	3.767219	3.558428	3.017722	3.53674
17249787	Gpx3	glutathione peroxidase 3	17.53627	19.06296	30.63906	18.57023	12.9171	13.74419
17235166	Gpx4	glutathione peroxidase 4	101.1211	105.2415	106.9239	105.4401	110.216	104.3611
17290913	Gpx5	glutathione peroxidase 5	3.133641	3.688643	2.554711	3.339703	2.953468	3.281624
17285631	Gpx6	glutathione peroxidase 6	5.103015	5.28235	5.27613	7.275316	6.616279	13.95616
17428054	Gpx7	glutathione peroxidase 7	11.27441	11.34308	10.95358	11.34308	9.22603	10.36257
17296281	Gpx8	glutathione peroxidase 8 (putative)	11.06514	12.16066	10.72749	12.94201	11.93701	13.42723
17500478	Gsr	glutathione reductase	168.4165	178.4208	174.1564	161.2539	134.1804	143.9616
17457876	Gstk1	glutathione S-transferase kappa 1	545.4261	524.8386	457.7259	533.1915	446.9687	533.5538
17451839	Gstm2-ps1	glutathione S-transferase mu 2 (muscle), pseudogene 1	49.07819	52.79575	48.16656	43.93291	29.98814	45.29438
17360216	Gsto1	glutathione S-transferase omega 1	188.1435	203.5388	200.2488	203.6206	163.7068	196.78
17360225	Gsto2	glutathione S-transferase omega 2	3.4335	4.20927	3.218526	4.465526	3.936627	5.438203
17519658	Gsta1	glutathione S-transferase, alpha 1 (Ya)	47.0046	53.88941	38.48942	37.57419	26.05918	44.17322
17529017	Gsta2	glutathione S-transferase, alpha 2 (Yc2)	2.723648	3.110745	3.208169	2.967072	3.192419	3.179478
17211405	Gsta3	glutathione S-transferase, alpha 3	321.8289	391.1993	357.5621	361.7828	310.5389	385.426
17519649	Gsta4	glutathione S-transferase, alpha 4	210.4422	292.7043	230.6274	218.4577	117.6331	204.5841
17410455	Gstcd	glutathione S-transferase, C-terminal domain containing	6.707848	6.191075	7.073332	6.943774	6.72871	6.373253
17409154	Gstm1	glutathione S-transferase, mu 1	150.851	206.6793	207.5449	175.2244	140.8621	174.5032
17409142	Gstm2	glutathione S-transferase, mu 2	46.91411	51.7641	57.07472	52.09453	46.04492	56.36118
17409130	Gstm3	glutathione S-transferase, mu 3	97.1741	160.1112	183.2171	117.5418	85.22058	102.6152
17409172	Gstm4	glutathione S-transferase, mu 4	34.8269	38.96157	37.26269	44.73152	26.44403	34.90883
17401610	Gstm5	glutathione S-transferase, mu 5	19.41366	31.80166	29.18004	30.38253	29.32705	26.43193
17409113	Gstm6	glutathione S-transferase, mu 6	14.77207	14.90175	13.78264	13.37243	11.69768	12.54178
17409099	Gstm7	glutathione S-transferase, mu 7	59.34399	70.39544	64.41121	81.18662	72.13562	76.4275
17361073	Gstp1	glutathione S-transferase, pi 1	1361.999	1262.038	851.1089	1343.76	788.0045	701.5198
17361081	Gstp2	glutathione S-transferase, pi 2	1030.244	877.1015	608.8361	996.7653	538.8239	476.1459
17241934	Gstt1	glutathione S-transferase, theta 1	180.4268	221.5487	236.2204	218.1263	221.833	254.378
17241954	Gstt2	glutathione S-transferase, theta 2	67.50582	107.8573	94.22709	89.48413	71.42415	93.22403
17241921	Gstt3	glutathione S-transferase, theta 3	28.94455	41.77101	64.0102	44.19006	31.81025	46.19645
17241946	Gstt4	glutathione S-transferase, theta 4	3.501177	4.100046	4.692771	6.416782	5.311171	6.347815
17393288	Gss	glutathione synthetase	123.6879	113.3311	107.1799	104.5805	110.2275	100.9327
17277592	Gstz1	glutathione transferase zeta 1 (maleylacetoacetate isomerase)	695.7571	662.9931	630.5994	672.5697	716.6426	731.3611
17260881	Gapdh	glyceraldehyde-3-phosphate dehydrogenase	2168.933	2497.052	2770.576	2335.394	2227.207	2288.394
17310462	Gapdh	glyceraldehyde-3-phosphate dehydrogenase	2024.107	2343.29	2633.124	2147.973	2112.873	2156.117
17427183	Gapdh	glyceraldehyde-3-phosphate dehydrogenase	2919.468	3235.65	3677.17	3115.461	3025.945	3161.339
17439361	Gapdh	glyceraldehyde-3-phosphate dehydrogenase	1463.848	1674.843	1848.193	1552.234	1477.538	1519.731
17471011	Gapdh	glyceraldehyde-3-phosphate dehydrogenase	4.801816	3.913502	6.166364	5.146744	3.417521	5.005451
17478474	Gapdh	glyceraldehyde-3-phosphate dehydrogenase	2919.468	3235.65	3677.17	3115.461	3025.945	3161.339
17531480	3000002C10Rik	glyceraldehyde-3-phosphate dehydrogenase pseudogene	41.71892	65.00866	65.94219	53.87672	49.5523	48.93009
17423189	4930448K20Rik	glyceraldehyde-3-phosphate dehydrogenase pseudogene	1.809599	2.207678	2.32545	2.135376	2.260481	2.591217
17509180	Gm10313	glyceraldehyde-3-phosphate dehydrogenase pseudogene	161.6993	188.8268	211.2445	170.9701	173.4998	183.8672
17247924	Gm12070	glyceraldehyde-3-phosphate dehydrogenase pseudogene	3.741742	3.151918	2.720855	3.064603	2.782767	2.513609
17230451	Gm5069	glyceraldehyde-3-phosphate dehydrogenase pseudogene	3.822006	3.565193	4.476807	5.229562	4.051399	3.943099
17231613	Gm5177	glyceraldehyde-3-phosphate dehydrogenase pseudogene	2.924926	2.74952	2.796554	2.749787	2.934409	2.772178
17221373	Gm5523	glyceraldehyde-3-phosphate dehydrogenase pseudogene	65.71086	74.89357	71.72529	81.22771	58.42322	63.72643
17299414	Gm6498	glyceraldehyde-3-phosphate dehydrogenase pseudogene	4.406735	5.275727	6.051698	4.289264	4.974909	4.928129
17527013	Gm6981	glyceraldehyde-3-phosphate dehydrogenase pseudogene	115.1994	111.7081	124.8628	115.5065	87.77694	115.9694
17232727	Gm8055	glyceraldehyde-3-phosphate dehydrogenase pseudogene	40.08221	49.92118	51.43398	47.66704	42.95547	47.18381
17239917	Gm8709	glyceraldehyde-3-phosphate dehydrogenase pseudogene	5.58323	6.457474	6.457474	7.464005	6.084337	9.291104
17233037	Gm9034	glyceraldehyde-3-phosphate dehydrogenase pseudogene	4.01461	3.170411	4.20927	3.305755	3.342204	3.354808
17546512	Gapdh-ps15	glyceraldehyde-3-phosphate dehydrogenase, pseudogene 15	163.361	217.3626	255.4753	202.7123	179.7901	183.9245
17489289	Gapdhs	glyceraldehyde-3-phosphate dehydrogenase, spermatogenic	4.513573	10.29863	5.023629	5.420177	6.270976	5.21006
17530623	Glyctk	glycerate kinase	202.8346	194.1018	180.2556	200.1045	201.4461	178.7327
17543045	Gk	glycerol kinase	171.8338	185.6022	197.6905	155.9425	140.5978	151.0152
17449935	Gk2	glycerol kinase 2	3.751714	3.222052	3.414774	3.751714	3.729055	3.28866
17520537	Gk5	glycerol kinase 5 (putative)	2.506128	2.800336	3.009972	3.009972	3.106478	3.138967
17350586	Gykl1	glycerol kinase-like 1	3.17646	3.499091	3.34958	3.636927	3.377931	3.842379
17370897	Gpd2	glycerol phosphate dehydrogenase 2, mitochondrial	65.29452	74.21146	62.03885	65.29452	65.29452	63.36306
17375885	Gpat2	glycerol-3-phosphate acyltransferase 2, mitochondrial	4.76911	6.191333	6.287864	6.287864	5.147529	6.467284
17508134	Gpat4	glycerol-3-phosphate acyltransferase 4	199.1608	269.1003	232.2718	250.8029	239.1265	271.5135
17365728	Gpam	glycerol-3-phosphate acyltransferase, mitochondrial	470.6497	347.9606	399.9345	345.6313	369.6792	334.1148

17314888	Gpd1	glycerol-3-phosphate dehydrogenase 1 (soluble)	641.8089	593.6505	477.5076	593.0418	459.9075	433.9455
17531939	Gpd1l	glycerol-3-phosphate dehydrogenase 1-like	25.96111	33.49445	36.03558	36.03558	37.59633	36.03558
17435419	1700022A21Rik	glycerol-3-phosphate dehydrogenase 1-like pseudogene	4.392128	3.702321	4.748066	4.087702	4.545157	4.54185
17506824	Gnpat	glyceronephosphate O-acyltransferase	69.6678	77.99934	71.25997	70.02606	64.04349	68.30882
17391997	Gpcpd1	glycerophosphocholine phosphodiesterase GDE1 homolog (S. cerevisiae)	65.60573	164.8831	143.5839	240.5495	168.8549	207.9251
17495586	Gde1	glycerophosphodiester phosphodiesterase 1	102.0272	107.1108	116.5116	101.4665	94.44418	97.27317
17267430	Gdpd1	glycerophosphodiester phosphodiesterase domain containing 1	11.31339	12.21879	11.12243	13.38393	11.49967	11.12243
17267443	Gdpd1	glycerophosphodiester phosphodiesterase domain containing 1	3.286792	5.831876	4.777381	4.979842	3.797658	4.527642
17536600	Gdpd2	glycerophosphodiester phosphodiesterase domain containing 2	5.264111	6.605922	6.044823	5.554434	5.636541	6.827181
17483098	Gdpd3	glycerophosphodiester phosphodiesterase domain containing 3	4.67673	4.773608	4.076384	5.216745	5.844826	5.422657
17480439	Gdpd4	glycerophosphodiester phosphodiesterase domain containing 4	4.516734	4.051286	4.004688	3.712652	4.457825	3.796263
17480534	Gdpd5	glycerophosphodiester phosphodiesterase domain containing 5	5.918986	6.137538	4.943113	5.499689	3.691891	6.331863
17390810	Gatm	glycine amidinotransferase (L-arginine:glycine amidinotransferase)	17.30096	15.40119	14.9538	11.66577	11.77382	12.533
17312884	Gcat	glycine C-acetyltransferase (2-amino-3-ketobutyrate-coenzyme A ligase)	67.82005	43.58138	50.28025	46.96975	49.82611	50.65771
17513325	Gcsh	glycine cleavage system protein H (aminomethyl carrier)	34.54536	35.79782	26.84348	32.86796	41.67961	41.29113
17301852	Gm9199	glycine cleavage system protein H (aminomethyl carrier) pseudogene	141.5669	130.4624	151.7381	141.5669	160.5279	137.9326
17363865	Gldc	glycine decarboxylase	365.2372	252.7295	242.028	323.2866	261.856	291.7381
17345562	Gnmt	glycine N-methyltransferase	920.5485	842.0138	876.1961	1059.494	1171.377	1188.462
17263011	Glra1	glycine receptor, alpha 1 subunit	4.924202	3.227932	3.45355	3.670941	3.406806	4.554649
17546001	Glra2	glycine receptor, alpha 2 subunit	3.316749	2.797252	2.797465	2.637608	3.083631	2.637608
17501234	Glra3	glycine receptor, alpha 3 subunit	2.946455	2.682373	2.996794	3.068982	3.035049	2.87669
17544768	Glra4	glycine receptor, alpha 4 subunit	3.266012	4.117276	4.551462	3.220132	3.713681	4.20927
17406165	Glrb	glycine receptor, beta subunit	2.870595	2.278525	2.326837	2.501009	2.04812	2.238357
17431266	Grrp1	glycine/arginine rich protein 1	11.31355	11.31355	10.76749	12.66873	10.24203	11.42989
17357837	Glyat	glycine-N-acyltransferase	92.0943	74.68983	70.62172	80.69738	70.57768	68.50322
17344982	Glyatl3	glycine-N-acyltransferase-like 3	3.429266	2.932904	2.953407	3.10325	3.729055	2.953407
17477811	Gys1	glycogen synthase 1, muscle	11.8659	14.89339	14.94717	15.06971	14.86142	14.70434
17472497	Gys2	glycogen synthase 2	319.4885	432.2224	433.741	371.2996	368.0276	524.0607
17487884	Gsk3a	glycogen synthase kinase 3 alpha	104.2476	118.1078	132.9122	107.0136	96.04509	94.83384
17325560	Gsk3b	glycogen synthase kinase 3 beta	110.3613	120.9192	108.0429	106.8942	92.89442	90.0541
17404329	Gyg	glycogenin	17.36632	21.38237	21.88196	16.58055	20.97036	12.52093
17451568	Gltp	glycolipid transfer protein	29.75665	31.75892	39.58577	31.863	31.49236	30.16471
17252065	Gltpd2	glycolipid transfer protein domain containing 2	132.6104	118.2282	112.225	115.1444	106.0624	105.4818
17502789	Gypa	glycophorin A	3.748621	5.360175	11.65308	5.621987	4.140572	3.149494
17353337	Gypc	glycophorin C	7.764268	8.108415	9.065459	9.199912	5.47121	6.221619
17458439	Gpnmb	glycoprotein (transmembrane) nmb	8.666263	13.01145	20.5864	10.11462	10.42735	8.483561
17252165	Gp1ba	glycoprotein 1b, alpha polypeptide	4.392493	4.372929	4.966984	4.392493	3.681924	5.290375
17495622	Gp2	glycoprotein 2 (zymogen granule membrane)	3.703322	3.729649	3.703322	3.703322	3.566008	4.012552
17329636	Gp5	glycoprotein 5 (platelet)	6.844523	6.251143	6.287864	6.287864	6.942378	6.474595
17485713	Gp6	glycoprotein 6 (platelet)	3.721051	5.361587	5.487123	5.396784	5.665173	6.318491
17460569	Gp9	glycoprotein 9 (platelet)	15.67453	20.76152	25.56114	20.76152	20.76152	18.97358
17219039	Gpa33	glycoprotein A33 (transmembrane)	7.75405	7.14503	6.508703	6.121096	5.787058	6.903892
17384484	Ggta1	glycoprotein galactosyltransferase alpha 1, 3	9.330213	9.614288	11.02472	10.09935	9.271868	10.94659
17356685	Gpha2	glycoprotein hormone alpha 2	14.46024	16.43079	10.47429	12.99919	12.15712	15.6216
17281941	Gphb5	glycoprotein hormone beta 5	7.870689	7.204359	7.34234	8.561123	8.786204	7.204359
17412681	Cga	glycoprotein hormones, alpha subunit	4.925056	5.139685	5.60273	6.013396	5.480813	6.347815
17328958	Gp1bb	glycoprotein Ib, beta polypeptide	10.21721	9.750053	9.630762	8.830836	7.216053	6.352965
17239077	Ginm1	glycoprotein integral membrane 1	96.43801	79.38695	77.08616	88.23811	79.61675	74.44949
17501191	Gpm6a	glycoprotein m6a	11.36748	10.20186	9.111698	12.36834	6.030928	10.20186
17539611	Gpm6b	glycoprotein m6b	5.539977	4.536816	5.21982	4.881079	4.179963	5.057379
17399033	Glmp	glycosylated lysosomal membrane protein	105.0913	98.97347	117.8526	94.57323	92.74837	84.03043
17322437	Glycam1	glycosylation dependent cell adhesion molecule 1	6.215326	6.215326	5.617429	6.624034	5.142429	6.384174
17318036	Gml	glycosylphosphatidylinositol anchored molecule like	4.77854	4.783312	5.42044	3.596017	4.206995	4.047048
17312200	Gml2	glycosylphosphatidylinositol anchored molecule like 2	6.380768	8.652818	6.160041	6.637178	6.924165	4.052662
17286055	Gpld1	glycosylphosphatidylinositol specific phospholipase D1	233.3886	232.2766	231.3014	275.8117	308.3959	245.0717
17442780	Glt1d1	glycosyltransferase 1 domain containing 1	30.66668	64.83316	68.56973	66.06938	78.23867	87.15491
17406423	Glt28d2	glycosyltransferase 28 domain containing 2	5.556244	6.01423	4.272735	5.440314	5.14835	5.447597
17382755	Glt6d1	glycosyltransferase 6 domain containing 1	3.970388	3.661894	4.019288	3.723373	4.50807	3.703322
17298312	Glt8d1	glycosyltransferase 8 domain containing 1	23.88677	15.53413	17.93269	18.40671	21.75492	19.08636
17243493	Glt8d2	glycosyltransferase 8 domain containing 2	2.757594	3.324483	3.24098	4.6569	3.484158	2.606909
17388353	Gyltl1b	glycosyltransferase-like 1B	10.23664	6.74281	8.917875	6.121308	6.87443	5.866459
17384927	Gtdc1	glycosyltransferase-like domain containing 1	19.23737	16.2878	19.13325	18.78202	18.53654	20.44194
17458771	Gars	glycyl-tRNA synthetase	108.7099	86.25104	91.58885	93.59438	104.14	86.80643
17343143	Glo1	glyoxalase 1	496.2145	415.1129	332.6774	369.1619	361.2866	368.1934
17266079	Glod4	glyoxalase domain containing 4	62.1542	57.09451	56.90368	56.79689	63.94679	55.73841
17539979	Glod5	glyoxalase domain containing 5	3.802057	4.695439	4.43674	4.627555	4.960309	5.120057
17327889	Glyr1	glyoxylate reductase 1 homolog (Arabidopsis)	120.6555	118.5112	116.156	109.8697	114.349	107.1568
17413573	Grhpr	glyoxylate reductase/hydroxypyruvate reductase	400.5532	430.7808	424.3439	422.2928	432.969	350.3687
17215820	Gpc1	glypican 1	49.3504	51.20242	52.18741	41.78201	34.31577	48.09417
17454310	Gpc2	glypican 2 (cerebroglycan)	6.686216	6.544759	6.995756	6.544033	8.529434	5.882135
17541681	Gpc3	glypican 3	6.42319	5.936777	5.311282	5.674212	5.489139	5.494583
17541668	Gpc4	glypican 4	76.67052	50.37269	58.16059	69.45083	90.84845	69.66103
17302645	Gpc5	glypican 5	7.814481	8.569316	7.527548	8.51685	7.261462	9.134274
17302656	Gpc6	glypican 6	10.83142	11.04307	14.65763	8.95231	10.83142	10.62823
17249801	Gm2a	GM2 ganglioside activator protein	224.713	197.7865	231.5227	220.9123	205.1677	169.9292
17536240	1700003E24Rik	Gm5941 pseudogene	6.403186	5.775397	6.464819	5.618442	5.653914	6.36513
17543126	1700084M14Rik	Gm5941 pseudogene	2.189908	2.074768	1.646006	1.909705	1.985992	1.865006
17536215	LOC101055749	Gm5941 pseudogene	1.989988	2.527463	1.969528	2.247218	2.216956	2.294834
17380306	Gnas	GNAS (guanine nucleotide binding protein, alpha stimulating) complex locus	9.2528	10.18215	10.83915	9.873626	8.162154	8.883326
17513031	Glg1	golgi apparatus protein 1	67.18513	64.59453	65.62347	63.7441	62.68985	64.26049
17240800	Gopc	golgi associated PDZ and coiled-coil motif containing	71.59912	55.35724	48.59311	51.57857	62.86216	63.72952
17312780	Gga1	golgi associated, gamma adaptin ear containing, ARF binding protein 1	34.61367	31.23821	40.77068	28.86942	31.47948	32.37751
17495890	Gga2	golgi associated, gamma adaptin ear containing, ARF binding protein 2	26.44238	26.76118	29.17054	25.38	23.8279	23.99713
17271938	Gga3	golgi associated, gamma adaptin ear containing, ARF binding protein 3	7.07397	8.038799	7.049349	6.299206	6.744587	6.688951

17384753	Golga1	golgi autoantigen, golgin subfamily a, 1	41.11378	26.67118	26.65252	31.18888	28.9241	30.0887
17369747	Golga2	golgi autoantigen, golgin subfamily a, 2	32.70547	31.33254	31.9395	32.62283	34.21128	33.62494
17440409	Golga3	golgi autoantigen, golgin subfamily a, 3	62.67682	55.13513	53.1803	55.24263	62.93105	62.57958
17522887	Golga4	golgi autoantigen, golgin subfamily a, 4	93.97792	87.54907	96.82784	92.04516	104.3705	93.79896
17278073	Golga5	golgi autoantigen, golgin subfamily a, 5	78.33309	60.7259	58.52495	62.36091	64.90735	63.03626
17508161	Golga7	golgi autoantigen, golgin subfamily a, 7	101.5432	118.0914	129.5307	106.9046	119.4084	119.5243
17359552	Golga7b	golgi autoantigen, golgin subfamily a, 7B	5.386134	5.347261	5.126059	5.670437	4.409332	6.313106
17325410	Golgb1	golgi autoantigen, golgin subfamily b, macrogolgin 1	50.6844	38.75687	42.40169	44.43301	48.373	47.38144
17465310	Gcc1	golgi coiled coil 1	18.32816	17.4399	15.37367	19.90446	17.69684	17.82426
17406002	Golim4	golgi integral membrane protein 4	118.6872	125.0698	121.8059	114.7357	119.6179	118.2208
17293237	Golm1	golgi membrane protein 1	11.83494	21.01387	16.10269	17.20886	15.42983	15.42983
17310452	Golph3	golgi phosphoprotein 3	158.229	131.563	139.9609	132.7069	122.428	135.5114
17400403	Golph3l	golgi phosphoprotein 3-like	45.49796	29.85209	35.62974	36.27918	36.84559	35.5407
17532246	Gorasp1	golgi reassembly stacking protein 1	21.6027	28.13356	27.77914	29.59579	33.50234	22.25859
17371661	Gorasp2	golgi reassembly stacking protein 2	138.6938	119.0522	132.672	119.6536	119.3513	107.6153
17266146	Gosr1	golgi SNAP receptor complex member 1	110.4562	107.6691	93.75281	109.3417	98.67345	109.3849
17270567	Gosr2	golgi SNAP receptor complex member 2	86.94252	77.47076	75.78971	67.19165	69.74414	68.69675
17443972	Get4	golgi to ER traffic protein 4 homolog (S. cerevisiae)	43.42217	33.31754	38.26782	38.26782	38.26782	35.43762
17217321	Golt1a	golgi transport 1 homolog A (S. cerevisiae)	79.4442	67.94427	63.75692	67.85908	75.56886	69.31713
17464128	Golt1b	golgi transport 1 homolog B (S. cerevisiae)	29.45111	19.72851	20.20289	16.74467	19.26566	15.59152
17490667	Gfy	golgi-associated olfactory signaling regulator	6.325458	8.172118	8.402508	7.677092	7.046613	6.236342
17229113	Gorab	golgin, RAB6-interacting	9.422566	8.51685	8.51685	8.51685	4.930999	10.47138
17359925	Gbf1	golgi-specific brefeldin A-resistance factor 1	139.3636	112.5288	106.7017	113.7592	100.7768	87.49326
17399194	Gon4l	gon-4-like (C.elegans)	34.03695	30.6278	38.76627	33.84709	21.72012	25.36118
17399176	5830417I10Rik	gon-4-like pseudogene	35.14774	24.83667	28.38919	38.40121	22.42864	23.79192
17301576	Gnrh1	gonadotropin releasing hormone 1	3.733115	5.929991	5.258605	4.614742	3.879552	4.528427
17449213	Gnrhr	gonadotropin releasing hormone receptor	3.224935	3.555593	3.297357	2.991336	3.42556	3.38792
17328823	Gsc2	goosecoid homebox 2	14.38557	14.49959	14.28838	13.95761	13.19994	16.93634
17283685	Gsc	goosecoid homeobox	19.59522	21.83938	19.61996	28.03156	30.45801	21.83454
17308746	Gpalpp1	GPALPP motifs containing 1	44.2756	43.637	40.53707	43.77878	44.2756	50.45866
17312374	Gpaa1	GPI anchor attachment protein 1	35.34343	37.33948	38.70613	34.94756	34.35719	30.64755
17312236	Gpihbp1	GPI-anchored HDL-binding protein 1	30.56122	33.90415	36.26058	29.22781	33.90415	32.82789
17436151	Gpn1	GPN-loop GTPase 1	22.23438	24.15132	16.7492	22.84696	17.64393	22.34546
17419636	Gpn2	GPN-loop GTPase 2	22.65378	22.54835	18.49957	24.27854	23.69449	22.2279
17442082	Gpn3	GPN-loop GTPase 3	23.65675	20.22854	18.93456	21.55185	24.55866	19.88874
17467232	Gprin3	GPRIN family member 3	45.14487	49.04826	43.88968	48.75371	45.89707	53.06063
17541465	Gpr119	G-protein coupled receptor 119	3.332928	4.064083	4.220781	3.552784	3.7117	4.191568
17455249	Gpr12	G-protein coupled receptor 12	5.18459	6.641136	5.687285	5.141681	7.439935	5.56106
17545450	Gpr173	G-protein coupled receptor 173	4.681043	4.681043	4.543582	4.963267	5.010797	4.658127
17430962	Gpr3	G-protein coupled receptor 3	8.864259	7.992241	8.176934	8.176934	7.220907	8.176934
17277794	Gpr65	G-protein coupled receptor 65	19.64159	15.72567	16.80513	11.59998	16.13151	16.09633
17409663	Gpr88	G-protein coupled receptor 88	7.181922	7.698994	6.434643	8.217332	7.698994	8.524706
17368260	Gpsm1	G-protein signalling modulator 1 (AGS3-like, C. elegans)	6.315732	7.002305	6.541085	7.047102	6.691177	6.937376
17409386	Gpsm2	G-protein signalling modulator 2 (AGS3-like, C. elegans)	21.21569	20.18791	18.36224	23.75172	17.66277	20.13216
17336595	Gpsm3	G-protein signalling modulator 3 (AGS3-like, C. elegans)	10.96383	13.27601	16.38746	13.27601	12.70452	17.69684
17535353	Gpr50	G-protein-coupled receptor 50	10.32958	8.782003	8.71252	8.901201	9.560725	8.651378
17274513	Grhl1	grainyhead-like 1 (Drosophila)	5.346668	6.224725	4.804246	5.313749	6.749871	5.614471
17311104	Grhl2	grainyhead-like 2 (Drosophila)	6.879625	7.036949	6.992896	6.67663	7.146268	6.992896
17431478	Grhl3	grainyhead-like 3 (Drosophila)	9.859674	8.327884	6.466343	8.553827	6.805211	7.483899
17489470	Gramd1a	GRAM domain containing 1A	30.29064	27.79956	24.67058	28.17239	20.13411	23.53474
17525803	Gramd1b	GRAM domain containing 1B	6.587632	6.575041	7.371662	5.613109	4.939449	6.869428
17330506	Gramd1c	GRAM domain containing 1C	43.23716	46.20445	45.02767	48.10117	50.82795	51.47179
17518021	Gramd2	GRAM domain containing 2	6.923877	6.171709	6.187643	7.533812	5.958214	6.171709
17350697	Gramd3	GRAM domain containing 3	65.91615	70.17183	76.37243	62.89311	52.47288	65.81388
17313926	Gramd4	GRAM domain containing 4	13.05934	10.6621	13.07492	10.49063	10.49063	7.154892
17371201	Gca	grancalcin	26.33975	22.33044	20.66346	17.97052	20.82335	19.75875
17256959	Grn	granulin	345.5115	483.6434	697.7614	394.4887	417.7946	333.2243
17296286	Gzma	granzyme A	3.133902	3.318957	2.979851	2.318095	2.987855	4.2325
17307033	Gzmb	granzyme B	4.139108	3.134641	3.390599	4.494031	3.230774	3.5534
17307025	Gzmc	granzyme C	2.515613	2.346629	2.2253	2.668428	2.458673	2.220077
17306991	Gzmd	granzyme D	2.548503	2.134458	2.243483	2.858087	3.301679	2.430225
17306983	Gzme	granzyme E	2.492079	3.160932	3.14415	3.125008	2.738641	2.546296
17307017	Gzmf	granzyme F	3.009972	2.260795	2.645628	2.779856	3.009972	4.645326
17306999	Gzmg	granzyme G	2.406006	1.91445	2.257256	2.507466	2.393646	2.072138
17296294	Gzmk	granzyme K	4.386499	5.631737	4.64362	5.450279	4.801649	5.096087
17234917	Gzmm	granzyme M (lymphocyte met-ase 1)	7.963375	9.182646	11.44036	9.404818	6.845235	9.808891
17307007	Gzmn	granzyme N	4.257274	3.856681	4.029973	4.802581	4.696773	6.036992
17443562	Gigyf1	GRB10 interacting GYF protein 1	13.95984	10.72652	14.67796	13.96893	12.57555	15.33685
17443565	Gigyf1	GRB10 interacting GYF protein 1	19.37884	29.56565	29.22336	26.61707	32.76764	30.71114
17215251	Gigyf2	GRB10 interacting GYF protein 2	42.19149	39.23883	45.99707	43.5014	42.23479	36.92664
17353032	Garem	GRB2 associated, regulator of MAPK1	86.97085	90.28159	112.2787	75.22759	83.78151	79.75096
17435737	Gareml	GRB2 associated, regulator of MAPK1-like	6.397064	6.039001	6.41318	7.686304	6.177572	7.059764
17296128	Gapt	Grb2-binding adaptor, transmembrane	8.346319	8.19742	6.82666	6.559746	8.022545	7.237242
17250615	Grap	GRB2-related adaptor protein	13.5976	11.88385	15.41796	11.50086	12.35189	14.34057
17313147	Grap2	GRB2-related adaptor protein 2	7.374064	6.687931	5.4376	6.047547	6.266718	5.474776
17389467	Grem1	gremlin 1	8.48862	8.807057	6.922005	9.703667	9.075141	7.061575
17230127	Grem2	gremlin 2 homolog, cysteine knot superfamily (Xenopus laevis)	21.69725	29.56954	32.11243	31.40821	73.46983	41.02354
17449456	Grsf1	G-rich RNA sequence binding factor 1	200.4446	181.2868	163.2014	189.0272	152.0793	189.7624
17233408	Gcc2	GRIP and coiled-coil domain containing 2	43.90003	46.14364	50.17268	49.16126	59.64131	42.92095
17532973	Gripap1	GRIP1 associated protein 1	25.89946	27.48228	25.14204	31.89857	24.11953	27.02495
17449473	Gc	group specific component	3482.989	3556.371	3475.223	3288.309	3497.359	3423.675
17467316	Gadd45a	growth arrest and DNA-damage-inducible 45 alpha	28.0792	29.71048	35.98366	35.62776	34.88996	41.04259
17235511	Gadd45b	growth arrest and DNA-damage-inducible 45 beta	20.98185	37.94216	27.29548	27.52079	17.45695	21.61918
17287361	Gadd45g	growth arrest and DNA-damage-inducible 45 gamma	94.96079	48.08317	27.83233	34.49702	76.84609	94.30485

17503217	Gadd45gip1	growth arrest and DNA-damage-inducible, gamma interacting protein 1	20.22489	16.83206	16.83206	18.49649	17.75631	15.15466
17293313	Gas1	growth arrest specific 1	23.14053	27.42786	30.02787	27.42786	30.31521	27.42786
17478476	Gas2	growth arrest specific 2	27.39746	28.38978	26.02182	24.45996	24.98655	28.26216
17218653	Gas5	growth arrest specific 5	13.63308	16.55896	18.71729	17.78452	22.19157	16.2596
17507605	Gas6	growth arrest specific 6	104.6146	116.9129	131.46	91.31817	135.3283	75.21769
17251222	Gas7	growth arrest specific 7	7.988364	11.21284	8.606878	8.594537	8.866041	9.073128
17506680	Gas8	growth arrest specific 8	19.83189	17.97799	17.14731	16.66489	15.83945	16.3601
17260075	Gas2l1	growth arrest-specific 2 like 1	30.19567	35.68832	33.57161	29.90697	25.49443	20.95104
17266903	Gas2l2	growth arrest-specific 2 like 2	11.12359	8.16804	9.583615	12.26819	6.486409	8.143675
17244057	Gas2l3	growth arrest-specific 2 like 3	9.668419	12.00564	15.37612	9.178001	12.36645	10.60373
17330443	Gap43	growth associated protein 43	2.737768	2.826369	3.236826	2.925595	2.932823	3.450798
17501905	Gdf1	growth differentiation factor 1	8.642987	8.790895	8.0349	10.15086	8.287173	9.5882
17298731	Gdf10	growth differentiation factor 10	14.75662	21.49039	33.28845	19.60623	10.24899	11.90116
17246352	Gdf11	growth differentiation factor 11	5.387366	5.209663	5.50812	7.119471	4.536281	6.623529
17510114	Gdf15	growth differentiation factor 15	15.7969	18.33007	13.17992	15.0383	11.09486	11.10071
17298738	Gdf2	growth differentiation factor 2	15.571	24.39105	22.96126	24.65195	15.42298	16.39859
17470592	Gdf3	growth differentiation factor 3	6.714221	6.681537	6.414959	5.785694	6.229688	6.236688
17393390	Gdf5	growth differentiation factor 5	5.523413	5.585475	5.987982	6.364071	6.393031	6.257863
17411727	Gdf6	growth differentiation factor 6	6.030928	6.030928	5.440201	4.90013	8.12642	7.135478
17279916	Gdf7	growth differentiation factor 7	12.95009	14.38616	12.79981	12.75871	13.11822	18.32206
17249515	Gdf9	growth differentiation factor 9	6.495632	7.031001	6.554928	6.105121	6.063917	5.390204
17450679	Gfi1	growth factor independent 1	18.00718	20.13076	22.1148	19.7778	14.62779	18.56045
17383413	Gfi1b	growth factor independent 1B	10.72124	10.65863	9.180101	9.022517	10.72124	13.97368
17260668	Grb10	growth factor receptor bound protein 10	15.69203	15.35323	22.60155	16.78185	15.50434	12.25433
17385853	Grb14	growth factor receptor bound protein 14	39.40509	32.55055	34.01337	41.32005	42.16167	44.8694
17272004	Grb2	growth factor receptor bound protein 2	67.40436	63.8082	65.69219	72.53071	69.86897	72.55635
17510823	Gab1	growth factor receptor bound protein 2-associated protein 1	35.00235	38.25642	47.02806	40.87424	33.86281	38.44063
17480312	Gab2	growth factor receptor bound protein 2-associated protein 2	8.401984	14.25316	15.81115	13.37799	8.567237	14.37121
17542732	Gab3	growth factor receptor bound protein 2-associated protein 3	3.002179	4.186661	3.568926	3.643715	3.643715	3.295666
17256033	Grb7	growth factor receptor bound protein 7	99.91158	108.2212	126.0253	121.0828	118.9359	118.5433
17341987	Gfer	growth factor, erv1 (S. cerevisiae)-like (augmenter of liver regeneration)	47.89456	41.90005	37.65553	41.61783	39.84316	44.85789
17270829	Gh	growth hormone	4.644972	5.711778	6.95331	6.82666	6.418339	7.833082
17305167	Ghitm	growth hormone inducible transmembrane protein	371.7425	375.8231	373.178	342.1014	269.6829	302.1037
17315635	Ghr	growth hormone receptor	123.585	104.3871	88.46715	112.1806	117.3342	130.1887
17393743	Ghrh	growth hormone releasing hormone	5.38942	5.578665	5.209374	5.19315	5.573679	6.221489
17458820	Ghrhr	growth hormone releasing hormone receptor	6.002735	6.226537	7.625701	5.46223	6.458683	6.226537
17396395	Ghsr	growth hormone secretagogue receptor	11.77839	11.26691	10.08249	12.12825	11.15872	9.360334
17348320	Greb1l	growth regulation by estrogen in breast cancer-like	3.392033	6.910595	5.134593	4.833506	3.983482	5.172565
17315167	Grasp	GRP1 (general receptor for phosphoinositides 1)-associated scaffold protein	25.90359	32.74448	29.95407	25.62162	25.07121	29.47664
17436931	Grpel1	GrpE-like 1, mitochondrial	115.8232	114.1874	143.3959	114.6188	131.1796	138.192
17354804	Grpel2	GrpE-like 2, mitochondrial	46.58327	64.4389	75.17286	59.52649	56.49182	64.9303
17444836	Gsx1	GS homeobox 1	5.764039	5.433644	5.298001	5.55871	5.433644	5.006457
17438233	Gsx2	GS homeobox 2	6.714128	6.992896	9.787565	6.816306	6.728104	8.215566
17496102	Gsg1l	GSG1-like	14.0397	19.94285	14.82305	18.65564	14.85998	15.90535
17251257	Gsg1l2	GSG1-like 2	5.338965	5.009338	5.203708	3.949254	4.779137	5.18459
17278410	Gskip	GSK3B interacting protein	23.49611	35.3324	31.03986	34.35885	35.91937	35.12923
17443083	Gtf2ird2	GTF2I repeat domain containing 2	19.76451	20.80892	17.94811	18.51214	19.54503	18.49085
17411840	Gem	GTP binding protein (gene overexpressed in skeletal muscle)	10.1025	10.41377	9.087289	10.1025	6.581608	10.1025
17313023	Gtpbp1	GTP binding protein 1	54.14927	47.83882	55.78673	53.38306	44.17475	53.09301
17337984	Gtpbp2	GTP binding protein 2	22.4291	27.64506	31.9847	30.8325	29.91236	27.65024
17502206	Gtpbp3	GTP binding protein 3	18.08047	17.22927	17.25294	20.13816	16.29401	13.8812
17290398	Gtpbp4	GTP binding protein 4	77.78122	70.25992	61.0877	73.45353	71.50687	71.05085
17450952	Gtpbp6	GTP binding protein 6 (putative)	22.17927	20.12714	18.29934	26.81819	18.12413	19.76533
17305748	Gch1	GTP cyclohydrolase 1	203.3808	207.4716	200.278	261.4552	228.0443	221.6946
17374738	Gchfr	GTP cyclohydrolase I feedback regulator	71.89203	72.28929	76.17312	78.05505	89.30815	102.7511
17249833	G3bp1	GTPase activating protein (SH3 domain) binding protein 1	66.36129	59.08541	60.56112	59.0723	79.46678	50.41007
17449627	G3bp2	GTPase activating protein (SH3 domain) binding protein 2	96.18033	94.47954	110.7429	122.6234	96.87416	112.4734
17384252	Gapvd1	GTPase activating protein and VPS9 domains 1	67.19957	65.9061	64.61289	66.57137	68.19528	69.79008
17384100	Garnl3	GTPase activating RANGAP domain-like 3	6.925412	6.54299	5.120306	5.967638	6.826377	6.54299
17458379	Gimap1	GTPase, IMAP family member 1	9.143459	6.706035	8.423151	8.51502	5.997495	6.885875
17466615	Gimap1os	GTPase, IMAP family member 1, opposite strand	6.860007	6.355431	6.503111	5.937312	7.500413	4.747178
17466618	Gimap3	GTPase, IMAP family member 3	8.717352	8.286024	12.25272	8.300855	7.891229	8.139838
17458362	Gimap4	GTPase, IMAP family member 4	27.29245	26.09045	28.01563	24.99885	21.74015	23.18179
17458385	Gimap5	GTPase, IMAP family member 5	6.066061	6.150655	6.337571	8.230614	5.120306	7.304421
17466606	Gimap6	GTPase, IMAP family member 6	9.488237	8.832427	11.25473	8.827531	9.488237	8.689481
17458372	Gimap7	GTPase, IMAP family member 7	3.488943	4.319218	2.948355	4.246252	2.848752	2.99881
17458345	Gimap8	GTPase, IMAP family member 8	7.539192	10.19903	11.31685	9.825018	11.90231	12.27848
17458355	Gimap9	GTPase, IMAP family member 9	7.743737	7.434522	7.299815	7.698994	10.51415	8.029944
17494656	Gvin1	GTPase, very large interferon inducible 1	78.59415	57.43467	66.9383	71.45931	83.64225	40.33275
17494649	Gm17757	GTPase, very large interferon inducible 1 pseudogene	56.91709	46.05609	56.52824	51.9575	49.89212	36.60858
17494662	Gm18853	GTPase, very large interferon inducible 1 pseudogene	64.94786	53.56096	64.47241	53.35198	55.53441	40.29475
17494668	Gm4759	GTPase, very large interferon inducible 1 pseudogene	4.413307	2.301014	3.009972	2.619806	3.009972	3.729055
17445381	Gtpbp10	GTP-binding protein 10 (putative)	30.73009	34.24164	27.26201	35.39417	34.61031	35.09394
17330602	Gtpbp8	GTP-binding protein 8 (putative)	36.37108	33.49445	28.27314	35.19601	25.94474	35.83283
17242842	Gamt	guanidinoacetate methyltransferase	156.2238	141.6081	161.5694	156.4502	220.9613	172.2954
17363470	Gda	guanine deaminase	48.35422	45.11953	49.03024	37.99374	37.80434	37.34569
17398020	Gmps	guanine monophosphate synthetase	10.28108	8.367925	8.974739	7.297033	6.923013	6.983741
17398023	Gmps	guanine monophosphate synthetase	126.6576	85.64814	84.34672	96.07439	96.12168	89.70273
17445735	Gnai1	guanine nucleotide binding protein (G protein), alpha inhibiting 1	18.62954	14.46345	19.14665	12.96814	17.7844	15.89135
17530955	Gnai2	guanine nucleotide binding protein (G protein), alpha inhibiting 2	154.1755	144.9378	142.0987	144.3963	117.0515	128.0284
17409217	Gnai3	guanine nucleotide binding protein (G protein), alpha inhibiting 3	190.8718	159.8384	155.7491	153.4825	140.7138	161.2997
17422573	Gnb1	guanine nucleotide binding protein (G protein), beta 1	229.839	261.017	297.2247	202.287	230.3877	198.6039
17454049	Gnb2	guanine nucleotide binding protein (G protein), beta 2	56.86583	52.88769	55.88736	53.21681	52.10464	52.59195
17470917	Gnb3	guanine nucleotide binding protein (G protein), beta 3	6.964791	7.513734	7.831562	6.943004	5.590472	7.795275

17404601	Gnb4	guanine nucleotide binding protein (G protein), beta 4	17.55804	16.33472	13.84295	14.31296	9.151575	16.34333
17519481	Gnb5	guanine nucleotide binding protein (G protein), beta 5	3.394808	3.243025	3.740082	4.461813	4.045001	3.729055
17323736	Gnb1l	guanine nucleotide binding protein (G protein), beta polypeptide 1-like	6.216748	8.310124	6.398262	7.251352	6.845519	9.445715
17248873	Gnb2l1	guanine nucleotide binding protein (G protein), beta polypeptide 2 like 1	37.42784	37.42784	39.95405	32.21007	37.42784	36.51988
17248890	Gnb2l1	guanine nucleotide binding protein (G protein), beta polypeptide 2 like 1	22.80487	26.79868	27.21764	37.11061	39.80092	29.85892
17414428	Gng10	guanine nucleotide binding protein (G protein), gamma 10	30.00643	27.93845	35.09005	27.93845	26.074	24.33009
17455793	Gng11	guanine nucleotide binding protein (G protein), gamma 11	22.62789	20.30397	16.26941	26.36112	12.96922	26.65307
17459250	Gng12	guanine nucleotide binding protein (G protein), gamma 12	91.20494	98.0367	90.08531	85.43113	87.72098	78.83258
17334716	Gng13	guanine nucleotide binding protein (G protein), gamma 13	7.129891	8.150621	5.88805	7.412345	7.452425	7.949643
17303706	Gng2	guanine nucleotide binding protein (G protein), gamma 2	10.71983	15.21508	16.43694	15.26388	15.46881	17.91592
17362511	Gng3	guanine nucleotide binding protein (G protein), gamma 3	3.279759	3.253494	5.301902	3.357622	3.939684	4.119702
17285322	Gng4	guanine nucleotide binding protein (G protein), gamma 4	14.57749	16.57492	15.40108	12.44807	13.30927	15.20738
17403543	Gng5	guanine nucleotide binding protein (G protein), gamma 5	12.64198	10.48228	12.26325	9.488237	9.500807	12.16226
17243147	Gng7	guanine nucleotide binding protein (G protein), gamma 7	17.96989	16.92038	21.23335	18.91095	15.29269	15.93338
17474211	Gng8	guanine nucleotide binding protein (G protein), gamma 8	3.763226	6.106814	4.83431	4.48569	3.953965	5.181644
17455787	Gngt1	guanine nucleotide binding protein (G protein), gamma transducing activity polyp	4.315268	3.753353	3.673792	4.053646	3.753353	4.041862
17255466	Gngt2	guanine nucleotide binding protein (G protein), gamma transducing activity polyp	11.93197	19.41689	19.18478	21.56605	16.45336	16.45336
17243392	Gna11	guanine nucleotide binding protein, alpha 11	137.2945	130.084	132.3129	109.3167	135.3143	115.9517
17454617	Gna12	guanine nucleotide binding protein, alpha 12	221.8945	221.0456	200.2488	201.2103	194.5409	181.4112
17257822	Gna13	guanine nucleotide binding protein, alpha 13	46.08611	59.71163	47.85839	44.67048	48.99661	41.58957
17357971	Gna14	guanine nucleotide binding protein, alpha 14	14.31296	15.60017	13.39423	11.96294	9.545495	14.78795
17243382	Gna15	guanine nucleotide binding protein, alpha 15	10.28707	11.04552	7.007549	7.698994	6.789804	8.979219
17503884	Gnao1	guanine nucleotide binding protein, alpha O	5.942541	6.127633	6.859198	6.171709	4.996161	6.341702
17357959	Gnaq	guanine nucleotide binding protein, alpha q polypeptide	52.0065	55.72257	54.13051	50.92104	66.69007	60.99588
17351426	Gnal	guanine nucleotide binding protein, alpha stimulating, olfactory type	4.420655	4.886969	4.558534	4.680199	5.39757	4.886969
17530990	Gnat1	guanine nucleotide binding protein, alpha transducing 1	11.23478	17.8968	21.96189	20.08769	25.78839	18.87415
17401629	Gnat2	guanine nucleotide binding protein, alpha transducing 2	4.65219	3.320637	3.8336	5.19722	3.843871	3.596242
17434995	Gnat3	guanine nucleotide binding protein, alpha transducing 3	3.150673	3.263862	3.430122	2.469519	3.198998	2.71782
17234264	Gnaz	guanine nucleotide binding protein, alpha z subunit	4.01461	3.191025	3.667889	4.801649	3.278896	3.544003
17337349	Gnl1	guanine nucleotide binding protein-like 1	19.6788	17.33649	25.33588	27.62189	24.55185	24.56326
17418413	Gnl2	guanine nucleotide binding protein-like 2 (nucleolar)	68.55167	59.87575	63.10533	49.07989	71.19924	54.94286
17304493	Gnl3	guanine nucleotide binding protein-like 3 (nucleolar)	70.66383	59.79571	55.69786	53.75921	62.76116	48.86501
17545381	Gnl3l	guanine nucleotide binding protein-like 3 (nucleolar)-like	38.55911	32.8748	29.23146	27.94252	30.6278	30.6278
17535808	Gdi1	guanosine diphosphate (GDP) dissociation inhibitor 1	107.7751	129.3548	115.8384	101.8266	109.888	112.436
17284880	Gdi2	guanosine diphosphate (GDP) dissociation inhibitor 2	375.5237	352.8986	330.3153	332.3225	319.9893	345.0424
17286973	Gmpr	guanosine monophosphate reductase	12.46698	12.60295	12.60295	13.21404	12.56353	13.21404
17300638	Gmpr2	guanosine monophosphate reductase 2	40.18318	41.22764	36.95557	41.81417	35.31894	43.41104
17450515	Gbp11	guanylate binding protein 11	31.29912	30.06389	36.31239	37.7164	25.66126	19.46243
17403268	Gbp2	guanylate binding protein 2	17.48469	12.24474	16.76941	12.31248	15.80107	17.07555
17403255	Gbp2b	guanylate binding protein 2b	20.36373	19.76738	21.00732	13.23081	11.81485	19.76738
17403237	Gbp3	guanylate binding protein 3	10.80068	10.12289	19.30349	16.71162	16.41451	23.80743
17450461	Gbp4	guanylate binding protein 4	3.828422	4.262678	7.787445	5.214685	4.377448	5.809927
17403205	Gbp5	guanylate binding protein 5	6.335726	6.935885	6.164312	5.322891	5.882176	5.909188
17450477	Gbp6	guanylate binding protein 6	2.714563	2.524697	2.435807	2.718536	2.491319	3.289641
17403224	Gbp7	guanylate binding protein 7	57.09055	36.6797	62.19299	55.44018	65.32349	42.72889
17514399	Gucy1a2	guanylate cyclase 1, soluble, alpha 2	11.5078	12.33298	10.98604	11.16654	11.79816	12.27516
17406221	Gucy1a3	guanylate cyclase 1, soluble, alpha 3	20.55133	20.16428	21.1615	22.29456	19.75273	17.81586
17307526	Gucy1b2	guanylate cyclase 1, soluble, beta 2	3.68024	3.457382	3.096868	3.688643	4.232823	3.381258
17406205	Gucy1b3	guanylate cyclase 1, soluble, beta 3	11.63194	24.14261	21.43312	14.98337	14.7604	19.20886
17472129	Gucy2c	guanylate cyclase 2c	11.28231	10.48198	13.1064	9.099706	9.25928	11.26238
17480466	Gucy2d	guanylate cyclase 2d	5.385462	5.780603	5.385462	4.974736	4.688739	6.635016
17264538	Gucy2e	guanylate cyclase 2e	5.087405	5.338965	5.338965	5.338965	5.55609	5.397458
17545023	Gucy2f	guanylate cyclase 2f	5.921612	5.604905	5.48933	6.341614	6.234181	5.48933
17365755	Gucy2g	guanylate cyclase 2g	3.729055	4.792705	3.729055	3.729055	4.536187	3.82137
17345670	Guca1a	guanylate cyclase activator 1a (retina)	4.316733	6.722277	7.241055	6.818432	5.556283	5.082295
17338245	Guca1b	guanylate cyclase activator 1B	8.536591	7.890901	7.313743	9.737895	9.005023	8.49745
17417946	Guca2a	guanylate cyclase activator 2a (guanylin)	5.772875	8.638495	5.525596	6.0951	4.501107	3.951855
17429441	Guca2b	guanylate cyclase activator 2b (retina)	7.381735	8.661459	7.843676	8.51685	6.326818	7.680605
17263362	Guk1	guanylate kinase 1	55.14201	48.86501	49.39421	56.8473	54.49606	57.43746
17450501	Gbp10	guanylate-binding protein 10	224.4266	138.2753	182.6857	236.4072	134.1674	222.9166
17450434	Gbp8	guanylate-binding protein 8	29.36061	26.04672	39.89677	20.95075	48.4989	28.3208
17450448	Gbp9	guanylate-binding protein 9	51.43149	43.09444	46.46717	54.41038	36.27943	47.83518
17241822	Gucd1	guanylyl cyclase domain containing 1	32.8338	25.70594	27.99156	26.66934	29.06941	27.80515
17437998	Guf1	GUF1 GTPase homolog (S. cerevisiae)	23.00667	22.62789	24.05392	25.83061	25.01324	28.14545
17307801	Gulo	gulonolactone (L-) oxidase	656.1128	516.3552	578.2903	616.2278	867.464	698.555
17212442	Gulp1	GULP, engulfment adaptor PTB domain containing 1	4.180861	3.920832	3.824842	4.196132	3.691891	5.082576
17216201	Gin1	gypsy retrotransposon integrase 1	14.33878	17.36608	12.57381	12.98397	19.20141	14.07107
17312882	H1f0	H1 histone family, member 0	122.6574	113.4592	135.4992	145.1831	152.5481	155.1683
17321199	H1fnt	H1 histone family, member N, testis-specific	4.318948	4.997962	4.637284	4.997962	5.120306	5.718077
17462044	H1foo	H1 histone family, member O, oocyte-specific	4.681043	4.231825	5.303299	4.801649	6.405051	5.262141
17468702	H1fx	H1 histone family, member X	9.501729	10.93226	10.77361	11.22567	9.08414	12.49518
17230715	Hlx	H2.0-like homeobox	16.69993	20.19141	18.85859	20.28175	17.70568	20.02319
17381988	H2afb1	H2A histone family, member B1	12.20508	8.726783	10.03466	14.24585	6.678481	10.29855
17548827	H2afb2	H2A histone family, member B2	11.64969	11.72146	11.79832	11.56513	11.64969	17.85678
17548877	H2afb3	H2A histone family, member B3	3.600557	3.864415	4.037046	3.870661	3.862273	3.451157
17463833	H2afj	H2A histone family, member J	10.79424	11.49457	8.196341	10.41665	10.77533	9.102545
17260357	H2afv	H2A histone family, member V	56.73275	52.36569	56.24138	46.74143	68.23405	61.49682
17516558	H2afx	H2A histone family, member X	65.22757	42.84129	50.89422	41.64467	58.69436	37.78548
17292974	H2afy	H2A histone family, member Y	71.81981	59.68804	65.28637	55.59757	73.29435	62.21541
17241240	H2afy2	H2A histone family, member Y2	3.445014	3.451013	5.383185	4.295154	4.325689	2.882219
17317415	H2afy3	H2A histone family, member Y3	17.7823	15.99579	27.89085	17.39475	18.48035	20.58726
17403004	H2afz	H2A histone family, member Z	23.12273	25.74713	22.16129	23.36893	17.33901	24.40441
17537978	H2bfm	H2B histone family, member M	3.532109	3.547321	3.661082	3.983012	4.035479	4.337188
17343735	H2-Ke6	H2-K region expressed gene 6	99.09566	91.56726	92.53582	98.06524	85.73724	95.71347

17230469	H3f3a	H3 histone, family 3A	10.80083	10.92938	10.1025	10.33467	8.415739	9.920137
17272095	H3f3b	H3 histone, family 3B	8.370188	10.42894	11.24366	12.82592	12.67426	13.53488
17436832	Hmx1	H6 homeobox 1	7.01095	6.884252	6.824957	5.295732	7.935109	6.588865
17484035	Hmx2	H6 homeobox 2	17.05934	14.95027	11.31465	13.53225	15.65205	14.59558
17484030	Hmx3	H6 homeobox 3	12.60295	13.34308	15.23196	13.23045	12.60295	13.12722
17301768	Hr	hairless	8.715842	7.466955	8.363808	7.287076	9.716589	7.730705
17324623	Hes1	hairy and enhancer of split 1 (Drosophila)	12.32837	13.45266	19.24471	25.10112	16.46317	20.24818
17422109	Hes2	hairy and enhancer of split 2 (Drosophila)	13.65739	10.94652	12.83695	14.33152	11.06108	11.36322
17433461	Hes3	hairy and enhancer of split 3 (Drosophila)	5.425214	5.730893	5.532264	5.36523	5.848311	5.819479
17422443	Hes5	hairy and enhancer of split 5 (Drosophila)	3.531375	4.663328	4.342151	4.853347	3.872298	4.534866
17225499	Hes6	hairy and enhancer of split 6	22.41014	20.25828	18.80742	19.86215	21.12047	27.05437
17251557	Hes7	hairy and enhancer of split 7 (Drosophila)	26.04113	27.58745	26.2079	30.60764	28.44947	27.53758
17404011	Hey1	hairy/enhancer-of-split related with YRPW motif 1	10.00452	15.067	20.28906	17.9271	14.25524	18.0831
17240077	Hey2	hairy/enhancer-of-split related with YRPW motif 2	7.341679	6.992896	5.456629	6.561884	5.407418	7.005655
17418177	Heyl	hairy/enhancer-of-split related with YRPW motif-like	9.240686	12.64127	10.68237	10.36034	9.734454	10.03376
17354356	Hdhd1a	haloacid dehalogenase-like hydrolase domain containing 1A	2.647095	2.469331	2.500281	2.64297	3.414821	2.194573
17351919	Hdhd2	haloacid dehalogenase-like hydrolase domain containing 2	45.28433	45.11202	51.40939	43.44445	45.51342	42.61059
17426198	Hdhd3	haloacid dehalogenase-like hydrolase domain containing 3	10.71099	9.848404	6.33498	9.639177	8.810171	7.581956
17512809	Hp	haptoglobin	2386.631	2572.419	2743.268	2045.333	2996.536	2200.202
17441524	Hrk	harakiri, BCL2 interacting protein (contains only BH3 domain)	3.071514	3.776186	4.648258	3.703322	4.123102	3.729055
17373406	Harbi1	harbinger transposase derived 1	19.67293	12.63287	16.59745	16.56045	18.65293	18.521
17477670	Rras	Harvey rat sarcoma oncogene, subgroup R	37.25701	61.4687	75.95853	53.99036	37.72084	42.35499
17497785	Hras	Harvey rat sarcoma virus oncogene	33.79154	32.2447	30.44092	32.93752	35.8455	28.25198
17355538	Haus1	HAUS augmin-like complex, subunit 1	15.60396	16.51414	15.19853	19.51998	11.89176	18.17823
17375100	Haus2	HAUS augmin-like complex, subunit 2	30.5119	27.96422	24.99643	29.13558	29.26248	31.02243
17306384	Haus4	HAUS augmin-like complex, subunit 4	25.25417	22.62789	17.58252	23.34805	24.88165	24.31913
17489251	Haus5	HAUS augmin-like complex, subunit 5	14.20936	13.48562	13.1551	13.39423	9.538153	12.72638
17426956	Haus6	HAUS augmin-like complex, subunit 6	11.79661	10.74944	9.84274	10.62308	10.47806	10.08158
17542349	Haus7	HAUS augmin-like complex, subunit 7	9.003338	8.624077	8.467112	7.130632	10.50752	8.211695
17232003	Hbs1l	Hbs1-like (S. cerevisiae)	136.5826	120.6932	105.7065	115.0965	115.6002	113.8452
17407190	Hax1	HCLS1 associated X-1	123.2651	113.8713	106.211	111.777	128.1189	107.7721
17273910	Hs1bp3	HCLS1 binding protein 3	22.22575	31.20034	25.37578	23.70714	26.50954	26.16343
17232444	Hddc2	HD domain containing 2	4.433082	5.300432	5.22659	6.260162	7.89473	6.196012
17479596	Hddc3	HD domain containing 3	11.99657	16.61322	15.73102	19.04922	11.40536	15.95437
17239493	Heca	headcase homolog (Drosophila)	57.20225	55.88309	50.04972	53.44007	58.73628	59.72073
17263063	Hand1	heart and neural crest derivatives expressed transcript 1	8.858669	10.10544	8.863644	9.533064	11.77969	15.72099
17501272	Hand2	heart and neural crest derivatives expressed transcript 2	23.21765	24.88009	23.4896	22.08676	26.9085	29.33152
17285157	Heatr1	HEAT repeat containing 1	46.2894	31.54261	28.19153	31.04825	27.69435	26.51745
17503572	Heatr3	HEAT repeat containing 3	44.99741	36.2744	30.02204	36.09007	41.64583	34.36124
17282450	Heatr4	HEAT repeat containing 4	7.45289	4.707333	4.350587	5.265243	4.982742	5.956934
17281025	Heatr5a	HEAT repeat containing 5A	33.73303	29.24423	28.35478	27.14811	27.57349	24.5085
17347307	Heatr5b	HEAT repeat containing 5B	4.748428	3.204413	3.567838	3.135227	3.413094	3.4027
17347311	Heatr5b	HEAT repeat containing 5B	36.91205	30.18647	35.53872	30.87998	29.47827	33.02782
17254305	Heatr6	HEAT repeat containing 6	36.5604	34.42894	36.17046	39.09765	36.00861	33.77866
17266928	Heatr9	HEAT repeat containing 9	3.276215	4.082209	4.183731	5.283595	3.806909	4.183731
17455507	Hsph1	heat shock 105kDa/110kDa protein 1	179.5411	97.92735	101.8202	113.9336	72.20816	71.78597
17312485	Hsf1	heat shock factor 1	32.3912	33.1788	42.0257	32.20762	28.08679	30.77549
17233357	Hsf2	heat shock factor 2	58.5631	61.10083	62.3259	57.37936	56.248	63.75869
17506067	Hsbp1	heat shock factor binding protein 1	147.5735	224.1095	250.9333	190.9141	188.8281	190.5968
17355634	Hsbp1l1	heat shock factor binding protein 1-like 1	4.358676	3.923442	4.536658	4.184079	4.20927	4.111315
17443342	Hspb1	heat shock protein 1	31.31062	31.98758	39.8357	31.55792	22.58589	20.59397
17212882	Hspe1	heat shock protein 1 (chaperonin 10)	87.6839	116.7056	91.2492	120.6764	108.0706	120.0399
17223228	Hspd1	heat shock protein 1 (chaperonin)	452.5646	514.7542	484.751	544.9047	469.9326	514.9469
17365992	Hspa12a	heat shock protein 12A	5.367015	5.328797	4.917926	5.528967	5.328797	5.211288
17366008	Hspa12a	heat shock protein 12A	4.572046	4.228307	4.664524	4.093316	4.085209	4.67242
17376423	Hspa12b	heat shock protein 12B	9.438123	12.26028	10.39285	12.17627	13.11576	10.74132
17381235	Hspa14	heat shock protein 14	14.86523	13.94601	14.96468	15.46034	12.1107	15.46034
17344132	Hspa1a	heat shock protein 1A	28.90426	10.21183	8.467112	4.256389	4.809977	4.681043
17344126	Hspa1b	heat shock protein 1B	271.5982	125.7679	69.86558	35.39221	27.8924	18.10542
17336824	Hspa1l	heat shock protein 1-like	4.240634	4.647678	4.978151	4.774898	4.857689	3.891186
17276565	Hspa2	heat shock protein 2	13.06658	10.83442	16.72367	11.07358	14.87874	14.37958
17526923	Hspb2	heat shock protein 2	9.995647	12.58209	11.36401	9.76033	10.48228	10.48228
17296317	Hspb3	heat shock protein 3	8.115219	6.877384	6.992896	6.992896	6.992896	8.100495
17262621	Hspa4	heat shock protein 4	226.6371	199.2727	187.242	218.7744	188.9538	199.2727
17397240	Hspa4l	heat shock protein 4 like	66.7155	51.13609	45.10483	42.61236	30.56122	37.83265
17370103	Hspa5	heat shock protein 5	364.8855	153.867	149.3538	167.4423	181.7964	120.1165
17331533	Hspa13	heat shock protein 70 family, member 13	77.72032	38.77756	33.83255	33.93119	43.1315	35.73783
17516365	Hspa8	heat shock protein 8	1606.906	1391.138	1528.224	1264.744	1151.198	1176.063
17451816	Hspb8	heat shock protein 8	137.9757	164.5988	154.8395	150.5637	134.6509	150.075
17353554	Hspa9	heat shock protein 9	1179.075	1207.46	1197.06	1142.185	1164.093	1219.201
17345186	Hsp90ab1	heat shock protein 90 alpha (cytosolic), class B member 1	298.24	293.7551	285.0517	268.318	206.9746	274.0395
17370727	Gm13498	heat shock protein 90 alpha (cytosolic), class B member 1 pseudogene	3.177716	2.701742	2.410463	2.42627	2.913837	2.630451
17284002	Hsp90aa1	heat shock protein 90, alpha (cytosolic), class A member 1	116.0297	94.96079	90.75153	82.98278	53.97689	61.29553
17243738	Hsp90b1	heat shock protein 90, beta (Grp94), member 1	1729.441	1085.828	1046.931	1147.581	1225.02	1110.89
17416448	Hspb11	heat shock protein family B (small), member 11	16.18348	14.61239	17.54721	11.96667	19.77191	13.15838
17421074	Hspb7	heat shock protein family, member 7 (cardiovascular)	7.291876	10.63369	10.66876	13.43682	10.66876	11.13307
17476456	Hspb6	heat shock protein, alpha-crystallin-related, B6	51.27878	73.54982	77.87401	59.98584	42.0598	43.73056
17256436	Hspb9	heat shock protein, alpha-crystallin-related, B9	9.731553	9.438974	8.916454	9.3628	10.64482	11.75164
17343278	Hsf2bp	heat shock transcription factor 2 binding protein	4.008076	4.566187	4.382184	3.611004	4.840748	3.800476
17543376	Hsf3	heat shock transcription factor 3	2.406006	2.406006	2.372306	2.458111	2.458673	2.210542
17504688	Hsf4	heat shock transcription factor 4	18.16312	18.78748	19.79782	15.68822	18.53795	18.3396
17254801	Hsf5	heat shock transcription factor family member 5	5.477585	5.032342	5.371444	5.097783	7.279503	5.893072
17223279	Hsfy2	heat shock transcription factor, Y-linked 2	2.328224	2.242596	2.255238	2.255238	1.875844	2.216019
17316392	Hrsp12	heat-responsive protein 12	1472.376	1422.487	1414.082	1317.63	1200.658	1345.96

17518694	Herc1	hect (homologous to the E6-AP (UBE3A) carboxyl terminus) domain and RCC1 (CH	59.18091	61.77024	65.27235	58.20858	57.04743	52.61054
17478601	Herc2	hect (homologous to the E6-AP (UBE3A) carboxyl terminus) domain and RCC1 (CH	41.59793	38.64956	39.1937	41.55759	40.56237	41.59793
17233113	Hace1	HECT domain and ankyrin repeat containing, E3 ubiquitin protein ligase 1	5.925143	6.649427	5.343741	7.698994	4.611736	8.153672
17459038	Herc3	hect domain and RLD 3	12.73697	15.10694	12.90171	13.90171	16.16633	15.10694
17233888	Herc4	hect domain and RLD 4	108.6527	112.3184	99.57281	114.8542	104.8774	106.8283
17458962	Herc6	hect domain and RLD 6	65.28049	48.0745	68.76202	78.06587	79.98403	75.82754
17280980	Hectd1	HECT domain containing 1	341.114	290.0122	241.0385	288.5285	272.1032	277.9112
17358905	Hectd2	HECT domain containing 2	6.992896	5.919847	4.984504	6.530122	6.145371	7.02126
17417573	Hectd3	HECT domain containing 3	42.59316	45.02299	45.93621	44.66863	49.79331	49.11494
17290738	Hecw1	HECT, C2 and WW domain containing E3 ubiquitin protein ligase 1	4.801649	4.801649	4.583279	4.801649	4.445606	4.801649
17223080	Hecw2	HECT, C2 and WW domain containing E3 ubiquitin protein ligase 2	10.1427	12.09719	14.88255	14.36643	11.68763	11.78386
17538744	Huwe1	HECT, UBA and WWE domain containing 1	162.0865	183.8608	182.6313	170.7474	175.1758	164.5931
17231135	Hhat	hedgehog acyltransferase	5.295585	6.992896	7.521029	7.812856	7.347024	6.216058
17532367	Hhatl	hedgehog acyltransferase-like	7.160697	6.71022	5.863329	6.115583	7.190639	6.111261
17278529	Hhipl1	hedgehog interacting protein-like 1	12.09711	10.62411	11.51394	11.13315	11.40765	11.46465
17220464	Hhipl2	hedgehog interacting protein-like 2	3.403337	4.20927	4.20927	3.909272	4.515013	4.20927
17510772	Hhip	Hedgehog-interacting protein	9.939202	7.70161	7.169189	7.803871	6.177144	7.169189
17325069	Heg1	HEG homolog 1 (zebrafish)	52.2772	52.36896	69.4908	52.48743	49.21479	36.20633
17245383	Helb	helicase (DNA) B	23.85252	25.40059	23.2006	27.83446	24.27451	21.23865
17395801	Helz2	helicase with zinc finger 2, transcriptional coactivator	70.13827	74.70071	72.63686	88.0945	74.19705	64.12211
17257683	Helz	helicase with zinc finger domain	37.69366	38.98399	39.48684	38.98399	39.87742	36.01535
17359212	Hells	helicase, lymphoid specific	4.966295	4.259193	5.120341	6.274324	4.912032	6.444373
17450157	Helq	helicase, POLQ-like	16.17989	15.82386	14.77504	17.62706	16.23595	16.23595
17396287	Hltf	helicase-like transcription factor	36.51077	36.72753	35.39712	33.6119	35.01813	34.22195
17509182	Helt	helt bHLH transcription factor	4.017617	3.371732	3.306099	3.894559	3.41861	3.41861
17342101	Hn1l	hematological and neurological expressed 1-like	66.50358	68.53742	58.71349	64.22798	81.03482	62.95242
17241799	1700049L16Rik	hematological and neurological expressed 1-like pseudogene	5.16386	7.520507	4.762701	4.67404	3.984172	7.462867
17271920	Hn1	hematological and neurological expressed sequence 1	20.12114	21.05061	27.65235	20.78744	19.74328	21.42911
17489046	Hcst	hematopoietic cell signal transducer	9.46131	8.967961	11.18025	10.10425	10.41167	7.472184
17325438	Hcls1	hematopoietic cell specific Lyn substrate 1	20.9615	23.45527	22.9285	20.29525	28.32728	21.96143
17467269	Hpgds	hematopoietic prostaglandin D synthase	5.48933	5.48933	5.916279	5.48933	6.450943	5.48933
17502406	Hsh2d	hematopoietic SH2 domain containing	4.844138	4.42464	4.844138	6.969668	4.401392	4.502793
17359045	Hhex	hematopoietically expressed homeobox	68.77251	58.35765	39.06244	50.10352	48.56348	44.4928
17472063	Hebp1	heme binding protein 1	169.0622	176.3637	171.4257	187.1356	191.9212	196.6982
17239539	Hebp2	heme binding protein 2	5.421304	5.820528	5.635095	5.800712	5.935872	5.702522
17502573	Hmox1	heme oxygenase 1	32.83289	75.00111	69.35558	36.19529	36.84406	24.31526
17322559	Hmox2	heme oxygenase 2	59.23878	60.63337	59.79902	63.44216	61.82335	61.30658
17227910	Hmcn1	hemicentin 1	5.042293	5.515073	5.159782	5.463366	4.63738	5.042293
17369379	Hmcn2	hemicentin 2	7.662951	7.698994	7.663322	8.49533	8.170985	7.663322
17530884	Hemk1	HemK methyltransferase family member 1	11.19009	12.58715	12.2841	18.11835	14.37709	15.66171
17291211	Hfe	hemochromatosis	60.56112	60.56112	59.69094	65.29452	59.47906	64.88261
17400592	Hfe2	hemochromatosis type 2 (juvenile) (human homolog)	146.9692	144.2153	182.0057	197.7961	155.3814	162.2877
17425028	Hemgn	hemogen	4.487805	3.984255	4.365812	4.814213	6.5129	5.792716
17248263	Hba-a1	hemoglobin alpha, adult chain 1	29.29454	49.21513	49.00986	41.93259	29.34433	19.66666
17248276	Hba-a2	hemoglobin alpha, adult chain 2	753.1482	1217.183	1798.315	980.6062	375.6434	87.20023
17494236	Hbb-bh2	hemoglobin beta, bh2	3.344638	3.362024	3.481165	4.110005	4.488054	3.38018
17248255	Hba-x	hemoglobin X, alpha-like embryonic chain in Hba complex	3.032211	3.006698	3.279123	3.263839	2.721402	2.795216
17494246	Hbb-y	hemoglobin Y, beta-like embryonic chain	3.29754	3.533162	2.67402	3.471767	3.209703	3.85206
17494240	Hbb-bh1	hemoglobin Z, beta-like embryonic chain	2.884517	4.263801	4.604996	4.508164	5.33508	5.492527
17494228	Hbb-bs	hemoglobin, beta adult s chain	3.315117	2.887738	3.615838	3.199885	2.703035	2.357291
17494221	Hbb-bt	hemoglobin, beta adult t chain	21.49352	31.5798	37.36304	20.79565	8.320556	2.736345
17248282	Hbq1a	hemoglobin, theta 1A	3.50601	5.248445	5.389121	4.129651	4.770069	5.011213
17248270	Hbq1b	hemoglobin, theta 1B	10.28272	13.02201	11.25949	7.764968	10.66876	10.98125
17384374	Hc	hemolytic complement	1493.229	1435.393	1336.533	1260.456	1461.607	1096.72
17494528	Hpx	hemopexin	3561.502	4074.056	4417.242	3212.053	4293.891	3345.184
17377870	Hck	hemopoietic cell kinase	33.99098	22.14824	27.89162	31.04459	22.03049	24.65964
17401805	Henmt1	HEN1 methyltransferase homolog 1 (Arabidopsis)	4.118332	4.080427	4.969153	4.046852	3.958682	4.078165
17464539	Hepacam2	HEPACAM family member 2	4.186603	3.450822	3.494752	3.559291	3.726471	3.531179
17447726	Hs3st1	heparan sulfate (glucosamine) 3-O-sulfotransferase 1	4.496897	3.655959	4.184514	3.787485	3.460739	4.20927
17482669	Hs3st2	heparan sulfate (glucosamine) 3-O-sulfotransferase 2	3.803137	4.532792	4.224879	5.071034	4.128907	5.362962
17250871	Hs3st3a1	heparan sulfate (glucosamine) 3-O-sulfotransferase 3A1	12.356	11.88187	11.15153	11.93867	7.870417	11.82723
17264102	Hs3st3b1	heparan sulfate (glucosamine) 3-O-sulfotransferase 3B1	100.7621	86.69218	101.7729	88.38012	117.7244	97.7368
17482907	Hs3st4	heparan sulfate (glucosamine) 3-O-sulfotransferase 4	16.55747	18.76757	16.72959	15.16633	16.91241	17.72803
17232549	Hs3st5	heparan sulfate (glucosamine) 3-O-sulfotransferase 5	2.781359	3.54506	3.302732	4.32494	3.263862	3.407444
17334427	Hs3st6	heparan sulfate (glucosamine) 3-O-sulfotransferase 6	19.01268	22.89324	18.79269	18.76536	16.28453	14.85905
17410818	Hs2st1	heparan sulfate 2-O-sulfotransferase 1	26.63497	23.22087	24.05442	23.69893	25.75124	20.78297
17211790	Hs6st1	heparan sulfate 6-O-sulfotransferase 1	136.6262	110.2787	128.7225	114.9849	148.3612	114.8375
17541636	Hs6st2	heparan sulfate 6-O-sulfotransferase 2	4.559893	3.883318	4.119102	3.89907	5.321268	3.610078
17302747	Hs6st3	heparan sulfate 6-O-sulfotransferase 3	7.316634	5.29687	6.139921	5.57681	4.974115	6.151635
17508445	Hgsnat	heparan-alpha-glucosaminide N-acetyltransferase	79.66477	78.07561	77.88157	68.95389	80.14219	80.09832
17450142	Hpse	heparanase	32.70796	34.30365	32.70796	33.6394	32.28137	21.34816
17364917	Hpse2	heparanase 2	6.78557	7.738532	6.78557	8.932971	7.546407	8.119101
17353699	Hbegf	heparin-binding EGF-like growth factor	13.45742	12.31803	14.46165	14.18988	14.31296	14.18988
17267702	Hlf	hepatic leukemia factor	45.09107	44.74951	56.24839	51.74796	52.63316	49.38531
17394041	Hnf4aos	hepatic nuclear factor 4 alpha, opposite strand	15.61521	21.09487	22.18481	22.61926	15.79231	13.74762
17379268	Hnf4a	hepatic nuclear factor 4, alpha	492.8552	535.5621	533.4429	517.2543	517.2543	476.2978
17248847	Havcr1	hepatitis A virus cellular receptor 1	3.460763	3.516256	4.337038	4.332651	4.420716	4.21926
17248809	Havcr2	hepatitis A virus cellular receptor 2	5.338965	4.022326	4.892019	4.330519	5.229888	4.606401
17516036	Hepacam	hepatocyte cell adhesion molecule	4.507133	4.860787	4.647678	4.647678	4.334694	5.737133
17434933	Hgf	hepatocyte growth factor	63.83121	64.24312	56.35571	50.10039	29.89599	35.21236
17436793	Hgfac	hepatocyte growth factor activator	142.1539	124.9477	124.3463	121.1609	167.5084	136.9903
17395955	Hnf4g	hepatocyte nuclear factor 4, gamma	3.211016	2.953407	3.009972	3.041768	3.533579	3.842779
17291408	Hdgfl1	hepatoma derived growth factor-like 1	7.206007	6.349443	6.552475	7.669593	6.043691	4.948221
17398863	Hdgf	hepatoma-derived growth factor	190.4238	157.593	168.7952	156.5631	145.2053	148.8123

17338715	Hdgfrp2	hepatoma-derived growth factor, related protein 2	48.19161	46.18972	46.20381	52.99153	50.30465	51.43826
17492861	Hdgfrp3	hepatoma-derived growth factor, related protein 3	5.901779	7.007889	6.07435	6.758251	5.772595	6.281852
17489363	Hamp	hepcidin antimicrobial peptide	422.7614	305.4368	106.3525	1263.246	1095.414	3871.186
17489357	Hamp2	hepcidin antimicrobial peptide 2	6.305409	6.075824	6.555019	16.37236	9.658104	134.2641
17536390	Heph	hephaestin	7.684226	6.288823	6.287864	6.287864	5.65756	8.420524
17524117	Hephl1	hephaestin-like 1	4.222069	4.486032	3.808492	4.162066	3.451898	3.874661
17489441	Hpn	hepsin	148.1906	176.3295	183.9245	190.2417	188.8164	173.3195
17364890	Hps1	Hermansky-Pudlak syndrome 1 homolog (human)	10.0091	9.210886	9.043116	11.29021	9.711472	10.35101
17404300	Hps3	Hermansky-Pudlak syndrome 3 homolog (human)	16.7822	19.07274	20.47157	18.57834	15.41411	17.26131
17440692	Hps4	Hermansky-Pudlak syndrome 4 homolog (human)	15.93061	21.83227	23.10879	24.57518	16.89307	36.62863
17491211	Hps5	Hermansky-Pudlak syndrome 5 homolog (human)	19.72318	25.11975	18.1372	18.17622	16.65115	15.61986
17359874	Hps6	Hermansky-Pudlak syndrome 6	9.596112	9.650877	7.708392	11.44638	12.44505	11.82657
17525075	Herpud2	HERPUD family member 2	123.2078	109.3508	118.9103	112.5787	112.2911	114.4941
17317574	Hhla1	HERV-H LTR-associating 1	3.789139	3.981881	3.613758	4.060423	3.922518	3.752313
17420425	Hp1bp3	heterochromatin protein 1, binding protein 3	53.77971	57.30345	64.31128	61.361	61.361	67.20935
17262478	Hnrnpab	heterogeneous nuclear ribonucleoprotein A/B	377.9836	345.5427	311.6213	325.5962	312.4085	327.0778
17293085	Hnrnpa0	heterogeneous nuclear ribonucleoprotein A0	65.16927	66.32358	58.46657	54.22777	73.69118	68.2279
17315546	Hnrnpa1	heterogeneous nuclear ribonucleoprotein A1	108.6798	141.8734	159.6258	140.9686	143.7392	143.9456
17532524	Gm10052	heterogeneous nuclear ribonucleoprotein A1 pseudogene	134.8994	183.6863	216.4095	182.3833	228.4952	188.8949
17538295	Gm5643	heterogeneous nuclear ribonucleoprotein A1 pseudogene	2.051743	2.371632	2.125791	2.337733	2.765039	2.255629
17466768	Hnrnpa2b1	heterogeneous nuclear ribonucleoprotein A2/B1	256.5436	302.0325	275.5729	249.9681	251.6893	291.0231
17372140	Hnrnpa3	heterogeneous nuclear ribonucleoprotein A3	57.98147	70.22535	63.14996	60.15614	78.68791	62.77508
17513228	Gm6793	heterogeneous nuclear ribonucleoprotein A3 pseudogene	3.435047	4.031481	5.063026	8.209191	7.627869	5.26725
17382354	LOC102638359	heterogeneous nuclear ribonucleoprotein A3 pseudogene	3.463883	4.889951	6.18537	5.415933	4.787094	7.977242
17306189	Hnrnpc	heterogeneous nuclear ribonucleoprotein C	163.0364	148.317	149.9877	175.1989	176.244	166.7127
17450022	Hnrnpd	heterogeneous nuclear ribonucleoprotein D	81.26431	84.62957	78.56746	82.95862	77.67293	80.87545
17450034	Hnrnpdl	heterogeneous nuclear ribonucleoprotein D-like	57.16103	69.00744	81.1253	74.26703	74.15849	70.02994
17462191	Hnrnpf	heterogeneous nuclear ribonucleoprotein F	107.4923	96.68901	102.5952	91.92084	99.45762	104.2165
17548525	Hnrnpf	heterogeneous nuclear ribonucleoprotein F	49.65063	50.03341	49.58837	46.21727	61.22971	49.61725
17249176	Hnrnph1	heterogeneous nuclear ribonucleoprotein H1	61.65517	73.60439	64.9303	59.64751	50.50555	69.07492
17537722	Hnrnph2	heterogeneous nuclear ribonucleoprotein H2	62.06896	57.60412	63.5064	62.06896	56.25736	63.9295
17241534	Hnrnph3	heterogeneous nuclear ribonucleoprotein H3	51.35348	79.14959	74.71417	81.84997	68.05787	91.57678
17293142	Hnrnpk	heterogeneous nuclear ribonucleoprotein K	58.15656	68.42777	75.08225	68.97301	72.00823	57.64845
17475956	Hnrnpl	heterogeneous nuclear ribonucleoprotein L	111.5696	140.95	132.4928	132.8947	141.8734	138.9691
17347475	Hnrnpll	heterogeneous nuclear ribonucleoprotein L-like	44.90923	44.90923	37.66911	39.93883	51.40262	49.3812
17343580	Hnrnpm	heterogeneous nuclear ribonucleoprotein M	113.9494	122.535	126.5514	122.637	128.0586	142.874
17420091	Hnrnpr	heterogeneous nuclear ribonucleoprotein R	37.25985	41.2225	40.74073	37.94558	43.35571	43.37795
17420106	Hnrnpr	heterogeneous nuclear ribonucleoprotein R	5.113	6.992896	5.937353	5.362218	8.580966	8.420524
17420108	Hnrnpr	heterogeneous nuclear ribonucleoprotein R	4.378024	4.883448	5.020914	4.80951	6.898296	5.063939
17230279	Hnrnpu	heterogeneous nuclear ribonucleoprotein U	35.47892	70.37202	57.66044	76.80509	59.63387	91.63393
17230282	Hnrnpu	heterogeneous nuclear ribonucleoprotein U	350.8839	404.0976	344.8583	388.489	382.5965	410.2473
17488013	Hnrnpul1	heterogeneous nuclear ribonucleoprotein U-like 1	137.5126	141.781	143.6775	128.4168	130.5465	140.3767
17357219	Hnrnpul2	heterogeneous nuclear ribonucleoprotein U-like 2	92.73423	110.7045	107.5989	108.8132	114.5989	125.2928
17257085	Hexim1	hexamethylene bis-acetamide inducible 1	70.39153	88.22894	71.79592	75.19371	75.49452	66.38107
17257089	Hexim2	hexamethylene bis-acetamide inducible 2	7.814535	7.667679	7.747495	7.311006	7.19767	10.58163
17241318	Hk1	hexokinase 1	7.003179	8.410899	10.84968	10.46217	7.228468	7.541963
17233783	Hk1os	hexokinase 1, opposite strand	9.731553	9.40384	10.12858	10.16043	10.44065	10.19952
17468018	Hk2	hexokinase 2	11.42704	11.62622	13.86245	12.23244	10.3961	13.92697
17292775	Hk3	hexokinase 3	11.7908	14.18073	12.43918	12.10927	9.889379	13.70025
17241355	Hkdc1	hexokinase domain containing 1	10.12725	11.02449	10.58713	8.857319	8.815852	8.787178
17259563	Hexdc	hexosaminidase (glycosyl hydrolase family 20, catalytic domain) containing	12.99883	16.87412	16.93117	16.2011	16.2011	14.7859
17517947	Hexa	hexosaminidase A	150.7851	179.4191	196.7541	163.1539	167.685	139.9085
17295278	Hexb	hexosaminidase B	34.82834	46.4131	54.72811	48.01423	53.08381	51.88588
17433251	H6pd	hexose-6-phosphate dehydrogenase (glucose 1-dehydrogenase)	280.0246	263.6737	237.753	253.7739	241.815	200.5808
17450572	Hfm1	HFM1, ATP-dependent DNA helicase homolog (S. cerevisiae)	2.508491	2.66139	2.255364	3.176416	2.713171	2.679883
17259344	Hgs	HGF-regulated tyrosine kinase substrate	77.44284	69.43976	73.51313	66.28773	72.79664	60.23583
17312418	Hgh1	HGH1 homolog	16.39064	16.09377	18.80338	16.90608	16.59205	14.17641
17271861	Hid1	HID1 domain containing	9.729462	10.01916	9.735331	7.688222	6.680981	8.05799
17532403	Higd1a	HIG1 domain family, member 1A	26.83548	24.99227	24.32504	32.52597	25.0436	26.51065
17257026	Higd1b	HIG1 domain family, member 1B	6.413092	6.008563	6.7635	6.7635	6.992896	7.630248
17315012	Higd1c	HIG1 domain family, member 1C	4.718146	4.412848	5.076274	4.20927	4.113852	3.89437
17287466	Higd2a	HIG1 domain family, member 2A	166.2799	208.7135	222.0914	203.4513	188.2949	200.8424
17225728	Hdlbp	high density lipoprotein (HDL) binding protein	892.6619	749.456	725.5946	683.7571	686.031	649.7946
17537527	4921511C20Rik	high density lipoprotein (HDL) binding protein pseudogene	3.531106	2.860882	2.360676	2.5951	3.131578	2.547196
17517576	Hmg20a	high mobility group 20A	20.26783	21.62368	27.42405	21.71349	17.67098	18.29212
17243286	Hmg20b	high mobility group 20B	31.55638	37.37107	36.37991	36.87957	41.47212	34.03908
17335129	Hmga1	high mobility group AT-hook 1	27.99466	28.56111	27.29264	32.29435	34.49439	27.07989
17245422	Hmga2	high mobility group AT-hook 2	9.772245	12.34136	8.945673	11.11434	11.49648	10.97524
17230225	Hmga2-ps1	high mobility group AT-hook 2, pseudogene 1	4.348567	5.101353	4.681043	4.801649	4.681043	4.612152
17259507	Hmga1-rs1	high mobility group AT-hook I, related sequence 1	55.68358	61.68766	58.55376	68.16267	51.53033	42.2064
17455476	Hmgb1	high mobility group box 1	55.29434	51.68737	51.5557	53.31095	60.61614	60.82785
17547917	Hmgb1	high mobility group box 1	506.0935	618.5644	624.1894	539.2797	784.5546	725.0014
17548488	Hmgb1	high mobility group box 1	592.0807	744.5784	740.9128	710.3174	944.7115	861.2331
17547787	Hmgb1-ps9	high mobility group box 1, pseudogene 9 [Source:MGI Symbol;Acc:MGI:96115]	349.1613	426.665	440.0397	389.8107	483.238	475.3512
17500451	Hmgb1-rs17	high mobility group box 1, related sequence 17	2.764349	2.538507	2.318401	2.249057	2.339289	2.393646
17501292	Hmgb2	high mobility group box 2	11.53999	11.63049	13.58855	9.474107	15.6829	13.44334
17237275	Gm5176	high mobility group box 2 pseudogene	4.626432	5.213275	4.586743	5.22938	5.908779	3.531938
17535344	Hmgb3	high mobility group box 3	10.10159	14.23075	12.94246	10.58405	16.33416	7.995731
17280577	Hbp1	high mobility group box transcription factor 1	147.364	135.4804	128.294	132.547	135.6128	126.9283
17332447	Hmgn1	high mobility group nucleosomal binding domain 1	63.49055	71.98777	67.86802	63.39073	95.2185	69.46238
17431136	Hmgn2	high mobility group nucleosomal binding domain 2	3.462322	3.480513	3.91703	4.801316	3.937364	5.005173
17510494	Hmgn2	high mobility group nucleosomal binding domain 2	4.278722	5.396709	4.531504	3.161107	4.720599	6.797951
17529276	Hmgn3	high mobility group nucleosomal binding domain 3	7.70177	7.356094	6.265632	8.626946	8.591023	10.19458
17544196	Hdx	highly divergent homeobox	3.197224	2.900577	2.734638	3.160494	3.892346	3.358577

17430253	Hmgb4	high-mobility group box 4	6.175261	5.831593	6.140048	5.347298	6.494147	6.157608
17418850	Hmgb4os	high-mobility group box 4, opposite strand	3.398175	3.54506	3.339703	3.255886	4.119474	3.490902
17476998	Hmgb1-ps7	high-mobility group high mobility group box 1, pseudogene 7	42.50469	52.63826	47.09298	47.79209	62.52105	52.09453
17544149	Hmgn5	high-mobility group nucleosome binding domain 5	23.31279	25.03683	24.55543	30.69943	32.34117	38.71632
17430307	Hpca	hippocalcin	9.79829	9.79829	9.79829	11.03466	7.268361	11.6342
17274310	Hpcal1	hippocalcin-like 1	17.10291	21.53558	21.698	14.38965	26.60748	17.21161
17418158	Hpcal4	hippocalcin-like 4	5.374945	5.380574	5.615444	6.174404	4.525728	5.959908
17409721	Hiat1	hippocampus abundant gene transcript 1	76.27826	97.23744	80.27562	82.08177	95.07736	86.80162
17293757	Hiatl1	hippocampus abundant transcript-like 1	37.04251	33.01798	32.9743	32.80901	31.169	30.41898
17483165	Hirip3	HIRA interacting protein 3	4.84058	5.03468	4.353181	3.824948	5.131782	6.018317
17382203	Hnmt	histamine N-methyltransferase	54.85077	53.69255	50.46915	56.57842	45.60184	51.66517
17461888	Hrh1	histamine receptor H1	9.361307	9.361307	7.645442	9.361307	12.74545	10.47726
17287426	Hrh2	histamine receptor H2	5.49584	6.905711	7.527809	6.951094	6.150569	6.780962
17395499	Hrh3	histamine receptor H3	11.66399	14.73393	13.47142	15.41529	16.08061	14.22947
17348618	Hrh4	histamine receptor H4	2.67402	2.693029	2.67402	2.72962	3.287065	3.496884
17236552	Hal	histidine ammonia lyase	571.722	605.5406	557.7134	634.2642	632.4817	674.381
17391094	Hdc	histidine decarboxylase	10.43725	11.49967	9.196277	10.66876	7.706149	7.881389
17477774	Hrc	histidine rich calcium binding protein	9.430864	7.22346	6.425996	9.711068	7.081574	8.438228
17413458	Hrct1	histidine rich carboxyl terminus 1	17.73245	16.77627	20.2399	18.52549	15.64761	16.61483
17249780	Hint1	histidine triad nucleotide binding protein 1	219.811	222.7405	217.4003	222.2439	242.0666	198.3961
17424782	Hint2	histidine triad nucleotide binding protein 2	14.02521	16.62232	14.15235	16.69518	16.58952	16.66986
17240045	Hint3	histidine triad nucleotide binding protein 3	35.89945	31.74594	30.44662	31.90875	32.42849	35.4364
17324344	Hrg	histidine-rich glycoprotein	1970.191	1897.179	1833.469	1819.315	1824.101	1891.166
17353765	Hars	histidyl-tRNA synthetase	100.0419	86.06651	78.98025	90.25219	104.4291	81.45267
17349774	Hars2	histidyl-tRNA synthetase 2, mitochondrial (putative)	24.82772	28.34181	24.20327	27.48266	27.36424	29.09864
17235136	Hmha1	histocompatibility (minor) HA-1	17.19384	17.19384	18.19689	13.43598	17.19384	16.95983
17377753	H13	histocompatibility 13	174.2204	153.5378	142.6731	140.1046	141.2013	123.224
17344578	H2-Bl	histocompatibility 2, blastocyst	4.208686	5.466093	4.371808	4.987027	4.520085	5.075676
17343813	H2-Aa	histocompatibility 2, class II antigen A, alpha	31.80166	31.44001	33.56347	24.47403	30.48928	29.14305
17336494	H2-Ab1	histocompatibility 2, class II antigen A, beta 1	105.0222	79.4431	86.79801	70.54539	80.09443	77.87023
17343827	H2-Ea-ps	histocompatibility 2, class II antigen E alpha, pseudogene	3.325497	4.866923	3.817134	4.441848	3.073601	3.736118
17336502	H2-Eb1	histocompatibility 2, class II antigen E beta	64.21908	55.19057	57.00909	42.52296	53.19357	56.36118
17336509	H2-Eb2	histocompatibility 2, class II antigen E beta2	4.446684	3.820523	4.580135	3.656466	4.553481	3.856575
17336407	H2-DMa	histocompatibility 2, class II, locus DMa	11.17111	11.9549	12.04915	11.50086	9.473516	11.66246
17336422	H2-DMb1	histocompatibility 2, class II, locus Mb1	4.884836	3.695245	4.69622	4.20927	4.813079	3.128476
17336414	H2-DMb2	histocompatibility 2, class II, locus Mb2	6.585578	6.889456	6.285903	6.889456	6.889456	6.170083
17337065	H2-D1	histocompatibility 2, D region locus 1	16.80292	18.64284	20.83822	17.76776	20.13886	18.64284
17343703	H2-K2	histocompatibility 2, K region locus 2	13.9434	19.17574	21.12457	18.29579	18.76679	17.79821
17343710	H2-K1	histocompatibility 2, K1, K region	461.1621	492.2304	481.7497	428.5828	475.6346	481.009
17344757	H2-M1	histocompatibility 2, M region locus 1	2.78433	2.239691	3.207991	2.960047	3.55902	2.735207
17344728	H2-M10.1	histocompatibility 2, M region locus 10.1	26.257	25.30271	27.19086	28.34122	17.03571	24.06309
17344720	H2-M10.2	histocompatibility 2, M region locus 10.2	2.913514	2.607234	2.506388	3.297151	4.156502	3.68877
17344736	H2-M10.3	histocompatibility 2, M region locus 10.3	5.852528	6.27911	4.654319	4.556576	4.632561	4.872729
17344743	H2-M10.4	histocompatibility 2, M region locus 10.4	3.403502	2.709488	2.979809	3.518499	3.117544	4.323501
17337427	H2-M10.5	histocompatibility 2, M region locus 10.5	3.02371	3.015339	3.366338	2.606909	3.451874	3.222343
17337434	H2-M10.6	histocompatibility 2, M region locus 10.6	2.821632	2.586695	2.809435	3.485462	3.623591	3.219106
17337411	H2-M11	histocompatibility 2, M region locus 11	2.826409	2.55487	3.035323	2.652826	2.825566	2.5205
17344873	H2-M2	histocompatibility 2, M region locus 2	5.456554	4.75093	5.771955	5.470869	6.70478	5.531114
17337557	H2-M3	histocompatibility 2, M region locus 3	15.37303	15.27023	15.30997	18.04003	15.65238	16.90514
17344811	H2-M5	histocompatibility 2, M region locus 5	8.237405	9.610757	8.512955	7.755609	8.512955	7.48914
17344750	H2-M9	histocompatibility 2, M region locus 9	3.009972	3.464795	3.009972	2.886758	3.009972	2.773351
17336396	H2-Oa	histocompatibility 2, O region alpha locus	6.700831	7.06652	6.38559	6.859769	6.318797	5.594077
17336476	H2-Ob	histocompatibility 2, O region beta locus	6.171709	6.61926	5.429879	6.943774	6.775794	6.42849
17343755	H2-Pb	histocompatibility 2, P region beta locus	3.223884	2.797717	2.588309	4.03028	3.339703	3.339703
17337089	H2-Q1	histocompatibility 2, Q region locus 1	25.11818	34.42775	33.06401	36.65175	32.50816	44.14628
17337142	H2-Q10	histocompatibility 2, Q region locus 10	42.97215	52.59122	57.93647	45.16803	43.31005	46.49456
17337100	H2-Q4	histocompatibility 2, Q region locus 4	32.49442	26.98377	32.18909	32.49442	37.29447	32.58283
17337110	H2-Q5	histocompatibility 2, Q region locus 5	8.607653	15.23925	13.75839	9.314317	9.310251	12.29645
17337118	H2-Q5	histocompatibility 2, Q region locus 5	14.91229	12.78961	7.769329	5.924322	7.19358	9.037727
17337120	H2-Q5	histocompatibility 2, Q region locus 5	668.3639	638.1006	677.1398	649.7676	640.5734	656.9319
17337133	H2-Q7	histocompatibility 2, Q region locus 7	24.74663	23.10671	24.17761	21.29481	16.90034	11.24366
17337122	H2-Q8	histocompatibility 2, Q region locus 8	51.80107	48.32273	54.69852	57.35113	48.90974	52.88585
17344642	H2-T10	histocompatibility 2, T region locus 10	15.58212	14.27254	15.01029	26.11651	22.75829	25.43759
17344593	H2-T22	histocompatibility 2, T region locus 22	124.7616	94.86342	130.2564	109.9611	104.8047	115.4945
17344568	H2-T23	histocompatibility 2, T region locus 23	94.71034	81.2339	87.1241	95.20464	104.0692	102.8523
17344558	H2-T24	histocompatibility 2, T region locus 24	10.2598	7.70193	11.43298	12.58096	5.777679	12.40836
17344608	H2-T3	histocompatibility 2, T region locus 3	3.839478	3.492621	3.893371	5.239903	3.111607	4.116619
17232102	H60b	histocompatibility 60b	5.064711	4.355294	3.387263	4.79018	3.904831	4.724331
17238722	H60c	histocompatibility 60c	5.074058	4.545818	5.589968	4.109891	3.390787	4.863079
17371739	Hat1	histone aminotransferase 1	30.24742	22.45788	24.11953	24.68239	25.26275	25.88707
17323782	Hira	histone cell cycle regulation defective homolog A (S. cerevisiae)	26.95816	33.7714	29.16103	27.37771	29.16103	26.56104
17285879	Hist1h1a	histone cluster 1, H1a	2.717029	3.030131	4.111743	2.953407	3.729055	2.703803
17291005	Hist1h1b	histone cluster 1, H1b	3.262731	2.162215	2.132758	2.328418	3.667508	2.396917
17285859	Hist1h1c	histone cluster 1, H1c	49.02888	31.0504	46.03662	44.78675	41.1691	34.22859
17285829	Hist1h1d	histone cluster 1, H1d	4.726591	4.50832	3.624068	5.208327	4.582707	4.689682
17285855	Hist1h1t	histone cluster 1, H1t	2.94862	3.84699	3.62447	3.916161	3.239206	3.193083
17285938	Hist1h2aa	histone cluster 1, H2aa	12.16572	8.039635	12.50662	11.12659	6.225803	17.38776
17285867	Hist1h2ab	histone cluster 1, H2ab	3.465347	4.353694	3.351879	2.490163	2.813527	3.682588
17291204	Hist1h2ac	histone cluster 1, H2ac	5.23326	6.611053	5.083739	5.056328	5.896137	4.594921
17285838	Hist1h2ad	histone cluster 1, H2ad	2.799308	2.248714	2.281828	2.42479	3.753483	2.143577
17291186	Hist1h2ae	histone cluster 1, H2ae	2.67402	2.672463	2.557599	2.49258	2.366902	2.221355
17285819	Hist1h2af	histone cluster 1, H2af	47.15504	63.02577	75.41032	55.49747	67.01165	77.48204
17291057	Hist1h2ag	histone cluster 1, H2ag	3.865326	3.049029	2.24549	2.370596	2.91212	2.994386
17291049	Hist1h2ah	histone cluster 1, H2ah	2.201001	2.049099	2.078338	1.874012	2.545608	2.248246

17285677	Hist1h2ai	histone cluster 1, H2ai	2.269509	2.171377	2.868825	2.085799	2.297731	2.771928
17291001	Hist1h2ak	histone cluster 1, H2ak	2.060051	2.060051	2.597349	1.78627	2.060051	2.024225
17292543	Hist1h2al	histone cluster 1, H2al	4.392493	3.883991	4.212597	4.732361	6.030928	5.646669
17291012	Hist1h2an	histone cluster 1, H2an	63.64962	89.44259	121.2121	84.20185	77.72032	83.75829
17285703	Hist1h2ao	histone cluster 1, H2ao	69.33923	69.4465	83.39795	70.55958	79.52243	86.16977
17291023	Hist1h2ao	histone cluster 1, H2ao	69.33923	69.4465	83.39795	70.55958	79.52243	86.16977
17291258	Hist1h2ba	histone cluster 1, H2ba	2.167452	2.312382	1.804664	2.111883	1.813316	2.360185
17285863	Hist1h2bb	histone cluster 1, H2bb	7.265842	7.698994	6.307682	5.275983	3.444752	6.110456
17285851	Hist1h2bc	histone cluster 1, H2bc	1152.755	1006.806	1001.94	973.5289	984.5154	1021.144
17291195	Hist1h2be	histone cluster 1, H2be	9.556948	10.22061	7.928621	11.32603	10.17002	11.52033
17291190	Hist1h2bf	histone cluster 1, H2bf	10.82339	8.832734	9.310251	8.964729	6.90351	9.920824
17285834	Hist1h2bg	histone cluster 1, H2bg	8.075099	13.39962	7.631571	9.764188	9.337394	13.78464
17291177	Hist1h2bh	histone cluster 1, H2bh	2.199248	2.40204	3.286655	3.080704	5.341	2.719271
17285746	Hist1h2bj	histone cluster 1, H2bj	2.995444	2.547708	2.154003	2.606909	3.200373	1.743214
17285742	Hist1h2bk	histone cluster 1, H2bk	2.846837	4.72073	5.186819	4.682244	7.760341	4.757257
17290997	Hist1h2bl	histone cluster 1, H2bl	376.3523	314.5226	359.7224	294.2462	243.0063	327.0257
17285683	Hist1h2bm	histone cluster 1, H2bm	2.351759	2.690119	4.780959	2.844391	3.328956	2.299053
17285691	Hist1h2bn	histone cluster 1, H2bn	310.6314	235.5991	289.2434	224.6943	218.1021	240.2096
17285696	Hist1h2bp	histone cluster 1, H2bp	2.860743	3.780744	3.966345	3.733814	3.716875	3.608952
17285710	Hist1h2bq	histone cluster 1, H2bq	9.123011	7.755501	8.295334	7.617301	6.27763	6.6928
17291014	Hist1h2bq	histone cluster 1, H2bq	9.123011	7.755501	8.295334	7.617301	6.27763	6.6928
17291233	Hist1h3a	histone cluster 1, H3a	2.255364	2.255364	2.255364	2.140919	2.462698	1.938392
17285871	Hist1h3b	histone cluster 1, H3b	3.937064	3.012915	3.435738	3.045375	3.435357	3.591608
17291222	Hist1h3c	histone cluster 1, H3c	2.245256	2.25552	1.907046	2.301445	3.269319	2.317244
17285842	Hist1h3d	histone cluster 1, H3d	3.452975	3.040545	2.962202	3.915075	3.316565	2.629503
17291183	Hist1h3e	histone cluster 1, H3e	3.470973	2.67402	3.060739	2.990942	2.360169	3.043835
17285825	Hist1h3f	histone cluster 1, H3f	2.719912	2.026682	2.255364	2.255364	2.608535	2.099171
17285821	Hist1h3g	histone cluster 1, H3g	1.942857	2.253707	2.228526	2.161511	2.476736	1.878707
17285680	Hist1h3h	histone cluster 1, H3h	18.16312	16.16935	15.74596	16.90444	16.16935	14.76849
17291008	Hist1h3i	histone cluster 1, H3i	2.769624	1.996454	2.52699	2.038249	1.88956	2.479931
17291229	Hist1h4a	histone cluster 1, H4a	2.888819	2.953407	3.091892	3.263862	3.558058	3.045396
17285875	Hist1h4b	histone cluster 1, H4b	2.429248	2.981379	2.346483	2.047694	2.281433	2.666413
17291208	Hist1h4c	histone cluster 1, H4c	3.751766	3.216652	3.090307	3.157231	3.266917	2.959985
17285846	Hist1h4d	histone cluster 1, H4d	4.519145	1.930133	2.992705	2.387201	3.79216	2.704628
17291180	Hist1h4f	histone cluster 1, H4f	2.790744	2.768338	5.286563	3.579778	3.286404	2.640351
17285815	Hist1h4h	histone cluster 1, H4h	2.990486	3.392973	3.397774	3.851125	2.505173	2.807177
17291053	Hist1h4i	histone cluster 1, H4i	17.77146	24.23852	23.07886	20.40315	13.54661	22.65488
17285687	Hist1h4j	histone cluster 1, H4j	2.411833	3.435881	3.157428	2.461623	2.835769	2.288592
17290999	Hist1h4k	histone cluster 1, H4k	166.6538	155.3609	194.4142	140.9071	138.0943	168.4481
17285706	Hist1h4m	histone cluster 1, H4m	6.262332	6.597823	8.171098	7.43679	7.43679	7.137209
17291019	Hist1h4n	histone cluster 1, H4n	9.341408	13.15564	6.992896	6.272889	3.729055	4.475379
17400545	Hist2h2aa1	histone cluster 2, H2aa1	312.2288	287.6658	345.7008	335.7492	382.83	404.7395
17408017	Hist2h2aa2	histone cluster 2, H2aa2	312.2288	287.6658	345.7008	335.7492	382.83	404.7395
17400539	Hist2h2ab	histone cluster 2, H2ab	3.422238	2.704028	2.877149	4.181382	3.863129	4.180223
17408011	Hist2h2ac	histone cluster 2, H2ac	4.412206	6.503156	5.558671	8.118088	7.377592	4.924646
17400562	Hist2h2bb	histone cluster 2, H2bb	4.547898	3.526898	3.828502	4.874824	3.424658	3.823887
17400541	Hist2h2be	histone cluster 2, H2be	6.299337	6.997599	7.915772	3.644624	6.686541	5.748717
17400559	Hist2h3b	histone cluster 2, H3b	4.38805	4.275313	4.045365	3.782238	4.014026	5.454663
17400555	Hist2h3c2	histone cluster 2, H3c2	65.48579	60.7937	67.49132	62.16023	59.49432	59.49432
17408015	Hist2h3c2	histone cluster 2, H3c2	5.942871	4.372808	4.246635	3.898178	5.823958	5.343704
17408021	Hist2h4	histone cluster 2, H4	5.916894	5.389345	6.671541	4.681043	3.107792	4.15924
17250178	Hist3h2a	histone cluster 3, H2a	3.061715	4.970462	5.722359	6.632671	3.867765	5.383819
17250162	Hist3h2ba	histone cluster 3, H2ba	2.591325	2.692861	2.481049	2.083357	2.188921	3.098178
17263218	Hist3h2bb-ps	histone cluster 3, H2bb, pseudogene	2.127988	2.089373	2.012669	2.089373	1.975366	2.053038
17472159	Hist4h4	histone cluster 4, H4	5.788743	4.303469	4.844138	4.326229	4.844138	5.358577
17548561	Hist4h4	histone cluster 4, H4	19.51985	10.32142	14.54529	16.75082	12.37254	14.44472
17430397	Hdac1	histone deacetylase 1	77.46324	52.24025	51.28447	49.62104	36.33858	56.60196
17320279	Hdac10	histone deacetylase 10	19.81196	23.88247	20.24958	27.63932	23.26888	16.23708
17460879	Hdac11	histone deacetylase 11	42.67088	51.26456	45.53835	44.50236	44.54742	46.68153
17232560	Hdac2	histone deacetylase 2	42.89686	41.15598	37.50757	43.6612	51.38837	46.95087
17353839	Hdac3	histone deacetylase 3	72.8946	89.13561	92.45632	84.65715	82.53762	77.54866
17225531	Hdac4	histone deacetylase 4	22.5693	21.52349	15.8299	16.07793	18.36058	20.00918
17225535	Hdac4	histone deacetylase 4	29.1275	26.58223	31.82768	32.53679	25.30236	26.36715
17270082	Hdac5	histone deacetylase 5	32.37998	49.87794	58.11586	45.70943	40.59922	46.65695
17539932	Hdac6	histone deacetylase 6	29.24869	27.48057	36.48395	32.62622	32.0953	33.06287
17321046	Hdac7	histone deacetylase 7	13.6415	13.5545	19.18424	11.54055	14.29818	13.47899
17543675	Hdac8	histone deacetylase 8	12.08462	12.5343	15.01601	18.53641	14.31316	13.84574
17280689	Hdac9	histone deacetylase 9	5.402511	6.698323	5.7632	5.991719	5.50354	5.725216
17255314	Hils1	histone H1-like protein in spermatids 1	6.201039	5.414695	5.613809	6.000697	4.461133	5.652817
17526195	Hinfp	histone H4 transcription factor	17.39186	14.37958	16.93528	11.00395	14.37958	14.37958
17535018	Htatsf1	HIV TAT specific factor 1	17.39125	17.39125	17.39125	14.34782	19.04249	17.67122
17478352	Htatip2	HIV-1 tat interactive protein 2, homolog (human)	72.96234	94.36108	91.12026	91.2043	66.19315	72.96234
17354703	Hmgxb3	HMG box domain containing 3	29.97484	33.14616	28.09439	25.18024	30.09037	28.21069
17502537	Hmgxb4	HMG box domain containing 4	10.88146	12.99919	14.60692	16.16689	9.598507	11.82903
17451629	Hnf1a	HNF1 homeobox A	32.25878	28.51521	30.71092	29.23896	34.36124	33.61679
17441072	Hnf1aos1	HNF1 homeobox A, opposite strand 1	18.42944	16.32487	13.19482	20.24874	13.19482	15.81213
17254332	Hnf1b	HNF1 homeobox B	33.27623	26.76118	26.26428	32.05372	25.69971	25.22898
17378272	Raly	hnRNP-associated with lethal yellow	55.24569	62.99695	52.45797	50.39994	55.06371	48.11217
17225329	Hjurp	Holliday junction recognition protein	63.16879	82.62807	69.5144	78.30432	66.24961	76.47043
17332299	Hlcs	holocarboxylase synthetase (biotin- [propriony-Coenzyme A-carboxylase (ATP-hyd	16.14886	15.47846	16.42737	15.52262	18.02366	16.46728
17546192	Hccs	holocytochrome c synthetase	60.9059	42.7055	45.89452	53.93762	48.3167	47.06067
17466798	Hoxa1	homeobox A1	3.339703	3.339703	3.276919	3.339703	3.339703	3.339703
17466878	Hoxa10	homeobox A10	4.33788	4.206062	4.363513	4.206062	3.880655	4.600211
17466887	Hoxa11	homeobox A11	4.537004	4.450816	4.235523	4.235523	3.729055	4.980394

17458616	Hoxa11os	homeobox A11, opposite strand	2.699646	4.457331	2.749921	2.989077	3.940531	3.065197
17466894	Hoxa13	homeobox A13	6.865097	7.57812	7.57812	9.951679	7.57812	8.417548
17466805	Hoxa2	homeobox A2	4.643009	4.299116	4.657288	3.840968	4.680946	5.09849
17466812	Hoxa3	homeobox A3	4.034948	4.20927	5.285207	3.720509	4.20927	4.136068
17466827	Hoxa4	homeobox A4	17.49341	16.33382	11.59901	10.77518	17.07377	12.53917
17466833	Hoxa5	homeobox A5	4.437017	4.402369	4.819154	4.15924	3.362047	4.631983
17466838	Hoxa6	homeobox A6	10.0005	10.80689	9.627025	11.24709	9.301478	10.34341
17466845	Hoxa7	homeobox A7	3.077588	3.718756	3.72167	4.308812	4.55446	3.287657
17466863	Hoxa9	homeobox A9	4.557808	4.008604	4.647678	3.789585	3.423471	3.756164
17466874	Hoxa9	homeobox A9	5.503235	5.503235	5.945549	6.589961	5.083457	6.189745
17255620	Hoxb1	homeobox B1	12.08596	14.63388	10.98658	14.31296	12.08596	13.77996
17255534	Hoxb13	homeobox B13	4.015278	3.74073	4.722497	4.109777	3.118582	5.652464
17255614	Hoxb2	homeobox B2	3.367365	3.388014	3.026793	4.112199	3.388014	3.53826
17255604	Hoxb3	homeobox B3	7.831127	4.90244	5.975544	6.002652	6.002652	6.63773
17255594	Hoxb4	homeobox B4	8.027329	6.992896	8.460835	5.990058	6.923733	7.169189
17255588	Hoxb5	homeobox B5	9.31109	7.143693	7.156281	7.706095	7.443546	7.709301
17268313	Hoxb5os	homeobox B5 and homeobox B6, opposite strand	14.75263	15.34227	14.61218	14.31296	14.31296	14.31296
17255581	Hoxb6	homeobox B6	4.514981	5.26371	4.374566	4.919938	4.4624	4.478296
17255572	Hoxb7	homeobox B7	3.254329	3.06335	3.351925	3.153185	4.556891	3.555149
17255565	Hoxb8	homeobox B8	6.209728	5.148207	5.870771	7.029977	7.041047	9.446828
17255558	Hoxb9	homeobox B9	6.405939	10.66713	8.402566	10.96603	10.68481	13.09333
17315500	Hoxc10	homeobox C10	8.436824	6.820228	6.394582	7.730062	9.622688	8.392787
17315495	Hoxc11	homeobox C11	3.819755	3.819755	3.221718	3.819755	3.912769	4.546984
17315490	Hoxc12	homeobox C12	7.022039	8.769898	7.096413	7.920162	7.890955	8.297921
17315485	Hoxc13	homeobox C13	4.616086	6.353009	4.832836	4.56625	6.850266	5.676887
17315538	Hoxc4	homeobox C4	6.171709	6.171709	5.946868	6.171709	5.726406	5.955241
17315530	Hoxc5	homeobox C5	3.753353	3.515476	2.390943	3.873264	4.069524	3.753353
17315519	Hoxc6	homeobox C6	3.927551	3.785464	3.927551	4.028716	3.927551	3.466765
17315514	Hoxc8	homeobox C8	4.638634	4.672129	5.017122	5.128653	5.548701	4.551462
17315507	Hoxc9	homeobox C9	4.744809	4.634745	3.702141	5.126059	5.120306	5.060535
17307709	Hmbox1	homeobox containing 1	35.99913	33.94436	37.84891	29.1166	31.96076	38.39882
17372114	Hoxd1	homeobox D1	4.854558	5.293603	4.794299	4.681043	4.681043	4.681043
17372054	Hoxd10	homeobox D10	10.07781	12.29262	13.45164	10.2684	11.00678	12.0489
17372045	Hoxd11	homeobox D11	4.442156	4.479973	4.132658	4.450971	4.533641	4.51639
17372039	Hoxd12	homeobox D12	6.663869	7.089383	6.61637	5.131925	7.532924	6.672836
17372035	Hoxd13	homeobox D13	5.506211	4.019873	4.701887	5.875412	3.676441	4.93695
17372080	Hoxd3	homeobox D3	5.965694	5.256199	5.050653	4.68299	5.284731	4.834578
17386672	Hoxd3os1	homeobox D3, opposite strand 1	10.81424	11.26769	13.18687	13.78579	11.03137	11.81076
17372092	Hoxd4	homeobox D4	10.5647	21.05951	17.34238	17.85109	17.74536	18.81524
17372072	Hoxd8	homeobox D8	14.57749	10.48089	10.66876	10.10201	13.19482	13.65824
17372066	Hoxd9	homeobox D9	2.46444	3.624369	2.653414	4.450662	3.790163	3.58154
17298014	Hesx1	homeobox gene expressed in ES cells	3.042274	3.443463	2.58553	2.822728	2.822728	3.193548
17408662	Hipk1	homeodomain interacting protein kinase 1	120.9544	142.4745	159.0427	131.6743	139.4873	131.9758
17466033	Hipk2	homeodomain interacting protein kinase 2	154.0377	174.0153	222.2901	158.0142	110.8635	127.1625
17388931	Hipk3	homeodomain interacting protein kinase 3	175.7718	184.5464	183.0203	164.6593	183.9054	199.4758
17475616	Hipk4	homeodomain interacting protein kinase 4	5.191891	5.091356	4.789317	5.062711	4.887477	4.66915
17306491	Homez	homeodomain leucine zipper-encoding gene	4.602475	3.973582	4.850825	3.942443	4.216658	4.722563
17289137	Homer1	homer homolog 1 (Drosophila)	16.13867	11.31073	12.20212	11.89596	13.63375	13.36761
17492826	Homer2	homer homolog 2 (Drosophila)	24.64392	22.44979	29.79875	31.27504	31.89437	22.78938
17501877	Homer3	homer homolog 3 (Drosophila)	3.499212	3.729055	3.815706	3.729055	3.729055	5.042817
17504005	Herpud1	homocysteine-inducible, endoplasmic reticulum stress-inducible, ubiquitin-like doı	1078.702	705.5233	633.0695	622.7287	798.6392	839.5195
17325497	Hgd	homogentisate 1, 2-dioxygenase	814.5277	934.3181	934.2663	997.8022	906.1399	1114.322
17415570	Hook1	hook homolog 1 (Drosophila)	98.12644	79.45521	71.23923	74.33914	97.42904	79.3193
17503300	Hook2	hook homolog 2 (Drosophila)	18.67362	27.55878	24.67657	19.97813	27.21236	28.07122
17508482	Hook3	hook homolog 3 (Drosophila)	77.41762	78.51737	74.76598	85.91809	74.54708	87.50418
17449052	Hopx	HOP homeobox	11.04989	15.76027	10.99519	16.22841	11.79759	14.01986
17438482	Hopxos	HOP homeobox, opposite strand	6.287864	6.639065	6.287864	6.287864	5.141502	6.817818
17400386	Hormad1	HORMA domain containing 1	2.598178	2.909114	2.472293	2.37614	2.465004	2.51333
17259975	Hormad2	HORMA domain containing 2	3.197136	3.755018	3.163233	3.245678	3.088936	4.394351
17326964	Hunk	hormonally upregulated Neu-associated kinase	15.72905	14.33232	17.18633	17.79845	15.5337	10.92847
17399972	Hrnr	hornerin	2.776428	2.719271	2.483492	2.211431	2.719271	2.719271
17542516	Hcfc1	host cell factor C1	94.0809	81.92433	78.11242	75.01879	81.06572	76.71517
17334091	Hcfc1r1	host cell factor C1 regulator 1 (XPO1-dependent)	43.86049	55.57946	67.83839	50.4765	67.64447	54.33576
17235890	Hcfc2	host cell factor C2	26.00667	22.57494	22.03401	21.84211	22.82796	22.86074
17322309	Hotair	HOX transcript antisense RNA (non-protein coding)	4.762998	4.681043	4.681043	5.217794	4.681043	4.20927
17458587	Halr1	Hoxa adjacent long noncoding RNA 1	3.270815	3.270815	3.270815	2.636036	3.270815	3.270815
17458598	Hoxaas2	Hoxa cluster antisense RNA 2	13.17892	12.98829	14.55568	13.71279	12.08939	15.52681
17458605	Hoxaas3	Hoxa cluster antisense RNA 3	5.669337	5.300432	6.287864	4.20927	4.676373	4.816483
17458626	Hottip	Hoxa distal transcript antisense RNA	3.673156	3.637583	3.772942	4.027431	3.432524	4.381151
17458592	Hotairm1	Hoxa transcript antisense RNA, myeloid-specific 1	5.071772	5.675116	4.63041	6.84272	5.953632	6.191419
17386679	Haglr	Hoxd antisense growth associated long non-coding RNA	4.079211	3.686802	3.931364	3.686802	4.109691	3.686802
17324576	Hrasls	HRAS-like suppressor	4.546984	5.537136	5.586443	6.69461	6.566206	7.565996
17357083	Hrasls5	HRAS-like suppressor family, member 5	4.26321	3.916487	4.21809	4.266314	4.14531	4.226636
17451171	Hscb	HscB iron-sulfur cluster co-chaperone homolog (E. coli)	9.827606	8.789554	10.72146	11.32917	11.14311	13.15446
17485880	Hspbp1	HSPA (heat shock 70kDa) binding protein, cytoplasmic cochaperone 1	46.62914	44.24278	51.24538	46.34912	40.34338	46.73009
17325261	Hspbap1	Hspb associated protein 1	8.793759	8.865303	8.730414	8.762849	8.488973	9.173931
17483912	Htra1	HtrA serine peptidase 1	8.962803	10.71723	9.666476	8.765826	10.36142	10.97889
17468075	Htra2	HtrA serine peptidase 2	40.56124	36.86526	40.01974	34.79529	47.51595	35.81272
17447365	Htra3	HtrA serine peptidase 3	9.372604	11.11511	8.995977	10.5356	10.21799	13.00983
17508325	Htra4	HtrA serine peptidase 4	5.295181	5.336967	5.746247	6.840302	6.155005	5.675471
17286818	Hivep1	human immunodeficiency virus type I enhancer binding protein 1	51.64225	56.01573	65.6444	64.75816	58.4216	60.82785
17231742	Hivep2	human immunodeficiency virus type I enhancer binding protein 2	15.80676	14.13226	22.00943	18.65965	14.35867	17.80092
17231754	Hivep2	human immunodeficiency virus type I enhancer binding protein 2	11.04131	9.729259	10.41665	11.46965	9.851955	12.45074
17417952	Hivep3	human immunodeficiency virus type I enhancer binding protein 3	7.764376	8.815424	8.533396	8.418248	8.276266	8.203616

17436683	Htt	huntingtin	41.58093	41.73165	41.3768	41.43305	30.2384	41.56681
17453573	Hip1	huntingtin interacting protein 1	20.00918	27.51316	26.95872	20.03596	19.91826	16.4683
17442428	Hip1r	huntingtin interacting protein 1 related	27.77028	27.73854	30.29232	27.3485	27.53835	24.02892
17375298	Hypk	huntingtin interacting protein K	24.82772	19.65862	22.94312	22.94312	25.45523	23.25872
17533226	Hypm	huntingtin interacting protein M	5.671066	6.204135	5.590665	6.163501	7.038412	8.769898
17269439	Hap1	huntingtin-associated protein 1	11.75156	14.10808	13.08489	14.10808	14.10808	17.42202
17260579	Hus1	Hus1 homolog (S. pombe)	13.86014	12.21879	13.66402	15.02236	11.66432	18.97963
17291609	Hus1b	Hus1 homolog b (S. pombe)	3.933899	4.801649	4.724429	4.344921	4.417438	4.255563
17289009	Hapln1	hyaluronan and proteoglycan link protein 1	4.98295	4.208395	4.806478	5.368615	4.170817	8.688457
17406646	Hapln2	hyaluronan and proteoglycan link protein 2	6.992896	6.840302	6.992896	8.314964	10.12577	7.698994
17492305	Hapln3	hyaluronan and proteoglycan link protein 3	7.656314	7.326784	7.301182	6.734963	7.069166	7.438182
17501800	Hapln4	hyaluronan and proteoglycan link protein 4	13.69711	9.936584	15.28972	13.77538	9.145487	10.4085
17261840	Hmmr	hyaluronan mediated motility receptor (RHAMM)	3.188593	2.454348	3.185832	2.946455	2.929268	3.105876
17317167	Has2	hyaluronan synthase 2	4.47107	5.036634	5.48933	5.971031	5.323887	5.556398
17311633	Has2os	hyaluronan synthase 2, opposite strand	2.560579	3.164571	2.887378	3.20004	4.23159	2.864652
17505191	Has3	hyaluronan synthase 3	8.099765	7.107588	9.078098	6.509064	6.952877	7.018584
17341269	Has1	hyaluronan synthase1	7.225313	6.238158	6.617746	6.394449	7.556457	6.576135
17360537	Habp2	hyaluronic acid binding protein 2	350.6991	344.5835	337.464	325.2714	379.288	342.6448
17288145	Habp4	hyaluronic acid binding protein 4	101.7468	89.9443	83.64863	96.33446	109.6833	87.80067
17521428	Hyal1	hyaluronoglucosaminidase 1	125.9956	132.6545	151.0696	125.4875	101.2052	103.2609
17521422	Hyal2	hyaluronoglucosaminidase 2	59.51577	58.57	62.98167	58.55052	56.16307	57.58296
17521448	Hyal3	hyaluronoglucosaminidase 3	8.349617	10.62669	8.754532	6.171709	6.788204	5.669455
17456440	Hyal4	hyaluronoglucosaminidase 4	3.643841	2.999641	2.764484	3.585241	2.733274	3.732908
17456460	Hyal5	hyaluronoglucosaminidase 5	2.67402	2.557173	3.227126	2.642274	2.287022	2.628045
17456434	Hyal6	hyaluronoglucosaminidase 6	2.917212	3.52905	2.987151	3.748075	2.648104	4.099534
17505505	Hydin	HYDIN, axonemal central pair apparatus protein	5.45905	5.523183	5.48933	5.48933	6.156882	5.563875
17452709	Hcar1	hydrocarboxylic acid receptor 1	13.19427	10.50359	11.37701	13.95007	12.77534	11.35795
17442046	Hvcn1	hydrogen voltage-gated channel 1	9.926465	8.649879	9.710328	9.051708	8.522697	8.592989
17525418	Hyls1	hydrolethalus syndrome 1	8.800772	10.9494	11.67508	9.700304	9.822839	8.912252
17392076	Hao1	hydroxyacid oxidase 1, liver	339.517	205.3655	191.1631	226.6151	267.4175	190.8665
17408360	Hao2	hydroxyacid oxidase 2	4.013915	2.903393	3.777076	4.536218	3.243789	3.789191
17334449	Hagh	hydroxyacyl glutathione hydrolase	272.7755	229.7275	203.5924	219.7151	240.6162	257.4449
17410390	Hadh	hydroxyacyl-Coenzyme A dehydrogenase	956.1877	1142.113	1163.625	1082.567	1018.394	1157.92
17446643	Hadha	hydroxyacyl-Coenzyme A dehydrogenase/3-ketoacyl-Coenzyme A thiolase/enoyl-C	1133.399	1074.813	1032.911	944.358	853.1408	819.3125
17374053	Hadhb	hydroxyacyl-Coenzyme A dehydrogenase/3-ketoacyl-Coenzyme A thiolase/enoyl-C	408.6721	423.2628	399.6131	372.1886	304.4645	326.4368
17435748	Hadhb	hydroxyacyl-Coenzyme A dehydrogenase/3-ketoacyl-Coenzyme A thiolase/enoyl-C	11.94546	10.06601	13.06224	10.94348	11.02235	8.94028
17342359	Haghl	hydroxyacylglutathione hydrolase-like	12.05701	11.6967	12.77145	13.69427	14.51115	14.37958
17452705	Hcar2	hydroxycarboxylic acid receptor 2	6.808703	7.104534	6.808703	7.034218	6.99435	6.505094
17408352	Hsd3b1	hydroxy-delta-5-steroid dehydrogenase, 3 beta- and steroid delta-isomerase 1	4.033354	2.737009	2.486575	3.54113	2.320346	2.752571
17408323	Hsd3b2	hydroxy-delta-5-steroid dehydrogenase, 3 beta- and steroid delta-isomerase 2	42.71942	32.95693	17.33925	36.44275	22.33462	32.71703
17408336	Hsd3b3	hydroxy-delta-5-steroid dehydrogenase, 3 beta- and steroid delta-isomerase 3	195.2921	121.9765	99.76764	189.8387	141.7338	160.9301
17408297	Hsd3b4	hydroxy-delta-5-steroid dehydrogenase, 3 beta- and steroid delta-isomerase 4	16.22605	6.009229	6.088724	7.698994	7.548186	6.055936
17408306	Hsd3b4	hydroxy-delta-5-steroid dehydrogenase, 3 beta- and steroid delta-isomerase 4	16.22605	6.009229	6.088724	7.698994	7.548186	6.055936
17408315	Hsd3b5	hydroxy-delta-5-steroid dehydrogenase, 3 beta- and steroid delta-isomerase 5	8.320672	2.572106	2.611358	3.276033	4.049433	2.781668
17408345	Hsd3b6	hydroxy-delta-5-steroid dehydrogenase, 3 beta- and steroid delta-isomerase 6	2.902327	2.519854	2.793144	3.54801	2.822982	3.104326
17483471	Hsd3b7	hydroxy-delta-5-steroid dehydrogenase, 3 beta- and steroid delta-isomerase 7	262.4611	251.1821	229.5795	264.6111	251.0899	287.7895
17517448	Hykk	hydroxylysine kinase 1	287.7596	280.808	316.9607	320.1224	278.2234	395.7869
17526225	Hmbs	hydroxymethylbilane synthase	59.8297	53.41711	55.69284	64.1639	59.79281	55.84747
17501250	Hpgd	hydroxyprostaglandin dehydrogenase 15 (NAD)	392.2936	427.3517	334.0268	410.5574	345.6576	523.0373
17417738	Hyi	hydroxypyruvate isomerase homolog (E. coli)	112.3511	107.6049	92.75223	107.5505	133.9471	159.5838
17256513	Hsd17b1	hydroxysteroid (17-beta) dehydrogenase 1	6.251013	7.592053	7.573447	6.721066	7.137704	7.169189
17538852	Hsd17b10	hydroxysteroid (17-beta) dehydrogenase 10	104.1725	109.2319	122.9945	141.2278	121.4038	128.5281
17450366	Hsd17b11	hydroxysteroid (17-beta) dehydrogenase 11	406.57	491.099	440.1923	390.3731	270.5966	330.8997
17388573	Hsd17b12	hydroxysteroid (17-beta) dehydrogenase 12	209.4337	187.3095	152.1742	171.4222	156.9118	169.8585
17450354	Hsd17b13	hydroxysteroid (17-beta) dehydrogenase 13	947.7351	886.9629	909.7208	806.5835	791.8042	752.0527
17477884	Hsd17b14	hydroxysteroid (17-beta) dehydrogenase 14	5.719504	5.719504	6.352656	6.227314	5.70521	5.889479
17506030	Hsd17b2	hydroxysteroid (17-beta) dehydrogenase 2	370.8881	327.0483	225.173	323.7912	344.6743	255.1391
17293635	Hsd17b3	hydroxysteroid (17-beta) dehydrogenase 3	5.042713	4.25571	4.053618	4.839339	5.006388	4.736922
17350517	Hsd17b4	hydroxysteroid (17-beta) dehydrogenase 4	995.8054	953.1504	946.888	954.8961	705.1176	805.5666
17246057	Hsd17b6	hydroxysteroid (17-beta) dehydrogenase 6	612.2385	826.6942	592.7172	548.326	396.5118	500.1684
17229466	Hsd17b7	hydroxysteroid (17-beta) dehydrogenase 7	70.6604	68.84786	90.66162	58.11546	63.81041	52.74235
17231229	Hsd11b1	hydroxysteroid 11-beta dehydrogenase 1	224.9624	217.5058	191.5716	191.1154	249.0307	278.0306
17504829	Hsd11b2	hydroxysteroid 11-beta dehydrogenase 2	7.464367	8.139725	7.795438	8.618997	7.922633	7.541283
17513446	Hsdl1	hydroxysteroid dehydrogenase like 1	7.44401	6.288387	8.192763	6.488073	6.930455	7.643164
17414456	Hsdl2	hydroxysteroid dehydrogenase like 2	291.9606	331.2278	307.4483	310.4786	217.7909	244.7508
17265912	Hic1	hypermethylated in cancer 1	4.414255	5.815527	4.761876	5.110059	5.068118	5.607081
17323432	Hic2	hypermethylated in cancer 2	5.530999	6.438212	8.550033	6.438212	8.023824	6.992896
17290093	Hcn1	hyperpolarization-activated, cyclic nucleotide-gated K+ 1	3.696936	2.855216	2.422757	2.842637	3.040482	2.784349
17234936	Hcn2	hyperpolarization-activated, cyclic nucleotide-gated K+ 2	7.368138	8.222403	6.235045	9.722788	8.312198	7.417176
17406925	Hcn3	hyperpolarization-activated, cyclic nucleotide-gated K+ 3	5.71158	4.875804	5.374982	6.887594	4.98105	5.242773
17517924	Hcn4	hyperpolarization-activated, cyclic nucleotide-gated K+ 4	4.329649	5.577351	3.648036	4.635163	4.327039	4.021462
17269672	Hcrt	hypocretin	7.594105	8.031559	6.992896	7.629243	5.985908	6.992896
17430561	Hcrtr1	hypocretin (orexin) receptor 1	4.715694	4.645487	4.160019	4.418081	4.040349	4.617558
17528913	Hcrtr2	hypocretin (orexin) receptor 2	5.923952	7.02384	6.659021	6.537097	5.481041	6.69062
17420500	AB041806	hypothetical protein, MNCb-2457	6.106222	5.527779	5.878956	6.275672	6.882964	6.466253
17534720	Hprt	hypoxanthine guanine phosphoribosyl transferase	154.8835	157.9342	163.2546	158.873	152.5607	158.0153
17276328	Hif1a	hypoxia inducible factor 1, alpha subunit	48.91042	47.85474	48.48747	45.0336	40.48063	44.35208
17487001	Hif3a	hypoxia inducible factor 3, alpha subunit	7.047248	6.416026	7.814535	6.891032	5.999366	7.590158
17456554	Hilpda	hypoxia inducible lipid droplet associated	35.56731	35.83457	35.30596	36.09607	34.49033	39.82107
17516564	Hyou1	hypoxia up-regulated 1	504.5628	197.1924	177.6274	215.9959	232.9231	170.159
17359716	Hif1an	hypoxia-inducible factor 1, alpha subunit inhibitor	27.24463	24.84787	21.51394	23.25292	21.53155	28.42818
17417422	Ipp	IAP promoted placental gene	13.68383	13.64973	15.07138	14.91777	15.07138	12.04581
17263345	Iba57	IBA57, iron-sulfur cluster assembly homolog (S. cerevisiae)	5.25456	5.101141	4.305677	4.68523	5.429427	3.849551
17234786	Icosl	icos ligand	6.403186	7.405669	8.049226	7.357267	7.030757	8.1351

17287869	Idnk	idnK gluconokinase homolog (E. coli)	40.72492	49.94644	67.56527	47.40574	56.48164	59.72487
17542149	Ids	iduronate 2-sulfatase	19.58626	18.78475	19.21019	18.89837	24.19438	16.38507
17440293	Idua	iduronidase, alpha-L-	28.02204	26.97816	38.01877	25.53882	25.92424	20.1854
17349627	Igip	IgA inducing protein	29.74427	24.10232	24.05925	28.35537	28.08874	25.52608
17474338	Igfl3	IGF-like family member 3	3.562747	3.339703	3.077012	3.339703	3.363492	3.339703
17476478	Igflr1	IGF-like family receptor 1	10.16607	9.732834	11.49002	10.01958	8.497686	10.09116
17490126	Iglon5	IgLON family member 5	9.721911	10.96353	10.55372	13.23806	10.99	9.128261
17349745	Ik	IK cytokine	170.6575	157.9988	146.732	142.6613	181.7448	164.0589
17247336	Ikzf1	IKAROS family zinc finger 1	9.123011	7.605853	6.992896	6.697023	6.589687	8.420524
17223985	Ikzf2	IKAROS family zinc finger 2	4.514856	4.775958	5.175937	5.175937	5.357946	5.643186
17268820	Ikzf3	IKAROS family zinc finger 3	6.016982	5.86215	6.702224	5.86215	6.193865	5.606421
17246268	Ikzf4	IKAROS family zinc finger 4	4.735675	6.535466	4.823098	6.914812	6.125935	6.125935
17497044	Ikzf5	IKAROS family zinc finger 5	37.46055	37.47171	36.37587	35.52075	39.67917	38.14547
17236476	Ikbip	IKBKB interacting protein	12.291	12.46923	12.78677	15.80753	13.87023	13.20589
17262102	Itk	IL2 inducible T cell kinase	5.146494	4.20927	4.239047	4.20927	4.160797	4.20927
17234814	Ilvbl	ilvB (bacterial acetolactate synthase)-like	27.09454	29.09945	29.31709	23.27179	26.88911	27.13156
17258186	Ict1	immature colon carcinoma transcript 1	68.67866	58.11183	57.01027	55.6153	58.13157	64.50459
17271888	Ict1os	immature colon carcinoma transcript 1, opposite strand [Source:MGI Symbol;Acc:N	3.703322	4.224469	3.703322	3.703322	4.504853	3.915645
17511130	Ier2	immediate early response 2	16.27708	18.24324	22.69307	19.85073	24.35507	26.59476
17337228	Ier3	immediate early response 3	11.24366	17.20076	21.66358	9.739718	14.36305	10.78863
17351913	Ier3ip1	immediate early response 3 interacting protein 1	47.39063	38.43157	40.03112	39.50984	40.13642	37.43615
17228349	Ier5	immediate early response 5	16.55219	16.07537	12.56518	15.02684	15.43967	13.78407
17383663	Ier5l	immediate early response 5-like	20.07224	19.3154	22.84711	20.60054	24.3392	20.34652
17262202	Irgm1	immunity-related GTPase family M member 1	44.10805	25.28062	25.20311	34.29437	26.86135	29.96113
17249990	Irgm2	immunity-related GTPase family M member 2	70.48967	80.42265	111.1412	120.5945	89.66481	98.4364
17487716	Irgc1	immunity-related GTPase family, cinema 1	5.558055	5.742226	5.772155	4.815448	4.970118	5.358466
17475001	Irgq	immunity-related GTPase family, Q	21.38652	19.92627	17.37077	17.62071	22.103	17.99058
17536519	Igbp1	immunoglobulin (CD79A) binding protein 1	88.51745	107.1761	95.19936	100.6107	116.5641	116.2897
17472303	Igbp1b	immunoglobulin (CD79A) binding protein 1b	3.37206	3.263862	3.339703	4.024222	3.339703	3.729055
17284334	Ighg	Immunoglobulin heavy chain (gamma polypeptide)	7.532141	40.42792	19.06785	7.698994	9.949334	5.954498
17284484	Ighg	Immunoglobulin heavy chain (gamma polypeptide)	2.44606	1.761116	2.142864	2.606909	2.643959	1.920564
17284314	Igh-VJ558	immunoglobulin heavy chain (J558 family)	6.970683	10.54794	8.174157	6.43906	8.031893	7.681936
17284321	Igh-VJ558	immunoglobulin heavy chain (J558 family)	3.539584	2.603243	2.428356	2.948048	3.263862	2.800996
17284379	Igh-VJ558	immunoglobulin heavy chain (J558 family)	3.916568	4.390697	3.438168	3.599958	3.4082	3.287908
17284450	Igh-VJ558	immunoglobulin heavy chain (J558 family)	3.09365	2.416686	3.004823	3.824842	2.110142	3.054995
17284493	Igh-VJ558	immunoglobulin heavy chain (J558 family)	2.649922	2.017432	2.23994	1.896409	2.877369	2.061965
17284536	Igh-VJ558	immunoglobulin heavy chain (J558 family)	3.069067	2.893128	2.669149	2.603171	2.893128	2.692712
17284447	Igh-V11	immunoglobulin heavy chain (V11 family)	106.721	92.5942	91.56092	101.9996	82.35704	85.44002
17284339	Igh-V3609N	immunoglobulin heavy chain (V3609N non-productive)	7.551745	158.0876	76.5521	6.631706	5.714471	6.105841
17284356	Igh-V7183	immunoglobulin heavy chain (V7183 family)	43.36112	75.7057	52.84848	41.28054	51.03058	37.41513
17284399	Igh-V7183	immunoglobulin heavy chain (V7183 family)	4.228864	3.0726	2.534199	3.932182	4.211225	3.547493
17284354	Igh-VX24	immunoglobulin heavy chain (X24 family)	15.60266	70.72459	55.94937	23.98615	21.1615	17.28849
17284376	Igh-VX24	immunoglobulin heavy chain (X24 family)	91.8616	96.7688	82.57425	80.03006	73.36093	96.83589
17284408	Igh-VX24	immunoglobulin heavy chain (X24 family)	63.35515	70.81485	68.70628	74.06448	60.11112	70.94111
17284327	Ighg2c	immunoglobulin heavy constant gamma 2C	20.26376	21.92447	16.59653	19.63873	17.78304	17.96168
17284344	Ighg3	Immunoglobulin heavy constant gamma 3	2.975207	4.43594	3.694144	2.606909	2.909114	4.128449
17284349	Ighm	immunoglobulin heavy constant mu	20.18805	23.97153	29.18206	21.81956	23.18516	14.06619
17284360	Ighm	immunoglobulin heavy constant mu	108.0676	183.0787	104.8127	126.9705	111.9942	132.6472
17284373	Ighm	immunoglobulin heavy constant mu	5.633182	4.037969	5.320346	4.411258	3.204457	5.972107
17284402	Ighm	immunoglobulin heavy constant mu	5.227894	6.177101	4.513323	6.293489	4.199624	10.15684
17284660	Ighm	immunoglobulin heavy constant mu	2.462357	13.06568	6.998181	2.351645	4.912509	2.897242
17284358	Ighj2	immunoglobulin heavy joining 2 [Source:MGI Symbol;Acc:MGI:4439803]	3.572837	3.200284	2.730017	3.322456	3.793133	3.533236
17284498	Ighv10-1	immunoglobulin heavy variable 10-1	2.285247	2.850984	3.51767	2.608192	2.458673	2.318578
17284439	Ighv11-1	immunoglobulin heavy variable 11-1	25.29657	21.22452	20.73161	30.2007	21.31785	22.20127
17284517	Ighv1-14	immunoglobulin heavy variable 1-14	2.096089	1.898013	1.915671	3.692275	2.228789	2.285991
17284519	Ighv1-15	immunoglobulin heavy variable 1-15	7.102023	7.698994	8.151017	6.957746	6.030928	7.019654
17284496	Ighv1-2	immunoglobulin heavy variable 1-2	2.086681	1.927285	1.855155	1.928635	2.121023	1.918249
17284544	Ighv1-30	immunoglobulin heavy variable 1-30	1.825232	1.558859	2.447807	1.797099	1.941457	2.048915
17284480	Ighv13-1	immunoglobulin heavy variable 13-1	2.953407	2.749273	2.156976	2.606909	3.326719	3.429718
17284546	Ighv1-31	immunoglobulin heavy variable 1-31	1.905342	2.009449	2.019209	2.304206	2.120126	2.256615
17284548	Ighv1-34	immunoglobulin heavy variable 1-34	3.292903	15.12004	16.33936	4.890832	4.961409	5.916894
17284551	Ighv1-36	immunoglobulin heavy variable 1-36	6.392765	5.098384	7.330848	7.076127	6.109905	6.13337
17284554	Ighv1-37	immunoglobulin heavy variable 1-37	8.693156	16.17002	9.158238	8.381567	8.476978	10.81724
17284557	Ighv1-39	immunoglobulin heavy variable 1-39	1.982801	2.265579	1.799679	1.979684	2.304446	2.283648
17284500	Ighv1-4	immunoglobulin heavy variable 1-4	3.383157	5.068188	4.065774	3.947393	5.101	3.139054
17284432	Ighv14-1	immunoglobulin heavy variable 14-1	5.950538	10.54794	6.533699	5.519739	8.472748	9.76527
17284442	Ighv14-2	immunoglobulin heavy variable 14-2	6.876479	8.78322	5.650075	4.767424	4.687504	4.557144
17284466	Ighv14-4	immunoglobulin heavy variable 14-4	3.871654	30.25329	17.84849	4.920654	6.230638	6.082692
17284564	Ighv1-47	immunoglobulin heavy variable 1-47	2.285279	2.406006	2.264527	2.089228	2.26759	2.688889
17284566	Ighv1-49	immunoglobulin heavy variable 1-49	1.756897	1.824676	2.423144	2.452155	1.927779	2.120273
17284571	Ighv1-50	immunoglobulin heavy variable 1-50	2.435588	2.569255	2.308555	2.445467	2.393646	2.242006
17284574	Ighv1-52	immunoglobulin heavy variable 1-52	2.772197	7.698994	5.120306	3.263862	3.729055	3.478439
17284577	Ighv1-53	immunoglobulin heavy variable 1-53	13.32801	16.36033	10.66876	13.63942	14.00335	11.93114
17284585	Ighv1-55	immunoglobulin heavy variable 1-55	2.16556	2.437952	1.898026	2.362722	2.07471	1.907298
17284591	Ighv1-56	immunoglobulin heavy variable 1-56	10.16607	23.31894	11.53287	9.604696	10.48228	9.250427
17284596	Ighv1-58	immunoglobulin heavy variable 1-58	2.414258	2.140266	1.993827	1.940475	2.517584	2.041076
17284453	Ighv16-1	immunoglobulin heavy variable 16-1	4.829487	4.847262	5.063658	3.626782	4.829487	4.468188
17284602	Ighv1-61	immunoglobulin heavy variable 1-61	2.74792	3.406476	2.973269	2.162185	2.895716	2.093853
17284605	Ighv1-62-1	immunoglobulin heavy variable 1-62-1	12.51928	35.71653	20.01306	11.28622	12.04314	9.418061
17284607	Ighv1-62-2	immunoglobulin heavy variable 1-62-2	7.033389	12.46231	11.42459	8.48362	10.07935	8.244089
17284609	Ighv1-62-3	immunoglobulin heavy variable 1-62-3	11.03489	10.96854	5.87305	7.595263	4.92666	4.005133
17284623	Ighv1-66	immunoglobulin heavy variable 1-66	3.763774	4.125789	4.103002	3.552833	3.620829	3.75619
17284628	Ighv1-69	immunoglobulin heavy variable 1-69	2.412468	4.105079	2.412468	2.668224	2.149096	2.470375
17284631	Ighv1-71	immunoglobulin heavy variable 1-71	7.033389	12.46231	11.42459	8.48362	10.07935	8.244089

17284633	Ighv1-72	immunoglobulin heavy variable 1-72	10.62742	23.59697	15.39159	13.20278	11.76721	12.11607
17284638	Ighv1-73	immunoglobulin heavy variable 1-73	2.027891	2.422925	3.635213	2.508109	2.65798	2.143205
17284645	Ighv1-75	immunoglobulin heavy variable 1-75	19.34931	45.32013	39.80755	15.1919	31.74374	19.85376
17284648	Ighv1-76	immunoglobulin heavy variable 1-76	4.884836	9.563045	5.269478	3.218124	4.085917	4.723414
17284650	Ighv1-77	immunoglobulin heavy variable 1-77	3.792266	6.44835	5.475915	3.278487	6.091426	6.119611
17284652	Ighv1-78	immunoglobulin heavy variable 1-78	1.779979	2.721006	2.769605	2.465688	2.298082	2.32099
17284654	Ighv1-80	immunoglobulin heavy variable 1-80	2.438645	6.373651	3.163913	3.460403	3.826035	3.460403
17284657	Ighv1-81	immunoglobulin heavy variable 1-81	2.402872	3.259589	2.595603	2.400675	2.455471	2.077718
17284662	Ighv1-83	immunoglobulin heavy variable 1-83	4.569987	3.924802	3.791451	2.921744	2.497544	3.278941
17284664	Ighv1-84	immunoglobulin heavy variable 1-84	3.049219	4.089629	4.64552	4.806944	3.308621	3.265106
17284667	Ighv1-85	immunoglobulin heavy variable 1-85	2.590373	4.024557	2.043454	2.103847	2.996005	2.02201
17284370	Ighv2-1	immunoglobulin heavy variable 2-1	6.171709	6.895332	4.729999	6.171709	6.171709	5.682832
17284382	Ighv2-3	immunoglobulin heavy variable 2-3	4.890154	4.950623	3.832803	4.787094	3.967802	4.270752
17284396	Ighv2-5	immunoglobulin heavy variable 2-5	5.506746	6.006606	6.577275	6.827181	6.145924	5.787499
17284411	Ighv2-6-8	immunoglobulin heavy variable 2-6-8	10.27666	8.935757	12.07968	10.33316	11.81985	10.31263
17284414	Ighv2-7	immunoglobulin heavy variable 2-7	11.71293	5.780603	7.219605	6.730809	8.022768	9.94437
17284426	Ighv2-9	immunoglobulin heavy variable 2-9	13.62949	24.84545	20.34751	13.14726	13.41756	25.52219
17284437	Ighv3-1	immunoglobulin heavy variable 3-1	2.597907	5.263856	5.961106	3.416431	6.494147	3.229185
17284445	Ighv3-2	immunoglobulin heavy variable 3-2	8.250891	6.720879	9.052084	9.703936	10.39862	11.32304
17284435	Ighv4-1	immunoglobulin heavy variable 4-1	1.933963	2.019965	2.258868	2.098255	2.332295	2.204635
17284417	Ighv5-15	immunoglobulin heavy variable 5-15	2.690921	1.809223	2.800588	2.337992	2.603135	2.089865
17284420	Ighv5-16	immunoglobulin heavy variable 5-16	4.551115	6.775277	3.48725	3.579629	4.660711	2.454399
17284423	Ighv5-17	immunoglobulin heavy variable 5-17	3.044109	2.797581	2.481049	2.805796	3.191512	2.447078
17284387	Ighv5-6	immunoglobulin heavy variable 5-6	5.879201	5.233514	3.823145	5.246117	3.986493	5.229054
17284393	Ighv5-9	immunoglobulin heavy variable 5-9	3.030215	5.020427	3.801266	4.20927	3.944767	4.696513
17284405	Ighv5-9-1	immunoglobulin heavy variable 5-9-1	3.527779	5.535524	3.883991	3.076116	3.883991	3.883991
17284490	Ighv6-3	immunoglobulin heavy variable 6-3	38.06967	43.59921	40.45259	29.69854	33.29445	32.5887
17284429	Ighv7-2	immunoglobulin heavy variable 7-2	1.82741	1.935049	1.922602	1.843158	1.969665	1.782819
17284463	Ighv7-3	immunoglobulin heavy variable 7-3	2.058566	2.561893	3.114887	2.07841	2.367788	1.953715
17284471	Ighv7-4	immunoglobulin heavy variable 7-4	1.906016	2.06421	2.899853	2.736591	2.150124	2.422069
17284636	Ighv8-13	immunoglobulin heavy variable 8-13	3.138032	3.574373	3.516816	2.256927	2.915877	2.946761
17284643	Ighv8-14	immunoglobulin heavy variable 8-14	4.49319	4.801649	4.471751	6.421187	4.235611	6.041597
17284593	Ighv8-8	immunoglobulin heavy variable 8-8	4.01461	18.35435	5.391363	3.436286	4.283173	3.168698
17284455	Ighv9-1	immunoglobulin heavy variable 9-1	5.338965	6.992896	6.232409	8.12079	5.840776	4.693617
17284482	Ighv9-4	immunoglobulin heavy variable 9-4	17.58545	24.65315	23.51615	15.34323	24.11936	21.78933
17284505	Ighv10-3	immunoglobulin heavy variable V10-3	3.023187	2.953407	2.670833	3.50142	1.969665	3.596017
17284515	Ighv1-12	immunoglobulin heavy variable V1-12	11.63355	13.04939	10.15283	8.51685	7.603955	13.81899
17279548	Ighv1-13	immunoglobulin heavy variable V1-13 [Source:MGI Symbol;Acc:MGI:5009907]	2.923811	3.009972	2.749006	2.953386	3.729055	2.285976
17284522	Ighv1-17-1	immunoglobulin heavy variable V1-17-1	2.341609	2.089272	2.130349	1.940475	2.14377	2.093301
17284527	Ighv1-18	immunoglobulin heavy variable V1-18	2.406006	2.406006	2.757422	2.304206	3.688822	2.393049
17284530	Ighv1-19	immunoglobulin heavy variable V1-19	2.444569	7.851945	3.687543	2.874518	2.846068	2.32929
17284533	Ighv1-20	immunoglobulin heavy variable V1-20	2.998332	5.806505	4.894257	5.041454	4.352276	5.313712
17284487	Ighv12-3	immunoglobulin heavy variable V12-3	3.860132	3.351275	5.523336	4.20927	4.20927	5.003716
17284539	Ighv1-23	immunoglobulin heavy variable V1-23	20.91346	19.33027	17.21972	17.13839	12.75013	19.67293
17284542	Ighv1-24	immunoglobulin heavy variable V1-24	1.836412	1.898013	1.919632	2.084455	1.977256	1.985277
17284560	Ighv1-42	immunoglobulin heavy variable V1-42	3.339703	8.167248	4.617558	3.417663	3.339703	2.635451
17284562	Ighv1-43	immunoglobulin heavy variable V1-43	3.537769	5.886418	4.233615	4.113681	4.352065	4.20927
17284503	Ighv1-5	immunoglobulin heavy variable V1-5	6.171709	6.122666	8.183738	7.754319	7.022477	7.754319
17284509	Ighv15-2	immunoglobulin heavy variable V15-2	2.549687	2.953407	3.083032	2.953407	3.623113	3.053513
17284582	Ighv1-54	immunoglobulin heavy variable V1-54	2.049369	2.145732	2.017865	1.945242	2.247156	1.780275
17284599	Ighv1-59	immunoglobulin heavy variable V1-59	2.395871	5.361401	2.911797	3.409121	2.193782	4.957456
17284617	Ighv1-62	immunoglobulin heavy variable V1-62	1.83162	3.433548	2.837027	1.795954	2.409911	2.070673
17284614	Ighv1-63	immunoglobulin heavy variable V1-63	10.05618	12.47614	8.805653	12.53213	10.92196	8.324421
17284626	Ighv1-67	immunoglobulin heavy variable V1-67	2.039888	5.775997	3.25584	2.923243	3.449889	3.622084
17284507	Ighv1-7	immunoglobulin heavy variable V1-7	3.962058	8.094377	3.817611	4.006271	4.20927	3.797316
17284640	Ighv1-74	immunoglobulin heavy variable V1-74	2.162035	2.356703	1.734428	1.961803	2.504148	1.930561
17284512	Ighv1-9	immunoglobulin heavy variable V1-9	6.692151	9.127059	8.467112	5.84029	3.241384	8.214712
17284390	Ighv2-4	immunoglobulin heavy variable V2-4	138.3722	168.0422	149.9274	138.0407	150.1062	161.6612
17284469	Ighv3-3	immunoglobulin heavy variable V3-3	2.066716	2.400292	2.329193	2.874657	3.104326	1.996925
17284474	Ighv3-4	immunoglobulin heavy variable V3-4	4.712557	3.458772	3.641013	3.203391	4.073051	3.622862
17279546	Ighv5-8	immunoglobulin heavy variable V5-8 [Source:MGI Symbol;Acc:MGI:5009890]	3.13962	3.66197	3.294935	3.937036	3.294935	3.339703
17284620	Ighv8-11	immunoglobulin heavy variable V8-11	2.253504	2.158995	2.335692	2.102652	2.149275	2.081841
17284568	Ighv8-5	immunoglobulin heavy variable V8-5	2.809941	2.003566	2.385431	2.279251	2.233428	2.751923
17284580	Ighv8-6	immunoglobulin heavy variable V8-6	6.992896	7.675603	7.334456	8.482385	4.773045	4.717524
17284612	Ighv8-9	immunoglobulin heavy variable V8-9	2.813469	2.88916	3.511117	4.39694	4.785567	3.861764
17284457	Ighv9-2	immunoglobulin heavy variable V9-2	3.196781	6.902409	3.122994	4.179818	3.271359	4.20927
17284460	Ighv9-3	immunoglobulin heavy variable V9-3	3.022747	8.334698	2.717669	3.363492	2.890983	4.597055
17449447	Jchain	immunoglobulin joining chain	3.919963	9.30825	4.930521	3.184419	3.942033	3.42397
17459338	Igk-V1	immunoglobulin kappa chain variable 1 (V1)	4.681043	13.19372	11.51626	4.482986	3.375006	3.144019
17459315	Igkv1-122	immunoglobulin kappa chain variable 1-122	4.061155	3.136531	2.347719	2.606909	2.098211	3.057813
17467499	Igkv12-38	immunoglobulin kappa chain variable 12-38	2.105773	2.175324	2.385679	2.030043	2.612516	1.992487
17467492	Igkv12-41	immunoglobulin kappa chain variable 12-41	2.721968	2.511989	1.706145	2.086247	2.713284	2.319012
17467404	Igkv12-89	immunoglobulin kappa chain variable 12-89	3.462322	2.953407	2.986364	2.571571	2.959431	2.770008
17459370	Igkv13-54-1	immunoglobulin kappa chain variable 13-54-1	2.060051	2.060051	2.060051	1.783795	1.673212	2.098124
17459365	Igkv13-84	immunoglobulin kappa chain variable 13-84	3.778202	3.561636	2.707404	2.817391	3.288022	4.390058
17459350	Igkv14-100	immunoglobulin kappa chain variable 14-100	2.481049	1.898013	2.481049	2.481049	4.901794	2.481049
17459347	Igkv15-103	immunoglobulin kappa chain variable 15-103	9.244338	31.89437	11.40828	9.275403	4.916767	8.428641
17467365	Igkv17-134	immunoglobulin kappa chain variable 17-134	2.381119	2.031915	2.237007	2.600682	2.476393	2.11364
17467505	Igkv18-36	immunoglobulin kappa chain variable 18-36	3.140665	3.353692	3.274399	2.955557	4.954571	3.216028
17467407	Igkv1-88	immunoglobulin kappa chain variable 1-88	5.548201	5.780603	5.817221	5.155027	3.935181	3.806355
17467392	Igkv19-93	immunoglobulin kappa chain variable 19-93	5.106731	14.81986	11.56746	8.372161	9.651813	9.186275
17459421	Igk-V21	immunoglobulin kappa chain variable 21 (V21)	61.2607	191.8015	117.4636	67.78011	57.17767	54.96228
17459288	Igkv2-137	immunoglobulin kappa chain variable 2-137	135.6852	152.0213	154.5972	137.628	125.7313	137.628
17459400	Igkv3-5	immunoglobulin kappa chain variable 3-5	2.88906	6.339548	5.075219	4.332321	4.216044	5.208327

17467464	Igkv4-56	immunoglobulin kappa chain variable 4-56	4.071132	4.024445	3.738683	4.436678	4.680426	4.084162
17467451	Igkv4-61	immunoglobulin kappa chain variable 4-61	3.443009	5.308632	4.634038	4.622041	3.410303	4.187154
17467435	Igkv4-70	immunoglobulin kappa chain variable 4-70	2.271885	6.999442	3.084101	2.770929	3.097684	2.306635
17467433	Igkv4-71	immunoglobulin kappa chain variable 4-71	2.489593	2.151362	1.961286	1.874609	2.035326	1.936793
17467430	Igkv4-72	immunoglobulin kappa chain variable 4-72	3.018852	16.969	5.702759	3.492863	3.225427	3.578785
17467401	Igkv4-90	immunoglobulin kappa chain variable 4-90	3.589518	3.37234	6.347551	3.156794	2.997064	4.339473
17467398	Igkv4-91	immunoglobulin kappa chain variable 4-91	5.153205	9.411013	8.18198	6.382404	4.836489	7.490749
17467489	Igkv5-43	immunoglobulin kappa chain variable 5-43	2.103599	4.023971	2.837027	2.372256	3.488701	2.52071
17467483	Igkv5-45	immunoglobulin kappa chain variable 5-45	3.150891	2.126145	2.304206	2.455727	1.56516	2.606909
17459377	Igkv6-25	immunoglobulin kappa chain variable 6-25	3.470564	4.041918	4.20927	3.263862	2.782767	3.342714
17467522	Igkv6-29	immunoglobulin kappa chain variable 6-29	5.213853	7.308625	3.850912	4.585662	3.982957	3.268684
17467513	Igkv7-33	immunoglobulin kappa chain variable 7-33	3.76701	2.749578	3.045861	3.200395	4.960275	3.729055
17467531	Igkv8-24	immunoglobulin kappa chain variable 8-24	3.38616	3.424492	2.308475	2.657611	2.912766	2.281448
17467529	Igkv8-27	immunoglobulin kappa chain variable 8-27	3.861684	2.281686	1.748369	2.387813	2.626224	2.490128
17467519	Igkv8-30	immunoglobulin kappa chain variable 8-30	6.878386	4.359129	6.190174	3.536862	4.399318	3.255209
17459321	Igkv9-120	immunoglobulin kappa chain variable 9-120	2.541147	2.762913	2.753716	3.267529	2.442977	2.576228
17467377	Igkv9-124	immunoglobulin kappa chain variable 9-124	2.376931	2.562976	3.251037	2.771967	2.517078	3.192773
17459415	Igkj1	immunoglobulin kappa joining 1	99.04347	245.5461	158.4474	106.2486	118.0398	102.7867
17459417	Igkj3	immunoglobulin kappa joining 3	8.150508	10.81431	8.686289	9.294389	10.48685	15.51218
17467389	Igkv10-94	immunoglobulin kappa variable 10-94	4.464999	8.925976	6.263808	3.990225	6.030928	3.727323
17459361	Igkv10-95	immunoglobulin kappa variable 10-95	5.141966	5.454738	5.245026	4.939996	3.935481	4.141921
17467384	Igkv10-96	immunoglobulin kappa variable 10-96	2.410212	11.32053	10.43429	4.572236	8.93743	2.692059
17459312	Igkv11-125	immunoglobulin kappa variable 11-125	10.75816	8.072245	6.879911	10.37285	8.51685	9.303606
17459330	Igkv1-115	immunoglobulin kappa variable 1-115	2.338883	2.250881	1.895541	2.27933	3.050932	2.223188
17459324	Igkv1-117	immunoglobulin kappa variable 1-117	2.452223	22.46598	2.915877	1.960729	2.985784	3.25584
17467368	Igkv1-131	immunoglobulin kappa variable 1-131	3.551184	2.317083	2.453685	2.725536	2.810954	2.698766
17459297	Igkv1-132	immunoglobulin kappa variable 1-132	5.510679	2.231479	2.57637	2.243529	2.045452	1.963476
17459294	Igkv1-133	immunoglobulin kappa variable 1-133	4.061605	2.602125	2.934694	2.526009	3.62344	2.718197
17459291	Igkv1-135	immunoglobulin kappa variable 1-135	4.82156	11.82961	8.81628	5.94217	3.965025	3.408531
17467486	Igkv12-44	immunoglobulin kappa variable 12-44	4.333732	6.536915	4.448534	9.009581	6.780727	4.20927
17467480	Igkv12-46	immunoglobulin kappa variable 12-46	3.376293	8.680511	4.315417	3.729055	3.353017	3.584247
17467477	Igkv12-47	immunoglobulin kappa variable 12-47	9.919999	10.41665	10.41665	15.56054	10.06273	11.37228
17459358	Igkv12-98	immunoglobulin kappa variable 12-98	19.23524	24.57007	22.0837	14.61563	26.05539	23.08702
17467508	Igkv1-35	immunoglobulin kappa variable 1-35	1.942857	1.820103	2.049014	1.746855	2.857176	2.236992
17459363	Igkv13-87	immunoglobulin kappa variable 13-87	2.579909	2.097208	2.542909	1.981208	1.990388	1.888826
17459335	Igkv14-111	immunoglobulin kappa variable 14-111	2.879942	2.884358	2.407223	2.750836	4.01105	3.04356
17459309	Igkv14-126	immunoglobulin kappa variable 14-126	2.472276	2.98386	2.045509	2.333362	1.765822	2.045509
17459300	Igkv14-130	immunoglobulin kappa variable 14-130	2.768184	2.544284	2.255364	2.774754	2.832802	1.903824
17459344	Igkv16-104	immunoglobulin kappa variable 16-104	3.792896	4.982501	2.691443	3.822801	3.753353	4.0802
17459318	Igkv17-121	immunoglobulin kappa variable 17-121	3.459971	4.943799	4.647678	3.867684	3.459971	3.098693
17459306	Igkv17-127	immunoglobulin kappa variable 17-127	3.654515	3.54506	3.607652	2.569558	2.295518	2.768914
17459355	Igkv1-99	immunoglobulin kappa variable 1-99	11.47299	9.148213	11.06921	8.624256	7.549128	8.735983
17459341	Igkv2-109	immunoglobulin kappa variable 2-109	6.992896	5.439975	5.301755	6.943774	4.259252	4.440678
17459332	Igkv2-112	immunoglobulin kappa variable 2-112	15.8908	14.71362	12.53708	12.53708	11.52744	16.32227
17459327	Igkv2-116	immunoglobulin kappa variable 2-116	5.470717	13.51547	8.75071	6.674501	9.710597	8.949208
17467387	Igkv2-95-2	immunoglobulin kappa variable 2-95-2	2.288544	1.906359	1.906359	2.119861	2.013813	1.906359
17459412	Igkv3-1	immunoglobulin kappa variable 3-1	2.089764	1.994255	1.945202	1.728703	2.164704	2.177224
17459392	Igkv3-10	immunoglobulin kappa variable 3-10	3.890916	4.783644	3.178751	3.103551	4.323111	3.33406
17459389	Igkv3-12	immunoglobulin kappa variable 3-12	23.52822	14.54882	14.31296	13.39423	12.47225	13.28835
17459409	Igkv3-2	immunoglobulin kappa variable 3-2	2.113919	2.435335	1.85342	1.864502	3.409925	2.202604
17459406	Igkv3-3	immunoglobulin kappa variable 3-3	2.031338	1.889992	2.29528	1.768676	2.00892	2.0903
17459403	Igkv3-4	immunoglobulin kappa variable 3-4	2.78912	2.953407	2.834747	2.606909	3.415886	2.953407
17459398	Igkv3-7	immunoglobulin kappa variable 3-7	3.73503	2.087375	2.216034	2.045878	2.614509	1.795705
17459395	Igkv3-9	immunoglobulin kappa variable 3-9	5.723628	4.84659	4.952648	5.463858	4.912781	3.401875
17467471	Igkv4-50	immunoglobulin kappa variable 4-50	3.425442	9.373449	2.891624	3.092428	2.88168	2.446399
17467469	Igkv4-53	immunoglobulin kappa variable 4-53	2.696915	2.516537	2.899994	2.18968	2.548521	2.085423
17467466	Igkv4-55	immunoglobulin kappa variable 4-55	8.36897	44.83054	9.820864	6.312362	8.015875	7.261411
17467461	Igkv4-57	immunoglobulin kappa variable 4-57	16.63927	19.25245	20.21368	17.67122	15.48855	16.77941
17467458	Igkv4-57-1	immunoglobulin kappa variable 4-57-1	3.021929	8.707992	5.35828	2.34689	3.220713	2.598358
17467456	Igkv4-58	immunoglobulin kappa variable 4-58	40.53876	41.27396	30.03598	39.56382	43.50683	43.81825
17467453	Igkv4-59	immunoglobulin kappa variable 4-59	2.236139	2.995237	3.051968	2.778392	2.134799	4.029107
17467449	Igkv4-62	immunoglobulin kappa variable 4-62	14.69008	32.69754	15.02403	13.06142	13.04441	12.9882
17467447	Igkv4-63	immunoglobulin kappa variable 4-63	8.327191	4.650513	5.722042	4.978462	7.299714	5.320641
17467441	Igkv4-68	immunoglobulin kappa variable 4-68	9.123011	27.9098	8.446303	6.529896	7.935109	8.722006
17467438	Igkv4-69	immunoglobulin kappa variable 4-69	3.062691	2.953407	2.790106	2.047963	2.118451	2.075458
17467427	Igkv4-73	immunoglobulin kappa variable 4-73	10.25382	19.42873	5.723232	6.943774	11.22684	7.045783
17467424	Igkv4-74	immunoglobulin kappa variable 4-74	53.23673	47.25451	43.79851	56.7717	48.81254	58.74687
17467421	Igkv4-78	immunoglobulin kappa variable 4-78	6.434107	5.780603	5.211469	3.837376	5.125597	5.610658
17467418	Igkv4-79	immunoglobulin kappa variable 4-79	11.04323	10.10537	8.619654	10.03668	8.580847	10.81064
17467415	Igkv4-80	immunoglobulin kappa variable 4-80	4.789284	4.800085	4.59075	5.994087	4.514324	3.29231
17467413	Igkv4-81	immunoglobulin kappa variable 4-81	2.9702	2.418965	2.988953	2.418965	2.601674	2.078943
17467410	Igkv4-86	immunoglobulin kappa variable 4-86	9.123011	7.498178	8.399713	8.637836	6.453447	8.399713
17467395	Igkv4-92	immunoglobulin kappa variable 4-92	6.35411	6.565205	7.5585	9.975713	6.381343	11.53663
17467502	Igkv5-37	immunoglobulin kappa variable 5-37	2.719554	3.883991	5.134487	4.142667	3.1104	5.129115
17467495	Igkv5-39	immunoglobulin kappa variable 5-39	3.53206	3.514185	3.129886	4.739287	3.729055	3.767089
17467474	Igkv5-48	immunoglobulin kappa variable 5-48	2.622586	2.7093	2.037514	2.070803	3.080618	3.06473
17467554	Igkv6-13	immunoglobulin kappa variable 6-13	3.196072	23.79341	4.710532	4.456744	4.956357	3.485656
17467551	Igkv6-14	immunoglobulin kappa variable 6-14	3.12096	2.111225	2.130837	2.332117	2.211293	2.211293
17467548	Igkv6-15	immunoglobulin kappa variable 6-15	2.435216	2.235519	2.284233	2.262801	2.919315	2.258571
17459383	Igkv6-17	immunoglobulin kappa variable 6-17	2.641651	4.208132	3.1624	2.935304	2.997978	2.530548
17467540	Igkv6-20	immunoglobulin kappa variable 6-20	5.08455	7.814535	10.07816	8.51685	6.030928	7.057709
17459423	Igkv6-23	immunoglobulin kappa variable 6-23	27.28999	365.3284	167.4109	33.75338	58.62646	26.77046
17467534	Igkv6-23	immunoglobulin kappa variable 6-23	2.210082	2.214652	2.130098	2.788211	2.849581	1.853587
17467516	Igkv6-32	immunoglobulin kappa variable 6-32	2.332117	3.387357	2.255364	2.375268	2.332117	2.285991

17467545	Igkv8-16	immunoglobulin kappa variable 8-16	3.738242	3.263862	4.647678	3.361488	4.089034	3.820734
17459380	Igkv8-18	immunoglobulin kappa variable 8-18	3.536127	3.046874	3.009972	3.007553	2.477852	2.874159
17467543	Igkv8-19	immunoglobulin kappa variable 8-19	6.25318	4.070652	3.826406	3.417331	5.38647	4.802681
17467537	Igkv8-21	immunoglobulin kappa variable 8-21	4.631405	5.539708	4.003328	5.069628	3.603303	5.280227
17459374	Igkv8-26	immunoglobulin kappa variable 8-26	2.449267	3.029249	3.295186	2.40936	2.458673	2.224822
17467525	Igkv8-28	immunoglobulin kappa variable 8-28	6.903988	10.5272	8.014486	7.926918	8.736952	9.12959
17467379	Igkv9-123	immunoglobulin kappa variable 9-123	3.739875	3.104476	3.274331	3.429314	2.28398	2.626006
17459303	Igkv9-129	immunoglobulin kappa variable 9-129	2.317999	2.078899	1.771867	1.722352	2.455063	1.789293
17329016	Iglc2	immunoglobulin lambda constant 2	4.464535	6.155944	5.376845	4.118389	5.798702	4.713569
17329009	Iglc3	immunoglobulin lambda constant 3	9.700506	6.140219	8.19708	8.036849	7.810203	11.82583
17329013	Iglv1	immunoglobulin lambda variable 1	7.716465	10.63774	13.13733	11.50086	8.785839	9.395435
17329023	Iglv2	immunoglobulin lambda variable 2	3.577843	4.20927	3.844671	3.901449	2.651962	3.84515
17329020	Iglv3	immunoglobulin lambda variable 3	2.812767	4.416887	3.822059	2.712588	3.145894	1.865006
17328567	Igll1	immunoglobulin lambda-like polypeptide 1	7.211753	7.07755	6.410203	6.943774	8.577873	5.866663
17360920	Ighmbp2	immunoglobulin mu binding protein 2	16.87938	15.93017	15.91516	17.7236	13.10295	15.93293
17527661	Islr	immunoglobulin superfamily containing leucine-rich repeat	10.54341	11.20383	11.11996	8.01732	6.870047	9.172786
17527666	Islr2	immunoglobulin superfamily containing leucine-rich repeat 2	4.297269	4.321613	4.4506	4.4506	4.74448	5.101106
17518483	Igdcc3	immunoglobulin superfamily, DCC subclass, member 3	11.00716	12.50801	10.76167	10.48438	8.482503	14.56173
17518458	Igdcc4	immunoglobulin superfamily, DCC subclass, member 4	7.150678	12.55709	17.18383	7.319829	20.26053	9.224048
17541528	Igsf1	immunoglobulin superfamily, member 1	4.702083	3.523746	3.509073	3.519109	4.176806	4.133317
17405489	Igsf10	immunoglobulin superfamily, member 10	3.771269	4.361638	4.072063	4.286797	3.703322	3.68739
17325651	Igsf11	immunoglobulin superfamily, member 11	181.126	176.0718	162.9765	139.4525	115.3369	136.6991
17431938	Igsf21	immunoglobulin superfamily, member 21	11.69995	11.32736	11.83838	12.50367	11.68439	12.01696
17487500	Igsf23	immunoglobulin superfamily, member 23	5.901943	5.664427	5.503083	6.811016	6.150228	5.96619
17401023	Igsf3	immunoglobulin superfamily, member 3	7.167152	7.532141	9.465246	7.698994	7.540081	7.698994
17327524	Igsf5	immunoglobulin superfamily, member 5	48.93619	45.06639	47.43796	39.7921	47.41067	40.35177
17495839	Igsf6	immunoglobulin superfamily, member 6	46.33145	60.89197	74.32677	53.34089	48.54867	49.76536
17219519	Igsf8	immunoglobulin superfamily, member 8	7.573762	8.933218	10.26847	7.05497	8.933218	8.813713
17219554	Igsf9	immunoglobulin superfamily, member 9	5.348113	6.960447	5.749235	7.613448	5.463555	5.295144
17515617	Igsf9b	immunoglobulin superfamily, member 9B	6.073424	6.992896	6.992896	7.988862	6.384749	6.238417
17227401	Igfn1	immunoglobulin-like and fibronectin type III domain containing 1	6.213043	6.437632	5.626041	5.528967	5.790508	7.211403
17325373	Ildr1	immunoglobulin-like domain containing receptor 1	6.061522	7.074411	6.891127	6.29061	5.112327	6.766313
17219053	Ildr2	immunoglobulin-like domain containing receptor 2	26.56804	43.1306	45.66351	24.46691	43.55269	23.14903
17344192	G6b	immunoreceptor tyrosine-based inhibitory motif (ITIM) containing platelet recepto	13.06323	18.60554	16.10269	13.56164	11.24982	13.56164
17302475	Irg1	immunoresponsive gene 1	4.557176	5.121016	5.268747	5.376957	4.350677	5.213709
17373984	Immp1l	IMP1 inner mitochondrial membrane peptidase-like (S. cerevisiae)	65.63029	59.78783	56.92301	61.70134	57.91399	70.4174
17275223	Immp2l	IMP2 inner mitochondrial membrane peptidase-like (S. cerevisiae)	64.09634	62.56657	55.05799	69.15252	72.65701	80.34186
17517605	Imp3	IMP3, U3 small nucleolar ribonucleoprotein, homolog (yeast)	113.4938	112.656	95.92068	99.09429	85.55439	114.4275
17211700	Imp4	IMP4, U3 small nucleolar ribonucleoprotein, homolog (yeast)	46.71487	49.11732	53.82782	49.46239	49.11732	44.96623
17548906	Imp4	IMP4, U3 small nucleolar ribonucleoprotein, homolog (yeast)	19.99753	17.93493	17.23632	12.71818	18.79035	16.93575
17348604	Impact	impact, RWD domain protein	61.56293	54.23755	48.57392	36.72346	41.94567	46.94436
17296013	Ipo11	importin 11	37.33689	35.31233	33.66272	31.77918	31.58418	39.41492
17428967	Ipo13	importin 13	23.62169	22.70503	21.1615	24.15885	17.01187	17.18014
17306719	Ipo4	importin 4	58.97044	55.59834	53.27844	56.89382	40.28358	52.4747
17306753	Ipo4	importin 4	15.70639	14.02161	16.55815	15.57057	11.02579	14.88606
17302773	Ipo5	importin 5	72.41316	67.66838	74.10968	74.7976	78.22457	74.24696
17481695	Ipo7	importin 7	185.0639	159.0416	134.6154	156.1252	154.8674	150.7872
17472990	Ipo8	importin 8	101.4996	94.41211	79.74433	82.97817	103.1414	87.40476
17227320	Ipo9	importin 9	45.26456	39.71522	44.06343	48.46663	38.62786	36.31969
17491529	Ipw	imprinted gene in the Prader-Willi syndrome region	2.885417	3.32635	3.009972	3.009972	3.705094	3.009972
17491535	Ipw	imprinted gene in the Prader-Willi syndrome region	4.681043	4.113681	4.950108	4.20927	2.549457	8.06928
17543785	Xist	inactive X specific transcripts	3.345264	3.724405	2.62026	2.468235	3.287384	2.639034
17415635	Inadl	InaD-like (Drosophila)	19.75957	17.58813	21.26959	19.44814	19.29667	19.51904
17486870	Inafm1	InaF motif containing 1	14.62404	15.186	15.45391	15.43197	16.33404	14.92563
17374610	Inafm2	InaF motif containing 2	43.85684	41.53858	32.9807	41.90266	35.11998	41.66286
17512833	Ist1	increased sodium tolerance 1 homolog (yeast)	78.40534	95.63322	92.45888	91.143	100.9341	99.22144
17224433	Ihh	Indian hedgehog	28.87602	23.74415	26.58812	31.29434	28.87602	32.09174
17508188	Ido1	indoleamine 2,3-dioxygenase 1	8.418948	6.349399	7.965197	8.51685	6.413136	8.115556
17508174	Ido2	indoleamine 2,3-dioxygenase 2	103.928	93.12135	83.48933	101.8209	89.88323	95.01675
17467062	Inmt	indolethylamine N-methyltransferase	419.3468	644.7786	550.5539	458.542	437.5488	605.4861
17213490	Icos	inducible T cell co-stimulator	7.74422	8.029221	7.394333	6.500272	9.42923	8.555072
17218149	Ivns1abp	influenza virus NS1A binding protein	154.4858	146.0998	164.0464	143.8917	184.5822	193.7456
17214588	Inha	inhibin alpha	8.75702	9.488237	7.97962	9.196341	7.767498	9.892259
17245890	Inhbe	inhibin beta E	116.573	185.6974	193.0352	162.8229	104.4023	84.30054
17285438	Inhba	inhibin beta-A	28.60787	23.39487	13.39247	21.94773	20.76814	15.21139
17226189	Inhbb	inhibin beta-B	8.680993	14.41141	13.10868	16.17787	17.64797	11.91379
17245895	Inhbc	inhibin beta-C	143.0673	84.6196	83.58893	77.01406	81.8063	65.17469
17529322	Ibtk	inhibitor of Bruton agammaglobulinemia tyrosine kinase	168.3734	125.8499	132.4175	123.3257	115.5794	94.26629
17265365	Inca1	inhibitor of CDK, cyclin A1 interacting protein 1	20.92898	30.73201	35.74724	51.84238	42.24885	37.56117
17377778	Id1	inhibitor of DNA binding 1	12.48384	14.41951	14.24684	14.29927	13.63129	20.14849
17280310	Id2	inhibitor of DNA binding 2	324.0831	289.5503	292.5968	310.0915	300.6039	326.8829
17420016	Id3	inhibitor of DNA binding 3	26.62778	23.30682	30.17078	23.30682	20.44491	26.62778
17287081	Id4	inhibitor of DNA binding 4	13.68297	7.813452	9.920343	10.79192	10.04559	8.522638
17499028	Ing1	inhibitor of growth family, member 1	32.45368	37.9611	37.9611	33.67719	42.82733	35.99863
17509274	Ing2	inhibitor of growth family, member 2	10.33674	11.1887	14.97475	15.40375	18.49649	14.79656
17456318	Ing3	inhibitor of growth family, member 3	19.28143	20.80531	19.68889	20.49216	17.70028	18.00019
17463020	Ing4	inhibitor of growth family, member 4	47.73912	57.09451	64.9357	67.43333	58.69436	65.64576
17216087	Ing5	inhibitor of growth family, member 5	14.67166	17.05946	12.85119	15.14889	11.98618	14.31961
17425425	Ikbkap	inhibitor of kappa light polypeptide enhancer in B cells, kinase complex-associatec	38.73699	26.9604	25.44764	28.34377	31.27223	26.14603
17508082	Ikbkb	inhibitor of kappaB kinase beta	48.79056	43.12253	47.38899	38.86905	36.84482	47.38899
17226771	Ikbke	inhibitor of kappaB kinase epsilon	32.76106	28.20013	30.54258	31.6497	33.35659	27.50744
17535877	Ikbkg	inhibitor of kappaB kinase gamma	39.1527	41.70909	39.12612	43.91951	37.87385	38.62786
17362559	Incenp	inner centromere protein	7.111579	7.225863	8.046715	7.452735	6.370736	7.405566
17459530	Immt	inner membrane protein, mitochondrial	191.7377	194.8297	187.438	208.8408	171.1218	199.1125

17468113	Ino80b	INO80 complex subunit B	22.57462	22.62789	21.13043	21.23938	23.85219	20.8518
17353131	Ino80c	INO80 complex subunit C	16.96759	12.60295	12.64276	12.60295	16.80071	13.91974
17223666	Ino80d	INO80 complex subunit D	52.91666	73.69424	78.107	63.78432	63.78432	65.72088
17213572	Ino80dos	INO80 complex subunit D, opposite strand	4.760556	3.706404	4.647678	3.82829	5.05363	4.334964
17496438	Ino80e	INO80 complex subunit E	18.64956	20.94727	19.25445	17.7568	18.71846	27.57311
17389861	Ino80	INO80 homolog (S. cerevisiae)	38.52973	49.41578	49.03092	52.29895	51.11837	51.52783
17465373	Impdh1	inosine 5-phosphate dehydrogenase 1	14.31296	14.60267	15.42961	12.45307	14.90299	15.96588
17521838	Impdh2	inosine 5-phosphate dehydrogenase 2	42.48377	48.14286	51.22762	38.32649	49.22775	44.04328
17376378	Itpa	inosine triphosphatase (nucleoside triphosphate pyrophosphatase)	23.50442	22.16129	22.61597	24.12488	21.1615	21.0146
17404109	Impa1	inositol (myo)-1(or 4)-monophosphatase 1	116.9923	104.2281	96.81643	104.2281	106.5362	117.4677
17351446	Impa2	inositol (myo)-1(or 4)-monophosphatase 2	19.70213	18.21355	19.95212	18.69901	18.83129	15.3594
17287126	Ippk	inositol 1,3,4,5,6-pentakisphosphate 2-kinase	13.25817	12.26725	9.375918	11.44607	15.14332	13.20617
17283463	Itpk1	inositol 1,3,4-triphosphate 5/6 kinase	47.32465	49.61382	54.87511	54.87511	47.83683	48.60423
17472777	Itpr2	inositol 1,4,5-triphosphate receptor 2	71.28072	57.42154	59.72115	62.52191	55.54942	61.16778
17335060	Itpr3	inositol 1,4,5-triphosphate receptor 3	12.08001	12.39452	12.12296	10.39422	9.631029	10.29534
17365617	Itprip	inositol 1,4,5-triphosphate receptor interacting protein	8.583286	9.035785	8.332908	7.293392	8.332908	8.332908
17391233	Itpripl1	inositol 1,4,5-triphosphate receptor interacting protein-like 1	21.20349	28.62989	34.13406	25.67353	40.06887	36.92894
17495549	Itpripl2	inositol 1,4,5-triphosphate receptor interacting protein-like 2	9.894316	9.894316	9.894316	9.894316	9.894316	10.39458
17374871	Itpka	inositol 1,4,5-trisphosphate 3-kinase A	5.51171	7.205258	5.51171	4.896633	7.659446	5.51171
17220110	Itpkb	inositol 1,4,5-trisphosphate 3-kinase B	27.84024	25.06461	28.38683	24.68461	17.94599	16.66478
17488167	Itpkc	inositol 1,4,5-trisphosphate 3-kinase C	11.89868	12.2455	10.20066	9.652415	12.22498	9.110056
17461341	Itpr1	inositol 1,4,5-trisphosphate receptor 1	88.54016	108.4856	107.8947	97.86425	93.45436	89.653
17521593	Ip6k1	inositol hexaphosphate kinase 1	42.35851	47.89224	40.05249	46.07557	49.22775	52.38384
17521902	Ip6k2	inositol hexaphosphate kinase 2	61.41674	63.40435	60.39637	59.68804	72.55736	69.94165
17342699	Ip6k3	inositol hexaphosphate kinase 3	14.53007	14.87915	12.17264	14.03386	11.43726	13.65209
17423037	Impad1	inositol monophosphatase domain containing 1	33.29445	33.29445	30.61125	31.71624	33.29445	36.79684
17259810	Inpp5j	inositol polyphosphate 5-phosphatase J	5.032272	5.669769	6.863146	6.265719	6.265719	5.651681
17253037	Inpp5k	inositol polyphosphate 5-phosphatase K	35.88154	51.92221	51.04543	47.8773	43.67906	44.91514
17234175	Ipmk	inositol polyphosphate multikinase	84.06014	98.89461	107.7826	90.40872	115.3569	123.6133
17234185	Ipmk	inositol polyphosphate multikinase	6.307945	7.846885	5.82735	6.911553	8.829734	8.106111
17493916	Inppl1	inositol polyphosphate phosphatase-like 1	34.05465	36.62483	40.64934	32.46785	29.75458	25.26696
17222940	Inpp1	inositol polyphosphate-1-phosphatase	23.03507	26.22262	21.16531	19.97163	19.05873	17.04882
17211939	Inpp4a	inositol polyphosphate-4-phosphatase, type I	33.62168	37.98848	36.88213	38.26782	45.52509	47.96167
17502816	Inpp4b	inositol polyphosphate-4-phosphatase, type II	5.394391	6.31871	4.571793	4.232999	3.830705	4.668147
17484382	Inpp5a	inositol polyphosphate-5-phosphatase A	34.34385	34.34385	40.77209	34.34385	32.5878	30.89454
17418306	Inpp5b	inositol polyphosphate-5-phosphatase B	22.54757	20.95133	22.54757	22.54757	21.11213	20.92376
17215309	Inpp5d	inositol polyphosphate-5-phosphatase D	10.98178	13.23733	17.37956	13.24394	12.66495	8.215851
17382993	Inpp5e	inositol polyphosphate-5-phosphatase E	10.11728	11.45488	11.45488	10.92098	10.79454	13.59317
17483740	Inpp5f	inositol polyphosphate-5-phosphatase F	31.19515	22.52117	20.74972	25.77195	23.04417	18.34583
17482047	Insc	inscuteable homolog (Drosophila)	25.27519	19.57594	19.12834	21.5305	18.95912	15.14311
17364251	Ide	insulin degrading enzyme	256.0532	212.851	184.0699	205.1734	174.8483	185.9215
17360301	Ins1	insulin I	3.229386	4.596545	3.595419	3.875923	3.977936	3.791162
17498160	Ins2	insulin II	4.832735	2.875454	3.037659	4.295988	4.532666	3.190826
17435584	Insig1	insulin induced gene 1	521.8314	272.645	442.2045	214.9936	460.0032	222.3071
17226346	Insig2	insulin induced gene 2	401.1811	263.4271	239.5462	319.4087	244.058	249.7672
17507057	Insr	insulin receptor	163.7306	184.8396	189.7637	177.7764	187.6434	188.3889
17214753	Irs1	insulin receptor substrate 1	4.277714	6.943918	5.295218	5.871381	5.120306	5.343926
17224878	Irs1	insulin receptor substrate 1	39.6899	53.42563	39.6899	45.31824	37.59763	44.14689
17507374	Irs2	insulin receptor substrate 2	83.82333	84.8175	81.21082	45.5213	59.9073	56.33306
17454114	Irs3	insulin receptor substrate 3	7.593105	8.535999	6.361072	7.024424	7.250297	7.088449
17545017	Irs4	insulin receptor substrate 4	3.445707	3.821423	3.566948	4.160307	3.758169	3.937992
17398825	Insrr	insulin receptor-related receptor	3.906807	4.184717	3.578587	4.135753	4.18202	4.20927
17517532	Isl2	insulin related protein 2 (islet 2)	7.569615	7.675975	7.075588	9.851204	6.958566	8.817135
17427655	Insl5	insulin-like 5	2.621477	2.591559	2.885237	3.329049	3.18846	2.953407
17363790	Insl6	insulin-like 6	3.555272	2.376272	2.886778	2.660228	2.513278	2.88914
17236288	Igf1	insulin-like growth factor 1	340.6697	293.9853	313.226	294.8035	397.4942	274.6442
17243845	Igf1os	insulin-like growth factor 1, opposite strand	3.948569	3.600607	4.37587	3.054635	3.916161	4.213941
17498142	Igf2	insulin-like growth factor 2	7.784368	8.281832	7.784368	7.594263	6.373165	6.135454
17268255	Igf2bp1	insulin-like growth factor 2 mRNA binding protein 1	5.386432	5.081167	4.95512	5.480889	4.896225	4.95512
17329258	Igf2bp2	insulin-like growth factor 2 mRNA binding protein 2	9.957406	14.27699	15.42608	14.14284	11.75376	11.10895
17466641	Igf2bp3	insulin-like growth factor 2 mRNA binding protein 3	22.18404	22.18404	24.7978	23.9093	25.22776	17.41816
17340845	Igf2r	insulin-like growth factor 2 receptor	63.32003	65.3081	72.16013	59.10056	56.02776	64.81384
17485266	Igf2os	insulin-like growth factor 2, opposite strand	5.17791	5.468973	5.57708	5.637596	5.318207	5.338965
17247208	Igfbp1	insulin-like growth factor binding protein 1	754.1773	812.9935	991.3976	502.3608	740.0044	716.6625
17214106	Igfbp2	insulin-like growth factor binding protein 2	445.7621	569.4992	559.5643	617.3223	453.7079	431.9618
17260474	Igfbp3	insulin-like growth factor binding protein 3	50.42335	52.49507	33.52185	44.6838	54.31015	61.5288
17256264	Igfbp4	insulin-like growth factor binding protein 4	564.2497	701.6657	795.9809	778.9463	845.9919	821.9176
17224180	Igfbp5	insulin-like growth factor binding protein 5	11.24732	8.738769	11.58134	10.77832	8.357492	12.18657
17315305	Igfbp6	insulin-like growth factor binding protein 6	8.294931	7.469336	7.612393	7.469336	7.469336	6.593022
17449084	Igfbp7	insulin-like growth factor binding protein 7	204.5897	218.8988	233.573	199.9853	94.00333	146.2852
17334468	Igfals	insulin-like growth factor binding protein, acid labile subunit	47.54132	40.34506	41.93782	50.72482	55.1294	32.13559
17424972	Igfbpl1	insulin-like growth factor binding protein-like 1	5.330312	7.731027	7.108672	7.467421	6.641366	7.408133
17479099	Igf1r	insulin-like growth factor I receptor	8.233353	8.488267	8.52571	8.314906	11.60175	8.900707
17377253	Insm1	insulinoma-associated 1	15.13892	15.78848	16.24293	16.24293	18.36708	14.17729
17275572	Insm2	insulinoma-associated 2	9.597975	8.469636	10.41037	8.480151	9.625423	8.666443
17544078	Itm2a	integral membrane protein 2A	4.136126	4.136126	3.909055	4.801649	3.947393	4.552377
17308603	Itm2b	integral membrane protein 2B	1568.99	1649.108	1560.367	1560.367	1410.744	1560.367
17214998	Itm2c	integral membrane protein 2C	42.56129	49.65476	52.34972	40.2512	38.22249	45.0992
17454450	Ints1	integrator complex subunit 1	32.11265	29.74407	29.18955	32.36853	26.54632	33.0102
17501609	Ints10	integrator complex subunit 10	16.6448	13.87158	12.44039	14.24417	12.98433	11.45789
17402746	Ints12	integrator complex subunit 12	32.49915	30.71923	26.91484	27.97527	35.43713	27.69339
17267237	Ints2	integrator complex subunit 2	13.26038	9.720294	12.88607	13.28338	10.82414	9.805016
17407272	Ints3	integrator complex subunit 3	55.33959	46.43047	47.69447	50.31198	45.09045	49.62792
17480358	Ints4	integrator complex subunit 4	50.76527	46.96942	47.15831	48.66862	44.26732	55.22961

17357276	Ints5	integrator complex subunit 5	20.14556	21.77544	24.97149	23.82625	23.22715	19.66148
17307550	Ints6	integrator complex subunit 6	65.65031	65.38374	60.1132	63.09134	52.09453	52.09453
17220809	Ints7	integrator complex subunit 7	38.16716	39.60443	38.01008	40.40187	39.60443	34.80469
17423228	Ints8	integrator complex subunit 8	32.77946	30.961	29.76407	35.13386	31.79526	25.78661
17301342	Ints9	integrator complex subunit 9	36.8944	35.23555	40.33611	35.87582	36.69064	36.8944
17296388	Itga1	integrin alpha 1	92.0675	92.0675	105.0469	92.0675	73.35991	83.68749
17518191	Itga11	integrin alpha 11	4.735511	5.146601	4.962682	4.693552	4.543361	4.936334
17296355	Itga2	integrin alpha 2	5.151669	5.018131	5.508578	7.338067	5.768355	5.346779
17270222	Itga2b	integrin alpha 2b	9.074386	10.96292	10.14045	10.49973	10.68237	12.17365
17268137	Itga3	integrin alpha 3	8.556317	10.97364	12.08169	12.87526	11.01174	12.71641
17372307	Itga4	integrin alpha 4	19.70063	13.49927	13.23568	12.60295	16.77929	12.60295
17322369	Itga5	integrin alpha 5 (fibronectin receptor alpha)	43.82281	28.33435	33.168	26.76118	23.98732	24.57756
17371800	Itga6	integrin alpha 6	9.06282	10.47124	13.23476	10.94432	10.94432	9.140607
17238605	Itga7	integrin alpha 7	14.64382	12.14297	17.70788	12.81774	19.44315	14.32973
17381717	Itga8	integrin alpha 8	30.19044	30.71241	30.38126	28.30431	21.85058	27.36159
17522916	Itga9	integrin alpha 9	32.73813	36.4064	42.41786	35.38681	33.93989	36.16895
17252574	Itgae	integrin alpha E, epithelial-associated	3.787905	5.352267	3.980032	4.090338	3.488701	4.203643
17511389	Itfg1	integrin alpha FG-GAP repeat containing 1	140.2202	148.4548	124.4886	134.8414	124.2541	119.4042
17471324	Itfg2	integrin alpha FG-GAP repeat containing 2	27.42786	24.69231	27.42786	28.79367	29.9273	28.75757
17483264	Itgal	integrin alpha L	32.79559	41.49599	45.24919	30.38358	38.97238	29.12871
17483577	Itgam	integrin alpha M	7.694086	8.174384	8.721643	7.50577	8.219155	7.276981
17372515	Itgav	integrin alpha V	100.603	86.07188	82.38844	82.40614	87.2099	78.56746
17483615	Itgax	integrin alpha X	5.568042	6.396089	6.943582	6.574677	6.413669	6.240753
17507014	Itgb1	integrin beta 1 (fibronectin receptor beta)	240.7063	221.0854	224.6943	220.1404	217.3054	224.6943
17280194	Itgb1bp1	integrin beta 1 binding protein 1	23.3495	24.36114	23.78549	24.49304	24.42726	22.47377
17536759	Itgb1bp2	integrin beta 1 binding protein 2	10.36422	11.4272	12.59518	11.08364	8.692855	11.05824
17234647	Itgb2	integrin beta 2	32.67171	44.00544	48.46966	32.88095	34.40294	31.96697
17332473	Itgb2l	integrin beta 2-like	3.240912	4.015362	4.636062	3.874768	3.954102	3.452664
17257235	Itgb3	integrin beta 3	19.68111	24.30279	20.45228	16.70989	17.43386	18.22908
17427563	Itgb3bp	integrin beta 3 binding protein (beta3-endonexin)	4.223591	5.374945	5.187718	5.469428	4.527266	5.330312
17258473	Itgb4	integrin beta 4	7.711171	8.51685	8.67997	8.51685	8.51685	6.743932
17325109	Itgb5	integrin beta 5	117.9245	119.5293	140.6007	118.1536	128.9985	118.5088
17385654	Itgb6	integrin beta 6	4.11856	3.703322	3.703322	3.809997	3.827069	3.703322
17322163	Itgb7	integrin beta 7	7.451805	7.900643	6.57878	6.582657	9.141241	6.479534
17284839	Itgb8	integrin beta 8	8.668786	9.187421	9.082692	11.42174	12.70399	10.5958
17439815	Ibsp	integrin binding sialoprotein	3.989727	3.581738	3.426843	4.513823	3.982819	3.188637
17481460	Ilk	integrin linked kinase	85.84606	102.8181	106.8668	102.8181	91.0546	102.8181
17400650	Itga10	integrin, alpha 10	6.745802	6.056986	7.449895	5.928224	5.117503	5.279605
17483648	Itgad	integrin, alpha D	4.20927	4.20927	4.20927	4.20927	5.368615	3.949856
17302936	Itgbl1	integrin, beta-like 1	3.343456	4.207899	4.197877	4.176169	3.984835	3.928858
17225478	Ilkap	integrin-linked kinase-associated serine/threonine phosphatase 2C	35.73907	34.32886	35.82885	37.66989	41.73367	37.99085
17229756	Itln1	intelectin 1 (galactofuranose binding)	3.691993	4.170875	4.626689	3.885445	5.331088	4.822763
17298267	Itih4	inter alpha-trypsin inhibitor, heavy chain 4	1788.693	2050.884	2217.933	1487.609	2089.497	1723.78
17239005	Ipcef1	interaction protein for cytohesin exchange factors 1	3.010431	3.517085	3.602379	3.749349	3.975262	3.749349
17294167	Ice1	interactor of little elongation complex ELL subunit 1	25.33377	26.13009	25.41855	29.72593	24.0746	26.56307
17518935	Ice2	interactor of little elongation complex ELL subunit 2	22.03996	26.75951	22.20973	28.4921	23.53001	28.07375
17366670	Itih5	inter-alpha (globulin) inhibitor H5	39.33168	49.65441	39.84343	54.34668	31.48516	38.59173
17538614	Itih5l-ps	inter-alpha (globulin) inhibitor H5-like, pseudogene	4.554997	4.271788	4.325959	4.992595	4.367901	4.392493
17304461	Itih1	inter-alpha trypsin inhibitor, heavy chain 1	648.845	565.1382	566.0125	579.6507	802.3845	581.8688
17381589	Itih2	inter-alpha trypsin inhibitor, heavy chain 2	814.2511	907.5542	949.4248	777.7755	1069.284	982.8517
17304434	Itih3	inter-alpha trypsin inhibitor, heavy chain 3	796.9415	895.0969	946.9668	663.2689	1114.878	717.1097
17515074	Icam1	intercellular adhesion molecule 1	28.54409	30.94834	29.97691	21.00251	18.79478	16.56608
17270877	Icam2	intercellular adhesion molecule 2	11.43473	10.20412	15.39788	13.23641	10.59778	8.863583
17515084	Icam4	intercellular adhesion molecule 4, Landsteiner-Wiener blood group	7.678742	7.678742	8.411773	7.678742	7.678742	7.104288
17515090	Icam5	intercellular adhesion molecule 5, telencephalin	6.992896	6.287864	5.721011	5.291585	6.752679	8.371522
17327069	Ifnar1	interferon (alpha and beta) receptor 1	118.7201	110.3551	99.50727	102.1532	111.4552	100.028
17327038	Ifnar2	interferon (alpha and beta) receptor 2	275.8003	303.4384	319.7233	287.4625	273.6845	263.2501
17230102	Ifi202b	interferon activated gene 202B	2.121508	2.628737	2.403072	2.110464	2.357683	3.281351
17230087	Ifi203	interferon activated gene 203	17.68605	26.7699	28.73446	26.92025	21.4202	27.19463
17230045	Ifi204	interferon activated gene 204	17.13436	15.89521	17.50943	22.35569	17.50943	18.62954
17230111	Ifi205	interferon activated gene 205	15.65813	14.35001	22.09258	18.35626	22.39384	17.03287
17415373	Ifna1	interferon alpha 1	27.20274	34.23405	35.99214	22.29456	27.17786	22.52148
17415361	Ifna11	interferon alpha 11	2.224529	1.910341	1.931632	1.775826	1.842954	1.837202
17427077	Ifna12	interferon alpha 12	2.623986	3.05847	3.281943	3.047508	4.103315	3.415957
17427092	Ifna13	interferon alpha 13	3.138184	2.058381	2.213869	2.377986	2.702229	2.218093
17427073	Ifna14	interferon alpha 14	18.86683	14.46315	19.49537	17.40669	14.374	19.1752
17427067	Ifna15	interferon alpha 15	9.970114	10.25524	11.23906	11.16422	10.39199	14.21439
17427096	Ifna16	interferon alpha 16	2.269068	2.597349	2.039577	2.111429	2.255364	2.351433
17427100	Ifna2	interferon alpha 2	4.241251	3.739175	3.956652	3.94846	3.585713	6.410247
17415369	Ifna4	interferon alpha 4	24.55049	29.89723	34.81386	25.52449	20.12476	31.24839
17415367	Ifna5	interferon alpha 5	17.16229	22.17005	18.80338	20.12365	12.04665	12.49639
17415365	Ifna6	interferon alpha 6	4.20927	4.571729	4.20927	4.103657	3.709771	4.997962
17415359	Ifna7	interferon alpha 7	8.657017	8.931546	8.986816	7.656632	6.142304	12.33675
17427075	Ifna9	interferon alpha 9	2.613567	2.596575	3.044869	2.657095	2.574817	2.498721
17427104	Ifnab	interferon alpha B	3.338986	2.986654	2.9314	3.174083	2.109031	3.359112
17427064	Ifnb1	interferon beta 1, fibroblast	4.146804	4.146804	4.220547	3.728874	4.497178	4.146804
17427128	Ifne	interferon epsilon	6.958132	7.005995	7.009735	6.944833	6.775089	8.835612
17237589	Ifng	interferon gamma	3.29866	3.883991	3.516548	3.077012	3.339703	3.977412
17249980	Igtp	interferon gamma induced GTPase	64.4398	52.90529	61.25476	80.43101	69.98675	58.56107
17510136	Ifi30	interferon gamma inducible protein 30	68.5417	83.69851	131.7227	75.0604	80.59341	69.35317
17248911	Ifi47	interferon gamma inducible protein 47	11.45932	8.725695	9.15773	12.25756	11.18893	9.016578
17231859	Ifngr1	interferon gamma receptor 1	57.01185	77.29429	72.04967	53.59439	61.65859	53.90884
17327084	Ifngr2	interferon gamma receptor 2	71.91795	80.48344	68.93238	66.65079	71.39445	74.43917
17364133	Ifit1bl2	interferon induced protein with tetratricopeptide repeats 1B like 2	5.353306	4.259281	4.207082	5.567849	5.456327	5.515952

17364126	Ifit1bl1	interferon induced protein with tetratricpeptide repeats 1B like 1	3.346702	2.803113	2.368281	3.07424	4.428291	3.137075
17484701	Ifitm1	interferon induced transmembrane protein 1	3.642503	3.531497	4.602443	4.641593	3.861951	3.643538
17498086	Ifitm10	interferon induced transmembrane protein 10	8.459134	5.525979	7.992628	6.992896	8.395405	8.270245
17497713	Ifitm2	interferon induced transmembrane protein 2	68.61918	91.31753	101.8534	73.90192	84.3549	56.41747
17357211	Gm2518	interferon induced transmembrane protein 2 pseudogene	10.7902	12.36971	14.78405	12.01046	8.295851	12.26793
17497718	Ifitm3	interferon induced transmembrane protein 3	903.199	1020.825	1154.114	1056.751	1038.856	1063.195
17497709	Ifitm5	interferon induced transmembrane protein 5	5.688428	5.576036	5.520504	5.460526	6.178215	6.421365
17497724	Ifitm6	interferon induced transmembrane protein 6	5.097041	4.12113	4.756598	3.864629	4.12113	3.933327
17328326	Ifitm7	interferon induced transmembrane protein 7	4.505196	3.918387	3.315508	3.243429	3.759759	2.558131
17385797	Ifih1	interferon induced with helicase C domain 1	119.0753	93.32295	95.97389	134.0168	129.9065	123.85
17350925	Iigp1	interferon inducible GTPase 1	726.9438	667.7388	623.8217	766.398	779.6215	699.5581
17412692	Ifnk	interferon kappa	2.877069	3.344591	2.482098	3.49829	4.920995	4.359552
17488485	Ifnl2	interferon lambda 2	41.42587	58.0084	47.7805	45.92284	48.6167	63.5945
17475863	Ifnl3	interferon lambda 3	17.37113	25.96111	21.74105	21.31164	17.04291	18.82881
17419880	Ifnlr1	interferon lambda receptor 1	5.330682	5.936489	6.743418	5.1983	6.040299	4.567928
17249593	Irf1	interferon regulatory factor 1	27.01521	30.13003	41.93404	32.88984	40.36044	37.53436
17501050	Irf2	interferon regulatory factor 2	91.62758	95.50604	96.23501	92.96012	128.1411	118.152
17474396	Irf2bp1	interferon regulatory factor 2 binding protein 1	23.26034	20.28498	22.92596	24.24054	21.87089	21.7919
17506938	Irf2bp2	interferon regulatory factor 2 binding protein 2	16.2834	16.67078	16.27167	21.18528	15.77316	16.13532
17514243	Irf2bp2	interferon regulatory factor 2 binding protein 2	138.7515	151.9118	125.6511	112.4944	124.9217	151.612
17282776	Irf2bpl	interferon regulatory factor 2 binding protein-like	30.00997	27.2001	35.09443	35.90319	38.11614	43.79244
17477655	Irf3	interferon regulatory factor 3	39.38051	32.48856	26.03246	33.95566	27.94561	29.41602
17286295	Irf4	interferon regulatory factor 4	4.971117	4.838702	4.026901	4.507602	4.570525	5.799908
17456692	Irf5	interferon regulatory factor 5	7.08673	7.866435	7.585583	9.989275	8.713305	8.775614
17220907	Irf6	interferon regulatory factor 6	76.96443	58.66955	78.00529	72.53674	69.76396	57.93326
17497813	Irf7	interferon regulatory factor 7	95.55172	66.9267	84.76871	115.1292	94.9858	104.4885
17506279	Irf8	interferon regulatory factor 8	15.52186	16.62405	18.24197	14.94685	19.62866	14.2585
17300591	Irf9	interferon regulatory factor 9	48.85926	48.85926	54.68677	56.96287	41.03122	55.66351
17398888	Isg20l2	interferon stimulated exonuclease gene 20-like 2	26.96732	29.20351	32.9583	33.2993	35.572	36.6162
17415319	Ifnz	interferon zeta	3.644169	4.165588	4.437601	3.263862	5.409555	5.048238
17278200	Ifi27	interferon, alpha-inducible protein 27	248.8961	208.8828	160.4111	230.8721	236.2385	234.5432
17283549	Ifi27l2a	interferon, alpha-inducible protein 27 like 2A	18.76432	20.22854	20.14961	22.95219	20.22854	20.22854
17283556	Ifi27l2b	interferon, alpha-inducible protein 27 like 2B	37.96794	38.10953	43.31095	36.77236	32.8732	40.96103
17230055	Gm16340	interferon-activable protein 203-like	28.84301	31.89437	33.9931	38.80687	41.4773	40.25539
17256734	Ifi35	interferon-induced protein 35	65.15934	73.0276	67.71343	90.09468	78.2875	78.85826
17411147	Ifi44	interferon-induced protein 44	87.67903	52.32614	70.08044	85.9729	50.12332	83.49223
17411159	Ifi44l	interferon-induced protein 44 like	2.300185	2.655825	2.914079	2.509465	2.40189	1.978463
17358832	Ifit1	interferon-induced protein with tetratricopeptide repeats 1	36.97838	19.98825	26.98302	50.09032	32.19512	52.09453
17358815	Ifit2	interferon-induced protein with tetratricopeptide repeats 2	6.992896	5.760324	5.824806	7.589106	5.700269	5.900675
17358821	Ifit3	interferon-induced protein with tetratricopeptide repeats 3	14.67898	21.78691	13.96042	14.5017	17.01706	23.88942
17358825	Ifit3b	interferon-induced protein with tetratricopeptide repeats 3B	4.13581	4.17342	3.501177	3.960822	3.817425	5.621987
17280842	Ifrd1	interferon-related developmental regulator 1	31.60257	29.36509	35.22432	23.52985	30.22771	23.0867
17521454	Ifrd2	interferon-related developmental regulator 2	109.7031	112.503	100.4943	93.74371	75.31263	95.47294
17479317	Isg20	interferon-stimulated protein	26.39843	31.66594	41.30143	31.46116	28.427	18.31305
17391554	Il1a	interleukin 1 alpha	16.72425	16.82903	14.80333	17.16467	12.21041	13.86812
17391565	Il1b	interleukin 1 beta	9.404948	14.67959	16.6229	16.56608	15.60882	16.56608
17376159	Il1bos	interleukin 1 beta, opposite strand	4.563972	4.588333	3.598261	3.703322	5.318244	5.692135
17367676	Il1f10	interleukin 1 family, member 10	8.609563	8.817746	7.900369	9.51933	10.65294	8.914476
17367666	Il1f5	interleukin 1 family, member 5 (delta)	3.689742	4.093883	3.293542	2.904078	3.263862	3.353738
17367659	Il1f6	interleukin 1 family, member 6	4.031593	3.979204	4.452668	4.720894	4.16862	4.06321
17367644	Il1f8	interleukin 1 family, member 8	2.321006	2.650896	2.481049	2.657851	2.755779	2.942801
17367652	Il1f9	interleukin 1 family, member 9	3.272765	3.517426	3.50917	4.047048	4.414377	3.368088
17324522	Il1rap	interleukin 1 receptor accessory protein	68.63916	53.48565	48.40788	61.17668	57.28041	63.45623
17324542	Il1rap	interleukin 1 receptor accessory protein	4.349712	3.669262	3.379594	4.258632	4.910875	4.886969
17543087	Il1rapl1	interleukin 1 receptor accessory protein-like 1	2.636456	2.655954	2.99146	3.178773	2.483371	2.925615
17538004	Il1rapl2	interleukin 1 receptor accessory protein-like 2	2.458673	2.408542	2.727993	2.210879	2.493375	2.643721
17367686	Il1rn	interleukin 1 receptor antagonist	10.20893	19.33027	22.54976	16.4078	17.16526	18.68813
17212185	Il1r1	interleukin 1 receptor, type I	77.78284	88.82907	82.49473	107.7183	128.6119	90.61324
17212174	Il1r2	interleukin 1 receptor, type II	4.337368	3.799817	4.20927	4.20927	3.967114	4.136068
17212211	Il1rl1	interleukin 1 receptor-like 1	3.89356	3.156291	3.120398	3.411154	5.205656	3.623842
17212199	Il1rl2	interleukin 1 receptor-like 2	3.714582	3.189345	3.364099	4.078108	3.187731	3.320568
17216990	Il10	interleukin 10	2.979169	2.829094	3.08378	3.361488	3.207213	2.885457
17526492	Il10ra	interleukin 10 receptor, alpha	9.376243	10.54794	10.31785	11.41754	9.198573	7.901738
17327054	Il10rb	interleukin 10 receptor, beta	30.0764	34.20487	46.13085	34.99168	27.03038	28.96201
17245312	Iltifb	interleukin 10-related T cell-derived inducible factor beta	2.100671	1.799892	1.98912	1.860551	2.2349	2.115575
17485924	Il11	interleukin 11	2.960108	4.160336	3.672239	3.672239	3.986078	4.100387
17412913	Il11ra1	interleukin 11 receptor, alpha chain 1	11.35936	13.42667	13.25119	12.85333	12.81392	17.3508
17502051	Il12rb1	interleukin 12 receptor, beta 1	10.73225	10.7176	10.082	10.7176	10.40843	12.24975
17467323	Il12rb2	interleukin 12 receptor, beta 2	4.158202	3.891051	3.60198	3.747764	4.352909	2.97364
17398218	Il12a	interleukin 12a	4.136126	4.568308	4.596864	4.411288	3.899043	4.592692
17248654	Il12b	interleukin 12b	12.22693	13.66885	13.66885	11.99449	14.31296	16.00499
17262695	Il13	interleukin 13	5.821214	6.594942	7.484002	7.151967	6.917832	6.557337
17534051	Il13ra1	interleukin 13 receptor, alpha 1	136.8508	141.9658	140.7109	119.0299	213.1049	125.3615
17545181	Il13ra2	interleukin 13 receptor, alpha 2	4.273239	3.681771	3.282784	3.977936	3.507979	2.840451
17510856	Il15	interleukin 15	7.698994	7.698994	7.698994	5.634353	7.869162	7.657482
17367004	Il15ra	interleukin 15 receptor, alpha chain	26.36843	35.51755	35.78046	37.92954	34.27227	38.28082
17492905	Il16	interleukin 16	5.952229	6.508973	6.680796	7.226014	6.597548	6.482409
17462351	Il17ra	interleukin 17 receptor A	42.08663	41.9169	43.09444	47.34828	36.95916	43.09444
17304338	Il17rb	interleukin 17 receptor B	28.87142	28.4546	45.49922	24.61882	30.08912	29.96113
17461703	Il17rc	interleukin 17 receptor C	65.79427	73.71979	90.03537	76.33379	65.70585	74.5259
17298021	Il17rd	interleukin 17 receptor D	5.34063	5.808276	4.825539	5.952683	4.798089	5.602497
17461684	Il17re	interleukin 17 receptor E	6.39569	4.964437	6.350103	5.981967	5.097006	6.278674
17211369	Il17a	interleukin 17A	4.577024	4.276024	4.680978	2.956376	5.03475	3.874338
17351137	Il17b	interleukin 17B	3.377838	3.314198	3.401215	3.309538	3.393726	3.252885

17506392	Il17c	interleukin 17C	8.370246	10.03793	9.792722	7.848245	7.642634	9.442376
17300921	Il17d	interleukin 17D	9.18271	11.02816	10.20073	9.828287	9.814195	11.37512
17221627	Il17f	interleukin 17F	5.562371	5.526247	6.902792	5.72716	5.423334	4.870973
17517105	Il18	interleukin 18	30.09621	27.06901	38.31109	28.80505	31.95833	41.79504
17493995	Il18bp	interleukin 18 binding protein	41.68192	44.09429	65.7259	36.70793	52.67841	48.61131
17212229	Il18r1	interleukin 18 receptor 1	5.025545	3.905751	4.20927	4.199856	4.607678	4.20927
17212252	Il18rap	interleukin 18 receptor accessory protein	3.631285	3.885903	3.952869	3.781451	4.911147	3.88655
17226728	Il19	interleukin 19	7.069558	7.858097	10.00896	7.917363	6.016064	6.581151
17404880	Il2	interleukin 2	2.661003	2.235504	2.528129	2.791654	2.165995	2.28929
17366992	Il2ra	interleukin 2 receptor, alpha chain	4.39231	4.64581	4.39231	4.39231	4.904377	4.904377
17319009	Il2rb	interleukin 2 receptor, beta chain	7.517485	7.15633	6.848937	6.820985	5.514232	6.334628
17543572	Il2rg	interleukin 2 receptor, gamma chain	18.44605	19.34595	25.70559	17.29077	18.9385	17.14778
17226720	Il20	interleukin 20	4.681043	4.238695	3.948159	4.103799	4.732885	4.992526
17530115	Il20rb	interleukin 20 receptor beta	4.465216	4.20927	4.601391	4.20927	4.352065	3.366198
17231881	Il20ra	interleukin 20 receptor, alpha	4.303618	4.595526	4.250434	4.126389	5.152884	3.7081
17404888	Il21	interleukin 21	4.684386	4.412144	3.650262	4.56682	3.569569	4.886969
17482958	Il21r	interleukin 21 receptor	4.844138	5.03475	5.454549	6.665162	4.365873	5.883889
17237581	Il22	interleukin 22	2.086883	1.865006	1.714895	2.447875	2.188921	1.766691
17419890	Il22ra1	interleukin 22 receptor, alpha 1	23.30859	38.37806	39.97677	30.85388	24.83616	34.19041
17231869	Il22ra2	interleukin 22 receptor, alpha 2	2.825742	3.047233	2.982744	3.029207	3.56371	2.762664
17467345	Il23r	interleukin 23 receptor	3.331034	2.721345	2.452971	3.98992	2.942679	2.34156
17246091	Il23a	interleukin 23, alpha subunit p19	4.765805	4.536375	4.081134	4.359008	5.520428	4.137301
17226708	Il24	interleukin 24	5.30363	6.488612	5.946703	4.801649	5.751866	4.995226
17300376	Il25	interleukin 25	11.24467	14.07146	12.80531	12.60295	12.33059	12.21829
17496338	Il27	interleukin 27	14.8174	21.31578	18.58775	19.25472	18.69979	18.40021
17510994	Il27ra	interleukin 27 receptor, alpha	14.90526	14.84371	15.82452	16.96995	13.30171	13.81956
17262823	Il3	interleukin 3	3.871681	4.040517	3.319394	4.135495	3.362397	3.362397
17297211	Il3ra	interleukin 3 receptor, alpha chain	15.26674	17.60764	15.5655	11.51378	14.82079	18.42356
17452609	Il31	interleukin 31	4.357226	4.031928	4.119131	4.472433	5.425101	3.848457
17296203	Il31ra	interleukin 31 receptor A	5.601798	5.125917	5.331347	6.796396	5.601798	6.548071
17358598	Il33	interleukin 33	12.66223	11.46981	12.44936	11.11241	9.521969	8.956531
17512895	Il34	interleukin 34	6.30353	8.487796	7.814535	8.597159	6.565387	7.722564
17262683	Il4	interleukin 4	3.784073	3.563735	3.339703	3.106564	3.751766	3.3385
17482943	Il4ra	interleukin 4 receptor, alpha	45.04422	46.22592	59.26671	44.16097	46.07684	37.51875
17249586	Il5	interleukin 5	4.058706	4.131713	3.734823	3.852861	4.764022	5.022968
17469541	Il5ra	interleukin 5 receptor, alpha	3.437429	4.376143	4.986992	3.857564	4.469799	3.646494
17435725	Il6	interleukin 6	3.049748	2.005414	2.470923	2.335821	2.056384	3.081686
17407138	Il6ra	interleukin 6 receptor, alpha	86.45036	129.6421	136.094	140.9364	171.027	156.37
17289889	Il6st	interleukin 6 signal transducer	333.5733	312.3089	386.7239	297.1732	247.4066	223.4797
17403994	Il7	interleukin 7	3.79019	3.524943	3.556159	3.484085	3.348349	3.350833
17315891	Il7r	interleukin 7 receptor	3.244779	2.962058	2.696335	2.987813	3.68195	3.306512
17293013	Il9	interleukin 9	3.876487	2.996877	2.423832	2.801773	3.164352	3.656922
17261458	Il9r	interleukin 9 receptor	7.698994	7.698994	7.293392	7.998281	6.030928	8.140402
17399754	Ilf2	interleukin enhancer binding factor 2	39.34095	36.29452	41.44655	34.4163	35.56805	51.98091
17515170	Ilf3	interleukin enhancer binding factor 3	22.65158	27.66193	23.30181	24.51019	27.55286	27.99137
17542546	Irak1	interleukin-1 receptor-associated kinase 1	30.77059	41.86115	39.37178	35.29446	43.76999	40.7735
17519888	Irak1bp1	interleukin-1 receptor-associated kinase 1 binding protein 1	4.582326	4.339504	7.173613	5.222027	4.473395	6.124109
17461807	Irak2	interleukin-1 receptor-associated kinase 2	53.50011	48.19762	52.18235	48.90364	43.70056	44.45334
17245399	Irak3	interleukin-1 receptor-associated kinase 3	15.5791	15.5153	15.49327	11.13531	14.04028	9.853116
17314460	Irak4	interleukin-1 receptor-associated kinase 4	67.45203	61.98168	61.71803	53.39897	70.84087	51.94885
17463126	Iffo1	intermediate filament family orphan 1	15.31656	18.88318	18.15116	19.00794	17.26466	15.18589
17420761	Iffo2	intermediate filament family orphan 2	21.41901	25.33149	23.90168	25.33149	25.33149	30.50957
17472737	Ifltd1	intermediate filament tail domain containing 1	2.704928	3.263862	3.263862	3.558675	3.263862	3.414963
17360109	Ina	internexin neuronal intermediate filament protein, alpha	3.893183	4.192323	5.636541	5.2058	4.088127	5.412856
17529199	Impg1	interphotoreceptor matrix proteoglycan 1	5.144889	3.968077	3.289983	3.610378	3.703322	4.108979
17326295	Impg2	interphotoreceptor matrix proteoglycan 2	5.862679	6.142134	6.885732	6.472217	6.584619	8.323267
17327137	Itsn1	intersectin 1 (SH3 domain protein 1A)	25.83276	30.1008	30.96443	24.71012	26.73838	22.10958
17273751	Itsn2	intersectin 2	42.42962	50.91116	48.85282	43.42759	46.30641	45.54592
17519619	Ick	intestinal cell kinase	51.00193	36.41775	47.23944	43.75118	41.22764	40.39514
17502527	Isx	intestine specific homeobox	4.681043	5.073073	4.4778	5.032307	5.799345	5.050793
17461996	Ift122	intraflagellar transport 122	9.301672	7.377439	9.644457	11.85168	7.629402	7.176597
17334502	Ift140	intraflagellar transport 140	16.78395	13.82751	17.19742	17.49863	19.85623	18.89549
17446930	Ift172	intraflagellar transport 172	20.9039	19.75519	17.54624	22.89785	19.96222	16.85506
17253688	Ift20	intraflagellar transport 20	167.7094	170.0152	146.0228	154.5147	169.8044	161.1589
17443461	Ift22	intraflagellar transport 22	9.644524	13.39423	13.84152	12.76437	11.49066	13.01434
17318932	Ift27	intraflagellar transport 27	25.90844	23.78648	21.40194	26.01226	22.26244	15.43764
17277472	Ift43	intraflagellar transport 43	22.60829	21.52319	22.91626	26.97722	24.58864	19.81128
17516666	Ift46	intraflagellar transport 46	28.88563	26.11162	24.72965	27.19199	25.78553	29.61528
17379187	Ift52	intraflagellar transport 52	37.76741	33.63124	34.86071	41.62793	36.72168	37.91298
17326147	Ift57	intraflagellar transport 57	12.52935	12.56379	12.42927	12.62779	13.1045	14.32546
17415439	Ift74	intraflagellar transport 74	4.826141	5.685235	5.083281	5.722875	5.176977	5.647647
17405764	Ift80	intraflagellar transport 80	5.758488	5.722914	6.945892	6.212612	5.009095	6.275063
17452430	Ift81	intraflagellar transport 81	5.656501	7.271132	7.066961	6.27763	7.766421	6.943437
17300893	Ift88	intraflagellar transport 88	13.86831	11.52816	12.38584	11.01868	10.14383	14.73801
17307148	Ift88os	intraflagellar transport 88, opposite strand [Source:MGI Symbol;Acc:MGI:1919181]	6.96315	6.423769	7.792682	7.067255	6.867095	6.380679
17425855	Inip	INTS3 and NABP interacting protein	11.76346	10.24395	8.974179	7.255223	8.15113	8.356565
17397208	Intu	inturned planar cell polarity effector homolog (Drosophila)	7.846939	8.364446	8.084508	8.084508	7.846341	7.282178
17413978	Invs	inversin	17.27292	13.2047	13.34752	13.3805	17.89147	17.43205
17279324	Inf2	inverted formin, FH2 and WH2 domain containing	29.7746	30.02766	34.26016	31.86675	34.19468	37.21133
17407418	Ivl	involucrin	3.610378	3.883991	3.963651	3.32831	3.883991	5.243027
17231358	Iyd	iodotyrosine deiodinase	159.3406	160.4011	163.1709	148.1639	191.7682	187.3393
17325386	Iqcb1	IQ calmodulin-binding motif containing 1	9.622622	9.428185	10.18165	10.27966	9.511811	9.622622
17468933	Iqsec1	IQ motif and Sec7 domain 1	42.53209	49.18137	59.09442	45.74747	36.81878	43.33768
17538891	Iqsec2	IQ motif and Sec7 domain 2	12.63216	9.807055	11.34087	10.56368	7.852108	10.31413

17470515	Iqsec3	IQ motif and Sec7 domain 3	5.632088	7.027931	5.617429	6.585715	6.752117	6.229084
17465186	Iqub	IQ motif and ubiquitin domain containing	3.062691	2.67402	2.715673	2.67402	2.613404	2.736155
17430462	Iqcc	IQ motif containing C	12.02087	10.58897	11.21393	12.74978	12.55813	12.47926
17441692	Iqcd	IQ motif containing D	8.464472	10.45912	11.09302	10.78422	11.58551	11.24366
17454590	Iqce	IQ motif containing E	6.576728	11.37347	12.89706	10.56565	8.165946	11.61623
17521252	Iqcf1	IQ motif containing F1	4.490419	3.849844	4.490419	4.103657	3.629121	4.494685
17530719	Iqcf3	IQ motif containing F3	5.685038	6.702224	5.009546	5.761762	5.28063	7.491891
17530728	Iqcf4	IQ motif containing F4	4.444497	4.31156	4.946609	4.197906	4.074294	4.336226
17521256	Iqcf5	IQ motif containing F5	3.139468	3.661285	3.914668	3.419535	5.061553	3.840143
17521259	Iqcf6	IQ motif containing F6	6.171709	6.171709	5.394353	6.323617	6.644037	6.289564
17329906	Iqcg	IQ motif containing G	12.24635	9.15919	11.70838	12.89062	10.99884	13.55149
17492661	Iqgap1	IQ motif containing GTPase activating protein 1	27.2216	34.03459	46.50326	31.78204	29.2347	34.32672
17295136	Iqgap2	IQ motif containing GTPase activating protein 2	681.7836	696.8962	702.3956	669.1798	676.2299	700.0625
17398929	Iqgap3	IQ motif containing GTPase activating protein 3	4.910024	5.554742	5.545625	4.796127	5.09902	4.82166
17528084	Iqch	IQ motif containing H	5.338965	5.315885	5.120306	5.421455	4.875466	5.120306
17482218	Iqck	IQ motif containing K	10.28229	9.182646	7.134637	8.419007	9.276046	8.419007
17225391	Iqca	IQ motif containing with AAA domain	5.437864	5.396822	6.477917	7.458006	6.093495	6.347815
17517424	Ireb2	iron responsive element binding protein 2	102.38	102.0597	86.67175	96.39992	93.94927	106.5761
17488515	Papl	iron/zinc purple acid phospatase-like	3.926054	4.016949	3.750726	4.040545	3.852407	4.335385
17293273	Isca1	iron-sulfur cluster assembly 1 homolog (S. cerevisiae)	79.02734	88.46899	87.543	91.0748	103.0564	99.2235
17277294	Isca2	iron-sulfur cluster assembly 2 homolog (S. cerevisiae)	41.16054	48.52075	49.51281	50.64402	47.65944	48.15788
17503800	Irx3os	iroquois homeobox 3, opposite strand	7.095429	7.492307	9.770484	8.51685	9.786412	9.717532
17294215	Irx1	Iroquois related homeobox 1 (Drosophila)	11.77324	15.11564	12.73776	17.2362	13.58742	13.81564
17288442	Irx2	Iroquois related homeobox 2 (Drosophila)	10.4505	12.35592	12.86242	13.26442	8.662419	11.69768
17511623	Irx3	Iroquois related homeobox 3	8.480974	7.501141	6.892035	7.501141	7.068725	7.70679
17288454	Irx4	Iroquois related homeobox 4 (Drosophila)	6.664238	5.4656	5.451072	3.918414	5.803004	6.231632
17503810	Irx5	Iroquois related homeobox 5 (Drosophila)	8.297979	9.815352	8.288379	11.4234	11.43703	11.0861
17503816	Irx6	Iroquois related homeobox 6 (Drosophila)	5.798059	6.727638	8.20083	7.705775	6.479668	6.82666
17440757	Iscu	IscU iron-sulfur cluster scaffold homolog (E. coli)	78.27122	81.15792	65.2131	74.15387	74.42988	75.35389
17434023	Isg15	ISG15 ubiquitin-like modifier	28.98953	27.08984	30.93826	41.423	31.14309	39.63986
17296427	Isl1	ISL1 transcription factor, LIM/homeodomain	5.442879	6.288866	5.376324	5.690203	5.376324	5.376324
17464102	Iapp	islet amyloid polypeptide	2.569878	2.426236	2.621968	2.663162	2.663162	3.079658
17464803	Ica1	islet cell autoantigen 1	7.778921	6.660405	5.617429	6.471768	7.343766	6.91141
17223581	Ica1l	islet cell autoantigen 1-like	4.389176	5.03866	4.313802	4.790446	5.541744	4.858295
17274471	Iah1	isoamyl acetate-hydrolyzing esterase 1 homolog (S. cerevisiae)	138.5401	150.4176	146.1818	148.9165	165.6621	160.677
17350869	Isoc1	isochorismatase domain containing 1	171.9363	162.2877	134.4867	136.8603	146.4465	162.3541
17473402	Isoc2a	isochorismatase domain containing 2a	73.78369	66.03459	78.69445	73.79647	83.52001	92.78824
17485952	Isoc2b	isochorismatase domain containing 2b	6.208264	7.32526	7.583848	10.28579	6.79084	8.854311
17223863	Idh1	isocitrate dehydrogenase 1 (NADP+), soluble	701.9965	626.4085	559.068	562.0443	554.2335	562.3599
17492478	Idh2	isocitrate dehydrogenase 2 (NADP+), mitochondrial	280.2013	316.4887	349.3332	280.6484	221.4259	273.6902
17517390	Idh3a	isocitrate dehydrogenase 3 (NAD+) alpha	57.2955	55.82077	68.72771	75.86802	56.14673	69.676
17391608	Idh3b	isocitrate dehydrogenase 3 (NAD+) beta	493.8024	500.1025	433.1551	440.3357	377.9758	414.8857
17542392	Idh3g	isocitrate dehydrogenase 3 (NAD+), gamma	256.0266	278.9263	305.8732	289.2073	254.2546	306.7607
17287206	Iars	isoleucine-tRNA synthetase	162.7812	113.2063	108.3353	104.1161	107.5289	95.89675
17230780	Iars2	isoleucine-tRNA synthetase 2, mitochondrial	70.28817	72.16113	62.88482	65.35157	62.73332	64.45588
17285056	Idi1	isopentenyl-diphosphate delta isomerase	102.336	89.02138	95.40216	107.8947	103.4242	76.47202
17285067	Idi2	isopentenyl-diphosphate delta isomerase 2	5.162787	3.633702	4.488147	4.87959	5.323555	8.912005
17290388	LOC102642243	isopentenyl-diphosphate delta-isomerase 2-like	5.259698	9.349505	9.084203	6.256301	7.369006	6.769503
17275047	Ispd	isoprenoid synthase domain containing	4.47811	4.47811	4.990969	4.565396	4.296971	4.579119
17280790	Ispd	isoprenoid synthase domain containing	3.161151	3.081814	2.989968	3.081814	3.081814	3.339703
17422148	Icmt	isoprenylcysteine carboxyl methyltransferase	37.86755	36.37108	38.42251	36.73695	34.35933	36.29452
17374648	Ivd	isovaleryl coenzyme A dehydrogenase	135.4719	126.9371	113.6395	127.9157	100.3683	130.9117
17376908	Ism1	isthmin 1 homolog (zebrafish)	11.69768	13.70034	11.69768	11.28027	11.43084	12.91638
17282858	Ism2	isthmin 2 homolog (zebrafish)	10.2448	9.841171	9.375138	10.0348	11.23159	9.331571
17468678	Isy1	ISY1 splicing factor homolog (S. cerevisiae)	31.44001	34.49918	34.57986	31.33493	35.24777	31.38493
17378300	Itch	itchy, E3 ubiquitin protein ligase	79.55992	92.7233	89.15291	113.0635	108.0893	110.7567
17349223	Iws1	IWS1 homolog (S. cerevisiae)	48.2946	48.92127	48.2511	50.67	49.11222	48.56651
17477532	Izumo2	IZUMO family member 2	4.87256	3.553942	3.598137	4.39295	4.383855	5.239249
17427188	Izumo3	IZUMO family member 3	2.233258	2.677655	2.469382	3.514233	3.396644	2.693365
17235399	Izumo4	IZUMO family member 4	8.388774	8.911819	8.211239	6.723628	13.35418	12.28529
17477918	Izumo1	izumo sperm-egg fusion 1	4.326799	4.61471	4.768945	4.385192	4.858328	4.61471
17524092	Izumo1r	IZUMO1 receptor, JUNO	7.608542	9.660046	8.211752	8.467112	7.410855	8.467112
17397344	Jade1	jade family PHD finger 1	62.14257	55.1726	55.03395	43.91738	45.22692	63.08609
17262551	Jade2	jade family PHD finger 2	19.42873	25.89443	25.3946	26.24117	28.58963	31.1636
17533553	Jade3	jade family PHD finger 3	24.89148	26.23371	26.6383	20.37969	21.51394	27.90845
17392151	Jag1	jagged 1	13.61514	8.280857	11.27128	8.713969	9.126742	7.793006
17284253	Jag2	jagged 2	10.27011	10.70327	7.698994	7.789334	9.390357	11.01006
17461677	Jagn1	jagunal homolog 1 (Drosophila)	39.34041	35.71059	30.79235	34.92093	39.85918	25.36505
17427585	Jak1	Janus kinase 1	212.4133	214.7613	223.9123	204.1478	181.9288	171.7088
17358517	Jak2	Janus kinase 2	55.91022	49.92221	51.48499	54.18794	40.78594	60.81478
17502295	Jak3	Janus kinase 3	27.00678	32.58373	37.48444	28.66563	27.51411	25.33957
17436957	Jakmip1	janus kinase and microtubule interacting protein 1	9.930319	10.65885	10.73001	11.287	9.930319	10.90706
17354132	Jakmip2	janus kinase and microtubule interacting protein 2	4.004577	4.705213	4.740963	5.244554	4.428629	5.585707
17484309	Jakmip3	janus kinase and microtubule interacting protein 3	5.120306	4.879353	4.716217	5.241283	5.653091	5.218409
17466921	Jazf1	JAZF zinc finger 1	9.387689	10.47436	10.41665	9.768317	8.920163	11.31332
17317989	Jrk	jerky	5.050933	5.714115	7.111284	5.714115	5.747203	4.544117
17276182	Jkamp	JNK1/MAPK8-associated membrane protein	67.8224	67.10043	61.89453	67.43894	63.84094	65.14353
17319289	Josd1	Josephin domain containing 1	75.86644	68.62203	72.28678	75.72039	65.17244	69.85638
17477486	Josd2	Josephin domain containing 2	32.22593	38.96481	53.43637	39.65195	34.87352	47.23126
17536984	Jpx	Jpx transcript, Xist activator (non-protein coding)	4.597469	5.562602	7.445042	6.761953	6.849934	5.558324
17523990	Jrkl	Jrk-like	6.944352	8.645144	11.05671	12.44945	11.45448	12.22964
17233960	Jmjd1c	jumonji domain containing 1C	71.33014	73.64828	86.62671	73.03216	83.40373	72.2958
17250225	Jmjd4	jumonji domain containing 4	19.78411	23.21363	24.05458	22.75135	19.56224	16.56125
17272519	Jmjd6	jumonji domain containing 6	38.06228	41.55067	36.01385	35.67744	38.7789	33.6373

17374972	Jmjd7	jumonji domain containing 7	14.1825	15.33493	13.57011	17.74032	17.26011	13.98414
17334803	Jmjd8	jumonji domain containing 8	145.0061	125.5397	118.7711	118.0636	110.2687	117.5948
17286927	Jarid2	jumonji, AT rich interactive domain 2	20.72026	20.23513	19.49578	19.19834	18.01979	13.60646
17399663	Jtb	jumping translocation breakpoint	121.1508	111.7127	94.454	110.013	112.7317	108.7506
17511259	Junb	jun B proto-oncogene	39.56108	45.35847	49.45176	53.75697	57.58376	58.32652
17502001	Jund	jun D proto-oncogene	105.6091	106.4565	106.2309	99.36735	114.4719	97.80796
17277396	Jdp2	Jun dimerization protein 2	12.94103	12.27576	13.04993	11.90561	12.7519	10.5159
17427312	Jun	jun proto-oncogene	127.3813	125.4553	71.32718	42.79677	43.36593	34.34623
17415502	Junos	jun proto-oncogene, opposite strand	9.115362	9.460458	8.365084	8.712399	6.907674	9.688276
17326801	Jam2	junction adhesion molecule 2	49.28955	34.53171	44.32442	43.66635	24.4664	22.81483
17525186	Jam3	junction adhesion molecule 3	3.18294	3.778857	3.382641	3.533162	3.09335	3.31537
17269464	Jup	junction plakoglobin	73.4683	66.34381	91.85141	73.1568	72.01073	65.48851
17243102	Jsrp1	junctional sarcoplasmic reticulum protein 1	21.85452	19.17228	15.11449	18.45411	16.98571	13.12531
17294991	Jmy	junction-mediating and regulatory protein	68.39268	68.28326	61.43505	71.92992	62.47556	83.42975
17221502	Jph1	junctophilin 1	8.082379	8.751498	8.178918	8.558393	7.866381	8.433433
17394015	Jph2	junctophilin 2	4.379967	3.735859	4.454952	4.519083	3.98887	4.129136
17506313	Jph3	junctophilin 3	3.653122	5.171239	5.469959	4.364602	5.408918	4.092493
17306666	Jph4	junctophilin 4	14.02521	16.89097	14.31296	12.42669	16.47858	15.73047
17269615	Kat2a	K(lysine) acetyltransferase 2A	33.2481	33.38435	33.2481	34.18686	34.48411	31.03276
17338571	Kat2b	K(lysine) acetyltransferase 2B	113.7932	99.75588	80.31124	101.2045	110.9688	109.7517
17361663	Kat5	K(lysine) acetyltransferase 5	26.22753	27.91522	29.47316	32.70026	30.5394	28.29745
17499983	Kat6a	K(lysine) acetyltransferase 6A	31.49673	34.96113	34.29889	34.29889	34.29889	34.29889
17297595	Kat6b	K(lysine) acetyltransferase 6B	26.56325	27.92877	23.62791	24.40729	26.42222	21.1615
17268180	Kat7	K(lysine) acetyltransferase 7	44.69495	44.56441	41.60053	43.46734	42.82377	43.00761
17483532	Kat8	K(lysine) acetyltransferase 8	111.0226	71.11638	59.09442	73.52485	86.92745	72.77142
17394102	Kcns1	K+ voltage-gated channel, subfamily S, 1	4.01461	3.844751	3.844751	4.567073	4.854491	4.330849
17310977	Kcns2	K+ voltage-gated channel, subfamily S, 2	14.74987	14.50029	10.93742	15.83396	15.18705	20.16624
17329946	Kalrn	kalirin, RhoGEF kinase	10.83983	11.80929	13.11104	11.89984	12.43927	12.43927
17477391	Klk1	kallikrein 1	2.999288	1.972465	1.893611	2.450897	3.493807	2.496886
17477377	Klk1b4	kallikrein 1-related pepidase b4	13.65446	5.306277	6.870809	11.50086	5.120306	7.698994
17477299	Klk1b1	kallikrein 1-related peptidase b1	2.146729	2.587197	3.009972	2.933474	3.83828	2.317421
17477314	Klk1b11	kallikrein 1-related peptidase b11	5.912589	9.800939	13.18074	22.73873	11.444	14.37958
17477354	Klk1b16	kallikrein 1-related peptidase b16	2.632384	3.609978	2.963126	3.469842	5.252485	3.7169
17477339	Klk1b21	kallikrein 1-related peptidase b21	4.857722	3.185081	2.743828	3.227305	2.816239	3.088187
17477347	Klk1b22	kallikrein 1-related peptidase b22	4.322482	4.334243	5.020984	3.283695	4.681367	3.810948
17477362	Klk1b24	kallikrein 1-related peptidase b24	6.617379	9.457049	17.69684	8.694602	19.62866	9.98014
17477331	Klk1b27	kallikrein 1-related peptidase b27	3.506496	2.722326	2.687733	2.842795	2.31988	2.421062
17477369	Klk1b3	kallikrein 1-related peptidase b3	4.505509	4.315806	4.295631	4.445298	3.940258	3.480996
17477384	Klk1b5	kallikrein 1-related peptidase b5	5.696793	6.881199	6.992896	7.727973	5.813714	6.992896
17477288	Klk1b7-ps	kallikrein 1-related peptidase b7, pseudogene	4.670089	6.12963	4.801649	4.801649	4.390971	4.277536
17477292	Klk1b8	kallikrein 1-related peptidase b8	2.995631	2.149781	2.419686	2.258132	3.274194	2.789661
17477306	Klk1b9	kallikrein 1-related peptidase b9	2.562496	2.872785	2.91212	3.067046	3.206368	2.820146
17477322	Klk1b26	kallikrein 1-related petidase b26	7.987256	8.480386	7.057856	7.431019	6.297067	7.579959
17509052	Klkb1	kallikrein B, plasma 1	435.0116	336.8868	242.2999	347.3726	410.7225	407.4756
17477220	Klk10	kallikrein related-peptidase 10	5.650662	7.500101	7.418976	6.164697	6.970151	6.347375
17477209	Klk11	kallikrein related-peptidase 11	3.341463	3.757909	3.524039	3.381258	2.959739	3.381258
17477199	Klk12	kallikrein related-peptidase 12	9.52065	8.601093	10.1025	13.59458	11.31622	10.49333
17477191	Klk13	kallikrein related-peptidase 13	7.486596	5.669769	5.669769	5.364337	5.669769	5.27156
17477184	Klk14	kallikrein related-peptidase 14	5.443256	4.153824	4.193282	5.25824	5.29342	4.556544
17477282	Klk15	kallikrein related-peptidase 15	3.403337	4.245134	4.20927	4.10841	3.390528	4.20927
17477274	Klk4	kallikrein related-peptidase 4 (prostase, enamel matrix, prostate)	11.50086	11.66828	11.85957	11.26683	8.689541	11.86771
17477266	Klk5	kallikrein related-peptidase 5	5.48933	6.196442	4.153507	6.100807	9.531346	6.100807
17477254	Klk6	kallikrein related-peptidase 6	5.52414	5.52414	5.52414	5.52414	5.294153	4.946198
17477245	Klk7	kallikrein related-peptidase 7 (chymotryptic, stratum corneum)	9.488237	9.623022	9.903442	10.0776	9.809231	9.918005
17477237	Klk8	kallikrein related-peptidase 8	8.559639	7.892597	7.060351	6.685614	7.183167	6.497073
17477229	Klk9	kallikrein related-peptidase 9	7.453355	6.267282	6.171709	6.171709	7.379689	5.862638
17541705	Kis2	Kaplan integration site 2	3.004969	3.339703	3.339703	3.255886	3.070748	3.339703
17474052	Kptn	kaptin	16.69738	12.39443	16.85553	17.28442	16.19021	14.54942
17325291	Kpna1	karyopherin (importin) alpha 1	52.41326	46.16348	40.37079	50.39539	41.92474	36.95839
17271033	Kpna2	karyopherin (importin) alpha 2	20.0799	22.88944	24.73514	23.49986	22.34407	19.40854
17307472	Kpna3	karyopherin (importin) alpha 3	153.6592	119.6976	127.3239	127.3239	117.7155	121.547
17405795	Kpna4	karyopherin (importin) alpha 4	70.39788	58.12069	51.52462	59.98667	53.5335	56.72725
17430503	Kpna6	karyopherin (importin) alpha 6	53.8939	48.69832	50.92704	60.84513	47.42185	56.76776
17268469	Kpnb1	karyopherin (importin) beta 1	144.4544	137.2203	147.5602	140.405	129.6457	142.4656
17547634	Kpnb1	karyopherin (importin) beta 1	193.0245	179.7503	196.3794	186.2272	174.2385	193.9391
17455028	Kpna7	karyopherin alpha 7 (importin alpha 8)	3.919175	4.20927	4.177761	4.560747	4.637316	3.625173
17270621	Kansl1	KAT8 regulatory NSL complex subunit 1	43.54575	40.60344	45.02767	50.0747	41.538	38.54522
17223880	Kansl1l	KAT8 regulatory NSL complex subunit 1-like	17.54892	19.96208	16.44948	16.17675	19.51593	20.03596
17321227	Kansl2	KAT8 regulatory NSL complex subunit 2	30.59279	49.65889	52.98309	35.70217	52.022	40.91506
17479162	Kansl2-ps	KAT8 regulatory NSL complex subunit 2, pseudogene	27.35381	42.35675	31.82768	29.94888	37.0343	31.65672
17222156	Kansl3	KAT8 regulatory NSL complex subunit 3	56.44993	66.14224	69.02992	52.54494	58.36696	57.71322
17231558	Katna1	katanin p60 (ATPase-containing) subunit A1	36.08932	36.42078	40.40411	37.63101	35.89448	39.21055
17507485	Gm6524	katanin p60 (ATPase-containing) subunit A1 pseudogene	3.062861	2.810253	2.560295	2.510683	2.615705	3.498848
17455431	Katnal1	katanin p60 subunit A-like 1	13.524	12.21879	15.43678	13.524	13.524	12.84398
17355483	Katnal2	katanin p60 subunit A-like 2	4.811511	4.307289	4.394929	4.665592	4.582993	4.941949
17504242	Katnb1	katanin p80 (WD40-containing) subunit B 1	16.20088	18.18126	15.53855	15.39457	15.65357	15.07724
17374283	Katnbl1	katanin p80 subunit B like 1	12.87633	9.128324	8.33106	9.178128	6.581425	7.613343
17359816	Kazald1	Kazal-type serine peptidase inhibitor domain 1	7.782696	6.632211	6.355299	6.831015	7.047981	7.049202
17432397	Kazn	kazrin, periplakin interacting protein	6.699205	6.665578	7.561277	6.8431	5.554126	6.699205
17498239	Kcnq1ot1	KCNQ1 overlapping transcript 1	15.82145	19.0894	16.80408	13.94398	12.64224	11.86138
17222694	Kdelc1	KDEL (Lys-Asp-Glu-Leu) containing 1	14.87275	11.8415	12.38953	13.6698	13.59798	15.54501
17517300	Kdelc2	KDEL (Lys-Asp-Glu-Leu) containing 2	11.57877	8.079522	10.93825	10.81004	7.653979	10.29855
17478032	Kdelr1	KDEL (Lys-Asp-Glu-Leu) endoplasmic reticulum protein retention receptor 1	33.01478	33.62145	33.99593	38.15447	39.97566	31.25229
17444338	Kdelr2	KDEL (Lys-Asp-Glu-Leu) endoplasmic reticulum protein retention receptor 2	230.653	159.2335	168.6315	140.9295	180.4518	150.0355

17313000	Kdelr3	KDEL (Lys-Asp-Glu-Leu) endoplasmic reticulum protein retention receptor 3	5.885276	6.592611	6.287864	6.594485	5.799868	6.287864
17264651	Kdm6b	KDM1 lysine (K)-specific demethylase 6B	41.01643	65.10967	58.08002	54.18344	38.50543	50.54582
17251653	Kdm6bos	KDM1 lysine (K)-specific demethylase 6B, opposite strand [Source:MGI Symbol;Acc	8.058381	5.52326	6.029967	4.369627	5.554319	6.503336
17349474	Kdm3b	KDM3B lysine (K)-specific demethylase 3B	75.66845	79.06076	78.69445	70.74518	78.69445	78.69445
17545430	Kantr	Kdm5c adjacent non-coding transcript	3.623264	3.227663	3.599309	3.930928	3.391116	3.89062
17275890	Klhdc1	kelch domain containing 1	3.643967	3.322364	3.735755	3.735833	3.322364	3.829378
17456841	Klhdc10	kelch domain containing 10	63.66109	63.3736	60.96249	62.76116	48.71148	55.5733
17275909	Klhdc2	kelch domain containing 2	96.01714	98.94535	86.64472	98.86925	98.94535	98.51353
17345527	Klhdc3	kelch domain containing 3	131.1433	140.5685	159.8672	138.0292	114.5648	113.0024
17513625	Klhdc4	kelch domain containing 4	15.77152	14.38228	17.16526	17.38523	16.54427	17.16526
17431933	Klhdc7a	kelch domain containing 7A	10.56368	10.96345	12.89035	12.2579	12.65679	13.63195
17314157	Klhdc7b	kelch domain containing 7B	12.53144	11.22964	14.46496	15.18074	9.988306	12.81099
17217174	Klhdc8a	kelch domain containing 8A	8.208849	8.556436	8.615652	9.534584	13.37095	8.494035
17531193	Klhdc8b	kelch domain containing 8B	11.61542	12.58986	12.69053	13.12568	10.85133	14.98129
17229742	Klhdc9	kelch domain containing 9	8.077618	9.438189	9.427792	10.38522	8.963362	10.09697
17499480	Kbtbd11	kelch repeat and BTB (POZ) domain containing 11	9.895688	9.50746	8.987999	10.17178	9.987752	12.53743
17468747	Kbtbd12	kelch repeat and BTB (POZ) domain containing 12	4.01461	3.987792	3.455298	5.217866	6.366454	5.148849
17528247	Kbtbd13	kelch repeat and BTB (POZ) domain containing 13	5.275874	4.836287	4.697587	5.17192	4.868475	4.402735
17467115	Kbtbd2	kelch repeat and BTB (POZ) domain containing 2	19.42873	18.16815	18.01392	18.05504	18.01392	17.38812
17514410	Kbtbd3	kelch repeat and BTB (POZ) domain containing 3	3.787485	4.20927	3.787485	3.979646	4.352065	3.371873
17373110	Kbtbd4	kelch repeat and BTB (POZ) domain containing 4	15.90612	15.43764	15.09417	16.18258	14.31296	15.5858
17302234	Kbtbd6	kelch repeat and BTB (POZ) domain containing 6	3.51416	3.412171	3.773622	3.722986	2.691126	3.898286
17302226	Kbtbd7	kelch repeat and BTB (POZ) domain containing 7	46.78324	31.84202	39.8589	39.62777	34.12791	38.24131
17461103	Kbtbd8	kelch repeat and BTB (POZ) domain containing 8	4.822362	4.769969	4.949696	5.022028	5.031575	4.826509
17309024	Klhl1	kelch-like 1	4.12333	5.965405	3.795211	4.386773	4.078476	4.626977
17256378	Klhl10	kelch-like 10	4.426848	4.814013	4.418633	5.368094	5.642013	5.289898
17269516	Klhl11	kelch-like 11	13.73933	13.8586	17.58922	17.65604	19.32478	16.5771
17217469	Klhl12	kelch-like 12	15.528	14.95038	12.73573	16.05087	13.55159	14.33858
17540589	Klhl13	kelch-like 13	7.461729	7.294353	6.374755	6.946903	6.330634	7.092627
17353042	Klhl14	kelch-like 14	4.135839	4.763493	3.963321	3.962992	4.838299	4.502574
17536295	Klhl15	kelch-like 15	15.18737	12.92901	11.55592	12.22549	11.84774	14.78457
17434046	Klhl17	kelch-like 17	17.92175	19.08477	19.49537	19.36675	16.12257	19.42496
17531563	Klhl18	kelch-like 18	16.33314	16.13409	15.60828	17.53104	14.71535	15.5992
17509642	Klhl2	kelch-like 2, Mayven	52.10789	49.0469	55.87651	55.74614	57.12142	61.39163
17228825	Klhl20	kelch-like 20	33.11631	30.76419	30.22771	29.2566	30.22771	28.27078
17422039	Klhl21	kelch-like 21	47.19853	50.35419	48.63962	49.55814	49.30049	60.96672
17323589	Klhl22	kelch-like 22	10.23401	18.11484	14.49175	20.28357	18.90977	16.29808
17371495	Klhl23	kelch-like 23	4.409393	5.338965	5.338965	5.275069	5.628693	5.896791
17323838	Klhl24	kelch-like 24	139.8096	186.2207	167.3413	182.0499	165.731	209.9672
17479254	Klhl25	kelch-like 25	18.97174	17.14053	17.23776	17.12414	17.23776	16.70062
17510057	Klhl26	kelch-like 26	20.22363	17.17538	19.31005	15.11113	17.08076	16.30237
17281412	Klhl28	kelch-like 28	14.15696	15.20485	13.73362	13.39423	11.12929	12.92157
17279893	Klhl29	kelch-like 29	6.535964	8.815974	8.405887	7.453355	8.143675	8.007045
17293059	Klhl3	kelch-like 3	15.70814	19.14638	19.92793	15.14353	18.49649	22.4176
17215750	Klhl30	kelch-like 30	4.570906	6.049433	4.826141	5.733317	5.749036	6.092904
17519573	Klhl31	kelch-like 31	7.12031	7.474878	7.829825	6.549751	7.066912	5.765757
17423640	Klhl32	kelch-like 32	3.787485	3.808466	3.738786	3.787485	3.733866	3.787485
17306045	Klhl33	kelch-like 33	4.454335	4.051679	3.48667	4.455941	3.838387	4.068283
17539121	Klhl34	kelch-like 34	8.060727	9.246517	10.10068	10.17206	9.520716	8.807606
17480555	Klhl35	kelch-like 35	4.667273	5.17669	5.363371	4.922086	3.78444	6.347815
17506177	Klhl36	kelch-like 36	12.51868	14.41081	22.6665	16.72321	16.20312	14.37958
17317278	Klhl38	kelch-like 38	4.267526	4.006521	3.50956	4.513135	3.688489	3.664738
17537306	Klhl4	kelch-like 4	4.01461	3.889272	4.687699	4.218441	4.243133	4.281748
17523359	Klhl40	kelch-like 40	5.979356	6.131712	5.460943	5.795246	6.030928	5.82933
17371451	Klhl41	kelch-like 41	6.100933	5.496373	5.965198	6.100933	6.564886	7.387263
17464417	Klhl42	kelch-like 42	39.06623	33.49746	30.12481	36.16369	37.73026	35.50229
17437704	Klhl5	kelch-like 5	24.86906	22.77722	22.25673	29.18813	20.38053	24.92774
17329063	Klhl6	kelch-like 6	10.49369	9.362994	12.19189	10.41665	10.2773	8.179768
17435251	Klhl7	kelch-like 7	28.37778	25.48948	30.68752	28.13356	22.81673	30.73264
17450338	Klhl8	kelch-like 8	5.179777	6.242267	5.428863	7.748355	6.717386	5.854719
17427108	Klhl9	kelch-like 9	61.6945	59.47164	56.01417	59.30287	70.93865	69.27534
17524659	Keap1	kelch-like ECH-associated protein 1	60.56112	53.56096	61.73728	57.43825	64.86508	57.05811
17466360	Kel	Kell blood group	2.778007	3.264699	3.009972	3.125615	2.544143	3.452353
17544446	Xkrx	Kell blood group complex subunit-related, X-linked	3.580051	3.532917	3.753665	3.32808	3.826724	3.674098
17533207	Xk	Kell blood group precursor (McLeod phenotype) homolog	23.79093	16.70954	14.16079	14.80066	17.29412	11.00594
17322039	Krt1	keratin 1	3.569198	4.325269	4.342994	4.589573	3.185964	4.216716
17269064	Krt10	keratin 10	8.70932	11.94156	9.685255	10.7929	11.96004	12.73229
17269074	Krt12	keratin 12	6.347815	5.017504	6.992896	7.259851	6.347815	5.566382
17269347	Krt13	keratin 13	6.222395	5.928224	7.59658	7.336948	5.966893	9.214462
17269391	Krt14	keratin 14	5.092909	3.711237	4.697815	4.886055	6.22576	4.996196
17269358	Krt15	keratin 15	4.634552	4.932879	4.074802	3.954677	5.026485	4.458659
17269404	Krt16	keratin 16	4.368809	4.933974	4.557397	4.241869	3.810129	4.20927
17269415	Krt17	keratin 17	6.648459	4.900402	7.526765	5.185884	4.982742	6.367601
17315245	Krt18	keratin 18	348.7308	311.8871	324.7487	307.9986	321.7553	300.5039
17269368	Krt19	keratin 19	5.15467	5.647373	5.44386	5.458596	5.153741	5.896423
17322026	Krt2	keratin 2	6.171709	5.669258	6.655237	6.072035	5.147386	5.129364
17269085	Krt20	keratin 20	5.618286	5.300432	5.633221	6.366233	5.603818	6.191247
17268995	Krt222	keratin 222	4.789848	3.339703	3.341625	3.87356	4.352065	3.919936
17269096	Krt23	keratin 23	25.80091	31.01254	42.19207	48.02588	9.17304	64.599
17269006	Krt24	keratin 24	4.480159	5.29944	5.468215	4.698955	6.034691	4.551241
17269018	Krt25	keratin 25	3.036228	3.973334	3.703322	3.420933	4.015668	3.538848
17269030	Krt26	keratin 26	4.652093	3.067918	2.980863	3.067918	3.263862	3.633299
17269042	Krt27	keratin 27	4.187096	4.33803	3.941296	4.23702	7.099808	4.243104
17269052	Krt28	keratin 28	4.761546	5.206703	4.743296	6.996532	5.338632	5.610114

17269301	Krt31	keratin 31	4.285668	4.86291	3.813326	2.992394	4.576231	4.106644
17269311	Krt32	keratin 32	7.684546	9.177492	8.047385	6.761344	8.048166	14.53078
17269270	Krt33a	keratin 33A	8.044373	8.220066	7.07397	7.5552	7.333643	7.978735
17269281	Krt33b	keratin 33B	5.097147	6.992896	6.386608	7.139386	7.280866	9.221299
17269291	Krt34	keratin 34	5.291732	4.8868	6.858533	5.938382	6.444284	5.981967
17269322	Krt35	keratin 35	5.479826	5.337522	5.877123	5.208796	5.15971	5.678186
17269333	Krt36	keratin 36	3.129994	3.263862	4.872864	3.440313	7.962933	4.872864
17269343	Krt36	keratin 36	8.196852	10.07096	9.061564	9.061564	8.00588	9.310251
17269108	Krt39	keratin 39	5.211613	5.486857	4.643717	6.350059	5.236925	5.341297
17322075	Krt4	keratin 4	3.708922	4.074294	4.05387	4.17449	3.889218	3.426772
17269118	Krt40	keratin 40	5.814923	10.22472	6.724794	7.878822	5.896178	8.129463
17269424	Krt42	keratin 42	13.44474	13.44474	12.53708	12.57346	13.30863	15.00499
17321966	Krt5	keratin 5	9.226542	10.65671	8.473453	8.573356	9.308121	8.981522
17321951	Krt6a	keratin 6A	3.462322	4.702148	3.703322	4.068621	5.635056	2.886758
17321939	Krt6b	keratin 6B	4.609819	3.010932	3.339703	3.207546	4.151003	2.803793
17315200	Krt7	keratin 7	13.55469	16.22211	12.1535	13.71032	20.89926	14.89432
17321978	Krt71	keratin 71	5.989435	5.300432	5.124851	5.641231	5.529695	4.790845
17322002	Krt72	keratin 72	3.587478	3.798237	3.6199	3.799685	3.413945	4.144879
17322014	Krt73	keratin 73	9.399148	10.02771	10.41644	8.89935	7.805765	10.09284
17321989	Krt74	keratin 74	2.316859	3.658241	3.809601	4.514512	2.87161	6.185242
17321907	Krt75	keratin 75	3.69991	4.663167	3.744103	4.376538	4.56796	4.607838
17322063	Krt76	keratin 76	3.249392	3.04905	4.385101	3.256946	4.223122	3.877589
17322051	Krt77	keratin 77	6.470916	7.698994	7.488413	8.226735	6.161835	7.393872
17322099	Krt78	keratin 78	12.12918	16.44161	15.94818	16.62301	12.20694	13.39423
17322087	Krt79	keratin 79	11.58503	11.33844	11.57371	14.49607	10.19366	11.02938
17322113	Krt8	keratin 8	220.9001	250.8185	252.2954	220.8817	231.2116	229.2456
17321823	Krt80	keratin 80	8.835612	7.104386	6.760266	7.458265	7.086533	6.394493
17321849	Krt81	keratin 81	21.90077	23.22119	18.95452	20.48676	21.1615	16.13722
17321883	Krt82	keratin 82	11.28481	13.02598	10.7716	12.25297	10.60358	13.90103
17321838	Krt83	keratin 83	2.429316	2.994469	2.335676	2.740464	3.549978	3.339703
17321871	Krt84	keratin 84	5.08078	4.999903	5.09531	5.824523	5.004688	4.771325
17315225	Krt86	keratin 86	6.860673	14.3203	11.25216	15.01622	9.659175	11.51362
17269381	Krt9	keratin 9	8.277585	5.741668	5.793921	6.29314	5.741668	5.608869
17234736	Krtap10-10	keratin associated protein 10-10	3.915157	2.725536	2.729904	2.940497	3.504236	2.953325
17242328	Krtap10-4	keratin associated protein 10-4	6.171709	7.082555	6.171709	6.797527	6.171709	6.171709
17331914	Krtap11-1	keratin associated protein 11-1	2.922109	3.263862	2.722364	2.70148	2.690417	2.922109
17234713	Krtap12-1	keratin associated protein 12-1	2.810545	2.96843	2.420978	2.643886	2.9702	3.350183
17234707	Gm10272	keratin associated protein 12-4	4.943079	13.29858	11.30665	9.45895	6.886973	7.330898
17331852	Krtap13	keratin associated protein 13	2.111298	2.353846	2.439491	2.304206	2.44191	2.287592
17269146	Krtap1-3	keratin associated protein 1-3	2.956602	3.34958	2.561751	2.398795	3.079209	2.956602
17326914	Krtap13-1	keratin associated protein 13-1	4.099335	3.430907	3.892886	3.958709	3.36277	3.675702
17331861	Krtap14	keratin associated protein 14	2.933453	2.663568	2.55845	2.431337	2.810311	2.802258
17269143	Krtap1-4	keratin associated protein 1-4	3.729055	3.710054	2.782169	3.233059	3.807357	2.953407
17326922	Krtap15	keratin associated protein 15	4.090309	3.644523	3.94545	3.644523	3.644523	3.806144
17269139	Krtap1-5	keratin associated protein 1-5	4.611672	2.807741	2.868845	2.888599	2.75219	2.294803
17269263	Krtap16-1	keratin associated protein 16-1	4.01461	4.513855	4.349863	4.01461	4.076525	4.01461
17331886	Krtap16-3	keratin associated protein 16-3	2.285215	2.20239	2.105116	2.4325	2.214974	2.33021
17269266	Krtap17-1	keratin associated protein 17-1	25.88672	19.5773	24.11836	17.41115	16.45085	19.99005
17331863	Krtap19-1	keratin associated protein 19-1	3.244104	2.314611	2.406006	3.230662	2.406006	2.532812
17331866	Krtap19-2	keratin associated protein 19-2	2.620187	2.309611	3.146853	2.336631	2.509083	2.14664
17331869	Krtap19-3	keratin associated protein 19-3	2.483543	1.861157	2.32152	3.107749	2.197571	2.596359
17331873	Krtap19-4	keratin associated protein 19-4	31.37536	33.31592	37.02788	37.97373	38.65411	32.794
17331876	Krtap19-5	keratin associated protein 19-5	3.294752	2.592026	2.910183	2.635488	2.448367	3.126005
17331880	Krtap19-9a	keratin associated protein 19-9A [Source:MGI Symbol;Acc:MGI:3704466]	2.610941	2.34925	2.075933	2.673556	2.072655	2.606909
17331882	Krtap19-9b	keratin associated protein 19-9B	3.207013	2.369118	2.446535	2.210174	2.800472	2.613929
17326950	Krtap20-2	keratin associated protein 20-2	3.177694	3.476462	2.67402	2.510875	3.019877	2.760788
17331900	Krtap21-1	keratin associated protein 21-1	1.989533	2.659804	2.67402	2.606909	2.659804	2.434862
17331888	Krtap22-2	keratin associated protein 22-2	3.148512	2.463142	2.48239	2.953407	2.906595	2.855137
17269158	Krtap2-4	keratin associated protein 2-4	3.477258	2.096816	2.253442	2.880322	2.274391	2.307627
17331832	Krtap24-1	keratin associated protein 24-1	3.492863	4.86254	4.509977	4.190057	5.058431	4.509977
17331842	Krtap26-1	keratin associated protein 26-1	3.176328	3.863719	3.766332	3.39735	2.988538	3.448502
17331846	Krtap27-1	keratin associated protein 27-1	3.787485	3.787485	3.881677	2.576388	4.975874	4.065351
17548922	Krtap28-13	keratin associated protein 28-13	2.018369	2.237566	2.14545	2.134562	1.965069	2.43304
17269261	Krtap29-1	keratin associated protein 29-1 [Source:MGI Symbol;Acc:MGI:3652056]	6.303137	6.287864	5.246626	6.287864	7.69526	6.745662
17269136	Krtap3-1	keratin associated protein 3-1	3.794422	4.312845	3.765601	3.382078	3.660397	4.831195
17256323	Krtap31-1	keratin associated protein 31-1	6.171709	5.780603	4.821025	4.798821	4.522749	4.681043
17256332	Krtap31-2	keratin associated protein 31-2	3.919854	2.578604	2.949969	2.509604	3.177716	2.850312
17269130	Krtap3-2	keratin associated protein 3-2	2.762089	2.178251	2.727653	2.325934	3.04356	2.396751
17269126	Krtap3-3	keratin associated protein 3-3	3.023333	3.460715	3.339703	3.188527	3.308896	2.851755
17269164	Krtap4-1	keratin associated protein 4-1	4.239282	2.605319	2.93384	2.858285	3.150214	4.350074
17269246	Krtap4-13	keratin associated protein 4-13	21.58115	35.36107	34.36124	31.25966	30.87442	28.56111
17269256	Krtap4-16	keratin associated protein 4-16	7.264281	7.507122	6.074897	7.426384	7.748086	7.507122
17269167	Krtap4-2	keratin associated protein 4-2	2.051188	2.132565	2.150094	2.929897	2.197617	2.002178
17269184	Krtap4-6	keratin associated protein 4-6	3.379782	3.274671	2.560277	2.734581	2.844293	2.774754
17269171	Krtap4-7	keratin associated protein 4-7	2.435892	2.443383	2.459935	2.354564	2.454961	3.316979
17269229	Krtap4-8	keratin associated protein 4-8	3.141732	2.640443	2.236232	2.660173	2.425076	2.22436
17256292	Krtap4-9	keratin associated protein 4-9	23.89456	23.0939	18.13909	16.92906	20.76152	23.87734
17498084	Krtap5-1	keratin associated protein 5-1	12.20948	12.10088	10.15072	12.83081	5.637635	9.190797
17498059	Krtap5-2	keratin associated protein 5-2	4.346126	3.97237	4.989655	3.066855	3.981826	3.565242
17485187	Krtap5-3	keratin associated protein 5-3	4.499953	5.126769	3.899043	5.58203	6.132052	8.014597
17485194	Krtap5-4	keratin associated protein 5-4	25.96111	31.92822	24.67041	25.96111	35.45974	20.48676
17498072	Krtap5-5	keratin associated protein 5-5	6.319849	5.468329	6.784394	5.47994	6.748281	6.610869
17326930	Krtap6-1	keratin associated protein 6-1	3.808756	2.281211	2.780029	2.608698	2.396087	2.962017
17331903	Krtap6-2	keratin associated protein 6-2	3.190605	3.627134	2.327224	2.945556	3.561043	4.748165

17326933	Krtap6-3	keratin associated protein 6-3	2.243747	2.600556	2.137212	2.507622	2.287022	2.406006
17331894	Krtap6-5	keratin associated protein 6-5	1.824056	2.037514	1.874947	2.648912	1.894451	2.276378
17331910	Krtap7-1	keratin associated protein 7-1	2.908368	3.073516	2.782767	2.782767	2.782767	2.457021
17331907	Krtap8-1	keratin associated protein 8-1	2.56735	2.303919	2.529391	2.88144	2.406656	3.093264
17256314	Krtap9-1	keratin associated protein 9-1	24.29268	22.36127	23.89522	24.57927	16.70745	18.95912
17269149	Krtap9-3	keratin associated protein 9-3	3.39615	2.988932	2.898507	4.10653	3.152399	2.929856
17256336	Krtap9-5	keratin associated protein 9-5	3.039976	3.456447	4.556323	3.261894	3.567516	3.664204
17331859	Gm20741	keratin associated protein pseudogene	3.462322	3.826937	3.051799	3.018894	3.545601	3.324322
17242318	LOC102640500	keratin-associated protein 10-8-like	52.24822	66.21013	68.70532	56.0946	54.38776	56.57724
17326936	LOC102637070	keratin-associated protein 20-2-like	4.4676	3.900313	5.725374	3.102863	3.052349	3.350276
17326938	LOC102637192	keratin-associated protein 20-2-like	2.813118	2.933636	2.432821	2.690753	3.767872	3.216206
17326944	LOC102637417	keratin-associated protein 20-2-like	24.53841	31.39363	31.91737	32.471	25.22776	28.82063
17498075	LOC105243101	keratin-associated protein 5-4-like	8.925667	4.52485	7.88926	3.974546	4.880402	4.669441
17485192	LOC105243090	keratin-associated protein 5-5-like	3.359788	6.029841	3.520646	4.215022	3.64753	4.798821
17399386	Krtcap2	keratinocyte associated protein 2	40.91931	47.32301	57.73283	36.29452	51.60754	49.28819
17436099	Krtcap3	keratinocyte associated protein 3	17.94003	19.86022	19.54151	28.81104	22.38949	16.66408
17476557	Krtdap	keratinocyte differentiation associated protein	5.835717	6.346143	6.39875	6.198847	5.719187	7.657959
17419613	Kdf1	keratinocyte differentiation factor 1	17.25114	15.36398	16.31356	16.35092	16.70884	16.48601
17407476	Kprp	keratinocyte expressed, proline-rich	5.762281	5.447446	6.319761	5.531727	5.169519	5.261594
17236816	Kera	keratocan	5.611786	5.317212	6.323486	4.842862	5.120306	5.080463
17435934	Khk	ketohexokinase	363.4996	268.532	263.3997	329.9355	294.1422	361.8656
17300697	Khnyn	KH and NYN domain containing	17.91554	21.33722	26.35528	26.3361	23.62791	25.54165
17211416	Khdc1a	KH domain containing 1A	3.412384	2.833784	3.782657	3.220646	4.627779	2.742877
17211429	Khdc1b	KH domain containing 1B	3.147268	2.937095	2.746758	4.150917	3.627184	2.603748
17211423	Khdc1c	KH domain containing 1C	4.628517	5.137762	3.263862	6.943774	2.166911	3.520475
17519290	Khdc3	KH domain containing 3, subcortical maternal complex member	6.706314	6.831489	8.882895	6.42466	6.610136	6.161493
17430533	Khdrbs1	KH domain containing, RNA binding, signal transduction associated 1	21.7851	27.06507	22.94694	31.29434	26.87141	27.39309
17211548	Khdrbs2	KH domain containing, RNA binding, signal transduction associated 2	7.475189	5.558016	6.664931	6.94647	7.013526	7.334405
17312054	Khdrbs3	KH domain containing, RNA binding, signal transduction associated 3	44.14567	52.49834	44.30599	45.59709	35.14676	43.41856
17346427	Khsrp	KH-type splicing regulatory protein	73.51058	62.36696	70.62172	56.57999	65.41094	64.21997
17471980	Kap	kidney androgen regulated protein	4.143213	3.329418	3.507176	3.367388	3.890997	3.714325
17357861	Keg1	kidney expressed gene 1	227.4617	120.8237	58.2958	227.1167	107.2638	58.88752
17465407	Kcp	kielin/chordin-like protein	11.97165	21.0994	21.1615	18.36644	13.98482	14.32556
17241420	Kif1bp	KIF1 binding protein	23.04785	20.72356	24.37787	27.17466	21.77242	21.31947
17537906	Kir3dl1	killer cell immunoglobulin-like receptor, three domains, long cytoplasmic tail, 1	5.499346	5.300432	3.782841	4.846758	4.99675	4.603113
17544740	Kir3dl2	killer cell immunoglobulin-like receptor, three domains, long cytoplasmic tail, 2	2.575442	2.728977	3.401238	3.06728	2.460941	2.372585
17463557	Klre1	killer cell lectin-like receptor family E member 1	3.227484	2.830938	2.955864	3.238038	3.311558	3.277305
17471608	Klri1	killer cell lectin-like receptor family I member 1	3.757909	4.150543	3.339703	3.381258	3.381258	3.043265
17471616	Klri2	killer cell lectin-like receptor family I member 2	3.370564	2.423799	3.275488	2.953407	2.848278	2.661593
17471768	Klra10	killer cell lectin-like receptor subfamily A, member 10	2.050364	2.713378	2.083545	3.038396	2.462903	2.391656
17471717	Klra11-ps	killer cell lectin-like receptor subfamily A, member 11, pseudogene [Source:MGI Sy	4.315058	3.567145	2.728712	3.008658	3.008658	3.008658
17471744	Klra14-ps	killer cell lectin-like receptor subfamily A, member 14, pseudogene	3.738838	3.180955	5.776838	4.941914	2.800064	5.662032
17471757	Klra9	killer cell lectin-like receptor subfamily A, member 9	2.902568	2.211538	2.657777	2.620224	2.329386	2.586103
17471445	Klrb1	killer cell lectin-like receptor subfamily B member 1	3.607226	6.031346	6.589368	4.405788	7.377029	3.300352
17463493	Klrb1-ps1	killer cell lectin-like receptor subfamily B member 1, pseudogene 1	3.05472	2.623477	2.411933	2.606909	2.565162	3.959944
17471431	Klrb1a	killer cell lectin-like receptor subfamily B member 1A	2.891925	2.586982	2.601782	2.015545	4.511259	2.52671
17471464	Klrb1b	killer cell lectin-like receptor subfamily B member 1B	3.684145	3.629599	3.483144	5.333158	3.841128	3.853448
17471452	Klrb1c	killer cell lectin-like receptor subfamily B member 1C	4.349954	4.903901	4.423566	3.480778	3.902125	4.109378
17463482	Klrb1f	killer cell lectin-like receptor subfamily B member 1F	3.843232	4.659096	3.825345	4.453841	3.832246	3.151678
17471598	Klrc1	killer cell lectin-like receptor subfamily C, member 1	3.307979	4.90013	2.403889	3.233799	4.049658	4.97715
17471586	Klrc2	killer cell lectin-like receptor subfamily C, member 2	2.87697	2.714977	3.135401	2.602972	2.512285	2.393928
17471578	Klrc3	killer cell lectin-like receptor subfamily C, member 3	2.049526	1.918276	1.797971	2.60877	3.291146	2.86501
17470533	Klrg1	killer cell lectin-like receptor subfamily G, member 1	3.755617	3.108848	3.339703	3.544495	3.41236	3.334776
17466025	Klrg2	killer cell lectin-like receptor subfamily G, member 2	8.774884	10.56961	11.41477	11.14126	11.04384	11.04905
17471565	Klrk1	killer cell lectin-like receptor subfamily K, member 1	5.824079	5.794604	6.154962	5.607859	5.074761	6.700599
17471635	Klra17	killer cell lectin-like receptor, subfamily A, member 17	3.911928	3.587428	3.990003	3.573332	4.48597	3.607326
17471828	Klra2	killer cell lectin-like receptor, subfamily A, member 2	8.786813	10.60175	9.624289	10.16022	12.9128	9.333059
17471778	Klra3	killer cell lectin-like receptor, subfamily A, member 3	2.452937	2.460156	2.664805	3.934881	2.490491	2.937808
17471648	Klra5	killer cell lectin-like receptor, subfamily A, member 5	4.047497	3.347537	2.961237	3.309515	2.895395	2.545713
17471707	Klra6	killer cell lectin-like receptor, subfamily A, member 6	2.485524	2.404955	2.404872	2.582217	2.478092	3.33931
17471659	Klra7	killer cell lectin-like receptor, subfamily A, member 7	2.406006	2.191958	2.509865	2.406006	2.406006	2.393049
17463567	Klrd1	killer cell lectin-like receptor, subfamily D, member 1	4.945615	3.463138	4.360701	3.52089	4.153795	4.905941
17406492	Kirrel	kin of IRRE like (Drosophila)	8.664701	10.47196	10.22139	9.337394	7.566835	8.908546
17489071	Kirrel2	kin of IRRE like 2 (Drosophila)	7.19797	7.698994	7.698994	7.698994	7.698994	7.734886
17515843	Kirrel3	kin of IRRE like 3 (Drosophila)	5.878304	6.992896	6.035319	8.381625	7.888549	9.212163
17525324	Kirrel3os	kin of IRRE like 3 (Drosophila), opposite strand	4.341158	4.701301	4.750206	3.476221	4.386469	5.209193
17274574	Kidins220	kinase D-interacting substrate 220	134.9125	117.045	118.0047	105.7688	100.654	105.2444
17448924	Kdr	kinase insert domain protein receptor	104.1934	80.02784	106.0529	90.68047	56.4116	59.13908
17484419	Kndc1	kinase non-catalytic C-lobe domain (KIND) containing 1	5.94456	7.864309	6.597503	6.183913	4.588333	5.394092
17266534	Ksr1	kinase suppressor of ras 1	12.35189	11.36259	13.80204	12.6172	10.57906	13.79258
17441429	Ksr2	kinase suppressor of ras 2	7.680552	10.54794	9.62529	8.553471	7.919504	7.630936
17299353	Ktn1	kinectin 1	23.43739	31.40647	31.42346	32.59458	29.59292	28.00087
17359020	Kif11	kinesin family member 11	8.086638	6.193694	6.718318	5.934679	7.038119	6.193694
17426251	Kif12	kinesin family member 12	7.519361	7.519361	7.558657	7.953556	6.940791	7.841937
17292215	Kif13a	kinesin family member 13A	25.51723	31.27613	31.62777	31.83672	30.91532	31.62777
17301300	Kif13b	kinesin family member 13B	83.00003	86.41201	77.18133	85.29977	84.07996	80.36024
17217789	Kif14	kinesin family member 14	3.782107	4.743954	5.205331	4.355897	4.719716	4.514042
17523494	Kif15	kinesin family member 15	2.684884	3.374561	3.75161	3.06711	3.576628	2.964153
17392279	Kif16b	kinesin family member 16B	28.32649	31.89437	32.74063	35.90642	23.20767	28.75279
17376974	Kif16bos	kinesin family member 16B, opposite strand	4.01461	4.287242	4.345674	4.426081	4.256861	3.950294
17420462	Kif17	kinesin family member 17	8.933156	10.06092	10.9598	9.459278	7.142653	10.44203
17374058	Kif18a	kinesin family member 18A	3.941296	4.314939	4.314939	3.828794	4.521088	4.735248
17270369	Kif18b	kinesin family member 18B	6.663592	9.268719	7.698994	8.733379	7.260556	8.750892
17537207	Gm7134	kinesin family member 18B pseudogene	10.55431	10.47022	6.905663	10.93961	9.886706	6.447813

17258064	Kif19a	kinesin family member 19A	8.942821	9.057356	8.995229	9.806715	10.40107	11.01097
17225630	Kif1a	kinesin family member 1A	5.891806	5.891806	5.316438	5.868981	5.127978	5.891806
17433055	Kif1b	kinesin family member 1B	142.2259	186.945	200.7464	172.0209	170.1437	178.6324
17252200	Kif1c	kinesin family member 1C	155.6887	175.2888	182.9138	168.5614	166.3583	166.1716
17349433	Kif20a	kinesin family member 20A	6.293271	6.313019	6.05153	6.899778	6.245989	8.254266
17358838	Kif20b	kinesin family member 20B	4.21546	5.618558	4.423566	4.48426	3.379852	4.597215
17320614	Kif21a	kinesin family member 21A	96.84663	113.9021	116.9607	96.93663	106.8646	127.0736
17217728	Kif21b	kinesin family member 21B	10.87776	8.213744	9.515899	11.62243	10.4838	12.98874
17496568	Kif22	kinesin family member 22	9.898569	6.236082	6.214766	7.344224	9.46938	7.518683
17527934	Kif23	kinesin family member 23	4.952922	4.801649	4.801649	4.801649	4.014165	5.193114
17424219	Kif24	kinesin family member 24	3.65852	3.433524	3.776422	3.383696	3.729055	3.462322
17279299	Kif26a	kinesin family member 26A	8.29108	9.310251	10.03264	10.60652	9.310251	8.81127
17219997	Kif26b	kinesin family member 26B	10.49885	12.86857	12.53708	14.61999	13.09587	11.01174
17293116	Kif27	kinesin family member 27	3.545846	3.844724	3.541278	3.927959	3.844724	4.590305
17296052	Kif2a	kinesin family member 2A	15.21708	19.05965	16.22571	17.80425	15.12874	17.85802
17267768	Kif2b	kinesin family member 2B	4.505384	4.923963	5.620584	4.801649	4.27718	4.801649
17428803	Kif2c	kinesin family member 2C	5.122613	5.231302	5.556437	5.225395	4.555944	4.824669
17249561	Kif3a	kinesin family member 3A	10.77764	9.22603	10.33553	11.81133	8.845232	7.911493
17377926	Kif3b	kinesin family member 3B	18.08548	14.92925	16.19706	15.36813	12.04715	16.19706
17273591	Kif3c	kinesin family member 3C	9.512206	13.69654	10.93901	8.822026	9.973432	9.973432
17536566	Kif4	kinesin family member 4	3.263862	3.862781	3.421431	2.972692	4.067747	3.265627
17277976	Kif4-ps	kinesin family member 4, pseudogene	4.681238	2.550906	2.60299	4.010578	4.40124	2.856127
17245798	Kif5a	kinesin family member 5A	4.423597	5.653052	4.954193	4.738597	5.74143	5.18592
17352462	Kif5b	kinesin family member 5B	112.3674	102.9143	89.29392	92.08409	99.60871	89.91812
17370659	Kif5c	kinesin family member 5C	3.339703	3.003116	2.719007	4.263948	4.352065	3.433786
17338492	Kif6	kinesin family member 6	6.347551	6.14831	6.968509	6.395468	6.811205	6.417093
17492385	Kif7	kinesin family member 7	10.48228	10.48228	10.43957	10.34542	10.91606	8.776952
17522377	Kif9	kinesin family member 9	2.526973	3.883991	6.204436	5.716571	3.697628	4.297001
17343652	Kifc1	kinesin family member C1	3.784467	9.011267	4.316165	3.422736	4.17342	6.904467
17312531	Kifc2	kinesin family member C2	9.636037	12.88249	17.10054	13.72163	11.6509	12.28529
17511927	Kifc3	kinesin family member C3	27.29377	28.37896	30.07577	26.76118	26.76118	23.65331
17334983	Kifc5b	kinesin family member C5B	2.648673	5.550278	5.126307	5.704577	4.396605	8.607415
17279210	Klc1	kinesin light chain 1	36.39681	39.33086	38.24714	38.92001	44.03046	36.02584
17361470	Klc2	kinesin light chain 2	13.25192	17.00232	11.48708	14.97288	15.73363	14.76194
17487226	Klc3	kinesin light chain 3	14.37958	14.37958	14.35031	12.82876	13.96477	15.75677
17345499	Klc4	kinesin light chain 4	53.12761	49.76743	50.45271	54.96114	41.57459	61.20043
17435734	Gm4961	kinesin superfamily protein 2C pseudogene	2.590661	2.982516	2.669815	3.222522	2.976321	3.339703
17218765	Kifap3	kinesin-associated protein 3	23.61841	19.81238	24.56121	24.56121	20.31481	26.6588
17442332	Kntc1	kinetochore associated 1	3.669211	3.993241	3.719581	4.214028	3.975345	3.638743
17374632	Knstrn	kinetochore-localized astrin/SPAG5 binding	6.171709	6.903223	6.438167	6.501443	7.042609	6.54798
17324356	Kng1	kininogen 1	356.1621	374.0585	392.9604	348.7236	456.9239	329.6406
17329361	Kng2	kininogen 2	506.0268	509.6419	559.669	424.2821	657.784	462.7054
17417336	Kncn	kinocilin	10.63369	15.30679	8.630356	12.2221	8.407751	14.31951
17217329	Kiss1	KiSS-1 metastasis-suppressor	11.4571	12.64574	9.089116	12.97794	9.355469	12.67971
17235029	Kiss1r	KISS1 receptor	6.883632	9.391464	9.391464	9.391464	9.458884	14.6213
17236900	Kitl	kit ligand	5.562487	4.91704	5.128582	4.383217	5.889397	3.941733
17438272	Kit	kit oncogene	17.32796	15.09344	20.81483	21.81684	15.28177	13.11676
17377268	Kiz	kizuna centrosomal protein	34.33243	27.63702	32.12178	32.62826	36.70895	36.05182
17445238	Kl	klotho	4.519929	5.215986	4.665106	4.581246	6.992896	4.625278
17437765	Klb	klotho beta	77.71817	103.4737	91.21948	101.2887	118.439	98.06049
17358324	Kank1	KN motif and ankyrin repeat domains 1	63.95787	60.45333	62.6386	62.22791	42.69455	50.27328
17524760	Kank2	KN motif and ankyrin repeat domains 2	37.93191	40.23307	42.74459	36.57079	42.17073	42.35734
17336175	Kank3	KN motif and ankyrin repeat domains 3	12.23634	12.02096	17.74536	14.79082	12.66557	15.89466
17427476	Kank4	KN motif and ankyrin repeat domains 4	11.00327	11.47641	12.41851	12.09023	10.44623	12.21439
17415711	Kank4os	KN motif and ankyrin repeat domains 4, opposite strand	5.768555	6.320856	6.290436	8.269557	5.676415	6.67242
17458163	Krba1	KRAB-A domain containing 1	14.89287	11.95805	13.84401	10.94052	12.09467	15.83231
17524677	Kri1	KRI1 homolog (S. cerevisiae)	11.34567	14.75795	13.25128	14.58062	15.09187	16.45564
17260137	Kremen1	kringle containing transmembrane protein 1	33.41282	36.06031	34.37315	35.49638	31.33776	38.30418
17341595	Kremen2	kringle containing transmembrane protein 2	14.84608	15.43539	17.64613	18.49675	18.64194	18.8616
17434381	Krit1	KRIT1, ankyrin repeat containing	63.76929	62.66205	50.74698	62.84953	57.16103	57.16103
17237284	Krr1	KRR1, small subunit (SSU) processome component, homolog (yeast)	49.00408	38.62653	36.75249	47.83849	37.05535	41.29857
17503254	Klf1	Kruppel-like factor 1 (erythroid)	8.228959	8.346666	7.363543	8.526124	10.38932	8.806386
17316690	Klf10	Kruppel-like factor 10	20.57371	44.18761	41.91835	49.0503	54.76112	47.74044
17274532	Klf11	Kruppel-like factor 11	45.55918	73.53147	65.51621	57.27843	61.28576	55.96023
17309099	Klf12	Kruppel-like factor 12	46.53551	52.83639	51.96974	49.40893	52.32397	61.18177
17491788	Klf13	Kruppel-like factor 13	18.28451	20.05139	20.00266	21.40818	25.07278	24.03409
17465597	Klf14	Kruppel-like factor 14	12.22193	11.30509	11.82944	11.11395	9.634635	15.34844
17460826	Klf15	Kruppel-like factor 15	136.2942	181.9124	193.1182	178.401	206.5218	263.8108
17243010	Klf16	Kruppel-like factor 16	15.69094	19.28597	16.7019	13.02779	17.11762	15.99069
17428904	Klf17	Kruppel-like factor 17	9.123011	6.439193	11.798	7.340763	4.968705	8.119439
17502440	Klf2	Kruppel-like factor 2 (lung)	20.02388	26.01208	24.76619	28.22008	15.14154	23.59174
17437674	Klf3	Kruppel-like factor 3 (basic)	69.40992	80.31848	73.08229	70.93226	77.54759	83.89309
17425401	Klf4	Kruppel-like factor 4 (gut)	9.477129	13.16769	15.01987	13.98948	13.43813	13.9099
17302397	Klf5	Kruppel-like factor 5	7.698994	8.511008	7.88587	7.672677	6.951335	7.156777
17285000	Klf6	Kruppel-like factor 6	41.28655	64.35231	68.44817	37.95479	43.00493	41.35156
17223766	Klf7	Kruppel-like factor 7 (ubiquitous)	12.13549	13.86254	14.46105	12.44626	11.47061	12.76711
17539004	Klf8	Kruppel-like factor 8	3.062691	3.263862	3.566428	3.374047	2.997542	4.160134
17358214	Klf9	Kruppel-like factor 9	180.4693	220.5375	178.1946	197.9758	209.4424	231.396
17416761	Kti12	KTI12 homolog, chromatin associated (S. cerevisiae)	57.67243	56.07361	51.7763	53.40045	44.9893	53.49789
17391270	Kcnip3	Kv channel interacting protein 3, calsenilin	7.954659	10.9513	10.09144	9.633166	9.063888	10.79192
17447963	Kcnip4	Kv channel interacting protein 4	2.608789	3.126763	4.064647	4.622073	4.028548	3.063647
17261622	Kcnip1	Kv channel-interacting protein 1	5.605954	5.567964	5.984746	6.539317	6.038667	7.202811
17365270	Kcnip2	Kv channel-interacting protein 2	3.721102	3.827706	3.389165	3.422807	3.25654	4.196335
17510080	Kxd1	KxDL motif containing 1	10.9356	15.0694	18.92144	14.64778	18.86434	16.58101

17370527	Kynu	kynureninase (L-kynurenine hydrolase)	314.6491	246.7267	231.2453	295.6384	275.8576	323.5063
17219789	Kmo	kynurenine 3-monooxygenase (kynurenine 3-hydroxylase)	718.0199	721.3722	762.4823	791.8866	714.7129	687.0208
17520835	Ky	kyphoscoliosis peptidase	3.862166	3.837748	3.887466	4.038053	6.53062	3.771007
17542654	Lage3	L antigen family, member 3	6.411669	8.422742	7.064268	6.178343	9.034032	7.929995
17379139	L3mbtl1	l(3)mbt-like (Drosophila)	5.24779	4.129108	3.70494	5.719821	4.06138	4.643459
17313322	L3mbtl2	l(3)mbt-like 2 (Drosophila)	53.79201	50.49295	38.74344	46.02418	41.71198	45.99069
17239889	L3mbtl3	l(3)mbt-like 3 (Drosophila)	13.75811	18.09413	17.81475	14.17385	18.82477	18.78423
17339289	L3mbtl4	l(3)mbt-like 4 (Drosophila)	5.667608	4.830391	4.699476	5.112149	5.127445	4.016893
17542419	L1cam	L1 cell adhesion molecule	6.791216	6.287864	7.084078	8.055365	9.746539	7.879423
17281609	L2hgdh	L-2-hydroxyglutarate dehydrogenase	80.304	74.97096	62.68333	73.82154	68.25108	80.70633
17281791	L3hypdh	L-3-hydroxyproline dehydratase (trans-)	36.54013	31.36209	32.01975	33.47309	33.95142	35.72619
17249929	Larp1	La ribonucleoprotein domain family, member 1	278.6094	249.7308	266.8879	252.4406	285.3661	254.6126
17397320	Larp1b	La ribonucleoprotein domain family, member 1B	200.253	171.9148	152.6105	196.8278	185.7772	224.1033
17314910	Larp4	La ribonucleoprotein domain family, member 4	184.9434	170.6776	133.2083	160.7795	163.5434	157.9725
17285074	Larp4b	La ribonucleoprotein domain family, member 4B	212.2308	202.7011	197.2867	196.3563	186.9748	178.6757
17518095	Larp6	La ribonucleoprotein domain family, member 6	7.30017	10.55233	8.946417	12.99487	9.310251	8.047162
17410190	Larp7	La ribonucleoprotein domain family, member 7	28.72251	25.09486	28.11094	27.97391	29.39258	31.76883
17308772	Lacc1	laccase (multicopper oxidoreductase) domain containing 1	24.30599	31.85218	40.8989	26.16108	27.72182	23.93567
17321221	Lalba	lactalbumin, alpha	2.916969	3.338616	2.708173	3.337713	2.615705	4.182803
17528418	Lactb	lactamase, beta	75.02763	60.70906	61.90654	59.1354	65.75187	68.18157
17221360	Lactb2	lactamase, beta 2	778.4713	626.3607	556.524	695.0341	524.7367	619.4526
17420139	Lactbl1	lactamase, beta-like 1	6.584026	5.453264	6.902026	5.874883	6.049517	5.89675
17226531	Lct	lactase	4.20927	6.287864	4.20927	4.20927	3.971599	4.130167
17518278	Lctl	lactase-like	4.20927	4.339203	4.579849	3.988704	4.130167	4.20927
17478225	Ldha	lactate dehydrogenase A	21.2659	21.77031	19.28571	18.72923	21.32613	20.12016
17367190	Gm13315	lactate dehydrogenase A pseudogene	5.768595	7.114735	8.734772	9.314769	8.389065	6.373121
17340493	Ldhal6b	lactate dehydrogenase A-like 6B	6.089737	5.79103	7.223059	8.555309	6.666641	7.215653
17472517	Ldhb	lactate dehydrogenase B	6.464954	6.764109	6.555655	6.67825	6.46697	7.294757
17478243	Ldhc	lactate dehydrogenase C	5.338965	5.338965	5.809001	6.436204	5.338965	5.238632
17513135	Ldhd	lactate dehydrogenase D	325.2285	243.0619	202.8838	235.1129	248.672	209.9759
17240538	Lace1	lactation elevated 1	25.30008	20.95017	27.69839	25.81182	12.82165	25.37208
17267504	Lpo	lactoperoxidase	8.833897	9.182646	9.448793	6.488343	8.023713	9.514514
17522555	Ltf	lactotransferrin	4.01461	5.007811	5.617429	4.980222	5.525749	5.035029
17217639	Lad1	ladinin	35.42608	37.29913	42.29397	44.45612	31.71163	31.54217
17365193	Lbx1	ladybird homeobox homolog 1 (Drosophila)	6.929398	6.189659	4.913905	6.707895	6.805872	6.701946
17459929	Lbx2	ladybird homeobox homolog 2 (Drosophila)	4.768283	4.793867	5.635252	5.60576	4.469582	4.806877
17350378	Lvrn	laeverin	4.766268	4.20927	4.165385	5.079688	3.875547	4.18608
17406783	Lmna	lamin A	27.72643	25.43212	28.93793	28.07025	27.14642	27.95298
17230550	Lbr	lamin B receptor	44.67451	30.78701	31.82768	28.91367	31.33059	27.62476
17230566	Lbr	lamin B receptor	3.566799	2.855988	2.901703	3.182653	2.369972	3.41771
17350740	Lmnb1	lamin B1	20.93101	20.96571	20.901	18.85532	26.00199	23.13572
17243128	Lmnb2	lamin B2	15.29894	16.08785	14.1825	12.79448	14.50311	16.38348
17497796	Lmntd2	lamin tail domain containing 2	9.004399	7.992573	7.118928	9.182137	7.868235	7.158613
17274775	Lamb1	laminin B1	19.78191	23.2805	20.74224	18.62166	19.01729	17.88539
17369580	Lamc3	laminin gamma 3	7.776063	10.38745	8.435479	8.222061	6.579236	9.57087
17339223	Lama1	laminin, alpha 1	13.96264	17.23047	15.2714	14.20119	12.92399	17.71071
17239919	Lama2	laminin, alpha 2	6.791828	7.412396	7.48421	7.412396	7.412396	7.169189
17348492	Lama3	laminin, alpha 3	11.03412	7.208854	8.276381	9.13972	7.974256	8.384124
17232593	Lama4	laminin, alpha 4	12.13532	12.70769	14.10183	10.28693	12.33572	11.32195
17395512	Lama5	laminin, alpha 5	7.682788	10.00279	12.30131	10.48198	8.512424	9.398562
17521727	Lamb2	laminin, beta 2	11.24366	12.17669	12.04991	8.650539	8.232782	12.95251
17220944	Lamb3	laminin, beta 3	15.86538	18.49649	23.92671	15.361	13.9918	13.66913
17228164	Lamc1	laminin, gamma 1	31.52709	39.95489	48.70979	40.16119	32.3553	33.72742
17228136	Lamc2	laminin, gamma 2	6.992896	6.844855	6.170554	5.024361	7.904641	7.593474
17417826	Lao1	L-amino acid oxidase 1	3.387052	3.883991	3.729055	3.363259	3.461194	4.809244
17223932	Lancl1	LanC (bacterial lantibiotic synthetase component C)-like 1	41.52937	39.3126	46.71746	49.14354	38.4084	38.59173
17458990	Lancl2	LanC (bacterial lantibiotic synthetase component C)-like 2	25.8453	23.65298	22.34608	21.50306	25.52838	33.19951
17533199	Lancl3	LanC lantibiotic synthetase component C-like 3 (bacterial)	3.557121	3.263862	3.678481	3.427674	3.732986	3.40957
17234552	Lss	lanosterol synthase	45.50175	32.90534	41.43218	27.28886	44.09704	27.80495
17329692	Lsg1	large subunit GTPase 1 homolog (S. cerevisiae)	41.52534	31.88509	33.5358	33.306	32.66401	36.8944
17231545	Lats1	large tumor suppressor	50.32558	47.48862	53.77002	50.1612	51.63545	51.4486
17307184	Lats2	large tumor suppressor 2	19.08795	23.68776	18.28375	14.71657	19.08795	20.54065
17543336	Las1l	LAS1-like (S. cerevisiae)	25.21097	26.28704	29.44458	27.68092	32.03973	25.45187
17407431	Lce1a1	late cornified envelope 1A1	2.269965	2.042888	2.316024	2.419233	2.491907	2.269965
17407439	Lce1a2	late cornified envelope 1A2	4.268207	4.475193	4.204167	4.157568	3.263839	3.554533
17407435	Lce1b	late cornified envelope 1B	4.024697	3.943181	5.034994	3.889056	2.925047	4.024697
17399899	Lce1c	late cornified envelope 1C	3.46989	2.639894	3.009972	4.72682	2.71946	2.43628
17407444	Lce1d	late cornified envelope 1D	3.56334	3.484303	3.372761	3.418563	3.418563	3.885634
17407448	Lce1e	late cornified envelope 1E	3.344221	2.346743	2.233537	2.61105	2.782767	2.305932
17407452	Lce1f	late cornified envelope 1F	4.134836	5.939988	5.93686	3.842433	3.860827	4.055501
17407457	Lce1g	late cornified envelope 1G	2.132506	2.0075	2.251552	2.479244	2.241198	2.308171
17407461	Lce1h	late cornified envelope 1H	5.859388	4.681043	3.008282	4.681043	3.51745	3.623214
17407466	Lce1i	late cornified envelope 1I	2.101894	2.064611	2.365524	2.366476	1.854513	1.967072
17407471	Lce1j	late cornified envelope 1J	123.0184	124.7106	125.3284	111.4506	121.6228	117.5499
17407473	Lce1k	late cornified envelope 1K	2.147399	2.377112	2.252208	2.933392	2.238093	1.9438
17407481	Lce1l	late cornified envelope 1L	4.660452	2.527393	2.659804	2.341495	2.599402	2.504739
17407501	Lce1m	late cornified envelope 1M	3.050594	3.950787	3.426273	2.871352	2.430175	3.218258
17407489	Lce3a	late cornified envelope 3A	4.020653	3.460955	3.552316	3.417686	4.44311	2.710107
17399906	Lce3b	late cornified envelope 3B	2.387946	2.786743	2.953407	3.263862	2.188921	2.879304
17399910	Lce3c	late cornified envelope 3C	15.06188	23.2096	15.17085	14.64981	18.08724	13.09378
17399914	Lce3d	late cornified envelope 3D	23.26114	25.3473	23.42651	23.32896	36.24575	18.28755
17399917	Lce3e	late cornified envelope 3E	3.915075	2.794442	2.659804	2.126705	2.27365	2.659804
17399922	Lce3f	late cornified envelope 3F	2.406006	2.239691	2.285991	2.034015	2.458673	2.19439
17407426	Lce6a	late cornified envelope 6A	2.388161	2.426287	2.67402	2.606909	2.715824	2.897302

17407386	Lelp1	late cornified envelope-like proline-rich 1	2.856543	3.368485	3.374889	3.588547	3.458844	4.283203
17480978	Lamtor1	late endosomal/lysosomal adaptor, MAPK and MTOR activator 1	27.09454	22.87453	21.9231	28.47038	28.71654	26.74431
17406817	Lamtor2	late endosomal/lysosomal adaptor, MAPK and MTOR activator 2	27.468	28.68431	30.03952	30.96507	29.42315	27.10862
17403025	Lamtor3	late endosomal/lysosomal adaptor, MAPK and MTOR activator 3	33.94507	34.39626	38.15235	33.91402	44.66213	49.54783
17443818	Lamtor4	late endosomal/lysosomal adaptor, MAPK and MTOR activator 4	61.0276	56.25424	61.0276	61.48276	61.0276	63.21171
17401556	Lamtor5	late endosomal/lysosomal adaptor, MAPK and MTOR activator 5	9.490407	9.761616	9.016453	8.640591	10.47479	10.54751
17339729	Ltbp1	latent transforming growth factor beta binding protein 1	5.846001	6.494012	6.252703	7.008958	5.618987	6.987857
17282570	Ltbp2	latent transforming growth factor beta binding protein 2	9.394589	7.953446	9.05108	8.466995	7.512224	8.823738
17356526	Ltbp3	latent transforming growth factor beta binding protein 3	13.78436	13.78436	13.78436	12.543	11.87248	14.71872
17488179	Ltbp4	latent transforming growth factor beta binding protein 4	25.25137	25.85641	28.37759	23.32783	22.25149	24.93966
17405737	Lxn	latexin	12.99919	11.29099	12.74571	14.56749	11.83567	12.97749
17526956	Layn	layilin	8.119608	6.65639	6.992896	7.291825	6.695492	5.56106
17357270	Lbhd1	LBH domain containing 1	74.5874	56.67144	53.29285	66.65957	66.36129	62.63599
17381026	Lime1	Lck interacting transmembrane adaptor 1	11.24366	12.45869	13.4647	12.45869	11.88508	13.66932
17529290	Lca5	Leber congenital amaurosis 5 (human)	10.64165	10.87558	9.545164	10.86789	12.03021	11.59459
17332457	Lca5l	Leber congenital amaurosis 5-like	5.729543	4.801649	4.346548	4.999764	4.999764	4.999764
17512565	Lcat	lecithin cholesterol acyltransferase	704.9612	621.2155	610.1542	572.662	698.4389	571.6031
17406247	Lrat	lecithin-retinol acyltransferase (phosphatidylcholine-retinol-O-acyltransferase)	49.82162	59.54919	40.67696	39.2081	27.09341	35.60604
17312829	Lgals1	lectin, galactose binding, soluble 1	54.31091	112.3285	131.3234	89.7444	66.16884	79.109
17362342	Lgals12	lectin, galactose binding, soluble 12	6.843622	4.471875	5.172458	6.73543	5.033807	5.965074
17299329	Lgals3	lectin, galactose binding, soluble 3	21.22364	27.18521	37.34388	21.191	19.89177	21.56186
17475989	Lgals4	lectin, galactose binding, soluble 4	12.69202	28.35851	27.15696	27.05006	19.18597	29.18914
17476001	Lgals7	lectin, galactose binding, soluble 7	5.403297	4.893986	4.771193	4.771193	4.695341	4.419429
17290629	Lgals8	lectin, galactose binding, soluble 8	215.8088	218.5682	233.429	231.093	222.3749	220.4137
17266520	Lgals9	lectin, galactose binding, soluble 9	1264.157	1409.277	1518.436	1296.494	1137.783	1255.521
17319091	Lgals2	lectin, galactose-binding, soluble 2	4.070568	3.104046	3.336811	3.431002	3.365241	3.076841
17260898	Lgalsl	lectin, galactoside binding-like	17.72667	19.51566	20.55703	23.86261	27.25936	17.7913
17260907	Lgalsl	lectin, galactoside binding-like	2.625096	2.824352	2.63929	2.433748	3.658646	2.544902
17272785	Lgals3bp	lectin, galactoside-binding, soluble, 3 binding protein	172.6313	212.7463	253.5752	201.6417	158.5331	164.8706
17527548	Lman1l	lectin, mannose-binding 1 like	9.312768	9.209227	9.385477	9.121683	12.02571	10.04148
17292839	Lman2	lectin, mannose-binding 2	216.7262	181.9515	188.4032	187.1615	193.8948	173.8947
17222179	Lman2l	lectin, mannose-binding 2-like	24.91375	22.37181	23.99064	19.63696	26.47044	21.69845
17355092	Lman1	lectin, mannose-binding, 1	483.7641	368.6429	377.6484	372.8212	396.1739	364.3421
17220214	Lefty1	left right determination factor 1	6.738185	7.579906	5.239757	6.055139	6.992896	8.420524
17220197	Lefty2	left-right determination factor 2	6.992896	7.034754	5.801597	9.495737	10.53195	8.420524
17283445	Lgmn	legumain	182.7161	249.067	267.0433	175.9986	169.3566	170.4601
17217596	Lmod1	leiomodin 1 (smooth muscle)	10.99237	9.287047	10.28871	9.582553	7.245976	10.37695
17456428	Lmod2	leiomodin 2 (cardiac)	7.870689	7.542067	8.469401	7.323991	6.545348	9.292199
17469360	Lmod3	leiomodin 3 (fetal)	4.214028	3.79579	4.630924	3.971324	3.4851	5.587179
17324971	Lmln	leishmanolysin-like (metallopeptidase M8 family)	8.634783	6.486679	9.316577	8.192706	9.411535	8.839655
17217160	Lemd1	LEM domain containing 1	4.067437	5.167907	4.067437	3.725722	3.894748	4.067437
17342708	Lemd2	LEM domain containing 2	23.66922	22.50338	21.13629	26.19483	22.20758	26.67729
17245448	Lemd3	LEM domain containing 3	14.60732	23.3529	19.04249	22.19096	25.893	23.32815
17444464	Lmtk2	lemur tyrosine kinase 2	51.46679	49.89454	48.65412	51.09428	46.02737	45.4469
17478010	Lmtk3	lemur tyrosine kinase 3	7.128507	9.299286	8.636938	7.747334	9.657502	9.894796
17211533	Lgsn	lengsin, lens protein with glutamine synthetase domain	4.20927	4.20927	4.20927	3.847977	4.061999	4.017199
17407086	Lenep	lens epithelial protein	31.31235	30.16116	38.80606	29.13659	26.75618	31.57454
17407093	Lenep	lens epithelial protein	27.18088	24.48218	34.64968	24.48218	24.42624	23.73839
17477089	Lim2	lens intrinsic membrane protein 2	4.01461	4.01461	4.117932	3.732675	4.177848	4.01461
17519509	Leo1	Leo1, Paf1/RNA polymerase II complex component, homolog (S. cerevisiae)	48.10383	43.81491	36.30182	39.23855	35.03099	43.06011
17456545	Lep	leptin	3.541008	3.494776	3.933872	3.787485	2.518911	3.838919
17415979	Lepr	leptin receptor	42.97334	104.0591	186.7909	80.27284	100.9901	92.26042
17415973	Leprot	leptin receptor overlapping transcript	39.56629	62.22791	66.55661	56.82564	68.77537	58.7656
17508722	Leprotl1	leptin receptor overlapping transcript-like 1	69.2403	71.10603	64.52338	64.52338	83.70257	64.52338
17250475	Llgl1	lethal giant larvae homolog 1 (Drosophila)	13.321	14.50803	15.35365	14.43941	13.19482	14.23578
17258341	Llgl2	lethal giant larvae homolog 2 (Drosophila)	49.43188	45.41667	53.77561	47.19264	41.14057	46.20605
17493238	l7Rn6	lethal, Chr 7, Rinchik 6	42.6839	33.83654	32.11443	40.01752	27.83619	36.16043
17315033	Letmd1	LETM1 domain containing 1	73.32229	84.12251	80.65768	90.09468	77.90694	75.50394
17437247	Lap3	leucine aminopeptidase 3	556.3928	568.2689	573.357	536.1043	453.447	503.9127
17482884	Lcmt1	leucine carboxyl methyltransferase 1	23.88793	23.65445	24.68495	25.04395	21.18234	21.84816
17390424	Lcmt2	leucine carboxyl methyltransferase 2	15.07713	16.29141	14.37978	15.02195	14.30483	16.28961
17428582	Lurap1	leucine rich adaptor protein 1	6.060765	6.145243	6.096791	6.063497	6.314156	6.575816
17414984	Lurap1l	leucine rich adaptor protein 1-like	88.73922	101.7666	71.27084	71.63933	80.55823	96.32377
17440521	Lrcol1	leucine rich colipase-like 1	4.01461	3.68195	4.20927	4.20927	4.053224	4.20927
17215650	Lrrfip1	leucine rich repeat (in FLII) interacting protein 1	37.38221	33.20365	44.81128	35.88627	44.65903	41.43046
17522577	Lrrfip2	leucine rich repeat (in FLII) interacting protein 2	75.98854	75.56834	83.50496	78.51628	86.44197	83.92217
17396106	Lrrcc1	leucine rich repeat and coiled-coil domain containing 1	5.752185	6.104571	6.537278	5.629903	6.298988	5.73948
17475851	Lrfn1	leucine rich repeat and fibronectin type III domain containing 1	6.764719	5.552856	6.140133	6.154749	5.119348	7.786905
17338460	Lrfn2	leucine rich repeat and fibronectin type III domain containing 2	8.83206	7.611074	7.488932	6.715664	6.666179	8.060783
17489039	Lrfn3	leucine rich repeat and fibronectin type III domain containing 3	14.57749	11.03986	22.06075	12.68463	11.03986	6.285293
17361304	Lrfn4	leucine rich repeat and fibronectin type III domain containing 4	9.387233	11.0643	15.05426	11.83387	9.310251	11.23104
17275756	Lrfn5	leucine rich repeat and fibronectin type III domain containing 5	3.08378	3.624344	3.079957	3.843578	4.267437	3.354459
17444047	Elfn1	leucine rich repeat and fibronectin type III, extracellular 1	10.0483	10.64999	10.66876	9.902687	12.18446	9.21951
17319045	Elfn2	leucine rich repeat and fibronectin type III, extracellular 2	9.709655	12.08043	11.04537	12.73467	12.23227	9.973708
17527445	Lingo1	leucine rich repeat and Ig domain containing 1	5.673897	7.554834	8.071574	7.329526	6.076919	6.50266
17423939	Lingo2	leucine rich repeat and Ig domain containing 2	3.913312	3.78444	3.856681	3.730942	4.115649	3.400319
17243113	Lingo3	leucine rich repeat and Ig domain containing 3	11.08925	11.87701	13.51613	12.52562	12.97336	14.36733
17400066	Lingo4	leucine rich repeat and Ig domain containing 4	8.039691	9.276689	11.10664	10.15677	13.11122	9.20023
17384049	Lrsam1	leucine rich repeat and sterile alpha motif containing 1	14.83672	14.03892	14.54882	14.83672	13.58771	16.56608
17528963	Lrrc1	leucine rich repeat containing 1	5.637713	4.259399	3.942553	5.501176	5.604012	4.813479
17237537	Lrrc10	leucine rich repeat containing 10	7.543427	7.398075	5.519892	6.56525	8.829428	6.992896
17362671	Lrrc10b	leucine rich repeat containing 10B	4.811144	5.150884	6.323705	4.266816	6.47132	5.42811
17312589	Lrrc14	leucine rich repeat containing 14	6.992896	10.56126	10.1025	8.361895	8.415564	11.11387
17294397	Lrrc14b	leucine rich repeat containing 14B	12.52067	11.8029	13.39423	13.26589	10.64128	10.79881

17329631	Lrrc15	leucine rich repeat containing 15	5.405582	6.025037	5.25405	4.940955	4.310843	5.40311
17291275	Lrrc16a	leucine rich repeat containing 16A	20.27388	20.36853	15.40279	21.58833	13.72182	14.85308
17300441	Lrrc16b	leucine rich repeat containing 16B	13.62987	13.62987	13.50901	13.2343	13.04351	14.92946
17435107	Lrrc17	leucine rich repeat containing 17	6.171709	5.93106	7.265943	7.023694	6.171709	8.255582
17298642	Lrrc18	leucine rich repeat containing 18	4.724134	5.98006	6.161365	6.681028	5.490091	5.617663
17427262	Lrrc19	leucine rich repeat containing 19	3.462322	3.462322	3.382406	3.287635	3.027842	4.073814
17522536	Lrrc2	leucine rich repeat containing 2	3.852033	4.000749	4.501107	4.497396	3.922192	4.013553
17233708	Lrrc20	leucine rich repeat containing 20	15.83637	24.84218	31.32885	25.31218	23.75518	12.42445
17470867	Lrrc23	leucine rich repeat containing 23	3.729055	3.729055	4.147207	3.729055	3.729055	5.109492
17318695	Lrrc24	leucine rich repeat containing 24	9.73668	8.22189	8.467112	8.721825	9.26949	11.58969
17501989	Lrrc25	leucine rich repeat containing 25	13.2047	11.74748	12.15148	12.04131	9.602034	10.49267
17367916	Lrrc26	leucine rich repeat containing 26	9.265251	11.01937	8.78596	8.705336	10.52727	9.148277
17484353	Lrrc27	leucine rich repeat containing 27	7.232327	7.182271	6.639709	7.16025	7.092774	7.391771
17491999	Lrrc28	leucine rich repeat containing 28	40.25455	47.85607	54.02518	39.83598	37.79229	33.65572
17512381	Lrrc29	leucine rich repeat containing 29	30.21787	30.12794	28.29451	30.73456	25.45646	21.75432
17242330	Lrrc3	leucine rich repeat containing 3	79.32974	68.67866	83.80707	84.00481	83.56518	82.66186
17346894	Lrrc30	leucine rich repeat containing 30	5.270866	5.919971	5.195023	5.270866	4.124245	5.270866
17404534	Lrrc31	leucine rich repeat containing 31	2.896077	3.400154	3.705761	5.202157	3.64614	5.126059
17480485	Lrrc32	leucine rich repeat containing 32	31.50917	29.9871	33.49445	24.2355	16.93458	16.69842
17404520	Lrrc34	leucine rich repeat containing 34	2.408041	3.008741	2.421599	2.625842	3.507371	2.152898
17504811	Lrrc36	leucine rich repeat containing 36	3.062139	3.299277	4.071668	4.625823	4.379512	3.352227
17270523	Lrrc37a	leucine rich repeat containing 37A	2.813898	2.662497	2.540478	2.747863	2.802356	2.518317
17421174	Lrrc38	leucine rich repeat containing 38	6.615407	6.338933	5.880749	4.343566	5.74545	5.628459
17402039	Lrrc39	leucine rich repeat containing 39	5.937312	7.085354	8.18771	7.469336	5.545471	5.982962
17303593	Lrrc3b	leucine rich repeat containing 3B	3.500085	3.339703	3.70553	3.384986	3.339703	3.339703
17256066	Lrrc3c	leucine rich repeat containing 3C	19.81705	19.65439	18.78071	16.76186	15.45284	16.85822
17465332	Lrrc4	leucine rich repeat containing 4	5.338965	5.165257	3.561611	4.20927	5.27357	4.565871
17403895	Lrrc40	leucine rich repeat containing 40	28.09322	27.22896	29.08735	27.95646	26.65233	26.57707
17417349	Lrrc41	leucine rich repeat containing 41	40.82554	29.97089	30.27867	30.43522	27.60849	31.53583
17427917	Lrrc42	leucine rich repeat containing 42	28.18352	33.23727	29.37486	24.54862	30.6278	27.68457
17442307	Lrrc43	leucine rich repeat containing 43	4.064985	5.048483	5.487466	5.417736	4.861866	5.56106
17259475	Lrrc45	leucine rich repeat containing 45	17.43422	16.48258	18.20093	19.29025	21.57951	20.35371
17268430	Lrrc46	leucine rich repeat containing 46	17.3294	15.19822	11.01823	13.37215	15.0901	17.93455
17422302	Lrrc47	leucine rich repeat containing 47	27.02514	24.7007	17.97052	21.42183	28.33592	24.16672
17250344	Lrrc48	leucine rich repeat containing 48	5.93069	6.953118	5.347335	5.933363	6.133795	6.079278
17527883	Lrrc49	leucine rich repeat containing 49	8.626229	8.170079	7.264482	8.535644	5.594464	7.410701
17477468	Lrrc4b	leucine rich repeat containing 4B	16.23843	17.33	17.72778	18.14651	14.31296	17.81289
17373616	Lrrc4c	leucine rich repeat containing 4C	4.233527	3.806698	3.441053	3.249167	3.147552	2.986964
17493980	Lrrc51	leucine rich repeat containing 51	22.92071	20.84978	20.62597	19.25151	16.32555	20.27092
17229412	Lrrc52	leucine rich repeat containing 52	7.930325	8.359925	7.874182	7.250096	8.98202	7.28844
17387565	Lrrc55	leucine rich repeat containing 55	4.752643	3.191866	2.982061	2.787071	3.152552	3.743921
17484771	Lrrc56	leucine rich repeat containing 56	6.866239	9.045749	9.428903	9.848336	7.589685	9.240494
17390249	Lrrc57	leucine rich repeat containing 57	19.19103	18.38261	16.37258	16.91651	21.74422	15.73102
17325533	Lrrc58	leucine rich repeat containing 58	155.5894	146.3714	138.8468	135.5978	110.9149	108.8562
17255232	Lrrc59	leucine rich repeat containing 59	187.8347	142.9672	151.7634	147.22	155.8582	127.3584
17317608	Lrrc6	leucine rich repeat containing 6 (testis)	3.062691	2.953407	3.142385	2.606909	3.03507	3.185059
17458315	Lrrc61	leucine rich repeat containing 61	16.24338	13.26626	15.96289	13.82244	11.54911	11.25107
17308656	Lrrc63	leucine rich repeat containing 63	2.640608	2.276378	2.481049	2.459167	2.784137	2.681221
17448832	Lrrc66	leucine rich repeat containing 66	6.992896	5.576887	7.317902	6.992896	8.894108	4.886969
17423422	Lrrc69	leucine rich repeat containing 69	2.556695	2.6639	2.805193	2.682912	3.408554	2.153346
17411457	Lrrc7	leucine rich repeat containing 7	3.062691	2.918223	3.009972	3.027653	3.152246	3.177627
17406531	Lrrc71	leucine rich repeat containing 71	8.391158	8.072357	7.321961	7.698994	6.302351	9.766083
17280774	Lrrc72	leucine rich repeat containing 72	2.551596	2.493341	2.764484	2.260466	2.458673	2.120611
17338059	Lrrc73	leucine rich repeat containing 73	6.920182	7.698994	7.698994	8.064136	9.723933	7.826786
17277534	Lrrc74a	leucine rich repeat containing 74A	7.132461	5.830784	5.992924	6.171709	6.443658	6.296936
17328728	Lrrc74b	leucine rich repeat containing 74B	5.494735	6.318622	5.766436	5.65807	4.999417	5.77848
17264046	Lrrc75a	leucine rich repeat containing 75A	12.97884	12.26393	12.02579	12.00564	14.17916	13.33134
17250750	Lrrc75aos1	leucine rich repeat containing 75A, opposite strand 1 [Source:MGI Symbol;Acc:MG	4.681043	5.704024	5.084761	4.858093	5.084761	6.331205
17250746	Lrrc75aos2	leucine rich repeat containing 75A, opposite strand 2 [Source:MGI Symbol;Acc:MG	10.14741	9.981316	10.05255	8.474745	8.474745	8.616906
17241840	Lrrc75b	leucine rich repeat containing 75B	7.76061	8.941581	8.347591	7.590579	7.073283	9.038228
17439901	Lrrc8b	leucine rich repeat containing 8 family, member B	6.171709	7.177194	7.050717	5.885928	6.296849	6.335024
17439909	Lrrc8c	leucine rich repeat containing 8 family, member C	20.9615	22.50603	24.04592	22.30306	19.73275	13.06975
17498786	Lrrc8e	leucine rich repeat containing 8 family, member E	6.591469	6.994447	6.591469	7.095282	5.342852	7.6959
17369126	Lrrc8a	leucine rich repeat containing 8A	67.12043	73.84509	90.17527	71.17161	77.71763	60.98785
17439922	Lrrc8d	leucine rich repeat containing 8D	13.44642	13.71421	14.53279	14.36633	15.48264	14.44702
17450529	Lrrc8dos	leucine rich repeat containing 8D, opposite strand [Source:MGI Symbol;Acc:MGI:3	5.086065	4.508476	4.695439	5.215878	4.746519	5.150063
17276192	Lrrc9	leucine rich repeat containing 9	3.703322	3.170829	3.708819	3.696142	3.703322	3.703322
17245059	Lgr5	leucine rich repeat containing G protein coupled receptor 5	14.58517	12.13549	11.43703	10.83712	11.61752	14.83867
17392047	Lrrn4	leucine rich repeat neuronal 4	5.410493	5.925677	6.940021	5.453151	5.905258	6.283551
17275875	Lrr1	leucine rich repeat protein 1	2.947455	2.656101	2.672815	2.636749	2.353504	2.725309
17461330	Lrrn1	leucine rich repeat protein 1, neuronal	3.322041	3.208369	3.267144	3.263862	3.66644	3.512163
17217241	Lrrn2	leucine rich repeat protein 2, neuronal	17.67674	20.98825	20.39537	22.23931	19.21698	20.39537
17280888	Lrrn3	leucine rich repeat protein 3, neuronal	6.030928	6.578734	4.853212	7.505561	6.030928	5.342926
17459735	Lrrtm1	leucine rich repeat transmembrane neuronal 1	4.946952	5.352749	6.534288	5.796612	4.793236	5.232535
17353581	Lrrtm2	leucine rich repeat transmembrane neuronal 2	4.271788	6.084126	4.864259	5.684762	5.53027	5.253213
17241604	Lrrtm3	leucine rich repeat transmembrane neuronal 3	4.97929	4.995538	4.710891	4.801649	5.306351	4.801649
17459786	Lrrtm4	leucine rich repeat transmembrane neuronal 4	3.157385	3.061163	3.150673	3.116161	3.551651	3.340074
17434404	Lrrd1	leucine rich repeats and death domain containing 1	2.83459	2.395057	2.333604	2.234404	2.133497	2.865188
17421807	Lzic	leucine zipper and CTNNBIP1 domain containing	14.03814	17.09663	19.45623	15.05677	15.86153	15.57737
17420125	Luzp1	leucine zipper protein 1	18.20875	20.24551	17.83884	22.53897	17.08585	19.62839
17478496	Luzp2	leucine zipper protein 2	3.543758	3.09908	3.354087	3.117479	2.887918	3.158545
17538495	Luzp4	leucine zipper protein 4	5.425402	4.319128	4.209036	4.67848	6.252573	5.621441
17532526	Lztfl1	leucine zipper transcription factor-like 1	18.07383	18.2892	14.98046	16.62336	19.03299	17.69684
17535148	Ldoc1	leucine zipper, down-regulated in cancer 1	8.163852	6.907961	8.327769	6.825336	6.992896	10.29299

17319989	Ldoc1l	leucine zipper, down-regulated in cancer 1-like	4.20927	4.106047	4.20927	4.478607	4.20927	4.053758
17509801	Lzts1	leucine zipper, putative tumor suppressor 1	10.58508	12.46672	12.0464	11.93776	10.76271	13.07582
17509806	Lzts1	leucine zipper, putative tumor suppressor 1	3.681235	3.84699	3.237028	4.09553	4.798621	4.042618
17359796	Lzts2	leucine zipper, putative tumor suppressor 2	14.15765	16.10158	18.02616	20.69284	13.9461	19.21925
17391683	Lzts3	leucine zipper, putative tumor suppressor family member 3	11.46512	16.27979	16.37951	18.24185	20.05403	22.76097
17447150	Letm1	leucine zipper-EF-hand containing transmembrane protein 1	107.4841	92.35576	90.56992	109.0942	78.10538	93.18721
17508380	Letm2	leucine zipper-EF-hand containing transmembrane protein 2	18.74079	19.3489	29.80164	19.47674	22.75545	24.96509
17398095	Lekr1	leucine, glutamate and lysine rich 1	3.210838	3.604302	3.22422	3.25575	3.518157	3.218013
17346150	Lrg1	leucine-rich alpha-2-glycoprotein 1	537.6748	585.8794	646.7615	444.685	728.3257	383.478
17347751	Lrpprc	leucine-rich PPR-motif containing	184.4595	168.8912	152.8603	185.8635	155.2963	171.2618
17239257	LOC105245161	leucine-rich repeat extensin-like protein 5	3.830519	4.106872	3.883991	5.26247	3.291465	3.636272
17491898	Lrrk1	leucine-rich repeat kinase 1	23.24019	21.77469	20.8323	20.05097	20.90708	17.52473
17314260	Lrrk2	leucine-rich repeat kinase 2	6.825809	8.02021	6.559655	8.234779	7.229972	6.763641
17359143	Lgi1	leucine-rich repeat LGI family, member 1	2.529514	3.605002	2.81037	2.480619	2.994593	2.882519
17448064	Lgi2	leucine-rich repeat LGI family, member 2	3.462322	3.658976	3.729055	3.700371	3.729055	4.406032
17301746	Lgi3	leucine-rich repeat LGI family, member 3	6.299075	8.039468	6.992896	7.985983	4.59884	7.732259
17476578	Lgi4	leucine-rich repeat LGI family, member 4	5.175865	4.04716	4.614038	5.836567	5.557014	4.393346
17298910	Lrit1	leucine-rich repeat, immunoglobulin-like and transmembrane domains 1	26.18176	31.89061	23.77692	27.42786	25.28518	33.77608
17298917	Lrit2	leucine-rich repeat, immunoglobulin-like and transmembrane domains 2	5.864386	6.171709	6.287864	5.410343	6.171709	7.208055
17410306	Lrit3	leucine-rich repeat, immunoglobulin-like and transmembrane domains 3	4.030615	4.343867	4.403528	5.00219	4.800451	4.844138
17374098	Lgr4	leucine-rich repeat-containing G protein-coupled receptor 4	111.1381	123.0875	126.6269	113.6395	135.4278	126.6532
17227198	Lgr6	leucine-rich repeat-containing G protein-coupled receptor 6	9.037101	10.31241	11.71057	10.14685	7.247985	10.15529
17308626	Lrch1	leucine-rich repeats and calponin homology (CH) domain containing 1	23.27566	28.56111	28.29372	27.20632	26.20899	23.95459
17545199	Lrch2	leucine-rich repeats and calponin homology (CH) domain containing 2	5.120306	5.120306	5.120306	5.526362	5.120306	4.617558
17324932	Lrch3	leucine-rich repeats and calponin homology (CH) domain containing 3	27.08552	39.00535	35.77798	33.50582	42.69455	32.40647
17457092	Lrguk	leucine-rich repeats and guanylate kinase domain containing	3.516816	3.438645	3.516816	3.614359	3.516816	3.332235
17469217	Lrig1	leucine-rich repeats and immunoglobulin-like domains 1	49.00136	34.67514	40.9335	30.6586	31.19429	36.27038
17408729	Lrig2	leucine-rich repeats and immunoglobulin-like domains 2	12.78739	17.12141	16.18864	15.23914	17.11679	15.23914
17237811	Lrig3	leucine-rich repeats and immunoglobulin-like domains 3	27.93458	24.50663	25.68457	28.68212	27.87037	24.20327
17244623	Lrriq1	leucine-rich repeats and IQ motif containing 1	3.555913	2.890422	3.192729	2.820831	2.911051	2.952833
17403811	Lrriq3	leucine-rich repeats and IQ motif containing 3	7.870689	4.059185	5.617429	7.582639	5.021645	5.352712
17396564	Lrriq4	leucine-rich repeats and IQ motif containing 4	3.28308	3.058258	2.93384	3.406783	3.461938	3.362187
17298126	Lrtm1	leucine-rich repeats and transmembrane domains 1	13.78426	10.07348	11.9539	10.52428	22.02546	18.4205
17470291	Lrtm2	leucine-rich repeats and transmembrane domains 2	7.163973	7.34733	10.24523	6.271932	10.03098	7.608067
17453679	Lrwd1	leucine-rich repeats and WD repeat domain containing 1	16.76104	17.72434	18.18176	14.31296	17.55828	14.38646
17280836	Lsmem1	leucine-rich single-pass membrane protein 1	4.68679	5.046034	7.116313	5.210566	5.310398	4.20927
17530923	Lsmem2	leucine-rich single-pass membrane protein 2	4.322872	5.106837	4.772053	4.437816	4.231825	4.699509
17323503	Lztr1	leucine-zipper-like transcriptional regulator, 1	48.54598	50.56475	46.46685	52.11981	45.69391	39.79403
17341247	Lnpep	leucyl/cystinyl aminopeptidase	78.61812	94.74185	118.0431	93.49712	99.10047	112.9875
17354036	Lars	leucyl-tRNA synthetase	49.75743	44.41915	46.83548	49.75743	58.95491	42.56011
17523574	Lars2	leucyl-tRNA synthetase, mitochondrial	44.42192	40.58065	40.41979	40.23558	29.61241	36.19328
17246810	Lif	leukemia inhibitory factor	5.815487	6.287864	6.287864	6.310525	6.287864	5.952394
17310044	Lifr	leukemia inhibitory factor receptor	250.7594	191.727	198.0938	256.412	219.4289	219.3316
17331088	Lnp1	leukemia NUP98 fusion partner 1	5.338965	4.746256	5.393194	7.719728	5.854111	5.522265
17308928	Lect1	leukocyte cell derived chemotaxin 1	4.572268	4.73978	5.472765	5.85407	5.561215	5.46098
17293020	Lect2	leukocyte cell-derived chemotaxin 2	114.9275	90.20403	86.0856	113.167	122.428	182.7262
17473292	Lilra5	leukocyte immunoglobulin-like receptor, subfamily A (with TM domain), member !	9.487908	10.67164	12.12867	9.490473	10.14587	10.71055
17233226	Lilrb4a	leukocyte immunoglobulin-like receptor, subfamily B, member 4A	55.93774	82.62807	112.4446	69.49465	64.12078	51.54284
17233210	Lilr4b	leukocyte immunoglobulin-like receptor, subfamily B, member 4B	3.592505	6.385148	9.766692	5.825452	4.681984	5.532724
17485545	Leng1	leukocyte receptor cluster (LRC) member 1	14.31236	19.16325	15.96322	16.45974	21.34254	18.82033
17473269	Leng8	leukocyte receptor cluster (LRC) member 8	49.18341	67.95086	71.01737	58.78556	61.84907	63.15303
17485702	Leng9	leukocyte receptor cluster (LRC) member 9	21.20482	11.98934	11.50102	10.32207	7.595527	7.7633
17344303	Lst1	leukocyte specific transcript 1	5.730655	7.169189	8.055868	5.851514	5.804292	7.169189
17389938	Ltk	leukocyte tyrosine kinase	9.783699	10.01673	9.875816	11.39793	9.489881	11.45353
17485673	Lair1	leukocyte-associated Ig-like receptor 1	17.91554	20.68639	18.57744	17.18729	19.72154	15.0456
17236531	Lta4h	leukotriene A4 hydrolase	69.21391	56.62707	60.76126	66.1803	56.02233	56.49103
17300666	Ltb4r1	leukotriene B4 receptor 1	4.053927	4.464938	4.22002	4.79018	4.084105	5.061482
17300663	Ltb4r2	leukotriene B4 receptor 2	13.39423	13.67217	12.33358	17.20183	12.4416	13.24238
17262316	Ltc4s	leukotriene C4 synthase	6.992896	6.288169	9.033907	7.277283	7.277283	7.277283
17357872	Lpxn	leupaxin	9.205525	9.685993	10.17347	8.075883	8.184986	7.798572
17444106	Lfng	LFNG O-fucosylpeptide 3-beta-N-acetylglucosaminyltransferase	27.96034	30.32257	36.43821	33.96013	26.45026	31.30302
17359436	Lcor	ligand dependent nuclear receptor corepressor	84.28009	102.4496	83.57387	82.60401	70.14848	76.93456
17359449	Lcor	ligand dependent nuclear receptor corepressor	19.87744	17.56949	21.93344	20.20541	17.48057	16.91663
17359454	Lcor	ligand dependent nuclear receptor corepressor	7.12026	8.51685	9.403905	8.51685	7.545204	8.934333
17447940	Lcorl	ligand dependent nuclear receptor corepressor-like	25.33816	19.28143	21.73533	18.24678	22.31188	22.21481
17401514	Lrif1	ligand dependent nuclear receptor interacting factor 1	28.74024	24.9324	27.16825	28.29451	26.27812	26.56104
17448880	Lnx1	ligand of numb-protein X 1	7.531776	7.698994	5.879445	7.644012	8.166116	7.320438
17455281	Lnx2	ligand of numb-protein X 2	73.98444	50.335	42.01871	46.87153	42.97274	34.52525
17473856	Lig1	ligase I, DNA, ATP-dependent	20.38082	13.90787	18.326	12.8975	14.63418	16.22481
17254093	Lig3	ligase III, DNA, ATP-dependent	51.34885	36.9228	40.98829	49.16126	52.26235	40.47474
17507355	Lig4	ligase IV, DNA, ATP-dependent	14.01918	11.50644	11.93387	12.61904	9.098634	11.22388
17510586	Large	like-glycosyltransferase	17.34611	17.53809	18.49649	18.16312	12.75508	11.04935
17437887	Limch1	LIM and calponin homology domains 1	5.026869	5.617429	5.730973	4.553134	6.05023	7.198468
17461477	Lmcd1	LIM and cysteine-rich domains 1	5.411843	7.237091	7.370027	6.767486	7.131967	7.186703
17349209	Lims2	LIM and senescent cell antigen like domains 2	106.2943	116.3083	143.0713	123.7153	107.7064	106.6611
17233437	Lims1	LIM and senescent cell antigen-like domains 1	21.69243	25.07834	24.9921	21.62428	17.368	28.49743
17255877	Lasp1	LIM and SH3 protein 1	29.20695	28.55636	31.8107	32.42872	33.63334	30.63736
17321645	Lima1	LIM domain and actin binding 1	87.71429	90.99151	97.52499	84.00481	93.45112	78.92498
17365314	Ldb1	LIM domain binding 1	63.24984	80.03838	74.12252	78.35318	82.41471	66.23308
17447884	Ldb2	LIM domain binding 2	19.60813	21.29481	19.11496	21.29481	19.89315	21.29481
17305070	Ldb3	LIM domain binding 3	4.464504	6.041932	5.19297	4.82497	7.477417	5.190595
17270730	Limd2	LIM domain containing 2	31.02286	29.34027	31.34732	31.29434	25.72537	23.15802
17324451	Lpp	LIM domain containing preferred translocation partner in lipoma	54.62805	66.69885	83.31648	62.71071	74.9294	65.40368
17329445	Lppos	LIM domain containing preferred translocation partner in lipoma, opposite strand	15.1959	17.91828	20.20317	20.7059	23.69449	21.66133

17494842	Lmo1	LIM domain only 1	3.998669	3.585464	3.701936	4.04817	3.321235	3.017513
17373781	Lmo2	LIM domain only 2	22.76491	22.13627	26.09732	23.99746	23.2389	26.2834
17472288	Lmo3	LIM domain only 3	3.884772	3.100197	3.954568	3.726858	3.313142	4.431208
17410809	Lmo4	LIM domain only 4	40.12891	39.90368	40.12891	40.48653	37.84734	37.49666
17302429	Lmo7	LIM domain only 7	21.15989	31.55048	40.52415	32.08907	26.34414	30.41224
17523597	Limd1	LIM domains containing 1	51.64834	44.60953	45.96105	46.5949	40.55084	41.7302
17254458	Lhx1os	LIM homeobox 1, opposite strand	5.380424	4.636544	4.511259	5.559519	4.49724	4.39941
17267039	Lhx1	LIM homeobox protein 1	6.508161	10.30862	11.75221	10.04733	10.07522	9.161349
17370439	Lhx2	LIM homeobox protein 2	18.94598	19.06269	20.30327	17.06443	18.2835	20.22153
17382851	Lhx3	LIM homeobox protein 3	4.991938	4.771623	4.813813	4.813813	4.872087	5.575186
17228398	Lhx4	LIM homeobox protein 4	6.992896	6.992896	6.992896	6.992896	6.992896	6.992896
17441652	Lhx5	LIM homeobox protein 5	4.82273	4.360852	4.595176	5.856424	3.767298	4.435141
17384533	Lhx6	LIM homeobox protein 6	21.34432	22.89467	25.32359	24.2172	26.99967	19.34247
17411338	Lhx8	LIM homeobox protein 8	4.374597	4.350014	4.653932	4.161374	4.477613	4.74879
17227589	Lhx9	LIM homeobox protein 9	5.851149	6.109058	6.508567	7.419593	5.022759	5.907305
17219127	Lmx1a	LIM homeobox transcription factor 1 alpha	5.137441	5.586714	4.473798	5.008297	4.502761	4.886969
17384190	Lmx1b	LIM homeobox transcription factor 1 beta	29.40175	27.65982	29.02532	35.34098	31.452	24.50527
17259763	Limk2	LIM motif-containing protein kinase 2	7.671241	9.2989	8.983079	7.724117	5.703391	10.80907
17386639	Lnp	limb and neural patterns	11.45448	9.813107	9.215037	9.696136	9.307605	10.15156
17333686	Lix1	limb expression 1 homolog (chicken)	3.462322	3.957584	4.661163	4.553702	3.32363	3.400177
17446587	Lmbr1	limb region 1	10.97303	15.59963	17.20863	15.23914	13.8301	14.12521
17321440	Lmbr1l	limb region 1 like	14.05849	16.49938	19.51417	15.37623	15.27415	19.51417
17339554	Lbh	limb-bud and heart	45.63599	45.2972	56.8473	41.84316	45.2972	32.68145
17325671	Lsamp	limbic system-associated membrane protein	4.804113	6.140687	5.423898	5.20627	4.959621	6.695074
17453430	Limk1	LIM-domain containing, protein kinase	28.62552	27.69685	29.64774	31.91715	18.98516	26.10909
17431166	Lin28a	lin-28 homolog A (C. elegans)	10.21898	12.16446	10.16987	9.488237	4.644618	11.00304
17240670	Lin28b	lin-28 homolog B (C. elegans)	3.725619	4.398648	4.068875	3.515403	4.348537	4.46005
17489138	Lin37	lin-37 homolog (C. elegans)	35.09005	35.90816	44.07962	37.66232	37.14767	37.56117
17277270	Lin52	lin-52 homolog (C. elegans)	32.14918	35.53429	30.5411	33.36122	32.83244	34.37291
17450098	Lin54	lin-54 homolog (C. elegans)	31.06418	31.06418	28.01874	27.47124	31.95656	31.74814
17237132	Lin7a	lin-7 homolog A (C. elegans)	50.01469	41.83011	50.19494	46.20061	49.24618	47.91382
17490785	Lin7b	lin-7 homolog B (C. elegans)	6.992896	13.61391	9.66527	9.706627	9.706627	8.128787
17374089	Lin7c	lin-7 homolog C (C. elegans)	91.2492	82.72492	73.8282	74.57447	71.55149	81.55888
17220148	Lin9	lin-9 homolog (C. elegans)	11.84799	9.464852	12.25935	12.67664	14.41841	11.89844
17415702	L1td1	LINE-1 type transposase domain containing 1	5.429992	7.907929	7.902943	6.683158	7.072057	8.175517
17479012	Lins1	lines homolog (Drosophila) 1	11.35307	7.901355	11.13176	10.01604	9.312122	8.0216
17496150	Lat	linker for activation of T cells	6.149035	8.794003	9.014203	6.369632	6.992896	7.467214
17453406	Lat2	linker for activation of T cells family, member 2	13.45304	12.39237	14.10886	15.521	17.56303	15.17379
17334697	Lmf1	lipase maturation factor 1	104.1905	109.7228	111.307	102.1801	97.7944	107.3055
17320457	Lmf2	lipase maturation factor 2	51.70027	59.22523	71.26294	59.0936	48.49049	51.97983
17355443	Lipg	lipase, endothelial	25.74928	27.58056	22.96126	23.72457	25.67407	20.77231
17358737	Lipk	lipase, family member K	3.514867	3.780717	3.040861	3.481478	3.000993	3.422333
17358764	Lipm	lipase, family member M	3.906563	4.416336	3.968214	4.238313	4.742113	4.347391
17358749	Lipn	lipase, family member N	2.580964	3.361325	2.471505	2.570912	4.141777	2.7787
17358724	Lipf	lipase, gastric	3.588523	3.257082	3.39175	3.139729	3.485535	3.450009
17528629	Lipc	lipase, hepatic	211.5522	174.1661	172.428	191.6566	154.8567	173.1826
17487927	Lipe	lipase, hormone sensitive	18.72015	21.87347	28.62314	20.41914	22.24023	20.242
17329243	Liph	lipase, member H	3.711545	5.284401	3.843685	4.254354	4.354026	4.09465
17331518	Lipi	lipase, member I	2.705078	3.518352	3.182322	3.287612	2.626006	2.689727
17364026	Lipo1	lipase, member O1	9.426943	10.23025	8.636879	8.149096	6.70822	6.890124
17273899	Ldah	lipid droplet associated hydrolase	164.6296	134.9948	107.8334	116.1463	85.35537	110.4822
17280062	Lpin1	lipin 1	109.3182	165.1691	195.9049	210.2191	330.0498	319.3158
17339434	Lpin2	lipin 2	296.9076	414.558	439.1226	361.7903	406.1334	383.1778
17379077	Lpin3	lipin 3	12.12262	11.01212	10.86465	13.26148	10.51575	11.0851
17368154	Lcn10	lipocalin 10	4.120387	6.030928	4.975357	6.343592	6.030928	5.917632
17368193	Lcn11	lipocalin 11	6.405095	7.113157	6.801722	6.801722	6.377009	6.293707
17382603	Lcn12	lipocalin 12	11.46163	10.6016	8.84468	9.042615	9.943061	6.77927
17383892	Lcn2	lipocalin 2	134.9845	302.5689	484.8888	117.7701	349.1831	290.0685
17368184	Lcn3	lipocalin 3	3.115341	2.931522	3.891618	3.161677	3.84635	3.345287
17383131	Lcn4	lipocalin 4	2.406006	2.846166	2.545025	2.521094	4.194416	3.050128
17368130	Lcn5	lipocalin 5	7.103992	7.183864	7.717855	7.747227	10.06301	9.197935
17368141	Lcn6	lipocalin 6	8.3399	8.499865	7.05101	5.964908	7.455422	7.517276
17368122	Lcn8	lipocalin 8	5.157171	5.812424	4.937532	4.793934	6.491897	5.853583
17368204	Lcn9	lipocalin 9	4.025171	4.031034	4.089686	4.189302	4.10653	4.761975
17437773	Lias	lipoic acid synthetase	77.20113	79.47009	73.91576	87.02753	70.9598	79.126
17489384	Lsr	lipolysis stimulated lipoprotein receptor	139.674	144.0624	163.3486	136.3263	139.3588	95.13999
17397511	Lhfp	lipoma HMGIC fusion partner	27.3144	26.87606	25.43988	24.14161	18.69344	19.31273
17545150	Lhfpl1	lipoma HMGIC fusion partner-like 1	5.040476	4.955223	4.062929	4.631887	4.063407	4.714027
17289196	Lhfpl2	lipoma HMGIC fusion partner-like 2	11.88566	9.504298	8.854679	8.949208	6.266196	9.404557
17435180	Lhfpl3	lipoma HMGIC fusion partner-like 3	2.865725	3.470179	2.973867	3.051186	2.757193	3.008616
17335357	Lhfpl5	lipoma HMGIC fusion partner-like 5	5.717522	4.932811	5.476067	5.053805	6.973534	4.787326
17469691	Lhfpl4	lipoma HMGIC fusion partner-like protein 4	5.671656	6.992896	6.649657	5.75757	6.599972	5.907141
17378827	Lbp	lipopolysaccharide binding protein	241.614	296.5188	302.397	237.9097	259.056	146.1707
17501633	Lpl	lipoprotein lipase	105.0943	134.7807	143.4585	93.22791	137.5975	114.719
17351963	Loxhd1	lipoxygenase homology domains 1	5.829613	6.173463	4.465371	5.829613	5.829613	6.211579
17480636	Lipt2	lipoyl(octanoyl) transferase 2 (putative)	12.99919	17.69684	17.67416	11.23003	13.44008	12.75526
17211987	Lipt1	lipoyltransferase 1	7.865781	11.46123	10.66876	7.776818	10.17848	9.863502
17211990	Lipt1	lipoyltransferase 1	11.04912	8.177841	10.13771	7.698994	6.965756	10.9431
17331731	Ltn1	listerin E3 ubiquitin protein ligase 1	55.07975	40.6592	38.81117	52.4947	49.33501	48.52412
17281721	Pygl	liver glycogen phosphorylase	243.5763	207.6414	208.8741	265.983	273.5555	286.9946
17262644	Leap2	liver-expressed antimicrobial peptide 2	18.01691	14.29194	11.15292	18.04879	19.01466	17.4155
17400622	Lix1l	Lix1-like	17.69684	18.05404	19.32491	17.69684	14.48824	17.69684
17395948	Lkaaear1	LKAAEAR motif containing 1 (IKAAEAR murine motif)	3.787485	3.421953	3.787485	4.297447	3.787485	3.692429
17237665	Llph	LLP homolog, long-term synaptic facilitation (Aplysia)	14.92811	16.42703	13.2983	14.621	12.80522	17.91045

17548354	Llph-ps1	LLP homolog, pseudogene 1	79.51913	95.46632	89.16403	87.78971	103.549	106.2994
17540325	Llph-ps2	LLP homolog, pseudogene 2	12.94022	8.248375	11.07228	15.37101	16.21559	11.86903
17211498	Lmbrd1	LMBR1 domain containing 1	176.2281	167.0632	168.21	146.7656	159.0923	171.104
17310201	Lmbrd2	LMBR1 domain containing 2	213.6684	172.428	149.3434	160.9267	161.8586	174.1166
17346317	Lonp1	lon peptidase 1, mitochondrial	135.1455	123.7796	125.612	126.118	116.2591	114.4402
17503507	Lonp2	lon peptidase 2, peroxisomal	560.7524	587.5672	493.0636	566.4795	462.7342	464.0576
17508787	Lonrf1	LON peptidase N-terminal domain and ring finger 1	13.76087	10.96147	14.41701	11.48971	10.90328	12.91289
17222495	Lonrf2	LON peptidase N-terminal domain and ring finger 2	3.478511	3.869105	4.245869	4.299712	4.185645	4.735117
17534074	Lonrf3	LON peptidase N-terminal domain and ring finger 3	11.40647	7.550384	8.640591	8.307878	8.11595	9.405991
17373778	Lincred1	long intergenic non-protein coding RNA of erythroid differentiation 1	4.123816	7.82326	5.986322	5.708177	5.283815	6.966625
17289382	Lncenc1	long non-coding RNA, embryonic stem cells expressed 1	4.228629	4.228629	4.604294	3.933463	5.188941	5.852812
17465608	Lncpint	long non-protein coding RNA, Trp53 induced transcript	69.75477	84.27075	83.61733	52.57154	47.66406	66.16608
17407378	Lor	loricrin	3.954321	4.801649	4.835215	4.627907	5.031854	5.331642
17515315	Ldlr	low density lipoprotein receptor	107.3442	70.22778	123.9737	67.97065	103.2115	75.69783
17431708	Ldlrad2	low density lipoprotein receptor A domain containing 2	9.402602	6.777109	8.48315	9.540865	6.398173	10.0962
17431350	Ldlrap1	low density lipoprotein receptor adaptor protein 1	52.3853	62.37604	62.05863	68.27095	61.81864	59.16943
17416440	Ldlrad1	low density lipoprotein receptor class A domain containing 1	15.35589	12.24516	12.75349	10.66824	13.32266	14.13314
17388705	Ldlrad3	low density lipoprotein receptor class A domain containing 3	13.80233	12.44177	16.77592	14.52111	17.06786	21.58414
17351559	Ldlrad4	low density lipoprotein receptor class A domain containing 4	20.79392	26.87327	21.86361	18.34506	19.79632	23.41303
17245923	Lrp1	low density lipoprotein receptor-related protein 1	494.0078	523.3165	508.2555	473.9858	521.3144	438.3988
17231522	Lrp11	low density lipoprotein receptor-related protein 11	18.41884	17.46349	13.575	18.07671	18.88947	14.40203
17386210	Lrp2	low density lipoprotein receptor-related protein 2	4.20927	3.834796	4.20927	4.11408	4.485752	4.20927
17489713	Lrp3	low density lipoprotein receptor-related protein 3	17.90611	14.97548	16.83393	17.64992	14.91332	18.44746
17373283	Lrp4	low density lipoprotein receptor-related protein 4	36.70844	46.4427	47.26204	35.5974	34.1466	42.48142
17360977	Lrp5	low density lipoprotein receptor-related protein 5	109.3758	101.8082	102.8844	111.4768	112.9061	91.43281
17471995	Lrp6	low density lipoprotein receptor-related protein 6	154.9974	196.9056	224.4748	194.5288	168.385	180.4281
17416540	Lrp8	low density lipoprotein receptor-related protein 8, apolipoprotein e receptor	5.849649	8.032394	7.448449	8.032394	9.52527	9.119913
17427958	Lrp8os1	low density lipoprotein receptor-related protein 8, apolipoprotein e receptor, opp	3.481985	3.445635	2.787516	3.521719	3.505232	4.440124
17427951	Lrp8os2	low density lipoprotein receptor-related protein 8, apolipoprotein e receptor, opp	8.843577	9.443424	8.561657	9.259344	10.30741	10.51422
17427948	Lrp8os3	low density lipoprotein receptor-related protein 8, apolipoprotein e receptor, opp	5.144426	6.070982	6.184342	4.577691	6.030928	6.42506
17447310	Lrpap1	low density lipoprotein receptor-related protein associated protein 1	40.03722	43.14406	31.97539	37.22784	30.29736	32.50185
17316754	Lrp12	low density lipoprotein-related protein 12	11.21292	11.05134	10.11848	12.20111	11.50086	8.420524
17384826	Lrp1b	low density lipoprotein-related protein 1B (deleted in tumors)	3.674251	3.674251	3.674251	3.684171	4.332711	3.674251
17300292	Lrp10	low-density lipoprotein receptor-related protein 10	85.55736	79.16111	76.20427	76.87007	73.5116	69.1741
17328124	Litaf	LPS-induced TN factor	33.9646	37.82767	41.37823	33.25064	35.69623	30.25601
17398655	Lrba	LPS-responsive beige-like anchor	63.00612	60.21162	61.71845	54.76415	56.58626	64.62946
17500957	Lrp2bp	Lrp2 binding protein	6.981563	7.097053	8.499865	8.316405	7.981445	8.822271
17357255	Lrrn4cl	LRRN4 C-terminal like	12.49665	11.41311	11.46433	10.6655	11.96311	13.35307
17500188	Lsm1	LSM1 homolog, U6 small nuclear RNA associated (S. cerevisiae)	23.68317	23.92555	22.34515	25.55954	22.62757	26.07093
17270071	Lsm12	LSM12 homolog (S. cerevisiae)	210.5881	207.6802	209.3249	186.3421	179.6344	179.1929
17489668	Lsm14a	LSM14 homolog A (SCD6, S. cerevisiae)	55.38487	63.00481	58.22795	57.56181	56.0033	55.77551
17380666	Lsm14b	LSM14 homolog B (SCD6, S. cerevisiae)	22.44201	25.36118	25.36118	26.30108	23.80017	27.50134
17336829	Lsm2	LSM2 homolog, U6 small nuclear RNA associated (S. cerevisiae)	15.33004	15.77152	21.31105	18.37331	22.3653	17.72373
17460933	Lsm3	LSM3 homolog, U6 small nuclear RNA associated (S. cerevisiae)	18.16312	21.20217	18.56972	21.82954	22.90054	21.80913
17501994	Lsm4	LSM4 homolog, U6 small nuclear RNA associated (S. cerevisiae)	55.37143	57.71762	59.58924	51.19	65.01993	60.89028
17467108	Lsm5	LSM5 homolog, U6 small nuclear RNA associated (S. cerevisiae)	6.662991	4.747638	6.837552	6.274715	12.8585	10.36638
17510707	Lsm6	LSM6 homolog, U6 small nuclear RNA associated (S. cerevisiae)	47.13543	49.01291	54.10012	60.22581	61.79679	58.01001
17234800	Lsm7	LSM7 homolog, U6 small nuclear RNA associated (S. cerevisiae)	37.8358	35.36107	44.0445	38.49769	40.66315	46.88193
17243117	Lsm7	LSM7 homolog, U6 small nuclear RNA associated (S. cerevisiae)	24.35625	22.75829	27.59414	29.53451	26.33884	31.91472
17456285	Lsm8	LSM8 homolog, U6 small nuclear RNA associated (S. cerevisiae)	11.58238	18.01042	16.18685	16.47824	18.37446	9.775361
17307638	Tdh	L-threonine dehydrogenase	9.907767	9.276175	8.054751	9.533923	9.05541	10.97501
17239371	Ltv1	LTV1 homolog (S. cerevisiae)	30.65308	29.01908	26.67192	30.70709	28.27412	25.673
17334904	Luc7l	Luc7 homolog (S. cerevisiae)-like	36.88213	28.62016	32.48113	31.82768	29.77707	30.36758
17457436	Luc7l2	LUC7-like 2 (S. cerevisiae)	109.0012	131.553	133.1289	126.8729	138.494	146.3116
17267822	Luc7l3	LUC7-like 3 (S. cerevisiae)	60.4437	90.95304	83.2224	81.26544	86.33418	95.49412
17236811	Lum	lumican	15.3854	25.30517	26.81745	18.00356	14.76869	12.75305
17477800	Lhb	luteinizing hormone beta	6.171709	5.310729	5.412293	6.066734	6.285162	5.998742
17347990	Lhcgr	luteinizing hormone/choriogonadotropin receptor	3.832989	4.25996	4.231121	3.634911	3.994736	4.105961
17437072	Lyar	Ly1 antibody reactive clone	19.24751	23.25743	20.92825	18.49649	21.82848	18.19323
17318013	Lynx1	Ly6/neurotoxin 1	9.731215	10.12058	8.915032	8.51685	5.991719	8.467875
17226435	Lypd1	Ly6/Plaur domain containing 1	8.440333	8.363113	7.190739	8.56991	7.129347	7.482083
17317994	Lypd2	Ly6/Plaur domain containing 2	5.246481	5.161463	5.004688	5.338965	6.230898	5.338965
17475054	Lypd3	Ly6/Plaur domain containing 3	7.916101	8.393951	9.095544	8.299992	9.765067	10.0651
17487779	Lypd4	Ly6/Plaur domain containing 4	6.808184	8.63628	7.820712	6.344076	7.39341	7.089825
17474934	Lypd5	Ly6/Plaur domain containing 5	4.87675	6.769034	5.803286	6.210373	6.262506	6.1429
17370705	Lypd6	LY6/PLAUR domain containing 6	5.290338	6.982337	4.20927	5.174968	5.271049	5.465563
17370691	Lypd6b	LY6/PLAUR domain containing 6B	3.874607	5.145888	3.898448	4.39496	3.984504	4.212393
17250044	Lypd8	LY6/PLAUR domain containing 8	3.541376	5.025266	3.911386	4.322542	2.897322	3.754654
17495097	Lyve1	lymphatic vessel endothelial hyaluronan receptor 1	104.2115	135.2861	145.8711	97.7822	73.65798	81.57358
17503208	Lyl1	lymphoblastomic leukemia 1	7.673741	9.117574	9.231595	10.56778	7.426127	10.5798
17318118	9030619P08Rik	lymphocyte antigen 6 complex pseudogene	18.61934	20.29384	32.82516	30.57287	50.90445	35.10221
17318083	Ly6a	lymphocyte antigen 6 complex, locus A	118.0857	199.3625	339.6229	206.1442	103.8408	158.4946
17318089	Ly6c1	lymphocyte antigen 6 complex, locus C1	3.436977	2.228712	2.792021	3.253494	5.768835	2.82353
17318100	Ly6c2	lymphocyte antigen 6 complex, locus C2	5.423409	6.287864	11.29021	5.423409	4.499485	5.69857
17318020	Ly6d	lymphocyte antigen 6 complex, locus D	44.37545	128.1562	166.3629	119.0943	54.53498	44.72315
17312209	Ly6e	lymphocyte antigen 6 complex, locus E	715.7143	662.0104	808.4698	693.0665	801.6618	640.4801
17312229	Ly6f	lymphocyte antigen 6 complex, locus F	2.823667	2.77664	2.713923	2.217478	2.688069	3.014398
17312219	Ly6g	lymphocyte antigen 6 complex, locus G	3.476824	2.591289	2.788308	3.062522	2.720968	2.843623
17344216	Ly6g5b	lymphocyte antigen 6 complex, locus G5B	3.570386	6.119526	5.653287	5.151277	4.628709	7.13805
17336965	Ly6g5c	lymphocyte antigen 6 complex, locus G5C	5.463479	6.380281	6.528538	7.125889	7.362063	5.680154
17336921	Ly6g6c	lymphocyte antigen 6 complex, locus G6C	9.123011	9.123011	6.435445	8.282349	8.537833	9.904266
17344200	Ly6g6d	lymphocyte antigen 6 complex, locus G6D	4.712851	5.386656	5.537482	6.132732	5.651642	5.320383
17336929	Ly6g6e	lymphocyte antigen 6 complex, locus G6E	18.03928	18.81329	17.7096	19.47026	12.93027	14.92408
17344208	Ly6g6f	lymphocyte antigen 6 complex, locus G6F	7.169189	7.441946	6.241315	8.758963	10.06671	7.169189

17318123	Ly6h	lymphocyte antigen 6 complex, locus H	5.177946	4.681043	4.760457	4.705767	4.365812	5.610036
17318076	Ly6i	lymphocyte antigen 6 complex, locus I	2.982061	2.683638	3.299643	3.137271	3.68144	3.643008
17318029	Ly6k	lymphocyte antigen 6 complex, locus K	13.01984	13.17079	13.91192	13.91192	14.49848	14.96386
17385574	Ly75	lymphocyte antigen 75	6.992896	7.042072	5.960651	6.171709	7.682895	5.212011
17286587	Ly86	lymphocyte antigen 86	49.58837	49.66818	57.77086	42.89864	45.80204	42.06767
17229767	Ly9	lymphocyte antigen 9	10.8706	10.76958	18.00044	11.89126	12.87651	12.21041
17211286	Ly96	lymphocyte antigen 96	57.74083	75.52959	100.9411	63.40479	55.42443	51.78133
17301968	Lcp1	lymphocyte cytosolic protein 1	158.7563	141.5551	144.1873	123.0849	127.6261	121.8498
17248380	Lcp2	lymphocyte cytosolic protein 2	64.82148	70.30961	84.77459	55.40292	41.64525	55.11259
17427799	Lexm	lymphocyte expansion molecule	39.88211	43.21949	43.46674	39.61514	37.22552	31.23908
17427802	Lexm	lymphocyte expansion molecule	5.098914	5.120306	5.120306	5.120306	4.512697	4.480718
17430413	Lck	lymphocyte protein tyrosine kinase	5.765117	5.332973	5.332973	5.913614	5.416196	5.703035
17485226	Lsp1	lymphocyte specific 1	16.0462	15.11459	18.4173	17.88552	12.79404	16.62428
17227025	Lax1	lymphocyte transmembrane adaptor 1	4.975288	4.61471	4.149622	4.61471	5.423108	4.046627
17470976	Lag3	lymphocyte-activation gene 3	5.974674	5.848757	6.820323	7.324702	4.525979	6.260032
17402662	Lef1	lymphoid enhancer binding factor 1	5.976124	6.754037	6.318184	5.976124	6.135964	5.617312
17464210	Lrmp	lymphoid-restricted membrane protein	7.85984	9.208014	8.997287	8.298324	9.187676	9.534386
17344316	Lta	lymphotoxin A	5.650819	6.417271	5.733078	5.386134	6.44978	5.386134
17337024	Ltb	lymphotoxin B	9.885816	10.5291	11.33309	10.49543	9.217401	10.29855
17471094	Ltbr	lymphotoxin B receptor	70.09453	67.8652	73.30451	61.50407	68.77441	69.60842
17482355	Lyrm1	LYR motif containing 1	9.003775	9.182646	7.62327	9.003775	10.48228	9.003775
17412534	Lyrm2	LYR motif containing 2	17.6273	14.88616	15.14122	19.42873	23.49464	25.20521
17291866	Lyrm4	LYR motif containing 4	31.96697	36.96121	31.47228	33.87174	34.32315	36.22767
17464238	Lyrm5	LYR motif containing 5	86.45096	101.7892	91.35299	112.0944	92.09239	120.4491
17262848	Lyrm7	LYR motif containing 7	5.163717	4.271136	6.075445	7.999168	4.482365	7.786095
17249774	Lyrm7os	LYR motif containing 7, opposite strand	2.98233	3.078527	2.704028	2.837971	2.537609	2.849344
17253700	Lyrm9	LYR motif containing 9	10.3745	11.99491	11.9052	10.58185	7.919833	8.704853
17431562	Kdm1a	lysine (K)-specific demethylase 1A	35.07862	30.86308	29.42499	33.80583	30.56122	31.37123
17287041	Kdm1b	lysine (K)-specific demethylase 1B	51.01431	45.58477	50.81421	51.39728	56.30261	57.0676
17361250	Kdm2a	lysine (K)-specific demethylase 2A	93.11812	98.67618	86.67236	90.1909	97.15592	91.1727
17452498	Kdm2b	lysine (K)-specific demethylase 2B	12.35189	12.25128	11.82272	11.06131	10.46667	12.58925
17467624	Kdm3a	lysine (K)-specific demethylase 3A	46.21855	57.77086	54.23003	42.9936	59.11408	47.01796
17429026	Kdm4a	lysine (K)-specific demethylase 4A	65.65213	62.84125	63.43249	74.85569	72.16113	67.36092
17338767	Kdm4b	lysine (K)-specific demethylase 4B	20.89941	16.46785	20.37616	20.29525	21.63987	17.30947
17414935	Kdm4c	lysine (K)-specific demethylase 4C	27.77664	28.30608	27.38151	30.21138	31.14201	28.86721
17524022	Kdm4d	lysine (K)-specific demethylase 4D	4.754389	3.883991	3.892697	3.547198	3.305847	3.363516
17462318	Kdm5a	lysine (K)-specific demethylase 5A	34.61631	38.65599	37.20101	51.11058	44.36684	38.86528
17217500	Kdm5b	lysine (K)-specific demethylase 5B	41.26881	48.53185	49.73226	52.62075	37.15179	48.52681
17538912	Kdm5c	lysine (K)-specific demethylase 5C	76.69869	72.16413	71.34399	65.14443	71.42761	66.96986
17546287	Kdm5d	lysine (K)-specific demethylase 5D	15.21276	12.8077	15.28326	14.58496	17.94313	19.94368
17533498	Kdm6a	lysine (K)-specific demethylase 6A	57.16103	51.67663	39.87907	49.88002	38.36104	40.17483
17466075	Kdm7a	lysine (K)-specific demethylase 7A	33.84967	39.28046	40.2473	31.29434	34.90109	37.99322
17482932	Kdm8	lysine (K)-specific demethylase 8	10.18829	9.053339	7.672145	9.361307	9.788718	9.361307
17526382	Kmt2a	lysine (K)-specific methyltransferase 2A	34.3329	40.87991	42.42198	33.79458	32.12379	33.9858
17489161	Kmt2b	lysine (K)-specific methyltransferase 2B	33.63823	30.27028	27.89897	29.22781	32.1445	30.15886
17446354	Kmt2c	lysine (K)-specific methyltransferase 2C	41.70302	48.16122	49.09384	48.0775	49.09384	53.60033
17321365	Kmt2d	lysine (K)-specific methyltransferase 2D	41.70244	52.148	56.11054	45.37859	47.68521	53.57582
17435189	Kmt2e	lysine (K)-specific methyltransferase 2E	89.11213	144.9941	137.8609	108.3428	126.9072	123.3309
17495598	Knop1	lysine rich nucleolar protein 1	15.4627	17.9821	14.00218	14.58365	14.79554	16.10604
17459463	Krcc1	lysine-rich coiled-coil 1	54.91011	48.06018	50.69249	58.48643	38.72759	49.84511
17283082	Lysmd1	LysM, putative peptidoglycan-binding, domain containing 1	25.63441	22.66666	29.50444	29.7845	29.64301	29.64301
17400244	Lysmd1	LysM, putative peptidoglycan-binding, domain containing 1	19.48673	21.24189	19.64214	23.88777	22.22159	17.43313
17519533	Lysmd2	LysM, putative peptidoglycan-binding, domain containing 2	12.59291	9.999112	14.1028	9.891025	8.732835	14.41111
17288898	Lysmd3	LysM, putative peptidoglycan-binding, domain containing 3	104.8621	62.19902	65.71314	63.4426	69.29455	60.99208
17479069	Lysmd4	LysM, putative peptidoglycan-binding, domain containing 4	16.7122	16.32646	15.50455	18.27108	18.9749	21.19879
17339561	Lclat1	lysocardiolipin acyltransferase 1	120.3123	115.9332	119.8611	98.60644	84.02344	98.18019
17425686	Lpar1	lysophosphatidic acid receptor 1	7.495424	7.830802	8.187596	9.448924	6.688997	9.310251
17501748	Lpar2	lysophosphatidic acid receptor 2	6.295845	6.762328	4.874519	7.619995	5.431309	5.847338
17403509	Lpar3	lysophosphatidic acid receptor 3	3.638415	4.361154	3.2246	4.09411	4.782318	3.6769
17537112	Lpar4	lysophosphatidic acid receptor 4	7.076078	7.019557	5.638143	6.106814	4.703419	6.84144
17463051	Lpar5	lysophosphatidic acid receptor 5	17.42854	11.72869	16.83206	13.86033	12.37091	10.09809
17301886	Lpar6	lysophosphatidic acid receptor 6	71.51629	37.03147	65.57618	61.0276	51.79963	55.90634
17288467	Lpcat1	lysophosphatidylcholine acyltransferase 1	11.2641	9.909759	10.01701	10.21141	7.352883	7.991188
17503841	Lpcat2	lysophosphatidylcholine acyltransferase 2	7.612393	9.426224	12.68033	6.807287	8.933652	7.077305
17440037	Lpcat2b	lysophosphatidylcholine acyltransferase 2B	4.759533	4.902134	4.353422	3.867791	4.519302	6.243002
17462905	Lpcat3	lysophosphatidylcholine acyltransferase 3	255.1108	315.7634	308.8473	250.544	292.6596	277.8341
17374218	Lpcat4	lysophosphatidylcholine acyltransferase 4	6.054257	7.79868	7.65329	5.844299	6.347815	6.347815
17220833	Lpgat1	lysophosphatidylglycerol acyltransferase 1	172.9834	182.8251	190.1257	181.3056	197.2512	147.7117
17210855	Lypla1	lysophospholipase 1	301.286	287.6678	281.82	279.8733	257.3557	288.8407
17431502	Lypla2	lysophospholipase 2	73.58552	84.09453	88.70171	84.15925	84.09453	84.09453
17230823	Lyplal1	lysophospholipase-like 1	73.07875	69.52115	73.46677	82.39643	70.16939	70.78099
17364114	Lipa	lysosomal acid lipase A	770.0402	742.5683	689.6928	670.4379	611.4709	641.1997
17285267	Lyst	lysosomal trafficking regulator	53.89427	50.23009	50.12749	44.25627	41.92474	48.20297
17499279	Lamp1	lysosomal-associated membrane protein 1	865.83	894.5945	866.5024	818.7675	851.2741	803.6592
17541173	Lamp2	lysosomal-associated membrane protein 2	755.9201	780.5731	717.403	709.2891	678.4835	704.0285
17329046	Lamp3	lysosomal-associated membrane protein 3	4.11916	4.985022	3.12003	3.269795	5.130004	2.54792
17376783	Lamp5	lysosomal-associated membrane protein family, member 5	4.268917	4.681043	4.681043	4.563561	4.753269	4.617654
17273961	Laptm4a	lysosomal-associated protein transmembrane 4A	223.1732	253.4048	248.3636	207.3623	216.2026	216.495
17310912	Laptm4b	lysosomal-associated protein transmembrane 4B	46.81276	59.74226	62.00918	55.73455	43.46493	58.79331
17419287	Laptm5	lysosomal-associated protein transmembrane 5	11.20476	10.72734	16.9576	10.28557	9.813855	8.684483
17245231	Lyz1	lysozyme 1	4.400386	4.708084	4.269183	6.753803	7.936924	3.892454
17245223	Lyz2	lysozyme 2	86.74989	112.0105	121.1987	133.7338	127.4617	128.0302
17222420	Lyg1	lysozyme G-like 1	2.734866	2.693608	2.471745	2.602648	2.769566	2.172988
17222411	Lyg2	lysozyme G-like 2	5.044391	4.032767	4.214642	4.565333	4.079126	4.460885

17348113	Lyzl1	lysozyme-like 1	3.506496	3.110249	3.779355	3.405815	3.839664	4.238812
17532341	Lyzl4	lysozyme-like 4	3.946654	3.883991	5.033668	3.44812	3.733322	5.177839
17523303	Lyzl4os	lysozyme-like 4, opposite strand	9.18061	6.541675	6.287864	5.225684	6.135283	5.807914
17270558	Lyzl6	lysozyme-like 6	6.035361	5.908738	4.952304	5.142821	4.894122	4.557871
17354367	Lox	lysyl oxidase	4.388293	3.488991	3.703322	3.703322	3.828953	4.214145
17527694	Loxl1	lysyl oxidase-like 1	8.15226	8.5463	11.34984	11.9175	9.77936	9.539608
17301670	Loxl2	lysyl oxidase-like 2	17.43422	15.1999	15.63178	17.7445	12.70478	14.82561
17459870	Loxl3	lysyl oxidase-like 3	10.50031	11.10194	12.7656	12.7687	8.723155	11.85817
17364851	Loxl4	lysyl oxidase-like 4	11.71277	12.31479	13.4774	15.38476	10.53947	11.31222
17513209	Kars	lysyl-tRNA synthetase	83.82392	80.55488	81	70.84431	74.64222	71.2101
17513389	Mphosph6	M phase phosphoprotein 6	16.42407	12.51711	15.68681	16.83685	17.29496	18.76991
17529046	Mb21d1	Mab-21 domain containing 1	3.058533	3.661615	3.577793	4.076356	3.853742	3.339703
17329527	Mb21d2	Mab-21 domain containing 2	38.67662	34.34052	37.65997	40.30676	31.49847	29.97047
17397740	Mab21l1	mab-21-like 1 (C. elegans)	5.455721	3.613583	3.506715	3.479717	4.253233	4.08153
17406456	Mab21l2	mab-21-like 2 (C. elegans)	5.919847	6.012271	4.647678	6.109439	6.725866	4.312038
17408536	Mab21l3	mab-21-like 3 (C. elegans)	7.405463	11.0742	9.881911	8.701596	8.701596	10.45253
17357004	Macrod1	MACRO domain containing 1	45.32484	48.51066	52.09453	54.46774	46.16668	61.34994
17376933	Macrod2	MACRO domain containing 2	4.224908	5.379082	4.800385	4.39624	5.029099	4.948599
17392268	Macrod2os2	MACRO domain containing 2, opposite strand 2 [Source:MGI Symbol;Acc:MGI:365	4.243427	4.888019	4.214379	4.686562	3.555568	4.238959
17436427	Maea	macrophage erythroblast attacher	68.24872	68.45576	78.34069	67.8619	70.84038	56.37056
17357810	Mpeg1	macrophage expressed gene 1	121.1231	104.2678	132.8496	120.8271	157.3017	130.3982
17251959	Mgl2	macrophage galactose N-acetyl-galactosamine specific lectin 2	5.413006	4.344921	4.002025	4.707757	4.521621	3.859115
17241962	Mif	macrophage migration inhibitory factor	186.7714	222.9197	257.5734	209.0277	195.0987	164.2261
17226327	Marco	macrophage receptor with collagenous structure	72.67262	116.0393	208.8264	60.43699	57.35073	30.4405
17508850	Msr1	macrophage scavenger receptor 1	39.94713	40.63835	43.81704	28.71634	32.28227	23.35339
17521625	Mst1	macrophage stimulating 1 (hepatocyte growth factor-like)	147.8705	132.9611	134.9003	152.2364	182.1067	151.8318
17521485	Mst1r	macrophage stimulating 1 receptor (c-met-related tyrosine kinase)	7.743898	9.546487	8.409442	7.448036	7.311513	7.637074
17454521	Mad1l1	MAD1 mitotic arrest deficient 1-like 1	23.69449	19.62853	20.61782	20.61782	20.61782	20.29891
17459236	Mad2l1	MAD2 mitotic arrest deficient-like 1	8.328115	8.87914	9.595846	11.02846	6.039085	8.226165
17421571	Mad2l2	MAD2 mitotic arrest deficient-like 2	48.91279	41.4207	50.36955	47.49389	56.52276	67.644
17345315	Mad2l1bp	MAD2L1 binding protein	12.35189	12.79555	11.54439	13.39368	10.87301	12.28725
17229315	Mael	maelstrom homolog (Drosophila)	2.558166	3.345264	3.345264	2.953407	3.009972	3.09616
17351687	Mro	maestro	3.894991	4.439262	4.20927	4.327879	3.979563	4.228864
17312427	Mroh1	maestro heat-like repeat family member 1	35.70316	39.01697	36.96121	29.33355	34.85926	36.87906
17215462	Mroh2a	maestro heat-like repeat family member 2A	4.721843	5.56106	5.235982	5.927074	5.56106	5.56106
17309853	Mroh2b	maestro heat-like repeat family member 2B	5.60817	4.722727	4.630603	4.694951	4.24902	4.826308
17227436	Mroh3	maestro heat-like repeat family member 3	5.979356	7.173763	5.879853	5.829087	5.447333	5.732323
17317952	Mroh4	maestro heat-like repeat family member 4	3.766593	4.402521	4.042646	4.20927	3.663163	4.430532
17317925	Mroh5	maestro heat-like repeat family member 5	7.470009	6.760032	6.080837	7.733706	6.072582	6.638328
17317930	Mroh5	maestro heat-like repeat family member 5	11.00128	10.55409	10.66876	12.41162	8.469754	11.3437
17318179	Mroh6	maestro heat-like repeat family member 6	8.467112	8.467112	9.241391	8.254667	7.935109	8.467112
17427833	Mroh7	maestro heat-like repeat family member 7	4.401362	5.187898	4.64742	5.614704	5.370513	7.040803
17393709	Mroh8	maestro heat-like repeat family member 8	4.742968	6.655652	4.327579	4.694951	4.524192	4.886969
17229070	Mroh9	maestro heat-like repeat family member 9	2.708286	2.941618	3.009972	3.009972	3.713836	3.009972
17312405	Maf1	MAF1 homolog (S. cerevisiae)	17.81055	23.11248	21.00528	23.97402	22.36127	22.44932
17539888	Magix	MAGI family member, X-linked	18.87415	17.03606	17.38041	20.49685	23.66085	22.12185
17543974	Magt1	magnesium transporter 1	239.6807	151.2069	155.9825	155.164	170.4849	185.6987
17306775	Mdp1	magnesium-dependent phosphatase 1	27.77933	28.34181	27.13758	33.1765	31.51594	34.29485
17416566	Magoh	mago homolog, exon junction complex core component	34.55063	28.89865	27.55247	27.1903	27.70319	28.21323
17471840	Magohb	mago-nashi homolog B (Drosophila)	5.8219	5.422582	4.20927	3.579728	3.716437	3.872298
17322600	Mgrn1	mahogunin, ring finger 1	75.80494	87.60553	97.5304	82.18311	87.16578	98.45892
17476622	LOC102635992	major allergen I polypeptide chain 1-like	2.332117	2.332117	2.002289	2.334332	2.279157	2.059123
17476643	LOC102637163	major allergen I polypeptide chain 1-like	5.607548	6.160767	5.922762	5.548009	6.995562	5.170128
17398180	Mfsd1	major facilitator superfamily domain containing 1	190.9935	188.4686	157.4391	173.9839	179.4502	162.1123
17447269	Mfsd10	major facilitator superfamily domain containing 10	25.99136	24.97928	24.99989	19.43399	32.74789	25.36118
17258664	Mfsd11	major facilitator superfamily domain containing 11	33.91661	33.05531	29.38565	27.85028	25.516	31.45963
17235701	Mfsd12	major facilitator superfamily domain containing 12	21.49471	21.49471	25.44517	26.51818	18.19197	20.56558
17429632	Mfsd2a	major facilitator superfamily domain containing 2A	271.4288	414.1444	323.9147	237.3628	215.9974	248.9496
17279864	Mfsd2b	major facilitator superfamily domain containing 2B	7.287682	7.143098	7.891448	6.213129	6.833146	7.124309
17312581	Mfsd3	major facilitator superfamily domain containing 3	16.14371	17.23752	21.94423	17.6306	20.76612	19.6254
17226829	Mfsd4	major facilitator superfamily domain containing 4	8.467112	10.67778	7.903655	8.535348	7.048763	7.054041
17315349	Mfsd5	major facilitator superfamily domain containing 5	24.0933	29.1483	27.38398	35.52937	23.18291	17.10896
17222925	Mfsd6	major facilitator superfamily domain containing 6	42.57722	35.32138	34.49942	27.75643	26.66453	21.40936
17251354	Mfsd6l	major facilitator superfamily domain containing 6-like	7.738908	7.201613	7.73687	7.228368	6.26876	6.684316
17450748	Mfsd7a	major facilitator superfamily domain containing 7A	5.927197	10.39091	10.29905	12.71553	9.683711	10.34126
17231003	Mfsd7b	major facilitator superfamily domain containing 7B	11.64711	12.06612	12.17585	11.05058	12.49406	11.37709
17277411	Mfsd7c	major facilitator superfamily domain containing 7C	14.57749	19.01479	18.88344	19.10913	18.55196	20.33552
17404976	Mfsd8	major facilitator superfamily domain containing 8	35.71801	23.34659	23.06686	25.92299	19.07115	25.54342
17222606	Mfsd9	major facilitator superfamily domain containing 9	8.162607	9.361307	9.784038	7.701503	6.62927	7.867689
17228353	Mr1	major histocompatibility complex, class I-related	23.69449	30.05744	28.00262	23.69449	19.21978	16.97042
17238318	Mip	major intrinsic protein of lens fiber	5.254706	5.110307	5.289678	5.838145	5.110307	5.77868
17425941	Mup1	major urinary protein 1	6289.938	5658.877	5241.013	6510.037	5856.65	5575.402
17425954	Mup10	major urinary protein 10	1043.656	723.3149	692.5431	989.0365	567.6981	676.8911
17425978	Mup12	major urinary protein 12	3194.734	2866.332	2523.01	3204.914	2957.065	2620.107
17426000	Mup13	major urinary protein 13	6309.238	6326.755	5917.409	6549.822	6322.327	6194.362
17426011	Mup14	major urinary protein 14	6444.488	6197.626	5693.026	6790.678	6223.757	6001.303
17426022	Mup15	major urinary protein 15	6745.455	6310.287	5951.922	6686.243	6356.514	6033.168
17426032	Mup16	major urinary protein 16	4847.348	4466.317	4001.874	4905.383	4279.777	4175.548
17426043	Mup18	major urinary protein 18	6689.349	6205.363	5851.74	6597.94	6296.481	6148.718
17426062	Mup19	major urinary protein 19	6271.566	6262.617	5803.832	6813.782	6336.147	6129.909
17425900	Mup2	major urinary protein 2	4743.644	4398.97	4292.374	5166.602	4743.644	4654.273
17426086	Mup20	major urinary protein 20	4074.649	3131.526	1760.598	3802.625	3406.466	2537.145
17426108	Mup21	major urinary protein 21	94.31531	14.99334	3.139381	40.91421	30.40338	37.99374
17426097	Mup3	major urinary protein 3	6253.985	4157.412	2856.217	5159.015	5004.603	4090.808

17425878	Mup4	major urinary protein 4	3.462322	3.070301	2.271885	2.287164	2.782767	4.675109
17426076	Mup5	major urinary protein 5	5.931677	4.922291	5.354233	6.555883	9.310251	6.607205
17414497	Mup6	major urinary protein 6	9.917593	6.699948	3.427294	8.654257	6.058119	4.564162
17425888	Mup7	major urinary protein 7	6183.809	5636.563	5399.799	6623.05	6039.318	5825.879
17425915	Mup8	major urinary protein 8	6191.572	6013.545	5469.601	6583.55	6066.674	5828.504
17425926	Mup9	major urinary protein 9	326.8557	282.0232	201.5467	415.1733	245.2314	228.3875
17414512	Mup-ps12	major urinary protein, pseudogene 12	33.14892	47.26663	60.8831	82.9477	60.14946	91.65489
17414520	Mup-ps16	major urinary protein, pseudogene 16	63.8436	66.59814	81.00955	126.1704	110.5067	131.7766
17496514	Mvp	major vault protein	64.82552	75.49452	71.40088	56.35259	53.77635	55.20052
17508593	Mak16	MAK16 homolog (S. cerevisiae)	32.49194	25.22776	22.69716	22.29163	25.12793	22.94328
17469887	Mkrn2os	makorin, ring finger protein 2, opposite strand	94.09656	97.23205	112.1612	99.24758	73.92959	94.09656
17466119	Mkrn1	makorin, ring finger protein, 1	13.08852	13.00839	13.01046	12.86955	11.78672	14.09684
17461968	Mkrn2	makorin, ring finger protein, 2	24.82772	24.84976	25.64703	32.19489	33.85319	35.79311
17491755	Mkrn3	makorin, ring finger protein, 3	5.002399	4.267142	3.627762	5.048133	3.735186	6.147714
17311512	Mal2	mal, T cell differentiation protein 2	192.6916	116.7695	109.6947	143.4148	125.5362	117.0021
17391339	Mall	mal, T cell differentiation protein-like	4.771358	4.037186	3.729055	3.297745	3.729055	4.980567
17260944	Mdh1	malate dehydrogenase 1, NAD (soluble)	848.8466	768.4566	720.0733	791.9964	737.5311	937.0875
17223747	Mdh1b	malate dehydrogenase 1B, NAD (soluble)	3.097448	2.789932	3.274308	3.003386	3.687262	2.967463
17443310	Mdh2	malate dehydrogenase 2, NAD (mitochondrial)	388.7503	450.1057	555.8455	421.6085	339.0326	426.0031
17338168	Mea1	male enhanced antigen 1	54.081	53.21533	55.10648	50.37025	50.24402	50.68159
17292032	Mak	male germ cell-associated kinase	4.763791	3.999141	5.06429	4.186167	4.566124	5.002295
17451668	Mlec	malectin	252.0454	226.3451	239.8602	183.339	154.7033	145.2808
17256162	Msl1	male-specific lethal 1 homolog (Drosophila)	25.3749	35.31453	38.33765	37.66389	48.78954	50.15286
17520815	Msl2	male-specific lethal 2 homolog (Drosophila)	109.735	115.7028	96.8614	91.46513	86.94192	89.91376
17546160	Msl3	male-specific lethal 3 homolog (Drosophila)	99.84858	91.33083	93.97727	112.4149	124.5723	108.0167
17233342	Msl3l2	male-specific lethal 3-like 2 (Drosophila)	12.69985	11.19071	8.024547	10.83224	12.62157	8.830836
17529398	Me1	malic enzyme 1, NADP(+)-dependent, cytosolic	341.6843	266.1157	244.1713	251.5724	165.1874	236.809
17355391	Me2	malic enzyme 2, NAD(+)-dependent, mitochondrial	14.00966	12.29773	15.60796	10.28179	13.11849	13.39423
17480048	Me3	malic enzyme 3, NADP(+)-dependent, mitochondrial	3.768159	5.379007	5.156206	2.834983	2.477371	3.861737
17500551	Mfhas1	malignant fibrous histiocytoma amplified sequence 1	48.04585	37.3633	38.4108	33.89475	31.85129	39.30797
17534352	Mcts1	malignant T cell amplified sequence 1	24.09013	22.5496	23.48536	28.71276	19.26994	28.4473
17377774	Mcts2	malignant T cell amplified sequence 2	15.84065	14.33281	11.99241	15.02559	15.18821	14.13627
17319864	Mcat	malonyl CoA:ACP acyltransferase (mitochondrial)	57.74844	51.10314	41.15769	41.28398	43.88968	50.6542
17506073	Mlycd	malonyl-CoA decarboxylase	67.97395	63.65491	59.91478	74.32677	66.52801	62.669
17351330	Malt1	MALT1 paracaspase	21.94302	21.69243	21.82213	20.26797	23.88959	23.96455
17457609	Mgam	maltase-glucoamylase	196.1903	183.2615	169.6279	190.2378	183.0444	190.2378
17367203	Malrd1	MAM and LDL receptor class A domain containing 1	3.565835	3.381258	3.381258	3.381258	3.381258	3.47774
17367209	Malrd1	MAM and LDL receptor class A domain containing 1	3.736247	2.831644	2.831644	3.054974	2.748225	2.619806
17363533	Mamdc2	MAM domain containing 2	6.860863	8.870282	8.612129	8.295679	6.890459	8.096565
17382638	Mamdc4	MAM domain containing 4	6.506628	6.429382	6.071109	6.920998	5.481687	6.429382
17343088	Mdga1	MAM domain containing glycosylphosphatidylinositol anchor 1	7.042316	5.368615	5.582533	6.42417	8.119045	6.521799
17281459	Mdga2	MAM domain containing glycosylphosphatidylinositol anchor 2	3.779145	3.656187	3.656187	3.686444	3.18656	4.453779
17329403	Masp1	mannan-binding lectin serine peptidase 1	198.6617	174.6194	160.8386	163.4482	198.6961	151.4859
17421704	Masp2	mannan-binding lectin serine peptidase 2	434.7433	489.1455	492.5137	480.5291	447.0493	457.5546
17527532	Mpi	mannose phosphate isomerase	20.46164	21.0264	16.16453	17.39644	10.90335	15.62257
17367102	Mrc1	mannose receptor, C type 1	114.7349	123.2975	149.9482	112.239	85.85082	102.945
17257361	Mrc2	mannose receptor, C type 2	8.674917	7.753835	9.10406	11.42997	8.67119	9.109614
17462647	M6pr	mannose-6-phosphate receptor, cation dependent	112.5241	146.545	141.9609	121.5023	136.3102	130.3078
17298977	Mbl1	mannose-binding lectin (protein A) 1	292.2218	280.3043	249.5422	293.1977	260.1572	229.1232
17358640	Mbl2	mannose-binding lectin (protein C) 2	460.5998	485.9183	423.5798	379.3853	609.1104	491.6303
17264824	Mpdu1	mannose-P-dolichol utilization defect 1	135.1146	142.2091	151.2719	130.4551	140.0929	153.3995
17240880	Man1a	mannosidase 1, alpha	281.9724	240.9684	233.5455	238.8283	218.326	244.9681
17339108	Man2a1	mannosidase 2, alpha 1	285.2217	339.3923	363.2956	326.473	385.6611	352.7617
17492549	Man2a2	mannosidase 2, alpha 2	54.64017	66.50219	80.67613	59.96422	56.91196	60.15864
17503397	Man2b1	mannosidase 2, alpha B1	98.85829	94.24734	93.39672	92.65969	95.24094	96.55573
17447514	Man2b2	mannosidase 2, alpha B2	88.45121	92.08537	107.2653	92.25019	72.16113	64.4255
17408422	Man1a2	mannosidase, alpha, class 1A, member 2	64.77522	61.96106	57.02134	59.06125	62.87741	60.52419
17367926	Man1b1	mannosidase, alpha, class 1B, member 1	31.53364	28.56427	25.25523	25.44323	28.08738	22.36235
17431332	Man1c1	mannosidase, alpha, class 1C, member 1	18.97871	13.97639	19.53122	15.24326	8.236149	13.08262
17517659	Man2c1	mannosidase, alpha, class 2C, member 1	13.45873	17.1207	17.55111	17.55111	16.685	14.22493
17527474	Man2c1os	mannosidase, alpha, class 2C, member 1, opposite strand	3.627762	3.608277	3.584421	4.855299	5.216637	4.170239
17402905	Manba	mannosidase, beta A, lysosomal	45.34589	43.16111	43.16111	37.59268	38.89142	36.86219
17378716	Manbal	mannosidase, beta A, lysosomal-like	32.44918	41.31031	51.31576	48.58166	41.27654	43.46614
17423713	Manea	mannosidase, endo-alpha	117.1497	111.5611	105.0957	116.611	109.9253	110.3919
17429886	Maneal	mannosidase, endo-alpha-like	8.365258	9.408013	7.394487	9.294711	7.935659	8.2142
17248941	Mgat1	mannoside acetylglucosaminyltransferase 1	24.86458	26.76118	24.42082	30.22771	26.76118	31.77522
17275882	Mgat2	mannoside acetylglucosaminyltransferase 2	137.9747	77.35861	84.00481	88.23566	88.40402	82.43242
17313087	Mgat3	mannoside acetylglucosaminyltransferase 3	5.964536	8.269385	5.746924	5.743937	5.494393	5.766876
17222332	Mgat4a	mannoside acetylglucosaminyltransferase 4, isoenzyme A	4.431392	3.564081	3.32446	3.649832	4.752478	4.15803
17249151	Mgat4b	mannoside acetylglucosaminyltransferase 4, isoenzyme B	27.95491	32.00244	32.73949	28.82682	30.89754	26.30454
17216753	Mgat5	mannoside acetylglucosaminyltransferase 5	28.90526	26.34541	25.5911	20.96659	27.45753	17.22904
17258683	Mgat5b	mannoside acetylglucosaminyltransferase 5, isoenzyme B	6.790275	7.129496	6.570303	6.88263	6.347375	6.974501
17459952	Mogs	mannosyl-oligosaccharide glucosidase	43.52885	29.36102	25.08182	24.29454	33.20434	29.36102
17472023	Mansc1	MANSC domain containing 1	9.950783	8.717473	8.09174	9.02627	9.259986	7.705187
17472893	Mansc4	MANSC domain containing 4	10.83945	8.6233	8.500042	10.10089	6.781385	9.55271
17226736	Mapkapk2	MAP kinase-activated protein kinase 2	118.554	139.6537	163.8316	139.2844	156.5598	158.588
17452209	Mapkapk5	MAP kinase-activated protein kinase 5	42.56011	51.53248	54.90745	52.32215	46.35587	58.56107
17417314	Mknk1	MAP kinase-interacting serine/threonine kinase 1	15.87066	14.21035	18.3872	17.3668	14.5341	12.60112
17243034	Mknk2	MAP kinase-interacting serine/threonine kinase 2	119.6395	179.039	196.2053	154.2568	180.4331	205.9742
17230760	Mark1	MAP/microtubule affinity regulating kinase 1	5.418599	6.060765	7.018389	5.614548	6.253787	7.003956
17346004	Mark1-ps1	MAP/microtubule affinity regulating kinase 1, pseudogene 1	3.284537	2.414342	3.194987	2.757632	2.793977	2.503298
17362289	Mark2	MAP/microtubule affinity regulating kinase 2	35.01085	31.03749	39.37314	35.16577	35.4922	29.71563
17279169	Mark3	MAP/microtubule affinity regulating kinase 3	101.1912	97.68059	94.23493	103.0607	90.43567	91.59519
17487249	Mark4	MAP/microtubule affinity regulating kinase 4	29.84899	25.96111	34.54153	26.36112	23.67791	30.86714

17281280	Mbip	MAP3K12 binding inhibitory protein 1	19.69708	14.10515	13.48637	12.75932	14.68773	16.54427
17326887	Map3k7cl	Map3k7 C-terminal like	5.587179	4.222215	5.450845	5.375877	3.5801	3.473861
17329074	Map6d1	MAP6 domain containing 1	6.426708	7.456197	6.24482	7.19044	5.838226	8.076947
17430006	Map7d1	MAP7 domain containing 1	49.28648	46.75212	58.30388	36.51228	39.98508	39.89622
17539167	Map7d2	MAP7 domain containing 2	3.490249	3.842273	4.070737	3.631612	3.755175	3.755175
17541856	Mtap7d3	MAP7 domain containing 3	3.54506	3.54506	3.887681	3.54506	3.140621	3.54506
17388128	Madd	MAP-kinase activating death domain	15.70226	12.50333	15.70226	16.30158	17.91381	14.15981
17419020	Marcksl1	MARCKS-like 1	20.40739	20.29525	21.60899	20.29525	18.72235	21.83847
17284963	Marcksl1-ps4	MARCKS-like 1, pseudogene 4	20.87494	40.73819	31.03878	26.3966	13.46554	19.0082
17353619	Mzb1	marginal zone B and B1 cell-specific protein 1	7.570402	10.03501	12.45092	10.27709	8.063745	8.708293
17359531	Marveld1	MARVEL (membrane-associating) domain containing 1	34.25327	35.41037	44.00087	40.24646	32.62645	36.68606
17295685	Marveld2	MARVEL (membrane-associating) domain containing 2	31.38515	35.91663	31.3206	35.83481	39.81582	38.0512
17512860	Marveld3	MARVEL (membrane-associating) domain containing 3	10.53881	9.734184	8.319864	7.935109	7.935109	7.935109
17340902	Mas1	MAS1 oncogene	6.908775	6.032015	5.602691	6.472621	5.54624	3.748517
17491318	Mrgpra1	MAS-related GPR, member A1	31.49738	59.13458	50.65525	31.56754	55.38487	37.042
17491323	Mrgpra2a	MAS-related GPR, member A2A	2.70133	3.436905	2.685238	2.84966	3.756685	3.477788
17491329	Mrgpra2b	MAS-related GPR, member A2B	6.07195	5.780603	4.720108	5.410568	5.179992	5.67197
17491335	Mrgpra3	MAS-related GPR, member A3	3.131296	2.661279	2.914402	3.073452	3.167292	3.114693
17491340	Mrgpra4	MAS-related GPR, member A4	2.234094	2.853713	2.591397	2.591397	2.745236	2.60821
17491310	Mrgpra6	MAS-related GPR, member A6	2.983488	1.83933	2.120376	1.924749	2.465329	2.07661
17491315	Mrgpra9	MAS-related GPR, member A9	4.176313	3.224465	3.02738	3.727272	3.555716	2.763641
17491354	Mrgprb1	MAS-related GPR, member B1	3.250699	2.7808	2.49258	2.82306	2.443129	2.628227
17548579	Mrgprb13	MAS-related GPR, member B13	3.479428	2.931035	3.673003	4.195957	3.971929	3.389894
17491362	Mrgprb2	MAS-related GPR, member B2	2.243218	3.203791	2.733198	2.781937	2.735283	2.752934
17491366	Mrgprb3	MAS-related GPR, member B3	2.972486	2.497994	3.075732	3.348976	3.012999	2.948457
17491352	Mrgprb4	MAS-related GPR, member B4	2.900899	2.062137	2.633808	2.719271	3.173115	2.719271
17491349	Mrgprb5	MAS-related GPR, member B5	2.273224	2.104518	2.443078	2.681648	2.817528	2.036596
17478273	Mrgprb8	MAS-related GPR, member B8	2.890081	2.464884	2.482132	2.304206	2.604543	2.137701
17485471	Mrgprd	MAS-related GPR, member D	3.062691	3.403832	3.293885	3.759498	3.062691	3.045819
17498365	Mrgpre	MAS-related GPR, member E	11.747	12.09442	15.07964	9.898363	15.5925	11.10987
17485462	Mrgprf	MAS-related GPR, member F	5.253468	5.63955	8.05369	5.68488	4.860989	6.695538
17498360	Mrgprg	MAS-related GPR, member G	12.81623	11.24436	12.53708	12.05651	13.17262	10.94432
17333320	Mrgprh	MAS-related GPR, member H	3.664331	3.497927	3.095795	3.808888	3.615337	3.863397
17491344	Mrgprx1	MAS-related GPR, member X1	2.524469	2.431792	2.497977	2.167843	2.812903	2.724138
17491358	Mrgprx2	MAS-related GPR, member X2	6.139155	5.545279	5.125703	4.801649	5.125739	6.454476
17498730	Mcemp1	mast cell expressed membrane protein 1	10.28949	10.56016	8.446947	10.35022	9.44637	10.44326
17257599	Milr1	mast cell immunoglobulin like receptor 1	5.028332	4.300219	5.450241	4.730065	4.53386	4.801616
17300717	Mcpt1	mast cell protease 1	4.135409	4.708345	4.282283	3.978901	4.685295	5.346038
17300725	Mcpt2	mast cell protease 2	2.803191	2.948763	2.941068	2.761132	2.83031	2.704009
17306960	Mcpt4	mast cell protease 4	2.406006	2.997937	2.391689	2.406006	2.057454	2.464013
17306968	Mcpt8	mast cell protease 8	4.287064	3.853475	3.977688	4.207899	4.69199	4.743165
17306952	Mcpt9	mast cell protease 9	4.566884	3.071812	3.229789	2.691947	4.352065	2.513696
17306945	Mcpt-ps1	mast cell protease, pseudogene 1	5.125597	5.813109	5.48933	7.630777	6.261594	5.865931
17262326	Maml1	mastermind like 1 (Drosophila)	30.02724	20.47356	22.0163	20.82537	19.04816	19.93871
17514678	Maml2	mastermind like 2 (Drosophila)	10.89973	9.658439	13.80855	13.31057	10.14186	10.23522
17405154	Maml3	mastermind like 3 (Drosophila)	4.681043	8.596861	10.21629	7.596	8.398548	6.663545
17535284	Mamld1	mastermind-like domain containing 1	9.326657	10.20617	12.99334	9.995093	11.57331	10.41528
17413528	Melk	maternal embryonic leucine zipper kinase	3.865996	3.815547	3.729055	4.23118	3.729055	3.729055
17278662	Meg3	maternally expressed 3	12.93717	12.93717	12.93717	12.61344	12.07147	12.02312
17419305	Matn1	matrilin 1, cartilage matrix protein	3.452856	4.838869	3.949965	3.652514	3.83384	4.22482
17310922	Matn2	matrilin 2	9.310251	9.015578	9.310251	10.58222	7.673794	8.788031
17273973	Matn3	matrilin 3	6.451166	9.228141	6.328353	7.949092	7.721119	9.883486
17394160	Matn4	matrilin 4	4.74698	4.379056	4.065858	3.391327	3.9469	3.872566
17349554	Matr3	matrin 3	108.3511	110.0923	97.05227	93.50943	109.564	107.223
17439825	Mepe	matrix extracellular phosphoglycoprotein with ASARM motif (bone)	9.488237	7.748247	9.413884	10.59602	10.41218	9.488237
17472192	Mgp	matrix Gla protein	20.62468	22.46878	18.52845	20.35399	18.30036	20.1538
17514541	Mmp10	matrix metallopeptidase 10	7.548919	8.123773	8.627186	7.491736	5.621168	7.920547
17514495	Mmp12	matrix metallopeptidase 12	9.243697	10.6052	14.63946	10.36954	8.049784	9.626891
17514482	Mmp13	matrix metallopeptidase 13	4.603464	3.863879	5.119986	5.055487	4.062084	4.455786
17300279	Mmp14	matrix metallopeptidase 14 (membrane-inserted)	107.1264	124.1482	150.6858	107.9381	125.0629	92.95883
17504293	Mmp15	matrix metallopeptidase 15	61.75012	57.78487	55.93115	62.20678	43.97007	53.75958
17412013	Mmp16	matrix metallopeptidase 16	4.239341	4.035311	4.239341	3.867657	4.413827	4.577659
17442887	Mmp17	matrix metallopeptidase 17	7.664704	7.793331	6.70743	7.07598	7.415788	8.420524
17238558	Mmp19	matrix metallopeptidase 19	102.2318	118.9721	111.4289	105.859	87.6523	86.15902
17514528	Mmp1a	matrix metallopeptidase 1a (interstitial collagenase)	2.08917	2.08917	2.08917	2.08917	1.968968	2.08917
17523834	Mmp1b	matrix metallopeptidase 1b (interstitial collagenase)	2.415011	2.357781	2.718649	2.326176	2.639912	3.066005
17503825	Mmp2	matrix metallopeptidase 2	14.782	14.782	15.88177	14.782	14.02783	13.81793
17514580	Mmp20	matrix metallopeptidase 20 (enamelysin)	4.85678	5.690834	6.557337	7.684865	6.038541	6.234959
17497194	Mmp21	matrix metallopeptidase 21	5.082048	5.719147	5.617157	5.012534	5.212444	5.54305
17433815	Mmp23	matrix metallopeptidase 23	10.88184	13.0722	13.89044	12.15325	15.45177	11.43084
17378450	Mmp24	matrix metallopeptidase 24	12.57991	12.14104	10.62455	10.22862	11.63178	14.96603
17341508	Mmp25	matrix metallopeptidase 25	5.153991	5.525749	5.527588	5.525749	5.870852	6.195969
17514566	Mmp27	matrix metallopeptidase 27	6.255564	4.332591	7.657853	6.430496	5.265827	5.590549
17266911	Mmp28	matrix metallopeptidase 28 (epilysin)	8.462477	8.625093	8.312601	8.625093	9.206865	8.625093
17514515	Mmp3	matrix metallopeptidase 3	3.282853	3.59881	3.097126	3.097126	2.77214	3.038923
17514592	Mmp7	matrix metallopeptidase 7	7.764807	8.467112	8.467112	8.059107	8.467112	7.758405
17514553	Mmp8	matrix metallopeptidase 8	4.473426	4.404139	5.090298	3.919284	4.70003	4.20927
17379606	Mmp9	matrix metallopeptidase 9	8.463181	9.182646	11.36543	11.28145	6.355299	9.452264
17272509	Mxra7	matrix-remodelling associated 7	12.3394	11.78598	13.90315	12.71465	10.83495	16.49984
17422732	Mxra8	matrix-remodelling associated 8	19.47012	23.6556	23.43495	21.86589	17.62559	19.96194
17433895	Mxra8os	matrix-remodelling associated 8, opposite strand [Source:MGI Symbol;Acc:MGI:37(	3.062691	3.141514	3.079039	2.665673	3.362117	3.062691
17542815	Mtcp1	mature T cell proliferation 1	5.813149	3.893264	5.466358	6.943774	6.320287	9.310251
17458752	Mturn	maturin, neural progenitor differentiation regulator homolog (Xenopus)	8.312774	6.992896	6.883918	4.567897	6.377186	6.458191
17509923	Mau2	MAU2 chromatid cohesion factor homolog (C. elegans)	52.12993	70.39592	63.42633	59.82597	49.0299	54.6254

17252817	Mnt	max binding protein	19.89467	18.48355	21.99906	21.38652	25.32306	24.99591
17468511	Mxd1	MAX dimerization protein 1	32.58057	30.20007	29.92688	32.63482	37.27664	35.78468
17292828	Mxd3	Max dimerization protein 3	7.415428	7.505874	7.484158	7.601057	8.876986	7.599582
17447218	Mxd4	Max dimerization protein 4	69.25038	61.43547	57.5008	59.38967	64.3046	63.0909
17374906	Mga	MAX gene associated	35.92285	51.33497	44.69991	41.36905	47.43303	62.11501
17360331	Mxi1	MAX interactor 1, dimerization protein	38.22196	37.96268	48.24341	39.91475	43.52221	43.46825
17282090	Max	Max protein	39.68577	52.2341	45.15081	43.30675	45.89484	46.45011
17256537	Mlx	MAX-like protein X	57.18283	40.3957	55.33882	45.72052	47.68786	52.35625
17255118	Mbtd1	mbt domain containing 1	19.05728	20.97284	19.22978	20.57613	19.21352	20.57613
17541948	Mcf2	mcf.2 transforming sequence	5.509686	6.088302	6.719063	5.197111	6.822167	6.27215
17499155	Mcf2l	mcf.2 transforming sequence-like	5.608558	5.346519	5.608558	5.608558	4.696448	4.96509
17392138	Mkks	McKusick-Kaufman syndrome	29.77728	25.86519	20.32284	24.14462	22.28406	27.97313
17496920	Mcmbp	MCM (minichromosome maintenance deficient) binding protein	89.8745	81.60129	85.35833	84.27659	80.6711	78.62139
17311602	Mtbp	Mdm2, transformed 3T3 cell double minute p53 binding protein	3.984531	4.844138	6.347639	3.456711	4.291494	5.539363
17404478	Mecom	MDS1 and EVI1 complex locus	5.237397	5.137833	4.972633	5.237397	4.316225	5.237397
17404503	Mecom	MDS1 and EVI1 complex locus	3.03547	2.890221	2.943372	4.753269	3.135205	3.532942
17396546	Mecomos	MDS1 and EVI1 complex locus, opposite strand	5.622376	5.090051	5.409518	5.780122	6.836652	5.495954
17421600	Mtor	mechanistic target of rapamycin (serine/threonine kinase)	94.68868	110.46	99.79184	86.50071	99.76003	84.12193
17254895	Mks1	Meckel syndrome, type 1	13.79917	14.51346	13.976	15.51305	13.50077	14.18398
17268755	Med1	mediator complex subunit 1	51.71748	54.8637	52.98015	54.9619	50.83076	54.9981
17288414	Med10	mediator complex subunit 10	18.12664	22.88817	21.93633	25.36118	26.80704	25.36118
17252032	Med11	mediator complex subunit 11	24.87664	17.19027	25.584	20.69084	18.26715	19.23484
17536669	Med12	mediator complex subunit 12	30.83293	32.80082	35.49318	29.47092	33.84662	33.84662
17397827	Med12l	mediator complex subunit 12-like	12.50315	16.70097	13.99297	12.39434	11.61695	12.54221
17267268	Med13	mediator complex subunit 13	93.62033	132.2157	110.2275	113.0941	109.9779	111.4575
17441540	Med13l	mediator complex subunit 13-like	43.28724	41.27654	41.77593	34.76684	35.32236	40.06332
17540282	Med14	mediator complex subunit 14	46.13245	38.87121	45.57277	44.66553	44.62778	40.67893
17328756	Med15	mediator complex subunit 15	46.98082	44.46074	55.24607	41.64525	39.00643	40.09638
17242729	Med16	mediator complex subunit 16	21.71169	25.47623	30.41498	22.20404	29.37038	19.37279
17524141	Med17	mediator complex subunit 17	23.96056	22.05968	16.83206	20.33312	15.99911	16.56872
17430853	Med18	mediator complex subunit 18	6.698416	9.364228	8.596265	8.85339	6.472576	9.284859
17372584	Med19	mediator complex subunit 19	43.13658	47.3729	38.85801	46.73301	61.38907	45.99292
17338280	Med20	mediator complex subunit 20	37.34984	42.1199	40.04166	39.97871	33.33625	35.72891
17464303	Med21	mediator complex subunit 21	51.66123	65.07989	64.27029	56.64906	57.0953	49.52345
17383164	Med22	mediator complex subunit 22	22.62789	24.5107	24.9966	23.54176	24.01444	23.27824
17232244	Med23	mediator complex subunit 23	29.39278	27.742	24.78079	24.5636	22.00074	23.81189
17268849	Med24	mediator complex subunit 24	37.60389	30.79683	31.49345	38.23097	29.31973	28.35183
17268882	Med24	mediator complex subunit 24	8.057878	8.355928	6.992896	7.693126	5.972355	8.253408
17490432	Med25	mediator complex subunit 25	45.58129	49.72536	58.53794	45.76047	52.52127	40.59978
17510572	Med26	mediator complex subunit 26	19.18079	17.61289	18.49649	18.49649	18.49649	17.28466
17368834	Med27	mediator complex subunit 27	10.69	9.431322	11.67321	10.8496	10.5732	9.328984
17437263	Med28	mediator complex subunit 28	27.9309	23.30811	28.96061	25.36716	21.49024	25.77606
17488463	Med29	mediator complex subunit 29	28.81983	38.42651	44.59531	37.21675	42.20553	34.23144
17311492	Med30	mediator complex subunit 30	13.06876	10.24686	10.18095	11.75555	10.48228	10.89686
17265625	Med31	mediator complex subunit 31	15.6578	17.03311	12.35189	12.35189	19.84481	13.39423
17301890	Med4	mediator complex subunit 4	12.62105	10.86156	9.163635	11.37646	10.53216	14.85009
17282368	Med6	mediator complex subunit 6	28.96021	23.61874	23.01065	22.72912	23.64855	24.57399
17248800	Med7	mediator complex subunit 7	21.85589	18.83273	13.89535	15.79439	16.74653	15.58763
17417749	Med8	mediator complex subunit 8	36.88724	37.07925	35.00551	44.04023	33.93519	47.06687
17250325	Med9	mediator complex subunit 9	20.16065	25.99226	27.58783	25.16802	22.74173	22.11985
17263506	Med9os	mediator complex subunit 9, opposite strand	7.514567	8.118707	10.18292	8.451515	8.453859	8.517677
17347210	Memo1	mediator of cell motility 1	53.76629	38.69432	28.66285	39.23556	36.27692	41.83997
17244559	Gm18409	mediator of cell motility 1 pseudogene	2.723421	3.456855	2.465653	2.663106	2.777467	2.839919
17337250	Mdc1	mediator of DNA damage checkpoint 1	22.11035	22.02561	23.6597	22.48732	26.21281	20.37813
17327580	Mefv	Mediterranean fever	4.786364	5.157708	5.677477	4.31452	5.316549	4.523658
17477946	Mamstr	MEF2 activating motif and SAP domain containing transcriptional regulator	7.27335	7.872272	7.253966	7.667307	5.737491	7.172818
17235626	Matk	megakaryocyte-associated tyrosine kinase	8.389181	12.41541	10.12732	9.79829	8.315483	9.79829
17320225	Mlc1	megalencephalic leukoencephalopathy with subcortical cysts 1 homolog (human)	4.510164	7.214903	6.662252	6.776874	4.804179	5.217252
17459857	M1ap	meiosis 1 associated protein	4.046543	4.488583	4.435756	4.257628	4.1322	4.257628
17328335	Marf1	meiosis arrest female 1	133.4143	142.6464	137.3935	132.5893	157.2984	151.7129
17313461	Mei1	meiosis defective 1	5.120306	5.086806	5.321378	5.120306	5.76328	5.895115
17381215	Meig1	meiosis expressed gene 1	4.764913	4.045141	4.20927	4.20752	3.487396	4.20927
17256979	Meioc	meiosis specific with coiled-coil domain	2.606728	2.936749	2.721138	2.610163	3.783995	3.267778
17334433	Meiob	meiosis specific with OB domains	6.263504	6.642379	7.069656	7.035681	13.50133	8.175234
17519155	Mns1	meiosis-specific nuclear structural protein 1	9.614355	9.093842	9.614355	9.822566	9.614355	10.96193
17519875	Mei4	meiosis-specific, MEI4 homolog (S. cerevisiae)	3.12516	3.045333	3.339703	3.373486	4.692315	3.366315
17249684	Meikin	meiotic kinetochore factor	3.078441	2.520098	2.437783	2.533514	2.586354	2.977951
17406323	Mnd1	meiotic nuclear divisions 1 homolog (S. cerevisiae)	6.171709	8.104314	6.287864	6.588682	5.531497	8.566762
17514760	Mre11a	meiotic recombination 11 homolog A (S. cerevisiae)	22.46769	21.80278	19.81279	16.25983	13.44483	18.08084
17260815	Meis1	Meis homeobox 1	12.53874	11.42332	13.91231	14.31296	11.50939	11.24366
17389608	Meis2	Meis homeobox 2	23.64659	19.11694	24.24306	21.23305	14.01325	12.30694
17389635	Meis2	Meis homeobox 2	7.91078	9.41421	10.28536	9.71053	8.718198	7.326733
17474078	Meis3	Meis homeobox 3	8.162381	7.049935	6.109736	6.446294	9.172977	7.669806
17358591	Mlana	melan-A	5.215878	4.257894	5.215878	6.540224	4.88382	5.305027
17313254	Mchr1	melanin-concentrating hormone receptor 1	3.856842	3.73762	3.486936	4.226197	4.320325	3.784703
17506627	Mc1r	melanocortin 1 receptor	4.521182	4.681043	4.316943	4.48454	4.732197	4.681043
17355264	Mc2r	melanocortin 2 receptor	3.111349	3.516134	2.884417	2.907219	2.779547	2.606963
17326987	Mrap	melanocortin 2 receptor accessory protein	120.967	108.5917	111.1258	100.337	92.50632	83.57966
17520063	Mrap2	melanocortin 2 receptor accessory protein 2	3.695245	4.190754	3.721257	3.695245	2.849957	3.549436
17380089	Mc3r	melanocortin 3 receptor	3.799369	4.036626	3.641468	3.687134	4.478576	3.074965
17355135	Mc4r	melanocortin 4 receptor	3.790426	3.186229	3.140077	3.557663	3.485945	4.25061
17351587	Mc5r	melanocortin 5 receptor	6.223905	7.044318	5.234312	8.971816	5.13737	5.154956
17315397	Myg1	melanocyte proliferating gene 1	24.22996	32.00577	33.2405	33.28984	30.46329	23.14486
17548705	Mela	melanoma antigen	3.827945	3.360486	4.013581	4.24231	3.970608	3.729055

17542213	Magea10	melanoma antigen family A, 10	3.126221	3.0496	2.921785	2.985681	3.614109	3.009325
17543014	Mageb16	melanoma antigen family B, 16	6.187343	6.914668	8.000499	6.650994	6.489647	6.992896
17545148	Mageb16-ps1	melanoma antigen family B, 16, pseudogene 1	4.235904	5.266155	3.703322	3.703322	3.8745	3.629674
17548816	Mageb17-ps	melanoma antigen family B, 17, pseudogene [Source:MGI Symbol;Acc:MGI:364313	4.699476	3.622586	4.021741	4.021518	3.558329	4.087079
17543141	Mageb18	melanoma antigen family B, 18	2.318578	2.509726	2.435014	2.930445	2.763813	2.496402
17545582	Magea1	melanoma antigen, family A, 1	2.952342	3.890161	2.867135	3.001243	4.067127	2.962592
17545567	Magea2	melanoma antigen, family A, 2	5.378709	2.859474	3.339703	3.19045	3.038375	4.373354
17545553	Magea3	melanoma antigen, family A, 3	2.431607	2.357308	2.88172	3.321972	3.08378	2.627426
17535416	Magea4	melanoma antigen, family A, 4	3.550076	3.020694	3.008282	2.791363	2.443383	3.293862
17545574	Magea5	melanoma antigen, family A, 5	2.331082	2.278351	2.335902	2.917656	2.725668	2.779066
17545548	Magea6	melanoma antigen, family A, 6	2.245287	2.181	2.083357	2.083357	2.120126	2.045509
17545560	Magea8	melanoma antigen, family A, 8	2.572248	2.245863	2.745064	2.202253	2.615705	2.163774
17543139	Mageb1	melanoma antigen, family B, 1	2.204712	2.071061	2.075271	2.113274	2.459628	2.339483
17536218	Mageb2	melanoma antigen, family B, 2	1.777439	1.898013	1.87954	2.083357	1.969665	2.285991
17390670	Mageb3	melanoma antigen, family B, 3	2.724384	2.220785	1.943612	2.856859	2.09246	2.131797
17543069	Mageb4	melanoma antigen, family B, 4	3.171598	3.723553	3.857992	4.052073	3.435357	3.988621
17543082	Gm41	melanoma antigen, family B, 4 pseudogene	3.893803	3.824073	3.729055	3.729055	4.231502	3.729055
17543131	Mageb5	melanoma antigen, family B, 5	2.474762	1.709981	2.514096	2.464799	2.413171	2.219862
17543264	Maged1	melanoma antigen, family D, 1	161.3612	171.3616	172.1163	130.8591	120.6814	127.7607
17545353	Maged2	melanoma antigen, family D, 2	18.43186	27.62782	17.33469	23.51941	16.85822	21.24763
17537029	Magee1	melanoma antigen, family E, 1	5.347038	5.536676	4.886969	5.10008	4.418663	4.502793
17543885	Magee2	melanoma antigen, family E, 2	3.474704	3.428506	3.516816	3.422807	3.933736	3.468423
17545482	Mageh1	melanoma antigen, family H, 1	7.565	8.10027	8.162211	8.733924	9.157476	7.680286
17478793	Magel2	melanoma antigen, family L, 2	4.775494	5.102662	5.624169	5.183764	5.108359	6.092862
17235249	Mum1	melanoma associated antigen (mutated) 1	7.017027	9.001716	7.065933	5.536829	7.190639	9.906051
17538060	Mum1l1	melanoma associated antigen (mutated) 1-like 1	3.736636	4.076384	3.736636	3.35718	3.736636	4.376386
17516518	Mcam	melanoma cell adhesion molecule	21.67695	21.67695	29.10712	18.63457	21.67695	18.48201
17488151	Mia	melanoma inhibitory activity	3.509925	3.261555	3.872781	3.803005	4.673068	3.942635
17275718	Mia2	melanoma inhibitory activity 2	262.9965	265.3826	254.7362	266.5976	255.2788	275.4506
17230668	Mia3	melanoma inhibitory activity 3	187.4056	182.3745	159.4411	142.3778	187.5459	167.4376
17215629	Mlph	melanophilin	6.420875	8.589535	7.949532	7.679328	7.972819	5.988937
17224125	Mreg	melanoregulin	86.22952	79.48771	65.27687	79.33744	66.75111	81.15061
17500867	Mtnr1a	melatonin receptor 1A	24.82772	20.04041	18.97753	18.97753	20.84876	17.70531
17524228	Mtnr1b	melatonin receptor 1B	6.992896	6.037662	4.601199	6.059883	5.506784	6.193608
17469183	Magi1	membrane associated guanylate kinase, WW and PDZ domain containing 1	30.1175	34.02823	29.03095	26.50954	27.31251	22.67782
17435008	Magi2	membrane associated guanylate kinase, WW and PDZ domain containing 2	8.170645	7.595421	7.357879	7.834331	7.273602	8.290103
17408698	Magi3	membrane associated guanylate kinase, WW and PDZ domain containing 3	76.2513	75.37112	61.94732	64.73975	78.43252	63.0944
17286254	Mboat1	membrane bound O-acyltransferase domain containing 1	3.906157	3.836817	3.386488	3.691712	3.245836	2.515787
17274558	Mboat2	membrane bound O-acyltransferase domain containing 2	3.455441	3.610078	2.796573	3.585141	3.005052	4.478483
17500517	Mboat4	membrane bound O-acyltransferase domain containing 4	5.195887	6.614261	5.687363	6.614261	6.742249	6.576591
17485574	Mboat7	membrane bound O-acyltransferase domain containing 7	35.55573	33.47982	40.9213	32.28965	32.82652	31.71251
17541846	Mmgt1	membrane magnesium transporter 1	43.34068	41.81417	37.31982	40.27772	46.78389	35.85544
17250755	Mmgt2	membrane magnesium transporter 2	8.958704	6.196571	6.543489	6.943774	6.76819	6.286339
17397990	Mme	membrane metallo endopeptidase	120.9042	117.405	111.0927	137.882	80.41373	118.17
17422415	Mmel1	membrane metallo-endopeptidase-like 1	5.208074	5.364709	4.744579	5.340297	5.146851	4.846489
17542751	Mpp1	membrane protein, palmitoylated	87.30667	98.68918	107.1873	86.01283	87.0752	77.3377
17270028	Mpp2	membrane protein, palmitoylated 2 (MAGUK p55 subfamily member 2)	9.175393	8.850384	8.850384	7.626441	8.89676	8.850384
17269995	Mpp3	membrane protein, palmitoylated 3 (MAGUK p55 subfamily member 3)	8.561657	8.542806	6.777955	10.42431	8.749072	9.597176
17223509	Mpp4	membrane protein, palmitoylated 4 (MAGUK p55 subfamily member 4)	7.529218	7.930985	4.186748	7.229571	6.171709	5.937642
17276714	Mpp5	membrane protein, palmitoylated 5 (MAGUK p55 subfamily member 5)	34.98028	34.76395	26.48695	43.38577	29.01265	34.29366
17458520	Mpp6	membrane protein, palmitoylated 6 (MAGUK p55 subfamily member 6)	184.604	141.0849	118.2692	154.6175	151.2845	163.438
17352549	Mpp7	membrane protein, palmitoylated 7 (MAGUK p55 subfamily member 7)	23.97618	21.1032	16.76732	20.46831	17.16526	19.85775
17501520	March1	membrane-associated ring finger (C3HC4) 1	7.885324	7.423811	8.964046	7.584373	7.312172	7.753082
17270684	March10	membrane-associated ring finger (C3HC4) 10	6.494147	6.386387	6.494147	7.068774	6.564841	6.184084
17310661	March11	membrane-associated ring finger (C3HC4) 11	2.948416	3.730839	3.137401	4.043543	3.281033	3.518328
17343602	March2	membrane-associated ring finger (C3HC4) 2	50.98532	58.64068	50.72306	53.0496	51.38873	53.20427
17354463	March3	membrane-associated ring finger (C3HC4) 3	6.292049	6.992896	6.992896	7.977021	6.992896	5.782567
17224146	March4	membrane-associated ring finger (C3HC4) 4	5.149349	3.807885	4.247959	3.923959	5.292869	5.111051
17359008	March5	membrane-associated ring finger (C3HC4) 5	193.066	194.3819	184.5477	179.1544	186.9307	193.066
17316298	March6	membrane-associated ring finger (C3HC4) 6	199.855	230.9473	232.3877	227.7204	257.1364	239.4748
17371059	March7	membrane-associated ring finger (C3HC4) 7	27.2099	40.06971	31.92158	35.82016	42.11172	30.20991
17462113	March8	membrane-associated ring finger (C3HC4) 8	103.2831	86.01402	96.159	97.52296	92.38905	88.14825
17245692	March9	membrane-associated ring finger (C3HC4) 9	3.113376	4.20927	4.667565	4.886529	4.20927	5.840371
17513417	Mbtps1	membrane-bound transcription factor peptidase, site 1	63.92995	50.58788	60.48309	56.92301	46.77254	46.61137
17545675	Mbtps2	membrane-bound transcription factor peptidase, site 2	19.7789	19.61397	24.24054	18.92984	19.55465	23.78351
17362922	Ms4a1	membrane-spanning 4-domains, subfamily A, member 1	3.907484	3.77836	3.787485	3.449913	4.661519	3.787485
17362839	Ms4a10	membrane-spanning 4-domains, subfamily A, member 10	4.964471	4.938524	4.796892	4.750667	4.410249	5.265425
17362915	Ms4a12	membrane-spanning 4-domains, subfamily A, member 12	2.98477	2.589691	2.348501	3.169884	3.396573	2.654242
17362904	Ms4a13	membrane-spanning 4-domains, subfamily A, member 13	6.082313	5.672835	5.29709	5.36233	5.460829	5.288579
17362941	Ms4a14	membrane-spanning 4-domains, subfamily A, member 14	2.479604	2.778565	2.803852	2.634465	2.716935	3.033577
17362849	Ms4a15	membrane-spanning 4-domains, subfamily A, member 15	7.626547	7.775902	7.338423	7.476588	5.742663	8.689782
17362861	Ms4a18	membrane-spanning 4-domains, subfamily A, member 18	2.199522	2.67402	2.625842	2.682708	2.435925	3.125788
17362986	Ms4a2	membrane-spanning 4-domains, subfamily A, member 2	3.395796	3.738035	3.604602	3.845683	3.288159	4.058284
17362998	Ms4a3	membrane-spanning 4-domains, subfamily A, member 3	7.274459	8.166795	7.230974	8.073868	6.219118	8.15452
17357640	Ms4a4a	membrane-spanning 4-domains, subfamily A, member 4A	3.665297	4.376993	3.775349	5.465411	4.320865	4.095643
17357659	Ms4a4b	membrane-spanning 4-domains, subfamily A, member 4B	3.959477	2.746263	2.712908	2.3594	4.0319	2.667762
17357648	Ms4a4c	membrane-spanning 4-domains, subfamily A, member 4C	9.029462	7.620048	9.399344	11.38293	4.783942	9.222705
17357700	Ms4a4d	membrane-spanning 4-domains, subfamily A, member 4D	17.97637	17.94127	16.06791	17.58435	16.18505	14.37958
17362934	Ms4a5	membrane-spanning 4-domains, subfamily A, member 5	3.788981	3.542162	3.248041	3.123904	3.308139	3.339703
17357688	Ms4a6b	membrane-spanning 4-domains, subfamily A, member 6B	7.96056	7.423708	7.974754	9.34957	8.090843	8.062851
17357671	Ms4a6c	membrane-spanning 4-domains, subfamily A, member 6C	6.992896	8.071686	6.302525	7.166308	6.26637	7.344173
17362973	Ms4a6d	membrane-spanning 4-domains, subfamily A, member 6D	6.591103	5.979687	5.27474	4.035199	3.729055	4.701887
17362953	Ms4a7	membrane-spanning 4-domains, subfamily A, member 7	5.319018	5.506173	7.068382	4.971117	6.190775	5.506173
17362874	Ms4a8a	membrane-spanning 4-domains, subfamily A, member 8A	7.480424	8.591738	11.50812	7.108327	8.401867	7.761901

17276262	Mnat1	menage a trois 1	28.29451	25.62251	27.752	26.36752	31.86388	31.64181
17440633	Mn1	meningioma 1	7.154892	9.171451	7.217154	10.27923	8.299992	9.326657
17365250	Mgea5	meningioma expressed antigen 5 (hyaluronidase)	118.3323	127.0956	132.3441	134.579	137.2593	154.3413
17345066	Mep1a	meprin 1 alpha	3.200195	4.472557	4.26584	4.012635	4.118446	4.325509
17348860	Mep1b	meprin 1 beta	4.211225	3.54447	3.835753	3.809337	3.454771	4.284628
17312738	Mpst	mercaptopyruvate sulfurtransferase	42.89448	44.32043	46.84652	52.65469	49.8192	49.4497
17530782	Manf	mesencephalic astrocyte-derived neurotrophic factor	50.17789	21.44218	21.049	20.60853	24.66169	17.87684
17269974	Meox1	mesenchyme homeobox 1	5.725573	3.962827	5.974508	5.103475	3.723037	5.629591
17275062	Meox2	mesenchyme homeobox 2	2.884957	3.339703	4.45696	3.339703	3.372527	3.64609
17444970	Medag	mesenteric estrogen dependent adipogenesis	3.072941	3.910546	3.820178	4.272084	4.598712	3.130038
17492943	Mesdc1	mesoderm development candidate 1	9.178828	9.310251	9.310251	9.125098	9.898775	10.33796
17479899	Mesdc2	mesoderm development candidate 2	80.67501	50.40448	58.58218	57.21891	65.3561	65.32304
17416110	Mier1	mesoderm induction early response 1 homolog (Xenopus laevis	28.87642	32.09596	29.66295	31.04136	29.57959	30.56122
17242589	Mier2	mesoderm induction early response 1, family member 2	18.04641	17.43483	19.74807	16.48224	18.04641	13.5195
17289824	Mier3	mesoderm induction early response 1, family member 3	43.41916	41.4141	37.10136	38.99589	39.25379	38.99589
17492425	Mesp1	mesoderm posterior 1	4.68338	5.338447	4.799719	6.50523	5.120306	5.120306
17479474	Mesp2	mesoderm posterior 2	8.889055	11.28606	9.044809	9.750931	9.946094	10.21948
17456934	Mest	mesoderm specific transcript	23.64151	23.77939	27.50382	17.93294	17.06253	18.26741
17279957	Msgn1	mesogenin 1	6.015522	5.908861	6.588956	6.834094	6.44549	5.56106
17342340	Msln	mesothelin	5.19495	5.757051	6.036239	4.925158	4.083907	6.072329
17334730	Mslnl	mesothelin-like	7.304016	8.893739	6.451837	6.588363	6.996726	7.135329
17456176	Met	met proto-oncogene	170.8587	154.414	174.8362	174.8519	188.6607	162.3395
17310893	Mtdh	metadherin	137.8409	126.5777	133.2776	131.2406	120.5744	127.3822
17418339	Mtf1	metal response element binding transcription factor 1	33.82059	36.09182	33.4156	33.4156	29.16103	29.87362
17440136	Mtf2	metal response element binding transcription factor 2	10.52749	10.15839	9.327756	8.506113	9.656966	12.10365
17443797	Mblac1	metallo-beta-lactamase domain containing 1	8.263655	10.22664	7.116658	7.65918	10.71537	10.1434
17288903	Mblac2	metallo-beta-lactamase domain containing 2	31.05384	21.79462	23.38319	23.73312	25.36118	32.93592
17355145	Mppe1	metallophosphoesterase 1	15.60774	17.3514	17.11121	16.89764	16.87435	19.08583
17313661	Mpped1	metallophosphoesterase domain containing 1	4.574265	6.030928	5.018618	6.710871	6.030928	9.943819
17374028	Mpped2	metallophosphoesterase domain containing 2	5.101671	6.176074	5.765757	5.612019	5.604633	5.048833
17503942	Mt1	metallothionein 1	51.21448	321.7843	536.8592	176.4995	450.9551	442.3425
17503937	Mt2	metallothionein 2	64.7411	1220.139	1796.583	619.1821	1647.257	1654.753
17503932	Mt3	metallothionein 3	6.078435	5.742584	7.292078	6.5843	5.835717	6.088176
17503926	Mt4	metallothionein 4	5.063272	5.443596	7.06368	6.251793	3.729055	5.036355
17355935	Mtl5	metallothionein-like 5, testis-specific (tesmin)	6.778378	8.450929	6.778378	4.87023	6.61174	6.660175
17279471	Mta1	metastasis associated 1	88.12748	90.82767	107.336	78.63556	81.8046	86.91902
17340017	Mta3	metastasis associated 3	70.65844	78.29129	80.43882	68.64535	50.64542	62.14429
17477785	Mtag2	metastasis associated gene 2	2.937726	3.705094	3.197269	2.851399	3.356622	3.294022
17279724	Macc1	metastasis associated in colon cancer 1	2.910829	3.136944	2.996545	3.021322	3.362863	2.750245
17361798	Malat1	metastasis associated lung adenocarcinoma transcript 1 (non-coding RNA)	752.4438	1097.671	1192.811	1139.851	1261.942	1261.155
17317327	Mtss1	metastasis suppressor 1	192.7704	211.4906	235.3836	203.1498	336.152	255.0648
17505623	Mtss1l	metastasis suppressor 1-like	16.17294	16.36918	17.70739	15.2458	14.31296	15.31125
17357338	Mta2	metastasis-associated gene family, member 2	88.9708	88.9967	85.00819	89.50523	86.47792	88.89066
17406941	Mtx1	metaxin 1	30.27909	39.18827	27.92393	28.51047	30.72583	35.67422
17372119	Mtx2	metaxin 2	120.6062	108.2295	113.8436	120.0857	105.162	109.4463
17289118	Mtx3	metaxin 3	20.34581	20.41701	14.99812	18.49367	17.93319	28.80166
17342386	Metrn	meteorin, glial cell differentiation regulator	9.925089	9.454558	11.50086	13.07193	11.50086	12.47346
17259679	Metrnl	meteorin, glial cell differentiation regulator-like	12.13565	11.73894	11.79285	11.77659	11.92403	11.98294
17513560	Mthfsd	methenyltetrahydrofolate synthetase domain containing	28.37582	33.45129	29.67446	32.96264	24.34443	28.52174
17298956	Mat1a	methionine adenosyltransferase I, alpha	2497.311	3189.556	3431.039	3296.204	3146.364	3125.736
17467753	Mat2a	methionine adenosyltransferase II, alpha	141.0859	174.4041	195.8982	158.4342	160.4278	178.7736
17261825	Mat2b	methionine adenosyltransferase II, beta	41.53628	43.69875	44.88246	47.8186	53.85842	53.32093
17244378	Metap2	methionine aminopeptidase 2	154.0933	160.8252	149.5444	145.8316	156.7857	161.3064
17307695	Msra	methionine sulfoxide reductase A	199.1346	208.9813	209.8042	201.5676	190.7461	193.9996
17334419	Msrb1	methionine sulfoxide reductase B1	442.4958	431.2139	381.6801	419.2073	416.1933	393.6283
17367367	Msrb2	methionine sulfoxide reductase B2	34.25494	28.23573	29.43519	34.07472	34.22432	34.92141
17245433	Msrb3	methionine sulfoxide reductase B3	38.79046	38.40867	40.1495	34.53004	29.40949	30.66434
17245850	Mars	methionine-tRNA synthetase	111.688	89.43577	82.49473	93.38313	88.58865	76.01436
17212904	Mars2	methionine-tRNA synthetase 2 (mitochondrial)	7.385881	6.192277	5.776598	9.714973	7.214303	8.183341
17410680	Metap1	methionyl aminopeptidase 1	90.25031	83.62776	87.48538	90.16027	84.2468	71.06563
17371756	Metap1d	methionyl aminopeptidase type 1D (mitochondrial)	13.83605	20.84949	19.35186	21.80066	22.80677	21.74708
17371775	Metap1d	methionyl aminopeptidase type 1D (mitochondrial)	3.542505	2.838463	4.84864	5.661758	3.923986	4.79297
17542573	Mecp2	methyl CpG binding protein 2	19.7158	28.00087	26.69726	29.25842	23.62594	30.21494
17351726	Mbd1	methyl-CpG binding domain protein 1	87.05046	104.2086	40.85016	67.02001	62.41323	58.7086
17351668	Mbd2	methyl-CpG binding domain protein 2	93.85424	88.50763	85.09485	100.7461	96.55439	88.44324
17242910	Mbd3	methyl-CpG binding domain protein 3	32.15096	31.2887	36.00861	30.54999	33.23865	34.76419
17514899	Mbd3l1	methyl-CpG binding domain protein 3-like 1	2.642072	2.377376	2.045509	2.561591	2.466474	2.18965
17514892	Mbd3l2	methyl-CpG binding domain protein 3-like 2	2.954246	2.857335	3.153776	3.059212	2.954246	3.120982
17469952	Mbd4	methyl-CpG binding domain protein 4	5.48933	5.000977	6.557746	4.99135	4.437447	7.19039
17370612	Mbd5	methyl-CpG binding domain protein 5	20.05556	20.49486	21.76774	19.90005	17.75939	18.62954
17245831	Mbd6	methyl-CpG binding domain protein 6	28.91067	36.65352	41.13002	36.84891	44.1368	39.69458
17245847	Mbd6	methyl-CpG binding domain protein 6	7.115376	7.848136	7.164818	6.537822	9.916837	6.535058
17404750	Mccc1	methylcrotonoyl-Coenzyme A carboxylase 1 (alpha)	97.7368	81.36859	75.01775	86.5103	82.98105	93.32101
17396933	Mccc1os	methylcrotonoyl-Coenzyme A carboxylase 1 (alpha), opposite strand	3.840995	4.290215	4.659839	4.57639	3.840995	3.890997
17295503	Mccc2	methylcrotonoyl-Coenzyme A carboxylase 2 (beta)	101.5467	102.925	106.3916	112.2841	101.8767	101.2368
17468143	Mthfd2	methylenetetrahydrofolate dehydrogenase (NAD+ dependent), methenyltetrahydr	10.22969	8.730958	8.114094	7.690194	8.213061	6.329713
17231387	Mthfd1l	methylenetetrahydrofolate dehydrogenase (NADP+ dependent) 1-like	10.8566	13.94349	16.43444	9.960306	12.60776	9.310251
17439003	Mthfd2l	methylenetetrahydrofolate dehydrogenase (NADP+ dependent) 2-like	8.405828	8.077506	6.491402	9.209099	8.688337	7.906833
17276520	Mthfd1	methylenetetrahydrofolate dehydrogenase (NADP+ dependent), methenyltetrahyd	257.436	276.246	278.8103	263.9407	256.7553	314.3526
17385030	Mmadhc	methylmalonic aciduria (cobalamin deficiency) cblD type, with homocystinuria	93.09682	75.7876	84.32801	80.34687	68.58257	77.29161
17510726	Mmaa	methylmalonic aciduria (cobalamin deficiency) type A	30.8278	30.8278	32.61446	36.06131	23.1447	38.12459
17451536	Mmab	methylmalonic aciduria (cobalamin deficiency) type B homolog (human)	38.80122	41.81649	42.15758	42.87278	37.3861	45.90438
17428702	Mmachc	methylmalonic aciduria cblC type, with homocystinuria	21.04798	21.84604	21.29791	22.84521	22.93311	22.29286
17478883	Mcee	methylmalonyl CoA epimerase	79.94689	102.2339	100.3704	89.22277	93.66772	118.6633

17337719	Mut	methylmalonyl-Coenzyme A mutase	256.4742	246.6138	237.3611	281.0553	269.9952	320.5199
17454157	Mepce	methylphosphate capping enzyme	20.34779	26.73004	24.01394	23.56772	24.24878	20.12142
17509629	Msmo1	methylsterol monoxygenase 1	81.9107	57.76966	80.27618	60.2279	94.27086	54.70497
17415380	Mtap	methylthioadenosine phosphorylase	64.3416	58.76845	54.42019	61.97481	49.25642	48.95078
17511102	Mri1	methylthioribose-1-phosphate isomerase homolog (S. cerevisiae)	18.21759	27.62782	20.78758	23.29454	27.01015	24.36114
17237876	Mettl1	methyltransferase like 1	23.38319	17.72373	19.67975	20.63727	22.28946	12.71985
17497131	Mettl10	methyltransferase like 10	8.49533	11.02373	11.98385	11.1694	11.98419	14.74925
17229121	Mettl11b	methyltransferase like 11B	3.246488	3.487492	3.769466	3.663849	3.483651	3.711983
17228943	Mettl13	methyltransferase like 13	13.09306	15.12685	12.75075	11.38987	10.71782	10.84757
17410043	Mettl14	methyltransferase like 14	22.02347	20.14877	19.61982	22.02347	19.30537	21.94773
17389121	Mettl15	methyltransferase like 15	9.332024	10.48228	11.31222	10.54012	9.91505	10.33854
17252800	Mettl16	methyltransferase like 16	34.58753	26.52829	31.27721	29.20756	31.08917	27.3233
17299644	Mettl17	methyltransferase like 17	24.43589	24.65007	23.15048	27.86496	22.54257	25.09434
17218813	Mettl18	methyltransferase like 18	8.26102	6.138346	6.071067	7.256531	5.908984	8.132055
17257310	Mettl2	methyltransferase like 2	20.88912	20.33919	18.06882	21.02538	26.2288	19.24618
17464492	Mettl20	methyltransferase like 20	45.01457	59.76546	52.43216	49.0503	56.62275	32.90238
17223777	Mettl21a	methyltransferase like 21A	25.68261	26.79236	23.79407	27.80784	31.30237	27.53606
17245686	Mettl21b	methyltransferase like 21B	6.295452	4.95354	4.647678	5.279276	5.019209	4.95354
17222668	Mettl21c	methyltransferase like 21C	2.332763	2.50198	2.793706	2.625842	3.044784	2.641248
17222707	Mettl21e	methyltransferase like 21E	2.563367	2.8002	2.67402	3.012205	2.67402	2.67402
17322735	Mettl22	methyltransferase like 22	22.57619	21.76231	26.56104	23.59959	23.95475	19.19475
17258653	Mettl23	methyltransferase like 23	25.60032	24.53875	26.19428	27.6884	25.90054	25.33114
17232778	Mettl24	methyltransferase like 24	9.904197	10.56375	10.20271	9.800192	11.56746	8.449582
17244679	Mettl25	methyltransferase like 25	6.171709	5.284291	5.471438	5.696319	5.260354	6.602581
17306289	Mettl3	methyltransferase like 3	27.97488	26.33245	27.49448	37.80093	30.58029	34.2063
17348054	Mettl4	methyltransferase like 4	30.15113	28.94275	28.14701	30.5716	33.9679	31.19321
17386328	Mettl5	methyltransferase like 5	39.18501	36.47459	37.02788	37.07308	40.00033	39.21517
17371520	Mettl5os	methyltransferase like 5, opposite strand [Source:MGI Symbol;Acc:MGI:1922537]	5.068609	5.145709	5.300432	5.896137	5.300432	6.055097
17304757	Mettl6	methyltransferase like 6	31.81863	32.53363	29.40216	36.06581	31.16274	33.71994
17315002	Mettl7a1	methyltransferase like 7A1	374.6501	373.3617	357.5249	367.7624	375.1594	402.2866
17315007	Mettl7a3	methyltransferase like 7A3	110.2924	89.23205	110.6324	150.6879	87.28912	131.8743
17246381	Mettl7b	methyltransferase like 7B	606.5236	702.1084	683.8708	649.7361	616.0271	719.4148
17386377	Mettl8	methyltransferase like 8	15.96444	13.7856	18.10128	19.87386	13.50246	16.43523
17482626	Mettl9	methyltransferase like 9	98.84321	93.83603	87.96087	88.722	83.14053	88.89436
17513681	Mvd	mevalonate (diphospho) decarboxylase	26.62335	22.82353	24.91461	22.82353	25.95859	24.5733
17440954	Mvk	mevalonate kinase	25.09869	24.91599	26.72782	31.00932	30.05869	18.64349
17399086	Mex3a	mex3 homolog A (C. elegans)	13.43393	12.54508	11.71066	13.19171	12.34881	14.46205
17479834	Mex3b	mex3 homolog B (C. elegans)	8.467112	8.467112	9.047944	9.023893	7.055166	5.97397
17351681	Mex3c	mex3 homolog C (C. elegans)	66.53723	57.83536	58.56878	54.68715	62.49115	59.90647
17242902	Mex3d	mex3 homolog D (C. elegans)	12.48921	12.90385	11.36401	13.3577	13.55309	14.16423
17319050	Mfng	MFNG O-fucosylpeptide 3-beta-N-acetylglucosaminyltransferase	11.38467	13.21523	14.98108	13.1224	13.43431	13.1224
17237006	Mgat4c	MGAT4 family, member C	2.677506	2.824313	2.643098	2.630998	3.050742	2.563722
17502908	Mgat4d	MGAT4 family, member C	3.33138	3.194965	3.194965	3.194965	2.534041	4.155724
17227149	Mgat4e	MGAT4 family, member E	3.703322	3.363679	3.219485	3.703322	6.043231	2.943087
17474342	Mill1	MHC I like leukocyte 1	6.33059	5.481649	5.771275	6.171709	4.397794	5.261995
17474363	Mill2	MHC I like leukocyte 2	11.19234	8.874587	11.79734	10.59139	16.19403	10.29977
17481899	Micalcl	MICAL C-terminal like	15.14248	11.42261	11.23509	11.25411	8.836837	9.426159
17454428	Micall2	MICAL-like 2	7.040754	11.37559	10.71545	9.507065	10.22175	10.14052
17499540	Mcph1	microcephaly, primary autosomal recessive 1	8.357839	7.246327	8.443727	8.146554	6.966963	6.524964
17462663	Mfap5	microfibrillar associated protein 5	4.428536	5.570358	5.29342	4.495402	5.480927	5.231229
17390626	Mfap1a	microfibrillar-associated protein 1A	25.991	21.92386	27.21896	25.16715	36.12411	31.30042
17390614	Mfap1b	microfibrillar-associated protein 1B	13.63412	6.828695	11.87685	7.407003	7.863873	7.373195
17420951	Mfap2	microfibrillar-associated protein 2	11.96659	10.95639	11.09517	11.32147	10.08179	11.90883
17249888	Mfap3	microfibrillar-associated protein 3	35.66606	35.90319	32.17593	33.29445	31.60345	26.51285
17501346	Mfap3l	microfibrillar-associated protein 3-like	4.015807	4.086116	5.946126	5.144426	4.327879	4.609116
17250585	Mfap4	microfibrillar-associated protein 4	4.070511	4.754851	5.90624	4.925124	4.315178	5.00167
17461132	Mitf	microphthalmia-associated transcription factor	23.0723	18.57332	18.68618	17.28106	19.30028	18.145
17326032	Morc1	microrchidia 1	3.687952	3.144956	3.387662	4.148357	3.14415	3.463859
17246636	Morc2a	microrchidia 2A	23.24261	28.23984	30.12084	28.47907	29.64897	30.27846
17343542	Morc2b	microrchidia 2B	4.998343	4.425652	4.962716	4.801649	4.190551	4.536941
17327311	Morc3	microrchidia 3	19.00412	25.68546	27.24746	21.90715	22.03034	28.44829
17544878	Morc4	microrchidia 4	4.401545	7.776656	9.07055	5.402997	5.845312	5.547778
17516408	Mir100	microRNA 100	2.927157	2.446315	2.106927	2.531513	2.489162	2.597583
17427623	Mir101a	microRNA 101a	22.6497	24.77117	24.63777	28.50731	21.99357	25.04881
17358515	Mir101b	microRNA 101b	2.481049	2.481049	2.528567	2.271303	2.541781	2.623295
17523680	Mir101c	microRNA 101c	2.343703	2.703765	3.783575	3.24098	3.089578	2.953407
17248486	Mir103-1	microRNA 103-1	3.10009	2.332359	2.175565	2.278525	2.495156	2.288465
17376508	Mir103-2	microRNA 103-2	3.079999	3.134793	4.878271	4.759038	5.350078	4.073475
17542237	Mir105	microRNA 105	2.499553	2.285991	2.492753	2.304206	2.161256	2.285991
17454254	Mir106b	microRNA 106b	5.48933	4.95536	4.904071	6.780962	5.302821	3.674709
17364163	Mir107	microRNA 107	11.07834	14.56385	25.97767	13.1417	19.01545	11.65793
17255602	Mir10a	microRNA 10a	2.332117	2.332117	2.637132	2.233444	2.291909	2.332117
17372106	Mir10b	microRNA 10b	2.666339	2.42264	2.42264	2.42264	2.42264	3.339703
17449106	Mir1187	microRNA 1187	2.363951	2.032901	1.787707	2.429215	2.148396	2.410195
17278751	Mir1188	microRNA 1188	10.16233	10.99267	11.68358	12.86697	11.51386	12.03563
17283343	Mir1190	microRNA 1190	22.81578	27.09134	18.81798	21.71349	16.75465	15.19674
17475536	Mir1191	microRNA 1191	52.66199	60.42778	47.10636	40.79838	43.55028	34.65808
17358219	Mir1192	microRNA 1192	10.16607	9.684651	12.75437	8.893739	9.149989	17.3962
17278824	Mir1193	microRNA 1193	4.943764	4.649804	4.69082	4.817885	4.935273	6.510688
17346950	Mir1195	microRNA 1195	136.366	195.2272	203.5614	176.808	127.1652	206.6277
17278812	Mir1197	microRNA 1197	2.392535	2.039817	2.528462	2.20852	2.730945	2.356915
17533002	Mir1198	microRNA 1198	21.64407	32.82106	25.01913	25.70772	23.87006	17.12901
17351321	Mir122	microRNA 122	6.030928	5.671892	4.645166	8.55525	6.030928	5.917427
17323999	Mir1224	microRNA 1224	2.280357	2.513225	1.848865	2.551897	2.473819	3.187488

17320035	Mir1249	microRNA 1249	6.988874	4.790081	5.518668	4.535464	3.729055	5.56106
17301291	Mir124a-1	microRNA 124a-1	9.375138	8.072581	8.420524	8.253065	7.459195	8.253065
17396190	Mir124a-2	microRNA 124a-2	7.761201	11.85439	8.590785	9.986575	6.588591	6.100511
17380877	Mir124a-3	microRNA 124a-3	2.21163	2.118995	1.81162	1.724203	2.039775	2.029255
17244232	Mir1251	microRNA 1251	3.038249	3.187201	2.706147	3.462754	3.338963	2.953407
17333729	Mir125a	microRNA 125a	2.737331	3.057389	3.607351	3.923252	3.394714	2.894271
17516420	Mir125b-1	microRNA 125b-1	2.379503	2.095493	2.497475	2.439372	2.300217	2.594632
17326704	Mir125b-2	microRNA 125b-2	2.187677	2.198638	2.183118	2.161646	2.235566	2.124068
17538433	Mir1264	microRNA 1264	2.697289	2.245567	2.250367	2.088128	2.216249	2.530215
17368347	Mir126a	microRNA 126a	2.873721	3.554213	3.360044	3.263862	2.37764	2.840018
17216868	Mir128-1	microRNA 128-1	3.373392	3.059615	3.059615	3.059615	3.015736	2.933047
17531799	Mir128-2	microRNA 128-2	5.135554	4.890731	4.540528	7.522019	7.598423	3.238599
17456543	Mir129-1	microRNA 129-1	3.104864	2.67402	1.898013	2.230814	2.715824	2.688497
17388592	Mir129-2	microRNA 129-2	2.462869	1.864489	1.818211	2.90595	3.211417	2.24915
17538435	Mir1298	microRNA 1298	3.548133	3.129018	2.899512	2.771794	2.933697	3.069939
17387512	Mir130a	microRNA 130a	2.437631	1.946659	1.866778	2.151332	2.192201	2.821319
17328621	Mir130b	microRNA 130b	3.726703	3.790952	3.959971	4.33827	4.301441	3.959862
17252880	Mir132	microRNA 132	3.74566	3.429338	3.875977	3.153754	4.464814	3.473909
17352693	Mir133a-1	microRNA 133a-1	3.764478	2.316843	2.281385	2.036907	3.749479	2.297667
17380764	Mir133a-2	microRNA 133a-2	5.100363	4.723741	3.28481	3.847256	5.45765	4.156301
17211367	Mir133b	microRNA 133b	3.728951	4.92369	3.939793	4.729081	4.286292	4.344891
17278854	Mir134	microRNA 134	21.26561	26.15854	21.28979	22.98642	23.6812	22.59858
17521141	Mir135a-1	microRNA 135a-1	5.512474	3.485124	3.737828	3.26574	4.114879	4.030783
17244215	Mir135a-2	microRNA 135a-2	2.172702	2.172702	1.907377	1.95297	2.48239	2.082361
17217169	Mir135b	microRNA 135b	4.184253	4.801649	4.801649	4.031984	5.052299	3.944466
17278713	Mir136	microRNA 136	2.391921	2.387499	1.986254	2.076768	2.271145	2.325998
17402109	Mir137	microRNA 137	2.071061	1.938768	2.185813	2.185813	2.060051	1.865006
17523434	Mir138-1	microRNA 138-1	3.707714	3.211729	4.106844	4.181904	3.395043	3.440886
17503972	Mir138-2	microRNA 138-2	3.269251	3.016028	4.115136	3.628844	4.289115	3.818537
17480922	Mir139	microRNA 139	6.995562	10.32937	8.259704	7.645972	6.269281	14.27096
17505309	Mir140	microRNA 140	2.965407	2.426875	2.772832	3.27985	2.932803	3.597438
17470792	Mir141	microRNA 141	2.468167	2.192383	1.751899	2.45668	2.345946	2.154256
17254838	Mir142	microRNA 142	17.09864	10.1102	11.87676	11.50269	10.20066	11.47753
17354793	Mir143	microRNA 143	19.29948	30.54173	25.23685	15.43357	18.89719	19.88019
17253448	Mir144	microRNA 144	2.406006	1.898013	2.110156	2.31474	2.072138	2.072138
17354791	Mir145a	microRNA 145a	2.406006	2.420844	2.525344	2.332731	2.420844	2.150974
17261942	Mir146	microRNA 146	2.060051	2.060051	2.033704	2.060051	2.283584	2.024225
17360018	Mir146b	microRNA 146b	6.752679	5.080463	5.617429	6.708359	5.617429	5.912589
17466758	Mir148a	microRNA 148a	2.815498	2.221047	2.393646	2.412702	3.263862	2.672704
17315568	Mir148b	microRNA 148b	3.882403	2.542892	3.062691	2.652568	2.848021	3.618671
17215832	Mir149	microRNA 149	14.60085	23.24357	15.50015	11.97829	14.84433	21.58908
17477693	Mir150	microRNA 150	4.241487	5.769835	4.803913	3.703322	4.636544	3.535686
17317898	Mir151	microRNA 151	3.959285	4.118446	3.374374	3.365615	4.242839	4.683769
17255674	Mir152	microRNA 152	5.122471	6.004774	6.371796	6.073424	6.867952	6.025997
17279668	Mir153	microRNA 153	2.4474	2.420844	2.084368	2.606909	2.627754	2.729487
17278878	Mir154	microRNA 154	2.321987	2.882579	2.362084	2.533374	2.335692	2.435554
17326794	Mir155	microRNA 155	3.481671	3.401144	3.495261	3.636977	3.053619	3.850431
17307518	Mir15a	microRNA 15a	14.57749	14.37958	16.23595	16.23595	16.94303	17.83229
17398264	Mir15b	microRNA 15b	2.074797	1.870962	1.924815	2.187177	2.263131	2.075228
17307516	Mir16-1	microRNA 16-1	16.54989	26.23771	24.36689	16.90502	26.44274	19.21485
17398266	Mir16-2	microRNA 16-2	2.432922	3.869186	3.594447	2.967298	4.204721	3.480151
17302632	Mir17	microRNA 17	8.375759	6.791263	7.094938	5.932993	7.279756	6.233144
17302634	Mir18	microRNA 18	3.362397	2.828917	3.784834	3.737154	2.850134	3.362397
17217844	Mir181a-1	microRNA 181a-1	2.141854	2.084585	2.275305	2.606909	2.040652	2.04269
17370483	Mir181a-2	microRNA 181a-2	17.62364	17.77774	13.49217	14.74782	18.40212	13.21047
17217846	Mir181b-1	microRNA 181b-1	4.01461	2.917758	2.255364	2.67402	4.490139	2.178825
17370485	Mir181b-2	microRNA 181b-2	3.095173	3.095173	3.099746	2.716314	3.57886	2.410646
17511075	Mir181c	microRNA 181c	18.05579	21.69183	16.83206	20.56644	19.66925	29.98481
17511073	Mir181d	microRNA 181d	108.7062	96.18767	112.3729	125.4223	112.1806	114.6641
17465478	Mir182	microRNA 182	2.891784	3.362886	2.543544	2.851676	3.317186	2.370876
17465482	Mir183	microRNA 183	3.63063	3.437906	3.613683	3.560402	4.093259	3.713347
17479703	Mir1839	microRNA 1839	3.92385	3.654718	3.339703	3.107124	4.843769	4.083454
17529645	Mir184	microRNA 184	5.596908	5.279349	6.050398	5.335783	5.616339	5.694464
17328933	Mir185	microRNA 185	39.99894	55.74962	52.08081	62.77073	52.806	50.83323
17403864	Mir186	microRNA 186	8.387844	3.213376	2.960621	6.922773	2.044148	5.209446
17353141	Mir187	microRNA 187	126.8905	150.9775	115.5609	158.8179	132.7971	144.2532
17539824	Mir188	microRNA 188	10.16607	10.3546	9.421064	11.43473	16.43763	6.931656
17410508	Mir1895	microRNA 1895	11.19878	9.310251	9.166811	9.310251	9.47634	9.310251
17285654	Mir1896	microRNA 1896	6.511004	8.631911	6.860815	6.326861	6.860815	8.584952
17310430	Mir1898	microRNA 1898	14.28798	26.12846	22.96126	24.85407	24.43522	29.86057
17514621	Mir1899	microRNA 1899	6.936318	7.698994	6.029256	6.171709	5.74549	6.060513
17541703	Mir18b	microRNA 18b	2.67402	2.426505	2.038955	2.475311	2.445314	1.953133
17524582	Mir1900	microRNA 1900	3.062691	3.382688	4.003745	3.298111	3.062691	3.42276
17352710	Mir1901	microRNA 1901	8.85382	6.283159	3.611204	6.700552	5.741867	6.041472
17289792	Mir1904	microRNA 1904	2.655991	2.425143	2.576245	2.900939	3.38562	2.520937
17406805	Mir1905	microRNA 1905	14.99199	19.91081	18.49649	16.32544	19.82172	13.64698
17543104	Mir1906-1	microRNA 1906-1	7.407979	5.915787	6.448797	7.453975	4.514731	7.453975
17316977	Mir1907	microRNA 1907	3.200484	3.317255	3.106694	3.369069	3.264586	3.250023
17399581	Mir190b	microRNA 190b	2.536501	2.01581	1.749085	2.14487	2.830467	2.17644
17521854	Mir191	microRNA 191	3.190738	3.517621	4.289888	4.267822	4.162442	5.708295
17538431	Mir1912	microRNA 1912	2.852565	2.931543	2.938643	2.474042	2.638101	2.964235
17356741	Mir192	microRNA 192	2.627881	2.301317	3.563611	4.565396	3.388249	2.670093
17228788	Mir1927	microRNA 1927	6.992896	4.796294	6.231502	7.188546	6.177101	4.659871
17224254	Mir1928	microRNA 1928	2.933494	2.0097	1.994352	2.450422	2.458673	1.878759

17233072	Mir1929	microRNA 1929	10.04726	7.743684	10.04726	13.77423	7.634269	9.23102
17234705	Mir1930	microRNA 1930	4.289888	5.535524	3.536053	4.20927	3.966042	5.314743
17236525	Mir1931	microRNA 1931	5.386134	4.68562	6.464102	5.932252	4.120702	6.689832
17259076	Mir1932	microRNA 1932	21.87392	31.24471	30.96679	30.35474	31.78821	37.03789
17260931	Mir1933	microRNA 1933	17.89283	22.87928	24.90045	26.36112	17.22521	21.74754
17251719	Mir1934	microRNA 1934	2.474025	2.277988	2.652182	2.229608	2.322116	2.411248
17283475	Mir1936	microRNA 1936	4.007799	2.965098	2.989926	4.121673	3.404847	4.30001
17280867	Mir1938	microRNA 1938	15.82595	19.3288	14.35807	15.24865	10.97767	14.46425
17253835	Mir193a	microRNA 193a	2.927441	2.809065	3.036607	2.843209	3.040798	3.604827
17323038	Mir193b	microRNA 193b	7.175503	6.982676	7.543427	8.154859	6.979482	5.637869
17315198	Mir1941	microRNA 1941	4.22081	3.954458	5.233514	3.590961	3.173423	5.803567
17220529	Mir194-1	microRNA 194-1	16.88348	9.789804	8.905768	9.488237	12.3323	13.93422
17318405	Mir1942	microRNA 1942	2.176425	2.374922	2.793009	2.637644	2.485248	2.313777
17356739	Mir194-2	microRNA 194-2	6.753849	6.610274	5.369322	7.281875	4.147523	5.310545
17319205	Mir1943	microRNA 1943	12.84077	19.91661	25.39742	10.44717	5.914598	22.2279
17328220	Mir1945	microRNA 1945	17.60093	25.0966	21.55499	34.3579	22.82828	12.41085
17329842	Mir1946a	microRNA 1946a	77.99393	105.9463	117.6641	95.1611	82.57596	104.5645
17524746	Mir1946b	microRNA 1946b	3.712626	2.480516	2.461333	3.226142	2.618372	2.904601
17324996	Mir1947	microRNA 1947	2.659804	3.132642	2.95609	1.898013	3.295255	2.549227
17348589	Mir1948	microRNA 1948	25.96111	19.16192	17.22235	32.77673	21.69409	19.57527
17349549	Mir1949	microRNA 1949	2.816669	1.985896	3.009972	2.953407	2.584974	2.953407
17358872	Mir1950	microRNA 1950	3.234046	2.004219	1.70818	2.535886	2.184465	2.004219
17374421	Mir1951	microRNA 1951	7.521811	5.754258	7.521811	6.171709	7.935109	6.04277
17376889	Mir1952	microRNA 1952	4.371626	3.429956	2.847686	2.973949	2.994988	3.729055
17392892	Mir1953	microRNA 1953	4.642365	4.537256	6.488028	6.523065	5.265498	4.0847
17369948	Mir1954	microRNA 1954	81.88686	75.2229	77.60728	96.43066	92.59356	74.64274
17373482	Mir1955	microRNA 1955	5.338965	3.883991	3.164703	3.703322	3.729055	3.703322
17403148	Mir1956	microRNA 1956	67.45437	101.0125	84.94576	79.683	73.19332	75.99697
17417858	Mir1957a	microRNA 1957a	12.09199	18.98608	22.58542	21.21746	13.0799	9.901589
17425179	Mir1958	microRNA 1958	2.268785	2.232763	2.034395	2.147384	1.898908	2.15729
17252011	Mir195a	microRNA 195a	5.424951	5.555397	7.143148	5.507204	4.639148	4.94164
17435769	Mir1960	microRNA 1960	5.937971	8.829489	14.56506	10.95996	11.623	6.517596
17449793	Mir1961	microRNA 1961	2.778064	2.307179	2.486472	2.226535	2.160297	2.374938
17484261	Mir1962	microRNA 1962	4.214642	3.612431	3.703322	3.587453	3.703322	3.633626
17488713	Mir1963	microRNA 1963	27.72701	27.72701	31.58746	32.29435	31.89437	34.48483
17476186	Mir1964	microRNA 1964	15.87693	17.32184	19.42241	15.70139	12.3752	14.69354
17506718	Mir1967	microRNA 1967	2.566478	2.648233	2.646526	1.947428	2.438172	2.473956
17507571	Mir1968	microRNA 1968	8.991925	5.000561	6.218859	7.642793	7.820929	5.652935
17502084	Mir1969	microRNA 1969	6.117703	4.330279	3.476342	4.448318	3.654996	3.578661
17255556	Mir196a-1	microRNA 196a-1	3.987792	3.883991	3.699499	5.249063	4.407284	5.350783
17315505	Mir196a-2	microRNA 196a-2	12.08847	10.30448	10.34635	12.83614	9.310251	10.95153
17544687	Mir1970	microRNA 1970	8.660258	6.455058	4.619382	5.288065	5.699242	4.314849
17308815	Mir1971	microRNA 1971	2.760309	2.941618	3.521378	2.857236	3.713836	2.765442
17230750	Mir1981	microRNA 1981	14.14294	15.4371	18.72197	18.21809	18.49585	20.1383
17291026	Mir1983	microRNA 1983	2.647646	2.528812	2.578282	3.04867	3.206679	3.047698
17524722	Mir199a-1	microRNA 199a-1	3.374608	2.703016	2.684791	2.577192	2.919376	3.339703
17369787	Mir199b	microRNA 199b	2.522772	1.910433	2.653966	2.127693	2.941924	2.216679
17302636	Mir19a	microRNA 19a	2.518125	2.239691	2.188512	2.606909	2.314082	2.763468
17541699	Mir19b-2	microRNA 19b-2	2.021982	1.964565	1.77617	2.653856	2.853752	2.207189
17380762	Mir1a-1	microRNA 1a-1	1.851571	2.070774	1.973354	1.896908	1.791155	2.14481
17352695	Mir1a-2	microRNA 1a-2	2.114402	1.870637	1.964797	2.157484	2.263492	1.769571
17348398	Mir1b	microRNA 1b	11.5933	12.27422	11.5933	10.97158	10.48228	12.56135
17433973	Mir200a	microRNA 200a	4.319397	4.165299	3.107081	3.703322	5.453226	3.420649
17433975	Mir200b	microRNA 200b	6.222913	5.163324	6.193994	5.196931	5.595744	5.914188
17470794	Mir200c	microRNA 200c	6.376921	5.301608	5.787378	5.64213	5.517214	6.110498
17542115	Mir201	microRNA 201	2.130925	2.08174	2.08174	1.896028	2.020413	2.463074
17497509	Mir202	microRNA 202	5.001566	4.368022	4.980222	5.41875	5.43018	4.794067
17279297	Mir203	microRNA 203	2.946312	2.453719	2.036469	2.52876	2.782767	2.172702
17358210	Mir204	microRNA 204	2.204605	2.131886	2.00215	1.941713	2.015014	1.697545
17231272	Mir205	microRNA 205	3.2375	3.00297	2.954635	3.079444	3.453622	2.540637
17211365	Mir206	microRNA 206	1.785416	1.909679	2.160103	1.857729	2.188921	2.69785
17306575	Mir208a	microRNA 208a	2.283917	2.338543	2.743086	2.334074	2.709018	2.49037
17306623	Mir208b	microRNA 208b	2.188921	2.019741	2.170368	2.096307	2.188921	2.334591
17302638	Mir20a	microRNA 20a	3.409665	2.322229	2.67402	2.610688	3.331888	2.481049
17541701	Mir20b	microRNA 20b	5.625066	2.676208	2.261281	2.659804	3.197069	2.015461
17497811	Mir210	microRNA 210	31.09369	48.13619	44.98244	43.75603	41.53081	48.42399
17478862	Mir211	microRNA 211	4.01461	3.657429	3.640433	3.729055	4.545881	3.729055
17252878	Mir212	microRNA 212	9.848541	11.04055	8.394242	9.015828	9.535377	10.01514
17521041	Mir2136	microRNA 2136	2.872506	2.159519	2.293228	2.953407	5.538364	2.953407
17535471	Mir2137	microRNA 2137	27.69781	47.82656	41.42788	33.72181	22.97161	33.34134
17220531	Mir215	microRNA 215	3.878019	3.263862	2.855058	5.969044	3.962909	3.878019
17247944	Mir216a	microRNA 216a	1.981345	2.578461	2.404355	2.451407	2.388939	2.344077
17247937	Mir216b	microRNA 216b	2.67402	2.326337	2.775639	3.19076	3.127175	2.422203
17247946	Mir217	microRNA 217	2.888659	2.027483	2.589637	2.360398	2.483268	2.169782
17437353	Mir218-1	microRNA 218-1	4.217769	5.668865	7.11222	4.325	5.309367	5.528201
17248474	Mir218-2	microRNA 218-2	8.820864	11.87191	10.66166	10.55043	9.036036	10.37292
17343733	Mir219a-1	microRNA 219a-1	10.86977	8.8556	6.501353	7.814535	8.576328	7.179682
17383520	Mir219a-2	microRNA 219a-2	9.396803	9.475749	8.347765	8.420524	7.13622	9.310251
17252906	Mir22	microRNA 22	20.47242	25.37929	23.91692	25.29447	27.77914	24.01011
17540451	Mir221	microRNA 221	3.402158	2.35365	2.606909	2.606909	3.030719	3.077716
17540453	Mir222	microRNA 222	2.774927	2.662571	2.514898	2.982806	1.834148	3.177297
17536383	Mir223	microRNA 223	4.876649	5.952311	6.062866	5.48933	5.564492	5.538057
17542209	Mir224	microRNA 224	3.20408	2.988911	3.062691	3.758221	2.99314	3.040608
17503116	Mir23a	microRNA 23a	5.527741	3.369233	4.673586	3.789113	6.142688	3.127956

17503120	Mir24-2	microRNA 24-2	2.162035	2.580517	2.220785	2.154899	2.669353	2.220785
17454250	Mir25	microRNA 25	15.23154	18.8433	15.50713	15.34323	17.03524	15.11166
17522969	Mir26a-1	microRNA 26a-1	5.658933	6.13354	5.713006	7.571137	6.867381	6.352436
17214232	Mir26b	microRNA 26b	5.418562	3.265106	5.120306	4.681043	4.681043	5.320567
17503118	Mir27a	microRNA 27a	5.338965	6.383289	5.48933	5.48933	7.947549	7.169189
17324471	Mir28a	microRNA 28a	4.167523	5.458558	4.167523	3.499503	6.030928	2.606909
17502525	Mir28b	microRNA 28b	4.081983	4.414622	4.20927	4.20927	5.109174	4.759797
17317044	Mir28c	microRNA 28c	3.104864	2.67402	2.087534	2.304206	4.329889	2.67402
17473111	Mir290a	microRNA 290a	7.704439	4.873472	7.883739	6.075445	5.705843	6.171709
17395129	Mir296	microRNA 296	4.769341	7.27915	7.889315	5.79826	7.889315	3.792791
17366734	Mir297a-2	microRNA 297a-2	2.54039	5.021993	4.235493	5.119561	6.174148	5.329647
17366938	Mir297a-4	microRNA 297a-4	2.851874	3.740212	4.423045	4.658127	6.615912	4.154284
17366916	Mir297b	microRNA 297b	2.443434	3.879901	3.651603	2.885097	2.145494	2.403289
17395131	Mir298	microRNA 298	21.02917	23.39341	23.37979	27.57961	23.54812	27.85858
17278808	Mir299a	microRNA 299a	3.130537	2.699702	2.321199	2.345133	2.955967	2.038023
17283964	Mir299b	microRNA 299b	4.307109	4.566156	4.481091	4.157972	4.681043	5.164147
17465601	Mir29a	microRNA 29a	2.099506	2.060051	2.099506	2.099506	2.08684	1.811067
17465603	Mir29b-1	microRNA 29b-1	1.931725	2.045509	1.883766	2.720327	2.122729	2.060508
17278842	Mir300	microRNA 300	2.925818	2.60877	2.714676	2.714676	2.998789	2.3059
17254679	Mir301	microRNA 301	2.391689	2.391689	2.631563	2.438426	3.040903	2.32284
17328623	Mir301b	microRNA 301b	11.86459	15.68801	18.50098	12.74394	10.71322	9.716387
17402479	Mir302a	microRNA 302a	2.571749	1.749266	1.707127	2.102608	2.367066	2.306204
17402475	Mir302b	microRNA 302b	2.265501	1.86648	1.874298	2.032577	3.139772	1.981757
17402477	Mir302c	microRNA 302c	2.1978	1.795194	2.271429	1.9424	2.329112	1.756009
17402481	Mir302d	microRNA 302d	2.568632	2.590104	2.987089	2.488713	2.987089	2.316537
17244522	Mir3058	microRNA 3058	39.1565	61.04918	58.69762	60.32274	59.6657	59.53681
17237026	Mir3059	microRNA 3059	4.002219	2.847074	2.579301	3.401097	3.130927	3.786907
17246755	Mir3060	microRNA 3060	5.211505	4.115136	5.120306	4.394838	5.5098	3.993683
17249423	Mir3061	microRNA 3061	2.824822	3.544495	3.703322	4.20927	3.339703	3.339703
17259262	Mir3065	microRNA 3065	6.061648	5.922762	6.992896	7.42659	6.992896	7.630777
17274292	Mir3066	microRNA 3066	2.235194	2.268219	2.251677	2.407056	2.080644	2.137671
17276921	Mir3067	microRNA 3067	4.070088	4.764814	4.06975	5.903743	5.61237	5.513391
17282902	Mir3068	microRNA 3068	3.872137	4.385496	4.183731	4.12556	7.89019	5.746327
17278698	Mir3070a	microRNA 3070a	4.553324	3.906184	4.966502	4.49266	6.61229	4.511102
17278700	Mir3070b	microRNA 3070b	5.903743	7.136714	9.903785	3.926544	4.273683	4.03713
17279295	Mir3073a	microRNA 3073a	3.6742	4.019288	5.750072	6.656159	3.546707	3.924938
17293571	Mir3074-1	microRNA 3074-1	3.433096	2.111444	1.955097	2.258852	2.983674	2.139198
17511077	Mir3074-2	microRNA 3074-2	2.674539	2.810311	2.158816	2.368215	2.400109	2.384241
17297746	Mir3075	microRNA 3075	10.05555	10.52093	13.79324	9.851955	9.356896	10.60086
17318426	Mir3079	microRNA 3079	2.162035	2.045637	3.08299	1.990623	2.21324	2.716841
17319736	Mir3080	microRNA 3080	5.120306	4.604358	6.458772	5.679524	5.120306	4.536187
17330600	Mir3081	microRNA 3081	6.656621	6.552566	7.681404	4.210875	3.626254	3.915645
17342674	Mir3083	microRNA 3083	3.355505	3.883991	3.883991	4.794399	3.76957	5.431648
17364826	Mir3085	microRNA 3085	13.49516	11.66157	11.79186	13.97901	14.03853	8.694964
17366006	Mir3086	microRNA 3086	2.23202	2.134177	2.462784	2.503229	2.389436	2.302514
17368067	Mir3087	microRNA 3087	3.749998	2.240111	2.928232	3.441935	3.403101	3.009242
17368760	Mir3088	microRNA 3088	3.170235	6.503336	5.505677	4.166396	4.335685	4.274602
17369266	Mir3089	microRNA 3089	5.023003	5.992176	5.67429	5.954251	3.847977	5.447257
17376683	Mir3090	microRNA 3090	4.681043	4.109406	4.366326	4.801649	4.25173	4.249226
17380747	Mir3091	microRNA 3091	14.7778	21.32701	17.85529	18.62954	18.62954	16.98654
17404462	Mir3092	microRNA 3092	6.171709	6.628535	7.235837	5.677949	6.737204	5.641426
17399008	Mir3093	microRNA 3093	9.379428	7.053699	5.836243	5.418975	5.118851	6.113803
17412846	Mir3094	microRNA 3094	2.200559	4.080568	3.399188	2.708492	3.399188	3.7955
17425701	Mir3095	microRNA 3095	1.96875	2.746758	2.140028	2.224545	2.625296	2.8513
17446066	Mir3096	microRNA 3096 [Source:MGI Symbol;Acc:MGI:4834311]	4.98485	3.483361	3.919338	4.057441	3.501274	4.786596
17436791	Mir3097	microRNA 3097	3.745115	4.951961	3.909679	3.61148	5.174466	3.029669
17463148	Mir3098	microRNA 3098	5.651367	4.053899	6.442051	8.895403	11.40259	4.497926
17473648	Mir3099	microRNA 3099	2.108475	2.072138	2.058809	2.048091	2.542011	2.072138
17211441	Mir30a	microRNA 30a	2.537785	2.699908	2.479759	3.538064	2.523734	3.41681
17317706	Mir30b	microRNA 30b	3.603778	2.662866	2.736326	3.099617	2.65108	3.280714
17429512	Mir30c-1	microRNA 30c-1	3.857377	3.130146	3.774329	4.075876	3.625324	4.040377
17211443	Mir30c-2	microRNA 30c-2	13.41597	13.09033	11.59966	13.43012	13.1234	12.11103
17317708	Mir30d	microRNA 30d	6.343284	8.096397	7.683321	8.572346	6.92983	4.587188
17429514	Mir30e	microRNA 30e	2.458656	3.060357	2.379618	2.725706	3.091806	3.737413
17427131	Mir31	microRNA 31	4.681043	3.400036	4.037102	4.037102	3.764087	4.538545
17488148	Mir3101	microRNA 3101	2.495848	2.300998	2.526938	2.190135	2.514009	2.477852
17493867	Mir3102	microRNA 3102	2.517218	2.635086	2.056513	2.304206	4.180977	2.285991
17485419	Mir3105	microRNA 3105	2.447756	2.501234	2.264904	2.379288	2.413589	2.273807
17507786	Mir3106	microRNA 3106	3.083502	4.20927	3.362397	3.362397	3.476438	3.032232
17508132	Mir3107	microRNA 3107	23.49204	29.93663	32.01842	34.68885	33.64499	31.84864
17505326	Mir3108	microRNA 3108	2.191213	2.126543	2.422354	2.474814	2.640718	2.689746
17518980	Mir3109	microRNA 3109	4.102718	3.310915	4.214992	3.85735	3.532134	2.95001
17534335	Mir3110	microRNA 3110	27.46172	33.3825	28.85981	29.37588	37.5695	28.76116
17425521	Mir32	microRNA 32	3.162313	3.883991	3.494049	3.808044	3.780036	3.514574
17541723	Mir322	microRNA 322	5.528316	5.944024	5.176654	5.275252	7.206956	5.780643
17278814	Mir323	microRNA 323	2.702192	2.67402	2.891263	2.67402	2.547302	2.901965
17251898	Mir324	microRNA 324	57.37499	54.11513	42.31948	56.99052	39.04295	59.65909
17543928	Mir325	microRNA 325	3.280078	2.814366	3.357622	3.152836	3.230528	2.580374
17480588	Mir326	microRNA 326	4.991523	4.926353	6.992896	5.338965	5.98006	3.306558
17305590	Mir327	microRNA 327	3.36277	4.003162	3.460643	3.793238	3.1874	3.460643
17512379	Mir328	microRNA 328	7.187051	22.62789	22.9312	28.92771	17.69684	20.48264
17278818	Mir329	microRNA 329	8.355986	7.278999	8.51685	8.51685	6.614399	9.270197
17313531	Mir33	microRNA 33	2.3514	2.953407	2.729109	2.613295	2.505989	2.253629
17474527	Mir330	microRNA 330	13.02607	13.06568	14.83569	10.21396	10.93067	10.82189

17456963	Mir335	microRNA 335	2.406006	2.384538	2.037514	3.198377	3.605327	2.395206
17278692	Mir337	microRNA 337	2.671611	2.738489	2.347378	3.396573	2.782767	2.782767
17272969	Mir338	microRNA 338	23.1593	29.93186	22.75939	20.60653	25.87165	25.69917
17454414	Mir339	microRNA 339	18.28933	24.21837	17.16526	17.16526	14.2072	22.76554
17249108	Mir340	microRNA 340	4.299921	4.687537	4.372899	4.607327	4.80095	3.774198
17278749	Mir341	microRNA 341	16.14662	20.34186	15.23914	20.71523	16.02742	22.2279
17278612	Mir342	microRNA 342	6.742577	6.612565	8.079242	7.793493	9.26204	6.295801
17491728	Mir344	microRNA 344	4.207141	3.01812	3.342436	2.770315	2.821398	3.047487
17491739	Mir344-2	microRNA 344-2	3.220199	2.497717	2.24968	3.112945	2.363689	2.32152
17491724	Mir344b	microRNA 344b	2.162035	1.883988	2.045509	2.160492	2.249634	2.130098
17491726	Mir344c	microRNA 344c	3.383954	2.00104	2.77287	2.458673	2.492598	2.285991
17491697	Mir344d-1	microRNA 344d-1	3.335007	2.619098	2.498271	3.411627	4.977426	3.258505
17491699	Mir344d-2	microRNA 344d-2	24.36858	21.47997	17.94326	21.46791	22.84236	30.78147
17491712	Mir344d-3	microRNA 344d-3	2.246486	2.462698	3.105122	2.274785	2.224067	2.256802
17491716	Mir344e	microRNA 344e	3.136097	2.162755	2.529584	2.162755	2.213854	2.147547
17491718	Mir344h-1	microRNA 344h-1	2.67402	2.67402	2.411465	2.84966	2.707272	2.65962
17491720	Mir344h-1	microRNA 344h-1	2.67402	2.67402	2.411465	2.84966	2.707272	2.65962
17491749	Mir344i	microRNA 344i	6.769926	10.35539	6.943774	8.171381	8.587213	6.786417
17278626	Mir345	microRNA 345	20.56587	24.92342	33.39893	30.96336	21.27799	19.72386
17298895	Mir346	microRNA 346	6.992896	5.901002	9.45364	9.161285	9.804201	12.22523
17423207	Mir3471-1	microRNA 3471-1	8.863583	8.467112	9.790958	7.723582	6.786934	6.261855
17545897	Mir3473a	microRNA 3473a	3.920017	2.342388	2.523157	2.451203	3.606376	2.434862
17232910	Mir3473b	microRNA 3473b	2.162035	1.759262	3.201704	2.272563	2.741832	2.606909
17231114	Mir3473c	microRNA 3473c	25.85856	22.57697	29.03538	27.55897	41.22764	29.96113
17513006	Mir3473d	microRNA 3473d	21.74573	25.9079	21.66854	27.90651	23.34724	23.96123
17538150	Mir3475	microRNA 3475	6.54104	5.556129	5.211758	8.360852	4.914314	5.095769
17421865	Mir34a	microRNA 34a	3.242845	4.094877	3.565489	3.434071	3.193548	3.431906
17526980	Mir34b	microRNA 34b	12.19561	11.37055	11.37055	10.66802	10.48228	8.181413
17526978	Mir34c	microRNA 34c	2.447112	2.406089	3.352413	2.544567	2.414727	3.206546
17230223	Mir350	microRNA 350	3.145371	3.519011	2.959657	3.450439	5.379157	4.002774
17544265	Mir361	microRNA 361	3.286997	2.749025	1.991203	2.665562	2.592026	2.289734
17539822	Mir362	microRNA 362	2.67402	2.420844	2.844786	3.034124	2.844786	2.868706
17541695	Mir363	microRNA 363	2.390098	4.191481	3.402417	3.983675	3.367061	4.131656
17323040	Mir365-1	microRNA 365-1	4.351673	3.9495	6.00157	4.590019	3.256066	4.640982
17253837	Mir365-2	microRNA 365-2	3.453741	3.24973	3.201615	3.402228	3.463834	3.800818
17402483	Mir367	microRNA 367	2.266427	1.832051	2.04846	1.898066	2.282208	2.0702
17278755	Mir370	microRNA 370	2.614455	2.665119	2.680849	2.902226	3.214	2.85999
17543815	Mir374b	microRNA 374b	3.253765	2.660025	2.714112	2.738166	2.537398	2.782111
17536995	Mir374c	microRNA 374c	2.085047	1.754682	2.058381	2.304206	2.085047	2.072138
17224395	Mir375	microRNA 375	27.2811	44.20568	28.38034	27.91812	26.20663	32.39008
17278840	Mir376a	microRNA 376a	2.36079	1.895055	2.394625	2.301541	3.00424	2.36079
17278838	Mir376b	microRNA 376b	2.553525	2.535025	2.014497	2.707554	2.156258	2.239179
17278834	Mir376c	microRNA 376c	1.942857	2.032225	2.035749	2.083357	2.592169	2.208581
17278882	Mir377	microRNA 377	4.160913	2.893609	2.598412	3.266487	3.485462	3.557146
17354755	Mir378a	microRNA 378a	1.966323	2.944025	2.944025	2.67402	2.458673	2.463808
17254976	Mir378b	microRNA 378b	37.33353	44.85043	38.01693	36.7693	38.48088	42.58933
17245661	Mir378d	microRNA 378d	6.992896	8.614577	10.1025	9.972464	3.972811	7.169189
17278803	Mir379	microRNA 379	2.736003	2.076466	2.304206	2.322567	2.657372	2.250663
17278810	Mir380	microRNA 380	2.714356	2.206959	2.798997	2.515805	2.773774	2.288147
17278844	Mir381	microRNA 381	2.953079	2.67402	3.078548	3.502318	3.737102	2.834747
17278852	Mir382	microRNA 382	2.67402	2.739476	2.739476	2.905628	2.702285	2.233057
17508846	Mir383	microRNA 383	4.961169	4.993322	6.992896	4.765475	4.407132	4.929051
17543918	Mir384	microRNA 384	2.072138	2.072138	2.034494	1.996731	2.177149	2.060979
17294680	Mir3961	microRNA 3961	3.200373	5.967472	9.572329	6.804693	6.14388	7.010513
17221010	Mir3962	microRNA 3962	11.20577	4.685393	6.839496	8.466701	5.10001	7.602901
17411145	Mir3963	microRNA 3963	2.11515	1.894504	1.687234	2.121096	1.915605	1.879241
17316268	Mir3964	microRNA 3964	2.21163	2.060051	2.596089	2.349233	2.622513	2.815966
17443108	Mir3965	microRNA 3965	2.305964	2.19249	1.95339	2.495831	2.883098	2.293197
17236798	Mir3966	microRNA 3966	2.396751	2.822728	2.822728	2.571607	2.877668	2.312607
17367531	Mir3967	microRNA 3967	2.425311	2.722987	2.267653	2.735434	2.450371	2.384902
17271905	Mir3968	microRNA 3968	10.90298	10.0202	14.45473	9.8815	10.46355	9.741136
17449427	Mir3969	microRNA 3969	2.162035	2.040157	2.111883	2.123317	2.885197	2.044985
17358719	Mir3970	microRNA 3970	2.438747	2.344028	2.214944	2.226998	2.652587	2.198653
17278886	Mir409	microRNA 409	11.88574	14.68641	13.13724	15.36845	12.83926	12.09828
17278806	Mir411	microRNA 411	2.555862	2.512163	2.164299	2.466012	2.368888	2.507622
17543813	Mir421	microRNA 421	3.495382	2.174901	2.691331	4.682049	2.231247	2.283141
17266196	Mir423	microRNA 423	4.541598	5.11456	4.544653	6.29218	6.88263	7.323839
17521856	Mir425	microRNA 425	13.02011	12.8791	13.02011	13.90903	15.02924	15.91737
17433968	Mir429	microRNA 429	10.2294	8.51685	8.266061	9.235179	8.566109	8.51685
17278702	Mir431	microRNA 431	8.296024	10.41629	5.513506	6.980401	5.051844	8.729748
17278711	Mir432	microRNA 432	30.78659	25.22776	23.38352	23.38352	28.00728	25.07469
17278709	Mir434	microRNA 434	2.490974	2.443434	2.406673	2.380179	2.51077	2.481995
17538439	Mir448	microRNA 448	4.540496	4.699151	3.624294	3.624294	3.898043	3.674658
17290005	Mir449a	microRNA 449a	3.054508	4.047357	3.633802	3.575339	3.714582	3.842672
17290003	Mir449b	microRNA 449b	1168.401	1406.545	1221.747	1240.297	1024.859	1209.16
17290001	Mir449c	microRNA 449c	2.600484	2.9716	2.715673	2.54762	2.676708	2.593535
17541717	Mir450-1	microRNA 450-1	2.508144	2.953407	2.045509	2.173289	2.143829	2.255364
17541719	Mir450-2	microRNA 450-2	4.529777	4.428107	5.105987	4.772814	5.844015	4.066422
17541715	Mir450b	microRNA 450b	1.799679	1.879006	2.179006	1.728703	2.509117	2.0388
17253450	Mir451a	microRNA 451a	6.076708	4.401301	4.819154	4.38428	4.25173	4.20927
17542211	Mir452	microRNA 452	2.406006	2.139999	2.220785	1.951347	2.323436	2.182165
17278876	Mir453	microRNA 453	3.642932	4.727246	4.169574	4.185268	4.708541	3.25363
17414727	Mir455	microRNA 455	3.923687	4.939175	4.163134	4.23702	4.671449	4.754884
17542076	Mir463	microRNA 463	2.220785	2.197556	2.067017	2.220785	2.561165	1.871896

17542096	Mir465	microRNA 465	2.239691	2.239691	2.066043	2.239691	2.646508	2.41185
17542090	Mir465b-1	microRNA 465b-1	2.459645	2.473048	2.549298	2.640956	2.188921	2.815654
17542094	Mir465b-1	microRNA 465b-1	2.459645	2.473048	2.549298	2.640956	2.188921	2.815654
17542088	Mir465c-1	microRNA 465c-1	2.308011	1.898013	2.126278	2.041925	2.336712	2.257319
17542092	Mir465c-1	microRNA 465c-1	2.308011	1.898013	2.126278	2.041925	2.336712	2.257319
17366856	Mir466b-2	microRNA 466b-2	2.406006	1.512516	2.464457	2.304206	2.295073	2.242814
17366880	Mir466b-3	microRNA 466b-3	2.406006	1.759262	2.406006	2.304206	1.930454	3.628391
17366742	Mir466b-4	microRNA 466b-4	2.448198	1.997659	2.202573	2.304206	2.188921	2.406006
17366796	Mir466b-4	microRNA 466b-4	2.448198	1.997659	2.202573	2.304206	2.188921	2.406006
17366832	Mir466b-4	microRNA 466b-4	2.448198	1.997659	2.202573	2.304206	2.188921	2.406006
17366868	Mir466b-4	microRNA 466b-4	2.448198	1.997659	2.202573	2.304206	2.188921	2.406006
17366808	Mir466b-5	microRNA 466b-5	2.448198	1.997659	2.202573	2.304206	2.188921	2.406006
17366844	Mir466b-5	microRNA 466b-5	2.448198	1.997659	2.202573	2.304206	2.188921	2.406006
17366774	Mir466c-2	microRNA 466c-2	3.362397	2.458571	3.7126	2.953407	3.059021	2.978797
17366784	Mir466c-2	microRNA 466c-2	3.362397	2.458571	3.7126	2.953407	3.059021	2.978797
17366820	Mir466c-2	microRNA 466c-2	3.362397	2.458571	3.7126	2.953407	3.059021	2.978797
17366918	Mir466d	microRNA 466d	31.62777	31.62777	50.31268	21.29481	24.59375	36.52874
17366752	Mir466e	microRNA 466e	3.55308	2.042831	3.161304	3.043961	2.705096	3.380227
17366764	Mir466e	microRNA 466e	3.55308	2.042831	3.161304	3.043961	2.705096	3.380227
17288440	Mir466f-4	microRNA 466f-4	11.95971	17.46809	13.13988	10.39386	10.21941	10.66166
17366930	Mir466g	microRNA 466g	2.919335	1.734945	1.734945	2.471265	2.112469	2.262237
17366932	Mir466h	microRNA 466h	44.39176	36.73975	40.61751	33.14157	29.72717	49.52963
17285454	Mir466i	microRNA 466i	1.942857	1.838157	1.772346	1.876742	2.009895	2.173711
17233663	Mir466j	microRNA 466j	19.36178	28.22771	32.62803	19.40854	26.5966	18.62954
17366936	Mir466l	microRNA 466l	4.212276	2.723251	2.248776	2.743733	2.687566	2.684643
17366926	Mir466n	microRNA 466n	2.909578	2.374477	3.157122	3.432334	4.100842	3.102067
17366736	Mir466o	microRNA 466o	3.005385	1.886877	2.291036	2.209393	2.56518	1.969282
17366888	Mir466p	microRNA 466p	2.363066	2.019083	2.116191	2.08405	2.685815	2.229932
17366750	Mir467a-1	microRNA 467a-1	2.726386	3.04774	2.623077	4.078984	3.561266	2.35819
17366762	Mir467a-1	microRNA 467a-1	2.726386	3.04774	2.623077	4.078984	3.561266	2.35819
17366794	Mir467a-1	microRNA 467a-1	2.726386	3.04774	2.623077	4.078984	3.561266	2.35819
17366806	Mir467a-1	microRNA 467a-1	2.726386	3.04774	2.623077	4.078984	3.561266	2.35819
17366830	Mir467a-1	microRNA 467a-1	2.726386	3.04774	2.623077	4.078984	3.561266	2.35819
17366842	Mir467a-1	microRNA 467a-1	2.726386	3.04774	2.623077	4.078984	3.561266	2.35819
17366854	Mir467a-1	microRNA 467a-1	2.726386	3.04774	2.623077	4.078984	3.561266	2.35819
17366866	Mir467a-1	microRNA 467a-1	2.726386	3.04774	2.623077	4.078984	3.561266	2.35819
17366782	Mir467a-3	microRNA 467a-3	3.258279	4.216336	3.751636	5.567694	4.609244	3.355412
17366818	Mir467a-3	microRNA 467a-3	3.258279	4.216336	3.751636	5.567694	4.609244	3.355412
17366878	Mir467a-3	microRNA 467a-3	3.258279	4.216336	3.751636	5.567694	4.609244	3.355412
17366772	Mir467b	microRNA 467b	2.408809	3.029732	2.522685	4.194503	3.214936	2.294055
17366740	Mir467c	microRNA 467c	60.25295	58.45076	70.86395	57.55981	52.21708	85.07421
17366894	Mir467d	microRNA 467d	2.24862	2.453855	2.165034	2.931421	3.343085	2.599619
17366886	Mir467e	microRNA 467e	4.871378	10.54794	16.71336	11.50086	6.655145	7.117053
17264822	Mir467f	microRNA 467f	3.358949	2.242115	2.362935	2.334009	2.710446	2.560437
17522812	Mir467h	microRNA 467h	3.165931	5.531305	5.32171	3.038333	5.202626	2.824744
17467988	Mir468	microRNA 468	2.51333	2.51333	2.51333	2.458009	2.988062	2.426135
17542086	Mir470	microRNA 470	2.584598	2.562443	2.808247	2.449284	2.657022	2.362346
17542072	Mir471	microRNA 471	2.098866	1.998891	2.007027	1.898013	2.11804	2.136383
17498158	Mir483	microRNA 483	37.34078	53.94772	44.77837	34.76443	35.48605	28.77931
17278858	Mir485	microRNA 485	2.688945	2.67402	2.810993	2.67402	2.732554	2.67402
17500060	Mir486	microRNA 486	11.70709	15.36281	16.43341	12.1962	13.27031	14.84412
17278846	Mir487b	microRNA 487b	3.062691	2.921339	2.688684	3.145894	3.897449	3.145894
17218541	Mir488	microRNA 488	2.614038	1.920457	2.060608	2.372256	2.397449	2.206867
17464571	Mir489	microRNA 489	2.406006	2.543015	2.304574	2.755015	2.8481	2.4713
17457318	Mir490	microRNA 490	2.160477	2.038418	2.116807	2.953407	2.481977	2.435402
17415290	Mir491	microRNA 491	2.771352	2.583846	2.596971	2.726311	2.477302	2.701798
17278690	Mir493	microRNA 493	2.997521	3.409287	2.536677	3.425869	3.072345	3.689589
17278820	Mir494	microRNA 494	4.635131	4.801649	4.264747	5.76324	4.801649	6.105926
17278830	Mir495	microRNA 495	2.125379	2.281148	2.083357	1.779707	2.169737	2.285991
17278880	Mir496a	microRNA 496a	3.070131	2.791151	3.009972	2.822728	2.443569	2.984564
17252009	Mir497	microRNA 497	5.379157	5.214721	4.210233	4.492411	4.658902	4.603624
17378438	Mir499	microRNA 499	2.481049	2.502934	3.618245	2.606909	2.481049	2.705453
17539818	Mir500	microRNA 500	3.062691	3.344661	2.896519	2.844391	2.479312	3.638465
17539820	Mir501	microRNA 501	3.136618	3.078889	3.822006	2.597475	2.586749	3.185147
17541946	Mir504	microRNA 504	2.360545	2.338024	2.717123	3.138576	2.396867	3.079572
17542021	Mir505	microRNA 505	98.36138	165.954	130.5845	147.6277	167.2845	143.0276
17542119	Mir509	microRNA 509	2.038164	2.038164	1.947158	2.295932	2.038164	2.038164
17438494	Mir5098	microRNA 5098	3.504771	8.075659	6.603496	5.428562	5.48933	9.68163
17275060	Mir5099	microRNA 5099	3250.171	3904.927	3389.813	3705.365	2979.408	3677.705
17250507	Mir5100	microRNA 5100	17.41852	13.3185	15.44063	17.13376	14.66322	17.44897
17222070	Mir5103	microRNA 5103	19.59427	14.66546	25.8634	17.09568	15.14018	21.31785
17231600	Mir5104	microRNA 5104	2.813274	2.536765	3.901043	3.135358	2.782767	3.754654
17424876	Mir5106	microRNA 5106	3.584247	3.274853	3.120182	3.120182	3.204946	3.192685
17233767	Mir5108	microRNA 5108	5.619688	5.303777	4.990658	4.815448	6.150271	5.48933
17367137	Mir511	microRNA 511	6.034859	8.142659	7.490749	7.993349	6.943389	10.58633
17267184	Mir5110	microRNA 5110	3.180426	3.040166	3.368649	2.918911	4.134549	2.918911
17352196	Mir5112	microRNA 5112	199.491	157.1088	192.0649	233.7561	171.1989	216.9442
17326239	Mir5118	microRNA 5118	3.841581	3.975841	5.211505	4.406124	7.586529	3.549756
17255947	Mir5119	microRNA 5119	3.260696	2.652477	3.362397	3.282944	2.618027	3.664306
17424902	Mir5120	microRNA 5120	11.53471	16.92472	15.34153	13.07111	18.79699	11.68163
17424088	Mir5123	microRNA 5123	28.15931	24.36114	33.92013	31.00158	24.19908	24.21367
17286717	Mir5124a	microRNA 5124a	8.068945	28.22184	18.27526	19.28972	13.45658	14.54983
17334147	Mir5125	microRNA 5125	41.62793	114.8455	121.0895	88.69925	90.19028	101.2277
17355728	Mir5127	microRNA 5127	5.441823	3.237836	2.760482	3.227574	3.001097	3.056202

17384657	Mir5128	microRNA 5128	4.585757	4.58312	3.683175	4.265515	4.426695	3.909733
17527982	Mir5133	microRNA 5133	12.96913	16.59343	20.38082	21.10189	15.67442	16.68477
17282019	Mir5135	microRNA 5135	77.30555	111.5116	94.65652	102.3147	93.78011	104.0476
17539826	Mir532	microRNA 532	3.886631	3.883991	5.029029	4.157165	5.948599	4.157165
17278848	Mir539	microRNA 539	5.438882	7.200165	5.528201	6.566752	5.187826	4.361608
17278694	Mir540	microRNA 540	11.24366	10.69823	12.16285	10.29577	12.18361	10.6141
17278884	Mir541	microRNA 541	3.044953	2.976032	2.617846	3.17151	2.606023	2.372454
17541721	Mir542	microRNA 542	15.96201	12.50653	12.81063	15.04977	16.92143	16.0999
17278828	Mir543	microRNA 543	2.933575	3.365125	3.340676	3.254757	2.493773	3.28743
17278850	Mir544	microRNA 544	2.477474	1.501589	1.869432	2.083357	1.89302	2.164659
17542117	Mir547	microRNA 547	2.768875	2.814171	2.583828	2.41972	2.579784	2.23301
17396544	Mir551b	microRNA 551b	11.84634	10.72466	10.99138	12.35189	12.32803	13.76898
17325723	Mir568	microRNA 568	80.12497	112.6357	105.9963	99.3281	87.91516	77.23485
17437702	Mir574	microRNA 574	46.62268	67.44595	74.28968	74.77686	67.98808	81.89083
17289790	Mir582	microRNA 582	2.419233	2.124687	2.536712	2.278383	2.238031	2.425933
17465296	Mir592	microRNA 592	5.915746	6.287864	5.669769	6.52795	4.641529	6.411314
17301245	Mir598	microRNA 598	15.65238	13.74781	11.56746	17.4312	15.37101	15.37996
17316432	Mir599	microRNA 599	2.418798	1.930869	2.256098	2.636237	2.974341	2.761324
17315536	Mir615	microRNA 615	5.669769	6.213947	5.669769	5.463858	5.669769	5.927649
17538312	Mir652	microRNA 652	4.572712	4.831262	5.705487	4.909037	4.726755	4.854525
17464569	Mir653	microRNA 653	2.217709	2.021099	2.113245	1.916667	2.548538	2.072425
17278836	Mir654	microRNA 654	5.335857	5.780603	5.066116	4.300427	4.085266	4.20927
17278696	Mir665	microRNA 665	10.00133	18.893	30.83271	13.24458	10.91583	11.94512
17278826	Mir666	microRNA 666	14.85658	14.04418	11.27363	13.86937	9.21044	11.75791
17278832	Mir667	microRNA 667	5.725414	5.977864	6.886877	6.420519	7.284601	5.125988
17278856	Mir668	microRNA 668	5.635408	7.664279	7.346209	5.905176	6.030677	5.218915
17366754	Mir669a-1	microRNA 669a-1	3.300329	2.111576	1.992238	1.898013	2.147041	2.606909
17366766	Mir669a-1	microRNA 669a-1	3.300329	2.111576	1.992238	1.898013	2.147041	2.606909
17366776	Mir669a-1	microRNA 669a-1	3.300329	2.111576	1.992238	1.898013	2.147041	2.606909
17366786	Mir669a-1	microRNA 669a-1	3.300329	2.111576	1.992238	1.898013	2.147041	2.606909
17366798	Mir669a-1	microRNA 669a-1	3.300329	2.111576	1.992238	1.898013	2.147041	2.606909
17366822	Mir669a-1	microRNA 669a-1	3.300329	2.111576	1.992238	1.898013	2.147041	2.606909
17366834	Mir669a-1	microRNA 669a-1	3.300329	2.111576	1.992238	1.898013	2.147041	2.606909
17366858	Mir669a-1	microRNA 669a-1	3.300329	2.111576	1.992238	1.898013	2.147041	2.606909
17366870	Mir669a-1	microRNA 669a-1	3.300329	2.111576	1.992238	1.898013	2.147041	2.606909
17366882	Mir669a-1	microRNA 669a-1	3.300329	2.111576	1.992238	1.898013	2.147041	2.606909
17366920	Mir669a-1	microRNA 669a-1	3.300329	2.111576	1.992238	1.898013	2.147041	2.606909
17366908	Mir669a-2	microRNA 669a-2	3.300329	2.111576	1.992238	1.898013	2.147041	2.606909
17366744	Mir669a-3	microRNA 669a-3	11.45686	5.020601	6.629775	11.24787	6.109524	10.39999
17366724	Mir669b	microRNA 669b	2.472174	2.731437	2.762664	2.773543	2.670926	2.309227
17366902	Mir669c	microRNA 669c	3.767324	2.794655	4.336587	3.737983	3.488701	3.569742
17366728	Mir669d	microRNA 669d	2.793396	3.040672	3.489402	6.918935	3.729055	3.536789
17366722	Mir669e	microRNA 669e	2.543227	2.812202	2.595532	2.493825	2.998394	2.821769
17366720	Mir669f	microRNA 669f	20.09062	25.96921	31.51988	24.07544	28.93954	27.20123
17366756	Mir669g	microRNA 669g	2.049526	3.060697	2.459866	2.479656	2.493116	2.619461
17366944	Mir669h	microRNA 669h	5.624637	4.815882	2.429383	5.796733	4.536281	4.20927
17366942	Mir669i	microRNA 669i	2.162035	1.974312	2.074667	1.728703	1.816032	1.855541
17366760	Mir669j	microRNA 669j	2.772121	3.362374	2.835946	2.304206	1.92635	2.195075
17366746	Mir669k	microRNA 669k	2.406006	2.251271	2.617573	2.848219	2.363361	2.897824
17366732	Mir669l	microRNA 669l	3.324091	3.247366	2.91093	2.22308	3.009972	3.079914
17366922	Mir669m-1	microRNA 669m-1	8.272423	7.982552	9.622355	9.035785	16.20885	10.62006
17366924	Mir669m-2	microRNA 669m-2	3.449387	2.920368	3.509462	2.953407	2.970509	2.902045
17401999	Mir669n	microRNA 669n	1.982705	11.69135	18.88004	5.690952	3.341579	8.03039
17366928	Mir669o	microRNA 669o	2.850332	5.677123	8.896883	5.309183	9.842399	6.15876
17366810	Mir669p-1	microRNA 669p-1	3.067642	1.81275	1.85378	1.829133	1.941511	2.541693
17366846	Mir669p-1	microRNA 669p-1	3.067642	1.81275	1.85378	1.829133	1.941511	2.541693
17388594	Mir670	microRNA 670	4.360671	4.485752	4.042058	4.443018	4.084162	4.240017
17543850	Mir672	microRNA 672	5.541897	7.490905	7.060596	4.814046	6.171709	6.171709
17278688	Mir673	microRNA 673	37.89984	46.89525	51.57714	47.40344	49.94506	45.24825
17374468	Mir674	microRNA 674	21.22967	12.21879	10.66876	12.46473	11.83051	11.50086
17498126	Mir675	microRNA 675	11.25536	11.97264	12.52171	14.07917	31.82459	15.84384
17536515	Mir676	microRNA 676	2.715598	2.738432	3.312797	2.789758	2.552286	2.915958
17238293	Mir677	microRNA 677	3.285379	5.391923	5.635252	6.405406	6.529398	10.00702
17278822	Mir679	microRNA 679	3.935345	3.831077	3.615387	4.20927	3.966482	5.554665
17463577	Mir680-1	microRNA 680-1	4.550137	4.270485	10.08158	5.973059	3.112319	4.063661
17538354	Mir680-2	microRNA 680-2	185.2706	222.5306	194.9094	194.7798	217.3717	198.3342
17280727	Mir680-3	microRNA 680-3	9.337394	7.463022	9.462819	7.584899	9.645527	6.560065
17281635	Mir681	microRNA 681	3.033682	3.241923	3.448311	4.624348	3.81642	3.255886
17288695	Mir682	microRNA 682	210.1739	285.8432	340.5989	300.9291	436.295	381.606
17292527	Mir683-1	microRNA 683-1	12.83276	14.25593	16.84794	15.30966	15.68518	12.66671
17292529	Mir683-1	microRNA 683-1	12.83276	14.25593	16.84794	15.30966	15.68518	12.66671
17387353	Mir684-1	microRNA 684-1	14.31732	14.92263	14.92263	15.37101	14.92263	13.53985
17411749	Mir684-2	microRNA 684-2	10.54422	10.64755	9.799037	10.79723	10.48228	10.35948
17308598	Mir687	microRNA 687	2.611611	2.67402	2.67402	2.315943	3.441744	2.627517
17322287	Mir688	microRNA 688	6.236601	7.85777	7.519726	7.76879	8.698882	10.35101
17329525	Mir690	microRNA 690	1.9527	2.036808	2.232716	2.198485	2.883798	2.274485
17331516	Mir691	microRNA 691	3.396032	3.101293	3.235458	3.551405	3.14476	3.729055
17340629	Mir692-1	microRNA 692-1	1107.814	1247.946	1388.229	1202.574	1001.593	1121.279
17288650	Mir692-3	microRNA 692-3	2256.17	2433.184	2614.537	2351.018	2227.701	2204.751
17418483	Mir692-3	microRNA 692-3	2373.006	2634.439	2927.777	2567.663	2318.651	2356.533
17338041	Mir693	microRNA 693	3.27399	4.057525	4.094564	3.02843	5.120306	3.494655
17355131	Mir694	microRNA 694	6.45837	7.882919	5.912958	6.107661	6.412247	6.808939
17378346	Mir695	microRNA 695	2.953407	3.306947	3.080896	2.75532	3.206635	2.953407
17418304	Mir697	microRNA 697	4.079013	5.675471	3.12042	5.177444	6.293227	5.235945

17440618	Mir701	microRNA 701	2.529408	2.343265	2.436956	2.423026	2.438696	2.261155
17449958	Mir703	microRNA 703	418.8036	486.2451	477.8387	453.5664	490.7961	518.2089
17480290	Mir708	microRNA 708	2.636018	2.420844	2.131132	2.606909	2.188921	2.285991
17503092	Mir709	microRNA 709	4.107128	2.263994	4.01603	3.990003	5.74788	3.719555
17293163	Mir7-1	microRNA 7-1	10.2778	9.826175	8.92177	9.884856	11.01517	17.48408
17509615	Mir710	microRNA 710	2.481049	2.375004	2.423782	2.527183	2.782767	2.430697
17288077	Mir713	microRNA 713	3.77255	4.818854	4.439816	5.409443	5.120306	6.134476
17541693	Mir717	microRNA 717	7.04339	6.367336	6.857962	8.51685	6.844665	8.226849
17479301	Mir7-2	microRNA 7-2	2.933392	3.879606	3.411225	4.934692	3.319647	3.041662
17453771	Mir721	microRNA 721	5.399254	5.662778	5.805821	5.770995	4.619863	5.28605
17542074	Mir741	microRNA 741	2.282461	2.089344	2.162575	1.909626	2.194512	1.975722
17542066	Mir742	microRNA 742	2.481049	2.246595	2.481049	2.383183	3.377978	2.513347
17542062	Mir743	microRNA 743	3.133858	3.425727	3.111521	3.772079	3.263862	3.688643
17542064	Mir743b	microRNA 743b	2.334834	2.641413	2.91802	2.263413	2.503923	2.193478
17264206	Mir744	microRNA 744	3.555543	4.130195	4.364057	3.68532	3.082455	3.755851
17278816	Mir758	microRNA 758	8.042589	9.716387	7.555724	9.417799	8.455675	6.237812
17302271	Mir759	microRNA 759	2.371122	2.588525	2.329919	2.601728	2.820381	2.070846
17409958	Mir760	microRNA 760	19.75999	21.60974	21.06929	20.33002	22.93883	22.30909
17416811	Mir761	microRNA 761	29.00641	48.6322	64.81968	35.04678	34.60287	37.41306
17245431	Mir763	microRNA 763	15.47706	14.46105	16.1446	16.92671	17.29364	19.32505
17538429	Mir764	microRNA 764	4.268059	3.705479	4.211926	4.762998	3.870393	4.353754
17542235	Mir767	microRNA 767	2.211308	2.406006	2.295789	2.805232	2.406006	2.836595
17338741	Mir7b	microRNA 7b	6.681398	6.867905	6.424081	6.171709	7.138149	6.97895
17327253	Mir802	microRNA 802	2.447027	2.367952	2.367952	2.367952	2.782767	2.422976
17262355	Mir804	microRNA 804	2.410229	3.09777	1.878733	2.134562	2.194132	2.53376
17542084	Mir871	microRNA 871	2.456169	2.20062	1.987383	2.293737	2.254426	2.274627
17415467	Mir872	microRNA 872	2.253738	2.39164	2.39164	2.27753	2.258884	2.339143
17423960	Mir873a	microRNA 873a	2.811344	2.495415	2.486248	2.232051	5.222824	2.725347
17293083	Mir874	microRNA 874	16.70028	15.79581	20.50097	21.92037	14.24388	16.01553
17316434	Mir875	microRNA 875	2.139569	1.799941	1.747945	2.111883	2.148232	2.111883
17423958	Mir876	microRNA 876	2.108724	2.060051	1.898013	1.994725	2.473099	2.084296
17344536	Mir877	microRNA 877	5.337781	5.040371	4.799586	5.979356	6.38745	6.497704
17542080	Mir878	microRNA 878	2.406006	2.845082	2.481995	2.350471	2.694075	2.70791
17434653	Mir879	microRNA 879	2.406006	2.332117	1.987631	2.0468	2.332117	2.332117
17542078	Mir880	microRNA 880	2.263602	2.132654	2.152943	2.132654	2.223635	2.139865
17542082	Mir881	microRNA 881	2.82042	1.819144	1.990361	2.169436	2.643703	2.502326
17278789	Mir882	microRNA 882	2.216956	2.165845	2.368609	2.344077	2.188921	2.098415
17542068	Mir883a	microRNA 883a	2.288354	2.343037	2.782053	2.652293	2.685052	2.207311
17542070	Mir883b	microRNA 883b	3.456951	2.97919	3.42929	4.01475	4.394412	4.425775
17399010	Mir9-1	microRNA 9-1	8.296886	6.696791	6.022615	6.540813	6.104191	6.28538
17288946	Mir9-2	microRNA 9-2	2.461333	2.34868	2.174404	2.214637	1.85333	2.214637
17541697	Mir92-2	microRNA 92-2	2.311036	2.961073	2.574781	2.785816	3.952156	3.017597
17406960	Mir92b	microRNA 92b	38.0921	46.07014	32.23129	46.61008	25.4293	34.31173
17454252	Mir93	microRNA 93	2.953407	2.953407	2.953407	2.953407	3.698525	3.625374
17479423	Mir9-3	microRNA 9-3	8.813225	8.498805	7.698994	6.919271	7.988474	6.798988
17465480	Mir96	microRNA 96	3.106521	3.351298	2.872885	2.934918	2.835297	4.072063
17538848	Mir98	microRNA 98	3.241878	5.024883	3.867711	2.839545	4.742343	3.345565
17326700	Mir99a	microRNA 99a	4.681043	4.899587	4.239488	4.462493	4.979221	4.450878
17333705	Mir99b	microRNA 99b	24.18583	31.1636	24.10666	25.40534	28.71634	25.57017
17292329	Mirlet7a-1	microRNA let7a-1	12.80815	10.02382	9.315738	13.0616	15.14395	13.19482
17516410	Mirlet7a-2	microRNA let7a-2	6.858438	5.313196	5.045824	5.335117	3.665907	3.784099
17313860	Mirlet7b	microRNA let7b	2.67402	3.423424	2.758225	2.844155	5.455873	3.188151
17326702	Mirlet7c-1	microRNA let7c-1	5.396148	2.257944	2.930222	2.846324	3.074326	2.449165
17313858	Mirlet7c-2	microRNA let7c-2	2.525554	4.762866	2.922615	3.353947	2.328821	2.841357
17292325	Mirlet7d	microRNA let7d	3.077524	3.732417	3.4988	4.723709	4.330999	4.059241
17333707	Mirlet7e	microRNA let7e	8.592512	4.999348	5.334304	8.618937	6.828175	6.332258
17292327	Mirlet7f-1	microRNA let7f-1	2.23662	1.861493	4.217038	2.943209	3.132859	3.173599
17538846	Mirlet7f-2	microRNA let7f-2	3.733762	2.178281	2.256005	2.843031	1.934177	2.123435
17521157	Mirlet7g	microRNA let7g	3.575859	3.817717	3.290507	3.790084	3.65259	4.587442
17245585	Mirlet7i	microRNA let7i	7.198468	5.29933	5.785333	6.171709	5.614899	6.190861
17491745	Mir344f	microRNA Mir344f	6.017524	4.96998	5.617429	4.562359	2.778969	4.560336
17424762	Msmp	microseminoprotein, prostate associated	7.317141	6.639433	5.821052	8.006379	7.441843	9.040484
17463909	Mgst1	microsomal glutathione S-transferase 1	262.2702	304.2915	284.9253	308.8602	268.2176	285.4294
17397475	Mgst2	microsomal glutathione S-transferase 2	3.574496	4.818854	4.561569	5.767316	5.184159	6.150058
17229403	Mgst3	microsomal glutathione S-transferase 3	31.0504	45.57561	57.38374	35.73981	32.88118	49.11698
17410636	Mttp	microsomal triglyceride transfer protein	401.5261	370.0586	353.8294	351.2514	307.3162	323.7845
17321493	Mcrs1	microspherule protein 1	11.96194	12.30267	15.013	12.36748	11.28692	14.65133
17232853	Mical1	microtubule associated monooxygenase, calponin and LIM domain containing 1	6.727917	8.320902	8.576209	7.027298	6.5986	9.310251
17481863	Mical2	microtubule associated monooxygenase, calponin and LIM domain containing 2	24.82772	20.69284	21.75326	18.88344	19.26152	13.76326
17470468	Mical3	microtubule associated monooxygenase, calponin and LIM domain containing 3	36.14439	31.04158	31.50939	31.25381	25.26993	23.69778
17312920	Micall1	microtubule associated monooxygenase, calponin and LIM domain containing -lik	27.90303	32.16612	28.84161	28.94977	31.89437	27.86148
17511211	Mast1	microtubule associated serine/threonine kinase 1	7.93082	11.03841	8.467112	10.24395	5.549585	9.255879
17428600	Mast2	microtubule associated serine/threonine kinase 2	41.39602	35.91713	42.10559	38.89439	38.89439	32.62306
17510166	Mast3	microtubule associated serine/threonine kinase 3	23.76292	20.02069	29.62021	28.71296	34.98974	30.35453
17295817	Mast4	microtubule associated serine/threonine kinase family member 4	9.57983	11.50086	12.53074	9.95858	10.46326	10.499
17382169	Mastl	microtubule associated serine/threonine kinase-like	3.976971	4.106844	3.80873	4.991073	4.392493	4.392493
17444884	Mtus2	microtubule associated tumor suppressor candidate 2	5.432589	4.806278	4.647678	4.762503	4.762503	5.284877
17346817	Mtcl1	microtubule crosslinking factor 1	9.012641	9.579166	9.234795	9.234795	8.263884	9.234795
17429689	Macf1	microtubule-actin crosslinking factor 1	85.45127	89.2704	93.90434	79.78524	76.07708	72.51764
17375244	Map1a	microtubule-associated protein 1 A	7.255575	6.570531	8.59418	7.794303	8.187653	8.206631
17378337	Map1lc3a	microtubule-associated protein 1 light chain 3 alpha	29.65102	25.4554	32.27175	29.65102	26.49044	30.34443
17506306	Map1lc3b	microtubule-associated protein 1 light chain 3 beta	194.8675	254.5632	310.9179	221.5134	233.9019	216.0693
17506888	Map10	microtubule-associated protein 10	3.417923	2.711705	3.15159	3.722831	3.953115	4.003301
17295482	Map1b	microtubule-associated protein 1B	10.36429	11.32014	10.96011	11.00777	9.035973	10.41665

17502075	Map1s	microtubule-associated protein 1S	40.96103	45.55413	45.62207	41.53081	32.34453	35.10635
17213770	Map2	microtubule-associated protein 2	6.287864	6.287864	6.039755	6.566752	5.713164	5.855125
17522253	Map4	microtubule-associated protein 4	47.34007	62.87872	72.37653	49.00544	66.31025	49.46753
17480515	Map6	microtubule-associated protein 6	5.630917	4.525634	4.539395	5.478155	3.349394	4.611512
17480525	Map6	microtubule-associated protein 6	4.019427	3.597513	3.482154	3.854089	4.392919	3.89178
17231925	Map7	microtubule-associated protein 7	62.35053	55.91409	54.68109	55.92107	51.61935	52.93757
17398460	Map9	microtubule-associated protein 9	4.144506	3.858018	3.430693	4.447917	4.352065	4.11856
17257197	Mapt	microtubule-associated protein tau	6.992896	7.470372	6.992896	7.677252	5.965777	7.081476
17377989	Mapre1	microtubule-associated protein, RP/EB family, member 1	44.79761	43.88178	38.04408	29.75252	23.50588	35.17601
17348933	Mapre2	microtubule-associated protein, RP/EB family, member 2	9.22635	9.732834	12.48635	9.732834	7.495527	10.23806
17435855	Mapre3	microtubule-associated protein, RP/EB family, member 3	70.27891	72.21967	79.3721	77.64171	63.41358	67.36092
17533282	Mid1ip1	Mid1 interacting protein 1 (gastrulation specific G12-like (zebrafish))	30.73861	29.93165	40.16119	37.20385	44.17322	40.66991
17412418	Mdn1	midasin homolog (yeast)	17.69856	19.48848	17.69856	18.11007	17.48457	14.79789
17388284	Mdk	midkine	6.141112	4.897108	5.297237	6.141112	5.292099	6.485285
17539700	Mid1	midline 1	8.715902	8.715902	10.66876	10.63848	7.144683	9.027584
17546762	Mid1	midline 1	45.63725	48.16122	56.22734	52.32687	45.83984	37.92428
17538171	Mid2	midline 2	25.82453	25.73054	33.49445	34.29865	25.24437	23.54355
17235211	Midn	midnolin	39.31751	70.82516	82.17001	69.64945	68.72723	61.56549
17271968	Mif4gd	MIF4G domain containing	28.03778	32.12022	27.63395	29.14325	30.15886	33.38296
17268810	Mien1	migration and invasion enhancer 1	74.04395	74.55122	81.24404	81.68957	76.70294	90.14465
17432790	Miip	migration and invasion inhibitory protein	15.13734	14.6281	14.41071	14.06239	14.8287	15.73014
17492314	Mfge8	milk fat globule-EGF factor 8 protein	44.03656	48.84571	57.34994	38.32676	26.03427	25.48224
17348365	Mib1	mindbomb homolog 1 (Drosophila)	101.84	96.04909	84.52932	88.39544	104.1342	94.33754
17433826	Mib2	mindbomb homolog 2 (Drosophila)	22.01706	21.82183	22.01706	23.13347	22.99885	23.74711
17240840	Mcm9	minichromosome maintenance complex component 9	8.656657	7.504729	7.006627	6.102752	8.535289	7.28137
17381306	Mcm10	minichromosome maintenance deficient 10 (S. cerevisiae)	17.48287	15.96698	15.54264	17.15432	12.43031	15.82003
17468798	Mcm2	minichromosome maintenance deficient 2 mitotin (S. cerevisiae)	15.96798	20.73563	19.702	15.96798	13.77137	13.39423
17221633	Mcm3	minichromosome maintenance deficient 3 (S. cerevisiae)	13.524	15.20643	13.55168	12.55143	12.99361	11.29858
17234519	Mcm3ap	minichromosome maintenance deficient 3 (S. cerevisiae) associated protein	18.75366	17.68568	16.17171	17.76936	17.67588	17.43035
17328432	Mcm4	minichromosome maintenance deficient 4 homolog (S. cerevisiae)	9.531611	9.880198	14.2009	9.078035	8.064807	9.88424
17502583	Mcm5	minichromosome maintenance deficient 5, cell division cycle 46 (S. cerevisiae)	14.76009	10.73984	11.25481	10.17735	9.983876	9.73857
17226550	Mcm6	minichromosome maintenance deficient 6 (MIS5 homolog, S. pombe) (S. cerevisia	17.8454	23.55171	17.69083	15.46173	11.57949	16.07604
17454226	Mcm7	minichromosome maintenance deficient 7 (S. cerevisiae)	15.2678	19.29025	14.61492	14.45503	14.54085	15.01133
17376626	Mcm8	minichromosome maintenance deficient 8 (S. cerevisiae)	6.41278	5.525596	5.726724	6.729177	5.305689	6.466701
17211066	Mcmdc2	minichromosome maintenance domain containing 2	3.692198	3.131774	3.131774	3.326996	3.064751	3.337759
17254835	Mir142hg	Mir142 host gene (non-protein coding) [Source:MGI Symbol;Acc:MGI:1925841]	3.621281	3.263862	2.991647	3.531179	3.658444	3.244711
17354784	Mir143hg	Mir143 and Mir145 host gene (non-protein coding)	8.947905	7.931809	9.526591	8.51685	10.88085	9.514119
17302640	Mir17hg	Mir17 host gene (non-protein coding)	5.21982	5.128333	5.113354	5.895319	5.2123	5.128333
17302617	Mir17hg	Mir17 host gene (non-protein coding) [Source:MGI Symbol;Acc:MGI:1923207]	13.81123	17.23835	14.00248	14.23125	11.90141	16.84957
17302619	Mir17hg	Mir17 host gene (non-protein coding) [Source:MGI Symbol;Acc:MGI:1923207]	8.954979	10.54473	9.403123	7.060792	6.896192	8.534638
17326685	Mir99ahg	Mir99a and Mirlet7c-1 host gene (non-protein coding)	7.921041	7.770675	5.927115	7.403359	7.109017	7.736065
17278860	Mirg	miRNA containing gene	3.703322	4.0103	3.697987	4.257628	3.67229	3.703322
17252272	Mis12	MIS12 homolog (yeast)	26.83307	21.69574	22.38561	15.13105	18.25247	18.78358
17281428	Mis18bp1	MIS18 binding protein 1	3.275193	3.690893	3.85222	3.468039	3.766619	3.935399
17331987	Mis18a	MIS18 kinetochore protein homolog A (S. pombe)	12.85333	13.29655	11.2989	11.69589	10.95578	12.07064
17406846	Msto1	misato homolog 1 (Drosophila)	20.75404	21.79855	22.62256	21.98839	19.43156	23.24309
17252113	Mink1	misshapen-like kinase 1 (zebrafish)	66.45796	89.68532	83.82682	78.11133	67.88213	78.97642
17455971	Mios	missing oocyte, meiosis regulator, homolog (Drosophila)	17.35994	16.44481	18.81837	16.9703	19.77122	19.74027
17466861	Mira	mistral long non-coding RNA	3.334568	3.949035	3.729055	3.729055	3.070173	5.59051
17222396	Mitd1	MIT, microtubule interacting and transport, domain containing 1	23.5155	21.10554	20.07628	30.22771	17.68789	23.5155
17258272	Mrps7	mitchondrial ribosomal protein S7	79.76036	64.33402	62.78422	71.63238	59.46422	69.74317
17346001	Mrps36-ps1	mitichondrial ribosomal protein S36, pseudogene 1	7.709514	8.824044	10.58655	7.186454	6.833051	11.02067
17405119	Mgarp	mitochondria localized glutamic acid rich protein	3.482419	3.524039	3.339703	3.339703	3.69074	3.110594
17230725	Marc1	mitochondrial amidoxime reducing component 1	492.5888	482.8094	462.5835	483.8547	476.9684	509.257
17230735	Marc2	mitochondrial amidoxime reducing component 2	253.3749	331.3082	341.1187	354.0624	353.8809	360.3462
17376480	Mavs	mitochondrial antiviral signaling protein	243.7553	199.0932	211.35	216.39	210.9489	162.894
17458454	Malsu1	mitochondrial assembly of ribosomal large subunit 1	37.16235	33.77936	33.77936	35.19381	33.77936	36.22265
17241013	Mcu	mitochondrial calcium uniporter	95.26603	84.82926	69.46864	71.19283	70.00179	84.15459
17292139	Mcur1	mitochondrial calcium uniporter regulator 1	86.48092	77.29536	86.90396	89.63747	91.95652	96.13368
17233565	Micu1	mitochondrial calcium uptake 1	51.9683	45.99611	45.54087	40.27102	37.27742	34.39412
17307231	Micu2	mitochondrial calcium uptake 2	75.85067	77.47828	65.06095	61.74498	69.748	63.90469
17500644	Micu3	mitochondrial calcium uptake family, member 3	6.358648	5.527319	5.668276	5.955819	5.232644	3.871547
17343035	Mtch1	mitochondrial carrier homolog 1 (C. elegans)	87.38659	84.69471	86.54749	81.02471	84.69471	85.30628
17373086	Mtch2	mitochondrial carrier homolog 2 (C. elegans)	516.3266	488.9896	470.79	446.851	353.3343	453.004
17313091	Mief1	mitochondrial elongation factor 1	27.55286	20.89564	24.98482	24.35726	24.23533	22.13749
17250500	Mief2	mitochondrial elongation factor 2	20.85122	20.08574	17.1967	22.86422	13.54314	16.17563
17214825	Mff	mitochondrial fission factor	67.79138	57.63008	59.74268	53.80469	58.3528	55.45478
17259929	Mtfp1	mitochondrial fission process 1	43.66847	34.19824	35.01497	45.57434	36.28396	37.05664
17396205	Mtfr1	mitochondrial fission regulator 1	55.12023	54.18494	53.14198	53.9051	52.03931	56.67066
17431302	Mtfr1l	mitochondrial fission regulator 1-like	64.32555	54.99239	59.98251	57.08343	54.69132	53.82856
17231963	Mtfr2	mitochondrial fission regulator 2	3.929975	4.293488	4.526732	4.41453	4.136126	4.152413
17377028	Mgme1	mitochondrial genome maintainance exonuclease 1	15.17937	16.23595	16.23595	18.73845	11.56449	16.23595
17484494	Mtg1	mitochondrial GTPase 1 homolog (S. cerevisiae)	26.22771	27.16374	28.54864	27.9968	32.06217	32.77787
17431867	Minos1	mitochondrial inner membrane organizing system 1	19.87455	20.9788	19.69271	18.39383	17.24708	17.8823
17301061	Mipep	mitochondrial intermediate peptidase	42.49879	39.8799	47.75533	44.22806	36.33631	44.81501
17307428	Mipepos	mitochondrial intermediate peptidase, opposite strand	5.510717	4.640403	4.064619	4.440586	4.060761	4.503479
17518573	Mtfmt	mitochondrial methionyl-tRNA formyltransferase	37.06023	40.66794	35.57742	34.78709	25.9079	31.3295
17348126	Mtpap	mitochondrial poly(A) polymerase	49.90249	40.93265	40.1662	46.87543	47.56043	49.2793
17284037	Mpc1	mitochondrial pyruvate carrier 1	893.2623	1000.496	981.5514	864.5766	893.1942	1008.853
17333079	Mpc1	mitochondrial pyruvate carrier 1	32.30644	33.91661	34.37577	33.79973	35.47867	37.56117
17218955	Mpc2	mitochondrial pyruvate carrier 2	95.18221	85.17511	82.97932	82.91263	79.56765	88.14092
17439374	Mrpl1	mitochondrial ribosomal protein L1	41.82837	47.57099	35.157	41.30917	40.10388	42.31009
17255719	Mrpl10	mitochondrial ribosomal protein L10	77.77853	80.17441	81.18211	74.97616	83.7252	81.06459
17356314	Mrpl11	mitochondrial ribosomal protein L11	33.67929	44.82774	41.37049	42.48819	33.94954	47.26794

17259374	Mrpl12	mitochondrial ribosomal protein L12	60.85694	60.95531	60.52671	58.82836	54.02855	68.064
17317144	Mrpl13	mitochondrial ribosomal protein L13	51.61112	52.80124	53.68511	55.16266	51.93265	57.55902
17337955	Mrpl14	mitochondrial ribosomal protein L14	34.07142	30.96121	29.03659	32.00532	29.10268	33.73022
17221081	Mrpl15	mitochondrial ribosomal protein L15	73.79494	82.88218	82.88218	83.76583	69.77847	87.03538
17357741	Mrpl16	mitochondrial ribosomal protein L16	68.64202	60.90547	63.22968	72.25222	61.17244	69.33058
17494637	Mrpl17	mitochondrial ribosomal protein L17	55.67432	69.19999	70.69028	72.96891	77.82005	66.99864
17340943	Mrpl18	mitochondrial ribosomal protein L18	66.74001	69.68518	71.10455	63.36086	66.42065	66.16012
17467996	Mrpl19	mitochondrial ribosomal protein L19	21.46196	22.59341	29.16103	25.28938	31.66221	23.11552
17338124	Mrpl2	mitochondrial ribosomal protein L2	34.70592	33.29792	34.70592	37.27974	32.82243	33.07456
17422697	Mrpl20	mitochondrial ribosomal protein L20	25.88672	27.85491	23.71273	35.94776	33.41885	28.96121
17355903	Mrpl21	mitochondrial ribosomal protein L21	28.04672	27.29207	27.60045	27.98167	30.48273	26.78067
17249965	Mrpl22	mitochondrial ribosomal protein L22	16.36612	17.14457	16.36612	17.44885	17.93306	19.00109
17485258	Mrpl23	mitochondrial ribosomal protein L23	34.98368	41.68366	34.64463	33.93871	33.97661	39.775
17398873	Mrpl24	mitochondrial ribosomal protein L24	181.7045	167.1489	187.3121	213.439	193.6302	186.0234
17255243	Mrpl27	mitochondrial ribosomal protein L27	4.630988	5.866622	4.872222	5.438241	5.971527	7.310196
17334861	Mrpl28	mitochondrial ribosomal protein L28	28.81104	22.2065	29.08372	26.6956	29.80123	27.86129
17521070	Mrpl3	mitochondrial ribosomal protein L3	47.64457	43.68209	37.15205	44.75044	51.82334	52.45033
17211998	Mrpl30	mitochondrial ribosomal protein L30	20.3619	20.04749	22.70062	23.03539	18.13934	27.09547
17290770	Mrpl32	mitochondrial ribosomal protein L32	20.94697	17.38017	20.79767	20.94697	19.47485	14.37958
17464843	Gm6578	mitochondrial ribosomal protein L32 pseudogene	3.062691	2.809357	2.849937	2.67402	2.960457	2.67402
17436192	Mrpl33	mitochondrial ribosomal protein L33	9.208078	8.102235	9.064579	8.539905	9.409513	10.59873
17502191	Mrpl34	mitochondrial ribosomal protein L34	64.07146	65.94859	61.46188	60.2279	52.53802	63.80776
17467656	Mrpl35	mitochondrial ribosomal protein L35	72.16113	72.94666	66.22344	69.73688	72.16113	72.16113
17288463	Mrpl36	mitochondrial ribosomal protein L36	16.4382	17.33385	17.59093	18.97213	21.40269	18.09752
17427891	Mrpl37	mitochondrial ribosomal protein L37	58.85161	61.3861	50.38876	57.36584	55.69323	55.59757
17272186	Mrpl38	mitochondrial ribosomal protein L38	106.8283	106.0169	105.189	104.4226	78.69445	93.40255
17331642	Mrpl39	mitochondrial ribosomal protein L39	139.3182	142.9999	112.8967	138.8651	127.8989	140.3923
17515062	Mrpl4	mitochondrial ribosomal protein L4	33.30646	29.68866	37.14793	34.82859	28.58824	34.70376
17329001	Mrpl40	mitochondrial ribosomal protein L40	61.20298	67.29465	66.84186	73.27911	68.91948	78.36133
17384811	Gm16523	mitochondrial ribosomal protein L40 pseudogene	2.779933	3.603228	3.603977	3.515354	3.840569	2.848831
17382350	Mrpl41	mitochondrial ribosomal protein L41	25.98937	28.16204	25.0853	30.80964	34.26206	27.90013
17244506	Mrpl42	mitochondrial ribosomal protein L42	40.64793	51.00476	55.18445	56.6412	54.54859	63.1631
17365160	Mrpl43	mitochondrial ribosomal protein L43	42.96292	41.14257	39.09657	38.16901	38.55323	37.49016
17214722	Mrpl44	mitochondrial ribosomal protein L44	33.41444	33.07548	31.88398	32.18932	28.88283	31.09455
17255762	Mrpl45	mitochondrial ribosomal protein L45	36.97197	39.09331	39.24481	38.78213	38.671	40.39626
17492280	Mrpl46	mitochondrial ribosomal protein L46	23.96322	16.24642	15.89245	19.32572	10.95692	22.75923
17404616	Mrpl47	mitochondrial ribosomal protein L47	14.14892	12.55169	16.23595	16.18012	15.53413	16.594
17548266	Mrpl47	mitochondrial ribosomal protein L47	6.33498	4.706583	4.229596	3.751974	4.082662	4.695862
17493786	Mrpl48	mitochondrial ribosomal protein L48	19.42873	14.89958	15.11375	13.30144	16.98195	15.40365
17361915	Mrpl49	mitochondrial ribosomal protein L49	47.48763	37.16622	37.20256	42.59818	41.84954	36.83486
17425228	Mrpl50	mitochondrial ribosomal protein L50	132.3707	113.9558	86.92504	103.065	117.3708	104.14
17463140	Mrpl51	mitochondrial ribosomal protein L51	19.96097	21.5214	19.96097	18.76757	20.29764	23.27114
17300273	Mrpl52	mitochondrial ribosomal protein L52	105.2233	115.775	115.775	114.1985	119.1876	127.1291
17459947	Mrpl53	mitochondrial ribosomal protein L53	107.3531	80.09387	82.1404	104.9894	81.87834	96.94066
17300944	Mrpl57	mitochondrial ribosomal protein L53	17.84094	15.73331	15.56431	14.6741	11.84019	14.38287
17243247	Mrpl54	mitochondrial ribosomal protein L54	47.77686	50.08477	53.82782	53.82782	53.82782	52.23989
17250197	Mrpl55	mitochondrial ribosomal protein L55	71.00309	61.25306	68.75582	71.04839	62.06121	63.06554
17400091	Mrpl9	mitochondrial ribosomal protein L9	37.40321	33.86305	36.30081	39.43815	36.91589	44.56502
17338233	Mrps10	mitochondrial ribosomal protein S10	39.58577	39.35023	37.38299	40.06082	40.30006	43.11626
17479291	Mrps11	mitochondrial ribosomal protein S11	48.62647	48.05751	48.51234	43.04519	49.53204	43.42759
17488535	Mrps12	mitochondrial ribosomal protein S12	63.96097	67.29465	69.37577	67.29465	68.67961	72.66607
17218607	Mrps14	mitochondrial ribosomal protein S14	5.506593	8.73683	7.933734	4.688284	4.955876	8.423676
17418507	Mrps15	mitochondrial ribosomal protein S15	92.09749	113.4726	111.334	122.2415	116.9015	121.3946
17303765	Mrps16	mitochondrial ribosomal protein S16	53.60702	45.62776	37.34958	46.71358	38.52999	59.76132
17442898	Mrps17	mitochondrial ribosomal protein S17	51.23543	43.96123	50.37654	44.6386	48.20397	52.68572
17337974	Mrps18a	mitochondrial ribosomal protein S18A	44.12762	48.97692	39.03266	45.95755	37.1858	42.40287
17344497	Mrps18b	mitochondrial ribosomal protein S18B	56.59136	58.52414	57.71122	54.40925	54.96	58.24047
17329149	Gm15760	mitochondrial ribosomal protein S18B pseudogene	6.910547	6.383732	6.539725	4.746684	6.100764	7.04974
17439609	Mrps18c	mitochondrial ribosomal protein S18C	4.70593	4.410157	4.507852	4.018926	4.268414	4.203555
17368666	Mrps2	mitochondrial ribosomal protein S2	27.34831	31.43871	27.87249	30.04452	25.22776	20.58212
17407926	Mrps21	mitochondrial ribosomal protein S21	22.77233	26.79534	26.50073	29.22194	20.61997	20.11988
17529966	Mrps22	mitochondrial ribosomal protein S22	44.53569	45.2994	40.15506	38.9254	36.72626	35.70465
17254962	Mrps23	mitochondrial ribosomal protein S23	35.04338	30.56799	34.86143	36.7031	32.49464	35.3596
17260177	Mrps24	mitochondrial ribosomal protein S24	71.89502	71.37466	69.71755	71.69646	69.3676	84.421
17469089	Mrps25	mitochondrial ribosomal protein S25	23.2734	25.66837	24.3188	29.6463	23.67512	34.3853
17376365	Mrps26	mitochondrial ribosomal protein S26	44.09124	44.64851	36.60528	49.40825	50.04555	47.82954
17289439	Mrps27	mitochondrial ribosomal protein S27	29.87942	28.87042	29.20554	28.98069	31.28003	28.87042
17404021	Mrps28	mitochondrial ribosomal protein S28	44.91016	41.36963	29.97068	39.004	38.01403	41.51469
17296476	Mrps30	mitochondrial ribosomal protein S30	34.72614	39.32051	33.49445	35.48531	32.2561	38.69942
17499922	Mrps31	mitochondrial ribosomal protein S31	92.27066	75.33978	77.58146	81.32179	88.83831	90.95052
17466195	Mrps33	mitochondrial ribosomal protein S33	38.33526	44.43948	46.43884	43.44897	46.43884	49.28682
17334489	Mrps34	mitochondrial ribosomal protein S34	60.16114	49.0639	60.16114	57.00948	63.17623	60.36708
17464404	Mrps35	mitochondrial ribosomal protein S35	38.95563	36.81444	39.07435	43.15931	41.0944	49.30971
17295735	Mrps36	mitochondrial ribosomal protein S36	3.557787	3.186406	4.044048	4.907302	4.527517	3.920561
17375911	Mrps5	mitochondrial ribosomal protein S5	73.81336	78.16116	78.16116	82.22755	71.35982	80.50966
17212318	Mrps9	mitochondrial ribosomal protein S9	47.1351	44.83862	50.02024	45.95309	52.06096	54.99162
17380694	Mtg2	mitochondrial ribosome associated GTPase 2	23.36942	18.97213	20.5884	23.04529	20.91941	28.9263
17370285	Mrrf	mitochondrial ribosome recycling factor	65.69128	54.89033	47.98096	51.15559	46.05609	49.86861
17267057	Mrm1	mitochondrial rRNA methyltransferase 1 homolog (S. cerevisiae)	6.340515	6.21412	6.429827	8.414339	6.238936	7.385779
17419332	Mecr	mitochondrial trans-2-enoyl-CoA reductase	16.23595	16.23595	15.92366	17.74241	14.06805	16.23595
17445303	Mterf1a	mitochondrial transcription termination factor 1a	3.157363	3.607026	2.670204	3.182587	4.619991	2.95736
17434467	Mterf1b	mitochondrial transcription termination factor 1b	1.911864	2.184147	2.045509	2.357929	2.391524	3.248469
17243638	Mterf2	mitochondrial transcription termination factor 2	6.992896	7.698994	7.588843	10.37321	7.698994	8.865549
17293870	Mterf3	mitochondrial transcription termination factor 3	19.54219	22.31049	13.85102	19.62866	19.62866	19.42402

17225693	Mterf4	mitochondrial transcription termination factor 4	17.50518	23.30472	23.2497	23.46323	23.6853	23.09758
17519718	Mto1	mitochondrial translation optimization 1 homolog (S. cerevisiae)	28.81404	29.98585	29.16103	29.98585	25.26626	29.98585
17248064	Mtif2	mitochondrial translational initiation factor 2	44.59716	44.81873	44.31521	45.00957	47.02317	44.16097
17455274	Mtif3	mitochondrial translational initiation factor 3	32.90101	26.82209	26.79682	29.65226	33.37093	35.75666
17302213	Mtrf1	mitochondrial translational release factor 1	11.50006	11.44083	13.3404	14.34027	12.45256	12.65381
17238984	Mtrf1l	mitochondrial translational release factor 1-like	18.3429	19.13391	17.75311	16.71545	17.77121	17.75311
17508907	Mtus1	mitochondrial tumor suppressor 1	113.0541	118.968	102.9243	103.258	100.2814	96.69437
17420505	Mul1	mitochondrial ubiquitin ligase activator of NFKB 1	36.6853	40.24451	36.43846	39.33359	44.3484	41.37852
17532595	mt-Rnr1	mitochondrially encoded 12S rRNA [Source:MGI Symbol;Acc:MGI:102493]	6558.59	6791.337	6557.817	6775.116	6487.109	6983.139
17532599	mt-Rnr2	mitochondrially encoded 16S rRNA [Source:MGI Symbol;Acc:MGI:102492]	5274.286	5196.015	5012.588	5411.077	5272.275	5608.503
17532639	mt-Ta	mitochondrially encoded tRNA alanine [Source:MGI Symbol;Acc:MGI:102491]	279.4139	310.0635	546.1071	378.1514	372.6946	387.2281
17532623	mt-Tr	mitochondrially encoded tRNA arginine [Source:MGI Symbol;Acc:MGI:102476]	696.1768	532.1614	699.1654	816.6816	615.6088	765.2939
17532641	mt-Tn	mitochondrially encoded tRNA asparagine [Source:MGI Symbol;Acc:MGI:102479]	2046.368	1674.599	1850.385	1946.059	1980.309	2123.252
17532643	mt-Tc	mitochondrially encoded tRNA cysteine [Source:MGI Symbol;Acc:MGI:102490]	58.87732	120.2998	268.2325	123.0866	110.6447	105.8076
17532651	mt-Te	mitochondrially encoded tRNA glutamic acid [Source:MGI Symbol;Acc:MGI:102488	3364.345	3990.296	4618.856	3635.908	3630.066	3672.177
17532637	mt-Tq	mitochondrially encoded tRNA glutamine [Source:MGI Symbol;Acc:MGI:102477]	409.2816	363.2226	466.8709	416.1904	355.9523	353.3319
17532619	mt-Tg	mitochondrially encoded tRNA glycine [Source:MGI Symbol;Acc:MGI:102486]	1798.814	1779.949	1740.987	1928.79	1651.109	1921.57
17532627	mt-Th	mitochondrially encoded tRNA histidine [Source:MGI Symbol;Acc:MGI:102485]	1246.062	1200.059	1715.71	1602.402	1350.624	1583.938
17532601	mt-Tl1	mitochondrially encoded tRNA leucine 1 [Source:MGI Symbol;Acc:MGI:102482]	5325.421	5085.31	4879.61	5253.124	4807.796	4601.281
17532615	mt-Tk	mitochondrially encoded tRNA lysine [Source:MGI Symbol;Acc:MGI:102483]	373.372	551.0273	1029.801	757.0422	588.7576	738.109
17532605	mt-Tm	mitochondrially encoded tRNA methionine [Source:MGI Symbol;Acc:MGI:102480]	363.6307	492.6059	755.166	489.3626	410.0568	430.2556
17532593	mt-Tf	mitochondrially encoded tRNA phenylalanine [Source:MGI Symbol;Acc:MGI:102487	572.5667	724.3936	841.4245	664.387	354.1238	449.4011
17532653	mt-Tp	mitochondrially encoded tRNA proline [Source:MGI Symbol;Acc:MGI:102478]	24.3156	49.20183	93.54055	44.11997	58.80105	53.04997
17532647	mt-Ts1	mitochondrially encoded tRNA serine 1 [Source:MGI Symbol;Acc:MGI:102475]	61.82892	116.4454	240.773	112.9946	129.3064	141.3355
17532629	mt-Ts2	mitochondrially encoded tRNA serine 2 [Source:MGI Symbol;Acc:MGI:102474]	72.06516	175.1613	481.139	192.8814	200.6003	216.0603
17532635	mt-Tt	mitochondrially encoded tRNA threonine [Source:MGI Symbol;Acc:MGI:102473]	41.16939	81.84713	175.6512	89.16094	78.57726	100.2161
17532645	mt-Ty	mitochondrially encoded tRNA tyrosine [Source:MGI Symbol;Acc:MGI:102470]	80.98147	275.5079	619.4912	238.3239	208.5891	194.7258
17532597	mt-Tv	mitochondrially encoded tRNA valine [Source:MGI Symbol;Acc:MGI:102472]	3794.042	3854.372	3743.936	4100.545	3723.258	4076.372
17396710	Mfn1	mitofusin 1	119.3058	112.1628	96.00449	107.7863	113.3099	97.95587
17432808	Mfn2	mitofusin 2	105.0797	124.7002	132.3267	134.5007	86.89372	119.4713
17323371	Mapk1	mitogen-activated protein kinase 1	160.628	127.3301	119.2495	131.772	120.1481	148.5413
17497414	Mapk1ip1	mitogen-activated protein kinase 1 interacting protein 1	17.26897	16.10984	20.03083	19.00148	22.29132	18.62967
17299323	Mapk1ip1l	mitogen-activated protein kinase 1 interacting protein 1-like	205.8172	210.9606	243.8534	185.1627	181.1336	168.2532
17450283	Mapk10	mitogen-activated protein kinase 10	4.760259	5.451526	5.702996	6.37533	4.31826	4.898296
17320322	Mapk11	mitogen-activated protein kinase 11	8.268926	8.838674	8.822271	6.497749	10.42532	8.604313
17320307	Mapk12	mitogen-activated protein kinase 12	13.46255	13.65001	16.04398	12.36937	12.45411	11.04017
17335383	Mapk13	mitogen-activated protein kinase 13	4.208132	6.294318	6.240363	6.276977	7.941216	5.782487
17335364	Mapk14	mitogen-activated protein kinase 14	48.30799	45.99484	46.9932	50.33988	42.09976	39.90839
17312312	Mapk15	mitogen-activated protein kinase 15	11.09963	13.43924	11.08172	11.12058	10.9334	8.508
17483084	Mapk3	mitogen-activated protein kinase 3	135.1793	160.4077	244.063	166.7543	143.5202	132.9067
17355411	Mapk4	mitogen-activated protein kinase 4	9.88842	7.995565	7.995565	7.995565	6.531118	6.358427
17528836	Mapk6	mitogen-activated protein kinase 6	78.14058	82.80466	92.15688	68.82162	72.26975	72.46689
17263811	Mapk7	mitogen-activated protein kinase 7	15.21824	12.58384	12.2517	12.71906	13.67871	12.93322
17304976	Mapk8	mitogen-activated protein kinase 8	37.25443	34.57794	31.36796	29.63561	34.76925	30.85089
17388371	Mapk8ip1	mitogen-activated protein kinase 8 interacting protein 1	7.498593	7.499113	7.40223	8.51685	6.904275	7.498593
17314164	Mapk8ip2	mitogen-activated protein kinase 8 interacting protein 2	6.220239	7.618727	5.501023	7.157124	7.175802	6.536009
17342065	Mapk8ip3	mitogen-activated protein kinase 8 interacting protein 3	18.35308	23.64249	23.53311	22.24979	18.45935	25.39055
17249057	Mapk9	mitogen-activated protein kinase 9	66.42802	66.42802	69.77992	70.87771	59.96089	56.66398
17370079	Mapkap1	mitogen-activated protein kinase associated protein 1	85.2844	85.38436	78.70263	82.15691	70.78786	79.24236
17374939	Mapkbp1	mitogen-activated protein kinase binding protein 1	8.076779	9.353135	10.32736	10.76943	10.32736	10.89293
17528160	Map2k1	mitogen-activated protein kinase kinase 1	61.42866	51.42222	52.1838	50.4996	52.1368	56.02776
17235539	Map2k2	mitogen-activated protein kinase kinase 2	136.348	125.9834	131.1915	121.936	122.8411	115.3801
17250533	Map2k3	mitogen-activated protein kinase kinase 3	73.30552	85.03589	82.18824	88.96957	84.07471	84.85396
17263705	Map2k3os	mitogen-activated protein kinase kinase 3, opposite strand	12.06495	9.666007	11.93313	9.594316	6.84272	7.304016
17264189	Map2k4	mitogen-activated protein kinase kinase 4	60.90843	66.38981	56.85164	61.161	64.76534	65.42862
17528053	Map2k5	mitogen-activated protein kinase kinase 5	55.34995	49.68781	48.16957	57.98067	37.68321	62.56179
17257906	Map2k6	mitogen-activated protein kinase kinase 6	5.5228	8.679007	9.103365	8.12428	9.310251	10.50919
17498791	Map2k7	mitogen-activated protein kinase kinase 7	29.79524	26.34286	26.50183	25.42877	29.16103	27.03975
17296174	Map3k1	mitogen-activated protein kinase kinase kinase 1	33.20733	30.60001	35.81123	32.2485	48.02787	31.88288
17488312	Map3k10	mitogen-activated protein kinase kinase kinase 10	14.37739	14.12119	14.37739	15.56259	14.37739	14.37739
17356502	Map3k11	mitogen-activated protein kinase kinase kinase 11	63.22091	49.96098	56.17047	52.39437	38.62786	44.79544
17315463	Map3k12	mitogen-activated protein kinase kinase kinase 12	7.248186	4.474821	5.334821	4.258779	5.532724	5.296356
17322230	Map3k12	mitogen-activated protein kinase kinase kinase 12	9.684718	8.62312	9.438123	8.640351	8.763882	8.390519
17324259	Map3k13	mitogen-activated protein kinase kinase kinase 13	41.62764	43.96123	42.60675	45.36287	36.90745	33.32031
17270453	Map3k14	mitogen-activated protein kinase kinase kinase 14	7.621104	6.934107	7.837752	10.72295	6.837837	9.503046
17539241	Map3k15	mitogen-activated protein kinase kinase kinase 15	8.063968	9.879719	10.94432	9.606827	7.213003	9.421848
17226489	Map3k19	mitogen-activated protein kinase kinase kinase 19	7.935879	9.803113	6.589002	6.171709	8.441387	7.935879
17349243	Map3k2	mitogen-activated protein kinase kinase kinase 2	64.16613	67.49459	59.144	54.91582	48.79732	63.263
17257511	Map3k3	mitogen-activated protein kinase kinase kinase 3	41.62793	43.54907	41.83011	42.09421	42.82674	42.14063
17340787	Map3k4	mitogen-activated protein kinase kinase kinase 4	42.4161	28.37798	29.59743	32.29771	27.49715	32.63685
17231891	Map3k5	mitogen-activated protein kinase kinase kinase 5	75.01307	78.35536	72.84207	80.82726	70.90129	93.62553
17419553	Map3k6	mitogen-activated protein kinase kinase kinase 6	6.590509	9.263902	7.250347	10.11434	7.406695	8.193614
17412350	Map3k7	mitogen-activated protein kinase kinase kinase 7	37.88251	41.9541	38.36902	42.83416	44.3733	41.21421
17352401	Map3k8	mitogen-activated protein kinase kinase kinase 8	10.57327	12.23888	17.86805	11.19513	12.2433	8.677203
17282380	Map3k9	mitogen-activated protein kinase kinase kinase 9	4.362001	6.53755	5.130111	5.130111	5.185956	5.587024
17476036	Map4k1	mitogen-activated protein kinase kinase kinase kinase 1	5.403522	7.065688	5.403522	5.751108	4.295422	4.67994
17356811	Map4k2	mitogen-activated protein kinase kinase kinase kinase 2	11.88624	17.42528	17.42528	14.9063	17.42528	16.43352
17347571	Map4k3	mitogen-activated protein kinase kinase kinase kinase 3	62.69029	99.33361	93.29449	87.11988	86.21757	90.84782
17212138	Map4k4	mitogen-activated protein kinase kinase kinase kinase 4	31.38689	44.48817	40.28889	36.84916	27.90535	27.43451
17281637	Map4k5	mitogen-activated protein kinase kinase kinase kinase 5	38.44489	46.4485	45.5232	45.18807	37.36382	37.45302
17530863	Mapkapk3	mitogen-activated protein kinase-activated protein kinase 3	13.24045	18.15783	19.38945	16.97736	17.70739	20.82883
17309065	Mzt1	mitotic spindle organizing protein 1	88.42669	67.82993	79.04761	86.1035	88.39482	95.35125
17328451	Mzt2	mitotic spindle organizing protein 2	16.90971	15.3676	16.07715	13.39423	16.85471	16.48246
17234972	Misp	mitotic spindle positioning	8.973557	6.837884	8.71264	8.346203	9.310251	7.139238

17230463	Mixl1	Mix1 homeobox-like 1 (Xenopus laevis)	4.288015	5.919191	4.472929	4.055613	4.300725	4.065154
17513076	Mlkl	mixed lineage kinase domain-like	38.6903	40.28609	53.12982	44.54711	45.24919	30.16283
17319429	Mkl1	MKL (megakaryoblastic leukemia)/myocardin-like 1	18.32219	19.49375	25.00891	18.32219	19.41729	16.43193
17323013	Mkl2	MKL/myocardin-like 2	63.95078	67.17302	70.28525	66.49758	64.32288	63.88432
17442284	Mlxip	MLX interacting protein	22.79239	30.50915	30.63332	27.84816	26.89582	25.69579
17443185	Mlxipl	MLX interacting protein-like	102.0321	181.3308	145.3886	197.1719	172.3372	160.2988
17364772	Mms19	MMS19 (MET18 S. cerevisiae)	21.93176	28.74761	30.85367	30.88726	32.6631	33.83114
17412237	Mms22l	MMS22-like, DNA repair protein	6.287864	6.287864	6.287864	4.988825	5.325327	6.391125
17297112	Mnd1-ps	Mnd1 retrotransposed pseudogene	3.043307	2.470461	2.280278	2.445247	2.29746	2.522877
17212888	Mob4	MOB family member 4, phocein	45.231	39.60279	38.72598	37.89406	37.84445	42.09801
17460059	Mob1a	MOB kinase activator 1A	37.91035	42.20611	39.52901	34.66289	34.26444	40.65582
17438814	Mob1b	MOB kinase activator 1B	134.0818	100.4776	81.05336	101.4961	90.43003	100.0342
17498032	Mob2	MOB kinase activator 2	38.53907	34.22812	37.94164	38.38312	41.42788	47.04208
17243051	Mob3a	MOB kinase activator 3A	17.35477	17.69684	19.44881	15.07755	15.47374	19.74753
17423915	Mob3b	MOB kinase activator 3B	78.04855	77.62019	79.75262	70.35934	74.00239	62.07197
17417302	Mob3c	MOB kinase activator 3C	8.758355	11.00022	8.639513	10.18532	9.960789	8.982954
17417306	Mob3c	MOB kinase activator 3C	24.26979	20.50978	25.56114	17.52776	24.39917	21.36148
17283477	Moap1	modulator of apoptosis 1	21.64872	19.81114	21.68822	23.70649	23.35614	21.75673
17536364	Msn	moesin	54.95885	57.97303	67.63321	48.29895	44.27683	47.68488
17352517	Mkx	mohawk homeobox	3.006094	3.743869	3.743869	4.270722	4.352065	3.126828
17284020	Mok	MOK protein kinase	4.40301	5.960238	4.780528	4.883041	5.557053	4.872087
17408765	Mov10	Moloney leukemia virus 10	31.84577	28.24865	27.8982	27.10242	34.89988	27.64468
17314019	Mov10l1	Moloney leukemia virus 10-like 1	4.762371	4.91537	4.054938	4.942634	4.885818	4.749975
17422992	Mos	Moloney sarcoma oncogene	2.823197	3.710002	3.203502	3.150411	3.34538	2.966764
17349016	Mocos	molybdenum cofactor sulfurase	63.44392	51.19922	45.73035	60.27467	42.62654	42.64516
17338472	Mocs1	molybdenum cofactor synthesis 1	225.8483	154.8277	147.12	142.4785	113.3099	125.4684
17290056	Mocs2	molybdenum cofactor synthesis 2	171.7171	180.2768	180.1494	180.4593	179.2128	212.3853
17379986	Mocs3	molybdenum cofactor synthesis 3	12.99919	12.80699	9.991837	12.90788	17.43688	13.45397
17521475	Mon1a	MON1 homolog A (yeast)	37.29447	35.0208	36.94891	37.29447	37.29447	34.09244
17505826	Mon1b	MON1 homolog b (yeast)	49.65338	50.24611	47.28072	45.4822	41.69608	35.70564
17245587	Mon2	MON2 homolog (yeast)	114.1574	94.58569	92.91374	103.5648	98.41798	104.1032
17214672	Mogat1	monoacylglycerol O-acyltransferase 1	12.55204	14.10994	16.83206	10.25524	8.971007	9.111256
17493643	Mogat2	monoacylglycerol O-acyltransferase 2	10.31856	9.64566	11.54591	13.20068	8.768621	10.31856
17533474	Maoa	monoamine oxidase A	18.51689	26.51065	29.30774	22.08263	12.07005	12.35044
17540378	Maob	monoamine oxidase B	399.1397	419.5968	418.9371	380.4808	425.463	495.7641
17255064	Mmd	monocyte to macrophage differentiation-associated	58.26429	60.32107	59.77043	67.25036	51.67269	58.37465
17454692	Mmd2	monocyte to macrophage differentiation-associated 2	53.10074	58.82306	75.3915	61.46571	51.28731	59.51494
17460665	Mgll	monoglyceride lipase	125.1609	139.2738	138.4096	134.1832	84.93222	96.0391
17232215	Moxd1	monooxygenase, DBH-like 1	8.036961	4.925841	4.750337	4.762866	6.610274	4.711544
17466239	Moxd2	monooxygenase, DBH-like 2	3.236916	3.681669	3.990861	4.573346	3.30129	3.551578
17447510	Mrfap1	Morf4 family associated protein 1	109.3887	112.6099	116.0522	99.60319	120.1015	109.5982
17422488	Morn1	MORN repeat containing 1	7.561959	9.166303	9.401363	7.799221	7.761148	8.379708
17339934	Morn2	MORN repeat containing 2	16.09622	19.0619	21.76367	17.73134	20.2099	17.69684
17452540	Morn3	MORN repeat containing 3	10.74222	11.53287	9.094914	11.29929	8.680993	11.69865
17364805	Morn4	MORN repeat containing 4	7.098332	9.023018	9.229548	10.08626	9.023018	9.525864
17370277	Morn5	MORN repeat containing 5	6.305147	6.868619	6.532793	7.615137	6.430139	6.317615
17529647	Morf4l1	mortality factor 4 like 1	24.27888	21.99449	17.2368	19.57174	14.80487	25.46176
17324469	Morf4l1-ps1	mortality factor 4 like 1, pseudogene 1	5.396709	7.491788	7.742503	6.402565	4.352065	6.241748
17544754	Morf4l2	mortality factor 4 like 2	26.83009	22.55601	18.07633	22.6882	19.22978	20.16232
17541760	Mospd1	motile sperm domain containing 1	69.43013	66.07213	57.77406	53.5929	60.5351	55.63767
17545979	Mospd2	motile sperm domain containing 2	29.9134	29.6198	31.82768	32.3912	27.61729	33.65969
17454069	Mospd3	motile sperm domain containing 3	36.52621	38.98589	39.60526	36.77211	33.57045	36.45917
17354258	Mospd4	motile sperm domain containing 4	12.67584	9.795098	8.734953	11.57668	12.07265	10.15044
17446608	Mnx1	motor neuron and pancreas homeobox 1	3.318773	3.454292	3.318773	3.318773	3.318773	3.729055
17491816	Mphosph10	M-phase phosphoprotein 10 (U3 small nucleolar ribonucleoprotein)	17.41115	16.34922	12.33392	18.74196	17.36355	17.40609
17300839	Mphosph8	M-phase phosphoprotein 8	20.05597	16.34854	18.99727	17.81524	19.62866	19.94147
17452776	Mphosph9	M-phase phosphoprotein 9	10.4724	16.07782	14.57617	12.19696	12.53561	12.75225
17285448	Mplkip	M-phase specific PLK1 intereacting protein	46.04204	38.90787	46.20509	31.94681	41.16111	40.82526
17338684	Mpnd	MPN domain containing	55.92301	48.04985	55.59603	54.22063	62.00489	54.5849
17446836	Mpv17	MpV17 mitochondrial inner membrane protein	201.7689	176.6623	171.6766	142.288	136.7038	143.7472
17510129	Mpv17l2	MPV17 mitochondrial membrane protein-like 2	25.58382	37.64927	35.07497	37.64927	35.59493	39.07679
17323098	Mpv17l	Mpv17 transgene, kidney disease mutant-like	27.9935	33.19583	30.35285	33.16455	35.38142	37.9611
17380790	Mrgbp	MRG/MORF4L binding protein	17.1824	15.9754	16.46214	12.29713	17.70445	15.99235
17431895	Mrto4	mRNA turnover 4, homolog (S. cerevisiae)	47.84181	34.04214	39.24427	35.69574	34.5286	36.8627
17291374	Mrs2	MRS2 magnesium homeostasis factor homolog (S. cerevisiae)	37.47847	37.99032	36.08006	31.31322	28.95679	29.65513
17495106	Mrvi1	MRV integration site 1	10.16607	9.618287	7.711973	8.790346	8.790346	8.790346
17447610	Msx1	msh homeobox 1	16.37667	16.65046	15.96566	13.73552	15.96566	15.46505
17437049	Msx1os	msh homeobox 1 opposite strand	4.01461	4.647678	5.230105	4.401423	4.661583	5.108395
17292671	Msx2	msh homeobox 2	4.260846	4.642301	3.721876	3.040967	3.698551	3.616866
17497576	Msx3	msh homeobox 3	5.715541	7.189892	8.82851	6.093326	5.884541	6.486139
17303821	Mss51	MSS51 mitochondrial translational activator	3.754316	4.647678	6.001237	4.647678	6.982966	4.647678
17341872	Mlst8	MTOR associated protein, LST8 homolog (S. cerevisiae)	18.19512	18.76861	18.76926	16.70618	15.95338	16.10247
17399374	Muc1	mucin 1, transmembrane	8.381741	8.15322	8.917195	8.313984	6.428891	6.861053
17325093	Muc13	mucin 13, epithelial transmembrane	5.759246	5.617429	5.617429	5.971941	5.617429	5.617429
17374156	Muc15	mucin 15	2.619879	3.258369	2.613947	2.74111	3.331473	3.0899
17524311	Muc16	mucin 16	3.877294	3.287361	3.267212	3.267212	4.25008	3.21026
17314373	Muc19	mucin 19	3.787485	4.174664	4.607231	3.091485	5.120306	4.886969
17314375	Muc19	mucin 19	4.820524	6.517958	7.169338	5.043621	5.665723	6.252746
17484991	Muc2	mucin 2	9.774006	11.54831	10.77772	10.62006	9.581822	10.62006
17485019	Muc2	mucin 2	3.941104	3.922681	3.369676	3.935645	3.778255	3.764374
17329864	Muc20	mucin 20	4.832936	7.362675	7.436481	5.057029	5.533721	7.78561
17453839	Muc3	mucin 3, intestinal	5.56106	6.180528	6.252963	5.623039	5.56106	5.56106
17453874	Muc3a	mucin 3A, cell surface associated	3.799185	6.301695	6.087585	6.248544	4.58687	4.794765
17324883	Muc4	mucin 4	7.037339	6.724048	7.348399	6.735383	6.646938	6.247418

17485098	Muc5b	mucin 5, subtype B, tracheobronchial	6.995368	6.835941	8.013486	6.835941	6.083873	8.212093
17485039	Muc5ac	mucin 5, subtypes A and C, tracheobronchial/gastric	4.610681	5.780603	4.987615	4.682568	4.987615	4.987615
17497980	Muc6	mucin 6, gastric	5.675785	6.3921	6.297111	6.316608	6.362483	6.42702
17404958	LOC102632538	mucin-1-like	5.617429	6.616049	5.617429	5.777318	7.620312	5.337263
17322444	Mucl2	mucin-like 2	2.412502	2.67402	2.685257	2.316216	2.524312	2.67402
17498607	Mcoln1	mucolipin 1	57.21375	63.42194	64.9992	49.5032	49.08737	45.73574
17403490	Mcoln2	mucolipin 2	5.410418	8.538366	7.635645	8.667645	6.836605	7.256631
17403473	Mcoln3	mucolipin 3	7.90919	8.916145	9.310251	10.12184	9.379168	8.493328
17219751	Mptx1	mucosal pentraxin 1	3.062691	4.374475	2.752934	3.321328	3.263862	3.03848
17229981	Mptx2	mucosal pentraxin 2	8.701355	7.185507	9.655627	12.0765	10.2922	11.09179
17234898	Madcam1	mucosal vascular addressin cell adhesion molecule 1	9.43191	9.302316	9.43191	8.826491	8.041641	9.43191
17289977	Mcidas	multiciliate differentiation and DNA synthesis associated cell cycle protein	6.626881	8.015264	7.374269	7.392078	6.080542	7.176249
17459097	Mmrn1	multimerin 1	5.494812	4.948804	5.198192	5.564685	4.646035	4.457269
17298825	Mmrn2	multimerin 2	48.9854	44.9547	51.04756	46.30224	36.28698	39.46167
17288780	Mctp1	multiple C2 domains, transmembrane 1	3.244621	3.745271	4.269361	3.703322	3.703322	3.298134
17492078	Mctp2	multiple C2 domains, transmembrane 2	39.88903	35.41896	30.21996	33.72017	38.8109	31.08895
17347903	Mcfd2	multiple coagulation factor deficiency 2	63.2744	63.02796	63.2744	62.3622	70.4052	49.44902
17350762	Megf10	multiple EGF-like-domains 10	5.430895	6.900735	5.692491	5.60576	4.33809	5.934844
17518342	Megf11	multiple EGF-like-domains 11	9.299222	9.488237	9.897814	9.91182	9.310251	13.09796
17422337	Megf6	multiple EGF-like-domains 6	6.657867	6.746784	7.642052	6.589459	6.375993	6.746784
17475251	Megf8	multiple EGF-like-domains 8	11.02457	13.75038	12.51659	12.17635	11.79947	9.9463
17426497	Megf9	multiple EGF-like-domains 9	75.4443	72.49	87.13618	80.25003	90.95746	81.69353
17356794	Men1	multiple endocrine neoplasia 1	38.85801	36.96582	46.86406	43.90307	44.6934	42.8915
17358682	Minpp1	multiple inositol polyphosphate histidine phosphatase 1	81.1883	80.09609	80.3842	87.73557	89.75124	86.703
17426700	Mpdz	multiple PDZ domain protein	25.53935	24.236	26.7495	26.23553	26.45723	22.98467
17502221	Mvb12a	multivesicular body subunit 12A	75.65849	74.44949	85.09662	74.67586	74.76598	75.11296
17384205	Mvb12b	multivesicular body subunit 12B	8.655337	9.032028	11.35913	8.872434	6.191762	8.410433
17462529	Mug1	murinoglobulin 1	3887.67	3338.768	2704.395	3603.667	3510.328	3113.346
17462566	Mug2	murinoglobulin 2	831.4121	579.8958	362.2621	551.3406	1060.979	910.8944
17462602	Gm10319	murinoglobulin pseudogene	281.611	195.4926	146.5186	204.0388	199.1511	176.023
17462608	Mug-ps1	murinoglobulin, pseudogene 1	245.9464	244.7695	173.7621	222.3625	206.2943	208.4532
17361617	Mus81	MUS81 endonuclease homolog (yeast)	24.72502	20.11347	19.77519	28.55774	21.98092	29.14285
17441134	Msi1	musashi RNA-binding protein 1	2.938276	3.384071	2.827917	2.467637	3.261397	2.94011
17441140	Msi1	musashi RNA-binding protein 1	8.464472	10.54692	11.88113	10.48228	10.48228	10.48228
17267559	Msi2	musashi RNA-binding protein 2	36.16996	33.02187	37.02788	39.33223	37.71561	37.02788
17530059	Mras	muscle and microspikes RAS	9.511085	8.605267	8.324191	7.572869	5.639941	6.489557
17356874	Pygm	muscle glycogen phosphorylase	5.933733	6.075487	6.865715	6.951768	6.342361	6.73669
17414385	Musk	muscle, skeletal, receptor tyrosine kinase	3.216073	3.49977	3.171027	3.216073	3.078527	3.170587
17397936	Mbnl1	muscleblind-like 1 (Drosophila)	171.4709	166.1393	165.7414	144.3813	133.5069	141.7662
17302754	Mbnl2	muscleblind-like 2	136.8906	115.1747	151.632	130.0858	108.6143	104.8258
17541618	Mbnl3	muscleblind-like 3 (Drosophila)	4.549254	4.643073	5.000665	4.676762	3.902909	5.722399
17414032	Murc	muscle-related coiled-coil protein	3.620754	5.37029	3.675167	3.091421	3.563266	3.016007
17528947	Mlip	muscular LMNA-interacting protein	3.736325	4.183586	3.181705	3.787485	3.632745	3.918251
17221396	Msc	musculin	14.23825	12.19062	10.66876	14.43921	12.64627	14.23825
17298262	Mustn1	musculoskeletal, embryonic nuclear protein 1	31.65628	58.82796	68.08051	47.55417	45.7481	36.64209
17456995	Mkln1	muskelin 1, intracellular mediator containing kelch motifs	82.65728	83.62776	82.65728	91.53046	81.40469	82.02149
17465614	Mkln1os	muskelin 1, intracellular mediator containing kelch motifs, opposite strand	12.64574	10.95107	11.56746	12.23083	10.20546	12.15426
17354189	Mcc	mutated in colorectal cancers	95.5769	70.22778	88.21182	94.23036	94.42782	138.9257
17531709	Mlh1	mutL homolog 1 (E. coli)	31.83275	27.60696	26.1716	30.67157	35.55918	29.98356
17282673	Mlh3	mutL homolog 3 (E coli)	13.85054	13.29581	13.89034	17.58094	15.6334	10.21445
17340285	Msh2	mutS homolog 2 (E. coli)	9.756881	10.5356	11.39769	10.5356	9.777394	10.69941
17294860	Msh3	mutS homolog 3 (E. coli)	20.40852	22.73038	20.80748	22.5992	22.7255	21.93572
17411277	Msh4	mutS homolog 4 (E. coli)	5.717126	5.678697	5.456175	5.228909	5.92465	5.92465
17344158	Msh5	mutS homolog 5 (E. coli)	5.977988	6.975758	7.647669	6.893181	8.115275	7.14607
17340307	Msh6	mutS homolog 6 (E. coli)	12.60592	11.73219	12.10214	10.10012	10.26463	13.45807
17417530	Mutyh	mutY homolog (E. coli)	4.123387	4.612983	4.497147	4.497147	5.199634	5.522303
17332531	Mx1	MX dynamin-like GTPase 1	3.660143	2.780376	2.867851	3.168061	2.71637	3.114931
17327557	Mx2	MX dynamin-like GTPase 2	9.729529	10.08123	10.95168	10.06196	12.69405	13.73105
17252369	Mybbp1a	MYB binding protein (P160) 1a	86.20861	69.93438	70.42911	62.1542	77.3168	70.83694
17436755	Msantd1	Myb/SANT-like DNA-binding domain containing 1	6.289956	5.980599	5.534949	7.744918	6.868857	6.44759
17516068	Msantd2	Myb/SANT-like DNA-binding domain containing 2	11.00388	14.53602	16.19796	13.21972	13.21972	16.70653
17414009	Msantd3	Myb/SANT-like DNA-binding domain containing 3	7.22381	8.073924	9.62589	9.537426	9.277654	7.349724
17514417	Msantd4	Myb/SANT-like DNA-binding domain containing 4 with coiled-coils	74.09632	64.56006	65.59664	64.56006	61.9907	54.1579
17427284	Mysm1	myb-like, SWIRM and MPN domains 1	22.09457	20.81281	21.70311	23.05392	22.85456	23.25856
17474389	Mypop	Myb-related transcription factor, partner of profilin	45.45667	42.42933	47.30399	42.22981	44.64913	41.4727
17487144	Mypopos	Myb-related transcription factor, partner of profilin, opposite strand	9.933692	8.832183	6.509334	7.316887	8.455441	7.557348
17309172	Mycbp2	MYC binding protein 2	37.23455	38.31746	39.56986	39.56986	37.49588	39.38952
17326472	Mina	myc induced nuclear antigen	20.59697	18.6267	18.97384	19.04209	16.08328	16.05188
17467363	4930515G16Rik	myc induced nuclear antigen pseudogene	2.82398	2.628774	2.937502	2.683117	2.819071	2.668668
17231477	Myct1	myc target 1	19.36125	13.78445	20.88912	17.18157	14.34295	14.46506
17496555	Maz	MYC-associated zinc finger protein (purine-binding transcription factor)	41.4842	32.57809	42.43786	39.09223	38.14971	40.79555
17267970	Mycbpap	MYCBP associated protein	5.398655	5.398655	5.386134	5.386134	5.398655	5.826381
17330337	Maats1	MYCBP-associated, testis expressed 1	3.703322	3.726135	3.891968	3.703322	3.46775	3.207413
17325578	Maats1os	MYCBP-associated, testis expressed 1, opposite strand [Source:MGI Symbol;Acc:MG	3.25115	3.211729	3.462322	3.462322	3.787222	3.434952
17539758	Mycs	myc-like oncogene, s-myc protein	5.469428	4.326019	4.876581	5.386134	7.140475	4.910841
17391332	Mal	myelin and lymphocyte protein, T cell differentiation protein	8.744828	8.052518	8.007545	8.161758	7.880024	7.292937
17352175	Mbp	myelin basic protein	9.361307	8.286196	9.361307	9.37202	10.1617	9.310251
17390844	Myef2	myelin basic protein expression factor 2, repressor	20.79853	17.61265	17.75582	19.13855	18.02728	17.63183
17396064	Gm9833	myelin basic protein expression factor 2, repressor pseudogene	2.763526	2.377969	2.361183	2.659804	2.274485	3.409216
17344822	Mog	myelin oligodendrocyte glycoprotein	7.789442	8.380173	7.003034	6.316082	8.131942	7.205807
17219222	Mpz	myelin protein zero	6.242656	7.615454	8.286713	8.038409	8.212264	8.516024
17229259	Mpzl1	myelin protein zero-like 1	21.48265	21.68056	24.67897	27.56508	22.3746	16.18629
17516699	Mpzl2	myelin protein zero-like 2	62.19859	86.66815	74.54192	83.02765	95.90938	122.161
17516707	Mpzl3	myelin protein zero-like 3	28.88983	30.39959	29.52571	31.70767	37.86308	33.85554

17362616	Myrf	myelin regulatory factor	46.0893	47.48336	48.86569	47.76096	48.21466	50.29698
17245124	Myrfl	myelin regulatory factor-like	3.037785	3.337945	2.719271	2.93138	2.844963	2.719271
17381143	Myt1	myelin transcription factor 1	7.979011	8.476155	6.668351	6.298246	8.457082	7.167848
17274670	Myt1l	myelin transcription factor 1-like	4.647678	4.128936	4.647678	5.718275	4.386226	5.54305
17489341	Mag	myelin-associated glycoprotein	4.395204	4.717655	3.986244	4.395204	3.43967	4.395204
17523192	Mobp	myelin-associated oligodendrocytic basic protein	4.468529	5.368987	5.182578	5.420891	6.126232	5.630527
17239664	Myb	myeloblastosis oncogene	6.148183	6.431878	6.009688	5.957801	6.163672	6.158675
17221158	Mybl1	myeloblastosis oncogene-like 1	5.944807	11.28184	13.71802	7.917912	7.650001	8.417839
17379206	Mybl2	myeloblastosis oncogene-like 2	6.620637	7.336744	7.010707	7.762062	8.289356	7.835851
17311846	Myc	myelocytomatosis oncogene	27.21066	37.55544	46.90143	20.97821	28.36598	27.09078
17400418	Mcl1	myeloid cell leukemia sequence 1	153.2656	198.3617	167.1211	172.1807	169.5609	180.5419
17230078	Mnda	myeloid cell nuclear differentiation antigen	41.48506	43.09444	72.87287	53.26884	46.80498	41.87276
17346175	Mydgf	myeloid derived growth factor	63.40084	54.49984	50.06915	51.8812	58.9917	57.89753
17532137	Myd88	myeloid differentiation primary response gene 88	48.44816	54.80365	67.81253	51.38552	46.55519	45.26989
17398139	Mlf1	myeloid leukemia factor 1	3.569347	2.648949	2.462869	2.984419	2.828917	2.921947
17462975	Mlf2	myeloid leukemia factor 2	325.9123	290.6441	324.7285	286.758	247.4992	241.5286
17230067	Mndal	myeloid nuclear differentiation antigen like	7.586371	9.163063	13.22421	8.975921	12.77791	10.68185
17486635	Mzf1	myeloid zinc finger 1	8.054639	7.936539	9.978964	6.657082	6.914045	12.33632
17346387	Mllt1	myeloid/lymphoid or mixed-lineage leukemia (trithorax homolog, Drosophila); trar	25.70042	24.9464	27.60237	32.7193	23.47137	25.33096
17367243	Mllt10	myeloid/lymphoid or mixed-lineage leukemia (trithorax homolog, Drosophila); trar	52.13571	54.84165	55.06181	54.28155	50.32523	53.78903
17407738	Mllt11	myeloid/lymphoid or mixed-lineage leukemia (trithorax homolog, Drosophila); trar	8.429283	8.950449	11.77455	12.08587	11.63097	9.077909
17427033	Mllt3	myeloid/lymphoid or mixed-lineage leukemia (trithorax homolog, Drosophila); trar	11.18404	11.25856	13.2761	10.38932	10.05011	11.20492
17333418	Mllt4	myeloid/lymphoid or mixed-lineage leukemia (trithorax homolog, Drosophila); trar	65.22757	64.19771	69.78379	65.44223	62.76246	59.3333
17255828	Mllt6	myeloid/lymphoid or mixed-lineage leukemia (trithorax homolog, Drosophila); trar	32.85634	32.09752	36.51633	38.03221	34.9621	37.68896
17473127	Myadm	myeloid-associated differentiation marker	25.6819	32.46673	49.66439	29.10006	30.51782	35.81743
17273253	Myadml2	myeloid-associated differentiation marker-like 2	4.325899	4.801649	4.264599	4.706387	3.468039	4.681043
17259438	Myadml2os	myeloid-associated differentiation marker-like 2, opposite strand [Source:MGI Sym	8.261937	7.095577	8.052462	9.870478	7.69526	8.166512
17225586	Myeov2	myeloma overexpressed 2	23.80166	20.52428	19.39926	19.60745	17.97313	19.41729
17254877	Mpo	myeloperoxidase	5.658384	6.704408	5.571787	4.362152	5.337115	5.102273
17429191	Mpl	myeloproliferative leukemia virus oncogene	10.07767	12.22447	11.34386	10.68459	8.728175	10.05876
17451186	Miat	myocardial infarction associated transcript (non-protein coding)	4.681043	5.091321	4.681043	3.643816	4.562043	4.486405
17365700	Mirt1	myocardial infarction associated transcript 1	7.540081	7.095823	6.810072	7.32846	6.837552	5.694503
17318422	Mirt2	myocardial infraction associated transcript 2	12.45307	11.13176	14.31296	15.7771	9.519	17.49729
17528673	Myzap	myocardial zonula adherens protein	9.963827	8.907188	9.292972	9.292972	9.292972	9.560857
17264159	Myocd	myocardin	5.656501	6.17663	6.17663	6.706779	5.927033	5.59113
17218752	Myoc	myocilin	6.020945	5.292722	3.64791	5.488036	4.242398	4.358646
17491979	Mef2a	myocyte enhancer factor 2A	124.4783	160.4278	147.7936	123.1609	114.645	123.8955
17501828	Mef2b	myocyte enhancer factor 2B	3.719014	4.84387	3.883453	4.982086	6.01936	4.517235
17288917	Mef2c	myocyte enhancer factor 2C	12.35489	10.88817	12.47078	10.16297	10.13869	11.85702
17398970	Mef2d	myocyte enhancer factor 2D	69.73447	73.98136	83.84774	67.88872	50.82795	55.65309
17345728	Mdfi	MyoD family inhibitor	14.99584	11.01578	13.12359	12.60295	9.001154	11.21999
17456121	Mdfic	MyoD family inhibitor domain containing	47.3017	54.78162	52.09489	45.50774	48.6946	48.6946
17364280	Myof	myoferlin	9.117827	11.34646	11.53	7.720049	8.113419	9.183219
17478175	Myod1	myogenic differentiation 1	5.876104	7.120359	5.160926	7.758835	8.144917	7.047248
17244726	Myf5	myogenic factor 5	9.94244	9.03566	9.713896	9.029963	11.96576	8.337703
17244732	Myf6	myogenic factor 6	4.765277	5.974964	5.644282	5.31478	6.013938	5.31478
17217428	Myog	myogenin	15.86714	18.00094	10.39163	16.66501	10.61138	17.923
17318741	Mb	myoglobin	8.017987	7.386751	7.046222	6.022991	7.558395	8.688578
17501976	Isyna1	myo-inositol 1-phosphate synthase A1	41.62793	73.06608	117.5076	52.22215	45.43903	42.30335
17314120	Miox	myo-inositol oxygenase	9.344452	7.76061	7.274711	6.632717	7.298854	7.615031
17339395	Myom1	myomesin 1	3.720303	3.207279	4.83498	5.036215	3.489112	3.57353
17499485	Myom2	myomesin 2	3.260289	3.884907	4.462493	4.086625	3.587876	3.709899
17419902	Myom3	myomesin family, member 3	8.138258	8.352453	6.88745	8.138258	8.731987	8.138258
17396553	Mynn	myoneurin	14.95183	16.23595	11.03473	12.61764	15.53069	11.7944
17241553	Mypn	myopalladin	6.201856	6.050146	5.483055	5.952889	6.091848	5.568929
17234131	Mrln	myoregulin	3.861443	3.274989	3.703322	4.280561	3.160757	4.135094
17440885	Myo1h	myosin 1H	5.650114	5.916566	4.357619	5.50129	5.71558	6.276151
17373189	Mybpc3	myosin binding protein C, cardiac	7.087221	5.936078	6.429382	6.5765	7.008958	6.942042
17490245	Mybpc2	myosin binding protein C, fast-type	3.066281	4.485597	4.597151	5.088252	3.729055	4.485597
17243910	Mybpc1	myosin binding protein C, slow-type	6.225544	6.817865	6.185328	7.676986	7.204309	6.633545
17217415	Mybph	myosin binding protein H	9.98097	11.32618	9.658974	11.64993	11.0022	12.37108
17401699	Mybphl	myosin binding protein H-like	4.387868	4.595303	4.84874	3.953745	6.002485	6.421231
17300390	Mhrt	myosin heavy chain associated RNA transcript	6.431878	7.386034	6.716222	7.333643	6.865287	8.853083
17238111	Myo1a	myosin IA	3.016551	3.803164	4.235346	3.884153	5.180172	4.639052
17222837	Myo1b	myosin IB	253.1941	207.8343	167.3181	248.5289	247.6313	235.4978
17253055	Myo1c	myosin IC	50.86389	47.24501	47.81429	42.59375	38.39616	36.57383
17266698	Myo1d	myosin ID	52.70617	47.40443	66.06297	42.68508	39.49368	40.26767
17519022	Myo1e	myosin IE	82.28343	71.40534	79.23742	73.16694	81.07471	76.37137
17336114	Myo1f	myosin IF	9.488237	9.488237	10.66876	10.02639	7.905737	8.434836
17260369	Myo1g	myosin IG	4.788388	6.914189	9.561785	7.749429	5.611786	5.529772
17367473	Myo3a	myosin IIIA	3.276238	2.776197	3.024172	3.263862	2.456288	2.944025
17371574	Myo3b	myosin IIIB	2.797349	3.336927	2.852645	2.828035	2.862528	2.802394
17518038	Myo9a	myosin IXa	18.928	22.88071	27.38758	15.56777	21.17749	19.16032
17502102	Myo9b	myosin IXb	34.95144	42.5669	44.64417	40.66935	44.02741	35.40153
17511336	Mylk3	myosin light chain kinase 3	4.208482	4.015362	4.086229	3.884934	4.67968	4.424364
17291674	Mylk4	myosin light chain kinase family, member 4	3.630303	3.18656	3.263862	3.263862	3.625525	4.629672
17483242	Mylpf	myosin light chain, phosphorylatable, fast skeletal muscle	4.270189	3.232141	3.314084	3.666669	4.538325	3.326834
17250266	Mprip	myosin phosphatase Rho interacting protein	81.76661	100.0432	108.667	94.16702	82.05788	88.16414
17286962	Mylip	myosin regulatory light chain interacting protein	55.29856	54.87131	63.96097	59.0891	41.61524	44.19006
17519394	Myo5a	myosin VA	3.034481	3.248266	3.176923	3.567195	3.414158	3.335609
17351760	Myo5b	myosin VB	38.94483	40.00671	45.3151	42.24533	36.72371	38.90949
17519438	Myo5c	myosin VC	6.866953	7.408801	8.481974	6.258253	6.921429	6.369764
17519821	Myo6	myosin VI	124.1611	103.1321	96.4915	110.0542	99.48244	91.06912
17493467	Myo7a	myosin VIIA	27.30475	26.12828	29.00521	26.599	23.26824	24.71594

17523201	Myrip	myosin VIIA and Rab interacting protein	9.341084	8.832979	7.858206	8.310758	8.51685	7.495683
17353271	Myo7b	myosin VIIB	7.017854	8.6288	7.952729	7.085796	7.344427	8.01832
17310589	Myo10	myosin X	117.519	109.3637	107.0158	97.69684	91.52792	84.17442
17254473	Myo19	myosin XIX	23.82295	25.05332	25.05332	24.29454	22.06503	21.61139
17250392	Myo15	myosin XV	6.030928	6.47464	5.322928	6.47464	6.030928	5.56106
17258374	Myo15b	myosin XVB	11.1024	11.6631	11.85785	12.88982	13.06233	9.182456
17498906	Myo16	myosin XVI	3.328172	3.513892	3.225784	3.551307	3.144629	3.494001
17253316	Myo18a	myosin XVIIIA	64.50594	63.96097	60.01745	56.47851	66.98193	60.82785
17451251	Myo18b	myosin XVIIIb	7.56972	7.580011	6.953889	6.991781	8.032951	5.443219
17326105	Myh15	myosin, heavy chain 15	2.522388	2.679883	2.855671	3.634684	2.997791	2.986819
17378395	Myh7b	myosin, heavy chain 7B, cardiac muscle, beta	6.746176	5.509342	7.046906	6.350939	5.660188	6.081006
17251046	Myh1	myosin, heavy polypeptide 1, skeletal muscle, adult	5.074832	4.127476	2.837322	3.149887	3.640332	3.473283
17251370	Myh10	myosin, heavy polypeptide 10, non-muscle	21.21599	17.77084	25.04898	17.51076	16.6746	20.15352
17328368	Myh11	myosin, heavy polypeptide 11, smooth muscle	10.75242	11.50086	11.72048	11.50086	14.72484	11.48588
17251179	Myh13	myosin, heavy polypeptide 13, skeletal muscle	4.271136	3.67285	3.222499	3.614084	3.13147	5.479256
17490328	Myh14	myosin, heavy polypeptide 14	20.10998	27.04687	30.66987	30.96271	25.97533	28.61362
17251001	Myh2	myosin, heavy polypeptide 2, skeletal muscle, adult	6.006856	5.644908	5.072335	6.246595	3.980501	4.389449
17250958	Myh3	myosin, heavy polypeptide 3, skeletal muscle, embryonic	4.136584	3.665881	3.343201	3.112751	3.138358	3.296054
17251091	Myh4	myosin, heavy polypeptide 4, skeletal muscle	3.815732	4.852136	3.620252	4.115221	3.451587	4.154803
17306532	Myh6	myosin, heavy polypeptide 6, cardiac muscle, alpha	6.476166	6.640169	7.626547	5.651759	7.27799	6.798281
17306577	Myh7	myosin, heavy polypeptide 7, cardiac muscle, beta	5.554511	7.316684	7.093118	7.165314	6.501443	7.524731
17251133	Myh8	myosin, heavy polypeptide 8, skeletal muscle, perinatal	3.005198	2.710201	3.339703	3.222522	2.815459	3.41378
17318830	Myh9	myosin, heavy polypeptide 9, non-muscle	183.1142	203.0569	223.63	175.1698	234.924	177.1687
17443451	Myl10	myosin, light chain 10, regulatory	10.1837	13.5845	6.451569	12.4643	13.39293	15.01945
17346964	Myl12a	myosin, light chain 12A, regulatory, non-sarcomeric	5.823716	8.467112	5.617429	5.050898	6.452508	4.886969
17346956	Myl12b	myosin, light chain 12B, regulatory	123.9591	134.0279	140.8602	123.2992	112.0478	115.4945
17223916	Myl1	myosin, light polypeptide 1	4.25176	4.217038	3.672086	4.429028	3.904912	4.90754
17442010	Myl2	myosin, light polypeptide 2, regulatory, cardiac, slow	8.497509	8.779568	8.636819	7.193431	8.136171	8.257643
17522439	Myl3	myosin, light polypeptide 3	8.172854	8.702259	8.207142	8.001442	8.907558	8.885605
17257221	Myl4	myosin, light polypeptide 4	8.640891	5.199958	6.832057	5.619065	6.96962	6.87953
17246146	Myl6	myosin, light polypeptide 6, alkali, smooth muscle and non-muscle	8.189356	10.43226	11.74496	11.61398	9.888009	7.422525
17246154	Myl6b	myosin, light polypeptide 6B	9.328338	9.214399	8.057208	6.7635	6.992896	8.057208
17260236	Myl7	myosin, light polypeptide 7, regulatory	4.876784	5.100151	4.574646	4.480718	6.991684	5.749235
17378663	Myl9	myosin, light polypeptide 9, regulatory	17.5325	27.29548	28.14253	24.85131	19.75204	23.06015
17325159	Mylk	myosin, light polypeptide kinase	58.73832	62.3285	49.51007	62.33541	52.87339	63.18718
17377816	Mylk2	myosin, light polypeptide kinase 2, skeletal muscle	9.641315	10.05325	7.934669	10.28807	13.3078	13.69161
17212813	Mstn	myostatin	2.90589	2.251974	2.562372	4.237108	2.811929	2.563989
17350267	Myot	myotilin	4.849312	3.713733	4.647678	5.168193	5.189552	4.016086
17465830	Mtpn	myotrophin	220.1144	197.7316	178.874	185.5366	189.3787	195.9063
17535319	Mtmr1	myotubularin related protein 1	76.17153	62.27063	52.53875	67.45905	64.20661	62.95679
17478864	Mtmr10	myotubularin related protein 10	77.14175	81.63184	58.69802	70.95045	80.53366	82.85059
17400486	Mtmr11	myotubularin related protein 11	5.331679	7.698994	15.9156	6.926085	6.992896	8.297634
17310432	Mtmr12	myotubularin related protein 12	26.67026	30.00186	33.60095	27.89201	24.3701	26.91186
17461554	Mtmr14	myotubularin related protein 14	31.41932	19.42873	24.19639	22.64028	20.96862	22.64028
17514691	Mtmr2	myotubularin related protein 2	90.16902	70.55518	76.09449	80.46949	71.07006	67.43427
17259992	Mtmr3	myotubularin related protein 3	62.19471	60.72295	67.70075	63.12633	56.5796	60.28888
17254776	Mtmr4	myotubularin related protein 4	61.83192	40.60625	43.13509	48.19862	41.25051	42.07613
17301010	Mtmr6	myotubularin related protein 6	51.20526	53.3579	61.04876	52.21383	54.30375	48.71452
17508885	Mtmr7	myotubularin related protein 7	4.144362	4.801649	3.986244	3.855051	3.470251	4.592978
17307650	Mtmr9	myotubularin related protein 9	79.17648	73.81387	75.05415	73.02507	57.63767	72.66657
17303874	Myoz1	myozenin 1	3.552759	3.327618	4.016893	3.876299	4.002746	3.855612
17410022	Myoz2	myozenin 2	3.286655	3.175404	2.751484	3.095173	4.127162	3.472826
17354599	Myoz3	myozenin 3	5.607042	8.070958	8.108303	7.156132	6.992896	7.12579
17240226	Marcks	myristoylated alanine rich protein kinase C substrate	53.58993	59.41849	62.70854	50.12644	47.96998	56.1989
17418447	Meaf6	MYST/Esa1-associated factor 6	19.03431	18.96083	15.54598	18.90754	15.67963	22.1722
17542486	Naa10	N(alpha)-acetyltransferase 10, NatA catalytic subunit	5.521422	4.203351	7.326631	5.417022	6.350763	5.039253
17449931	Naa11	N(alpha)-acetyltransferase 11, NatA catalytic subunit	4.875229	5.149849	5.062781	5.808799	7.248035	5.753022
17397439	Naa15	N(alpha)-acetyltransferase 15, NatA auxiliary subunit	96.35315	70.704	71.66665	74.31492	72.02071	73.12283
17308888	Naa16	N(alpha)-acetyltransferase 16, NatA auxiliary subunit	33.73677	28.08407	23.78055	26.36112	25.06479	26.88538
17377199	Naa20	N(alpha)-acetyltransferase 20, NatB catalytic subunit	14.87358	21.30204	25.25768	22.32828	22.19434	24.55968
17441895	Naa25	N(alpha)-acetyltransferase 25, NatB auxiliary subunit	58.18236	67.97677	56.20747	82.21103	65.71679	64.39336
17299465	Naa30	N(alpha)-acetyltransferase 30, NatC catalytic subunit	73.00129	60.50532	50.7199	64.20172	62.30647	60.01287
17287928	Naa35	N(alpha)-acetyltransferase 35, NatC auxiliary subunit	61.74027	54.36288	46.27336	57.38374	57.16103	67.72281
17251643	Naa38	N(alpha)-acetyltransferase 38, NatC auxiliary subunit	56.19267	53.69627	51.71819	54.63146	57.50359	52.60835
17362279	Naa40	N(alpha)-acetyltransferase 40, NatD catalytic subunit, homolog (S. cerevisiae)	27.13776	23.51338	22.35739	26.55497	26.62778	30.59958
17325761	Naa50	N(alpha)-acetyltransferase 50, NatE catalytic subunit	174.4041	173.2474	148.5722	145.2103	152.4128	153.4591
17322476	Naa60	N(alpha)-acetyltransferase 60, NatF catalytic subunit	123.681	108.6655	113.6143	95.59147	90.39932	96.38455
17326846	N6amt1	N-6 adenine-specific DNA methyltransferase 1 (putative)	12.08596	12.08596	14.63388	12.40483	12.87321	12.39056
17307151	N6amt2	N-6 adenine-specific DNA methyltransferase 2 (putative)	22.66682	22.62789	22.42786	26.36112	18.54425	18.29681
17430645	Nkain1	Na+/K+ transporting ATPase interacting 1	6.880484	5.592952	5.749594	7.592211	5.69178	7.268361
17240109	Nkain2	Na+/K+ transporting ATPase interacting 2	6.2537	7.000025	5.210277	5.980682	5.87187	5.843003
17423601	Nkain3	Na+/K+ transporting ATPase interacting 3	7.750772	5.098066	5.386134	5.386134	7.824833	5.2288
17395707	Nkain4	Na+/K+ transporting ATPase interacting 4	11.89992	12.99712	12.13582	13.79449	11.7243	13.66733
17260406	Nacad	NAC alpha domain containing	6.460116	12.63978	11.56746	10.3293	11.56746	9.798698
17319607	Naga	N-acetyl galactosaminidase, alpha	53.40119	58.82796	57.31656	58.19648	60.48351	63.93305
17501565	Nat1	N-acetyl transferase 1	2.63918	2.96662	3.486115	2.969335	3.191976	3.187952
17524264	Naalad2	N-acetylated alpha-linked acidic dipeptidase 2	4.517705	5.661483	4.063069	4.941949	5.173318	5.050758
17356642	Naaladl1	N-acetylated alpha-linked acidic dipeptidase-like 1	4.441971	5.780603	5.602614	6.824342	5.26298	4.802914
17404373	Naaladl2	N-acetylated alpha-linked acidic dipeptidase-like 2	21.06258	24.36114	18.49649	20.12532	17.44437	27.47676
17460133	Nagk	N-acetylglucosamine kinase	18.43876	19.20873	18.53564	19.44423	16.37996	18.60167
17236339	Gnptab	N-acetylglucosamine-1-phosphate transferase, alpha and beta subunits	23.41011	25.97425	25.27466	33.84615	26.85465	26.59937
17327934	Nagpa	N-acetylglucosamine-1-phosphodiester alpha-N-acetylglucosaminidase	28.61183	30.14883	27.92954	26.54816	26.06009	33.26516
17342167	Gnptg	N-acetylglucosamine-1-phosphotransferase, gamma subunit	52.88585	53.34718	63.73438	57.41119	64.70162	60.37293
17256882	Nags	N-acetylglutamate synthase	97.40406	88.64762	88.50027	101.7377	95.26009	95.9526

17228234	Npl	N-acetylneuraminate pyruvate lyase	21.16121	15.90799	17.07768	15.90799	13.19812	17.61497
17392780	Nanp	N-acetylneuraminic acid phosphatase	6.941272	6.648874	8.00649	7.725402	7.097741	7.373093
17413832	Nans	N-acetylneuraminic acid synthase (sialic acid synthase)	19.63833	15.38732	10.9497	15.52681	14.71097	14.99303
17388821	Nat10	N-acetyltransferase 10	32.6964	36.01086	32.10531	36.74637	28.45401	33.47077
17473417	Nat14	N-acetyltransferase 14	7.226965	9.086911	7.703585	4.470791	7.235386	7.235386
17501569	Nat2	N-acetyltransferase 2 (arylamine N-acetyltransferase)	21.63282	17.09864	16.76883	19.04315	21.31475	21.97163
17501574	Nat3	N-acetyltransferase 3	3.787485	3.877992	3.589368	3.207546	4.352065	3.787485
17521441	Nat6	N-acetyltransferase 6	77.46324	106.8513	147.6728	99.12314	76.05546	93.17235
17468438	Nat8	N-acetyltransferase 8 (GCN5-related, putative)	4.77073	4.468374	2.67402	4.858968	3.048818	3.253742
17468467	Nat8b	N-acetyltransferase 8B [Source:MGI Symbol;Acc:MGI:3644831]	15.06898	10.07928	13.08779	11.50405	9.155889	10.29227
17436545	Nat8l	N-acetyltransferase 8-like	5.074023	6.139325	5.40858	6.338537	6.154066	6.054593
17271789	Nat9	N-acetyltransferase 9 (GCN5-related, putative)	18.29516	15.30509	17.87424	20.74541	22.55632	21.90837
17263698	Natd1	N-acetyltransferase domain containing 1	26.48842	26.66989	22.89626	28.62254	28.54666	26.76118
17502470	Nwd1	NACHT and WD repeat domain containing 1	5.937806	6.638328	6.701621	7.104288	7.495008	6.480836
17437585	Nwd2	NACHT and WD repeat domain containing 2	3.945286	4.384645	3.600232	3.076479	2.93325	3.755123
17445860	Napepld	N-acyl phosphatidylethanolamine phospholipase D	9.915393	6.481016	8.860327	6.391436	6.677	6.303006
17449673	Naaa	N-acylethanolamine acid amidase	19.36608	19.77328	20.71121	18.20799	17.50312	21.39127
17508949	Asah1	N-acylsphingosine amidohydrolase 1	77.24341	80.10442	82.55937	64.93255	68.47142	71.1292
17363927	Asah2	N-acylsphingosine amidohydrolase 2	66.36129	86.01283	75.09682	79.00105	63.09615	72.2362
17422587	Nadk	NAD kinase	187.451	205.2801	209.6327	197.4002	162.7925	183.2044
17310187	Nadk2	NAD kinase 2, mitochondrial	252.5456	220.1541	202.6744	232.5698	229.0296	258.5608
17498370	Nadsyn1	NAD synthetase 1	21.90077	23.34789	19.14479	23.04577	23.34789	23.34789
17535434	Nsdhl	NAD(P) dependent steroid dehydrogenase-like	126.9459	108.903	127.286	118.393	164.503	117.6217
17512732	Nqo1	NAD(P)H dehydrogenase, quinone 1	35.87955	39.40454	42.72327	39.07787	38.62786	33.67299
17286462	Nqo2	NAD(P)H dehydrogenase, quinone 2	99.6688	103.0828	84.46665	100.9859	86.40483	123.3505
17225564	Ndufa10	NADH dehydrogenase (ubiquinone) 1 alpha subcomplex 10	321.6148	321.3764	293.9201	310.5281	319.3512	324.4495
17338872	Ndufa11	NADH dehydrogenase (ubiquinone) 1 alpha subcomplex 11	14.80138	21.41975	22.26692	19.37709	21.6045	17.91716
17339864	Ndufaf7	NADH dehydrogenase (ubiquinone) 1 alpha subcomplex assembly factor 7	52.57664	46.97691	56.85006	59.52072	40.9213	51.27522
17541036	Ndufa1	NADH dehydrogenase (ubiquinone) 1 alpha subcomplex, 1	111.884	126.6919	137.3983	147.0222	172.6624	142.6484
17236681	Ndufa12	NADH dehydrogenase (ubiquinone) 1 alpha subcomplex, 12	69.78234	74.79034	74.79034	76.56589	76.07181	82.90344
17509891	Ndufa13	NADH dehydrogenase (ubiquinone) 1 alpha subcomplex, 13	11.82436	12.51599	12.64671	14.25781	11.74203	10.81191
17509899	Ndufa13	NADH dehydrogenase (ubiquinone) 1 alpha subcomplex, 13	178.7191	175.504	183.3123	201.0499	188.4947	199.5228
17353752	Ndufa2	NADH dehydrogenase (ubiquinone) 1 alpha subcomplex, 2	98.34979	92.49478	99.19668	111.7701	125.371	118.0161
17473171	Ndufa3	NADH dehydrogenase (ubiquinone) 1 alpha subcomplex, 3	244.2949	319.1365	299.8464	295.8209	298.7759	276.8921
17464836	Ndufa4	NADH dehydrogenase (ubiquinone) 1 alpha subcomplex, 4	716.5433	732.919	657.5287	732.5635	771.4987	854.8219
17238054	Ndufa4l2	NADH dehydrogenase (ubiquinone) 1 alpha subcomplex, 4-like 2	7.708873	11.28841	16.16565	9.89315	25.72929	11.72893
17465201	Ndufa5	NADH dehydrogenase (ubiquinone) 1 alpha subcomplex, 5	32.5747	47.59176	36.7059	44.83489	53.88344	52.05374
17319619	Ndufa6	NADH dehydrogenase (ubiquinone) 1 alpha subcomplex, 6 (B14)	167.5073	204.8366	204.9616	236.381	232.3281	260.1085
17336190	Ndufa7	NADH dehydrogenase (ubiquinone) 1 alpha subcomplex, 7 (B14.5a)	42.9942	35.36107	37.83134	36.31113	38.61582	37.0071
17384525	Ndufa8	NADH dehydrogenase (ubiquinone) 1 alpha subcomplex, 8	182.5351	241.4181	283.6288	237.3973	236.0895	237.3973
17471166	Ndufa9	NADH dehydrogenase (ubiquinone) 1 alpha subcomplex, 9	205.6276	198.2161	195.3016	209.8522	208.3565	220.0595
17389928	Ndufaf1	NADH dehydrogenase (ubiquinone) 1 alpha subcomplex, assembly factor 1	61.60861	59.71494	58.40419	62.92014	55.29243	61.30233
17296104	Ndufaf2	NADH dehydrogenase (ubiquinone) 1 alpha subcomplex, assembly factor 2	12.59405	15.75109	14.26977	11.50086	15.38337	16.94949
17531207	Ndufaf3	NADH dehydrogenase (ubiquinone) 1 alpha subcomplex, assembly factor 3	44.65222	33.53487	37.34699	45.39873	35.89448	37.82007
17412272	Ndufaf4	NADH dehydrogenase (ubiquinone) 1 alpha subcomplex, assembly factor 4	69.91112	57.16301	56.99763	69.50284	51.68522	56.30378
17213179	Ndufb3	NADH dehydrogenase (ubiquinone) 1 beta subcomplex 3	28.90826	32.26616	36.32775	30.88833	29.93684	35.98241
17330314	Ndufb4	NADH dehydrogenase (ubiquinone) 1 beta subcomplex 4	44.85447	46.66827	41.79707	43.66726	39.01021	42.93791
17365134	Ndufb8	NADH dehydrogenase (ubiquinone) 1 beta subcomplex 8	44.6742	37.92744	33.87479	40.77435	40.57277	47.15471
17475861	1700028B04Rik	NADH dehydrogenase (ubiquinone) 1 beta subcomplex 8 pseudogene	13.66658	13.06568	8.467112	13.39423	12.35189	13.792
17342029	Ndufb10	NADH dehydrogenase (ubiquinone) 1 beta subcomplex, 10	58.93325	68.21703	73.09699	77.16528	60.37042	84.38765
17540490	Ndufb11	NADH dehydrogenase (ubiquinone) 1 beta subcomplex, 11	11.74455	11.04951	10.18991	12.35189	11.46934	11.74455
17457540	Ndufb2	NADH dehydrogenase (ubiquinone) 1 beta subcomplex, 2	34.00889	31.89437	28.99797	34.20582	39.24372	38.32835
17396750	Ndufb5	NADH dehydrogenase (ubiquinone) 1 beta subcomplex, 5	76.47626	58.0772	63.80422	67.95369	64.21551	75.19266
17424023	Ndufb6	NADH dehydrogenase (ubiquinone) 1 beta subcomplex, 6	95.61864	88.97388	91.46133	108.0361	98.70081	107.0737
17502944	Ndufb7	NADH dehydrogenase (ubiquinone) 1 beta subcomplex, 7	42.89091	43.59649	50.44048	52.28844	53.369	55.45286
17311796	Ndufb9	NADH dehydrogenase (ubiquinone) 1 beta subcomplex, 9	986.6195	967.9509	940.6407	926.3605	875.9411	933.4442
17495921	Ndufab1	NADH dehydrogenase (ubiquinone) 1, alpha/beta subcomplex, 1	37.00504	38.00507	36.6825	35.6945	29.22113	34.27489
17405133	Ndufc1	NADH dehydrogenase (ubiquinone) 1, subcomplex unknown, 1	84.52405	95.03651	95.03651	95.03651	95.03651	93.65863
17480343	Ndufc2	NADH dehydrogenase (ubiquinone) 1, subcomplex unknown, 2	241.7614	228.9153	214.327	258.6666	228.7598	287.0106
17376917	Ndufaf5	NADH dehydrogenase (ubiquinone) complex I, assembly factor 5	8.230385	6.103387	6.55661	9.009019	6.497479	7.423605
17423215	Ndufaf6	NADH dehydrogenase (ubiquinone) complex I, assembly factor 6	24.97876	21.28861	23.11696	24.41236	19.18065	26.17776
17223685	Ndufs1	NADH dehydrogenase (ubiquinone) Fe-S protein 1	175.6951	168.5508	166.3191	186.1936	154.9813	192.9843
17229665	Ndufs2	NADH dehydrogenase (ubiquinone) Fe-S protein 2	231.5982	243.7942	224.8283	245.4151	220.3037	256.277
17388074	Ndufs3	NADH dehydrogenase (ubiquinone) Fe-S protein 3	33.33348	34.47551	30.70347	34.27988	34.27988	35.19918
17296336	Ndufs4	NADH dehydrogenase (ubiquinone) Fe-S protein 4	84.70058	93.9662	95.25745	92.38073	90.98394	95.29443
17429803	Ndufs5	NADH dehydrogenase (ubiquinone) Fe-S protein 5	3.727969	3.501881	3.458652	3.924231	3.517401	2.903675
17294235	Ndufs6	NADH dehydrogenase (ubiquinone) Fe-S protein 6	39.21028	48.18693	50.71674	46.11806	49.39387	47.30825
17235268	Ndufs7	NADH dehydrogenase (ubiquinone) Fe-S protein 7	195.6037	208.4215	203.4697	203.5416	176.8276	204.3219
17361032	Ndufs8	NADH dehydrogenase (ubiquinone) Fe-S protein 8	188.4072	143.7592	149.1851	154.3434	147.4774	144.6618
17361056	Ndufv1	NADH dehydrogenase (ubiquinone) flavoprotein 1	210.5691	194.7528	193.9297	213.3547	193.086	205.6076
17346802	Ndufv2	NADH dehydrogenase (ubiquinone) flavoprotein 2	159.8993	153.7508	144.4414	177.0545	160.559	191.1922
17335865	Ndufv3	NADH dehydrogenase (ubiquinone) flavoprotein 3	40.92385	38.55778	51.09428	45.44092	41.57228	49.25506
17325719	BC002163	NADH dehydrogenase Fe-S protein 5 pseudogene	104.2974	80.77517	86.08978	95.66836	85.64458	89.09669
17532603	ND1	NADH dehydrogenase subunit 1	5689.831	5940.835	5776.981	5776.981	5770.498	5872.992
17532607	ND2	NADH dehydrogenase subunit 2	5434.72	5677.342	5454.042	5432.498	5227.915	5504.02
17532621	ND3	NADH dehydrogenase subunit 3	4715.091	5008.698	5165.205	5013.387	4850.171	5148.977
17532625	ND4	NADH dehydrogenase subunit 4	7163.555	7322.142	7111.608	7260.484	7120.141	7374.86
17221871	ND4L	NADH dehydrogenase subunit 4L	5387.648	5328.708	5184.036	5378.805	5203.26	5396.731
17532631	ND5	NADH dehydrogenase subunit 5	6547.734	6731.909	6316.107	6431.591	6398.021	6592.911
17532649	ND6	NADH dehydrogenase subunit 6	1007.637	1253.964	1384.05	1218.128	1313.654	1328.323
17282828	Noxred1	NADP+ dependent oxidoreductase domain containing 1	7.218905	5.741071	5.418261	6.785758	4.625919	5.443634
17382459	Ndor1	NADPH dependent diflavin oxidoreductase 1	29.00239	26.45099	23.60744	31.63786	25.39583	20.50622
17544424	Nox1	NADPH oxidase 1	3.39874	3.366175	3.479162	3.280351	3.594921	3.273741
17340458	Nox3	NADPH oxidase 3	5.671892	6.492572	6.037662	8.688578	5.480699	6.958855

17479974	Nox4	NADPH oxidase 4	57.99393	32.31451	18.70588	39.67697	49.19603	38.62786
17382363	Noxa1	NADPH oxidase activator 1	6.519675	6.113252	6.168287	6.212139	6.603816	6.704315
17334381	Noxo1	NADPH oxidase organizer 1	7.778705	7.778705	7.778705	10.44673	7.329222	7.778705
17503650	Nkd1	naked cuticle 1 homolog (Drosophila)	15.47342	13.96071	16.77569	14.39325	13.64462	14.35449
17294289	Nkd2	naked cuticle 2 homolog (Drosophila)	9.749917	11.03015	11.06691	10.48722	8.311276	11.30195
17545824	Nhs	Nance-Horan syndrome (human)	4.851901	4.700942	4.584327	4.50957	6.108211	4.386834
17462694	Nanog	Nanog homeobox	3.753353	4.577976	4.208949	4.147667	3.880493	3.998669
17360873	Nanos1	nanos homolog 1 (Drosophila)	14.64676	13.08734	11.88879	11.16832	9.201697	11.02709
17474385	Nanos2	nanos homolog 2 (Drosophila)	4.475783	4.392919	4.232265	4.592883	3.613157	4.399715
17511069	Nanos3	nanos homolog 3 (Drosophila)	5.394839	5.079653	5.788863	4.639277	4.340376	5.531804
17477508	Napsa	napsin A aspartic peptidase	5.053875	5.385835	4.786928	4.864967	4.786762	3.823304
17416773	Nrd1	nardilysin, N-arginine dibasic convertase, NRD convertase 1	143.9027	135.2317	136.2205	136.5097	142.2594	137.69
17238226	Naca	nascent polypeptide-associated complex alpha polypeptide	289.8314	284.7792	328.095	289.6004	315.4986	320.0204
17407306	Npr1	natriuretic peptide receptor 1	22.96349	14.75958	15.87451	16.36668	15.26229	17.36788
17413403	Npr2	natriuretic peptide receptor 2	60.35662	49.92152	54.1185	73.63961	53.48639	52.00001
17316043	Npr3	natriuretic peptide receptor 3	8.428115	11.12914	9.344193	11.24171	9.456983	8.139612
17421545	Nppa	natriuretic peptide type A	8.679669	7.450256	7.008278	8.51685	9.326657	9.888077
17421540	Nppb	natriuretic peptide type B	7.177742	4.988341	5.683935	6.085771	5.287919	5.882625
17225191	Nppc	natriuretic peptide type C	9.73189	11.71577	11.41208	10.28807	9.788311	12.85003
17473300	Ncr1	natural cytotoxicity triggering receptor 1	3.972563	6.781009	4.923178	6.970345	6.767533	4.994014
17477101	Nkg7	natural killer cell group 7 sequence	10.52976	9.832444	10.29399	10.72778	9.310251	9.466493
17523332	Nktr	natural killer tumor recognition sequence	37.17189	35.69673	35.36107	40.47586	37.877	33.30992
17315570	Nckap1l	NCK associated protein 1 like	23.69449	35.92908	35.95723	27.88776	26.08123	24.68033
17521917	Nckipsd	NCK interacting protein with SH3 domain	17.25449	19.4708	24.76464	14.00772	14.48271	18.04854
17387316	Nckap1	NCK-associated protein 1	235.8948	238.4826	227.0223	238.8266	222.8918	235.5811
17226447	Nckap5	NCK-associated protein 5	4.947021	4.801649	5.049743	4.97515	4.384523	4.10488
17321561	Nckap5l	NCK-associated protein 5-like	12.98577	12.65609	12.93071	13.07909	13.96874	12.65469
17416481	Ndc1	NDC1 transmembrane nucleoporin	22.64264	19.0384	18.24096	20.33143	19.83147	19.19262
17347042	Ndc80	NDC80 homolog, kinetochore complex component (S. cerevisiae)	5.482941	5.000838	6.245772	3.703322	6.223603	6.31998
17340411	2700099C18Rik	NDC80 homolog, kinetochore complex component pseudogene	4.676081	3.568629	2.887398	4.259576	4.114309	4.533766
17354629	Ndst1	N-deacetylase/N-sulfotransferase (heparan glucosaminyl) 1	147.4498	158.3958	165.5531	154.7612	142.3827	147.3732
17303897	Ndst2	N-deacetylase/N-sulfotransferase (heparan glucosaminyl) 2	28.11757	25.72805	26.42021	26.64476	29.14426	28.20052
17410067	Ndst3	N-deacetylase/N-sulfotransferase (heparan glucosaminyl) 3	4.397642	3.334291	3.995123	5.200715	5.173892	3.726574
17402412	Ndst4	N-deacetylase/N-sulfotransferase (heparin glucosaminyl) 4	2.318787	2.604434	2.477577	2.909699	2.691182	2.773985
17381940	Nebl	nebulette	3.925401	4.42234	4.20927	3.944821	4.20927	4.20927
17385099	Neb	nebulin	20.64757	23.15433	21.25441	29.9605	51.37662	30.00789
17365803	Nrap	nebulin-related anchoring protein	4.636416	4.409974	4.966949	4.859002	5.048063	4.931751
17462719	Necap1	NECAP endocytosis associated 1	135.3555	128.9118	139.8649	130.1318	140.5442	146.7493
17432124	Necap2	NECAP endocytosis associated 2	47.93043	46.77546	48.87653	44.81811	40.15729	38.32994
17478789	Ndn	necdin	5.377703	7.111136	5.592642	6.186056	4.830625	5.518935
17491846	Ndnl2	necdin-like 2	33.8539	30.20049	29.31282	30.75822	31.86631	28.4923
17511453	N4bp1	NEDD4 binding protein 1	30.21808	31.73318	39.30552	27.8924	30.865	29.76118
17437803	N4bp2	NEDD4 binding protein 2	44.2664	40.38199	34.73384	43.13479	40.30173	52.96546
17448369	N4bp2os	NEDD4 binding protein 2, opposite strand [Source:MGI Symbol;Acc:MGI:1921484]	6.001445	6.318973	5.788341	5.926499	7.74143	5.27357
17455554	N4bp2l1	NEDD4 binding protein 2-like 1	64.09589	98.448	80.81941	101.0643	124.9459	114.4275
17455562	N4bp2l2	NEDD4 binding protein 2-like 2	70.34666	67.7322	46.18172	57.66804	58.10579	63.38546
17262506	N4bp3	NEDD4 binding protein 3	14.90971	15.51875	15.36142	16.81515	15.17011	15.83385
17350011	Ndfip1	Nedd4 family interacting protein 1	224.819	203.0949	185.304	195.1555	159.2788	213.3754
17302550	Ndfip2	Nedd4 family interacting protein 2	60.94813	64.02795	64.02795	60.74695	67.9066	66.10466
17512275	Nae1	NEDD8 activating enzyme E1 subunit 1	45.10952	39.13208	36.32019	40.97352	43.00642	42.16547
17447170	Nelfa	negative elongation factor complex member A, Whsc2	42.24885	41.0964	39.97151	36.30182	36.8944	40.74949
17382392	Nelfb	negative elongation factor complex member B	39.42968	33.63427	32.36786	34.60959	33.98014	25.01983
17380340	Nelfcd	negative elongation factor complex member C/D, Th1l	46.88128	37.27897	41.09098	36.77542	38.77191	43.87417
17336738	Nelfe	negative elongation factor complex member E, Rdbp	38.6201	35.90368	30.15865	46.43047	35.89448	32.72089
17329819	Nrros	negative regulator of reactive oxygen species	11.40386	12.03263	13.39423	11.26191	13.43198	10.28778
17435422	Nub1	negative regulator of ubiquitin-like proteins 1	59.57644	65.38419	58.31318	63.46459	59.72984	71.52323
17527481	Neil1	nei endonuclease VIII-like 1 (E. coli)	14.57749	13.37067	13.37067	14.49045	11.04269	11.69695
17307598	Neil2	nei like 2 (E. coli)	10.67778	11.63033	10.72986	9.888557	9.399409	10.71946
17509361	Neil3	nei like 3 (E. coli)	7.112171	8.250491	6.929734	6.864478	6.762609	7.215953
17256754	Nbr1	neighbor of Brca1 gene 1	281.6813	255.3567	250.3374	273.6523	271.6321	277.4108
17478406	Nell1	NEL-like 1	4.377509	4.551367	4.653674	4.633107	4.025115	4.265131
17491406	Nell1os	NEL-like 1, opposite strand	4.130596	3.654946	3.958489	4.337429	3.914125	4.526983
17320813	Nell2	NEL-like 2	4.664136	5.391101	5.374386	4.664654	4.420808	5.429352
17266498	Nlk	nemo like kinase	40.95705	36.31466	44.6807	34.52358	30.39222	35.15018
17527735	Neo1	neogenin	34.97034	35.52419	34.96792	34.86578	31.88995	30.37474
17465733	Npn2	neoplastic progression 2	3.055101	2.794713	3.659001	3.068195	4.219085	3.299735
17311519	Nov	nephroblastoma overexpressed gene	5.735702	5.735702	5.225467	5.735702	5.596093	5.704854
17233286	Nepn	nephrocan	3.603128	3.536176	3.11301	3.354063	3.644094	2.749635
17410435	Npnt	nephronectin	9.95858	11.2393	9.220212	9.888214	7.93027	8.878217
17391347	Nphp1	nephronophthisis 1 (juvenile) homolog (human)	15.33291	14.15834	17.28705	16.31944	11.55135	10.26271
17520983	Nphp3	nephronophthisis 3 (adolescent)	9.767437	7.927137	7.614873	9.733104	10.166	10.14101
17422218	Nphp4	nephronophthisis 4 (juvenile) homolog (human)	6.379441	5.598188	4.937771	5.486857	5.726128	4.925568
17489094	Nphs1os	nephrosis 1 homolog, nephrin, opposite strand	4.681043	4.30905	4.255976	5.132103	4.416214	4.482955
17476389	Nphs1	nephrosis 1, nephrin	6.317352	7.714753	7.433337	8.40781	7.9189	7.574602
17218412	Nphs2	nephrosis 2, podocin	5.052474	4.435295	4.012413	4.088892	3.035596	3.813353
17401066	Ngf	nerve growth factor	12.63856	12.91343	11.84979	12.03972	9.064893	10.36667
17268210	Ngfr	nerve growth factor receptor (TNFR superfamily, member 16)	8.720676	12.337	13.75277	14.24783	7.616721	11.67111
17537895	Ngfrap1	nerve growth factor receptor (TNFRSF16) associated protein 1	6.738091	6.791687	6.791687	6.171709	6.690528	5.56106
17229813	Nhlh1	nescient helix loop helix 1	3.586235	3.517182	3.270588	3.511092	3.621456	3.656162
17401036	Nhlh2	nescient helix loop helix 2	4.20927	4.113681	4.20927	4.20927	5.059483	7.692806
17398903	Nes	nestin	4.954261	4.114337	7.400742	4.841755	5.179562	4.381242
17270583	Nsf	N-ethylmaleimide sensitive fusion protein	35.23238	36.28396	40.20464	35.30645	33.59769	37.72764
17474035	Napa	N-ethylmaleimide sensitive fusion protein attachment protein alpha	45.68378	53.56096	50.29524	46.46975	41.96108	50.77477
17392578	Napb	N-ethylmaleimide sensitive fusion protein attachment protein beta	4.088325	5.907591	6.637178	5.666233	7.322367	5.07237

17351204	Napg	N-ethylmaleimide sensitive fusion protein attachment protein gamma	28.01097	25.0502	21.53976	22.27078	18.27488	21.60435
17264405	Ntn1	netrin 1	19.09059	24.29016	22.0163	19.78082	19.11814	19.40115
17341758	Ntn3	netrin 3	8.760966	7.853523	10.38479	10.0806	9.146438	11.8016
17236604	Ntn4	netrin 4	31.74044	22.15929	25.39707	18.98897	16.13219	21.61049
17477960	Ntn5	netrin 5	7.770137	7.935109	8.600258	8.769168	7.509204	6.613849
17409480	Ntng1	netrin G1	2.845969	2.797891	3.421692	2.822806	2.7751	2.847567
17409491	Ntng1	netrin G1	12.32777	11.27535	12.53708	12.20948	12.20948	10.24913
17383467	Ntng2	netrin G2	5.73761	5.032272	5.419651	4.975426	5.358429	4.384979
17479540	Ngrn	neugrin, neurite outgrowth associated	36.85249	30.52163	39.03212	32.11622	27.42577	33.43205
17526776	Ncam1	neural cell adhesion molecule 1	5.158995	6.377938	5.316217	5.53587	5.005832	4.14261
17326756	Ncam2	neural cell adhesion molecule 2	4.450045	3.650135	3.58375	3.795974	4.888121	4.292446
17519184	Nedd4	neural precursor cell expressed, developmentally down-regulated 4	532.8221	557.5317	521.6614	502.6429	478.4618	448.1631
17244244	Nedd1	neural precursor cell expressed, developmentally down-regulated gene 1	26.62076	29.13113	28.77273	20.33045	33.17145	35.71232
17351282	Nedd4l	neural precursor cell expressed, developmentally down-regulated gene 4-like	178.2922	170.231	190.0217	173.9272	182.7161	138.7554
17306785	Nedd8	neural precursor cell expressed, developmentally down-regulated gene 8	127.3955	130.4642	120.0399	120.3899	117.6657	129.3405
17292071	Nedd9	neural precursor cell expressed, developmentally down-regulated gene 9	18.19929	16.76395	17.83835	14.54075	11.90042	19.51281
17367978	Npdc1	neural proliferation, differentiation and control 1	10.99046	13.19537	11.77471	13.31112	13.55704	12.28529
17306680	Nrl	neural retina leucine zipper gene	7.78453	8.98775	8.467112	7.844383	9.456393	10.39357
17360166	Neurl1a	neuralized E3 ubiquitin protein ligase 1A	13.48627	12.12598	12.12716	17.85789	13.61306	15.43764
17334922	Neurl1b	neuralized E3 ubiquitin protein ligase 1B	8.7685	9.018766	7.603323	8.917257	8.767223	10.02799
17394292	Neurl2	neuralized E3 ubiquitin protein ligase 2	6.764719	7.781563	7.183167	7.106405	7.275014	6.189187
17222149	Neurl3	neuralized E3 ubiquitin protein ligase 3	52.99447	61.68125	75.30272	52.87156	48.37903	50.52095
17251767	Neurl4	neuralized E3 ubiquitin protein ligase 4	20.5004	29.97193	38.89412	33.22737	32.49059	33.16317
17336811	Neu1	neuraminidase 1	65.22757	69.9116	66.45519	60.47219	66.45519	62.89747
17215298	Neu2	neuraminidase 2	5.802843	6.034608	6.206758	5.813311	6.347815	6.347815
17493718	Neu3	neuraminidase 3	12.56179	10.41434	14.63631	11.27996	12.08797	14.45143
17508609	Nrg1	neuregulin 1	10.79709	10.67238	17.38969	10.69749	9.859674	6.233014
17353676	Nrg2	neuregulin 2	10.16607	11.94984	11.31324	11.21183	13.40148	13.02381
17305182	Nrg3	neuregulin 3	4.20927	3.842166	4.20927	4.20927	5.485222	4.789716
17298929	Nrg3os	neuregulin 3, opposite strand	3.049072	2.271885	2.597781	2.98014	2.626497	2.98014
17527332	Nrg4	neuregulin 4	21.80323	34.01832	29.35756	18.91882	34.98537	25.85784
17291386	Nrsn1	neurensin 1	4.772483	5.599624	5.517635	5.199958	5.671892	6.003484
17392932	Nrsn2	neurensin 2	7.966688	7.429628	9.027021	9.316383	6.723722	7.654987
17348016	Nrxn1	neurexin I	16.36101	15.8299	17.54283	15.0528	11.87569	18.60824
17356924	Nrxn2	neurexin II	10.67926	9.37189	7.586634	8.51685	7.738425	9.401754
17277712	Nrxn3	neurexin III	5.965363	5.510602	5.838873	6.420386	5.080041	6.414025
17456015	Nxph1	neurexophilin 1	3.315324	3.787485	3.787485	4.152068	4.229069	3.473909
17367637	Nxph2	neurexophilin 2	8.802114	10.45253	10.44326	9.214846	9.736073	10.10537
17268205	Nxph3	neurexophilin 3	16.98289	19.16829	18.78566	17.68446	18.17181	20.81483
17245917	Nxph4	neurexophilin 4	9.73803	9.42544	11.03068	10.02875	11.69873	9.431126
17526663	Nxpe2	neurexophilin and PC-esterase domain family, member 2	14.27709	13.06568	10.00827	15.37453	9.757895	12.64373
17330988	Nxpe3	neurexophilin and PC-esterase domain family, member 3	8.420524	8.24586	9.183028	8.420524	11.56746	7.548605
17516978	Nxpe4	neurexophilin and PC-esterase domain family, member 4	5.308521	4.644876	5.303777	4.016559	5.948764	4.75818
17443803	Nxpe5	neurexophilin and PC-esterase domain family, member 5	3.462322	3.897908	3.156466	3.287635	3.48102	3.287635
17291874	Nrn1	neuritin 1	295.5729	304.2282	311.3169	319.2206	514.0518	511.9539
17504998	Nrn1l	neuritin 1-like	8.554123	12.85698	10.68104	10.46311	10.13223	11.17676
17405285	Nbea	neurobeachin	7.936869	5.866866	6.112193	5.997661	5.997661	5.997661
17213369	Nbeal1	neurobeachin like 1	54.30639	52.63607	45.58414	49.19637	52.09453	49.89454
17531591	Nbeal2	neurobeachin-like 2	14.38666	14.86245	16.6305	15.39041	14.21872	11.17839
17274123	Nbas	neuroblastoma amplified sequence	57.89071	39.70971	38.82328	45.85445	39.40318	35.35837
17401117	Nras	neuroblastoma ras oncogene	35.12339	42.28371	36.6797	33.0482	37.0628	38.93997
17431861	Nbl1	neuroblastoma, suppression of tumorigenicity 1	7.344427	6.974404	7.914839	7.812423	7.552896	9.69533
17316574	Ncald	neurocalcin delta	8.80779	7.698994	7.387519	8.672753	7.935109	8.469871
17509944	Ncan	neurocan	9.320323	6.277891	8.595431	8.509415	6.558746	10.11749
17430140	Ncdn	neurochondrin	20.17798	22.84379	20.45143	18.7573	18.50227	21.36326
17395133	Nespas	neuroendocrine secretory protein antisense	5.67669	4.60902	4.489703	4.474573	5.334267	4.743526
17290242	Net1	neuroepithelial cell transforming gene 1	132.8496	150.0449	153.8361	137.188	111.6756	133.0191
17226916	Nfasc	neurofascin	12.86037	14.37958	14.31296	13.39423	10.84719	11.93255
17253746	Nf1	neurofibromatosis 1	81.53796	84.80339	85.42462	85.13674	87.69544	84.04733
17260036	Nf2	neurofibromatosis 2	30.92068	30.44915	33.72859	38.98318	32.08418	30.13003
17260066	Nefh	neurofilament, heavy polypeptide	9.182646	9.182646	9.244466	10.07564	8.90225	9.414079
17301582	Nefl	neurofilament, light polypeptide	3.059763	3.627285	3.253607	4.20927	2.920408	3.085512
17308046	Nefm	neurofilament, medium polypeptide	3.194987	3.131687	3.647227	3.590637	4.632401	3.509949
17387267	Neurod1	neurogenic differentiation 1	6.558655	6.479758	7.434677	6.368263	6.388734	7.7909
17268786	Neurod2	neurogenic differentiation 2	8.467112	7.228318	9.528176	10.00944	8.508884	8.467112
17246469	Neurod4	neurogenic differentiation 4	3.586981	3.837588	3.80435	3.790426	3.912932	4.419858
17467068	Neurod6	neurogenic differentiation 6	3.277033	3.756789	3.642073	3.337135	4.2044	3.355319
17293002	Neurog1	neurogenin 1	7.17426	7.578645	6.979144	5.572791	6.949986	6.378557
17402513	Neurog2	neurogenin 2	16.12928	14.91146	17.75877	16.72391	19.10635	13.76936
17233771	Neurog3	neurogenin 3	4.521652	4.509414	4.518989	4.790878	6.443256	7.210753
17282789	Ngb	neuroglobin	6.938963	5.764238	6.107407	6.459489	4.633974	7.730544
17525649	Nrgn	neurogranin	4.746881	5.982299	4.613527	5.433644	8.144973	5.570589
17300396	Ngdn	neuroguidin, EIF4E binding protein	65.92529	51.66517	50.65771	52.99631	50.47125	39.84592
17404388	Nlgn1	neuroligin 1	3.284423	3.2227	3.61148	3.154891	3.927224	3.482468
17264984	Nlgn2	neuroligin 2	10.02152	10.87347	7.544002	10.24757	11.04774	10.26186
17536720	Nlgn3	neuroligin 3	10.69334	11.44178	10.06252	12.18353	10.43609	11.49536
17295907	Nln	neurolysin (metallopeptidase M3 family)	58.45684	57.28835	53.72866	53.28066	51.16197	49.91599
17492722	Nmb	neuromedin B	16.67113	11.70782	12.46715	14.35598	13.10813	14.61684
17231774	Nmbr	neuromedin B receptor	5.40266	4.82507	3.697474	5.295585	3.407515	4.20927
17212069	Nms	neuromedin S	5.075395	4.954296	5.184662	4.670121	5.397345	5.584507
17448997	Nmu	neuromedin U	7.258744	7.045783	6.955142	7.230623	7.066031	9.017078
17225175	Nmur1	neuromedin U receptor 1	13.57265	11.60738	12.08596	12.84478	12.55361	12.58209
17263024	Nmur2	neuromedin U receptor 2	3.832963	5.300432	4.289888	3.690254	3.119706	4.789882
17231040	Nenf	neuron derived neurotrophic factor	13.03971	23.24454	18.23072	18.96937	17.98086	17.39993

17227348	Nav1	neuron navigator 1	21.29481	19.34233	22.94169	21.29481	19.03285	16.11308
17227379	Nav1	neuron navigator 1	23.77099	25.43635	24.96284	21.83726	22.86169	20.05542
17478301	Nav2	neuron navigator 2	31.97694	27.8063	33.57231	34.05607	26.79812	26.74097
17244864	Nav3	neuron navigator 3	4.857453	5.093545	4.909717	4.943011	4.553103	3.800581
17447615	Nsg1	neuron specific gene family member 1	10.68933	11.59556	13.80357	10.7607	10.05443	11.9395
17248231	Nsg2	neuron specific gene family member 2	5.923172	5.386134	5.14439	5.386134	4.994049	4.727574
17369368	Ncs1	neuronal calcium sensor 1	5.389607	4.804079	5.708018	4.887037	4.579817	4.804079
17275255	Nrcam	neuronal cell adhesion molecule	2.990507	3.557885	2.713472	3.052455	2.999828	3.852086
17403829	Negr1	neuronal growth regulator 1	3.592156	4.72361	3.549609	4.272854	4.352065	5.269368
17225261	Ngef	neuronal guanine nucleotide exchange factor	33.64266	35.42264	36.65352	33.97614	30.49033	38.72035
17486915	Npas1	neuronal PAS domain protein 1	5.280813	5.010623	4.561505	4.801649	4.766301	5.79087
17212087	Npas2	neuronal PAS domain protein 2	17.47257	14.23322	15.91593	19.55099	12.39546	13.75
17275454	Npas3	neuronal PAS domain protein 3	4.876987	4.578357	4.830425	4.413307	4.901217	5.296392
17361435	Npas4	neuronal PAS domain protein 4	6.82666	6.064716	6.82666	6.82666	6.671633	7.068774
17272926	Nptx1	neuronal pentraxin 1	4.019121	5.161034	5.265389	5.179059	4.960481	5.111264
17444498	Nptx2	neuronal pentraxin 2	12.16108	14.39634	15.56183	13.3355	14.83775	13.40556
17319339	Npcd	neuronal pentraxin chromo domain	9.215868	10.82121	11.73755	8.681113	10.87075	10.32872
17353370	Nrep	neuronal regeneration related protein	28.99877	32.22704	34.1935	38.37939	38.62117	34.21009
17214735	Nyap2	neuronal tyrosine-phophorylated phosphoinositide 3-kinase adaptor 2	2.777371	3.250789	2.909417	2.95976	3.151394	2.82263
17454145	Nyap1	neuronal tyrosine-phosphorylated phosphoinositide 3-kinase adaptor 1	4.897447	6.210933	5.535524	8.323325	5.489139	6.347815
17378741	Nnat	neuronatin	18.19689	22.88547	19.54381	20.42452	17.63733	19.72044
17459207	Ndnf	neuron-derived neurotrophic factor	2.930364	3.164549	3.24316	2.988642	4.233321	3.39808
17280913	Nova1	neuro-oncological ventral antigen 1	4.447054	5.120306	5.120306	5.120306	5.120306	5.120306
17474377	Nova2	neuro-oncological ventral antigen 2	8.890903	9.587469	8.92443	7.90119	8.467112	8.467112
17259430	Npb	neuropeptide B	5.741151	4.771854	11.31638	7.207505	6.172094	5.797175
17233716	Npffr1	neuropeptide FF receptor 1	13.65162	14.17818	13.90855	14.64098	13.90855	12.45454
17438869	Npffr2	neuropeptide FF receptor 2	6.002361	7.307612	6.002361	6.310525	5.26725	6.887594
17322254	Npff	neuropeptide FF-amide peptide precursor	14.23647	13.13624	16.84385	13.38792	12.98217	13.24697
17484221	Nps	neuropeptide S	3.885526	3.786566	3.461698	4.625117	4.091387	4.575534
17515496	Npsr1	neuropeptide S receptor 1	7.333795	6.031973	5.289788	6.32866	6.214249	5.869347
17466743	Npvf	neuropeptide VF precursor	3.725644	3.246466	3.703758	3.76795	3.81449	3.727814
17341975	Npw	neuropeptide W	9.263196	9.055285	7.049544	8.405712	7.799761	7.827274
17458514	Npy	neuropeptide Y	7.804953	9.450627	7.330848	7.36293	9.836125	9.514712
17501544	Npy1r	neuropeptide Y receptor Y1	3.269523	4.055894	3.430574	3.584495	3.81145	4.599127
17406233	Npy2r	neuropeptide Y receptor Y2	3.123276	2.96058	2.353683	2.74634	2.776697	3.34698
17305005	Npy4r	neuropeptide Y receptor Y4	3.019291	3.822377	4.605411	3.396668	3.201926	3.609978
17509706	Npy5r	neuropeptide Y receptor Y5	7.785879	7.538774	8.546774	7.232127	9.321939	9.699565
17350262	Npy6r	neuropeptide Y receptor Y6	2.20699	2.007792	2.448401	2.404972	2.434389	2.734733
17221110	Npbwr1	neuropeptides B/W receptor 1	4.01461	4.57024	4.20927	3.106715	5.726406	6.347815
17352252	Neto1	neuropilin (NRP) and tolloid (TLL)-like 1	4.147293	4.20927	3.536053	3.258053	2.62264	3.258053
17511377	Neto2	neuropilin (NRP) and tolloid (TLL)-like 2	9.980347	7.781294	8.68659	7.781294	7.781294	7.474204
17506991	Nrp1	neuropilin 1	154.7741	144.9217	139.0953	134.6061	142.5861	106.9187
17213548	Nrp2	neuropilin 2	46.8946	46.62785	53.82782	38.88387	28.00592	29.61262
17517914	Nptn	neuroplastin	94.24081	95.26537	93.78856	85.504	93.2473	76.19846
17244601	Nts	neurotensin	4.48625	3.898854	3.549215	4.279523	3.729055	3.549215
17380782	Ntsr1	neurotensin receptor 1	5.113213	5.092062	5.48933	5.947692	5.596171	5.969293
17274195	Ntsr2	neurotensin receptor 2	10.41196	12.33948	13.0464	11.28043	12.03655	12.99036
17525198	Ntm	neurotrimin	9.882253	9.021454	11.21564	11.75759	9.290524	10.16424
17548736	Ntm	neurotrimin	4.859676	5.119809	4.957697	5.547047	4.957422	5.168695
17406586	Ntrk1	neurotrophic tyrosine kinase, receptor, type 1	6.525507	7.153206	6.240277	7.981224	7.960009	9.969354
17287901	Ntrk2	neurotrophic tyrosine kinase, receptor, type 2	5.47231	4.437878	4.597693	5.581914	10.32872	8.795954
17492244	Ntrk3	neurotrophic tyrosine kinase, receptor, type 3	4.270604	4.270604	5.252193	4.270604	4.195986	4.608956
17471140	Ntf3	neurotrophin 3	13.85803	14.21616	20.59069	13.84401	12.41403	13.16249
17477796	Ntf5	neurotrophin 5	8.691891	7.678476	8.460541	9.768655	8.563497	8.875817
17531581	Nradd	neurotrophin receptor associated death domain	6.539544	6.325984	7.901519	7.391361	6.568528	6.866857
17346344	Nrtn	neurturin	9.822907	8.140289	10.50439	10.52392	10.50439	9.913607
17396369	Nceh1	neutral cholesterol ester hydrolase 1	44.58882	52.1162	54.77554	47.72092	33.53347	29.9329
17423066	Nsmaf	neutral sphingomyelinase (N-SMase) activation associated factor	43.90399	52.56425	53.42303	57.4092	54.39756	46.49456
17453288	Ncf1	neutrophil cytosolic factor 1	22.1401	16.85156	20.24803	16.16846	9.392505	18.24741
17218261	Ncf2	neutrophil cytosolic factor 2	18.60051	21.16561	21.95945	19.49537	23.22586	15.5912
17312700	Ncf4	neutrophil cytosolic factor 4	8.210443	13.82589	12.56728	9.941613	13.49104	10.2136
17522369	Ngp	neutrophilic granule protein	6.883632	7.647032	9.633032	6.733516	10.45195	6.106518
17411201	Nexn	nexilin	5.115198	5.445219	6.032015	5.002711	5.148635	4.176516
17319738	Nfam1	Nfat activating molecule with ITAM motif 1	12.94856	14.13147	13.37911	14.31038	13.7223	13.55394
17540977	Nkrf	NF-kappaB repressing factor	13.44325	15.83308	10.47988	14.76132	18.22618	14.9923
17534142	Nkap	NFKB activating protein	15.0157	10.81641	14.7817	14.37241	13.72477	13.06115
17290957	Nkapl	NFKB activating protein-like	5.858535	5.736656	5.617429	4.998239	6.328923	5.337263
17297374	Nkiras1	NFKB inhibitor interacting Ras-like protein 1	39.16546	32.18552	30.15907	31.74418	30.25245	34.36124
17256414	Nkiras2	NFKB inhibitor interacting Ras-like protein 2	16.25464	16.79349	17.77959	16.57641	17.36523	17.36523
17460494	Nfu1	NFU1 iron-sulfur cluster scaffold homolog (S. cerevisiae)	68.65534	71.25898	68.78967	67.25455	67.35952	69.03567
17283088	1700019M22Rik	NFX-like protein	2.629412	2.300376	3.014064	2.511797	3.670356	2.246891
17222913	Nab1	Ngfi-A binding protein 1	55.84747	53.56096	51.6734	44.25811	49.1524	49.96098
17238088	Nab2	Ngfi-A binding protein 2	15.91064	18.14638	17.58045	16.51712	15.50939	17.74696
17246019	Nab2	Ngfi-A binding protein 2	76.01594	83.63066	77.84918	86.35812	97.9939	100.9075
17213153	Nif3l1	Ngg1 interacting factor 3-like 1 (S. pombe)	29.2714	23.42488	28.1318	26.72819	27.29472	29.13154
17297305	Ngly1	N-glycanase 1	102.4638	91.46957	78.47167	94.49067	96.6401	92.47234
17292258	Nhlrc1	NHL repeat containing 1	10.47145	10.14629	11.17141	8.33909	9.865143	10.69096
17360585	Nhlrc2	NHL repeat containing 2	37.33275	46.18364	57.22446	41.37221	46.82769	61.11904
17405197	Nhlrc3	NHL repeat containing 3	31.5142	40.38759	37.22036	34.54201	30.72008	34.06835
17342529	Nhlrc4	NHL repeat containing 4	4.267704	5.219422	5.686536	4.729377	3.870017	3.332165
17319576	Nhp2l1	NHP2 non-histone chromosome protein 2-like 1 (S. cerevisiae)	10.34556	9.97039	9.488171	8.522106	8.686831	9.630896
17249347	Nhp2	NHP2 ribonucleoprotein	36.69471	39.69458	30.71646	40.62089	37.76558	39.69458
17516993	LOC100862468	NHP2-like protein 1	76.754	89.11337	87.01728	79.7283	85.70753	89.65673
17231826	Nhsl1	NHS-like 1	18.32016	17.45998	19.724	19.41702	15.71837	12.92847

17536861	Nhsl2	NHS-like 2	5.652033	5.801315	5.42059	5.652033	6.030928	4.862169
17411981	Nbn	nibrin	38.89762	32.72815	30.94126	35.53799	30.86479	35.82339
17243361	Ncln	nicalin homolog (zebrafish)	63.71981	58.1356	59.57974	56.53138	68.86457	58.12029
17229818	Ncstn	nicastrin	94.90749	102.7974	93.76256	83.38465	78.30921	82.1609
17521652	Nicn1	nicolin 1	62.62471	65.43905	74.76598	63.17842	70.06247	62.15808
17526695	Nnmt	nicotinamide N-methyltransferase	47.34335	52.09056	59.90108	51.25888	53.22345	61.96106
17433151	Nmnat1	nicotinamide nucleotide adenylyltransferase 1	29.87155	31.99623	32.89189	30.04785	20.80113	28.76615
17218277	Nmnat2	nicotinamide nucleotide adenylyltransferase 2	9.808755	10.68481	12.28486	10.41665	10.68481	10.6587
17520611	Nmnat3	nicotinamide nucleotide adenylyltransferase 3	11.26386	8.9971	11.52312	12.62446	12.38215	13.67586
17296489	Nnt	nicotinamide nucleotide transhydrogenase	61.28788	64.57617	70.43839	56.6683	52.26959	59.68349
17274875	Nampt	nicotinamide phosphoribosyltransferase	73.07672	82.417	99.69436	85.10193	99.69436	87.06072
17358020	Nmrk1	nicotinamide riboside kinase 1	13.93286	25.04811	33.37394	20.08324	25.7959	20.87378
17243219	Nmrk2	nicotinamide riboside kinase 2	5.397607	5.8377	4.256359	5.433681	5.347631	4.930999
17318194	Naprt	nicotinate phosphoribosyltransferase	111.3563	122.745	112.0633	101.2277	107.6609	103.1357
17285244	Nid1	nidogen 1	26.88445	29.12548	36.45387	20.31791	22.40098	22.24887
17297391	Nid2	nidogen 2	14.83024	15.46366	13.02733	12.50679	12.50679	13.41523
17285225	LOC105245453	nidogen-1-like	61.38184	68.94815	68.56213	46.61848	37.40632	39.88405
17352731	Npc1	Niemann-Pick type C1	261.0405	236.832	252.6121	219.3529	215.1889	228.2862
17282563	Npc2	Niemann-Pick type C2	99.81606	112.9915	114.1645	106.4764	131.3425	99.78285
17538018	Nrk	Nik related kinase	3.25496	3.301565	3.25496	3.38616	3.329187	3.460403
17296532	Nim1k	NIM1 serine/threonine protein kinase	11.50493	10.35223	11.48461	10.94606	9.083322	9.543973
17297258	Nek10	NIMA (never in mitosis gene a)- related kinase 10	4.349893	3.343409	3.230842	3.441315	3.441315	3.361232
17501367	Nek1	NIMA (never in mitosis gene a)-related expressed kinase 1	31.38689	26.40209	23.11424	30.91168	22.91055	22.64201
17530436	Nek11	NIMA (never in mitosis gene a)-related expressed kinase 11	3.623214	4.486934	4.486934	4.748757	4.486934	5.239104
17220844	Nek2	NIMA (never in mitosis gene a)-related expressed kinase 2	6.223905	6.600887	6.385635	6.786793	7.126432	7.928566
17508006	Nek3	NIMA (never in mitosis gene a)-related expressed kinase 3	10.70231	10.88757	10.36616	13.80377	8.690083	11.34701
17298292	Nek4	NIMA (never in mitosis gene a)-related expressed kinase 4	32.81992	34.01903	32.57177	34.22408	30.73115	30.50069
17507982	Nek5	NIMA (never in mitosis gene a)-related expressed kinase 5	5.061868	6.247245	6.4223	5.440767	6.449065	6.899539
17370455	Nek6	NIMA (never in mitosis gene a)-related expressed kinase 6	92.0726	58.50021	60.50154	53.79425	76.24601	46.5978
17227570	Nek7	NIMA (never in mitosis gene a)-related expressed kinase 7	154.5854	167.0782	137.0027	144.3623	147.7977	147.5786
17266344	Nek8	NIMA (never in mitosis gene a)-related expressed kinase 8	9.534254	9.041925	9.422109	8.494859	10.22409	10.01284
17282698	Nek9	NIMA (never in mitosis gene a)-related expressed kinase 9	67.24383	62.85606	56.97748	48.87552	54.97829	59.09483
17512740	Nob1	NIN1/RPN12 binding protein 1 homolog (S. cerevisiae)	32.85202	33.42973	36.74281	29.08675	36.1662	28.75359
17281686	Nin	ninein	8.353611	11.31661	13.43375	10.30648	11.20569	15.30414
17392748	Ninl	ninein-like	7.70695	8.248547	9.623956	8.215111	7.935109	8.100663
17287107	Ninj1	ninjurin 1	55.087	61.13557	58.16099	63.20383	66.80712	53.86439
17462310	Ninj2	ninjurin 2	7.994013	8.289816	8.289816	8.081819	8.289816	8.134874
17438062	Nipal1	NIPA-like domain containing 1	27.09454	75.51598	78.06695	58.39731	129.2966	83.29454
17316402	Nipal2	NIPA-like domain containing 2	3.324644	3.583005	3.285129	3.304312	3.780979	3.941596
17431454	Nipal3	NIPA-like domain containing 3	23.59697	22.11679	29.79978	25.83473	25.08547	25.83473
17262055	Nipal4	NIPA-like domain containing 4	12.61423	9.041675	10.43884	9.871505	10.20879	11.87668
17315810	Nipbl	Nipped-B homolog (Drosophila)	105.6281	109.9436	96.89431	104.4429	105.6281	112.1682
17414161	Nipsnap3a	nipsnap homolog 3A (C. elegans)	3.440099	3.263862	2.857176	3.959313	2.890342	3.079402
17414173	Nipsnap3b	nipsnap homolog 3B (C. elegans)	91.14679	66.04603	71.46079	76.08657	80.69179	68.90611
17304596	Nisch	nischarin	89.36141	96.12768	111.6021	95.02005	89.67911	81.10731
17449073	Noa1	nitric oxide associated 1	30.89454	31.10641	30.89454	29.4703	29.27181	26.8273
17229537	Nos1ap	nitric oxide synthase 1 (neuronal) adaptor protein	22.16713	21.64347	27.48533	23.79456	24.87785	20.94204
17441453	Nos1	nitric oxide synthase 1, neuronal	5.764079	6.831394	6.408337	6.639433	6.346979	6.862004
17253707	Nos2	nitric oxide synthase 2, inducible	4.951858	5.726525	8.349501	6.171709	6.171709	7.612657
17435282	Nos3	nitric oxide synthase 3, endothelial cell	17.62706	19.487	20.67478	15.07295	14.63043	14.56668
17477679	Nosip	nitric oxide synthase interacting protein	32.93432	29.54107	23.29761	27.37202	28.34024	32.44625
17371390	Nostrin	nitric oxide synthase trafficker	46.00568	38.52946	46.65598	49.16944	43.46493	40.31207
17229729	Nit1	nitrilase 1	207.3666	202.4217	205.6632	228.416	234.1485	222.7328
17331102	Nit2	nitrilase family, member 2	190.766	150.499	135.6034	187.5368	198.3342	211.5024
17393483	Nfs1	nitrogen fixation gene 1 (S. cerevisiae)	75.81493	70.10571	73.07723	74.88579	61.97309	62.82383
17521376	Nprl2	nitrogen permease regulator-like 2	43.37705	35.06306	43.37705	46.47652	44.89242	43.37705
17261494	Nprl3	nitrogen permease regulator-like 3	27.28923	21.22629	23.89456	25.21762	22.14072	24.02975
17447110	Nkx1-1	NK1 transcription factor related, locus 1 (Drosophila)	17.90909	19.66298	16.52055	19.80908	17.07235	17.61741
17497104	Nkx1-2	NK1 transcription factor related, locus 2 (Drosophila)	6.05002	6.844428	6.36663	7.467265	8.396569	6.826944
17281299	Nkx2-1	NK2 homeobox 1	4.603113	5.246553	5.383222	3.700269	4.949696	5.945673
17392529	Nkx2-2	NK2 homeobox 2	5.531305	6.7788	6.201555	6.049475	5.742902	7.605484
17377317	Nkx2-2os	NK2 homeobox 2, opposite strand	5.54847	6.800968	6.387184	6.429382	6.315382	6.562293
17359599	Nkx2-3	NK2 homeobox 3	4.377782	4.377782	4.058988	5.331014	4.148529	4.20962
17392525	Nkx2-4	NK2 homeobox 4	10.24736	10.58068	11.49034	11.67678	9.554233	8.881849
17342656	Nkx2-5	NK2 homeobox 5	31.36536	30.09496	27.40923	27.89452	31.91361	28.96964
17301605	Nkx2-6	NK2 homeobox 6	8.698641	9.607493	9.914981	10.14481	9.648469	10.23025
17281306	Nkx2-9	NK2 homeobox 9	8.249691	10.3966	9.558803	8.706965	9.831489	10.18751
17447749	Nkx3-2	NK3 homeobox 2	7.437718	8.599245	8.721462	8.127885	9.000655	9.258253
17301610	Nkx3-1	NK-3 transcription factor, locus 1 (Drosophila)	7.172818	9.79829	8.71264	7.484106	7.262015	7.262015
17450199	Nkx6-1	NK6 homeobox 1	6.935068	6.587815	6.345835	6.476929	6.051446	6.368043
17497449	Nkx6-2	NK6 homeobox 2	9.827742	11.15903	11.14033	11.48875	11.07942	11.28364
17500062	Nkx6-3	NK6 homeobox 3	13.47796	12.97219	10.92279	13.3126	14.81052	17.50033
17526160	Nlrx1	NLR family member X1	22.41061	25.50114	19.49808	19.25966	19.20553	16.21391
17295627	Naip1	NLR family, apoptosis inhibitory protein 1	2.67402	3.54506	3.54506	4.909615	3.263862	2.74712
17295569	Naip2	NLR family, apoptosis inhibitory protein 2	6.747579	6.801722	6.535013	3.397892	3.797421	5.12716
17295588	Naip5	NLR family, apoptosis inhibitory protein 5	4.818019	4.909071	10.54049	4.511947	7.993349	6.90351
17295607	Naip6	NLR family, apoptosis inhibitory protein 6	3.968104	5.386582	3.7072	4.656707	10.02945	3.318658
17327613	Nlrc3	NLR family, CARD domain containing 3	4.681043	5.919724	4.327519	5.190703	4.681043	4.807944
17347236	Nlrc4	NLR family, CARD domain containing 4	4.863787	5.32278	4.763526	6.499235	5.489939	5.135127
17504023	Nlrc5	NLR family, CARD domain containing 5	19.08967	22.96126	24.37432	22.7422	22.96126	22.65127
17494822	Nlrp10	NLR family, pyrin domain containing 10	4.218207	4.327129	4.022661	4.183122	4.009632	3.725128
17485497	Nlrp12	NLR family, pyrin domain containing 12	58.30873	77.99069	78.85334	97.29879	96.167	73.89936
17481514	Nlrp14	NLR family, pyrin domain containing 14	3.172676	2.683731	3.069024	2.935365	3.381938	3.035975
17265473	Nlrp1a	NLR family, pyrin domain containing 1A	4.520963	3.65994	2.933677	2.929735	3.672443	3.273332

17265496	Nlrp1b	NLR family, pyrin domain containing 1B	3.538186	3.263862	4.056878	2.879823	4.154688	3.074965
17265526	Nlrp1c-ps	NLR family, pyrin domain containing 1C, pseudogene	4.220372	7.034218	5.09245	3.717235	4.876513	4.674882
17486025	Nlrp2	NLR family, pyrin domain containing 2	3.457694	3.952019	3.82959	3.972535	3.438883	3.04044
17250249	Nlrp3	NLR family, pyrin domain containing 3	4.102405	4.107954	5.367908	3.725515	3.706661	5.422432
17475410	Nlrp4a	NLR family, pyrin domain containing 4A	2.936851	3.695271	2.668983	2.70236	2.546208	3.043455
17473671	Nlrp4b	NLR family, pyrin domain containing 4B	3.147835	1.830389	2.093708	2.481702	2.564167	2.294675
17473525	Nlrp4c	NLR family, pyrin domain containing 4C	2.422741	3.024465	2.09349	2.939397	2.899572	2.641853
17486436	Nlrp4d	NLR family, pyrin domain containing 4D	3.434571	4.320805	4.141261	3.061864	2.931238	3.296671
17474809	Nlrp4e	NLR family, pyrin domain containing 4E	3.481937	2.306012	3.131014	2.754671	3.250924	1.97386
17474732	Gm4541	NLR family, pyrin domain containing 4E pseudogene	4.03109	3.7878	3.374351	3.374351	3.374351	3.581292
17474752	Gm4541	NLR family, pyrin domain containing 4E pseudogene	3.033935	3.582359	3.246533	3.258776	3.122713	3.670992
17474767	Gm4541	NLR family, pyrin domain containing 4E pseudogene	3.033935	3.582359	3.246533	3.258776	3.122713	3.670992
17474791	Gm4541	NLR family, pyrin domain containing 4E pseudogene	4.03109	3.7878	3.374351	3.374351	3.374351	3.581292
17474803	Gm4541	NLR family, pyrin domain containing 4E pseudogene	4.03109	3.7878	3.374351	3.374351	3.374351	3.581292
17293774	Nlrp4f	NLR family, pyrin domain containing 4F	2.495364	2.50243	3.128953	2.448248	2.546031	2.02194
17474820	Nlrp5	NLR family, pyrin domain containing 5	4.717328	4.563846	4.512916	5.785894	4.49347	7.350182
17484670	Nlrp6	NLR family, pyrin domain containing 6	64.49476	71.29851	71.60657	84.12834	105.2948	106.2214
17475431	Nlrp9a	NLR family, pyrin domain containing 9A	3.85433	4.871952	4.19151	4.031481	4.549128	4.086172
17474708	Nlrp9b	NLR family, pyrin domain containing 9B	3.153295	2.641743	2.496055	2.606493	2.878625	2.971003
17488078	Nlrp9c	NLR family, pyrin domain containing 9C	2.406006	2.451798	2.952854	2.153525	2.344223	2.47902
17398287	Nmd3	NMD3 homolog (S. cerevisiae)	53.46193	59.43826	41.85767	52.41108	62.48769	57.38533
17367807	Nsmf	NMDA receptor synaptonuclear signaling and neuronal migration factor	34.89576	32.68394	41.75451	35.77277	32.92268	30.89454
17353444	Nme5	NME/NM23 family member 5	5.540899	5.210566	5.653013	5.810571	5.040965	5.036285
17218907	Nme7	NME/NM23 family member 7	20.3619	20.92796	18.49649	18.49649	16.52399	17.17407
17290871	Nme8	NME/NM23 family member 8	4.307289	3.424112	3.892238	3.706661	3.942771	3.567467
17520706	Nme9	NME/NM23 family member 9	3.465347	3.72722	3.580795	3.59243	4.098682	3.616114
17267804	Nme1	NME/NM23 nucleoside diphosphate kinase 1	93.82757	94.70968	85.3749	96.24502	76.75666	83.67241
17267794	Nme2	NME/NM23 nucleoside diphosphate kinase 2	35.18796	25.99802	35.63369	31.53714	32.04084	34.92722
17334495	Nme3	NME/NM23 nucleoside diphosphate kinase 3	38.57542	35.32481	37.87883	35.32481	39.89317	35.32481
17342595	Nme4	NME/NM23 nucleoside diphosphate kinase 4	16.47367	15.79931	16.04053	17.23047	16.36067	13.78522
17522219	Nme6	NME/NM23 nucleoside diphosphate kinase 6	23.98665	12.86384	13.70224	13.20452	17.07235	14.14608
17248249	Mpg	N-methylpurine-DNA glycosylase	11.8319	12.29406	12.337	12.31248	12.61939	12.41722
17327802	Nmral1	NmrA-like family domain containing 1	11.8808	12.63146	14.36594	16.29605	11.79546	14.10446
17385085	Nmi	N-myc (and STAT) interactor	58.51238	53.49195	59.29054	70.0819	67.11903	65.12773
17317652	Ndrg1	N-myc downstream regulated gene 1	53.22049	41.6721	37.67642	39.93412	32.78059	43.74451
17306147	Ndrg2	N-myc downstream regulated gene 2	838.3448	918.1143	968.0448	1016.159	982.4295	947.5183
17393592	Ndrg3	N-myc downstream regulated gene 3	52.1668	47.57	52.89392	41.93317	38.71901	43.83769
17504327	Ndrg4	N-myc downstream regulated gene 4	10.89504	12.87375	11.26714	10.74751	10.23061	11.06852
17257051	Nmt1	N-myristoyltransferase 1	154.9093	153.6187	163.6977	151.632	138.3626	152.4973
17366373	Nmt2	N-myristoyltransferase 2	46.86146	64.1817	56.24177	54.28795	46.75731	47.79938
17466476	Nobox	NOBOX oogenesis homeobox	3.515622	3.850058	4.687732	3.659179	3.265265	3.753353
17397426	Noct	nocturnin	16.67887	31.03448	24.50238	13.08498	18.65551	19.48281
17233702	Nodal	nodal	7.6491	5.698452	5.698452	4.428813	4.8588	5.698452
17478082	Nomo1	nodal modulator 1	68.93668	57.79128	52.6452	50.86566	52.23627	52.49507
17267656	Nog	noggin	6.513532	6.941368	6.688858	7.24457	6.941368	7.702677
17288387	Nsun2	NOL1/NOP2/Sun domain family member 2	89.34468	91.94696	87.29396	94.77929	95.19738	83.51017
17331350	Nsun3	NOL1/NOP2/Sun domain family member 3	56.15996	50.9281	58.01564	52.8298	34.48316	52.56716
17381911	Nsun6	NOL1/NOP2/Sun domain family member 6	9.830195	8.946169	7.768467	7.881663	7.64629	11.05901
17428529	Nsun4	NOL1/NOP2/Sun domain family, member 4	6.028128	6.189616	6.166706	5.299991	6.026666	5.381581
17443261	Nsun5	NOL1/NOP2/Sun domain family, member 5	17.09864	17.09864	23.05967	18.48906	17.09864	16.13722
17437857	Nsun7	NOL1/NOP2/Sun domain family, member 7	2.640351	2.770161	2.378942	2.62932	3.263862	2.606909
17491454	Nipa1	non imprinted in Prader-Willi/Angelman syndrome 1 homolog (human)	12.08144	11.7317	14.52262	11.35551	13.14261	12.59885
17491440	Nipa2	non imprinted in Prader-Willi/Angelman syndrome 2 homolog (human)	24.71046	27.23462	23.40103	24.71046	22.36127	20.59226
17378287	a	nonagouti	8.379418	8.379418	8.379418	8.329558	8.286139	10.04085
17378296	a	nonagouti	2.034085	1.859429	1.943113	1.762911	1.814523	2.141557
17530128	Nck1	non-catalytic region of tyrosine kinase adaptor protein 1	38.3903	31.0924	29.30023	30.05764	31.74484	28.87202
17212355	Nck2	non-catalytic region of tyrosine kinase adaptor protein 2	6.028546	5.264476	5.149884	5.149884	4.404444	5.149884
17498122	Nctc1	non-coding transcript 1	5.742862	4.031705	5.54897	6.12343	4.131226	4.855837
17431429	Ncmap	noncompact myelin associated protein	5.482865	6.055726	6.171709	6.909542	6.219247	7.189393
17224440	Nhej1	nonhomologous end-joining factor 1	10.36121	11.57965	9.889105	10.19649	9.702322	9.090376
17536742	Nono	non-POU-domain-containing, octamer binding protein	120.1306	139.9114	140.3972	133.1151	124.0459	127.1035
17370068	Nron	non-protein coding RNA, repressor of NFAT	4.389145	6.06148	6.549342	5.069172	4.993738	5.725335
17471023	Ncapd2	non-SMC condensin I complex, subunit D2	13.53009	14.21429	17.53238	15.76245	12.76339	11.6459
17437278	Ncapg	non-SMC condensin I complex, subunit G	4.01461	4.24637	4.598585	3.801134	3.904425	4.567992
17391212	Ncaph	non-SMC condensin I complex, subunit H	3.75494	3.591235	3.745167	3.779643	3.667126	3.706172
17515577	Ncapd3	non-SMC condensin II complex, subunit D3	23.00858	26.32734	23.02549	25.5606	28.82543	26.76823
17279608	Ncapg2	non-SMC condensin II complex, subunit G2	4.221308	4.684126	5.629044	3.403101	3.990916	4.604486
17314134	Ncaph2	non-SMC condensin II complex, subunit H2	44.69526	50.00776	47.70372	43.47156	44.69526	40.80149
17340495	Gm8363	non-SMC condensin II complex, subunit H2 pseudogene	5.365007	6.724281	4.09712	7.523636	7.920656	5.690439
17496049	Nsmce1	non-SMC element 1 homolog (S. cerevisiae)	38.60618	40.16119	40.16119	36.35546	40.16119	33.79435
17311821	Nsmce2	non-SMC element 2 homolog (MMS21, S. cerevisiae)	38.41932	37.82583	31.25511	40.05055	52.35553	37.05176
17496989	Nsmce4a	non-SMC element 4 homolog A (S. cerevisiae)	51.75692	43.03266	45.64738	64.11544	57.05732	62.91927
17488492	Nccrp1	non-specific cytotoxic cell receptor protein 1 homolog (zebrafish)	13.58074	12.29321	11.92147	11.18722	12.79333	12.46162
17374238	Nop10	NOP10 ribonucleoprotein	261.9413	265.5777	240.1913	240.0831	245.839	294.3543
17447289	Nop14	NOP14 nucleolar protein	37.6018	33.66856	30.97602	33.76063	38.90032	34.22313
17292712	Nop16	NOP16 nucleolar protein	41.62793	30.39854	40.32353	41.0944	44.42285	34.95653
17463108	Nop2	NOP2 nucleolar protein	52.28735	47.90818	46.19709	48.78548	42.29045	48.54329
17376252	Nop56	NOP56 ribonucleoprotein	37.64823	38.31746	35.35225	33.29445	36.82363	32.1853
17376272	Nop56	NOP56 ribonucleoprotein	27.73873	25.96111	21.20996	23.68103	43.65515	37.79779
17376274	Nop56	NOP56 ribonucleoprotein	14.59933	12.87857	13.13523	14.12481	17.652	18.75418
17213295	Nop58	NOP58 ribonucleoprotein	56.57371	57.39368	52.33993	47.09461	51.69382	48.06684
17213315	Nop58	NOP58 ribonucleoprotein	18.55171	19.22404	17.85963	19.70732	23.22232	22.12522
17300650	Nop9	NOP9 nucleolar protein	27.45981	25.72929	24.69128	29.0046	34.6975	27.58783
17540396	Ndp	Norrie disease (pseudoglioma) (human)	4.215168	3.668804	4.925636	4.801649	4.307498	3.958133

17383052	Notch1	notch 1	33.06952	37.38636	41.06849	38.22779	40.17678	39.39471
17400813	Notch2	notch 2	80.78301	82.8259	94.72544	74.95174	85.33999	70.87181
17343299	Notch3	notch 3	10.65619	10.93067	10.93067	9.343869	15.49596	10.93067
17336559	Notch4	notch 4	16.66466	16.56608	18.53667	16.06635	14.75212	16.56608
17266830	Nle1	notchless homolog 1 (Drosophila)	14.19617	13.13505	15.13126	17.06691	11.40686	8.561657
17367852	Nrarp	Notch-regulated ankyrin repeat protein	9.833534	10.68104	9.018828	8.239347	9.020891	10.16543
17460267	Noto	notochord homolog (Xenopus laevis)	6.290959	4.647871	3.559661	4.645455	4.330609	4.511915
17273260	Notum	notum pectinacetylesterase homolog (Drosophila)	32.57673	33.59187	32.39906	40.1036	30.33455	38.44063
17259443	Notumos	notum pectinacetylesterase homolog (Drosophila), opposite strand [Source:MGI Sy	5.430293	4.706191	4.767854	5.737769	5.510755	7.478091
17533183	B630019K06Rik	novel protein similar to F-box and leucine-rich repeat protein 17 (Fbxl17)	8.327191	6.171709	6.178172	6.171709	5.161463	6.687097
17260308	Npc1l1	NPC1-like 1	6.171709	8.818052	6.171709	6.171709	4.954571	6.171709
17283225	Nrde2	nrde-2 necessary for RNA interference, domain containing	18.15695	15.97717	15.75371	15.48705	15.3241	15.48705
17295270	Nsa2	NSA2 ribosome biogenesis homolog (S. cerevisiae)	236.1386	187.542	182.8428	192.0995	198.4635	186.124
17220591	5033404E19Rik	NSA2 ribosome biogenesis homolog pseudogene	4.804346	5.65807	6.949793	6.076708	4.889815	6.108381
17377583	Nsfl1c	NSFL1 (p97) cofactor (p47)	128.2336	129.4795	116.0031	105.9867	106.9884	111.236
17220776	Nsl1	NSL1, MIND kinetochore complex component, homolog (S. cerevisiae)	3.43731	3.648567	2.958734	3.522061	3.788088	3.455873
17272913	Sgsh	N-sulfoglucosamine sulfohydrolase (sulfamidase)	22.33664	20.21494	22.8666	18.02628	18.71029	20.74296
17323082	Ntan1	N-terminal Asn amidase	28.39589	28.66344	35.48334	32.3331	35.01618	35.22505
17423444	Necab1	N-terminal EF-hand calcium binding protein 1	190.6985	150.0085	134.093	137.6442	136.2942	139.6944
17506095	Necab2	N-terminal EF-hand calcium binding protein 2	5.05801	4.552408	4.910228	5.054926	5.446729	4.20927
17393143	Necab3	N-terminal EF-hand calcium binding protein 3	4.736266	4.801649	5.553164	5.339372	4.801649	4.4251
17369271	Ntmt1	N-terminal Xaa-Pro-Lys N-methyltransferase 1	25.36259	15.49531	18.24539	19.35695	16.20368	17.95931
17377354	Nxt1	NTF2-related export protein 1	11.79832	13.5845	13.5845	13.5845	12.52189	14.00966
17334356	Nthl1	nth (endonuclease III)-like 1 (E.coli)	15.02872	15.64381	13.22513	15.02528	15.74694	15.43764
17474657	Nkpd1	NTPase, KAP family P-loop domain containing 1	6.42043	7.800897	6.441247	6.25474	6.092988	6.798752
17243604	Nuak1	NUAK family, SNF1-like kinase, 1	11.50086	12.9819	10.48068	11.50086	11.54791	12.2017
17217182	Nuak2	NUAK family, SNF1-like kinase, 2	11.17691	16.8302	21.05951	19.09192	23.16669	23.91013
17214948	Sp100	nuclear antigen Sp100	25.70897	22.61425	37.29137	28.5083	20.82161	29.2424
17214979	Sp100	nuclear antigen Sp100	3.703322	2.420844	3.373719	4.280561	3.946572	4.36197
17369832	Naif1	nuclear apoptosis inducing factor 1	15.65531	19.4282	15.75808	17.11228	17.01199	15.19095
17501550	Naf1	nuclear assembly factor 1 homolog (S. cerevisiae)	15.35897	15.95227	15.51165	17.14338	13.57857	13.03808
17275234	Gm2027	nuclear assembly factor 1 homolog pseudogene	2.62535	2.71897	2.358795	2.369216	3.453334	3.117069
17428659	Nasp	nuclear autoantigenic sperm protein (histone-binding)	9.599438	12.13271	11.75042	15.13168	13.10023	13.05943
17366185	LOC100041708	nuclear body protein SP140-like	5.331605	4.943867	4.33163	6.786793	3.875789	4.850051
17366201	LOC101055672	nuclear body protein SP140-like	13.48151	19.41366	13.40045	13.79449	18.32321	14.94333
17413789	Ncbp1	nuclear cap binding protein subunit 1	48.02022	41.92678	41.77882	44.27622	34.72638	36.25002
17324722	Ncbp2	nuclear cap binding protein subunit 2	28.80865	32.57854	31.0018	32.64907	33.78826	32.8131
17252557	Ncbp3	nuclear cap binding subunit 3	24.83822	25.69989	29.65637	25.10008	23.97984	22.44683
17217127	Nucks1	nuclear casein kinase and cyclin-dependent kinase substrate 1	96.71984	115.8456	111.5139	106.2773	133.2083	120.5828
17431036	Nudc	nuclear distribution gene C homolog (Aspergillus)	66.84464	62.31467	50.2928	67.01351	64.75053	59.23221
17323120	Nde1	nuclear distribution gene E homolog 1 (A nidulans)	18.28654	15.36036	21.18175	24.61627	17.98796	20.12044
17264424	Ndel1	nuclear distribution gene E-like homolog 1 (A. nidulans)	46.21759	63.89982	55.47093	49.29502	49.30596	59.17066
17355364	n-R5-8s1	nuclear encoded rRNA 5.8S 1 [Source:MGI Symbol;Acc:MGI:4421735]	1130.378	985.6147	997.1384	1223.408	999.9	1154.802
17546790	n-R5s1	nuclear encoded rRNA 5S 1 [Source:MGI Symbol;Acc:MGI:4421733]	257.8664	266.5403	282.4595	306.3145	319.1498	379.6852
17543085	n-R5s10	nuclear encoded rRNA 5S 10 [Source:MGI Symbol;Acc:MGI:4421744]	14.57021	19.03972	11.57131	11.78794	12.4007	18.66457
17537901	n-R5s12	nuclear encoded rRNA 5S 12 [Source:MGI Symbol;Acc:MGI:4421746]	35.43984	21.27858	14.00131	21.1615	21.1615	23.9292
17513974	n-R5s127	nuclear encoded rRNA 5S 127 [Source:MGI Symbol;Acc:MGI:4421983]	83.02593	76.21061	66.19361	111.8863	89.36141	92.58522
17538742	n-R5s13	nuclear encoded rRNA 5S 13 [Source:MGI Symbol;Acc:MGI:4421747]	3.308575	3.809786	2.483767	2.816083	1.827574	3.214268
17513976	n-R5s136	nuclear encoded rRNA 5S 136	2382.037	2127.126	1833.202	2632.997	2380.551	2416.495
17538850	n-R5s14	nuclear encoded rRNA 5S 14 [Source:MGI Symbol;Acc:MGI:4421748]	5.842031	5.502549	4.306393	7.609017	8.925358	4.992561
17538945	n-R5s15	nuclear encoded rRNA 5S 15 [Source:MGI Symbol;Acc:MGI:4421749]	2.788076	2.553047	2.552091	1.904761	2.373408	2.66056
17473633	n-R5s150	nuclear encoded rRNA 5S 150 [Source:MGI Symbol;Acc:MGI:4422012]	2.162035	1.898013	2.206837	2.220785	1.661401	2.235566
17474128	n-R5s151	nuclear encoded rRNA 5S 151 [Source:MGI Symbol;Acc:MGI:4422013]	955.2801	805.8793	743.5675	955.8961	946.7371	899.9306
17487507	n-R5s152	nuclear encoded rRNA 5S 152 [Source:MGI Symbol;Acc:MGI:4422015]	6.502435	3.927088	4.30702	3.123298	4.292892	3.51175
17488848	n-R5s153	nuclear encoded rRNA 5S 153 [Source:MGI Symbol;Acc:MGI:4422016]	2.453243	2.100845	1.992445	2.295725	2.307195	2.723119
17492890	n-R5s154	nuclear encoded rRNA 5S 154 [Source:MGI Symbol;Acc:MGI:4422017]	2.401507	2.120332	2.700039	3.019584	2.21022	2.953407
17480100	n-R5s155	nuclear encoded rRNA 5S 155 [Source:MGI Symbol;Acc:MGI:4422018]	5.336375	4.377873	5.617429	3.494098	8.626289	7.391976
17480566	n-R5s156	nuclear encoded rRNA 5S 156 [Source:MGI Symbol;Acc:MGI:4422019]	397.6347	269.9166	284.5543	353.7828	350.2934	354.3693
17495452	n-R5s157	nuclear encoded rRNA 5S 157 [Source:MGI Symbol;Acc:MGI:4422020]	2.318578	2.269084	2.034917	2.243312	2.650326	2.594201
17496945	n-R5s158	nuclear encoded rRNA 5S 158 [Source:MGI Symbol;Acc:MGI:4422021]	4.987511	4.486841	4.862641	4.482644	5.170737	4.578706
17484056	n-R5s159	nuclear encoded rRNA 5S 159 [Source:MGI Symbol;Acc:MGI:4422022]	2.045509	2.060051	1.759262	2.301748	1.804676	2.606909
17539084	n-R5s16	nuclear encoded rRNA 5S 16 [Source:MGI Symbol;Acc:MGI:4421750]	31.55376	35.14214	35.30621	49.09588	28.99596	45.78141
17456051	n-R5s160	nuclear encoded rRNA 5S 160 [Source:MGI Symbol;Acc:MGI:4422024]	6.630281	14.82757	10.91235	12.15451	16.4119	13.34724
17456839	n-R5s161	nuclear encoded rRNA 5S 161 [Source:MGI Symbol;Acc:MGI:4422025]	8.960194	5.316733	6.82666	7.56993	6.336605	8.465293
17459095	n-R5s162	nuclear encoded rRNA 5S 162 [Source:MGI Symbol;Acc:MGI:4422026]	354.362	354.362	267.4935	406.8068	388.8312	371.8843
17460536	n-R5s164	nuclear encoded rRNA 5S 164 [Source:MGI Symbol;Acc:MGI:4422028]	2.593589	2.776062	2.840254	2.682708	2.625842	3.303534
17469605	n-R5s165	nuclear encoded rRNA 5S 165 [Source:MGI Symbol;Acc:MGI:4422029]	6.556791	6.035654	6.028546	6.171709	5.705052	7.184262
17463609	n-R5s166	nuclear encoded rRNA 5S 166 [Source:MGI Symbol;Acc:MGI:4422030]	3.418302	4.663328	4.19942	3.701089	3.091614	3.362397
17463617	n-R5s167	nuclear encoded rRNA 5S 167 [Source:MGI Symbol;Acc:MGI:4422031]	20.54051	17.4532	12.75897	18.94375	16.23021	14.53239
17463971	n-R5s168	nuclear encoded rRNA 5S 168 [Source:MGI Symbol;Acc:MGI:4422032]	4.005632	4.731049	4.05418	5.549085	4.766169	5.268528
17434322	n-R5s169	nuclear encoded rRNA 5S 169 [Source:MGI Symbol;Acc:MGI:4422033]	8.536946	7.797491	7.524105	8.580014	4.218295	11.37031
17445994	n-R5s170	nuclear encoded rRNA 5S 170 [Source:MGI Symbol;Acc:MGI:4422035]	2.741376	1.90903	2.296521	2.47538	2.332893	2.788521
17437479	n-R5s171	nuclear encoded rRNA 5S 171 [Source:MGI Symbol;Acc:MGI:4422036]	3.614084	3.887089	4.117875	4.610841	4.029777	5.027147
17448133	n-R5s172	nuclear encoded rRNA 5S 172 [Source:MGI Symbol;Acc:MGI:4422037]	3.230035	2.726254	3.414892	2.42024	2.900577	2.791151
17440022	n-R5s173	nuclear encoded rRNA 5S 173 [Source:MGI Symbol;Acc:MGI:4422038]	6.333004	4.957387	5.473714	6.287864	9.345423	8.420524
17441394	n-R5s174	nuclear encoded rRNA 5S 174 [Source:MGI Symbol;Acc:MGI:4422039]	6.95119	7.587686	8.772634	6.815928	7.380968	10.70988
17441585	n-R5s175	nuclear encoded rRNA 5S 175 [Source:MGI Symbol;Acc:MGI:4422040]	6.992896	5.261339	7.782372	7.498593	8.296081	6.741175
17441622	n-R5s176	nuclear encoded rRNA 5S 176 [Source:MGI Symbol;Acc:MGI:4422041]	78.74792	59.84546	41.28455	85.96098	77.7074	88.03468
17443110	n-R5s178	nuclear encoded rRNA 5S 178 [Source:MGI Symbol;Acc:MGI:4422043]	4.07743	12.32632	7.892323	5.0969	8.077283	8.924368
17455429	n-R5s179	nuclear encoded rRNA 5S 179 [Source:MGI Symbol;Acc:MGI:4422044]	3.193393	3.427437	3.373462	2.873422	3.236153	3.270724
17539492	n-R5s18	nuclear encoded rRNA 5S 18 [Source:MGI Symbol;Acc:MGI:4421752]	2.623095	2.882759	2.915291	3.162839	3.146919	3.168654
17455496	n-R5s180	nuclear encoded rRNA 5S 180 [Source:MGI Symbol;Acc:MGI:4422045]	21.56142	21.56142	20.90708	21.56142	25.22776	21.56142
17411905	n-R5s182	nuclear encoded rRNA 5S 182 [Source:MGI Symbol;Acc:MGI:4422047]	6.739119	4.488054	3.426629	5.190631	3.626807	4.20927
17412858	n-R5s183	nuclear encoded rRNA 5S 183 [Source:MGI Symbol;Acc:MGI:4422048]	3.075477	3.703322	2.67402	3.703322	3.784388	3.703322

17414993	n-R5s187	nuclear encoded rRNA 5S 187 [Source:MGI Symbol;Acc:MGI:4422052]	2.287022	2.279472	2.32545	2.588004	2.966866	1.920218
17415001	n-R5s188	nuclear encoded rRNA 5S 188 [Source:MGI Symbol;Acc:MGI:4422053]	2.071779	2.045509	2.018103	2.083357	2.093751	2.22
17356670	n-R5s19	nuclear encoded rRNA 5S 19 [Source:MGI Symbol;Acc:MGI:4421753]	3.883991	3.883991	4.615606	4.072317	2.871969	3.229006
17428251	n-R5s191	nuclear encoded rRNA 5S 191 [Source:MGI Symbol;Acc:MGI:4422056]	4.414653	4.367265	5.619922	6.211493	10.35288	6.76275
17418355	n-R5s192	nuclear encoded rRNA 5S 192 [Source:MGI Symbol;Acc:MGI:4422057]	3.254938	4.678934	2.215113	3.031916	5.552394	3.014043
17421856	n-R5s193	nuclear encoded rRNA 5S 193 [Source:MGI Symbol;Acc:MGI:4422058]	145.4813	125.431	139.4912	152.9695	123.3215	136.7313
17395971	n-R5s194	nuclear encoded rRNA 5S 194 [Source:MGI Symbol;Acc:MGI:4422059]	2.068393	2.238714	2.125187	2.591756	2.570217	2.052099
17404963	n-R5s195	nuclear encoded rRNA 5S 195 [Source:MGI Symbol;Acc:MGI:4422060]	6.240406	4.949593	4.39091	5.424311	6.767251	5.004237
17397492	n-R5s196	nuclear encoded rRNA 5S 196 [Source:MGI Symbol;Acc:MGI:4422061]	12.36465	6.763922	5.206306	7.810094	7.159555	7.020433
17399235	n-R5s197	nuclear encoded rRNA 5S 197 [Source:MGI Symbol;Acc:MGI:4422062]	12.61913	16.65889	9.605029	13.33911	11.83444	14.35877
17386683	n-R5s198	nuclear encoded rRNA 5S 198 [Source:MGI Symbol;Acc:MGI:4422063]	3.040608	2.18392	2.133157	3.263862	3.00649	2.421498
17532737	n-R5s2	nuclear encoded rRNA 5S 2 [Source:MGI Symbol;Acc:MGI:4421736]	10.5089	8.14983	9.976958	15.3394	14.96728	12.36868
17387502	n-R5s200	nuclear encoded rRNA 5S 200 [Source:MGI Symbol;Acc:MGI:4422065]	4.804679	4.339654	5.422845	5.401275	4.747671	4.81011
17373994	n-R5s201	nuclear encoded rRNA 5S 201 [Source:MGI Symbol;Acc:MGI:4422066]	2.386341	1.982719	2.057696	2.252489	2.45852	2.257725
17389333	n-R5s202	nuclear encoded rRNA 5S 202 [Source:MGI Symbol;Acc:MGI:4422067]	3.339703	4.20927	3.339703	3.412124	3.621883	3.339703
17374848	n-R5s204	nuclear encoded rRNA 5S 204 [Source:MGI Symbol;Acc:MGI:4422069]	10.91946	10.41904	4.43259	4.776024	10.56382	7.417227
17390261	n-R5s205	nuclear encoded rRNA 5S 205 [Source:MGI Symbol;Acc:MGI:4422070]	2.696878	3.055948	2.94066	3.452497	3.247906	3.551011
17394100	n-R5s207	nuclear encoded rRNA 5S 207 [Source:MGI Symbol;Acc:MGI:4422072]	3.003116	3.292583	2.230968	2.393298	3.220958	2.905809
17221923	n-R5s209	nuclear encoded rRNA 5S 209 [Source:MGI Symbol;Acc:MGI:4422074]	26.37538	34.14494	18.69758	36.52722	23.61596	31.12517
17359676	n-R5s21	nuclear encoded rRNA 5S 21 [Source:MGI Symbol;Acc:MGI:4421755]	3.204568	2.320813	2.733558	2.480928	2.887618	2.958919
17211883	n-R5s210	nuclear encoded rRNA 5S 210 [Source:MGI Symbol;Acc:MGI:4422075]	2.36897	2.169692	2.283347	3.091914	2.568489	2.57521
17214823	n-R5s213	nuclear encoded rRNA 5S 213 [Source:MGI Symbol;Acc:MGI:4422078]	23.11392	9.447417	10.36214	18.15318	21.94499	20.65244
17224952	n-R5s214	nuclear encoded rRNA 5S 214 [Source:MGI Symbol;Acc:MGI:4422079]	2.67402	2.262974	2.350113	2.083357	2.262974	2.00172
17225988	n-R5s216	nuclear encoded rRNA 5S 216 [Source:MGI Symbol;Acc:MGI:4422081]	1.942857	1.988086	2.011651	2.124068	2.260419	2.36043
17227614	n-R5s217	nuclear encoded rRNA 5S 217 [Source:MGI Symbol;Acc:MGI:4422082]	6.348475	5.046489	5.469011	5.527703	4.648451	6.884682
17227826	n-R5s218	nuclear encoded rRNA 5S 218 [Source:MGI Symbol;Acc:MGI:4422083]	2.143146	2.065513	2.421465	2.314499	2.197785	3.221695
17218058	n-R5s219	nuclear encoded rRNA 5S 219 [Source:MGI Symbol;Acc:MGI:4422084]	3.097169	1.998767	2.386274	2.722647	2.523997	2.419284
17228291	n-R5s220	nuclear encoded rRNA 5S 220 [Source:MGI Symbol;Acc:MGI:4422085]	3.328795	3.147748	2.70474	2.466183	3.833893	2.57596
17353167	n-R5s23	nuclear encoded rRNA 5S 23 [Source:MGI Symbol;Acc:MGI:4421758]	3.611905	4.20927	2.362935	2.056242	2.32603	2.972527
17349421	n-R5s24	nuclear encoded rRNA 5S 24 [Source:MGI Symbol;Acc:MGI:4421759]	4.882331	5.457083	3.486864	3.460955	4.622842	5.167477
17332891	n-R5s26	nuclear encoded rRNA 5S 26 [Source:MGI Symbol;Acc:MGI:4421871]	3.379009	3.978791	4.20927	3.881865	3.780822	3.684631
17345150	n-R5s27	nuclear encoded rRNA 5S 27 [Source:MGI Symbol;Acc:MGI:4421872]	2.406006	2.234652	2.285991	2.144543	2.232794	3.014461
17338353	n-R5s28	nuclear encoded rRNA 5S 28 [Source:MGI Symbol;Acc:MGI:4421873]	114.1178	57.21534	45.5434	87.68511	97.07649	90.62455
17339852	n-R5s29	nuclear encoded rRNA 5S 29 [Source:MGI Symbol;Acc:MGI:4421874]	3.56497	3.190118	4.4469	3.920914	3.290279	6.168587
17540323	n-R5s3	nuclear encoded rRNA 5S 3 [Source:MGI Symbol;Acc:MGI:4421737]	27.56164	18.78267	35.32359	27.54064	17.90288	34.41916
17327972	n-R5s30	nuclear encoded rRNA 5S 30 [Source:MGI Symbol;Acc:MGI:4421875]	1.811168	2.70399	2.623877	2.571143	3.029501	2.166941
17328482	n-R5s31	nuclear encoded rRNA 5S 31 [Source:MGI Symbol;Acc:MGI:4421876]	3.244891	3.274989	2.67402	3.044531	3.680368	3.907322
17329622	n-R5s32	nuclear encoded rRNA 5S 32 [Source:MGI Symbol;Acc:MGI:4421877]	3.468063	3.836073	2.962572	3.154169	2.962572	3.39469
17324959	n-R5s33	nuclear encoded rRNA 5S 33 [Source:MGI Symbol;Acc:MGI:4421878]	556.7902	506.3601	471.8322	577.4331	552.971	563.0815
17325531	n-R5s34	nuclear encoded rRNA 5S 34 [Source:MGI Symbol;Acc:MGI:4421879]	2.436601	2.059623	2.106941	2.230829	2.332634	2.106941
17330803	n-R5s36	nuclear encoded rRNA 5S 36 [Source:MGI Symbol;Acc:MGI:4421881]	3.28727	2.191092	2.255364	1.868927	2.241431	2.580767
17331348	n-R5s37	nuclear encoded rRNA 5S 37 [Source:MGI Symbol;Acc:MGI:4421882]	1.833042	1.751232	1.787954	1.999667	2.457038	1.843976
17316735	n-R5s39	nuclear encoded rRNA 5S 39 [Source:MGI Symbol;Acc:MGI:4421884]	2.953407	2.814093	2.835789	3.625374	2.858127	3.240688
17313037	n-R5s40	nuclear encoded rRNA 5S 40 [Source:MGI Symbol;Acc:MGI:4421885]	3.042232	4.097546	3.829962	5.458256	3.040545	3.420175
17313575	n-R5s41	nuclear encoded rRNA 5S 41 [Source:MGI Symbol;Acc:MGI:4421886]	2.938195	2.446586	2.339256	3.117479	2.574764	3.134684
17313974	n-R5s42	nuclear encoded rRNA 5S 42 [Source:MGI Symbol;Acc:MGI:4421887]	4.381759	5.143891	4.980291	4.208949	6.303705	6.347815
17315031	n-R5s43	nuclear encoded rRNA 5S 43 [Source:MGI Symbol;Acc:MGI:4421888]	2.347248	2.359318	2.481049	2.118099	2.827486	2.841751
17303536	n-R5s45	nuclear encoded rRNA 5S 45 [Source:MGI Symbol;Acc:MGI:4421890]	5.501748	3.748959	3.836073	4.31461	3.197778	4.878914
17305114	n-R5s46	nuclear encoded rRNA 5S 46 [Source:MGI Symbol;Acc:MGI:4421891]	23.90483	16.95877	22.57368	24.86837	24.58847	27.78145
17301190	n-R5s47	nuclear encoded rRNA 5S 47 [Source:MGI Symbol;Acc:MGI:4421892]	4.182832	5.938671	5.241937	5.241937	5.989103	4.94517
17302136	n-R5s48	nuclear encoded rRNA 5S 48 [Source:MGI Symbol;Acc:MGI:4421893]	9.777394	9.294775	5.140291	8.818724	8.069224	8.872742
17540434	n-R5s5	nuclear encoded rRNA 5S 5 [Source:MGI Symbol;Acc:MGI:4421739]	3.002387	3.268208	2.050165	2.024927	3.706609	2.213133
17302594	n-R5s50	nuclear encoded rRNA 5S 50 [Source:MGI Symbol;Acc:MGI:4421895]	197.5755	195.8561	203.5614	240.8348	189.3419	257.9558
17309455	n-R5s51	nuclear encoded rRNA 5S 51 [Source:MGI Symbol;Acc:MGI:4421896]	5.502244	4.20927	4.20927	4.620311	4.147638	4.20927
17290736	n-R5s52	nuclear encoded rRNA 5S 52 [Source:MGI Symbol;Acc:MGI:4421897]	3.462322	3.462322	3.897314	3.462322	3.383837	3.060548
17292632	n-R5s54	nuclear encoded rRNA 5S 54 [Source:MGI Symbol;Acc:MGI:4421899]	8.500278	5.648783	5.022271	5.648783	4.90822	4.451989
17289549	n-R5s56	nuclear encoded rRNA 5S 56 [Source:MGI Symbol;Acc:MGI:4421901]	7.120655	2.335805	4.724822	5.774316	4.915506	6.143071
17281079	n-R5s58	nuclear encoded rRNA 5S 58 [Source:MGI Symbol;Acc:MGI:4421903]	2.225208	2.342989	2.32795	2.269697	2.558982	2.346938
17534669	n-R5s6	nuclear encoded rRNA 5S 6 [Source:MGI Symbol;Acc:MGI:4421740]	5.975627	6.633039	6.175945	5.738048	6.164953	5.366866
17281479	n-R5s60	nuclear encoded rRNA 5S 60 [Source:MGI Symbol;Acc:MGI:4421905]	3.891186	3.320867	3.682588	3.532109	3.386535	3.94023
17275873	n-R5s61	nuclear encoded rRNA 5S 61 [Source:MGI Symbol;Acc:MGI:4421906]	2.777352	2.836241	2.156333	2.494499	1.779041	3.019082
17276983	n-R5s62	nuclear encoded rRNA 5S 62 [Source:MGI Symbol;Acc:MGI:4421907]	11.01242	13.06568	12.08847	16.02808	17.18776	13.06568
17282395	n-R5s63	nuclear encoded rRNA 5S 63 [Source:MGI Symbol;Acc:MGI:4421908]	2.247156	2.420844	2.335417	2.409594	2.729847	2.371139
17282730	n-R5s64	nuclear encoded rRNA 5S 64 [Source:MGI Symbol;Acc:MGI:4421909]	29.64589	33.51767	22.61127	25.21971	32.15743	26.62778
17277754	n-R5s65	nuclear encoded rRNA 5S 65 [Source:MGI Symbol;Acc:MGI:4421910]	7.467421	5.065238	4.199827	3.455274	7.070244	4.166136
17247078	n-R5s67	nuclear encoded rRNA 5S 67 [Source:MGI Symbol;Acc:MGI:4421912]	2.33278	2.036356	2.469656	2.584365	3.345032	2.078352
17247927	n-R5s68	nuclear encoded rRNA 5S 68 [Source:MGI Symbol;Acc:MGI:4421913]	6.992896	6.992896	8.918926	9.908386	7.209804	6.980885
17249916	n-R5s69	nuclear encoded rRNA 5S 69 [Source:MGI Symbol;Acc:MGI:4421914]	21.94226	22.25442	22.71447	18.69797	19.84674	20.00654
17542133	n-R5s7	nuclear encoded rRNA 5S 7 [Source:MGI Symbol;Acc:MGI:4421741]	2.947598	4.502543	2.789139	4.383004	2.842007	3.469385
17252339	n-R5s70	nuclear encoded rRNA 5S 70 [Source:MGI Symbol;Acc:MGI:4421915]	62.25207	36.79455	41.08216	80.87153	37.04508	59.93804
17252754	n-R5s71	nuclear encoded rRNA 5S 71 [Source:MGI Symbol;Acc:MGI:4421916]	391.4434	398.6586	346.2907	427.9208	379.0068	394.6719
17267700	n-R5s72	nuclear encoded rRNA 5S 72 [Source:MGI Symbol;Acc:MGI:4421917]	5.106731	5.124602	6.33946	4.271729	5.124602	5.163896
17268784	n-R5s73	nuclear encoded rRNA 5S 73 [Source:MGI Symbol;Acc:MGI:4421918]	1.804051	2.090561	1.801602	1.898013	1.843733	2.017222
17272337	n-R5s74	nuclear encoded rRNA 5S 74 [Source:MGI Symbol;Acc:MGI:4421919]	2.406006	2.874259	3.183977	2.957032	4.442063	2.933677
17240087	n-R5s76	nuclear encoded rRNA 5S 76 [Source:MGI Symbol;Acc:MGI:4421924]	8.101449	9.706828	8.236378	9.264352	10.20497	11.38206
17234798	n-R5s77	nuclear encoded rRNA 5S 77 [Source:MGI Symbol;Acc:MGI:4421925]	4.712557	3.410776	5.35082	4.239517	2.058338	6.347815
17243953	n-R5s79	nuclear encoded rRNA 5S 79 [Source:MGI Symbol;Acc:MGI:4421927]	32.50748	30.16618	31.6155	33.94624	38.48755	33.14318
17535296	n-R5s8	nuclear encoded rRNA 5S 8 [Source:MGI Symbol;Acc:MGI:4421742]	2.255895	2.406006	2.290559	3.02694	2.876611	2.947169
17244693	n-R5s80	nuclear encoded rRNA 5S 80 [Source:MGI Symbol;Acc:MGI:4421928]	6.513171	4.791542	6.287864	6.882773	5.320235	10.29855
17516028	n-R5s82	nuclear encoded rRNA 5S 82 [Source:MGI Symbol;Acc:MGI:4421930]	2.830702	2.674353	2.690268	3.329141	2.503662	3.128237
17518189	n-R5s84	nuclear encoded rRNA 5S 84 [Source:MGI Symbol;Acc:MGI:4421932]	4.759797	6.797197	4.834779	4.821126	4.759797	5.36285
17518634	n-R5s85	nuclear encoded rRNA 5S 85 [Source:MGI Symbol;Acc:MGI:4421933]	2.806982	3.781451	3.258505	4.366659	2.785218	2.953407
17519314	n-R5s86	nuclear encoded rRNA 5S 86 [Source:MGI Symbol;Acc:MGI:4421934]	8.638555	6.243565	4.605699	6.201469	8.915712	9.10204
17529842	n-R5s87	nuclear encoded rRNA 5S 87 [Source:MGI Symbol;Acc:MGI:4421935]	2.090242	2.220524	2.264873	2.251147	1.882787	2.366344

17530227	n-R5s89	nuclear encoded rRNA 5S 89 [Source:MGI Symbol;Acc:MGI:4421937]	416.0174	293.7938	348.2815	462.8946	349.1565	458.364
17536163	n-R5s9	nuclear encoded rRNA 5S 9 [Source:MGI Symbol;Acc:MGI:4421743]	3.883991	3.883991	3.883991	3.617818	3.797974	3.729055
17531749	n-R5s90	nuclear encoded rRNA 5S 90 [Source:MGI Symbol;Acc:MGI:4421938]	2.704965	3.22069	3.602254	3.643437	3.180867	3.446161
17531981	n-R5s92	nuclear encoded rRNA 5S 92 [Source:MGI Symbol;Acc:MGI:4421940]	5.338965	20.79983	14.7078	18.49649	17.15836	16.56608
17507306	n-R5s93	nuclear encoded rRNA 5S 93 [Source:MGI Symbol;Acc:MGI:4421941]	5.127267	3.563883	4.20927	5.772675	3.354528	3.752469
17507317	n-R5s94	nuclear encoded rRNA 5S 94 [Source:MGI Symbol;Acc:MGI:4421942]	4.058341	3.117328	3.009972	3.207124	3.30548	3.712369
17507353	n-R5s95	nuclear encoded rRNA 5S 95 [Source:MGI Symbol;Acc:MGI:4421943]	1.924508	1.837686	2.002525	2.031845	2.064053	2.202589
17501094	n-R5s96	nuclear encoded rRNA 5S 96 [Source:MGI Symbol;Acc:MGI:4421944]	4.974805	3.916541	4.257186	5.670398	4.043992	5.024326
17501102	n-R5s97	nuclear encoded rRNA 5S 97 [Source:MGI Symbol;Acc:MGI:4421945]	3.462322	2.932904	2.322406	5.073355	3.603128	3.134488
17501202	n-R5s98	nuclear encoded rRNA 5S 98 [Source:MGI Symbol;Acc:MGI:4421946]	25.16593	24.31121	27.89646	22.95601	29.72304	31.13186
17281529	Nemf	nuclear export mediator factor	41.7962	36.03458	30.4348	28.65411	34.70063	36.03458
17415606	Nfia	nuclear factor I/A	53.6758	69.5708	74.41079	55.05837	69.29935	56.8406
17426765	Nfib	nuclear factor I/B	207.6889	265.4378	276.338	219.3423	230.1132	251.2762
17243330	Nfic	nuclear factor I/C	66.94944	76.88232	80.81437	73.62634	67.76648	76.13458
17511142	Nfix	nuclear factor I/X	43.876	39.23692	47.64325	39.63354	40.61413	42.13859
17505260	Nfat5	nuclear factor of activated T cells 5	31.66309	49.32134	47.70042	37.97689	32.12557	31.61637
17355670	Nfatc1	nuclear factor of activated T cells, cytoplasmic, calcineurin dependent 1	27.81999	29.9273	32.9131	31.24081	24.07927	29.64835
17394806	Nfatc2	nuclear factor of activated T cells, cytoplasmic, calcineurin dependent 2	9.640313	10.01479	9.933898	10.17827	9.810455	11.93544
17496183	Nfatc2ip	nuclear factor of activated T cells, cytoplasmic, calcineurin dependent 2 interacting	9.126046	11.12621	7.379024	8.204014	11.18691	9.759316
17505034	Nfatc3	nuclear factor of activated T cells, cytoplasmic, calcineurin dependent 3	62.81294	61.12582	60.86327	62.5644	68.85025	59.52237
17300672	Nfatc4	nuclear factor of activated T cells, cytoplasmic, calcineurin dependent 4	11.39927	11.53519	11.39927	11.39927	11.39927	9.956302
17410542	Nfkb1	nuclear factor of kappa light polypeptide gene enhancer in B cells 1, p105	55.72721	50.68019	73.05191	53.29987	54.07538	56.8879
17359967	Nfkb2	nuclear factor of kappa light polypeptide gene enhancer in B cells 2, p49/p100	18.69979	17.38728	22.36297	22.59795	21.0528	18.2215
17344322	Nfkbil1	nuclear factor of kappa light polypeptide gene enhancer in B cells inhibitor like 1	14.47268	14.5689	15.79701	14.85061	11.79865	14.41131
17281219	Nfkbia	nuclear factor of kappa light polypeptide gene enhancer in B cells inhibitor, alpha	83.08119	99.65429	98.56886	79.84112	109.3235	118.12
17488544	Nfkbib	nuclear factor of kappa light polypeptide gene enhancer in B cells inhibitor, beta	51.99857	51.79604	57.04466	52.07756	51.57464	51.67448
17476372	Nfkbid	nuclear factor of kappa light polypeptide gene enhancer in B cells inhibitor, delta	6.007897	6.162689	6.177829	7.812802	6.463609	5.813069
17337927	Nfkbie	nuclear factor of kappa light polypeptide gene enhancer in B cells inhibitor, epsilc	7.319423	9.324911	9.086911	7.372327	8.113419	9.332477
17330967	Nfkbiz	nuclear factor of kappa light polypeptide gene enhancer in B cells inhibitor, zeta	13.35983	16.22448	15.49596	17.06608	14.01422	16.73272
17515758	Nfrkb	nuclear factor related to kappa B binding protein	23.3853	33.08488	33.24142	26.58997	29.76737	27.63453
17322337	Nfe2	nuclear factor, erythroid derived 2	3.789953	4.167436	4.068621	4.206791	3.783627	3.967857
17386700	Nfe2l2	nuclear factor, erythroid derived 2, like 2	152.1457	163.4403	151.8118	120.0124	120.9964	120.5009
17458550	Nfe2l3	nuclear factor, erythroid derived 2, like 3	8.487031	7.384755	8.763213	7.76486	8.499512	10.69356
17268356	Nfe2l1	nuclear factor, erythroid derived 2,-like 1	51.01572	57.09451	70.81289	55.46478	54.60761	62.84386
17292634	Nfil3	nuclear factor, interleukin 3, regulated	57.70082	27.74258	31.71141	31.29434	35.09516	34.66866
17302061	Nufip1	nuclear fragile X mental retardation protein interacting protein 1	17.64992	17.09355	17.60935	19.71921	19.85637	19.22991
17253307	Nufip2	nuclear fragile X mental retardation protein interacting protein 2	49.18444	49.09452	49.18444	51.73756	42.63836	49.18444
17301391	Nuggc	nuclear GTPase, germinal center associated	3.883991	3.89356	3.494316	3.883991	4.375901	4.067832
17505239	Nip7	nuclear import 7 homolog (S. cerevisiae)	30.2449	32.12134	31.99268	36.65048	33.81215	34.32648
17480986	Numa1	nuclear mitotic apparatus protein 1	68.76965	70.75646	75.1541	66.30565	72.65398	69.90773
17361805	Neat1	nuclear paraspeckle assembly transcript 1 (non-protein coding)	83.04952	189.6269	195.1338	172.6157	161.0629	142.02
17453557	Pom121	nuclear pore membrane protein 121	49.1991	47.83451	51.26705	40.18848	43.1641	33.80465
17259585	Narf	nuclear prelamin A recognition factor	59.83219	58.66304	56.96129	49.99979	51.49855	58.33824
17334749	Narfl	nuclear prelamin A recognition factor-like	35.78096	35.4143	29.88376	37.70803	29.09542	33.39662
17517323	Npat	nuclear protein in the AT region	18.19866	16.38598	14.43921	19.60827	18.84043	13.56672
17273086	Nploc4	nuclear protein localization 4 homolog (S. cerevisiae)	309.531	300.5539	297.2556	276.6657	213.6285	220.8159
17496345	Nupr1	nuclear protein transcription regulator 1	20.92448	28.99696	38.53106	20.5884	22.10346	22.95681
17442966	Nupr1l	nuclear protein transcriptional regulator 1 like	12.13044	11.50086	11.20546	11.35417	11.6363	12.5814
17501810	Nr2c2ap	nuclear receptor 2C2-associated protein	18.36287	15.85285	13.18294	13.53901	14.02725	15.45348
17241637	Nrbf2	nuclear receptor binding factor 2	70.56447	69.11467	59.88488	65.88052	70.45499	68.31072
17436077	Nrbp1	nuclear receptor binding protein 1	158.1347	128.4755	119.4315	135.929	145.6831	137.7883
17318312	Nrbp2	nuclear receptor binding protein 2	45.91648	51.49249	59.09688	47.96433	45.29469	48.80713
17279805	Ncoa1	nuclear receptor coactivator 1	57.47689	57.47689	57.36504	51.50248	57.43905	57.47689
17279830	Ncoa1	nuclear receptor coactivator 1	8.330886	7.728241	10.78796	7.45196	8.350369	7.255827
17221317	Ncoa2	nuclear receptor coactivator 2	86.60389	107.9411	105.8972	84.48305	99.15956	103.4859
17320858	D030018L15Rik	nuclear receptor coactivator 2 pseudogene	4.147839	3.235862	3.68343	3.601006	2.667559	3.197269
17379719	Ncoa3	nuclear receptor coactivator 3	48.2946	47.33253	55.66814	47.4064	44.83178	45.5494
17298510	Ncoa4	nuclear receptor coactivator 4	26.20155	25.62944	25.36136	20.62854	24.15433	20.08087
17394349	Ncoa5	nuclear receptor coactivator 5	34.30365	28.56111	26.23335	21.4636	21.4636	24.79539
17393244	Ncoa6	nuclear receptor coactivator 6	45.48377	65.98655	57.06997	67.4539	49.5389	48.01855
17240053	Ncoa7	nuclear receptor coactivator 7	12.42824	11.62872	15.23914	12.84469	15.26431	11.11665
17263959	Ncor1	nuclear receptor co-repressor 1	147.4672	160.0923	170.5274	155.0597	144.7912	148.9743
17452897	Ncor2	nuclear receptor co-repressor 2	41.66257	46.36486	56.9309	54.43339	48.52479	45.50932
17331564	Nrip1	nuclear receptor interacting protein 1	48.48041	61.19068	60.26673	47.4064	41.70447	43.36082
17463422	Nrip2	nuclear receptor interacting protein 2	11.33506	10.19818	10.68778	9.647064	10.91038	14.05294
17494940	Nrip3	nuclear receptor interacting protein 3	4.577437	3.372574	2.979975	4.912917	4.627972	3.489161
17536201	Nr0b1	nuclear receptor subfamily 0, group B, member 1	2.747806	2.488351	2.824509	2.804104	2.529286	2.630195
17419622	Nr0b2	nuclear receptor subfamily 0, group B, member 2	106.8216	47.157	44.15852	117.9613	75.97696	92.06813
17268884	Nr1d1	nuclear receptor subfamily 1, group D, member 1	71.28615	166.1209	166.2695	130.3024	144.6678	195.9104
17303625	Nr1d2	nuclear receptor subfamily 1, group D, member 2	50.56124	108.1013	86.31025	74.98084	101.2866	104.14
17490312	Nr1h2	nuclear receptor subfamily 1, group H, member 2	90.6302	103.3891	119.0901	107.1605	101.2277	98.10536
17388177	Nr1h3	nuclear receptor subfamily 1, group H, member 3	68.61442	72.21317	70.04062	73.76988	68.71056	64.60975
17244070	Nr1h4	nuclear receptor subfamily 1, group H, member 4	132.2707	156.0376	202.8838	141.3639	157.7624	162.2933
17408625	Nr1h5	nuclear receptor subfamily 1, group H, member 5	4.660291	4.8555	4.757026	5.192754	4.363331	5.100681
17330323	Nr1i2	nuclear receptor subfamily 1, group I, member 2	95.07539	126.3682	130.232	128.4827	128.9153	100.4699
17219231	Nr1i3	nuclear receptor subfamily 1, group I, member 3	136.647	246.9115	280.5161	209.9599	249.5854	277.1609
17236662	Nr2c1	nuclear receptor subfamily 2, group C, member 1	59.2741	46.30577	41.31632	47.54132	58.38072	52.11295
17461023	Nr2c2	nuclear receptor subfamily 2, group C, member 2	40.48681	51.43755	51.09428	42.86892	47.37224	51.09428
17240556	Nr2e1	nuclear receptor subfamily 2, group E, member 1	4.824602	5.009373	5.127871	5.031017	4.783047	4.951343
17527830	Nr2e3	nuclear receptor subfamily 2, group E, member 3	4.511696	4.239517	4.239517	4.239517	4.131283	4.165819
17294547	Nr2f1	nuclear receptor subfamily 2, group F, member 1	7.863382	6.88578	8.152542	9.431518	8.011709	11.45623
17492066	Nr2f2	nuclear receptor subfamily 2, group F, member 2	28.82503	38.13728	40.1999	28.44218	30.21326	33.67019
17510280	Nr2f6	nuclear receptor subfamily 2, group F, member 6	46.71552	58.73343	49.25779	49.62173	45.17585	43.60314
17353985	Nr3c1	nuclear receptor subfamily 3, group C, member 1	83.96115	92.18499	92.77988	89.3515	85.00584	100.1522

17502626	Nr3c2	nuclear receptor subfamily 3, group C, member 2	18.76068	19.71074	20.21984	21.00484	21.00484	20.87291
17315178	Nr4a1	nuclear receptor subfamily 4, group A, member 1	21.08785	34.22978	17.43748	13.5862	10.9212	9.648335
17385374	Nr4a2	nuclear receptor subfamily 4, group A, member 2	17.43108	16.33427	12.56684	8.911078	13.08172	18.34061
17413945	Nr4a3	nuclear receptor subfamily 4, group A, member 3	5.953095	5.435188	6.225199	4.726231	5.088816	6.75619
17384705	Nr5a1	nuclear receptor subfamily 5, group A, member 1	6.44214	6.90418	6.588271	6.262332	6.44214	7.311006
17370475	Nr5a1os	nuclear receptor subfamily 5, group A, member 1, opposite strand [Source:MGI Sy	4.154515	2.992352	3.550224	3.56334	2.832351	3.681133
17227515	Nr5a2	nuclear receptor subfamily 5, group A, member 2	126.684	150.2145	136.6565	124.8498	145.8923	174.5988
17384717	Nr6a1	nuclear receptor subfamily 6, group A, member 1	9.077594	7.685824	6.713244	7.698994	7.299461	7.640145
17287613	Nsd1	nuclear receptor-binding SET-domain protein 1	73.62736	76.43704	78.25006	76.43704	72.64139	84.10152
17456804	Nrf1	nuclear respiratory factor 1	22.48265	24.78972	23.83385	22.92008	28.52055	22.4389
17357183	Nxf1	nuclear RNA export factor 1	50.91292	52.64848	50.0036	50.49295	49.02413	50.0036
17544558	Nxf2	nuclear RNA export factor 2	2.690846	2.704984	2.525064	2.627462	2.602702	2.611864
17544696	Nxf3	nuclear RNA export factor 3	3.199752	2.920368	2.782805	2.953407	2.792254	2.937523
17544631	Nxf7	nuclear RNA export factor 7	3.872728	3.245813	3.446017	3.381258	3.71224	3.317623
17266185	Nsrp1	nuclear speckle regulatory protein 1	49.63583	49.85236	46.94729	58.87119	44.09735	50.52411
17412813	Nfx1	nuclear transcription factor, X-box binding 1	45.86431	44.11049	38.68842	41.58064	31.93795	41.52188
17448645	Nfxl1	nuclear transcription factor, X-box binding-like 1	43.96123	22.59983	23.40346	32.22704	28.06091	26.84776
17345782	Nfya	nuclear transcription factor-Y alpha	28.43626	22.69087	29.08191	24.55645	24.49593	26.25681
17243509	Nfyb	nuclear transcription factor-Y beta	30.91596	29.8819	33.07525	36.79072	26.32534	35.7408
17429495	Nfyc	nuclear transcription factor-Y gamma	29.49462	30.72817	31.02308	26.11126	22.73637	30.29127
17504958	Nutf2	nuclear transport factor 2	24.82772	17.16526	17.16526	16.23595	15.80369	17.16526
17365714	Nutf2-ps1	nuclear transport factor 2, pseudogene 1	113.1325	123.1609	146.9977	121.7958	113.361	116.9583
17352656	Nutf2-ps2	nuclear transport factor 2, pseudogene 2	145.9055	204.9986	233.9603	173.4745	169.695	177.095
17538284	Nxt2	nuclear transport factor 2-like export factor 2	41.10352	37.42914	27.49448	32.0615	32.16322	32.67579
17233294	Nus1	nuclear undecaprenyl pyrophosphate synthase 1 homolog (S. cerevisiae)	97.15727	93.51591	98.17951	100.9369	91.63139	87.75017
17230499	Nvl	nuclear VCP-like	34.95798	23.11136	29.94057	34.49272	26.54411	31.47294
17222825	Nabp1	nucleic acid binding protein 1	52.84774	45.01706	32.46853	37.01223	40.81931	36.7479
17246131	Nabp2	nucleic acid binding protein 2	55.89704	57.84579	58.40419	48.93077	45.36098	41.96574
17490859	Nucb1	nucleobindin 1	112.1604	99.34049	114.2009	80.29343	94.37678	91.28399
17482095	Nucb2	nucleobindin 2	53.41637	27.23009	29.12407	37.43251	29.16103	22.49621
17359902	Nolc1	nucleolar and coiled-body phosphoprotein 1	44.71106	47.70141	39.36878	46.3382	48.02088	41.76869
17533743	Gm5124	nucleolar and coiled-body phosphoprotein 1 pseudogene	9.701582	9.477654	11.24366	13.0175	11.62509	11.24366
17374833	Nusap1	nucleolar and spindle associated protein 1	7.863001	6.70901	12.14037	12.80788	7.796951	8.302582
17422914	Noc2l	nucleolar complex associated 2 homolog (S. cerevisiae)	47.17661	50.27537	51.60146	46.22784	50.27537	56.38228
17364367	Noc3l	nucleolar complex associated 3 homolog (S. cerevisiae)	32.63323	30.18961	27.70895	28.52727	29.14063	27.64717
17451043	Noc4l	nucleolar complex associated 4 homolog (S. cerevisiae)	11.03649	11.7531	10.39783	11.16453	9.828628	11.1252
17274267	Nol10	nucleolar protein 10	15.15204	14.31296	13.2773	16.37962	14.31296	13.52859
17271091	Nol11	nucleolar protein 11	34.78661	27.2067	23.23343	27.2067	27.2067	28.94776
17547640	Nol11	nucleolar protein 11	7.942317	9.169036	7.097741	9.077531	7.894621	10.84584
17312835	Nol12	nucleolar protein 12	24.71903	19.74123	23.8279	22.04195	24.1463	21.84952
17504707	Nol3	nucleolar protein 3 (apoptosis repressor with CARD domain)	17.13364	16.93012	17.25413	14.41032	15.04519	15.09041
17353079	Nol4	nucleolar protein 4	3.060209	3.254148	3.214312	3.294798	3.185324	3.639348
17393051	Nol4l	nucleolar protein 4-like	4.681043	4.894054	4.651481	5.058641	4.767622	5.75769
17286882	Nol7	nucleolar protein 7	25.49443	24.57603	25.71093	23.90764	34.57147	23.39405
17287185	Nol8	nucleolar protein 8	56.0946	42.98049	42.30599	49.11085	46.05769	40.61611
17422047	Nol9	nucleolar protein 9	41.26395	40.30704	31.43479	32.38783	30.39812	34.36457
17424128	Nol6	nucleolar protein family 6 (RNA-associated)	62.7468	58.02771	58.97166	62.1598	49.94402	51.06384
17216563	Nifk	nucleolar protein interacting with the FHA domain of MKI67	68.64106	61.81864	52.87779	60.51454	66.25374	63.93039
17435660	Nom1	nucleolar protein with MIF4G domain 1	23.27695	25.66321	25.36118	20.29525	21.66584	33.1611
17225153	Ncl	nucleolin	130.2383	139.7205	123.4275	120.3798	141.8832	123.8431
17325849	Nepro	nucleolus and neural progenitor protein	16.73515	21.22364	23.37898	17.59569	19.06824	19.72523
17261550	Npm1	nucleophosmin 1	7.948486	10.31885	8.268067	8.982768	10.62124	9.365526
17308442	Npm2	nucleophosmin/nucleoplasmin 2	15.05468	13.59439	15.12172	13.63082	14.31187	14.31504
17548852	LOC105243690	nucleophosmin-like	116.9485	116.5843	115.4104	119.4299	150.783	127.6297
17365243	Npm3	nucleoplasmin 3	83.12151	97.38516	82.7708	94.04178	106.8972	84.38063
17460254	Npm3-ps1	nucleoplasmin 3, pseudogene 1	2.344516	2.094869	2.09811	2.245318	2.005205	2.072138
17245272	Nup107	nucleoporin 107	17.09864	13.29047	12.65872	15.52735	13.92495	14.19479
17514004	Nup133	nucleoporin 133	24.73205	24.40103	22.30786	25.0148	21.34121	29.90677
17292190	Nup153	nucleoporin 153	63.03407	62.99869	61.44868	63.03407	68.26906	64.41211
17310078	Nup155	nucleoporin 155	80.07778	58.56107	54.25221	58.60086	62.12276	54.75391
17372984	Nup160	nucleoporin 160	19.71156	19.34126	18.60515	19.36084	20.30904	21.27888
17369147	Nup188	nucleoporin 188	29.14568	29.14366	29.14568	29.14568	29.14568	25.81325
17457249	Nup205	nucleoporin 205	23.70879	28.38978	22.96126	23.22393	24.66118	22.98021
17468961	Nup210	nucleoporin 210	31.44655	27.77182	30.86479	26.32333	26.93649	25.42613
17399604	Nup210l	nucleoporin 210-like	3.564624	3.564624	3.564624	3.785149	3.501905	3.775218
17369627	Nup214	nucleoporin 214	29.50873	29.50035	33.29284	33.43761	27.51812	32.45188
17372444	Nup35	nucleoporin 35	3.568753	2.955864	4.189767	4.511915	3.202747	3.548674
17495368	Nup35	nucleoporin 35	7.308878	9.341667	10.2333	10.59704	7.814535	6.850503
17236313	Nup37	nucleoporin 37	14.79769	13.07157	14.51517	14.96977	14.44512	15.05677
17231534	Nup43	nucleoporin 43	10.15691	7.038851	9.810455	11.07113	9.402862	9.112077
17313767	Nup50	nucleoporin 50	60.93883	54.14965	50.97543	57.80691	66.80434	60.34867
17469274	1700123L14Rik	nucleoporin 50 pseudogene	7.370538	6.360102	6.241921	5.96309	5.706436	5.862029
17449731	Nup54	nucleoporin 54	49.51281	43.02997	41.76001	42.11377	46.64143	40.81535
17544919	Nup62cl	nucleoporin 62 C-terminal like	3.493686	4.801649	4.348959	4.61567	4.309767	4.569607
17258239	Nup85	nucleoporin 85	20.22727	23.98682	23.61154	21.11227	19.17773	23.51403
17265393	Nup88	nucleoporin 88	25.36118	24.16153	25.36118	25.36118	20.76181	25.06166
17503948	Nup93	nucleoporin 93	18.3807	14.13353	11.49457	13.96322	14.48251	9.424199
17494041	Nup98	nucleoporin 98	95.50273	86.77274	83.49686	77.00125	76.40155	72.28728
17307403	Nupl1	nucleoporin like 1	66.37969	70.15432	61.9718	64.35052	62.90662	52.20442
17435267	Nupl2	nucleoporin like 2	14.31683	15.77426	13.33467	14.11424	12.9588	14.44812
17266095	Nxn	nucleoredoxin	8.898363	8.216705	6.599103	8.136679	8.935819	8.819764
17510358	Nxnl1	nucleoredoxin-like 1	5.854557	5.975875	4.047273	5.309404	5.002641	4.805178
17287320	Nxnl2	nucleoredoxin-like 2	6.552748	6.506853	5.629981	5.716056	5.412969	5.829491
17506891	Ntpcr	nucleoside-triphosphatase, cancer-related	15.60461	11.52241	15.45659	13.14361	13.45322	12.85796

17237252	Nap1l1	nucleosome assembly protein 1-like 1	50.5122	54.87853	54.87967	46.58069	52.65396	41.07304
17543781	Nap1l2	nucleosome assembly protein 1-like 2	2.704553	2.628355	2.553418	2.621368	2.732478	2.686746
17544284	Nap1l3	nucleosome assembly protein 1-like 3	4.643717	4.33136	4.06017	4.301441	4.191626	5.242155
17498255	Nap1l4	nucleosome assembly protein 1-like 4	68.28042	56.56548	64.49431	66.23813	64.95326	62.30172
17467205	Nap1l5	nucleosome assembly protein 1-like 5	4.681043	4.681043	5.509304	5.494964	4.381607	4.681043
17322829	Nubp1	nucleotide binding protein 1	40.06248	40.03223	40.42792	42.85376	40.88756	37.56117
17342042	Nubp2	nucleotide binding protein 2	69.40896	62.25639	52.0967	51.08118	56.74888	51.29869
17275408	Nubpl	nucleotide binding protein-like	31.27006	18.80768	16.33755	25.85354	21.31711	21.62488
17467015	Nod1	nucleotide-binding oligomerization domain containing 1	32.70932	27.48724	31.66199	36.33832	33.03263	34.1492
17503664	Nod2	nucleotide-binding oligomerization domain containing 2	18.01042	16.09533	15.95614	19.28838	18.34316	16.40303
17511134	Nacc1	nucleus accumbens associated 1, BEN and BTB (POZ) domain containing	61.49128	57.10915	65.99569	53.99336	45.7275	51.87509
17382822	Nacc2	nucleus accumbens associated 2, BEN and BTB (POZ) domain containing	27.31043	27.27864	30.88833	29.22781	29.42213	28.98832
17316831	Nudcd1	NudC domain containing 1	45.32955	34.66337	30.62526	33.69494	37.29447	35.36082
17248512	Nudcd2	NudC domain containing 2	29.23551	29.36102	30.55253	33.57115	28.43804	33.2147
17260295	Nudcd3	NudC domain containing 3	32.62826	35.58433	32.18218	28.29451	30.81904	32.51763
17444068	Nudt1	nudix (nucleoside diphosphate linked moiety X)-type motif 1	16.78953	16.25859	17.23584	19.42873	13.19482	16.52261
17539768	Nudt10	nudix (nucleoside diphosphate linked moiety X)-type motif 10	3.021594	3.034082	2.653341	3.451396	2.615705	3.09513
17532732	Nudt11	nudix (nucleoside diphosphate linked moiety X)-type motif 11	5.303372	4.936813	4.971358	5.09637	7.443494	4.20927
17346618	Nudt12	nudix (nucleoside diphosphate linked moiety X)-type motif 12	132.7621	138.2178	128.063	133.7041	116.2003	130.4298
17339066	Nudt12os	nudix (nucleoside diphosphate linked moiety X)-type motif 12, opposite strand	4.367356	4.20927	3.793712	4.113681	4.085266	4.58474
17297413	Nudt13	nudix (nucleoside diphosphate linked moiety X)-type motif 13	33.37856	32.68734	31.28372	35.9002	29.16103	36.6063
17284280	Nudt14	nudix (nucleoside diphosphate linked moiety X)-type motif 14	13.36928	14.31395	16.21481	16.80047	16.3828	15.10998
17308612	Nudt15	nudix (nucleoside diphosphate linked moiety X)-type motif 15	4.01461	3.472826	4.3749	3.703322	3.44542	3.656796
17530424	Nudt16	nudix (nucleoside diphosphate linked moiety X)-type motif 16	25.47146	27.15865	25.84745	25.47146	22.89118	20.45881
17322621	Nudt16l1	nudix (nucleoside diphosphate linked moiety X)-type motif 16-like 1	33.39847	28.93573	28.89484	26.55884	31.3219	27.70607
17408090	Nudt17	nudix (nucleoside diphosphate linked moiety X)-type motif 17	8.292057	6.945218	6.992896	7.322976	6.992896	7.025496
17301796	Nudt18	nudix (nucleoside diphosphate linked moiety X)-type motif 18	31.3471	23.44519	28.19779	29.54496	36.32447	19.87331
17489801	Nudt19	nudix (nucleoside diphosphate linked moiety X)-type motif 19	50.61489	39.63299	43.26325	45.08045	43.78425	56.88633
17412871	Nudt2	nudix (nucleoside diphosphate linked moiety X)-type motif 2	25.31762	20.22966	19.91578	26.36076	21.67845	24.90287
17511825	Nudt21	nudix (nucleoside diphosphate linked moiety X)-type motif 21	41.04146	45.53456	40.29754	40.85753	57.29431	45.33961
17362216	Nudt22	nudix (nucleoside diphosphate linked moiety X)-type motif 22	18.70199	13.35604	23.4046	18.03978	21.58848	21.30736
17244514	Nudt4	nudix (nucleoside diphosphate linked moiety X)-type motif 4	496.865	551.7152	530.3901	498.5761	576.014	601.1495
17366542	Nudt5	nudix (nucleoside diphosphate linked moiety X)-type motif 5	16.19437	19.34099	18.47138	14.47067	12.19806	17.75902
17404911	Nudt6	nudix (nucleoside diphosphate linked moiety X)-type motif 6	13.16705	10.54685	12.29151	12.06763	15.02913	11.75359
17505851	Nudt7	nudix (nucleoside diphosphate linked moiety X)-type motif 7	238.177	106.3739	97.36558	198.2092	113.3319	107.6444
17356061	Nudt8	nudix (nucleoside diphosphate linked moiety X)-type motif 8	19.32478	18.11572	16.67726	24.48659	17.77601	18.20951
17439769	Nudt9	nudix (nucleoside diphosphate linked moiety X)-type motif 9	34.60863	41.55442	39.54628	40.41783	31.0952	39.78217
17342750	Nudt3	nudix (nucleotide diphosphate linked moiety X)-type motif 3	40.53988	43.12941	46.49456	45.86749	41.79041	46.49456
17229433	Nuf2	NUF2, NDC80 kinetochore complex component, homolog (S. cerevisiae)	4.603273	4.603273	4.69762	5.270902	4.166425	4.603273
17282435	Numb	numb gene homolog (Drosophila)	51.71819	61.80664	59.25315	46.7531	37.2379	47.74938
17475564	Numbl	numb-like	5.211144	9.341084	7.118583	7.013429	6.545303	10.29855
17337269	Nrm	nurim (nuclear envelope membrane protein)	11.43251	14.05323	9.899187	11.51203	8.748344	13.68003
17287272	Nutm2	NUT family member 2	3.202836	2.908711	3.08609	2.656506	3.136227	4.249137
17389315	Nutm1	NUT midline carcinoma, family member 1	7.218905	9.190988	8.571217	8.631253	6.546846	6.943196
17533440	Nyx	nyctalopin	5.384976	5.617429	5.617429	5.264549	6.123812	5.617429
17300686	Nynrin	NYN domain and retroviral integrase containing	6.288866	6.851691	10.3177	6.241704	7.024863	7.227416
17484271	Mgmt	O-6-methylguanine-DNA methyltransferase	70.19372	68.42777	67.56106	78.70372	59.04119	68.29131
17338434	Oard1	O-acyl-ADP-ribose deacylase 1	28.89504	31.13122	32.61695	37.63805	33.27346	33.33024
17351364	Oacyl	O-acyltransferase like	2.949274	2.995735	3.254396	2.820889	4.381485	3.286268
17526102	Oaf	OAF homolog (Drosophila)	293.5454	304.6798	310.4356	333.962	329.4647	376.6263
17386481	Ola1	Obg-like ATPase 1	47.0323	40.38927	36.85427	37.42421	39.62365	38.26226
17263223	Obscn	obscurin, cytoskeletal calmodulin and titin-interacting RhoGEF	5.510411	5.510411	5.55301	5.510411	5.131107	5.125526
17224629	Obsl1	obscurin-like 1	6.239282	9.488237	9.488237	10.771	9.488237	9.488237
17295670	Ocln	occludin	48.52277	46.28234	46.28234	52.24786	64.27162	55.16877
17502157	Ocel1	occludin/ELL domain containing 1	23.24035	17.65971	16.46385	20.61453	20.91042	16.97936
17438101	Ociad1	OCIA domain containing 1	204.2681	221.8453	215.3411	204.1676	192.6395	206.7782
17448821	Ociad2	OCIA domain containing 2	71.7611	58.21544	39.19207	58.21544	62.61429	54.75049
17534497	Ocrl	oculocerebrorenal syndrome of Lowe	12.76366	10.7911	13.32192	13.1005	11.19847	9.952093
17478698	Oca2	oculocutaneous albinism II	4.97446	3.8745	5.269149	3.87767	5.232245	5.652269
17274021	Osr1	odd-skipped related 1 (Drosophila)	10.22607	11.83666	9.941613	9.941613	9.310251	12.08965
17310982	Osr2	odd-skipped related 2	4.80338	5.060921	5.123856	4.600147	5.266703	5.549393
17438684	Odam	odontogenic, ameloblast asssociated	2.490594	2.458741	2.611358	2.69856	2.724308	2.633388
17368163	Obp2a	odorant binding protein 2A	28.24395	7.665979	4.013804	16.23595	11.56746	8.928142
17368175	Obp2b	odorant binding protein 2B	5.163288	4.651545	5.292649	5.287259	5.537059	6.261985
17542933	Obp1a	odorant binding protein IA	3.530885	2.67402	2.793396	2.346873	2.103629	2.953407
17536052	Obp1b	odorant binding protein IB	3.776134	2.543544	2.53225	2.966394	2.758435	2.562532
17381204	Olah	oleoyl-ACP hydrolase	2.953407	2.586803	2.953407	2.953407	4.270278	3.258211
17368646	Olfm1	olfactomedin 1	13.60146	14.04622	13.80999	14.63986	12.17331	13.54971
17524441	Olfm2	olfactomedin 2	4.653286	5.997245	4.653286	4.213327	3.729313	5.353009
17401970	Olfm3	olfactomedin 3	8.844496	7.075146	7.279756	6.692012	9.363254	6.603816
17302279	Olfm4	olfactomedin 4	3.254148	3.779433	3.281215	3.513576	5.067872	3.414466
17494307	Olfm5	olfactomedin 5	4.763824	4.763824	6.298988	4.763824	4.763824	4.296048
17481550	Olfml1	olfactomedin-like 1	17.8818	21.82788	21.21011	21.21011	19.22564	19.06652
17370487	Olfml2a	olfactomedin-like 2A	6.882535	6.834473	7.772615	6.518229	7.726634	7.73183
17219185	Olfml2b	olfactomedin-like 2B	16.64261	17.48614	19.56469	18.17344	14.04213	17.5952
17408648	Olfml3	olfactomedin-like 3	14.24921	14.52997	14.31296	15.94542	13.3615	17.02379
17493547	Omp	olfactory marker protein	7.404642	8.855293	8.372451	10.17806	11.26707	10.35532
17265761	Olfr1	olfactory receptor 1	2.65119	2.331196	2.12908	2.31474	2.458673	2.072138
17248961	Olfr10	olfactory receptor 10	2.721647	2.17359	2.531145	2.98078	4.163163	3.198599
17387610	Olfr1000	olfactory receptor 1000	4.881045	5.914844	4.358495	4.230183	4.142064	4.351703
17387612	Olfr1002	olfactory receptor 1002	2.418043	2.07346	2.099084	2.224082	2.305069	2.497301
17387617	Olfr1006	olfactory receptor 1006	3.045354	2.040171	2.534463	2.61614	2.242488	3.259137
17372751	Olfr1008	olfactory receptor 1008	2.953407	2.592744	2.821535	2.879304	6.184084	2.953407
17372753	Olfr1009	olfactory receptor 1009	4.231502	3.939029	4.140629	4.822195	4.352065	4.37105

17344862	Olfr101	olfactory receptor 101	2.49931	2.314772	2.455676	2.251427	2.314772	2.19328
17372755	Olfr1010	olfactory receptor 1010	3.285584	3.339703	3.009972	4.120045	3.811503	3.321719
17387620	Olfr1012	olfactory receptor 1012	2.186722	2.65119	2.62799	2.09423	2.38644	2.809941
17372757	Olfr1013	olfactory receptor 1013	2.480894	2.631381	2.743219	2.5355	2.606963	2.831762
17372759	Olfr1014	olfactory receptor 1014	3.310846	3.049896	2.785642	2.450897	3.255457	3.167644
17372761	Olfr1015	olfactory receptor 1015	4.680491	4.908595	4.15973	3.696526	5.120306	3.81182
17387622	Olfr1016	olfactory receptor 1016	2.951033	2.926406	3.065962	3.019229	3.415957	3.204568
17372765	Olfr1018	olfactory receptor 1018	3.427722	2.420844	2.403739	2.932559	2.926102	2.485765
17387624	Olfr1019	olfactory receptor 1019	2.728598	3.296808	2.220016	2.333507	2.107379	2.099288
17344866	Olfr102	olfactory receptor 102	2.67402	2.315767	2.271728	2.466388	1.990747	2.301317
17372767	Olfr1020	olfactory receptor 1020	2.162035	2.047481	2.181711	2.812202	2.162035	2.162035
17372771	Olfr1022	olfactory receptor 1022	3.95646	3.104692	3.951937	3.156553	2.957135	3.918931
17372773	Olfr1023	olfactory receptor 1023	2.544902	2.810818	2.481049	2.327514	2.257193	2.481049
17387628	Olfr1024	olfactory receptor 1024	4.096836	4.517047	3.873372	4.701822	4.325479	3.44824
17372775	Olfr1025-ps1	olfactory receptor 1025, pseudogene 1	2.372552	2.503714	3.218927	3.427247	3.086582	4.622073
17372777	Olfr1026	olfactory receptor 1026	2.625478	2.420844	2.67402	2.578103	2.338462	2.565304
17372779	Olfr1028	olfactory receptor 1028	2.311597	2.465072	2.155227	2.25366	3.540222	2.705303
17372781	Olfr1029	olfactory receptor 1029	2.798338	2.196581	1.924402	2.752247	2.410179	2.100816
17344870	Olfr103	olfactory receptor 103	2.700825	2.812182	2.185495	2.360201	2.584025	2.430107
17372785	Olfr1030	olfactory receptor 1030	1.980425	2.25238	2.313713	2.19544	2.255035	1.908157
17372788	Olfr1031	olfactory receptor 1031	3.402983	2.551013	2.140296	2.719271	2.188921	2.719271
17372790	Olfr1032	olfactory receptor 1032	2.391606	1.959615	1.886144	2.099317	2.081596	2.170263
17372792	Olfr1033	olfactory receptor 1033	6.608624	5.89814	4.464783	6.024243	5.583927	6.070562
17372797	Olfr1034	olfactory receptor 1034	41.68221	33.01661	36.41296	36.73797	49.24789	39.55615
17372801	Olfr1036	olfactory receptor 1036	2.134991	2.171964	2.365557	2.234528	2.384324	1.865419
17387630	Olfr1037	olfactory receptor 1037	2.342729	2.404822	2.204406	2.024422	2.70624	1.960104
17372805	Olfr1038-ps	olfactory receptor 1038, pseudogene	2.953407	3.102906	3.281397	2.953407	2.953407	3.191025
17387634	Olfr1039	olfactory receptor 1039	4.622041	7.727544	4.158347	4.844138	5.358466	5.243536
17387636	Olfr1040	olfactory receptor 1040	3.375427	2.606909	2.702847	2.297556	2.712927	2.425849
17387638	Olfr1042	olfactory receptor 1042	1.914331	2.62799	1.95937	2.336226	2.177043	2.07612
17387642	Olfr1043	olfactory receptor 1043	2.572605	2.474214	3.036985	2.953407	2.276267	2.841396
17387644	Olfr1044	olfactory receptor 1044	2.270642	3.306328	2.481049	2.606909	3.220847	2.516049
17387648	Olfr1045	olfactory receptor 1045	2.82635	2.885037	2.67402	2.18883	2.771563	2.361429
17387650	Olfr1046	olfactory receptor 1046	3.311764	5.780603	4.501731	3.737024	3.332581	3.559217
17387652	Olfr1047	olfactory receptor 1047	6.459892	5.495954	4.086569	5.32278	4.447239	5.288249
17387654	Olfr1048	olfactory receptor 1048	2.432568	1.793279	1.898237	2.656248	2.239691	2.335093
17387656	Olfr1049	olfactory receptor 1049	2.244431	2.27813	2.36061	2.383993	3.554755	2.394327
17387658	Olfr1051	olfactory receptor 1051	2.633735	2.596845	2.209179	2.56767	2.56767	2.908933
17372808	Olfr1052	olfactory receptor 1052	3.484665	3.212508	3.071365	2.394609	2.718574	3.056456
17387660	Olfr1053	olfactory receptor 1053	2.564949	2.434035	2.432264	1.828081	2.090054	2.020959
17387662	Olfr1054	olfactory receptor 1054	2.250133	2.35873	1.974066	2.563633	2.32908	2.447519
17387664	Olfr1055	olfactory receptor 1055	2.366902	2.239971	2.382275	2.511884	4.609787	2.418278
17387666	Olfr1056	olfactory receptor 1056	2.461009	2.791035	2.622877	2.409009	2.376074	3.559661
17387670	Olfr1057	olfactory receptor 1057	5.200535	5.614665	3.703322	3.833999	3.612456	4.623803
17387672	Olfr1058	olfactory receptor 1058	2.129493	2.166806	1.886563	1.839355	2.218062	2.503124
17387674	Olfr1061	olfactory receptor 1061	2.60765	2.787149	2.64286	3.119598	2.735795	2.903554
17387676	Olfr1062	olfactory receptor 1062	3.0739	2.929247	2.768472	2.899351	2.797639	2.611919
17387678	Olfr1065	olfactory receptor 1065	2.857077	4.531096	2.531531	2.937543	2.461999	2.598412
17387681	Olfr1066	olfactory receptor 1066	1.603595	2.615887	3.009972	2.645481	3.829962	3.334961
17337566	Olfr107	olfactory receptor 107	2.414509	2.256505	2.298767	1.895423	2.295662	2.22214
17372810	Olfr1076	olfactory receptor 1076	3.758664	4.78318	3.739097	3.663138	4.44462	3.626681
17387685	Olfr1077-ps1	olfactory receptor 1077, pseudogene 1	27.78819	44.82774	38.19124	33.54254	25.13577	33.93824
17387688	Olfr1079	olfactory receptor 1079	2.741547	2.239691	2.203245	2.101108	2.490974	2.666117
17337570	Olfr108	olfactory receptor 108	2.680496	2.6144	2.939886	4.724364	3.338245	4.442648
17387692	Olfr1080	olfactory receptor 1080	2.416703	3.141209	2.937075	2.097455	2.682206	3.523282
17387694	Olfr1082	olfactory receptor 1082	2.379799	2.106576	2.241633	2.365229	2.619824	2.261876
17387697	Olfr1084	olfactory receptor 1084	2.343541	1.886733	2.164374	2.191001	1.938096	1.865006
17387700	Olfr1085	olfactory receptor 1085	4.209299	3.414584	3.796447	3.815918	4.245516	3.124987
17387702	Olfr1086	olfactory receptor 1086	2.406006	2.291703	1.965015	2.406006	2.471111	1.9358
17387704	Olfr1087	olfactory receptor 1087	2.11515	2.690641	2.10944	2.073172	1.956371	2.69929
17387706	Olfr1089	olfactory receptor 1089	2.406006	2.150601	1.957483	2.148634	1.552561	2.830938
17337574	Olfr109	olfactory receptor 109	2.950583	2.443502	2.13505	2.963598	2.782767	2.212151
17387708	Olfr1090	olfactory receptor 1090	2.162035	2.690641	1.831137	2.502829	2.458673	2.410346
17372812	Olfr1093	olfactory receptor 1093	3.253427	2.453328	2.481049	2.586839	2.633297	2.832371
17372815	Olfr1094	olfactory receptor 1094	2.682968	2.855394	2.00846	2.440573	4.205304	1.972616
17387712	Olfr1095	olfactory receptor 1095	2.28216	2.332117	2.583416	3.067918	2.710934	2.072138
17387714	Olfr1096-ps1	olfactory receptor 1096, pseudogene 1	2.441842	2.045226	1.955856	1.898013	2.089909	2.372125
17387717	Olfr1097	olfactory receptor 1097	2.596683	2.117556	2.261798	1.981784	1.994214	2.291242
17387720	Olfr1098	olfactory receptor 1098	3.32043	2.057211	2.763487	2.964852	2.236093	2.255442
17387723	Olfr1099	olfactory receptor 1099	2.406006	2.36079	2.481049	2.664787	3.187289	2.662959
17290990	Olfr11	olfactory receptor 11	3.134836	3.134836	2.80255	3.134836	2.729828	3.539977
17337576	Olfr110	olfactory receptor 110	2.784059	2.506684	3.257375	2.775504	2.645188	2.922879
17387726	Olfr1100	olfactory receptor 1100	2.481049	2.239691	3.300558	2.481049	2.375696	2.633169
17387728	Olfr1101	olfactory receptor 1101	2.367017	2.615107	1.922229	2.255364	2.292974	2.18392
17372819	Olfr1102	olfactory receptor 1102	2.039619	2.207357	2.365524	2.745331	2.36746	2.013506
17387730	Olfr1104	olfactory receptor 1104	2.882039	2.582557	2.694019	2.396003	2.462989	2.243125
17387732	Olfr1105	olfactory receptor 1105	3.048564	3.822801	3.25381	3.465924	2.953448	2.647095
17387734	Olfr1106	olfactory receptor 1106	3.467486	2.746168	2.349543	2.89911	2.791596	3.220512
17387736	Olfr1107	olfactory receptor 1107	2.429535	1.968109	1.978957	2.092837	2.520518	2.082418
17387740	Olfr1109	olfactory receptor 1109	3.116377	2.854524	1.954379	2.29021	2.865248	3.084422
17337580	Olfr111	olfactory receptor 111	2.280768	1.85441	1.722794	2.005233	3.333551	2.404205
17387744	Olfr1110	olfactory receptor 1110	2.318449	2.256239	2.253973	2.075933	2.384935	2.377491
17387748	Olfr1111	olfactory receptor 1111	2.562674	2.30261	2.953202	3.000972	2.979272	2.721251
17372823	Olfr1112	olfactory receptor 1112	2.691219	2.595208	2.147964	2.595208	2.208076	3.153579

17372825	Olfr1113	olfactory receptor 1113	2.705396	1.923295	2.060936	2.374346	2.135109	2.057482
17372828	Olfr1115	olfactory receptor 1115	2.938928	2.913958	2.887358	3.570213	3.161502	2.727955
17372831	Olfr1116	olfactory receptor 1116	2.488661	2.112396	2.102725	2.371056	2.818954	2.905628
17372833	Olfr1118	olfactory receptor 1118	1.997146	2.041161	2.255364	2.083357	2.188921	2.141958
17344881	Olfr112	olfactory receptor 112	2.387648	2.420844	2.387648	2.107262	2.235767	2.31612
17372837	Olfr1120	olfactory receptor 1120	2.273587	1.942238	1.838208	2.458145	2.111883	1.961245
17372840	Olfr1121	olfactory receptor 1121	2.173741	2.303679	2.056584	2.083357	2.458673	2.072138
17372842	Olfr1122	olfactory receptor 1122	3.792975	3.556677	4.186167	3.500789	3.202192	3.283035
17372846	Olfr1123	olfactory receptor 1123	2.155123	2.7124	1.779843	1.932663	2.120655	2.381846
17372848	Olfr1124	olfactory receptor 1124	2.869064	2.151615	2.307323	2.443214	3.386207	2.394011
17372850	Olfr1126	olfactory receptor 1126	2.406006	2.552941	2.99505	2.508596	2.45173	2.497804
17387758	Olfr1128	olfactory receptor 1128	2.222248	2.764005	3.021532	3.173929	2.922433	4.34468
17372866	Olfr1129	olfactory receptor 1129	2.676801	2.164254	2.321054	2.620968	2.743429	2.177073
17344892	Olfr113	olfactory receptor 113	3.894532	4.78434	5.28268	5.400451	4.78434	5.152955
17372870	Olfr1130	olfactory receptor 1130	2.708567	2.437868	2.276236	2.074322	2.455693	2.057796
17372874	Olfr1131	olfactory receptor 1131	5.317249	3.316221	2.460668	2.899934	2.876571	2.480584
17387760	Olfr1132	olfactory receptor 1132	3.33302	1.748575	2.174329	3.660118	2.457293	2.328144
17387762	Olfr1133	olfactory receptor 1133	2.760271	3.307795	2.135657	2.659804	2.550889	3.162422
17387764	Olfr1134	olfactory receptor 1134	1.942857	2.445874	2.667355	2.2398	2.782767	2.134784
17387768	Olfr1135	olfactory receptor 1135	2.457975	2.883998	2.632147	2.179973	1.927846	2.380822
17387770	Olfr1136	olfactory receptor 1136	2.261406	1.787149	1.907351	2.209209	2.567617	1.938956
17387774	Olfr1137	olfactory receptor 1137	2.527621	2.451237	2.451101	2.553861	2.287006	2.032619
17387776	Olfr1138	olfactory receptor 1138	2.102375	2.329467	2.832488	2.395771	1.831188	2.290575
17344896	Olfr114	olfactory receptor 114	2.616739	2.435858	2.427195	2.953407	2.848377	2.456782
17372876	Olfr1140	olfactory receptor 1140	2.67402	2.606909	2.892486	2.573586	2.606909	2.256771
17387778	Olfr1141	olfactory receptor 1141	4.007632	4.178543	2.728693	6.590464	3.689205	4.742014
17372882	Olfr1143	olfactory receptor 1143	5.017991	5.398281	6.93401	4.881146	4.098511	4.330339
17372884	Olfr1145	olfactory receptor 1145	2.376206	2.551136	2.67402	2.153152	2.458673	2.102215
17372886	Olfr1148	olfactory receptor 1148	2.953407	2.953407	2.709206	3.251623	2.953407	2.811617
17344899	Olfr115	olfactory receptor 115	2.36382	2.625769	1.968832	2.794597	2.686951	1.760494
17372888	Olfr1151	olfactory receptor 1151	4.876547	3.282056	2.572284	2.143131	3.565736	3.338245
17372890	Olfr1152	olfactory receptor 1152	4.162961	2.455097	2.237953	3.671577	3.717261	2.841869
17372892	Olfr1153	olfactory receptor 1153	2.987399	2.235829	2.120449	2.991564	2.052753	2.481049
17387780	Olfr1154	olfactory receptor 1154	2.110288	2.345247	2.667318	2.464321	2.042704	2.401091
17387782	Olfr1155	olfactory receptor 1155	3.135053	2.332505	2.39818	2.404222	2.73263	2.606909
17387784	Olfr1156	olfactory receptor 1156	2.367755	2.298209	2.078611	2.743809	2.472482	2.206653
17387788	Olfr1157	olfactory receptor 1157	3.117739	2.76343	2.67402	2.657151	3.179412	3.140404
17372894	Olfr1158	olfactory receptor 1158	4.535841	3.460331	4.20927	3.044404	2.826546	4.144132
17344905	Olfr116	olfactory receptor 116	8.12276	4.579182	5.861744	5.783048	4.022744	5.707623
17387794	Olfr1160	olfactory receptor 1160	2.008655	1.824094	1.982952	2.954246	2.034832	2.125644
17372896	Olfr1161	olfactory receptor 1161	2.519103	2.146461	2.146461	2.605807	2.188921	2.481031
17387800	Olfr1162	olfactory receptor 1162	2.503419	2.754003	3.256811	3.153863	3.130255	2.906675
17387802	Olfr1163	olfactory receptor 1163	2.550836	2.524959	2.342729	2.449318	1.887622	2.606909
17387806	Olfr1164	olfactory receptor 1164	2.162035	2.414225	2.204146	2.930892	2.701087	3.024067
17387810	Olfr1166	olfactory receptor 1166	3.366361	3.827865	2.717086	4.3492	2.755664	3.366361
17387813	Olfr1167	olfactory receptor 1167	3.369536	3.830041	3.380555	3.846003	4.378905	3.579406
17372898	Olfr1168	olfactory receptor 1168	2.929958	2.535095	2.714638	3.488266	2.953734	3.569619
17344907	Olfr117	olfactory receptor 117	2.150169	2.228511	2.198516	2.592547	2.355184	2.345442
17387817	Olfr1170	olfactory receptor 1170	2.3715	2.309611	2.218355	2.759544	2.273792	1.951374
17387822	Olfr1173	olfactory receptor 1173	2.288036	2.129877	2.165019	2.165019	3.096332	2.075156
17387824	Olfr1174-ps	olfactory receptor 1174, pseudogene	6.84125	7.296477	8.560411	8.51685	5.88499	8.968272
17387826	Olfr1175-ps	olfactory receptor 1175, pseudogene	3.493057	3.010827	3.016112	2.584544	3.23288	2.639327
17372900	Olfr1176	olfactory receptor 1176	2.67402	2.274611	2.675187	2.656598	2.916342	2.287957
17372902	Olfr1178	olfactory receptor 1178	4.535589	3.045185	2.818114	2.80634	3.833441	2.931522
17387829	Olfr1179	olfactory receptor 1179	3.67843	3.024423	2.889721	2.889721	3.504673	2.803171
17337583	Olfr118	olfactory receptor 118	3.365755	2.477148	2.56824	2.418781	2.559177	2.238046
17387831	Olfr1180	olfactory receptor 1180	2.263178	2.713096	2.492321	2.163159	2.363852	1.822072
17387835	Olfr1181	olfactory receptor 1181	1.866946	2.413907	2.67402	2.047027	2.67402	3.19953
17387837	Olfr1182	olfactory receptor 1182	2.548362	2.354744	1.868551	2.165049	2.566798	2.285991
17372904	Olfr1183	olfactory receptor 1183	2.794965	3.173357	3.88208	3.224667	3.171906	3.318658
17372906	Olfr1184	olfactory receptor 1184	1.940368	2.659804	2.92596	2.199644	2.836339	2.659804
17372908	Olfr1186	olfactory receptor 1186	1.979245	1.791142	1.928702	1.736317	2.147949	2.146283
17387841	Olfr1187-ps1	olfactory receptor 1187, pseudogene 1 [Source:MGI Symbol;Acc:MGI:3031021]	2.646123	2.263727	2.388442	2.381251	1.973081	2.684717
17372912	Olfr1188	olfactory receptor 1188	2.069353	2.25541	2.898025	2.48873	2.665655	2.265501
17372914	Olfr1189	olfactory receptor 1189	3.893479	3.21253	2.645169	2.538718	3.610729	2.252707
17337585	Olfr119	olfactory receptor 119	1.932074	2.239691	2.020147	2.396917	1.979368	2.38401
17372916	Olfr1192-ps1	olfactory receptor 1192, pseudogene 1	2.258821	2.597187	3.137358	2.355837	2.07277	2.958672
17372919	Olfr1193	olfactory receptor 1193	5.338965	4.801649	4.881417	5.536253	6.103641	5.693595
17387844	Olfr1195	olfactory receptor 1195	2.440472	2.440472	3.171159	2.621023	1.642405	2.606909
17387846	Olfr1196	olfactory receptor 1196	2.770699	3.86155	3.094615	8.936439	4.624188	4.562422
17387848	Olfr1197	olfactory receptor 1197	4.117161	2.758053	3.247884	2.72512	3.067408	3.211372
17387850	Olfr1198	olfactory receptor 1198	2.16205	2.661464	1.980425	2.304206	1.735739	2.334057
17387852	Olfr1199	olfactory receptor 1199	3.357599	3.335655	2.981565	3.016405	3.234539	2.669482
17215817	Olfr12	olfactory receptor 12	6.171709	6.086488	7.827763	6.171709	5.188329	6.171709
17337589	Olfr120	olfactory receptor 120	4.7058	2.906111	2.611919	3.141296	2.743752	2.901904
17387856	Olfr1200	olfactory receptor 1200	2.367772	2.349412	2.716991	2.318803	2.812884	2.496263
17372921	Olfr1201	olfactory receptor 1201	2.685238	2.138901	1.950332	2.196993	2.661298	2.519295
17372923	Olfr1202	olfactory receptor 1202	2.264904	1.787769	1.916402	1.68817	1.955422	1.904246
17372927	Olfr1204	olfactory receptor 1204	2.311982	2.59314	2.080038	2.55356	3.224197	2.40936
17372925	Olfr1205	olfactory receptor 1205	2.944025	3.03926	3.77891	2.825155	3.142276	4.655061
17372929	Olfr1206	olfactory receptor 1206	2.805368	2.94015	2.270421	2.378695	2.477217	2.839427
17387858	Olfr1208	olfactory receptor 1208	2.572748	2.543368	2.53125	3.03808	2.28615	2.398014
17387862	Olfr1209	olfactory receptor 1209	3.294159	2.38396	2.03183	2.616757	2.089214	1.800565
17337591	Olfr121	olfactory receptor 121	2.264339	2.23724	1.932703	2.181182	2.877688	1.915804

17387864	Olfr1211	olfactory receptor 1211	1.980068	2.724988	2.725234	2.348338	4.792904	3.729055
17372931	Olfr1212	olfactory receptor 1212	2.27794	2.869163	2.371665	2.083357	2.37058	2.181076
17387870	Olfr1213	olfactory receptor 1213	1.959778	2.084917	2.185359	1.927499	2.451339	2.072138
17387876	Olfr1214	olfactory receptor 1214	2.870436	2.775524	2.514288	2.497007	2.935955	2.982909
17387878	Olfr1215	olfactory receptor 1215	3.062691	3.230035	3.062691	3.421242	3.10495	3.062691
17387880	Olfr1216	olfactory receptor 1216	2.137849	2.060051	2.282034	2.060051	1.990388	2.402389
17387882	Olfr1217	olfactory receptor 1217	2.10411	2.425765	2.045367	1.846675	2.259808	1.851854
17387886	Olfr1218	olfactory receptor 1218	2.772428	2.67402	2.772428	2.214667	4.236403	3.135032
17387888	Olfr1219	olfactory receptor 1219	8.89787	7.487634	6.141708	10.92181	18.21191	8.89787
17337595	Olfr122	olfactory receptor 122	2.872287	2.434946	4.004688	2.957504	2.528514	3.554853
17387890	Olfr1220	olfactory receptor 1220	5.336338	6.094256	5.894911	4.512791	3.806566	4.20927
17387894	Olfr1221	olfactory receptor 1221	2.551614	2.239691	2.092953	2.304206	2.239691	2.681481
17387900	Olfr1222	olfactory receptor 1222	3.116961	2.843386	2.593014	2.997438	3.063498	3.496739
17387902	Olfr1223	olfactory receptor 1223	2.475809	2.785546	2.501858	2.121317	2.662866	3.190782
17387906	Olfr1224-ps1	olfactory receptor 1224, pseudogene 1	6.979676	3.868784	3.966042	3.887035	5.120306	5.314743
17387908	Olfr1225	olfactory receptor 1225	2.308651	1.82038	2.469194	2.135864	2.782767	2.656469
17387912	Olfr1226	olfactory receptor 1226	10.60814	17.44752	10.96049	10.82466	6.423858	15.43764
17387914	Olfr1228	olfactory receptor 1228	2.579462	1.710253	2.28512	2.469947	2.072885	2.188724
17387918	Olfr1229	olfactory receptor 1229	2.371287	2.342908	2.311838	2.81501	2.708511	2.387582
17337599	Olfr123	olfactory receptor 123	3.525701	3.097448	4.050107	3.668067	3.467894	4.024836
17387926	Olfr1230	olfactory receptor 1230	2.215681	2.774408	2.467791	2.757422	2.945597	3.210082
17387928	Olfr1231	olfactory receptor 1231	2.680347	2.365492	2.659325	2.246984	2.350227	2.930283
17387930	Olfr1232	olfactory receptor 1232	3.070641	2.508196	2.637169	2.723931	2.733766	2.637169
17387932	Olfr1233	olfactory receptor 1233	3.395396	2.67695	2.67695	2.606909	2.749673	2.180668
17387936	Olfr1234	olfactory receptor 1234	2.257741	2.215389	2.597709	2.352427	2.256427	2.584956
17387940	Olfr1238	olfactory receptor 1238	2.775158	3.451707	4.379603	2.676746	2.549704	2.781687
17387942	Olfr1239	olfactory receptor 1239	2.162035	2.953407	2.082837	2.801035	2.295264	2.953407
17337604	Olfr124	olfactory receptor 124	2.999745	2.420844	2.317662	2.389403	2.645444	2.229546
17387944	Olfr1240	olfactory receptor 1240	2.072842	2.220785	1.897711	2.261876	1.94209	2.024815
17387948	Olfr1241	olfactory receptor 1241	2.585691	2.41739	2.226241	2.934735	2.639162	2.639162
17387950	Olfr1242	olfactory receptor 1242	2.783114	2.733046	3.248064	3.032652	3.433024	2.783268
17387956	Olfr1243	olfactory receptor 1243	2.096452	2.447129	2.110127	2.155003	2.886237	2.502847
17387961	Olfr1245	olfactory receptor 1245	2.564718	2.239241	1.863235	2.36682	2.220785	2.162305
17387965	Olfr1246	olfactory receptor 1246	3.49296	2.409527	2.370432	2.616104	3.155853	2.452087
17387969	Olfr1247	olfactory receptor 1247	2.280768	2.308571	2.441842	2.328838	2.356719	2.489628
17387975	Olfr1248	olfactory receptor 1248	2.271917	2.271917	2.72822	2.189225	2.763296	2.005372
17387979	Olfr1249	olfactory receptor 1249	3.365965	2.480309	2.452155	3.648921	2.953407	2.953407
17337608	Olfr125	olfactory receptor 125	2.195075	2.133793	1.98985	2.283663	2.275337	2.032309
17387981	Olfr1250	olfactory receptor 1250	2.07507	2.143086	2.192231	2.36682	2.108709	2.249696
17387987	Olfr1251	olfactory receptor 1251	3.225717	2.289797	2.601259	2.433512	2.28829	2.567208
17387991	Olfr1252	olfactory receptor 1252	2.637151	2.767225	2.670297	2.586121	2.640406	2.96251
17387995	Olfr1253	olfactory receptor 1253	1.881796	1.898013	2.045509	1.898013	2.056142	2.953407
17387997	Olfr1254	olfactory receptor 1254	2.420508	2.306332	2.022893	2.306332	2.450608	3.10452
17372934	Olfr1255	olfactory receptor 1255	2.162035	1.768223	1.768223	2.111883	2.049668	1.700985
17388001	Olfr1256	olfactory receptor 1256	2.860783	1.940825	2.285991	2.335498	2.34811	2.285991
17372936	Olfr1257	olfactory receptor 1257	1.969241	1.906055	1.79852	2.2959	2.805893	2.297413
17372942	Olfr1258	olfactory receptor 1258	2.379156	2.298512	2.528006	2.535324	3.095023	2.241058
17388008	Olfr1259	olfactory receptor 1259	2.780164	2.55627	2.818524	2.611629	2.311293	2.954717
17337612	Olfr126	olfactory receptor 126	2.398363	1.995693	2.365524	2.167752	2.671	2.140711
17372949	Olfr1260	olfactory receptor 1260	3.374023	3.225494	2.796825	2.498877	3.065006	2.826154
17372954	Olfr1261	olfactory receptor 1261	3.495794	3.66065	3.569223	3.684145	3.711185	3.765105
17372959	Olfr1262	olfactory receptor 1262	2.571856	2.953407	2.740274	3.936873	3.00066	2.465004
17372966	Olfr1263	olfactory receptor 1263	2.102579	2.282144	1.790534	1.81329	2.937828	1.746093
17388010	Olfr1264	olfactory receptor 1264	2.885737	3.100863	3.153032	3.176042	3.919746	3.253359
17372970	Olfr1265	olfactory receptor 1265	3.652059	2.145762	3.281601	2.422052	2.367805	4.127219
17388016	Olfr1269	olfactory receptor 1269	19.15262	20.65086	21.13511	19.80784	23.89439	19.41083
17337614	Olfr127	olfactory receptor 127	6.304011	5.390279	6.935116	9.670095	11.33757	6.94363
17388026	Olfr1270	olfactory receptor 1270	2.67402	2.578621	2.481049	2.849858	2.578621	2.391142
17388035	Olfr1271	olfactory receptor 1271	3.161502	2.793589	2.171602	4.69443	2.924338	3.305595
17388037	Olfr1272	olfactory receptor 1272	4.01667	4.672841	4.352065	4.352065	4.645616	4.739845
17388039	Olfr1273-ps	olfactory receptor 1273, pseudogene	2.724384	1.966432	1.957823	1.953783	2.949867	2.444044
17372977	Olfr1274-ps	olfactory receptor 1274, pseudogene	1.799679	2.083357	2.224899	2.044205	2.083357	2.072138
17389256	Olfr1275	olfactory receptor 1275	2.112088	3.246848	2.638449	2.705059	2.991149	2.694355
17374167	Olfr1276	olfactory receptor 1276	2.219816	1.976585	2.183269	2.282018	2.167512	2.226349
17389266	Olfr1277	olfactory receptor 1277	2.16894	1.969269	2.448282	2.17955	2.422976	1.89264
17374170	Olfr1278	olfactory receptor 1278	2.115077	2.893729	2.250101	2.128814	2.266003	1.961748
17374172	Olfr1279	olfactory receptor 1279	2.774158	2.169947	3.132382	2.455744	2.731134	3.611505
17337618	Olfr128	olfactory receptor 128	2.369331	2.511971	2.457906	2.677878	2.57273	4.106132
17374174	Olfr1280	olfactory receptor 1280	2.22251	1.88757	2.07096	2.179806	2.485937	2.260951
17374178	Olfr1281	olfactory receptor 1281	2.700881	2.301174	2.256458	1.745633	2.167707	3.276896
17389269	Olfr1282	olfactory receptor 1282	2.832449	2.737692	2.85999	3.329072	2.420223	1.740799
17374180	Olfr1283	olfactory receptor 1283	2.140296	1.920537	1.839865	1.882383	3.071216	1.979821
17374184	Olfr1284	olfactory receptor 1284	2.406006	2.65176	2.377788	2.11408	2.332052	2.097368
17374188	Olfr1285	olfactory receptor 1285	2.347703	1.910341	1.617662	1.877145	3.231401	1.995071
17389271	Olfr1286	olfactory receptor 1286	2.596935	3.558354	3.336719	3.409901	3.508198	4.155062
17374190	Olfr1287	olfactory receptor 1287	2.505259	2.637626	3.339703	3.324391	2.539827	2.637626
17374192	Olfr1288	olfactory receptor 1288	2.71069	2.631819	2.61469	2.754862	2.8817	2.299372
17374194	Olfr1289	olfactory receptor 1289	3.117933	2.636091	3.544249	2.884937	2.609277	4.395173
17344911	Olfr129	olfactory receptor 129	4.048311	3.246871	2.995174	3.369349	3.390364	3.246871
17389273	Olfr1290	olfactory receptor 1290	4.200439	2.554941	3.24098	2.638833	3.959669	3.162685
17389275	Olfr1294	olfactory receptor 1294	2.815751	3.699012	3.231245	2.683322	2.660468	3.29183
17389277	Olfr1295	olfactory receptor 1295	2.752247	2.049057	2.260372	2.485472	2.487885	2.185889
17389281	Olfr1297	olfactory receptor 1297	3.123124	2.67402	2.135361	2.266082	2.899552	2.729298
17389283	Olfr1298	olfactory receptor 1298	12.30131	10.52924	9.361307	9.293809	8.541444	9.922956

17374198	Olfr1299	olfactory receptor 1299	6.88578	8.863153	6.123897	7.602005	6.131967	8.383949
17458006	Olfr13	olfactory receptor 13	3.00168	2.67402	4.09482	2.894592	3.263862	2.894592
17337622	Olfr130	olfactory receptor 130	5.197616	10.48787	8.127153	8.98638	8.672152	8.120621
17374201	Olfr1300-ps1	olfactory receptor 1300, pseudogene 1	6.034022	5.756413	4.668568	5.398356	5.943365	6.037034
17374206	Olfr1301	olfactory receptor 1301	2.646563	3.331657	3.009972	2.626879	2.475757	2.867891
17374208	Olfr1302	olfactory receptor 1302	3.656517	3.161283	2.617864	2.481496	3.538309	2.953796
17389285	Olfr1303	olfactory receptor 1303	4.681043	4.681043	4.742902	5.118567	4.681043	4.949422
17389291	Olfr1305	olfactory receptor 1305	2.525869	2.386192	2.420844	3.176328	2.301126	2.996981
17389293	Olfr1306	olfactory receptor 1306	2.000638	1.759262	2.058167	1.875064	2.782767	2.058167
17389295	Olfr1307	olfactory receptor 1307	2.808423	1.890215	2.431674	2.431674	2.092939	2.441182
17389297	Olfr1308	olfactory receptor 1308	3.44017	2.216326	2.880981	2.650877	2.714149	2.962736
17389299	Olfr1309	olfactory receptor 1309	2.638193	2.365393	2.0291	2.523454	3.263862	2.304446
17344917	Olfr131	olfactory receptor 131	2.671778	3.104756	2.861933	2.752476	2.898427	2.98742
17389301	Olfr1310	olfactory receptor 1310	4.01461	2.54769	2.891905	2.049582	2.915069	2.285991
17389305	Olfr1311	olfactory receptor 1311	3.065729	3.025388	3.140425	3.038691	3.164593	3.159902
17389307	Olfr1312	olfactory receptor 1312	2.67402	2.279157	2.332117	2.332117	3.525554	2.604001
17389309	Olfr1313	olfactory receptor 1313	3.318796	2.676504	2.023594	4.20927	2.438358	2.194436
17389311	Olfr1314	olfactory receptor 1314	2.620787	2.237953	1.782584	2.625642	3.009263	1.877015
17389313	Olfr1316	olfactory receptor 1316	2.578818	3.104756	3.116464	2.630287	2.935386	3.286678
17374212	Olfr1317	olfactory receptor 1317	1.829158	1.815126	2.700525	2.282999	1.884942	2.613494
17374214	Olfr1318	olfactory receptor 1318	2.063266	2.135243	2.045509	2.054361	3.206412	2.427666
17344923	Olfr132	olfactory receptor 132	2.67402	2.442452	2.6146	3.257872	3.072621	2.67402
17534667	Olfr1320	olfactory receptor 1320	3.227059	2.903655	3.009972	2.880781	3.009972	3.143453
17541525	Olfr1321	olfactory receptor 1321	2.690828	3.012936	3.468015	3.068301	2.724308	2.606909
17541559	Olfr1322	olfactory receptor 1322	3.120917	2.953407	2.953407	2.953407	2.953407	2.937523
17541561	Olfr1323	olfactory receptor 1323	3.062691	2.943944	2.943944	2.647242	3.308781	3.11547
17541563	Olfr1324	olfactory receptor 1324	2.332117	1.996786	2.278288	2.577246	2.332117	2.233088
17535898	Olfr1325	olfactory receptor 1325	2.128135	2.493151	2.412468	3.010306	2.802258	2.27039
17542716	Olfr1326-ps1	olfactory receptor 1326, pseudogene 1	5.851636	5.871626	4.62656	5.9901	4.322033	8.006657
17429290	Olfr1328	olfactory receptor 1328	2.406006	2.328063	2.205247	2.143874	2.967442	2.552622
17429288	Olfr1329	olfactory receptor 1329	2.421834	2.821515	3.227238	3.420222	3.77493	4.742672
17337625	Olfr133	olfactory receptor 133	2.420844	1.960416	2.242441	2.971621	2.56207	2.673779
17417824	Olfr1330	olfactory receptor 1330	4.413643	3.119922	2.621677	2.30558	2.744532	2.352199
17417821	Olfr1331	olfactory receptor 1331	4.681043	4.612823	3.434333	7.935274	3.787485	4.282847
17429285	Olfr1333	olfactory receptor 1333	3.735005	3.893884	4.103543	5.047503	3.075626	3.905995
17429281	Olfr1335	olfactory receptor 1335	2.897041	2.897282	2.696803	3.328933	3.350694	2.919133
17473629	Olfr1336	olfactory receptor 1336	3.769727	5.710076	4.056091	3.844271	3.767611	3.536396
17429279	Olfr1337	olfactory receptor 1337	14.84608	16.09064	28.90185	20.63827	18.24223	13.99549
17429277	Olfr1338	olfactory receptor 1338	3.792187	2.012083	3.009972	2.36079	2.461419	3.28645
17417819	Olfr1339	olfactory receptor 1339	3.062691	4.641464	2.97331	3.723786	2.885717	4.030615
17337631	Olfr134	olfactory receptor 134	2.736421	2.579122	1.950738	2.748777	1.799006	2.477217
17417817	Olfr1340	olfactory receptor 1340	2.621405	2.953407	2.926163	2.544972	2.458673	4.056513
17417814	Olfr1341	olfactory receptor 1341	3.251127	2.852308	3.014106	2.971806	3.460931	3.331934
17429274	Olfr1342	olfactory receptor 1342	2.67402	2.489179	2.533146	3.090821	2.595244	2.46957
17473625	Olfr1344	olfactory receptor 1344	6.992896	5.135946	5.107864	5.721724	5.344778	6.129503
17473631	Olfr1346	olfactory receptor 1346	3.174523	4.113681	3.643866	3.674913	4.113681	4.113681
17486092	Olfr1347	olfactory receptor 1347	2.707723	2.97793	2.717104	2.521182	2.46403	2.752476
17486094	Olfr1348	olfactory receptor 1348	10.46609	13.29609	10.81019	9.014391	7.060009	7.821254
17486096	Olfr1349	olfactory receptor 1349	4.633235	2.325482	2.739723	2.692171	2.802861	3.488749
17337633	Olfr135	olfactory receptor 135	2.09468	1.962605	1.839075	1.713433	2.812026	2.230365
17473635	Olfr1350	olfactory receptor 1350	5.338965	7.709461	6.34412	5.92075	6.300996	5.792234
17234859	Olfr1351	olfactory receptor 1351	2.930994	2.953407	2.829349	2.606909	2.437513	2.606909
17234856	Olfr1352	olfactory receptor 1352	1.993315	2.812884	3.461242	2.093606	4.114223	2.895415
17234852	Olfr1353	olfactory receptor 1353	2.607866	2.239691	2.808014	2.607866	2.547178	2.914483
17234848	Olfr1354	olfactory receptor 1354	2.319317	3.488749	2.951565	2.60346	3.578314	2.857691
17234845	Olfr1355	olfactory receptor 1355	5.366271	3.352041	4.173969	2.621677	4.230476	4.867632
17242568	Olfr1356	olfactory receptor 1356	3.742572	2.77412	2.546967	4.092748	3.001721	4.238959
17242545	Olfr1357	olfactory receptor 1357	4.806278	4.806278	4.728033	4.961409	5.453831	4.20927
17234809	Olfr1358	olfactory receptor 1358	3.108848	3.108848	2.611611	3.16637	3.108848	3.149319
17285674	Olfr1359	olfactory receptor 1359	7.550332	6.150655	4.20927	5.133775	5.120306	6.209297
17337643	Olfr136	olfactory receptor 136	2.06318	2.249524	1.908501	2.031535	2.487609	2.033578
17290994	Olfr1360	olfactory receptor 1360	28.17747	37.13635	35.39197	43.41916	38.76331	38.47288
17290992	Olfr1361	olfactory receptor 1361	5.633143	6.992896	6.508658	6.072329	6.986113	6.460832
17290987	Olfr1362	olfactory receptor 1362	4.620663	7.826298	5.48933	4.579531	5.140718	5.48933
17290985	Olfr1364	olfactory receptor 1364	2.65623	3.135401	2.690175	2.815576	2.188921	2.752648
17290980	Olfr1366	olfactory receptor 1366	2.474419	2.462579	2.309339	2.666838	2.782767	3.274921
17285641	Olfr1367	olfactory receptor 1367	5.050863	3.71417	4.130052	5.107333	4.975978	4.681043
17290906	Olfr1368	olfactory receptor 1368	2.606909	2.700563	2.669556	2.345686	2.695345	2.75639
17285614	Olfr1369-ps1	olfactory receptor 1369, pseudogene 1	2.634008	2.226025	1.977435	2.076782	3.367645	2.094448
17344925	Olfr137	olfactory receptor 137	3.350578	2.913655	3.373158	2.52869	2.695812	3.149581
17290904	Olfr1370	olfactory receptor 1370	5.799667	4.525383	5.932952	5.520466	5.558825	5.56106
17262598	Olfr1371	olfactory receptor 1371	6.817298	6.992896	7.168742	6.992896	6.643069	8.847562
17262596	Olfr1372-ps1	olfactory receptor 1372, pseudogene 1	2.816239	2.441995	2.593661	2.75322	2.916706	2.698598
17262594	Olfr1373	olfactory receptor 1373	16.10102	21.75522	21.73653	25.36927	27.26182	24.54403
17249260	Olfr1375	olfactory receptor 1375	3.078185	3.263862	4.20927	2.830074	3.263862	4.036011
17249252	Olfr1377	olfactory receptor 1377	2.987006	3.225807	3.042337	4.141921	2.368166	3.484279
17249250	Olfr1378	olfactory receptor 1378	2.90863	2.846304	2.462118	2.840412	2.871491	2.848456
17337637	Olfr138	olfactory receptor 138	2.103395	2.020049	1.900949	2.103395	2.188921	2.22399
17248990	Olfr1380	olfactory receptor 1380	4.00233	4.13604	3.87939	4.403498	3.878557	4.753236
17248988	Olfr1381	olfactory receptor 1381	6.772319	7.698994	7.416199	7.698994	6.927045	7.893089
17248986	Olfr1382	olfactory receptor 1382	11.09317	9.182646	9.421195	7.166258	10.77488	9.185638
17248984	Olfr1383	olfactory receptor 1383	3.629422	2.883458	4.414714	2.731323	3.285926	3.500862
17248981	Olfr1384	olfactory receptor 1384	2.535482	2.696803	2.120964	3.04941	3.263862	2.67402
17248979	Olfr1385	olfactory receptor 1385	2.622386	2.276394	2.19815	1.81888	2.927928	3.303877

17248975	Olfr1386	olfactory receptor 1386	2.698261	2.585691	2.270217	2.564789	2.928983	2.286594
17248973	Olfr1387	olfactory receptor 1387	2.332311	2.420844	2.492701	2.420844	2.364492	2.452359
17248971	Olfr1388	olfactory receptor 1388	3.6255	6.828601	3.854276	6.093369	5.411168	4.20927
17248967	Olfr1389	olfactory receptor 1389	3.825213	5.313638	4.690494	5.659364	4.848942	5.288798
17265806	Olfr139	olfactory receptor 139	4.376022	3.784939	3.405248	3.655959	3.200195	3.991248
17248965	Olfr1390	olfactory receptor 1390	4.235611	3.456447	4.440955	3.890161	3.679373	5.56106
17248963	Olfr1391	olfactory receptor 1391	2.274312	2.883338	2.969315	1.9484	2.06921	2.018803
17248957	Olfr1392	olfactory receptor 1392	2.313024	2.214683	2.280484	2.236868	3.23353	2.25951
17248955	Olfr1393	olfactory receptor 1393	3.462322	4.267792	4.077288	4.20927	4.248018	5.16694
17248927	Olfr1394	olfactory receptor 1394	4.06755	4.245134	4.20927	3.703322	4.469799	4.886969
17248925	Olfr1395	olfactory receptor 1395	5.901575	8.287058	5.800029	6.171709	4.996265	5.986862
17262255	Olfr1396	olfactory receptor 1396	3.709102	4.213911	3.119511	3.609502	3.516353	3.957145
17388012	Olfr140	olfactory receptor 140	3.842726	3.677002	3.489185	3.293725	3.027905	4.150859
17408182	Olfr1402	olfactory receptor 1402	3.79266	2.797814	2.997688	2.765538	3.263862	2.509257
17219635	Olfr1404	olfactory receptor 1404	4.143529	2.278872	2.394576	2.569237	2.708661	2.285231
17229970	Olfr1406	olfactory receptor 1406	2.132625	1.82283	1.929263	2.082014	2.153585	1.774325
17229967	Olfr1408	olfactory receptor 1408	1.975845	1.906557	2.352068	2.069023	2.156916	1.936042
17387710	Olfr141	olfactory receptor 141	2.639034	2.124112	2.452869	2.282239	2.860743	2.671333
17215815	Olfr1410	olfactory receptor 1410	3.380883	2.953407	3.229118	3.211551	2.417641	3.047255
17215813	Olfr1411	olfactory receptor 1411	16.1095	21.11696	20.87335	23.74086	30.54935	26.64993
17215811	Olfr1412	olfactory receptor 1412	6.997599	5.519777	7.101531	6.280328	6.829595	5.557169
17215807	Olfr1413	olfactory receptor 1413	2.953407	2.953407	2.953407	2.627863	2.745845	2.598069
17225582	Olfr1414	olfactory receptor 1414	7.0884	9.070487	5.715145	6.687097	6.915579	6.687097
17225580	Olfr1415	olfactory receptor 1415	4.435356	3.286199	3.480055	2.522562	2.188921	2.975083
17225578	Olfr1416	olfactory receptor 1416	3.003345	2.095508	2.425732	2.698766	2.458673	2.198988
17363044	Olfr1417	olfactory receptor 1417	4.629094	6.430585	5.617429	5.357389	5.307123	5.564184
17363046	Olfr1418	olfactory receptor 1418	2.710089	2.477388	2.974609	2.835317	2.475517	3.095495
17363050	Olfr1419	olfactory receptor 1419	3.444537	2.433327	2.559567	2.586874	2.707066	2.831212
17388033	Olfr142	olfactory receptor 142	2.81302	2.652955	2.727464	2.773139	2.231989	2.993763
17357749	Olfr1420	olfactory receptor 1420	3.022453	2.675595	2.929268	3.1411	3.520938	3.478101
17363056	Olfr1423	olfactory receptor 1423	2.872028	2.925047	2.698972	3.82882	2.303456	2.733956
17363058	Olfr1424	olfactory receptor 1424	2.536519	2.49931	2.671667	3.091506	3.072004	2.791983
17363060	Olfr1425	olfactory receptor 1425	2.498652	1.873622	2.427818	2.304206	1.716131	1.908594
17363062	Olfr1426	olfactory receptor 1426	3.366035	2.545272	3.186892	3.403667	3.079252	2.753926
17363068	Olfr1427	olfactory receptor 1427	2.007166	1.960838	1.898013	1.934124	1.848032	2.646893
17363070	Olfr1428	olfactory receptor 1428	4.542574	5.373604	3.547002	5.224634	4.464195	4.194445
17516147	Olfr143	olfactory receptor 143	3.058491	3.384799	2.80564	6.100003	3.132686	3.856762
17357788	Olfr1431	olfactory receptor 1431	2.539492	3.058618	2.738071	2.450897	2.928679	2.450167
17363076	Olfr1432	olfactory receptor 1432	2.935386	3.02239	2.67402	3.757857	3.263862	2.430377
17357794	Olfr1434	olfactory receptor 1434	2.294675	2.654776	2.204696	2.811948	2.550341	2.476307
17363080	Olfr1436	olfactory receptor 1436	2.585781	1.898671	2.092837	1.830554	3.04044	2.676541
17363082	Olfr1437	olfactory receptor 1437	2.984791	3.044826	3.622034	2.536677	2.267009	3.862058
17357799	Olfr1440	olfactory receptor 1440	3.283854	3.164878	2.863679	2.926994	3.677996	3.128931
17357801	Olfr1441	olfactory receptor 1441	3.247839	3.158238	2.859157	3.530127	3.171027	4.011773
17357851	Olfr1442	olfactory receptor 1442	2.342972	2.099739	1.898013	2.031521	2.411783	2.259385
17357855	Olfr1443	olfactory receptor 1443	2.502968	2.362657	2.716634	2.631837	2.543949	2.497578
17357887	Olfr1444	olfactory receptor 1444	7.925819	5.981179	8.467112	9.977304	6.351732	11.62541
17357891	Olfr1445	olfactory receptor 1445	2.738109	2.448096	2.21754	2.264229	4.867092	2.579623
17363125	Olfr1446	olfactory receptor 1446	2.619407	2.240654	2.207082	2.243265	2.027441	1.801614
17363129	Olfr1447	olfactory receptor 1447	2.554056	2.531671	2.158696	3.04698	2.531285	3.057622
17363132	Olfr1448	olfactory receptor 1448	2.161241	2.907199	1.942655	2.078208	3.071131	2.353324
17357897	Olfr1449	olfactory receptor 1449	3.179963	3.729055	3.815706	3.247816	3.576702	3.729055
17516120	Olfr145	olfactory receptor 145	6.719063	5.300432	5.276824	7.106849	9.182901	6.35856
17357899	Olfr1450	olfactory receptor 1450	2.402906	2.03338	2.019083	1.883766	1.926337	2.072138
17357902	Olfr1451	olfactory receptor 1451	2.245754	2.077574	2.199735	2.63673	2.275841	2.81035
17363134	Olfr1453	olfactory receptor 1453	1.942857	1.831493	1.634705	2.344613	1.807668	2.547178
17357904	Olfr1454	olfactory receptor 1454	38.84723	26.69412	30.89454	49.09656	36.67487	48.16957
17363136	Olfr1456-ps1	olfactory receptor 1456, pseudogene 1 [Source:MGI Symbol;Acc:MGI:3031290]	11.4234	8.461245	10.02097	15.41272	9.64058	9.168209
17363139	Olfr1457	olfactory receptor 1457	2.075487	2.342875	2.28235	1.956412	2.020203	1.956642
17363141	Olfr1458	olfactory receptor 1458	2.325531	2.316457	1.994227	2.044517	2.276094	2.421196
17363144	Olfr1459	olfactory receptor 1459	2.565678	2.092097	2.444044	2.101064	2.807761	2.127265
17525706	Olfr146	olfactory receptor 146	1.676567	2.160312	1.953837	2.050947	2.339337	2.257585
17357906	Olfr1461	olfactory receptor 1461	2.162035	1.933132	2.083357	2.083357	2.834256	1.820305
17357908	Olfr1462	olfactory receptor 1462	2.289829	2.13156	2.481049	2.630633	2.481049	2.308715
17357910	Olfr1463	olfactory receptor 1463	23.70649	20.36288	22.2279	29.53431	20.17393	21.0877
17363150	Olfr1465	olfactory receptor 1465	4.365903	3.100455	3.116118	4.840681	4.642655	3.549215
17357912	Olfr1466	olfactory receptor 1466	2.471248	2.110786	2.202237	2.644033	2.651649	2.408709
17357914	Olfr1467	olfactory receptor 1467	3.130428	3.323216	3.607852	2.655199	2.478522	2.296107
17357916	Olfr1469	olfactory receptor 1469	2.78186	2.060051	2.105919	2.606909	2.474831	2.769873
17516161	Olfr147	olfactory receptor 147	3.20997	2.617628	2.670926	2.434541	3.309171	2.268754
17357918	Olfr1471	olfactory receptor 1471	1.986171	2.492321	3.050424	2.479879	3.771583	1.704431
17363152	Olfr1472	olfactory receptor 1472	3.652742	2.412452	1.789541	2.00757	2.512668	2.218447
17357920	Olfr1474	olfactory receptor 1474	2.754251	4.4778	4.458598	3.4108	3.278805	3.4108
17363154	Olfr1475	olfactory receptor 1475	3.353296	1.945135	2.449895	2.931482	2.944739	2.915695
17357922	Olfr1477	olfactory receptor 1477	5.338965	6.068374	4.17504	5.338965	3.925945	5.858251
17516267	Olfr148	olfactory receptor 148	1.983997	2.170113	2.255004	2.019419	2.710483	2.116015
17357926	Olfr1480	olfactory receptor 1480	3.486767	2.93683	2.744322	2.623168	2.967998	3.11506
17357928	Olfr1484	olfactory receptor 1484	2.737293	4.013943	2.61868	2.93136	2.998228	2.552304
17357930	Olfr1487	olfactory receptor 1487	5.124496	6.379927	4.608637	3.153273	4.200788	4.527642
17357932	Olfr1489	olfactory receptor 1489	1.942857	1.759262	2.404705	2.304206	1.969665	1.916375
17525776	Olfr149	olfactory receptor 149	2.613947	2.163894	2.134917	2.994552	2.471677	2.420911
17357934	Olfr1490	olfactory receptor 1490	3.448216	3.305778	3.307406	2.957791	3.595444	3.340004
17357936	Olfr1491	olfactory receptor 1491	6.848082	6.251403	6.56871	10.86638	9.425506	8.194182
17357938	Olfr1494	olfactory receptor 1494	4.681043	4.647678	5.001913	4.591769	4.739254	4.590559

17357940	Olfr1495	olfactory receptor 1495	2.249057	2.097194	2.231556	2.531022	2.695102	2.864136
17357943	Olfr1496	olfactory receptor 1496	2.538964	1.85648	2.255364	2.002899	2.272689	2.151928
17363162	Olfr1497	olfactory receptor 1497	2.981565	2.514184	3.787668	3.298065	3.24226	3.589916
17363164	Olfr1499	olfactory receptor 1499	2.936729	2.279978	3.152748	2.667133	3.598486	2.464013
17322465	Olfr15	olfactory receptor 15	3.228334	3.84715	4.355445	3.787485	4.580103	2.710408
17516280	Olfr150	olfactory receptor 150	3.63481	2.67402	3.023752	3.247208	3.376293	3.023752
17363168	Olfr1500	olfactory receptor 1500	2.611303	2.471163	2.543614	3.633122	2.761764	2.541729
17363171	Olfr1501	olfactory receptor 1501	2.835081	2.167858	2.164629	2.379865	2.128962	2.091285
17357947	Olfr1502	olfactory receptor 1502	6.415448	8.374772	6.366454	4.621112	7.90634	6.038332
17363173	Olfr1504	olfactory receptor 1504	2.505954	2.521391	2.051771	1.932074	2.199613	2.429434
17357949	Olfr1505	olfactory receptor 1505	2.504426	2.565073	2.459406	2.563296	2.841416	2.586408
17306325	Olfr1507	olfactory receptor 1507	2.406006	2.622749	2.452988	2.298368	1.940408	2.285991
17306321	Olfr1508	olfactory receptor 1508	2.516084	2.572177	2.647902	2.717971	2.566211	2.46246
17299767	Olfr1509	olfactory receptor 1509	2.938439	2.938439	2.922717	2.744246	2.954246	2.887778
17525684	Olfr151	olfactory receptor 151	3.062691	3.009972	3.009972	3.009972	3.16251	3.009972
17306318	Olfr1510	olfactory receptor 1510	1.811833	1.942857	1.682842	2.024913	2.649188	1.739232
17306316	Olfr1511	olfactory receptor 1511	2.721345	2.567635	3.009972	2.748796	3.393114	2.513678
17306314	Olfr1512	olfactory receptor 1512	2.397183	3.075263	2.481049	2.57052	3.216206	3.03179
17306312	Olfr1513	olfactory receptor 1513	2.345003	2.036187	2.003663	2.115619	2.246065	2.094027
17372880	Olfr152	olfactory receptor 152	2.610018	3.137814	2.802414	3.533138	2.944739	2.918263
17372862	Olfr153	olfactory receptor 153	2.71099	2.282587	2.122802	2.201566	3.028304	2.187541
17481502	Olfr1532-ps1	olfactory receptor 1532, pseudogene 1	3.241856	2.953407	2.953407	2.81113	2.953407	2.545378
17290983	Olfr1535	olfactory receptor 1535	2.798299	2.782767	3.009972	2.782767	2.782767	2.691462
17525718	Olfr1537	olfactory receptor 1537	3.087844	3.132772	3.107598	2.927725	4.867058	2.36874
17387614	Olfr154	olfactory receptor 154	2.123361	2.079476	2.043001	1.960457	2.076552	2.060551
17413470	Olfr155	olfactory receptor 155	5.237542	4.213678	4.183934	3.720973	4.448627	4.648548
17424815	Olfr156	olfactory receptor 156	3.231961	4.184746	5.617429	4.03783	3.935181	3.23501
17424818	Olfr157	olfactory receptor 157	2.419888	2.897483	2.295359	2.679586	2.115209	2.064325
17424811	Olfr159	olfactory receptor 159	2.415179	2.7641	2.267637	3.323055	3.906861	2.17231
17219629	Olfr16	olfactory receptor 16	2.172927	2.690585	1.983502	2.122773	2.1007	2.603568
17525680	Olfr160	olfactory receptor 160	4.421942	2.047084	2.48704	2.729052	4.609084	2.658016
17322453	Olfr161	olfactory receptor 161	2.395754	2.750702	2.690716	3.86412	3.363679	2.415748
17329026	Olfr164	olfactory receptor 164	2.011888	2.020819	2.123774	2.332472	2.326401	2.750321
17329030	Olfr165	olfactory receptor 165	1.900383	2.004219	1.992293	2.004219	2.643831	1.865006
17323824	Olfr166	olfactory receptor 166	4.583946	3.493589	3.716694	3.905264	3.626505	3.626505
17329034	Olfr167	olfactory receptor 167	2.742972	2.739951	2.275195	2.27682	3.313578	2.418378
17329036	Olfr168	olfactory receptor 168	2.138516	2.255364	2.033916	2.163039	2.494568	2.255364
17329038	Olfr169	olfactory receptor 169	2.406006	2.045807	2.569469	2.406006	3.151372	2.269461
17481508	Olfr17	olfactory receptor 17	2.449216	3.274716	2.717349	2.114388	2.752533	2.743828
17329040	Olfr170	olfactory receptor 170	1.901977	1.941619	1.955205	2.131221	2.906031	3.081238
17329043	Olfr171	olfactory receptor 171	2.86964	2.155496	2.111795	2.351889	1.91042	2.025025
17331217	Olfr172	olfactory receptor 172	5.615249	6.287864	6.992896	7.698994	5.588263	11.64961
17331221	Olfr173	olfactory receptor 173	3.593476	2.220785	1.823892	2.192383	2.921461	2.393514
17331225	Olfr175-ps1	olfactory receptor 175, pseudogene 1	2.446298	2.088157	2.365475	2.494464	2.766746	2.40926
17547980	Olfr175-ps1	olfactory receptor 175, pseudogene 1	3.518767	3.770877	3.279714	3.451276	3.611329	3.26694
17331228	Olfr176	olfactory receptor 176	1.864579	1.910778	1.759701	2.027202	1.910579	1.80906
17331230	Olfr178	olfactory receptor 178	3.772759	2.255364	1.823399	1.979945	2.615125	1.921616
17524363	Olfr18	olfactory receptor 18	2.004649	2.156468	2.503281	2.283727	2.57364	2.250959
17331232	Olfr180	olfactory receptor 180	2.540108	2.164914	2.483664	2.981772	2.188921	3.065006
17331235	Olfr181	olfactory receptor 181	2.196932	1.713837	2.059251	2.231216	2.059251	1.865006
17326445	Olfr183	olfactory receptor 183	2.492148	3.123059	2.187814	2.187814	2.212243	3.004969
17331238	Olfr186	olfactory receptor 186	2.474128	2.51659	2.063395	2.850549	2.382985	3.050551
17331241	Olfr187	olfactory receptor 187	2.13258	2.593032	2.197449	2.2868	2.402672	2.861477
17328539	Olfr19	olfactory receptor 19	2.777968	2.795275	2.737047	2.4217	3.678991	2.621804
17331245	Olfr190	olfactory receptor 190	2.061236	1.769719	2.295837	1.769719	2.228202	1.932074
17331247	Olfr191	olfactory receptor 191	10.72288	6.023617	11.37047	5.665919	6.14912	4.937532
17331249	Olfr192	olfactory receptor 192	2.274674	2.263664	2.177149	2.328321	1.994711	2.421851
17331251	Olfr193	olfactory receptor 193	2.225254	2.014972	1.889939	2.355299	3.388625	2.072138
17331254	Olfr194	olfactory receptor 194	4.20041	5.642248	3.217433	4.20927	4.14017	5.586133
17326448	Olfr195	olfactory receptor 195	2.197617	2.072138	1.972096	2.317726	2.188921	1.875948
17331256	Olfr196	olfactory receptor 196	3.370424	4.135438	3.924204	3.099746	3.727504	3.365941
17331258	Olfr197	olfactory receptor 197	2.39956	3.005448	3.06301	2.849858	2.935467	2.741433
17331260	Olfr198	olfactory receptor 198	2.105656	2.671407	2.394625	2.304206	1.967481	2.182664
17331264	Olfr199	olfactory receptor 199	2.625114	2.197267	1.931163	2.083357	2.418378	2.066701
17494725	Olfr2	olfactory receptor 2	2.486472	2.732516	3.20559	2.335255	2.672982	3.16376
17252702	Olfr20	olfactory receptor 20	3.349301	2.636584	2.668816	2.666727	2.843327	2.641834
17331266	Olfr201	olfactory receptor 201	2.457736	2.646343	2.256427	2.61076	2.235721	2.089315
17331268	Olfr202	olfactory receptor 202	2.267166	2.642329	2.58125	2.205399	2.383596	2.068966
17326451	Olfr203	olfactory receptor 203	4.372081	2.832508	2.911091	2.852308	3.907403	2.887578
17331270	Olfr204	olfactory receptor 204	1.930333	2.119524	3.158764	2.233041	2.262237	1.931096
17331272	Olfr205	olfactory receptor 205	5.725295	5.270902	5.13591	8.591381	5.763719	5.744375
17331274	Olfr206	olfactory receptor 206	2.498565	2.194786	2.38244	2.310972	2.682727	2.571428
17331278	Olfr208	olfactory receptor 208	2.370284	2.953407	2.30622	2.460259	2.402989	2.474694
17462131	Olfr211	olfactory receptor 211	3.573704	4.127677	4.292506	4.729737	3.711211	3.521719
17462133	Olfr212	olfactory receptor 212	3.349951	4.772814	3.703322	5.156206	4.15368	3.737879
17462139	Olfr213	olfactory receptor 213	3.022076	2.867652	4.055922	3.253066	3.160362	3.547297
17462141	Olfr214	olfactory receptor 214	3.297768	3.79245	3.268548	2.826252	3.720767	2.724214
17470050	Olfr215	olfactory receptor 215	3.442699	4.068649	3.25803	3.59131	4.352065	3.330087
17219633	Olfr218	olfactory receptor 218	3.264043	3.381867	3.655376	4.261526	3.501759	2.866102
17219764	Olfr220	olfactory receptor 220	3.075114	2.826213	3.036501	3.278782	3.416478	2.905004
17306186	Olfr221	olfactory receptor 221	2.391358	3.098736	2.391888	2.970097	2.802763	3.304221
17263440	Olfr222	olfactory receptor 222	4.874384	7.198868	7.458627	6.141368	5.326434	6.988632
17263444	Olfr223	olfactory receptor 223	2.449046	2.476736	2.515055	2.31779	2.89905	2.483991
17263168	Olfr224	olfactory receptor 224	3.156991	2.847429	2.610362	3.155437	2.645866	2.516782

17250264	Olfr225	olfactory receptor 225	4.934316	5.484728	5.022376	3.088893	3.597513	4.808677
17387683	Olfr228	olfactory receptor 228	2.540672	2.238776	2.081524	2.153197	2.132092	1.727349
17516292	Olfr229	olfactory receptor 229	2.391209	2.508352	1.965369	2.443366	2.424757	1.849827
17252724	Olfr23	olfactory receptor 23	2.480326	2.184177	2.255364	1.893257	2.636383	2.285991
17230120	Olfr231	olfactory receptor 231	3.655959	3.494776	3.787485	3.896584	3.569569	3.90456
17357790	Olfr235	olfactory receptor 235	3.062691	2.951892	2.840569	3.261826	2.449573	2.984688
17458000	Olfr237-ps1	olfactory receptor 237, pseudogene 1	3.062691	2.239691	2.688497	2.304206	6.030928	2.304206
17336073	Olfr239	olfactory receptor 239	3.436548	3.514112	3.653831	3.231894	3.010181	2.810233
17524322	Olfr24	olfactory receptor 24	2.153614	2.169526	2.181257	2.169526	2.825586	2.06653
17481225	Olfr243	olfactory receptor 243	2.279931	1.812599	2.492891	2.048077	2.403006	2.14319
17246453	Olfr247	olfactory receptor 247	4.157511	3.585216	3.735937	3.756164	4.323831	3.735937
17246455	Olfr247	olfactory receptor 247	4.157511	3.585216	3.735937	3.756164	4.323831	3.735937
17219758	Olfr248	olfactory receptor 248	4.103258	2.67402	3.323999	3.062691	2.934084	3.062691
17516151	Olfr25	olfactory receptor 25	2.409477	3.184816	2.727123	2.985474	4.219026	3.281829
17516157	Olfr250	olfactory receptor 250	3.530885	2.67402	3.009972	2.892626	2.67402	2.813137
17516159	Olfr251	olfactory receptor 251	3.77616	6.740287	4.810544	2.859593	4.383126	4.344048
17387742	Olfr259	olfactory receptor 259	5.759366	3.686725	4.88907	4.378055	2.754461	3.439503
17516237	Olfr26	olfactory receptor 26	2.888859	2.636091	2.272027	2.756447	4.520743	2.812981
17363078	Olfr262	olfactory receptor 262	3.391421	3.041768	4.685945	2.711103	3.274285	3.13999
17285616	Olfr263	olfactory receptor 263	2.076091	1.665771	1.604318	1.98613	2.84887	1.784525
17408970	Olfr266	olfactory receptor 266	3.96992	4.092975	3.097899	4.472898	3.825425	4.20927
17425706	Olfr267	olfactory receptor 267	10.11784	7.276678	7.546931	7.175105	7.384243	7.940336
17516253	Olfr27	olfactory receptor 27	2.075516	2.108563	1.898013	2.109264	2.331648	1.865006
17414159	Olfr270	olfactory receptor 270	2.331292	2.458673	2.458673	2.540108	2.583273	2.482683
17425296	Olfr272	olfactory receptor 272	2.289623	2.236775	2.045509	2.168308	2.289623	3.274331
17425291	Olfr273	olfactory receptor 273	4.199391	2.804785	2.76119	2.822728	3.312843	2.963085
17414152	Olfr275	olfactory receptor 275	2.920368	2.804435	2.870118	2.953407	3.22735	2.920368
17314624	Olfr279	olfactory receptor 279	12.16159	11.10179	14.32308	13.14653	8.491974	11.17358
17314621	Olfr281	olfactory receptor 281	4.647678	3.458293	4.647678	4.096154	4.790214	3.752729
17314619	Olfr282	olfactory receptor 282	7.9551	7.9551	6.992896	8.892013	9.617287	8.082939
17321217	Olfr283	olfactory receptor 283	6.743605	7.184661	6.294536	6.688627	8.551989	8.88659
17321215	Olfr284	olfactory receptor 284	4.534269	4.155177	4.418847	4.620471	3.954951	5.789465
17321213	Olfr285	olfactory receptor 285	5.310177	5.109174	5.854882	5.599702	4.995226	6.041137
17321194	Olfr286	olfactory receptor 286	3.099896	2.46738	3.885526	2.214606	2.39956	3.104541
17321186	Olfr287	olfactory receptor 287	2.803482	2.976506	3.723682	2.976506	3.263862	2.606909
17321179	Olfr288	olfactory receptor 288	2.659546	2.309499	2.695812	3.626028	3.609402	4.270633
17424813	Olfr29-ps1	olfactory receptor 29, pseudogene 1	3.085298	3.723837	4.197528	3.524699	5.038101	3.426273
17479927	Olfr290	olfactory receptor 290	3.116702	2.174947	2.472482	2.393397	2.249649	2.445569
17479923	Olfr291	olfactory receptor 291	2.112835	2.258226	2.028734	2.392817	2.302913	2.191426
17479949	Olfr292	olfactory receptor 292	2.073	1.974818	1.777747	2.094709	2.287355	1.925442
17479947	Olfr293	olfactory receptor 293	2.187768	1.821908	2.482511	2.013772	2.130379	2.09484
17493143	Olfr294	olfactory receptor 294	2.600069	2.608011	2.618426	2.66246	2.725952	2.081192
17479944	Olfr295	olfactory receptor 295	1.965205	1.885569	2.569878	1.887701	1.957022	2.241384
17479942	Olfr297	olfactory receptor 297	2.406006	2.57662	2.09423	2.248714	2.016579	2.123214
17493141	Olfr298	olfactory receptor 298	1.670732	2.012069	2.16529	2.612045	3.12956	2.544267
17479940	Olfr299	olfactory receptor 299	2.301557	1.787087	2.007263	3.059615	2.175957	2.175866
17384561	Olfr3	olfactory receptor 3	4.714484	5.060711	4.810277	5.037577	5.916156	5.401275
17263148	Olfr30	olfactory receptor 30	2.850707	2.929694	2.67402	2.552357	3.64925	2.776678
17479936	Olfr301	olfactory receptor 301	2.690194	2.548644	3.55685	2.084354	2.810272	2.512285
17493137	Olfr303	olfactory receptor 303	4.306393	4.356924	3.309951	3.313418	2.875654	4.055838
17493135	Olfr304	olfactory receptor 304	3.71971	2.590212	2.680236	2.56751	2.528181	3.409287
17493133	Olfr305	olfactory receptor 305	2.162035	2.102186	2.112439	2.314996	2.059523	2.115223
17493130	Olfr307	olfactory receptor 307	3.275193	2.220785	1.795431	1.990651	1.906478	2.623204
17493126	Olfr308	olfactory receptor 308	2.66926	2.402956	2.263413	2.536994	2.859237	2.34632
17493121	Olfr309	olfactory receptor 309	2.934979	2.583147	2.973166	2.901462	2.723912	3.002262
17297209	Olfr31	olfactory receptor 31	3.101186	3.111284	3.599833	2.426926	3.337759	4.076978
17493116	Olfr310	olfactory receptor 310	3.667787	3.766958	5.319276	3.314773	4.521339	4.574709
17250115	Olfr311	olfactory receptor 311	3.685984	3.578885	2.610579	2.688013	3.241429	3.940995
17250113	Olfr312	olfactory receptor 312	3.394126	2.04085	2.255364	2.606909	2.294262	2.285991
17250109	Olfr313	olfactory receptor 313	4.732262	3.55047	4.189535	4.359945	4.333882	5.486211
17250105	Olfr314	olfactory receptor 314	4.564289	5.834504	4.113681	5.459088	6.857677	4.472867
17250101	Olfr315	olfactory receptor 315	7.382298	7.791926	5.793278	7.310044	2.468954	6.442586
17250097	Olfr316	olfactory receptor 316	2.662774	3.226679	4.083624	4.159298	3.477523	4.493003
17263189	Olfr317	olfactory receptor 317	4.320296	6.479938	5.651759	4.681043	4.116106	4.793867
17263187	Olfr318	olfactory receptor 318	13.86783	17.61875	16.22054	31.01985	25.97929	20.14444
17250095	Olfr319	olfactory receptor 319	2.147012	1.898013	2.239691	1.833322	2.512058	2.635269
17388019	Olfr32	olfactory receptor 32	2.678397	2.464867	2.638138	2.290369	2.800278	2.197556
17250093	Olfr320	olfactory receptor 320	6.034608	4.98181	5.085255	4.651545	6.81281	5.099338
17250089	Olfr322	olfactory receptor 322	6.133243	6.922773	5.654149	5.500108	8.22377	4.886969
17263185	Olfr323	olfactory receptor 323	5.599547	6.886114	4.909922	5.401238	6.030928	5.675234
17250076	Olfr324	olfactory receptor 324	2.930994	2.67402	2.606909	2.083357	2.636931	2.606909
17250072	Olfr325	olfactory receptor 325	3.382641	2.708004	2.514794	2.676115	2.617174	2.872765
17263164	Olfr328	olfactory receptor 328	2.412903	2.174419	2.092228	3.335909	2.512947	1.910619
17263160	Olfr329-ps	olfactory receptor 329, pseudogene	2.788521	2.632475	3.180823	2.7702	2.489973	2.589063
17494126	Olfr33	olfactory receptor 33	2.406006	2.274879	2.466662	2.112191	2.435925	1.61087
17263157	Olfr330	olfactory receptor 330	2.273744	1.898724	1.981056	2.347785	1.825283	2.105073
17263154	Olfr331	olfactory receptor 331	4.526607	5.370625	10.10747	5.137513	7.920656	6.294841
17263151	Olfr332	olfactory receptor 332	9.159507	9.764323	11.8715	9.293938	10.59205	15.43764
17370326	Olfr338	olfactory receptor 338	2.484335	1.897171	2.121346	2.866956	3.204191	2.606909
17370330	Olfr339	olfactory receptor 339	1.999958	1.85903	1.833258	3.020003	2.188921	2.435807
17370332	Olfr340	olfactory receptor 340	2.854464	2.456799	2.817216	3.872003	2.202298	2.599979
17384559	Olfr341	olfactory receptor 341	2.953407	2.796922	2.953407	3.018496	2.973949	1.874921
17370334	Olfr342	olfactory receptor 342	4.048058	2.293324	2.348908	2.908993	2.477423	2.284471
17370337	Olfr344	olfactory receptor 344	2.988373	4.115877	3.158873	2.112703	2.737597	5.223548

17370339	Olfr345	olfactory receptor 345	3.575091	3.376644	4.285549	2.919457	3.515866	3.701166
17370341	Olfr346	olfactory receptor 346	2.533479	2.304206	1.86903	2.304206	2.804415	1.797971
17370343	Olfr347	olfactory receptor 347	2.489852	2.809961	3.040334	2.406723	2.303552	2.309307
17370345	Olfr348	olfactory receptor 348	2.764292	2.925007	2.577639	2.341057	2.047367	2.680961
17370350	Olfr350	olfactory receptor 350	2.875813	1.904523	2.078208	2.090561	3.236961	2.101443
17384563	Olfr351	olfactory receptor 351	2.679697	2.347052	2.520535	2.852032	2.187935	3.439074
17370352	Olfr352	olfactory receptor 352	3.282011	3.701423	3.662148	3.221963	2.540267	3.495261
17384565	Olfr353	olfactory receptor 353	3.777155	2.942495	3.208169	2.953407	3.02948	2.995652
17370354	Olfr354	olfactory receptor 354	2.170715	2.048247	2.035058	2.035058	2.230396	2.111883
17384567	Olfr355	olfactory receptor 355	2.67402	2.906655	2.658035	2.658035	3.611429	2.532988
17370358	Olfr356	olfactory receptor 356	3.051228	2.690678	3.481961	2.953407	2.831036	2.991875
17370360	Olfr357	olfactory receptor 357	2.220785	2.132787	2.241384	2.083357	2.740635	2.220785
17384569	Olfr358	olfactory receptor 358	2.899632	2.304191	1.960661	2.083357	2.53863	2.060822
17370364	Olfr360	olfactory receptor 360	2.241726	2.533391	2.330145	2.436517	2.313585	2.379255
17384572	Olfr361	olfactory receptor 361	2.895094	2.318642	3.165317	1.973559	3.786461	2.605283
17384576	Olfr362	olfactory receptor 362	4.532415	4.913258	3.960246	5.12446	4.88382	6.524738
17370367	Olfr364-ps1	olfactory receptor 364, pseudogene 1	2.758225	2.638339	2.481049	2.659804	2.274485	2.659804
17370369	Olfr365	olfactory receptor 365	2.050449	2.230102	2.67426	2.844175	2.316232	3.375872
17370371	Olfr366	olfactory receptor 366	4.720894	4.780694	5.055136	4.759104	7.18481	4.264008
17370373	Olfr367-ps	olfactory receptor 367, pseudogene	3.191844	2.429787	2.481049	2.496246	2.891845	2.822728
17370376	Olfr368	olfactory receptor 368	5.183512	4.292387	4.911658	5.752384	6.21567	5.598421
17502940	Olfr370	olfactory receptor 370	9.824473	12.3118	7.766152	11.61325	11.91825	11.32399
17503429	Olfr371	olfactory receptor 371	4.599956	3.873264	5.344741	5.080569	4.717459	5.574104
17502366	Olfr372	olfactory receptor 372	3.021113	3.29754	2.67402	2.12877	4.446654	2.593553
17502369	Olfr373	olfactory receptor 373	6.0805	4.486654	4.96571	5.405919	8.50741	5.422432
17502371	Olfr374	olfactory receptor 374	6.828979	6.951624	6.33801	7.765614	6.030928	5.144497
17252706	Olfr376	olfactory receptor 376	2.680589	2.916585	2.809338	2.428962	3.255863	3.333575
17265770	Olfr378	olfactory receptor 378	7.622002	3.446877	4.385253	5.712927	5.126876	4.465866
17457969	Olfr38	olfactory receptor 38	5.553241	5.574066	6.40656	4.6664	6.09113	5.412631
17265773	Olfr380	olfactory receptor 380	2.432972	2.124053	2.492166	2.255364	2.348777	2.488127
17265777	Olfr381	olfactory receptor 381	2.391225	1.848365	2.325176	2.304206	1.978449	2.304206
17265781	Olfr382	olfactory receptor 382	2.904158	3.53069	2.889	3.526263	2.526465	2.816513
17252710	Olfr384	olfactory receptor 384	2.957483	2.081856	2.632475	2.304206	3.052307	2.666745
17265783	Olfr385	olfactory receptor 385	3.381258	3.764713	3.481165	3.257443	4.308095	2.781552
17265785	Olfr389	olfactory receptor 389	2.036441	2.265422	2.137153	2.092228	2.917677	1.766238
17514977	Olfr39	olfactory receptor 39	2.164059	2.013199	2.635049	2.195151	2.906434	2.200727
17252721	Olfr390	olfactory receptor 390	4.66265	5.500756	5.120306	4.261821	5.048973	5.166474
17265789	Olfr391-ps	olfactory receptor 391, pseudogene	3.994652	2.835848	2.855691	2.627571	2.833804	2.506649
17265792	Olfr392	olfactory receptor 392	2.516799	2.722836	3.962882	3.262007	4.118703	2.373984
17265796	Olfr393	olfactory receptor 393	3.164681	2.457634	2.67402	2.004927	3.117263	2.420844
17265798	Olfr394	olfactory receptor 394	5.297934	4.540307	7.179234	6.704222	6.62904	7.496774
17265800	Olfr395	olfactory receptor 395	2.083357	2.083357	1.960838	2.557351	2.57052	2.083357
17252726	Olfr397	olfactory receptor 397	2.094724	2.430798	2.05281	2.255364	3.257556	1.991713
17265802	Olfr398	olfactory receptor 398	4.801549	4.941709	3.881919	6.633315	3.910817	3.865942
17265808	Olfr399	olfactory receptor 399	3.40565	3.54506	4.1404	4.801649	3.462634	4.068396
17252737	Olfr401	olfactory receptor 401	3.263862	3.263862	3.009972	2.953407	3.056117	2.446925
17252739	Olfr402	olfactory receptor 402	3.052286	2.353879	2.432568	3.108352	2.861854	2.032817
17252742	Olfr403	olfactory receptor 403	3.419037	3.331981	2.533497	2.97921	2.605066	3.77891
17252744	Olfr406	olfactory receptor 406	2.123862	2.292831	2.35052	2.853554	2.583219	3.339703
17265818	Olfr410	olfactory receptor 410	2.640388	2.693272	2.298161	2.533128	2.861755	2.606909
17265821	Olfr411	olfactory receptor 411	3.262686	3.867899	3.053936	4.365509	3.525456	4.162384
17252756	Olfr412	olfactory receptor 412	2.261939	2.333362	2.359253	2.581823	2.522947	2.404905
17219762	Olfr414	olfactory receptor 414	4.335385	3.54506	4.647678	4.514668	6.045954	5.020601
17219756	Olfr417	olfactory receptor 417	12.32888	10.64696	6.776451	11.39746	8.320326	15.55752
17219639	Olfr418	olfactory receptor 418	4.893206	9.118396	6.104106	4.270337	5.981138	5.56106
17230123	Olfr419	olfactory receptor 419	6.248977	4.717263	7.039437	4.015167	5.774636	6.169912
17219694	Olfr420	olfactory receptor 420	3.82683	2.857751	3.649807	4.46633	3.639323	2.588955
17219690	Olfr421-ps1	olfactory receptor 421, pseudogene 1	2.497353	2.220785	2.220785	2.392751	2.528409	2.154256
17219688	Olfr424	olfactory receptor 424	3.520158	3.120506	3.320568	3.694784	3.003345	3.741249
17219686	Olfr427	olfactory receptor 427	4.329919	4.290483	4.717884	4.148558	4.777216	7.602163
17219684	Olfr429	olfactory receptor 429	2.829466	2.829466	3.362187	2.606909	2.115282	3.599509
17265814	Olfr43	olfactory receptor 43	2.989512	2.989512	3.461242	2.76734	2.674557	3.01327
17219682	Olfr430	olfactory receptor 430	3.141645	2.607577	2.891885	3.672417	2.863521	2.774331
17219680	Olfr432	olfactory receptor 432	4.430102	3.154191	3.295004	3.295004	2.659564	3.39874
17219677	Olfr433	olfactory receptor 433	2.654813	2.878825	2.529163	2.846877	3.508587	2.867414
17458012	Olfr434	olfactory receptor 434	1.942857	2.16184	2.091865	1.663901	1.971509	2.204589
17458010	Olfr435	olfactory receptor 435	2.028256	2.281148	3.127565	2.161631	2.053479	2.606909
17458003	Olfr437	olfactory receptor 437	2.530022	3.54506	3.890647	5.821617	3.129148	2.831526
17525733	Olfr44	olfactory receptor 44	2.354009	2.015824	1.836489	2.606909	2.102739	1.865006
17457997	Olfr441	olfactory receptor 441	2.255911	2.771102	2.871809	2.551295	2.914948	3.320269
17457987	Olfr444	olfactory receptor 444	2.025699	2.295471	1.984245	2.077747	2.383415	2.294198
17457985	Olfr446	olfactory receptor 446	5.94077	4.286618	3.057452	3.714428	4.384037	4.978703
17457994	Olfr446	olfactory receptor 446	3.824815	4.646905	5.957306	4.481619	4.733804	4.865034
17457983	Olfr447	olfactory receptor 447	3.955801	3.642149	4.047862	4.18579	3.147333	3.566033
17457981	Olfr448	olfactory receptor 448	4.889646	2.939172	3.340282	3.18369	4.018842	2.866182
17457977	Olfr449	olfactory receptor 449	3.893803	3.825133	3.902828	2.978488	6.50744	3.729055
17484580	Olfr45	olfactory receptor 45	2.356654	5.454436	5.425402	3.966262	4.191103	3.875923
17457973	Olfr450	olfactory receptor 450	2.912766	3.213621	2.690902	3.521451	3.263862	5.981677
17457971	Olfr452	olfactory receptor 452	7.225463	9.026708	9.085022	12.94076	11.20655	12.50567
17457967	Olfr453	olfactory receptor 453	2.776794	2.79859	4.442525	2.705021	2.623568	2.95857
17466426	Olfr455	olfactory receptor 455	6.609495	8.440977	9.581755	9.294582	9.874584	9.377348
17466421	Olfr456	olfactory receptor 456	3.356692	2.294691	2.821007	2.139776	2.731948	2.714525
17466418	Olfr457	olfactory receptor 457	2.67402	2.292513	2.16235	2.292513	2.732554	2.360725
17466416	Olfr458	olfactory receptor 458	2.619933	3.904722	2.0753	3.159048	4.037186	2.265689

17466382	Olfr459	olfactory receptor 459	2.820674	2.778315	2.716464	2.753468	2.701705	2.794035
17484565	Olfr46	olfactory receptor 46	3.981826	2.656469	2.411432	2.555083	2.620424	2.708436
17457605	Olfr460	olfactory receptor 460	3.978515	3.709848	3.662072	4.448596	5.088851	4.40127
17466226	Olfr461	olfactory receptor 461	3.620227	3.007594	2.754862	2.932132	2.790551	2.214053
17267536	Olfr462	olfactory receptor 462	3.620026	2.695868	2.777641	3.563809	2.876391	3.768995
17267538	Olfr463	olfactory receptor 463	2.645298	2.234141	2.411766	2.215128	3.34958	3.669211
17267540	Olfr464	olfactory receptor 464	2.50224	2.148411	2.820283	2.197815	2.612679	2.301429
17288185	Olfr466	olfactory receptor 466	5.998077	5.722081	5.894829	6.02249	6.918599	4.476962
17481588	Olfr467	olfactory receptor 467	4.441694	2.67402	3.551651	5.084973	7.610124	3.576355
17494770	Olfr469	olfactory receptor 469	5.120306	5.120306	6.056356	5.490585	5.219965	4.388598
17458016	Olfr47	olfactory receptor 47	2.067217	1.898013	1.987659	1.987659	2.229809	2.072138
17494772	Olfr470	olfactory receptor 470	2.52741	2.282461	2.668705	2.267858	2.297476	2.176425
17494774	Olfr471	olfactory receptor 471	2.575888	2.385398	2.242286	2.709525	2.905568	2.699646
17481590	Olfr472	olfactory receptor 472	3.734927	4.135954	3.413661	4.758444	3.539879	4.763526
17481594	Olfr473	olfactory receptor 473	2.301429	2.466491	2.051871	3.402464	2.289052	2.551331
17481596	Olfr474	olfactory receptor 474	2.875474	2.372322	2.289258	2.91707	2.833745	3.3898
17481598	Olfr476	olfactory receptor 476	3.478632	2.487765	2.487765	2.606909	2.487765	2.259776
17481600	Olfr477	olfactory receptor 477	2.135953	2.286403	2.072138	2.083357	2.379272	1.957076
17494777	Olfr478	olfactory receptor 478	2.483406	3.851686	3.133902	3.144019	3.660625	2.705565
17481602	Olfr479	olfactory receptor 479	3.511482	3.201216	3.44542	3.697551	4.587442	3.354947
17388006	Olfr48	olfactory receptor 48	3.41035	3.62339	3.215047	4.76148	3.84539	3.375474
17494779	Olfr480	olfactory receptor 480	2.049526	2.691686	2.161421	1.983062	2.282904	2.21608
17481606	Olfr481	olfactory receptor 481	4.166685	6.100511	4.269745	4.037522	4.670639	3.940176
17494781	Olfr482	olfactory receptor 482	2.951994	2.798435	4.479724	3.220824	2.75322	2.606909
17481608	Olfr483	olfactory receptor 483	2.356687	2.007097	2.017614	2.332117	2.364295	2.513504
17494785	Olfr484	olfactory receptor 484	4.628196	5.973473	6.373916	6.714267	10.03911	7.981943
17494789	Olfr485	olfactory receptor 485	4.043823	3.802452	3.334245	4.147983	2.954738	2.953407
17494791	Olfr486	olfactory receptor 486	2.402339	1.978915	3.276624	2.676504	2.679363	2.720459
17494793	Olfr487	olfactory receptor 487	2.060051	1.80827	1.796638	2.209025	1.862629	1.886641
17494795	Olfr488	olfactory receptor 488	2.420844	2.420844	2.420844	2.798182	2.639052	2.072138
17306341	Olfr49	olfactory receptor 49	3.121674	2.452274	2.823648	2.953407	2.90104	3.086154
17494797	Olfr490	olfactory receptor 490	2.698149	4.615414	2.368412	2.626297	3.450511	2.735852
17481612	Olfr491	olfactory receptor 491	5.234349	4.020765	3.377931	4.389419	3.729055	5.149385
17494799	Olfr492	olfactory receptor 492	3.204435	5.870323	5.386134	5.386134	4.771656	7.170979
17494801	Olfr493	olfactory receptor 493	2.733141	1.945445	1.981249	2.355152	2.179187	2.336534
17481614	Olfr494	olfactory receptor 494	2.406006	2.712588	2.043709	2.276283	2.421381	2.375482
17481616	Olfr495	olfactory receptor 495	3.433786	2.469211	2.477612	1.752567	3.01373	2.684624
17481618	Olfr497	olfactory receptor 497	2.808131	2.164134	2.444433	2.285136	2.36097	2.480017
17481620	Olfr498	olfactory receptor 498	3.527754	2.641248	2.553259	3.09835	3.794685	3.289891
17486089	Olfr5	olfactory receptor 5	1.826751	2.362984	2.450863	2.58795	1.966486	2.093577
17370348	Olfr50	olfactory receptor 50	2.821046	3.145305	3.203524	2.443298	3.46989	2.868885
17494803	Olfr502	olfactory receptor 502	2.552498	2.503263	2.450099	2.53132	3.084165	2.503697
17481622	Olfr503	olfactory receptor 503	6.962956	4.381759	5.812384	6.369058	8.490267	6.82969
17494806	Olfr504	olfactory receptor 504	2.672834	2.720308	2.354613	2.358958	3.131622	2.806282
17481624	Olfr506	olfactory receptor 506	2.911253	1.926671	2.134103	3.047867	2.458673	1.966336
17481628	Olfr507	olfactory receptor 507	2.513696	2.182936	2.003011	2.48499	2.49277	2.563722
17481630	Olfr508	olfactory receptor 508	1.783622	2.553896	2.405122	3.050192	4.248666	3.007553
17494808	Olfr509	olfactory receptor 509	4.762437	4.712655	5.023246	6.679638	5.838104	5.844259
17249256	Olfr51	olfactory receptor 51	2.198958	2.362444	2.67402	2.950542	2.782767	7.004247
17481632	Olfr510	olfactory receptor 510	4.051399	4.05182	5.47121	4.812678	3.256653	4.969119
17481634	Olfr512	olfactory receptor 512	3.579976	4.033102	3.232208	3.889568	3.526018	4.532195
17481638	Olfr513	olfactory receptor 513	3.414135	4.27016	3.245678	4.210437	4.227134	3.319417
17494811	Olfr514	olfactory receptor 514	3.582086	2.406356	2.672259	2.87408	3.443343	3.407208
17494813	Olfr516	olfactory receptor 516	3.279305	2.559017	2.327789	3.172148	2.696149	2.784754
17494815	Olfr517	olfactory receptor 517	2.612625	2.253879	2.226828	2.047623	2.645866	2.273051
17494818	Olfr518	olfactory receptor 518	10.30891	7.565786	8.793698	9.166303	10.22324	7.612551
17494820	Olfr519	olfactory receptor 519	10.30627	7.144287	10.43819	8.808278	11.35244	4.717067
17387646	Olfr52	olfactory receptor 52	1.942857	1.791118	2.056584	2.408091	3.221405	2.111649
17480596	Olfr520	olfactory receptor 520	2.67402	2.608391	2.67402	3.282398	3.263862	2.606909
17480598	Olfr521	olfactory receptor 521	3.008282	3.883991	3.221383	3.179302	3.752	4.608062
17497633	Olfr522	olfactory receptor 522	8.673895	11.87479	10.85961	9.29207	6.995902	11.25863
17484510	Olfr523	olfactory receptor 523	4.629672	7.700222	9.232875	7.307257	9.330989	6.59764
17497637	Olfr524	olfactory receptor 524	4.783511	4.188634	6.168501	6.830873	4.21695	5.17002
17484550	Olfr525	olfactory receptor 525	16.95396	15.01851	14.45774	9.417147	14.84227	20.81382
17484553	Olfr527	olfactory receptor 527	8.672813	12.79067	10.8363	12.38378	11.93983	13.02724
17484573	Olfr53	olfactory receptor 53	2.903011	2.643684	2.721477	2.743067	3.342274	2.790744
17497643	Olfr530	olfactory receptor 530	2.553153	3.9327	2.689336	2.665544	3.078655	2.535078
17497647	Olfr531	olfactory receptor 531	3.59891	4.358797	3.511993	4.224059	5.080991	3.949418
17497649	Olfr532	olfactory receptor 532	3.424777	3.060994	3.372995	3.124922	3.583825	3.822562
17484556	Olfr533	olfactory receptor 533	2.594308	2.090488	2.09275	2.713604	2.79417	2.243218
17484560	Olfr535	olfactory receptor 535	6.669506	5.230178	5.553164	7.717534	8.90262	9.191243
17497651	Olfr536	olfactory receptor 536	7.599792	6.137921	8.181129	9.228013	8.16538	6.681722
17484563	Olfr538	olfactory receptor 538	4.870534	7.189343	5.338965	6.171709	4.911964	5.338965
17484578	Olfr539	olfactory receptor 539	2.438933	2.793338	2.8138	3.272538	3.738138	3.337783
17249258	Olfr54	olfactory receptor 54	4.478576	4.708639	4.457207	3.26617	7.398947	4.433912
17484583	Olfr541	olfactory receptor 541	4.835617	4.801649	4.20927	3.634357	5.537597	4.95749
17494087	Olfr543	olfactory receptor 543	5.154563	5.605255	4.16732	5.566537	3.570386	4.243663
17494091	Olfr544	olfactory receptor 544	5.767915	5.450997	5.599857	5.044775	5.301498	4.589128
17494095	Olfr545	olfactory receptor 545	2.720836	2.857453	2.67402	2.814854	2.780395	2.446552
17481123	Olfr547	olfactory receptor 547	3.709539	4.200642	4.290066	5.159531	6.644267	3.781189
17481125	Olfr548-ps1	olfactory receptor 548, pseudogene 1	2.755569	2.436297	2.481049	2.674242	2.359057	2.659804
17481127	Olfr549	olfactory receptor 549	14.9126	19.8488	18.27602	16.86254	17.43579	17.14956
17336071	Olfr55	olfactory receptor 55	3.182521	4.487494	3.39874	2.939804	2.998623	2.961483
17481129	Olfr550	olfactory receptor 550	2.269619	2.519435	2.255364	2.266003	2.418865	2.237891

17494111	Olfr551	olfactory receptor 551	6.435267	5.726724	6.171709	6.171709	5.834949	6.992023
17481133	Olfr552	olfactory receptor 552	3.721902	3.715999	3.778962	4.28442	3.309424	3.388766
17494115	Olfr553	olfactory receptor 553	2.390016	2.744265	2.418311	2.59287	2.989885	2.528742
17481135	Olfr554	olfactory receptor 554	4.832199	4.345222	5.120306	3.263862	4.352065	4.128478
17481137	Olfr555	olfactory receptor 555	2.749578	2.424538	2.980057	3.063668	3.028283	2.81829
17481139	Olfr556	olfactory receptor 556	3.413259	2.740559	3.723192	3.126395	3.109559	2.547426
17481141	Olfr557	olfactory receptor 557	2.67402	2.727483	2.819013	3.369349	2.739077	2.619279
17481144	Olfr558	olfactory receptor 558	5.003404	6.188244	5.003404	6.217006	5.689887	4.976495
17494128	Olfr559	olfactory receptor 559	2.595244	2.31219	2.126086	2.406006	2.782767	2.968677
17481149	Olfr561	olfactory receptor 561	2.105656	2.294659	2.359989	2.087028	2.188921	2.212443
17481153	Olfr564	olfactory receptor 564	3.968297	3.261013	3.520109	3.674964	3.396856	3.685703
17494142	Olfr566	olfactory receptor 566	5.403971	3.218191	3.035912	3.339703	3.339703	3.125312
17481155	Olfr568	olfactory receptor 568	3.870607	3.716849	4.299473	3.585365	2.784349	3.849871
17494144	Olfr569	olfactory receptor 569	2.954164	2.67402	2.630195	2.28031	2.119685	2.460105
17234861	Olfr57	olfactory receptor 57	2.370596	2.221617	2.250866	2.700432	2.820479	3.723398
17481157	Olfr570	olfactory receptor 570	3.198532	2.789101	2.339856	2.645371	2.021379	2.787187
17494146	Olfr571	olfactory receptor 571	8.84229	7.507018	7.865999	9.360853	6.962522	8.461949
17481161	Olfr572	olfactory receptor 572	2.994593	2.606909	2.606909	2.197891	2.046218	2.75639
17494148	Olfr573-ps1	olfactory receptor 573, pseudogene 1	3.785044	2.658422	2.67402	2.955086	3.263862	2.67402
17481163	Olfr574	olfactory receptor 574	3.726497	3.792344	3.930519	3.873721	3.324967	3.14729
17494150	Olfr575	olfactory receptor 575	3.428577	4.415663	3.52954	4.61855	4.021267	4.238841
17481165	Olfr576	olfactory receptor 576	3.936736	3.661919	3.497417	3.98851	2.614509	3.898232
17494152	Olfr577	olfactory receptor 577	4.465773	4.801649	4.315178	4.575629	4.20927	4.711054
17494154	Olfr578	olfactory receptor 578	3.31344	3.219909	2.997127	2.86503	2.952526	3.045122
17514948	Olfr58	olfactory receptor 58	2.423866	2.040906	2.166941	3.049262	2.027343	2.072138
17481167	Olfr582	olfactory receptor 582	2.517061	2.895736	2.458673	2.3692	2.458673	3.046051
17481169	Olfr583	olfactory receptor 583	3.078228	2.228387	2.173259	2.992477	3.51248	2.568988
17481171	Olfr584	olfactory receptor 584	6.017399	15.01789	9.688746	8.574663	9.120039	7.62734
17481173	Olfr585	olfactory receptor 585	2.589781	2.157155	2.275873	1.858618	2.199141	2.029072
17494156	Olfr586	olfactory receptor 586	2.914564	2.566514	2.831036	2.741262	2.844214	2.711723
17494158	Olfr589	olfactory receptor 589	3.546805	2.843938	3.140077	3.410871	3.303785	2.813781
17252749	Olfr59	olfactory receptor 59	3.240081	2.744418	3.032253	3.938537	3.437977	2.45394
17494160	Olfr591	olfactory receptor 591	2.246392	2.669852	2.673408	3.029837	3.197712	2.806515
17481175	Olfr592	olfactory receptor 592	4.751688	4.418357	3.866478	4.859743	3.509827	4.596131
17481180	Olfr593	olfactory receptor 593	3.274194	2.953407	3.182918	4.451125	4.908867	3.596765
17481182	Olfr594	olfactory receptor 594	5.126059	5.406631	4.810177	5.057379	5.444841	5.155706
17481188	Olfr596	olfactory receptor 596	4.090111	5.1801	3.338708	5.070858	2.615705	4.588174
17481190	Olfr597	olfactory receptor 597	2.624695	2.882639	2.643648	2.773254	3.698679	2.512685
17481194	Olfr598	olfactory receptor 598	3.4406	3.243384	2.67402	3.699807	2.982186	3.243384
17481196	Olfr599	olfactory receptor 599	2.692581	2.538137	2.798726	2.556606	3.126221	2.346044
17494717	Olfr6	olfactory receptor 6	3.085127	2.606547	2.481049	2.952076	3.26762	3.161984
17497641	Olfr60	olfactory receptor 60	2.606909	2.170564	2.792776	2.319558	3.799369	2.6466
17494166	Olfr600	olfactory receptor 600	2.999309	2.608373	2.531741	2.600772	2.668613	2.346532
17494168	Olfr601	olfactory receptor 601	5.362925	7.330543	5.391288	5.179526	3.959148	4.929188
17494170	Olfr603	olfactory receptor 603	4.658902	3.070024	2.67402	2.432989	3.229386	2.985784
17494172	Olfr605	olfactory receptor 605	5.146137	4.725248	5.95966	5.089486	5.512474	6.136177
17481205	Olfr606	olfactory receptor 606	2.497371	3.537892	3.118409	4.971634	3.539634	2.833608
17494175	Olfr607	olfactory receptor 607	2.402073	2.544284	2.307691	2.160867	2.725914	2.534463
17481207	Olfr608	olfactory receptor 608	2.524819	2.438257	2.67402	3.090071	3.567689	2.02229
17494177	Olfr609	olfactory receptor 609	5.236018	6.280067	6.788393	6.388424	5.67728	5.200355
17484569	Olfr61	olfactory receptor 61	2.925737	3.229185	3.16365	3.242912	4.377235	3.36249
17494179	Olfr610	olfactory receptor 610	2.829427	2.614545	3.277351	2.766726	2.906313	3.400837
17494181	Olfr611	olfactory receptor 611	3.360067	3.412881	3.128324	2.830369	3.398128	2.939763
17494183	Olfr612	olfactory receptor 612	8.245117	11.31567	6.850313	8.999345	12.15375	6.763359
17481209	Olfr613	olfactory receptor 613	3.470155	3.883991	4.913292	4.897583	4.122416	3.918387
17481213	Olfr615	olfactory receptor 615	4.181846	4.485162	4.026482	4.915438	5.573911	4.453038
17494185	Olfr616	olfactory receptor 616	2.688385	2.709225	2.516258	2.369315	2.153868	2.071822
17481215	Olfr617	olfactory receptor 617	4.992838	8.098417	7.278898	7.774932	7.922798	7.412704
17481217	Olfr618	olfactory receptor 618	2.67402	2.463791	3.307245	3.487492	4.419031	2.344142
17481219	Olfr619	olfactory receptor 619	2.172686	2.066845	2.033422	2.315558	2.199705	2.020175
17417812	Olfr62	olfactory receptor 62	4.559735	4.564921	4.874891	4.487059	5.755016	4.45276
17494187	Olfr620	olfactory receptor 620	2.45525	2.476341	2.150646	2.953407	3.423875	2.549545
17494189	Olfr622	olfactory receptor 622	4.776918	3.344962	3.71757	2.97919	3.344962	2.885057
17494193	Olfr623	olfactory receptor 623	2.39695	2.02917	1.982609	2.069023	1.96695	2.373112
17494197	Olfr624	olfactory receptor 624	3.232835	3.266578	3.009972	3.05616	3.211016	3.794948
17481223	Olfr625-ps1	olfactory receptor 625, pseudogene 1	2.45071	1.933574	2.962346	2.413254	3.284287	2.201093
17481227	Olfr628	olfactory receptor 628	2.776928	2.303855	2.358484	2.697251	2.089706	2.575835
17494199	Olfr629	olfactory receptor 629	3.673537	1.909202	1.896922	2.108446	2.14322	2.272452
17336075	Olfr63	olfactory receptor 63	5.539516	3.318658	4.691307	4.12193	3.44922	4.174403
17494203	Olfr630	olfactory receptor 630	3.062691	3.520572	4.533703	3.703322	3.880143	3.703322
17481240	Olfr631	olfactory receptor 631	3.00822	2.32845	2.68414	2.991129	3.622511	2.873701
17481248	Olfr632	olfactory receptor 632	4.719389	3.492863	3.696782	5.62608	3.873184	4.361336
17481250	Olfr633	olfactory receptor 633	2.94623	3.012518	3.164286	3.852353	2.729866	3.182477
17481252	Olfr635	olfactory receptor 635	1.977051	2.463433	2.652789	2.463706	2.710859	2.520465
17481254	Olfr638	olfactory receptor 638	2.441741	2.12731	2.869183	2.379288	4.358646	3.237926
17494263	Olfr639	olfactory receptor 639	3.467846	3.664154	3.766436	4.456064	3.44824	3.89151
17494256	Olfr64	olfactory receptor 64	2.917333	2.609801	2.909376	3.315347	3.066536	2.650492
17494267	Olfr640	olfactory receptor 640	2.521916	3.554287	3.810235	3.763044	3.554287	2.827878
17481256	Olfr641	olfactory receptor 641	3.728254	3.39121	2.978137	3.70032	3.126221	4.283411
17494273	Olfr642	olfactory receptor 642	4.018842	4.994603	3.899908	6.345043	4.501482	3.922871
17494277	Olfr643	olfactory receptor 643	2.638869	2.701424	2.229036	3.095452	2.507344	2.406756
17494296	Olfr643	olfactory receptor 643	4.753434	7.576807	4.952922	8.007212	6.404385	7.316583
17494282	Olfr644	olfactory receptor 644	3.5568	3.484834	4.519835	4.173073	6.824531	4.932982
17494286	Olfr645	olfactory receptor 645	2.773197	2.801365	2.249259	2.224498	2.459543	2.674965

17481258	Olfr646	olfactory receptor 646	5.624364	5.780603	5.765797	5.772075	3.617116	3.870849
17494315	Olfr648	olfactory receptor 648	3.08269	2.70281	3.381281	3.911359	2.423379	2.446908
17494319	Olfr649	olfactory receptor 649	2.374856	2.097092	2.359564	2.056384	2.163549	2.374856
17481236	Olfr65	olfactory receptor 65	4.886969	5.622298	4.886969	5.923295	5.145317	5.60747
17481309	Olfr651	olfactory receptor 651	1.907589	2.710427	2.218678	1.862706	2.479329	2.74341
17481313	Olfr652	olfactory receptor 652	2.613929	3.775532	3.540983	3.540983	3.490201	3.46177
17481317	Olfr653	olfactory receptor 653	3.286587	5.780603	3.339703	2.953407	4.543393	3.306168
17481320	Olfr654	olfactory receptor 654	2.921117	2.911253	2.483612	2.743314	2.82447	3.188969
17494422	Olfr655	olfactory receptor 655	4.490606	3.335169	3.398387	4.210583	4.195899	2.632366
17481324	Olfr656	olfactory receptor 656	5.462343	4.163249	6.121563	4.097233	3.37765	3.610503
17481326	Olfr657	olfactory receptor 657	3.662529	3.184043	2.850846	2.903615	2.717764	5.401949
17494424	Olfr658	olfactory receptor 658	2.691966	2.402623	2.591702	2.392751	3.077374	2.454876
17481328	Olfr659	olfactory receptor 659	2.624714	2.540795	2.544373	2.665821	2.362362	2.666782
17494254	Olfr66	olfactory receptor 66	5.034435	5.62413	3.554385	5.932376	6.874239	7.391976
17481330	Olfr661	olfactory receptor 661	7.6058	6.559564	5.620077	4.61551	4.450755	7.222258
17481332	Olfr663	olfactory receptor 663	2.655107	2.128696	2.065942	2.46945	3.122994	2.19325
17494429	Olfr664	olfactory receptor 664	2.298336	2.623768	2.156094	2.260904	2.573604	2.36079
17481340	Olfr665	olfactory receptor 665	5.338965	5.301865	5.30091	5.476484	9.446566	4.369475
17494443	Olfr666	olfactory receptor 666	2.122405	2.37619	2.701237	3.388273	2.458673	2.290734
17494445	Olfr667	olfactory receptor 667	3.83036	3.992715	3.774381	4.718375	4.216044	5.95999
17494447	Olfr668	olfactory receptor 668	2.424236	2.202421	2.62264	2.188921	2.188921	2.209439
17481342	Olfr669	olfactory receptor 669	1.934982	2.358615	2.481049	2.481049	3.24098	2.67402
17494216	Olfr67	olfactory receptor 67	2.220785	2.083415	2.101078	2.394675	2.362247	2.165049
17494451	Olfr670	olfactory receptor 670	3.74169	2.479312	3.088658	2.953407	2.782767	3.676696
17494453	Olfr671	olfactory receptor 671	4.427953	4.800351	5.848149	5.308889	4.246458	4.406979
17494455	Olfr672	olfactory receptor 672	3.651603	2.759143	3.78885	3.392174	3.143823	3.005865
17494457	Olfr675	olfactory receptor 675	1.844948	2.345198	2.302594	2.690753	3.122367	1.865006
17481344	Olfr676	olfactory receptor 676	3.590861	3.731977	3.884934	3.630605	6.320725	3.803796
17481346	Olfr677	olfactory receptor 677	3.082733	2.889721	2.962428	2.823863	2.424236	3.005177
17481348	Olfr678	olfactory receptor 678	3.308942	4.005382	4.02545	3.618571	3.46357	3.531179
17481350	Olfr679	olfactory receptor 679	4.233409	3.347305	4.922462	4.20927	4.541472	5.949465
17494214	Olfr68	olfactory receptor 68	2.67402	1.987796	2.271114	3.076799	2.461999	2.703522
17494459	Olfr680-ps1	olfactory receptor 680, pseudogene 1	2.930324	2.060051	2.587072	2.525082	2.901683	3.23111
17481352	Olfr681	olfactory receptor 681	6.206242	8.88924	6.29314	5.015175	6.413758	5.305468
17494462	Olfr682-ps1	olfactory receptor 682, pseudogene 1 [Source:MGI Symbol;Acc:MGI:3030516]	5.851474	4.697392	4.108552	3.742702	6.578369	4.013525
17494465	Olfr683	olfactory receptor 683	2.982764	3.379173	2.655052	3.378564	3.726187	2.932965
17494467	Olfr684	olfactory receptor 684	3.178729	2.592547	2.287799	2.790899	3.073239	3.255118
17494469	Olfr685	olfactory receptor 685	3.791267	3.351089	3.703322	4.224469	3.767742	3.554927
17494473	Olfr686	olfactory receptor 686	3.29037	3.898313	3.370354	3.336233	3.177539	3.46602
17481357	Olfr688	olfactory receptor 688	3.062691	4.933461	4.188838	4.110119	4.721548	3.258866
17481359	Olfr689	olfactory receptor 689	2.879284	3.11735	2.793202	3.552218	2.717368	3.342367
17494208	Olfr69	olfactory receptor 69	3.205146	2.212489	2.115575	2.268062	2.120097	2.381152
17494476	Olfr690	olfactory receptor 690	3.52255	4.745631	4.420716	4.332651	4.420716	5.454512
17494480	Olfr691	olfactory receptor 691	5.433192	2.992954	2.786067	3.66644	2.90732	2.489214
17481362	Olfr692	olfactory receptor 692	3.766488	5.478686	4.307587	3.468688	3.920669	4.167378
17494684	Olfr693	olfactory receptor 693	5.662386	5.460375	5.617429	5.496488	5.662386	5.460375
17494686	Olfr694	olfactory receptor 694	3.062691	2.05811	1.917438	1.820027	2.127929	2.217279
17494688	Olfr695	olfactory receptor 695	3.181903	2.656193	2.810467	3.767298	3.100391	2.660781
17494692	Olfr697	olfactory receptor 697	3.142015	2.434828	2.119465	2.350406	2.328256	2.5839
17494695	Olfr698	olfactory receptor 698	8.426246	6.265111	10.55958	6.40967	8.261879	8.551633
17494697	Olfr699	olfactory receptor 699	3.413354	3.443009	2.726103	3.90456	3.632644	3.89753
17424802	Olfr70	olfactory receptor 70	9.91237	9.45043	7.88352	9.502651	14.45223	9.346719
17494699	Olfr700	olfactory receptor 700	8.211467	7.794681	8.200376	8.489385	7.728616	14.19253
17481487	Olfr701	olfactory receptor 701	4.230388	4.74596	4.904853	3.892886	4.186487	4.879793
17494701	Olfr702	olfactory receptor 702	2.204849	2.066687	1.857394	2.066687	2.206087	1.787025
17481497	Olfr703	olfactory receptor 703	4.309738	3.688949	3.04259	3.001492	3.452975	3.304495
17481499	Olfr704	olfactory receptor 704	2.296314	2.334106	2.448741	2.417373	2.764503	2.904661
17494706	Olfr705	olfactory receptor 705	3.361395	3.54506	3.264744	3.053746	5.535755	4.605092
17494708	Olfr706	olfactory receptor 706	4.490419	3.44683	3.367271	4.207111	4.352065	3.308713
17494710	Olfr707	olfactory receptor 707	3.652439	2.775293	2.855889	2.458673	2.458673	2.445433
17494712	Olfr709-ps1	olfactory receptor 709, pseudogene 1 [Source:MGI Symbol;Acc:MGI:3030543]	1.955585	1.973477	2.241042	2.235039	3.088829	2.631654
17424807	Olfr71	olfactory receptor 71	7.934449	8.452452	7.877894	5.401462	5.740395	8.366418
17494714	Olfr710	olfactory receptor 710	2.693589	2.304989	2.605734	2.755836	2.609584	3.305641
17494719	Olfr711	olfactory receptor 711	2.993452	3.873694	4.083454	2.739951	3.219485	3.250046
17481504	Olfr713	olfactory receptor 713	2.953407	2.519103	2.953407	2.953407	2.782767	2.932132
17481506	Olfr714	olfactory receptor 714	2.985764	3.062691	2.809435	3.898746	3.263862	3.636524
17494731	Olfr715	olfactory receptor 715	2.330824	2.190242	2.141898	2.404122	2.629995	2.24767
17481510	Olfr716	olfactory receptor 716	2.134976	2.029339	2.259917	2.292481	2.275179	2.245241
17454787	Olfr718-ps1	olfactory receptor 718, pseudogene 1	5.570821	5.044391	5.853664	6.738605	5.853664	6.390682
17303589	Olfr720	olfactory receptor 720	2.364639	2.808676	2.32136	3.226992	3.213176	2.568133
17297225	Olfr721-ps1	olfactory receptor 721, pseudogene 1 [Source:MGI Symbol;Acc:MGI:3030555]	2.991751	2.935508	2.490767	3.264269	2.80671	2.952731
17305921	Olfr722	olfactory receptor 722	2.374132	2.22402	1.873986	2.479759	2.497942	2.473459
17305925	Olfr723	olfactory receptor 723	2.768587	3.401049	2.614165	3.368275	2.971744	2.468372
17305929	Olfr724	olfactory receptor 724	8.273513	8.459896	7.724759	12.65609	6.932521	7.683907
17305933	Olfr725	olfactory receptor 725	2.083992	2.073747	2.303711	2.257866	2.339483	2.075775
17305937	Olfr726	olfactory receptor 726	9.523488	9.523488	9.523488	9.523488	10.06859	12.28529
17299473	Olfr727	olfactory receptor 727	3.163036	3.338083	4.761843	5.746566	2.485197	2.646031
17305939	Olfr728	olfactory receptor 728	2.782767	2.965653	2.802239	2.782767	2.782767	2.68347
17305941	Olfr729	olfactory receptor 729	2.319204	2.066329	2.309803	1.891211	2.853001	2.610634
17387796	Olfr73	olfactory receptor 73	3.130016	2.613186	1.975804	2.406006	2.302546	2.082852
17305943	Olfr730	olfactory receptor 730	2.594614	2.747444	2.754404	2.46957	2.597331	2.354711
17305945	Olfr731	olfactory receptor 731	2.575906	2.591774	2.080601	2.032042	2.737066	2.356033
17305947	Olfr732	olfactory receptor 732	1.968259	1.898013	1.7279	1.749303	1.922602	1.813492
17305950	Olfr733	olfactory receptor 733	8.849341	7.723582	9.310251	7.532402	10.23501	8.995042

17305953	Olfr734	olfactory receptor 734	3.347421	3.350857	3.071897	3.219708	3.656593	3.558773
17305957	Olfr735	olfactory receptor 735	3.091271	2.679103	2.615524	3.133163	3.263862	3.268503
17299481	Olfr736	olfactory receptor 736	2.691891	2.169646	1.838208	2.419988	2.082346	1.90199
17299483	Olfr738	olfactory receptor 738	3.705453	3.145065	3.725257	3.458461	3.483216	3.764218
17299485	Olfr739	olfactory receptor 739	3.777967	3.779669	4.054938	4.228336	4.127162	4.551651
17387792	Olfr74	olfactory receptor 74	2.064811	2.17085	2.150422	2.587646	2.100642	2.387995
17299487	Olfr740	olfactory receptor 740	2.819873	2.817177	3.261013	2.163219	2.367805	2.38639
17299489	Olfr741	olfactory receptor 741	3.01768	2.739913	2.266537	1.810214	2.136338	2.296044
17299493	Olfr742	olfactory receptor 742	2.593014	2.262692	2.338413	2.728655	2.451509	2.707085
17299500	Olfr743	olfactory receptor 743	3.856869	3.590264	4.419643	4.734362	3.323654	3.720277
17299502	Olfr744	olfactory receptor 744	1.942857	2.424622	1.954893	2.572766	2.220277	2.345475
17299506	Olfr745	olfactory receptor 745	3.181925	2.955188	3.70286	3.405838	3.635138	4.076299
17299510	Olfr746	olfactory receptor 746	2.371073	2.147562	1.885347	2.503055	2.485128	2.418144
17305962	Olfr747	olfactory receptor 747	2.003635	1.976311	2.000804	1.988527	1.790236	1.944339
17299512	Olfr748	olfactory receptor 748	2.310428	2.217955	2.941333	2.705509	3.070045	3.671883
17305964	Olfr749	olfactory receptor 749	9.259152	6.258253	5.591014	7.395614	5.021297	7.018779
17265765	Olfr75-ps1	olfactory receptor 75, pseudogene 1	8.843025	9.342832	8.638735	10.90509	6.79536	11.28583
17306094	Olfr750	olfactory receptor 750	4.894936	6.315251	5.277154	5.372971	6.326511	5.66427
17363072	Olfr76	olfactory receptor 76	6.0367	6.579373	6.528674	6.86819	6.580148	7.909026
17344909	Olfr761	olfactory receptor 761	7.759964	7.355941	7.092528	7.695313	8.922265	7.374167
17238642	Olfr763	olfactory receptor 763	4.56644	4.739188	3.983758	4.839641	4.437724	3.55372
17246386	Olfr764-ps1	olfactory receptor 764, pseudogene 1	2.070616	1.87457	3.009784	2.347182	2.52734	2.262472
17246388	Olfr765	olfactory receptor 765	3.947365	5.159925	4.5152	2.681072	3.239385	4.131026
17246390	Olfr766	olfactory receptor 766	6.888692	5.223331	3.598212	7.381889	8.247461	6.079742
17246392	Olfr767	olfactory receptor 767	4.840748	5.169913	5.431309	6.407937	5.967679	5.48758
17246396	Olfr768	olfactory receptor 768	2.807216	3.229319	2.604615	4.016113	2.604615	3.795816
17246398	Olfr769	olfactory receptor 769	3.671908	2.544849	2.18604	2.591666	2.892386	2.16523
17514953	Olfr77	olfactory receptor 77	2.631819	2.647921	2.481049	2.726367	2.260293	2.623295
17246402	Olfr770	olfactory receptor 770	2.174404	2.880861	2.685927	1.886824	2.971229	2.879823
17246404	Olfr771	olfactory receptor 771	4.845985	4.441478	4.778673	4.086937	3.743817	4.474573
17246408	Olfr772	olfactory receptor 772	2.953407	3.009117	2.297906	2.953407	3.147399	2.204437
17246412	Olfr773	olfactory receptor 773	4.069665	2.259823	1.762447	2.457855	1.726942	2.266679
17238646	Olfr774	olfactory receptor 774	2.809357	2.17807	2.481049	2.054789	3.020191	3.905507
17238648	Olfr775	olfactory receptor 775	2.67402	2.67402	2.67402	3.703322	2.649904	2.67402
17238650	Olfr776	olfactory receptor 776	2.672074	1.85778	2.284835	1.831429	2.742763	2.175565
17246416	Olfr777	olfactory receptor 777	2.044092	1.947118	2.50191	1.682411	2.259588	2.259432
17494133	Olfr78	olfactory receptor 78	2.583183	2.578639	2.708455	2.657556	3.674123	2.953407
17238656	Olfr780	olfactory receptor 780	2.607505	2.092866	2.09349	2.422187	2.219124	2.270059
17238659	Olfr781	olfactory receptor 781	2.201841	2.406322	2.210358	2.235891	2.079908	2.267779
17238661	Olfr782	olfactory receptor 782	2.863263	1.709056	2.337425	2.348891	2.023047	2.40149
17238663	Olfr784	olfactory receptor 784	1.978326	2.098968	1.898013	2.778392	2.350015	2.220785
17238665	Olfr786	olfactory receptor 786	2.631125	2.701705	2.521706	3.003469	3.283057	2.399161
17238667	Olfr787	olfactory receptor 787	2.101923	3.151132	2.512546	2.691313	2.166956	2.706691
17238669	Olfr788	olfactory receptor 788	3.132751	2.638577	2.627954	2.529338	3.166524	4.165328
17238671	Olfr790	olfactory receptor 790	2.395007	2.697233	2.626716	2.86803	2.297094	2.463159
17238675	Olfr791	olfactory receptor 791	3.382055	2.122346	2.289734	2.428087	2.428087	2.288227
17238677	Olfr792	olfactory receptor 792	5.502625	4.20927	3.719555	4.084049	5.653326	4.20927
17238679	Olfr794	olfactory receptor 794	3.04905	3.38832	3.134836	3.134836	2.679846	3.581664
17246418	Olfr796	olfactory receptor 796	5.344519	7.182818	5.67315	7.951682	6.352833	11.27152
17246420	Olfr798	olfactory receptor 798	2.212504	2.020777	1.962279	1.928702	2.069898	1.908091
17238681	Olfr799	olfactory receptor 799	2.368658	1.864075	2.011093	2.041076	2.458673	2.282777
17514922	LOC100503048	olfactory receptor 7G2-like	2.757173	2.830957	2.820694	2.988476	3.42435	2.690175
17234850	Olfr8	olfactory receptor 8	7.276527	6.775418	11.71447	10.20589	13.19482	10.97683
17238683	Olfr800	olfactory receptor 800	3.30947	2.138664	2.05281	2.681983	4.010077	2.677302
17246422	Olfr801	olfactory receptor 801	2.894472	1.967413	2.522213	2.083357	2.909074	2.474454
17246424	Olfr802	olfactory receptor 802	1.729158	2.256098	2.790744	2.043284	3.558033	3.04103
17246426	Olfr803	olfactory receptor 803	2.728882	1.793316	1.770258	2.102477	2.64363	1.740546
17238654	Olfr804	olfactory receptor 804	3.304954	2.953407	2.953407	2.913514	3.406051	3.167534
17238687	Olfr804	olfactory receptor 804	2.304654	1.785638	2.053977	2.282492	2.171121	2.054689
17246428	Olfr805	olfactory receptor 805	1.980384	1.887531	2.135435	2.014735	2.327192	1.681851
17246430	Olfr806	olfactory receptor 806	3.351252	2.564149	2.163429	2.573301	2.57207	2.107014
17246432	Olfr807	olfactory receptor 807	2.417473	2.682559	2.674909	2.770737	3.213889	2.948028
17238689	Olfr808	olfactory receptor 808	2.804882	2.115326	2.75074	3.058067	3.699653	2.766841
17238691	Olfr809	olfactory receptor 809	2.548468	2.214253	2.067017	1.918621	2.02539	2.062208
17246435	Olfr810	olfactory receptor 810	2.35494	4.253027	2.911898	2.812533	3.153513	2.673186
17246438	Olfr811	olfactory receptor 811	5.395625	3.222857	3.396997	4.365237	4.915983	2.606909
17246440	Olfr812	olfactory receptor 812	2.356736	2.483561	2.309883	2.335708	2.334429	2.808637
17238695	Olfr813	olfactory receptor 813	2.003149	1.797859	1.901608	2.032154	2.121964	2.109952
17246443	Olfr814	olfactory receptor 814	2.773466	1.918768	2.013534	2.523944	1.928247	1.933762
17246445	Olfr815	olfactory receptor 815	2.295996	2.258508	2.214621	2.492062	2.76008	2.286816
17246447	Olfr816	olfactory receptor 816	4.495028	2.184919	1.994214	2.304206	2.89391	2.195151
17246449	Olfr818	olfactory receptor 818	6.107619	3.352994	4.320535	5.146601	5.75749	4.743724
17246451	Olfr819	olfactory receptor 819	6.074392	5.748717	5.231084	5.463252	6.733283	7.828251
17238697	Olfr820	olfactory receptor 820	4.238313	2.554729	2.894512	2.894512	3.098779	3.198466
17238701	Olfr821	olfactory receptor 821	2.010118	3.630857	2.493116	2.074552	3.010869	2.10906
17238703	Olfr822	olfactory receptor 822	2.136856	2.255645	1.84266	2.196247	2.437125	2.31816
17246457	Olfr823	olfactory receptor 823	2.470392	2.415179	2.675429	2.083357	1.706547	1.830554
17246459	Olfr824	olfactory receptor 824	4.547489	3.263862	3.756398	3.826698	3.617116	3.866237
17246461	Olfr825	olfactory receptor 825	1.675719	4.407315	5.948558	2.632603	4.732164	3.019187
17246464	Olfr826	olfactory receptor 826	2.496402	2.591648	2.159938	2.526622	2.410713	2.211875
17246466	Olfr827	olfactory receptor 827	2.394028	2.323887	2.197312	2.586283	2.016467	2.338413
17524326	Olfr828	olfactory receptor 828	2.573675	3.54506	2.99003	3.46739	3.415886	2.712268
17514908	Olfr829	olfactory receptor 829	3.007511	2.521793	3.010827	2.575174	2.625842	2.606909
17514910	Olfr830	olfactory receptor 830	3.03343	2.908832	3.763774	2.38773	4.062985	2.955577

17514912	Olfr832	olfactory receptor 832	2.012948	1.79195	1.966486	2.265611	2.267889	2.076509
17514914	Olfr834	olfactory receptor 834	2.581626	2.004441	2.010578	2.148232	1.98232	2.050378
17514916	Olfr835	olfactory receptor 835	2.452376	2.897985	2.536484	2.665359	4.214817	2.255004
17514918	Olfr836	olfactory receptor 836	1.977681	2.145792	2.636401	1.954379	1.902623	2.108475
17514920	Olfr837	olfactory receptor 837	2.579515	2.32429	2.2003	2.113992	1.781213	2.150392
17524328	Olfr843	olfactory receptor 843	1.623863	2.094433	1.721934	1.845037	2.831193	2.033902
17514924	Olfr845	olfactory receptor 845	3.556406	4.224323	3.071684	3.796105	3.729055	3.482588
17524332	Olfr846	olfactory receptor 846	2.015363	1.739268	1.968559	2.614473	3.210438	2.079894
17524334	Olfr847	olfactory receptor 847	3.578066	2.420844	1.99543	2.420844	2.625023	2.420844
17514928	Olfr849	olfactory receptor 849	2.435149	2.392137	2.332117	2.348256	2.245645	2.332117
17524336	Olfr850	olfactory receptor 850	4.66585	4.170383	3.88884	3.60203	3.688591	3.070216
17514930	Olfr851	olfactory receptor 851	2.559763	2.586498	2.609277	2.513382	2.352851	2.653893
17524338	Olfr853	olfactory receptor 853	3.797211	3.307337	3.099789	3.276942	3.704477	3.062055
17524340	Olfr854	olfactory receptor 854	2.965016	2.527498	3.294227	3.7169	3.17272	3.12241
17514933	Olfr855	olfactory receptor 855	3.062691	2.312142	3.455537	2.739913	3.729055	2.576174
17524342	Olfr856-ps1	olfactory receptor 856, pseudogene 1	2.357732	3.43281	2.631545	2.558166	4.028548	4.063464
17514946	Olfr857	olfactory receptor 857	2.388359	2.72839	2.750493	2.286483	2.578335	3.120874
17514951	Olfr859	olfactory receptor 859	5.48583	4.049995	5.157207	5.243172	6.160255	6.391258
17524344	Olfr860	olfactory receptor 860	3.062691	2.367296	1.898013	2.132832	2.968656	2.047822
17524348	Olfr862	olfactory receptor 862	1.849211	2.019195	1.814975	2.095058	2.188921	2.087636
17524351	Olfr866	olfactory receptor 866	2.37698	2.049426	2.202436	2.522965	2.294532	2.039577
17524355	Olfr867	olfactory receptor 867	2.94719	2.235581	2.103089	2.026556	2.190621	1.966459
17514958	Olfr868	olfactory receptor 868	2.437615	2.463808	2.19812	2.724195	2.994489	2.957237
17514961	Olfr869	olfactory receptor 869	7.106356	12.35891	4.23426	3.87931	5.682753	7.179035
17524357	Olfr870	olfactory receptor 870	2.838011	2.953407	3.703322	3.703322	2.547125	2.953407
17514967	Olfr871	olfactory receptor 871	2.673037	2.398163	1.607323	2.962387	2.306028	2.012292
17514971	Olfr872	olfactory receptor 872	3.462322	2.437327	2.953407	2.886337	2.791305	2.953407
17514980	Olfr873	olfactory receptor 873	1.916561	1.869095	1.898013	2.210358	2.083299	2.255051
17516110	Olfr874	olfactory receptor 874	1.926524	1.871532	2.024043	1.969269	2.118539	1.850301
17516112	Olfr875	olfactory receptor 875	4.570969	7.814318	5.113532	3.632997	8.013264	3.971517
17516115	Olfr876	olfactory receptor 876	2.500593	3.244441	2.782767	3.389189	2.782767	2.377903
17516118	Olfr877	olfactory receptor 877	3.097577	3.540689	4.461318	5.205476	2.924216	3.131166
17516124	Olfr878	olfactory receptor 878	2.969521	2.347199	2.881999	2.527621	3.091892	3.47815
17516127	Olfr881	olfactory receptor 881	3.200084	2.237473	3.16433	2.526657	3.263839	2.606909
17516129	Olfr883	olfactory receptor 883	2.406006	2.953673	2.944025	2.897302	3.027548	2.953407
17516131	Olfr884	olfactory receptor 884	6.551204	6.322828	6.241358	4.888866	5.68358	4.456744
17516133	Olfr885	olfactory receptor 885	2.538049	2.449318	2.449318	2.676727	2.160507	2.213348
17516135	Olfr887	olfactory receptor 887	6.751883	5.602186	3.787485	3.604802	3.787485	2.847054
17516137	Olfr888	olfactory receptor 888	2.020791	2.104431	2.214084	2.293499	2.361478	2.112176
17516139	Olfr889	olfactory receptor 889	2.162305	2.366607	1.950995	1.825485	2.534147	2.0363
17516141	Olfr890	olfactory receptor 890	2.135213	2.957053	2.040666	2.574389	2.752667	2.526622
17525690	Olfr891	olfactory receptor 891	2.647022	3.024947	2.674761	2.811656	2.695401	2.415865
17516143	Olfr893	olfactory receptor 893	2.591612	2.272169	2.661593	2.610091	2.481203	3.30736
17516145	Olfr894	olfactory receptor 894	3.462322	1.844922	1.996994	2.883858	1.978847	1.947617
17516149	Olfr895	olfactory receptor 895	2.846364	3.916622	3.121133	2.745673	2.330404	2.47267
17516155	Olfr898	olfactory receptor 898	3.339703	4.142639	4.026036	2.98771	3.367855	3.339703
17238638	Olfr9	olfactory receptor 9	3.885553	3.822244	3.520011	7.613237	5.746207	5.662857
17344829	Olfr90	olfactory receptor 90	2.802744	1.990595	2.205644	2.65704	2.268235	2.551968
17516164	Olfr901	olfactory receptor 901	5.98541	5.98541	5.129755	5.835232	4.72515	9.721372
17516166	Olfr902	olfactory receptor 902	1.929437	2.160777	2.133526	2.345442	2.095508	2.360856
17516170	Olfr904	olfactory receptor 904	3.15837	2.357896	2.19214	2.255332	2.845259	2.694617
17516172	Olfr905	olfactory receptor 905	2.564078	2.297731	1.944973	2.504895	2.36079	2.36079
17516174	Olfr906	olfactory receptor 906	2.569469	2.420844	2.318208	1.697228	2.155332	2.666191
17516176	Olfr907	olfactory receptor 907	2.842578	2.828525	2.92055	3.389424	2.759755	2.243685
17516180	Olfr908	olfactory receptor 908	2.606909	2.577764	2.606909	2.606909	5.445747	2.020147
17344833	Olfr91	olfactory receptor 91	6.487758	6.771944	5.858738	4.518644	5.617079	5.952559
17516184	Olfr910	olfactory receptor 910	1.981867	2.122155	1.586503	1.843823	2.02525	1.898013
17516182	Olfr911-ps1	olfactory receptor 911, pseudogene 1	2.419451	2.178402	2.026879	2.620387	3.465371	2.094608
17516186	Olfr912	olfactory receptor 912	12.35686	7.574864	9.53855	9.450365	13.92804	8.707207
17516189	Olfr913	olfactory receptor 913	2.343037	1.975544	2.021071	2.929308	2.782767	2.143621
17516192	Olfr914	olfactory receptor 914	5.829289	10.21134	4.568055	6.287864	6.719156	4.714027
17525692	Olfr915	olfactory receptor 915	1.944892	2.632384	2.191912	2.082519	3.943236	2.447977
17525694	Olfr916	olfactory receptor 916	2.232376	2.481049	2.481049	2.481049	3.061206	2.514741
17525696	Olfr917	olfactory receptor 917	3.536176	6.83021	4.151665	6.366939	3.091635	4.083001
17525698	Olfr918	olfactory receptor 918	3.135249	3.194699	2.464645	2.312527	2.060965	2.062551
17525700	Olfr919	olfactory receptor 919	4.845515	3.696321	4.269361	4.571634	5.060921	6.561884
17344835	Olfr92	olfactory receptor 92	4.750239	4.219319	6.022824	6.574814	6.20749	6.129375
17516217	Olfr920	olfactory receptor 920	3.155744	3.402794	2.788849	2.607903	2.921117	2.911838
17516222	Olfr921	olfactory receptor 921	2.303009	2.208352	2.152361	2.23428	2.198455	2.566763
17516227	Olfr922	olfactory receptor 922	2.435453	2.239691	2.243234	2.249696	2.590984	2.815068
17516231	Olfr923	olfactory receptor 923	2.620242	2.67402	1.947806	2.704271	2.67402	3.072643
17516235	Olfr924	olfactory receptor 924	2.258038	2.525817	1.697263	2.398662	2.241897	2.245941
17516239	Olfr926	olfactory receptor 926	1.915817	1.999986	2.045509	2.161316	2.458673	2.642622
17344837	Olfr93	olfactory receptor 93	13.89824	13.24109	10.77996	17.16181	13.35594	16.65554
17516243	Olfr930	olfactory receptor 930	3.22574	2.561502	2.558308	3.421716	2.981999	2.757135
17516247	Olfr933	olfactory receptor 933	3.062691	2.204742	2.230381	2.304206	2.031929	2.420508
17525702	Olfr934	olfactory receptor 934	2.871153	2.170624	2.890983	2.759066	3.936109	2.606909
17525704	Olfr935	olfactory receptor 935	5.00913	4.903222	3.928994	4.673197	3.536617	3.277305
17525710	Olfr936	olfactory receptor 936	2.248854	1.751268	1.951996	2.06391	2.138768	2.122626
17525712	Olfr937	olfactory receptor 937	2.348501	2.260983	2.261406	2.955639	2.819169	2.616068
17525714	Olfr938	olfactory receptor 938	2.152764	1.970293	1.789703	2.111883	2.188921	2.066501
17344846	Olfr94	olfactory receptor 94	3.10009	4.294857	4.173535	4.709618	3.613408	3.318497
17516257	Olfr943	olfactory receptor 943	2.132432	2.67402	2.481049	2.470187	2.203016	2.96095
17516261	Olfr944	olfactory receptor 944	3.053492	2.818329	2.765212	3.805141	3.183933	4.485037

17525720	Olfr945	olfactory receptor 945	3.764557	3.19295	2.733748	3.446232	3.071152	2.836142
17525722	Olfr947-ps1	olfactory receptor 947, pseudogene 1	3.193614	3.226232	2.665526	3.202037	4.912509	3.172082
17525725	Olfr948	olfactory receptor 948	2.887078	2.190226	2.441571	2.461674	2.395256	2.79448
17344850	Olfr95	olfactory receptor 95	5.718632	6.045954	8.670108	5.568775	5.192035	7.637762
17516263	Olfr951	olfactory receptor 951	2.606276	2.397981	2.298416	2.423849	3.246736	2.348094
17525727	Olfr952	olfactory receptor 952	5.469542	3.988123	4.827111	4.149737	6.353537	5.346482
17516265	Olfr954	olfactory receptor 954	3.080021	2.159594	2.261359	2.808072	2.902186	2.575335
17525731	Olfr955	olfactory receptor 955	5.176547	4.334784	5.94353	4.402369	3.225896	4.074181
17525736	Olfr957	olfactory receptor 957	8.646522	5.11222	5.669651	5.110874	5.735264	3.427199
17525740	Olfr958	olfactory receptor 958	4.462524	4.179731	4.462524	4.462524	3.59896	4.788056
17525742	Olfr959	olfactory receptor 959	2.67402	2.750073	3.95454	3.324483	3.009972	3.009972
17337549	Olfr96	olfactory receptor 96	3.646949	3.437882	3.437882	3.520572	3.437882	3.437882
17516271	Olfr960	olfactory receptor 960	4.032236	4.059888	3.339703	3.625852	4.448041	3.557836
17516274	Olfr961	olfactory receptor 961	3.631561	4.43259	3.87042	3.066728	3.566132	3.566132
17516276	Olfr963	olfactory receptor 963	3.789612	2.348501	3.566156	3.263862	2.952772	2.996441
17516278	Olfr965	olfactory receptor 965	2.206439	2.55395	1.997589	2.802239	2.406006	3.339703
17516282	Olfr967	olfactory receptor 967	2.235085	2.08917	1.905725	1.927339	3.797079	2.109469
17525780	Olfr968	olfactory receptor 968	4.838098	3.803638	3.200506	4.722857	3.204924	5.622298
17516284	Olfr969	olfactory receptor 969	1.809423	1.807054	2.083357	1.931083	2.034875	2.072138
17344852	Olfr97	olfactory receptor 97	2.760941	3.263862	2.481049	4.548623	2.515282	4.821293
17516286	Olfr970	olfactory receptor 970	2.109864	1.852213	1.747945	2.083357	2.304574	2.18162
17516288	Olfr971	olfactory receptor 971	2.446518	2.260215	2.220031	2.821202	2.403022	2.265234
17516290	Olfr972	olfactory receptor 972	2.941108	1.995403	2.59924	1.943086	2.83084	2.727237
17516294	Olfr974	olfactory receptor 974	3.752937	3.321535	2.72027	2.594075	3.000432	2.647829
17525782	Olfr975	olfactory receptor 975	3.989755	4.817551	4.688999	4.109378	3.917437	3.802136
17525784	Olfr976	olfactory receptor 976	3.544126	2.795837	3.271291	2.96173	3.422499	3.347305
17516298	Olfr978	olfactory receptor 978	11.81322	9.731957	11.39998	7.798464	8.420232	9.712751
17525788	Olfr979	olfactory receptor 979	2.383382	1.848673	2.229376	2.060608	2.801307	2.636109
17344855	Olfr98	olfactory receptor 98	6.627202	5.651093	9.298706	5.302747	7.809986	5.169125
17525791	Olfr980	olfactory receptor 980	2.691033	2.331664	2.705734	2.428104	3.295963	2.18094
17516300	Olfr981	olfactory receptor 981	3.009972	3.345264	2.739647	2.882619	2.464116	4.41949
17516305	Olfr982	olfactory receptor 982	3.63549	4.161028	3.371522	3.29037	4.658676	2.736819
17525794	Olfr983	olfactory receptor 983	2.706147	2.202436	2.790551	2.380888	2.85302	3.546436
17525797	Olfr984	olfactory receptor 984	2.255364	2.178779	2.588148	2.006763	2.439355	2.309739
17525799	Olfr985	olfactory receptor 985	3.182874	3.264586	4.273802	3.92317	2.876033	3.229095
17516316	Olfr986	olfactory receptor 986	4.091444	2.778873	2.88912	5.007568	4.387837	4.192527
17387571	Olfr987	olfactory receptor 987	3.188549	3.232723	4.015752	3.208525	3.114952	2.647902
17387578	Olfr988	olfactory receptor 988	4.405513	4.944724	8.514135	5.002329	3.739071	4.669798
17344860	Olfr99	olfactory receptor 99	2.320716	2.171467	2.62912	3.155612	2.977559	3.473259
17387583	Olfr992	olfactory receptor 992	1.863804	1.819018	2.086883	2.10035	3.263862	2.293038
17387585	Olfr993	olfactory receptor 993	2.364197	2.866499	2.364197	2.364197	2.646967	2.674798
17387587	Olfr994	olfactory receptor 994	2.406006	2.956192	3.063541	2.215619	2.203841	2.091387
17387589	Olfr995	olfactory receptor 995	2.375037	2.124274	1.946875	2.387168	2.471608	2.265831
17372744	Olfr996	olfactory receptor 996	2.162035	2.230118	1.747945	2.720006	2.054162	2.3059
17372747	Olfr998	olfactory receptor 998	2.550836	2.420844	2.104226	2.060894	2.582557	2.176545
17266586	Omg	oligodendrocyte myelin glycoprotein	3.851579	4.726263	4.358646	3.851579	5.399927	4.072402
17327035	Olig1	oligodendrocyte transcription factor 1	14.31296	17.12961	17.33036	18.26209	12.80806	19.27822
17327030	Olig2	oligodendrocyte transcription factor 2	5.89536	7.299411	7.930875	7.9908	8.741374	6.945651
17231855	Olig3	oligodendrocyte transcription factor 3	3.331103	4.087419	3.413472	3.564402	2.868268	3.384892
17364588	Opalin	oligodendrocytic myelin paranodal and inner loop protein	2.868805	3.547174	3.128433	3.405343	2.959698	3.765601
17365493	Obfc1	oligonucleotide/oligosaccharide-binding fold containing 1	9.351968	6.455595	8.307187	9.502519	8.059442	9.840762
17543407	Ophn1	oligophrenin 1	8.101505	11.68706	12.22134	8.044652	14.23539	12.11792
17446760	Ost4	oligosaccharyltransferase 4 homolog (S. cerevisiae)	164.5212	138.6669	141.723	126.342	130.6714	119.6304
17548444	Ost4	oligosaccharyltransferase 4 homolog (S. cerevisiae)	873.3645	831.1874	774.6281	741.9098	743.0214	684.549
17410376	Ostc	oligosaccharyltransferase complex subunit	146.5653	103.7351	110.8281	101.2487	107.8947	82.44728
17536818	Ogt	O-linked N-acetylglucosamine (GlcNAc) transferase (UDP-N-acetylglucosamine:pol	73.22579	89.73009	94.57454	90.40245	92.29752	94.57454
17416141	Oma1	OMA1 homolog, zinc metallopeptidase (S. cerevisiae)	36.73542	30.82139	34.0129	36.99222	34.29437	35.70811
17454936	Ocm	oncomodulin	3.402865	3.326004	3.56905	4.801649	3.402865	3.955006
17303442	Oit1	oncoprotein induced transcript 1	4.391945	4.753467	4.48078	4.820958	5.563952	5.948929
17241001	Oit3	oncoprotein induced transcript 3	82.5382	70.13779	81.72072	64.36971	43.53851	45.50048
17246803	Osm	oncostatin M	7.107736	6.573811	5.366532	5.466396	4.32395	6.164398
17315743	Osmr	oncostatin M receptor	18.20168	22.62789	27.31251	18.65034	20.86582	21.66043
17519357	Onecut1	one cut domain, family member 1	47.55978	43.43542	41.42788	21.20746	22.29673	12.1013
17351273	Onecut2	one cut domain, family member 2	78.36785	81.68674	78.83203	69.30944	83.86925	59.71784
17235360	Onecut3	one cut domain, family member 3	15.28262	17.75779	18.38096	20.05722	19.1241	18.35689
17529035	Ooep	oocyte expressed protein	17.19813	17.19813	17.12806	21.91702	19.45474	16.77615
17529003	Omt2a	oocyte maturation, alpha	3.446089	3.673206	3.8978	3.646191	3.720019	3.261239
17519692	Omt2b	oocyte maturation, beta	2.030986	2.327353	2.457515	2.190803	2.847212	2.292815
17363016	Oosp1	oocyte secreted protein 1	3.259205	4.063605	3.334591	3.164856	3.225784	3.326627
17363009	Oosp2	oocyte secreted protein 2	4.01461	3.54506	4.256035	3.744544	3.418397	4.130997
17357719	Oosp3	oocyte secreted protein 3	4.226226	3.867497	3.112211	4.609691	3.088936	3.889487
17473971	Obox1	oocyte specific homeobox 1	4.123244	2.436905	2.443247	2.378118	2.422136	2.370728
17473959	Obox2	oocyte specific homeobox 2	3.179368	3.824232	3.526776	5.492451	4.106929	5.296576
17486748	Obox3	oocyte specific homeobox 3	4.688999	4.093089	4.127791	4.571317	7.631518	7.381121
17473985	Obox4-ps33	oocyte specific homeobox 4, pseudogene 33	13.39423	11.75856	13.53422	13.3947	11.89102	13.70547
17473988	Obox5	oocyte specific homeobox 5	3.248289	4.413184	5.240048	3.030278	4.113254	3.356924
17486764	Obox6	oocyte specific homeobox 6	5.766196	5.078069	4.430809	5.143392	5.050408	5.2427
17473948	Obox7	oocyte specific homeobox 7	2.719761	2.702061	2.493427	2.729222	2.653709	3.43143
17486704	Obox8	oocyte specific homeobox 8 [Source:MGI Symbol;Acc:MGI:3645855]	4.681043	4.20927	4.012858	4.681043	4.364541	5.85614
17277646	Oog1	oogenesin 1	3.118646	3.513917	2.882918	3.417449	4.380939	5.604828
17421271	Oog2	oogenesin 2	6.12861	3.379758	5.543857	4.715955	3.467846	4.352065
17432514	Oog3	oogenesin 3	2.513609	3.909001	3.149123	3.869776	2.801831	3.778229
17432449	Oog4	oogenesin 4	2.378183	2.116323	2.75681	2.393978	2.91517	2.579801
17389920	Oip5	Opa interacting protein 5	3.193238	3.385855	3.119533	2.717236	3.193238	3.193238

17515653	Opcml	opioid binding protein/cell adhesion molecule-like	4.01461	4.518926	4.01461	3.775977	4.570114	4.218266
17380799	Ogfr	opioid growth factor receptor	67.56527	53.6494	57.59014	71.48259	62.17618	63.83342
17221756	Ogfrl1	opioid growth factor receptor-like 1	22.34639	17.05118	15.54383	18.51445	28.05411	25.20765
17430762	Oprd1	opioid receptor, delta 1	11.04101	8.798881	9.532469	9.568084	9.784174	8.927276
17210904	Oprk1	opioid receptor, kappa 1	3.744596	4.030671	3.548034	3.470155	4.12905	3.038796
17231495	Oprm1	opioid receptor, mu 1	4.681043	4.177269	5.06752	4.681043	4.681043	4.681043
17381128	Oprl1	opioid receptor-like 1	5.077541	7.703371	5.617429	6.141538	4.352065	5.51928
17535722	Opn1mw	opsin 1 (cone pigments), medium-wave-sensitive (color blindness, deutan)	3.062691	3.600532	2.867732	3.137488	2.458673	3.289686
17465396	Opn1sw	opsin 1 (cone pigments), short-wave-sensitive (color blindness, tritan)	7.153156	6.588956	8.353438	7.504105	7.32658	6.333882
17230166	Opn3	opsin 3	12.86518	12.14129	11.63686	10.44767	13.44185	12.16943
17305091	Opn4	opsin 4 (melanopsin)	8.856459	7.80041	10.14397	8.356912	6.949841	8.783038
17345007	Opn5	opsin 5	4.856847	5.014619	4.20927	5.110095	4.368173	4.683574
17324583	Opa1	optic atrophy 1	133.8163	122.1144	109.6947	120.7802	116.891	130.2798
17474534	Opa3	optic atrophy 3	42.1681	40.89039	44.90331	39.85918	39.53449	41.41926
17227063	Optc	opticin	8.850445	10.11848	9.889859	7.949587	6.732582	8.578944
17381330	Optn	optineurin	77.17705	91.64981	89.32054	91.21632	76.12561	91.09437
17442177	Orai1	ORAI calcium release-activated calcium modulator 1	14.57749	15.2231	17.80771	12.78305	15.2231	11.63646
17453698	Orai2	ORAI calcium release-activated calcium modulator 2	15.776	14.9247	13.97969	19.38072	13.18513	12.24228
17483437	Orai3	ORAI calcium release-activated calcium modulator 3	23.69449	21.34432	28.25472	30.78744	29.26714	25.07208
17485449	Oraov1	oral cancer overexpressed 1	16.41372	13.01136	10.63509	13.39423	13.6175	13.28835
17546016	Ofd1	oral-facial-digital syndrome 1 gene homolog (human)	6.483667	6.632028	5.515111	6.191762	6.030928	7.287379
17418519	Oscp1	organic solute carrier partner 1	6.703525	6.992896	6.992896	6.992896	6.235002	9.228013
17416690	Orc1	origin recognition complex, subunit 1	4.312068	3.702064	3.703322	3.487468	3.476944	3.315186
17223390	Orc2	origin recognition complex, subunit 2	17.82389	14.82551	17.69684	18.77902	14.68275	24.26559
17423823	Orc3	origin recognition complex, subunit 3	40.94854	41.11207	49.00034	38.22779	36.8318	39.86001
17384994	Orc4	origin recognition complex, subunit 4	38.84185	38.6659	32.06772	36.3794	37.01428	34.36124
17445996	Orc5	origin recognition complex, subunit 5	29.22498	27.61461	30.07682	26.53565	30.94576	33.80864
17503431	Orc6	origin recognition complex, subunit 6	9.032717	10.31878	9.960651	8.925482	13.44698	9.607027
17212821	Ormdl1	ORM1-like 1 (S. cerevisiae)	28.39313	28.59518	25.68261	30.6433	22.36127	34.80735
17246345	Ormdl2	ORM1-like 2 (S. cerevisiae)	34.44422	24.27804	25.56114	30.91768	34.28938	30.1643
17268834	Ormdl3	ORM1-like 3 (S. cerevisiae)	61.99844	51.88732	44.23235	55.01183	65.54255	49.24652
17497091	Oat	ornithine aminotransferase	953.3089	1081.194	956.1015	1353.566	813.1795	1447.051
17235462	Oaz1	ornithine decarboxylase antizyme 1	71.65572	66.02864	68.47569	99.77455	69.3979	78.2159
17341240	Oaz1-ps	ornithine decarboxylase antizyme 1, pseudogene	928.2696	806.6617	829.1964	857.6053	752.976	823.6799
17518628	Oaz2	ornithine decarboxylase antizyme 2	8.834999	10.69756	9.233387	4.827579	10.5639	10.37874
17548287	LOC102633210	ornithine decarboxylase antizyme 2 pseudogene	23.92505	24.55287	33.28476	32.71885	26.75061	28.68133
17407542	Oaz3	ornithine decarboxylase antizyme 3	4.051623	3.7194	3.259024	4.317811	3.88235	4.575312
17274294	Odc1	ornithine decarboxylase, structural 1	54.59587	56.01029	51.16126	47.06654	54.06713	56.21215
17533253	Otc	ornithine transcarbamylase	1060.045	814.189	715.1638	868.6854	776.6496	856.1561
17291992	Ofcc1	orofacial cleft 1 candidate 1	4.74935	4.789749	5.859754	4.548403	4.548403	4.469303
17414729	Orm1	orosomucoid 1	704.1017	779.1137	795.8595	673.428	778.2609	674.6334
17414747	Orm2	orosomucoid 2	108.8607	259.3614	416.9383	177.251	360.2463	339.9267
17414738	Orm3	orosomucoid 3	23.46421	29.86989	43.6494	38.55297	27.73181	29.79297
17260959	Otx1	orthodenticle homolog 1	23.19754	23.16813	23.25582	25.99658	20.62611	23.86939
17305830	Otx2	orthodenticle homolog 2	2.989885	4.142151	3.703322	4.279701	3.729055	3.338245
17299443	Otx2os1	orthodenticle homolog 2 opposite strand 1	4.595558	3.980804	4.13819	4.390301	4.114651	4.329739
17289245	Otp	orthopedia homolog (Drosophila)	9.685792	9.78994	9.361307	12.2444	9.361307	12.36765
17306054	Osgep	O-sialoglycoprotein endopeptidase	16.90373	17.36523	21.24778	14.3526	20.14835	19.99725
17212828	Osgepl1	O-sialoglycoprotein endopeptidase-like 1	49.52792	45.96201	43.24795	57.27605	49.81195	51.6512
17485520	Oscar	osteoclast associated receptor	2.663863	3.94299	4.423628	4.369627	4.141175	3.01948
17363374	Ostf1	osteoclast stimulating factor 1	91.4683	99.08536	104.9123	87.22864	96.38054	99.68952
17394454	Ocstamp	osteoclast stimulatory transmembrane protein	10.96725	18.36071	11.3522	12.55578	12.47554	12.01979
17324546	Ostn	osteocrin	3.197269	3.143758	4.036262	3.029858	3.46888	4.313413
17287175	Ogn	osteoglycin	6.158334	4.211546	5.831108	4.865338	4.028269	5.435151
17287170	Omd	osteomodulin	6.542128	3.405697	2.412	5.639472	6.463161	5.095628
17232937	Ostm1	osteopetrosis associated transmembrane protein 1	18.75873	17.14362	21.32967	20.44676	19.51566	22.12139
17482634	Otoa	otoancorin	3.618546	3.887223	3.526849	3.526849	3.823278	3.496811
17317555	Oc90	otoconin 90	4.545062	4.574836	4.477738	5.505067	4.134979	4.132257
17446688	Otof	otoferlin	13.38096	10.79866	9.275468	11.42609	9.614554	10.16607
17478116	Otog	otogelin	8.270474	9.451085	8.895835	9.292263	6.992896	9.457442
17244785	Otogl	otogelin-like	3.480344	3.354784	3.389189	3.263862	3.263862	2.935141
17398308	Otol1	otolin 1 homolog (zebrafish)	8.993047	7.769167	9.664801	10.00445	9.67177	9.004711
17437097	Otop1	otopetrin 1	5.393605	6.751228	5.513544	5.508655	4.549191	4.464319
17258155	Otop2	otopetrin 2	9.287627	8.356739	7.983603	8.504757	8.578706	9.564172
17258168	Otop3	otopetrin 3	4.471906	5.086383	5.225576	5.837376	6.168843	6.875144
17376988	Otor	otoraplin	6.144987	8.048835	6.304841	6.943774	5.766476	4.763295
17225595	Otos	otospiralin	8.011431	9.596046	8.380115	6.607296	7.40762	7.871999
17316189	Otulin	OTU deubiquitinase with linear linkage specificity	55.29243	52.43943	49.94436	61.41631	56.71939	52.09453
17367385	Otud1	OTU domain containing 1	25.36434	21.38445	14.84052	21.73367	18.61921	19.315
17431841	Otud3	OTU domain containing 3	21.85604	17.664	16.27606	14.70087	18.61121	17.555
17502754	Otud4	OTU domain containing 4	82.7731	104.083	93.79181	93.55417	89.28278	92.35064
17533015	Otud5	OTU domain containing 5	51.56606	52.03931	49.80574	51.56606	58.69436	49.9281
17536517	Otud6a	OTU domain containing 6A	9.933692	8.51685	8.51685	7.902724	9.536435	10.12289
17423433	Otud6b	OTU domain containing 6B	113.9897	114.7412	110.3299	115.4032	124.0897	119.0868
17478801	Otud7a	OTU domain containing 7A	6.02249	5.717799	6.84718	5.387739	4.266846	5.717799
17400467	Otud7b	OTU domain containing 7B	25.32973	28.56111	32.74176	25.84942	26.82526	23.11921
17362261	Otub1	OTU domain, ubiquitin aldehyde binding 1	51.62615	63.29502	63.12633	53.26626	65.81753	60.74863
17278188	Otub2	OTU domain, ubiquitin aldehyde binding 2	10.29541	6.928774	7.630195	7.18018	8.100944	5.858535
17311147	Odf1	outer dense fiber of sperm tails 1	3.880897	4.708149	3.791319	3.404729	3.791319	3.725386
17368947	Odf2	outer dense fiber of sperm tails 2	6.81451	8.928204	6.856394	8.263769	7.528435	7.09907
17403340	Odf2l	outer dense fiber of sperm tails 2-like	17.44039	21.56994	20.55333	19.85472	19.50511	21.4012
17484615	Odf3	outer dense fiber of sperm tails 3	5.704063	6.16786	5.617429	7.337405	8.692312	8.420524
17320486	Odf3b	outer dense fiber of sperm tails 3B	45.70278	50.11533	49.48914	66.80249	43.90003	50.92881
17527453	Odf3l1	outer dense fiber of sperm tails 3-like 1	5.449145	5.468405	5.586366	6.482948	4.736003	5.134771

17242644	Odf3l2	outer dense fiber of sperm tails 3-like 2	16.43717	16.977	17.76949	18.07596	15.67442	17.15848
17264440	Odf4	outer dense fiber of sperm tails 4	4.583279	5.445219	4.497147	5.000804	4.497147	3.827918
17401447	Ovgp1	oviductal glycoprotein 1	3.686496	3.971737	2.933291	3.346099	3.569322	3.664611
17361656	Ovol1	OVO homolog-like 1 (Drosophila)	4.409882	4.921165	3.322202	4.80951	3.761166	4.616598
17488986	Ovol3	OVO homolog-like 3 (Drosophila)	10.45477	14.49657	12.7967	14.53672	10.28757	11.77773
17494752	Ovch2	ovochymase 2	3.973361	4.392493	4.392493	4.717197	3.817108	4.976323
17392403	Ovol2	ovo-like 2 (Drosophila)	11.2151	12.79129	11.14597	11.90347	13.67321	13.57387
17300261	Oxa1l	oxidase assembly 1-like	168.5765	164.1112	170.732	174.9526	159.2147	159.742
17311286	Oxr1	oxidation resistance 1	85.0135	73.69169	65.4445	77.00552	80.4628	78.73319
17506081	Osgin1	oxidative stress induced growth inhibitor 1	602.3967	383.5976	281.1605	394.076	289.9921	186.1588
17423479	Osgin2	oxidative stress induced growth inhibitor family member 2	7.532559	6.754037	7.650956	6.956155	8.024436	7.112171
17394024	Oser1	oxidative stress responsive serine rich 1	6.97716	5.169196	4.727017	5.132885	5.135732	5.140968
17532113	Oxsr1	oxidative-stress responsive 1	41.20907	44.61386	39.20865	43.97403	31.89437	31.48298
17471550	Olr1	oxidized low density lipoprotein (lectin-like) receptor 1	3.838147	3.92211	2.849562	3.249167	2.871829	4.167263
17273116	Oxld1	oxidoreductase like domain containing 1	12.15072	14.19214	13.15683	14.49607	14.67959	14.14578
17298488	Oxnad1	oxidoreductase NAD-binding domain containing 1	57.09134	46.80822	58.71389	59.57892	45.95564	56.74573
17247080	Ogdh	oxoglutarate (alpha-ketoglutarate) dehydrogenase (lipoamide)	592.9596	570.0719	578.607	577.4931	516.2586	522.6531
17309528	Oxgr1	oxoglutarate (alpha-ketoglutarate) receptor 1	4.328479	4.06645	4.262323	3.907213	4.372747	4.196539
17298556	Ogdhl	oxoglutarate dehydrogenase-like	6.19129	7.247181	6.74931	6.546392	8.022601	6.844523
17357771	Osbp	oxysterol binding protein	265.7029	157.7471	160.6336	146.1798	186.6989	131.2697
17259873	Osbp2	oxysterol binding protein 2	11.52728	11.50086	11.50086	12.5456	8.873295	10.4518
17522791	Osbpl10	oxysterol binding protein-like 10	10.70832	11.83887	11.85793	9.03685	9.684919	12.02788
17324998	Osbpl11	oxysterol binding protein-like 11	55.25411	51.79999	54.81428	54.34668	46.5197	50.11498
17352779	Osbpl1a	oxysterol binding protein-like 1A	24.81585	27.74393	29.20797	25.14623	28.34751	28.34751
17380711	Osbpl2	oxysterol binding protein-like 2	65.31534	52.40563	53.25666	50.9567	57.83817	46.93232
17466687	Osbpl3	oxysterol binding protein-like 3	11.18311	15.91869	18.12024	15.13535	7.068921	13.15957
17498331	Osbpl5	oxysterol binding protein-like 5	10.87603	8.375179	10.1025	6.46119	9.196915	8.539313
17372197	Osbpl6	oxysterol binding protein-like 6	4.902066	4.402277	4.873472	4.315327	4.890527	4.295929
17255727	Osbpl7	oxysterol binding protein-like 7	12.75915	13.35113	13.52944	10.53998	13.52006	11.82018
17237219	Osbpl8	oxysterol binding protein-like 8	133.5587	87.33391	85.65526	93.17171	93.21951	106.9505
17428142	Osbpl9	oxysterol binding protein-like 9	261.3972	304.1987	295.6856	262.7414	302.4326	264.3673
17376372	Oxt	oxytocin	11.48684	12.42548	11.69768	10.37033	11.69768	11.69768
17469627	Oxtr	oxytocin receptor	11.63767	11.80282	11.62179	11.66278	11.18017	13.13888
17480420	Pak1	p21 protein (Cdc42/Rac)-activated kinase 1	14.17503	16.60263	20.37248	15.46495	14.6912	20.21508
17329790	Pak2	p21 protein (Cdc42/Rac)-activated kinase 2	111.2946	117.8853	114.0688	126.3314	112.3308	111.4506
17538320	Pak3	p21 protein (Cdc42/Rac)-activated kinase 3	3.568258	4.61823	4.787094	3.870876	4.147667	4.049124
17488501	Pak4	p21 protein (Cdc42/Rac)-activated kinase 4	31.85792	31.38515	28.43114	24.52311	29.12649	26.87122
17374594	Pak6	p21 protein (Cdc42/Rac)-activated kinase 6	12.88401	8.712701	14.67807	13.73905	10.24345	12.28529
17392123	Pak7	p21 protein (Cdc42/Rac)-activated kinase 7	3.156772	3.412077	3.323538	3.044953	3.286678	3.030278
17443286	Por	P450 (cytochrome) oxidoreductase	175.8023	310.6702	398.1698	292.1104	353.6577	270.2255
17393022	Pdrg1	p53 and DNA damage regulated 1	32.76106	38.38152	30.15572	31.94194	34.5042	38.44596
17497924	Pidd1	p53 induced death domain protein 1	13.1114	15.52014	14.31296	13.37558	13.64452	17.73208
17475821	Paf1	Paf1, RNA polymerase II associated factor, homolog (S. cerevisiae)	77.22789	68.8808	73.22782	66.72706	70.90424	66.72706
17377328	Pax1	paired box 1	6.992411	5.117148	6.080584	6.748281	5.640058	6.080584
17359726	Pax2	paired box 2	2.633297	3.484882	3.103853	3.503362	2.945474	2.68347
17224684	Pax3	paired box 3	9.529893	9.162428	7.86818	7.318307	6.186657	6.26611
17465319	Pax4	paired box 4	6.992896	5.139614	6.397774	7.271838	5.815769	5.240338
17424880	Pax5	paired box 5	4.240899	4.310813	3.249145	3.574918	3.403384	4.381789
17424884	Pax5	paired box 5	7.870689	6.679546	6.583478	7.416148	6.487983	8.742343
17373943	Pax6	paired box 6	4.756828	7.061575	5.617429	5.989809	6.02959	7.438904
17389042	Pax6os1	paired box 6 opposite strand 1	3.213711	3.387827	3.550248	4.20927	4.20927	3.729055
17431910	Pax7	paired box 7	7.802519	9.826312	9.459802	11.19389	9.505418	14.14902
17382224	Pax8	paired box 8	2.996607	3.137075	3.114434	3.317945	2.675039	3.387286
17275604	Pax9	paired box 9	7.698994	8.50346	6.189831	8.379882	8.184249	6.757689
17485589	Pirb	paired Ig-like receptor B	9.306315	14.52735	12.8038	10.54122	9.291297	11.00845
17454166	Pilra	paired immunoglobin-like type 2 receptor alpha	7.285914	8.693517	12.95664	8.424961	8.444137	6.772319
17454179	Pilrb1	paired immunoglobin-like type 2 receptor beta 1	17.62278	27.15056	20.03721	30.98311	32.03662	14.48422
17454187	Pilrb2	paired immunoglobin-like type 2 receptor beta 2	4.681043	4.31159	7.367678	3.263839	5.253977	5.542704
17262457	Prop1	paired like homeodomain factor 1	7.265641	6.452508	7.439935	6.396976	6.992896	6.861291
17229096	Prrx1	paired related homeobox 1	9.123011	8.561657	10.60167	8.561657	8.438871	8.339032
17369286	Prrx2	paired related homeobox 2	12.61878	13.16203	13.89785	11.23938	11.84134	12.61878
17298608	Prrxl1	paired related homeobox protein-like 1	11.76452	14.68203	13.99239	15.88276	15.18	13.01641
17480955	Phox2a	paired-like homeobox 2a	4.444373	6.305759	5.240665	5.882462	4.664945	4.819689
17448458	Phox2b	paired-like homeobox 2b	6.915147	6.915147	6.746644	8.514253	7.935109	6.254307
17292967	Pitx1	paired-like homeodomain transcription factor 1	5.020984	7.704706	6.569939	8.783951	8.191173	6.655329
17402541	Pitx2	paired-like homeodomain transcription factor 2	4.329259	3.819172	3.795763	3.559291	3.948733	3.729055
17365331	Pitx3	paired-like homeodomain transcription factor 3	7.864582	6.551204	6.512223	7.025009	5.773795	8.053579
17286719	Pak1ip1	PAK1 interacting protein 1	78.18229	71.33459	78.78833	70.69273	71.60012	83.89599
17509537	Palld	palladin, cytoskeletal associated protein	28.47098	18.81355	23.81849	20.2427	18.25424	15.89576
17409792	Palmd	palmdelphin	133.5429	155.0952	134.525	125.2442	117.9728	134.525
17418101	Ppt1	palmitoyl-protein thioesterase 1	72.68773	82.76506	76.77262	70.10474	56.72922	67.07066
17343897	Ppt2	palmitoyl-protein thioesterase 2	48.3673	45.11233	39.55971	54.11588	43.90764	50.44362
17238394	Pan2	PAN2 polyA specific ribonuclease subunit homolog (S. cerevisiae)	42.89448	49.59422	47.54956	43.04131	51.15169	53.49121
17444855	Pan3	PAN3 polyA specific ribonuclease subunit homolog (S. cerevisiae)	55.56097	75.19162	71.40583	67.47448	67.47448	73.12841
17382054	Ptf1aos	pancreas specific transcription factor 1a, opposite strand	3.637557	3.337065	3.494824	3.45858	2.91972	3.873345
17367376	Ptf1a	pancreas specific transcription factor, 1a	4.704398	4.704398	4.804213	4.704398	4.675984	4.875161
17444841	Pdx1	pancreatic and duodenal homeobox 1	5.899857	5.300432	5.299881	5.288945	5.191171	6.159401
17360751	Pnlip	pancreatic lipase	2.521898	2.822415	2.481049	2.867771	3.729055	2.427346
17360770	Pnliprp1	pancreatic lipase related protein 1	3.934199	4.178167	4.121873	4.105307	3.581987	3.599509
17360785	Pnliprp2	pancreatic lipase-related protein 2	3.964311	4.906894	4.734789	4.585948	5.922187	5.738406
17270048	Ppy	pancreatic polypeptide	12.51252	15.14784	9.804472	10.34972	10.5437	10.29855
17380953	Ppdpf	pancreatic progenitor cell differentiation and proliferation factor homolog (zebrafi	21.57652	24.60859	27.78222	25.70345	29.2027	31.60279
17524107	Panx1	pannexin 1	12.09291	12.65539	14.0219	15.71619	9.592587	12.16884
17314051	Panx2	pannexin 2	12.78987	11.40773	11.50086	12.95152	11.83715	12.64785

17525673	Panx3	pannexin 3	4.203147	4.482427	4.623707	4.45273	3.960932	4.255474
17364150	Pank1	pantothenate kinase 1	450.7426	549.4293	623.1778	585.4694	585.226	546.7888
17376493	Pank2	pantothenate kinase 2	38.21402	38.6019	40.47615	38.63563	41.06109	35.74328
17248476	Pank3	pantothenate kinase 3	105.7475	90.92972	106.1389	85.51645	102.4893	111.1689
17422449	Pank4	pantothenate kinase 4	25.43988	24.36114	24.36114	19.42873	25.52608	23.96355
17294974	Papd4	PAP associated domain containing 4	57.94451	60.11862	60.07113	60.07113	58.53713	60.07113
17503596	Papd5	PAP associated domain containing 5	89.75622	90.49712	94.6106	91.00223	75.31002	87.13799
17294114	Papd7	PAP associated domain containing 7	38.41373	39.28046	43.51135	41.2125	31.50677	34.15323
17277101	Papln	papilin, proteoglycan-like sulfated glycoprotein	8.062795	10.86932	8.740707	8.721281	8.319922	9.446173
17406604	Prcc	papillary renal cell carcinoma (translocation-associated)	32.48001	40.71335	40.35149	47.81893	46.14812	34.86699
17228667	Pappa2	pappalysin 2	3.684682	4.01258	3.009972	3.703322	3.749375	3.906103
17228694	Pappa2	pappalysin 2	3.753457	4.102917	4.40005	6.185285	4.355475	4.089176
17506949	Pard3	par-3 family cell polarity regulator	81.06459	87.63772	88.94182	83.5617	86.05875	87.05891
17213515	Pard3b	par-3 family cell polarity regulator beta	13.60241	12.99163	13.14088	13.40937	13.29093	12.6123
17223649	Pard3bos1	par-3 family cell polarity regulator beta, opposite strand 1	3.177473	3.011746	2.589799	2.659804	2.621659	2.659804
17223655	Pard3bos2	par-3 family cell polarity regulator beta, opposite strand 2	3.178046	2.457753	3.076713	2.677135	2.531882	2.660413
17223659	Pard3bos3	par-3 family cell polarity regulator beta, opposite strand 3 [Source:MGI Symbol;Ac	3.558601	2.887418	3.007553	3.000244	3.041304	3.308667
17504922	Pard6a	par-6 family cell polarity regulator alpha	8.637298	9.948714	7.72326	6.722836	11.35173	9.798018
17379981	Pard6b	par-6 family cell polarity regulator beta	18.1834	14.06736	13.70766	15.92454	15.8984	19.67211
17352120	Pard6g	par-6 family cell polarity regulator gamma	3.834079	4.646582	3.656365	4.256094	4.61887	4.668309
17234963	Palm	paralemmin	22.59169	22.00196	26.19192	29.32868	23.84145	30.98354
17503060	Palm3	paralemmin 3	17.26634	14.69782	16.6545	19.05331	19.43102	15.50434
17414351	Pakap	paralemmin A kinase anchor protein	7.10675	7.394384	6.940935	7.110988	6.858248	5.970989
17542249	Pnma5	paraneoplastic antigen family 5	3.682409	2.797542	3.806539	3.629272	3.355505	2.528374
17282461	Pnma1	paraneoplastic antigen MA1	5.121726	5.877489	6.196399	6.006689	7.135181	6.008646
17301523	Pnma2	paraneoplastic antigen MA2	5.715382	5.234675	5.630644	5.250009	5.021819	5.706911
17535473	Pnma3	paraneoplastic antigen MA3	4.089799	2.67402	3.402252	2.732781	2.70701	2.575138
17464614	Pon1	paraoxonase 1	1257.506	1335.552	1340.523	1329.705	1425.014	1577.255
17464638	Pon2	paraoxonase 2	93.989	89.67103	84.9917	71.94936	88.4831	63.56805
17464626	Pon3	paraoxonase 3	264.1164	256.9778	244.0005	219.9375	199.9853	203.8946
17307080	Pspc1	paraspeckle protein 1	16.27031	16.57791	13.89544	16.35228	16.84607	19.26286
17470988	Ptms	parathymosin	39.5019	30.81263	34.77142	42.51824	39.06541	42.18973
17495242	Pth	parathyroid hormone	3.270407	2.818661	2.705922	2.818661	3.519011	2.925027
17531650	Pth1r	parathyroid hormone 1 receptor	15.46302	17.11323	15.89356	18.19109	17.09473	21.62128
17477728	Pth2	parathyroid hormone 2	13.71003	18.09213	19.87771	17.28118	16.40223	16.95866
17213747	Pth2r	parathyroid hormone 2 receptor	5.123253	3.946927	4.342512	4.254855	4.647001	4.254855
17472903	Pthlh	parathyroid hormone-like peptide	6.745942	8.356739	8.467112	7.189044	5.63869	7.858751
17340774	Pacrg	PARK2 co-regulated	5.374945	5.160425	4.244604	4.72895	5.373232	4.873641
17437355	Pacrgl	PARK2 co-regulated-like	14.07126	13.48104	12.36714	13.11531	13.8053	13.30153
17433310	Park7	Parkinson disease (autosomal recessive, early onset) 7	75.4919	73.09699	67.09856	73.97162	76.30047	87.69727
17333206	Park2	Parkinson disease (autosomal recessive, juvenile) 2, parkin	7.207655	9.822498	12.14853	8.406469	8.887946	8.972438
17497885	Pddc1	Parkinson disease 7 domain containing 1	29.47766	29.47766	29.17903	27.24142	33.39986	27.56107
17214166	Pnkd	paroxysmal nonkinesiogenic dyskinesia	186.9061	153.9609	152.6528	162.1876	146.0319	148.3838
17243850	Parpbp	PARP1 binding protein	5.038834	3.692326	4.275343	4.017144	3.463378	4.539993
17495932	Palb2	partner and localizer of BRCA2	5.725057	5.405507	5.730218	5.790067	5.93398	5.561176
17260775	Pno1	partner of NOB1 homolog (S. cerevisiae)	77.98528	58.66223	58.36332	66.62215	52.39183	61.97825
17318942	Pvalb	parvalbumin	5.097076	5.415333	4.916597	5.097076	5.079688	5.282533
17481916	Parva	parvin, alpha	99.32259	117.7326	98.89255	97.48917	126.7823	107.6198
17313705	Parvb	parvin, beta	13.17663	10.33094	11.09563	11.25161	12.46905	10.28742
17313721	Parvg	parvin, gamma	11.61357	9.178256	9.178256	8.528192	10.08095	9.976197
17225705	Pask	PAS domain containing serine/threonine kinase	3.462322	3.731201	3.410303	3.31583	4.352065	3.203302
17335419	Pnpla1	patatin-like phospholipase domain containing 1	4.681043	4.735543	4.515263	5.006873	4.115335	3.993019
17342940	Pnpla1os	patatin-like phospholipase domain containing 1, opposite strand [Source:MGI Sym	5.089028	5.48933	5.195707	6.056188	5.48933	7.462971
17484909	Pnpla2	patatin-like phospholipase domain containing 2	110.4049	218.6546	248.2638	210.8875	167.1709	165.6036
17313677	Pnpla3	patatin-like phospholipase domain containing 3	10.20186	9.854277	10.58816	8.606401	7.974201	9.789126
17319968	Pnpla5	patatin-like phospholipase domain containing 5	7.123963	6.898009	6.287864	5.06587	5.563104	6.593022
17498625	Pnpla6	patatin-like phospholipase domain containing 6	40.26906	36.39857	44.12334	36.13112	39.46413	28.67
17367765	Pnpla7	patatin-like phospholipase domain containing 7	43.9716	37.47795	38.75069	39.12666	39.2459	37.81797
17275237	Pnpla8	patatin-like phospholipase domain containing 8	104.5479	112.4305	102.5832	109.6073	103.6266	103.7336
17545618	Ptchd1	patched domain containing 1	9.98706	11.41802	10.55497	11.89167	9.856804	9.39993
17432941	Ptchd2	patched domain containing 2	9.333706	8.967029	8.967029	8.51685	8.967029	8.967029
17259693	Ptchd3	patched domain containing 3	2.804085	2.813839	2.255364	2.218262	2.565073	2.664879
17337735	Ptchd4	patched domain containing 4	6.123727	5.846933	5.48933	4.838735	4.436371	5.459088
17293607	Ptch1	patched homolog 1	39.55861	38.31985	51.53248	38.60003	37.07282	40.76248
17417622	Ptch2	patched homolog 2	10.48714	13.76784	13.79506	14.40762	11.46759	14.14284
17455878	Peg10	paternally expressed 10	18.30137	20.95424	17.29568	15.38412	14.30542	18.58581
17491759	Peg12	paternally expressed 12	22.07375	17.13008	13.17417	15.76322	11.46655	17.14909
17317835	Peg13	paternally expressed 13	13.10659	11.03887	12.89804	10.49689	13.34151	16.3609
17486110	Peg3	paternally expressed 3	34.21744	44.89709	53.60145	29.52858	25.60671	23.90102
17446539	Paxip1	PAX interacting (with transcription-activation domain) protein 1	26.71041	29.11922	37.32784	33.29445	28.47808	31.96542
17332084	Paxbp1	PAX3 and PAX7 binding protein 1	21.99342	21.92644	21.40625	25.20521	23.44746	24.27703
17441168	Pxn	paxillin	31.59162	32.56499	36.26661	35.21236	39.75212	34.3822
17496532	Pagr1a	PAXIP1 associated glutamate rich protein 1A	15.06334	17.16526	18.51189	16.09689	17.26825	15.81981
17335878	Pknox1	Pbx/knotted 1 homeobox	13.53216	11.79285	9.655895	16.32601	17.56534	14.08463
17525561	Pknox2	Pbx/knotted 1 homeobox 2	12.10121	12.2933	13.15373	11.0109	9.965278	11.00899
17426890	Psip1	PC4 and SFRS1 interacting protein 1	28.36913	28.34574	25.86501	28.34574	27.5372	25.06287
17391640	Pced1a	PC-esterase domain containing 1A	23.59452	23.59452	22.01889	28.79667	25.96561	19.38327
17314541	Pced1b	PC-esterase domain containing 1B	8.240546	9.848131	11.25629	8.858546	8.622642	9.397129
17507537	Pcid2	PCI domain containing 2	30.11332	24.15383	22.72518	25.85623	31.0033	26.4943
17455061	Pdap1	PDGFA associated protein 1	48.37266	51.64118	40.38507	53.76107	53.76107	55.01031
17431272	Pdik1l	PDLIM1 interacting kinase 1 like	27.32974	36.03058	29.12286	26.44678	27.01502	36.12711
17448335	Pds5a	PDS5, regulator of cohesion maintenance, homolog A (S. cerevisiae)	129.7427	118.8848	114.9275	121.5048	115.7678	122.062
17445193	Pds5b	PDS5, regulator of cohesion maintenance, homolog B (S. cerevisiae)	46.77935	40.85923	39.03537	41.5645	44.78582	37.66963
17379582	Pcif1	PDX1 C-terminal inhibiting factor 1	28.18391	29.64342	32.52258	31.7543	34.49439	32.86728

17364474	Pdlim1	PDZ and LIM domain 1 (elfin)	51.078	69.36135	91.40999	70.88999	75.67737	66.49251
17308242	Pdlim2	PDZ and LIM domain 2	12.14819	14.40842	11.53279	11.77953	14.02025	10.85757
17500922	Pdlim3	PDZ and LIM domain 3	6.171709	5.831027	6.123388	5.716175	5.277081	7.668423
17262798	Pdlim4	PDZ and LIM domain 4	5.212083	5.274886	6.187129	5.391886	5.067872	5.554011
17410748	Pdlim5	PDZ and LIM domain 5	87.21413	75.48353	80.16719	78.58489	77.1471	75.5254
17292891	Pdlim7	PDZ and LIM domain 7	13.27942	20.24467	19.62458	17.19062	17.07164	19.3912
17346603	Pdzph1	PDZ and pleckstrin homology domains 1	6.13116	4.339353	4.167811	4.758807	4.814413	5.256709
17301428	Pbk	PDZ binding kinase	5.338965	4.964333	4.75983	4.57896	4.124617	5.231374
17400732	Pdzk1	PDZ domain containing 1	37.61145	41.79707	43.16111	46.76509	33.05829	42.01376
17543479	Pdzd11	PDZ domain containing 11	24.37989	21.28566	30.32026	28.08465	31.16079	31.54982
17316068	Pdzd2	PDZ domain containing 2	5.8022	5.587411	5.22058	6.151593	6.283681	6.533291
17526149	Pdzd3	PDZ domain containing 3	6.57878	6.783313	5.53967	7.88844	4.797491	4.550957
17542408	Pdzd4	PDZ domain containing 4	12.4235	10.58963	10.85773	13.84737	11.57371	11.65987
17365167	Pdzd7	PDZ domain containing 7	6.332785	7.251251	7.698994	6.820701	6.341306	7.698994
17366039	Pdzd8	PDZ domain containing 8	105.462	106.7913	93.02135	110.3743	85.26076	90.60445
17495802	Pdzd9	PDZ domain containing 9	4.732754	4.125389	4.584486	5.180567	5.161463	4.235669
17495812	Pdzd9	PDZ domain containing 9	5.08388	5.33301	4.831161	5.051774	6.100088	4.927036
17469488	Pdzrn3	PDZ domain containing RING finger 3	19.4542	24.53858	24.85269	21.82258	18.16035	20.06877
17314421	Pdzrn4	PDZ domain containing RING finger 4	5.006284	4.692868	4.604805	4.599541	4.762173	5.56106
17417146	Pdzk1ip1	PDZK1 interacting protein 1	14.35111	15.78454	11.55896	13.39423	9.159444	13.1438
17276940	Pcnx	pecanex homolog (Drosophila)	48.04885	56.55529	57.07433	53.5903	57.39289	54.11963
17514170	Pcnxl2	pecanex-like 2 (Drosophila)	5.660345	5.914065	5.882625	6.757127	6.481016	6.155005
17361703	Pcnxl3	pecanex-like 3 (Drosophila)	41.61062	32.38536	41.85186	30.15217	36.01086	33.57324
17276235	Pcnxl4	pecanex-like 4 (Drosophila)	9.549068	10.5784	11.77577	10.81784	8.384472	9.756407
17473637	Peg3os	Peg3 opposite strand	6.36288	7.742717	8.718138	6.242872	4.810377	4.833874
17247550	Peli1	pellino 1	76.5574	67.29465	67.29465	77.24288	78.80253	83.10193
17299401	Peli2	pellino 2	36.02783	41.87682	43.89242	46.71228	45.99069	39.16302
17361424	Peli3	pellino 3	15.47653	14.51296	15.2624	14.2205	15.72589	12.85511
17296422	Pelo	pelota homolog (Drosophila)	39.00183	38.05806	35.58975	37.99822	37.00145	36.22968
17419206	Pef1	penta-EF hand domain containing 1	25.67976	20.18414	21.37941	23.6438	16.86605	18.58259
17455071	Ptcd1	pentatricopeptide repeat domain 1	25.83885	26.56031	42.35411	37.41487	30.41245	33.59
17295463	Ptcd2	pentatricopeptide repeat domain 2	63.64874	43.37284	47.82888	60.59051	55.08433	57.41039
17467664	Ptcd3	pentatricopeptide repeat domain 3	75.27558	74.17546	63.47471	78.03179	59.81643	63.11277
17467690	Ptcd3	pentatricopeptide repeat domain 3	381.2438	305.9665	305.2442	477.8454	519.1257	462.8754
17334538	Ptx4	pentraxin 4	6.325064	4.028883	4.028883	3.706738	4.624637	4.024892
17398115	Ptx3	pentraxin related gene	6.775324	6.098735	6.569757	6.579783	6.992896	6.579783
17362741	Pga5	pepsinogen 5, group I	4.746947	7.287783	4.930555	5.501557	5.63826	5.280264
17368302	Pmpca	peptidase (mitochondrial processing) alpha	48.46328	53.34829	51.66696	52.99411	45.73225	48.73479
17435130	Pmpcb	peptidase (mitochondrial processing) beta	242.3167	246.5421	253.7493	253.7493	214.2736	244.9885
17476709	Pepd	peptidase D	67.09903	62.7964	58.22795	54.43716	55.44863	53.55131
17373680	Pamr1	peptidase domain containing associated with muscle regeneration 1	7.870689	11.33836	9.188249	9.188249	7.935109	9.864186
17211305	Pi15	peptidase inhibitor 15	2.841652	2.841652	2.841652	3.00749	2.841652	3.015924
17335506	Pi16	peptidase inhibitor 16	9.18653	8.908978	8.60008	8.60008	8.60008	8.852654
17217083	Pm20d1	peptidase M20 domain containing 1	96.52428	94.06656	87.4587	85.65823	106.3481	114.9331
17423781	Pm20d2	peptidase M20 domain containing 2	13.86273	15.32505	15.55903	12.98604	18.49649	18.96556
17512691	Pdf	peptide deformylase (mitochondrial)	4.546322	7.080052	7.851618	7.683853	6.799459	6.282418
17270053	Pyy	peptide YY	3.671526	6.167774	5.482865	5.379828	5.433418	5.848878
17474371	Pglyrp1	peptidoglycan recognition protein 1	14.56304	15.64988	14.51759	15.74815	16.40848	18.49444
17343449	Pglyrp2	peptidoglycan recognition protein 2	197.3401	184.2525	232.0336	192.0383	228.4239	170.9073
17399845	Pglyrp3	peptidoglycan recognition protein 3	3.998531	3.996453	3.709976	3.998531	5.439711	5.211577
17399828	Pglyrp4	peptidoglycan recognition protein 4	3.206124	3.63282	2.861001	3.240598	3.076521	3.478511
17432058	Padi1	peptidyl arginine deiminase, type I	11.02006	11.1401	12.63549	12.40904	9.87698	12.77446
17420874	Padi2	peptidyl arginine deiminase, type II	4.01461	4.489703	4.39228	3.868784	4.489703	6.462624
17432038	Padi3	peptidyl arginine deiminase, type III	6.126827	7.757759	6.533473	7.790792	7.118583	8.369434
17432014	Padi4	peptidyl arginine deiminase, type IV	3.586881	4.185587	4.367658	4.199856	3.881273	3.787485
17431992	Padi6	peptidyl arginine deiminase, type VI	4.763692	5.036041	4.592119	4.559609	4.229362	4.559609
17429371	Ppih	peptidyl prolyl isomerase H	4.99007	4.801649	5.692214	4.868644	6.171709	10.07027
17225929	Pam	peptidylglycine alpha-amidating monooxygenase	40.56799	43.89364	49.31142	38.55858	36.64616	37.56117
17343026	Ppil1	peptidylprolyl isomerase (cyclophilin)-like 1	11.80315	9.244594	11.99316	9.719216	11.37283	9.176411
17328582	Ppil2	peptidylprolyl isomerase (cyclophilin)-like 2	110.3613	91.69429	80.69459	87.82258	84.02752	64.08301
17223378	Ppil3	peptidylprolyl isomerase (cyclophilin)-like 3	20.83836	15.77119	18.6845	20.3578	20.17952	24.29908
17231574	Ppil4	peptidylprolyl isomerase (cyclophilin)-like 4	31.10318	25.01203	22.19603	29.01667	30.56122	22.78228
17232881	Ppil6	peptidylprolyl isomerase (cyclophilin)-like 6	6.781432	5.178269	6.992896	5.809645	5.952435	5.952435
17247140	Ppia	peptidylprolyl isomerase A	748.4333	727.6596	688.4032	671.2377	667.8036	631.4392
17520588	Ppia	peptidylprolyl isomerase A	582.4418	598.1112	593.5764	553.6191	550.3555	524.9732
17357969	E030024N20Rik	peptidylprolyl isomerase A pseudogene 8	7.571452	9.938237	7.814047	8.859345	7.065982	8.290563
17518664	Ppib	peptidylprolyl isomerase B	356.5177	280.557	249.1309	287.2036	329.6314	269.4736
17354378	Ppic	peptidylprolyl isomerase C	21.20041	25.22776	31.70657	21.23291	20.56829	22.16037
17398384	Ppid	peptidylprolyl isomerase D (cyclophilin D)	35.02784	35.8142	31.24991	40.07276	33.85812	36.68402
17295944	Ppwd1	peptidylprolyl isomerase domain and WD repeat containing 1	23.35015	25.13107	22.88515	23.21733	23.37315	27.49829
17429659	Ppie	peptidylprolyl isomerase E (cyclophilin E)	11.02143	9.120545	10.09501	11.56113	14.71341	11.60456
17297750	Ppif	peptidylprolyl isomerase F (cyclophilin F)	88.53279	85.92107	119.2834	93.52434	97.54392	87.57881
17304141	Ppifos	peptidylprolyl isomerase F, opposite strand	10.3172	11.70782	11.67508	9.757151	11.29764	11.89184
17371460	Ppig	peptidyl-prolyl isomerase G (cyclophilin G)	16.30497	17.47415	19.17786	20.5425	21.21569	20.18833
17369986	Ptrh1	peptidyl-tRNA hydrolase 1 homolog (S. cerevisiae)	20.63698	17.16157	17.69684	16.43546	15.61326	17.16157
17254662	Ptrh2	peptidyl-tRNA hydrolase 2	5.883767	5.357389	5.857195	4.179731	4.191452	5.259115
17273743	Ptrhd1	peptidyl-tRNA hydrolase domain containing 1	11.40599	10.7341	9.911477	12.64189	11.61993	11.69816
17233694	Prf1	perforin 1 (pore forming protein)	13.42732	13.43198	13.39423	12.16581	13.62175	12.16581
17366423	Phxr1	per-hexamer repeat gene 1	10.62404	6.261638	8.854004	6.618113	9.689284	6.447456
17244551	Phxr2	per-hexamer repeat gene 2	2.567457	2.653911	2.964153	2.792486	3.158063	2.606909
17514687	Phxr4	per-hexamer repeat gene 4	3.54506	3.62824	3.371452	3.907132	3.043856	3.326857
17475603	Prx	periaxin	4.820023	5.7632	5.311907	3.98246	5.573679	4.072289
17242088	Pcnt	pericentrin (kendrin)	13.97504	14.93401	19.03668	15.83023	21.50291	18.5056
17500761	Pcm1	pericentriolar material 1	58.35199	66.22757	62.45001	57.81252	66.32312	66.22757

17492406	Plin1	perilipin 1	7.819411	8.490326	8.960629	10.13476	7.150926	9.116816
17426981	Plin2	perilipin 2	636.6956	612.9349	674.7129	520.881	362.7068	466.3826
17346216	Plin3	perilipin 3	54.19471	59.44527	84.95282	53.07682	65.91432	56.13933
17346125	Plin4	perilipin 4	12.95476	27.9009	31.95811	18.62515	18.49649	21.68281
17346136	Plin5	perilipin 5	103.6345	96.22768	80.30345	115.0303	76.16784	96.99846
17251527	Per1	period circadian clock 1	28.50099	82.78687	69.09168	39.11879	61.10506	61.10506
17225506	Per2	period circadian clock 2	14.03522	18.77993	20.54578	13.94601	24.31509	21.38371
17433328	Per3	period circadian clock 3	10.26527	14.62313	17.17383	11.50573	12.26989	16.56608
17397575	Postn	periostin, osteoblast specific factor	20.57585	32.64771	28.66642	24.9966	19.40195	35.04557
17404077	Pmp2	peripheral myelin protein 2	2.539827	2.655438	2.704478	2.606909	2.160552	2.67402
17250839	Pmp22	peripheral myelin protein 22	14.35339	16.5686	19.51836	14.5473	11.32375	19.31059
17314699	Prph	peripherin	5.889071	5.944436	5.656305	6.488253	5.737491	4.67174
17338203	Prph2	peripherin 2	8.467171	8.544701	10.79312	7.678955	9.659175	10.87241
17314439	Pphln1	periphilin 1	35.38731	28.68968	30.41013	34.5085	29.64383	25.46017
17327909	Ppl	periplakin	41.45948	39.49012	43.46041	36.45842	32.96424	31.66396
17420171	Hspg2	perlecan (heparan sulfate proteoglycan 2)	41.33294	46.92972	57.98348	41.3043	33.40495	38.71069
17274703	Pxdn	peroxidasin homolog (Drosophila)	20.29525	17.92151	22.13642	23.29454	16.74978	18.61418
17417480	Prdx1	peroxiredoxin 1	29.93974	27.64047	25.05766	25.78446	28.42306	21.66839
17503287	Prdx2	peroxiredoxin 2	54.76112	54.76112	57.59853	54.06751	53.69255	47.16223
17366141	Prdx3	peroxiredoxin 3	220.3236	220.6476	209.9701	230.0877	222.0391	238.1093
17545605	Prdx4	peroxiredoxin 4	32.04883	20.95511	18.82098	26.11795	28.87622	24.84632
17362101	Prdx5	peroxiredoxin 5	139.4912	135.7012	145.1228	143.2062	160.4278	140.2707
17228846	Prdx6	peroxiredoxin 6	34.79046	39.33986	41.19679	40.40327	37.29784	45.42045
17372388	Prdx6b	peroxiredoxin 6B	3.401969	3.602629	3.401969	3.401969	3.401969	2.972424
17434333	Pex1	peroxisomal biogenesis factor 1	35.25461	39.06325	37.27122	41.55413	33.08992	34.55494
17422476	Pex10	peroxisomal biogenesis factor 10	13.60674	13.82397	11.35133	13.51847	13.87081	13.82819
17492417	Pex11a	peroxisomal biogenesis factor 11 alpha	154.322	196.2733	215.8776	167.5108	134.5828	133.2009
17400638	Pex11b	peroxisomal biogenesis factor 11 beta	32.22436	31.53539	35.15529	31.96276	26.50201	31.50131
17507082	Pex11g	peroxisomal biogenesis factor 11 gamma	49.9506	54.49153	54.2571	50.58578	58.09773	56.7902
17266891	Pex12	peroxisomal biogenesis factor 12	27.48095	25.49019	28.66265	28.2606	29.70698	31.23843
17261098	Pex13	peroxisomal biogenesis factor 13	50.61559	40.65723	42.74874	45.10327	38.10531	40.75598
17433009	Pex14	peroxisomal biogenesis factor 14	94.24865	92.64556	98.17951	88.16414	74.56465	78.22837
17373484	Pex16	peroxisomal biogenesis factor 16	70.44083	70.87328	75.60239	73.95317	80.90405	78.45481
17219489	Pex19	peroxisomal biogenesis factor 19	153.3538	168.0248	155.2006	157.4445	141.5168	140.4419
17403973	Pex2	peroxisomal biogenesis factor 2	38.77675	27.03975	26.14929	36.01111	22.56805	32.11955
17462420	Pex26	peroxisomal biogenesis factor 26	30.37726	41.62909	45.41667	34.07708	34.45998	41.62707
17239401	Pex3	peroxisomal biogenesis factor 3	123.9831	117.488	97.65351	97.69481	114.1083	110.6408
17470689	Pex5	peroxisomal biogenesis factor 5	106.2022	96.27304	95.46037	100.3516	89.97985	87.23287
17404628	Pex5l	peroxisomal biogenesis factor 5-like	5.608325	6.517461	6.999394	6.183313	6.095607	6.707569
17338174	Pex6	peroxisomal biogenesis factor 6	59.79695	59.29629	53.0897	64.17413	53.76443	56.91433
17239616	Pex7	peroxisomal biogenesis factor 7	58.65491	54.73493	48.34115	61.29383	58.56351	68.00364
17450989	Pxmp2	peroxisomal membrane protein 2	194.8365	200.5725	238.7588	265.3256	283.3163	317.7504
17393173	Pxmp4	peroxisomal membrane protein 4	455.1001	340.75	324.8748	386.0757	298.2793	263.345
17224133	Pecr	peroxisomal trans-2-enoyl-CoA reductase	210.8817	198.8794	169.9327	217.7124	182.7034	195.0297
17342958	Pxt1	peroxisomal, testis specific 1	19.42873	13.8232	14.67857	15.04404	15.37101	13.15747
17448001	Ppargc1a	peroxisome proliferative activated receptor, gamma, coactivator 1 alpha	38.79503	79.58088	85.88117	65.38102	71.33954	95.25283
17354739	Ppargc1b	peroxisome proliferative activated receptor, gamma, coactivator 1 beta	9.979794	11.41153	10.52676	13.42444	11.3559	18.79921
17359878	Pprc1	peroxisome proliferative activated receptor, gamma, coactivator-related 1	23.04178	22.28931	22.03951	23.80925	25.97713	21.60285
17313862	Ppara	peroxisome proliferator activated receptor alpha	147.4897	165.451	179.3694	148.2152	161.2405	172.7162
17461942	Pparg	peroxisome proliferator activated receptor gamma	37.7991	42.39758	39.76149	28.01913	28.63445	37.27432
17335297	Ppard	peroxisome proliferator activator receptor delta	23.02996	20.62125	25.27904	27.67862	24.94744	25.82703
17231844	Perp	PERP, TP53 apoptosis effector	191.2041	178.3528	169.6279	170.1744	138.4556	132.7511
17360532	Ppnr	per-pentamer repeat gene	3.994071	5.789906	6.671726	4.854424	6.992896	5.104324
17548110	Ppnr	per-pentamer repeat gene	18.86212	25.98919	31.16576	29.82789	20.09159	34.7895
17346409	Pspn	persephin	14.98555	17.87374	16.35829	18.2911	15.23914	14.2762
17246682	Pes1	pescadillo homolog 1, containing BRCT domain (zebrafish)	68.46525	68.46525	73.53912	62.76986	68.46525	70.19664
17331021	Pcnp	PEST proteolytic signal containing nuclear protein	17.58289	15.37378	16.27866	17.33241	15.2193	16.286
17498695	Pet100	PET100 homolog (S. cerevisiae)	15.04915	14.23865	14.10046	15.36398	19.93	15.35312
17377041	Pet117	PET117 homolog (S. cerevisiae)	41.10409	34.66001	45.89452	40.33835	41.03548	38.35546
17515045	Ppan	peter pan homolog (Drosophila)	22.68206	24.26088	24.26088	23.7784	22.11219	24.26088
17515058	Ppan	peter pan homolog (Drosophila)	16.82845	17.67269	20.77015	23.03587	21.05557	20.97371
17216373	Phlpp1	PH domain and leucine rich repeat protein phosphatase 1	64.53635	74.15027	81.34603	70.62563	60.65522	69.38731
17505455	Phlpp2	PH domain and leucine rich repeat protein phosphatase 2	11.73837	13.87812	12.77189	9.488237	11.42783	8.046771
17484802	Phrf1	PHD and ring finger domains 1	23.27953	28.3051	24.1979	28.28058	23.76489	26.42699
17335000	Phf1	PHD finger protein 1	26.77714	35.2507	28.91628	23.75271	28.34731	28.91628
17341132	Phf10	PHD finger protein 10	45.08482	51.19958	52.4536	51.49106	44.79016	50.78956
17341148	Phf10	PHD finger protein 10	4.717557	4.204925	4.182165	4.992665	6.03444	5.056153
17307269	Phf11a	PHD finger protein 11A	13.83327	15.25637	16.62705	12.39048	20.41376	14.31097
17307280	Phf11b	PHD finger protein 11B	16.37962	20.65587	26.99593	19.4658	16.28148	9.894248
17307305	Phf11c	PHD finger protein 11C	7.751632	13.5845	10.9554	14.96012	13.30568	12.5476
17307290	Phf11d	PHD finger protein 11D	6.110244	6.736784	6.703897	8.379418	8.060001	8.976108
17253396	Phf12	PHD finger protein 12	45.54813	50.87906	53.82782	54.73645	55.44325	55.97303
17433405	Phf13	PHD finger protein 13	33.80606	32.59209	32.8181	28.19427	30.39095	29.42071
17456025	Phf14	PHD finger protein 14	34.22242	35.16212	33.45268	35.22115	36.42356	35.03561
17384334	Phf19	PHD finger protein 19	5.483017	6.586673	5.376324	7.476329	4.829152	5.310729
17222094	AA619741	PHD finger protein 19 pseudogene	3.651148	2.889721	2.616358	3.441864	2.458673	3.036333
17292346	Phf2	PHD finger protein 2	55.66312	56.17904	60.59933	58.94755	48.0855	53.24964
17378548	Phf20	PHD finger protein 20	12.30319	13.06568	12.21829	12.21829	12.488	11.82362
17311945	Phf20l1	PHD finger protein 20-like 1	56.0946	52.9317	47.87199	50.07192	54.98858	46.67086
17373450	Phf21a	PHD finger protein 21A	18.8807	20.13132	28.15716	20.184	25.85336	16.87774
17319997	Phf21b	PHD finger protein 21B	7.975915	10.36588	9.455869	9.977027	9.932521	9.47588
17251866	Phf23	PHD finger protein 23	30.81648	37.75589	29.15558	30.7804	32.05217	34.65136
17413173	Phf24	PHD finger protein 24	6.461817	6.621784	7.462402	6.580923	6.621784	6.992896
17221939	Phf3	PHD finger protein 3	97.57232	86.01641	84.59028	84.47895	84.59028	84.59028

17319539	Phf5a	PHD finger protein 5A	43.88512	37.2614	38.35891	42.94118	35.52493	45.16646
17534706	Phf6	PHD finger protein 6	45.33332	33.70896	34.33838	34.41248	30.04681	37.95926
17304646	Phf7	PHD finger protein 7	21.24277	22.46427	23.51745	23.85054	23.3073	29.14022
17538704	Phf8	PHD finger protein 8	20.81873	19.42873	17.85208	12.67831	17.2122	14.92615
17233873	Pbld1	phenazine biosynthesis-like protein domain containing 1	202.0152	224.8938	208.3781	228.8502	247.8769	242.3856
17233860	Pbld2	phenazine biosynthesis-like protein domain containing 2	9.232555	14.06922	9.428838	11.8715	9.310251	8.759327
17236269	Pah	phenylalanine hydroxylase	1098.539	1041.322	1071.889	1323.8	1313.5	1575.79
17286575	Fars2	phenylalanine-tRNA synthetase 2 (mitochondrial)	52.40382	53.38047	57.92041	55.09617	53.38047	43.45921
17503226	Farsa	phenylalanyl-tRNA synthetase, alpha subunit	24.77323	22.96126	25.8324	22.18634	29.74139	20.79133
17224702	Farsb	phenylalanyl-tRNA synthetase, beta subunit	44.84888	39.9535	39.46194	39.88875	35.37921	37.56117
17540050	Ebp	phenylalkylamine Ca2+ antagonist (emopamil) binding protein	319.3047	269.5128	254.9252	263.8694	282.3127	279.9664
17255992	Pnmt	phenylethanolamine-N-methyltransferase	5.428073	5.561446	5.120306	4.881688	5.120306	5.034959
17351414	Pmaip1	phorbol-12-myristate-13-acetate-induced protein 1	7.250196	11.8539	12.37623	11.24655	13.30964	11.16483
17218073	Pdc	phosducin	4.566662	4.599191	4.751227	3.789375	3.98086	5.972066
17384578	Pdcl	phosducin-like	34.46285	29.71851	28.71077	29.74592	40.35624	30.41498
17448987	Pdcl2	phosducin-like 2	2.353096	3.238644	2.485455	3.76641	2.57964	3.932373
17212080	Pdcl3	phosducin-like 3	43.70511	41.36274	45.68948	39.23393	51.56499	52.26887
17286838	Phactr1	phosphatase and actin regulator 1	3.932781	4.279404	4.288491	3.764792	4.288491	4.788454
17239384	Phactr2	phosphatase and actin regulator 2	15.02153	18.10693	14.69782	14.47489	14.18398	10.1382
17380584	Phactr3	phosphatase and actin regulator 3	18.69253	21.57457	17.79007	22.92977	19.84179	23.12353
17430835	Phactr4	phosphatase and actin regulator 4	50.05215	58.46738	56.71742	51.2539	61.07796	65.47944
17358706	Pten	phosphatase and tensin homolog	550.2906	544.5686	480.2494	491.0956	481.189	524.4786
17241206	Pald1	phosphatase domain containing, paladin 1	10.69274	13.25303	15.57748	18.51189	11.88154	16.07882
17255458	Phospho1	phosphatase, orphan 1	49.38942	70.32667	64.8988	56.5694	23.23423	32.9839
17371485	Phospho2	phosphatase, orphan 2	44.65501	34.18188	41.06593	43.62793	41.97301	44.29924
17324803	Pcyt1a	phosphate cytidylyltransferase 1, choline, alpha isoform	69.8162	71.76607	77.59383	69.22782	66.91279	61.91856
17536264	Pcyt1b	phosphate cytidylyltransferase 1, choline, beta isoform	3.298591	3.392597	3.54226	4.242222	3.425869	3.587329
17273200	Pcyt2	phosphate cytidylyltransferase 2, ethanolamine	248.8703	272.9154	278.1019	280.9618	272.0485	264.2959
17545629	Phex	phosphate regulating endopeptidase homolog, X-linked	3.672672	2.542804	3.265536	3.288592	3.732675	3.408271
17267685	Pctp	phosphatidylcholine transfer protein	181.8216	194.04	230.5251	219.0946	194.2189	205.3071
17451854	Pebp1	phosphatidylethanolamine binding protein 1	9.123011	7.33227	9.123011	10.09767	9.123011	17.69684
17472085	Pbp2	phosphatidylethanolamine binding protein 2	3.630127	2.815166	4.277269	3.237073	3.318543	3.610729
17301708	Pebp4	phosphatidylethanolamine binding protein 4	5.252885	5.118035	5.118035	5.118035	4.773376	4.905227
17263516	Pemt	phosphatidylethanolamine N-methyltransferase	252.5613	225.5682	226.6638	198.7967	242.3889	229.5604
17258887	Pgs1	phosphatidylglycerophosphate synthase 1	24.56683	24.5539	23.23761	23.26179	16.48349	23.87419
17417407	Pik3r3	phosphatidylinositol 3 kinase, regulatory subunit, polypeptide 3 (p55)	8.545767	6.198847	5.142001	8.758477	4.846187	3.7644
17521105	Pik3r4	phosphatidylinositol 3 kinase, regulatory subunit, polypeptide 4, p150	41.85825	41.79041	34.2737	46.1542	46.99092	40.59021
17433169	Pik3cd	phosphatidylinositol 3-kinase catalytic delta polypeptide	8.937244	9.515899	11.14697	8.558275	8.0349	8.691951
17495414	Pik3c2a	phosphatidylinositol 3-kinase, C2 domain containing, alpha polypeptide	105.2255	101.1751	81.55775	87.88165	87.48416	73.70752
17463930	Pik3c2g	phosphatidylinositol 3-kinase, C2 domain containing, gamma polypeptide	9.51214	11.07719	13.04287	11.12829	10.95722	13.38653
17396677	Pik3ca	phosphatidylinositol 3-kinase, catalytic, alpha polypeptide	85.78479	90.67042	84.16975	88.07679	78.62684	97.05227
17530000	Pik3cb	phosphatidylinositol 3-kinase, catalytic, beta polypeptide	50.21582	38.79933	41.67615	43.21619	43.14825	47.22274
17295796	Pik3r1	phosphatidylinositol 3-kinase, regulatory subunit, polypeptide 1 (p85 alpha)	96.151	96.08305	103.4852	82.45357	97.96537	92.40827
17510145	Pik3r2	phosphatidylinositol 3-kinase, regulatory subunit, polypeptide 2 (p85 beta)	26.86377	22.03172	24.56121	25.03076	24.56121	22.53273
17359520	Pi4k2a	phosphatidylinositol 4-kinase type 2 alpha	141.3179	114.8638	124.5844	125.1782	116.4922	121.4771
17437390	Pi4k2b	phosphatidylinositol 4-kinase type 2 beta	44.68411	42.35029	42.74637	39.01806	36.52216	47.76229
17328654	Pi4ka	phosphatidylinositol 4-kinase, catalytic, alpha polypeptide	81.75301	78.68736	85.09427	82.99198	81.56679	74.19808
17400198	Pi4kb	phosphatidylinositol 4-kinase, catalytic, beta polypeptide	34.42632	33.86352	31.28458	33.49445	31.50808	28.59518
17480078	Picalm	phosphatidylinositol binding clathrin assembly protein	358.709	399.1452	416.2481	345.9501	302.2482	307.0096
17539548	Piga	phosphatidylinositol glycan anchor biosynthesis, class A	15.10087	12.21879	10.02166	14.44832	9.291748	12.21879
17528778	Pigb	phosphatidylinositol glycan anchor biosynthesis, class B	21.99311	22.62789	22.80898	22.80898	19.64268	22.07299
17528783	Pigb	phosphatidylinositol glycan anchor biosynthesis, class B	32.15208	24.30987	26.83269	31.91251	24.73599	27.01128
17218713	Pigc	phosphatidylinositol glycan anchor biosynthesis, class C	12.35189	13.63497	13.78426	11.39066	11.56545	11.30101
17347888	Pigf	phosphatidylinositol glycan anchor biosynthesis, class F	16.99549	16.23595	16.23595	14.8288	16.23595	20.93391
17440201	Pigg	phosphatidylinositol glycan anchor biosynthesis, class G	24.4586	23.93052	25.78589	25.13734	20.41263	24.3919
17282139	Pigh	phosphatidylinositol glycan anchor biosynthesis, class H	9.601435	13.9347	11.32414	11.74602	9.716589	11.03183
17403706	Pigk	phosphatidylinositol glycan anchor biosynthesis, class K	76.47573	71.44692	74.06756	72.74872	61.91384	63.21829
17250682	Pigl	phosphatidylinositol glycan anchor biosynthesis, class L	17.32004	18.50727	23.07582	19.09973	19.67375	18.81433
17219542	Pigm	phosphatidylinositol glycan anchor biosynthesis, class M	17.76431	20.64199	19.39765	15.30838	19.07023	19.00451
17225999	Pign	phosphatidylinositol glycan anchor biosynthesis, class N	26.16652	27.71164	25.95013	29.68393	27.27827	22.15115
17424536	Pigo	phosphatidylinositol glycan anchor biosynthesis, class O	22.19219	26.95461	27.51125	25.76695	25.00648	23.82377
17332317	Pigp	phosphatidylinositol glycan anchor biosynthesis, class P	27.69167	33.76461	32.24537	32.40445	30.65754	26.90645
17342509	Pigq	phosphatidylinositol glycan anchor biosynthesis, class Q	95.26009	102.5043	123.2266	115.134	103.2709	104.2982
17253616	Pigs	phosphatidylinositol glycan anchor biosynthesis, class S	31.71448	35.55154	39.65003	37.06588	39.58056	32.83722
17379439	Pigt	phosphatidylinositol glycan anchor biosynthesis, class T	104.0015	115.7525	100.832	103.9164	104.2743	102.3594
17393225	Pigu	phosphatidylinositol glycan anchor biosynthesis, class U	45.48251	44.27223	40.213	31.28003	45.3613	39.09548
17431065	Pigv	phosphatidylinositol glycan anchor biosynthesis, class V	16.31266	18.3321	23.8279	22.24147	16.27652	18.42126
17267102	Pigw	phosphatidylinositol glycan anchor biosynthesis, class W	12.82414	14.94976	14.31296	13.99045	15.98537	16.54015
17329809	Pigx	phosphatidylinositol glycan anchor biosynthesis, class X	25.72163	19.9791	17.14849	22.91007	24.32268	21.49471
17515413	Pigyl	phosphatidylinositol glycan anchor biosynthesis, class Y-like	68.0291	72.16113	51.30295	67.92214	67.58588	69.71465
17324714	Pigz	phosphatidylinositol glycan anchor biosynthesis, class Z	8.32171	9.646797	7.863655	6.844665	7.570875	6.756612
17253024	Pitpna	phosphatidylinositol transfer protein, alpha	139.0433	133.2609	130.2627	115.9613	103.4644	107.3196
17440620	Pitpnb	phosphatidylinositol transfer protein, beta	150.228	128.96	117.0921	117.2497	106.869	108.2438
17271118	Pitpnc1	phosphatidylinositol transfer protein, cytoplasmic 1	95.62527	103.4658	128.0967	103.4866	111.7267	116.3704
17356108	Pitpnm1	phosphatidylinositol transfer protein, membrane-associated 1	12.64452	16.47024	19.89164	17.39354	12.09241	14.78867
17452741	Pitpnm2	phosphatidylinositol transfer protein, membrane-associated 2	42.32212	45.60753	49.00306	44.75261	43.83921	39.43733
17442491	Pitpnm2os1	phosphatidylinositol transfer protein, membrane-associated 2, opposite strand 1	4.754916	4.570145	6.105375	3.943455	3.787878	4.232764
17394582	Prex1	phosphatidylinositol-3,4,5-trisphosphate-dependent Rac exchange factor 1	19.40841	25.05732	25.68315	19.93263	17.18812	18.21898
17211131	Prex2	phosphatidylinositol-3,4,5-trisphosphate-dependent Rac exchange factor 2	25.44588	23.03938	25.14727	24.26778	14.91095	17.26586
17407684	Pip5k1a	phosphatidylinositol-4-phosphate 5-kinase, type 1 alpha	36.14214	32.69436	32.69436	31.24601	30.52247	30.59576
17363605	Pip5k1b	phosphatidylinositol-4-phosphate 5-kinase, type 1 beta	3.312017	3.526336	4.197266	3.21095	3.526336	3.61158
17235663	Pip5k1c	phosphatidylinositol-4-phosphate 5-kinase, type 1 gamma	37.37392	36.02783	36.95993	27.78915	28.01913	32.16032
17369868	Pip5kl1	phosphatidylinositol-4-phosphate 5-kinase-like 1	8.491974	7.537364	7.913303	8.380463	8.441504	9.39993
17382038	Pip4k2a	phosphatidylinositol-5-phosphate 4-kinase, type II, alpha	24.99383	28.75219	34.92335	22.0015	24.99383	24.99383

17268598	Pip4k2b	phosphatidylinositol-5-phosphate 4-kinase, type II, beta	43.40081	32.45638	46.00887	37.81089	39.5323	37.56117
17245785	Pip4k2c	phosphatidylinositol-5-phosphate 4-kinase, type II, gamma	26.47961	35.55006	32.37571	32.3004	35.29079	35.99389
17440361	Plcxd1	phosphatidylinositol-specific phospholipase C, X domain containing 1	12.31692	14.00587	14.91136	14.13167	12.50454	10.23373
17330742	Plcxd2	phosphatidylinositol-specific phospholipase C, X domain containing 2	69.25326	68.8808	64.099	70.5767	59.2741	52.74235
17309825	Plcxd3	phosphatidylinositol-specific phospholipase C, X domain containing 3	2.953407	2.935833	2.704721	3.237702	2.953407	3.730658
17447045	Pisd	phosphatidylserine decarboxylase	33.03813	42.86743	32.68235	35.97044	30.56122	27.68034
17246508	Pisd-ps1	phosphatidylserine decarboxylase, pseudogene 1	6.940983	12.72955	8.89972	11.3349	7.540551	6.152574
17340435	Pisd-ps2	phosphatidylserine decarboxylase, pseudogene 2	4.534646	8.010154	7.478765	6.945459	6.463878	7.169189
17532694	Pisd-ps3	phosphatidylserine decarboxylase, pseudogene 3	26.2288	41.43678	52.71604	62.09994	51.18361	36.19855
17288278	Ptdss1	phosphatidylserine synthase 1	69.9213	71.84023	62.11199	67.93815	62.0909	70.8389
17484752	Ptdss2	phosphatidylserine synthase 2	20.81238	25.23423	26.30162	25.36118	22.51524	24.89959
17333133	Pde10a	phosphodiesterase 10A	10.31727	10.54794	13.84651	10.09228	10.01493	9.816713
17386725	Pde11a	phosphodiesterase 11A	4.695764	4.560209	5.007637	4.204342	4.322842	4.322842
17304240	Pde12	phosphodiesterase 12	28.19427	20.66446	16.98383	21.59851	18.74326	23.44356
17387276	Pde1a	phosphodiesterase 1A, calmodulin-dependent	5.607276	5.913655	5.913655	5.386134	4.840882	7.270779
17315606	Pde1b	phosphodiesterase 1B, Ca2+-calmodulin dependent	5.995541	6.629178	5.995541	4.233057	5.011561	4.704593
17467072	Pde1c	phosphodiesterase 1C	3.004885	3.167556	2.94223	3.217656	3.27785	3.453502
17467100	Pde1c	phosphodiesterase 1C	26.83102	29.96964	24.19455	25.40957	23.9516	29.59251
17480880	Pde2a	phosphodiesterase 2A, cGMP-stimulated	48.2946	41.76058	57.93125	52.57518	31.41823	34.54057
17464040	Pde3a	phosphodiesterase 3A, cGMP inhibited	8.287115	12.79422	9.820184	9.407426	10.39026	9.814195
17482021	Pde3b	phosphodiesterase 3B, cGMP-inhibited	191.3606	190.8202	185.6035	158.3771	224.6663	191.0028
17515106	Pde4a	phosphodiesterase 4A, cAMP specific	14.49406	16.45621	18.17533	16.06423	9.692105	10.87015
17416023	Pde4b	phosphodiesterase 4B, cAMP specific	8.028943	8.028943	7.038412	7.406387	7.781725	8.215509
17502016	Pde4c	phosphodiesterase 4C, cAMP specific	18.18214	20.90361	23.50947	22.81926	23.53441	19.73781
17408211	Pde4dip	phosphodiesterase 4D interacting protein (myomegalin)	61.74241	49.576	59.1272	61.74241	62.78422	51.46929
17289742	Pde4d	phosphodiesterase 4D, cAMP specific	5.395699	6.942378	6.287864	5.810531	6.246552	6.35759
17402321	Pde5a	phosphodiesterase 5A, cGMP-specific	5.319129	7.483017	8.568425	8.15927	5.500528	5.046349
17351082	Pde6a	phosphodiesterase 6A, cGMP-specific, rod, alpha	2.882799	2.64704	3.339703	3.011934	2.567813	3.311213
17440232	Pde6b	phosphodiesterase 6B, cGMP, rod receptor, beta polypeptide	2.55395	3.296374	2.759889	2.88162	2.938134	3.467534
17359118	Pde6c	phosphodiesterase 6C, cGMP specific, cone, alpha prime	4.01461	3.492451	3.452257	4.278277	4.661777	4.818052
17225179	Pde6d	phosphodiesterase 6D, cGMP-specific, rod, delta	25.7063	27.29567	34.74106	31.29434	24.8532	29.31099
17273107	Pde6g	phosphodiesterase 6G, cGMP-specific, rod, gamma	7.870635	9.390813	8.145877	9.491855	7.996563	9.559068
17463842	Pde6h	phosphodiesterase 6H, cGMP-specific, cone, gamma	9.282735	10.04921	9.732834	9.258766	6.868476	9.919999
17404269	Pde7a	phosphodiesterase 7A	11.12821	9.050767	10.00098	9.999944	7.839708	10.48228
17239638	Pde7b	phosphodiesterase 7B	28.85281	34.6492	58.07237	34.6492	29.78161	30.37474
17479668	Pde8a	phosphodiesterase 8A	69.10509	70.27112	79.58419	66.40776	59.13294	73.25016
17295059	Pde8b	phosphodiesterase 8B	7.943473	7.921041	7.925105	7.533812	10.92643	8.666143
17335842	Pde9a	phosphodiesterase 9A	75.03595	49.09145	49.09145	49.09145	47.42283	49.89938
17380222	Pck1	phosphoenolpyruvate carboxykinase 1, cytosolic	2013.263	2608.51	2647.95	2427.137	2449.48	2565.955
17300504	Pck2	phosphoenolpyruvate carboxykinase 2 (mitochondrial)	15.46195	26.25845	20.31214	23.77082	21.66839	23.77082
17242376	Pfkl	phosphofructokinase, liver, B-type	45.37388	45.7684	54.13126	49.68264	78.89216	44.3361
17314577	Pfkm	phosphofructokinase, muscle	12.9465	13.90942	14.21833	12.28469	13.17161	11.91949
17290324	Pfkp	phosphofructokinase, platelet	7.910945	9.488237	11.74952	12.02162	8.119439	8.294586
17361494	Pacs1	phosphofurin acidic cluster sorting protein 1	14.57749	11.72398	18.04616	12.84763	16.69252	17.2661
17279434	Pacs2	phosphofurin acidic cluster sorting protein 2	49.96791	55.0912	75.43698	59.35016	59.96089	52.0202
17437611	Pgm1	phosphoglucomutase 1	25.84763	34.39579	40.45315	27.05025	27.205	24.88579
17415815	Pgm2	phosphoglucomutase 2	277.5935	261.8016	245.5018	239.1861	290.0525	273.4096
17480652	Pgm2l1	phosphoglucomutase 2-like 1	14.32834	17.41043	16.05054	16.27821	20.12993	18.92787
17529383	Pgm3	phosphoglucomutase 3	64.50012	57.08461	47.56604	58.80513	47.11975	47.97364
17363626	Pgm5	phosphoglucomutase 5	9.492776	7.921096	6.709987	6.977451	5.549701	4.973185
17433040	Pgd	phosphogluconate dehydrogenase	77.22789	77.22789	98.59824	65.53756	65.79153	68.17071
17537088	Pgk1	phosphoglycerate kinase 1	627.4123	628.4438	644.113	596.0502	656.6451	711.8059
17344939	Pgk2	phosphoglycerate kinase 2	4.145712	2.970632	3.147879	4.074068	3.263839	3.779198
17359466	Pgam1	phosphoglycerate mutase 1	38.79853	25.13385	35.63344	27.99156	29.56565	24.84132
17260192	Pgam2	phosphoglycerate mutase 2	4.261526	4.132744	3.774407	4.757191	3.48887	3.774407
17450979	Pgam5	phosphoglycerate mutase family member 5	32.66446	34.80518	35.73386	34.46094	28.24141	33.1441
17334262	Pgp	phosphoglycolate phosphatase	59.54465	46.571	54.59625	47.66208	43.63912	44.71447
17382673	Phpt1	phosphohistidine phosphatase 1	14.03357	16.11955	15.8534	19.00306	20.92985	20.80704
17213702	Pikfyve	phosphoinositide kinase, FYVE finger containing	46.46975	43.09444	36.90693	44.16618	46.93785	38.50276
17364642	Pik3ap1	phosphoinositide-3-kinase adaptor protein 1	186.1653	186.1201	186.4416	176.4151	196.6205	186.5386
17246577	Pik3ip1	phosphoinositide-3-kinase interacting protein 1	6.029967	5.507395	5.043552	5.638651	5.647412	5.312018
17280609	Pik3cg	phosphoinositide-3-kinase, catalytic, gamma polypeptide	9.244338	8.70769	10.17777	10.92272	10.48983	9.444864
17217247	Pik3c2b	phosphoinositide-3-kinase, class 2, beta polypeptide	12.06269	14.05518	17.04681	14.81801	12.30574	14.37829
17349100	Pik3c3	phosphoinositide-3-kinase, class 3	44.77868	49.56157	37.33845	47.37782	45.28245	47.601
17251303	Pik3r5	phosphoinositide-3-kinase, regulatory subunit 5, p101	9.626958	9.461048	10.96854	9.869931	8.317616	7.37013
17251328	Pik3r6	phosphoinositide-3-kinase, regulatory subunit 6	11.74561	17.81573	17.4962	13.03339	15.90127	16.72379
17250924	Pirt	phosphoinositide-interacting regulator of transient receptor potential channels	6.752398	5.386134	4.524662	5.386134	7.446487	5.015453
17233323	Pln	phospholamban	6.793147	5.731688	5.611241	4.315746	5.992425	6.671263
17330359	Pla1a	phospholipase A1 member A	43.57866	50.32453	53.55428	48.3435	53.9537	52.41944
17487722	Pinlyp	phospholipase A2 inhibitor and LY6/PLAUR domain containing	20.77908	13.52053	15.319	17.84911	16.36294	15.81454
17385614	Pla2r1	phospholipase A2 receptor 1	9.123011	9.353589	10.01215	9.308637	8.597934	8.497215
17427240	Plaa	phospholipase A2, activating protein	88.92086	74.35923	62.21239	69.27918	64.77746	63.40304
17441160	Pla2g1b	phospholipase A2, group IB, pancreas	3.062691	2.37726	2.630597	2.247857	2.846304	2.598015
17420539	Pla2g2a	phospholipase A2, group IIA (platelets, synovial fluid)	6.831725	6.551612	8.410199	7.933239	7.244821	7.606485
17420519	Pla2g2c	phospholipase A2, group IIC	8.804249	6.552702	8.306208	8.306208	7.263777	9.216059
17420530	Pla2g2d	phospholipase A2, group IID	23.89654	25.79036	24.6711	28.85101	25.34993	28.82223
17420546	Pla2g2e	phospholipase A2, group IIE	17.15765	15.93028	15.66888	17.2655	16.16666	14.1304
17431811	Pla2g2f	phospholipase A2, group IIF	4.776189	4.861866	4.52871	4.96368	4.352065	4.861866
17246602	Pla2g3	phospholipase A2, group III	8.653178	7.081083	8.667945	8.477624	7.712775	7.294201
17227828	Pla2g4a	phospholipase A2, group IVA (cytosolic, calcium-dependent)	13.16176	14.10945	14.91301	12.33486	13.16176	11.32532
17374984	Pla2g4b	phospholipase A2, group IVB (cytosolic)	4.490014	3.473307	5.002919	5.485944	4.890798	4.447825
17473889	Pla2g4c	phospholipase A2, group IVC (cytosolic, calcium-independent)	4.323321	4.100074	3.227976	4.957181	4.404078	4.159557
17390106	Pla2g4d	phospholipase A2, group IVD	3.912444	3.440146	3.833468	3.833468	3.852887	3.833468
17390079	Pla2g4e	phospholipase A2, group IVE	10.17657	8.592155	9.447745	9.628359	8.330771	9.215101

17390131	Pla2g4f	phospholipase A2, group IVF	4.360278	5.020427	5.095452	7.028954	5.020427	6.201469
17431820	Pla2g5	phospholipase A2, group V	3.893183	4.395843	4.68101	3.940722	3.652996	4.635902
17319168	Pla2g6	phospholipase A2, group VI	61.56464	49.979	48.32842	54.94743	43.62097	56.52707
17337796	Pla2g7	phospholipase A2, group VII (platelet-activating factor acetylhydrolase, plasma)	6.171709	9.50746	8.635023	8.182093	6.486364	6.94777
17328277	Pla2g10	phospholipase A2, group X	12.23015	8.269098	10.48228	10.48228	9.218487	11.03473
17323057	Pla2g10os	phospholipase A2, group X, opposite strand	2.606692	2.348631	2.420827	2.348354	2.406006	1.904127
17402588	Pla2g12a	phospholipase A2, group XIIA	15.20612	19.71539	17.64246	14.50089	14.29412	14.12658
17233554	Pla2g12b	phospholipase A2, group XIIB	147.4232	119.5649	107.7422	105.3437	121.2642	101.6284
17505060	Pla2g15	phospholipase A2, group XV	38.02404	38.86905	44.44318	39.03077	37.27483	36.38293
17357072	Pla2g16	phospholipase A2, group XVI	10.64637	19.43399	16.56309	14.31296	14.31296	14.37958
17472114	Plbd1	phospholipase B domain containing 1	222.5908	246.4088	251.562	243.6388	216.0797	217.5963
17451972	Plbd2	phospholipase B domain containing 2	63.96097	66.04512	64.49252	70.01926	53.52051	52.01623
17436246	Plb1	phospholipase B1	3.623314	3.623314	3.941487	3.623314	3.939411	3.618872
17376685	Plcb1	phospholipase C, beta 1	33.00608	36.23369	37.7232	31.88642	31.62361	32.79946
17389740	Plcb2	phospholipase C, beta 2	8.686711	12.5074	12.58392	11.18133	10.39379	9.033907
17362156	Plcb3	phospholipase C, beta 3	35.57791	29.09441	33.38851	28.65589	29.09441	29.34637
17376728	Plcb4	phospholipase C, beta 4	11.24818	10.28443	10.31842	11.24818	7.567045	11.24818
17532045	Plcd1	phospholipase C, delta 1	5.684802	6.677787	5.579052	5.223367	5.684802	5.439032
17270410	Plcd3	phospholipase C, delta 3	9.421717	10.11819	9.759654	9.421717	8.877356	10.47095
17214269	Plcd4	phospholipase C, delta 4	4.272262	4.98295	5.653875	5.394578	4.98295	4.98295
17359160	Plce1	phospholipase C, epsilon 1	23.86112	28.26804	33.46729	27.6215	36.4064	26.84869
17405549	Plch1	phospholipase C, eta 1	4.148759	3.852113	4.631469	4.144534	3.746621	3.985664
17433699	Plch2	phospholipase C, eta 2	5.994419	7.644489	8.066428	7.340814	5.894339	6.746036
17379037	Plcg1	phospholipase C, gamma 1	32.04928	32.76514	37.68661	34.27346	26.89806	29.75314
17505995	Plcg2	phospholipase C, gamma 2	16.23213	12.38498	17.71709	14.03941	11.88797	14.01141
17472321	Plcz1	phospholipase C, zeta 1	3.268775	2.788327	2.67402	2.819873	2.114593	2.853119
17212908	Plcl1	phospholipase C-like 1	4.679194	4.263032	3.786592	3.609528	4.189477	3.933681
17338521	Plcl2	phospholipase C-like 2	21.43431	21.1615	25.29394	20.29525	18.56779	17.67833
17488292	Pld3	phospholipase D family, member 3	41.39458	50.92775	48.93077	40.45427	32.54987	34.78154
17279404	Pld4	phospholipase D family, member 4	32.96516	38.31772	56.0911	25.78875	32.79127	26.67655
17230176	Pld5	phospholipase D family, member 5	3.487226	3.788088	3.176306	3.406995	3.366945	2.963598
17263459	Pld6	phospholipase D family, member 6	18.02841	16.28712	22.62412	23.82146	19.207	21.42391
17396403	Pld1	phospholipase D1	28.26569	20.77649	24.62787	22.07467	21.56082	21.07178
17252081	Pld2	phospholipase D2	13.12122	18.0742	18.83455	15.80369	17.16526	17.53627
17289928	Plpp1	phospholipid phosphatase 1	34.80035	25.87129	41.84809	36.34991	23.06862	18.36415
17242578	Plpp2	phospholipid phosphatase 2	56.63924	51.64977	48.1482	50.15321	68.02957	34.21626
17416234	Plpp3	phospholipid phosphatase 3	166.409	230.2839	237.4286	214.1919	216.1921	204.3587
17483793	Plpp4	phospholipid phosphatase 4	4.395722	5.492679	4.664104	5.156457	4.455385	4.664104
17500172	Plpp5	phospholipid phosphatase 5	106.6596	92.87317	90.27033	75.89958	98.74666	48.75235
17358482	Plpp6	phospholipid phosphatase 6	42.33708	35.44278	37.75406	40.89691	46.21118	40.16481
17369672	Plpp7	phospholipid phosphatase 7 (inactive)	8.99816	7.135923	8.106055	7.880297	7.935109	8.25215
17414045	Plppr1	phospholipid phosphatase related 1	9.328596	10.04719	10.2667	9.784377	9.272832	10.45477
17515358	Plppr2	phospholipid phosphatase related 2	13.60458	20.9676	27.24973	20.01001	12.87839	15.6628
17242707	Plppr3	phospholipid phosphatase related 3	5.66914	7.993626	7.027541	6.171709	6.974743	5.500337
17409816	Plppr4	phospholipid phosphatase related 4	6.081343	6.487263	6.98074	6.690249	7.41548	6.262723
17402099	Plppr5	phospholipid phosphatase related 5	8.249862	6.590372	8.325114	7.57686	7.157719	6.81744
17520315	Plscr1	phospholipid scramblase 1	12.65469	12.25331	17.13222	14.88688	14.11679	15.2696
17520326	Plscr2	phospholipid scramblase 2	44.36438	60.42778	64.98388	70.22778	75.23333	66.58752
17251756	Plscr3	phospholipid scramblase 3	12.03755	17.61533	17.64846	15.66041	15.92024	12.53161
17520353	Plscr4	phospholipid scramblase 4	6.514435	7.626336	7.045148	8.184135	5.553587	6.615912
17520307	Plscr5	phospholipid scramblase family, member 5	5.661483	5.736258	4.509977	5.894829	3.728254	7.087958
17394297	Pltp	phospholipid transfer protein	138.2734	181.131	291.637	170.2216	135.0257	135.5489
17484068	Lhpp	phospholysine phosphohistidine inorganic pyrophosphate phosphatase	98.7624	124.9165	113.91	139.1937	96.75404	132.0948
17319554	Pmm1	phosphomannomutase 1	13.18495	19.68193	22.28591	17.44401	16.22538	17.36499
17322774	Pmm2	phosphomannomutase 2	143.7482	140.0638	124.797	148.054	134.7116	130.6597
17399474	Pmvk	phosphomevalonate kinase	75.02763	63.98359	88.59172	65.01001	91.93422	65.01001
17527511	Ppcdc	phosphopantothenoylcysteine decarboxylase	28.29451	26.12918	26.08774	28.46841	27.69435	32.32078
17429414	Ppcs	phosphopantothenoylcysteine synthetase	34.10544	32.58983	30.2449	34.50085	30.56122	36.15517
17404058	Pag1	phosphoprotein associated with glycosphingolipid microdomains 1	8.344352	8.494329	8.856152	8.600556	8.494329	9.290331
17229842	Pea15a	phosphoprotein enriched in astrocytes 15A	39.34477	46.58262	52.6817	33.25778	35.57446	38.01851
17449091	Pea15b	phosphoprotein enriched in astrocytes 15B	4.568657	4.356652	4.886969	4.886969	4.71455	5.736696
17449033	Ppat	phosphoribosyl pyrophosphate amidotransferase	52.0624	63.39908	60.14321	38.06465	43.08548	59.0236
17538161	Prps1	phosphoribosyl pyrophosphate synthetase 1	36.01161	66.23583	38.34908	30.6705	46.04332	45.33615
17274965	Prps1l1	phosphoribosyl pyrophosphate synthetase 1-like 1	5.478686	5.300432	5.300432	4.896192	4.965744	5.815971
17275612	Prps1l3	phosphoribosyl pyrophosphate synthetase 1-like 3	16.66778	17.77614	13.23944	14.17454	16.36612	16.1352
17546119	Prps2	phosphoribosyl pyrophosphate synthetase 2	18.23085	18.68813	19.12569	20.04902	18.10957	16.9576
17272396	Prpsap1	phosphoribosyl pyrophosphate synthetase-associated protein 1	63.45008	65.14308	65.24566	64.22887	53.63601	67.62524
17263877	Prpsap2	phosphoribosyl pyrophosphate synthetase-associated protein 2	17.36523	16.23595	17.36523	15.98792	20.44477	16.57389
17438423	Paics	phosphoribosylaminoimidazole carboxylase, phosphoribosylaminoribosylaminoimic	193.3392	174.6533	210.5574	179.2277	164.4391	184.7192
17264482	Pfas	phosphoribosylformylglycinamidine synthase (FGAR amidotransferase)	12.41059	12.77366	12.53708	13.09996	10.80218	13.83355
17332140	Gart	phosphoribosylglycinamide formyltransferase	59.16122	50.46216	48.36327	50.29315	56.20319	55.09617
17543691	Phka1	phosphorylase kinase alpha 1	23.21781	16.36532	16.0941	19.42873	18.10153	18.8616
17539303	Phka2	phosphorylase kinase alpha 2	36.85708	37.82374	39.43268	39.81582	40.64849	47.23093
17503462	Phkb	phosphorylase kinase beta	46.71681	43.75906	39.35404	42.27609	31.89437	38.31082
17453140	Phkg1	phosphorylase kinase gamma 1	6.187557	5.398356	6.942378	5.045265	5.293933	4.930418
17483385	Phkg2	phosphorylase kinase, gamma 2 (testis)	56.38541	45.10733	43.02639	45.11827	45.56929	35.58013
17350718	Phax	phosphorylated adaptor for RNA export	15.02736	18.60076	16.99714	19.19129	19.15886	17.65078
17363204	Psat1	phosphoserine aminotransferase 1	5.441295	7.206306	5.849162	5.625027	6.723815	5.835272
17453124	Psph	phosphoserine phosphatase	17.74475	14.52615	14.61158	14.88224	13.57857	14.91105
17484005	Pstk	phosphoseryl-tRNA kinase	27.81845	18.00593	18.48432	22.00547	23.82278	21.53901
17367036	Pter	phosphotriesterase related	128.391	91.968	107.0959	101.0195	132.1479	117.322
17224971	Pid1	phosphotyrosine interaction domain containing 1	119.4961	132.9592	113.7466	125.4675	140.2114	120.4825
17369131	Phyhd1	phytanoyl-CoA dioxygenase domain containing 1	49.88935	55.92339	69.20527	59.03833	62.88395	55.80722
17366511	Phyh	phytanoyl-CoA hydroxylase	872.0578	875.1825	742.1876	907.1517	721.4322	776.908

17301738	Phyhip	phytanoyl-CoA hydroxylase interacting protein	5.036355	4.500858	5.036355	5.036355	5.945384	4.886969
17241709	Phyhipl	phytanoyl-CoA hydroxylase interacting protein-like	4.739221	4.086767	5.442615	4.657255	4.061605	4.769672
17241729	Phyhipl	phytanoyl-CoA hydroxylase interacting protein-like	2.367214	2.031	2.056427	1.993647	2.031	2.098168
17434833	Pclo	piccolo (presynaptic cytomatrix protein)	3.757231	3.551233	4.149421	3.908161	4.074689	3.822801
17513713	Piezo1	piezo-type mechanosensitive ion channel component 1	13.64443	17.32724	20.91303	17.4364	12.52892	14.78159
17354906	Piezo2	piezo-type mechanosensitive ion channel component 2	4.728426	5.722676	6.018233	5.335783	5.821294	5.722676
17518596	Pif1	PIF1 5-to-3 DNA helicase homolog (S. cerevisiae)	5.036355	5.036355	5.452168	5.036355	4.796659	5.036355
17290939	Pgbd1	piggyBac transposable element derived 1	3.735652	3.442484	5.336745	4.186429	4.149967	4.023637
17514063	Pgbd5	piggyBac transposable element derived 5	5.29687	6.208006	5.292502	6.318929	5.516373	5.629786
17467166	Pyurf	Pigy upstream reading frame	31.80959	34.13098	31.38362	32.04572	31.37318	38.46782
17477698	Pih1d1	PIH1 domain containing 1	47.63467	45.51846	51.71067	42.29367	46.0925	45.12328
17517120	Pih1d2	PIH1 domain containing 2	3.005885	3.263862	3.134575	3.263862	2.881859	3.757726
17211541	Pih1d3	PIH1 domain containing 3	2.646783	2.8568	2.529742	2.326643	2.526342	1.975804
17538141	Pih1h3b	PIH1 domain containing 3B	6.280807	8.358361	10.38054	11.50852	10.91137	12.28529
17462987	Pianp	PILR alpha associated neural protein	6.003567	8.765583	9.189587	11.5078	9.53313	9.095103
17301247	Pinx1	PIN2/TERF1 interacting, telomerase inhibitor 1	16.48669	17.03512	16.48669	14.47489	14.39823	15.69605
17275706	Pnn	pinin	44.33948	56.64709	60.71411	50.70022	49.22775	57.49442
17266302	Pipox	pipecolic acid oxidase	371.4824	425.5663	405.5792	409.2135	410.8278	408.0805
17539519	Pir	pirin	38.02167	43.60314	34.92262	37.22784	34.64271	39.33086
17431516	Pithd1	PITH (C-terminal proteasome-interacting domain of thioredoxin-like) domain cont	15.51713	14.3076	15.174	16.44697	16.36362	18.12526
17265570	Pitpnm3	PITPNM family member 3	4.883481	4.155379	5.400901	4.994188	4.913428	3.959999
17285007	Pitrm1	pitrilysin metallepetidase 1	127.7722	114.9617	122.1796	125.1609	111.7236	108.6738
17234667	Pttg1ip	pituitary tumor-transforming 1 interacting protein	229.7451	258.2832	234.1177	250.5891	228.6948	238.1093
17261944	Pttg1	pituitary tumor-transforming gene 1	16.77732	19.48673	25.69312	17.19456	20.39693	20.39693
17442800	Piwil1	piwi-like RNA-mediated gene silencing 1	3.605352	4.165588	4.117989	3.821052	3.870661	3.928667
17308319	Piwil2	piwi-like RNA-mediated gene silencing 2	3.311328	4.929803	4.321344	4.884768	3.861844	4.941127
17524049	Piwil4	piwi-like RNA-mediated gene silencing 4	3.255637	3.888571	3.06979	3.797053	5.206558	4.160768
17354029	Plac8l1	PLAC8-like 1	2.611701	2.384125	2.269886	2.603153	2.970035	2.384125
17517097	Plet1	placenta expressed transcript 1	17.50336	12.98352	15.2769	14.10877	10.47029	16.60217
17526809	Plet1os	placenta expressed transcript 1, opposite strand	5.300432	5.300432	6.287254	4.737349	5.300432	4.531284
17304155	Plac9b	placenta specific 9b	18.08648	19.72427	17.84255	18.46383	18.28083	20.87407
17304170	Plac9b	placenta specific 9b	18.08648	19.72427	17.84255	18.46383	18.28083	20.87407
17304186	Plac9b	placenta specific 9b	20.14193	20.14193	18.45628	20.00918	19.06838	21.6048
17282664	Pgf	placental growth factor	3.463114	3.560648	3.898259	4.003301	4.167956	3.764818
17541736	Plac1	placental specific protein 1	4.075621	3.80807	3.745946	3.337528	4.558534	3.656948
17450121	Plac8	placenta-specific 8	15.8678	17.94114	23.30552	18.89235	17.90325	16.97406
17227384	Pkp1	plakophilin 1	6.395335	6.022114	5.712491	7.498593	6.236385	5.707188
17323302	Pkp2	plakophilin 2	83.59994	68.97157	66.67297	68.18441	74.99071	61.23141
17484731	Pkp3	plakophilin 3	8.74501	13.19949	10.37148	10.41665	10.37148	11.12945
17370977	Pkp4	plakophilin 4	76.27667	84.88337	84.90514	76.19741	67.30212	80.60235
17511878	Pllp	plasma membrane proteolipid	47.70141	46.47104	44.48262	46.47104	44.02771	36.90438
17543106	Pet2	plasmacytoma expressed transcript 2	4.717982	5.576384	4.270929	4.016197	6.534378	5.092592
17311853	Pvt1	plasmacytoma variant translocation 1	5.044565	5.396896	6.026373	5.442879	6.494147	5.537482
17510338	Plvap	plasmalemma vesicle associated protein	60.58043	73.69424	73.82717	58.21988	39.70421	54.59928
17333267	Plg	plasminogen	1922.556	1851.617	1858.96	1864.729	2291.807	1916.237
17499962	Plat	plasminogen activator, tissue	12.55535	13.39052	19.69776	13.16395	10.48228	8.480915
17297537	Plau	plasminogen activator, urokinase	8.714875	9.361307	9.91237	10.00341	10.15086	11.24366
17474974	Plaur	plasminogen activator, urokinase receptor	6.909973	9.426159	8.561657	8.493564	11.06414	8.821842
17363799	Plgrkt	plasminogen receptor, C-terminal lysine transmembrane protein	9.075329	16.88699	18.383	14.22158	13.0607	19.08967
17529764	Pls1	plastin 1 (I-isoform)	18.43902	23.29454	23.94263	26.61357	25.61967	28.72012
17542853	Pls3	plastin 3 (T-isoform)	71.11589	82.02717	72.06016	74.61894	73.05545	80.70969
17438246	Pdgfra	platelet derived growth factor receptor, alpha polypeptide	18.84252	25.49213	22.19988	16.17877	19.03708	17.69684
17351027	Pdgfrb	platelet derived growth factor receptor, beta polypeptide	23.14903	25.10756	25.92209	24.96959	17.26095	17.55877
17454345	Pdgfa	platelet derived growth factor, alpha	19.27061	24.54181	22.21373	23.74991	21.21393	25.63068
17319380	Pdgfb	platelet derived growth factor, B polypeptide	10.61601	13.66241	11.58696	13.05798	8.532331	10.27211
17406552	Pear1	platelet endothelial aggregation receptor 1	11.18249	16.23145	15.45262	13.86658	15.50434	15.45262
17438969	Pf4	platelet factor 4	5.434962	6.595079	7.698994	5.403484	6.22494	8.368622
17270937	Pecam1	platelet/endothelial cell adhesion molecule 1	42.26613	56.45502	64.72629	45.97762	35.88403	35.775
17419754	Pafah2	platelet-activating factor acetylhydrolase 2	64.60885	55.49824	53.00072	61.11311	58.78679	62.41929
17265853	Pafah1b1	platelet-activating factor acetylhydrolase, isoform 1b, subunit 1	89.12387	102.5433	91.88707	104.2208	105.2138	119.1075
17526614	Pafah1b2	platelet-activating factor acetylhydrolase, isoform 1b, subunit 2	76.69444	76.69444	78.15737	75.76292	64.43712	71.16175
17487905	Pafah1b3	platelet-activating factor acetylhydrolase, isoform 1b, subunit 3	9.018203	8.631911	7.592	8.377037	7.76373	9.021767
17419437	Ptafr	platelet-activating factor receptor	11.60223	15.95095	11.42973	9.553835	12.70796	17.18097
17500735	Pdgfrl	platelet-derived growth factor receptor-like	13.11713	13.5437	16.97136	8.938669	11.80986	11.52992
17398416	Pdgfc	platelet-derived growth factor, C polypeptide	36.12235	32.52484	35.15944	23.23117	20.43698	17.46434
17514460	Pdgfd	platelet-derived growth factor, D polypeptide	5.577273	5.313307	6.345659	6.03829	4.662843	6.617471
17260761	Plek	pleckstrin	18.16312	22.48545	25.21744	16.92589	18.17697	18.76913
17282117	Plek2	pleckstrin 2	12.37014	11.30085	12.48272	9.885062	11.30085	12.88482
17365347	Psd	pleckstrin and Sec7 domain containing	4.516797	7.282531	6.943774	6.943774	6.293663	7.625595
17349607	Psd2	pleckstrin and Sec7 domain containing 2	4.20927	4.316524	5.120306	4.308214	4.20927	3.786251
17509723	Psd3	pleckstrin and Sec7 domain containing 3	50.35315	48.55541	54.30413	45.42548	43.19493	47.36403
17367698	Psd4	pleckstrin and Sec7 domain containing 4	8.98476	9.542452	9.227373	8.570207	12.96464	9.227181
17483891	Plekha1	pleckstrin homology domain containing, family A (phosphoinositide binding speci	23.78747	29.3936	30.2384	24.82153	24.28326	22.82432
17477856	Plekha4	pleckstrin homology domain containing, family A (phosphoinositide binding speci	10.7772	9.902412	10.67601	9.902412	12.7741	9.902412
17458734	Plekha8	pleckstrin homology domain containing, family A (phosphoinositide binding speci	40.50281	30.00768	36.98504	31.6361	28.90365	29.01285
17463973	Plekha5	pleckstrin homology domain containing, family A member 5	54.37268	59.74433	60.31772	62.10554	53.04482	50.74838
17217291	Plekha6	pleckstrin homology domain containing, family A member 6	45.43367	51.59502	50.62401	52.4089	42.69455	60.13654
17495373	Plekha7	pleckstrin homology domain containing, family A member 7	14.57062	12.54169	14.57062	14.36743	10.7481	12.23719
17493807	Plekhb1	pleckstrin homology domain containing, family B (evectins) member 1	45.58256	53.14934	56.54196	38.72384	30.27636	31.23323
17211774	Plekhb2	pleckstrin homology domain containing, family B (evectins) member 2	40.62962	39.85807	38.93512	29.11075	21.11593	21.67921
17276864	Plekhd1	pleckstrin homology domain containing, family D (with coiled-coil domains) meml	3.558798	4.148184	3.894397	3.883991	3.883991	3.707534
17282281	Plekhd1os	pleckstrin homology domain containing, family D (with coiled-coil domains) meml	5.728153	7.639404	6.103217	6.648689	6.841724	5.249827
17489905	Plekhf1	pleckstrin homology domain containing, family F (with FYVE domain) member 1	35.88254	25.72394	28.61342	28.1888	32.25028	35.0026
17423209	Plekhf2	pleckstrin homology domain containing, family F (with FYVE domain) member 2	35.91862	37.45795	33.23405	35.97643	32.37324	38.60699

17231366	Plekhg1	pleckstrin homology domain containing, family G (with RhoGef domain) member 1	13.30208	13.15409	14.06532	10.51677	7.946557	11.15408
17488436	Plekhg2	pleckstrin homology domain containing, family G (with RhoGef domain) member 2	12.80389	14.21025	12.96006	13.66999	15.03987	13.81209
17276591	Plekhg3	pleckstrin homology domain containing, family G (with RhoGef domain) member 3	29.66171	32.11332	34.50731	23.97136	23.88495	33.6849
17504780	Plekhg4	pleckstrin homology domain containing, family G (with RhoGef domain) member 4	5.028925	3.931037	4.173535	3.703322	3.703322	4.212977
17422066	Plekhg5	pleckstrin homology domain containing, family G (with RhoGef domain) member 5	12.65679	15.59812	15.40717	11.65858	13.81027	12.78978
17471107	Plekhg6	pleckstrin homology domain containing, family G (with RhoGef domain) member 6	16.00299	23.38287	18.47638	21.03034	23.51322	21.51215
17276743	Plekhh1	pleckstrin homology domain containing, family H (with MyTH4 domain) member 1	5.644516	5.300432	6.45224	5.885031	5.578008	5.644516
17340050	Plekhh2	pleckstrin homology domain containing, family H (with MyTH4 domain) member 2	5.27262	7.139881	6.447635	5.382178	6.730016	6.15637
17269785	Plekhh3	pleckstrin homology domain containing, family H (with MyTH4 domain) member 3	6.152829	6.938337	6.890315	7.045636	6.466119	8.044485
17243093	Plekhj1	pleckstrin homology domain containing, family J member 1	28.01874	25.9255	28.11133	24.45385	19.62676	26.26664
17270508	Plekhm1	pleckstrin homology domain containing, family M (with RUN domain) member 1	51.95317	52.51945	52.31744	46.74953	45.45005	50.15738
17432264	Plekhm2	pleckstrin homology domain containing, family M (with RUN domain) member 2	26.62778	24.47963	26.62778	26.11669	24.7643	20.22699
17223799	Plekhm3	pleckstrin homology domain containing, family M, member 3	10.91031	10.2917	11.91015	11.42007	9.872668	10.58258
17434027	Plekhn1	pleckstrin homology domain containing, family N member 1	4.334633	5.285976	5.617429	5.241319	5.32577	5.277776
17407969	Plekho1	pleckstrin homology domain containing, family O member 1	24.69317	24.73257	31.64531	28.87882	21.96265	27.18577
17528274	Plekho2	pleckstrin homology domain containing, family O member 2	17.84094	19.14811	20.71552	17.64283	17.33601	17.53311
17360570	Plekhs1	pleckstrin homology domain containing, family S member 1	5.526017	5.676336	5.011839	6.127718	6.494057	7.533394
17529231	Phip	pleckstrin homology domain interacting protein	39.40864	47.72655	45.86336	43.91281	45.51626	51.66409
17508336	Plekha2	pleckstrin homology domain-containing, family A (phosphoinositide binding speci	12.58968	15.73745	14.71902	13.23659	13.72838	13.72838
17372242	Plekha3	pleckstrin homology domain-containing, family A (phosphoinositide binding speci	46.85854	53.30504	47.01274	46.16668	41.39458	45.56013
17237280	Phlda1	pleckstrin homology-like domain, family A, member 1	99.97462	38.4337	63.18937	53.10037	58.53753	61.01068
17498251	Phlda2	pleckstrin homology-like domain, family A, member 2	3.4988	3.179522	3.483602	3.348233	3.78906	3.140621
17217619	Phlda3	pleckstrin homology-like domain, family A, member 3	19.0516	19.22791	20.01473	18.61186	24.57007	20.04916
17526315	Phldb1	pleckstrin homology-like domain, family B, member 1	9.416298	12.41859	16.4634	11.36354	11.80225	10.9817
17330708	Phldb2	pleckstrin homology-like domain, family B, member 2	54.61253	49.96098	48.09517	45.09326	53.36123	54.04278
17475037	Phldb3	pleckstrin homology-like domain, family B, member 3	10.90706	12.60295	13.97445	12.54595	11.38901	13.26902
17318350	Plec	plectin	42.31361	37.903	43.04489	41.60572	41.35099	36.95788
17422996	Plag1	pleiomorphic adenoma gene 1	6.648597	7.18008	6.695445	5.781605	4.714844	6.537912
17231694	Plagl1	pleiomorphic adenoma gene-like 1	6.992896	7.004004	6.992896	5.962304	6.361998	6.992896
17393036	Plagl2	pleiomorphic adenoma gene-like 2	11.17033	12.62735	14.31296	13.39423	12.70294	10.70594
17465856	Ptn	pleiotrophin	3.08823	4.66	3.787485	3.803981	3.915971	3.180735
17398512	Plrg1	pleiotropic regulator 1, PRL1 homolog (Arabidopsis)	80.24558	76.63545	71.23232	73.22782	81.57301	66.32956
17468828	Plxna1	plexin A1	27.09454	28.58983	27.09454	25.36118	27.09454	26.24499
17220974	Plxna2	plexin A2	62.89441	36.44073	41.69753	28.53518	35.70193	30.74735
17535841	Plxna3	plexin A3	4.101751	5.9695	6.677509	4.705246	5.686654	4.705246
17465636	Plxna4	plexin A4	8.338686	8.743495	7.359613	8.51685	8.886344	12.09065
17457056	Plxna4os1	plexin A4, opposite strand 1	4.943525	4.408384	4.297686	3.558601	4.297686	3.847256
17457052	Plxna4os2	plexin A4, opposite strand 2 [Source:MGI Symbol;Acc:MGI:3045237]	19.40814	19.42873	17.7252	18.07746	15.2678	17.7252
17457047	Plxna4os3	plexin A4, opposite strand 3 [Source:MGI Symbol;Acc:MGI:3651753]	3.121068	2.840687	3.155109	3.058279	3.831794	3.647328
17522167	Plxnb1	plexin B1	32.45098	30.69624	31.63852	34.87618	54.76112	36.97735
17320337	Plxnb2	plexin B2	213.7855	226.8477	233.8501	227.61	211.878	188.1957
17535644	Plxnb3	plexin B3	4.682114	5.35108	4.800451	5.102839	4.800451	5.113886
17244439	Plxnc1	plexin C1	73.66615	57.77086	83.85123	75.54844	40.99909	38.84185
17469967	Plxnd1	plexin D1	33.63147	29.97047	38.24104	31.70064	21.39527	26.16761
17268661	Plxdc1	plexin domain containing 1	6.072708	5.569122	6.618113	7.219405	9.546355	6.707987
17367216	Plxdc2	plexin domain containing 2	12.73714	12.50177	14.26037	12.17466	11.08187	12.1418
17405998	Platr10	pluripotency associated transcript 10	4.499485	4.057188	4.804379	4.281837	4.702148	4.113795
17248522	Platr13	pluripotency associated transcript 13 [Source:MGI Symbol;Acc:MGI:3651752]	19.44261	25.53015	21.17016	26.37977	19.9057	27.22179
17373844	Platr14	pluripotency associated transcript 14	2.953407	2.452274	3.142538	2.913514	2.926792	2.937482
17392718	Platr15	pluripotency associated transcript 15 [Source:MGI Symbol;Acc:MGI:3650105]	99.62804	100.019	113.4946	90.08469	96.161	94.51622
17421327	Platr16	pluripotency associated transcript 16	2.812533	4.204488	4.045702	4.371868	3.431002	3.584172
17336209	Platr17	pluripotency associated transcript 17	3.275375	2.727464	3.250564	3.615287	2.919497	2.751141
17274114	Platr19	pluripotency associated transcript 19 [Source:MGI Symbol;Acc:MGI:4936971]	4.20927	3.914668	4.11237	4.726787	4.352065	4.14462
17288223	Platr2	pluripotency associated transcript 2 [Source:MGI Symbol;Acc:MGI:5477252]	1.941269	1.95381	2.747177	2.126513	2.188163	2.801482
17262464	Platr20	pluripotency associated transcript 20	7.320032	8.158647	7.448087	8.844803	7.600741	8.201683
17544845	Platr21	pluripotency associated transcript 21	3.379547	3.757179	3.80836	3.753613	3.783863	3.721644
17217830	Platr22	pluripotency associated transcript 22	4.329019	4.681043	3.477523	5.873783	4.681043	4.358918
17227512	Platr23	pluripotency associated transcript 23 [Source:MGI Symbol;Acc:MGI:5477092]	2.04327	2.355837	2.206883	2.276094	2.61143	1.978394
17293521	Platr25	pluripotency associated transcript 25	3.405555	4.136126	3.222008	4.857386	5.873865	2.918223
17293534	Platr25	pluripotency associated transcript 25	2.732118	1.765039	1.889913	1.978339	3.438907	2.241897
17371790	Platr26	pluripotency associated transcript 26	8.611413	5.586249	6.185199	7.446332	10.51699	8.052741
17393766	Platr27	pluripotency associated transcript 27	8.673474	9.401168	10.99069	13.22513	9.453575	11.86319
17481535	Platr28	pluripotency associated transcript 28 [Source:MGI Symbol;Acc:MGI:3645719]	2.865486	2.450676	2.909881	3.592754	2.99748	3.09908
17394529	Platr29	pluripotency associated transcript 29 [Source:MGI Symbol;Acc:MGI:3650001]	4.05986	3.776501	3.767611	3.662046	2.938745	4.185471
17376723	Platr3	pluripotency associated transcript 3	4.4851	4.878677	5.776317	4.08136	4.741028	4.540276
17400570	Platr30	pluripotency associated transcript 30	3.652008	3.981439	4.095757	4.555091	3.665093	3.399942
17463344	Platr31	pluripotency associated transcript 31 [Source:MGI Symbol;Acc:MGI:5477339]	5.443747	5.956397	5.940894	5.914557	7.589685	4.644972
17479644	Platr32	pluripotency associated transcript 32 [Source:MGI Symbol;Acc:MGI:3801726]	2.2594	1.968887	2.343086	2.871789	2.357193	2.252551
17405017	Platr4	pluripotency associated transcript 4	14.0508	14.31296	14.31296	16.04998	11.64073	11.51554
17215557	Platr5	pluripotency associated transcript 5	3.843418	3.472705	3.783522	5.281691	3.589518	4.44653
17239687	Platr6	pluripotency associated transcript 6	6.001113	4.84528	4.623771	4.82497	5.120306	5.975709
17243621	Platr7	pluripotency associated transcript 7	3.753353	3.813696	3.715638	4.039117	4.914007	4.039117
17389132	Platr8	pluripotency associated transcript 8	3.322156	2.845377	2.858285	3.58621	2.985557	3.49846
17423910	Platr9	pluripotency associated transcript 9	2.51734	2.419921	2.255364	2.478264	2.216679	2.220108
17216169	Panct2	pluripotency-associated noncoding transcript 2	4.921609	4.806877	5.592216	4.954845	4.868644	5.062675
17489273	Pmis2	Pmis2, sperm specific protein	2.188421	2.175716	2.402339	2.448333	2.178522	2.34645
17336157	Pram1	PML-RAR alpha-regulated adaptor molecule 1	4.2352	4.658999	4.140543	4.309618	5.727319	4.1855
17474227	Pnmal1	PNMA-like 1	4.291673	4.191306	4.074294	4.376841	4.775825	4.785402
17474224	Pnmal2	PNMA-like 2	7.796843	7.00959	7.12826	6.973293	6.953359	6.170725
17412166	Pnisr	PNN interacting serine/arginine-rich	24.36402	31.17375	29.59907	27.4701	30.97903	34.0129
17521178	Poc1a	POC1 centriolar protein homolog A (Chlamydomonas)	10.87422	14.31246	12.17871	14.65773	15.06898	13.21844
17236860	Poc1b	POC1 centriolar protein homolog B (Chlamydomonas)	19.36259	19.16776	14.80703	17.70531	24.37026	20.20653
17289289	Poc5	POC5 centriolar protein homolog (Chlamydomonas)	9.393742	8.997723	9.400581	7.788794	13.12277	10.99092
17465620	Podxl	podocalyxin-like	14.50944	15.04831	14.85771	13.69778	9.961272	12.7143

17468780	Podxl2	podocalyxin-like 2	10.03	10.51641	10.17975	9.361307	10.14819	7.770945
17427970	Podn	podocan	7.102171	10.32579	9.361307	8.417839	9.091132	9.450561
17503094	Podnl1	podocan-like 1	5.610425	5.263126	5.584817	6.820843	4.504291	7.105667
17432440	Pdpn	podoplanin	10.56419	11.40995	9.095292	11.48127	10.72793	11.16561
17229336	Pogk	pogo transposable element with KRAB domain	3.794316	4.960447	5.596054	4.013052	4.215752	4.997027
17400139	Pogz	pogo transposable element with ZNF domain	23.48976	26.50091	31.94792	25.50061	26.42113	29.27708
17487489	Pvr	poliovirus receptor	38.58986	41.27053	34.02728	33.25455	29.67529	33.10139
17516450	Pvrl1	poliovirus receptor-related 1	93.93103	91.51904	95.71546	93.46472	113.361	88.45305
17487408	Pvrl2	poliovirus receptor-related 2	58.22916	53.19357	52.77746	58.29499	51.21165	53.09448
17330773	Pvrl3	poliovirus receptor-related 3	58.71674	58.18196	51.27985	55.50785	68.06494	64.25069
17219308	Pvrl4	poliovirus receptor-related 4	7.655518	9.152907	6.459981	7.269116	7.269116	7.563794
17482739	Plk1	polo-like kinase 1	10.3067	9.16065	9.797068	11.06675	8.799247	8.847562
17289794	Plk2	polo-like kinase 2	45.5715	44.3275	33.60398	48.32005	40.51376	37.02737
17428766	Plk3	polo-like kinase 3	144.4273	204.0502	164.258	43.34789	63.49935	73.78164
17397268	Plk4	polo-like kinase 4	4.497677	3.851259	3.807252	3.948159	3.93101	4.31817
17235329	Plk5	polo-like kinase 5	10.03703	8.532036	7.941822	9.502519	13.20507	9.945611
17278436	Papola	poly (A) polymerase alpha	200.5322	187.3056	180.4769	177.4895	173.2606	158.4946
17454687	Papolb	poly (A) polymerase beta (testis specific)	3.453693	4.80841	3.479596	3.709256	5.056643	4.491384
17298528	Parg	poly (ADP-ribose) glycohydrolase	26.56638	28.29411	27.56107	27.56107	26.61431	25.56982
17220122	Parp1	poly (ADP-ribose) polymerase family, member 1	77.5046	75.32569	75.22655	77.22789	69.44361	73.58042
17318407	Parp10	poly (ADP-ribose) polymerase family, member 10	38.36051	35.90443	32.5677	35.12314	30.72391	29.75768
17463358	Parp11	poly (ADP-ribose) polymerase family, member 11	16.26152	12.56789	11.71455	20.77764	14.42161	16.75837
17466060	Parp12	poly (ADP-ribose) polymerase family, member 12	116.1399	107.1316	125.2138	141.8203	104.398	141.1015
17330099	Parp14	poly (ADP-ribose) polymerase family, member 14	92.59677	77.39508	85.00996	107.1539	90.11779	80.26727
17518500	Parp16	poly (ADP-ribose) polymerase family, member 16	46.09377	35.5789	45.13642	39.04051	41.22307	42.5259
17299518	Parp2	poly (ADP-ribose) polymerase family, member 2	18.64116	19.94935	21.1615	18.43787	17.99782	25.79805
17530703	Parp3	poly (ADP-ribose) polymerase family, member 3	38.67475	41.7574	39.31015	39.12042	40.86801	28.13453
17300802	Parp4	poly (ADP-ribose) polymerase family, member 4	32.61966	30.16116	32.97544	36.86424	29.14366	32.0253
17517981	Parp6	poly (ADP-ribose) polymerase family, member 6	17.41091	17.11904	19.2829	20.57128	19.64186	18.3246
17296436	Parp8	poly (ADP-ribose) polymerase family, member 8	7.364768	11.84166	7.20311	8.600795	8.404314	9.396868
17325274	Parp9	poly (ADP-ribose) polymerase family, member 9	74.97096	59.14195	64.90105	81.3077	78.41567	69.74994
17468261	Paip2b	poly(A) binding protein interacting protein 2B	7.555514	6.110795	6.693125	9.355145	7.506394	7.0272
17316521	Pabpc1	poly(A) binding protein, cytoplasmic 1	13.43645	13.43645	13.21184	12.01005	13.01118	12.40767
17379337	Pabpc1l	poly(A) binding protein, cytoplasmic 1-like	7.188845	6.113421	6.065094	5.458294	5.251101	6.364909
17548821	Pabpc1l2b-ps	poly(A) binding protein, cytoplasmic 1-like 2B, pseudogene	7.329628	7.63718	7.64576	7.698994	6.900735	9.801007
17548871	Pabpc1l2b-ps	poly(A) binding protein, cytoplasmic 1-like 2B, pseudogene	7.329628	7.63718	7.64576	7.698994	6.900735	9.801007
17350074	Pabpc2	poly(A) binding protein, cytoplasmic 2	3.326212	4.138649	2.809046	3.178597	3.000556	2.994469
17418187	Pabpc4	poly(A) binding protein, cytoplasmic 4	14.9483	14.52222	13.39423	11.14759	12.82521	13.5545
17405062	Pabpc4l	poly(A) binding protein, cytoplasmic 4-like	3.023207	3.712446	3.657125	3.284491	3.680291	4.478265
17537351	Pabpc5	poly(A) binding protein, cytoplasmic 5	4.163509	4.743263	4.029274	4.204604	4.833305	4.495994
17340748	Pabpc6	poly(A) binding protein, cytoplasmic 6	5.399778	4.358797	5.439937	5.0969	4.333552	5.462911
17300360	Pabpn1	poly(A) binding protein, nuclear 1	44.90798	63.77239	66.79786	64.25202	61.32783	76.58925
17261122	Papolg	poly(A) polymerase gamma	13.08271	13.08271	10.39703	12.99081	9.18411	11.04958
17513796	Pabpn1l	poly(A)binding protein nuclear 1-like	11.84963	10.95107	11.8906	10.45108	10.41333	10.90192
17302331	Gm5088	poly(A)-binding protein, cytoplasmic pseudogene	24.44504	21.39838	25.00717	23.35436	20.34144	23.15561
17328251	Parn	poly(A)-specific ribonuclease (deadenylation nuclease)	48.30163	50.21651	49.17523	51.09428	64.9177	53.45711
17340917	Pnldc1	poly(A)-specific ribonuclease (PARN)-like domain containing 1	6.200738	9.020016	8.271907	6.291308	11.87981	8.52506
17468505	Pcbp1	poly(rC) binding protein 1	168.8256	130.0462	117.1627	137.6595	120.5301	112.763
17315447	Pcbp2	poly(rC) binding protein 2	450.802	453.7142	498.4725	412.2884	474.8243	474.7091
17242207	Pcbp3	poly(rC) binding protein 3	7.051548	7.593895	7.894074	8.42829	5.737093	7.607856
17521218	Pcbp4	poly(rC) binding protein 4	31.72042	41.79533	48.36596	45.93717	40.97977	41.6288
17290121	Paip1	polyadenylate binding protein-interacting protein 1	115.9035	115.881	122.5087	122.2313	124.2171	114.8439
17349572	Paip2	polyadenylate-binding protein-interacting protein 2	62.71419	67.29186	52.00398	59.51412	59.85998	68.62489
17505345	Pmfbp1	polyamine modulated factor 1 binding protein 1	4.035088	3.68739	4.20927	4.17892	5.231991	4.091812
17484484	Paox	polyamine oxidase (exo-N4-amino)	36.22164	33.49445	35.36107	34.60911	46.57003	52.73687
17406745	Pmf1	polyamine-modulated factor 1	25.35028	21.82213	30.91125	22.94281	26.87253	28.14936
17298324	Pbrm1	polybromo 1	82.41757	79.97848	79.33744	80.57665	89.63623	82.52332
17459918	Pcgf1	polycomb group ring finger 1	12.85457	15.67474	20.92042	13.38041	18.13796	13.80176
17268581	Pcgf2	polycomb group ring finger 2	8.640351	10.20469	9.27894	8.204583	7.445816	8.640351
17440257	Pcgf3	polycomb group ring finger 3	44.00819	43.18954	44.81936	42.07963	42.15348	43.94417
17358891	Pcgf5	polycomb group ring finger 5	33.90627	35.75145	30.0441	34.1916	37.19637	34.28487
17365444	Pcgf6	polycomb group ring finger 6	18.90754	17.7557	16.60735	18.19601	19.06613	12.74615
17221553	Pkhd1	polycystic kidney and hepatic disease 1	10.60785	14.31236	13.10895	11.81903	11.5015	13.01172
17311350	Pkhd1l1	polycystic kidney and hepatic disease 1-like 1	2.738584	3.223236	3.154541	3.019794	2.825919	2.899492
17320100	Pkdrej	polycystic kidney disease (polycystin) and REJ (sperm receptor for egg jelly homol	6.80757	8.224854	7.198468	6.80757	6.170212	6.702596
17334304	Pkd1	polycystic kidney disease 1 homolog	26.15618	25.13891	28.29451	28.29451	25.1342	28.29451
17260524	Pkd1l1	polycystic kidney disease 1 like 1	5.051809	5.362702	5.125313	4.913905	6.284422	4.544905
17513334	Pkd1l2	polycystic kidney disease 1 like 2	8.540438	7.100251	6.739773	6.114057	7.418667	7.858261
17505375	Pkd1l3	polycystic kidney disease 1 like 3	4.01461	4.377691	4.377691	5.659168	4.537413	4.835114
17439842	Pkd2	polycystic kidney disease 2	44.09827	41.10893	42.72563	36.85632	42.4982	41.9669
17365080	Pkd2l1	polycystic kidney disease 2-like 1	4.20752	5.676218	4.006104	4.801649	4.719716	4.670704
17349402	Pkd2l2	polycystic kidney disease 2-like 2	4.062478	4.02785	3.912227	4.353211	3.676722	3.593277
17539914	Pqbp1	polyglutamine binding protein 1	63.97428	62.73549	66.46348	54.56183	60.42987	57.71762
17470540	Phc1	polyhomeotic-like 1 (Drosophila)	5.523145	6.407093	8.606759	5.829693	5.406182	6.981272
17418857	Phc2	polyhomeotic-like 2 (Drosophila)	23.16621	24.35473	25.13926	23.96588	24.14797	23.7374
17404548	Phc3	polyhomeotic-like 3 (Drosophila)	28.61421	34.98246	39.25379	32.77764	40.3417	31.56382
17543150	Pola1	polymerase (DNA directed), alpha 1	11.74154	10.91681	10.98985	9.35521	11.04981	13.04107
17361855	Pola2	polymerase (DNA directed), alpha 2	17.60215	15.57284	18.05379	16.64365	17.2576	14.48502
17508063	Polb	polymerase (DNA directed), beta	24.65674	24.18499	24.27198	26.11524	21.384	24.39325
17490282	Pold1	polymerase (DNA directed), delta 1, catalytic subunit	10.6658	10.61873	10.32285	11.51714	9.734723	9.02965
17260221	Pold2	polymerase (DNA directed), delta 2, regulatory subunit	21.64722	18.25146	19.29828	14.63033	15.63156	18.19122
17440465	Pole	polymerase (DNA directed), epsilon	4.022019	4.508977	4.326949	4.508977	4.508977	5.187754
17281506	Pole2	polymerase (DNA directed), epsilon 2 (p59 subunit)	2.776524	3.542039	2.996607	3.135162	2.932457	3.258663
17426222	Pole3	polymerase (DNA directed), epsilon 3 (p17 subunit)	45.35595	46.95543	37.98874	48.16122	57.16658	48.16122

17345321	Polh	polymerase (DNA directed), eta (RAD 30 related)	20.99887	15.37101	14.31971	20.27163	12.34847	13.84507
17492340	Polg	polymerase (DNA directed), gamma	28.47788	29.19056	31.6745	33.62658	26.62261	31.87956
17270962	Polg2	polymerase (DNA directed), gamma 2, accessory subunit	16.97665	17.36199	17.11987	19.39967	16.70942	20.73103
17355316	Poli	polymerase (DNA directed), iota	10.16212	10.31113	11.23719	9.870889	6.791828	12.81152
17295212	Polk	polymerase (DNA directed), kappa	11.62888	13.80951	11.44472	16.12783	14.14088	15.90094
17365200	Poll	polymerase (DNA directed), lambda	7.631888	7.994457	8.748344	7.631888	6.421276	7.440347
17260198	Polm	polymerase (DNA directed), mu	10.66772	8.530794	9.488237	10.3366	9.06326	9.729732
17325454	Polq	polymerase (DNA directed), theta	4.399257	4.165357	4.684808	4.264983	3.59522	4.186951
17493739	Pold3	polymerase (DNA-directed), delta 3, accessory subunit	20.68166	22.64185	29.98772	21.29274	20.45455	23.87618
17356202	Pold4	polymerase (DNA-directed), delta 4	32.3461	33.86023	34.81821	34.80831	41.38913	37.05176
17253674	Poldip2	polymerase (DNA-directed), delta interacting protein 2	82.76851	81.84316	93.22791	93.22791	96.54636	97.36963
17319770	Poldip3	polymerase (DNA-directed), delta interacting protein 3	57.84258	60.96925	57.00118	53.78977	54.60496	59.91519
17468007	Pole4	polymerase (DNA-directed), epsilon 4 (p12 subunit)	16.21717	16.63154	18.49649	20.52784	18.49649	18.86487
17459550	Polr1a	polymerase (RNA) I polypeptide A	46.16124	44.85821	38.93889	44.85821	42.19821	42.55598
17376096	Polr1b	polymerase (RNA) I polypeptide B	17.04917	14.64209	13.65058	17.87176	18.15242	17.16526
17345335	Polr1c	polymerase (RNA) I polypeptide C	46.57068	34.60335	33.21125	33.7473	40.15451	35.05018
17444828	Polr1d	polymerase (RNA) I polypeptide D	31.5	34.99192	39.06027	35.15334	38.46408	36.96121
17413591	Polr1e	polymerase (RNA) I polypeptide E	9.034094	8.67606	9.870136	9.034094	9.639712	12.13044
17264906	Polr2a	polymerase (RNA) II (DNA directed) polypeptide A	81.40018	80.22166	87.76963	82.73638	99.49968	77.92585
17438496	Polr2b	polymerase (RNA) II (DNA directed) polypeptide B	155.7135	138.2437	143.3889	144.6929	135.3011	124.9849
17504160	Polr2c	polymerase (RNA) II (DNA directed) polypeptide C	54.85762	55.72026	45.91138	53.16113	40.91166	51.24147
17349174	Polr2d	polymerase (RNA) II (DNA directed) polypeptide D	10.17269	7.536371	8.548552	7.610441	7.377848	10.82991
17242773	Polr2e	polymerase (RNA) II (DNA directed) polypeptide E	33.13858	43.51527	44.413	50.9454	45.89452	46.29999
17312939	Polr2f	polymerase (RNA) II (DNA directed) polypeptide F	42.0805	38.98724	42.98019	41.18166	42.80389	37.00786
17362494	Polr2g	polymerase (RNA) II (DNA directed) polypeptide G	31.00911	39.08519	39.2968	31.92091	40.35568	42.92095
17324125	Polr2h	polymerase (RNA) II (DNA directed) polypeptide H	8.935571	10.99191	10.03925	17.20529	7.473272	16.26828
17476297	Polr2i	polymerase (RNA) II (DNA directed) polypeptide I	31.45244	30.33581	28.41695	29.15861	30.25329	30.33581
17443430	Polr2j	polymerase (RNA) II (DNA directed) polypeptide J	46.27978	50.779	48.99423	47.47809	40.98432	48.69967
17311064	Polr2k	polymerase (RNA) II (DNA directed) polypeptide K	5.532724	5.051634	4.500483	4.143213	4.736692	5.289532
17497953	Polr2l	polymerase (RNA) II (DNA directed) polypeptide L	8.631073	7.116017	6.658605	7.501609	7.875437	9.562713
17528663	Polr2m	polymerase (RNA) II (DNA directed) polypeptide M	128.0985	121.1928	121.403	127.0295	132.2762	131.3452
17304089	Polr3a	polymerase (RNA) III (DNA directed) polypeptide A	31.5249	36.08757	24.93949	29.45989	29.70554	37.6018
17236018	Polr3b	polymerase (RNA) III (DNA directed) polypeptide B	54.01919	49.74432	48.79732	52.69704	56.248	49.09792
17408099	Polr3c	polymerase (RNA) III (DNA directed) polypeptide C	36.7818	31.96808	35.13897	38.71981	35.1475	36.76956
17308349	Polr3d	polymerase (RNA) III (DNA directed) polypeptide D	17.33637	16.48086	15.52617	16.70305	17.33637	19.32679
17482590	Polr3e	polymerase (RNA) III (DNA directed) polypeptide E	19.00939	17.53992	18.07783	18.35397	17.41779	19.05094
17377071	Polr3f	polymerase (RNA) III (DNA directed) polypeptide F	9.87965	11.17831	8.559936	11.50732	11.93825	11.98925
17294664	Polr3g	polymerase (RNA) III (DNA directed) polypeptide G	18.4265	18.29478	22.2538	13.40714	18.38325	16.69183
17408074	Polr3gl	polymerase (RNA) III (DNA directed) polypeptide G like	49.30254	40.99369	53.67766	58.17873	53.63824	60.8696
17319545	Polr3h	polymerase (RNA) III (DNA directed) polypeptide H	19.20753	22.67955	23.37396	25.31218	28.92089	22.6395
17381184	Polr3k	polymerase (RNA) III (DNA directed) polypeptide K	46.66439	42.91678	40.08471	40.60766	40.24841	43.5977
17242653	Polrmt	polymerase (RNA) mitochondrial (DNA directed)	34.0438	35.8455	35.8614	30.22771	31.03061	38.0388
17269748	Ptrf	polymerase I and transcript release factor	11.24366	14.31296	19.95641	14.31296	13.32617	11.36511
17216960	Pigr	polymeric immunoglobulin receptor	1670.345	1517.478	1501.533	1587.84	1861.616	1657.404
17241157	Pldi	polymorphic derived intron containing	6.043231	6.785194	6.377054	6.771474	7.515765	8.61398
17477597	Pnkp	polynucleotide kinase 3- phosphatase	39.30361	32.20784	33.97637	35.90916	32.19646	35.37602
17234980	Ptbp1	polypyrimidine tract binding protein 1	100.6958	122.631	131.7884	100.8138	113.4058	103.1664
17409839	Ptbp2	polypyrimidine tract binding protein 2	26.18158	38.36982	27.56107	24.94277	25.0292	24.6841
17425836	Ptbp3	polypyrimidine tract binding protein 3	137.1328	202.1889	182.6135	167.5305	183.9232	177.1896
17247962	Pnpt1	polyribonucleotide nucleotidyltransferase 1	48.8139	50.94258	45.184	52.99925	44.40099	51.1641
17537019	Pbdc1	polysaccharide biosynthesis domain containing 1	36.86322	40.87254	36.30459	37.68687	44.13343	32.49194
17318299	Puf60	poly-U binding splicing factor 60	90.00854	83.0196	92.9801	83.36789	88.82476	75.89222
17247371	Pom121l12	POM121 membrane glycoprotein-like 12	4.094678	4.110774	3.80095	4.347241	4.092153	5.514385
17285725	Pom121l2	POM121 membrane glycoprotein-like 2 (rat)	7.380047	7.379484	7.843241	8.182434	5.931924	8.063354
17325592	Popdc2	popeye domain containing 2	6.35759	7.837372	8.172628	7.390336	7.393564	12.67532
17233095	Popdc3	popeye domain containing 3	3.746387	3.084913	2.931177	3.518279	2.697738	3.204257
17540059	Porcn	porcupine homolog (Drosophila)	11.37252	12.98847	12.47044	13.39423	12.47044	12.47044
17357805	Pfpl	pore forming protein-like	5.542205	4.20927	5.263892	4.133059	5.229888	5.367834
17223138	Pgap1	post-GPI attachment to proteins 1	51.11625	49.4569	39.53614	43.30975	40.18263	35.82587
17481066	Pgap2	post-GPI attachment to proteins 2	20.45313	23.32492	27.79667	29.02311	26.90719	26.71429
17268794	Pgap3	post-GPI attachment to proteins 3	15.06679	15.15603	15.98149	13.0897	10.34635	16.86511
17222968	Pms1	postmeiotic segregation increased 1 (S. cerevisiae)	10.43009	12.16446	11.01655	10.68778	10.91719	11.53079
17444416	Pms2	postmeiotic segregation increased 2 (S. cerevisiae)	5.014862	5.025614	5.702561	4.362696	4.729803	4.232294
17467823	Kcmf1	potassium channel modulatory factor 1	279.3519	288.4065	266.5902	251.5777	242.8699	267.323
17301123	Kcnrg	potassium channel regulator	2.921846	2.966887	3.025492	3.182587	2.749177	3.048374
17352850	Kctd1	potassium channel tetramerisation domain containing 1	7.781671	5.718434	7.866544	5.350783	6.716595	6.015272
17451526	Kctd10	potassium channel tetramerisation domain containing 10	17.81191	19.99739	22.62397	15.21055	19.3873	17.04268
17265026	Kctd11	potassium channel tetramerisation domain containing 11	10.16607	11.02319	13.40296	12.42281	14.36474	13.53038
17309154	Kctd12	potassium channel tetramerisation domain containing 12	27.64679	35.73733	34.45688	23.57278	24.24155	29.45356
17545488	Kctd12b	potassium channel tetramerisation domain containing 12b	4.714223	5.444955	6.051446	5.519815	5.444955	4.419368
17483180	Kctd13	potassium channel tetramerisation domain containing 13	12.25442	21.24792	14.48773	16.23595	16.23595	14.23342
17483190	Kctd13	potassium channel tetramerisation domain containing 13	6.175432	6.111684	5.222679	5.932129	5.948682	5.414432
17483192	Kctd13	potassium channel tetramerisation domain containing 13	14.20355	10.17023	10.52808	11.16468	14.37311	16.01254
17480349	Kctd14	potassium channel tetramerisation domain containing 14	4.278277	4.702735	3.339703	4.381819	4.278277	4.139309
17489682	Kctd15	potassium channel tetramerisation domain containing 15	11.9385	18.4426	17.23919	21.62113	14.62404	21.31933
17350081	Kctd16	potassium channel tetramerisation domain containing 16	4.193224	3.719194	3.817478	5.056047	3.489886	3.885984
17312746	Kctd17	potassium channel tetramerisation domain containing 17	25.92047	32.69436	41.77709	32.69436	28.20345	22.93994
17223332	Kctd18	potassium channel tetramerisation domain containing 18	14.24763	12.20457	16.38859	13.86543	12.32751	14.59639
17512415	Kctd19	potassium channel tetramerisation domain containing 19	5.417886	7.326174	6.124534	7.158166	7.234634	6.379264
17258197	Kctd2	potassium channel tetramerisation domain containing 2	15.64728	15.29735	16.33404	14.84999	15.17201	14.43431
17335441	Kctd20	potassium channel tetramerisation domain containing 20	52.29786	56.40808	52.29786	56.53765	41.92503	50.45971
17480324	Kctd21	potassium channel tetramerisation domain containing 21	15.96577	15.05353	18.77199	17.23477	11.18474	11.40188
17230879	Kctd3	potassium channel tetramerisation domain containing 3	25.56114	27.24312	25.56114	28.93934	21.7765	25.59624
17302056	Kctd4	potassium channel tetramerisation domain containing 4	3.952649	3.258843	4.288223	3.425299	3.355901	3.37379

17341681	Kctd5	potassium channel tetramerisation domain containing 5	13.44847	15.43913	13.42937	13.74905	15.8231	14.01121
17297100	Kctd6	potassium channel tetramerisation domain containing 6	18.80898	21.61484	17.79463	21.05513	22.65331	26.22517
17442995	Kctd7	potassium channel tetramerisation domain containing 7	9.932384	10.53019	10.82376	9.441002	9.713222	10.47
17448526	Kctd8	potassium channel tetramerisation domain containing 8	3.424183	4.222917	3.80319	4.230564	4.455879	3.820682
17301558	Kctd9	potassium channel tetramerisation domain containing 9	37.90142	39.06	34.0811	35.065	32.04017	33.78568
17506917	Kcnk1	potassium channel, subfamily K, member 1	5.338965	7.098676	5.464691	7.1874	6.065977	6.42653
17283117	Kcnk10	potassium channel, subfamily K, member 10	4.862843	6.677694	5.643499	6.949022	8.315656	5.557631
17347948	Kcnk12	potassium channel, subfamily K, member 12	3.927061	4.432744	3.310387	4.735182	4.592055	4.219582
17277900	Kcnk13	potassium channel, subfamily K, member 13	5.581644	8.995541	9.076462	8.674857	6.550023	8.121409
17379323	Kcnk15	potassium channel, subfamily K, member 15	14.27689	20.54236	13.88139	14.93401	14.22947	16.49309
17303732	Kcnk16	potassium channel, subfamily K, member 16	9.147389	6.504238	6.765422	7.178687	8.365664	8.249004
17360817	Kcnk18	potassium channel, subfamily K, member 18	4.268739	4.760391	5.069664	3.905778	4.873269	6.837789
17230902	Kcnk2	potassium channel, subfamily K, member 2	6.51457	12.31684	6.594805	10.47973	6.574859	7.118089
17435811	Kcnk3	potassium channel, subfamily K, member 3	23.23375	22.00135	20.98054	22.04363	21.31224	26.66989
17362128	Kcnk4	potassium channel, subfamily K, member 4	18.36007	14.49929	13.67539	14.93712	14.75212	12.28529
17303722	Kcnk5	potassium channel, subfamily K, member 5	51.42792	51.42792	31.84533	51.42792	42.73333	47.80601
17356517	Kcnk7	potassium channel, subfamily K, member 7	5.9707	7.364308	6.00569	6.606609	7.4437	8.8456
17317801	Kcnk9	potassium channel, subfamily K, member 9	5.850541	6.154237	5.303115	5.860525	4.349622	5.876308
17368212	Kcnt1	potassium channel, subfamily T, member 1	7.127914	7.183167	7.337456	6.666687	5.176475	7.794951
17217969	Kcnt2	potassium channel, subfamily T, member 2	6.867	7.433646	8.582988	5.373194	7.935109	5.921899
17500209	Kcnu1	potassium channel, subfamily U, member 1	3.023941	2.802919	2.583828	2.677766	2.701087	3.049431
17316859	Kcnv1	potassium channel, subfamily V, member 1	7.006675	7.028223	7.006675	6.96962	6.241834	7.04339
17358448	Kcnv2	potassium channel, subfamily V, member 2	2.72259	3.54506	3.753353	3.482709	3.855345	3.564328
17510212	Kcnn1	potassium intermediate/small conductance calcium-activated channel, subfamily N	12.64417	18.49649	18.36211	19.42873	16.28204	20.94465
17350332	Kcnn2	potassium intermediate/small conductance calcium-activated channel, subfamily N	25.21657	23.76868	26.36112	29.22781	23.4756	42.10734
17399485	Kcnn3	potassium intermediate/small conductance calcium-activated channel, subfamily N	4.886969	4.689974	4.766003	3.721953	5.75733	7.707644
17474941	Kcnn4	potassium intermediate/small conductance calcium-activated channel, subfamily N	11.0954	12.43634	11.42839	11.71707	12.99775	12.06278
17491068	Kcnj11	potassium inwardly rectifying channel, subfamily J, member 11	9.11631	9.29426	8.86979	9.162936	9.513129	9.731013
17515813	Kcnj1	potassium inwardly-rectifying channel, subfamily J, member 1	4.457856	4.083256	3.480127	4.20927	4.36061	3.651983
17219536	Kcnj10	potassium inwardly-rectifying channel, subfamily J, member 10	16.04264	17.78464	12.81916	16.26174	26.29105	12.95161
17250549	Kcnj12	potassium inwardly-rectifying channel, subfamily J, member 12	3.991746	3.861925	3.527094	3.861925	3.729055	3.879498
17225253	Kcnj13	potassium inwardly-rectifying channel, subfamily J, member 13	3.582409	3.54506	3.771687	3.499721	3.917681	3.295506
17490978	Kcnj14	potassium inwardly-rectifying channel, subfamily J, member 14	17.21805	12.50367	12.46413	13.05997	16.52399	14.85143
17327450	Kcnj15	potassium inwardly-rectifying channel, subfamily J, member 15	5.882625	5.772075	5.469428	7.167401	5.882625	5.780323
17257928	Kcnj16	potassium inwardly-rectifying channel, subfamily J, member 16	6.721671	5.755176	7.723903	6.097974	8.112013	6.81744
17257937	Kcnj2	potassium inwardly-rectifying channel, subfamily J, member 2	4.690559	4.352065	4.399623	4.352065	4.352065	4.411166
17370883	Kcnj3	potassium inwardly-rectifying channel, subfamily J, member 3	4.702604	6.484791	5.586327	5.906486	5.009269	5.920504
17319226	Kcnj4	potassium inwardly-rectifying channel, subfamily J, member 4	6.766501	6.405273	6.465626	6.647906	5.741191	7.387161
17525304	Kcnj5	potassium inwardly-rectifying channel, subfamily J, member 5	5.991968	5.127871	5.261302	5.338965	5.696872	6.752726
17332346	Kcnj6	potassium inwardly-rectifying channel, subfamily J, member 6	4.579754	4.471503	4.901591	4.268414	6.175646	5.105032
17472530	Kcnj8	potassium inwardly-rectifying channel, subfamily J, member 8	21.30219	26.01749	19.79028	20.44378	18.37344	23.49334
17229917	Kcnj9	potassium inwardly-rectifying channel, subfamily J, member 9	10.62868	9.799037	9.531677	12.58471	9.458491	8.651738
17488792	Kcnk6	potassium inwardly-rectifying channel, subfamily K, member 6	30.56122	30.56122	42.33973	30.56122	30.26923	31.4337
17304010	Kcnma1	potassium large conductance calcium-activated channel, subfamily M, alpha meml	4.732787	3.716591	4.334663	4.809577	4.075367	4.24634
17304012	Kcnma1	potassium large conductance calcium-activated channel, subfamily M, alpha meml	4.389024	4.20927	4.20927	4.584327	4.20927	4.20927
17248374	Kcnmb1	potassium large conductance calcium-activated channel, subfamily M, beta memb	4.650384	5.132956	5.752863	5.559596	7.215153	5.912179
17396662	Kcnmb2	potassium large conductance calcium-activated channel, subfamily M, beta memb	5.689295	4.473302	4.352065	4.038249	4.352065	4.239899
17404596	Kcnmb3	potassium large conductance calcium-activated channel, subfamily M, beta memb	6.331775	7.201762	5.762321	6.156072	6.656482	9.080993
17245085	Kcnmb4	potassium large conductance calcium-activated channel, subfamily M, beta memb	6.939588	7.23674	7.726848	8.450988	6.105672	7.576072
17237492	Kcnmb4os1	potassium large conductance calcium-activated channel, subfamily M, beta memb	9.45718	8.741192	9.45954	9.338689	8.409325	11.61196
17237496	Kcnmb4os2	potassium large conductance calcium-activated channel, subfamily M, beta memb	4.609946	5.009651	4.647678	4.647678	4.961203	4.110489
17394668	Kcnb1	potassium voltage gated channel, Shab-related subfamily, member 1	10.50235	12.34564	9.42433	11.75514	9.86316	10.27688
17211235	Kcnb2	potassium voltage gated channel, Shab-related subfamily, member 2	7.000655	5.848716	5.869022	6.345791	5.25875	7.424737
17478181	Kcnc1	potassium voltage gated channel, Shaw-related subfamily, member 1	5.414207	5.779321	7.019411	5.587644	6.460877	5.675116
17237336	Kcnc2	potassium voltage gated channel, Shaw-related subfamily, member 2	3.004969	3.387122	3.645761	3.618571	3.644447	3.071408
17477520	Kcnc3	potassium voltage gated channel, Shaw-related subfamily, member 3	26.98321	38.83243	37.33431	30.51761	36.44427	32.02774
17408999	Kcnc4	potassium voltage gated channel, Shaw-related subfamily, member 4	6.982095	8.813103	7.023986	7.550436	7.688915	6.842436
17279950	Kcns3	potassium voltage-gated channel, delayed-rectifier, subfamily S, member 3	12.32991	15.82485	14.80395	13.96719	15.43582	13.65077
17545045	Kcne1l	potassium voltage-gated channel, Isk-related family, member 1-like, pseudogene	6.463878	9.507328	9.224815	9.016016	12.46275	11.58078
17327218	Kcne2	potassium voltage-gated channel, Isk-related subfamily, gene 2	6.030928	4.941058	5.989311	6.863479	6.030928	5.766117
17480641	Kcne3	potassium voltage-gated channel, Isk-related subfamily, gene 3	8.832611	12.68481	12.4044	10.50592	10.50592	10.52151
17214711	Kcne4	potassium voltage-gated channel, Isk-related subfamily, gene 4	5.041804	7.185159	4.435787	5.041804	5.041804	5.432778
17332223	Kcne1	potassium voltage-gated channel, Isk-related subfamily, member 1	3.741145	5.031854	5.409443	3.703322	3.832617	4.889646
17398052	Kcnab1	potassium voltage-gated channel, shaker-related subfamily, beta member 1	3.90995	4.912952	5.705091	4.801649	4.565459	4.886969
17433493	Kcnab2	potassium voltage-gated channel, shaker-related subfamily, beta member 2	4.700649	5.436695	5.649135	4.801649	5.476218	5.747322
17251617	Kcnab3	potassium voltage-gated channel, shaker-related subfamily, beta member 3	4.294678	4.027376	4.349351	4.20927	4.294678	4.098966
17264585	Kcnab3os	potassium voltage-gated channel, shaker-related subfamily, beta member 3, oppo	6.21244	6.583706	5.335339	5.892582	6.598051	5.387254
17471155	Kcna1	potassium voltage-gated channel, shaker-related subfamily, member 1	4.626721	3.717751	4.20927	3.947283	4.300427	4.20927
17401545	Kcna10	potassium voltage-gated channel, shaker-related subfamily, member 10	4.386712	4.761777	5.918288	4.684516	4.140285	5.788462
17401535	Kcna2	potassium voltage-gated channel, shaker-related subfamily, member 2	2.884577	3.693248	3.647455	3.605327	3.14777	3.24098
17401539	Kcna2	potassium voltage-gated channel, shaker-related subfamily, member 2	6.759048	8.482444	9.780376	7.548186	7.867798	7.267605
17401526	Kcna3	potassium voltage-gated channel, shaker-related subfamily, member 3	6.461862	4.875364	5.219241	5.618987	5.299955	6.349223
17374047	Kcna4	potassium voltage-gated channel, shaker-related subfamily, member 4	3.272379	3.432667	2.884058	3.277351	3.999667	3.349905
17471151	Kcna5	potassium voltage-gated channel, shaker-related subfamily, member 5	6.46455	3.976888	4.262619	4.792007	5.271122	6.192921
17477791	Kcna7	potassium voltage-gated channel, shaker-related subfamily, member 7	4.01461	5.190056	4.653641	4.254619	4.144534	3.043771
17471160	Kcna6	potassium voltage-gated channel, shaker-related, subfamily, member 6	4.836522	5.562872	7.862946	7.751685	6.19932	5.618442
17533004	Kcnd1	potassium voltage-gated channel, Shal-related family, member 1	4.36669	5.367983	4.860922	4.189767	4.847799	4.798588
17456308	Kcnd2	potassium voltage-gated channel, Shal-related family, member 2	5.760444	4.927753	4.946747	4.745829	4.905499	4.980153
17401394	Kcnd3	potassium voltage-gated channel, Shal-related family, member 3	4.176632	6.337395	5.758248	8.423676	6.088007	7.096954
17408821	Kcnd3os	potassium voltage-gated channel, Shal-related family, member 3, opposite strand	4.681043	5.119703	4.1369	4.435418	4.801649	4.520806
17280144	Kcnf1	potassium voltage-gated channel, subfamily F, member 1	4.465649	4.602443	5.384566	4.893172	4.124502	4.804046
17394795	Kcng1	potassium voltage-gated channel, subfamily G, member 1	14.69751	13.86562	11.56746	10.40165	10.3953	11.56746
17355641	Kcng2	potassium voltage-gated channel, subfamily G, member 2	9.703129	10.87023	8.647901	10.62728	9.703129	12.12716
17347645	Kcng3	potassium voltage-gated channel, subfamily G, member 3	11.94314	11.76868	11.56401	10.72421	8.530676	10.29855

17513472	Kcng4	potassium voltage-gated channel, subfamily G, member 4	5.333269	7.218855	7.688062	6.368617	6.171709	6.484836
17220882	Kcnh1	potassium voltage-gated channel, subfamily H (eag-related), member 1	6.936702	4.183296	7.003034	5.502091	6.192577	5.833857
17446123	Kcnh2	potassium voltage-gated channel, subfamily H (eag-related), member 2	10.75533	10.55131	11.52456	12.05617	8.853758	11.81707
17314755	Kcnh3	potassium voltage-gated channel, subfamily H (eag-related), member 3	4.977944	4.801649	5.120306	4.801649	4.727574	6.445222
17269652	Kcnh4	potassium voltage-gated channel, subfamily H (eag-related), member 4	6.32923	5.931307	8.100045	5.366904	6.768143	8.582988
17281927	Kcnh5	potassium voltage-gated channel, subfamily H (eag-related), member 5	3.776344	5.11463	4.330009	3.974932	3.9434	4.422524
17257473	Kcnh6	potassium voltage-gated channel, subfamily H (eag-related), member 6	4.424732	4.28968	5.526247	5.670477	5.459353	4.944176
17385816	Kcnh7	potassium voltage-gated channel, subfamily H (eag-related), member 7	3.653603	4.688349	4.20927	4.166078	4.892019	4.852472
17338545	Kcnh8	potassium voltage-gated channel, subfamily H (eag-related), member 8	4.681043	5.176547	4.342783	4.71723	4.52849	4.970428
17485320	Kcnq1	potassium voltage-gated channel, subfamily Q, member 1	10.02833	12.97884	11.06637	7.546251	8.882895	12.01954
17395732	Kcnq2	potassium voltage-gated channel, subfamily Q, member 2	4.462339	4.085011	4.13994	4.593329	4.20927	4.164202
17317593	Kcnq3	potassium voltage-gated channel, subfamily Q, member 3	6.452687	5.332899	5.178449	6.211923	5.175506	4.953197
17429476	Kcnq4	potassium voltage-gated channel, subfamily Q, member 4	12.02846	14.19322	16.48475	13.9669	15.64685	16.04431
17221686	Kcnq5	potassium voltage-gated channel, subfamily Q, member 5	8.171665	8.491974	8.430335	8.491974	8.561894	8.491974
17500349	Poteg	POTE ankyrin domain family, member G	4.751786	4.20306	4.484944	5.672167	4.192963	4.653899
17288854	Pou5f2	POU domain class 5, transcription factor 2	9.015016	9.355469	10.79791	12.55082	15.70509	11.66747
17326534	Pou1f1	POU domain, class 1, transcription factor 1	3.037049	2.92375	2.64827	3.243249	2.880781	2.590535
17517209	Pou2af1	POU domain, class 2, associating factor 1	6.046708	7.567779	5.652073	7.414452	4.224967	6.394936
17229282	Pou2f1	POU domain, class 2, transcription factor 1	14.31971	27.66922	33.12343	24.90252	25.20451	28.64874
17487855	Pou2f2	POU domain, class 2, transcription factor 2	9.755393	11.03649	9.987752	9.755393	9.755393	11.24366
17526086	Pou2f3	POU domain, class 2, transcription factor 3	3.362397	4.189593	3.025052	2.666172	3.828157	3.903748
17418268	Pou3f1	POU domain, class 3, transcription factor 1	9.749917	10.80967	9.063825	10.25688	12.09627	10.47116
17423631	Pou3f2	POU domain, class 3, transcription factor 2	3.446758	4.253528	4.090253	3.749349	3.773779	3.976861
17212313	Pou3f3	POU domain, class 3, transcription factor 3	7.036705	6.162305	7.979841	6.328528	5.922926	7.403924
17222618	Pantr1	POU domain, class 3, transcription factor 3 adjacent noncoding transcript 1	8.339842	8.791992	9.480216	8.774519	7.268965	9.175266
17537209	Pou3f4	POU domain, class 3, transcription factor 4	3.817637	4.92789	4.416918	5.010449	5.080217	4.666044
17309287	Pou4f1	POU domain, class 4, transcription factor 1	5.608675	7.333948	6.203877	4.340135	5.66058	7.289198
17510696	Pou4f2	POU domain, class 4, transcription factor 2	5.300432	5.300432	5.300432	5.629513	5.487694	5.200823
17350134	Pou4f3	POU domain, class 4, transcription factor 3	15.22954	8.473747	9.310251	11.47395	9.310251	8.11882
17337155	Pou5f1	POU domain, class 5, transcription factor 1	9.35132	8.157573	7.523062	8.382729	6.704501	8.019321
17402556	Gm5712	POU domain, class 5, transcription factor 1 pseudogene	5.430745	4.625695	4.450786	5.453415	5.266557	5.483359
17321740	Pou6f1	POU domain, class 6, transcription factor 1	19.56184	17.79722	16.34797	15.65108	14.55285	17.30983
17290835	Pou6f2	POU domain, class 6, transcription factor 2	5.352378	4.918949	4.647678	6.63189	6.114099	5.2773
17246547	Patz1	POZ (BTB) and AT hook containing zinc finger 1	7.68396	8.581977	10.25667	8.61607	9.410556	8.354769
17422907	Perm1	PPARGC1 and ESRR induced regulator, muscle 1	3.279759	2.490422	1.954839	2.622404	2.700769	2.917535
17391781	Gm14057	Ppp1r14c pseudogene	4.639534	5.10574	4.734198	6.198031	3.95731	3.798527
17280125	Pqlc3	PQ loop repeat containing	6.984806	6.287864	6.338845	6.900591	7.698994	5.259151
17352146	Pqlc1	PQ loop repeat containing 1	52.64556	35.84276	35.65716	39.09386	41.74669	41.21507
17431880	Pqlc2	PQ loop repeat containing 2	25.33711	27.70031	23.87188	24.20797	23.36715	22.83999
17240645	Prdm1	PR domain containing 1, with ZNF domain	17.17705	17.91666	20.29525	20.51532	17.49232	17.49232
17515729	Prdm10	PR domain containing 10	13.51238	14.79738	18.64969	13.48618	11.56746	14.52564
17388417	Prdm11	PR domain containing 11	9.449317	5.715343	9.126995	6.763734	5.477699	7.531671
17369532	Prdm12	PR domain containing 12	13.49291	13.24302	13.01605	13.4453	13.01605	12.90242
17423612	Prdm13	PR domain containing 13	5.304071	5.51492	5.865117	5.539171	5.921079	7.974146
17221307	Prdm14	PR domain containing 14	5.518821	6.287864	6.217222	6.86681	7.227917	6.645925
17332595	Prdm15	PR domain containing 15	11.40362	15.42673	11.57115	11.56281	12.02563	10.18991
17433631	Prdm16	PR domain containing 16	6.993041	8.064024	7.671932	7.816323	5.355384	8.471985
17432426	Prdm2	PR domain containing 2, with ZNF domain	12.02463	11.30618	9.423611	13.58441	13.17645	15.19484
17243661	Prdm4	PR domain containing 4	29.69092	35.8013	28.83382	29.69092	19.62866	30.38084
17459213	Prdm5	PR domain containing 5	6.060513	6.64528	5.969251	6.133115	6.171709	6.048972
17350639	Prdm6	PR domain containing 6	8.82117	8.467112	8.358303	10.12163	8.467112	8.584833
17439511	Prdm8	PR domain containing 8	6.955046	5.4845	6.915914	6.769785	6.933482	5.024778
17341214	Prdm9	PR domain containing 9	9.164715	12.37966	12.53708	10.46028	17.94811	9.537294
17532947	Praf2	PRA1 domain family 2	13.10886	11.26043	18.58453	12.28248	8.398199	14.59326
17346679	Pja2	praja 2, RING-H2 motif containing	136.7531	97.87375	97.06841	94.35781	109.372	109.2417
17543451	Pja1	praja ring finger 1, E3 ubiquitin protein ligase	27.61308	26.71115	27.59089	27.59089	27.59089	27.53548
17432546	Pramef12	PRAME family member 12	2.893469	2.794965	3.587155	2.495727	4.767094	2.98742
17421297	Pramef12os	PRAME family member 12, opposite strand [Source:MGI Symbol;Acc:MGI:1925973]	3.176923	3.131557	2.953407	2.658182	3.801371	3.122756
17432509	Pramef17	PRAME family member 17	3.099617	3.150411	2.592744	3.374632	2.419535	2.953407
17432496	Pramef25	PRAME family member 25	2.494758	2.253254	2.320941	3.254554	2.175987	2.319237
17455713	LOC620551	PRAME family member 5-like	2.213716	2.08392	2.199674	1.898013	3.471791	2.2751
17432488	Pramef6	PRAME family member 6	4.228981	4.251229	5.059378	4.445637	4.109207	4.601933
17421187	Pramef8	PRAME family member 8	20.58098	18.95846	17.74376	17.53104	17.11192	23.53164
17455726	LOC100039010	PRAME family member 8-like	1.763278	2.420844	2.589727	2.577924	2.033155	2.185722
17455668	LOC100862202	PRAME family member 8-like	4.01784	5.752743	4.144735	4.144735	4.493127	4.574836
17455740	LOC101055951	PRAME family member 8-like	3.414395	2.420844	2.879843	2.605572	2.710483	3.40872
17449816	LOC102634314	PRAME family member 8-like	2.423043	2.979541	4.126046	2.248402	3.072153	2.567564
17455639	LOC624931	PRAME family member 8-like	3.268797	2.308683	2.122861	2.268565	2.782767	2.857236
17455627	LOC665755	PRAME family member 8-like	3.54506	3.387662	4.647678	4.04273	3.793606	3.54506
17439207	LOC102641711	PRAME family member 9/15-like	3.432263	2.820166	3.977191	2.606909	4.033661	3.661564
17229417	Pbx1	pre B cell leukemia homeobox 1	28.13746	30.71816	33.59815	27.75066	30.43902	27.96092
17336602	Pbx2	pre B cell leukemia homeobox 2	8.820375	11.33891	9.958511	8.658817	8.050286	8.658817
17384234	Pbx3	pre B cell leukemia homeobox 3	14.23746	12.58986	13.48282	10.43696	11.3585	12.68103
17501755	Pbx4	pre B cell leukemia homeobox 4	7.43241	6.691409	7.179284	7.117596	5.847257	8.767892
17399460	Pbxip1	pre B cell leukemia transcription factor interacting protein 1	17.44196	21.09282	21.1615	16.41315	20.78585	16.87412
17345581	Ptcra	pre T cell antigen receptor alpha	9.123011	7.543165	8.813042	8.89898	9.599305	9.123011
17328573	Vpreb1	pre-B lymphocyte gene 1	3.692736	4.318888	4.192759	3.389118	4.161086	3.58159
17323627	Vpreb2	pre-B lymphocyte gene 2	9.635169	12.55291	11.63944	8.477095	12.0398	10.3858
17234441	Vpreb3	pre-B lymphocyte gene 3	4.769341	4.20927	4.20927	4.10841	3.618069	4.199129
17278335	Gm10000	predicted gene 10000 [Source:MGI Symbol;Acc:MGI:3641968]	3.619599	2.806399	4.114537	3.45154	3.888086	3.212998
17360731	Gm10007	predicted gene 10007	38.64983	27.85125	39.2625	41.47327	27.05419	32.00177
17471274	Gm10010	predicted gene 10010 [Source:MGI Symbol;Acc:MGI:3641978]	5.94526	4.507195	5.099055	6.574905	4.684256	4.584581
17498080	Gm10013	predicted gene 10013	27.85994	30.60722	39.21599	32.93158	19.68166	28.0722
17234709	Gm10024	predicted gene 10024	4.741949	4.260492	4.498862	6.789946	4.260492	5.271999

17290353	Gm10029	predicted gene 10029 [Source:MGI Symbol;Acc:MGI:3641706]	18.2783	19.88336	18.29021	18.80872	20.23415	22.65268
17522550	Gm10030	predicted gene 10030	9.711741	7.539976	8.091292	6.895475	9.388274	9.924194
17301514	Gm10032	predicted gene 10032 [Source:MGI Symbol;Acc:MGI:3642901]	9.277525	8.342154	9.708578	12.35189	9.963206	8.189356
17509809	Gm10033	predicted gene 10033	13.16358	13.03149	13.50545	12.42531	15.82946	12.21024
17348674	Gm10036	predicted gene 10036 [Source:MGI Symbol;Acc:MGI:3642334]	594.2845	560.4221	608.642	593.1117	617.8231	629.7607
17288350	Gm10037	predicted gene 10037	3.510168	2.343265	2.504322	1.808633	2.187086	3.260493
17303373	Gm10044	predicted gene 10044	8.545767	10.54794	9.743634	9.871231	8.029054	10.38752
17475695	Gm10046	predicted gene 10046	5.332566	6.011479	3.917736	6.849506	6.426486	4.890765
17358271	Gm10053	predicted gene 10053 [Source:MGI Symbol;Acc:MGI:3704493]	259.2446	295.6302	208.9784	265.2318	196.0177	277.2147
17548102	Gm10053	predicted gene 10053 [Source:MGI Symbol;Acc:MGI:3704493]	157.9769	144.2293	125.8263	128.5014	117.2651	152.8847
17545508	Gm10057	predicted gene 10057	2.213378	2.162035	1.95751	2.162035	2.128696	2.121346
17446420	Gm10062	predicted gene 10062 [Source:MGI Symbol;Acc:MGI:3641967]	2.790493	4.147667	2.552198	2.635725	3.508441	3.358228
17452453	Gm10064	predicted gene 10064 [Source:MGI Symbol;Acc:MGI:3645905]	6.687468	9.909484	9.645861	9.310251	7.605009	8.27902
17290961	Gm10065	predicted gene 10065 [Source:MGI Symbol;Acc:MGI:3642433]	5.338965	3.726264	5.009165	3.154082	3.127435	3.753353
17463434	Gm10069	predicted gene 10069	20.08658	20.85585	19.35199	17.22963	20.49372	17.84787
17526261	Gm10080	predicted gene 10080	21.86361	29.46969	32.14428	28.87002	27.53567	32.41815
17494729	Gm10081	predicted gene 10081	2.52223	3.250519	2.347231	3.142799	5.557014	4.535967
17323816	Gm10088	predicted gene 10088 [Source:MGI Symbol;Acc:MGI:3641695]	2.028256	2.688497	2.216541	2.094637	1.938392	2.477148
17325321	Gm10092	predicted gene 10092 [Source:MGI Symbol;Acc:MGI:3642362]	2.59296	2.400159	2.21048	2.148485	2.203092	2.07612
17504367	Gm10094	predicted gene 10094	452.4893	510.0306	492.2543	446.1732	528.6393	532.0065
17231473	Gm10097	predicted gene 10097 [Source:MGI Symbol;Acc:MGI:3642295]	9.118459	10.48228	12.50558	10.93196	11.63202	13.92929
17234716	Gm10100	predicted gene 10100	3.916568	9.508778	4.788786	5.399291	3.89626	5.365825
17305738	Gm10101	predicted gene 10101 [Source:MGI Symbol;Acc:MGI:3641713]	4.15155	4.292952	4.293904	3.620402	4.232382	3.256788
17336062	Gm10103	predicted gene 10103	2.961709	2.288592	2.722043	2.823726	2.782767	3.12659
17499732	Gm10104	predicted gene 10104	4.139969	5.511099	4.750798	4.402369	5.120306	5.120306
17490180	Gm10109	predicted gene 10109 [Source:MGI Symbol;Acc:MGI:3647742]	7.923677	9.79829	6.219204	9.889242	8.157347	7.641893
17309006	Gm10110	predicted gene 10110 [Source:MGI Symbol;Acc:MGI:3641718]	54.95962	48.04452	50.62998	59.45722	55.17642	48.47907
17292185	Gm10113	predicted gene 10113 [Source:MGI Symbol;Acc:MGI:3642714]	3.483941	5.126876	3.040461	4.199886	4.278989	3.984006
17381515	Gm10115	predicted gene 10115 [Source:MGI Symbol;Acc:MGI:3641675]	2.708286	2.892606	3.009972	2.805504	2.680515	3.009972
17241600	Gm10118	predicted gene 10118 [Source:MGI Symbol;Acc:MGI:3642340]	3.574794	2.915655	3.339703	2.953407	2.832763	2.953407
17352427	Gm10125	predicted gene 10125	6.543126	7.035095	5.790468	6.6533	8.641789	5.236199
17294318	Gm10126	predicted gene 10126 [Source:MGI Symbol;Acc:MGI:3809072]	24.95972	25.61612	30.27049	26.24626	31.05406	29.34169
17377459	Gm10130	predicted gene 10130 [Source:MGI Symbol;Acc:MGI:3642547]	5.622727	9.119849	8.037016	5.949754	9.611956	14.17189
17308783	Gm10132	predicted gene 10132 [Source:MGI Symbol;Acc:MGI:3704449]	27.12987	38.56205	39.13778	31.87846	41.95614	34.40151
17368683	Gm10134	predicted gene 10134 [Source:MGI Symbol;Acc:MGI:3642322]	7.870689	10.92855	11.11989	10.67689	6.823112	8.728235
17288219	Gm10139	predicted gene 10139	6.813707	17.03146	11.17629	6.366939	11.69516	11.90759
17234711	Gm10142	predicted gene 10142	11.68836	13.08126	13.99918	10.85283	12.26257	13.11258
17357475	Gm10143	predicted gene 10143 [Source:MGI Symbol;Acc:MGI:3704492]	5.338965	5.0755	5.394129	6.132222	4.146373	5.125668
17498067	Gm10153	predicted gene 10153 [Source:MGI Symbol;Acc:MGI:3642359]	7.02647	8.554657	6.994447	9.176665	7.584373	7.76061
17426915	Gm10154	predicted gene 10154 [Source:MGI Symbol;Acc:MGI:3642271]	26.79887	33.66366	43.16111	26.15437	30.79171	29.67446
17496146	Gm10155	predicted gene 10155 [Source:MGI Symbol;Acc:MGI:3642914]	702.717	766.1643	831.2566	771.4025	755.0666	754.3342
17548777	Gm10157	predicted gene 10157 [Source:MGI Symbol;Acc:MGI:3642264]	1495.643	1391.958	1346.436	1455.763	1520.247	1545.052
17492858	Gm10160	predicted gene 10160 [Source:MGI Symbol;Acc:MGI:3642884]	1.986639	3.047423	2.390993	2.291623	1.873466	2.077157
17240595	Gm10180	predicted gene 10180 [Source:MGI Symbol;Acc:MGI:3701608]	601.5997	730.5099	843.8074	771.3009	858.1345	840.2064
17525073	Gm10180	predicted gene 10180 [Source:MGI Symbol;Acc:MGI:3701608]	389.6162	545.6946	604.2744	605.1798	621.7066	677.0554
17226870	Gm10188	predicted gene 10188 [Source:MGI Symbol;Acc:MGI:3704196]	8.356681	7.854721	6.301477	6.125298	8.360852	8.755624
17347531	Gm10190	predicted gene 10190	3.418634	3.020505	3.392291	3.835275	3.548305	4.193486
17427441	Gm10192	predicted gene 10192 [Source:MGI Symbol;Acc:MGI:3642867]	5.197904	3.953143	3.374164	4.80318	3.488701	3.811899
17365690	Gm10197	predicted gene 10197 [Source:MGI Symbol;Acc:MGI:3704501]	18.12137	18.43276	22.79318	15.39532	22.84442	20.76368
17464490	Gm10203	predicted gene 10203 [Source:MGI Symbol;Acc:MGI:3642365]	2.57855	2.554304	2.264747	2.527919	5.033144	3.1156
17446507	Gm10220	predicted gene 10220	2.675762	2.697345	2.566816	4.713439	3.584893	4.026175
17331890	Gm10228	predicted gene 10228	2.874797	2.715937	2.242969	2.15889	3.091806	2.689672
17326926	Gm10229	predicted gene 10229	2.381417	2.650069	3.041093	3.593326	2.458673	2.285991
17325271	Gm10237	predicted gene 10237 [Source:MGI Symbol;Acc:MGI:3641816]	3.293611	3.954266	6.311968	5.120199	4.085577	4.432437
17312050	Gm10240	predicted gene 10240 [Source:MGI Symbol;Acc:MGI:3704454]	1260.902	1313.081	1384.021	1294.079	1267.386	1277.591
17466639	Gm10242	predicted gene 10242 [Source:MGI Symbol;Acc:MGI:3642310]	8.456613	4.432099	5.553472	2.877349	3.45008	4.421114
17457511	Gm10244	predicted gene 10244	8.467112	8.333197	8.467112	7.437254	11.68293	10.0467
17548504	Gm10244	predicted gene 10244 [Source:MGI Symbol;Acc:MGI:3642504]	14.2586	11.33954	12.35369	13.17262	10.52479	11.8972
17328042	Gm10247	predicted gene 10247 [Source:MGI Symbol;Acc:MGI:3641838]	264.9911	255.8936	282.0994	290.8981	321.4922	312.6121
17304005	Gm10248	predicted gene 10248	2.770795	3.301496	3.217923	3.034502	3.156225	3.057664
17546345	Gm10256	predicted gene 10256	3.262708	2.983281	3.228446	3.351345	3.710362	3.339703
17546355	Gm10256	predicted gene 10256	2.979479	2.694897	2.75704	3.009972	3.332419	3.009972
17295788	Gm10257	predicted gene 10257	13.524	19.92365	22.03996	19.42873	20.5351	19.74014
17404509	Gm10258	predicted gene 10258	3.070833	2.740768	2.961052	3.009972	3.133858	2.618572
17295304	Gm10260	predicted gene 10260 [Source:MGI Symbol;Acc:MGI:3642298]	9.55622	6.495227	7.953997	7.560177	7.560177	6.477693
17354170	Gm10267	predicted gene 10267	2.115033	2.511014	2.071377	2.084888	2.656101	2.540179
17349977	Gm10268	predicted gene 10268 [Source:MGI Symbol;Acc:MGI:3642637]	321.383	312.6273	359.0647	317.1057	267.1618	318.5619
17266298	Gm10277	predicted gene 10277	8.794795	8.491268	6.089822	5.684644	7.401974	6.406827
17505821	Gm10280	predicted gene 10280	2.238078	2.725177	2.385133	2.476118	3.091014	2.580499
17509500	Gm10283	predicted gene 10283	2.67402	2.332763	2.420844	2.332763	2.420844	2.36459
17411135	Gm10287	predicted gene 10287 [Source:MGI Symbol;Acc:MGI:3642138]	4.819188	4.734231	3.984117	4.923758	4.046543	4.301799
17411099	Gm10288	predicted gene 10288 [Source:MGI Symbol;Acc:MGI:3704227]	941.9456	810.2818	795.8595	1035.434	917.9934	1023.376
17492074	Gm10295	predicted gene 10295 [Source:MGI Symbol;Acc:MGI:3642770]	2.67402	2.696803	2.67402	2.43547	2.67402	2.406723
17419374	Gm10300	predicted gene 10300 [Source:MGI Symbol;Acc:MGI:3641670]	20.05514	17.78994	20.08867	20.22111	23.01113	23.91195
17415434	Gm10306	predicted gene 10306 [Source:MGI Symbol;Acc:MGI:3704234]	4.20927	5.747044	3.973251	3.703322	3.796605	4.56739
17340393	Gm10308	predicted gene 10308 [Source:MGI Symbol;Acc:MGI:3641943]	14.18889	11.0141	10.5806	13.88004	11.22489	11.48405
17347877	Gm10309	predicted gene 10309 [Source:MGI Symbol;Acc:MGI:3641941]	3.552981	4.20927	4.20927	4.20927	5.482599	4.033689
17234738	Gm10318	predicted gene 10318	3.017053	2.579426	2.897744	3.647455	3.344707	3.891726
17233563	Gm10322	predicted gene 10322 [Source:MGI Symbol;Acc:MGI:3704363]	36.86066	44.58264	38.8335	44.0949	30.6839	34.49296
17293854	Gm10323	predicted gene 10323	2.342372	2.162035	1.955991	1.991976	2.3715	2.072138
17293856	Gm10323	predicted gene 10323	2.507935	2.177934	2.058681	2.036032	2.737654	2.218816
17288239	Gm10324	predicted gene 10324	3.477836	4.476341	2.990548	2.46969	2.509813	3.247546
17280275	Gm10330	predicted gene 10330 [Source:MGI Symbol;Acc:MGI:3642221]	2.406006	2.320587	2.133807	2.237907	2.188921	1.912248
17466311	Gm10334	predicted gene 10334	6.478231	6.287864	5.927197	6.568573	6.992896	6.614261

17231759	Gm10335	predicted gene 10335 [Source:MGI Symbol;Acc:MGI:3641693]	32.04262	36.21938	32.21945	27.84372	27.67497	31.26572
17290563	Gm10336	predicted gene 10336	8.803639	9.345294	7.971217	8.591381	8.903731	11.70733
17303368	Gm10340	predicted gene 10340	2.698018	2.367115	2.616431	2.948355	2.730169	2.513661
17322911	Gm10343	predicted gene 10343 [Source:MGI Symbol;Acc:MGI:3642690]	63.72157	82.88045	99.39215	92.63914	87.06977	110.3926
17445651	Gm10354	predicted gene 10354	6.181599	8.337934	12.53708	7.592211	8.337934	10.68096
17408507	Gm10355	predicted gene 10355 [Source:MGI Symbol;Acc:MGI:3641762]	10.39646	12.4554	11.3474	16.34106	6.237379	13.45248
17510618	Gm10358	predicted gene 10358 [Source:MGI Symbol;Acc:MGI:3708786]	150.5606	143.0276	167.0806	166.8283	147.8151	149.7882
17459386	Gm10360	predicted gene 10360 [Source:MGI Symbol;Acc:MGI:3708738]	2.238652	2.027764	2.117204	2.740141	2.147562	2.358043
17317900	Gm10362	predicted gene 10362	14.37958	14.37958	14.37958	14.09127	14.03007	16.85296
17443128	Gm10369	predicted gene 10369	4.710891	6.816778	4.705833	6.988486	5.862679	5.966356
17305742	Gm10371	predicted gene 10371	4.20927	4.20927	4.20927	4.112113	4.20927	5.023943
17316820	Gm10373	predicted gene 10373	2.754728	2.673464	3.006719	2.354172	2.401557	2.832881
17305493	Gm10375	predicted gene 10375	2.770545	2.878605	2.764407	3.240261	2.423228	2.801948
17305432	Gm10376	predicted gene 10376	2.737217	2.856008	3.010869	2.849562	2.408692	2.729979
17305310	Gm10377	predicted gene 10377	3.2265	2.431657	2.702641	2.78487	3.009575	2.79543
17305413	Gm10377	predicted gene 10377	3.163804	3.076777	3.125138	3.107835	2.6794	3.189256
17299039	Gm10378	predicted gene 10378	3.028157	2.847962	3.414064	3.062691	2.773774	2.963742
17299044	Gm10378	predicted gene 10378	3.822589	4.278485	5.033284	4.192934	4.489236	4.170615
17442709	Gm10382	predicted gene 10382 [Source:MGI Symbol;Acc:MGI:3647829]	23.69449	21.30042	21.57726	19.50308	18.42139	20.82854
17316559	Gm10384	predicted gene 10384	3.601805	3.483699	3.508392	3.069131	2.786878	3.138489
17310316	Gm10389	predicted gene 10389	5.051143	5.398131	4.667532	5.978444	4.609084	4.376841
17451952	Gm10390	predicted gene 10390	8.134987	7.710904	7.377643	9.51458	7.701129	11.48533
17266259	Gm10392	predicted gene 10392 [Source:MGI Symbol;Acc:MGI:3704377]	15.44406	20.22854	18.19185	22.9285	16.07236	18.19185
17297707	Gm10398	predicted gene 10398 [Source:MGI Symbol;Acc:MGI:3642254]	3.814727	4.044552	3.729055	3.729055	3.329441	3.16034
17451865	Gm10399	predicted gene 10399 [Source:MGI Symbol;Acc:MGI:3704255]	11.7828	14.02073	12.55056	14.01384	10.39703	10.73649
17464061	Gm10400	predicted gene 10400	4.887477	3.883991	4.139797	3.637104	4.622425	3.683967
17451645	Gm10401	predicted gene 10401 [Source:MGI Symbol;Acc:MGI:3704254]	12.00347	10.30884	12.12187	12.00347	11.51841	12.25009
17303204	Gm10406	predicted gene 10406	3.013897	3.384071	3.548305	2.92525	3.551701	3.529001
17296639	Gm10408	predicted gene 10408	4.552598	5.10712	4.746848	5.180567	4.643266	6.626559
17296836	Gm10410	predicted gene 10410	4.960756	5.551663	5.428486	5.603895	6.873762	5.37379
17296912	Gm10410	predicted gene 10410	2.582521	2.420844	1.8995	2.420844	2.986923	2.453719
17296943	Gm10410	predicted gene 10410	2.294993	2.868248	3.152311	2.925879	4.276587	3.208258
17296625	Gm10413	predicted gene 10413	2.953407	2.519103	2.860585	3.177826	2.953407	2.953407
17296682	Gm10413	predicted gene 10413	2.953407	2.519103	2.860585	3.177826	2.953407	2.953407
17471146	Gm10415	predicted gene 10415	3.021008	3.339703	3.158238	3.339703	3.897638	3.339703
17440218	Gm10419	predicted gene 10419	3.278168	3.26445	4.148731	4.083624	3.607627	2.814971
17284702	Gm10421	predicted gene 10421	2.675484	2.112439	2.264104	2.296951	2.203657	1.914556
17449880	Gm10424	predicted gene 10424	10.97257	8.467112	8.692674	9.224687	10.54787	8.692674
17284105	Gm10425	predicted gene 10425 [Source:MGI Symbol;Acc:MGI:3642471]	6.97542	8.62581	8.62581	8.074092	11.48421	9.970321
17436987	Gm1043	predicted gene 1043	7.199866	6.787922	5.845798	8.925605	7.085453	7.181972
17548394	Gm1043	predicted gene 1043	13.3149	14.40722	12.5146	14.74864	12.40173	16.56458
17277934	Gm10432	predicted gene 10432	7.630037	6.362439	5.881279	7.700702	6.916873	7.265137
17277919	Gm10433	predicted gene 10433	6.312756	9.789601	6.865382	7.550332	7.746475	7.602796
17250193	Gm10435	predicted gene 10435 [Source:MGI Symbol;Acc:MGI:3642820]	5.320899	6.107957	6.861005	5.901861	4.65803	7.71732
17282942	Gm10436	predicted gene 10436	2.513051	2.2751	2.510683	2.736421	2.704496	2.352737
17538538	Gm10439	predicted gene 10439	5.076837	4.993738	4.41294	5.284548	6.080163	5.95293
17437547	Gm10440	predicted gene 10440	2.855276	2.298862	3.033157	2.523944	2.413355	2.285991
17468281	Gm10445	predicted gene 10445	3.604302	3.513698	4.136642	3.723837	3.104154	3.078398
17262658	Gm10447	predicted gene 10447 [Source:MGI Symbol;Acc:MGI:3642647]	6.517054	6.538683	6.045493	8.934642	5.203419	5.150527
17439054	Gm1045	predicted gene 1045	4.136126	4.570874	3.672519	4.570874	4.059776	4.136126
17276583	Gm10451	predicted gene 10451	4.446376	5.276568	4.982363	6.422567	6.152701	5.581721
17261173	Gm10466	predicted gene 10466	6.627019	6.798045	8.976232	7.297084	13.98337	7.379996
17446490	Gm10471	predicted gene 10471	2.768299	2.765365	3.132751	3.258392	2.730055	3.43519
17435162	Gm10475	predicted gene 10475 [Source:MGI Symbol;Acc:MGI:3642283]	7.667679	7.343918	7.087221	7.534543	6.595674	7.341068
17534935	Gm10477	predicted gene 10477	2.3928	2.239691	2.623713	2.937197	5.56835	2.659343
17445620	Gm10481	predicted gene 10481 [Source:MGI Symbol;Acc:MGI:3708707]	3041.337	3280.455	3657.849	3236.458	3079.819	3032.496
17434655	Gm10482	predicted gene 10482 [Source:MGI Symbol;Acc:MGI:3644444]	3.638743	3.263862	4.002413	3.582558	3.063562	3.582558
17533911	Gm10488	predicted gene 10488	3.437358	3.437358	3.437358	3.449411	3.453239	3.623088
17533965	Gm10488	predicted gene 10488	3.437358	3.437358	3.437358	3.449411	3.453239	3.623088
17533981	Gm10488	predicted gene 10488	3.437358	3.437358	3.437358	3.449411	3.453239	3.623088
17540774	Gm10488	predicted gene 10488	3.563142	3.521573	3.38018	3.025639	3.641165	3.658545
17540793	Gm10488	predicted gene 10488	3.437358	3.437358	3.437358	3.449411	3.453239	3.623088
17540824	Gm10488	predicted gene 10488	3.437358	3.437358	3.437358	3.449411	3.453239	3.623088
17540237	Gm10489	predicted gene 10489 [Source:MGI Symbol;Acc:MGI:3704505]	7.212153	7.716465	7.794789	6.384263	8.222061	7.218755
17533065	Gm10490	predicted gene 10490 [Source:MGI Symbol;Acc:MGI:3642001]	3.513016	5.300432	3.779669	4.801649	3.289071	4.474728
17548014	Gm10493	predicted gene 10493 [Source:MGI Symbol;Acc:MGI:3644330]	7.584899	7.159059	7.846667	7.148596	9.098319	8.706181
17347443	Gm10494	predicted gene 10494	2.248698	2.56168	2.801928	2.352802	3.821291	2.297253
17344686	Gm10499	predicted gene 10499	29.29454	25.96111	30.56333	30.13943	24.93776	27.17164
17344137	Gm10501	predicted gene 10501 [Source:MGI Symbol;Acc:MGI:3704476]	5.49664	4.825405	4.727148	5.036355	4.574455	5.682241
17335124	Gm10505	predicted gene 10505	5.073706	6.587313	4.896735	4.588778	4.785203	5.073706
17341175	Gm10510	predicted gene 10510	4.557239	5.780603	4.721057	6.260032	4.681043	4.681043
17333373	Gm10512	predicted gene 10512	3.302892	3.765392	4.356683	3.690868	4.131312	3.664814
17220877	Gm10516	predicted gene 10516	6.160938	9.002527	7.56264	7.56264	6.030928	7.733331
17220051	Gm10518	predicted gene 10518 [Source:MGI Symbol;Acc:MGI:3704205]	13.73828	14.73954	13.96797	14.85957	17.10137	14.78252
17219431	Gm10521	predicted gene 10521 [Source:MGI Symbol;Acc:MGI:3642358]	3.554508	4.158116	3.366151	3.477643	3.603553	3.120182
17218594	Gm10530	predicted gene 10530 [Source:MGI Symbol;Acc:MGI:3641765]	3.263862	3.321604	3.075306	3.464243	3.447809	3.263862
17351873	Gm10532	predicted gene 10532	7.873145	6.303705	6.594394	5.367462	5.902966	5.902966
17217562	Gm10535	predicted gene 10535 [Source:MGI Symbol;Acc:MGI:3642442]	10.80599	9.573391	9.452264	12.36097	11.24982	10.84005
17350760	Gm10536	predicted gene 10536	3.462322	3.462322	3.570287	3.462322	3.643058	3.056117
17217237	Gm10538	predicted gene 10538	6.606197	7.510818	8.147514	5.032203	6.384351	5.986779
17350258	Gm10542	predicted gene 10542 [Source:MGI Symbol;Acc:MGI:3642604]	1.999556	2.048957	1.862293	2.072138	2.188921	2.395123
17353786	Gm10544	predicted gene 10544 [Source:MGI Symbol;Acc:MGI:3642892]	3.417473	3.569792	2.929329	3.136553	3.136901	2.765058
17353781	Gm10545	predicted gene 10545 [Source:MGI Symbol;Acc:MGI:3704485]	12.9334	11.24366	11.56249	10.04148	11.51833	12.47528
17349357	Gm10549	predicted gene 10549	11.65898	11.41374	10.96702	12.35566	11.02334	12.5563

17348145	Gm10556	predicted gene 10556	3.354761	2.978859	2.852961	3.495551	3.694502	3.416076
17223876	Gm10558	predicted gene 10558 [Source:MGI Symbol;Acc:MGI:3642906]	4.904887	3.94146	3.134315	3.509146	4.991384	4.115621
17433934	Gm10560	predicted gene 10560	6.992896	7.077893	9.187358	9.450692	6.720833	6.934443
17212900	Gm10561	predicted gene 10561	4.181498	3.125528	3.462322	3.401285	3.263862	4.009938
17422471	Gm10564	predicted gene 10564 [Source:MGI Symbol;Acc:MGI:3704240]	8.644245	8.634245	8.555843	9.370006	8.342848	10.48228
17221432	Gm10566	predicted gene 10566 [Source:MGI Symbol;Acc:MGI:3642220]	443.7305	647.9686	652.7334	538.7977	501.9605	559.0099
17548309	Gm10571	predicted gene 10571 [Source:MGI Symbol;Acc:MGI:3708701]	4.661777	3.263862	5.005763	3.789979	3.70494	3.702193
17497947	Gm10575	predicted gene 10575	9.062192	10.32751	8.2644	6.400391	7.342188	8.086358
17427579	Gm10577	predicted gene 10577 [Source:MGI Symbol;Acc:MGI:3642534]	5.383894	5.011665	4.850993	5.605799	4.946987	4.182107
17484278	Gm10578	predicted gene 10578	6.569302	8.494977	6.228566	5.791753	5.812465	6.728337
17484061	Gm10584	predicted gene 10584	4.783544	5.451185	5.75063	4.963095	5.48933	4.539238
17423333	Gm10604	predicted gene 10604 [Source:MGI Symbol;Acc:MGI:3642593]	3.129343	2.01299	2.366065	2.360627	2.285817	3.294547
17523038	Gm10608	predicted gene 10608 [Source:MGI Symbol;Acc:MGI:3642009]	4.880537	4.116448	4.116448	4.116448	4.352065	4.202972
17492897	Gm10610	predicted gene 10610	2.749787	2.642567	3.111801	3.442723	2.718122	2.707385
17506439	Gm10612	predicted gene 10612 [Source:MGI Symbol;Acc:MGI:3708796]	6.440845	6.131797	6.131797	6.131797	5.780042	5.43225
17506270	Gm10614	predicted gene 10614	4.417561	3.611079	3.376059	3.553104	3.256879	3.692429
17522280	Gm10615	predicted gene 10615	6.171709	6.830684	4.904785	6.034231	6.07654	5.345297
17548652	Gm10617	predicted gene 10617 [Source:MGI Symbol;Acc:MGI:3708793]	4.01461	4.473953	4.766036	3.720303	4.295512	3.982957
17492179	Gm10619	predicted gene 10619	4.507133	4.507133	3.238689	3.929049	5.180962	4.194067
17505111	Gm10629	predicted gene 10629 [Source:MGI Symbol;Acc:MGI:3642307]	3.777312	3.381258	3.504722	2.896358	3.455274	3.418255
17512177	Gm10631	predicted gene 10631 [Source:MGI Symbol;Acc:MGI:3642022]	3.508781	4.392706	3.283103	3.809232	5.120306	3.827281
17520116	Gm10634	predicted gene 10634	3.042189	2.827898	2.615198	5.374796	3.084165	3.244621
17529095	Gm10635	predicted gene 10635	4.01461	3.437739	3.373486	3.9363	3.854944	4.17287
17411014	Gm10636	predicted gene 10636	2.775004	2.489179	2.481049	2.67402	3.071621	2.65962
17511466	Gm10637	predicted gene 10637	2.941944	3.46876	3.46876	3.46876	3.268322	3.7825
17503528	Gm10638	predicted gene 10638	6.992896	5.91427	6.129715	6.108508	5.48952	7.274409
17519682	Gm10639	predicted gene 10639	13.524	14.85864	18.33807	16.02031	10.20419	16.97653
17476599	Gm10640	predicted gene 10640	6.265762	6.265762	7.072646	6.930407	6.265762	6.706174
17519084	Gm10642	predicted gene 10642 [Source:MGI Symbol;Acc:MGI:3704338]	19.90129	19.71443	19.68302	20.29525	22.50432	20.29525
17511014	Gm10643	predicted gene 10643 [Source:MGI Symbol;Acc:MGI:3642507]	12.18091	12.68138	8.721885	12.43462	11.3232	11.04583
17510975	Gm10644	predicted gene 10644	4.896938	7.134983	6.801675	5.672128	6.80441	6.648782
17510881	Gm10645	predicted gene 10645 [Source:MGI Symbol;Acc:MGI:3704313]	13.55328	15.32186	14.3354	15.56852	16.63696	17.47027
17518793	Gm10647	predicted gene 10647 [Source:MGI Symbol;Acc:MGI:3642036]	3.233844	3.417639	2.627918	3.008074	2.905568	2.955762
17488561	Gm10648	predicted gene 10648	5.433531	5.069874	3.95465	3.644776	4.58846	5.025649
17510635	Gm10649	predicted gene 10649	6.928774	7.595685	7.456972	7.691314	8.724788	8.36375
17502086	Gm10654	predicted gene 10654	7.230824	6.485285	7.34346	8.24306	7.077648	7.612551
17527923	Gm10655	predicted gene 10655 [Source:MGI Symbol;Acc:MGI:3642575]	7.470941	7.646078	7.729366	7.694726	5.848189	6.937279
17527465	Gm10658	predicted gene 10658	14.44051	12.70919	15.01008	14.78426	13.3921	15.38145
17509716	Gm10660	predicted gene 10660 [Source:MGI Symbol;Acc:MGI:3642761]	2.67402	2.935935	2.502257	2.67402	2.519504	2.866559
17487531	Gm10665	predicted gene 10665	2.798648	2.967463	3.213287	2.900698	3.702372	2.900698
17487621	Gm10665	predicted gene 10665	2.798648	2.967463	3.213287	2.900698	3.702372	2.900698
17487511	Gm10666	predicted gene 10666	2.849502	2.971621	3.094015	2.953407	3.686189	2.939539
17487605	Gm10666	predicted gene 10666	2.849502	2.971621	3.094015	2.953407	3.686189	2.939539
17487545	Gm10668	predicted gene 10668	5.280447	4.504322	4.590591	4.504322	3.591857	4.590591
17487140	Gm10676	predicted gene 10676 [Source:MGI Symbol;Acc:MGI:3704283]	6.992896	8.662119	7.122531	6.943774	9.033719	5.705447
17526659	Gm10677	predicted gene 10677	9.123011	8.492327	7.09784	10.04419	10.05813	7.479179
17526633	Gm10680	predicted gene 10680 [Source:MGI Symbol;Acc:MGI:3704335]	64.88801	64.09545	85.48741	72.16113	69.70934	57.65485
17526473	Gm10684	predicted gene 10684	2.578514	3.883991	3.182786	3.497878	3.497878	3.965135
17526127	Gm10687	predicted gene 10687	5.163073	6.498334	5.062009	5.141039	3.995705	7.577805
17407531	Gm10696	predicted gene 10696	5.75067	9.603897	7.070146	7.08231	6.702503	8.180052
17407522	Gm10697	predicted gene 10697	3.991193	3.792765	4.642108	4.287153	4.514731	3.729055
17407529	Gm10697	predicted gene 10697	3.991193	3.792765	4.642108	4.287153	4.514731	3.729055
17459470	Gm1070	predicted gene 1070	10.74669	11.84799	10.00986	9.771161	10.79013	12.33888
17525235	Gm10700	predicted gene 10700	4.774038	4.90363	4.002441	6.010979	4.004966	4.451711
17515510	Gm10701	predicted gene 10701 [Source:MGI Symbol;Acc:MGI:3642532]	4.876784	3.140447	4.144046	3.447116	4.604358	3.860908
17406512	Gm10705	predicted gene 10705	3.792003	2.067289	2.281638	2.393713	2.45428	2.06759
17514754	Gm10706	predicted gene 10706	7.753297	5.734787	4.523972	5.734787	5.860322	5.44873
17406264	Gm10710	predicted gene 10710	3.461794	4.499953	5.789946	4.027962	3.842513	5.650937
17395124	Gm10714	predicted gene 10714	5.394391	5.952807	5.338965	5.709087	5.075641	5.784772
17514352	Gm10715	predicted gene 10715 [Source:MGI Symbol;Acc:MGI:3642376]	21.85361	45.47305	62.14257	26.9935	43.33708	51.77343
17514355	Gm10715	predicted gene 10715 [Source:MGI Symbol;Acc:MGI:3642376]	2.995984	6.763828	9.310251	4.801649	6.106603	6.471768
17514343	Gm10717	predicted gene 10717 [Source:MGI Symbol;Acc:MGI:3642031]	18.58891	47.06589	78.16116	34.31244	42.18798	40.95223
17514346	Gm10717	predicted gene 10717 [Source:MGI Symbol;Acc:MGI:3642031]	19.58612	32.20092	49.45313	25.15704	34.54368	43.03862
17514349	Gm10717	predicted gene 10717 [Source:MGI Symbol;Acc:MGI:3642031]	23.84839	59.89941	82.42328	35.01716	56.99802	78.64974
17514340	Gm10718	predicted gene 10718 [Source:MGI Symbol;Acc:MGI:3642028]	31.62777	86.63812	151.5111	52.09453	95.73072	83.29454
17514330	Gm10719	predicted gene 10719 [Source:MGI Symbol;Acc:MGI:3641690]	13.98938	29.48788	39.77996	20.72773	27.52155	28.43528
17514333	Gm10719	predicted gene 10719 [Source:MGI Symbol;Acc:MGI:3641690]	55.51824	112.7005	193.9243	77.50137	136.112	133.176
17514337	Gm10719	predicted gene 10719 [Source:MGI Symbol;Acc:MGI:3641690]	6.246725	11.40338	17.70531	9.219701	10.9576	14.14176
17514326	Gm10722	predicted gene 10722 [Source:MGI Symbol;Acc:MGI:3642024]	2.694803	2.038249	3.766488	2.677785	4.113111	2.57289
17296277	Gm10735	predicted gene 10735 [Source:MGI Symbol;Acc:MGI:3642476]	3.947584	4.132887	3.473957	4.84246	4.11582	4.724298
17404156	Gm10745	predicted gene 10745	2.305548	2.275195	2.792699	2.277893	2.387201	2.502882
17237534	Gm10747	predicted gene 10747	5.998659	7.452322	6.031095	5.818309	7.104829	7.675709
17392621	Gm10750	predicted gene 10750 [Source:MGI Symbol;Acc:MGI:3641996]	9.33733	8.817624	8.717956	8.810049	10.18772	10.55167
17244533	Gm10754	predicted gene 10754	4.765706	2.920368	2.684773	2.953407	3.064921	2.612444
17294514	Gm10760	predicted gene 10760	23.5852	21.11842	21.16546	22.39136	19.68534	21.09501
17391506	Gm10762	predicted gene 10762 [Source:MGI Symbol;Acc:MGI:3704209]	14.29888	11.3988	9.055473	13.49235	13.29342	16.0922
17288269	Gm10767	predicted gene 10767	9.182646	9.182646	9.182646	9.182646	9.012267	9.182646
17359672	Gm10768	predicted gene 10768	175.5855	233.7836	198.2188	193.6503	234.5904	247.621
17392642	Gm10770	predicted gene 10770 [Source:MGI Symbol;Acc:MGI:3642046]	2.406006	2.06537	1.955924	1.881118	1.807881	1.847226
17288189	Gm10775	predicted gene 10775 [Source:MGI Symbol;Acc:MGI:3642324]	8.298497	8.224512	8.676782	9.00209	6.464371	9.885199
17235821	Gm10778	predicted gene 10778	14.04963	12.38232	12.58462	10.66986	9.104438	10.1991
17287794	Gm10782	predicted gene 10782	5.117751	5.722319	4.348054	4.712622	4.616406	5.724144
17327130	Gm10785	predicted gene 10785	4.602922	4.801649	4.486157	4.504509	4.924817	5.137085
17234606	Gm10787	predicted gene 10787	4.421451	4.334663	4.201953	3.703322	4.283203	3.514599

17326954	Gm10789	predicted gene 10789	4.043767	3.856601	3.852327	3.500376	3.877562	4.063745
17286808	Gm10790	predicted gene 10790	4.590114	3.54506	4.427554	4.308722	4.943764	5.140398
17326834	Gm10791	predicted gene 10791	4.01461	3.872405	3.560155	3.872405	3.573307	3.872405
17388899	Gm10799	predicted gene 10799 [Source:MGI Symbol;Acc:MGI:3642868]	19.21246	27.21613	15.47921	25.15721	25.5282	26.96133
17388663	Gm10800	predicted gene 10800 [Source:MGI Symbol;Acc:MGI:3641657]	507.7414	1399.892	1552.567	921.4742	1267.922	1343.621
17373634	Gm10801	predicted gene 10801 [Source:MGI Symbol;Acc:MGI:3641656]	2.799269	2.375333	2.739685	2.260904	2.260544	2.36079
17373580	Gm10803	predicted gene 10803 [Source:MGI Symbol;Acc:MGI:3641754]	3.566552	4.517329	4.07356	4.875736	3.488701	5.12549
17373572	Gm10804	predicted gene 10804	4.295154	4.844675	4.108011	4.283351	3.313624	4.093571
17356577	Gm10814	predicted gene 10814	6.235564	5.225105	6.380104	6.260075	6.577229	7.472391
17363237	Gm10819	predicted gene 10819 [Source:MGI Symbol;Acc:MGI:3642135]	47.69447	58.82796	40.42792	46.18172	46.60523	50.87765
17372024	Gm10822	predicted gene 10822 [Source:MGI Symbol;Acc:MGI:3641955]	3.353064	3.287885	3.395279	3.803928	3.412006	4.356803
17324570	Gm10823	predicted gene 10823	4.580833	3.525236	3.693299	4.521997	3.977412	4.253145
17232132	Gm10824	predicted gene 10824	3.340537	3.675167	2.940517	3.263862	4.277862	3.795895
17232116	Gm10825	predicted gene 10825	4.299801	4.693747	4.689194	4.610745	4.352065	6.101779
17231823	Gm10827	predicted gene 10827 [Source:MGI Symbol;Acc:MGI:3642321]	9.754784	8.529079	9.138517	9.091447	8.632689	9.441198
17302885	Gm10837	predicted gene 10837 [Source:MGI Symbol;Acc:MGI:3642026]	5.266447	5.998742	5.789585	7.992351	5.053069	6.837458
17257532	Gm10840	predicted gene 10840 [Source:MGI Symbol;Acc:MGI:3642918]	27.78415	31.70525	32.24917	30.24784	28.81664	26.74579
17257329	Gm10842	predicted gene 10842 [Source:MGI Symbol;Acc:MGI:3642916]	2.374576	2.782362	2.007918	2.100656	2.140043	1.900673
17308946	Gm10845	predicted gene 10845	4.702833	3.830971	3.394196	2.898768	3.140491	3.204746
17301943	Gm10847	predicted gene 10847	3.230976	3.634609	3.975014	3.702039	3.263115	3.366828
17366988	Gm10851	predicted gene 10851	6.992896	6.992896	7.869544	6.992896	7.347279	7.748784
17381511	Gm10855	predicted gene 10855	4.01461	3.127153	3.703322	3.02801	2.947231	3.100563
17366586	Gm10857	predicted gene 10857	3.755956	5.978361	5.18168	4.342753	5.558363	5.162071
17307963	Gm10860	predicted gene 10860	3.096138	2.967401	3.588423	2.890141	3.520938	2.967401
17548131	Gm10862	predicted gene 10862 [Source:MGI Symbol;Acc:MGI:3641622]	3.354598	4.382457	4.469087	4.651739	3.786487	4.137588
17312946	Gm10863	predicted gene 10863	9.332994	15.33855	12.09057	11.31536	9.519066	12.04606
17319103	Gm10865	predicted gene 10865	28.09088	21.53155	30.43143	18.63457	22.18373	34.10402
17443874	Gm10874	predicted gene 10874 [Source:MGI Symbol;Acc:MGI:3704261]	21.80429	18.48996	19.37494	19.10449	18.37815	17.77959
17373813	Gm10912	predicted gene 10912 [Source:MGI Symbol;Acc:MGI:3809094]	3.359508	3.363842	3.112298	3.401403	3.734772	3.24226
17299516	Gm10916	predicted gene 10916 [Source:MGI Symbol;Acc:MGI:3779123]	20.03055	24.78783	40.1495	40.42399	38.77971	28.59934
17539754	Gm10921	predicted gene 10921	3.102798	3.14561	3.39735	3.755825	4.552945	3.429766
17540885	Gm10921	predicted gene 10921	3.102798	3.14561	3.39735	3.755825	4.552945	3.429766
17532714	Gm10922	predicted gene 10922	3.545699	4.249432	3.66972	4.340617	2.883858	7.468042
17548294	Gm10931	predicted gene 10931	10.20108	9.06747	9.96438	10.23004	12.22227	7.44752
17548498	Gm10931	predicted gene 10931	10.20108	9.06747	9.96438	10.23004	12.22227	7.44752
17488366	Gm10935	predicted gene 10935 [Source:MGI Symbol;Acc:MGI:3809041]	3.239744	3.016049	3.391915	2.78107	3.742857	3.023333
17237545	Gm10936	predicted gene 10936 [Source:MGI Symbol;Acc:MGI:3779146]	2.341641	2.819579	2.455471	2.534937	3.166392	3.342065
17242276	Gm10941	predicted gene 10941	7.787553	8.42683	9.51966	7.076912	8.400819	7.388031
17231619	Gm10944	predicted gene 10944 [Source:MGI Symbol;Acc:MGI:3779154]	3.174875	2.88162	3.516816	4.115621	2.267323	2.995631
17239003	Gm10945	predicted gene 10945 [Source:MGI Symbol;Acc:MGI:3779155]	1.898776	1.660894	2.005108	2.41195	3.193636	1.780263
17475064	Gm1096	predicted gene 1096	4.228277	4.188489	3.341417	3.687927	4.229772	3.309401
17401586	Gm10961	predicted gene 10961	9.071493	14.41881	9.900079	6.844333	10.67223	10.92151
17400118	Gm10972	predicted gene 10972 [Source:MGI Symbol;Acc:MGI:3779183]	3.062691	3.643336	2.788289	2.687286	2.786222	4.110232
17380526	Gm11009	predicted gene 11009	2.559284	2.523804	2.751427	2.711404	2.308427	2.534902
17395342	Gm11009	predicted gene 11009	2.461674	2.427548	2.698822	2.435672	2.19611	2.438223
17394908	Gm11011	predicted gene 11011 [Source:MGI Symbol;Acc:MGI:3779229]	9.181883	7.536214	7.343155	8.317961	8.317443	7.840252
17504434	Gm11020	predicted gene 11020 [Source:MGI Symbol;Acc:MGI:3779240]	8.560529	8.914229	9.821477	10.27324	9.077972	6.602031
17279582	Gm11027	predicted gene 11027 [Source:MGI Symbol;Acc:MGI:3779250]	3.812375	3.54506	1.969665	2.579283	1.969665	2.953407
17376441	Gm11037	predicted gene 11037 [Source:MGI Symbol;Acc:MGI:3779261]	4.730524	2.694	2.694	2.322921	2.317116	2.727539
17464037	Gm11077	predicted gene 11077 [Source:MGI Symbol;Acc:MGI:3779304]	2.04802	2.431405	2.347296	2.152719	2.133231	1.737641
17525141	Gm1110	predicted gene 1110	3.171928	3.031391	3.009972	3.566033	3.226858	3.421408
17297189	Gm11100	predicted gene 11100	10.82534	9.014141	11.9033	10.43559	11.9636	9.70703
17346510	Gm11110	predicted gene 11110	4.994949	5.160783	5.587101	6.221144	5.198949	6.626697
17449445	Gm11115	predicted gene 11115 [Source:MGI Symbol;Acc:MGI:3779367]	2.461623	1.646805	1.92131	1.825738	2.813371	2.204971
17344620	Gm11127	predicted gene 11127	4.05109	3.573233	4.027041	3.22612	3.729055	4.20927
17468425	Gm11128	predicted gene 11128	3.516207	7.206606	3.893749	3.893317	4.169516	5.586211
17525395	Gm1113	predicted gene 1113 [Source:MGI Symbol;Acc:MGI:2685959]	3.729055	4.801649	3.622737	4.028799	3.729055	3.574001
17344331	Gm11131	predicted gene 11131 [Source:MGI Symbol;Acc:MGI:3779386]	9.900422	11.71731	9.098823	8.797051	6.039253	9.310251
17331581	Gm11146	predicted gene 11146 [Source:MGI Symbol;Acc:MGI:3779400]	2.406006	2.786936	2.67402	2.786936	2.782767	2.806321
17517084	Gm11149	predicted gene 11149	4.200264	4.600211	4.111515	4.667079	4.9872	4.271581
17341000	Gm11166	predicted gene 11166	5.416947	6.192277	5.382663	6.580741	5.120306	6.198676
17514328	Gm11168	predicted gene 11168 [Source:MGI Symbol;Acc:MGI:3779420]	16.73887	40.41755	63.13596	26.4609	36.04407	48.62681
17510127	Gm11175	predicted gene 11175 [Source:MGI Symbol;Acc:MGI:3809055]	16.84198	18.46153	19.75889	17.90512	20.58541	15.25013
17415012	Gm11184	predicted gene 11184	2.305948	3.108632	3.310525	3.618295	3.187157	2.868825
17262616	Gm11186	predicted gene 11186	6.723442	4.286232	5.455154	5.413757	4.051427	5.142893
17266315	Gm11190	predicted gene 11190	14.29709	16.23595	14.93753	17.60435	17.88874	18.62954
17253645	Gm11195	predicted gene 11195 [Source:MGI Symbol;Acc:MGI:3649277]	5.487237	5.60308	4.886529	5.794041	5.049568	5.039777
17253739	Gm11201	predicted gene 11201	12.67303	15.37431	10.54926	12.97669	10.4142	13.00036
17253832	Gm11205	predicted gene 11205	9.815284	9.882322	10.39696	9.815284	10.86669	7.546774
17426230	Gm11209	predicted gene 11209 [Source:MGI Symbol;Acc:MGI:3650632]	6.502435	6.992896	6.785194	6.735663	4.007187	7.540185
17414642	Gm11210	predicted gene 11210	4.818687	5.473107	6.541448	6.14895	5.978402	6.126529
17426233	Gm11211	predicted gene 11211	8.720555	8.163399	7.181574	7.246277	7.698994	7.545884
17426336	Gm11213	predicted gene 11213	2.686132	2.888859	2.872845	2.601926	3.100219	3.195208
17414796	Gm11216	predicted gene 11216 [Source:MGI Symbol;Acc:MGI:3651044]	2.475329	2.754575	2.555791	2.984895	2.782767	2.285991
17426397	Gm11217	predicted gene 11217	2.938439	2.741376	2.828329	2.717594	3.294958	2.857077
17426507	Gm11228	predicted gene 11228	2.046403	2.239691	2.724459	2.107993	2.688926	2.599384
17529990	Gm1123	predicted gene 1123	7.688062	8.110776	7.356043	8.032227	8.282062	8.71983
17414855	Gm11234	predicted gene 11234 [Source:MGI Symbol;Acc:MGI:3651441]	3.706404	3.134836	2.741566	3.134836	3.319624	3.086475
17414884	Gm11236	predicted gene 11236	3.582483	4.077656	4.568403	3.648542	4.423965	4.494903
17414891	Gm11238	predicted gene 11238	4.56043	5.560097	6.970538	4.992249	6.263504	7.702838
17414908	Gm11240	predicted gene 11240	2.405939	2.318385	1.894648	2.318385	2.18604	2.366311
17414858	Gm11250	predicted gene 11250 [Source:MGI Symbol;Acc:MGI:3651181]	2.447281	2.575442	2.393895	1.926591	2.928009	2.722364
17426695	Gm11261	predicted gene 11261 [Source:MGI Symbol;Acc:MGI:3650239]	1.862009	2.474334	2.159953	2.197846	2.751999	5.592875
17426756	Gm11264	predicted gene 11264	3.086518	2.685461	2.822395	3.666745	3.077694	3.52839

17426786	Gm11267	predicted gene 11267	2.824724	2.824724	2.97397	3.157582	3.566577	2.94999
17415006	Gm11269	predicted gene 11269	3.920044	3.887062	3.899286	4.098426	3.00574	4.039621
17285693	Gm11274	predicted gene 11274 [Source:MGI Symbol;Acc:MGI:3649225]	2.360627	2.549916	2.219139	2.19249	2.219893	2.717312
17291028	Gm11278	predicted gene 11278	4.519584	3.301382	3.788929	4.771788	3.376597	3.325451
17285731	Gm11290	predicted gene 11290 [Source:MGI Symbol;Acc:MGI:3651659]	4.420532	3.880708	3.880708	5.101813	3.396926	3.880708
17285736	Gm11291	predicted gene 11291 [Source:MGI Symbol;Acc:MGI:3651661]	10.53516	10.21176	11.37899	13.71089	12.12372	11.91279
17285739	Gm11292	predicted gene 11292 [Source:MGI Symbol;Acc:MGI:3651660]	5.471286	8.133239	6.908823	5.93069	7.077354	6.916202
17291078	Gm11300	predicted gene 11300 [Source:MGI Symbol;Acc:MGI:3651398]	3.458221	2.341106	2.373638	2.23662	2.242798	2.187177
17285777	Gm11309	predicted gene 11309 [Source:MGI Symbol;Acc:MGI:3650169]	4.681043	4.735773	5.728947	5.489025	3.263862	12.28529
17285780	Gm11314	predicted gene 11314 [Source:MGI Symbol;Acc:MGI:3650397]	7.985872	6.263938	5.20259	9.332218	8.594895	6.256908
17291174	Gm11335	predicted gene 11335	2.983633	3.263862	3.437143	4.079861	2.411131	3.339703
17291237	Gm11337	predicted gene 11337	10.51342	9.433479	9.633834	10.29877	8.980091	8.472102
17285940	Gm11339	predicted gene 11339 [Source:MGI Symbol;Acc:MGI:3652101]	3.082455	2.934612	2.562781	3.275148	2.901422	3.176372
17291325	Gm11345	predicted gene 11345	7.251955	6.932761	8.465058	8.602166	9.885404	7.526296
17291397	Gm11351	predicted gene 11351	2.355299	3.052497	2.598646	2.601385	3.284765	2.735984
17286104	Gm11353	predicted gene 11353 [Source:MGI Symbol;Acc:MGI:3649794]	112.8263	116.5787	129.6708	106.424	117.6143	112.19
17286210	Gm11360	predicted gene 11360 [Source:MGI Symbol;Acc:MGI:3649916]	22.16621	30.57202	34.50922	30.17831	21.71485	38.84562
17291531	Gm11364	predicted gene 11364 [Source:MGI Symbol;Acc:MGI:3652071]	3.062691	3.263862	2.832979	3.009972	3.009972	3.166568
17286246	Gm11365	predicted gene 11365 [Source:MGI Symbol;Acc:MGI:3652070]	3.729055	3.671552	3.729055	4.118703	3.671552	3.589169
17286249	Gm11368	predicted gene 11368 [Source:MGI Symbol;Acc:MGI:3652073]	2.981441	3.107361	3.163518	2.906635	3.583626	2.981441
17286311	Gm11376	predicted gene 11376 [Source:MGI Symbol;Acc:MGI:3650400]	2.921744	3.442174	3.339703	3.263862	3.69074	3.431668
17286324	Gm11377	predicted gene 11377	4.112085	4.227427	3.760697	5.080393	3.712189	4.154198
17286329	Gm11378	predicted gene 11378 [Source:MGI Symbol;Acc:MGI:3650784]	8.6184	6.137368	6.646938	10.52757	9.635837	6.661144
17286337	Gm11379	predicted gene 11379 [Source:MGI Symbol;Acc:MGI:3650783]	5.027217	4.166656	5.04481	4.495308	4.779368	4.209912
17291667	Gm11381	predicted gene 11381 [Source:MGI Symbol;Acc:MGI:3650781]	4.249402	4.155177	3.703322	5.078421	2.943168	3.415839
17246516	Gm11399	predicted gene 11399 [Source:MGI Symbol;Acc:MGI:3650744]	2.597187	2.851024	3.033998	3.074219	2.925757	3.608527
17535196	Gm1140	predicted gene 1140	2.846877	2.953407	2.370448	2.846877	3.283217	3.308254
17542100	Gm1140	predicted gene 1140	2.846877	2.953407	2.370448	2.846877	3.283217	3.308254
17535376	Gm1141	predicted gene 1141	2.978426	3.474078	3.121415	3.263862	3.468039	3.246286
17426886	Gm11413	predicted gene 11413	4.369778	4.114765	4.20927	4.114765	3.967114	4.20927
17426919	Gm11415	predicted gene 11415	4.400508	3.360812	3.968324	5.255544	4.243516	4.69212
17254015	Gm11417	predicted gene 11417 [Source:MGI Symbol;Acc:MGI:3651779]	4.7407	3.875332	3.560722	4.810277	3.961701	5.078527
17254023	Gm11418	predicted gene 11418	1.96905	2.78001	2.006192	2.159773	2.435993	2.548521
17254037	Gm11419	predicted gene 11419	3.588572	2.771525	3.016593	2.771525	2.771525	2.771525
17266792	Gm11423	predicted gene 11423	11.47005	11.05518	10.14193	10.78459	11.05518	14.37958
17254085	Gm11426	predicted gene 11426	5.95194	5.646708	5.513812	5.480737	7.000219	5.015488
17267053	Gm11431	predicted gene 11431 [Source:MGI Symbol;Acc:MGI:3650047]	9.079986	8.652158	8.581144	11.70928	10.56236	8.281603
17267011	Gm11437	predicted gene 11437	16.06167	15.78115	16.10269	20.27584	27.15094	16.06167
17267198	Gm11444	predicted gene 11444 [Source:MGI Symbol;Acc:MGI:3651161]	4.868813	4.868813	4.485846	4.853683	3.651856	6.25266
17379224	Gm11454	predicted gene 11454	4.088014	4.883684	5.807954	4.549916	5.289495	5.373641
17379375	Gm11455	predicted gene 11455 [Source:MGI Symbol;Acc:MGI:3650216]	4.654383	5.914516	4.168938	5.499155	5.991885	4.840178
17394259	Gm11457	predicted gene 11457	5.601099	4.801649	4.579055	4.975633	5.311245	6.861481
17394535	Gm11464	predicted gene 11464 [Source:MGI Symbol;Acc:MGI:3650572]	2.67402	3.02216	3.02216	3.680776	2.515909	3.337945
17379716	Gm11465	predicted gene 11465	4.309289	3.54506	2.606565	4.056878	3.217299	3.56018
17394568	Gm11466	predicted gene 11466 [Source:MGI Symbol;Acc:MGI:3650521]	3.499212	4.123416	3.280828	4.70003	3.729055	3.729055
17394571	Gm11467	predicted gene 11467 [Source:MGI Symbol;Acc:MGI:3650520]	12.64311	11.31716	13.60278	14.65773	15.1192	16.46397
17379746	Gm11468	predicted gene 11468	3.462322	3.380344	3.285493	3.418421	2.951012	3.7289
17379928	Gm11476	predicted gene 11476	6.992896	5.885194	5.568003	6.766689	7.649524	5.603895
17414782	Gm11482	predicted gene 11482	5.833897	6.129163	5.641387	5.833897	5.833897	5.331347
17426555	Gm11487	predicted gene 11487	3.268571	3.103078	3.236782	2.99775	2.938073	3.114434
17267466	Gm11491	predicted gene 11491 [Source:MGI Symbol;Acc:MGI:3651138]	3.3429	3.27835	2.802938	2.92594	3.593775	3.179831
17267581	Gm11493	predicted gene 11493 [Source:MGI Symbol;Acc:MGI:3651324]	2.811363	3.461338	3.461338	3.8382	3.912525	4.962372
17267695	Gm11494	predicted gene 11494	4.190987	4.054826	3.721051	3.414632	2.7549	3.569173
17267651	Gm11497	predicted gene 11497 [Source:MGI Symbol;Acc:MGI:3651117]	8.962182	9.861246	9.861246	9.385672	10.6587	10.29855
17267501	Gm11505	predicted gene 11505 [Source:MGI Symbol;Acc:MGI:3651152]	3.550322	3.744492	3.516816	3.516816	2.59287	3.781949
17267553	Gm11508	predicted gene 11508 [Source:MGI Symbol;Acc:MGI:3650928]	10.3356	7.330593	8.583881	6.814463	7.573237	7.169189
17255348	Gm11513	predicted gene 11513	10.0389	9.652616	9.777936	13.60863	7.654934	11.0798
17268202	Gm11521	predicted gene 11521 [Source:MGI Symbol;Acc:MGI:3651772]	3.005281	3.390293	2.652072	2.67402	2.612915	2.627517
17268401	Gm11525	predicted gene 11525	6.820039	6.113421	6.31652	6.363454	5.383036	6.972954
17268240	Gm11527	predicted gene 11527 [Source:MGI Symbol;Acc:MGI:3649799]	6.845187	7.097053	6.005149	6.0044	7.714432	7.96343
17255423	Gm11528	predicted gene 11528 [Source:MGI Symbol;Acc:MGI:3649788]	6.813944	8.249919	9.241839	8.101674	7.985042	10.37486
17268328	Gm11529	predicted gene 11529	5.891357	7.423194	5.120306	6.854588	6.561611	8.616309
17268427	Gm11532	predicted gene 11532	3.459803	4.126532	3.703322	3.459803	2.863382	3.339703
17255479	Gm11534	predicted gene 11534 [Source:MGI Symbol;Acc:MGI:3650775]	3.540713	4.066563	3.339703	3.276942	3.339703	2.947885
17255482	Gm11534	predicted gene 11534 [Source:MGI Symbol;Acc:MGI:3650775]	3.016467	2.204482	2.866817	3.059827	2.845555	3.132686
17255531	Gm11535	predicted gene 11535 [Source:MGI Symbol;Acc:MGI:3650113]	3.155394	3.7334	3.54506	3.54506	4.100159	3.495139
17268318	Gm11536	predicted gene 11536 [Source:MGI Symbol;Acc:MGI:3650116]	4.874824	5.867598	6.373783	6.155645	5.116368	5.803688
17255650	Gm11537	predicted gene 11537 [Source:MGI Symbol;Acc:MGI:3705160]	5.501252	6.964598	6.895762	5.71776	7.396537	6.725539
17255540	Gm11538	predicted gene 11538	3.354226	3.938783	4.222742	4.285103	3.219932	4.219816
17268061	Gm11541	predicted gene 11541	3.18539	4.013748	3.477499	3.825823	3.237544	3.576876
17255251	Gm11542	predicted gene 11542 [Source:MGI Symbol;Acc:MGI:3650067]	4.401911	3.703322	3.703322	3.415129	3.703322	3.269817
17268088	Gm11543	predicted gene 11543	5.457953	3.071067	2.48661	2.694392	3.963486	3.249933
17268093	Gm11544	predicted gene 11544	5.16039	4.529651	4.966398	6.102456	5.478686	6.784347
17268066	Gm11545	predicted gene 11545	11.15633	6.992896	8.467112	5.592526	7.207355	6.992896
17268079	Gm11546	predicted gene 11546	2.070587	2.088417	2.897201	2.627335	2.447553	2.455539
17404811	Gm11548	predicted gene 11548	2.82635	2.421062	2.67402	2.67402	2.338462	3.023752
17404816	Gm11549	predicted gene 11549	2.497959	2.933677	3.277101	3.366805	3.200351	3.220222
17256854	Gm11551	predicted gene 11551	6.063539	7.30903	5.051529	6.063539	5.730416	6.063539
17269241	Gm11554	predicted gene 11554	6.464057	6.150868	6.515519	5.696122	6.525145	5.132707
17269175	Gm11555	predicted gene 11555 [Source:MGI Symbol;Acc:MGI:3651823]	1.932074	2.074236	1.543302	1.932074	2.342274	2.038164
17256306	Gm11559	predicted gene 11559	2.328821	2.938174	3.146199	2.229299	2.844569	2.864454
17269161	Gm11562	predicted gene 11562	2.442502	2.953407	3.15406	3.143453	3.47078	3.118603
17269181	Gm11563	predicted gene 11563	2.324838	2.99395	4.327759	3.24107	3.447594	2.561182
17269250	Gm11564	predicted gene 11564	7.698994	7.698994	9.171578	7.698994	5.799707	10.86842

17256328	Gm11565	predicted gene 11565	3.250812	2.398163	3.134836	3.017701	3.464339	2.888379
17256317	Gm11567	predicted gene 11567	2.935121	2.781803	3.214914	2.431674	2.904359	3.61173
17256296	Gm11568	predicted gene 11568	3.942252	2.501251	2.290782	2.830074	2.900879	2.998747
17269237	Gm11569	predicted gene 11569	4.06614	3.48143	4.063802	3.575587	3.733607	3.123492
17256338	Gm11571	predicted gene 11571	5.139792	5.382625	5.564261	6.095776	6.11906	4.293666
17268441	Gm11574	predicted gene 11574 [Source:MGI Symbol;Acc:MGI:3705166]	11.96966	9.672575	9.386973	10.50504	9.705483	10.09053
17223643	Gm11579	predicted gene 11579 [Source:MGI Symbol;Acc:MGI:3650510]	2.797465	3.164856	2.902387	3.084101	3.168236	5.292246
17255755	Gm11583	predicted gene 11583 [Source:MGI Symbol;Acc:MGI:3705153]	25.20276	23.0667	26.8824	25.36118	22.54757	28.76116
17270060	Gm11586	predicted gene 11586	29.48256	29.48256	34.94151	28.21284	29.2104	30.64246
17213499	Gm11587	predicted gene 11587 [Source:MGI Symbol;Acc:MGI:3652110]	3.136314	2.748853	2.620932	2.684177	2.982537	2.345637
17213509	Gm11588	predicted gene 11588 [Source:MGI Symbol;Acc:MGI:3650951]	2.642549	3.024863	2.375284	2.968142	2.940293	2.10169
17255759	Gm11592	predicted gene 11592	5.352563	5.655129	4.067042	6.157864	5.952972	4.628357
17269220	Gm11595	predicted gene 11595	2.212105	2.361854	4.350014	2.864255	3.289641	3.386089
17269233	Gm11596	predicted gene 11596	5.67917	5.292319	4.176719	4.886969	4.649224	5.304328
17223717	Gm11608	predicted gene 11608	4.515294	3.850084	3.544372	4.107897	3.968324	4.107897
17255819	Gm11612	predicted gene 11612 [Source:MGI Symbol;Acc:MGI:3651820]	14.01102	12.10189	12.7741	12.6632	13.82656	18.7534
17268576	Gm11613	predicted gene 11613 [Source:MGI Symbol;Acc:MGI:3651819]	5.212516	5.407418	4.357045	5.551163	5.336227	4.676276
17255871	Gm11614	predicted gene 11614 [Source:MGI Symbol;Acc:MGI:3651813]	4.86008	3.442436	3.138075	3.263862	3.207146	2.849621
17269836	Gm11615	predicted gene 11615 [Source:MGI Symbol;Acc:MGI:3652280]	4.20927	4.348687	4.20927	4.300427	3.713681	4.560747
17269908	Gm11626	predicted gene 11626 [Source:MGI Symbol;Acc:MGI:3699632]	14.57749	15.50015	14.57749	13.94146	14.57749	14.57749
17270286	Gm11627	predicted gene 11627	3.297037	3.167051	3.031664	3.448885	3.297037	3.297037
17255904	Gm11630	predicted gene 11630 [Source:MGI Symbol;Acc:MGI:3650475]	3.287316	3.989284	3.798843	4.279286	4.352065	4.100017
17269948	Gm11634	predicted gene 11634	8.910461	10.46536	10.1025	10.1025	11.87224	11.63734
17270294	Gm11636	predicted gene 11636 [Source:MGI Symbol;Acc:MGI:3649522]	3.796553	3.54506	3.218481	4.043936	3.369746	3.661107
17257398	Gm11638	predicted gene 11638 [Source:MGI Symbol;Acc:MGI:3651788]	3.883641	4.085096	3.724612	4.146891	5.193726	4.707268
17257403	Gm11640	predicted gene 11640 [Source:MGI Symbol;Acc:MGI:3649238]	3.043202	2.031845	2.357618	3.005344	2.892406	3.277873
17270721	Gm11646	predicted gene 11646 [Source:MGI Symbol;Acc:MGI:3649612]	7.378615	9.182646	14.41181	8.515906	12.54873	9.54179
17257730	Gm11650	predicted gene 11650 [Source:MGI Symbol;Acc:MGI:3650132]	9.178383	11.29537	10.36149	13.77614	11.35669	11.40346
17270715	Gm11651	predicted gene 11651	2.906776	3.68486	3.642856	4.421972	2.95484	3.524797
17257738	Gm11655	predicted gene 11655 [Source:MGI Symbol;Acc:MGI:3650998]	4.893918	5.438165	5.879323	6.219334	7.696326	4.649869
17257756	Gm11656	predicted gene 11656 [Source:MGI Symbol;Acc:MGI:3651000]	2.522003	2.659804	3.079252	2.615796	2.883398	2.460821
17257735	Gm11657	predicted gene 11657 [Source:MGI Symbol;Acc:MGI:3649241]	6.171709	5.780603	6.497929	6.171709	6.171709	5.661875
17270609	Gm11659	predicted gene 11659 [Source:MGI Symbol;Acc:MGI:3649220]	3.150586	3.535269	3.120636	3.709591	3.904262	3.889299
17270673	Gm11665	predicted gene 11665	2.379354	2.479604	2.755263	2.545131	2.745883	3.660016
17270676	Gm11665	predicted gene 11665	2.830153	2.618898	2.840707	2.597637	2.542116	2.951197
17271197	Gm11670	predicted gene 11670 [Source:MGI Symbol;Acc:MGI:3649868]	9.859811	13.87591	15.06094	12.59937	10.56697	14.78805
17270727	Gm11672	predicted gene 11672 [Source:MGI Symbol;Acc:MGI:3651419]	2.448741	2.690492	2.532636	2.67402	2.342161	3.797421
17271534	Gm11674	predicted gene 11674 [Source:MGI Symbol;Acc:MGI:3650971]	2.642256	2.369282	2.566816	2.900497	2.668705	2.623531
17271531	Gm11677	predicted gene 11677	12.51659	9.9308	7.507331	11.74521	7.474101	8.800222
17257942	Gm11680	predicted gene 11680 [Source:MGI Symbol;Acc:MGI:3650354]	2.491509	3.053936	2.870118	2.946087	3.856922	3.356017
17271568	Gm11681	predicted gene 11681	11.86771	15.23165	13.70243	11.76468	11.39051	12.90215
17257924	Gm11682	predicted gene 11682	5.024987	5.848068	5.963586	4.820157	6.581836	5.119277
17257896	Gm11683	predicted gene 11683	5.646043	3.95391	5.88185	6.45291	6.694656	4.274483
17257893	Gm11684	predicted gene 11684 [Source:MGI Symbol;Acc:MGI:3651604]	16.23595	16.23595	17.3246	15.75295	17.8527	17.8527
17377009	Gm11686	predicted gene 11686 [Source:MGI Symbol;Acc:MGI:3652311]	2.620369	2.661925	2.900698	3.253855	3.315186	2.977992
17271692	Gm11690	predicted gene 11690	15.89411	18.26639	19.20127	17.39149	13.11576	16.03464
17258012	Gm11691	predicted gene 11691	4.052044	3.474896	3.016028	3.766958	3.061545	3.51209
17547598	Gm11694	predicted gene 11694 [Source:MGI Symbol;Acc:MGI:3650243]	2.361642	2.09955	2.435402	2.592816	2.458673	2.483784
17271907	Gm11695	predicted gene 11695	27.24142	89.11646	192.7517	87.14041	116.9607	81.82501
17271237	Gm11696	predicted gene 11696	5.534335	6.515564	6.150825	5.422845	7.30589	6.87829
17257903	Gm11697	predicted gene 11697 [Source:MGI Symbol;Acc:MGI:3650098]	12.68991	8.56599	9.20208	8.476978	7.669752	10.21601
17271001	Gm11706	predicted gene 11706 [Source:MGI Symbol;Acc:MGI:3651837]	8.933404	6.961653	9.849292	10.57576	7.733867	9.125351
17271030	Gm11707	predicted gene 11707 [Source:MGI Symbol;Acc:MGI:3651836]	2.041529	2.456629	2.128298	2.395688	2.264951	2.653341
17271741	Gm11709	predicted gene 11709	8.068833	5.300432	7.258342	5.237942	6.445222	5.207172
17271751	Gm11710	predicted gene 11710	35.53946	35.8532	37.14716	36.73975	46.53938	41.3897
17271757	Gm11711	predicted gene 11711	31.62777	32.86249	34.04828	33.67509	33.47379	34.29271
17257654	Gm11712	predicted gene 11712 [Source:MGI Symbol;Acc:MGI:3652138]	8.782368	10.39956	7.74143	9.553901	8.390519	12.69616
17257657	Gm11713	predicted gene 11713	6.714035	8.894355	7.944189	8.590904	6.39733	8.4088
17257660	Gm11714	predicted gene 11714 [Source:MGI Symbol;Acc:MGI:3649214]	4.24131	6.691084	4.072938	5.657756	4.177761	4.497147
17257647	Gm11715	predicted gene 11715	3.363679	3.257375	3.703322	4.21774	3.363679	2.797232
17257651	Gm11716	predicted gene 11716	5.338965	4.895377	5.065975	4.466176	4.681043	4.140629
17257680	Gm11722	predicted gene 11722 [Source:MGI Symbol;Acc:MGI:3649870]	3.548821	2.450031	2.576495	2.604236	2.470803	2.567706
17272587	Gm11723	predicted gene 11723 [Source:MGI Symbol;Acc:MGI:3650120]	6.321294	6.558791	6.208264	7.551431	7.016006	5.938588
17272591	Gm11724	predicted gene 11724 [Source:MGI Symbol;Acc:MGI:3650123]	8.197023	11.06269	12.09794	8.735438	6.700413	8.735438
17258882	Gm11725	predicted gene 11725	8.903916	8.531503	7.86044	8.113025	7.278191	8.056314
17258761	Gm11729	predicted gene 11729	2.947251	2.901844	2.658293	3.529295	3.463042	3.308896
17272544	Gm11730	predicted gene 11730 [Source:MGI Symbol;Acc:MGI:3650941]	4.543393	3.495139	3.873318	4.175879	4.441509	5.7784
17258704	Gm11731	predicted gene 11731 [Source:MGI Symbol;Acc:MGI:3650739]	4.671481	4.013219	3.697551	4.20927	4.20927	4.670931
17258765	Gm11732	predicted gene 11732	8.456262	7.526087	7.643217	8.223884	8.863153	7.687796
17258771	Gm11733	predicted gene 11733	6.668952	7.031975	8.467112	7.031975	5.756333	8.254667
17258777	Gm11734	predicted gene 11734	5.819318	4.688837	3.442938	6.81281	3.59689	4.546039
17258899	Gm11738	predicted gene 11738	5.284841	5.988024	5.988024	5.505486	5.120306	6.876383
17258580	Gm11739	predicted gene 11739 [Source:MGI Symbol;Acc:MGI:3650571]	10.1382	5.184914	5.620545	6.171709	4.768019	6.171709
17258615	Gm11744	predicted gene 11744	4.751358	4.885716	5.03852	4.553008	4.800984	4.875364
17272810	Gm11747	predicted gene 11747	4.654286	6.069762	5.22717	4.935137	5.27251	4.707072
17272814	Gm11748	predicted gene 11748 [Source:MGI Symbol;Acc:MGI:3704384]	4.951103	5.952683	5.582533	5.42811	5.349522	4.960034
17258930	Gm11750	predicted gene 11750 [Source:MGI Symbol;Acc:MGI:3650640]	7.120853	7.211153	4.821059	5.678382	7.69814	7.062065
17414846	Gm11751	predicted gene 11751 [Source:MGI Symbol;Acc:MGI:3650096]	2.99059	3.263862	2.943372	3.263862	3.156662	3.263862
17258961	Gm11752	predicted gene 11752 [Source:MGI Symbol;Acc:MGI:3651700]	10.96071	13.39423	13.39423	13.98124	11.64735	16.30441
17272892	Gm11753	predicted gene 11753	4.681043	4.795097	5.844259	4.870062	4.957765	4.747836
17272866	Gm11754	predicted gene 11754	6.492707	6.304404	5.562757	5.165113	5.983792	6.423546
17272870	Gm11755	predicted gene 11755	6.385059	7.875273	6.907052	9.066087	8.849832	7.051401
17426613	Gm11756	predicted gene 11756	7.120112	6.90217	7.013283	7.413629	7.019606	6.914189
17426597	Gm11757	predicted gene 11757	6.890076	7.080788	6.90217	6.775418	6.460429	6.685799

17426589	Gm11758	predicted gene 11758	6.827512	7.287379	6.950612	7.019071	6.712639	6.601116
17426605	Gm11758	predicted gene 11758	6.827512	7.287379	6.950612	7.019071	6.712639	6.601116
17259172	Gm11762	predicted gene 11762	2.728693	2.829447	3.74579	3.168764	2.700694	3.513673
17259232	Gm11766	predicted gene 11766 [Source:MGI Symbol;Acc:MGI:3650673]	8.239061	9.606827	9.488697	9.365721	9.606827	9.492578
17259271	Gm11769	predicted gene 11769	7.217904	6.287864	7.217904	7.217904	7.903984	7.169189
17273053	Gm11770	predicted gene 11770	41.80055	47.48171	45.13517	43.95849	34.71146	36.41902
17259531	Gm11773	predicted gene 11773 [Source:MGI Symbol;Acc:MGI:3651310]	6.292049	6.063539	6.992896	7.553053	6.813519	7.169189
17259546	Gm11775	predicted gene 11775	3.770014	2.961689	2.928131	2.605843	2.907058	3.845417
17423047	Gm11782	predicted gene 11782 [Source:MGI Symbol;Acc:MGI:3651017]	2.309915	2.67402	2.072396	3.008762	2.533427	3.008762
17411492	Gm11783	predicted gene 11783	2.798764	3.28841	2.930019	3.23176	3.359042	3.21605
17548289	Gm11783	predicted gene 11783	9.048571	10.66876	8.895958	12.42109	9.402667	10.66876
17422949	Gm11787	predicted gene 11787	10.87218	9.79829	9.824337	10.62809	9.310251	9.79829
17273135	Gm11788	predicted gene 11788	9.856804	9.182646	12.28427	12.36054	11.58953	11.58953
17273140	Gm11788	predicted gene 11788	14.22198	22.06152	20.61496	19.24978	12.48644	18.64194
17273144	Gm11789	predicted gene 11789 [Source:MGI Symbol;Acc:MGI:3650495]	46.65468	56.81382	45.88784	51.75298	43.79305	38.66483
17411630	Gm11795	predicted gene 11795 [Source:MGI Symbol;Acc:MGI:3650854]	3.786356	2.521077	3.666491	2.95527	2.6316	3.441625
17411621	Gm11802	predicted gene 11802	4.680524	3.972976	4.864394	4.454273	4.039425	4.67323
17548330	Gm11810	predicted gene 11810 [Source:MGI Symbol;Acc:MGI:3652275]	101.0895	107.675	109.7084	111.1928	116.7177	119.5127
17423205	Gm11815	predicted gene 11815 [Source:MGI Symbol;Acc:MGI:3650511]	16.93035	19.64268	24.9039	19.17294	19.13365	22.32735
17423136	Gm11816	predicted gene 11816	2.67402	2.625915	2.909982	2.732554	2.732554	3.072408
17423141	Gm11817	predicted gene 11817 [Source:MGI Symbol;Acc:MGI:3650295]	4.625278	2.562727	2.338802	2.675039	2.804143	2.835258
17411913	Gm11820	predicted gene 11820	4.134549	2.239428	2.045509	2.333297	2.936057	2.463638
17411783	Gm11821	predicted gene 11821	5.366457	7.834602	7.439522	9.174439	8.344236	5.419463
17423386	Gm11823	predicted gene 11823	2.898045	3.33813	2.910748	3.278896	4.731147	3.355087
17411744	Gm11827	predicted gene 11827	6.663037	7.402589	6.424704	8.175461	6.937856	8.966283
17423314	Gm11831	predicted gene 11831 [Source:MGI Symbol;Acc:MGI:3650069]	6.699531	7.380456	6.810685	6.920998	6.054257	6.257386
17423317	Gm11832	predicted gene 11832	4.522781	4.122816	3.115341	6.551204	3.263862	5.385648
17411948	Gm11837	predicted gene 11837	5.551663	5.763559	7.295566	4.755246	7.382298	6.013104
17411881	Gm11842	predicted gene 11842 [Source:MGI Symbol;Acc:MGI:3651165]	3.512237	2.868348	3.384189	3.381023	3.499479	2.977827
17411955	Gm11844	predicted gene 11844	16.68026	10.56646	10.1025	15.35014	12.78252	18.57474
17411901	Gm11846	predicted gene 11846 [Source:MGI Symbol;Acc:MGI:3651794]	3.519182	4.478948	3.753353	3.703322	3.668118	3.779119
17412009	Gm11861	predicted gene 11861 [Source:MGI Symbol;Acc:MGI:3650457]	2.953407	2.971621	2.953407	2.828447	3.297745	2.953407
17412033	Gm11867	predicted gene 11867 [Source:MGI Symbol;Acc:MGI:3650506]	2.598412	2.685257	2.588273	2.595999	3.262324	2.465688
17412115	Gm11872	predicted gene 11872 [Source:MGI Symbol;Acc:MGI:3649333]	5.339631	7.302903	4.86008	5.339631	5.618403	4.847027
17412231	Gm11884	predicted gene 11884	2.525694	2.019405	1.88638	2.496384	2.298926	2.634922
17548334	Gm11893	predicted gene 11893 [Source:MGI Symbol;Acc:MGI:3651418]	559.4944	478.5348	414.1645	439.3692	376.029	433.6418
17412269	Gm11899	predicted gene 11899 [Source:MGI Symbol;Acc:MGI:3649624]	7.090169	5.75059	5.698689	5.600633	4.661648	6.164612
17423732	Gm11915	predicted gene 11915 [Source:MGI Symbol;Acc:MGI:3650126]	3.624671	4.404353	4.980429	5.821093	4.803946	5.56106
17412327	Gm11916	predicted gene 11916 [Source:MGI Symbol;Acc:MGI:3650125]	2.406006	2.18277	2.351498	1.987714	2.056399	2.450727
17412323	Gm11917	predicted gene 11917	3.339703	3.040988	3.280828	3.69074	3.339703	3.581813
17412330	Gm11924	predicted gene 11924 [Source:MGI Symbol;Acc:MGI:3649863]	3.019857	3.203147	3.888409	2.484439	3.15999	3.380977
17423799	Gm11934	predicted gene 11934 [Source:MGI Symbol;Acc:MGI:3649212]	3.655934	2.884038	3.176791	3.413567	2.992912	2.764694
17269156	Gm11937	predicted gene 11937	10.91243	26.93724	26.56104	20.96048	21.12852	19.84385
17269153	Gm11938	predicted gene 11938	7.115179	6.992896	7.77186	9.692709	6.992896	7.29314
17269134	Gm11939	predicted gene 11939 [Source:MGI Symbol;Acc:MGI:3651443]	4.306244	4.533798	4.364995	4.282965	3.438311	4.370051
17256273	Gm11940	predicted gene 11940 [Source:MGI Symbol;Acc:MGI:3702271]	7.379945	5.151919	5.632167	5.016287	5.458407	7.111185
17246569	Gm11944	predicted gene 11944	5.780603	6.060891	5.811457	5.780603	5.343444	7.080788
17246624	Gm11946	predicted gene 11946	5.154884	4.7407	4.884192	3.955774	4.313952	4.272262
17259792	Gm11948	predicted gene 11948 [Source:MGI Symbol;Acc:MGI:3650068]	2.720911	2.838443	2.67402	2.96516	3.065516	2.377359
17547565	Gm11954	predicted gene 11954 [Source:MGI Symbol;Acc:MGI:3649565]	108.0152	105.359	115.3281	96.66019	102.4979	100.7719
17246822	Gm11958	predicted gene 11958	4.229714	5.979977	4.647678	4.594889	4.138965	5.030877
17246818	Gm11959	predicted gene 11959	2.953407	3.358111	2.916726	2.953407	2.973949	2.606909
17260016	Gm11961	predicted gene 11961	3.086646	3.118387	2.500315	2.918203	2.74912	3.145916
17246963	Gm11963	predicted gene 11963	14.58648	11.33875	15.62452	17.13934	19.10595	22.21019
17547567	Gm11964	predicted gene 11964 [Source:MGI Symbol;Acc:MGI:3652241]	53.23267	49.22502	57.60372	54.22777	46.64531	56.56705
17247064	Gm11967	predicted gene 11967	5.760204	6.713244	7.043878	5.905299	8.82747	6.939203
17247148	Gm11973	predicted gene 11973 [Source:MGI Symbol;Acc:MGI:3650058]	4.20927	4.20927	4.236345	4.839339	4.46695	4.344108
17260396	Gm11974	predicted gene 11974	7.93973	17.13531	11.26277	13.30641	13.62571	11.49082
17260453	Gm11978	predicted gene 11978	10.91969	9.346525	8.023657	11.28379	7.647722	7.987478
17260466	Gm11979	predicted gene 11979 [Source:MGI Symbol;Acc:MGI:3650694]	11.93635	7.767391	14.04573	16.21885	16.95454	15.66258
17260460	Gm11981	predicted gene 11981	6.060177	5.230758	4.782716	4.847363	5.0276	5.199994
17260469	Gm11985	predicted gene 11985	2.771659	2.299675	2.377442	2.481049	2.87681	2.481049
17260482	Gm11986	predicted gene 11986 [Source:MGI Symbol;Acc:MGI:3650604]	2.953407	2.953407	3.182918	2.953407	2.953407	2.993514
17247215	Gm11992	predicted gene 11992	3.933545	4.681043	3.905886	4.681043	4.396544	5.612525
17247332	Gm11998	predicted gene 11998	2.596755	2.257538	2.817762	3.100713	3.005094	3.684452
17260625	Gm11999	predicted gene 11999	7.378973	6.407182	7.044415	6.943774	6.836889	7.632629
17260630	Gm12000	predicted gene 12000	5.379567	5.255361	4.547678	5.923131	3.415768	3.925809
17247356	Gm12002	predicted gene 12002	4.767028	3.383649	3.928232	4.157511	4.220079	3.596042
17260720	Gm12003	predicted gene 12003 [Source:MGI Symbol;Acc:MGI:3651520]	13.46414	12.99325	14.9335	12.09216	12.79768	10.76928
17247490	Gm12018	predicted gene 12018	3.135857	3.260131	2.720497	3.390481	3.003698	3.62354
17260795	Gm12022	predicted gene 12022	2.236713	2.276646	2.151779	2.304206	2.7874	2.451645
17260798	Gm12023	predicted gene 12023 [Source:MGI Symbol;Acc:MGI:3650977]	3.983537	3.40624	4.784838	4.807111	3.743039	3.783785
17247515	Gm12026	predicted gene 12026 [Source:MGI Symbol;Acc:MGI:3651190]	6.394139	5.506479	6.896622	6.59124	6.353317	6.364954
17260834	Gm12027	predicted gene 12027 [Source:MGI Symbol;Acc:MGI:3651189]	7.386393	7.565943	6.992896	6.153128	6.491492	6.210761
17260850	Gm12031	predicted gene 12031	4.558376	4.380544	6.766266	5.907509	5.120306	4.20927
17247593	Gm12043	predicted gene 12043 [Source:MGI Symbol;Acc:MGI:3651843]	21.68116	24.75709	21.87817	32.05639	24.65298	29.42295
17261001	Gm12050	predicted gene 12050 [Source:MGI Symbol;Acc:MGI:3652080]	3.500789	3.356017	3.033493	3.206612	4.547236	2.973578
17247623	Gm12051	predicted gene 12051 [Source:MGI Symbol;Acc:MGI:3652078]	3.597414	3.81441	3.261487	4.764946	3.729055	3.787485
17247635	Gm12052	predicted gene 12052	3.324022	3.798685	4.716249	4.156013	4.466021	4.213853
17261006	Gm12055	predicted gene 12055 [Source:MGI Symbol;Acc:MGI:3650012]	4.844541	3.774433	3.774433	4.100131	4.039089	4.159961
17247638	Gm12056	predicted gene 12056	7.694566	8.919606	9.155255	6.750994	8.46412	7.814535
17247688	Gm12059	predicted gene 12059	5.767556	6.287864	6.301783	6.146393	7.320844	5.561793
17247845	Gm12061	predicted gene 12061 [Source:MGI Symbol;Acc:MGI:3651662]	4.316225	3.706301	3.106823	3.492209	3.553794	3.100133
17261156	Gm12064	predicted gene 12064 [Source:MGI Symbol;Acc:MGI:3649477]	3.091828	2.833863	2.981979	4.027068	2.605229	3.133272

17261166	Gm12065	predicted gene 12065 [Source:MGI Symbol;Acc:MGI:3649819]	3.264541	3.83852	2.968883	3.694938	2.756122	3.729055
17247867	Gm12066	predicted gene 12066	4.607966	3.385268	3.432382	2.772716	3.325174	2.387962
17247872	Gm12068	predicted gene 12068 [Source:MGI Symbol;Acc:MGI:3649823]	2.322003	2.889721	3.01066	2.781051	3.263862	3.363516
17247882	Gm12069	predicted gene 12069	17.71181	19.99573	15.048	18.35524	17.54916	22.65708
17247929	Gm12081	predicted gene 12081 [Source:MGI Symbol;Acc:MGI:3650553]	3.327895	2.788327	2.332666	2.606909	2.772812	2.606909
17247939	Gm12082	predicted gene 12082	3.841341	3.744855	3.600856	3.895423	6.01961	5.650153
17248102	Gm12088	predicted gene 12088 [Source:MGI Symbol;Acc:MGI:3650337]	3.328933	3.048691	2.67402	2.835867	3.302435	3.519792
17248061	Gm12089	predicted gene 12089 [Source:MGI Symbol;Acc:MGI:3650341]	3.8419	3.765418	4.253351	4.253676	4.677183	3.554631
17261285	Gm12092	predicted gene 12092	3.073282	3.64379	3.061248	3.795	3.218816	3.009117
17248132	Gm12100	predicted gene 12100 [Source:MGI Symbol;Acc:MGI:3651586]	3.02673	2.922433	2.483062	3.664458	3.689947	3.031223
17248209	Gm12107	predicted gene 12107	6.647676	4.469613	4.014054	6.497974	8.090787	5.538057
17261455	Gm12108	predicted gene 12108	6.542536	5.338965	4.261851	5.633104	6.199621	6.153469
17261539	Gm12111	predicted gene 12111	6.870714	6.435267	6.028337	6.213947	6.574176	6.601299
17248351	Gm12114	predicted gene 12114 [Source:MGI Symbol;Acc:MGI:3649344]	3.949226	4.035899	3.009972	3.462322	4.109834	3.462322
17547571	Gm12115	predicted gene 12115 [Source:MGI Symbol;Acc:MGI:3652288]	49.08363	54.18644	63.17492	74.96265	63.71318	83.00866
17248355	Gm12116	predicted gene 12116 [Source:MGI Symbol;Acc:MGI:3652289]	4.403895	4.814947	5.2788	4.983675	5.12375	4.983675
17248367	Gm12119	predicted gene 12119	4.668568	4.79922	5.62842	5.335413	4.863349	4.617878
17248371	Gm12120	predicted gene 12120 [Source:MGI Symbol;Acc:MGI:3651991]	4.181469	3.886334	4.181469	4.150629	4.844541	4.181469
17248406	Gm12121	predicted gene 12121	9.278361	9.887734	6.256692	10.41665	7.751954	9.650408
17248472	Gm12122	predicted gene 12122 [Source:MGI Symbol;Acc:MGI:3650741]	3.315738	2.940986	2.686039	2.606909	2.663974	3.084464
17248488	Gm12126	predicted gene 12126	3.477595	3.256766	3.317347	3.256766	2.858543	3.256766
17248491	Gm12127	predicted gene 12127 [Source:MGI Symbol;Acc:MGI:3652208]	4.76716	6.789946	6.674964	5.708335	6.473922	8.278848
17248496	Gm12128	predicted gene 12128	3.78738	4.20927	4.307498	4.20927	3.870286	2.965386
17261818	Gm12130	predicted gene 12130	3.203569	3.263862	3.224823	3.263862	3.263862	3.475017
17248503	Gm12132	predicted gene 12132	3.019982	2.961771	3.19191	3.19191	3.517889	4.091217
17261936	Gm12144	predicted gene 12144 [Source:MGI Symbol;Acc:MGI:3649685]	3.772315	3.883991	4.346819	4.710989	3.102089	4.865237
17261926	Gm12145	predicted gene 12145 [Source:MGI Symbol;Acc:MGI:3649686]	3.091871	3.436286	4.647678	3.110421	3.576033	3.8156
17261939	Gm12147	predicted gene 12147 [Source:MGI Symbol;Acc:MGI:3649954]	3.688668	3.500934	3.075455	3.663646	2.587807	2.528865
17248589	Gm12148	predicted gene 12148 [Source:MGI Symbol;Acc:MGI:3649953]	10.84404	8.012375	9.664734	11.98311	10.90933	12.57834
17261959	Gm12150	predicted gene 12150 [Source:MGI Symbol;Acc:MGI:3650526]	2.754289	3.54506	3.54506	3.433595	3.170477	3.54506
17248646	Gm12153	predicted gene 12153	7.412961	5.548932	9.198955	6.038667	6.351071	7.073234
17261990	Gm12154	predicted gene 12154 [Source:MGI Symbol;Acc:MGI:3649295]	3.227529	3.419179	3.032295	3.176372	3.252502	3.011287
17262013	Gm12158	predicted gene 12158 [Source:MGI Symbol;Acc:MGI:3649487]	3.408956	3.109387	3.810605	3.603303	4.455045	3.618746
17262016	Gm12159	predicted gene 12159	3.724637	3.227954	3.050826	3.078206	2.966908	3.255321
17248716	Gm12160	predicted gene 12160 [Source:MGI Symbol;Acc:MGI:3649485]	3.407444	2.666838	3.100219	2.621041	3.029354	2.904822
17262020	Gm12162	predicted gene 12162 [Source:MGI Symbol;Acc:MGI:3649481]	4.272054	2.712287	2.639126	3.216608	3.395796	2.89385
17262023	Gm12162	predicted gene 12162 [Source:MGI Symbol;Acc:MGI:3649481]	2.537961	2.546719	3.876568	3.062182	3.326535	3.347537
17262046	Gm12166	predicted gene 12166 [Source:MGI Symbol;Acc:MGI:3650635]	26.70633	17.3186	12.14777	18.7016	19.64745	26.31056
17248790	Gm12167	predicted gene 12167	2.121111	2.725593	2.639309	2.995839	2.822728	2.518981
17248821	Gm12169	predicted gene 12169	3.753353	4.642172	3.915374	3.753353	3.489572	3.356924
17248831	Gm12171	predicted gene 12171	2.851004	2.953407	2.860585	3.703322	4.364027	2.606854
17547573	Gm12174	predicted gene 12174	229.8964	187.194	193.0579	186.9502	202.6814	185.2796
17262218	Gm12185	predicted gene 12185	3.321443	3.535318	4.385466	5.426417	5.574838	4.050837
17262297	Gm12195	predicted gene 12195 [Source:MGI Symbol;Acc:MGI:3651100]	8.281775	15.0695	13.48721	13.11595	11.23556	15.05624
17262381	Gm12198	predicted gene 12198 [Source:MGI Symbol;Acc:MGI:3650407]	16.73643	19.61615	21.9526	20.30172	14.8842	19.06494
17262384	Gm12199	predicted gene 12199 [Source:MGI Symbol;Acc:MGI:3650414]	8.223941	8.223941	8.179088	8.223941	8.094265	8.125688
17262387	Gm12200	predicted gene 12200 [Source:MGI Symbol;Acc:MGI:3650413]	25.96111	20.39891	23.50083	27.03488	22.61425	22.88642
17262586	Gm12205	predicted gene 12205 [Source:MGI Symbol;Acc:MGI:3650261]	3.182764	2.999496	2.670112	3.470059	6.287908	3.644498
17249434	Gm12207	predicted gene 12207 [Source:MGI Symbol;Acc:MGI:3702399]	4.563529	5.063483	5.482371	5.202482	4.330699	4.61935
17262662	Gm12212	predicted gene 12212 [Source:MGI Symbol;Acc:MGI:3652263]	9.28782	6.743605	5.878834	6.146649	8.528074	7.199417
17262733	Gm12214	predicted gene 12214 [Source:MGI Symbol;Acc:MGI:3652265]	2.467209	2.420844	2.467209	2.512859	2.196353	2.623586
17262740	Gm12216	predicted gene 12216	4.757719	4.998517	4.757719	4.757719	5.077963	4.886969
17249636	Gm12223	predicted gene 12223	5.163252	5.386134	5.120306	5.386134	4.306065	5.459277
17262830	Gm12224	predicted gene 12224 [Source:MGI Symbol;Acc:MGI:3651369]	2.46403	2.752877	2.714676	2.679251	2.784368	3.319693
17249769	Gm12227	predicted gene 12227	3.119425	2.812728	2.717745	2.792118	3.235458	3.41726
17249853	Gm12236	predicted gene 12236 [Source:MGI Symbol;Acc:MGI:3651548]	6.713569	5.67433	5.620818	6.135794	9.614688	5.661758
17249851	Gm12238	predicted gene 12238	30.87313	33.21355	26.28923	31.29434	42.09742	39.6712
17249895	Gm12243	predicted gene 12243 [Source:MGI Symbol;Acc:MGI:3649716]	3.173291	2.435132	2.851004	2.304206	3.015485	2.517026
17249962	Gm12246	predicted gene 12246 [Source:MGI Symbol;Acc:MGI:3651154]	3.364565	3.339703	3.339703	3.559785	3.241317	3.664535
17263087	Gm12247	predicted gene 12247	24.36114	20.27598	21.89835	25.36118	21.55305	24.36114
17263073	Gm12248	predicted gene 12248	5.433343	5.533223	5.373232	5.767316	5.64346	4.673748
17263070	Gm12249	predicted gene 12249 [Source:MGI Symbol;Acc:MGI:3649298]	4.679486	5.462798	5.383372	6.171709	4.976978	6.347815
17249977	Gm12250	predicted gene 12250	10.26392	7.360532	7.697447	7.977021	10.26392	10.13813
17250063	Gm12253	predicted gene 12253	2.62026	2.572141	2.980016	2.872685	2.452767	2.56808
17250141	Gm12256	predicted gene 12256 [Source:MGI Symbol;Acc:MGI:3649604]	9.153985	10.94447	12.40724	13.37846	12.92399	11.79244
17250145	Gm12257	predicted gene 12257	4.488334	5.750391	5.168157	5.147921	4.908152	5.457953
17250126	Gm12259	predicted gene 12259 [Source:MGI Symbol;Acc:MGI:3649826]	2.711253	2.460139	2.453685	2.956213	2.596647	2.615161
17263456	Gm12264	predicted gene 12264 [Source:MGI Symbol;Acc:MGI:3702404]	10.9814	7.935274	9.314963	8.976917	8.250949	8.549085
17250341	Gm12265	predicted gene 12265 [Source:MGI Symbol;Acc:MGI:3651913]	8.553708	17.1523	16.38575	17.467	17.467	17.467
17547630	Gm12271	predicted gene 12271	2.935324	2.083487	2.953407	2.953407	2.427397	2.953407
17250611	Gm12272	predicted gene 12272 [Source:MGI Symbol;Acc:MGI:3650798]	4.34914	4.06062	3.459396	4.029358	3.505742	5.254852
17250634	Gm12273	predicted gene 12273	4.926558	4.779932	5.400414	6.330415	4.446376	4.884632
17250695	Gm12278	predicted gene 12278 [Source:MGI Symbol;Acc:MGI:3650383]	3.375942	2.885197	3.185699	3.291214	4.120587	3.291214
17264031	Gm12279	predicted gene 12279	6.745381	5.142322	4.379663	5.197616	4.791841	4.32482
17264039	Gm12280	predicted gene 12280 [Source:MGI Symbol;Acc:MGI:3652011]	6.572216	8.41924	8.187369	6.447948	7.643217	10.33932
17250860	Gm12289	predicted gene 12289	4.663781	3.53674	3.905562	3.716643	3.716643	3.987792
17250867	Gm12290	predicted gene 12290	2.504617	2.659804	3.61178	2.528725	2.5612	2.726859
17264121	Gm12292	predicted gene 12292	3.219017	3.219017	3.219017	3.219017	3.328218	3.07985
17264180	Gm12295	predicted gene 12295	4.520085	4.812211	4.900232	4.939586	6.978515	5.653326
17264290	Gm12298	predicted gene 12298	3.484279	4.421788	4.553545	3.418539	3.753353	4.034668
17250935	Gm12299	predicted gene 12299 [Source:MGI Symbol;Acc:MGI:3652304]	2.686672	3.767219	3.687543	3.586558	2.782767	4.297954
17264320	Gm12300	predicted gene 12300	4.130138	3.627813	3.68578	3.72549	4.285282	3.212864
17264335	Gm12301	predicted gene 12301 [Source:MGI Symbol;Acc:MGI:3650426]	10.29449	12.27159	14.30473	12.7549	16.27979	10.43747
17264418	Gm12305	predicted gene 12305 [Source:MGI Symbol;Acc:MGI:3650835]	15.00603	11.42364	16.19324	12.57765	12.09836	16.79989

17265302	Gm12315	predicted gene 12315	8.179995	10.82136	8.776952	9.297804	8.435713	9.735196
17265282	Gm12316	predicted gene 12316 [Source:MGI Symbol;Acc:MGI:3651760]	8.563853	8.011709	7.040461	8.682678	10.39538	7.71946
17265384	Gm12318	predicted gene 12318	125.5676	185.6254	182.692	198.8091	280.4131	237.8948
17265326	Gm12319	predicted gene 12319 [Source:MGI Symbol;Acc:MGI:3649749]	7.935109	7.618569	7.403513	6.943774	9.239277	8.243974
17265379	Gm12320	predicted gene 12320	4.266668	4.20927	5.26174	5.216528	4.807411	5.060009
17265545	Gm12324	predicted gene 12324 [Source:MGI Symbol;Acc:MGI:3651352]	4.484167	5.926416	4.484167	5.035482	4.612056	4.64185
17265548	Gm12326	predicted gene 12326	5.025754	5.287845	5.287845	5.277702	6.045242	6.258904
17265910	Gm12333	predicted gene 12333	3.379196	3.653122	2.886177	3.236221	2.793318	2.782825
17265973	Gm12336	predicted gene 12336 [Source:MGI Symbol;Acc:MGI:3650722]	3.277396	3.543512	4.835751	4.043067	2.531829	5.517176
17253118	Gm12339	predicted gene 12339	11.05449	10.48228	10.30148	12.66434	10.82564	10.96239
17253125	Gm12339	predicted gene 12339	7.140574	7.676826	7.269973	7.119619	7.852162	7.748516
17256362	Gm12348	predicted gene 12348	4.198517	4.133288	4.133288	3.564303	5.215805	4.41851
17256342	Gm12349	predicted gene 12349	20.60996	18.49649	19.63805	20.43783	18.56341	20.12295
17256030	Gm12352	predicted gene 12352 [Source:MGI Symbol;Acc:MGI:3705172]	15.97573	15.44716	16.8148	17.12284	17.94674	16.02042
17423572	Gm12354	predicted gene 12354	6.883346	7.516182	7.57791	6.171709	10.37191	9.529827
17268842	Gm12355	predicted gene 12355 [Source:MGI Symbol;Acc:MGI:3649769]	38.38977	39.85227	37.06075	46.53196	44.02771	46.31572
17268898	Gm12359	predicted gene 12359	12.30088	9.801958	10.1025	7.832647	8.468227	10.17516
17412689	Gm12364	predicted gene 12364 [Source:MGI Symbol;Acc:MGI:3649785]	4.408018	5.363966	5.688665	6.620316	5.350523	6.044823
17412705	Gm12371	predicted gene 12371	2.4898	2.625842	2.771717	2.897945	2.782767	2.285991
17412708	Gm12374	predicted gene 12374	2.708455	2.953407	3.230573	2.953407	2.597331	3.319233
17423966	Gm12381	predicted gene 12381	6.515699	4.839943	4.297299	4.801649	6.448305	6.007938
17413170	Gm12395	predicted gene 12395 [Source:MGI Symbol;Acc:MGI:3650610]	19.46972	13.06568	11.30869	10.37723	13.19519	8.561479
17424075	Gm12396	predicted gene 12396 [Source:MGI Symbol;Acc:MGI:3649201]	325.8491	375.1854	407.2046	350.206	359.0871	374.1933
17412879	Gm12404	predicted gene 12404	15.91704	11.76941	12.02287	11.81445	10.80158	12.95763
17412907	Gm12405	predicted gene 12405	11.51714	12.10491	14.66647	10.6116	11.56882	9.820592
17412910	Gm12405	predicted gene 12405	3.271313	3.292583	3.516816	3.516816	3.149843	3.602903
17424346	Gm12407	predicted gene 12407	20.28287	20.49457	26.059	19.06401	19.62866	17.76087
17413662	Gm12408	predicted gene 12408	9.193601	7.461522	7.453097	9.107342	5.991345	7.274258
17424968	Gm12409	predicted gene 12409	2.785449	3.444346	3.345102	3.874741	2.29148	2.948641
17413671	Gm12410	predicted gene 12410 [Source:MGI Symbol;Acc:MGI:3650364]	11.42071	12.78039	11.96302	9.398497	9.714636	9.357999
17426926	Gm12414	predicted gene 12414	4.459092	5.468367	4.816249	5.535448	5.125099	7.38368
17415073	Gm12415	predicted gene 12415	3.552242	4.232558	3.648972	3.58375	3.965987	3.703938
17425147	Gm12426	predicted gene 12426	4.528741	4.323381	5.026834	4.261939	3.663519	4.252143
17413942	Gm12434	predicted gene 12434 [Source:MGI Symbol;Acc:MGI:3649453]	3.318382	2.028566	2.020595	2.587592	2.458673	2.99908
17414040	Gm12436	predicted gene 12436	2.916605	4.401789	4.390423	3.98017	4.880334	4.515013
17400881	Gm12440	predicted gene 12440	5.360175	4.364753	5.175112	4.074774	3.686112	5.393082
17425010	Gm12446	predicted gene 12446 [Source:MGI Symbol;Acc:MGI:3651433]	5.805901	5.833008	6.901022	7.077108	6.232539	5.91009
17413399	Gm12472	predicted gene 12472 [Source:MGI Symbol;Acc:MGI:3649439]	6.033813	9.830808	7.345649	8.069336	9.271804	7.105814
17424768	Gm12473	predicted gene 12473 [Source:MGI Symbol;Acc:MGI:3652337]	24.65503	18.73105	26.12429	22.09457	24.04858	22.68426
17400994	Gm12474	predicted gene 12474	8.909534	4.419643	6.667658	7.357777	8.802663	7.984876
17414270	Gm12478	predicted gene 12478 [Source:MGI Symbol;Acc:MGI:3651221]	2.427902	2.481049	2.241369	2.744056	2.481049	2.148813
17414273	Gm12480	predicted gene 12480 [Source:MGI Symbol;Acc:MGI:3651952]	2.706147	2.424471	2.44506	2.568596	3.432858	2.654997
17425382	Gm12484	predicted gene 12484 [Source:MGI Symbol;Acc:MGI:3651626]	5.397271	4.29081	4.340677	4.845213	4.916222	5.88801
17413570	Gm12493	predicted gene 12493 [Source:MGI Symbol;Acc:MGI:3651194]	6.442765	4.728983	4.811144	5.132494	4.347361	5.897322
17425371	Gm12496	predicted gene 12496	5.750909	5.211613	5.866012	5.257803	7.482135	5.241646
17409166	Gm12498	predicted gene 12498 [Source:MGI Symbol;Acc:MGI:3650533]	3.39801	3.062691	3.463258	3.318428	2.849562	3.463258
17401625	Gm12500	predicted gene 12500	7.687956	9.187612	8.862539	9.737018	8.062962	8.420524
17413523	Gm12503	predicted gene 12503	6.992896	6.1332	8.186859	6.992896	6.992896	7.589001
17425391	Gm12505	predicted gene 12505	8.525356	9.672441	9.488237	8.116625	9.085337	9.488237
17414321	Gm12506	predicted gene 12506	2.522353	3.168632	2.865506	3.356017	2.840943	3.253291
17414299	Gm12508	predicted gene 12508 [Source:MGI Symbol;Acc:MGI:3651359]	34.14163	32.01886	59.97752	38.0338	36.22767	40.9792
17425396	Gm12510	predicted gene 12510 [Source:MGI Symbol;Acc:MGI:3651360]	2.598033	2.900839	3.13751	2.674576	2.927096	2.81228
17414318	Gm12511	predicted gene 12511	4.621048	4.824335	5.326803	5.823433	5.688231	5.364263
17425384	Gm12514	predicted gene 12514	7.430195	6.995077	6.106518	8.42683	5.264914	5.46651
17425408	Gm12519	predicted gene 12519	3.816314	3.795369	5.255871	4.743428	4.360429	5.727359
17409280	Gm12522	predicted gene 12522	5.551432	5.604944	5.068364	5.03866	5.583927	5.023455
17409277	Gm12523	predicted gene 12523 [Source:MGI Symbol;Acc:MGI:3649602]	6.748936	4.599924	5.478231	4.586965	6.539	6.368528
17401647	Gm12524	predicted gene 12524 [Source:MGI Symbol;Acc:MGI:3651331]	3.47054	5.286856	4.082831	4.362424	3.729055	3.95391
17401696	Gm12525	predicted gene 12525 [Source:MGI Symbol;Acc:MGI:3649596]	2.527656	2.620968	2.359302	2.278304	2.526395	2.649206
17425606	Gm12526	predicted gene 12526 [Source:MGI Symbol;Acc:MGI:3651778]	3.548969	5.929868	4.654899	4.874249	4.443511	4.848001
17414342	Gm12530	predicted gene 12530	3.335285	3.854036	3.339703	3.316772	2.862489	3.621055
17404877	Gm12531	predicted gene 12531 [Source:MGI Symbol;Acc:MGI:3650531]	3.432691	2.759582	2.728825	4.148989	3.673487	2.583577
17397111	Gm12532	predicted gene 12532	3.462322	3.664179	2.32458	2.610471	2.745845	2.953407
17397117	Gm12534	predicted gene 12534 [Source:MGI Symbol;Acc:MGI:3650303]	3.978432	3.837535	3.558502	4.31458	3.837535	3.837535
17414348	Gm12536	predicted gene 12536	18.93062	16.3439	16.43273	18.75496	11.48453	16.43273
17425603	Gm12538	predicted gene 12538 [Source:MGI Symbol;Acc:MGI:3650715]	12.0729	14.79718	9.258125	12.20601	15.72927	12.28103
17425867	Gm12542	predicted gene 12542 [Source:MGI Symbol;Acc:MGI:3651424]	6.058035	5.249536	4.962028	7.397357	4.858631	5.851798
17249277	Gm12569	predicted gene 12569	5.64616	5.261521	4.374112	5.539516	5.485222	4.928334
17266975	Gm12576	predicted gene 12576	4.805545	6.876717	6.690249	7.042609	6.690249	6.690249
17425703	Gm12580	predicted gene 12580 [Source:MGI Symbol;Acc:MGI:3651872]	5.018339	4.827245	5.805056	7.093561	6.513667	5.981635
17259689	Gm12589	predicted gene 12589	3.629674	3.932427	4.117675	3.745167	3.143888	3.33369
17273562	Gm12590	predicted gene 12590 [Source:MGI Symbol;Acc:MGI:3650370]	85.29859	99.33912	147.2119	115.3049	73.13905	107.464
17259760	Gm12592	predicted gene 12592 [Source:MGI Symbol;Acc:MGI:3649892]	5.793479	6.317221	6.512223	6.146734	5.733873	6.517777
17425813	Gm12594	predicted gene 12594 [Source:MGI Symbol;Acc:MGI:3649447]	2.957586	3.571921	2.739989	3.135814	3.407775	3.38318
17414450	Gm12596	predicted gene 12596	7.62586	7.427105	7.150083	7.773423	7.542485	6.668859
17415233	Gm12600	predicted gene 12600 [Source:MGI Symbol;Acc:MGI:3651471]	5.568273	3.36256	3.366081	4.793568	3.46602	4.573251
17427133	Gm12602	predicted gene 12602	2.534393	3.063583	2.646563	3.264631	3.232432	2.918081
17415375	Gm12603	predicted gene 12603	2.652421	2.888259	3.746984	3.047952	2.897483	3.173951
17427141	Gm12606	predicted gene 12606	8.189753	8.677022	7.863491	8.480386	12.04348	9.113657
17415391	Gm12610	predicted gene 12610	3.550076	3.729055	3.283376	5.704617	5.295768	5.744256
17415396	Gm12610	predicted gene 12610	5.061202	5.423146	4.303737	5.061202	5.061202	5.973059
17547628	Gm12614	predicted gene 12614	8.517972	9.7535	8.625332	8.51685	7.992407	8.029722
17547626	Gm12622	predicted gene 12622	10.54853	12.41601	10.54707	10.41442	10.29584	10.21162
17415407	Gm12629	predicted gene 12629 [Source:MGI Symbol;Acc:MGI:3650349]	2.539052	2.878965	3.02216	3.568506	3.029417	3.517401

17415236	Gm12631	predicted gene 12631 [Source:MGI Symbol;Acc:MGI:3650969]	4.575216	5.054436	5.570898	5.617507	6.927429	6.105037
17415424	Gm12637	predicted gene 12637 [Source:MGI Symbol;Acc:MGI:3650764]	4.060564	4.354207	3.619399	4.394442	4.010133	4.13389
17427213	Gm12648	predicted gene 12648 [Source:MGI Symbol;Acc:MGI:3649948]	4.312068	3.66131	3.846803	3.358716	6.127464	4.02598
17415437	Gm12657	predicted gene 12657	3.286951	2.321279	2.469125	2.120699	1.864088	2.176364
17262413	Gm12660	predicted gene 12660 [Source:MGI Symbol;Acc:MGI:3650844]	4.183209	3.883991	3.626405	4.025924	5.102485	4.26871
17427197	Gm12666	predicted gene 12666	8.470341	9.408013	8.819641	13.64263	11.84758	10.10747
17415417	Gm12668	predicted gene 12668 [Source:MGI Symbol;Acc:MGI:3651936]	4.432928	4.688707	3.703322	4.051707	5.07114	4.20927
17415421	Gm12670	predicted gene 12670 [Source:MGI Symbol;Acc:MGI:3651682]	2.192216	2.060051	2.05496	2.442333	2.442333	2.690977
17413567	Gm12678	predicted gene 12678	4.033857	4.703354	4.275046	3.437262	3.527583	4.192905
17424904	Gm12679	predicted gene 12679 [Source:MGI Symbol;Acc:MGI:3650924]	5.828966	5.530615	5.390092	4.777712	5.162536	4.0936
17427550	Gm12681	predicted gene 12681 [Source:MGI Symbol;Acc:MGI:3650649]	3.54506	4.015418	3.16354	3.54506	4.880537	3.936354
17427557	Gm12682	predicted gene 12682 [Source:MGI Symbol;Acc:MGI:3650643]	3.502803	4.175416	3.248357	2.826644	3.84747	3.123774
17427554	Gm12688	predicted gene 12688	4.310723	3.433381	3.477836	3.702193	4.214992	3.752859
17415771	Gm12690	predicted gene 12690 [Source:MGI Symbol;Acc:MGI:3650444]	13.1285	15.47288	14.66109	14.18506	13.30485	14.44762
17415496	Gm12694	predicted gene 12694	2.305724	2.061951	3.260764	2.606909	2.697214	3.15826
17427427	Gm12695	predicted gene 12695	3.578066	3.28242	3.349301	3.678455	3.293633	2.795662
17415847	Gm12701	predicted gene 12701	2.88952	3.408838	3.211372	2.710013	2.782767	3.813088
17415832	Gm12702	predicted gene 12702 [Source:MGI Symbol;Acc:MGI:3650874]	3.012309	3.263862	3.472513	3.285425	3.263862	3.6944
17427309	Gm12703	predicted gene 12703 [Source:MGI Symbol;Acc:MGI:3650667]	3.311374	3.196227	3.785044	3.786802	4.742869	3.896206
17548346	Gm12704	predicted gene 12704 [Source:MGI Symbol;Acc:MGI:3650671]	35.88627	40.97807	63.74631	41.99163	42.78075	43.30495
17415741	Gm12705	predicted gene 12705 [Source:MGI Symbol;Acc:MGI:3650670]	3.972673	3.350694	3.350694	3.305435	3.121826	3.350694
17415538	Gm12707	predicted gene 12707	2.917596	4.245134	3.945724	4.20927	4.794698	3.285698
17415529	Gm12708	predicted gene 12708	5.69399	5.207858	6.092693	5.350264	5.818632	5.060921
17427645	Gm12709	predicted gene 12709	2.810253	2.810253	2.900758	2.900658	2.827663	2.599709
17248421	Gm12711	predicted gene 12711 [Source:MGI Symbol;Acc:MGI:3649928]	7.214103	9.89802	7.814535	11.45265	6.047086	8.672633
17250317	Gm12714	predicted gene 12714 [Source:MGI Symbol;Acc:MGI:3651102]	4.12905	4.142639	4.648322	4.370747	5.226807	4.681043
17416154	Gm12718	predicted gene 12718	4.72361	5.370513	4.116591	4.981741	5.484119	4.077628
17427722	Gm12720	predicted gene 12720 [Source:MGI Symbol;Acc:MGI:3651748]	4.404597	5.300432	3.725335	4.584264	4.45523	4.358857
17416243	Gm12724	predicted gene 12724	3.170653	2.963948	2.694355	2.963948	2.963948	3.107684
17427761	Gm12729	predicted gene 12729 [Source:MGI Symbol;Acc:MGI:3649488]	3.575612	3.055927	3.703322	4.20927	3.64518	4.190609
17416248	Gm12730	predicted gene 12730 [Source:MGI Symbol;Acc:MGI:3651116]	2.598412	2.389684	2.598412	2.598412	2.838011	2.553224
17416687	Gm12737	predicted gene 12737 [Source:MGI Symbol;Acc:MGI:3651021]	3.255344	5.073495	4.71638	4.20927	4.173159	4.090168
17548350	Gm12739	predicted gene 12739 [Source:MGI Symbol;Acc:MGI:3651018]	3.703322	3.528634	3.703322	3.703322	3.787275	3.901557
17428082	Gm12743	predicted gene 12743 [Source:MGI Symbol;Acc:MGI:3651449]	3.391539	3.991359	3.891968	3.412999	5.129009	4.033606
17427883	Gm12746	predicted gene 12746 [Source:MGI Symbol;Acc:MGI:3652135]	3.023752	3.63602	3.339703	3.255886	3.862647	3.011015
17412640	Gm12753	predicted gene 12753 [Source:MGI Symbol;Acc:MGI:3652140]	4.352065	5.050548	5.159925	4.313862	4.450107	4.390545
17489696	Gm12756	predicted gene 12756 [Source:MGI Symbol;Acc:MGI:3649709]	3.602704	3.88418	4.966536	3.703322	5.585669	4.290007
17476690	Gm12758	predicted gene 12758	7.596264	8.683881	7.880898	7.957967	8.407052	8.653357
17489642	Gm12762	predicted gene 12762	10.01826	9.948714	12.07081	8.73677	12.36097	10.01826
17476661	Gm12763	predicted gene 12763	2.2325	2.146491	1.97073	2.161406	2.483148	2.578657
17476695	Gm12764	predicted gene 12764 [Source:MGI Symbol;Acc:MGI:3649980]	4.266225	4.511071	3.670967	3.276238	4.976426	4.062844
17476698	Gm12766	predicted gene 12766 [Source:MGI Symbol;Acc:MGI:3652050]	2.909397	3.275579	4.695895	5.502358	2.963907	3.960438
17476705	Gm12781	predicted gene 12781	15.86692	25.22776	22.08477	24.51665	30.83699	27.75893
17476702	Gm12784	predicted gene 12784	5.338965	4.20927	4.20927	4.292744	5.417773	4.034249
17427886	Gm12786	predicted gene 12786	4.436094	4.170701	4.117218	3.96269	4.859911	4.395661
17416014	Gm12789	predicted gene 12789	2.604741	2.82445	2.440793	2.398729	2.208796	3.226589
17427641	Gm12790	predicted gene 12790 [Source:MGI Symbol;Acc:MGI:3649398]	5.205043	6.037788	4.857217	7.802465	4.52849	4.853145
17416005	Gm12794	predicted gene 12794	3.344754	2.313874	3.339703	2.431135	2.719271	3.506958
17416009	Gm12794	predicted gene 12794	2.343037	1.898013	3.339703	3.249573	1.926097	2.689243
17427619	Gm12796	predicted gene 12796 [Source:MGI Symbol;Acc:MGI:3649207]	6.135156	6.720041	5.96404	5.727399	7.985319	5.896546
17415928	Gm12798	predicted gene 12798 [Source:MGI Symbol;Acc:MGI:3651483]	6.139069	9.182646	6.139069	7.120408	5.510144	7.558815
17407742	Gm128	predicted gene 128	6.037536	6.282288	5.08233	6.239541	5.087617	6.356797
17415919	Gm12801	predicted gene 12801 [Source:MGI Symbol;Acc:MGI:3652119]	3.729055	3.572738	3.729055	3.729055	3.576355	3.752911
17416422	Gm12802	predicted gene 12802	4.01539	4.335144	4.188896	3.858847	3.462346	4.315088
17416893	Gm12811	predicted gene 12811 [Source:MGI Symbol;Acc:MGI:3650795]	6.473339	7.248186	7.35222	7.070832	7.879805	9.29046
17417059	Gm12814	predicted gene 12814	2.885497	2.612299	2.255645	2.973496	2.188921	2.953407
17548352	Gm12816	predicted gene 12816 [Source:MGI Symbol;Acc:MGI:3649628]	172.8911	178.4159	181.4867	165.5772	179.8487	183.6939
17417078	Gm12823	predicted gene 12823 [Source:MGI Symbol;Acc:MGI:3651665]	2.788482	2.307195	2.663402	2.590014	2.597313	2.630761
17417673	Gm12828	predicted gene 12828 [Source:MGI Symbol;Acc:MGI:3650145]	4.209708	4.020932	3.740238	3.749765	5.116474	4.937737
17417099	Gm12829	predicted gene 12829	4.300487	3.129951	3.577595	4.069693	2.997501	4.658611
17417102	Gm12830	predicted gene 12830	3.684809	3.776919	3.540321	5.274813	4.471999	3.802267
17417699	Gm12840	predicted gene 12840 [Source:MGI Symbol;Acc:MGI:3650917]	50.0407	80.96127	112.6786	62.24733	48.50293	62.50415
17417729	Gm12841	predicted gene 12841 [Source:MGI Symbol;Acc:MGI:3651048]	9.401689	8.568662	8.718379	7.936044	11.01983	10.7292
17429270	Gm12853	predicted gene 12853	4.077317	3.994182	3.943673	4.874317	5.857317	4.428721
17418030	Gm12860	predicted gene 12860 [Source:MGI Symbol;Acc:MGI:3651651]	5.901248	4.693844	4.089544	4.220196	5.155134	4.9152
17417837	Gm12861	predicted gene 12861 [Source:MGI Symbol;Acc:MGI:3651891]	3.115319	2.363295	2.186859	2.427195	3.202215	2.414124
17429298	Gm12862	predicted gene 12862 [Source:MGI Symbol;Acc:MGI:3651209]	5.545279	4.801649	4.801649	4.040825	5.04495	5.458218
17429292	Gm12863	predicted gene 12863	3.489402	4.079578	3.9138	3.734435	3.537549	3.374865
17429295	Gm12865	predicted gene 12865	3.450104	5.35453	3.875386	6.515609	5.08871	4.761381
17429301	Gm12866	predicted gene 12866	7.446229	7.241457	7.448655	7.61081	7.23965	7.617407
17429304	Gm12867	predicted gene 12867	4.055023	4.151895	4.777249	4.209124	5.207894	3.625148
17429307	Gm12868	predicted gene 12868 [Source:MGI Symbol;Acc:MGI:3651004]	6.285424	6.671217	8.125744	7.597054	7.826406	7.668423
17427936	Gm12869	predicted gene 12869 [Source:MGI Symbol;Acc:MGI:3649409]	5.203203	4.310514	3.655022	4.383612	4.200875	4.420072
17429596	Gm12880	predicted gene 12880	4.221864	3.720225	2.999828	3.682103	3.802874	3.475691
17429591	Gm12886	predicted gene 12886	2.953407	2.28745	2.953407	3.476944	2.953407	4.140687
17429600	Gm12887	predicted gene 12887	2.271917	2.158112	2.382391	2.277104	2.257804	2.12644
17429581	Gm12888	predicted gene 12888	2.639656	2.334996	2.22638	2.974361	2.639656	2.639656
17548303	Gm12891	predicted gene 12891 [Source:MGI Symbol;Acc:MGI:3649646]	45.60247	51.10031	61.20001	68.6839	54.31919	62.49202
17417869	Gm12898	predicted gene 12898 [Source:MGI Symbol;Acc:MGI:3650325]	6.360631	5.106058	6.273454	5.615132	5.017539	5.532571
17429829	Gm12905	predicted gene 12905 [Source:MGI Symbol;Acc:MGI:3702581]	11.17382	13.61042	11.45535	12.07424	11.77455	13.86225
17416533	Gm12907	predicted gene 12907	4.490419	4.235229	3.516353	5.272181	3.849284	4.867564
17414529	Gm12909	predicted gene 12909	31.64202	42.59405	35.65074	47.37388	40.16536	48.1502
17414533	Gm12910	predicted gene 12910 [Source:MGI Symbol;Acc:MGI:3651134]	5.456364	3.93439	3.190649	4.529086	3.609327	4.732131
17426429	Gm12911	predicted gene 12911	3.720303	3.709976	3.170543	3.290051	3.425988	3.565415

17426752	Gm12914	predicted gene 12914 [Source:MGI Symbol;Acc:MGI:3650483]	2.674001	2.526377	2.578604	2.578604	2.272594	2.578604
17429875	Gm12915	predicted gene 12915	6.577549	6.577549	5.72593	7.99069	7.166109	6.743371
17429832	Gm12916	predicted gene 12916	2.899411	3.491991	3.339703	2.813508	4.154572	3.303602
17429650	Gm12923	predicted gene 12923	5.022063	4.804512	3.397586	4.071301	4.20927	4.20927
17418217	Gm12925	predicted gene 12925	3.729055	3.375989	4.20927	4.280561	3.805563	4.295631
17429352	Gm12927	predicted gene 12927	11.58062	12.56405	11.28762	12.31735	11.01151	11.06215
17429918	Gm12930	predicted gene 12930 [Source:MGI Symbol;Acc:MGI:3650529]	3.324022	3.210393	3.650641	4.397001	3.430931	3.820152
17429930	Gm12932	predicted gene 12932 [Source:MGI Symbol;Acc:MGI:3651151]	9.034282	13.15482	10.77757	11.90363	15.13524	11.90363
17418724	Gm12942	predicted gene 12942	12.27405	14.16639	12.75075	13.36243	12.6827	11.43417
17418589	Gm12945	predicted gene 12945 [Source:MGI Symbol;Acc:MGI:3704237]	5.338965	5.682517	6.22205	5.682517	6.493742	6.587404
17430231	Gm12947	predicted gene 12947 [Source:MGI Symbol;Acc:MGI:3649527]	3.287019	4.666432	3.009972	3.715612	3.500934	3.742027
17250698	Gm12949	predicted gene 12949 [Source:MGI Symbol;Acc:MGI:3649756]	2.700338	2.243483	2.792989	3.010431	2.101151	3.797026
17417404	Gm12951	predicted gene 12951 [Source:MGI Symbol;Acc:MGI:3649505]	9.13877	8.212492	8.512365	6.230682	8.050118	9.372994
17417434	Gm12953	predicted gene 12953 [Source:MGI Symbol;Acc:MGI:3649497]	2.679437	2.842795	3.657531	2.563545	2.757498	3.488774
17418854	Gm12958	predicted gene 12958	6.734496	7.698994	7.33161	8.727449	6.516061	8.097632
17430549	Gm12963	predicted gene 12963	14.16865	14.34623	16.00743	16.71464	13.66999	15.42619
17430657	Gm12970	predicted gene 12970	10.64239	10.61292	9.983391	11.50086	14.33828	13.29176
17419265	Gm12971	predicted gene 12971 [Source:MGI Symbol;Acc:MGI:3650041]	8.730595	16.28554	14.22286	13.24559	9.394328	7.549285
17419272	Gm12972	predicted gene 12972 [Source:MGI Symbol;Acc:MGI:3650043]	14.02627	13.37864	15.24622	16.73504	15.21582	14.59073
17419316	Gm12973	predicted gene 12973 [Source:MGI Symbol;Acc:MGI:3650042]	4.046796	3.19871	2.953284	3.615612	3.252931	4.212305
17419648	Gm12974	predicted gene 12974	8.80663	10.45456	12.81152	10.04454	6.650118	10.88342
17418972	Gm12976	predicted gene 12976 [Source:MGI Symbol;Acc:MGI:3650499]	5.225286	4.103344	5.016253	4.898126	4.898126	5.037891
17419653	Gm12977	predicted gene 12977	9.702994	9.475421	9.410361	8.621507	8.848543	10.06859
17430393	Gm12979	predicted gene 12979 [Source:MGI Symbol;Acc:MGI:3651303]	4.01461	4.216775	4.874891	4.102491	4.102491	4.679259
17430875	Gm12981	predicted gene 12981 [Source:MGI Symbol;Acc:MGI:3651528]	9.532932	8.209589	11.27652	10.58266	8.889732	10.05966
17419853	Gm12984	predicted gene 12984	4.585948	4.152068	3.651831	4.509852	3.863129	4.013915
17430722	Gm12992	predicted gene 12992	5.616923	5.835394	4.547993	5.06429	4.142868	5.096299
17417548	Gm12996	predicted gene 12996	8.194182	6.859198	6.583022	7.067402	6.498515	7.067402
17419420	Gm12999	predicted gene 12999 [Source:MGI Symbol;Acc:MGI:3651167]	8.995042	9.657702	9.657702	9.899736	10.61064	10.44217
17431663	Gm13001	predicted gene 13001	5.797175	5.486743	5.591828	5.243827	4.723643	6.657313
17431667	Gm13003	predicted gene 13003	6.565614	6.33138	5.732244	6.33138	5.51343	6.206457
17420011	Gm13008	predicted gene 13008 [Source:MGI Symbol;Acc:MGI:3702670]	9.563243	7.712614	8.534638	14.7468	9.057796	15.12297
17431703	Gm13010	predicted gene 13010	4.349351	6.477468	5.606343	5.042118	5.670909	5.86467
17420827	Gm13016	predicted gene 13016	11.86936	8.960318	8.917628	10.09039	13.35752	11.21992
17420834	Gm13021	predicted gene 13021	15.84483	44.9575	40.42792	26.65511	16.17081	17.94587
17421233	Gm13023	predicted gene 13023	4.34923	2.490335	2.427767	3.1658	2.046091	2.513365
17420841	Gm13025	predicted gene 13025	8.384066	8.140853	8.399363	8.843148	7.878331	7.945896
17420838	Gm13026	predicted gene 13026 [Source:MGI Symbol;Acc:MGI:3650881]	9.902138	9.427074	11.96534	10.29855	8.473042	8.920101
17420820	Gm13027	predicted gene 13027 [Source:MGI Symbol;Acc:MGI:3651912]	21.67124	14.99802	16.20133	14.99802	10.17657	16.52846
17431836	Gm13030	predicted gene 13030	3.374912	2.796263	3.524772	3.120052	2.661298	2.85581
17432077	Gm13031	predicted gene 13031	7.725348	6.906764	9.487908	7.725348	6.606197	7.500673
17420866	Gm13032	predicted gene 13032	4.028325	4.082011	4.021295	3.618571	3.934772	3.975372
17421196	Gm13043	predicted gene 13043	3.615186	5.016739	4.306602	4.26729	4.61711	4.853515
17421203	Gm13043	predicted gene 13043	2.602756	3.009868	3.704452	3.176548	3.392385	3.217923
17421209	Gm13043	predicted gene 13043	3.115168	3.516914	3.974932	3.438359	3.859891	4.098199
17421968	Gm13049	predicted gene 13049	4.6442	3.710645	3.987239	4.6442	3.881166	5.641778
17421396	Gm13051	predicted gene 13051 [Source:MGI Symbol;Acc:MGI:3702694]	2.813839	3.741897	2.549704	3.608002	2.220924	3.037722
17432418	Gm13052	predicted gene 13052 [Source:MGI Symbol;Acc:MGI:3651990]	3.559834	4.181295	3.676594	3.81679	4.386986	4.151147
17432861	Gm13054	predicted gene 13054 [Source:MGI Symbol;Acc:MGI:3702728]	8.51685	7.959291	8.555724	8.51685	8.171721	9.489815
17432145	Gm13055	predicted gene 13055 [Source:MGI Symbol;Acc:MGI:3649999]	4.095813	3.996536	3.727969	4.039117	4.067183	2.953407
17421030	Gm13056	predicted gene 13056	7.974146	10.99145	13.62808	11.87833	9.279262	10.74557
17432458	Gm13058	predicted gene 13058 [Source:MGI Symbol;Acc:MGI:3650002]	3.500862	3.457765	4.854693	4.204196	5.021854	4.865675
17419658	Gm13061	predicted gene 13061 [Source:MGI Symbol;Acc:MGI:3650259]	17.34358	15.21223	14.3753	16.26907	18.02428	17.56218
17421164	Gm13062	predicted gene 13062 [Source:MGI Symbol;Acc:MGI:3649476]	4.801649	4.20927	4.742606	4.286232	5.755934	5.821617
17419371	Gm13063	predicted gene 13063 [Source:MGI Symbol;Acc:MGI:3649474]	13.63488	14.86039	13.5717	13.5717	13.01632	11.773
17433166	Gm13066	predicted gene 13066	4.146258	3.609828	4.081983	4.982363	4.171308	4.699965
17421862	Gm13067	predicted gene 13067	13.91472	13.75601	16.03231	15.21086	12.95422	16.71765
17421850	Gm13068	predicted gene 13068 [Source:MGI Symbol;Acc:MGI:3651967]	3.022537	3.361418	3.69074	2.934328	4.01258	3.79019
17421858	Gm13070	predicted gene 13070	6.171709	6.165295	7.640251	5.386134	7.166705	8.190037
17433231	Gm13073	predicted gene 13073	3.544225	3.991774	3.952978	3.744414	3.653198	3.092299
17421057	Gm13074	predicted gene 13074 [Source:MGI Symbol;Acc:MGI:3651959]	9.788311	10.41665	13.75162	10.41665	10.07599	10.15529
17421068	Gm13075	predicted gene 13075 [Source:MGI Symbol;Acc:MGI:3651958]	14.7942	12.21879	14.7942	14.7942	13.43207	14.7942
17421227	Gm13078	predicted gene 13078	7.201663	6.298464	7.309588	7.683268	6.039713	5.175542
17421222	Gm13083	predicted gene 13083	2.922433	2.945005	2.789681	2.718932	4.239106	2.234853
17432477	Gm13084	predicted gene 13084	3.040081	2.474385	2.716615	3.393255	2.967278	2.693179
17432463	Gm13088	predicted gene 13088	2.373161	1.898013	1.860667	2.083357	4.57658	2.606909
17432470	Gm13089	predicted gene 13089	3.041852	2.038588	2.245287	2.610272	2.009574	2.669094
17422006	Gm13090	predicted gene 13090	5.180495	7.235286	5.197472	7.319373	6.701156	7.422782
17433283	Gm13091	predicted gene 13091 [Source:MGI Symbol;Acc:MGI:3649653]	6.014272	6.370559	7.250347	6.844286	6.000489	6.954998
17433261	Gm13093	predicted gene 13093	3.97653	5.002086	4.485379	4.689779	4.932914	4.32464
17422135	Gm13096	predicted gene 13096 [Source:MGI Symbol;Acc:MGI:3649461]	8.87834	10.04127	8.453859	7.262972	9.378323	11.0479
17421801	Gm13097	predicted gene 13097 [Source:MGI Symbol;Acc:MGI:3701129]	11.6463	15.25616	9.268591	10.93067	13.30605	10.93067
17432503	Gm13101	predicted gene 13101 [Source:MGI Symbol;Acc:MGI:3650204]	11.05518	15.50788	16.12917	7.723474	11.87133	12.20559
17421263	Gm13102	predicted gene 13102	2.663789	2.358762	2.57905	3.035281	2.868089	2.563722
17421242	Gm13103	predicted gene 13103	2.002247	2.094564	1.949548	2.083357	2.058467	2.389055
17433658	Gm13110	predicted gene 13110 [Source:MGI Symbol;Acc:MGI:3651716]	3.351995	3.472392	3.254622	3.827069	3.90827	3.472392
17422398	Gm13111	predicted gene 13111	7.501557	7.972875	6.687236	7.939125	8.153503	8.559521
17422266	Gm13115	predicted gene 13115 [Source:MGI Symbol;Acc:MGI:3649445]	6.171709	6.171709	6.621784	6.784582	6.973728	5.630956
17421290	Gm13119	predicted gene 13119	2.341771	2.34117	1.805239	2.458009	2.022781	2.106153
17421283	Gm13123	predicted gene 13123 [Source:MGI Symbol;Acc:MGI:3651473]	80.14552	68.81828	72.17163	86.44616	78.94303	83.10538
17432572	Gm13124	predicted gene 13124	2.827251	2.840431	2.780472	2.873223	2.840431	3.243812
17432541	Gm13125	predicted gene 13125	2.721308	2.721308	2.721308	2.590158	2.223974	2.345524
17421285	Gm13128	predicted gene 13128	3.487637	2.925554	2.726084	3.087138	3.29409	3.199641
17422389	Gm13133	predicted gene 13133	4.544527	4.614838	4.263948	4.834645	6.387804	4.597087

17422394	Gm13134	predicted gene 13134 [Source:MGI Symbol;Acc:MGI:3650641]	6.171709	6.171709	5.726327	8.247346	5.142536	6.891271
17421476	Gm13139	predicted gene 13139	7.441688	3.868649	5.396709	6.675241	7.341831	5.466964
17548375	Gm13141	predicted gene 13141 [Source:MGI Symbol;Acc:MGI:3651744]	15.21234	15.21234	15.23914	15.21234	10.27082	13.70718
17421511	Gm13145	predicted gene 13145 [Source:MGI Symbol;Acc:MGI:3701123]	8.665963	7.248236	8.51685	6.372502	6.956926	8.51685
17548371	Gm13149	predicted gene 13149 [Source:MGI Symbol;Acc:MGI:3651738]	24.35473	21.64662	19.71238	17.26681	18.01879	18.81407
17421491	Gm13152	predicted gene 13152	14.63205	12.46905	7.005267	10.95206	5.735344	7.57119
17421521	Gm13154	predicted gene 13154	2.652863	2.27663	1.897658	2.297094	2.854148	2.476684
17432770	Gm13157	predicted gene 13157	13.10886	11.29443	9.13478	11.0828	8.802541	8.524883
17432718	Gm13165	predicted gene 13165 [Source:MGI Symbol;Acc:MGI:3650210]	2.847785	1.882657	2.181046	2.521007	1.769363	2.261124
17421423	Gm13166	predicted gene 13166 [Source:MGI Symbol;Acc:MGI:3705130]	4.517047	6.484521	4.234231	4.502137	4.275995	4.795296
17433525	Gm13173	predicted gene 13173 [Source:MGI Symbol;Acc:MGI:3650870]	3.385268	3.516743	3.714119	3.43998	3.535343	2.936362
17422261	Gm13174	predicted gene 13174 [Source:MGI Symbol;Acc:MGI:3650869]	9.930594	6.089653	9.201187	7.46975	6.030928	6.102879
17381277	Gm13175	predicted gene 13175 [Source:MGI Symbol;Acc:MGI:3651286]	5.665801	5.560174	5.187538	5.682556	5.268601	6.302001
17421307	Gm13177	predicted gene 13177	3.485535	3.406263	3.008282	2.933392	3.28481	4.190813
17432582	Gm13178	predicted gene 13178	3.683737	2.78001	2.884797	4.122559	4.406124	3.367505
17381280	Gm13179	predicted gene 13179	9.248889	6.992896	5.634978	6.921669	9.165731	8.721764
17366448	Gm13180	predicted gene 13180 [Source:MGI Symbol;Acc:MGI:3650463]	6.943774	4.980429	6.943774	6.943774	5.760843	6.379662
17381256	Gm13185	predicted gene 13185 [Source:MGI Symbol;Acc:MGI:3651411]	10.87218	9.310251	9.255879	9.317158	9.310251	9.706156
17366481	Gm13189	predicted gene 13189 [Source:MGI Symbol;Acc:MGI:3650860]	3.110917	3.794764	3.812005	3.960932	3.612231	3.794764
17381259	Gm13191	predicted gene 13191 [Source:MGI Symbol;Acc:MGI:3650045]	4.01461	4.475814	3.452449	4.33133	4.14623	4.150629
17548133	Gm13194	predicted gene 13194	138.1125	144.6117	169.3131	191.8786	169.5233	189.7742
17381397	Gm13199	predicted gene 13199	3.263862	3.263862	3.263862	3.334522	3.263862	3.339703
17432964	Gm13200	predicted gene 13200 [Source:MGI Symbol;Acc:MGI:3650195]	3.91456	3.619198	3.197158	3.07407	3.001638	3.592156
17432891	Gm13201	predicted gene 13201	2.899552	3.263862	3.214914	3.692096	3.263862	3.241811
17421596	Gm13206	predicted gene 13206 [Source:MGI Symbol;Acc:MGI:3651440]	5.205945	5.523183	5.200138	5.70699	5.418299	5.56106
17432985	Gm13209	predicted gene 13209 [Source:MGI Symbol;Acc:MGI:3651228]	7.950855	10.33266	12.36088	10.6303	11.98967	12.28529
17381521	Gm13211	predicted gene 13211 [Source:MGI Symbol;Acc:MGI:3651019]	2.51279	2.031718	2.481049	2.969973	1.990678	2.360725
17421364	Gm13212	predicted gene 13212	2.214161	2.116045	2.040128	2.058067	2.433023	2.19908
17381530	Gm13218	predicted gene 13218 [Source:MGI Symbol;Acc:MGI:3651679]	2.406006	2.302003	2.592978	2.406006	2.188921	2.532426
17366636	Gm13219	predicted gene 13219	2.111883	2.111883	1.934419	2.467158	2.158112	2.111883
17371291	Gm1322	predicted gene 1322	3.009972	2.653396	2.628009	3.123059	3.360906	3.098371
17548365	Gm13229	predicted gene 13229 [Source:MGI Symbol;Acc:MGI:3651933]	26.46475	30.95091	40.31682	33.29445	25.15233	27.26749
17421392	Gm13235	predicted gene 13235 [Source:MGI Symbol;Acc:MGI:3649925]	2.656101	2.027933	2.393646	2.611738	2.440218	2.376387
17421380	Gm13236	predicted gene 13236 [Source:MGI Symbol;Acc:MGI:3705163]	3.582409	3.54506	3.187621	3.724896	3.340213	3.519499
17421334	Gm13242	predicted gene 13242	4.138076	3.684145	3.364145	3.974876	6.398528	4.751325
17421437	Gm13247	predicted gene 13247	3.677002	2.716747	2.948661	2.161196	2.443197	2.441182
17421444	Gm13248	predicted gene 13248	33.19215	27.09848	21.8518	29.4748	26.59126	29.84071
17548367	Gm13249	predicted gene 13249	31.3721	32.80514	27.45087	26.75859	27.69858	28.85461
17421432	Gm13251	predicted gene 13251	11.24366	15.76813	8.719528	18.84592	17.847	14.55073
17366644	Gm13256	predicted gene 13256 [Source:MGI Symbol;Acc:MGI:3651464]	13.09732	9.153732	10.38508	13.39423	10.72213	9.241775
17431022	Gm13257	predicted gene 13257	8.007434	8.137468	7.442256	7.315619	7.64274	7.328612
17381617	Gm13261	predicted gene 13261	3.953033	4.394655	4.988237	4.623194	4.433113	4.640885
17366657	Gm13262	predicted gene 13262	4.801649	4.801649	5.41522	4.801649	4.801649	5.361438
17366949	Gm13264	predicted gene 13264	6.992896	6.694935	5.015592	6.82316	5.649057	8.537833
17367157	Gm13266	predicted gene 13266	2.800802	3.262007	2.953407	2.953407	2.171768	2.547673
17366558	Gm13267	predicted gene 13267	2.727067	2.790435	2.694953	2.569736	3.058067	2.79206
17381906	Gm13269	predicted gene 13269	3.279441	4.849043	3.782107	4.240781	3.774041	3.827255
17367047	Gm13270	predicted gene 13270	2.603081	2.670982	2.575728	2.967319	2.748491	2.845752
17415292	Gm13271	predicted gene 13271	21.11183	30.0004	32.24738	41.27139	24.23449	27.05681
17415315	Gm13272	predicted gene 13272	12.41188	10.22083	19.20766	14.31365	10.53728	11.77398
17427113	Gm13274	predicted gene 13274	9.792587	9.046	9.105827	8.818969	7.555567	8.818969
17415338	Gm13275	predicted gene 13275	5.777318	4.494778	5.01375	4.494778	3.91798	4.964437
17415323	Gm13276	predicted gene 13276	13.56512	11.25099	13.70234	15.50702	13.18467	13.17654
17415328	Gm13277	predicted gene 13277	10.06461	9.52296	10.66876	10.53961	11.38656	12.57093
17415333	Gm13278	predicted gene 13278	13.07827	10.88508	13.71117	15.91582	14.01422	13.05798
17415340	Gm13279	predicted gene 13279	9.94244	10.96429	9.696338	11.25692	9.266792	8.710165
17415296	Gm13283	predicted gene 13283	7.689554	8.769168	10.97174	8.715479	7.526765	8.715479
17415345	Gm13285	predicted gene 13285 [Source:MGI Symbol;Acc:MGI:3701981]	8.103808	10.92499	7.120162	7.946557	10.2008	5.937683
17415294	Gm13286	predicted gene 13286	59.79156	55.32808	50.71849	41.96166	44.34256	82.1609
17415349	Gm13287	predicted gene 13287	8.467112	8.467112	11.40172	8.313466	8.467112	10.22543
17415353	Gm13288	predicted gene 13288	4.830726	5.780603	4.483576	5.112327	5.59113	6.448976
17415305	Gm13290	predicted gene 13290	12.60409	10.65324	11.3393	12.96922	11.10664	10.56455
17415310	Gm13290	predicted gene 13290	12.7811	11.29553	11.61639	13.33504	11.28051	10.38788
17366985	Gm13291	predicted gene 13291 [Source:MGI Symbol;Acc:MGI:3649284]	7.761148	5.91009	6.483263	7.988142	7.051792	7.717641
17366980	Gm13293	predicted gene 13293	2.505971	2.711742	3.798132	2.935284	2.782767	2.960519
17424384	Gm13299	predicted gene 13299 [Source:MGI Symbol;Acc:MGI:3701133]	7.668742	8.933156	9.049261	7.896591	8.164474	9.797339
17392617	Gm1330	predicted gene 1330 [Source:MGI Symbol;Acc:MGI:2686176]	3.339703	3.880681	2.894752	2.399544	3.15745	3.339703
17424407	Gm13303	predicted gene 13303 [Source:MGI Symbol;Acc:MGI:3705775]	25.22618	40.944	65.41502	47.71331	37.88619	28.37444
17412965	Gm13304	predicted gene 13304	20.79032	19.09417	29.05612	25.3814	16.58331	22.47081
17413025	Gm13304	predicted gene 13304	20.79032	19.09417	29.05612	25.3814	16.58331	22.47081
17413078	Gm13304	predicted gene 13304	26.90757	19.42873	32.67035	29.41439	24.63129	19.84798
17413123	Gm13304	predicted gene 13304	20.79032	19.09417	29.05612	25.3814	16.58331	22.47081
17434150	Gm13304	predicted gene 13304	20.79032	19.09417	29.05612	25.3814	16.58331	22.47081
17434230	Gm13304	predicted gene 13304	26.90757	19.42873	32.67035	29.41439	24.63129	19.84798
17424389	Gm13305	predicted gene 13305	22.59607	20.47881	25.2561	20.58541	25.04412	20.68811
17424431	Gm13305	predicted gene 13305	22.59607	20.47881	25.2561	20.58541	25.04412	20.68811
17434174	Gm13305	predicted gene 13305	22.59607	20.47881	25.2561	20.58541	25.04412	20.68811
17434259	Gm13305	predicted gene 13305	22.59607	20.47881	25.2561	20.58541	25.04412	20.68811
17413039	Gm13306	predicted gene 13306	15.03976	13.524	12.91925	12.46024	12.57355	14.53178
17413137	Gm13306	predicted gene 13306	28.68889	26.79942	24.43234	23.6379	23.756	27.53434
17434210	Gm13306	predicted gene 13306	28.29451	29.46479	26.6734	26.93444	24.67845	30.40275
17434254	Gm13306	predicted gene 13306	28.2017	26.39971	21.07382	23.0089	22.04378	27.22519
17424352	Gm13307	predicted gene 13307 [Source:MGI Symbol;Acc:MGI:3701135]	3.115578	3.373345	4.956151	3.114326	2.542839	3.719374
17412979	Gm13308	predicted gene 13308	19.2821	20.07962	19.34864	19.14559	19.2821	19.2821

17413090	Gm13308	predicted gene 13308	8.595908	9.247606	11.08433	8.398607	8.473864	8.220408
17413103	Gm13308	predicted gene 13308	8.595908	9.247606	11.08433	8.398607	8.473864	8.220408
17434137	Gm13308	predicted gene 13308	28.48065	26.18248	30.66647	28.48065	23.66019	26.08665
17381937	Gm13322	predicted gene 13322 [Source:MGI Symbol;Acc:MGI:3650424]	4.232441	4.728688	4.20927	5.35019	3.978129	4.490419
17367234	Gm13324	predicted gene 13324 [Source:MGI Symbol;Acc:MGI:3650422]	5.447824	5.598343	6.482409	6.807334	8.088039	5.889928
17382151	Gm13327	predicted gene 13327 [Source:MGI Symbol;Acc:MGI:3651762]	6.090286	5.167405	4.713112	4.69082	4.175416	6.22671
17367239	Gm13330	predicted gene 13330 [Source:MGI Symbol;Acc:MGI:3649452]	4.351703	4.685198	4.667209	4.820958	4.667209	4.667209
17382098	Gm13335	predicted gene 13335 [Source:MGI Symbol;Acc:MGI:3651482]	2.67402	3.339703	3.185633	4.099477	3.318221	3.484327
17382051	Gm13344	predicted gene 13344 [Source:MGI Symbol;Acc:MGI:3649239]	4.177935	4.258838	4.092493	3.219039	4.578325	5.62608
17382089	Gm13346	predicted gene 13346 [Source:MGI Symbol;Acc:MGI:3649698]	3.00374	3.504819	4.334633	4.121444	4.434957	3.344499
17382080	Gm13348	predicted gene 13348	2.428036	2.726424	2.603929	2.857374	2.547002	2.238496
17548195	Gm13351	predicted gene 13351 [Source:MGI Symbol;Acc:MGI:3651067]	4.681043	4.945547	5.399216	6.627616	6.018483	5.297751
17382101	Gm13362	predicted gene 13362 [Source:MGI Symbol;Acc:MGI:3652150]	3.392103	4.346668	4.340556	3.695296	4.130167	5.116864
17367436	Gm13363	predicted gene 13363	142.8404	218.5183	264.0779	170.4636	223.827	223.0278
17383366	Gm13371	predicted gene 13371 [Source:MGI Symbol;Acc:MGI:3650807]	4.292982	3.432025	3.889218	5.088322	4.09065	6.024285
17383350	Gm13372	predicted gene 13372	5.057309	5.517291	6.064632	5.932047	5.456894	5.61887
17383369	Gm13373	predicted gene 13373	4.550547	4.375233	4.229333	5.225503	4.235992	4.235992
17367440	Gm13375	predicted gene 13375	14.63793	14.20001	14.1826	15.47138	18.10957	15.62961
17367443	Gm13376	predicted gene 13376 [Source:MGI Symbol;Acc:MGI:3649912]	2.947067	3.203191	3.998392	3.468784	3.962333	3.680725
17382136	Gm13380	predicted gene 13380 [Source:MGI Symbol;Acc:MGI:3649647]	3.025409	3.762287	3.347189	2.868686	3.696833	3.422997
17368710	Gm13381	predicted gene 13381 [Source:MGI Symbol;Acc:MGI:3649650]	5.440616	8.368912	9.310251	6.614261	5.797979	6.573948
17381468	Gm13383	predicted gene 13383	7.364717	7.313541	8.998035	7.698994	9.916699	7.264784
17382378	Gm13387	predicted gene 13387	2.651116	4.387016	3.593202	3.624294	4.664815	3.184706
17381471	Gm13388	predicted gene 13388	3.754186	6.155176	4.172292	4.503323	4.56929	3.132078
17366623	Gm13389	predicted gene 13389 [Source:MGI Symbol;Acc:MGI:3649902]	10.42821	9.59691	7.426281	10.16896	7.25095	10.48395
17366618	Gm13391	predicted gene 13391	6.953648	5.875941	6.178129	6.190517	6.290698	6.178129
17548141	Gm13392	predicted gene 13392 [Source:MGI Symbol;Acc:MGI:3649898]	70.32667	94.51294	78.14708	91.51396	111.8987	100.1341
17368799	Gm13393	predicted gene 13393 [Source:MGI Symbol;Acc:MGI:3649783]	2.181484	2.167422	2.113802	2.494447	2.36966	2.285991
17368473	Gm13398	predicted gene 13398	4.544338	6.660452	5.225902	7.529844	6.626559	4.313085
17383498	Gm13402	predicted gene 13402 [Source:MGI Symbol;Acc:MGI:3649787]	2.672463	2.459202	2.211155	2.293785	2.631545	2.692264
17384220	Gm13403	predicted gene 13403	3.308506	3.232096	3.717184	3.500934	4.623419	3.016509
17383781	Gm13405	predicted gene 13405 [Source:MGI Symbol;Acc:MGI:3649515]	5.747601	5.080463	6.084295	6.265545	5.595356	6.14912
17367683	Gm13411	predicted gene 13411 [Source:MGI Symbol;Acc:MGI:3651013]	20.39919	15.46645	12.82449	19.06216	14.80467	16.88465
17369838	Gm13412	predicted gene 13412 [Source:MGI Symbol;Acc:MGI:3650612]	3.407846	3.253201	2.882479	3.324598	3.709334	4.566472
17367726	Gm13415	predicted gene 13415	2.814815	3.613458	2.888079	3.703322	3.625349	3.729055
17382244	Gm13417	predicted gene 13417 [Source:MGI Symbol;Acc:MGI:3652159]	4.30505	4.224469	3.843925	4.774799	3.453933	3.610378
17383501	Gm13420	predicted gene 13420	8.026049	6.897148	6.897148	6.135709	6.28538	6.927621
17383785	Gm13425	predicted gene 13425	3.425679	3.738216	3.647126	4.437909	3.456208	4.583882
17383788	Gm13427	predicted gene 13427 [Source:MGI Symbol;Acc:MGI:3650808]	9.450496	9.800124	8.458724	10.56851	9.157793	8.159779
17370297	Gm13429	predicted gene 13429	3.536568	3.879713	3.681133	3.729055	3.729055	3.063243
17383808	Gm13441	predicted gene 13441 [Source:MGI Symbol;Acc:MGI:3649406]	12.59361	12.44669	12.72153	16.55735	10.93954	14.30116
17383813	Gm13442	predicted gene 13442 [Source:MGI Symbol;Acc:MGI:3650661]	14.88988	12.48748	11.37094	11.37094	13.88158	8.610697
17384502	Gm13446	predicted gene 13446	6.582429	6.612748	6.71571	7.203809	8.343774	7.326377
17370136	Gm13448	predicted gene 13448 [Source:MGI Symbol;Acc:MGI:3651456]	5.846001	7.173613	4.930862	5.846001	5.38229	6.173249
17370141	Gm13449	predicted gene 13449 [Source:MGI Symbol;Acc:MGI:3651453]	3.708948	5.253613	6.992896	4.225904	4.424241	4.491259
17370522	Gm13456	predicted gene 13456 [Source:MGI Symbol;Acc:MGI:3651389]	1005.195	1085.016	1037.841	1024.206	1133.58	1086.22
17370588	Gm13465	predicted gene 13465 [Source:MGI Symbol;Acc:MGI:3649598]	2.971003	4.006188	3.157603	3.413898	3.436357	3.54506
17384976	Gm13467	predicted gene 13467 [Source:MGI Symbol;Acc:MGI:3649595]	3.0092	3.380063	3.693324	4.054123	3.668499	5.039184
17384979	Gm13470	predicted gene 13470	3.890269	3.933109	5.966976	3.89861	4.011773	4.70593
17370580	Gm13479	predicted gene 13479	3.857804	4.240252	4.264274	4.127362	4.220957	3.629347
17385024	Gm13480	predicted gene 13480	2.38778	2.264967	2.727842	2.797542	2.859514	3.339703
17384985	Gm13481	predicted gene 13481	4.918812	4.064929	3.374187	4.31159	2.964133	4.197935
17370715	Gm13483	predicted gene 13483	5.520428	6.270715	5.120306	5.120306	5.120306	5.031261
17370721	Gm13485	predicted gene 13485	3.027863	2.885037	3.148053	2.897302	3.571723	2.65962
17385065	Gm13490	predicted gene 13490	4.046487	3.951198	4.273269	4.428107	4.423934	4.197732
17384741	Gm13496	predicted gene 13496 [Source:MGI Symbol;Acc:MGI:3651033]	2.882039	3.361488	4.02188	3.50324	2.869879	4.549128
17370729	Gm13497	predicted gene 13497	2.651723	2.144543	2.283758	2.283758	2.446908	2.283758
17385054	Gm13499	predicted gene 13499 [Source:MGI Symbol;Acc:MGI:3650382]	3.791714	3.238487	2.944025	2.807196	3.730373	2.86807
17370794	Gm13522	predicted gene 13522 [Source:MGI Symbol;Acc:MGI:3651428]	2.745007	4.946644	3.760436	6.632855	4.352065	4.382305
17384046	Gm13523	predicted gene 13523 [Source:MGI Symbol;Acc:MGI:3651426]	8.674977	9.614421	9.614421	9.399344	7.101727	10.29855
17369999	Gm13524	predicted gene 13524	9.923163	10.45978	13.5285	13.65304	14.04739	14.74598
17370040	Gm13528	predicted gene 13528	13.17846	13.58008	13.67928	21.1051	11.43417	12.26878
17370057	Gm13530	predicted gene 13530	11.68333	11.52736	11.42855	11.68333	13.1234	10.88116
17370054	Gm13536	predicted gene 13536	3.161151	3.805801	3.520426	3.520426	3.963596	3.533505
17368200	Gm13539	predicted gene 13539	2.67402	2.667818	2.67402	3.012205	2.999205	2.693627
17370947	Gm13544	predicted gene 13544	3.459252	4.04402	3.589791	3.776946	3.876031	3.489693
17548147	Gm13545	predicted gene 13545 [Source:MGI Symbol;Acc:MGI:3649667]	52.57992	83.57676	107.3129	71.143	72.90723	75.8102
17370941	Gm13546	predicted gene 13546	3.703322	2.808384	3.461218	2.950828	3.915292	3.047402
17368887	Gm13547	predicted gene 13547	5.104077	5.571362	6.171709	6.134986	6.636166	8.307647
17385483	Gm13552	predicted gene 13552	118.9878	112.713	130.9425	101.0706	112.9249	110.1839
17382848	Gm13553	predicted gene 13553 [Source:MGI Symbol;Acc:MGI:3650620]	5.377069	5.781284	5.942953	6.259641	5.11988	5.926211
17368254	Gm13554	predicted gene 13554 [Source:MGI Symbol;Acc:MGI:3650406]	4.833941	8.291597	4.872999	4.742047	5.025371	8.213289
17370437	Gm13556	predicted gene 13556 [Source:MGI Symbol;Acc:MGI:3651929]	3.024067	3.54506	3.726833	3.297585	3.032022	3.340676
17371195	Gm13561	predicted gene 13561	5.394652	7.521967	5.795407	5.333527	5.1425	7.852598
17368286	Gm13562	predicted gene 13562	10.65944	15.11082	12.86866	13.95442	12.35189	11.5552
17368292	Gm13563	predicted gene 13563 [Source:MGI Symbol;Acc:MGI:3652052]	12.23956	17.33757	13.5514	13.97823	9.682436	15.16938
17385753	Gm13564	predicted gene 13564 [Source:MGI Symbol;Acc:MGI:3649652]	4.511102	5.664702	6.278892	5.406631	6.470019	7.342595
17385750	Gm13565	predicted gene 13565 [Source:MGI Symbol;Acc:MGI:3649651]	2.242441	2.420844	2.420844	1.970375	2.281559	2.420844
17371212	Gm13569	predicted gene 13569 [Source:MGI Symbol;Acc:MGI:3649495]	2.248402	2.18224	1.88757	1.823285	2.053194	2.705265
17548203	Gm13570	predicted gene 13570	26.56104	33.43622	28.49822	35.63542	30.56122	26.56104
17371078	Gm13571	predicted gene 13571	2.737464	3.351275	3.338685	3.176923	2.809902	3.897503
17371053	Gm13572	predicted gene 13572	2.769566	2.76995	2.438662	2.975619	2.906132	3.399447
17385850	Gm13575	predicted gene 13575	2.406006	2.031718	2.197404	2.545766	2.137405	2.54046
17371088	Gm13580	predicted gene 13580	4.255003	4.225963	4.23611	3.713836	3.930465	3.843365

17385712	Gm13582	predicted gene 13582	5.066291	6.859531	5.803487	6.126232	5.695845	6.851406
17371092	Gm13583	predicted gene 13583	3.171093	2.746568	3.458628	3.765418	3.263862	3.478367
17384689	Gm13584	predicted gene 13584	4.09641	3.339703	2.830192	3.281374	3.339703	3.339703
17370450	Gm13589	predicted gene 13589 [Source:MGI Symbol;Acc:MGI:3649991]	7.247483	7.752008	7.752008	8.073924	8.416731	7.801492
17371218	Gm13595	predicted gene 13595 [Source:MGI Symbol;Acc:MGI:3650913]	2.578979	2.416401	2.040864	2.228542	2.379156	2.275621
17371315	Gm13596	predicted gene 13596	18.05805	18.49649	16.36612	16.40428	20.06473	17.76222
17386117	Gm13598	predicted gene 13598 [Source:MGI Symbol;Acc:MGI:3649281]	3.462322	3.20519	3.072536	3.337991	3.145131	2.796011
17386121	Gm13599	predicted gene 13599	5.141075	4.066901	3.607777	3.171972	3.443391	3.54506
17423885	Gm136	predicted gene 136	3.739538	2.796922	2.44445	3.202525	2.953407	2.953407
17386130	Gm13601	predicted gene 13601	4.515858	4.260403	3.088615	2.701911	2.689877	3.248469
17370206	Gm13605	predicted gene 13605	4.617558	5.24819	5.24819	6.315294	5.24819	5.24819
17383816	Gm13609	predicted gene 13609	21.8468	16.32872	22.3791	24.33768	22.92929	25.94995
17383832	Gm13610	predicted gene 13610	17.2122	9.702254	11.06775	10.81079	12.44876	12.24847
17371421	Gm13613	predicted gene 13613 [Source:MGI Symbol;Acc:MGI:3650687]	8.161872	6.672928	5.407306	5.517903	5.489558	5.952105
17385964	Gm13617	predicted gene 13617 [Source:MGI Symbol;Acc:MGI:3650300]	3.009972	3.193503	3.009972	2.953407	3.009972	3.05182
17371267	Gm13618	predicted gene 13618 [Source:MGI Symbol;Acc:MGI:3650299]	4.622618	3.212129	3.818484	4.246841	4.193341	4.373141
17385961	Gm13619	predicted gene 13619 [Source:MGI Symbol;Acc:MGI:3702958]	3.913854	2.793299	2.606909	2.606909	2.712927	2.947885
17371056	Gm13620	predicted gene 13620 [Source:MGI Symbol;Acc:MGI:3650077]	15.21287	18.10593	15.6334	19.44625	17.90983	19.90391
17371281	Gm13629	predicted gene 13629	6.287864	6.287864	5.611553	8.045879	6.030928	6.335682
17371272	Gm13630	predicted gene 13630	5.117858	3.297951	3.834796	4.20927	4.226256	3.678073
17371682	Gm13632	predicted gene 13632 [Source:MGI Symbol;Acc:MGI:3651357]	3.448981	3.453645	3.011307	3.643159	3.263342	3.362397
17433490	Gm13645	predicted gene 13645 [Source:MGI Symbol;Acc:MGI:3650945]	4.383217	5.255908	5.246808	5.750989	5.945631	5.257839
17386685	Gm13652	predicted gene 13652	5.061974	4.41998	4.714975	5.081344	4.144247	4.717688
17386711	Gm13656	predicted gene 13656 [Source:MGI Symbol;Acc:MGI:3650910]	3.146025	3.164132	3.062691	3.645786	3.204257	3.326396
17372156	Gm13657	predicted gene 13657	8.884681	14.67756	13.74848	10.5272	14.06395	10.20985
17372136	Gm13658	predicted gene 13658 [Source:MGI Symbol;Acc:MGI:3649763]	2.334721	2.241679	2.018467	2.189953	2.431826	2.756524
17386697	Gm13660	predicted gene 13660 [Source:MGI Symbol;Acc:MGI:3649762]	3.449267	2.645298	2.760175	2.912686	2.912686	4.519145
17372271	Gm13661	predicted gene 13661 [Source:MGI Symbol;Acc:MGI:3649760]	4.264244	3.626757	3.165076	3.703322	3.703322	3.646191
17371839	Gm13663	predicted gene 13663 [Source:MGI Symbol;Acc:MGI:3650323]	6.291831	6.645326	5.609258	6.965901	6.055516	8.018654
17372026	Gm13667	predicted gene 13667 [Source:MGI Symbol;Acc:MGI:3650879]	3.067216	2.962058	2.762549	2.762549	2.88946	2.979438
17387363	Gm13680	predicted gene 13680 [Source:MGI Symbol;Acc:MGI:3652285]	97.93143	106.9261	109.8522	116.2462	109.9794	147.5418
17387368	Gm13684	predicted gene 13684 [Source:MGI Symbol;Acc:MGI:3649340]	3.165032	3.134358	3.375076	3.287293	3.613909	3.68172
17387365	Gm13686	predicted gene 13686 [Source:MGI Symbol;Acc:MGI:3651825]	10.76368	11.1503	12.35189	18.84226	12.2405	10.90796
17372564	Gm13691	predicted gene 13691 [Source:MGI Symbol;Acc:MGI:3702053]	29.46949	28.95579	23.87303	27.25162	27.25162	27.25162
17372566	Gm13691	predicted gene 13691 [Source:MGI Symbol;Acc:MGI:3702053]	29.46949	28.95579	23.87303	27.25162	27.25162	27.25162
17372570	Gm13691	predicted gene 13691 [Source:MGI Symbol;Acc:MGI:3702053]	29.46949	28.95579	23.87303	27.25162	27.25162	27.25162
17372572	Gm13691	predicted gene 13691 [Source:MGI Symbol;Acc:MGI:3702053]	29.46949	28.95579	23.87303	27.25162	27.25162	27.25162
17372574	Gm13691	predicted gene 13691 [Source:MGI Symbol;Acc:MGI:3702053]	29.46949	28.95579	23.87303	27.25162	27.25162	27.25162
17372576	Gm13691	predicted gene 13691 [Source:MGI Symbol;Acc:MGI:3702053]	29.46949	28.95579	23.87303	27.25162	27.25162	27.25162
17372568	Gm13695	predicted gene 13695 [Source:MGI Symbol;Acc:MGI:3702066]	29.46949	28.95579	23.87303	27.25162	27.25162	27.25162
17371990	Gm13703	predicted gene 13703 [Source:MGI Symbol;Acc:MGI:3651307]	4.527517	3.775689	3.59243	4.026399	4.631341	4.468684
17371996	Gm13707	predicted gene 13707	4.51301	4.452236	4.838433	4.867395	4.589764	4.131999
17372002	Gm13707	predicted gene 13707	2.850174	4.023915	3.703322	4.223151	3.79082	3.808466
17372007	Gm13708	predicted gene 13708 [Source:MGI Symbol;Acc:MGI:3651236]	4.352035	4.315866	3.703322	4.830893	3.563735	4.276943
17371993	Gm13709	predicted gene 13709	9.898226	9.032404	11.56746	11.07358	11.56746	14.33093
17387426	Gm13710	predicted gene 13710	3.069875	2.964133	3.212441	3.576603	6.505275	2.617936
17372729	Gm13713	predicted gene 13713 [Source:MGI Symbol;Acc:MGI:3650490]	3.884799	5.225648	4.684289	5.520848	5.120306	4.472929
17372738	Gm13715	predicted gene 13715	3.539437	4.20927	4.303379	4.135552	5.196607	3.973224
17372735	Gm13716	predicted gene 13716 [Source:MGI Symbol;Acc:MGI:3650432]	3.696859	4.793602	4.693389	4.376143	4.172639	4.051679
17371870	Gm13746	predicted gene 13746 [Source:MGI Symbol;Acc:MGI:3651629]	5.784772	9.496132	7.3888	6.143837	5.386171	7.386802
17213669	Gm13748	predicted gene 13748	3.411793	3.388484	2.873123	3.815151	3.528952	4.065718
17213663	Gm13749	predicted gene 13749	5.002711	3.920235	3.55473	3.181043	4.186109	3.947447
17372420	Gm13752	predicted gene 13752	2.482786	2.228434	2.531425	2.531425	2.883658	2.536378
17387819	Gm13753	predicted gene 13753 [Source:MGI Symbol;Acc:MGI:3713298]	2.162035	2.005567	2.290686	2.162035	2.188921	2.162035
17387866	Gm13763	predicted gene 13763 [Source:MGI Symbol;Acc:MGI:3650763]	2.365721	2.174223	2.105248	2.509222	2.39514	2.285991
17372974	Gm13769	predicted gene 13769	6.453581	7.588002	9.69775	6.643254	6.017941	5.828643
17388030	Gm13769	predicted gene 13769	6.453581	7.588002	9.69775	6.643254	6.017941	5.828643
17548213	Gm13770	predicted gene 13770 [Source:MGI Symbol;Acc:MGI:3651719]	51.72321	109.8377	194.4466	109.6605	93.65668	110.1855
17414507	Gm13773	predicted gene 13773 [Source:MGI Symbol;Acc:MGI:3712316]	176.7161	112.9437	138.1441	253.1344	186.514	182.6275
17388347	Gm13780	predicted gene 13780 [Source:MGI Symbol;Acc:MGI:3650707]	2.54261	3.346586	3.252434	3.168785	2.782767	3.117847
17465532	Gm13781	predicted gene 13781	4.20927	3.88523	5.142108	4.20927	4.115392	4.20927
17456931	Gm13782	predicted gene 13782	8.252779	8.668366	12.79404	8.827225	6.157096	8.827225
17440971	Gm13790	predicted gene 13790	6.408648	6.408648	6.992896	6.943774	5.246408	8.397966
17388414	Gm13791	predicted gene 13791	4.987442	4.430839	4.9972	5.127445	3.71484	3.881488
17373607	Gm13794	predicted gene 13794	2.162035	2.634648	2.101617	2.608897	2.84378	2.753602
17373611	Gm13799	predicted gene 13799	3.286928	2.45088	2.727294	2.542328	2.782362	3.456208
17388432	Gm13802	predicted gene 13802 [Source:MGI Symbol;Acc:MGI:3651560]	4.528145	5.001081	4.668115	5.682123	3.92483	4.376356
17373565	Gm13807	predicted gene 13807	4.5209	4.071696	3.787485	3.787485	2.414258	4.062056
17373513	Gm13814	predicted gene 13814	3.687109	3.074198	3.156925	5.386134	4.11699	2.602936
17548151	Gm13815	predicted gene 13815 [Source:MGI Symbol;Acc:MGI:3649318]	40.97381	42.49438	38.97211	39.7637	46.46782	33.47797
17373527	Gm13816	predicted gene 13816	6.171709	6.712267	5.771995	5.546701	5.739599	5.843772
17451626	Gm13822	predicted gene 13822	5.873457	7.909574	6.479848	7.691633	10.05876	5.299404
17441114	Gm13830	predicted gene 13830	4.615446	4.65348	4.40124	4.88846	3.924775	4.776156
17456969	Gm13833	predicted gene 13833 [Source:MGI Symbol;Acc:MGI:3650920]	6.544033	9.143078	7.80544	7.18008	7.289349	6.486184
17465605	Gm13834	predicted gene 13834 [Source:MGI Symbol;Acc:MGI:3650919]	14.60236	14.73485	14.62353	15.8512	18.27095	14.27659
17441340	Gm13837	predicted gene 13837 [Source:MGI Symbol;Acc:MGI:3650972]	3.186097	3.560476	3.68854	3.703322	4.214291	3.398575
17451811	Gm13838	predicted gene 13838 [Source:MGI Symbol;Acc:MGI:3649377]	4.82293	4.025645	5.731767	5.765797	6.23591	6.34113
17441343	Gm13839	predicted gene 13839 [Source:MGI Symbol;Acc:MGI:3650513]	6.315732	5.290778	6.120417	6.0648	6.255217	5.37137
17441188	Gm13840	predicted gene 13840	4.01461	3.373953	3.130472	2.92448	3.729055	3.03886
17451780	Gm13842	predicted gene 13842 [Source:MGI Symbol;Acc:MGI:3650892]	3.157275	3.703784	3.43143	3.664306	4.133174	3.958188
17457027	Gm13846	predicted gene 13846	3.456519	3.229341	3.486356	3.886954	3.44351	3.352413
17465632	Gm13848	predicted gene 13848	19.43493	22.62789	26.89321	22.37429	22.25766	23.40379
17457031	Gm13849	predicted gene 13849 [Source:MGI Symbol;Acc:MGI:3651992]	3.759289	4.20927	4.165328	3.363912	3.916378	4.20927
17457044	Gm13850	predicted gene 13850	5.093474	4.624092	5.075043	5.486211	4.473022	6.307245

17465692	Gm13853	predicted gene 13853	2.753335	2.812124	2.934796	2.663217	3.427817	2.67708
17457122	Gm13855	predicted gene 13855	3.604177	2.69176	3.366385	3.069918	2.779374	4.184137
17457116	Gm13856	predicted gene 13856 [Source:MGI Symbol;Acc:MGI:3652199]	3.90613	3.482902	3.157034	3.461122	3.74431	2.846304
17457180	Gm13860	predicted gene 13860 [Source:MGI Symbol;Acc:MGI:3649464]	3.263862	3.263862	3.263862	3.263862	3.660752	3.166195
17465728	Gm13861	predicted gene 13861	3.062691	2.907421	3.085705	3.261329	3.009972	3.494606
17388771	Gm13869	predicted gene 13869	4.537728	4.315567	4.764319	4.647291	5.977739	4.20927
17373732	Gm13874	predicted gene 13874 [Source:MGI Symbol;Acc:MGI:3650729]	4.637091	3.293359	4.017895	3.703322	4.16097	5.54647
17373837	Gm13883	predicted gene 13883 [Source:MGI Symbol;Acc:MGI:3650051]	5.338965	5.13349	5.338965	5.338965	4.314819	6.137325
17548156	Gm13884	predicted gene 13884	21.86862	22.87104	15.49102	18.26196	21.87923	28.77731
17388548	Gm13889	predicted gene 13889	17.77564	22.76649	22.71825	15.10977	15.33185	12.43169
17373657	Gm13905	predicted gene 13905 [Source:MGI Symbol;Acc:MGI:3651198]	2.67402	2.523874	2.580266	2.407173	2.915574	3.270021
17389117	Gm13912	predicted gene 13912	2.406006	2.285991	2.121023	2.304206	2.372306	2.396851
17374055	Gm13923	predicted gene 13923	2.389452	2.304206	2.071894	2.304206	2.499553	2.436314
17374132	Gm13936	predicted gene 13936 [Source:MGI Symbol;Acc:MGI:3651878]	7.127124	6.256778	3.799396	4.130024	2.847133	4.834779
17372255	Gm13938	predicted gene 13938 [Source:MGI Symbol;Acc:MGI:3651046]	9.276303	10.02847	8.571633	10.03501	8.171042	7.621738
17389195	Gm13941	predicted gene 13941	3.088144	2.67402	2.692731	2.67402	2.351628	2.219139
17372261	Gm13943	predicted gene 13943 [Source:MGI Symbol;Acc:MGI:3650360]	15.08361	15.37101	15.63915	15.2768	13.89853	19.5883
17372264	Gm13944	predicted gene 13944	5.338965	4.801649	4.385314	5.170701	5.234457	4.801649
17373981	Gm13954	predicted gene 13954 [Source:MGI Symbol;Acc:MGI:3649858]	3.174633	3.263862	2.902005	2.643116	2.782767	2.379305
17389260	Gm13962	predicted gene 13962 [Source:MGI Symbol;Acc:MGI:3650830]	4.831965	3.090885	3.429861	2.885097	3.997505	3.989589
17389449	Gm13963	predicted gene 13963 [Source:MGI Symbol;Acc:MGI:3650832]	4.439509	4.525979	4.132228	3.6447	4.123387	4.044384
17389456	Gm13964	predicted gene 13964	2.217924	2.555579	3.258821	2.778584	2.727842	2.80998
17374388	Gm13972	predicted gene 13972 [Source:MGI Symbol;Acc:MGI:3649226]	4.079295	3.763252	4.20927	4.20927	5.095204	4.20927
17374391	Gm13974	predicted gene 13974	2.277435	2.606909	2.854088	2.606909	2.712927	2.432433
17374401	Gm13976	predicted gene 13976 [Source:MGI Symbol;Acc:MGI:3651280]	4.086965	3.675702	3.787485	3.907457	4.186545	3.383555
17548164	Gm13981	predicted gene 13981 [Source:MGI Symbol;Acc:MGI:3652167]	14.67603	15.98238	16.54599	17.27531	16.64146	19.47903
17389668	Gm13982	predicted gene 13982	2.841002	2.580052	2.35636	2.784079	2.490594	2.321601
17389672	Gm13985	predicted gene 13985	3.050086	4.136126	3.858152	4.136126	4.136126	5.798863
17389675	Gm13986	predicted gene 13986	4.506508	5.467533	4.181962	3.691328	3.227462	3.971021
17389640	Gm13990	predicted gene 13990	2.83626	2.746796	2.871511	2.858424	2.821652	2.868447
17375587	Gm13994	predicted gene 13994 [Source:MGI Symbol;Acc:MGI:3651835]	2.874877	2.874877	2.586014	3.163957	3.248987	3.575042
17389994	Gm13999	predicted gene 13999 [Source:MGI Symbol;Acc:MGI:3651905]	5.05342	3.914098	3.361325	4.01461	4.79038	5.229888
17390876	Gm14003	predicted gene 14003 [Source:MGI Symbol;Acc:MGI:3649824]	3.778595	2.871312	3.022265	2.32624	2.899693	4.030559
17390866	Gm14004	predicted gene 14004 [Source:MGI Symbol;Acc:MGI:3651365]	5.245717	4.423536	4.423536	5.023769	3.622285	3.946462
17391406	Gm14005	predicted gene 14005	4.20927	4.20927	4.886326	3.876917	4.267349	4.413398
17548172	Gm14007	predicted gene 14007 [Source:MGI Symbol;Acc:MGI:3652193]	3.475041	2.83687	3.351414	2.997916	3.132816	3.287726
17375976	Gm14009	predicted gene 14009	3.647278	3.162817	3.592554	3.289732	3.178883	3.652489
17375981	Gm14010	predicted gene 14010	26.69523	27.94833	26.69523	31.65497	26.60914	29.76304
17391487	Gm14011	predicted gene 14011 [Source:MGI Symbol;Acc:MGI:3649272]	23.69449	23.89489	25.2808	29.48685	25.89282	25.87685
17389079	Gm14014	predicted gene 14014 [Source:MGI Symbol;Acc:MGI:3649268]	5.953384	6.175346	5.234385	4.533075	5.846122	5.136551
17389075	Gm14015	predicted gene 14015	4.924782	6.524874	5.617429	5.779401	5.000873	5.211938
17390665	Gm14019	predicted gene 14019 [Source:MGI Symbol;Acc:MGI:3651448]	3.594248	3.883991	4.172378	4.55244	4.172378	5.492831
17376153	Gm14023	predicted gene 14023	4.030866	3.964448	5.063588	4.009299	4.136126	4.82594
17376149	Gm14024	predicted gene 14024 [Source:MGI Symbol;Acc:MGI:3649704]	6.810827	10.08333	8.912128	8.420524	9.570272	8.420524
17391510	Gm14025	predicted gene 14025	3.604927	3.051101	3.961728	4.257894	4.540717	4.05387
17391518	Gm14025	predicted gene 14025	4.632401	5.709522	3.557984	5.408655	4.901183	5.797737
17391528	Gm14029	predicted gene 14029 [Source:MGI Symbol;Acc:MGI:3650450]	3.588075	3.225717	2.999974	3.061885	3.750336	3.292287
17391583	Gm14040	predicted gene 14040	4.652448	4.891137	5.521537	4.171655	4.213269	5.521537
17391587	Gm14041	predicted gene 14041	4.645037	4.886969	4.886969	4.886969	5.506898	4.886969
17376557	Gm14051	predicted gene 14051	4.801649	4.801649	4.767722	4.801649	4.906724	5.042573
17376856	Gm14055	predicted gene 14055	7.945291	8.2301	7.177742	7.177742	6.737064	7.527131
17376883	Gm14061	predicted gene 14061	4.334213	3.556135	4.316913	3.552809	3.124077	3.745712
17392180	Gm14062	predicted gene 14062	2.250647	2.436837	2.174314	2.346076	2.103118	2.46135
17376862	Gm14064	predicted gene 14064	3.79287	5.00278	5.135305	5.464199	4.090309	4.191365
17375480	Gm14085	predicted gene 14085	4.681043	2.383894	2.552286	2.4755	2.188921	2.641963
17374683	Gm14089	predicted gene 14089	11.22606	12.68041	15.57176	16.23258	14.9394	18.21632
17389792	Gm14091	predicted gene 14091 [Source:MGI Symbol;Acc:MGI:3650488]	3.703322	3.703322	3.522354	3.272266	3.423258	3.268843
17392448	Gm14092	predicted gene 14092	4.981396	4.34697	5.332049	4.856376	5.025266	4.614166
17377164	Gm14093	predicted gene 14093 [Source:MGI Symbol;Acc:MGI:3651964]	2.406006	2.444349	2.606565	2.594434	3.239879	2.845121
17391993	Gm14095	predicted gene 14095	2.991937	2.910345	2.844313	2.921846	3.111952	3.746595
17376672	Gm14100	predicted gene 14100	2.307643	2.61498	3.261668	2.604777	2.280184	2.700226
17376661	Gm14102	predicted gene 14102 [Source:MGI Symbol;Acc:MGI:3651501]	5.280923	4.291673	4.55285	4.910024	4.670607	4.493719
17392043	Gm14104	predicted gene 14104 [Source:MGI Symbol;Acc:MGI:3651500]	14.74465	12.21879	13.97387	16.15333	14.23036	14.23036
17392543	Gm14110	predicted gene 14110 [Source:MGI Symbol;Acc:MGI:3649328]	6.501894	5.667097	6.489917	5.840371	5.35338	5.942665
17392522	Gm14114	predicted gene 14114	2.97597	2.494205	2.735245	2.402839	3.176835	2.480773
17377349	Gm14120	predicted gene 14120 [Source:MGI Symbol;Acc:MGI:3651530]	3.980556	3.851579	5.134985	4.450169	3.483144	4.783113
17377471	Gm14123	predicted gene 14123 [Source:MGI Symbol;Acc:MGI:3650509]	2.753774	3.239161	3.006344	3.053915	3.218102	3.35023
17377454	Gm14124	predicted gene 14124	2.886117	2.421985	2.754117	2.705696	2.436094	1.886471
17392627	Gm14133	predicted gene 14133	15.10161	17.11762	21.83832	16.91416	15.194	17.08312
17377420	Gm14135	predicted gene 14135 [Source:MGI Symbol;Acc:MGI:3649300]	2.330323	2.366869	2.005775	2.393646	2.467038	2.590984
17374743	Gm14137	predicted gene 14137	7.526296	8.309836	7.123025	9.776445	7.006335	8.142377
17377443	Gm14139	predicted gene 14139	2.432467	2.194192	2.597637	2.482339	2.432349	2.120067
17392794	Gm14144	predicted gene 14144	3.703065	3.276283	4.017394	3.090457	3.663722	3.461194
17377540	Gm14147	predicted gene 14147	7.870689	7.840252	8.759509	7.698994	7.523375	7.124555
17377547	Gm14147	predicted gene 14147	2.686169	2.561395	2.414493	2.815732	2.690138	3.412053
17377553	Gm14149	predicted gene 14149	3.358344	3.596017	3.48073	2.733425	2.984957	3.339703
17392787	Gm14151	predicted gene 14151	8.24146	7.698994	7.843894	7.893855	5.927485	7.892597
17377627	Gm14155	predicted gene 14155 [Source:MGI Symbol;Acc:MGI:3651338]	3.342529	4.01475	3.739538	4.589573	4.205858	3.882376
17392965	Gm14161	predicted gene 14161	8.191797	5.8219	6.441024	6.992896	7.321352	6.524874
17377703	Gm14164	predicted gene 14164	8.467112	8.467112	7.009007	9.488237	8.858546	7.864909
17392828	Gm14167	predicted gene 14167	8.526715	10.22926	11.43465	11.02594	8.907743	9.354821
17393549	Gm14169	predicted gene 14169	14.13402	12.16463	11.44829	15.46152	11.96451	13.19482
17393535	Gm14170	predicted gene 14170	5.12201	4.281155	4.6664	4.212189	3.773335	3.56999
17393554	Gm14174	predicted gene 14174 [Source:MGI Symbol;Acc:MGI:3650931]	19.34703	16.66686	19.37199	21.47312	17.47645	21.88712

17269206	Gm14180	predicted gene 14180 [Source:MGI Symbol;Acc:MGI:3650017]	48.89246	56.76737	53.7894	42.04639	36.99504	52.45833
17269218	Gm14188	predicted gene 14188 [Source:MGI Symbol;Acc:MGI:3651817]	6.236515	11.81698	9.705887	10.0159	8.625571	7.517485
17269194	Gm14190	predicted gene 14190 [Source:MGI Symbol;Acc:MGI:3651811]	12.67163	15.08539	16.3311	12.26402	10.91523	16.73202
17256290	Gm14193	predicted gene 14193 [Source:MGI Symbol;Acc:MGI:3651818]	22.31528	24.1612	26.29634	24.04925	21.191	24.46182
17393019	Gm14199	predicted gene 14199	6.394626	10.49027	6.832625	7.560282	9.797543	7.106652
17393843	Gm14204	predicted gene 14204	4.123044	4.73515	3.182918	3.384259	3.739979	3.509462
17393851	Gm14205	predicted gene 14205 [Source:MGI Symbol;Acc:MGI:3702167]	7.870689	7.698994	6.790652	6.814841	8.702501	7.86698
17389855	Gm14207	predicted gene 14207	4.684094	7.647085	4.818987	6.941272	6.820323	5.189264
17376780	Gm14209	predicted gene 14209 [Source:MGI Symbol;Acc:MGI:3649779]	3.195585	3.25873	2.67402	3.261329	2.962222	2.953407
17392118	Gm14210	predicted gene 14210	2.692171	2.486765	2.508613	3.048839	2.844234	3.007845
17392115	Gm14211	predicted gene 14211 [Source:MGI Symbol;Acc:MGI:3652008]	4.591005	4.777878	4.76247	4.777878	5.185058	4.710499
17393187	Gm14216	predicted gene 14216	9.87171	9.206546	9.280934	9.571997	8.345972	9.87698
17376795	Gm14218	predicted gene 14218 [Source:MGI Symbol;Acc:MGI:3650517]	2.86801	2.655309	2.850194	2.681239	2.810662	2.798842
17393872	Gm14221	predicted gene 14221	6.661421	7.110051	7.309233	6.267717	9.238317	7.261663
17379008	Gm14222	predicted gene 14222 [Source:MGI Symbol;Acc:MGI:3651737]	3.497708	3.927714	3.624646	3.706532	4.20787	4.290423
17393854	Gm14223	predicted gene 14223 [Source:MGI Symbol;Acc:MGI:3650698]	3.062691	3.417142	3.062691	3.29457	3.339703	3.339703
17393546	Gm14224	predicted gene 14224 [Source:MGI Symbol;Acc:MGI:3649242]	4.467879	3.768368	3.768368	4.193224	3.802241	4.693259
17393543	Gm14225	predicted gene 14225 [Source:MGI Symbol;Acc:MGI:3649784]	21.85271	30.85901	26.04004	26.37428	27.44478	26.54963
17393906	Gm14228	predicted gene 14228 [Source:MGI Symbol;Acc:MGI:3649960]	2.67402	2.953407	2.491026	2.728768	3.073516	3.332743
17393562	Gm14230	predicted gene 14230	5.613576	6.789051	6.004316	6.004316	7.550279	5.009928
17376443	Gm14232	predicted gene 14232	4.224381	3.750648	3.456064	4.203934	3.143976	3.599958
17379978	Gm14236	predicted gene 14236 [Source:MGI Symbol;Acc:MGI:3651467]	6.282592	6.171709	6.756893	6.171709	5.120306	6.171709
17379110	Gm14241	predicted gene 14241 [Source:MGI Symbol;Acc:MGI:3651060]	5.337263	5.360249	5.614043	7.627604	5.389009	6.432636
17393957	Gm14242	predicted gene 14242 [Source:MGI Symbol;Acc:MGI:3651063]	2.867751	3.546854	3.546854	3.466308	3.450391	4.010744
17379106	Gm14243	predicted gene 14243 [Source:MGI Symbol;Acc:MGI:3651252]	2.718442	3.366758	2.815166	2.888239	3.416976	3.096375
17375929	Gm14244	predicted gene 14244	5.120306	4.791442	5.149135	5.595861	5.370178	4.604294
17380011	Gm14249	predicted gene 14249	10.08039	10.75466	11.76346	9.73135	9.573523	10.68022
17380007	Gm14250	predicted gene 14250	4.074604	4.283708	5.012117	4.709357	3.967884	6.284117
17393531	Gm14252	predicted gene 14252 [Source:MGI Symbol;Acc:MGI:3649403]	5.537405	6.456624	5.537405	6.015022	4.148558	6.347815
17394012	Gm14254	predicted gene 14254 [Source:MGI Symbol;Acc:MGI:3650986]	3.882296	3.825319	4.162038	3.684784	3.851846	3.840915
17394000	Gm14255	predicted gene 14255 [Source:MGI Symbol;Acc:MGI:3651202]	5.753501	5.582379	6.251056	6.171709	5.083739	6.290349
17393307	Gm14257	predicted gene 14257 [Source:MGI Symbol;Acc:MGI:3652145]	3.017178	2.998269	2.747368	2.791867	4.198983	2.53297
17380001	Gm14258	predicted gene 14258 [Source:MGI Symbol;Acc:MGI:3652141]	3.703322	3.391962	3.581465	3.703322	4.199245	4.299563
17394902	Gm14260	predicted gene 14260 [Source:MGI Symbol;Acc:MGI:3650157]	5.159209	5.58474	5.260573	7.907217	5.221159	6.793806
17379992	Gm14261	predicted gene 14261	2.591954	3.185213	2.691294	3.147006	3.100434	3.809839
17394803	Gm14262	predicted gene 14262 [Source:MGI Symbol;Acc:MGI:3651915]	13.64197	22.02195	17.36523	23.74267	17.36523	18.42394
17394989	Gm14264	predicted gene 14264	8.086077	8.730172	8.065254	9.157285	8.086077	11.20499
17380071	Gm14266	predicted gene 14266 [Source:MGI Symbol;Acc:MGI:3649850]	4.566979	4.965607	4.183006	4.682244	4.946129	4.682244
17394578	Gm14267	predicted gene 14267 [Source:MGI Symbol;Acc:MGI:3649851]	3.153054	3.961756	2.983571	3.4858	3.706095	4.197761
17394574	Gm14268	predicted gene 14268	10.21906	10.14305	12.3566	12.83979	10.21906	10.51947
17380085	Gm14271	predicted gene 14271 [Source:MGI Symbol;Acc:MGI:3649854]	7.713898	5.481193	4.647678	4.119759	5.732323	5.56106
17395003	Gm14272	predicted gene 14272 [Source:MGI Symbol;Acc:MGI:3651834]	3.009972	3.009972	3.009972	2.957422	3.009972	3.26916
17380096	Gm14273	predicted gene 14273	4.681043	5.074339	5.239794	3.824895	3.942061	5.453793
17380093	Gm14275	predicted gene 14275 [Source:MGI Symbol;Acc:MGI:3649537]	3.807806	2.336728	2.810935	3.038881	2.70519	2.909114
17391905	Gm14280	predicted gene 14280 [Source:MGI Symbol;Acc:MGI:3650415]	5.357352	5.762001	6.050649	6.413003	6.242743	5.914229
17376535	Gm14285	predicted gene 14285	10.37141	11.60496	10.26563	9.365007	9.973086	9.976543
17393752	Gm14286	predicted gene 14286 [Source:MGI Symbol;Acc:MGI:3650190]	6.710313	7.46944	9.855165	8.632868	7.897576	8.486855
17380399	Gm14288	predicted gene 14288	177.8565	224.0334	178.3256	183.0964	200.2905	185.561
17380472	Gm14288	predicted gene 14288	145.674	183.1015	143.9386	150.1354	155.7675	151.9887
17395277	Gm14288	predicted gene 14288	131.266	164.9357	132.8146	135.0276	138.0503	135.0631
17380518	Gm14295	predicted gene 14295	83.30147	82.80064	70.44522	85.16803	66.03825	85.42462
17380464	Gm14296	predicted gene 14296	48.37501	58.00639	38.6911	48.72465	43.28664	42.09013
17380466	Gm14296	predicted gene 14296	192.2287	193.1998	185.8404	196.5606	182.2279	205
17395359	Gm14296	predicted gene 14296	90.39869	89.26793	79.32535	79.63882	82.25492	77.94745
17548186	Gm14299	predicted gene 14299 [Source:MGI Symbol;Acc:MGI:3650442]	4.364813	5.921079	4.422401	4.511603	4.23159	3.66766
17394182	Gm14302	predicted gene 14302 [Source:MGI Symbol;Acc:MGI:3701953]	23.3806	25.71164	23.09454	24.35895	22.48311	20.42522
17380509	Gm14305	predicted gene 14305	24.76035	34.74106	30.30828	33.29445	35.89448	35.01012
17380427	Gm14308	predicted gene 14308	327.1866	351.4121	339.6417	315.84	306.7884	330.1185
17380434	Gm14308	predicted gene 14308	327.1866	351.4121	339.6417	315.84	306.7884	330.1185
17395251	Gm14308	predicted gene 14308	325.2601	346.3916	332.8412	318.6591	310.5023	339.6229
17395260	Gm14308	predicted gene 14308	219.4913	240.4378	244.6152	235.4326	238.9508	237.4385
17395345	Gm14308	predicted gene 14308	325.2173	367.5916	348.1536	326.2762	315.3172	347.0934
17379456	Gm14317	predicted gene 14317	5.447975	5.513812	7.127223	5.565109	5.24779	8.260849
17379957	Gm14319	predicted gene 14319 [Source:MGI Symbol;Acc:MGI:3649450]	5.015766	5.365416	5.350709	5.020566	6.599881	5.526745
17379949	Gm14321	predicted gene 14321	9.361307	9.028648	10.23337	10.7713	12.35806	7.835634
17380567	Gm14322	predicted gene 14322	5.120767	7.915772	8.588523	10.03132	7.539454	7.59837
17395304	Gm14325	predicted gene 14325	63.42106	119.3653	71.23084	74.1914	77.22896	84.11376
17380572	Gm14327	predicted gene 14327	12.69642	16.29119	18.56457	18.65577	16.70467	16.39643
17463797	Gm14330	predicted gene 14330	3.107986	2.942964	2.453328	2.720157	2.444501	2.697364
17395692	Gm14340	predicted gene 14340 [Source:MGI Symbol;Acc:MGI:3650817]	3.407964	3.784126	3.686214	3.520768	3.407964	3.784126
17380888	Gm14341	predicted gene 14341 [Source:MGI Symbol;Acc:MGI:3651041]	18.49649	19.66611	20.23415	18.30239	18.49649	20.75288
17395695	Gm14342	predicted gene 14342	5.40963	6.87219	6.089568	7.744006	5.87586	6.389619
17380868	Gm14344	predicted gene 14344	5.338965	5.548355	3.339703	4.048255	4.336316	4.062281
17532702	Gm14345	predicted gene 14345	2.7211	2.953407	3.005802	2.888499	3.579654	2.859157
17539740	Gm14346	predicted gene 14346	3.062691	3.54506	3.612957	3.821979	4.22239	3.757466
17532710	Gm14347	predicted gene 14347	2.655309	2.129434	2.654684	2.056313	3.241294	3.365078
17539744	Gm14351	predicted gene 14351	2.589871	2.689168	2.959616	2.933738	3.800344	2.860743
17498394	Gm14372	predicted gene 14372 [Source:MGI Symbol;Acc:MGI:3649263]	5.488873	4.827479	4.219377	5.379455	5.248736	5.215408
17532728	Gm14374	predicted gene 14374	2.162035	2.012599	1.747945	1.898013	1.966977	2.054191
17498423	Gm14376	predicted gene 14376 [Source:MGI Symbol;Acc:MGI:3651797]	4.146632	5.780603	4.647678	4.765904	5.720654	5.173964
17478268	Gm14377	predicted gene 14377	3.403927	3.975014	3.703322	4.148098	4.423566	4.610394
17532799	Gm14379	predicted gene 14379	2.866221	3.095173	2.540971	3.221048	3.175426	3.221048
17395189	Gm14393	predicted gene 14393	5.719345	5.662935	4.51179	4.426941	4.956941	4.035255
17372060	Gm14396	predicted gene 14396 [Source:MGI Symbol;Acc:MGI:3649369]	18.95846	17.10659	19.83409	13.22119	19.1127	24.5825

17379674	Gm14397	predicted gene 14397 [Source:MGI Symbol;Acc:MGI:3649371]	10.69979	9.403645	10.10138	10.61079	9.362994	9.149672
17395227	Gm14399	predicted gene 14399 [Source:MGI Symbol;Acc:MGI:3650082]	183.1333	212.1896	201.1879	193.9808	187.4718	193.1797
17380544	Gm14401	predicted gene 14401 [Source:MGI Symbol;Acc:MGI:3650075]	3.243339	2.769067	3.552095	2.658975	2.591846	2.885297
17395298	Gm14402	predicted gene 14402 [Source:MGI Symbol;Acc:MGI:3649812]	3.861095	3.04259	3.339703	3.265469	3.04413	3.315623
17380555	Gm14403	predicted gene 14403	16.45073	38.73511	19.58219	29.04343	34.03813	27.65196
17380468	Gm14405	predicted gene 14405	4.986439	6.026874	4.616694	4.440863	5.552587	4.886393
17395354	Gm14405	predicted gene 14405	30.78637	46.37868	31.26074	32.58893	40.0289	37.44004
17395389	Gm14406	predicted gene 14406 [Source:MGI Symbol;Acc:MGI:3649838]	20.74052	12.93833	14.00005	21.23276	15.10443	22.20865
17395366	Gm14407	predicted gene 14407 [Source:MGI Symbol;Acc:MGI:3649809]	51.80502	70.65942	83.62602	84.33912	57.9425	85.73842
17395376	Gm14409	predicted gene 14409	3.19574	1.958162	2.024394	2.304206	3.482444	3.052413
17395379	Gm14412	predicted gene 14412 [Source:MGI Symbol;Acc:MGI:3652251]	20.12602	32.34588	28.63346	30.08787	25.00787	12.76888
17395382	Gm14418	predicted gene 14418 [Source:MGI Symbol;Acc:MGI:3702408]	24.31627	18.75509	18.80312	17.25413	26.53326	21.22128
17380549	Gm14420	predicted gene 14420	45.46486	84.9022	55.88271	65.29452	67.24849	50.63279
17372111	Gm14426	predicted gene 14426 [Source:MGI Symbol;Acc:MGI:3652025]	6.472576	6.059967	6.298769	4.898126	6.427154	7.099267
17380410	Gm14431	predicted gene 14431	97.7639	111.0488	90.75467	96.56845	94.48871	95.49412
17380491	Gm14431	predicted gene 14431	148.7381	184.636	146.9661	161.5156	161.3668	153.5889
17395272	Gm14431	predicted gene 14431	148.7381	184.636	146.9661	161.5156	161.3668	153.5889
17395290	Gm14431	predicted gene 14431	195.5807	247.2746	193.2507	209.861	200.142	199.1884
17380418	Gm14436	predicted gene 14436	123.7951	154.3017	135.3368	134.6957	148.0027	131.6424
17380496	Gm14436	predicted gene 14436	94.30615	107.0232	97.80186	95.75262	102.702	90.65282
17379680	Gm14437	predicted gene 14437	4.681043	4.105961	4.362303	4.23206	4.90047	4.17892
17380441	Gm14442	predicted gene 14442 [Source:MGI Symbol;Acc:MGI:3652323]	20.974	25.36118	23.98965	23.05152	24.26004	24.06109
17395193	Gm14443	predicted gene 14443	3.059424	2.906595	4.02732	3.120701	2.519714	2.994718
17380392	Gm14444	predicted gene 14444 [Source:MGI Symbol;Acc:MGI:3652325]	3.485728	2.252583	2.608897	2.375416	3.488701	2.474385
17395060	Gm14453	predicted gene 14453 [Source:MGI Symbol;Acc:MGI:3702165]	20.56002	17.72962	21.27534	25.92281	27.0585	25.84261
17395029	Gm14455	predicted gene 14455 [Source:MGI Symbol;Acc:MGI:3711286]	6.176331	8.325114	7.076569	6.943774	6.483532	7.713844
17540102	Gm14459	predicted gene 14459	2.274706	3.032547	3.377112	1.990471	5.578279	3.248694
17372284	Gm14461	predicted gene 14461	3.529956	3.373158	2.977291	3.53042	3.754368	3.280828
17383616	Gm14471	predicted gene 14471 [Source:MGI Symbol;Acc:MGI:3650368]	4.01461	4.209503	4.72954	5.110378	4.854659	4.209503
17533294	Gm14474	predicted gene 14474	9.223856	10.88855	9.993084	12.1902	11.07397	11.05993
17533303	Gm14477	predicted gene 14477	8.37663	12.55308	10.23862	13.13278	11.20134	13.50227
17533316	Gm14477	predicted gene 14477	7.913413	10.80487	10.09326	11.17149	11.3489	12.59911
17533322	Gm14477	predicted gene 14477	7.913413	10.80487	10.09326	11.17149	11.3489	12.59911
17533328	Gm14477	predicted gene 14477	9.045498	10.8642	9.30967	12.03088	10.53801	12.35866
17533289	Gm14483	predicted gene 14483	9.237036	11.85686	10.36056	13.04007	11.0821	12.33623
17533307	Gm14484	predicted gene 14484	10.64748	15.3377	13.16687	15.6855	17.61253	13.73895
17383667	Gm14486	predicted gene 14486	4.307736	4.272884	4.234437	4.802049	4.70903	4.157194
17369252	Gm14487	predicted gene 14487	14.76756	17.47766	14.79564	14.97506	12.33563	13.23485
17383672	Gm14488	predicted gene 14488	3.008262	4.545881	3.703322	3.703322	5.566189	2.76617
17540240	Gm14493	predicted gene 14493 [Source:MGI Symbol;Acc:MGI:3705313]	11.50078	8.704853	9.012704	11.73577	10.43993	10.00951
17381190	Gm14496	predicted gene 14496	1.942857	2.220785	2.098313	2.220785	2.353487	2.424521
17533219	Gm14501	predicted gene 14501	14.57749	16.8183	13.11467	17.68348	20.13802	14.84515
17533466	Gm14505	predicted gene 14505 [Source:MGI Symbol;Acc:MGI:3705173]	3.693324	4.293577	3.83384	3.845923	4.449675	3.662478
17441337	Gm14507	predicted gene 14507 [Source:MGI Symbol;Acc:MGI:3705240]	2.406006	2.953407	2.826585	2.084339	1.909321	2.372339
17441331	Gm14508	predicted gene 14508 [Source:MGI Symbol;Acc:MGI:3705279]	4.661163	4.83086	5.348929	5.386134	5.386096	4.071894
17548834	Gm14517	predicted gene 14517	2.507744	2.260152	2.103774	2.448622	2.419083	2.251147
17456988	Gm14532	predicted gene 14532	2.641651	3.085598	2.320282	2.614871	3.387263	2.854761
17456991	Gm14532	predicted gene 14532	6.314638	3.54506	3.627913	3.326811	4.694918	3.406854
17457060	Gm14540	predicted gene 14540 [Source:MGI Symbol;Acc:MGI:3705140]	4.019595	3.924095	4.589382	4.20927	5.314743	4.20927
17457125	Gm14546	predicted gene 14546	4.159038	3.515573	4.877257	5.751666	4.417132	4.614614
17465735	Gm14547	predicted gene 14547 [Source:MGI Symbol;Acc:MGI:3705289]	8.222232	8.115781	6.949697	8.116456	7.625225	9.613488
17485609	Gm14548	predicted gene 14548	2.796205	2.831154	3.800818	3.01699	2.682503	3.00118
17534119	Gm14549	predicted gene 14549 [Source:MGI Symbol;Acc:MGI:3705175]	4.405085	4.151607	4.721908	3.628668	5.038241	5.720099
17540870	Gm14552	predicted gene 14552 [Source:MGI Symbol;Acc:MGI:3801915]	2.566905	2.617374	2.4519	2.685685	2.536009	2.644363
17540914	Gm14552	predicted gene 14552 [Source:MGI Symbol;Acc:MGI:3801915]	2.978343	2.951462	3.078206	3.81404	2.803385	2.644436
17540929	Gm14553	predicted gene 14553 [Source:MGI Symbol;Acc:MGI:3801869]	2.159459	2.331276	3.493831	3.896179	2.294484	2.485903
17541222	Gm14565	predicted gene 14565 [Source:MGI Symbol;Acc:MGI:3705304]	4.350587	4.419827	3.861041	4.485162	4.158375	5.576114
17548806	Gm14567	predicted gene 14567 [Source:MGI Symbol;Acc:MGI:3705732]	58.85447	65.05283	71.01097	93.62488	86.87927	86.00866
17540937	Gm14569	predicted gene 14569	2.891484	5.084726	3.894343	2.647113	3.07211	4.79513
17469013	Gm14573	predicted gene 14573 [Source:MGI Symbol;Acc:MGI:3705150]	11.29115	13.21542	14.33162	11.71537	10.20561	11.71537
17534691	Gm14582	predicted gene 14582 [Source:MGI Symbol;Acc:MGI:3705178]	3.080191	2.836241	2.791325	2.843012	2.975475	2.428087
17541713	Gm14586	predicted gene 14586	232.0529	260.9591	296.1389	257.345	327.1322	315.9801
17534813	Gm14590	predicted gene 14590	2.992518	2.351808	2.204528	2.356491	3.066749	2.328386
17534791	Gm14594	predicted gene 14594	2.662257	2.549139	2.213869	2.402872	2.904943	2.383877
17534806	Gm14595	predicted gene 14595	2.992518	2.351808	2.204528	2.356491	3.066749	2.328386
17534774	Gm14596	predicted gene 14596	2.959472	2.343346	2.170459	2.378348	3.055694	2.426556
17541769	Gm14597	predicted gene 14597 [Source:MGI Symbol;Acc:MGI:3711766]	4.647678	3.263862	5.389644	3.059763	4.138965	4.046151
17534767	Gm14598	predicted gene 14598	3.039787	2.360643	2.386671	2.50691	3.092321	2.429888
17541818	Gm14601	predicted gene 14601 [Source:MGI Symbol;Acc:MGI:3705772]	5.400863	5.771595	5.108961	4.873269	6.523562	6.124279
17380388	Gm14616	predicted gene 14616	5.98458	5.917632	6.242916	5.98458	7.87189	6.327695
17395182	Gm14617	predicted gene 14617 [Source:MGI Symbol;Acc:MGI:3705092]	11.71756	12.35189	12.35189	12.35189	14.07156	11.2634
17395186	Gm14618	predicted gene 14618 [Source:MGI Symbol;Acc:MGI:3705277]	4.01461	3.363726	4.01461	3.857617	4.603433	3.610003
17534849	Gm14625	predicted gene 14625	3.015046	2.443925	2.262974	2.726008	3.03053	2.566603
17533350	Gm14634	predicted gene 14634	4.986405	4.442895	5.794805	5.233405	5.042293	4.916563
17540267	Gm14635	predicted gene 14635	5.886418	6.965322	5.357537	5.316807	4.881586	5.854679
17380057	Gm14637	predicted gene 14637	5.30624	4.801649	5.049673	4.347663	4.772218	3.726755
17380079	Gm14640	predicted gene 14640 [Source:MGI Symbol;Acc:MGI:3705244]	3.135618	2.96366	2.821046	3.615662	2.86112	2.953407
17395006	Gm14641	predicted gene 14641 [Source:MGI Symbol;Acc:MGI:3705300]	6.25435	4.601136	5.954003	6.961702	5.896954	6.819661
17380240	Gm14642	predicted gene 14642	2.474642	2.493704	2.805115	3.225471	2.902407	2.527936
17534376	Gm14643	predicted gene 14643 [Source:MGI Symbol;Acc:MGI:3705095]	2.682429	2.658827	2.649426	2.962325	2.183193	3.218347
17535445	Gm14684	predicted gene 14684 [Source:MGI Symbol;Acc:MGI:3705272]	4.448472	6.100807	4.647678	5.386134	4.899383	5.815689
17535480	Gm14685	predicted gene 14685	11.41089	8.720555	9.339401	8.899103	8.720555	10.692
17541516	Gm14696	predicted gene 14696	2.882419	2.768415	2.770526	2.996233	2.474196	2.768415
17535241	Gm14698	predicted gene 14698 [Source:MGI Symbol;Acc:MGI:3709613]	4.808144	8.629578	7.910506	7.177692	6.123642	7.813939

17539875	Gm14703	predicted gene 14703 [Source:MGI Symbol;Acc:MGI:3705165]	3.12607	3.695783	3.744726	3.845364	4.558187	4.153622
17542135	Gm14705	predicted gene 14705 [Source:MGI Symbol;Acc:MGI:3705152]	3.339703	3.339703	3.395679	3.733219	4.064365	2.876272
17542881	Gm14707	predicted gene 14707	3.2408	3.08793	2.845042	2.896097	2.458673	3.083802
17542878	Gm14715	predicted gene 14715 [Source:MGI Symbol;Acc:MGI:3705290]	2.805212	2.938195	2.953407	3.867497	2.670463	3.014461
17535045	Gm14718	predicted gene 14718	2.719271	3.162444	3.139707	2.607939	2.782767	2.172927
17534665	Gm14719	predicted gene 14719	321.2672	287.1041	324.9694	327.071	327.2116	283.9258
17535280	Gm14725	predicted gene 14725	5.602109	3.270543	4.129165	4.909411	3.903829	3.967912
17542913	Gm14742	predicted gene 14742 [Source:MGI Symbol;Acc:MGI:3705303]	3.329303	3.211372	2.712607	3.211372	2.833274	3.396644
17542941	Gm14743	predicted gene 14743	2.363558	2.480945	2.230473	3.024046	2.26139	2.907582
17536028	Gm14744	predicted gene 14744	3.965163	3.75031	4.116905	4.514324	5.743101	4.187357
17536036	Gm14750	predicted gene 14750 [Source:MGI Symbol;Acc:MGI:3705827]	2.1256	2.375317	2.15729	2.013046	2.324934	1.852791
17536187	Gm14762	predicted gene 14762	3.773936	3.42929	2.727294	3.636952	3.392268	3.595768
17536184	Gm14764	predicted gene 14764 [Source:MGI Symbol;Acc:MGI:3705248]	4.65348	4.558882	5.740276	3.299712	4.447208	5.224417
17536225	Gm14781	predicted gene 14781	1.962836	1.964456	1.740799	2.384638	3.137097	2.566514
17536292	Gm14798	predicted gene 14798 [Source:MGI Symbol;Acc:MGI:3705098]	5.036914	5.523949	4.613559	5.357834	5.488683	3.912227
17536492	Gm14808	predicted gene 14808	6.093537	5.048763	5.267798	6.009104	5.364635	6.397419
17543446	Gm14809	predicted gene 14809	3.504819	4.167205	4.333161	4.036095	3.900692	4.718702
17543438	Gm14812	predicted gene 14812	3.363259	4.344108	2.776178	3.682537	3.607552	3.717338
17548867	Gm14816	predicted gene 14816 [Source:MGI Symbol;Acc:MGI:3705674]	12.35189	9.941338	17.01918	13.96003	12.99919	12.99919
17535707	Gm14817	predicted gene 14817 [Source:MGI Symbol;Acc:MGI:3705159]	3.584669	5.597529	4.846859	2.747577	4.495931	2.673742
17536346	Gm14818	predicted gene 14818 [Source:MGI Symbol;Acc:MGI:3801996]	2.983447	3.356063	2.940354	3.556628	3.342065	3.231581
17533073	Gm14820	predicted gene 14820 [Source:MGI Symbol;Acc:MGI:3713695]	3.822509	3.635692	3.912037	4.407376	4.358404	4.160913
17536324	Gm14827	predicted gene 14827	3.791766	5.649566	3.249483	4.204808	4.340316	4.753038
17499765	Gm14850	predicted gene 14850	4.745138	3.760436	4.334694	4.341369	4.76792	3.95191
17499780	Gm14850	predicted gene 14850	4.949319	4.55386	4.647678	4.567453	5.544702	4.196743
17507901	Gm14851	predicted gene 14851	3.294844	3.94176	3.743687	3.254193	2.920246	4.017506
17536887	Gm14858	predicted gene 14858	6.579145	5.249937	4.315686	5.076274	5.120306	5.076274
17543491	Gm14866	predicted gene 14866 [Source:MGI Symbol;Acc:MGI:3704514]	6.205167	7.948375	6.934058	6.869523	6.934058	8.420524
17536620	Gm14902	predicted gene 14902	3.166063	3.099746	2.793202	2.939478	2.99451	2.889861
17536381	Gm14925	predicted gene 14925 [Source:MGI Symbol;Acc:MGI:3801742]	2.003344	2.393928	2.209852	2.223727	2.474488	1.979464
17544279	Gm14929	predicted gene 14929 [Source:MGI Symbol;Acc:MGI:3705167]	2.406006	2.892266	2.944495	2.691387	3.637658	2.580839
17544239	Gm14936	predicted gene 14936	2.391093	2.160223	2.669556	2.275447	2.380113	3.248312
17544308	Gm14950	predicted gene 14950	1.87777	1.898013	2.067131	2.156363	2.38396	2.366098
17537388	Gm14951	predicted gene 14951 [Source:MGI Symbol;Acc:MGI:3705251]	4.05986	3.832458	3.832458	3.915455	2.87691	4.01667
17362028	Gm14964	predicted gene 14964 [Source:MGI Symbol;Acc:MGI:3641621]	12.57355	15.95183	13.48422	13.54727	11.38759	14.13167
17362024	Gm14965	predicted gene 14965	4.91537	6.324362	6.083451	6.533699	6.324362	7.169189
17362044	Gm14967	predicted gene 14967 [Source:MGI Symbol;Acc:MGI:3705318]	4.681043	5.443936	4.596992	3.322594	5.71356	4.098767
17356958	Gm14968	predicted gene 14968 [Source:MGI Symbol;Acc:MGI:3705315]	4.737382	4.034584	5.617429	6.356577	4.886969	4.544212
17390485	Gm14978	predicted gene 14978 [Source:MGI Symbol;Acc:MGI:3705122]	5.048553	4.125817	4.252467	4.374506	4.352698	5.152669
17548879	Gm14989	predicted gene 14989 [Source:MGI Symbol;Acc:MGI:3705366]	34.82086	27.49448	27.49448	22.41667	27.97275	21.20129
17534130	Gm15008	predicted gene 15008	3.113398	2.640718	2.441402	2.361052	2.787245	2.441402
17537808	Gm15015	predicted gene 15015 [Source:MGI Symbol;Acc:MGI:3709319]	2.251131	2.03307	2.22456	2.398679	2.243172	1.978641
17544611	Gm15023	predicted gene 15023	2.761592	3.066728	2.965715	3.136423	3.680062	3.17151
17537975	Gm15026	predicted gene 15026 [Source:MGI Symbol;Acc:MGI:3705439]	4.016614	4.917858	6.464729	4.266698	6.797668	5.56106
17545075	Gm15032	predicted gene 15032	4.043095	3.374023	4.450693	4.551115	4.776587	4.423229
17544936	Gm15046	predicted gene 15046 [Source:MGI Symbol;Acc:MGI:3705253]	5.775877	5.718592	4.847531	6.106476	5.9071	4.466702
17458602	Gm15050	predicted gene 15050 [Source:MGI Symbol;Acc:MGI:3705180]	5.973556	6.363718	6.289695	7.389773	6.381829	6.24915
17507882	Gm15056	predicted gene 15056	2.244058	2.259432	2.421028	2.51044	3.331426	2.820264
17535984	Gm15063	predicted gene 15063	2.11162	2.765193	2.313505	2.38277	2.854504	3.339703
17548881	Gm15067	predicted gene 15067 [Source:MGI Symbol;Acc:MGI:3705530]	2.24283	2.257788	2.126219	2.206806	2.67695	2.351954
17538452	Gm15080	predicted gene 15080	3.943537	2.728277	2.585835	3.019019	3.761375	3.770877
17461845	Gm15082	predicted gene 15082 [Source:MGI Symbol;Acc:MGI:3705124]	3.152442	3.220199	4.229978	3.583055	3.697935	4.482147
17461848	Gm15083	predicted gene 15083	3.437382	3.417331	3.437382	3.131578	3.123579	3.437382
17545269	Gm15086	predicted gene 15086 [Source:MGI Symbol;Acc:MGI:3705246]	3.439336	3.316634	3.548551	3.491314	3.316634	3.479741
17545273	Gm15086	predicted gene 15086 [Source:MGI Symbol;Acc:MGI:3705246]	3.439336	3.316634	3.548551	3.491314	3.316634	3.479741
17538562	Gm15091	predicted gene 15091	5.807471	6.010063	5.097677	5.294887	6.95225	6.404207
17545259	Gm15092	predicted gene 15092 [Source:MGI Symbol;Acc:MGI:3705242]	2.303376	2.075041	2.809065	2.361543	2.684922	1.969282
17538505	Gm15093	predicted gene 15093	6.015939	5.939041	5.196175	6.518274	7.474619	7.002305
17538518	Gm15093	predicted gene 15093	6.981369	6.892083	6.03005	7.021504	8.674136	8.126026
17538528	Gm15093	predicted gene 15093	6.956974	6.868	6.521392	7.537886	10.16248	8.025437
17538549	Gm15097	predicted gene 15097	6.83978	4.077034	3.51923	4.354388	4.067775	5.530424
17545253	Gm15099	predicted gene 15099 [Source:MGI Symbol;Acc:MGI:3705260]	2.878266	1.976777	2.72633	2.329451	4.479414	3.568382
17545287	Gm15104	predicted gene 15104	2.752915	1.769069	2.131014	2.521164	2.546772	2.095784
17538461	Gm15107	predicted gene 15107	4.342573	3.992659	3.7776	4.287599	5.182506	4.270633
17538470	Gm15109	predicted gene 15109	5.370253	5.304953	4.69095	5.48933	6.820323	6.389354
17538477	Gm15114	predicted gene 15114	2.095101	2.046743	2.328418	2.215174	2.799696	2.965736
17242853	Gm15122	predicted gene 15122 [Source:MGI Symbol;Acc:MGI:3705268]	2.77714	2.77714	2.724724	2.802744	2.489766	2.952301
17235347	Gm15123	predicted gene 15123 [Source:MGI Symbol;Acc:MGI:3705256]	3.954074	4.956116	6.246768	6.151337	4.075198	4.395173
17538486	Gm15127	predicted gene 15127	3.016467	3.331888	2.778623	3.261623	4.695927	2.606909
17538441	Gm15128	predicted gene 15128	2.608807	4.136126	3.752365	3.981605	3.903396	3.95013
17481297	Gm15133	predicted gene 15133	4.176285	4.202652	4.801083	3.666288	4.112142	4.295452
17494322	Gm15135	predicted gene 15135 [Source:MGI Symbol;Acc:MGI:3705309]	2.602467	3.009972	3.727737	2.992062	3.255525	3.266555
17232199	Gm15137	predicted gene 15137 [Source:MGI Symbol;Acc:MGI:3705316]	3.994846	4.753269	3.550347	4.012552	3.639474	3.598561
17545518	Gm15140	predicted gene 15140	2.162035	2.280168	2.11263	2.132832	2.050392	2.121346
17539046	Gm15142	predicted gene 15142	2.394725	2.690492	4.087645	2.989118	3.541499	2.606909
17545514	Gm15143	predicted gene 15143	3.393444	2.553135	2.00215	2.127236	2.340408	2.504999
17539086	Gm15156	predicted gene 15156 [Source:MGI Symbol;Acc:MGI:3705293]	15.7618	13.87206	15.04904	12.37443	12.63392	13.61816
17545708	Gm15169	predicted gene 15169	4.512822	4.667565	4.667565	5.683777	4.667565	7.041438
17224614	Gm15178	predicted gene 15178 [Source:MGI Symbol;Acc:MGI:3705132]	4.299623	4.953094	5.640488	5.158208	4.358434	5.481345
17224608	Gm15179	predicted gene 15179	5.026904	5.844988	5.841343	6.138644	7.009055	6.961895
17214650	Gm15182	predicted gene 15182 [Source:MGI Symbol;Acc:MGI:3705533]	2.731569	2.287894	2.838168	2.731569	3.150236	2.982165
17547539	Gm15197	predicted gene 15197 [Source:MGI Symbol;Acc:MGI:3708123]	4.916426	6.062782	4.679745	5.329684	5.043621	6.011854
17240577	Gm15199	predicted gene 15199	13.18961	12.19045	20.39961	13.30835	7.148844	14.19351
17240572	Gm15200	predicted gene 15200 [Source:MGI Symbol;Acc:MGI:3705136]	6.287864	6.54907	6.287864	6.489962	7.353545	6.17342

17545862	Gm15201	predicted gene 15201	4.295809	6.654868	5.681965	5.331199	6.460384	5.480206
17539400	Gm15202	predicted gene 15202 [Source:MGI Symbol;Acc:MGI:3705278]	18.17886	21.91596	15.3918	19.33818	16.78627	20.23036
17545817	Gm15205	predicted gene 15205	2.568347	2.229113	2.429922	2.16517	2.158008	2.317774
17283868	Gm15208	predicted gene 15208 [Source:MGI Symbol;Acc:MGI:3705292]	2.512546	2.822786	2.547284	2.854187	2.508318	2.850016
17299250	Gm15217	predicted gene 15217	4.087334	4.695439	4.678675	4.368112	3.921784	3.944384
17299243	Gm15218	predicted gene 15218 [Source:MGI Symbol;Acc:MGI:3705128]	2.175369	2.420844	2.67402	2.205476	3.007824	2.420844
17299261	Gm15221	predicted gene 15221 [Source:MGI Symbol;Acc:MGI:3705247]	4.002746	4.715171	4.21546	4.086767	4.597756	4.249196
17546070	Gm15226	predicted gene 15226 [Source:MGI Symbol;Acc:MGI:3705187]	3.762313	3.859944	3.660321	4.571983	4.151694	4.055922
17539646	Gm15228	predicted gene 15228 [Source:MGI Symbol;Acc:MGI:3705118]	3.36277	2.602936	2.809104	3.720741	3.1137	2.953407
17546073	Gm15230	predicted gene 15230	2.500645	2.500645	2.344337	2.569486	2.421414	2.534498
17546088	Gm15232	predicted gene 15232	3.878584	2.734733	2.831782	3.002408	2.444637	3.471526
17400121	Gm15234	predicted gene 15234 [Source:MGI Symbol;Acc:MGI:3705137]	3.104154	3.31475	3.738631	3.358879	3.756086	2.993742
17539653	Gm15239	predicted gene 15239	3.808994	2.964153	2.931888	3.335377	3.608052	3.248807
17545756	Gm15241	predicted gene 15241	2.274958	2.240732	2.312767	2.312767	2.230334	2.274659
17545761	Gm15243	predicted gene 15243 [Source:MGI Symbol;Acc:MGI:3705116]	3.426748	3.426748	3.952485	3.426748	4.109663	4.139223
17539693	Gm15245	predicted gene 15245	2.028369	2.20062	2.291512	2.450405	2.199004	2.782169
17546219	Gm15247	predicted gene 15247 [Source:MGI Symbol;Acc:MGI:3705183]	2.919194	3.457981	2.874	3.512602	2.632092	3.474728
17546175	Gm15261	predicted gene 15261 [Source:MGI Symbol;Acc:MGI:3705201]	3.807621	3.883991	3.81441	4.098114	6.982434	6.347815
17539391	Gm15262	predicted gene 15262 [Source:MGI Symbol;Acc:MGI:3705232]	3.312637	3.874902	3.12399	3.317784	3.75096	3.347514
17548885	Gm15266	predicted gene 15266	2.67402	2.67402	3.009972	3.009972	2.836064	3.009972
17396504	Gm1527	predicted gene 1527	2.606728	2.308267	2.255364	2.731323	2.273949	2.622622
17232231	Gm15271	predicted gene 15271 [Source:MGI Symbol;Acc:MGI:3705206]	5.813915	4.502949	5.813915	6.336649	4.956975	5.35802
17281911	Gm15283	predicted gene 15283	4.094508	3.929539	4.187357	4.257864	4.387594	4.392432
17289817	Gm15286	predicted gene 15286	2.67402	2.629084	3.006594	2.691201	2.709901	3.227372
17289840	Gm15287	predicted gene 15287 [Source:MGI Symbol;Acc:MGI:3705156]	6.287864	6.493202	5.282167	6.944833	4.852271	6.287864
17296164	Gm15288	predicted gene 15288 [Source:MGI Symbol;Acc:MGI:3705195]	2.435216	2.420844	2.481049	2.748434	2.458673	2.481049
17296161	Gm15290	predicted gene 15290	5.684211	3.819755	3.442341	4.813913	6.22274	4.247165
17499753	Gm15292	predicted gene 15292	12.02579	13.23247	19.82886	21.5405	16.25284	18.53898
17499748	Gm15293	predicted gene 15293	3.1498	3.171137	2.890562	3.167753	2.403772	3.435262
17538281	Gm15295	predicted gene 15295 [Source:MGI Symbol;Acc:MGI:3705286]	4.656416	4.16348	5.120306	4.801649	5.938094	4.621368
17545020	Gm15298	predicted gene 15298 [Source:MGI Symbol;Acc:MGI:3705276]	8.32927	9.124845	9.406513	12.26768	10.76085	6.88535
17499796	Gm15315	predicted gene 15315	15.49037	19.35078	20.28287	13.20059	14.16973	16.26637
17332454	Gm15317	predicted gene 15317 [Source:MGI Symbol;Acc:MGI:3705262]	9.858649	9.075958	9.361307	10.37371	13.05481	9.855985
17499650	Gm15319	predicted gene 15319	3.673181	3.443821	3.604652	4.273772	3.706378	4.014304
17507860	Gm15319	predicted gene 15319	3.804403	3.195873	3.677002	3.86806	4.097688	3.86806
17289857	Gm15322	predicted gene 15322	3.331242	3.926762	3.780508	4.364329	4.197441	3.863424
17289860	Gm15323	predicted gene 15323	6.448797	6.427332	6.915914	7.812585	9.729934	8.514489
17289848	Gm15324	predicted gene 15324 [Source:MGI Symbol;Acc:MGI:3705211]	4.871615	4.894291	3.520084	6.787122	4.78507	3.757961
17296200	Gm15325	predicted gene 15325	4.138965	3.862594	3.740004	3.693888	4.246694	4.18031
17289851	Gm15326	predicted gene 15326	8.736407	9.79829	9.526591	8.664161	7.185358	9.965139
17289843	Gm15327	predicted gene 15327	3.039281	3.616866	3.843125	3.703322	3.843125	3.935754
17348684	Gm15328	predicted gene 15328	3.691021	3.522794	3.406783	4.224762	4.920551	3.666821
17353973	Gm15334	predicted gene 15334 [Source:MGI Symbol;Acc:MGI:3705284]	15.19137	10.35833	11.17629	10.46819	11.69135	11.84339
17353976	Gm15336	predicted gene 15336 [Source:MGI Symbol;Acc:MGI:3705096]	5.713798	5.109882	6.178986	4.99135	5.254232	6.443792
17353980	Gm15337	predicted gene 15337	2.79543	3.54506	3.465347	3.339889	2.780415	3.736092
17482914	Gm15338	predicted gene 15338 [Source:MGI Symbol;Acc:MGI:3708108]	2.892947	2.592708	3.219574	2.776486	3.026667	2.442553
17496031	Gm15339	predicted gene 15339 [Source:MGI Symbol;Acc:MGI:3705108]	13.99714	9.872736	10.03536	9.824473	7.194927	12.5264
17327479	Gm15340	predicted gene 15340 [Source:MGI Symbol;Acc:MGI:3705158]	6.104571	4.81448	5.527741	4.691535	5.48933	6.443792
17327487	Gm15341	predicted gene 15341 [Source:MGI Symbol;Acc:MGI:3705258]	11.48015	14.17159	11.05242	11.91949	13.18276	13.86668
17327482	Gm15342	predicted gene 15342 [Source:MGI Symbol;Acc:MGI:3705188]	10.16438	6.82666	6.738885	6.82666	7.11804	6.82666
17242145	Gm15343	predicted gene 15343	59.80773	35.77575	45.86272	61.34356	50.21199	50.30256
17350754	Gm15345	predicted gene 15345	14.48542	17.09864	15.45498	19.24791	17.33445	20.13593
17499959	Gm15346	predicted gene 15346	4.571793	5.516296	4.681043	4.573853	4.871412	5.132529
17507524	Gm15347	predicted gene 15347 [Source:MGI Symbol;Acc:MGI:3705310]	8.768925	8.678165	9.081181	9.847312	8.157912	9.183792
17507515	Gm15348	predicted gene 15348	4.211196	3.567838	4.193079	3.39255	3.420933	3.805194
17507520	Gm15350	predicted gene 15350	4.01461	4.071612	4.01461	4.36678	3.978405	4.261791
17507532	Gm15351	predicted gene 15351	7.868944	7.214003	6.682649	8.080194	7.331864	5.56106
17509691	Gm15354	predicted gene 15354 [Source:MGI Symbol;Acc:MGI:3705093]	3.004198	3.235862	3.276124	2.894873	2.458673	3.009972
17509694	Gm15356	predicted gene 15356 [Source:MGI Symbol;Acc:MGI:3705102]	2.659878	3.265469	2.797465	3.359531	3.111219	2.757345
17535926	Gm15363	predicted gene 15363	4.427677	5.570435	6.188244	4.681043	6.030928	4.235816
17535982	Gm15384	predicted gene 15384 [Source:MGI Symbol;Acc:MGI:3705249]	2.587161	2.640278	2.393298	2.369282	2.167091	3.091549
17221536	Gm15386	predicted gene 15386	2.406006	2.492425	2.16235	2.408091	2.435925	3.032484
17216490	Gm15389	predicted gene 15389	5.030563	4.685977	4.369384	5.159138	4.487525	4.861023
17226120	Gm15391	predicted gene 15391 [Source:MGI Symbol;Acc:MGI:3705314]	2.920287	3.00012	2.581572	3.048966	2.968101	2.920287
17226361	Gm15392	predicted gene 15392 [Source:MGI Symbol;Acc:MGI:3705317]	4.395204	5.69403	4.829521	4.228072	5.48933	5.310398
17505934	Gm15395	predicted gene 15395	5.765797	7.844057	5.663995	5.873539	5.997245	4.794964
17490324	Gm15396	predicted gene 15396	10.45086	8.924987	9.788108	9.205589	12.97965	9.232171
17234210	Gm15397	predicted gene 15397 [Source:MGI Symbol;Acc:MGI:3705298]	2.484043	2.393646	2.393646	2.180109	2.744094	2.744094
17241806	Gm15398	predicted gene 15398 [Source:MGI Symbol;Acc:MGI:3705254]	4.469892	3.51667	5.145246	3.204657	3.234001	3.331981
17459696	Gm15401	predicted gene 15401	4.038725	4.179499	4.402582	4.561569	4.224996	5.042817
17459706	Gm15402	predicted gene 15402 [Source:MGI Symbol;Acc:MGI:3707435]	4.364602	4.146546	4.642301	5.518935	4.345072	3.140621
17340401	Gm15404	predicted gene 15404 [Source:MGI Symbol;Acc:MGI:3705186]	2.191305	2.815225	2.316345	2.644143	2.85997	2.508996
17444920	Gm15407	predicted gene 15407 [Source:MGI Symbol;Acc:MGI:3705245]	8.440392	7.78221	7.319626	7.984433	7.926368	10.13736
17444933	Gm15408	predicted gene 15408	7.822935	13.05744	15.96455	16.33665	22.76775	20.20948
17444930	Gm15409	predicted gene 15409 [Source:MGI Symbol;Acc:MGI:3705229]	17.71365	15.64283	18.01766	16.52708	20.38576	18.53692
17455459	Gm15411	predicted gene 15411	7.892487	9.938375	8.403382	6.881199	9.17552	7.892487
17493382	Gm15412	predicted gene 15412	6.557837	7.269872	9.635036	6.991781	5.650858	7.606116
17493390	Gm15413	predicted gene 15413	5.753022	5.61813	5.3008	5.770275	6.24257	6.006106
17493386	Gm15414	predicted gene 15414 [Source:MGI Symbol;Acc:MGI:3705307]	4.722039	5.780603	4.716053	5.225395	5.295732	7.164917
17493400	Gm15415	predicted gene 15415 [Source:MGI Symbol;Acc:MGI:3713275]	6.495182	5.937024	7.138495	7.068578	7.118583	8.473923
17493395	Gm15416	predicted gene 15416 [Source:MGI Symbol;Acc:MGI:3705308]	3.168039	2.810253	2.373539	2.839782	3.632317	2.809026
17399426	Gm15417	predicted gene 15417	13.4104	14.31723	13.86149	11.70627	12.08152	14.67664
17498958	Gm15418	predicted gene 15418	6.171709	6.988341	6.116897	7.503221	6.551885	6.372767
17507431	Gm15419	predicted gene 15419 [Source:MGI Symbol;Acc:MGI:3705320]	6.008063	5.123963	4.738531	5.540246	5.165829	5.474473

17335142	Gm15420	predicted gene 15420	11.11719	13.44344	14.84423	19.22604	20.27289	15.82496
17230227	Gm15423	predicted gene 15423	10.75429	9.459015	11.08456	9.511811	9.727169	10.38976
17408067	Gm15441	predicted gene 15441	44.7185	89.79605	65.66124	55.73918	15.11155	13.88832
17408008	Gm15444	predicted gene 15444 [Source:MGI Symbol;Acc:MGI:3705796]	3.062691	3.575711	3.339703	3.945095	3.263862	3.464843
17217590	Gm15445	predicted gene 15445 [Source:MGI Symbol;Acc:MGI:3707341]	4.681043	4.157684	4.116448	3.802478	4.326709	3.007448
17440342	Gm15446	predicted gene 15446	3.570386	3.077055	3.089429	5.223801	3.329026	2.911051
17485649	Gm15448	predicted gene 15448	3.971159	3.091485	2.507188	2.24482	2.824254	3.081003
17222051	Gm15455	predicted gene 15455	5.178305	4.232441	5.37163	3.675906	3.478801	3.966922
17212009	Gm15457	predicted gene 15457 [Source:MGI Symbol;Acc:MGI:3705190]	2.761209	2.953407	2.612389	3.885257	2.458673	3.10325
17335165	Gm15458	predicted gene 15458 [Source:MGI Symbol;Acc:MGI:3705291]	29.0541	24.23651	23.76555	22.4515	23.57229	24.91461
17446757	Gm15461	predicted gene 15461 [Source:MGI Symbol;Acc:MGI:3705274]	7.383629	11.3924	11.36787	12.81969	9.674452	10.80038
17396576	Gm15462	predicted gene 15462 [Source:MGI Symbol;Acc:MGI:3705271]	3.603903	2.895595	3.855612	3.55865	3.285539	4.281392
17548262	Gm15466	predicted gene 15466 [Source:MGI Symbol;Acc:MGI:3705491]	86.76793	96.29373	102.9643	94.348	107.4365	93.46278
17446828	Gm15469	predicted gene 15469 [Source:MGI Symbol;Acc:MGI:3705154]	4.371929	5.03161	5.169662	4.152413	6.431165	4.794299
[truncated: 2,132,802 more chars]
